# Exploration of the gene fusion landscape of glioblastoma using transcriptome sequencing and copy number data

**DOI:** 10.1186/1471-2164-14-818

**Published:** 2013-11-22

**Authors:** Nameeta Shah, Michael Lankerovich, Hwahyung Lee, Jae-Geun Yoon, Brett Schroeder, Greg Foltz

**Affiliations:** The Ben and Catherine Ivy Center for Advanced Brain Tumor Treatment, Swedish Neuroscience Institute, Seattle, WA USA

**Keywords:** Gene fusion, Glioblastoma, RNA-seq, EGFR fusions, NTRK1, ROS1, FGFR3-TACC3, PIK3C2B, Non-coding gene fusions

## Abstract

**Background:**

RNA-seq has spurred important gene fusion discoveries in a number of different cancers, including lung, prostate, breast, brain, thyroid and bladder carcinomas. Gene fusion discovery can potentially lead to the development of novel treatments that target the underlying genetic abnormalities.

**Results:**

In this study, we provide comprehensive view of gene fusion landscape in 185 glioblastoma multiforme patients from two independent cohorts. Fusions occur in approximately 30-50% of GBM patient samples. In the Ivy Center cohort of 24 patients, 33% of samples harbored fusions that were validated by qPCR and Sanger sequencing. We were able to identify high-confidence gene fusions from RNA-seq data in 53% of the samples in a TCGA cohort of 161 patients. We identified 13 cases (8%) with fusions retaining a tyrosine kinase domain in the TCGA cohort and one case in the Ivy Center cohort. Ours is the first study to describe recurrent fusions involving non-coding genes. Genomic locations 7p11 and 12q14-15 harbor majority of the fusions. Fusions on 7p11 are formed in focally amplified EGFR locus whereas 12q14-15 fusions are formed by complex genomic rearrangements. All the fusions detected in this study can be further visualized and analyzed using our website: http://ivygap.swedish.org/fusions.

**Conclusions:**

Our study highlights the prevalence of gene fusions as one of the major genomic abnormalities in GBM. The majority of the fusions are private fusions, and a minority of these recur with low frequency. A small subset of patients with fusions of receptor tyrosine kinases can benefit from existing FDA approved drugs and drugs available in various clinical trials. Due to the low frequency and rarity of clinically relevant fusions, RNA-seq of GBM patient samples will be a vital tool for the identification of patient-specific fusions that can drive personalized therapy.

**Electronic supplementary material:**

The online version of this article (doi:10.1186/1471-2164-14-818) contains supplementary material, which is available to authorized users.

## Background

Cancers result from the accumulation of genomic mutations and epigenetic alterations that change gene expression and function. In particular, gene fusions have been recognized as an associated and significant feature of cancer since the characterization of the Philadelphia chromosome [1]. The occurrence of gene fusions in solid tumors has long been noted, but their importance has been appreciated only recently, largely due to high throughput technologies such as transcriptome sequencing (RNA-seq) [2–5]. RNA-seq permits genome-wide transcription analysis for novel transcript discovery.

RNA-seq has spurred important gene fusion discoveries for a number of different cancers, including lung [6, 7], prostate [3, 8, 9], breast [10–12], brain [13], thyroid [14] and bladder carcinomas [15]. One obvious benefit from gene fusion discovery is the potential to develop novel treatments that target these genetic abnormalities. The EML4-ALK translocation fusion is an example in which the fusion causes constitutive kinase activity. Mouse fibroblasts transfected with EML4-ALK formed tumors when this fusion was injected into nude mice, thus demonstrating the oncogenic activity of the resultant protein [6]. Crizotinib, a competitive inhibitor of ALK, has recently been granted FDA approval for the treatment of specific late-stage, non-small cell lung cancers, and presently there are two phase 3 trials in progress [16].

Glioblastoma multiforme (GBM), a grade IV astrocytoma, is the most common form of primary brain cancer, with a median survival of approximately 1 year after multi-modal treatments [17]. Recent studies suggest that nearly 80% of all malignant brain tumors are accounted for by the broad category of gliomas, and 54% of all malignant brain tumors are GBM [18]. The first fusion protein discovered in glioblastoma was the FIG-ROS1 fusion, in which an intra-chromosomal deletion of 240 kb leads to a constitutively active kinase, suggesting oncogenic activity [19]. Two more studies reported fusions of PDGFRA-KDR [20] and LEO1-SLC12A1 [21], each in a single patient sample. The FGFR-TACC fusion is one of the recurrent fusions in GBM and it has been reported in three studies [13, 22, 23]. The oral administration of an FGFR inhibitor has been shown to prolong the survival of mice harboring intracranial FGFR-TACC-initiated glioma [13]. EGFR-SEPT14 fusions present in about 4% of GBMs were shown to be functional and sensitive to EGFR inhibition in a recent study [24].

In this study, we focus on identification of gene fusion events from GBM transcriptome data. Using our in-house pipeline, we identify and validate 13 fusion events in 24 GBM samples by analyzing SOLiD single-end 50 bp data. We also identify 175 high-confidence gene fusion events in 161 GBM samples by analyzing TCGA Illumina HiSeq paired-end 75 bp transcriptome data. We integrate gene fusion data with copy number data to elucidate fusion mechanisms in GBM.

## Results

### Gene fusion discovery pipeline for SOLiD single-end 50 bp data

**Figure 1:**
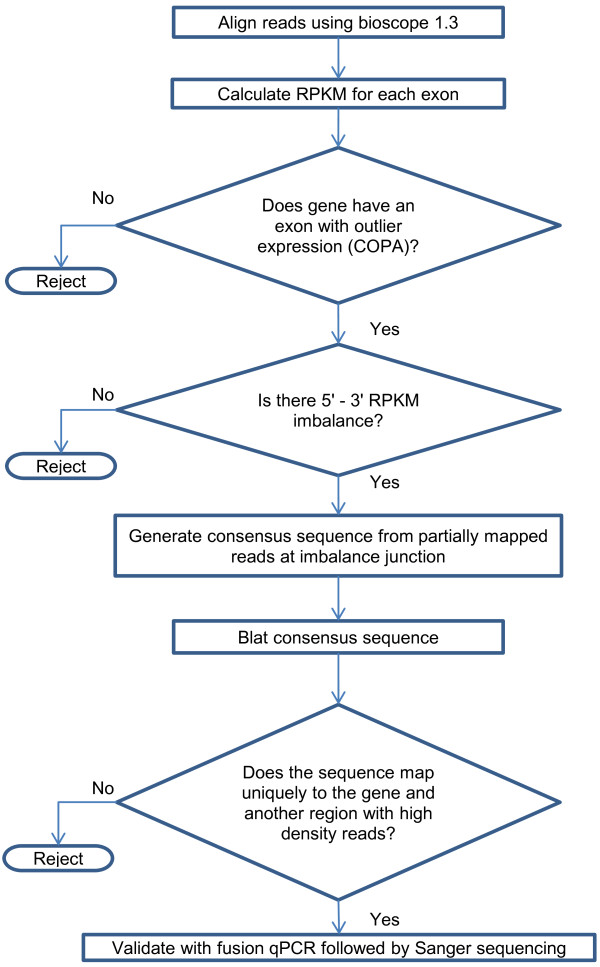
**Gene fusion discovery pipeline for the Ivy Center SOLiD single-end data.** Reads were aligned to the hg19 assembly using bioscope-1.3 software by Life Technologies. RPKM values were calculated for each exon, followed by modified cancer outlier profile analysis (COPA). If any of the exons of a gene displayed outlier expression in a sample, then the read distribution across that gene was evaluated for that sample. If either the 3′ or 5′ end of the gene had a considerably lower RPKM value compared to the other, the gene was further evaluated for fusion. All partially mapped sequences to a potential fusion breakpoint were extracted. One or more consensus sequence was generated and translated to base space format from the color space format. The consensus sequences were then aligned to the hg19 human genome using UCSC BLAT. If part of the consensus sequence mapped to the known exon and the other part uniquely mapped to the genome, the sequence was considered a potential fusion sequence. All potential fusion sequences were validated with fusion qPCR followed by Sanger sequencing.

**Figure 2:**
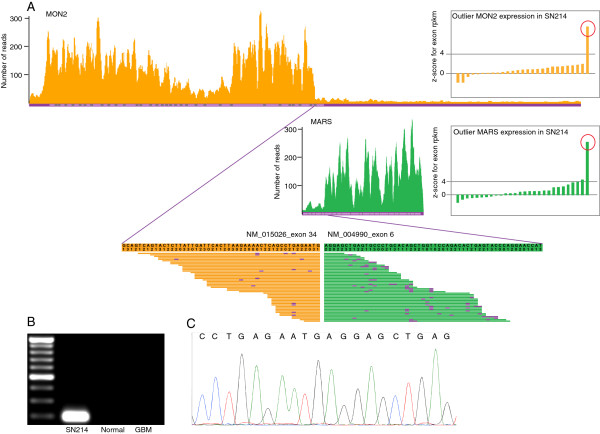
**Example of a gene fusion identified in the Ivy Center SOLiD single-end data.** This figure illustrates the MON2-MARS gene fusion. **A**. Outlier expression of the MON2 and MARS exons can be observed with a z-score > 4. MON2 read distribution shows a drop after exon 34, and MARS read distribution shows a rise at exon 6. Partially mapped reads at exon 34 of MON2 and exon 6 of MARS map to the MON2-MARS fusion sequence. The sequence is represented both in color space and base space format. The purple color indicates a mismatch in the color space format. **B**. Gel images showing the fusion qPCR result. The product can be observed in sample SN214 (TCGA-74-6583) but not in the non-tumor brain and MON2-MARS fusion negative GBM samples. **C**. Trace from Sanger sequencing of the fusion PCR product.

We profiled the transcriptome of 24 GBM samples and 4 non-tumor samples using the SOLiD sequencer. We generated 50 bp single-end RNA-seq reads with sequencing depths ranging from 126 to 205 million reads (details provided in Additional file 1). A variety of software packages are available for gene fusion discovery for Illumina paired-end, Illumina single-end and SOLiD paired-end data [25–29]. We developed an in-house gene fusion discovery pipeline, as no software package was available for single-end SOLiD data (see Figure 1). First, we aligned all the reads and calculated reads per kilobase per million (RPKM) for each exon using Bioscope 1.3 software package [30]. Gene annotations were combined from three databases: Ensembl gene annotation version 66, UCSC and RefSeq genes (the tracks were downloaded on April 4th, 2012, from the UCSC genome browser [31]). Cancer Outlier Profile Analysis (COPA) [3] was performed for each exon to identify exons with substantially higher expression in a small set of samples. We evaluated the expression variation at 5′ and 3′ exons of all genes that had at least one exon with outlier expression levels. We extracted reads that partially mapped to the junction where there was a significant change in expression levels for a given gene. We then constructed a consensus sequence from the partially extracted reads. After converting the consensus sequence from color space to base space format, we used the UCSC BLAT tool [32] to map the consensus sequence to the human genome (hg19 assembly). If part of the consensus sequence mapped to the original gene and the rest mapped uniquely to another region in the genome, then the sequence was considered a fusion sequence. We identified 13 such sequences (see Table 1) in eight samples. We were able to validate all of the 13 fusion transcripts using fusion qPCR followed by Sanger sequencing. Figure 2 illustrates the MON2-MARS gene fusion as one example of a fusion transcript. The outlier expression of the MON2 and MARS exons can be observed with a z-score > 4. Panel A shows the RNA-seq read distribution across all exons for both genes. The MON2 read distribution shows higher 5′ expression relative to its 3′ expression, and the MARS read distribution shows higher 3′ expression relative to its 5′ expression. Partially mapped reads at exon 34 of MON2 and exon 6 of MARS map to the MON2-MARS fusion sequence. Panel B shows the gel image for fusion qPCR. The product can be observed in sample SN214 (TCGA-74-6583) but not in non-tumor brain and MON2-MARS fusion negative GBM samples. Panel C shows the Sanger sequencing trace of the fusion PCR product. Detailed images for the other 12 fusion sequences are available in Additional file 2.

**Table 1:** **Ivy Center fusions**

Ivy Center sample id	Fusion gene symbol (5′ → 3′)	Fusion junction reads	Genomic location (hg19) chromosome (strand)	Type
(TCGA sample id)	Fusion transcripts (5′ → 3′) (UAR-genomic sequence without gene annotation)	WT junction reads (5′,3′)	Coordinates (5′,3′)	
SN214	MON2 → MARS	47	12q14 (+/+)	In-frame fusion
(TCGA-74-6583)	(NM_015026 → NM_004990)	(6, 3)	(62981936, 57883990)	
SN214	MDM1 → UAR	196	12q15 (−/−)	Extended 3′ UTR
(TCGA-74-6583)	(NM_020128 → NA)	(4, NA)	(68717849, 68876024)	
SN214	SLC35E3 → UAR	470	12q15 (+/−)	Truncated gene
(TCGA-74-6583)	(NM_018656 → NA)	(0, NA)	(69145972, 68489752)	
SN238	YEATS4 → SLC35E3	232	12q15 (+/+)	In-frame fusion
	(NM_006530 → NM_018656)	(6, 4)	(69753803, 69152935)	
SN161	PIK3C2B → DSTYK	95	1q32 (−/−)	In-frame fusion
	(NM_002646 → NM_199462)	(3, 0)	(204426856, 205119924)	
SN195-1	PLEKHA6 → PIK3C2B	23	1q32 (−/−)	5′ UTR
	(novel 5′ UTR → NM_002646)	(0, 13)	(204320007, 204439018)	
SN161	CREB1 → PARD3B	65	2q33 (+/+)	Out-of-frame fusion
	(NM_004379 → NM_057177)	(10, 1)	(208442379, 205829875)	
SN214	SCFD2 → CLOCK	10	4q12 (−/−)	In-frame fusion
(TCGA-74-6583)	(NM_152540 → NM_004898)	(4, 6)	(53786892, 56301763)	
SN159	SEC61G → UAR	484	7p11 (+/−)	No protein product
	(ENST00000480303 → NA)	(44, NA)	(51654097, 54821716)	
SN161	LANCL2 → RP11-745C15	274	7p11 (+/+)	Truncated gene
	(NM_018697 → ENST00000439413)	(8, 0)	(55469013, 54872359)	
SN218	ZNF713 → UAR	51	7p11 (+/+)	Truncated gene
	(uc003tra → NA)	(2, NA)	(55991300, 56082944)	
SN154	ZNF713 → UAR	14	7p11 (+/−)	Truncated gene
(TCGA-74-6573)	(uc003tra → NA)	(10, NA)	(55980418, 55945274)	
SN187	FGFR3 → TACC3	13	4p16 (+/+)	In-frame fusion
(TCGA-74-6578)	(NM_000142 → NM_006342)	(31, 0)	(1808661, 1737458)	

Fusions identified in the Ivy Center SOLiD single-end dataset.

### Gene fusions in the Ivy Center SOLiD dataset

We identified 13 fusion transcripts in eight out of the 24 GBM samples (see Table 1). Two samples, SN214 and SN161, harbored multiple fusions. Both fusion partners in eight of the fusion transcripts are well annotated genes. Five (MON2 → MARS, YEATS4 → SLC35E3, PIK3C2B → DSTYK, SCFD2 → CLOCK, FGFR3 → TACC3) out of these eight transcripts are predicted to be in-frame fusions coding for a chimeric protein product. Transcript CREB1 → PARD3B results in a C-terminal truncation of the 5′ fusion gene partner due to a frameshift. In transcript PLEKHA6 → PIK3C2B, the entire PIK3C2B coding sequence is preserved, but the fusion junction is at a novel 5′ UTR exon for PLEKHA6. In transcript LANCL2 → RP11-745C15, the 5′ partner gene fuses with a non-coding RNA resulting in C-terminal truncation. For the other five fusion transcripts, the 5′ partner gene fuses with genomic sequence without gene annotation, denoted as “UAR” in Table 1. Three of these transcripts (SLC35E3 → UAR, ZNF713 → UAR in two samples) result in C-terminal truncation of the 5′ partner genes. One transcript (MDM1 → UAR) is predicted to result in a shorter isoform with an extended 3′ UTR, and one transcript does not have any predicted protein product (SEC61G → UAR). We had tissue available from surgery at recurrence for patient SN159, and we were able to validate the presence of SEC61G → UAR at recurrence. Predicted protein sequences are provided in Additional file 2. All fusions are intra-chromosomal in our cohort, and fusion partners are in close proximity, ranging from a distance of 5.1 million base pairs to 35 kilo base pairs between the two partners. Although there are no recurrent fusions in our small cohort, there are multiple genes, SLC35E3, PIK3C2B and ZNF713, that occurred in more than one fusion transcript. An FGFR3 → TACC3 fusion was recently reported by three independent studies as a recurrent gene fusion [13, 22, 23]. All our fusion transcripts are highly overexpressed compared to their wildtype gene partners, as is evident in the third column in Table 1, which shows a much higher number of reads spanning the fusion junction compared to the number of reads spanning the known wildtype junctions. One of the fusions, SLC35E3 → UAR, has two isoforms.

### Gene fusions in the Illumina HiSeq TCGA dataset

We downloaded RNA-seq data for 169 TCGA samples from CGHub [33] to explore the gene fusion landscape of GBM beyond our cohort. The TCGA transcriptome data are 75 bp paired-end reads generated using Illumina HiSeq with sequencing depths ranging from 54 to 252 million reads per sample. We used the TopHat-Fusion [25] and SnowShoes-FTD [29] software packages to identify fusions because both packages are expected to have a very low false-positive rate. There were 882 and 492 fusion sequences identified by TopHat-Fusion and SnowShoes FTD suggesting a large number of false positives (results are available in Additional files 3 and 4). The number of fusion sequences could be reduced by increasing the threshold for the minimum number of fusion spanning reads, but this modification can lead to the failure to identify some truly important fusions, such as CEP85L → ROS1. In our SOLiD dataset, the FGFR3 → TACC3 fusion has the second lowest number of junction spanning reads. Because our method resulted in a 100% validation rate, we applied filtering steps based on our method to the fusion sequences identified by both packages. We required that at least one of the breakpoints must be a known exon boundary. To reduce the likelihood of identifying passenger fusions [34], we required that at least one of the fusion spanning reads must have a ratio of greater than two compared with its corresponding wild-type exon-exon spanning reads. We discarded gene fusions involving adjacent genes. Exact details are provided in the methods section. After curating fusion sequences from both packages, we obtained a set of 175 high-confidence fusion sequences, which was referred to as the curated set. Curated fusions were present in 53% (85/161) of patients, and 22% (35/161) of these patients harbored more than one fusion. The curated fusion set is available in Additional file 5.

### Gene fusions and copy number changes

**Figure 3:**
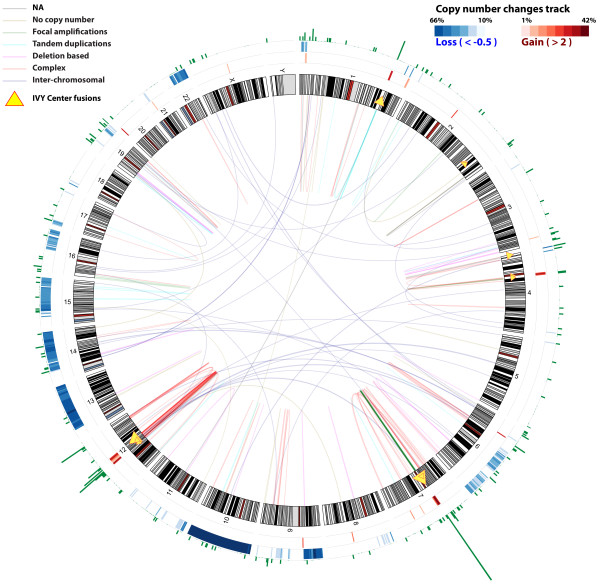
**Gene fusions identified in the TCGA Illumina HiSeq paired-end data and the Ivy Center SOLiD single-end data.** This Circos plot shows all the curated fusions identified in TCGA dataset. Fusions are represented by arcs. The thickness of the arc represents the number of fusion-spanning reads. The colors of the arc represent the likely mechanism of the fusion formation. Green arcs represent fusions formed by focal amplifications, cyan arcs represent fusions formed by tandem duplications, magenta arcs represent deletion-based fusions, dark orange arcs represent fusions formed by complex genomic rearrangements and blue arcs show the fusions formed by inter-chromosomal rearrangements. The outer ring shows the fusion breakpoint density histogram at a given genomic location. Yellow triangles represent the fusions detected in the Ivy Center samples. The size of the triangle indicates the number of breakpoints in that location. The two rings outside of the ideogram show frequency of the copy number gain and loss in TCGA samples. Two major genomic hotspots for fusions can be observed on chromosomes 7 (7p11) and 12 (12q14-15). These regions also show frequent focal gains.

**Figure 4:**
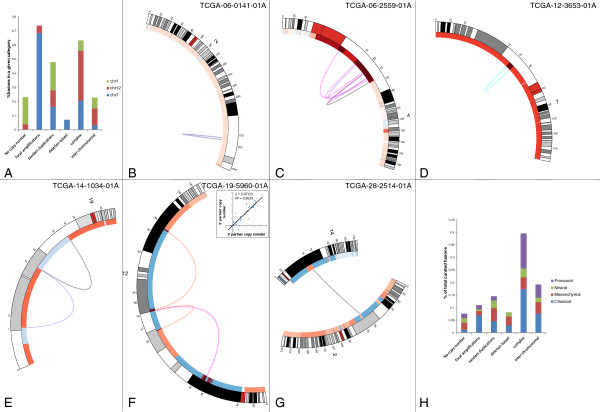
**Association between gene fusions and copy number changes in the TCGA dataset. A**. Distribution of each fusion mechanism over three chromosomes (7, 12 and 1) with most fusions. Majority of fusions due to focal amplifications (68%) are found on chromosome 7. Fusions formed due to complex genomic rearrangements are mostly present on chromosomes 12 and 7. **B**. Fusion without associated copy number changes. **C**. Fusion points within a genomic amplicon. **D**. Fusions due to tandem duplications. **E**. Black arc showing fusion due to interstitial genomic deletion. **F**. Fusions due to complex genomic rearrangements. **G**. Inter-chromosomal fusion with at least one fusion point near a genomic breakpoint. **H**. Distribution of fusion mechanisms for 175 fusion sequences. Eight percent of the fusion sequences do not have associated copy number changes. Forty percent of fusion sequences are formed by complex genomic rearrangements. Fusions in the proneural subtype are mostly formed by complex genomic rearrangements.

The Circos plot of all curated fusions (see Figure 3) shows specific genomic hotspots where fusions occur in GBM. Two major genomic hotspots are on chromosomes 7 (7p11) and 12 (12q14-15). In our SOLiD dataset, 8 of 13 validated fusions were located on 7p11 and 12q14-15. Other regions with higher frequency of fusions are on chromosomes 1, 4, 6 and 19. These genomic hotspots for fusions are the regions that are frequently amplified in GBM, as observed in Figure 3[35]. Because Affymetrix SNP array data were available for all but two TCGA samples, we looked for associations between fusion points and copy number data. We downloaded level 3 segmented copy number data from TCGA [36]. The start and end points of each segment were considered to be the genomic breakpoints. For the curated set, copy number data were available for 172 fusion sequences, out of which at least one of the partner genes harbored a genomic breakpoint in 135 cases (78%). We also predicted the fusion mechanism for each of the fusion sequences based on the copy number data. Figure 4 shows the distribution of different fusion mechanisms for all curated fusions. We binned fusion mechanisms into six types:

1. No copy number changes - There are no genomic breakpoints around fusion points. These could be either inter- or intra-chromosomal fusions, see Figure 4B.
2. Focal amplifications - Fusion points are within a genomic amplicon, see Figure 4C.
3. Tandem duplications - Fusion points are around the start and end of an amplified genomic segment, see Figure 4D.
4. Deletion-based - Fusion points are around the start and end of a relatively deleted genomic segment, see Figure 4E.
5. Complex genomic rearrangements - Both fusion points are around genomic breakpoints with multiple segments between the two breakpoints, see Figure 4F.
6. Inter-chromosomal - Fusion partners are located on different chromosomes with at least one fusion point near a genomic breakpoint, see Figure 4G.

Only 8% of the fusions are without accompanying copy number changes suggesting that the majority of the fusions in GBM are associated with unbalanced genomic rearrangements. Majority of the fusions in focal amplicons are present on chromosome 7 and restricted to the EGFR locus, see Figure 3 and Figure 4A. Approximately 40% of all the fusions in GBM result from complex genomic rearrangements (CGR), see Figure 4H. Some of the inter-chromosomal rearrangements also display complex fusion mechanisms, see Figure 4G. A recent study analyzed whole genome sequencing data and showed a high incidence of CGR in GBM resulting from chromothripsis—39% in GBM compared to 9% in other tumor types [37]. Fusions generated through CGRs are largely present on chromosomes 12 and 7, see Figure 4A. The distribution of CGR based fusions on chromosome 12 is largely restricted to 12q14-15 amplicon, see Figure 3. Even though partners of fusion sequences formed due to CGRs belong to different copy number segments, they have highly correlated copy number value, see Figure 4F. This suggests co-amplification of segments involving fusion.

### Gene fusions and molecular features

**Figure 5:**
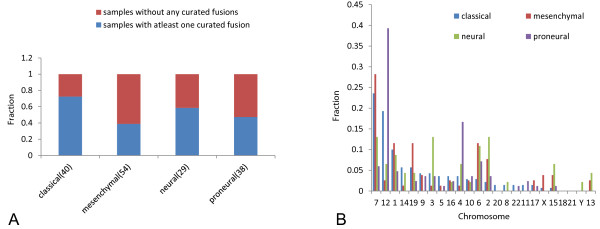
**Association between gene fusions and molecular subtype in the TCGA dataset. A**. Seventy-two percent of the samples with a classical subtype had at least one fusion event compared to 39% of samples with a mesenchymal subtype (Chi-square test p-value = 0.01). **B**. Chromosomal distribution of fusion events for different molecular subtypes.

We checked to see if samples with at least one curated fusion were enriched in any clinically associated molecular features. We did not find any association with presence of EGFR vIII, mutation/homozygous deletion of PTEN or TP53, mutation of IDH1 or G-cimp status (data obtained from CBio portal [38]). Amplifications of EGFR were more prevalent in samples with at least one fusion compared to samples with no fusions (63% vs. 38%, p = 0.0016, Fisher’s exact test). We observed that the samples with classical subtype were more likely to have fusions (72%) and that samples with mesenchymal subtype were less likely to have fusions (39%), see Figure 5A. This result can be explained by the association of genomic fusion hotspots with subtypes. Almost all of the samples with a classical subtype have focal amplification of the EGFR locus, and samples with a mesenchymal subtype have a much lower incidence of focal amplifications on chromosomes 4 and 12 [35]. Figure 5B shows the chromosomal distribution of fusion breakpoints for each subtype. The majority of the fusions in the classical subtype are located on chromosomes 7, 12 and 1. Fusions in the mesenchymal subtype are mostly present on chromosomes 7, 1, 6 and 19, whereas chromosomes 12 and 4 harbor the majority of the fusions with the proneural subtype. Proneural subtype shows enrichment of fusions formed by complex genomic rearrangements, see Figure 4H. Gene fusions in samples with the neural subtype have a broader chromosomal distribution, with the majority of breakpoints on chromosomes 3, 2, 7, 6, 1, 15, 4 and 12.

### Predicted structure of the curated fusions set

We predicted the amino acid sequence of all curated fusions based on their chimeric nucleotide sequence. A significant portion of the fusions (37%) were predicted to be in-frame fusions with amino acid sequences present from both fusion partner genes. Another 18% were predicted to have C-terminal truncation due to either the out of frame fusion with another gene or fusion with an unannotated region. Approximately 8% of fusions are predicted to result in the same protein product as its 3′ partner gene by borrowing only the promoter from the 5′ partner. In approximately 10% of the fusions, the 5′ gene partner is predicted to contribute only the promoter, but the N-terminal of the 3′ gene is truncated. We also observed another novel class of fusions that involve non-coding RNA genes. In approximately 14% (25/175) of the fusions, the 5′ partner gene is predicted to have a C-terminal truncation due to fusion with a non-coding RNA. These fusions also result in the expression of non-coding RNAs that are not expressed in other samples. Another important set of fusions involve tyrosine kinases. In 13 cases, the fusion sequences retained the tyrosine kinase domain. EGFR (6 samples), FGFR3 (2 samples) and NTRK1 (2 samples) were recurrently fused. Other kinase genes included EPHB2, FLT4 and ROS1.

### Recurrent gene fusions

Although more than half of GBMs showed evidence of gene fusions, there were very few fusions that were present in more than one sample. One of those fusions is the already reported FGFR3 → TACC3 fusion, which was found in two patients in the TCGA cohort and in one patient in the Ivy Center cohort. EGFR → SEPT14, an in-frame fusion with C-terminal deletion of EGFR, was found in three patients in the TCGA cohort. LANCL2 → SEPT14, an out of frame fusion that leads to the C-terminal truncation of LANCL2, was found in two TCGA patients. Two additional patients, one TCGA and one Ivy Center, had fusions of LANCL2 with non-coding RNA RP11-745C15.2, which also resulted in the C-terminal truncation of LANCL2. The same non-coding RNA RP11-745C15.2 fused with EGFR in two TCGA patients, resulting in the C-terminal truncation of EGFR. There are 27 genes that are fusion partners in more than one patient sample (see Table 2). The majority of these genes (18/27) are on genomic fusion hotspots located on chromosomes 7 and 12.

**Table 2:** **Fusions involving genes that partner in more than one fusion**

Sample	5′ partner gene	Genomic location		3′ partner gene	Genomic location	
TCGA-06-5856-01A	TSFM	chr12	58180073	IFNG	chr12	68549194
TCGA-28-5207-01A	TSFM	chr12	58191066	TJAP1	chr6	43473030
TCGA-19-2624-01A	PPM1H	chr12	63182005	MDM2	chr12	69202987
TCGA-06-5856-01A	C12orf49	chr12	117175594	MDM2	chr12	69229608
TCGA-27-1835-01A	FGFR3	chr4	1808660	TACC3	chr4	1741428
TCGA-76-4925-01A	FGFR3	chr4	1808660	TACC3	chr4	1739324
TCGA-74-6578 (SN187)	FGFR3	chr4	1808661	TACC3	chr4	1737458
TCGA-06-0129-01A	FRS2	chr12	69864309	KIF5A	chr12	57957221
TCGA-41-2571-01A	FRS2	chr12	69864309	DTX3	chr12	58002302
TCGA-06-0141-01A	GIGYF2	chr2	233562102	ECEL1	chr2	233345866
TCGA-28-2499-01A	GIGYF2	chr2	233613791	PPP1R7	chr2	242107151
TCGA-06-0187-01A	HMGA2	chr12	66232348	NUP107	chr12	69109406
TCGA-06-0686-01A	NUP107	chr12	69096563	RP11-123O10	chr12	67302585
TCGA-14-1034-02B	ADAMTS17	chr15	100589061	LPAR1	chr9	113638001
TCGA-06-0129-01A	NAA15	chr4	140222984	LPAR1	chr9	113638001
TCGA-06-0125-01A	ARID1A	chr1	27094489	RNF31	chr14	24624365
TCGA-06-0125-02A	ARID1A	chr1	27094489	RNF31	chr14	24624365
TCGA-28-2514-01A	ARID1A	chr1	27024031	BEND5	chr1	49202124
TCGA-19-2619-01A	BCAN	chr1	156628525	NTRK1	chr1	156844697
TCGA-06-5411-01A	NFASC	chr1	204951147	NTRK1	chr1	156844362
TCGA-06-0157-01A	NFASC	chr1	204797909	SOX13	chr1	204082042
TCGA-06-0210-01A	NFASC	chr1	204797910	PRELP	chr1	203452296
TCGA-12-1597-01B	NFASC	chr1	204951147	RTN3	chr11	63525627
TCGA-06-5418-01A	CEP85L	chr6	118802941	ROS1	chr6	117641192
TCGA-14-2554-01A	CEP85L	chr6	118953615	SYTL3	chr6	159166511
TCGA-06-2559-01A	CTDSP2	chr12	58240154	LOC100422737	chr6	107172534
TCGA-41-2571-01A	CTDSP2	chr12	58240154	C12orf10	chr12	53699691
TCGA-19-2624-01A	EGFR	chr7	55087057	PPM1H	chr12	63195939
TCGA-28-5209-01A	EGFR	chr7	55268105	PSPHP1	chr7	55840873
TCGA-27-1837-01A	EGFR	chr7	55268106	SEPT14	chr7	55863785
TCGA-28-2513-01A	EGFR	chr7	55268106	SEPT14	chr7	55863785
TCGA-32-5222-01A	EGFR	chr7	55268106	SEPT14	chr7	55863785
TCGA-12-5299-01A	EGFR	chr7	55087057	RP11-436 F9	chr7	54414986
TCGA-06-0219-01A	EGFR	chr7	55240816	RP11-745C15.2	chr7	54860605
TCGA-12-3653-01A	EGFR	chr7	55269474	RP11-745C15.2	chr7	54850284
TCGA-12-3652-01A	VOPP1	chr7	55639963	RP11-745C15.2	chr7	54850800
TCGA-32-2638-01A	LANCL2	chr7	55433921	RP11-745C15.2	chr7	54850800
SN161	LANCL2	chr7	55469013	RP11-745C15.2	chr7	54872357
TCGA-06-0211-01A	LANCL2	chr7	55433921	GS1-18A18	chr7	54643985
TCGA-06-0211-01B	LANCL2	chr7	55433921	GS1-18A18	chr7	54643985
TCGA-06-0211-01A	LANCL2	chr7	55479782	SEPT14	chr7	55886916
TCGA-06-0211-01B	LANCL2	chr7	55479782	SEPT14	chr7	55886916
TCGA-28-2513-01A	LANCL2	chr7	55433922	SEPT14	chr7	55914330
TCGA-14-0817-01A	LANCL2	chr7	55469012	PSPH	chr7	56082822
TCGA-28-5209-01A	LANCL2	chr7	55433921	RP11-310H4	chr7	55714590
TCGA-14-1829-01A	SEC61G	chr7	54823471	RP11-310H4	chr7	55727802
TCGA-06-0211-02A	SEC61G	chr7	54825187	EGFR	chr7	55224225
SN159	SEC61G	chr7	51654097	UAR	chr7	54821716
TCGA-06-0211-01B	MRPS17	chr7	56019622	RP11-436 F9	chr7	54411333
TCGA-06-5856-01A	XRCC6BP1	chr12	58335421	SRRM4	chr12	119583185
TCGA-06-0138-01A	YEATS4	chr12	69764754	XRCC6BP1	chr12	58339410
TCGA-26-5135-01A	SLC16A7	chr12	59990016	RP11-362 K2.2	chr12	59206195
TCGA-02-2485-01A	MARS	chr12	57898081	RP11-362 K2.2	chr12	59195041
TCGA-74-6583 (SN214)	MON2	chr12	62981936	MARS	chr12	57883989
TCGA-74-6583 (SN214)	SLC35E3	chr12	69145972	UAR	chr12	68489752
SN238	YEATS4	chr12	69753803	SLC35E3	chr12	69152935
SN161	PIK3C2B	chr1	204426856	DSTYK	chr1	205119924
SN195-1	PLEKHA6	chr1	204320007	PIK3C2B	chr1	204439018
SN218	ZNF713	chr7	55991300	UAR	chr7	56082944
TCGA-74-6573 (SN154)	ZNF713	chr7	55980418	UAR	chr7	55945274

## Discussion

Our study highlights the prevalence of gene fusions as one of the major genomic abnormalities in GBM. Fusions occur in approximately 30-50% of GBM patient samples. In the Ivy Center cohort of 24 patients, 33% of samples harbored fusions that were validated by qPCR and Sanger sequencing. We were able to identify high-confidence gene fusions from RNA-seq data in 53% of samples in a TCGA cohort of 161 patients. We identified 13 cases (8%) with fusions retaining the tyrosine kinase domain in the TCGA cohort and one case in the Ivy Center cohort. Recent advances in the development of tyrosine kinase inhibitors (TKIs) have demonstrated that these drugs can provide significant benefit to patients whose tumors have a specific genetic abnormality. We also identified a novel class of fusions (14%) that result in the C-terminal truncation of its 5′ partner due to fusion with non-coding RNA genes. One such case was also present in the Ivy Center cohort. This study reveals the diversity of gene fusions in GBM samples. The majority of the fusions are private fusions occurring in one patient. There are a few fusions that recur at low frequency in GBM.

Our study is the first to provide a comprehensive view of the gene fusion landscape in GBM by examining sequences from 185 patients from two independent cohorts. We successfully utilized our in-house pipeline for fusion discovery using SOLiD single-end, 50 bp RNA-seq data with a 100% validation rate. For the TCGA cohort, we used two different gene fusion detection software packages to comprehensively identify fusions from Illumina paired-end, 75 bp RNA-seq data. Ours is the first study to describe recurrent fusions involving non-coding genes. We combined copy number data with gene fusion discovery to elucidate mechanisms of the formation of gene fusions in GBM. All of the fusions detected in this study can be further visualized and analyzed on our website (http://ivygap.swedish.org/fusions).

We were able to validate all of the fusions in our SOLiD single-end RNA-seq data by using strict filtering criteria. It is likely that we may have underestimated fusions for Ivy Center data. Due to lack of access to the tissue samples, we could not determine the validation rate for our set of curated fusions in the TCGA cohort. The curated fusion set did have a significantly higher percentage of fusions associated with copy number changes relative to the low-confidence set. We applied filters to discard likely passenger fusions [34, 39], but the functional significance of these fusions still needs to be evaluated.

Singh et al. was the first study to describe multiple fusions of FGFR-TACC in GBM, reporting this phenomenon in 3 of the 97 tumors examined. They showed that the fusion protein has oncogenic activity when introduced into astrocytes and oral administration of an FGFR inhibitor prolongs the survival of mice harboring intracranial FGFR-TACC-initiated glioma [13]. A second study by Parker et al. showed that the fusion gene is overexpressed by escaping miR-99a regulation due to loss of the 3′ UTR of FGFR3 [22]. In their cohort, 4 out of 48 samples harbored the FGFR3 → TACC3 fusion. In our Ivy Center cohort, the FGFR3 → TACC3 fusion was detected in one out of 72 samples. We tested for this fusion in an additional 48 samples in addition to the 24 RNA-seq samples, but did not detect any fusion events. In the TCGA cohort, 2 of 161 samples harbored the FGFR3 → TACC3 fusion. Fusions of FGFR genes are identified in other cancers, including bladder cancer, cholangiocarcinoma, squamous lung cancer, breast cancer, thyroid cancer, oral cancer, head and neck squamous cell carcinoma and prostate cancer [15, 23]. Tropomyosin-Receptor Kinases (Trk) are known to play a role in cancer biology. Rearrangements of the NTRK1 gene are consistently observed in a small fraction of papillary thyroid carcinomas [40]. We identified two cases of NTRK1 fusions in the TCGA cohort. Frattini et al. [24] screened 248 samples for NFASC-NTRK1 fusion but did not find any. We identified a single case of a CEP85L-ROS1 fusion in the TCGA patient samples. In a recent study by Giacomini et al. [41], a CEP85L-ROS1 fusion was detected for angiosarcoma. There have been two more reported cases, one angiosarcoma and one epithelioid hemangioendothelioma, with ROS1 rearrangements. ROS1 rearrangements also define a unique molecular subclass of lung cancer that may respond to an ALK inhibitor [42]. We identified fusions of EGFR in nine patient samples from the TCGA cohort, out of which six retained the tyrosine kinase domain and resulted in a carboxyl-terminal truncation. A study by Cho et al. has shown that cetuximab prolonged the survival of intracranially xenografted mice with oncogenic EGFR carboxyl-terminal deletion mutants compared with untreated control mice [43]. It is likely that patients with fusions of EGFR leading to carboxyl-terminal truncation will show sensitivity to EGFR inhibitors. Frattini et al. [24] showed that EGFR-SEPT14 fusions which occur in about 4% of GBMs was a functional gene fusion in GBM and confers mitogen independence and sensitivity to EGFR inhibition. A total of 13 cases from both cohorts have fusions of genes involved in chromatin remodeling and modification. These genes include ARID1A, ARID1B, ASH1L, CHD4, HDAC1, HMGA2, JMJD1C, KDM4B, RERE, SETD1B and YEATS4. ARID1A-MAST2 fusion has been shown to be a critical driver fusion in an MDA-MB-468 breast cancer cell line [10]. In 27 samples, the 5′ partner gene fuses with non-coding RNA. These fusions are predicted to have a C-terminal truncation. These cases also have highly expressed non-coding RNAs that are not expressed in other samples. A recent study by Zhang et al. [44] discovered a signature comprising of six long non-coding RNA that predicts survival in GBM. There is now growing evidence of an oncogenic and tumor suppressive role for long, non-coding RNAs in tumor biology [45]. Their identification in gene fusion events has thus far been neglected, as most studies focus on fusions of the coding genes.

Even though gene fusion events in GBM are abundant with scarce recurrent events, they are not random events. Majority of the fusion events occur at 7p11, 12q14-15, 1q32 and 4q12 which are also recurrently amplified regions in GBM. These fusion hotspots are consistent in both Ivy and TCGA cohorts. Also majority of the fusion events are due to unbalanced genomic rearrangements. Analysis of whole genome sequencing data also showed that 88% of genic rearrangements in GBM are associated with copy number alterations [46]. Some of the key genes implicated in GBM biology within these hotspots are EGFR, MDM2, CDK4, PIK3C2B, MDM4 and PDGFRA. A recent study [46] identified a dense breakpoint pattern on 12q14-15 indicative of local chromosome instability and defined this region as “breakpoint enriched region” (BER). They showed that patients with BER pattern had poor survival and this pattern was associated with MDM2/CDK4 co-amplification. There are two other cancers, dedifferentiated liposarcomas and lung adenocarcinomas that also show MDM2/CDK4 co-amplification in 90% and 4% of cases respectively [38, 47]. All three types of cancer display distinct genomic aberration patterns in 12q14-15 region in spite of having MDM2/CDK4 co-amplification. GBM samples show shattering of the region with alternate high level deletions and gains, lung adenocarcinomas mostly contain large amplified segments and dedifferentiated liposarcomas contain multiple amplified segments (see Additional file 6). Whole genome sequencing, copy number and RNA-seq datasets show that GBMs contain deletion bridges that connect these amplified segments and generate a large number of fusion transcripts. Such complex genomic rearrangements are more prevalent on chromosome 12 but not limited to as shown in the study by Malhotra et al. [37] where they analyzed whole genome sequencing data of 18 GBMs. About 40% of fusion transcripts are formed due to such complex genomic rearrangements. With the advent of RNA-seq technology the list of fusion sequences in solid tumors is growing exponentially but little is known about the mechanisms that facilitate fusion events. The formation of the TMPRSS2-ERG gene fusion that occurs in about 50% of prostate cancers has been shown to be facilitated by androgen signaling which induces proximity of the TMPRSS2 and ERG genomic loci and then exposure to gamma irradiation which causes DNA double-strand breaks [48]. The overview of the fusion landscape in GBM leads to questions about what mechanisms are responsible for generating highly site specific DNA double-strand breaks and then joining of these breaks that result in complex genomic rearrangements.

## Conclusions

Gene fusions are frequent genomic abnormalities in GBM. The majority of the fusions are private fusions, with a minority recurring in multiple patients. Complex genomic rearrangements are the major mechanism by which fusions are formed in GBM. Due to the low frequency and rarity of clinically relevant fusions, RNA-seq of GBM patient samples is an essential tool for the identification of patient specific fusions that can drive personalized therapy.

## Methods

### Ethics statement

This study was reviewed and approved by Western IRB (IRB00000533) in compliance with the ethical principles set forth in the report of the National Commission for the Protection of Human Subjects of Biomedical and Behavioral Research, titled “Ethical Principles and Guidelines for the Protection of Human Subjects of Research (Belmont Report)”. The research protocol was also approved by the Swedish Neuroscience Institute research steering committee. All participants provided written informed consent according to IRB guidelines prior to their participation in this study.

### Patient samples

Tumors were obtained from surgeries performed in the years 2009 through 2011 at the Swedish Medical Center (Seattle, WA) according to institutional guidelines. Patient samples used in this study had a histopathology diagnosis of WHO grade IV glioblastoma multiforme.

### Transcriptome sequencing on SOLiD 5500

#### RNA isolation and purification

Total RNA was extracted from human brain tumor tissues with Trizol (Life Technologies, CA) and then purified using the MEGAclear kit (Life Technologies) as per the manufacturer’s instructions. The integrity and quantity of RNA was assessed on the Agilent 2100 Bioanalyzer (Agilent, CA) as per the manufacturer’s recommendations.

### Ribosomal RNA depletion from total RNA

Qualified total RNA was subjected to depletion of ribosomal RNA by using the Ribo-Zero rRNA removal Kit (Epicentre, IL). A total of 5 μg of purified total RNA was mixed with rRNA removal reagents for 25 minutes, added to prepared Ribo-Zero microspheres according to the manufacturer’s instructions, and then incubated for 20 minutes. The mixture was applied to a spin-filter column and centrifuged for 2 minutes to remove the microspheres. rRNA-depleted total RNA was concentrated using the Ribominus concentration module (Life Technologies, CA) and assessed on the Agilent 2100 Bioanalyzer for the confirmation of rRNA removal.

### RNA fragmentation

A total of 500 ng of rRNA-depleted total RNA was subjected to fragmentation by chemical hydrolysis using the SOLiD Total RNA-Seq kit (Life Technologies, CA) according to the manufacturer’s instruction and was assessed on the Agilent 2100 Bioanalyzer for fragment yield and size distribution.

### Construction of an amplified whole transcriptome library

The fragmented rRNA-depleted total RNA samples were used for the construction of an amplified library using the SOLiD Total RNA-Seq kit (Life Technologies, CA) according to the manufacturer’s instruction. Briefly, 100 ng of fragmented RNA was hybridized with SOLiD adaptor mix and followed by ligation of the fragments. Reverse transcription was performed with SOLiD RT primers to generate the cDNA library. The cDNA library was then purified and size selected using AMPure XP reagent (Agencourt, CA) as per the manufacturer’s instruction. Amplification of the cDNA library was performed for multiplex SOLiD sequencing using barcoded 3′ primers. Purification of the amplified DNA was performed using the PureLink PCR micro kit (Life Technologies, CA). Purified DNA was assessed on the Agilent Bioanalyzer 2100 for yield and size distribution.

### Sequencing

The bar-coded libraries were quantified by using the SOLiD Library TaqMan Quantitation kit (Life Technologies, CA), and four bar-coded libraries were pooled together in equal concentrations into one pool. The pooled libraries were used as the template for the next step of emulsion PCR and were followed by enrichment. Emulsion PCR and enrichment were performed at the E120 scale in SOLiD EZ Bead System (Life Technologies, CA) according to the manufacturer’s instructions. Each pool was sequenced in a SOLiD FlowChip on the SOLiD 5500 (Life Technologies, CA) according to the manufacturer’s instructions.

### TCGA transcriptome and copy number data

TCGA transcriptome data were downloaded from CGHub [33]. The level 3 copy number data were obtained from the TCGA data portal [36].

### Gene fusion discovery process for SOLiD 5500 data

Reads were aligned to hg19 assembly using bioscope 1.3 software by Life Technologies [30]. The gene annotation file was obtained by combining annotations from Ensembl gene annotation version 66, UCSC and RefSeq genes (the tracks were downloaded on April 4th, 2012, from the UCSC genome browser [31]). RPKM values were calculated for each exon, followed by a modified cancer outlier profile analysis (COPA) [3]. If any of the exons of a gene displayed outlier expression in a sample, then the read distribution across that gene was evaluated for that sample. If either the 3′ or 5′ end of the gene had a considerably lower RPKM value compared to the other end, the gene was further evaluated for fusion events.

### Alignments

Bioscope 1.3 was run using its default settings. The RPKM values were calculated using the “Count Known Exons” tool with quality cutoffs minMapq = 10 and scoreClearZone = 5. Exons that have an RPKM value greater than 20 in at least one of the samples were evaluated for outlier expression.

### Cancer outlier profile analysis (COPA)

For each exon, RPKM values are sorted in ascending order. We calculate z-score z_i_ in sample *i* as *Z*_*i*_ = (*x*_*i*_ − *μ*)/*σ*

where average and standard deviation are calculated as follows:

where n = number of samples and k = index to the array of sorted RPKM values.

An exon is considered to have an outlier expression if the z-score is greater than 4.

### Exon-walking RNA-seq expression pattern

Earlier studies have utilized exon-walk PCR to identify fusion breakpoints [3]. We used a similar approach using RNA-seq RPKM data for each exon. For each exon number *j* of gene *i* and sample *k* RPKM is normalized by the 7th quantile RPKM values for exon number *j* of gene *i* as follows:

When walking from *j*^*th*^ exon to the *(j + 1)*^*th*^ exon, if there is a two-fold drop or rise in normalized RPKM value, then the exon-exon boundary is considered a potential fusion breakpoint.

### Consensus sequence

All partially mapped sequences to a potential fusion breakpoint were extracted. These sequences have less than 35 matches to the known exon. One or more consensus sequences were generated and translated to base space format from the color space format. At least two sequences were used to generate a consensus sequence.

### Blat

The consensus sequences were then aligned to the hg19 human genome using BLAT [32].

If the part of the consensus sequence mapped to the known exon and the other part uniquely mapped to the genome, the sequence was considered a fusion sequence.

### Fusion qPCR

cDNAs were synthesized by using the High Capacity cDNA Reverse Transcription Kit (Life Technologies) with 1 μg of purified total RNA. Primers specific for fusion genes that were used in RT-qPCR are listed in Additional file 7. GUSB was used as the internal reference gene. For each fusion sequence three samples were used: the GBM sample containing the fusion, the GBM sample without that fusion and the non-tumor brain sample.

### Sanger sequencing

The RT-PCR products were selectively extracted from an agarose gel and cloned into the pCR2.1-TOPO cloning vector (Life Technologies). All clones were confirmed by sequencing using 3130 Genetic Analyzer (Life Technologies).

### Gene fusion discovery process for TCGA Illumina HiSeq data

#### TopHat

We used TopHat-2.0.4. Linux_x86_64 version of the TopHat software. The following command was used to generate alignments:

*tophat -o****OUTDIRECTORY****-p****12****–fusion-search –keep-fasta-order –bowtie1 –no-coverage-search -r****300****–mate-std-dev****500****–fusion-min-dist****100000****–fusion-anchor-length****20****–fusion-ignore-chromosomes chrM hg19 samplename_1.fastq samplename_2.fastq*

After generating alignments for all samples, the following command was used to the generate fusion transcript output:

*tophat-fusion-post2 -p****12****–num-fusion-reads****1****–num-fusion-pairs****2****–num-fusion-both****10****hg19*

### SnowShoes-FTD

We used SnowShoes-FTD_2.0_Build37 version of the SnowShoes-FTD software. We followed the instructions provided in the user manual (filename – User_Manual_Build37_06-04-2012.pdf). We trimmed the RNA-seq reads to a 50-bp read length, as per the recommendations in the manual. The following parameters were set in the configure_file.txt:

- $read_length = 50
- $distance = 50000
- $lib_size = 300
- $minimal = 5
- $max_fusion_isoform = 5

### Curated fusions

For TopHat fusions, we considered all of the potential fusions in the output file, potential_fusion.txt, and not just the fusions reported in result.txt. We used the output file final_fusion_report_RNA.txt for SnowShoes-Ftd fusions. For all exons of the genes involved in fusion transcripts, we calculated z-scores as described in the above section. Exon RPKM for the TCGA data was calculated using script *coverageBed* in package BEDTools-Version-2.16.2 [49]. The following conditions were met by the fusion transcripts in the curated set:

1. At least one of the breakpoints was a known exon boundary.
2. At least one of the ratios of fusion spanning reads vs. corresponding wild-type exon-exon spanning reads was greater than 2.
3. Number of fusion spanning reads ≥ 100 or outlier z-score value ≥ 5.
4. If only present in potential_fusion.txt then outlier z-score value ≥ 10.
5. Fusion sequence maintains the 5′ → 3′ direction.
6. Not identified in normal tissues (TFG → GPR128 [50]).

## Electronic supplementary material

Additional file 1: **Is a table listing RNA-seq depth of sequencing and clinical data for the Ivy Center cohort.** (XLSX 15 KB)

Additional file 2: **Contains details of Ivy Center fusions with predicted protein sequences.** (GZ 9 MB)

Additional file 3: **Contains output from TopHat software for the TCGA cohort.** (GZ 5 MB)

Additional file 4: **Contains output from SnowShoes-FTD software for the TCGA cohort.** (GZ 14 MB)

Additional file 5: **Is a table listing curated fusion set for the TCGA cohort.** (XLSX 27 KB)

Additional file 6: **Is a snapshot of Integrated Genome Viewer showing genomic rearrangements on 12q14-15 in GBM, lung adenocarcinomas and sarcomas.** (PNG 85 KB)

Additional file 7: **Is a table listing fusion qPCR primers.** (XLS 31 KB)
