# Supplementary material for: Exploration of the gene fusion landscape of glioblastoma using transcriptome sequencing and copy number data
Source: BMC Genomics. 2013 Nov 22;14(1):818. doi: 10.1186/1471-2164-14-818 (PMC4046790; doi:10.1186/1471-2164-14-818)
Supplement: Supplementary file 2 — Additional file 2: Contains details of Ivy Center fusions with predicted protein sequences. (GZ 9 MB) [file 12864_2013_5514_MOESM2_ESM.gz › s2/fgfr3-tacc3.pdf]

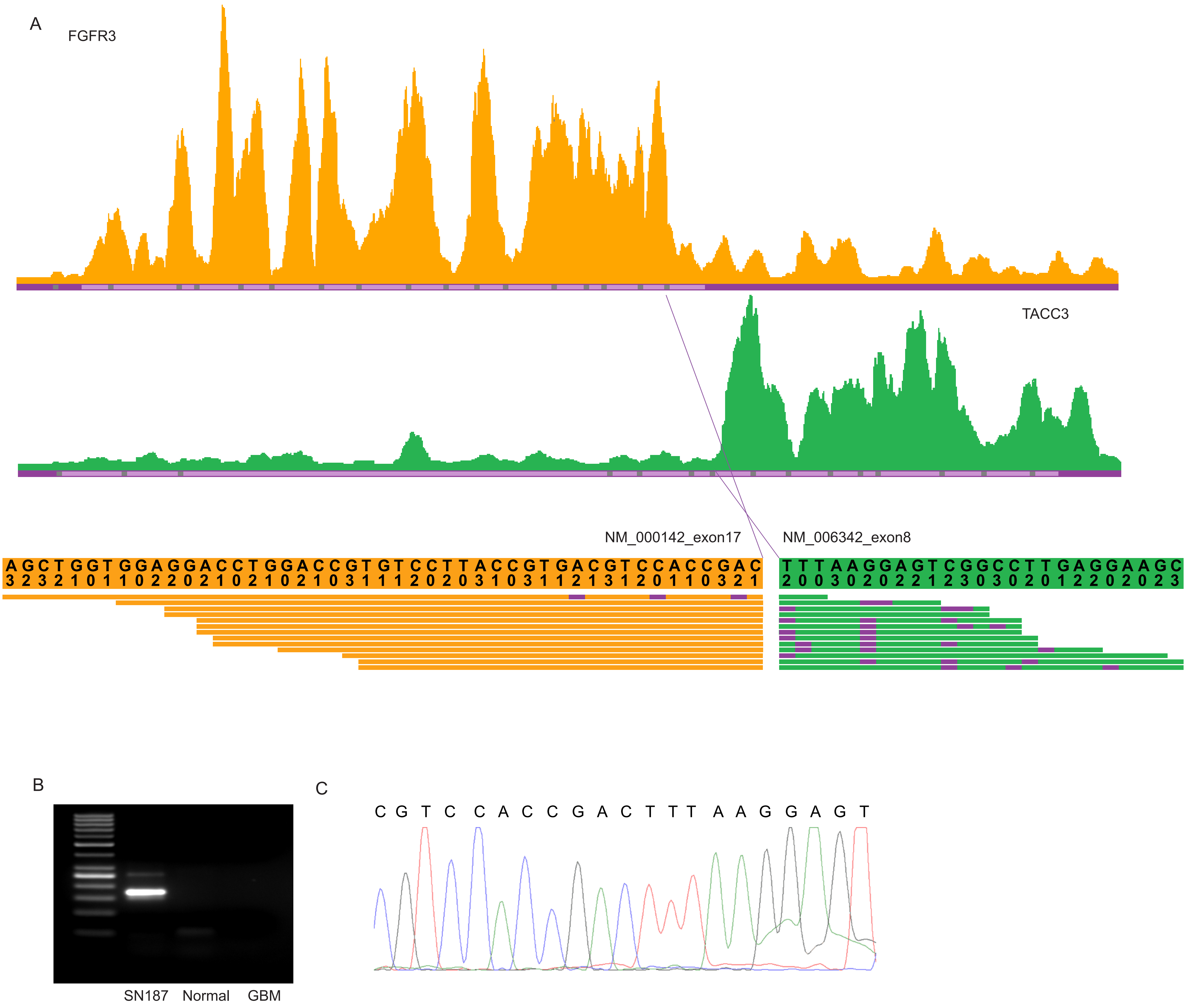
A
FGFR3
TACC3
NM_000142_exon17
NM_006342_exon8
AGCTGGTGGAGGACCTGGACCGTGTCCTTACCGTGACGTCCACCGAC
32321011022021021021031112020310311213120110321
TTTAAGGAGTCGGCCTTGAGGAAGC
2003020221230302012220202 3
B
SN187
Normal
GBM
C
C G T C C A C C G A C T T T A A G G A G T
