# Supplementary material for: Exploration of the gene fusion landscape of glioblastoma using transcriptome sequencing and copy number data
Source: BMC Genomics. 2013 Nov 22;14(1):818. doi: 10.1186/1471-2164-14-818 (PMC4046790; doi:10.1186/1471-2164-14-818)
Supplement: Supplementary file 2 — Additional file 2: Contains details of Ivy Center fusions with predicted protein sequences. (GZ 9 MB) [file 12864_2013_5514_MOESM2_ESM.gz › s2/proteinsequences.docx]

>mon2-mars

MSGTSSPEAVKKLLENMQSDLRALSLECKKKFPPVKEAAESGIIKVKTIAARNTEILAALKENSSEVVQPFLMGCGTKEPKITQLCLAAIQRLMSHEVVSETAAGNIINMLWQLMENSLEELKLLQTVLVLLTTNTVVHDEALSKAIVLCFRLHFTKDNITNNTAAATVRQVVTVVFERMVAEDERHRDIIEQPVLVQGNSNRRSVSTLKPCAKDAYMLFQDLCQLVNADAPYWLVGMTEMTRTFGLELLESVLNDFPQVFLQHQEFSFLLKERVCPLVIKLFSPNIKFRQGSSTSSSPAPVEKPYFPICMRLLRVVSVLIKQFYSLLVTECEIFLSLLVKFLDADKPQWLRAVAVESIHRFCVQPQLLRSFCQSYDMKQHSTKVFRDIVNALGSFIQSLFLVPPTGNPATSNQAGNNNLGGSVSAPANSGMVGIGGGVTLLPAFEYRGTWIPILTITVQGSAKATYLEMLDKVEPPTIPEGYAMSVAFHCLLDLVRGITSMIEGELGELETECQTTTEEGSSPTQSTEQQDLQSTSDQMDKEIVSRAVWEEMVNACWCGLLAALSLLLDASTDEAATENILKAELTMAALCGRLGLVTSRDAFITAICKGSLPPHYALTVLNTTTAATLSNKSYSVQGQSVMMISPSSESHQQVVAVGQPLAVQPQGTVMLTSKNIQCMRTLLNLAHCHGAVLGTSWQLVLATLQHLVWILGLKPSSGGALKPGRAVEGPSTVLTTAVMTDLPVISNILSRLFESSQYLDDVSLHHLINALCSLSLEAMDMAYGNNKEPSLFAVAKLLETGLVNMHRIEILWRPLTGHLLEVCQHPNSRMREWGAEALTSLIKAGLTFNHDPPLSQNQRLQLLLLNPLKEMSNINHPDIRLKQLECVLQILQSQGDSLGPGWPLVLGVMGAIRNDQGESLIRTAFQCLQLVVTDFLPTMPCTCLQIVVDVAGSFGLHNQELNISLTSIGLLWNISDYFFQRGETIEKELNKEEAAQQKQAEEKGVVLNRPFHPAPPFDCLWLCLYAKLGELCVDPRPAVRKSAGQTLFSTIGAHGTLLQHSTWHTVIWKVLFHLLDRVRESSTTADKEKIESGGGNILIHHSRDTAEKQWAETWVLTLAGVARIFNTRRYLLQPLGDFSRAWDVLLDHIQSAALSKNNEVSLAALKSFQEILQIVSPVRDSDKPETPPVVNVPVPVLIGPISGMSRPFVRTDSIGEKLGRYSSSEPPIVTDELEDLNLWWAAWNTWYRIGSESTKPPITFDKLTFIPSQPFLTALIQIFPALYQHIKTGFNMDDLQKLGVILHSAISVPISSDASPFILPSYTEAVLTSLQEAVLTALDVLQKAICVGPENMQIMYPAIFDQLLAFVEFSCKPPQYGQLETKHIANAKYNQIQLFAPAEWVALNYVPFAERSLEVVVDLYQKTACHKAVVNEKVLQNIIKTLRVPLSLKYSCPSESTWKLAVSSLLRVLSIGLPVARQHASSGKFDSMWPELANTFEDFLFTKSIPPDNLSIQEFQRNENIDVEVVQLISNEILPYANFIPKEFVGQIMTMLNKGSIHSQSSSFTEAEIDIRLREEFSKMCFETLLQFSFSNKVTTPQEGYISRMALSVLLKRSQDVLHRYIEDERLSGKCPLPRQQVTEIIFVLKAVSTLIDSLKKTQPEN

EELSALHSWFQTLSTQEPCQRAAETVLKQQGVLALRPYLQKQPQPSPAEGRAVTNEPEEEELATLSEEEIAMAVTAWEKGLESLPPLRPQQNPVLPVAGERNVLITSALPYVNNVPHLGNIIGCVLSADVFARYSRLRQWNTLYLCGTDEYGTATETKALEEGLTPQEICDKYHIIHADIYRWFNISFDIFGRTTTPQQTKITQDIFQQLLKRGFVLQDTVEQLRCEHCARFLADRFVEGVCPFCGYEEARGDQCDKCGKLINAVELKKPQCKVCRSCPVVQSSQHLFLDLPKLEKRLEEWLGRTLPGSDWTPNAQFITRSWLRDGLKPRCITRDLKWGTPVPLEGFEDKVFYVWFDATIGYLSITANYTDQWERWWKNPEQVDLYQFMAKDNVPFHSLVFPCSALGAEDNYTLVSHLIATEYLNYEDGKFSKSRGVGVFGDMAQDTGIPADIWRFYLLYIRPEGQDSAFSWTDLLLKNNSELLNNLGNFINRAGMFVSKFFGGYVPEMVLTPDDQRLLAHVTLELQHYHQLLEKVRIRDALRSILTISRHGNQYIQVNEPWKRIKGSEADRQRAGTVTGLAVNIAALLSVMLQPYMPTVSATIQAQLQLPPPACSILLTNFLCTLPAGHQIGTVSPLFQKLENDQIESLRQRFGGGQAKTSPKPAVVETVTTAKPQQIQALMDEVTKQGNIVRELKAQKADKNEVAAEVAKLLDLKKQLAVAEGKPPEAPKGKKKK

>mdm1-utr1 (same as NP_001191958.1)

MPVRFKGLSEYQRNFLWKKSYLSESCNSSVGRKYPWAGLRSDQLGNQGRCRTKIQHSDISSLLILVCST

>slc35e3-utr

MALLVDRVRGHWRIAAGLLFNLLVSICIVFLNKWIYVYHGFPNMSLTLVHFVVTWLGLYICQKLDIFAPKSLPPSRLLLLALSFCGFVVFTNLSLQNNTIGTYQLAKAMTTPVIIAIQTFCYQKTFSTRIQLTLIPITLGVILNSYYDVKFNFLGMVFAALGVLVTSLYQVWVGAKQHELQVNSMQLLYYQAPMSSAMLLVAVPFFEPVFGEGGIFGPWSVSAL

VACPEKTQRQWCQGRTEFHK

>yeats4-slc35e3

MFKRMAEFGPDSGGRVK

LMVLLSGVIAFMVNLSIYWIIGNTSPVTYNMFGHFKFCITLFGGYVLFKDPLSINQALGILCTLFGILAYTHFKLSEQEGSRSKLAQRP

>pik3c2b-dstyk

MSSTQGNGEHWKSLESVGISRKELAMAEALQMEYDALSRLRHDKEENRAKQNADPSLISWDEPGVDFYSKPAGRRTDLKLLRGLSGSDPTLNYNSLSPQEGPPNHSTSQGPQPGSDPWPKGSLSGDYLYIFDGSDGGVSSSPGPGDIEGSCKKLSPPPLPPRASIWDTPPLPPRKGSPSSSKISQPSDINTFSLVEQLPGKLLEHRILEEEEVLGGGGQGRLLGSVDYDGINDAITRLNLKSTYDAEMLRDATRGWKEGRGPLDFSKDTSGKPVARSKTMPPQVPPRTYASRYGNRKNATPGKNRRISAAPVGSRPHTVANGHELFEVSEERDEEVAAFCHMLDILRSGSDIQDYFLTGYVWSAVTPSPEHLGDEVNLKVTVLCDRLQEALTFTCNCSSTVDLLIYQTLCYTHDDLRNVDVGDFVLKPCGLEEFLQNKHALGSHEYIQYCRKFDIDIRLQLMEQKVVRSDLARTVNDDQSPSTLNYLVHLQERPVKQTISRQALSLLFDTYHNEVDAFLLADGDFPLKADRVVQSVKAICNALAAVETPEITSALNQLPPCPSRMQPKIQK

LDKQNRAKITDLGFCKPEAMMSGSIVGTPIHMAPELFTGARPERLPVFDEECWQLMEACWDGDPLKRPLLGIVQPMLQGIMNRLCKSNSEQPNRGLDDST

>plekha6-pik3c2b

MSSTQGNGEHWKSLESVGISRKELAMAEALQMEYDALSRLRHDKEENRAKQNADPSLISWDEPGVDFYSKPAGRRTDLKLLRGLSGSDPTLNYNSLSPQEGPPNHSTSQGPQPGSDPWPKGSLSGDYLYIFDGSDGGVSSSPGPGDIEGSCKKLSPPPLPPRASIWDTPPLPPRKGSPSSSKISQPSDINTFSLVEQLPGKLLEHRILEEEEVLGGGGQGRLLGSVDYDGINDAITRLNLKSTYDAEMLRDATRGWKEGRGPLDFSKDTSGKPVARSKTMPPQVPPRTYASRYGNRKNATPGKNRRISAAPVGSRPHTVANGHELFEVSEERDEEVAAFCHMLDILRSGSDIQDYFLTGYVWSAVTPSPEHLGDEVNLKVTVLCDRLQEALTFTCNCSSTVDLLIYQTLCYTHDDLRNVDVGDFVLKPCGLEEFLQNKHALGSHEYIQYCRKFDIDIRLQLMEQKVVRSDLARTVNDDQSPSTLNYLVHLQERPVKQTISRQALSLLFDTYHNEVDAFLLADGDFPLKADRVVQSVKAICNALAAVETPEITSALNQLPPCPSRMQPKIQKDPSVLAVRENREKVVEALTAAILDLVELYCNTFNADFQTAVPGSRKHDLVQEACHFARSLAFTVYATHRIPIIWATSYEDFYLSCSLSHGGKELCSPLQTRRAHFSKYLFHLIVWDQQICFPVQVNRLPRETLLCATLYALPIPPPGSSSEANKQRRVPEALGWVTTPLFNFRQVLTCGRKLLGLWPATQENPSARWSAPNFHQPDSVILQIDFPTSAFDIKFTSPPGDKFSPRYEFGSLREEDQRKLKDIMQKESLYWLTDADKKRLWEKRYYCHSEVSSLPLVLASAPSWEWACLPDIYVLLKQWTHMNHQDALGLLHATFPDQEVRRMAVQWIGSLSDAELLDYLPQLVQALKYECYLDSPLVRFLLKRAVSDLRVTHYFFWLLKDGLKDSQFSIRYQYLLAALLCCCGKGLREEFNRQCWLVNALAKLAQQVREAAPSARQGILRTGLEEVKQFFALNGSCRLPLSPSLLVKGIVPRDCSYFNSNAVPLKLSFQNVDPLGENIRVIFKCGDDLRQDMLTLQMIRIMSKIWVQEGLDMRMVIFRCFSTGRGRGMVEMIPNAETLRKIQVEHGVTGSFKDRPLADWLQKHNPGEDEYEKAVENFIYSCAGCCVATYVLGICDRHNDNIMLKTTGHMFHIDFGRFLGHAQMFGNIKRDRAPFVFTSDMAYVINGGDKPSSRFHDFVDLCCQAYNLIRKHTHLFLNLLGLMLSCGIPELSDLEDLKYVYDALRPQDTEANATTYFTRLIESSLGSVATKLNFFIHNLAQMKFTGSDDRLTLSFASRTHTLKSSGRISDVFLCRHEKIFHPNKGYIYVVKVMRENTHEATYIQRTFEEFQELHNKLRLLFPSSHLPSFPSRFVIGRSRGEAVAERRREELNGYIWHLIHAPPEVAECDLVYTFFHPLPRDEKAMGTSPAPKSSDGTWARPVGKVGGEVKLSISYKNNKLFIMVMHIRGLQLLQDGNDPDPYVKIYLLPDPQKTTKRKTKVARKTCNPTYNEMLVYDGIPKGDLQQRELQLSVLSEQGFWENVLLGEVNIRLRELDLAQEKTGWFALGSRSHGTL

>creb1-pard3b 5'3' Frame 2

MTRTVEISGEGGPLGIHVVPFFSSLSGRILGLFIRGIEDNSRSKREGLFHENECIVKINNVDLVDKTFAQAQDVFRQAMKSPSVLLHVLPPQNREQYEKSVIGSLNIFGNNDGVLKTKVPPPVHGKSGLKTANLTGTDSPETDASASLQQNKSPRVPRLGGKPSSPSLSPLMGFGSNKNAKKIKIDLKKGPEGLGFTVVTRDSSIHGPGPIFVKNILPKGAAIKDGRLQSGDRILEVNGRDVTGRTQEELVAMLRSTKQGETASLVIARQEGHFLPRELKGEPDCCALSLETSEQLTFEIPLNDSGSAGLGVSLKGNKSRETGTDLGIFIKSIIHGGAAFKDGRLRMNDQLIAVNGESLLGKSNHEAMETLRRSMSMEGNIRGMIQLVILRRPERPMEDPAECGAFSKPCFENCQNAVTTSRRNDNSILHPLGTCSPQDKQKGLLLPNDGWAESEVPPSPTPHSALGLGLEDYSHSSGVDSAVYFPDQHINFRSVTPARQPESINLKASKSMDLVPDESKVHSLAGQKSDGLSDKSSHSGQGALNCESAPQGNSELEDMENKARKVKKTKEKEKKKEKGKLKVKEKKRKEENEDPERKIKKKGFGAMLRFGKKKEDKGGKAEQKGTLKHGGLREEELEKMKEERERIGAKHQELREKQARGLLDYATGAIGSVYDMDDDEMDPNYARVNHFREPCTSANVFRSPSPPRAGPFGYPRDGHPLSPERDHLEGLYAKVNKPYHPLVPADSGRPTGGSTDRIQKLRKEYYQARREGFPLYEDDEGRARPSEYDLLWVPGRGPDGNAHNLRFEGMERQYASLPRGGPADPVDYLPAAPRGLYKERELPYYPGAHPMHPPKGSYPRPTELRVADLRYPQHYPPPPAPQHKGPFRQDVPPSPPQHQRMPAYQETGRPGPRGGSPDQYPYRTQDSRQKNPMTAAV

>creb1-pard3b 5'3' Frame 3

MTMESGAENQQSGDAAVTEAENQQMTVQAQPQIATLAQVSMPAAHATSSAPTVTLVQLPNGQTVQVHGVIQAAQPSVIQSPQVQTVQISTIAESEDSQESVDSVTDSQKRREILSRRPSYRKILNDLSSDAPGVPRIEEEKSEEETSAPAITTVTVPTPIYQTSSGQYIAITQGGAIQLANNGTDGVQGLQTLTMTNAAATQPGTTILQYAQTTDGQQILVPSNQVVVQAASGDVQTYQIRTAPTSTIAPGVVMASSPALPTQPAEEAARKREVRLMKN

S

>scfd2-clock

MSASGVLSFTQQGWEQVLAKVKRAVVYLDAACAESLHWGCGSTRLLEAVGGPDCHLREFEPDAIGGGAKQPKAVFVLSCLLKGRTVEILRDIICRSHFQYCVVVTTVSHAVHLTANHVPAAAAAEMEGQQPVFEQLEEKLCEWMGNMNYTAEVFHVPLLLAPVAPHFALTPAFASLFPLLPQDVHLLNSARPDKRKLGSLGDVDSTTLTPELLLQIRCLVSGLSSLCEHLGVREECFAVGSLSQVIAADLANYAPAKNRKKTAAGRASVVFVDRTLDLTGAVGHHGDNLVEKIISALPQLPGHTNDVMVNMIALTALHTEEENYNVVAPGCLSQSSDTTAKALWEALLNTKHKEAVMEVRRHLVEAASRENLPIKMSMGRVTPGQLMSYIQLFKNNLKALMNHCGLLQLGLATAQTLKHPQTAKWDNFLAFERLLLQSIGESAMSVVLNQLLPMIKPVTQRTNEDYSPEELLILLIYIYSVTGELTVDKDLCEAEEKVKKALAQVFCEESGLSPLLQKITDWDSSINLTFHKSKIAVDELFTSLRDIAGARSLLKQFKSVYVPGNHTHQ

TSRLLHGNPSTQLILSAAFPLQQSTFPQSHHQQHQSQQQQQLSRHRTDSLPDPSKVQPQ

>sec61g-utr

MDQVMQFVEPSRQFVKDSIRLVKRCTKPDRKEFQKIAMATAIGFAIMGFIGFFVKLIHIPINNIIV

>lancl2-RP11-715C15

MGETMSKRLKLHLGGEAEMEERAFVNPFPDYEAAAGALLASGAAEETGCVRPPATTDEPGLPFHQDGKIIHNFIRRIQTKIKDLLQQMEEGLKTADPHDCSAYTGWTGIALLYLQLYRVTCDQTYLLRSLDYVKRTLRNLNGRRVTFLCGDAGPLAVGAVIYHKLRSDCESQECVTKLLQLQRSVVCQESDLPDELLYGRAGYLYALLYLNTEIGPGTVCESAIKEVVNAIIESGKTLSREERKTERCPLLYQWHRKQYVGAAHGMAGIYYMLMQ

SED

>ZNF713-utr

MPSQNAVFSQEGNMEEEEMNDGSQMVRSQESLTFQDVAVDFTREEWDQLYPAQKNLYRDVMLENYRNLVALGYQLCKPEVIAQLELEEEWVIERDSLLDTHP

DSAVGCVSSKPAYSP

>ZNF713-utr (SN154)

MPSQNAVFSQEGNMEEEEMNDGSQMVRSQ

V

>fgfr3-tacc3

MGAPACALALCVAVAIVAGASSESLGTEQRVVGRAAEVPGPEPGQQEQLVFGSGDAVELSCPPPGGGPMGPTVWVKDGTGLVPSERVLVGPQRLQVLNASHEDSGAYSCRQRLTQRVLCHFSVRVTDAPSSGDDEDGEDEAEDTGVDTGAPYWTRPERMDKKLLAVPAANTVRFRCPAAGNPTPSISWLKNGREFRGEHRIGGIKLRHQQWSLVMESVVPSDRGNYTCVVENKFGSIRQTYTLDVLERSPHRPILQAGLPANQTAVLGSDVEFHCKVYSDAQPHIQWLKHVEVNGSKVGPDGTPYVTVLKTAGANTTDKELEVLSLHNVTFEDAGEYTCLAGNSIGFSHHSAWLVVLPAEEELVEADEAGSVYAGILSYGVGFFLFILVVAAVTLCRLRSPPKKGLGSPTVHKISRFPLKRQVSLESNASMSSNTPLVRIARLSSGEGPTLANVSELELPADPKWELSRARLTLGKPLGEGCFGQVVMAEAIGIDKDRAAKPVTVAVKMLKDDATDKDLSDLVSEMEMMKMIGKHKNIINLLGACTQGGPLYVLVEYAAKGNLREFLRARRPPGLDYSFDTCKPPEEQLTFKDLVSCAYQVARGMEYLASQKCIHRDLAARNVLVTEDNVMKIADFGLARDVHNLDYYKKTTNGRLPVKWMAPEALFDRVYTHQSDVWSFGVLLWEIFTLGGSPYPGIPVEELFKLLKEGHRMDKPANCTHDLYMIMRECWHAAPSQRPTFKQLVEDLDRVLTVTSTD

FKESALRKQSLYLKFDPLLRDSPGRPVPVATETSSMHGANETPSGRPREAKLVEFDFLGALDIPVPGPPPGVPAPGGPPLSTGPIVDLLQYSQKDLDAVVKATQEENRELRSRCEELHGKNLELGKIMDRFEEVVYQAMEEVQKQKELSKAEIQKVLKEKDQLTTDLNSMEKSFSDLFKRFEKQKEVIEGYRKNEESLKKCVEDYLARITQEGQRYQALKAHAEEKLQLANEEIAQVRSKAQAEALALQASLRKEQMRIQSLEKTVEQKTKENEELTRICDDLISKMEKISRLLHGNPSTQLILSAAFPLQQSTFPQSHHQQHQSQQQQQLSRHRTDSLPDPSKVQPQ
