# Supplementary figures and images for: Exploration of the gene fusion landscape of glioblastoma using transcriptome sequencing and copy number data
Source: BMC Genomics. 2013 Nov 22;14(1):818. doi: 10.1186/1471-2164-14-818 (PMC4046790; doi:10.1186/1471-2164-14-818)

A

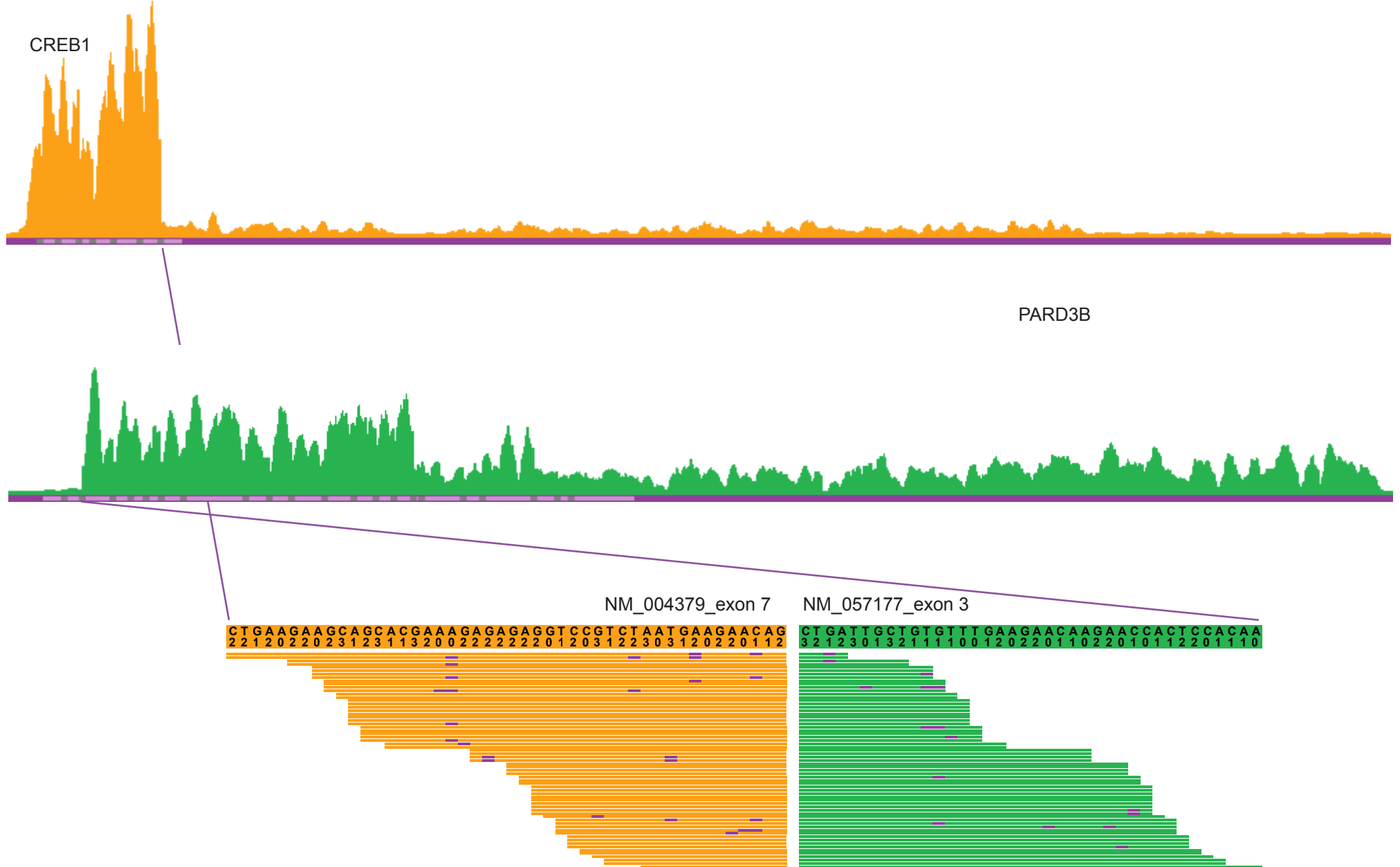

B

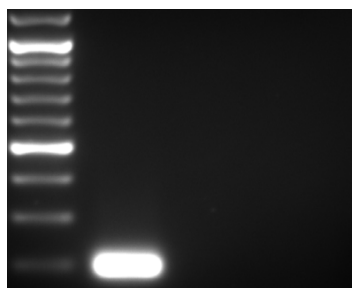

SN161 Normal GBM

C

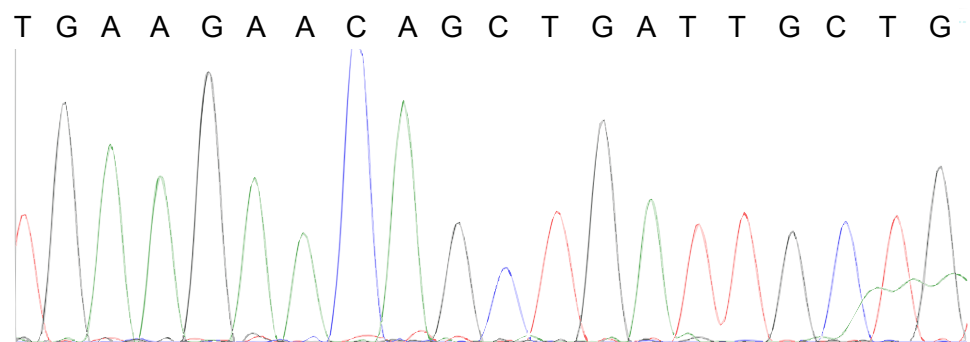

Supplement: Supplementary file 2 — Additional file 2: Contains details of Ivy Center fusions with predicted protein sequences. (GZ 9 MB) [file 12864_2013_5514_MOESM2_ESM.gz › s2/creb1-pard3b.pdf]

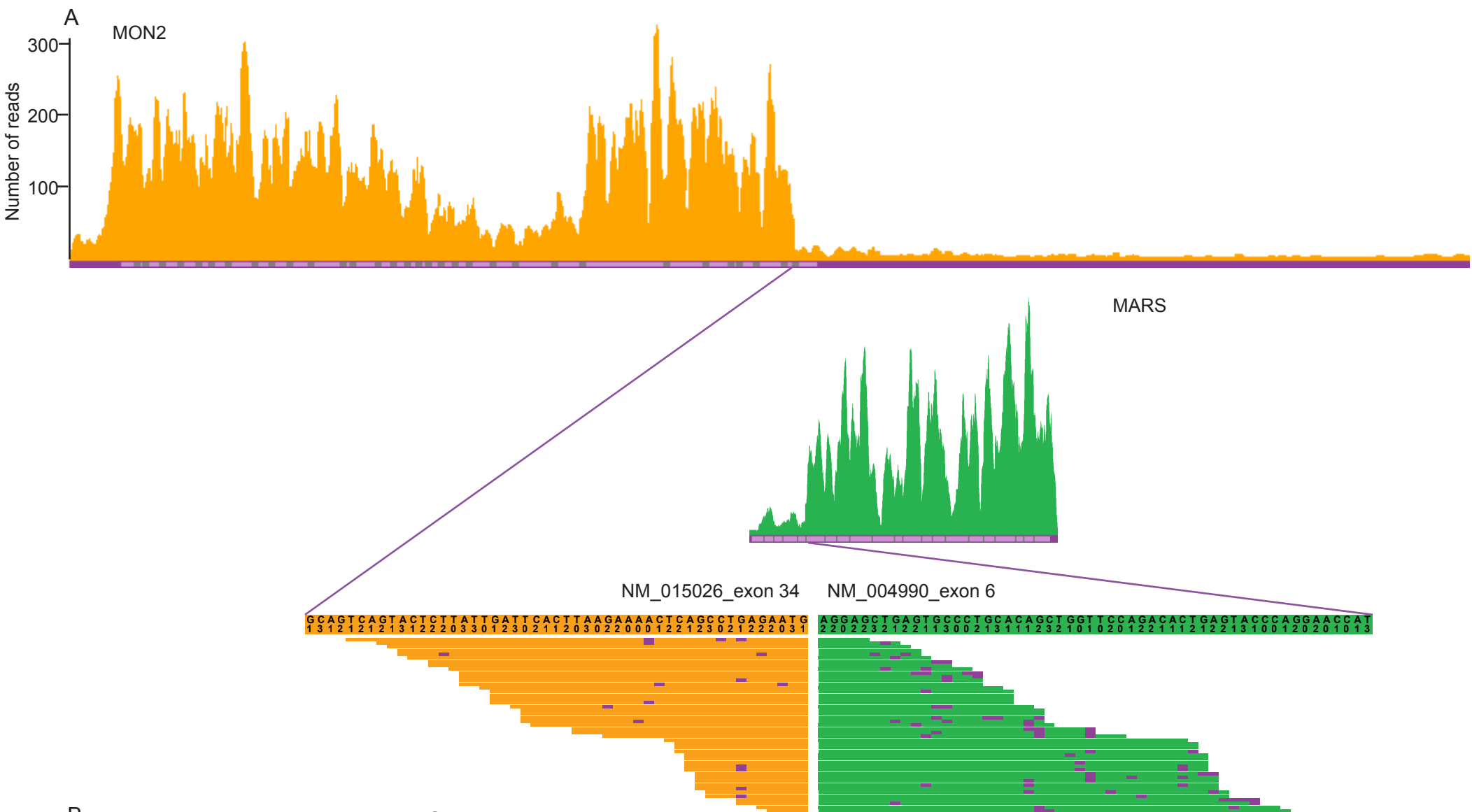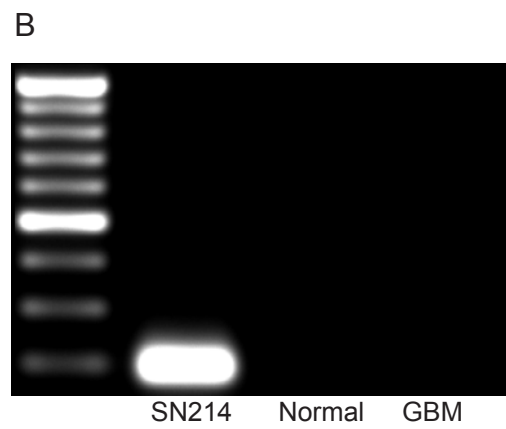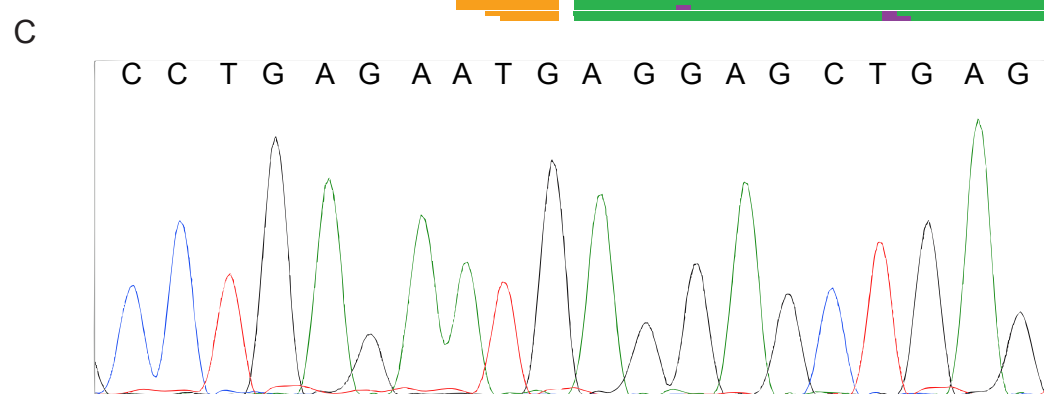

Supplement: Supplementary file 2 — Additional file 2: Contains details of Ivy Center fusions with predicted protein sequences. (GZ 9 MB) [file 12864_2013_5514_MOESM2_ESM.gz › s2/figure2_mon2-mars.pdf]

A

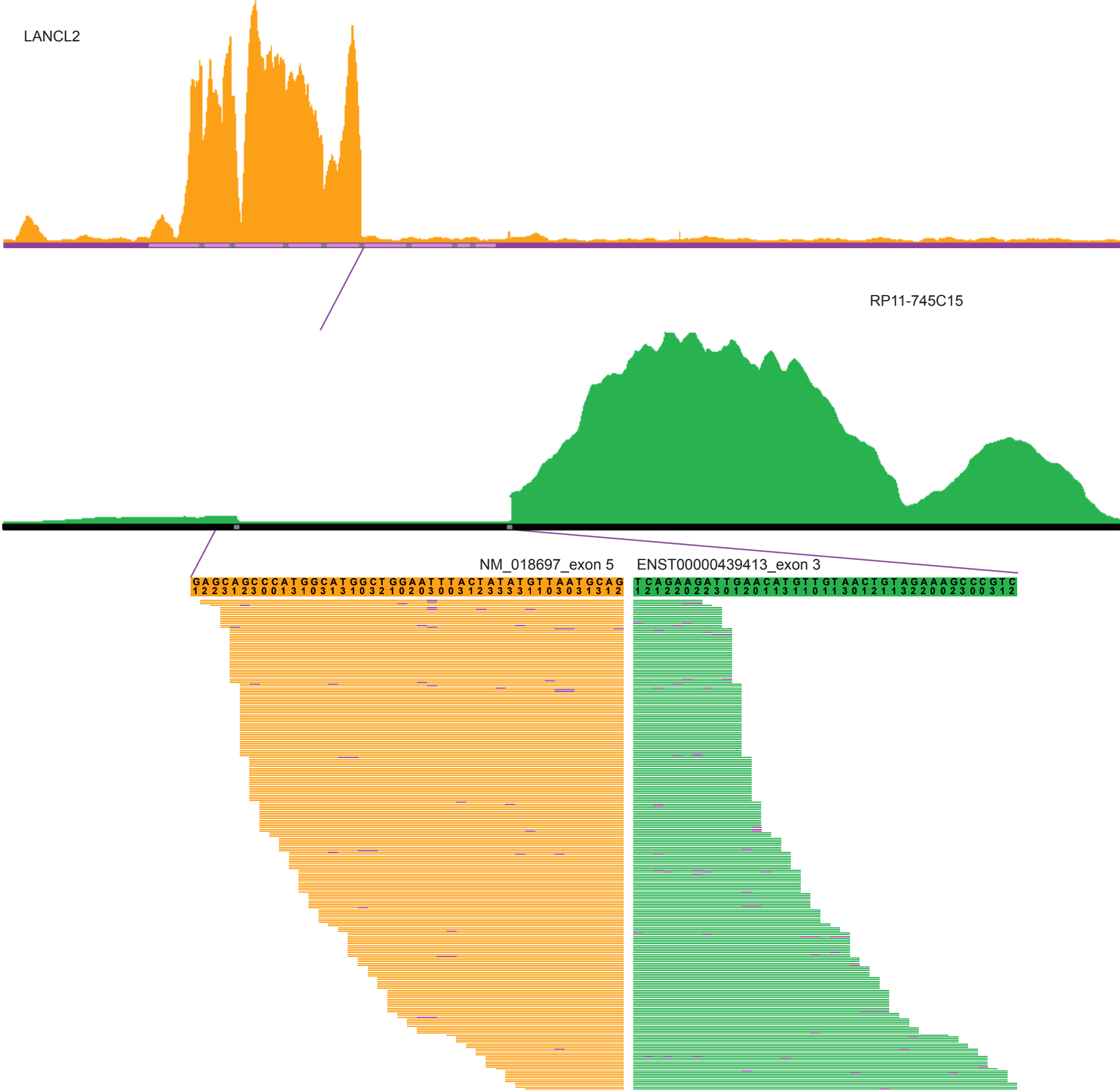

B

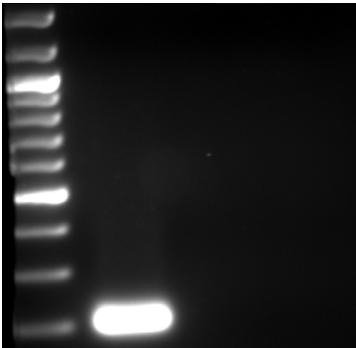

C

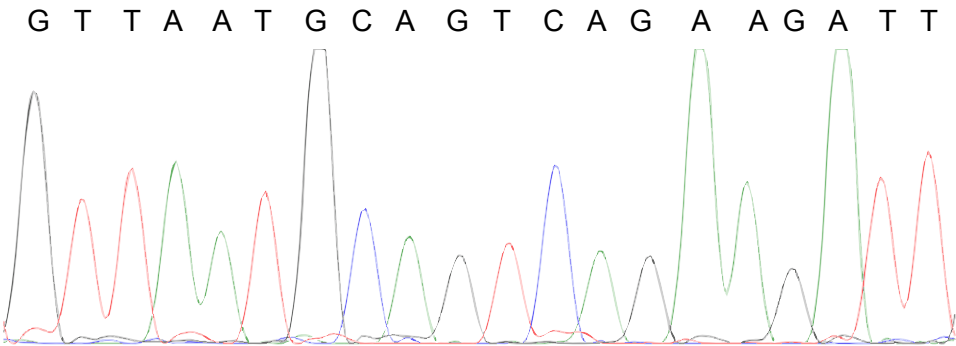

Supplement: Supplementary file 2 — Additional file 2: Contains details of Ivy Center fusions with predicted protein sequences. (GZ 9 MB) [file 12864_2013_5514_MOESM2_ESM.gz › s2/lancl2-rp11-745c15.pdf]

A

MDM1

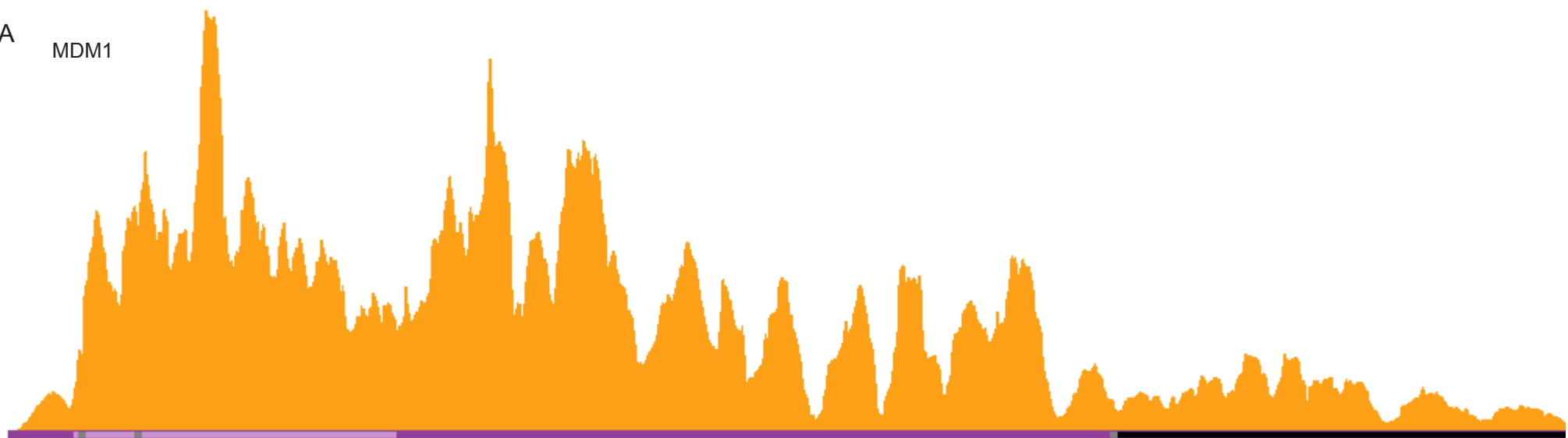unannotated  
region

NM\_020128\_3' utr chr12:68,876,024

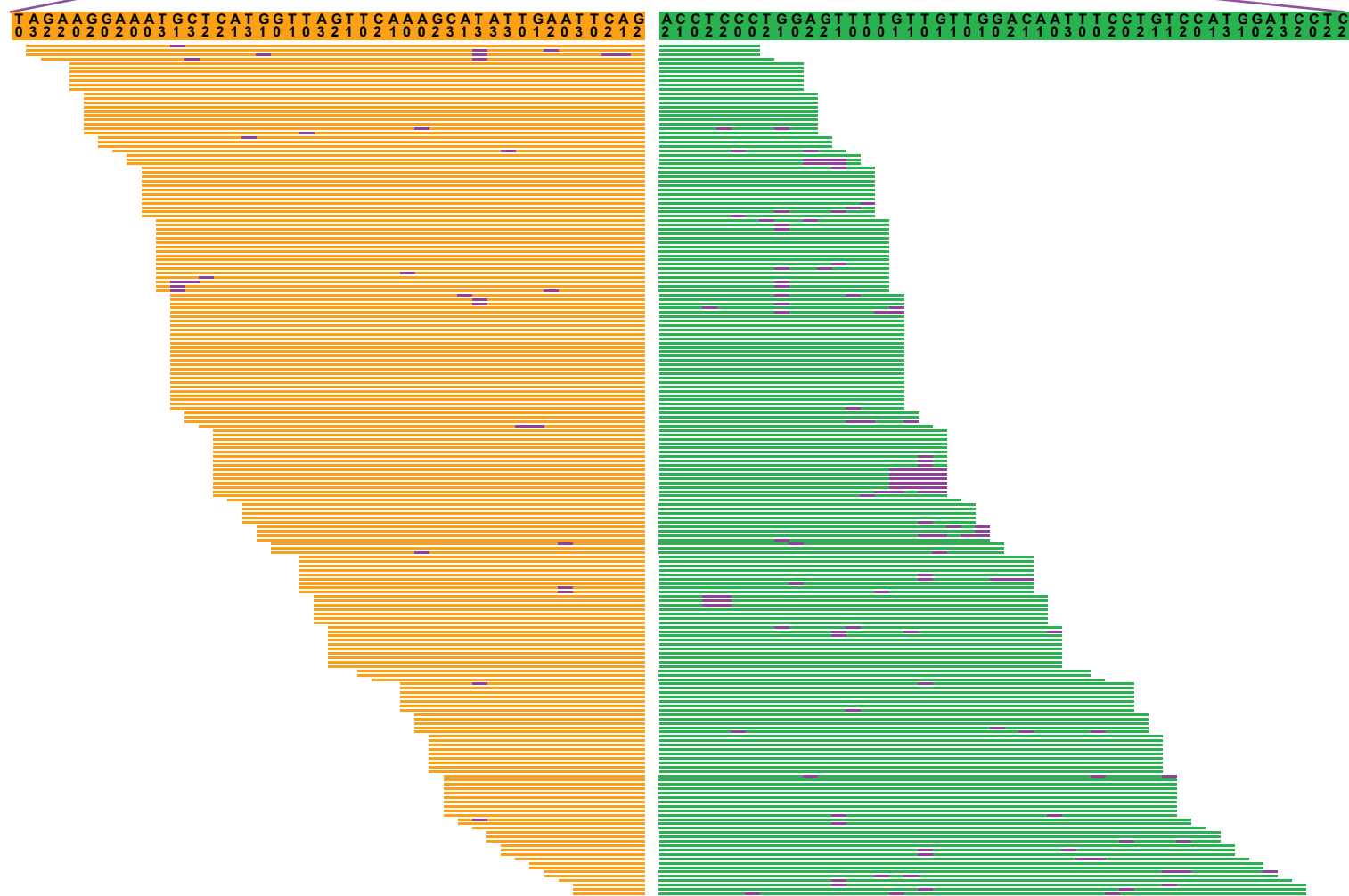

B

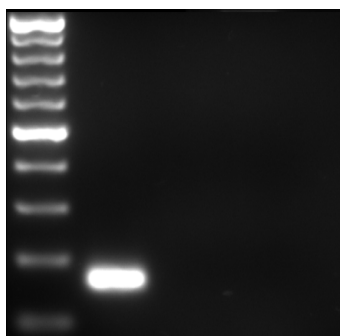

SN214 Normal GBM

C

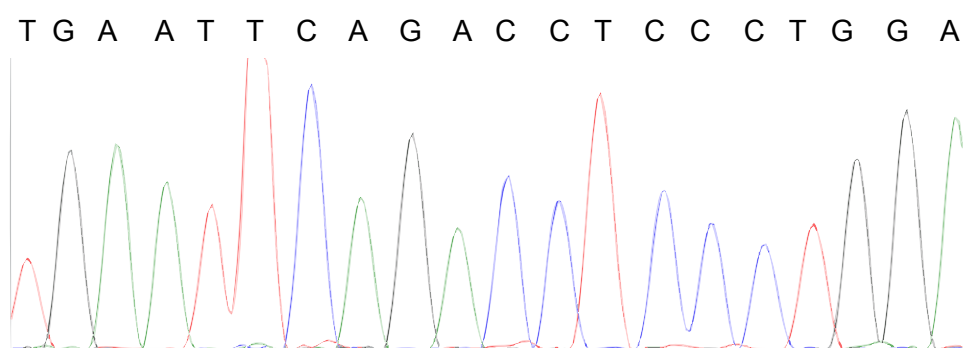

Supplement: Supplementary file 2 — Additional file 2: Contains details of Ivy Center fusions with predicted protein sequences. (GZ 9 MB) [file 12864_2013_5514_MOESM2_ESM.gz › s2/mdm1.pdf]

A

PIK3C2B

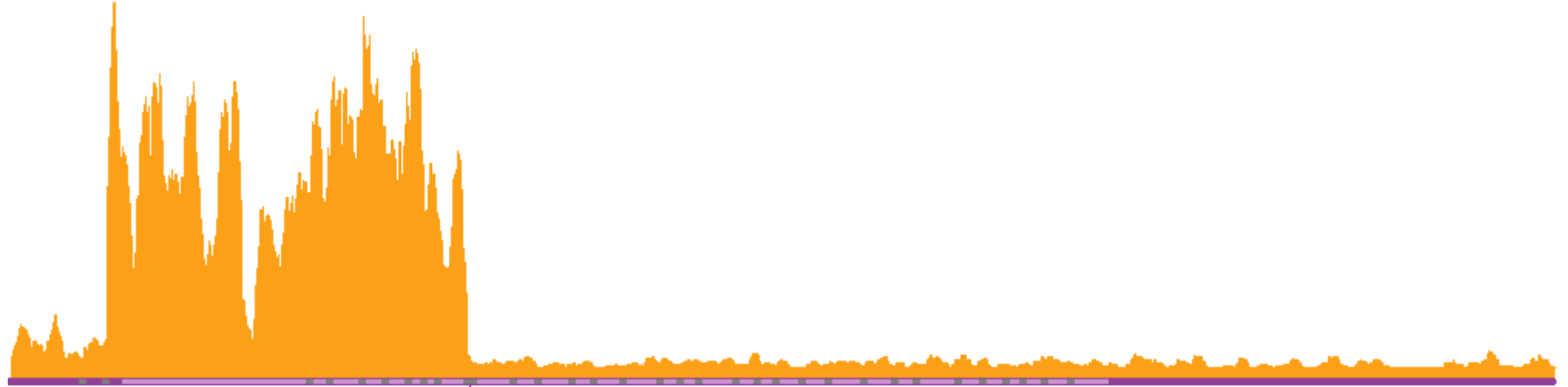

DSTYK

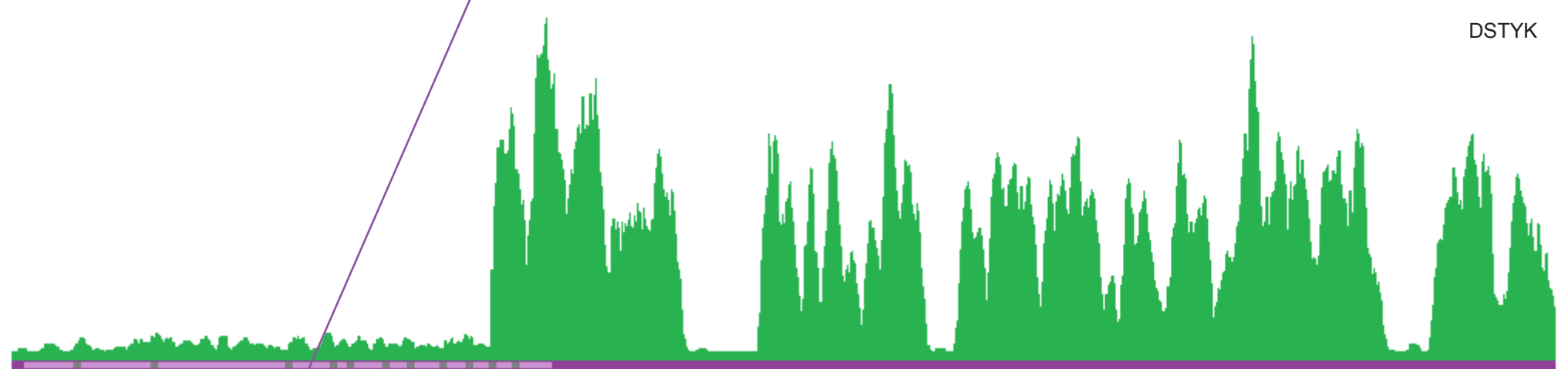

NM\_002646\_exon 10

NM\_199462\_exon 11

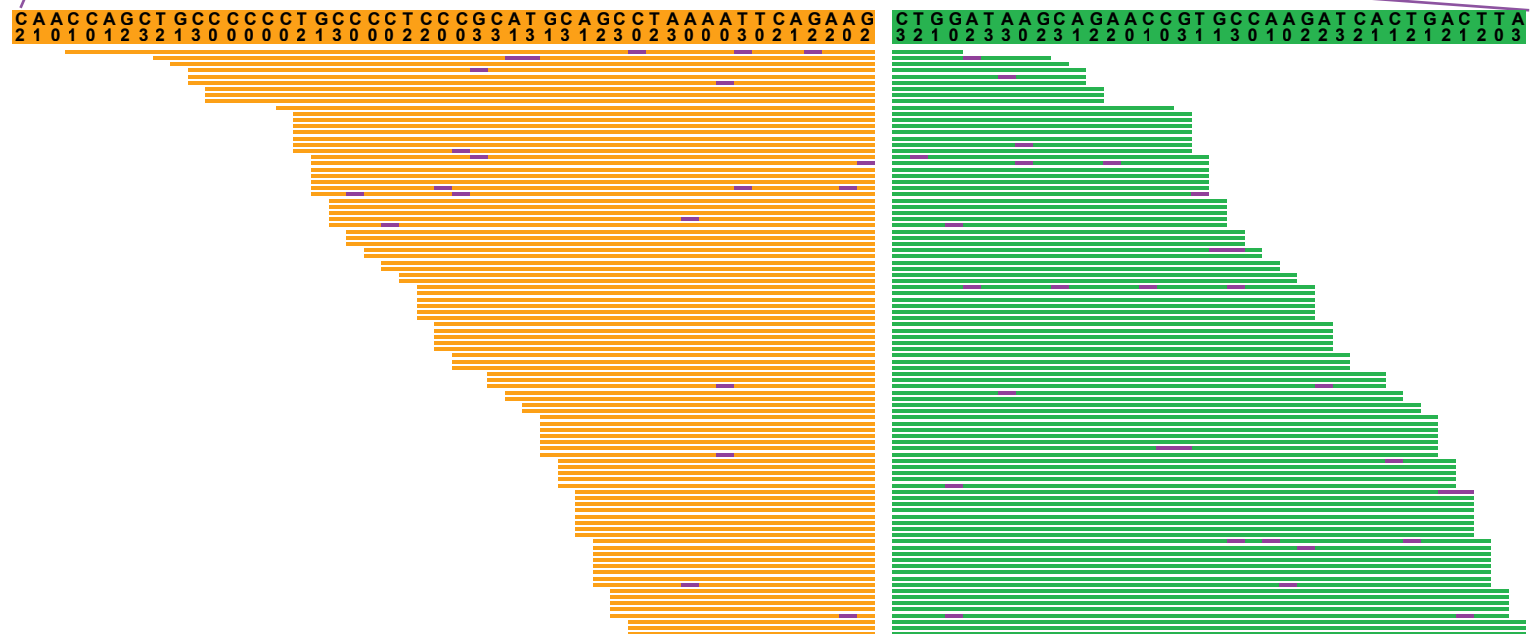

B

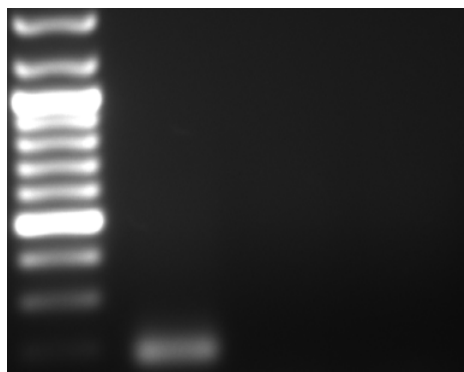

SN161 Normal GBM

C

A A T T C A G A A G C T G G A T A A G C

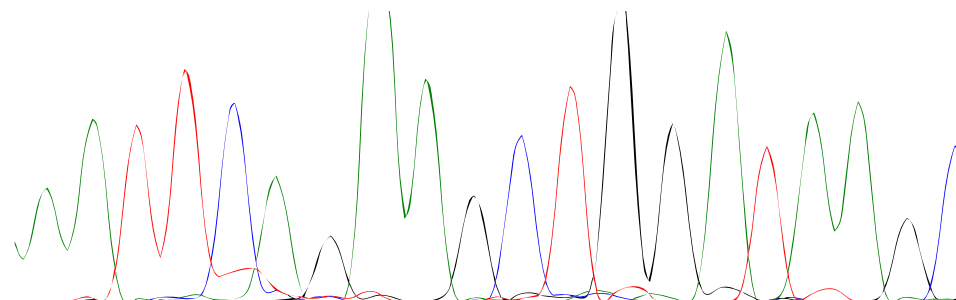

Supplement: Supplementary file 2 — Additional file 2: Contains details of Ivy Center fusions with predicted protein sequences. (GZ 9 MB) [file 12864_2013_5514_MOESM2_ESM.gz › s2/pik3c2b-dstyk.pdf]

A

PLEKHA6

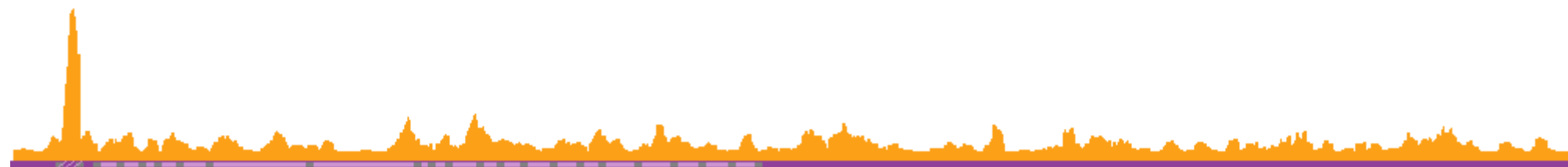

PIK3C2B

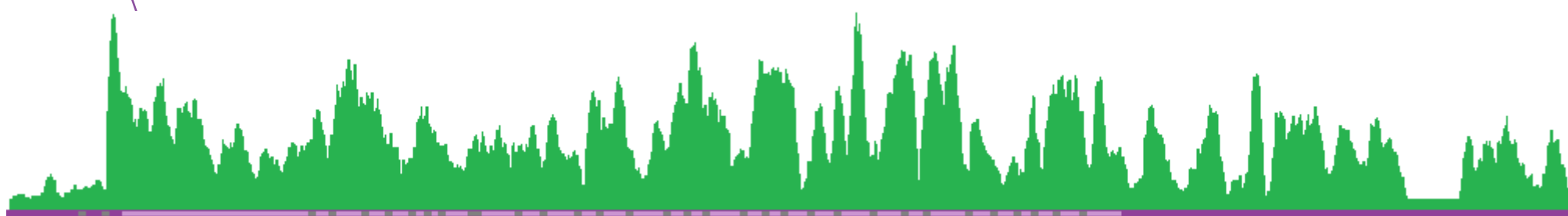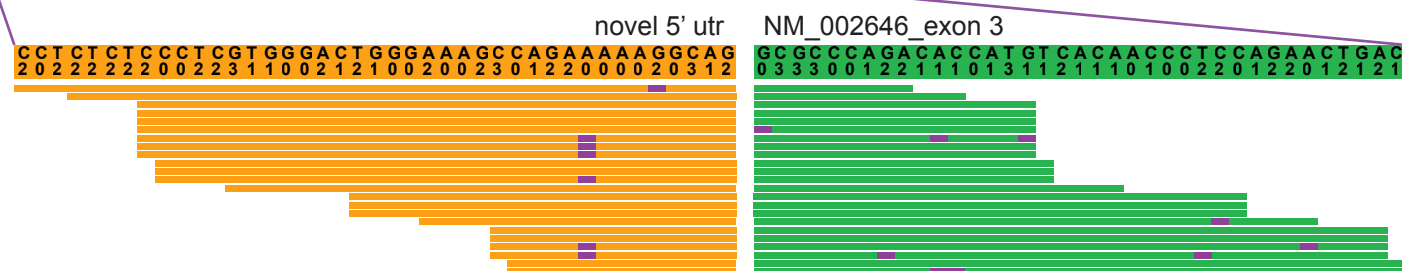

B

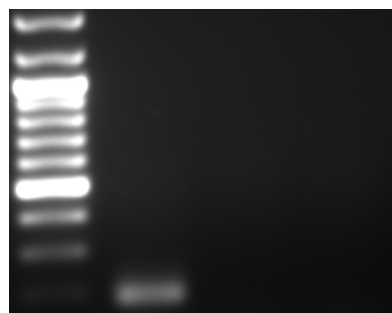

SN195 Normal GBM

C

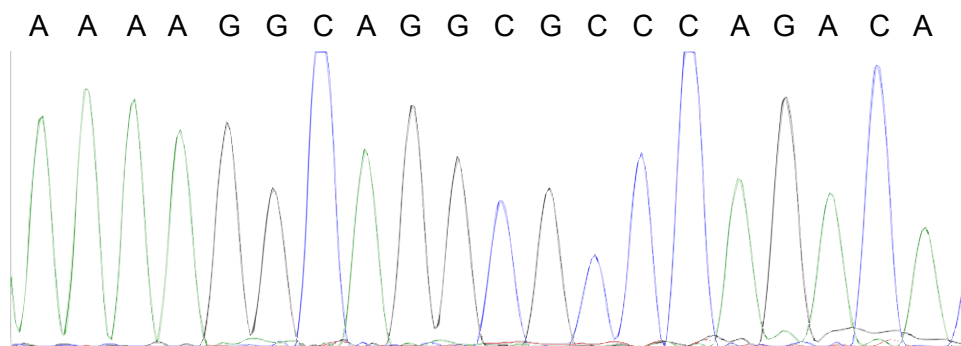

Supplement: Supplementary file 2 — Additional file 2: Contains details of Ivy Center fusions with predicted protein sequences. (GZ 9 MB) [file 12864_2013_5514_MOESM2_ESM.gz › s2/plekha6-pik3c2b.pdf]

A

SCFD2

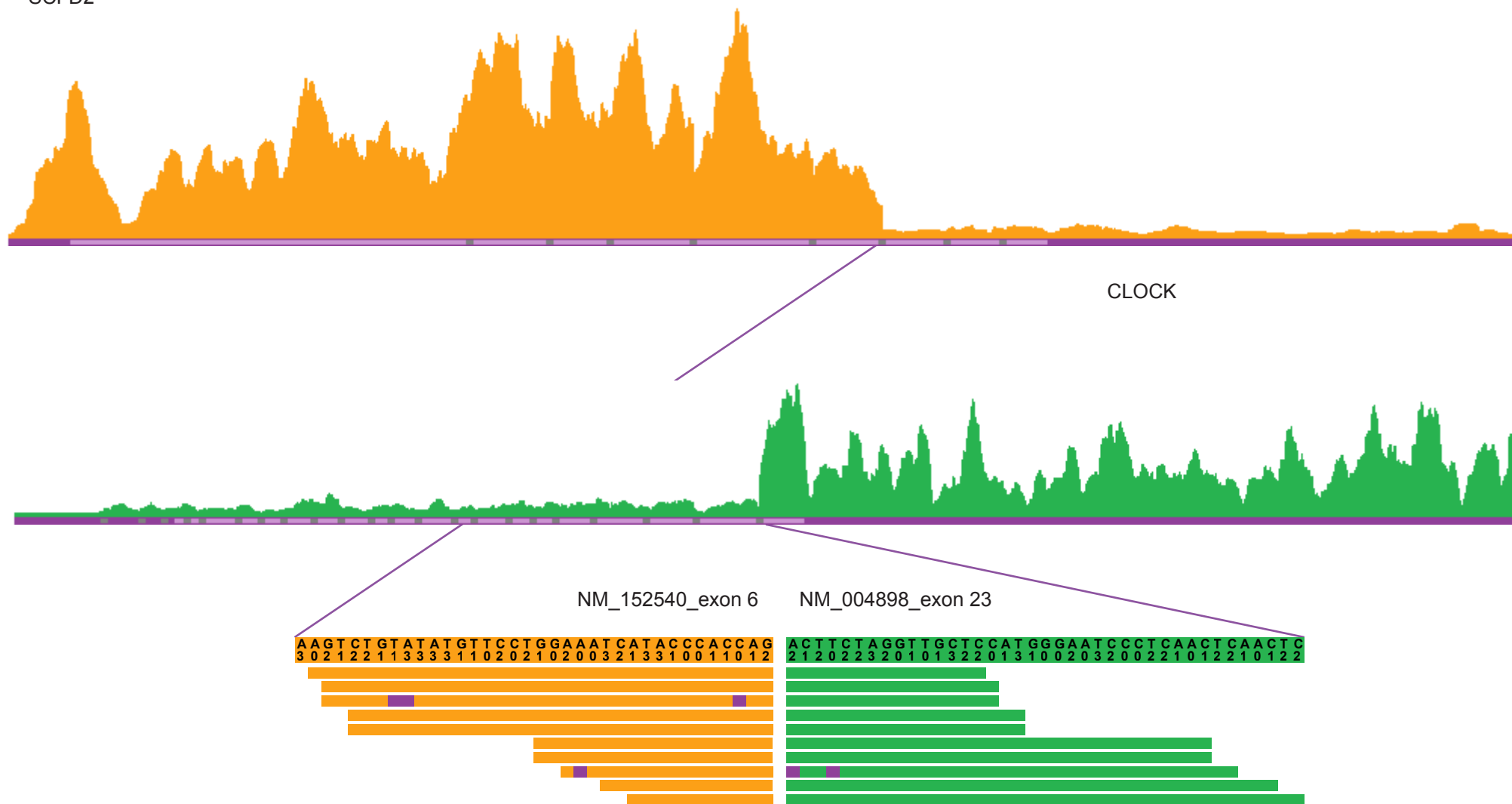

B

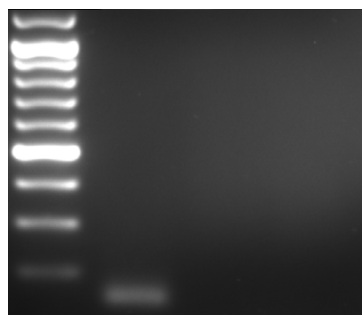

SN214 Normal GBM

C

A A T T C A G A A G C T G G A T A A G C

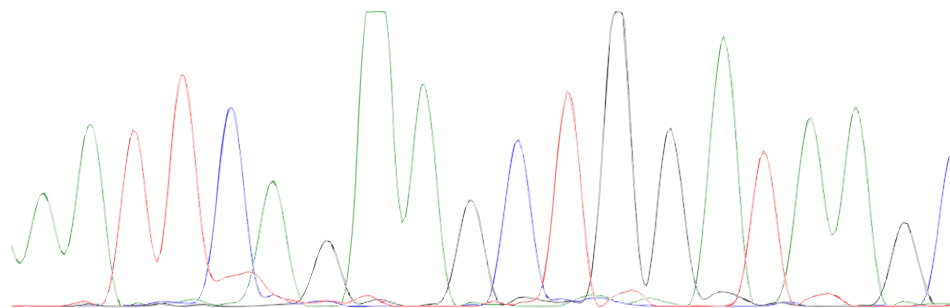

Supplement: Supplementary file 2 — Additional file 2: Contains details of Ivy Center fusions with predicted protein sequences. (GZ 9 MB) [file 12864_2013_5514_MOESM2_ESM.gz › s2/scfd2-clock.pdf]

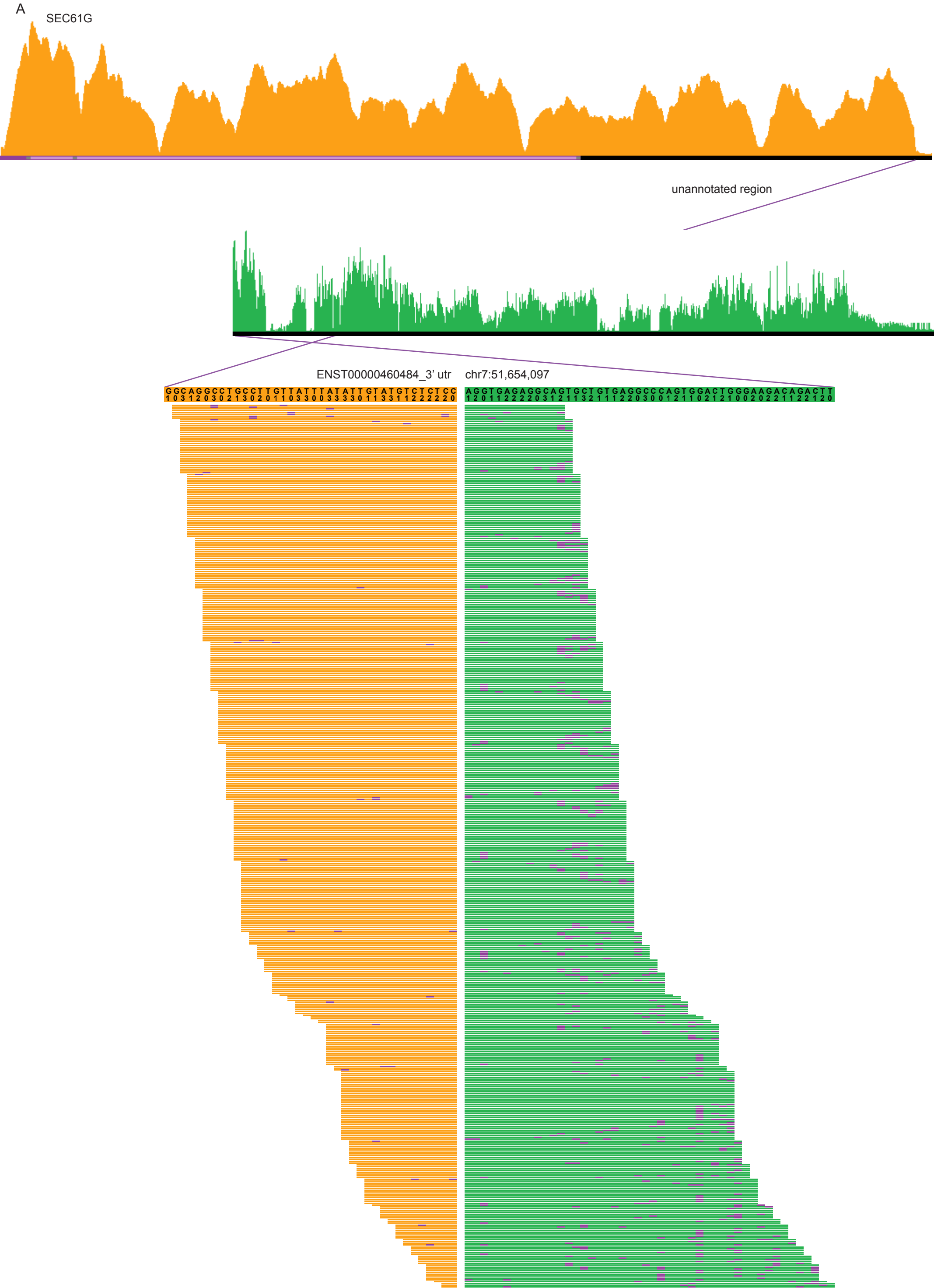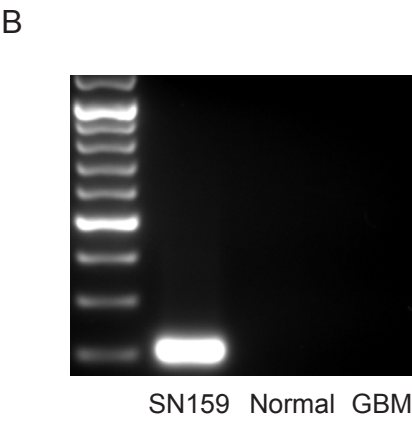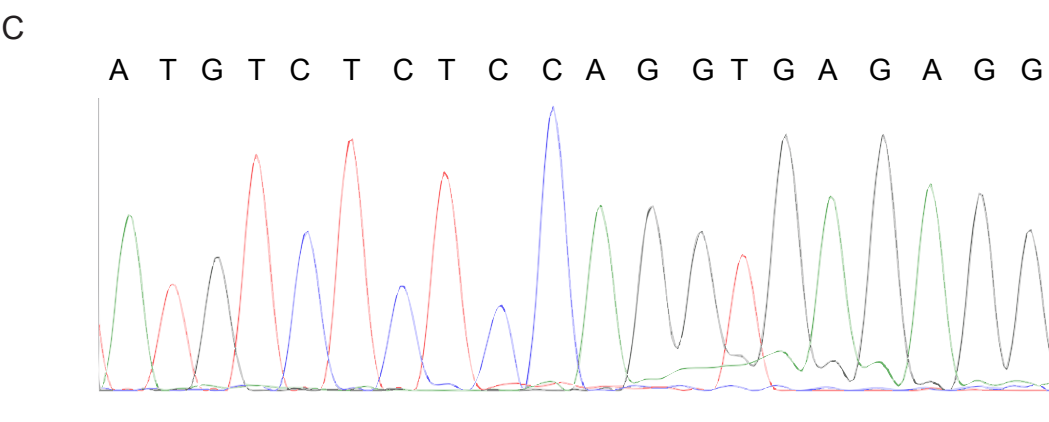

Supplement: Supplementary file 2 — Additional file 2: Contains details of Ivy Center fusions with predicted protein sequences. (GZ 9 MB) [file 12864_2013_5514_MOESM2_ESM.gz › s2/sec61g.pdf]

A

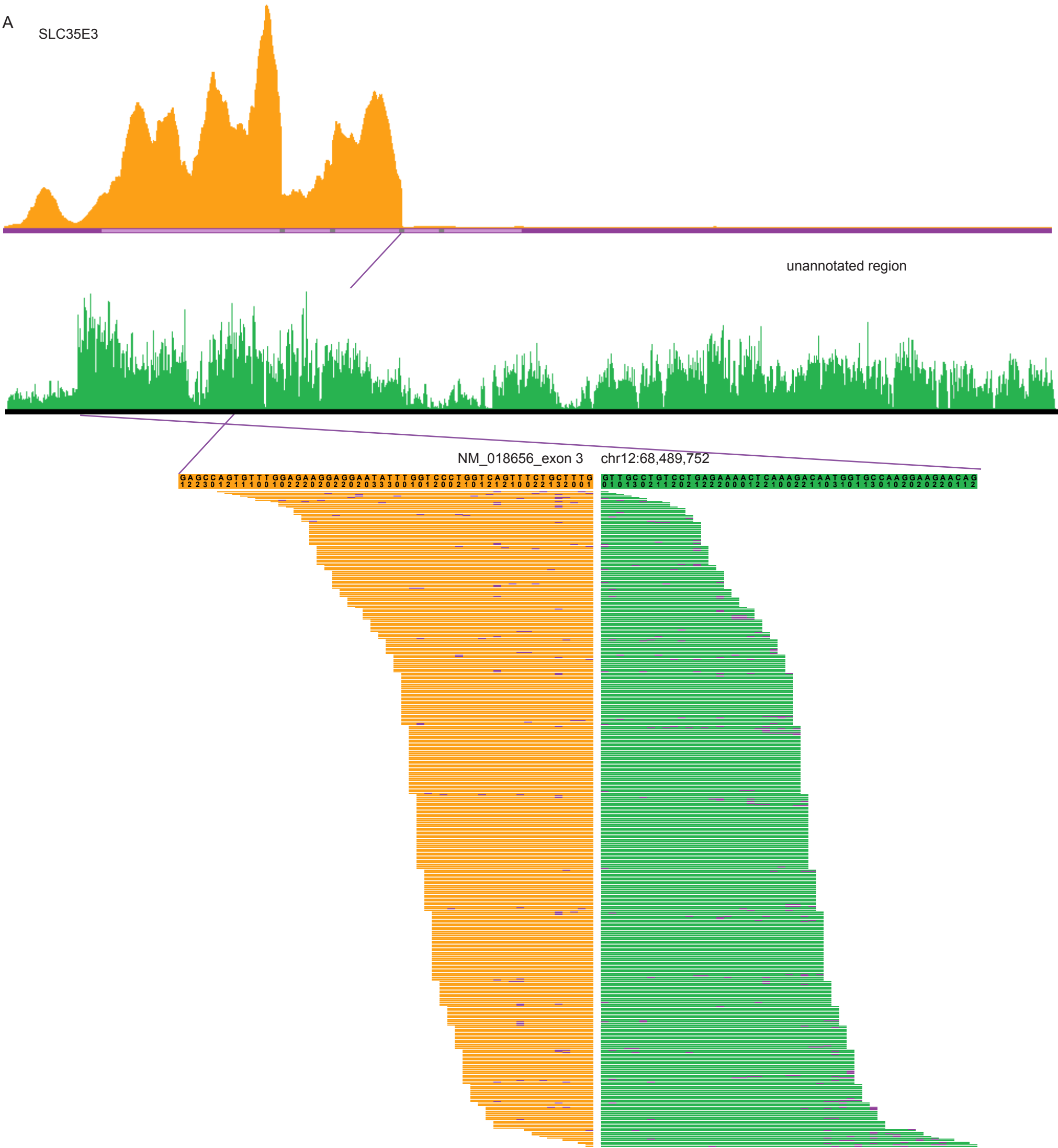

B

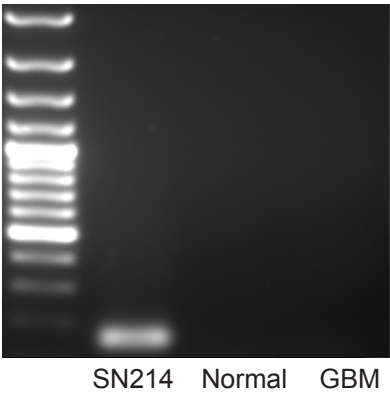

C

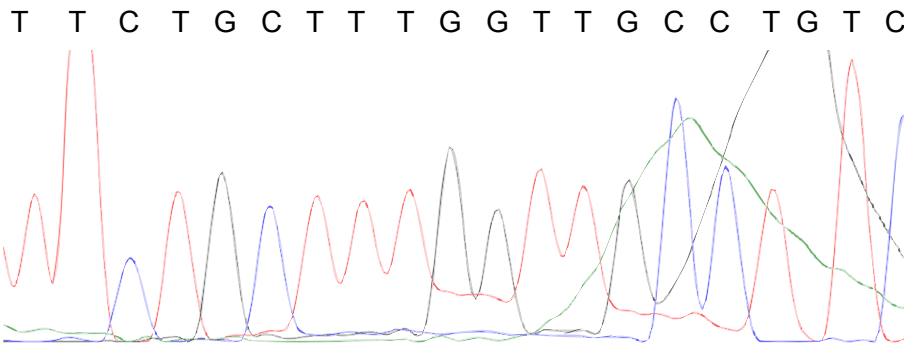

Supplement: Supplementary file 2 — Additional file 2: Contains details of Ivy Center fusions with predicted protein sequences. (GZ 9 MB) [file 12864_2013_5514_MOESM2_ESM.gz › s2/slc35e3.pdf]

A

YEATS4

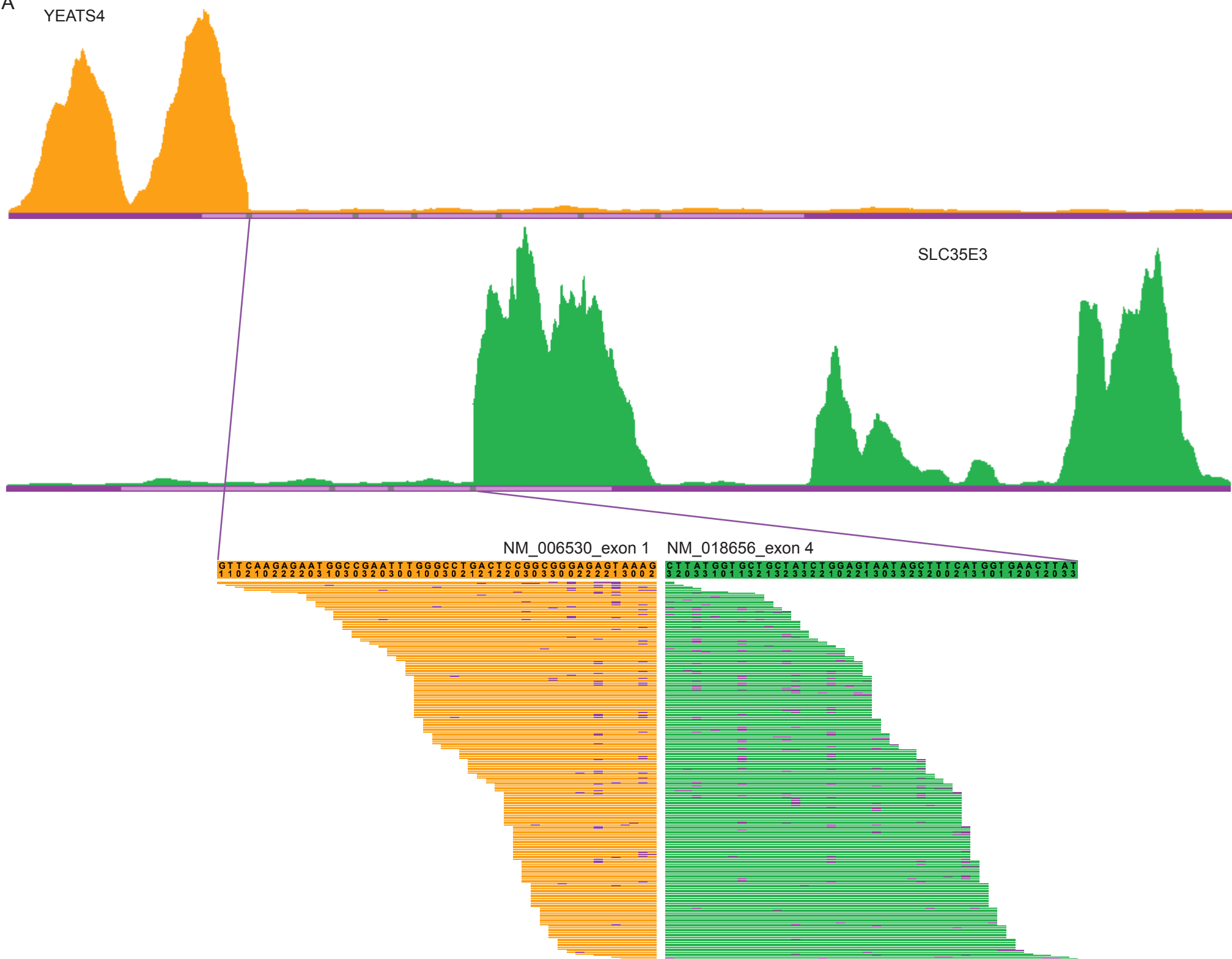

B

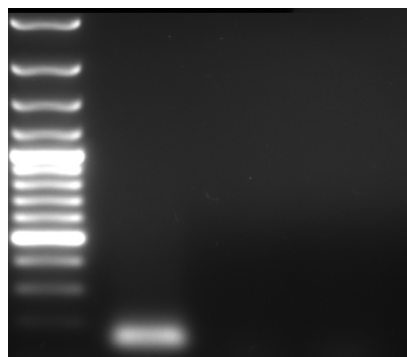

SN238 Normal GBM

C

G A G A G T A A A G C T T A T G G T G C

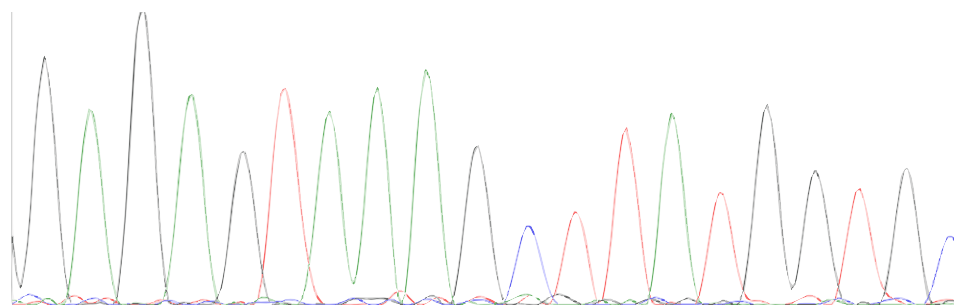

Supplement: Supplementary file 2 — Additional file 2: Contains details of Ivy Center fusions with predicted protein sequences. (GZ 9 MB) [file 12864_2013_5514_MOESM2_ESM.gz › s2/yeats4-slc35e3.pdf]

A

ZNF713

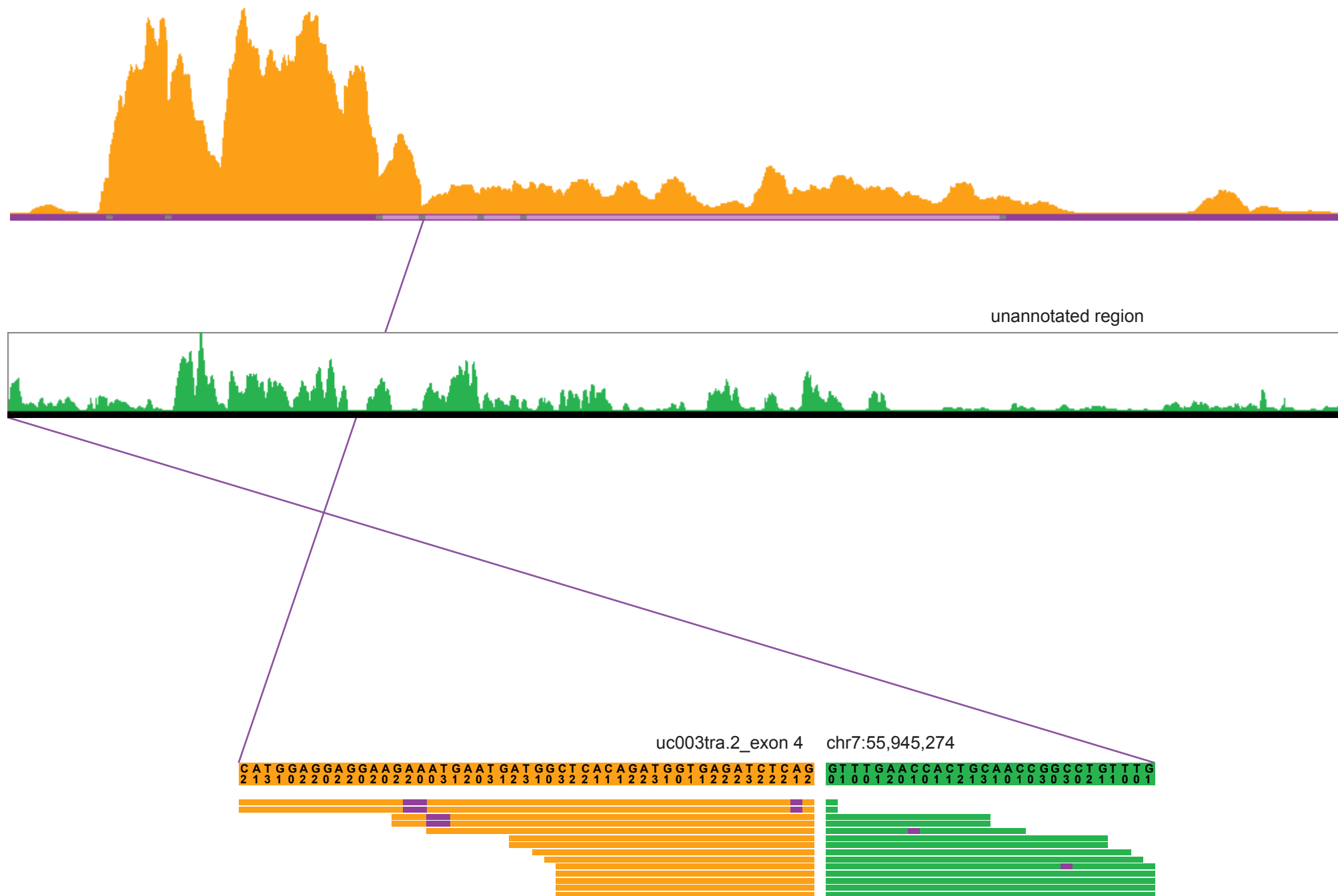

B

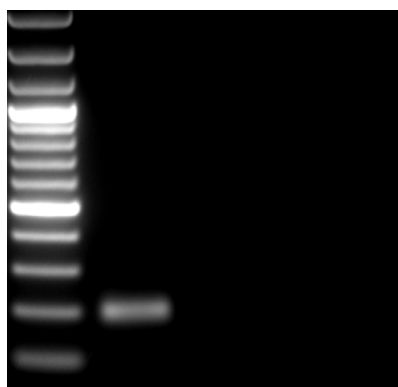

Normal GBM

C

G A G A T C T C A G G T T T G A A C C A

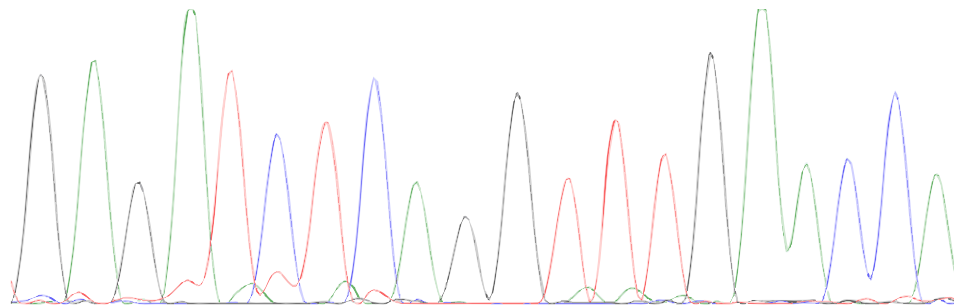

Supplement: Supplementary file 2 — Additional file 2: Contains details of Ivy Center fusions with predicted protein sequences. (GZ 9 MB) [file 12864_2013_5514_MOESM2_ESM.gz › s2/znf713_sn154.pdf]

A

ZNF713

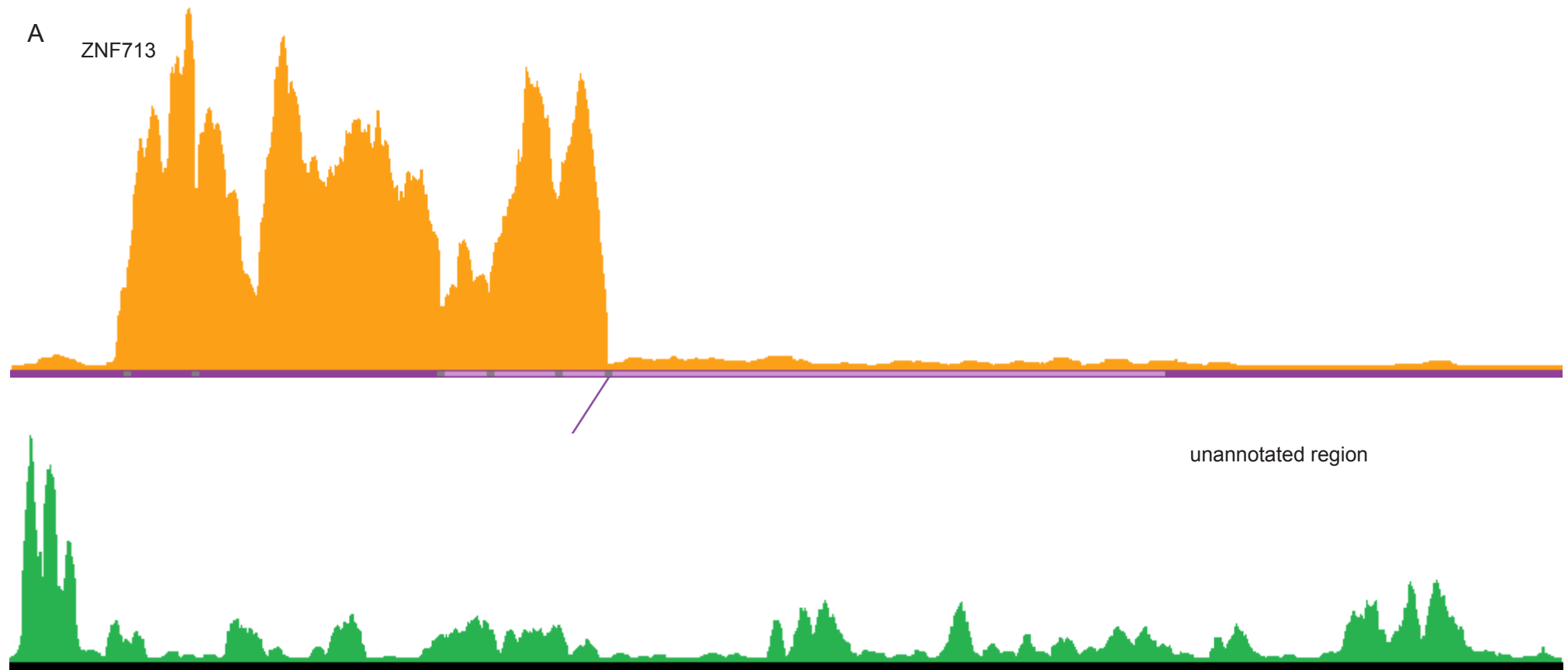

uc003tra.2\_exon 6

chr7:56,082,944

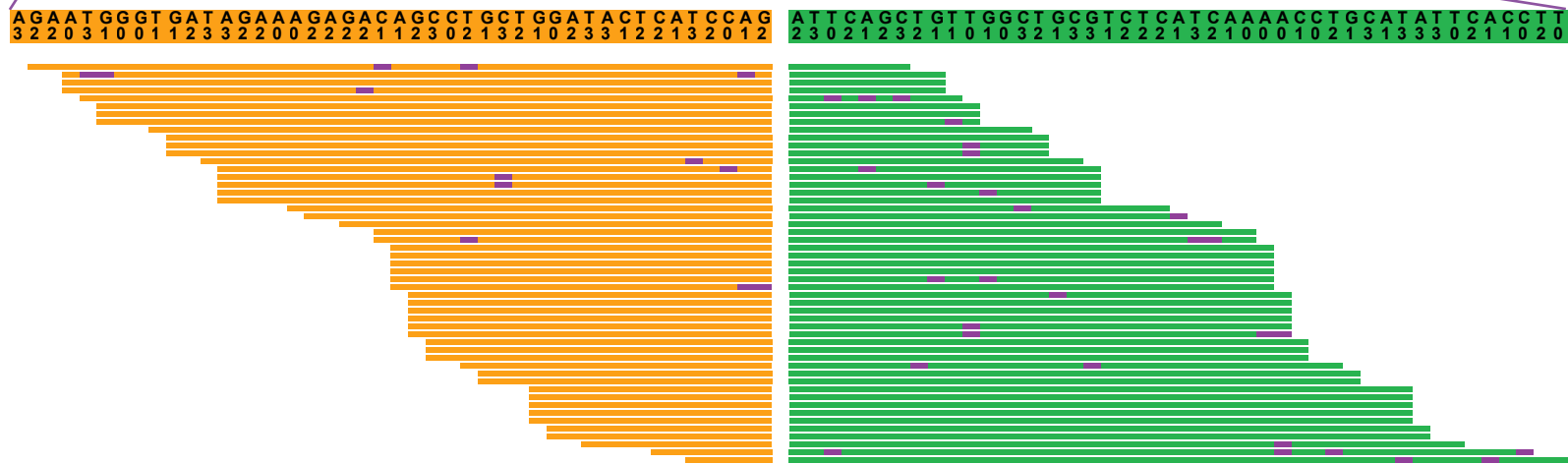

B

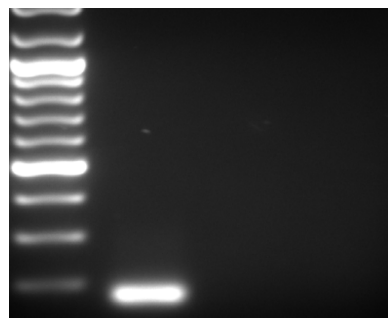

SN218 Normal GBM

C

T C A T C C A G A T T C A G C T G T T G

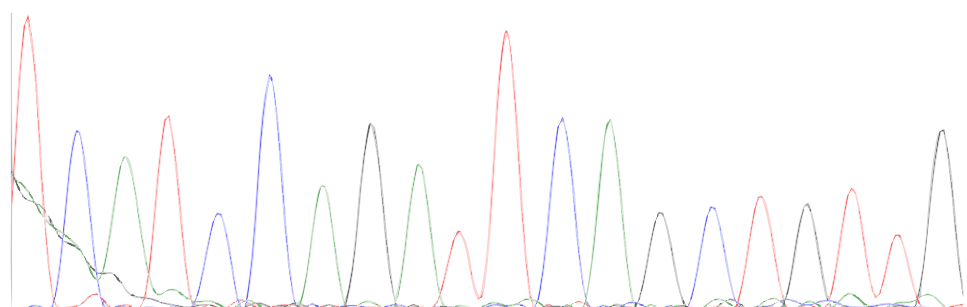

Supplement: Supplementary file 2 — Additional file 2: Contains details of Ivy Center fusions with predicted protein sequences. (GZ 9 MB) [file 12864_2013_5514_MOESM2_ESM.gz › s2/znf713_sn218.pdf]

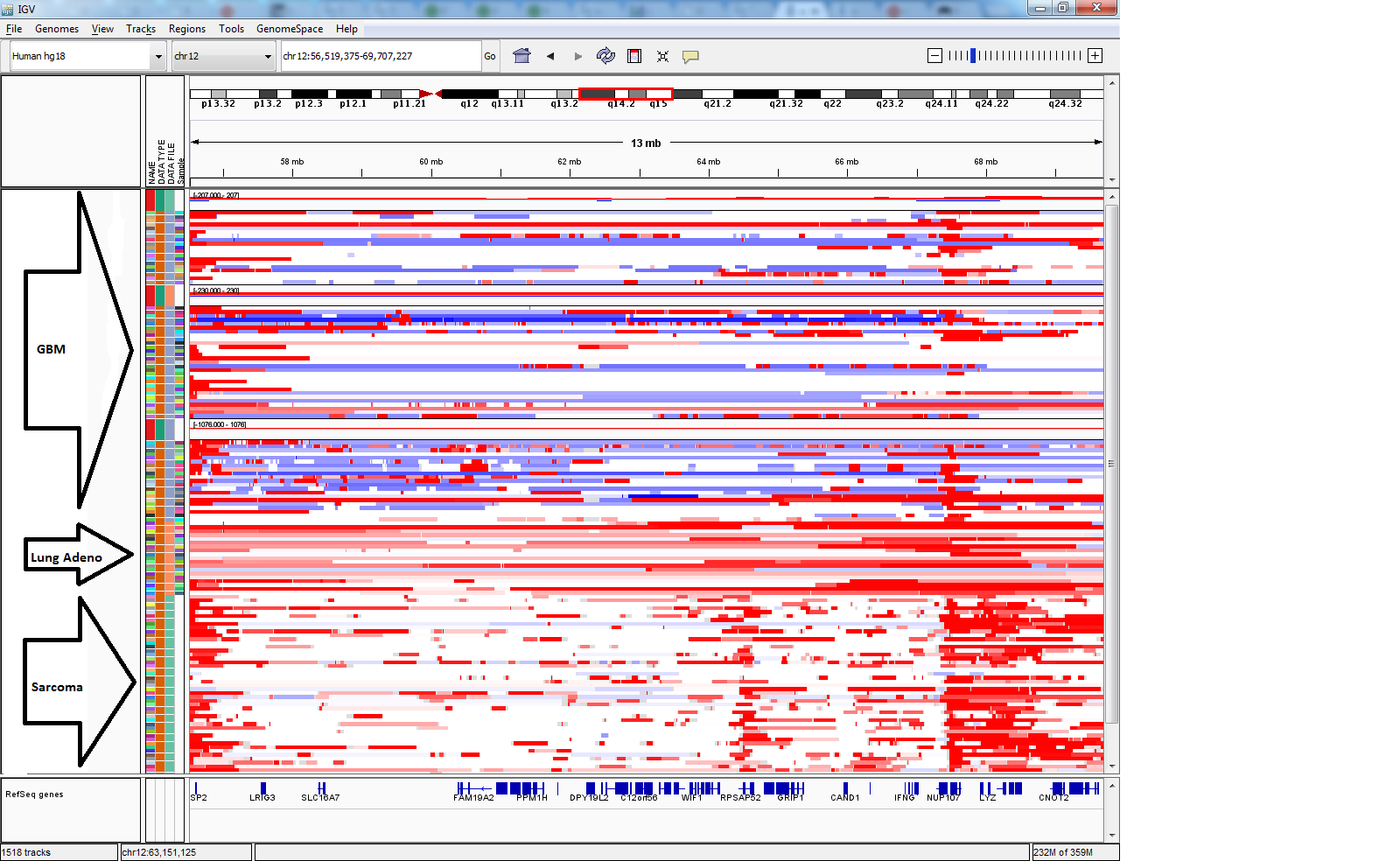

Supplement: Supplementary file 6 — Additional file 6: Is a snapshot of Integrated Genome Viewer showing genomic rearrangements on 12q14-15 in GBM, lung adenocarcinomas and sarcomas. (PNG 85 KB) [file 12864_2013_5514_MOESM6_ESM.png]
