# Supplementary material for: Exploration of the gene fusion landscape of glioblastoma using transcriptome sequencing and copy number data
Source: BMC Genomics. 2013 Nov 22;14(1):818. doi: 10.1186/1471-2164-14-818 (PMC4046790; doi:10.1186/1471-2164-14-818)
Supplement: Supplementary file 3 — Additional file 3: Contains output from TopHat software for the TCGA cohort. (GZ 5 MB) [file 12864_2013_5514_MOESM3_ESM.gz › tophat.tar!/tophatfusion_out/result.html]

 
 
 result 
 
 
 
 
 
 
 
  candidate fusion list 
The following tables show fusion candidates where fusions are grouped based on their genomic locations ( table description ) 
 sample information  
Known fusions are found at  Mitelman Database  as of Feb 21, 2012 
    
1. chr12-chr12 rr 
 
   TCGA-26-5135-01A  
 ENSG00000258231 
 chr12 
 59169742 
 SLC16A7 
 chr12 
 59991753 
  170  
  61  
 93 
 1206.86  
   TCGA-26-5135-01A  
 ENSG00000258231 
 chr12 
 59195041 
 SLC16A7 
 chr12 
 59990016 
  7  
  8  
 1 
 307.90  
   TCGA-26-5135-01A  
 ENSG00000258231 
 chr12 
 59199722 
 SLC16A7 
 chr12 
 59990016 
  32  
  41  
 33 
 471.66  
   TCGA-26-5135-01A  
 ENSG00000258231 
 chr12 
 59205703 
 SLC16A7 
 chr12 
 59990016 
  1  
  37  
 0 
 -152.71  
   TCGA-26-5135-01A  
 ENSG00000258231 
 chr12 
 59206195 
 SLC16A7 
 chr12 
 59990016 
  129  
  37  
 135 
 390.34  
 
    
2. chr3-chr9 ff 
 
   TCGA-06-5415-01A  
 FYTTD1 
 chr3 
 197502495 
 ABCA2 
 chr9 
 139909141 
  207  
  65  
 236 
 1155.10  
 
    
3. chr14-chr14 fr 
 
   TCGA-06-5413-01A  
 AKAP6 
 chr14 
 32903022 
 EGLN3 
 chr14 
 34400420 
  142  
  40  
 132 
 1119.28  
 
    
4. chr19-chr19 rr 
 
   TCGA-32-1970-01A  
 ZNF550 
 chr19 
 58063994 
 CXCL17 
 chr19 
 42938007 
  179  
  92  
 252 
 1109.54  
   TCGA-32-1970-01A  
 ZNF550 
 chr19 
 58067598 
 CXCL17 
 chr19 
 42938007 
  2  
  92  
 2 
 863.20  
 
    
5. chr2-chr2 fr 
 
   TCGA-32-2615-01A  
 ATG4B 
 chr2 
 242590749 
 DTYMK 
 chr2 
 242615651 
  92  
  42  
 96 
 1064.41  
   TCGA-32-2615-01A  
 ATG4B 
 chr2 
 242590749 
 DTYMK 
 chr2 
 242616788 
  1  
  42  
 0 
 -100.13  
   TCGA-32-2615-01A  
 ATG4B 
 chr2 
 242591157 
 DTYMK 
 chr2 
 242616794 
  3  
  42  
 4 
 400.36  
 
    
6. chr6-chr12 rr 
 
   TCGA-28-5207-01A  
 TJAP1 
 chr6 
 43473030 
 TSFM 
 chr12 
 58191066 
  165  
  68  
 213 
 1044.29  
 
    
7. chr10-chr10 rr 
 
   TCGA-12-5299-01A  
 SLIT1 
 chr10 
 98790513 
 SFRP5 
 chr10 
 99527616 
  283  
  87  
 276 
 1024.89  
 
    
8. chr12-chr12 rf 
 
   TCGA-06-2570-01A  
 ENSG00000179912 
 chr12 
 57789531 
 LRIG3 
 chr12 
 59276289 
  1  
  47  
 0 
 -1014.30  
   TCGA-06-2570-01A  
 ENSG00000179912 
 chr12 
 57789531 
 LRIG3 
 chr12 
 59278133 
  251  
  47  
 143 
 1008.77  
   TCGA-41-5651-01A  
 CTDSP2 
 chr12 
 58240154 
 ENSG00000258231 
 chr12 
 58937906 
  23  
  3  
 3 
 232.15  
 
    
9. chr12-chr12 rf 
 
   TCGA-06-0129-01A  
 EFCAB4B 
 chr12 
 3803613 
 MPHOSPH9 
 chr12 
 123702922 
  50  
  68  
 48 
 976.94  
   TCGA-06-0129-01A  
 EFCAB4B 
 chr12 
 3803613 
 MPHOSPH9 
 chr12 
 123702945 
  4  
  68  
 2 
 943.50  
 
    
10. chr4-chr4 rr 
 
   TCGA-76-4925-01A  
 TACC3 
 chr4 
 1739210 
 FGFR3 
 chr4 
 1809719 
  17  
  345  
 30 
 -286.85  
   TCGA-76-4925-01A  
 TACC3 
 chr4 
 1739324 
 FGFR3 
 chr4 
 1808660 
  631  
  345  
 560 
 972.70  
   TCGA-76-4925-01A  
 TACC3 
 chr4 
 1739324 
 FGFR3 
 chr4 
 1809010 
  9  
  345  
 9 
 646.43  
   TCGA-27-1835-01A  
 TACC3 
 chr4 
 1740074 
 FGFR3 
 chr4 
 1808660 
  164  
  7465  
 118 
 708.84  
   TCGA-14-0789-01A  
 TACC3 
 chr4 
 1741428 
 FGFR3 
 chr4 
 1808660 
  5  
  4  
 3 
 370.42  
   TCGA-27-1835-01A  
 TACC3 
 chr4 
 1741428 
 FGFR3 
 chr4 
 1808660 
  20848  
  7465  
 13644 
 772.62  
 
    
11. chr12-chr8 rf 
 
   TCGA-06-0178-01A  
 AGAP2 
 chr12 
 58123421 
 TMEM67 
 chr8 
 94827532 
  59  
  64  
 66 
 964.70  
 
    
12. chr19-chr19 rf 
 
   TCGA-06-5415-01A  
 CARD8 
 chr19 
 48752779 
 SULT2B1 
 chr19 
 49099995 
  122  
  53  
 92 
 956.66  
   TCGA-06-5415-01A  
 CARD8 
 chr19 
 48753007 
 SULT2B1 
 chr19 
 49099995 
  37  
  53  
 37 
 814.88  
 
    
13. chr12-chr12 rf 
 
   TCGA-06-5856-01A  
 LOC100506844 
 chr12 
 58325415 
 SRRM4 
 chr12 
 119574011 
  104  
  80  
 99 
 725.76  
   TCGA-06-5856-01A  
 ENSG00000166896 
 chr12 
 58335421 
 SRRM4 
 chr12 
 119583185 
  39  
  93  
 26 
 953.60  
   TCGA-06-5856-01A  
 ENSG00000166896 
 chr12 
 58335421 
 SRRM4 
 chr12 
 119588821 
  2  
  73  
 1 
 860.79  
 
    
14. chr7-chr7 ff 
 
   TCGA-06-0211-01B  
 EGFR 
 chr7 
 55151378 
 SEPT14 
 chr7 
 55886819 
  30  
  3  
 21 
 156.31  
   TCGA-28-5209-01A  
 EGFR 
 chr7 
 55268105 
 ENSG00000226278 
 chr7 
 55840873 
  10119  
  2422  
 10184 
 936.91  
   TCGA-28-5215-01A  
 EGFR 
 chr7 
 55268105 
 ENSG00000226278 
 chr7 
 55840873 
  42  
  9  
 42 
 533.63  
   TCGA-19-2624-01A  
 LANCL2 
 chr7 
 55433921 
 ENSG00000223475 
 chr7 
 55654413 
  3  
  63  
 6 
 252.35  
   TCGA-14-0817-01A  
 LANCL2 
 chr7 
 55469012 
 PSPH 
 chr7 
 56082822 
  1266  
  511  
 1356 
 741.04  
   TCGA-14-0817-01A  
 LANCL2 
 chr7 
 55469012 
 PSPH 
 chr7 
 56088795 
  56  
  500  
 85 
 609.77  
 
    
15. chr7-chr7 fr 
 
   TCGA-06-0219-01A  
 ENSG00000234707 
 chr7 
 54850035 
 EGFR 
 chr7 
 55240816 
  19  
  16  
 20 
 603.79  
   TCGA-06-0744  
 ENSG00000234707 
 chr7 
 54850035 
 EGFR 
 chr7 
 55087057 
  8  
  2  
 7 
 318.74  
   TCGA-06-0744-01A  
 ENSG00000234707 
 chr7 
 54850035 
 EGFR 
 chr7 
 55087057 
  8  
  2  
 7 
 318.74  
   TCGA-14-0790-01B  
 ENSG00000234707 
 chr7 
 54850035 
 EGFR 
 chr7 
 55268105 
  12  
  2  
 9 
 355.83  
   TCGA-06-0219-01A  
 ENSG00000234707 
 chr7 
 54860605 
 EGFR 
 chr7 
 55240816 
  68  
  47  
 66 
 929.94  
   TCGA-06-2564-01A  
 ENSG00000234707 
 chr7 
 54865084 
 EGFR 
 chr7 
 55221844 
  9  
  4  
 7 
 385.54  
 
    
16. chr4-chr4 ff 
 
   TCGA-28-5204-01A  
 TBC1D14 
 chr4 
 6996028 
 HTRA3 
 chr4 
 8288287 
  73  
  41  
 117 
 926.61  
 
    
17. chr1-chr1 rf 
 
   TCGA-32-5222-01A  
 MTOR 
 chr1 
 11313895 
 CAMTA1 
 chr1 
 6867042 
  26  
  2  
 19 
 451.74  
   TCGA-32-5222-01A  
 MTOR 
 chr1 
 11313895 
 CAMTA1 
 chr1 
 6880240 
  64  
  35  
 67 
 922.04  
   TCGA-32-5222-01A  
 MTOR 
 chr1 
 11313895 
 CAMTA1 
 chr1 
 6885151 
  1  
  35  
 1 
 706.95  
 
    
18. chr6-chr12 rr 
 
   TCGA-06-0129-01A  
 USP49 
 chr6 
 41859531 
 ENSG00000257817 
 chr12 
 115159681 
  40  
  10  
 62 
 432.13  
   TCGA-06-0129-01A  
 USP49 
 chr6 
 41861670 
 ENSG00000257817 
 chr12 
 115191648 
  98  
  456  
 156 
 667.52  
   TCGA-06-0129-01A  
 USP49 
 chr6 
 41863061 
 ENSG00000257817 
 chr12 
 115191648 
  24  
  456  
 28 
 -973.29  
   TCGA-06-0129-01A  
 MED20 
 chr6 
 41884522 
 ENSG00000257817 
 chr12 
 115191648 
  16  
  17  
 28 
 917.65  
 
    
19. chr10-chr10 rr 
 
   TCGA-02-2483-01A  
 ENSG00000166171 
 chr10 
 103336915 
 DOCK1 
 chr10 
 128799871 
  5  
  27  
 4 
 -289.27  
   TCGA-02-2483-01A  
 POLL 
 chr10 
 103340004 
 DOCK1 
 chr10 
 128799872 
  65  
  33  
 30 
 893.57  
 
    
20. chr1-chr1 rr 
 
   TCGA-06-0157-01A  
 SOX13 
 chr1 
 204082042 
 NFASC 
 chr1 
 204797909 
  39  
  14  
 52 
 53.51  
   TCGA-06-0157-01A  
 SOX13 
 chr1 
 204083448 
 NFASC 
 chr1 
 204797909 
  8  
  14  
 5 
 -47.63  
   TCGA-27-1834-01A  
 PLEKHA6 
 chr1 
 204259704 
 NFASC 
 chr1 
 204890200 
  8  
  12  
 5 
 463.62  
   TCGA-27-1834-01A  
 PLEKHA6 
 chr1 
 204261201 
 NFASC 
 chr1 
 204889867 
  4  
  12  
 0 
 36.52  
   TCGA-27-1834-01A  
 PLEKHA6 
 chr1 
 204263197 
 NFASC 
 chr1 
 204889867 
  9  
  12  
 10 
 449.34  
   TCGA-27-1834-01A  
 PLEKHA6 
 chr1 
 204298341 
 NFASC 
 chr1 
 204889867 
  3  
  9  
 1 
 411.50  
   TCGA-27-1834-01A  
 PLEKHA6 
 chr1 
 204298806 
 NFASC 
 chr1 
 204889867 
  4  
  9  
 4 
 427.65  
   TCGA-27-1834-01A  
 PLEKHA6 
 chr1 
 204300421 
 NFASC 
 chr1 
 204889867 
  32  
  9  
 12 
 547.22  
   TCGA-27-1834-01A  
 PLEKHA6 
 chr1 
 204319491 
 NFASC 
 chr1 
 204889867 
  102  
  35  
 55 
 889.79  
 
    
21. chr9-chr9 rf 
 
   TCGA-06-0129-01A  
 ENSG00000227531 
 chr9 
 113998287 
 ZFP37 
 chr9 
 115811641 
  257  
  104  
 226 
 887.54  
 
    
22. chr7-chr7 ff 
 
   TCGA-27-2528-01A  
 ENSG00000106066 
 chr7 
 29186574 
 TSPAN33 
 chr7 
 128801518 
  161  
  81  
 248 
 880.92  
 
    
23. chr12-chr12 fr 
 
   TCGA-06-0129-01A  
 DIP2B 
 chr12 
 51128912 
 RAB3IP 
 chr12 
 70179327 
  33  
  52  
 35 
 479.96  
   TCGA-06-0129-01A  
 DIP2B 
 chr12 
 51130429 
 RAB3IP 
 chr12 
 70180389 
  63  
  52  
 57 
 879.83  
 
    
24. chr1-chr1 rr 
 
   TCGA-06-5411-01A  
 NTRK1 
 chr1 
 156844167 
 NFASC 
 chr1 
 204951824 
  7  
  238  
 5 
 -195.75  
   TCGA-06-5411-01A  
 NTRK1 
 chr1 
 156844362 
 NFASC 
 chr1 
 204951147 
  901  
  238  
 541 
 866.46  
 
    
25. chr1-chr1 rr 
 
   TCGA-26-5136-01B  
 CNN3 
 chr1 
 95392393 
 ALG14 
 chr1 
 95448861 
  50  
  37  
 40 
 862.60  
   TCGA-26-5136-01B  
 CNN3 
 chr1 
 95392393 
 ALG14 
 chr1 
 95449188 
  1  
  37  
 1 
 106.32  
 
    
26. chr19-chr19 rr 
 
   TCGA-06-0125-02A  
 SLC39A3 
 chr19 
 2737045 
 SGTA 
 chr19 
 2765282 
  17  
  8  
 12 
 848.97  
   TCGA-06-0125-02A  
 SLC39A3 
 chr19 
 2737045 
 SGTA 
 chr19 
 2765325 
  2  
  8  
 0 
 790.16  
   TCGA-06-0125-01A  
 SLC39A3 
 chr19 
 2737045 
 SGTA 
 chr19 
 2765282 
  29  
  14  
 10 
 845.45  
 
    
27. chr7-chr7 rr 
 
   TCGA-14-1823-01A  
 ENSG00000242870 
 chr7 
 91456728 
 VOPP1 
 chr7 
 55540737 
  12  
  5  
 14 
 582.37  
   TCGA-14-1823-01A  
 MTERF 
 chr7 
 91509368 
 VOPP1 
 chr7 
 55540737 
  14  
  11  
 14 
 848.01  
   TCGA-14-1823-01A  
 MTERF 
 chr7 
 91509970 
 VOPP1 
 chr7 
 55540737 
  34  
  11  
 20 
 -1136.33  
 
    
28. chr5-chr1 rr 
 
   TCGA-41-4097-01A  
 PPP2R2B 
 chr5 
 146017813 
 CCT3 
 chr1 
 156294876 
  3  
  14  
 4 
 785.50  
   TCGA-41-4097-01A  
 PPP2R2B 
 chr5 
 146017813 
 CCT3 
 chr1 
 156294879 
  33  
  14  
 50 
 847.88  
 
    
29. chr12-chr12 fr 
 
   TCGA-06-5856-01A  
 SETD1B 
 chr12 
 122248740 
 KDM2B 
 chr12 
 122012497 
  18  
  31  
 6 
 458.73  
   TCGA-06-5856-01A  
 SETD1B 
 chr12 
 122248740 
 KDM2B 
 chr12 
 122013763 
  125  
  31  
 88 
 833.40  
   TCGA-06-5856-01A  
 MLXIP 
 chr12 
 122600253 
 KDM2B 
 chr12 
 121966103 
  7178  
  496  
 3097 
 74.07  
 
    
30. chr12-chr12 fr 
 
   TCGA-06-5856-01A  
 MARCH9 
 chr12 
 58150777 
 SUDS3 
 chr12 
 118830686 
  1083  
  412  
 1170 
 827.37  
 
    
31. chr12-chr12 ff 
 
   TCGA-06-5856-01A  
 CCDC60 
 chr12 
 119826132 
 KDM2B 
 chr12 
 122012451 
  64  
  9  
 88 
 816.70  
   TCGA-06-5856-01A  
 CCDC60 
 chr12 
 119840234 
 KDM2B 
 chr12 
 122012451 
  7  
  3  
 9 
 392.98  
   TCGA-06-5856-01A  
 CCDC60 
 chr12 
 119841629 
 KDM2B 
 chr12 
 122012451 
  8  
  3  
 4 
 362.75  
   TCGA-06-5856-01A  
 CCDC60 
 chr12 
 119873809 
 KDM2B 
 chr12 
 122012451 
  24  
  3  
 32 
 263.39  
 
    
32. chr14-chr14 rf 
 
   TCGA-12-3653-01A  
 HNRNPC 
 chr14 
 21698475 
 KLHL28 
 chr14 
 45400546 
  48  
  10  
 17 
 367.95  
   TCGA-12-3653-01A  
 HNRNPC 
 chr14 
 21698477 
 KLHL28 
 chr14 
 45395485 
  12  
  10  
 10 
 231.02  
   TCGA-12-3653-01A  
 HNRNPC 
 chr14 
 21698477 
 KLHL28 
 chr14 
 45414926 
  142  
  20  
 85 
 813.19  
 
    
33. chr13-chr13 rr 
 
   TCGA-06-5856-01A  
 PSPC1 
 chr13 
 20304378 
 CRYL1 
 chr13 
 20987525 
  60  
  20  
 19 
 810.87  
 
    
34. chr3-chr3 rr 
 
   TCGA-14-2554-01A  
 CAMP 
 chr3 
 48265843 
 C3orf71 
 chr3 
 48956430 
  9  
  51  
 10 
 92.87  
   TCGA-14-2554-01A  
 CAMP 
 chr3 
 48265843 
 ARIH2 
 chr3 
 48960243 
  3  
  51  
 0 
 600.89  
   TCGA-14-2554-01A  
 CAMP 
 chr3 
 48265843 
 ARIH2 
 chr3 
 48965245 
  97  
  51  
 64 
 809.06  
 
    
35. chr9-chr16 rf 
 
   TCGA-02-2483-01A  
 ASTN2 
 chr9 
 119976636 
 ARHGDIG 
 chr16 
 331745 
  12  
  24  
 17 
 649.46  
   TCGA-02-2483-01A  
 ASTN2 
 chr9 
 119976636 
 ARHGDIG 
 chr16 
 332024 
  39  
  24  
 38 
 808.75  
 
    
36. chr2-chr2 rr 
 
   TCGA-76-4929-01A  
 ADAM17 
 chr2 
 9675962 
 YWHAQ 
 chr2 
 9731643 
  197  
  93  
 145 
 806.28  
 
    
37. chr5-chr5 rr 
 
   TCGA-06-5856-01A  
 LIFR 
 chr5 
 38496483 
 ENSG00000249740 
 chr5 
 38744118 
  88  
  22  
 71 
 804.82  
 
    
38. chr7-chr7 fr 
 
   TCGA-06-2557-01A  
 SDK1 
 chr7 
 4298755 
 EGFR 
 chr7 
 55268105 
  708  
  228  
 624 
 801.47  
   TCGA-06-2557-01A  
 SDK1 
 chr7 
 4300566 
 EGFR 
 chr7 
 55268105 
  9  
  228  
 15 
 610.79  
   TCGA-06-2557-01A  
 SDK1 
 chr7 
 4303082 
 EGFR 
 chr7 
 55268363 
  43  
  228  
 37 
 432.72  
 
    
39. chr7-chr7 ff 
 
   TCGA-28-1747-01C  
 SP4 
 chr7 
 21468409 
 GPNMB 
 chr7 
 23313139 
  4  
  81  
 5 
 309.40  
   TCGA-28-1747-01C  
 SP4 
 chr7 
 21469209 
 GPNMB 
 chr7 
 23313139 
  149  
  81  
 208 
 800.82  
 
    
40. chr16-chr16 rf 
 
   TCGA-06-5856-01A  
 LUC7L 
 chr16 
 249060 
 LOC652276 
 chr16 
 2663960 
  16  
  2  
 13 
 693.28  
   TCGA-06-5856-01A  
 LUC7L 
 chr16 
 249060 
 KCTD5 
 chr16 
 2745935 
  16  
  2  
 13 
 786.98  
 
    
41. chr12-chr12 ff 
 
   TCGA-26-5132-01A  
 PDZRN4 
 chr12 
 41949561 
 ENSG00000257747 
 chr12 
 82348026 
  215  
  56  
 364 
 785.40  
 
    
42. chr12-chr12 rr 
 
   TCGA-06-0686-01A  
 ENSG00000256248 
 chr12 
 67302585 
 NUP107 
 chr12 
 69094632 
  15  
  110  
 8 
 657.25  
   TCGA-06-0686-01A  
 ENSG00000256248 
 chr12 
 67302585 
 NUP107 
 chr12 
 69096563 
  416  
  110  
 445 
 775.54  
 
    
43. chr6-chr6 ff 
 
   TCGA-06-0686-01A  
 HSP90AB1 
 chr6 
 44214931 
 PTCHD4 
 chr6 
 47882266 
  4606  
  2515  
 6681 
 -75.62  
   TCGA-06-0686-01A  
 HSP90AB1 
 chr6 
 44216512 
 PTCHD4 
 chr6 
 47882266 
  3106  
  2515  
 3614 
 771.78  
 
    
44. chr11-chr11 rf 
 
   TCGA-06-2557-01A  
 ENSG00000254919 
 chr11 
 35939560 
 TRIM44 
 chr11 
 35827907 
  18  
  5  
 15 
 766.32  
 
    
45. chr14-chr19 ff 
 
   TCGA-14-0736-02A  
 WDR20 
 chr14 
 102606508 
 ASNA1 
 chr19 
 12856190 
  109  
  81  
 143 
 761.26  
 
    
46. chr12-chr12 rr 
 
   TCGA-06-0138-01A  
 MARS 
 chr12 
 57905480 
 YEATS4 
 chr12 
 69764754 
  104  
  50  
 96 
 674.24  
   TCGA-06-0129-01A  
 KIF5A 
 chr12 
 57957221 
 FRS2 
 chr12 
 69864309 
  38  
  18  
 29 
 120.46  
   TCGA-41-2571-01A  
 DTX3 
 chr12 
 58002302 
 FRS2 
 chr12 
 69864309 
  1207  
  1115  
 1008 
 405.07  
   TCGA-41-2571-01A  
 DTX3 
 chr12 
 58002302 
 FRS2 
 chr12 
 69864309 
  1207  
  1115  
 1008 
 405.07  
   TCGA-41-2571-01A  
 DTX3 
 chr12 
 58002859 
 FRS2 
 chr12 
 69864309 
  512  
  1115  
 491 
 351.72  
   TCGA-41-2571-01A  
 DTX3 
 chr12 
 58002859 
 FRS2 
 chr12 
 69864309 
  512  
  1115  
 491 
 351.72  
   TCGA-06-0138-01A  
 XRCC6BP1 
 chr12 
 58339410 
 YEATS4 
 chr12 
 69764754 
  658  
  394  
 660 
 760.18  
 
    
47. chr11-chr11 rf 
 
   TCGA-26-5132-01A  
 PATL1 
 chr11 
 59434325 
 TECTA 
 chr11 
 121058540 
  134  
  33  
 98 
 759.18  
 
    
48. chr1-chr14 ff 
 
   TCGA-06-0125-02A  
 ARID1A 
 chr1 
 27094489 
 RNF31 
 chr14 
 24624365 
  28  
  16  
 50 
 722.43  
   TCGA-06-0125-01A  
 ARID1A 
 chr1 
 27094489 
 RNF31 
 chr14 
 24624365 
  52  
  11  
 70 
 754.13  
 
    
49. chr12-chr12 fr 
 
   TCGA-06-0174  
 OS9 
 chr12 
 58089625 
 C12orf66 
 chr12 
 64588437 
  76  
  443  
 62 
 625.84  
   TCGA-06-0174-01A  
 OS9 
 chr12 
 58089625 
 C12orf66 
 chr12 
 64588437 
  76  
  443  
 62 
 625.84  
   TCGA-06-0174  
 OS9 
 chr12 
 58090155 
 C12orf66 
 chr12 
 64588437 
  766  
  443  
 772 
 753.16  
   TCGA-06-0174-01A  
 OS9 
 chr12 
 58090155 
 C12orf66 
 chr12 
 64588437 
  766  
  443  
 772 
 753.16  
 
    
50. chr2-chr2 rf 
 
   TCGA-14-0871-01A  
 ZEB2 
 chr2 
 145274844 
 ENSG00000226674 
 chr2 
 145939770 
  125  
  30  
 65 
 752.86  
 
    
51. chr17-chr17 ff 
 
   TCGA-14-0790-01B  
 ITGB4 
 chr17 
 73752328 
 TRIM65 
 chr17 
 73885805 
  79  
  58  
 122 
 748.30  
 
    
52. chr12-chr12 rf 
 
   TCGA-02-2485-01A  
 R3HDM2 
 chr12 
 57660513 
 XRCC6BP1 
 chr12 
 58339410 
  10  
  5  
 9 
 414.02  
   TCGA-06-0174  
 R3HDM2 
 chr12 
 57682791 
 PIP4K2C 
 chr12 
 57989670 
  191  
  132  
 115 
 744.01  
   TCGA-06-0174-01A  
 R3HDM2 
 chr12 
 57682791 
 PIP4K2C 
 chr12 
 57989670 
  191  
  132  
 115 
 744.01  
   TCGA-06-0174  
 R3HDM2 
 chr12 
 57686354 
 PIP4K2C 
 chr12 
 57989670 
  108  
  132  
 91 
 745.04  
   TCGA-06-0174  
 R3HDM2 
 chr12 
 57686354 
 PIP4K2C 
 chr12 
 57992847 
  28  
  132  
 18 
 680.20  
   TCGA-06-0174-01A  
 R3HDM2 
 chr12 
 57686354 
 PIP4K2C 
 chr12 
 57989670 
  108  
  132  
 91 
 745.04  
   TCGA-06-0174-01A  
 R3HDM2 
 chr12 
 57686354 
 PIP4K2C 
 chr12 
 57992847 
  28  
  132  
 18 
 680.20  
   TCGA-06-0174  
 R3HDM2 
 chr12 
 57689181 
 PIP4K2C 
 chr12 
 57989670 
  110  
  132  
 84 
 702.03  
   TCGA-06-0174  
 R3HDM2 
 chr12 
 57689181 
 PIP4K2C 
 chr12 
 57992847 
  15  
  132  
 16 
 658.98  
   TCGA-06-0174-01A  
 R3HDM2 
 chr12 
 57689181 
 PIP4K2C 
 chr12 
 57989670 
  110  
  132  
 84 
 702.03  
   TCGA-06-0174-01A  
 R3HDM2 
 chr12 
 57689181 
 PIP4K2C 
 chr12 
 57992847 
  15  
  132  
 16 
 658.98  
   TCGA-06-2570-01A  
 ENSG00000179912 
 chr12 
 57789531 
 TSFM 
 chr12 
 58176892 
  66  
  13  
 33 
 734.26  
   TCGA-02-2485-01A  
 ENSG00000179912 
 chr12 
 57824502 
 MARS 
 chr12 
 57898007 
  271  
  2  
 2 
 -67.76  
   TCGA-06-5859-01A  
 DDIT3 
 chr12 
 57914200 
 GLI1 
 chr12 
 57857447 
  5  
  4  
 6 
 -16.88  
 
    
53. chr2-chr19 fr 
 
   TCGA-76-4925-01A  
 FMNL2 
 chr2 
 153475102 
 WDR62 
 chr19 
 36574142 
  48  
  20  
 37 
 741.63  
   TCGA-76-4925-01A  
 FMNL2 
 chr2 
 153479727 
 WDR62 
 chr19 
 36574465 
  9  
  20  
 6 
 483.02  
 
    
54. chr7-chr7 rf 
 
   TCGA-06-0211-01A  
 SEC61G 
 chr7 
 54825187 
 EGFR 
 chr7 
 55224225 
  26  
  15  
 22 
 627.88  
   TCGA-06-0211-02A  
 SEC61G 
 chr7 
 54825187 
 EGFR 
 chr7 
 55224225 
  585  
  389  
 509 
 740.76  
   TCGA-06-0211-02A  
 SEC61G 
 chr7 
 54825187 
 EGFR 
 chr7 
 55224451 
  12  
  389  
 14 
 562.17  
 
    
55. chr6-chr6 fr 
 
   TCGA-06-0129-01A  
 DNAH8 
 chr6 
 38919295 
 TMEM217 
 chr6 
 37180771 
  50  
  27  
 67 
 735.26  
 
    
56. chr19-chr19 rr 
 
   TCGA-27-1834-01A  
 ENSG00000239736 
 chr19 
 42110518 
 MEGF8 
 chr19 
 42848984 
  60  
  24  
 35 
 734.64  
 
    
57. chr6-chr14 rr 
 
   TCGA-27-1835-01A  
 ENSG00000181355 
 chr6 
 10141263 
 RHOJ 
 chr14 
 63671764 
  28  
  76  
 37 
 684.33  
   TCGA-27-1835-01A  
 ENSG00000181355 
 chr6 
 10141853 
 RHOJ 
 chr14 
 63671764 
  341  
  76  
 289 
 734.17  
 
    
58. chr6-chr12 ff 
 
   TCGA-06-0129-01A  
 ENSG00000232598 
 chr6 
 36908138 
 MPHOSPH9 
 chr12 
 123678932 
  78  
  62  
 146 
 731.66  
 
    
59. chr11-chr22 ff 
 
   TCGA-06-1804-01A  
 CCDC83 
 chr11 
 85593777 
 SYN3 
 chr22 
 33315739 
  53  
  23  
 70 
 728.98  
 
    
60. chr12-chr12 ff 
 
   TCGA-02-2485-01A  
 MARS 
 chr12 
 57898081 
 ENSG00000258231 
 chr12 
 59195041 
  427  
  28  
 418 
 723.40  
   TCGA-02-2485-01A  
 MARS 
 chr12 
 57898081 
 ENSG00000258231 
 chr12 
 59199722 
  141  
  54  
 126 
 655.77  
   TCGA-02-2485-01A  
 MARS 
 chr12 
 57898081 
 ENSG00000258231 
 chr12 
 59199727 
  3  
  54  
 3 
 630.19  
   TCGA-02-2485-01A  
 MARS 
 chr12 
 57898081 
 ENSG00000258231 
 chr12 
 59205703 
  1  
  37  
 1 
 354.29  
   TCGA-02-2485-01A  
 MARS 
 chr12 
 57898081 
 ENSG00000258231 
 chr12 
 59206195 
  287  
  37  
 341 
 564.56  
   TCGA-02-2485-01A  
 MARS 
 chr12 
 57898081 
 LRIG3 
 chr12 
 59278133 
  18  
  3  
 15 
 348.76  
   TCGA-06-2570-01A  
 DDIT3 
 chr12 
 57912882 
 ENSG00000258231 
 chr12 
 59093541 
  14  
  2  
 18 
 505.26  
   TCGA-06-0138-01A  
 TSFM 
 chr12 
 58186855 
 ENSG00000258231 
 chr12 
 59128624 
  33  
  15  
 43 
 446.46  
 
    
61. chr11-chr22 rr 
 
   TCGA-06-1804-01A  
 CREBZF 
 chr11 
 85373713 
 SYN3 
 chr22 
 33316392 
  33  
  22  
 48 
 722.47  
 
    
62. chr12-chr12 ff 
 
   TCGA-06-0174  
 OS9 
 chr12 
 58090155 
 CPSF6 
 chr12 
 69642119 
  132  
  61  
 167 
 616.37  
   TCGA-06-0174  
 OS9 
 chr12 
 58090155 
 CPSF6 
 chr12 
 69644908 
  30  
  61  
 60 
 416.91  
   TCGA-06-0174-01A  
 OS9 
 chr12 
 58090155 
 CPSF6 
 chr12 
 69642119 
  132  
  61  
 167 
 616.37  
   TCGA-06-0174-01A  
 OS9 
 chr12 
 58090155 
 CPSF6 
 chr12 
 69644908 
  30  
  61  
 60 
 416.91  
   TCGA-06-0138-01A  
 METTL21B 
 chr12 
 58166910 
 LYZ 
 chr12 
 69743887 
  14  
  5  
 16 
 719.80  
   TCGA-06-0138-01A  
 TSFM 
 chr12 
 58186855 
 LYZ 
 chr12 
 69743887 
  12  
  9  
 16 
 605.53  
 
    
63. chr12-chr12 fr 
 
   TCGA-06-0138-01A  
 HMGA2 
 chr12 
 66267968 
 YEATS4 
 chr12 
 69764754 
  143  
  57  
 169 
 718.93  
 
    
64. chr7-chr7 rf 
 
   TCGA-06-5410-01A  
 TMEM176B 
 chr7 
 150497835 
 TMEM176A 
 chr7 
 150499324 
  1  
  39  
 0 
 -342.07  
   TCGA-06-0238-01A  
 TMEM176B 
 chr7 
 150497851 
 TMEM176A 
 chr7 
 150498645 
  1  
  22  
 1 
 -295.60  
   TCGA-41-2572-01A  
 TMEM176B 
 chr7 
 150497853 
 TMEM176A 
 chr7 
 150498643 
  1  
  15  
 1 
 718.02  
   TCGA-14-0736-02A  
 TMEM176B 
 chr7 
 150498015 
 TMEM176A 
 chr7 
 150498635 
  1  
  29  
 1 
 439.53  
 
    
65. chr3-chr16 fr 
 
   TCGA-06-5414-01A  
 PLA1A 
 chr3 
 119341433 
 TCF25 
 chr16 
 89952362 
  240  
  43  
 221 
 718.12  
 
    
66. chr10-chr10 ff 
 
   TCGA-02-2483-01A  
 LHPP 
 chr10 
 126186696 
 FANK1 
 chr10 
 127668729 
  4  
  5  
 5 
 584.93  
   TCGA-02-2483-01A  
 LHPP 
 chr10 
 126205839 
 FANK1 
 chr10 
 127668729 
  27  
  14  
 51 
 717.16  
   TCGA-02-2483-01A  
 LHPP 
 chr10 
 126214832 
 FANK1 
 chr10 
 127668729 
  1  
  14  
 1 
 333.94  
   TCGA-02-2483-01A  
 LHPP 
 chr10 
 126234757 
 FANK1 
 chr10 
 127668729 
  27  
  22  
 20 
 197.60  
   TCGA-02-2483-01A  
 LHPP 
 chr10 
 126234757 
 FANK1 
 chr10 
 127677119 
  40  
  23  
 31 
 220.28  
   TCGA-02-2483-01A  
 LHPP 
 chr10 
 126234809 
 FANK1 
 chr10 
 127668729 
  8  
  22  
 10 
 224.73  
   TCGA-02-2483-01A  
 LHPP 
 chr10 
 126234809 
 FANK1 
 chr10 
 127677119 
  19  
  23  
 17 
 227.62  
 
    
67. chr10-chr10 fr 
 
   TCGA-12-0619-01A  
 VIM 
 chr10 
 17271983 
 MRC1 
 chr10 
 17855220 
  140  
  72  
 82 
 715.45  
   TCGA-12-0619-01A  
 VIM 
 chr10 
 17271983 
 MRC1 
 chr10 
 18102207 
  140  
  68  
 80 
 716.40  
 
    
68. chr12-chr6 rf 
 
   TCGA-06-0129-01A  
 MPHOSPH9 
 chr12 
 123678932 
 ENSG00000198663 
 chr6 
 36846783 
  85  
  44  
 95 
 714.07  
 
    
69. chrX-chrX rf 
 
   TCGA-06-2569-01A  
 NLGN4X 
 chrX 
 6134247 
 ARHGAP6 
 chrX 
 11272669 
  50  
  30  
 45 
 710.23  
   TCGA-06-2569-01A  
 NLGN4X 
 chrX 
 6144294 
 ARHGAP6 
 chrX 
 11270468 
  2  
  53  
 1 
 -517.77  
   TCGA-06-2569-01A  
 NLGN4X 
 chrX 
 6144674 
 ARHGAP6 
 chrX 
 11270468 
  5  
  43  
 6 
 84.69  
   TCGA-06-2569-01A  
 NLGN4X 
 chrX 
 6144905 
 ARHGAP6 
 chrX 
 11270468 
  10  
  43  
 7 
 317.00  
   TCGA-06-2569-01A  
 NLGN4X 
 chrX 
 6144919 
 ARHGAP6 
 chrX 
 11270468 
  19  
  43  
 18 
 329.76  
   TCGA-06-2569-01A  
 NLGN4X 
 chrX 
 6145709 
 ARHGAP6 
 chrX 
 11270468 
  1  
  43  
 0 
 -645.93  
   TCGA-06-2569-01A  
 NLGN4X 
 chrX 
 6145728 
 ARHGAP6 
 chrX 
 11270468 
  2  
  43  
 1 
 -494.13  
   TCGA-06-2569-01A  
 NLGN4X 
 chrX 
 6146047 
 ARHGAP6 
 chrX 
 11270468 
  2  
  43  
 1 
 -74.84  
   TCGA-06-2569-01A  
 NLGN4X 
 chrX 
 6146581 
 ARHGAP6 
 chrX 
 11270467 
  67  
  43  
 41 
 665.45  
 
    
70. chr9-chr9 rf 
 
   TCGA-28-1753-01A  
 VAV2 
 chr9 
 136804224 
 NOTCH1 
 chr9 
 139389937 
  55  
  11  
 42 
 708.58  
 
    
71. chr20-chrX fr 
 
   TCGA-06-5856-01A  
 RALGAPB 
 chr20 
 37126158 
 BCORL1 
 chrX 
 129181491 
  38  
  16  
 41 
 706.27  
 
    
72. chr19-chr20 rf 
 
   TCGA-06-0238-01A  
 MOB3A 
 chr19 
 2076809 
 BMP7 
 chr20 
 55802327 
  29  
  23  
 31 
 702.85  
 
    
73. chr12-chr12 rr 
 
   TCGA-06-5856-01A  
 KDM2B 
 chr12 
 121986781 
 KSR2 
 chr12 
 118298235 
  40  
  10  
 52 
 702.45  
 
    
74. chr9-chr9 rr 
 
   TCGA-28-5204-01A  
 UBE2R2 
 chr9 
 33911961 
 CLTA 
 chr9 
 36204175 
  79  
  26  
 59 
 702.34  
   TCGA-28-5204-01A  
 UBE2R2 
 chr9 
 33917015 
 CLTA 
 chr9 
 36204175 
  2  
  26  
 1 
 644.72  
 
    
75. chr5-chr6 ff 
 
   TCGA-06-0129-01A  
 PCDHGA3 
 chr5 
 140858112 
 QKI 
 chr6 
 163956013 
  80  
  26  
 115 
 701.31  
   TCGA-06-0129-01A  
 PCDHGA3 
 chr5 
 140867181 
 QKI 
 chr6 
 163956013 
  5  
  24  
 7 
 465.92  
 
    
76. chr16-chr16 rr 
 
   TCGA-26-5133-01A  
 DDX19B 
 chr16 
 70358487 
 SF3B3 
 chr16 
 70595686 
  6  
  3  
 6 
 699.85  
 
    
77. chr11-chr11 rr 
 
   TCGA-06-0744  
 ENSG00000255367 
 chr11 
 3566487 
 RRM1 
 chr11 
 4144613 
  24  
  18  
 14 
 698.11  
   TCGA-06-0744-01A  
 ENSG00000255367 
 chr11 
 3566487 
 RRM1 
 chr11 
 4144613 
  24  
  18  
 14 
 698.11  
 
    
78. chr19-chr19 fr 
 
   TCGA-19-2620-01A  
 SCAF1 
 chr19 
 50150086 
 TRAPPC6A 
 chr19 
 45668227 
  16  
  19  
 13 
 697.45  
   TCGA-19-2620-01A  
 SCAF1 
 chr19 
 50150086 
 TRAPPC6A 
 chr19 
 45668451 
  17  
  19  
 14 
 556.79  
 
    
79. chr4-chr1 fr 
 
   TCGA-76-4925-01A  
 FGFR3 
 chr4 
 1805615 
 KCND3 
 chr1 
 112396666 
  665  
  163  
 606 
 697.99  
 
    
80. chr12-chr12 fr 
 
   TCGA-06-5856-01A  
 SUDS3 
 chr12 
 118829086 
 CDK4 
 chr12 
 58145124 
  9  
  412  
 10 
 668.12  
   TCGA-06-5856-01A  
 SUDS3 
 chr12 
 118829086 
 CDK4 
 chr12 
 58145518 
  137  
  412  
 129 
 696.81  
 
    
81. chr1-chr11 ff 
 
   TCGA-12-1597-01B  
 NFASC 
 chr1 
 204951147 
 RTN3 
 chr11 
 63525627 
  78  
  42  
 120 
 696.71  
   TCGA-12-1597-01B  
 NFASC 
 chr1 
 204953269 
 RTN3 
 chr11 
 63525627 
  2  
  42  
 4 
 -612.00  
 
    
82. chr1-chr1 rf 
 
   TCGA-26-5139-01A  
 CCT3 
 chr1 
 156303337 
 ENSG00000236947 
 chr1 
 156425587 
  7  
  4  
 4 
 8.97  
   TCGA-19-2619-01A  
 C1orf61 
 chr1 
 156383204 
 RHBG 
 chr1 
 156347091 
  5  
  141  
 5 
 630.29  
   TCGA-19-2619-01A  
 C1orf61 
 chr1 
 156384445 
 RHBG 
 chr1 
 156347091 
  3  
  141  
 2 
 692.43  
 
    
83. chr7-chr7 rr 
 
   TCGA-06-2557-01A  
 VWC2 
 chr7 
 49951629 
 CASD1 
 chr7 
 94146967 
  7  
  9  
 2 
 471.47  
   TCGA-06-2557-01A  
 VWC2 
 chr7 
 49951629 
 CASD1 
 chr7 
 94147634 
  38  
  9  
 29 
 691.32  
 
    
84. chr12-chr12 ff 
 
   TCGA-06-0129-01A  
 PEX5 
 chr12 
 7344295 
 MED13L 
 chr12 
 116543971 
  17  
  17  
 19 
 167.92  
   TCGA-06-0129-01A  
 PEX5 
 chr12 
 7344295 
 MED13L 
 chr12 
 116544027 
  5  
  17  
 6 
 264.08  
   TCGA-06-0129-01A  
 PEX5 
 chr12 
 7344295 
 MED13L 
 chr12 
 116544030 
  21  
  17  
 20 
 -29.31  
   TCGA-06-0129-01A  
 PEX5 
 chr12 
 7347258 
 MED13L 
 chr12 
 116549232 
  57  
  17  
 66 
 685.50  
 
    
85. chr12-chr12 rr 
 
   TCGA-06-0138-01A  
 OS9 
 chr12 
 58109542 
 SLC35E3 
 chr12 
 69145969 
  49  
  52  
 31 
 682.36  
 
    
86. chr7-chr7 rr 
 
   TCGA-14-1402-02A  
 H2AFV 
 chr7 
 44875127 
 PPP1R9A 
 chr7 
 94666092 
  20  
  80  
 14 
 679.26  
   TCGA-14-1402-02A  
 H2AFV 
 chr7 
 44875127 
 PPP1R9A 
 chr7 
 94666422 
  25  
  80  
 13 
 515.77  
   TCGA-14-1402-02A  
 H2AFV 
 chr7 
 44875130 
 PPP1R9A 
 chr7 
 94666092 
  20  
  80  
 3 
 675.48  
 
    
87. chr19-chr19 rr 
 
   TCGA-32-1970-01A  
 AP2A1 
 chr19 
 50285013 
 ZNF544 
 chr19 
 58757792 
  1  
  21  
 0 
 678.34  
 
    
88. chr7-chr7 fr 
 
   TCGA-32-5222-01A  
 ENSG00000249773 
 chr7 
 55974071 
 SEPT14 
 chr7 
 55912410 
  10  
  70  
 9 
 677.56  
   TCGA-32-5222-01A  
 ENSG00000249773 
 chr7 
 55974071 
 SEPT14 
 chr7 
 55914329 
  138  
  70  
 128 
 659.25  
   TCGA-06-0743-01A  
 GBAS 
 chr7 
 56032393 
 PSPH 
 chr7 
 56079561 
  115  
  2  
 1 
 -712.84  
 
    
89. chr7-chr7 fr 
 
   TCGA-28-5209-01A  
 EGFR 
 chr7 
 55268105 
 PSPH 
 chr7 
 56079561 
  9667  
  703  
 3979 
 640.40  
   TCGA-28-5215-01A  
 EGFR 
 chr7 
 55268105 
 PSPH 
 chr7 
 56079561 
  42  
  5  
 21 
 676.07  
   TCGA-28-2513-01A  
 LANCL2 
 chr7 
 55433921 
 ZNF713 
 chr7 
 55980652 
  44  
  13  
 53 
 515.95  
 
    
90. chr12-chr12 rf 
 
   TCGA-19-2624-01A  
 PPM1H 
 chr12 
 63182005 
 MDM2 
 chr12 
 69202987 
  21  
  8  
 19 
 671.42  
 
    
91. chr2-chr5 rf 
 
   TCGA-12-1597-01B  
 NRXN1 
 chr2 
 50758364 
 DIAPH1 
 chr5 
 140995579 
  12  
  10  
 9 
 496.20  
   TCGA-12-1597-01B  
 NRXN1 
 chr2 
 50758364 
 HDAC3 
 chr5 
 141005622 
  5  
  5  
 6 
 666.83  
 
    
92. chr7-chr12 fr 
 
   TCGA-19-2624-01A  
 EGFR 
 chr7 
 55240816 
 GNS 
 chr12 
 65110598 
  48  
  29  
 50 
 666.41  
 
    
93. chr2-chr4 ff 
 
   TCGA-06-2565-01A  
 RAMP1 
 chr2 
 238786003 
 ENSG00000250266 
 chr4 
 171197732 
  49  
  25  
 52 
 664.21  
 
    
94. chr7-chr7 ff 
 
   TCGA-06-2557-01A  
 GBAS 
 chr7 
 56052570 
 CYP51A1 
 chr7 
 91760082 
  365  
  131  
 546 
 662.80  
   TCGA-06-2557-01A  
 GBAS 
 chr7 
 56059056 
 CYP51A1 
 chr7 
 91759718 
  62  
  129  
 55 
 636.53  
 
    
95. chr12-chr12 rf 
 
   TCGA-06-5856-01A  
 CTDSP2 
 chr12 
 58240154 
 MDM2 
 chr12 
 69202987 
  36  
  19  
 22 
 661.94  
 
    
96. chr16-chr16 rr 
 
   TCGA-06-5856-01A  
 IL32 
 chr16 
 3115560 
 NAA60 
 chr16 
 3498539 
  3  
  41  
 2 
 347.36  
   TCGA-06-5856-01A  
 IL32 
 chr16 
 3115560 
 NAA60 
 chr16 
 3508180 
  4  
  37  
 3 
 -112.38  
   TCGA-06-5856-01A  
 IL32 
 chr16 
 3115781 
 NAA60 
 chr16 
 3498539 
  5  
  41  
 4 
 590.46  
   TCGA-06-5856-01A  
 IL32 
 chr16 
 3115781 
 NAA60 
 chr16 
 3508180 
  14  
  37  
 9 
 64.45  
   TCGA-06-5856-01A  
 IL32 
 chr16 
 3115784 
 NAA60 
 chr16 
 3498539 
  45  
  41  
 39 
 661.64  
   TCGA-06-5856-01A  
 IL32 
 chr16 
 3115784 
 NAA60 
 chr16 
 3508180 
  52  
  37  
 49 
 136.98  
   TCGA-06-5856-01A  
 IL32 
 chr16 
 3117377 
 NAA60 
 chr16 
 3508180 
  1  
  37  
 0 
 49.48  
 
    
97. chr3-chr3 rr 
 
   TCGA-06-0645-01A  
 GPR128 
 chr3 
 100348441 
 TFG 
 chr3 
 100438901 
  16  
  17  
 18 
 520.05  
   TCGA-19-0957-02A  
 GPR128 
 chr3 
 100348441 
 TFG 
 chr3 
 100438901 
  14  
  7  
 4 
 508.62  
   TCGA-19-1787-01B  
 GPR128 
 chr3 
 100348441 
 TFG 
 chr3 
 100432712 
  1  
  18  
 1 
 467.32  
   TCGA-19-1787-01B  
 GPR128 
 chr3 
 100348441 
 TFG 
 chr3 
 100438901 
  44  
  18  
 46 
 656.92  
   TCGA-27-1830-01A  
 GPR128 
 chr3 
 100348441 
 TFG 
 chr3 
 100438901 
  9  
  3  
 10 
 442.24  
   TCGA-28-5216-01A  
 GPR128 
 chr3 
 100348441 
 TFG 
 chr3 
 100438901 
  14  
  3  
 15 
 394.44  
 
    
98. chr12-chr12 fr 
 
   TCGA-06-0129-01A  
 DIP2B 
 chr12 
 51034634 
 DNAH10 
 chr12 
 124249794 
  11  
  8  
 9 
 485.45  
   TCGA-06-0129-01A  
 DIP2B 
 chr12 
 51034634 
 DNAH10 
 chr12 
 124273143 
  104  
  45  
 100 
 654.10  
   TCGA-06-0129-01A  
 DIP2B 
 chr12 
 51034634 
 DNAH10 
 chr12 
 124273148 
  32  
  45  
 28 
 592.52  
   TCGA-06-0129-01A  
 DIP2B 
 chr12 
 51034634 
 DNAH10 
 chr12 
 124273171 
  2  
  45  
 2 
 445.14  
   TCGA-06-0129-01A  
 DIP2B 
 chr12 
 51034634 
 DNAH10 
 chr12 
 124274718 
  15  
  45  
 13 
 443.40  
 
    
99. chr7-chr7 rr 
 
   TCGA-06-0184-01A  
 CAV1 
 chr7 
 116198999 
 ST7-AS1 
 chr7 
 116593744 
  178  
  56  
 115 
 650.57  
 
    
100. chr7-chr7 rf 
 
   TCGA-32-1970-01A  
 HIBADH 
 chr7 
 27668989 
 C7orf10 
 chr7 
 40723654 
  7  
  3  
 8 
 449.06  
   TCGA-32-1970-01A  
 HIBADH 
 chr7 
 27668989 
 C7orf10 
 chr7 
 40789032 
  35  
  8  
 33 
 649.29  
 
    
101. chr15-chr11 rf 
 
   TCGA-06-0174  
 MEIS2 
 chr15 
 37329014 
 ZNF143 
 chr11 
 9516192 
  24  
  9  
 32 
 648.87  
   TCGA-06-0174-01A  
 MEIS2 
 chr15 
 37329014 
 ZNF143 
 chr11 
 9516192 
  24  
  9  
 32 
 648.87  
 
    
102. chr3-chr19 ff 
 
   TCGA-06-5859-01A  
 MAP3K13 
 chr3 
 185003430 
 CD37 
 chr19 
 49840165 
  30  
  8  
 33 
 647.49  
 
    
103. chr6-chr12 rf 
 
   TCGA-06-0129-01A  
 TFEB 
 chr6 
 41682958 
 DIP2B 
 chr12 
 51115168 
  42  
  47  
 41 
 641.59  
   TCGA-06-0129-01A  
 TFEB 
 chr6 
 41703087 
 DIP2B 
 chr12 
 51116984 
  65  
  31  
 71 
 105.01  
 
    
104. chr4-chr9 fr 
 
   TCGA-06-0129-01A  
 NAA15 
 chr4 
 140222984 
 LPAR1 
 chr9 
 113638001 
  46  
  18  
 39 
 640.59  
 
    
105. chr7-chr7 rf 
 
   TCGA-12-0616-01A  
 TPK1 
 chr7 
 144462972 
 ENSG00000233969 
 chr7 
 119825025 
  36  
  3  
 24 
 640.60  
 
    
106. chr12-chr12 rr 
 
   TCGA-06-0129-01A  
 USP5 
 chr12 
 6964564 
 COQ10A 
 chr12 
 56663344 
  26  
  6  
 16 
 639.25  
 
    
107. chr15-chr15 fr 
 
   TCGA-12-5295-01A  
 AKAP13 
 chr15 
 86103356 
 IQGAP1 
 chr15 
 90999546 
  74  
  17  
 65 
 639.84  
 
    
108. chr9-chr9 ff 
 
   TCGA-27-2523-01A  
 NUDT2 
 chr9 
 34329596 
 GLIPR2 
 chr9 
 36162358 
  5  
  14  
 9 
 -71.01  
   TCGA-27-2523-01A  
 NUDT2 
 chr9 
 34339163 
 GLIPR2 
 chr9 
 36162228 
  1  
  14  
 1 
 591.67  
   TCGA-27-2523-01A  
 NUDT2 
 chr9 
 34339163 
 GLIPR2 
 chr9 
 36162358 
  29  
  14  
 37 
 638.93  
 
    
109. chr1-chr15 rf 
 
   TCGA-06-0125-02A  
 PAFAH2 
 chr1 
 26323665 
 BTBD1 
 chr15 
 83735502 
  51  
  10  
 59 
 526.76  
   TCGA-06-0125-01A  
 PAFAH2 
 chr1 
 26323665 
 BTBD1 
 chr15 
 83735502 
  98  
  21  
 93 
 633.89  
 
    
110. chr19-chr19 rr 
 
   TCGA-14-1034-02B  
 FKBP8 
 chr19 
 18644079 
 SLC1A6 
 chr19 
 15061201 
  127  
  59  
 135 
 434.68  
   TCGA-14-1034-01A  
 FKBP8 
 chr19 
 18644079 
 SLC1A6 
 chr19 
 15061201 
  154  
  71  
 190 
 633.36  
   TCGA-14-1034-02B  
 FKBP8 
 chr19 
 18648410 
 SLC1A6 
 chr19 
 15061201 
  5  
  59  
 6 
 324.02  
   TCGA-14-1034-01A  
 FKBP8 
 chr19 
 18648410 
 SLC1A6 
 chr19 
 15061201 
  6  
  71  
 11 
 571.45  
 
    
111. chr12-chr12 fr 
 
   TCGA-06-0138-01A  
 METTL21B 
 chr12 
 58166910 
 CPM 
 chr12 
 69326619 
  17  
  9  
 13 
 454.17  
   TCGA-06-0138-01A  
 METTL21B 
 chr12 
 58166910 
 CPM 
 chr12 
 69340433 
  37  
  6  
 27 
 632.33  
 
    
112. chr12-chr12 rf 
 
   TCGA-06-0129-01A  
 SLC11A2 
 chr12 
 51386592 
 PFKM 
 chr12 
 48516549 
  15  
  7  
 10 
 631.32  
 
    
113. chr12-chr12 rr 
 
   TCGA-06-0138-01A  
 ENSG00000135678 
 chr12 
 69357942 
 AGAP2 
 chr12 
 58131983 
  18  
  19  
 34 
 628.56  
   TCGA-06-0138-01A  
 ENSG00000135678 
 chr12 
 69357942 
 AGAP2 
 chr12 
 58132952 
  3  
  19  
 5 
 240.38  
   TCGA-06-0138-01A  
 ENSG00000135678 
 chr12 
 69357942 
 AGAP2 
 chr12 
 58133752 
  1  
  19  
 1 
 35.60  
   TCGA-06-0138-01A  
 ENSG00000135678 
 chr12 
 69363875 
 AGAP2 
 chr12 
 58131983 
  1  
  19  
 2 
 -17.20  
 
    
114. chr12-chr4 rf 
 
   TCGA-06-2559-01A  
 CTDSP2 
 chr12 
 58240154 
 DCUN1D4 
 chr4 
 52765435 
  36  
  12  
 32 
 627.34  
 
    
115. chr17-chr17 fr 
 
   TCGA-14-1034-01A  
 POLR2A 
 chr17 
 7412718 
 KPNB1 
 chr17 
 45742523 
  2  
  11  
 1 
 -521.71  
   TCGA-14-1034-02B  
 POLR2A 
 chr17 
 7414610 
 KPNB1 
 chr17 
 45742523 
  27  
  24  
 13 
 627.89  
   TCGA-14-1034-02B  
 POLR2A 
 chr17 
 7415049 
 KPNB1 
 chr17 
 45742523 
  1  
  24  
 1 
 25.98  
   TCGA-14-1034-02B  
 POLR2A 
 chr17 
 7416012 
 KPNB1 
 chr17 
 45742523 
  2  
  24  
 2 
 -400.87  
 
    
116. chr15-chr12 fr 
 
   TCGA-06-5415-01A  
 APBA2 
 chr15 
 29213951 
 CHD4 
 chr12 
 6710931 
  10  
  4  
 7 
 625.01  
 
    
117. chr12-chr2 rf 
 
   TCGA-06-0221-02A  
 C12orf4 
 chr12 
 4639805 
 ENSG00000225942 
 chr2 
 546657 
  3  
  7  
 2 
 417.73  
   TCGA-06-0221-02A  
 C12orf4 
 chr12 
 4643322 
 ENSG00000233633 
 chr2 
 559108 
  15  
  22  
 24 
 623.10  
   TCGA-06-0221-02A  
 C12orf4 
 chr12 
 4643322 
 ENSG00000233633 
 chr2 
 560077 
  15  
  21  
 11 
 561.74  
 
    
118. chr19-chr19 ff 
 
   TCGA-14-1034-02B  
 ZNF557 
 chr19 
 7081453 
 ENSG00000246896 
 chr19 
 15052293 
  1  
  28  
 1 
 487.03  
   TCGA-14-1034-02B  
 ZNF557 
 chr19 
 7081465 
 ENSG00000246896 
 chr19 
 15052293 
  6  
  28  
 13 
 511.32  
   TCGA-14-1034-01A  
 ZNF557 
 chr19 
 7081465 
 ENSG00000246896 
 chr19 
 15052293 
  3  
  22  
 3 
 450.50  
   TCGA-14-1034-02B  
 ZNF557 
 chr19 
 7082062 
 ENSG00000246896 
 chr19 
 15052293 
  13  
  28  
 42 
 623.84  
   TCGA-14-1034-01A  
 ZNF557 
 chr19 
 7082062 
 ENSG00000246896 
 chr19 
 15052293 
  17  
  22  
 24 
 586.47  
 
    
119. chr8-chr8 rr 
 
   TCGA-28-2513-01A  
 MSR1 
 chr8 
 15971876 
 VPS37A 
 chr8 
 17104877 
  20  
  7  
 7 
 620.34  
 
    
120. chr12-chr12 rf 
 
   TCGA-19-2624-01A  
 PPM1H 
 chr12 
 63182003 
 IL26 
 chr12 
 68610942 
  23  
  3  
 17 
 343.81  
   TCGA-19-2624-01A  
 PPM1H 
 chr12 
 63182003 
 RAP1B 
 chr12 
 69042487 
  2  
  37  
 1 
 618.76  
   TCGA-19-2624-01A  
 PPM1H 
 chr12 
 63182005 
 RAP1B 
 chr12 
 69042478 
  740  
  37  
 397 
 240.72  
   TCGA-19-2624-01A  
 PPM1H 
 chr12 
 63182005 
 RAP1B 
 chr12 
 69047891 
  2  
  37  
 0 
 289.96  
 
    
121. chr12-chr12 rf 
 
   TCGA-06-0129-01A  
 RECQL 
 chr12 
 21652488 
 ENSG00000184752 
 chr12 
 95344741 
  16  
  9  
 12 
 615.26  
 
    
122. chr6-chr12 rf 
 
   TCGA-06-0129-01A  
 PPIL1 
 chr6 
 36833724 
 MPHOSPH9 
 chr12 
 123678932 
  75  
  45  
 81 
 614.50  
 
    
123. chr12-chr12 rr 
 
   TCGA-06-0878-01A  
 NCOR2 
 chr12 
 124950718 
 DHX37 
 chr12 
 125450357 
  19  
  16  
 16 
 614.20  
   TCGA-06-0878-01A  
 NCOR2 
 chr12 
 124957497 
 DHX37 
 chr12 
 125450357 
  1  
  16  
 1 
 397.65  
 
    
124. chr11-chr11 rr 
 
   TCGA-14-2554-01A  
 LOC643037 
 chr11 
 94250748 
 ENSG00000166025 
 chr11 
 94474153 
  48  
  13  
 10 
 614.72  
 
    
125. chr7-chr7 rf 
 
   TCGA-32-2615-01A  
 KRIT1 
 chr7 
 91866980 
 GNGT1 
 chr7 
 93536047 
  60  
  26  
 46 
 613.06  
   TCGA-32-2615-01A  
 KRIT1 
 chr7 
 91866980 
 GNGT1 
 chr7 
 93536050 
  28  
  26  
 22 
 558.91  
   TCGA-32-2615-01A  
 KRIT1 
 chr7 
 91870306 
 GNGT1 
 chr7 
 93536047 
  1  
  26  
 3 
 531.65  
 
    
126. chr5-chr5 rr 
 
   TCGA-06-1804-01A  
 MGAT1 
 chr5 
 180235647 
 FLT4 
 chr5 
 180058777 
  13  
  13  
 13 
 612.21  
 
    
127. chr1-chr1 fr 
 
   TCGA-27-2523-01A  
 RPS6KC1 
 chr1 
 213303231 
 LYST 
 chr1 
 235840918 
  29  
  15  
 29 
 612.04  
 
    
128. chr13-chr13 ff 
 
   TCGA-12-0619-01A  
 ENSG00000136156 
 chr13 
 48836856 
 MYCBP2 
 chr13 
 77847606 
  20  
  7  
 41 
 610.82  
 
    
129. chr4-chr4 rf 
 
   TCGA-19-0957-02A  
 CHIC2 
 chr4 
 54880003 
 ENSG00000150471 
 chr4 
 62248841 
  80  
  24  
 61 
 608.20  
 
    
130. chr12-chr12 fr 
 
   TCGA-19-2624-01A  
 MDM2 
 chr12 
 69230528 
 PPM1H 
 chr12 
 63195939 
  25  
  8  
 23 
 608.77  
 
    
131. chr1-chr5 ff 
 
   TCGA-27-1835-01A  
 MIIP 
 chr1 
 12090180 
 CMYA5 
 chr5 
 79084793 
  8  
  4  
 10 
 607.75  
 
    
132. chr14-chr17 fr 
 
   TCGA-32-2616-01A  
 GPHN 
 chr14 
 67391008 
 SPECC1 
 chr17 
 19927780 
  41  
  12  
 43 
 607.13  
 
    
133. chr12-chr12 fr 
 
   TCGA-02-2485-01A  
 GLI1 
 chr12 
 57856658 
 MARS 
 chr12 
 57892405 
  12  
  46  
 19 
 512.45  
   TCGA-06-5856-01A  
 METTL1 
 chr12 
 58165072 
 CTDSP2 
 chr12 
 58221372 
  15  
  4  
 9 
 552.59  
   TCGA-41-5651-01A  
 METTL21B 
 chr12 
 58166910 
 METTL1 
 chr12 
 58163460 
  8  
  39  
 8 
 558.79  
   TCGA-41-5651-01A  
 METTL21B 
 chr12 
 58166910 
 METTL1 
 chr12 
 58163738 
  22  
  39  
 20 
 605.11  
   TCGA-41-5651-01A  
 METTL21B 
 chr12 
 58168470 
 METTL1 
 chr12 
 58163738 
  2  
  39  
 2 
 433.03  
   TCGA-41-5651-01A  
 METTL21B 
 chr12 
 58168549 
 METTL1 
 chr12 
 58163738 
  11  
  39  
 14 
 460.68  
 
    
134. chr6-chr6 rr 
 
   TCGA-06-0129-01A  
 SLC22A23 
 chr6 
 3410421 
 TNFRSF21 
 chr6 
 47221256 
  21  
  16  
 15 
 605.15  
 
    
135. chr9-chr9 ff 
 
   TCGA-06-0129-01A  
 CTNNAL1 
 chr9 
 111754902 
 RGS3 
 chr9 
 116291500 
  28  
  7  
 29 
 605.32  
 
    
136. chr7-chr7 rr 
 
   TCGA-06-0210-01A  
 TMED4 
 chr7 
 44620691 
 ENSG00000232006 
 chr7 
 43012441 
  13  
  17  
 28 
 407.09  
   TCGA-06-0210-01A  
 TMED4 
 chr7 
 44620691 
 ENSG00000232006 
 chr7 
 43012442 
  1  
  17  
 1 
 363.27  
   TCGA-06-0210-01A  
 TMED4 
 chr7 
 44620691 
 ENSG00000232006 
 chr7 
 43012446 
  26  
  17  
 34 
 419.91  
   TCGA-06-0210-02A  
 TMED4 
 chr7 
 44620691 
 ENSG00000232006 
 chr7 
 43012441 
  32  
  41  
 43 
 517.53  
   TCGA-06-0210-02A  
 TMED4 
 chr7 
 44620691 
 ENSG00000232006 
 chr7 
 43012446 
  32  
  41  
 42 
 509.06  
   TCGA-06-0210-01A  
 TMED4 
 chr7 
 44621047 
 ENSG00000232006 
 chr7 
 43012446 
  6  
  17  
 7 
 438.28  
   TCGA-06-0210-02A  
 TMED4 
 chr7 
 44621047 
 ENSG00000232006 
 chr7 
 43012446 
  10  
  41  
 20 
 604.80  
 
    
137. chr3-chr3 rr 
 
   TCGA-06-0219-01A  
 SEC62 
 chr3 
 169710381 
 YEATS2 
 chr3 
 183442318 
  22  
  23  
 19 
 602.61  
 
    
138. chr7-chr7 rr 
 
   TCGA-06-0211-01A  
 ENSG00000231427 
 chr7 
 54406822 
 MRPS17 
 chr7 
 56019622 
  37  
  7  
 49 
 535.42  
   TCGA-06-0211-01B  
 ENSG00000231427 
 chr7 
 54411333 
 MRPS17 
 chr7 
 56019622 
  261  
  9  
 415 
 600.58  
   TCGA-06-0211-01A  
 ENSG00000231427 
 chr7 
 54411333 
 MRPS17 
 chr7 
 56019622 
  169  
  7  
 221 
 574.81  
   TCGA-14-1402-02A  
 SEC61G 
 chr7 
 54823471 
 ENSG00000231317 
 chr7 
 55727802 
  35  
  36  
 38 
 587.12  
   TCGA-14-1829-01A  
 SEC61G 
 chr7 
 54823471 
 ENSG00000231317 
 chr7 
 55727802 
  13  
  28  
 16 
 526.05  
   TCGA-14-1402-02A  
 SEC61G 
 chr7 
 54825187 
 ENSG00000231317 
 chr7 
 55727802 
  16  
  36  
 13 
 557.69  
   TCGA-14-1829-01A  
 SEC61G 
 chr7 
 54825187 
 ENSG00000231317 
 chr7 
 55727802 
  9  
  28  
 12 
 492.45  
   TCGA-14-1402-02A  
 SEC61G 
 chr7 
 54826850 
 ENSG00000231317 
 chr7 
 55727802 
  8  
  36  
 0 
 59.67  
   TCGA-14-1829-01A  
 SEC61G 
 chr7 
 54826850 
 ENSG00000231317 
 chr7 
 55727802 
  24  
  28  
 1 
 -843.62  
   TCGA-28-2513-01A  
 SEC61G 
 chr7 
 54826850 
 CHCHD2 
 chr7 
 56170703 
  38  
  158  
 0 
 -345.96  
   TCGA-28-2513-01A  
 SEC61G 
 chr7 
 54826850 
 ENSG00000106153 
 chr7 
 56174262 
  4810  
  166  
 65 
 -502.94  
 
    
139. chr12-chr12 ff 
 
   TCGA-41-2571-01A  
 FRS2 
 chr12 
 69864309 
 RAB3IP 
 chr12 
 70178499 
  1  
  22  
 4 
 241.49  
   TCGA-41-2571-01A  
 FRS2 
 chr12 
 69864309 
 RAB3IP 
 chr12 
 70178499 
  1  
  22  
 4 
 241.49  
   TCGA-41-2571-01A  
 FRS2 
 chr12 
 69925835 
 RAB3IP 
 chr12 
 70193988 
  3  
  87  
 4 
 599.93  
   TCGA-41-2571-01A  
 FRS2 
 chr12 
 69925835 
 RAB3IP 
 chr12 
 70193988 
  3  
  87  
 4 
 599.93  
 
    
140. chr13-chr13 fr 
 
   TCGA-06-2558-01A  
 AKAP11 
 chr13 
 42882744 
 MRPS31 
 chr13 
 41303736 
  45  
  11  
 38 
 596.19  
 
    
141. chr12-chr12 rr 
 
   TCGA-06-0129-01A  
 LOC256021 
 chr12 
 92469836 
 VEZT 
 chr12 
 95665442 
  25  
  11  
 9 
 593.39  
 
    
142. chr12-chr12 ff 
 
   TCGA-19-2624-01A  
 TSFM 
 chr12 
 58180944 
 NUP107 
 chr12 
 69103017 
  26  
  84  
 42 
 580.56  
   TCGA-19-2624-01A  
 TSFM 
 chr12 
 58186855 
 NUP107 
 chr12 
 69103017 
  154  
  89  
 189 
 592.47  
   TCGA-19-2624-01A  
 TSFM 
 chr12 
 58186855 
 NUP107 
 chr12 
 69103784 
  2  
  89  
 2 
 529.69  
 
    
143. chr4-chr4 rf 
 
   TCGA-06-0174  
 SGCB 
 chr4 
 52894895 
 FIP1L1 
 chr4 
 54319086 
  4  
  3  
 9 
 590.78  
   TCGA-06-0174-01A  
 SGCB 
 chr4 
 52894895 
 FIP1L1 
 chr4 
 54319086 
  4  
  3  
 9 
 590.78  
 
    
144. chr16-chr16 rf 
 
   TCGA-06-5856-01A  
 UNKL 
 chr16 
 1460860 
 ZNF598 
 chr16 
 2052206 
  1  
  32  
 1 
 -948.06  
   TCGA-06-5856-01A  
 UNKL 
 chr16 
 1461929 
 ZNF598 
 chr16 
 2052206 
  10  
  32  
 13 
 475.67  
   TCGA-06-5856-01A  
 UNKL 
 chr16 
 1461929 
 ZNF598 
 chr16 
 2053616 
  38  
  32  
 30 
 588.20  
 
    
145. chr9-chr9 fr 
 
   TCGA-12-3652-01A  
 NFX1 
 chr9 
 33295424 
 C9orf24 
 chr9 
 34382866 
  13  
  7  
 13 
 588.53  
 
    
146. chr7-chr7 fr 
 
   TCGA-12-0616-01A  
 AVL9 
 chr7 
 32564277 
 C7orf10 
 chr7 
 40357004 
  46  
  12  
 42 
 586.18  
 
    
147. chr2-chr2 ff 
 
   TCGA-28-2499-01A  
 DGKD 
 chr2 
 234299128 
 SPP2 
 chr2 
 234967479 
  25  
  7  
 41 
 586.81  
 
    
148. chr6-chr6 rf 
 
   TCGA-06-0129-01A  
 MDGA1 
 chr6 
 37623475 
 PTK7 
 chr6 
 43126554 
  15  
  6  
 14 
 583.26  
 
    
149. chr1-chr1 ff 
 
   TCGA-06-2565-01A  
 KIF1B 
 chr1 
 10381914 
 EPB41 
 chr1 
 29313942 
  8  
  13  
 7 
 402.99  
   TCGA-06-2565-01A  
 KIF1B 
 chr1 
 10384119 
 EPB41 
 chr1 
 29313942 
  19  
  13  
 33 
 581.29  
 
    
150. chr3-chr2 fr 
 
   TCGA-41-4097-01A  
 SLC25A26 
 chr3 
 66313802 
 IMMT 
 chr2 
 86374955 
  28  
  5  
 29 
 581.20  
 
    
151. chr2-chr2 fr 
 
   TCGA-27-1830-01A  
 PSMD1 
 chr2 
 231945027 
 DNER 
 chr2 
 230377651 
  17  
  7  
 13 
 577.02  
 
    
152. chr17-chr17 ff 
 
   TCGA-76-4926-01B  
 LRRC37BP1 
 chr17 
 28937959 
 ENSG00000185158 
 chr17 
 30344990 
  24  
  2  
 20 
 577.72  
 
    
153. chr9-chr15 rf 
 
   TCGA-06-0190-01A  
 FOCAD 
 chr9 
 20993251 
 THSD4 
 chr15 
 71810760 
  39  
  8  
 40 
 575.95  
 
    
154. chr2-chr2 rr 
 
   TCGA-14-0789-01A  
 PUM2 
 chr2 
 20487451 
 C2orf43 
 chr2 
 20885283 
  26  
  20  
 16 
 552.67  
   TCGA-14-0789-01A  
 PUM2 
 chr2 
 20490412 
 ENSG00000118961 
 chr2 
 20884682 
  21  
  20  
 15 
 574.46  
 
    
155. chr7-chr7 fr 
 
   TCGA-06-0744  
 EGFR 
 chr7 
 55087057 
 SEC61G 
 chr7 
 54825286 
  102  
  24  
 108 
 573.65  
   TCGA-06-0744-01A  
 EGFR 
 chr7 
 55087057 
 SEC61G 
 chr7 
 54825286 
  102  
  24  
 108 
 573.65  
   TCGA-06-0211-01B  
 LANCL2 
 chr7 
 55433921 
 ENSG00000228735 
 chr7 
 54643985 
  91  
  49  
 82 
 296.44  
   TCGA-06-0211-01A  
 LANCL2 
 chr7 
 55433921 
 ENSG00000228735 
 chr7 
 54643985 
  40  
  11  
 23 
 287.73  
   TCGA-06-0219-01A  
 LANCL2 
 chr7 
 55496139 
 ENSG00000228735 
 chr7 
 54643985 
  7  
  2  
 6 
 194.67  
 
    
156. chr12-chr12 rr 
 
   TCGA-06-0129-01A  
 ANKRD52 
 chr12 
 56638645 
 SH2B3 
 chr12 
 111855575 
  23  
  14  
 12 
 572.37  
 
    
157. chr1-chr1 rr 
 
   TCGA-06-0190-02A  
 CAMTA1 
 chr1 
 7309550 
 PGD 
 chr1 
 10459760 
  22  
  11  
 8 
 570.92  
   TCGA-06-0190-01A  
 CAMTA1 
 chr1 
 7309550 
 PGD 
 chr1 
 10459760 
  12  
  3  
 4 
 369.91  
 
    
158. chr12-chr12 ff 
 
   TCGA-41-2571-01A  
 RAB3IP 
 chr12 
 70188293 
 CNOT2 
 chr12 
 70737907 
  9  
  2  
 9 
 569.09  
   TCGA-41-2571-01A  
 RAB3IP 
 chr12 
 70188293 
 CNOT2 
 chr12 
 70737907 
  9  
  2  
 9 
 569.09  
 
    
159. chr8-chr8 rr 
 
   TCGA-28-5204-01A  
 ENSG00000254300 
 chr8 
 77414912 
 PMP2 
 chr8 
 82357223 
  1  
  14  
 1 
 304.22  
   TCGA-28-5204-01A  
 ENSG00000254300 
 chr8 
 77414978 
 PMP2 
 chr8 
 82357223 
  36  
  14  
 12 
 568.52  
   TCGA-28-5204-01A  
 ENSG00000253661 
 chr8 
 77509569 
 PMP2 
 chr8 
 82357223 
  14  
  7  
 11 
 457.06  
 
    
160. chr4-chr4 rf 
 
   TCGA-27-1830-01A  
 SCFD2 
 chr4 
 53751913 
 ENSG00000248869 
 chr4 
 138076343 
  32  
  8  
 24 
 566.98  
 
    
161. chr2-chr14 rf 
 
   TCGA-28-2514-01A  
 SPRED2 
 chr2 
 65659095 
 G2E3 
 chr14 
 31050281 
  31  
  8  
 29 
 566.84  
   TCGA-28-2514-01A  
 SPRED2 
 chr2 
 65659095 
 G2E3 
 chr14 
 31055923 
  2  
  8  
 2 
 325.27  
 
    
162. chr4-chr12 rr 
 
   TCGA-12-0616-01A  
 ENSG00000250546 
 chr4 
 85174071 
 TMTC3 
 chr12 
 88570092 
  32  
  21  
 41 
 564.16  
 
    
163. chr4-chr16 fr 
 
   TCGA-06-0157-01A  
 NAT8L 
 chr4 
 2062888 
 DBNDD1 
 chr16 
 90075837 
  23  
  10  
 26 
 563.77  
 
    
164. chr19-chr19 ff 
 
   TCGA-06-5415-01A  
 RPL36 
 chr19 
 5690610 
 TTYH1 
 chr19 
 54946721 
  37  
  25  
 37 
 563.34  
   TCGA-06-5415-01A  
 LONP1 
 chr19 
 5699215 
 TTYH1 
 chr19 
 54945024 
  1  
  24  
 0 
 -13.58  
   TCGA-06-5415-01A  
 LONP1 
 chr19 
 5699215 
 TTYH1 
 chr19 
 54945705 
  3  
  24  
 3 
 279.96  
   TCGA-06-5415-01A  
 LONP1 
 chr19 
 5699215 
 TTYH1 
 chr19 
 54945821 
  31  
  24  
 45 
 506.87  
 
    
165. chr15-chr9 rr 
 
   TCGA-14-1034-02B  
 ADAMTS17 
 chr15 
 100589061 
 LPAR1 
 chr9 
 113638001 
  20  
  22  
 30 
 563.40  
 
    
166. chr12-chr12 rr 
 
   TCGA-06-5859-01A  
 ENSG00000179912 
 chr12 
 57824502 
 XRCC6BP1 
 chr12 
 58335660 
  11  
  2  
 0 
 358.94  
   TCGA-41-5651-01A  
 INHBC 
 chr12 
 57844596 
 TSFM 
 chr12 
 58188060 
  3  
  15  
 2 
 528.41  
   TCGA-41-5651-01A  
 INHBE 
 chr12 
 57849150 
 TSFM 
 chr12 
 58186855 
  12  
  15  
 6 
 16.70  
   TCGA-41-5651-01A  
 INHBE 
 chr12 
 57849252 
 TSFM 
 chr12 
 58186855 
  1  
  15  
 1 
 88.02  
   TCGA-41-5651-01A  
 INHBE 
 chr12 
 57849526 
 TSFM 
 chr12 
 58186855 
  4  
  15  
 3 
 356.44  
   TCGA-02-2485-01A  
 INHBE 
 chr12 
 57849876 
 METTL21B 
 chr12 
 58166910 
  8  
  6  
 11 
 478.99  
   TCGA-41-5651-01A  
 INHBE 
 chr12 
 57849876 
 TSFM 
 chr12 
 58186855 
  15  
  15  
 8 
 307.09  
   TCGA-41-5651-01A  
 KIF5A 
 chr12 
 57960908 
 OS9 
 chr12 
 58090149 
  2  
  34  
 0 
 534.42  
   TCGA-41-5651-01A  
 KIF5A 
 chr12 
 57960908 
 OS9 
 chr12 
 58090155 
  53  
  34  
 59 
 553.34  
   TCGA-27-1835-01A  
 OS9 
 chr12 
 58109874 
 MARCH9 
 chr12 
 58152082 
  2  
  83  
 0 
 243.72  
   TCGA-27-1835-01A  
 OS9 
 chr12 
 58110194 
 MARCH9 
 chr12 
 58152082 
  2  
  83  
 0 
 561.03  
   TCGA-12-1597-01B  
 AGAP2 
 chr12 
 58127689 
 ENSG00000257152 
 chr12 
 58161358 
  24  
  57  
 19 
 363.76  
   TCGA-12-1597-01B  
 AGAP2 
 chr12 
 58127808 
 CYP27B1 
 chr12 
 58159979 
  84  
  57  
 81 
 512.88  
   TCGA-12-1597-01B  
 AGAP2 
 chr12 
 58127808 
 CYP27B1 
 chr12 
 58160747 
  3  
  57  
 4 
 408.57  
   TCGA-12-1597-01B  
 AGAP2 
 chr12 
 58127808 
 CYP27B1 
 chr12 
 58160826 
  6  
  57  
 5 
 444.97  
   TCGA-12-1597-01B  
 AGAP2 
 chr12 
 58127808 
 CYP27B1 
 chr12 
 58160839 
  9  
  57  
 7 
 441.85  
   TCGA-27-1835-01A  
 CTDSP2 
 chr12 
 58220128 
 ENSG00000245651 
 chr12 
 58280478 
  3  
  16  
 2 
 277.08  
   TCGA-27-1835-01A  
 CTDSP2 
 chr12 
 58220128 
 ENSG00000245651 
 chr12 
 58280558 
  6  
  16  
 7 
 469.36  
   TCGA-27-1835-01A  
 CTDSP2 
 chr12 
 58221334 
 ENSG00000245651 
 chr12 
 58280478 
  1  
  16  
 0 
 119.91  
 
    
167. chr11-chr1 fr 
 
   TCGA-06-0125-02A  
 FDX1 
 chr11 
 110327770 
 SLC35E2 
 chr1 
 1666273 
  9  
  4  
 6 
 561.50  
 
    
168. chr1-chr10 fr 
 
   TCGA-19-0957-02A  
 NFIA 
 chr1 
 61554351 
 RNLS 
 chr10 
 90122481 
  14  
  14  
 15 
 561.32  
 
    
169. chr20-chr20 rr 
 
   TCGA-06-2567-01A  
 APCDD1L-AS1 
 chr20 
 57094559 
 STX16-NPEPL1 
 chr20 
 57227142 
  3  
  17  
 3 
 423.25  
   TCGA-06-2567-01A  
 APCDD1L-AS1 
 chr20 
 57094559 
 STX16-NPEPL1 
 chr20 
 57227193 
  2  
  17  
 0 
 254.85  
   TCGA-06-2567-01A  
 APCDD1L-AS1 
 chr20 
 57098224 
 STX16-NPEPL1 
 chr20 
 57227142 
  20  
  17  
 9 
 556.24  
   TCGA-06-2567-01A  
 APCDD1L-AS1 
 chr20 
 57098224 
 STX16-NPEPL1 
 chr20 
 57227193 
  9  
  17  
 4 
 377.93  
 
    
170. chr2-chr2 rr 
 
   TCGA-06-0743-01A  
 GPN1 
 chr2 
 27869732 
 SLC4A1AP 
 chr2 
 27892278 
  1  
  17  
 0 
 -571.90  
   TCGA-06-0743-01A  
 GPN1 
 chr2 
 27870702 
 SLC4A1AP 
 chr2 
 27892253 
  27  
  17  
 18 
 554.81  
 
    
171. chr6-chr6 rf 
 
   TCGA-14-2554-01A  
 ENSG00000111860 
 chr6 
 118953615 
 SYTL3 
 chr6 
 159166511 
  16  
  11  
 20 
 552.39  
 
    
172. chr15-chr15 ff 
 
   TCGA-28-1747-01C  
 BLM 
 chr15 
 91312815 
 ENSG00000185442 
 chr15 
 93326406 
  8  
  24  
 16 
 519.24  
   TCGA-28-1747-01C  
 BLM 
 chr15 
 91312815 
 ENSG00000185442 
 chr15 
 93326414 
  12  
  24  
 21 
 550.72  
   TCGA-28-1747-01C  
 BLM 
 chr15 
 91312815 
 ENSG00000185442 
 chr15 
 93330540 
  1  
  24  
 1 
 69.46  
 
    
173. chr6-chr6 ff 
 
   TCGA-16-1045-01B  
 PLEKHG1 
 chr6 
 151122004 
 ENSG00000236627 
 chr6 
 166188340 
  13  
  16  
 20 
 549.04  
   TCGA-16-1045-01B  
 PLEKHG1 
 chr6 
 151122004 
 ENSG00000256956 
 chr6 
 166204816 
  9  
  8  
 12 
 364.60  
 
    
174. chr9-chr22 rf 
 
   TCGA-19-2625-01A  
 UBAP2 
 chr9 
 33986757 
 NCF4 
 chr22 
 37271694 
  14  
  5  
 14 
 549.79  
 
    
175. chr12-chr12 rr 
 
   TCGA-41-2571-01A  
 ENSG00000257815 
 chr12 
 70635960 
 SLC38A4 
 chr12 
 47186965 
  7  
  42  
 9 
 549.44  
   TCGA-41-2571-01A  
 ENSG00000257815 
 chr12 
 70635960 
 SLC38A4 
 chr12 
 47197576 
  9  
  11  
 10 
 354.85  
   TCGA-41-2571-01A  
 ENSG00000257815 
 chr12 
 70635960 
 SLC38A4 
 chr12 
 47186965 
  7  
  42  
 9 
 549.44  
   TCGA-41-2571-01A  
 ENSG00000257815 
 chr12 
 70635960 
 SLC38A4 
 chr12 
 47197576 
  9  
  11  
 10 
 354.85  
 
    
176. chr4-chr4 rr 
 
   TCGA-06-2559-01A  
 PDGFRA 
 chr4 
 55133455 
 TMEM165 
 chr4 
 56262562 
  17  
  14  
 33 
 548.16  
 
    
177. chr3-chr3 rr 
 
   TCGA-14-2554-01A  
 PRKAR2A 
 chr3 
 48884767 
 SPINK8 
 chr3 
 48351435 
  39  
  9  
 61 
 548.26  
   TCGA-14-2554-01A  
 PRKAR2A 
 chr3 
 48884767 
 SPINK8 
 chr3 
 48351970 
  1  
  9  
 1 
 -202.22  
 
    
178. chr12-chr12 rf 
 
   TCGA-19-5960-01A  
 NELL2 
 chr12 
 45269007 
 OS9 
 chr12 
 58109542 
  15  
  2  
 12 
 548.49  
 
    
179. chr4-chr4 fr 
 
   TCGA-06-2559-01A  
 KIAA1211 
 chr4 
 57045595 
 NMU 
 chr4 
 56496626 
  78  
  15  
 52 
 547.28  
 
    
180. chr1-chr1 rr 
 
   TCGA-14-0787-01A  
 ASH1L 
 chr1 
 155385534 
 C1orf61 
 chr1 
 156384544 
  50  
  30  
 17 
 543.66  
 
    
181. chr7-chr7 rr 
 
   TCGA-28-5208-01A  
 COBL 
 chr7 
 51203854 
 SEPT14 
 chr7 
 55886915 
  25  
  4  
 14 
 543.84  
 
    
182. chr1-chr1 rf 
 
   TCGA-32-5222-01A  
 RERE 
 chr1 
 8484661 
 HIVEP3 
 chr1 
 42384083 
  8  
  3  
 3 
 416.19  
   TCGA-32-5222-01A  
 RERE 
 chr1 
 8525984 
 HIVEP3 
 chr1 
 42312094 
  23  
  8  
 16 
 543.80  
 
    
183. chr22-chr22 rf 
 
   TCGA-41-2572-01A  
 AP1B1 
 chr22 
 29734978 
 SGSM3 
 chr22 
 40796699 
  7  
  3  
 7 
 541.58  
 
    
184. chr1-chr1 rr 
 
   TCGA-12-3650-01A  
 TMEM51 
 chr1 
 15536985 
 PLEKHM2 
 chr1 
 16045119 
  1  
  13  
 0 
 -650.95  
   TCGA-12-3650-01A  
 TMEM51 
 chr1 
 15536985 
 PLEKHM2 
 chr1 
 16046414 
  10  
  13  
 2 
 429.21  
   TCGA-12-3650-01A  
 TMEM51 
 chr1 
 15536985 
 PLEKHM2 
 chr1 
 16047882 
  1  
  13  
 1 
 -718.91  
   TCGA-12-3650-01A  
 TMEM51 
 chr1 
 15541390 
 PLEKHM2 
 chr1 
 16043310 
  1  
  13  
 1 
 -56.45  
   TCGA-12-3650-01A  
 TMEM51 
 chr1 
 15541390 
 PLEKHM2 
 chr1 
 16045119 
  3  
  13  
 1 
 319.40  
   TCGA-12-3650-01A  
 TMEM51 
 chr1 
 15541390 
 PLEKHM2 
 chr1 
 16046414 
  8  
  13  
 1 
 538.47  
   TCGA-12-3650-01A  
 TMEM51 
 chr1 
 15545821 
 PLEKHM2 
 chr1 
 16046414 
  2  
  13  
 1 
 539.58  
 
    
185. chr18-chr18 rr 
 
   TCGA-76-4929-01A  
 KCTD1 
 chr18 
 24085254 
 GALNT1 
 chr18 
 33243765 
  18  
  3  
 9 
 538.69  
 
    
186. chr4-chr4 ff 
 
   TCGA-06-0749-01A  
 DCLK2 
 chr4 
 151023963 
 LRBA 
 chr4 
 151833340 
  18  
  11  
 27 
 534.53  
 
    
187. chr9-chr9 rf 
 
   TCGA-41-3915-01A  
 POLE3 
 chr9 
 116171889 
 ENSG00000119440 
 chr9 
 136101033 
  20  
  10  
 21 
 533.35  
 
    
188. chr12-chr12 fr 
 
   TCGA-06-0129-01A  
 ARID2 
 chr12 
 46161765 
 MARS 
 chr12 
 57906746 
  6  
  17  
 6 
 401.68  
   TCGA-06-0129-01A  
 ARID2 
 chr12 
 46162287 
 MARS 
 chr12 
 57906746 
  36  
  17  
 38 
 531.18  
 
    
189. chr1-chr16 rf 
 
   TCGA-16-1045-01B  
 BAI2 
 chr1 
 32221599 
 HS3ST4 
 chr16 
 26146932 
  16  
  19  
 13 
 531.58  
   TCGA-16-1045-01B  
 BAI2 
 chr1 
 32229484 
 HS3ST4 
 chr16 
 26146932 
  3  
  19  
 3 
 -85.62  
 
    
190. chr14-chr14 fr 
 
   TCGA-32-2616-01A  
 FUT8 
 chr14 
 65922435 
 ENSG00000259146 
 chr14 
 71762172 
  10  
  6  
 14 
 531.82  
 
    
191. chr12-chr12 fr 
 
   TCGA-19-2624-01A  
 HOXC10 
 chr12 
 54379793 
 WIBG 
 chr12 
 56296138 
  1  
  26  
 0 
 487.62  
   TCGA-19-2624-01A  
 HOXC10 
 chr12 
 54379793 
 WIBG 
 chr12 
 56297263 
  11  
  26  
 11 
 528.20  
   TCGA-19-2624-01A  
 ENSG00000180806 
 chr12 
 54388799 
 WIBG 
 chr12 
 56297263 
  52  
  26  
 52 
 233.32  
   TCGA-19-2624-01A  
 ENSG00000180806 
 chr12 
 54389332 
 WIBG 
 chr12 
 56297263 
  3  
  26  
 5 
 333.45  
 
    
192. chr9-chr22 rr 
 
   TCGA-19-2625-01A  
 UBE2R2 
 chr9 
 33911961 
 MYO18B 
 chr22 
 26228963 
  25  
  8  
 26 
 527.50  
 
    
193. chr16-chr16 rf 
 
   TCGA-06-0882-01A  
 ZNF689 
 chr16 
 30616401 
 ZNF48 
 chr16 
 30409175 
  17  
  3  
 10 
 525.64  
 
    
194. chr7-chr7 rr 
 
   TCGA-32-2615-01A  
 CYP51A1 
 chr7 
 91753544 
 PPP1R9A 
 chr7 
 94854489 
  6  
  48  
 4 
 467.54  
   TCGA-32-2615-01A  
 ENSG00000240720 
 chr7 
 91768080 
 PPP1R9A 
 chr7 
 94827795 
  21  
  4  
 13 
 524.76  
 
    
195. chr9-chr9 rr 
 
   TCGA-06-0129-01A  
 TTLL11 
 chr9 
 124854965 
 GGTA1P 
 chr9 
 124222496 
  12  
  6  
 18 
 523.58  
 
    
196. chr7-chr7 rr 
 
   TCGA-06-0184-01A  
 PPP1R17 
 chr7 
 31732019 
 SRRT 
 chr7 
 100479861 
  18  
  11  
 16 
 523.11  
   TCGA-06-0184-01A  
 PPP1R17 
 chr7 
 31732019 
 SRRT 
 chr7 
 100480710 
  3  
  11  
 4 
 416.50  
 
    
197. chr1-chr1 rr 
 
   TCGA-12-0618-01A  
 KCNN3 
 chr1 
 154744450 
 DCST1 
 chr1 
 155007161 
  14  
  11  
 20 
 523.85  
 
    
198. chr1-chr1 ff 
 
   TCGA-76-4925-01A  
 ENSG00000196660 
 chr1 
 219921557 
 RNU5F-1 
 chr1 
 220231594 
  13  
  4  
 32 
 521.52  
 
    
199. chr7-chr7 rr 
 
   TCGA-14-0736-02A  
 TMEM106B 
 chr7 
 12254434 
 KLHL7 
 chr7 
 23191827 
  4  
  3  
 9 
 520.35  
 
    
200. chr7-chr7 rf 
 
   TCGA-14-0736-02A  
 GRB10 
 chr7 
 50737418 
 EEPD1 
 chr7 
 36278593 
  6  
  2  
 5 
 520.26  
 
    
201. chr17-chr17 rr 
 
   TCGA-16-1045-01B  
 GSDMA 
 chr17 
 38121739 
 THRA 
 chr17 
 38233860 
  4  
  46  
 3 
 197.52  
   TCGA-16-1045-01B  
 GSDMA 
 chr17 
 38121786 
 THRA 
 chr17 
 38233860 
  1  
  46  
 0 
 257.00  
   TCGA-16-1045-01B  
 GSDMA 
 chr17 
 38121935 
 THRA 
 chr17 
 38233190 
  7  
  46  
 5 
 461.73  
   TCGA-16-1045-01B  
 GSDMA 
 chr17 
 38121935 
 THRA 
 chr17 
 38233860 
  93  
  46  
 45 
 520.87  
 
    
202. chr12-chr6 rr 
 
   TCGA-06-2559-01A  
 CTDSP2 
 chr12 
 58240154 
 LOC100422737 
 chr6 
 107172534 
  30  
  4  
 37 
 519.48  
 
    
203. chr19-chr19 rf 
 
   TCGA-12-3653-01A  
 PTPRS 
 chr19 
 5336698 
 LRRC4B 
 chr19 
 51051798 
  8  
  5  
 9 
 519.11  
 
    
204. chr6-chr6 rr 
 
   TCGA-32-2632-01A  
 ZNRD1-AS1 
 chr6 
 29976056 
 HLA-E 
 chr6 
 30458928 
  22  
  2  
 0 
 519.65  
 
    
205. chr12-chr12 rr 
 
   TCGA-06-0129-01A  
 CAMKK2 
 chr12 
 121682942 
 NR2C1 
 chr12 
 95461166 
  21  
  5  
 28 
 518.67  
 
    
206. chr1-chr1 rr 
 
   TCGA-16-1045-01B  
 UBE4B 
 chr1 
 10238701 
 KIF1B 
 chr1 
 10292491 
  214  
  115  
 139 
 518.41  
 
    
207. chr6-chr6 rr 
 
   TCGA-06-0125-02A  
 ATXN1 
 chr6 
 16753463 
 FLJ23152 
 chr6 
 17111194 
  6  
  7  
 5 
 494.71  
   TCGA-06-0125-01A  
 ATXN1 
 chr6 
 16753463 
 FLJ23152 
 chr6 
 17111194 
  6  
  4  
 9 
 514.45  
   TCGA-06-0125-02A  
 ATXN1 
 chr6 
 16761528 
 FLJ23152 
 chr6 
 17111194 
  3  
  7  
 1 
 145.81  
 
    
208. chr12-chr12 rf 
 
   TCGA-06-0138-01A  
 LRIG3 
 chr12 
 59307762 
 ENSG00000258231 
 chr12 
 58937906 
  23  
  3  
 5 
 514.54  
 
    
209. chr2-chr3 rr 
 
   TCGA-06-0171-02A  
 KIDINS220 
 chr2 
 8952524 
 C3orf70 
 chr3 
 184801350 
  17  
  3  
 27 
 512.28  
 
    
210. chr11-chr11 rr 
 
   TCGA-06-0141-01A  
 POLA2 
 chr11 
 65049949 
 DPF2 
 chr11 
 65113829 
  21  
  8  
 22 
 510.81  
 
    
211. chr8-chr8 fr 
 
   TCGA-06-5414-01A  
 CHD7 
 chr8 
 61655655 
 TOX 
 chr8 
 59872566 
  16  
  4  
 11 
 510.60  
 
    
212. chr1-chr1 rf 
 
   TCGA-02-2486-01A  
 ENSG00000134686 
 chr1 
 33886018 
 CSMD2 
 chr1 
 34242070 
  5  
  4  
 3 
 509.11  
 
    
213. chr12-chr12 fr 
 
   TCGA-26-5135-01A  
 RAP1B 
 chr12 
 69004822 
 CTDSP2 
 chr12 
 58223378 
  17  
  3  
 16 
 -73.78  
   TCGA-26-5135-01A  
 RAP1B 
 chr12 
 69004822 
 ENSG00000245651 
 chr12 
 58265870 
  60  
  22  
 38 
 35.02  
   TCGA-26-5135-01A  
 RAP1B 
 chr12 
 69004822 
 ENSG00000245651 
 chr12 
 58280558 
  10  
  9  
 12 
 -115.61  
   TCGA-26-5135-01A  
 RAP1B 
 chr12 
 69004822 
 ENSG00000245651 
 chr12 
 58287757 
  11  
  8  
 10 
 -8.73  
   TCGA-26-5135-01A  
 RAP1B 
 chr12 
 69004831 
 CTDSP2 
 chr12 
 58223378 
  9  
  3  
 11 
 -74.97  
   TCGA-26-5135-01A  
 RAP1B 
 chr12 
 69004831 
 ENSG00000245651 
 chr12 
 58265870 
  27  
  22  
 16 
 2.35  
   TCGA-26-5135-01A  
 RAP1B 
 chr12 
 69004831 
 ENSG00000245651 
 chr12 
 58270009 
  1  
  22  
 2 
 -44.76  
   TCGA-26-5135-01A  
 RAP1B 
 chr12 
 69004831 
 ENSG00000245651 
 chr12 
 58280558 
  5  
  9  
 5 
 -121.99  
   TCGA-26-5135-01A  
 RAP1B 
 chr12 
 69004831 
 ENSG00000245651 
 chr12 
 58287757 
  9  
  8  
 6 
 -4.92  
   TCGA-06-0138-01A  
 NUP107 
 chr12 
 69121168 
 ENSG00000245651 
 chr12 
 58265870 
  41  
  67  
 42 
 508.24  
 
    
214. chr7-chr9 fr 
 
   TCGA-06-0744  
 EGFR 
 chr7 
 55087057 
 ERP44 
 chr9 
 102784507 
  30  
  15  
 32 
 507.39  
   TCGA-06-0744-01A  
 EGFR 
 chr7 
 55087057 
 ERP44 
 chr9 
 102784507 
  30  
  15  
 32 
 507.39  
 
    
215. chr16-chrY ff 
 
   TCGA-06-0132-01A  
 SRCAP 
 chr16 
 30727799 
 RPS4Y1 
 chrY 
 2722640 
  44  
  14  
 44 
 506.02  
 
    
216. chr1-chr1 rr 
 
   TCGA-06-2565-01A  
 SRRM1 
 chr1 
 24972474 
 CLIC4 
 chr1 
 25140709 
  29  
  8  
 10 
 506.05  
 
    
217. chr6-chr6 rr 
 
   TCGA-06-0129-01A  
 PPIL1 
 chr6 
 36824361 
 PGC 
 chr6 
 41710226 
  8  
  27  
 0 
 505.89  
 
    
218. chr12-chr12 fr 
 
   TCGA-19-2624-01A  
 FLJ41278 
 chr12 
 65314849 
 MDM2 
 chr12 
 69207407 
  149  
  91  
 167 
 505.46  
   TCGA-19-2624-01A  
 FLJ41278 
 chr12 
 65314849 
 MDM2 
 chr12 
 69210724 
  17  
  91  
 19 
 449.04  
   TCGA-19-2624-01A  
 FLJ41278 
 chr12 
 65315445 
 MDM2 
 chr12 
 69207407 
  2  
  91  
 3 
 -221.71  
 
    
219. chr5-chr5 ff 
 
   TCGA-28-2499-01A  
 ENSG00000249306 
 chr5 
 173944075 
 NOP16 
 chr5 
 175813840 
  18  
  13  
 31 
 504.94  
   TCGA-28-2499-01A  
 ENSG00000249306 
 chr5 
 173944075 
 NOP16 
 chr5 
 175815235 
  6  
  13  
 12 
 505.19  
   TCGA-28-2499-01A  
 ENSG00000249306 
 chr5 
 173944075 
 NOP16 
 chr5 
 175815433 
  3  
  13  
 9 
 179.35  
 
    
220. chr1-chr1 ff 
 
   TCGA-28-2513-01A  
 NBPF3 
 chr1 
 21771711 
 EPHB2 
 chr1 
 23189529 
  12  
  6  
 18 
 505.43  
 
    
221. chr12-chr12 rf 
 
   TCGA-41-2571-01A  
 CTDSP2 
 chr12 
 58240154 
 C12orf10 
 chr12 
 53699691 
  200  
  112  
 166 
 488.84  
   TCGA-41-2571-01A  
 CTDSP2 
 chr12 
 58240154 
 C12orf10 
 chr12 
 53699981 
  121  
  112  
 92 
 504.61  
   TCGA-41-2571-01A  
 CTDSP2 
 chr12 
 58240154 
 C12orf10 
 chr12 
 53699985 
  5  
  112  
 5 
 480.06  
   TCGA-41-2571-01A  
 CTDSP2 
 chr12 
 58240154 
 C12orf10 
 chr12 
 53700463 
  9  
  112  
 7 
 473.29  
   TCGA-41-2571-01A  
 CTDSP2 
 chr12 
 58240154 
 C12orf10 
 chr12 
 53699691 
  200  
  112  
 166 
 488.84  
   TCGA-41-2571-01A  
 CTDSP2 
 chr12 
 58240154 
 C12orf10 
 chr12 
 53699981 
  121  
  112  
 92 
 504.61  
   TCGA-41-2571-01A  
 CTDSP2 
 chr12 
 58240154 
 C12orf10 
 chr12 
 53699985 
  5  
  112  
 5 
 480.06  
   TCGA-41-2571-01A  
 CTDSP2 
 chr12 
 58240154 
 C12orf10 
 chr12 
 53700463 
  9  
  112  
 7 
 473.29  
 
    
222. chr12-chr12 fr 
 
   TCGA-06-0882-01A  
 TMEM132B 
 chr12 
 125856041 
 SCARB1 
 chr12 
 125332058 
  9  
  2  
 5 
 503.80  
 
    
223. chr1-chr1 ff 
 
   TCGA-06-5411-01A  
 ATP2B4 
 chr1 
 203596346 
 NFASC 
 chr1 
 204889759 
  17  
  4  
 16 
 503.81  
 
    
224. chr22-chr22 rr 
 
   TCGA-32-2638-01A  
 COMT 
 chr22 
 19948721 
 MED15 
 chr22 
 20891490 
  18  
  4  
 6 
 503.15  
 
    
225. chr12-chr12 rr 
 
   TCGA-06-0129-01A  
 LGR5 
 chr12 
 71928894 
 KIAA1033 
 chr12 
 105538649 
  15  
  4  
 0 
 502.24  
 
    
226. chr6-chr15 ff 
 
   TCGA-76-4925-01A  
 BRPF3 
 chr6 
 36185786 
 STARD9 
 chr15 
 42987940 
  12  
  3  
 10 
 502.24  
 
    
227. chr7-chr7 rf 
 
   TCGA-12-0616-01A  
 MPLKIP 
 chr7 
 40173827 
 MAGI2 
 chr7 
 77973124 
  7  
  3  
 5 
 501.54  
 
    
228. chr17-chr9 rf 
 
   TCGA-06-0219-01A  
 SRP68 
 chr17 
 74039909 
 URM1 
 chr9 
 131150094 
  18  
  9  
 17 
 500.71  
 
    
229. chr12-chr12 rr 
 
   TCGA-19-2624-01A  
 PPM1H 
 chr12 
 63171491 
 DPY19L2 
 chr12 
 64045493 
  8  
  6  
 5 
 500.65  
 
    
230. chr1-chr7 ff 
 
   TCGA-06-0649-01B  
 LEPR 
 chr1 
 66088184 
 TMEM209 
 chr7 
 129815351 
  11  
  2  
 11 
 499.78  
 
    
231. chr15-chr15 rr 
 
   TCGA-06-2562-01A  
 NARG2 
 chr15 
 60766441 
 ENSG00000128918 
 chr15 
 58413213 
  9  
  2  
 13 
 284.75  
   TCGA-06-2562-01A  
 NARG2 
 chr15 
 60768261 
 ALDH1A2 
 chr15 
 58306478 
  7  
  4  
 7 
 434.84  
   TCGA-06-2562-01A  
 NARG2 
 chr15 
 60768261 
 ENSG00000128918 
 chr15 
 58399666 
  21  
  11  
 33 
 499.48  
   TCGA-06-2562-01A  
 NARG2 
 chr15 
 60771201 
 ENSG00000128918 
 chr15 
 58399666 
  1  
  10  
 1 
 -49.20  
 
    
232. chr2-chr2 rr 
 
   TCGA-16-0846-01A  
 FARP2 
 chr2 
 242432708 
 ENSG00000115694 
 chr2 
 242434290 
  1  
  17  
 1 
 -513.38  
   TCGA-32-2615-01A  
 ING5 
 chr2 
 242662353 
 D2HGDH 
 chr2 
 242695428 
  4  
  4  
 7 
 498.97  
 
    
233. chr2-chr2 rr 
 
   TCGA-19-1787-01B  
 PGAP1 
 chr2 
 197729710 
 PLCL1 
 chr2 
 198851814 
  14  
  9  
 14 
 278.26  
   TCGA-19-1787-01B  
 PGAP1 
 chr2 
 197729710 
 PLCL1 
 chr2 
 198860094 
  10  
  9  
 7 
 498.96  
 
    
234. chr3-chr3 rr 
 
   TCGA-27-1832-01A  
 OGG1 
 chr3 
 9807500 
 CAMK1 
 chr3 
 9809439 
  2  
  45  
 0 
 240.08  
   TCGA-28-5207-01A  
 RPUSD3 
 chr3 
 9881867 
 EMC3 
 chr3 
 10005880 
  3  
  8  
 3 
 497.08  
   TCGA-14-1402-02A  
 BRK1 
 chr3 
 10167295 
 IRAK2 
 chr3 
 10264514 
  1  
  9  
 1 
 286.73  
   TCGA-14-1402-02A  
 BRK1 
 chr3 
 10167309 
 IRAK2 
 chr3 
 10264514 
  12  
  9  
 7 
 374.31  
 
    
235. chr12-chr12 ff 
 
   TCGA-06-0129-01A  
 ESPL1 
 chr12 
 53663767 
 ENSG00000060709 
 chr12 
 131132036 
  12  
  4  
 22 
 496.42  
 
    
236. chr19-chr7 fr 
 
   TCGA-32-1970-01A  
 LOC100287534 
 chr19 
 55315153 
 ENSG00000106066 
 chr7 
 29186574 
  9  
  3  
 7 
 495.95  
 
    
237. chr12-chr12 rr 
 
   TCGA-06-0129-01A  
 ARID2 
 chr12 
 46169860 
 PITPNM2 
 chr12 
 123562196 
  6  
  3  
 3 
 494.33  
 
    
238. chr20-chr20 rr 
 
   TCGA-06-0747-01A  
 TMC2 
 chr20 
 2621779 
 EBF4 
 chr20 
 2674427 
  16  
  3  
 15 
 494.58  
 
    
239. chr1-chr1 rr 
 
   TCGA-19-2619-01A  
 LINC00624 
 chr1 
 146989120 
 C1orf61 
 chr1 
 156375074 
  6  
  198  
 4 
 494.15  
 
    
240. chr12-chr12 rr 
 
   TCGA-06-0129-01A  
 SLC2A13 
 chr12 
 40345058 
 ANAPC7 
 chr12 
 110834256 
  16  
  4  
 3 
 493.78  
 
    
241. chr1-chr1 fr 
 
   TCGA-06-0158-01A  
 VAMP3 
 chr1 
 7834244 
 PARK7 
 chr1 
 8022934 
  38  
  13  
 41 
 493.28  
   TCGA-06-0158-01A  
 VAMP3 
 chr1 
 7837377 
 PARK7 
 chr1 
 8022158 
  1  
  13  
 2 
 -684.34  
 
    
242. chr7-chr7 rr 
 
   TCGA-14-0736-02A  
 ENSG00000234710 
 chr7 
 9780582 
 LINC00265 
 chr7 
 39773642 
  9  
  13  
 2 
 491.24  
 
    
243. chr1-chr1 rf 
 
   TCGA-27-2523-01A  
 NSL1 
 chr1 
 212960900 
 ENSG00000228067 
 chr1 
 212719035 
  20  
  16  
 19 
 491.91  
   TCGA-27-2523-01A  
 NSL1 
 chr1 
 212960900 
 ENSG00000228067 
 chr1 
 212727753 
  4  
  16  
 1 
 354.36  
 
    
244. chr6-chr6 rr 
 
   TCGA-32-2638-01A  
 LOC100507557 
 chr6 
 146068282 
 IGF2R 
 chr6 
 160455553 
  13  
  9  
 9 
 491.67  
 
    
245. chr22-chr22 ff 
 
   TCGA-06-0745-01A  
 SPECC1L 
 chr22 
 24709433 
 PPP6R2 
 chr22 
 50852980 
  8  
  2  
 11 
 490.86  
 
    
246. chr14-chr14 ff 
 
   TCGA-27-1835-01A  
 TTLL5 
 chr14 
 76211923 
 DIO2-AS1 
 chr14 
 80839729 
  44  
  18  
 61 
 489.67  
   TCGA-27-1835-01A  
 TTLL5 
 chr14 
 76211926 
 DIO2-AS1 
 chr14 
 80839727 
  5  
  18  
 10 
 371.13  
 
    
247. chr7-chr7 rr 
 
   TCGA-06-0184-01A  
 ENSG00000236318 
 chr7 
 17490618 
 RBM33 
 chr7 
 155538295 
  9  
  2  
 0 
 488.68  
 
    
248. chr17-chr22 fr 
 
   TCGA-14-0787-01A  
 ATP6V0A1 
 chr17 
 40620124 
 ENSG00000198125 
 chr22 
 36033254 
  1  
  11  
 0 
 226.87  
   TCGA-14-0787-01A  
 ATP6V0A1 
 chr17 
 40622235 
 ENSG00000198125 
 chr22 
 36033254 
  23  
  11  
 19 
 488.54  
 
    
249. chr12-chr12 ff 
 
   TCGA-06-0686-01A  
 CYP27B1 
 chr12 
 58158103 
 ENSG00000257354 
 chr12 
 62997347 
  16  
  5  
 23 
 487.28  
 
    
250. chr1-chr1 rr 
 
   TCGA-06-0878-01A  
 THBS3 
 chr1 
 155165754 
 GON4L 
 chr1 
 155733927 
  35  
  4  
 21 
 202.04  
   TCGA-28-5207-01A  
 ASH1L-AS1 
 chr1 
 155531943 
 YY1AP1 
 chr1 
 155640254 
  8  
  18  
 7 
 487.98  
 
    
251. chr4-chr4 rr 
 
   TCGA-06-2559-01A  
 AASDH 
 chr4 
 57217507 
 LRRC66 
 chr4 
 52864102 
  7  
  7  
 6 
 487.15  
   TCGA-06-2559-01A  
 AASDH 
 chr4 
 57219569 
 LRRC66 
 chr4 
 52869557 
  2  
  7  
 3 
 48.66  
 
    
252. chr6-chr6 ff 
 
   TCGA-06-0129-01A  
 ZFAND3 
 chr6 
 37897774 
 DNAH8 
 chr6 
 38747741 
  12  
  5  
 9 
 359.83  
   TCGA-06-0129-01A  
 ZFAND3 
 chr6 
 37897774 
 DNAH8 
 chr6 
 38754540 
  9  
  5  
 18 
 485.88  
 
    
253. chr12-chr12 rf 
 
   TCGA-06-0129-01A  
 CS 
 chr12 
 56674999 
 ENSG00000179088 
 chr12 
 103632928 
  4  
  25  
 7 
 485.22  
   TCGA-06-0129-01A  
 CS 
 chr12 
 56676203 
 ENSG00000179088 
 chr12 
 103636090 
  125  
  25  
 66 
 -24.90  
 
    
254. chr1-chr14 ff 
 
   TCGA-12-3652-01A  
 STX12 
 chr1 
 28128325 
 CEP128 
 chr14 
 81217344 
  15  
  8  
 11 
 51.49  
   TCGA-12-3652-01A  
 STX12 
 chr1 
 28128325 
 CEP128 
 chr14 
 81254314 
  11  
  4  
 20 
 474.58  
   TCGA-12-3652-01A  
 STX12 
 chr1 
 28128325 
 CEP128 
 chr14 
 81300255 
  16  
  6  
 27 
 485.30  
 
    
255. chr15-chr10 rr 
 
   TCGA-76-4925-01A  
 PEAK1 
 chr15 
 77657504 
 TM9SF3 
 chr10 
 98292946 
  6  
  6  
 8 
 485.45  
 
    
256. chr6-chr6 rr 
 
   TCGA-76-4929-01A  
 GPLD1 
 chr6 
 24433414 
 TDP2 
 chr6 
 24651296 
  8  
  3  
 4 
 484.86  
 
    
257. chr12-chr12 rr 
 
   TCGA-06-5856-01A  
 ENSG00000166881 
 chr12 
 57477350 
 LRP1 
 chr12 
 57593565 
  9  
  3  
 5 
 387.95  
   TCGA-14-1402-02A  
 ENSG00000166881 
 chr12 
 57481379 
 LRP1 
 chr12 
 57532363 
  5  
  4  
 3 
 374.45  
   TCGA-06-0219-01A  
 ENSG00000179912 
 chr12 
 57789531 
 SDR9C7 
 chr12 
 57324267 
  4  
  7  
 5 
 483.94  
   TCGA-06-0219-01A  
 ENSG00000179912 
 chr12 
 57789531 
 SDR9C7 
 chr12 
 57327950 
  6  
  7  
 5 
 481.40  
 
    
258. chr13-chr13 rr 
 
   TCGA-14-0789-01A  
 ALG11 
 chr13 
 52586644 
 UTP14C 
 chr13 
 52604101 
  12  
  6  
 8 
 483.96  
 
    
259. chr9-chr9 rr 
 
   TCGA-06-0744  
 MRPL50 
 chr9 
 104160782 
 ERP44 
 chr9 
 102822453 
  380  
  281  
 538 
 482.20  
   TCGA-06-0744-01A  
 MRPL50 
 chr9 
 104160782 
 ERP44 
 chr9 
 102822453 
  380  
  281  
 538 
 482.20  
 
    
260. chr4-chr4 rr 
 
   TCGA-06-2559-01A  
 HOPX 
 chr4 
 57547157 
 PDCL2 
 chr4 
 56448403 
  6  
  46  
 3 
 -12.58  
   TCGA-06-2559-01A  
 HOPX 
 chr4 
 57547420 
 PDCL2 
 chr4 
 56448403 
  258  
  46  
 171 
 482.24  
 
    
261. chr17-chr17 rr 
 
   TCGA-27-2524-01A  
 VAT1 
 chr17 
 41173952 
 MRC2 
 chr17 
 60708715 
  11  
  12  
 8 
 481.08  
   TCGA-27-2524-01A  
 RND2 
 chr17 
 41177976 
 MRC2 
 chr17 
 60705280 
  73  
  12  
 76 
 9.72  
 
    
262. chr19-chr16 rr 
 
   TCGA-06-5856-01A  
 RANBP3 
 chr19 
 5927978 
 PRM1 
 chr16 
 11375086 
  8  
  3  
 14 
 478.31  
 
    
263. chr12-chr12 rr 
 
   TCGA-06-0129-01A  
 NTN4 
 chr12 
 96131622 
 GLT1D1 
 chr12 
 129432002 
  19  
  6  
 14 
 476.94  
 
    
264. chr17-chr17 ff 
 
   TCGA-06-0130-01A  
 CDK12 
 chr17 
 37619369 
 CDC6 
 chr17 
 38445659 
  13  
  6  
 16 
 476.47  
 
    
265. chr3-chr3 rr 
 
   TCGA-14-1829-01A  
 ULK4 
 chr3 
 41996463 
 TRAK1 
 chr3 
 42167105 
  8  
  3  
 2 
 476.30  
 
    
266. chr1-chr5 rr 
 
   TCGA-27-1835-01A  
 SRRM1 
 chr1 
 24998672 
 CMYA5 
 chr5 
 79084926 
  7  
  4  
 13 
 476.28  
 
    
267. chr7-chr7 rf 
 
   TCGA-06-2557-01A  
 VOPP1 
 chr7 
 55618310 
 HIP1 
 chr7 
 75206786 
  12  
  11  
 5 
 475.47  
   TCGA-06-2557-01A  
 VOPP1 
 chr7 
 55618793 
 HIP1 
 chr7 
 75210552 
  8  
  11  
 7 
 -748.42  
 
    
268. chr13-chr13 rr 
 
   TCGA-12-0619-01A  
 MED4 
 chr13 
 48653979 
 HTR2A 
 chr13 
 47409773 
  6  
  4  
 13 
 475.82  
 
    
269. chr12-chr12 rf 
 
   TCGA-19-2624-01A  
 GNS 
 chr12 
 65130783 
 ENSG00000256248 
 chr12 
 67417993 
  27  
  10  
 22 
 475.92  
 
    
270. chr4-chr4 rf 
 
   TCGA-76-4928-01B  
 SCFD2 
 chr4 
 54218764 
 GSX2 
 chr4 
 54967748 
  11  
  4  
 10 
 475.13  
 
    
271. chr5-chr5 rr 
 
   TCGA-06-2564-01A  
 CCDC127 
 chr5 
 216843 
 TERT 
 chr5 
 1282738 
  17  
  10  
 2 
 473.56  
   TCGA-06-2564-01A  
 CCDC127 
 chr5 
 216843 
 TERT 
 chr5 
 1287200 
  2  
  10  
 0 
 100.16  
 
    
272. chr6-chr6 rr 
 
   TCGA-06-5418-01A  
 CEP85L 
 chr6 
 118802941 
 ROS1 
 chr6 
 117641192 
  8  
  7  
 11 
 473.65  
 
    
273. chr12-chr12 ff 
 
   TCGA-06-0238-01A  
 MARS 
 chr12 
 57898081 
 AVIL 
 chr12 
 58201770 
  7  
  21  
 16 
 472.31  
 
    
274. chr14-chr14 ff 
 
   TCGA-06-5413-01A  
 AKAP6 
 chr14 
 33243071 
 EGLN3 
 chr14 
 34419601 
  7  
  3  
 11 
 472.80  
   TCGA-06-5413-01A  
 ENSG00000151322 
 chr14 
 33404208 
 EGLN3 
 chr14 
 34409743 
  8  
  2  
 6 
 -240.55  
 
    
275. chr4-chr4 rr 
 
   TCGA-26-5134-01A  
 LNX1 
 chr4 
 54373483 
 USP46 
 chr4 
 53494329 
  23  
  6  
 37 
 468.80  
 
    
276. chr1-chr1 rr 
 
   TCGA-06-5856-01A  
 RPRD2 
 chr1 
 150443036 
 TARS2 
 chr1 
 150464964 
  7  
  3  
 6 
 467.02  
 
    
277. chr2-chr2 rr 
 
   TCGA-14-0736-02A  
 ENSG00000234198 
 chr2 
 69947905 
 ANXA4 
 chr2 
 70008705 
  17  
  6  
 1 
 467.83  
 
    
278. chr6-chr6 rr 
 
   TCGA-02-2483-01A  
 SYNE1 
 chr6 
 152607923 
 SCAF8 
 chr6 
 155138912 
  10  
  5  
 7 
 464.66  
 
    
279. chr10-chr10 fr 
 
   TCGA-06-2563-01A  
 ENTPD7 
 chr10 
 101439631 
 IDE 
 chr10 
 94250351 
  7  
  9  
 8 
 464.67  
 
    
280. chr12-chr3 rf 
 
   TCGA-06-5415-01A  
 CHD4 
 chr12 
 6710813 
 FYTTD1 
 chr3 
 197501019 
  17  
  2  
 15 
 464.84  
 
    
281. chr12-chr22 ff 
 
   TCGA-06-5856-01A  
 LRP1 
 chr12 
 57578987 
 ENSG00000253874 
 chr22 
 22412890 
  6  
  3  
 7 
 464.73  
 
    
282. chr2-chrX fr 
 
   TCGA-06-0158-01A  
 EML4 
 chr2 
 42396775 
 CASK 
 chrX 
 41420896 
  71  
  17  
 66 
 463.95  
 
    
283. chr4-chr3 rf 
 
   TCGA-06-0644-01A  
 DCTD 
 chr4 
 183836075 
 ECE2 
 chr3 
 184001572 
  32  
  14  
 31 
 463.86  
 
    
284. chr22-chr22 rr 
 
   TCGA-06-0649-01B  
 GNB1L 
 chr22 
 19841965 
 CLTCL1 
 chr22 
 19178946 
  4  
  6  
 11 
 463.39  
 
    
285. chr2-chr2 rf 
 
   TCGA-06-2561-01A  
 SERTAD2 
 chr2 
 64871491 
 SPRED2 
 chr2 
 65659095 
  16  
  7  
 12 
 462.58  
   TCGA-06-2561-01A  
 SERTAD2 
 chr2 
 64873558 
 SPRED2 
 chr2 
 65659095 
  4  
  7  
 1 
 -518.87  
 
    
286. chr1-chr1 fr 
 
   TCGA-06-2565-01A  
 SKI 
 chr1 
 2161173 
 SSU72 
 chr1 
 1500295 
  7  
  8  
 10 
 462.31  
 
    
287. chr11-chr11 ff 
 
   TCGA-06-2561-01A  
 LRP5 
 chr11 
 68207383 
 ATG16L2 
 chr11 
 72539403 
  10  
  4  
 6 
 461.48  
 
    
288. chr11-chr11 rr 
 
   TCGA-06-5410-01A  
 CTSC 
 chr11 
 88029300 
 RAB38 
 chr11 
 87883122 
  3  
  10  
 3 
 216.73  
   TCGA-06-5410-01A  
 CTSC 
 chr11 
 88033697 
 RAB38 
 chr11 
 87883122 
  6  
  10  
 7 
 420.07  
   TCGA-19-4065-02A  
 CTSC 
 chr11 
 88033697 
 RAB38 
 chr11 
 87883122 
  4  
  6  
 8 
 387.31  
   TCGA-19-4065-01A  
 CTSC 
 chr11 
 88033697 
 RAB38 
 chr11 
 87883122 
  6  
  8  
 7 
 449.02  
   TCGA-32-2616-01A  
 CTSC 
 chr11 
 88033697 
 RAB38 
 chr11 
 87883122 
  7  
  4  
 11 
 460.17  
 
    
289. chr3-chr3 rr 
 
   TCGA-12-1597-01B  
 CDC25A 
 chr3 
 48226142 
 SCN10A 
 chr3 
 38748869 
  12  
  2  
 14 
 460.99  
 
    
290. chr11-chr11 rr 
 
   TCGA-02-2486-01A  
 SYT12 
 chr11 
 66812063 
 KDM2A 
 chr11 
 66999430 
  8  
  3  
 6 
 459.46  
 
    
291. chr2-chr2 ff 
 
   TCGA-19-2629-01A  
 TAF1B 
 chr2 
 10053650 
 NHEJ1 
 chr2 
 220022953 
  53  
  17  
 65 
 459.55  
 
    
292. chr7-chr7 rr 
 
   TCGA-27-2528-01A  
 JAZF1-AS1 
 chr7 
 28280075 
 AGAP3 
 chr7 
 150784158 
  1117  
  637  
 1307 
 459.43  
 
    
293. chr4-chr4 rr 
 
   TCGA-02-0055-01A  
 LOC731424 
 chr4 
 185765275 
 SNX25 
 chr4 
 186231969 
  9  
  3  
 16 
 455.97  
 
    
294. chr4-chr4 fr 
 
   TCGA-06-2559-01A  
 KIAA1211 
 chr4 
 57045595 
 ENSG00000251264 
 chr4 
 55312592 
  23  
  17  
 16 
 455.92  
   TCGA-06-2559-01A  
 KIAA1211 
 chr4 
 57045595 
 ENSG00000251264 
 chr4 
 55312617 
  5  
  17  
 2 
 309.78  
 
    
295. chr6-chr6 rr 
 
   TCGA-06-0129-01A  
 RPS10 
 chr6 
 34390530 
 BTBD9 
 chr6 
 38139492 
  20  
  6  
 15 
 454.33  
 
    
296. chr2-chr3 fr 
 
   TCGA-06-0747-01A  
 CCDC104 
 chr2 
 55750957 
 MYLK-AS1 
 chr3 
 123312523 
  21  
  8  
 18 
 454.72  
 
    
297. chr12-chr12 rr 
 
   TCGA-06-0129-01A  
 PPFIA2 
 chr12 
 82070569 
 DPY19L2 
 chr12 
 63954441 
  9  
  3  
 8 
 453.61  
 
    
298. chr7-chr7 fr 
 
   TCGA-27-2528-01A  
 CREB5 
 chr7 
 28610154 
 KCNH2 
 chr7 
 150647507 
  3  
  30  
 5 
 453.36  
 
    
299. chr6-chr6 fr 
 
   TCGA-28-5204-01A  
 ENSG00000118482 
 chr6 
 64346054 
 EYS 
 chr6 
 64436572 
  42  
  6  
 31 
 453.37  
 
    
300. chr17-chr19 rr 
 
   TCGA-76-4925-01A  
 SRP68 
 chr17 
 74046508 
 AP1M2 
 chr19 
 10694745 
  22  
  4  
 26 
 452.62  
 
    
301. chr5-chr14 rr 
 
   TCGA-27-1835-01A  
 CLPTM1L 
 chr5 
 1344465 
 PPP2R5E 
 chr14 
 63842825 
  9  
  2  
 11 
 451.43  
 
    
302. chr5-chr5 fr 
 
   TCGA-06-2564-01A  
 TERT 
 chr5 
 1282738 
 LOC728613 
 chr5 
 1598363 
  17  
  6  
 6 
 450.54  
 
    
303. chr19-chr19 rr 
 
   TCGA-06-5415-01A  
 LONP1 
 chr19 
 5700933 
 INSR 
 chr19 
 7215509 
  7  
  8  
 8 
 444.58  
   TCGA-06-5415-01A  
 LONP1 
 chr19 
 5705782 
 INSR 
 chr19 
 7203670 
  6  
  9  
 2 
 363.09  
   TCGA-06-5415-01A  
 LONP1 
 chr19 
 5705782 
 INSR 
 chr19 
 7212548 
  3  
  12  
 10 
 450.98  
 
    
304. chr12-chr12 rf 
 
   TCGA-06-0129-01A  
 AVIL 
 chr12 
 58200142 
 HIP1R 
 chr12 
 123332577 
  13  
  3  
 14 
 448.97  
 
    
305. chr6-chr6 ff 
 
   TCGA-32-2634-01A  
 DSE 
 chr6 
 116672489 
 SOGA3 
 chr6 
 127835967 
  14  
  4  
 22 
 447.15  
 
    
306. chr7-chr7 rr 
 
   TCGA-06-0157-01A  
 SEC61G 
 chr7 
 54823471 
 ENSG00000234707 
 chr7 
 54850035 
  21  
  17  
 19 
 446.57  
 
    
307. chr14-chr14 fr 
 
   TCGA-06-0743-01A  
 LTB4R 
 chr14 
 24785571 
 STXBP6 
 chr14 
 25452889 
  5  
  3  
 7 
 446.44  
 
    
308. chr22-chr22 ff 
 
   TCGA-06-0649-01B  
 DGCR6 
 chr22 
 18898540 
 C22orf25 
 chr22 
 20039987 
  6  
  2  
 5 
 445.19  
 
    
309. chr17-chr19 ff 
 
   TCGA-19-4065-01A  
 SARM1 
 chr17 
 26727630 
 ZNF544 
 chr19 
 58761472 
  9  
  4  
 10 
 444.79  
 
    
310. chr9-chr9 rr 
 
   TCGA-32-5222-01A  
 MLLT3 
 chr9 
 20620652 
 ENSG00000205442 
 chr9 
 24545058 
  11  
  5  
 5 
 444.17  
 
    
311. chr19-chr19 fr 
 
   TCGA-14-1402-02A  
 FCGRT 
 chr19 
 50017742 
 BCAT2 
 chr19 
 49303553 
  11  
  6  
 5 
 443.16  
 
    
312. chr21-chr21 rr 
 
   TCGA-06-0645-01A  
 ENSG00000231620 
 chr21 
 20675685 
 NCAM2 
 chr21 
 22790888 
  11  
  7  
 4 
 442.60  
 
    
313. chr4-chr4 rr 
 
   TCGA-06-5416-01A  
 SCD5 
 chr4 
 83601859 
 TMEM150C 
 chr4 
 83425819 
  4  
  6  
 9 
 442.44  
 
    
314. chr19-chr19 fr 
 
   TCGA-32-1970-01A  
 SPINT2 
 chr19 
 38773463 
 CLPTM1 
 chr19 
 45488560 
  13  
  21  
 13 
 442.22  
   TCGA-32-1970-01A  
 SPINT2 
 chr19 
 38773463 
 CLPTM1 
 chr19 
 45489832 
  10  
  21  
 9 
 418.77  
   TCGA-32-1970-01A  
 SPINT2 
 chr19 
 38782980 
 CLPTM1 
 chr19 
 45489832 
  4  
  21  
 3 
 323.26  
   TCGA-32-1970-01A  
 SPINT2 
 chr19 
 38783028 
 CLPTM1 
 chr19 
 45489832 
  1  
  21  
 1 
 221.08  
   TCGA-32-1970-01A  
 SPINT2 
 chr19 
 38783065 
 CLPTM1 
 chr19 
 45490310 
  5  
  21  
 4 
 -253.97  
 
    
315. chr17-chr17 rf 
 
   TCGA-06-0221-02A  
 STX8 
 chr17 
 9395145 
 TRIM16L 
 chr17 
 18638189 
  11  
  3  
 3 
 440.20  
 
    
316. chr4-chr4 rr 
 
   TCGA-14-0781-01B  
 SCD5 
 chr4 
 83626435 
 ENSG00000145287 
 chr4 
 84039123 
  14  
  2  
 2 
 440.87  
 
    
317. chr12-chr12 ff 
 
   TCGA-06-0129-01A  
 YEATS4 
 chr12 
 69756686 
 EP400 
 chr12 
 132522226 
  2  
  5  
 6 
 439.26  
 
    
318. chr17-chr17 rf 
 
   TCGA-06-2558-01A  
 DLG4 
 chr17 
 7096263 
 ZBTB4 
 chr17 
 7379118 
  1  
  13  
 1 
 123.52  
   TCGA-06-2558-01A  
 DLG4 
 chr17 
 7096263 
 ZBTB4 
 chr17 
 7379673 
  2  
  13  
 2 
 30.91  
   TCGA-06-2558-01A  
 DLG4 
 chr17 
 7096263 
 ZBTB4 
 chr17 
 7379989 
  4  
  13  
 2 
 425.66  
   TCGA-06-2558-01A  
 ACAP1 
 chr17 
 7251057 
 WRAP53 
 chr17 
 7592332 
  14  
  11  
 10 
 439.08  
 
    
319. chr1-chr11 rf 
 
   TCGA-27-2524-01A  
 MEF2D 
 chr1 
 156438571 
 ZDHHC5 
 chr11 
 57449893 
  6  
  2  
 7 
 438.97  
 
    
320. chr19-chr19 rr 
 
   TCGA-06-5415-01A  
 MLLT1 
 chr19 
 6214852 
 GRIN2D 
 chr19 
 48941313 
  11  
  4  
 6 
 437.73  
 
    
321. chr7-chr19 rf 
 
   TCGA-32-1970-01A  
 JAZF1 
 chr7 
 28210261 
 CPT1C 
 chr19 
 50210774 
  6  
  20  
 7 
 437.64  
   TCGA-32-1970-01A  
 JAZF1 
 chr7 
 28220081 
 CPT1C 
 chr19 
 50211979 
  18  
  19  
 18 
 237.58  
 
    
322. chr4-chr11 rf 
 
   TCGA-76-4926-01B  
 LINC00290 
 chr4 
 182022720 
 IFT46 
 chr11 
 118424176 
  26  
  7  
 17 
 437.94  
 
    
323. chr16-chr16 rf 
 
   TCGA-06-0210-01A  
 CHMP1A 
 chr16 
 89715758 
 SPIRE2 
 chr16 
 89924745 
  9  
  2  
 10 
 436.14  
 
    
324. chr14-chr22 ff 
 
   TCGA-06-1804-01A  
 SLC39A9 
 chr14 
 69908982 
 ENSG00000099968 
 chr22 
 18120867 
  3  
  7  
 5 
 436.91  
 
    
325. chr2-chr2 fr 
 
   TCGA-28-2499-01A  
 GIGYF2 
 chr2 
 233613791 
 PPP1R7 
 chr2 
 242107151 
  14  
  4  
 21 
 436.16  
 
    
326. chr7-chr7 ff 
 
   TCGA-32-2615-01A  
 ENSG00000237819 
 chr7 
 92480199 
 SGCE 
 chr7 
 94248069 
  17  
  3  
 26 
 435.87  
 
    
327. chr1-chr1 rr 
 
   TCGA-27-1835-01A  
 YARS 
 chr1 
 33276191 
 RNF19B 
 chr1 
 33415374 
  7  
  3  
 4 
 434.13  
 
    
328. chr12-chr12 rf 
 
   TCGA-02-2485-01A  
 CTDSP2 
 chr12 
 58240154 
 ENSG00000245651 
 chr12 
 58284095 
  10  
  3  
 12 
 301.91  
   TCGA-16-0846-01A  
 CTDSP2 
 chr12 
 58240154 
 OS9 
 chr12 
 58088532 
  10  
  5  
 9 
 320.05  
   TCGA-16-0846-01A  
 CTDSP2 
 chr12 
 58240154 
 OS9 
 chr12 
 58109542 
  17  
  4  
 9 
 416.84  
   TCGA-16-0846-01A  
 CTDSP2 
 chr12 
 58240154 
 TSPAN31 
 chr12 
 58139527 
  8  
  8  
 8 
 431.23  
   TCGA-16-0846-01A  
 CTDSP2 
 chr12 
 58240154 
 TSPAN31 
 chr12 
 58140371 
  20  
  8  
 13 
 360.88  
 
    
329. chr1-chr1 fr 
 
   TCGA-14-0787-01A  
 ATP2B4 
 chr1 
 203596344 
 MDM4 
 chr1 
 204522701 
  14  
  9  
 11 
 431.61  
   TCGA-14-0787-01A  
 ATP2B4 
 chr1 
 203596346 
 MDM4 
 chr1 
 204518934 
  2  
  9  
 2 
 306.21  
   TCGA-14-0787-01A  
 ATP2B4 
 chr1 
 203596346 
 MDM4 
 chr1 
 204518954 
  2  
  9  
 2 
 267.61  
 
    
330. chr19-chr19 rr 
 
   TCGA-14-0787-01A  
 LPHN1 
 chr19 
 14294344 
 EMR3 
 chr19 
 14779663 
  6  
  5  
 2 
 431.93  
 
    
331. chr13-chr13 fr 
 
   TCGA-27-1831-01A  
 FREM2 
 chr13 
 39438742 
 MTRF1 
 chr13 
 41808142 
  10  
  2  
 12 
 431.05  
 
    
332. chr12-chr12 rf 
 
   TCGA-28-5209-01A  
 CCNT1 
 chr12 
 49086021 
 OS9 
 chr12 
 58109542 
  22  
  7  
 15 
 393.63  
   TCGA-28-5209-01A  
 CCNT1 
 chr12 
 49086037 
 OS9 
 chr12 
 58109542 
  4  
  7  
 5 
 365.07  
   TCGA-28-5209-01A  
 CCNT1 
 chr12 
 49086373 
 OS9 
 chr12 
 58109542 
  11  
  7  
 8 
 431.50  
 
    
333. chr12-chr12 fr 
 
   TCGA-06-0129-01A  
 NOC4L 
 chr12 
 132629149 
 SDS 
 chr12 
 113837514 
  12  
  10  
 10 
 103.78  
   TCGA-06-0129-01A  
 NOC4L 
 chr12 
 132630209 
 SDS 
 chr12 
 113837514 
  4  
  10  
 3 
 429.03  
   TCGA-06-0129-01A  
 NOC4L 
 chr12 
 132632523 
 SDS 
 chr12 
 113837514 
  2  
  10  
 1 
 226.76  
 
    
334. chr12-chr12 rf 
 
   TCGA-06-0138-01A  
 CPM 
 chr12 
 69326457 
 LYZ 
 chr12 
 69743887 
  44  
  22  
 40 
 429.95  
   TCGA-06-0138-01A  
 CPM 
 chr12 
 69326457 
 LYZ 
 chr12 
 69746932 
  1  
  22  
 0 
 324.82  
 
    
335. chr4-chr4 fr 
 
   TCGA-27-1830-01A  
 PIGG 
 chr4 
 502758 
 SPINK2 
 chr4 
 57676349 
  16  
  7  
 12 
 429.14  
 
    
336. chr5-chr5 ff 
 
   TCGA-14-0790-01B  
 FAF2 
 chr5 
 175927440 
 ENSG00000248484 
 chr5 
 176150319 
  12  
  13  
 12 
 428.71  
 
    
337. chr10-chr10 fr 
 
   TCGA-76-4929-01A  
 ADO 
 chr10 
 64565548 
 REEP3 
 chr10 
 65349209 
  7  
  2  
 8 
 428.73  
 
    
338. chr12-chr12 fr 
 
   TCGA-06-0129-01A  
 EFCAB4B 
 chr12 
 3792568 
 PITPNM2 
 chr12 
 123562202 
  3  
  6  
 2 
 -515.80  
   TCGA-06-0129-01A  
 EFCAB4B 
 chr12 
 3794245 
 PITPNM2 
 chr12 
 123562213 
  6  
  6  
 8 
 427.50  
 
    
339. chr12-chr12 rr 
 
   TCGA-06-0125-02A  
 SPATS2 
 chr12 
 49854552 
 COX14 
 chr12 
 50506083 
  4  
  2  
 8 
 323.65  
   TCGA-06-0125-01A  
 SPATS2 
 chr12 
 49854552 
 COX14 
 chr12 
 50506083 
  4  
  9  
 8 
 425.60  
   TCGA-06-1804-01A  
 TMBIM6 
 chr12 
 50146246 
 DIP2B 
 chr12 
 50899022 
  20  
  5  
 22 
 236.14  
 
    
340. chr12-chr12 rf 
 
   TCGA-19-2624-01A  
 PPM1H 
 chr12 
 63182005 
 ENSG00000256248 
 chr12 
 67359560 
  17  
  5  
 9 
 414.44  
   TCGA-19-2624-01A  
 PPM1H 
 chr12 
 63182005 
 ENSG00000256248 
 chr12 
 67417993 
  6  
  4  
 9 
 425.15  
   TCGA-19-2624-01A  
 PPM1H 
 chr12 
 63182005 
 ENSG00000257083 
 chr12 
 67435904 
  13  
  4  
 5 
 397.51  
   TCGA-19-2624-01A  
 PPM1H 
 chr12 
 63213554 
 ENSG00000257083 
 chr12 
 67462829 
  5  
  7  
 3 
 155.72  
 
    
341. chr7-chr7 rr 
 
   TCGA-06-0184-01A  
 CACNA2D1 
 chr7 
 81799865 
 CPED1 
 chr7 
 120797851 
  9  
  3  
 0 
 424.45  
 
    
342. chr5-chr5 rr 
 
   TCGA-02-2486-01A  
 ENSG00000253959 
 chr5 
 173069540 
 CPEB4 
 chr5 
 173317860 
  10  
  4  
 2 
 422.73  
 
    
343. chr12-chr12 rr 
 
   TCGA-06-5856-01A  
 EFCAB4B 
 chr12 
 3782611 
 C12orf4 
 chr12 
 4600399 
  8  
  8  
 9 
 422.94  
 
    
344. chr7-chr7 ff 
 
   TCGA-19-2619-01A  
 WBSCR17 
 chr7 
 70800718 
 PPP1R9A 
 chr7 
 94827660 
  13  
  2  
 22 
 422.41  
 
    
345. chr12-chr12 ff 
 
   TCGA-06-0129-01A  
 ETV6 
 chr12 
 12044530 
 KIAA0528 
 chr12 
 22635490 
  6  
  2  
 10 
 420.23  
 
    
346. chr7-chr7 rf 
 
   TCGA-06-0184-01A  
 IMMP2L 
 chr7 
 110526648 
 DYNC1I1 
 chr7 
 95657486 
  8  
  5  
 8 
 420.78  
 
    
347. chr7-chr7 rf 
 
   TCGA-12-3652-01A  
 VOPP1 
 chr7 
 55639963 
 ENSG00000234707 
 chr7 
 54850284 
  35  
  141  
 31 
 321.78  
   TCGA-12-3652-01A  
 VOPP1 
 chr7 
 55639963 
 ENSG00000234707 
 chr7 
 54850800 
  632  
  141  
 611 
 274.82  
   TCGA-27-1832-01A  
 ENSG00000231317 
 chr7 
 55744392 
 VSTM2A 
 chr7 
 54612314 
  9  
  6  
 7 
 420.61  
 
    
348. chr14-chr14 ff 
 
   TCGA-15-0742-01A  
 NUBPL 
 chr14 
 32068556 
 SNX6 
 chr14 
 35036891 
  6  
  3  
 7 
 418.41  
 
    
349. chr3-chr11 ff 
 
   TCGA-26-5135-01A  
 LMLN 
 chr3 
 197751656 
 MAP6 
 chr11 
 75355935 
  5  
  2  
 6 
 418.58  
 
    
350. chr6-chr18 rr 
 
   TCGA-14-1034-02B  
 FILIP1 
 chr6 
 76167877 
 TUBB6 
 chr18 
 12311051 
  1  
  8  
 4 
 417.23  
 
    
351. chr15-chr19 fr 
 
   TCGA-06-2570-01A  
 THSD4 
 chr15 
 71447375 
 KDM4B 
 chr19 
 5047679 
  5  
  5  
 3 
 360.29  
   TCGA-06-2570-01A  
 THSD4 
 chr15 
 71458308 
 KDM4B 
 chr19 
 5061235 
  10  
  3  
 8 
 416.74  
 
    
352. chr4-chr4 rr 
 
   TCGA-19-1390-01A  
 USP46 
 chr4 
 53524340 
 LOC152578 
 chr4 
 53665475 
  2  
  8  
 1 
 416.28  
   TCGA-19-1390-01A  
 USP46 
 chr4 
 53525281 
 LOC152578 
 chr4 
 53665475 
  13  
  8  
 7 
 318.50  
 
    
353. chr19-chr19 fr 
 
   TCGA-32-1970-01A  
 AP2A1 
 chr19 
 50285872 
 ZNF135 
 chr19 
 58575295 
  81  
  36  
 96 
 415.28  
 
    
354. chr6-chr6 ff 
 
   TCGA-06-0129-01A  
 SYNGAP1 
 chr6 
 33393679 
 HSP90AB1 
 chr6 
 44220781 
  14  
  7  
 14 
 413.32  
 
    
355. chr17-chr6 fr 
 
   TCGA-06-0221-02A  
 MAP2K4 
 chr17 
 11999010 
 HCG17 
 chr6 
 30264013 
  4  
  4  
 4 
 412.31  
 
    
356. chr1-chr14 rr 
 
   TCGA-08-0386-01A  
 SRM 
 chr1 
 11118856 
 SMEK1 
 chr14 
 91957145 
  12  
  5  
 17 
 412.84  
 
    
357. chr4-chr12 rf 
 
   TCGA-06-0178-01A  
 SORBS2 
 chr4 
 186578577 
 OS9 
 chr12 
 58109542 
  5  
  2  
 7 
 411.99  
 
    
358. chr7-chr7 rf 
 
   TCGA-27-1832-01A  
 ENSG00000235664 
 chr7 
 22950728 
 FAM126A 
 chr7 
 23015828 
  20  
  11  
 8 
 411.29  
 
    
359. chr12-chr12 fr 
 
   TCGA-06-5856-01A  
 TSFM 
 chr12 
 58193702 
 TMEM120B 
 chr12 
 122150869 
  63  
  18  
 73 
 410.17  
 
    
360. chr11-chr11 rr 
 
   TCGA-27-2524-01A  
 STX5 
 chr11 
 62592188 
 SLC3A2 
 chr11 
 62652881 
  1  
  9  
 1 
 -46.12  
   TCGA-27-2524-01A  
 STX5 
 chr11 
 62592322 
 SLC3A2 
 chr11 
 62652828 
  20  
  9  
 27 
 409.15  
 
    
361. chr1-chr1 rf 
 
   TCGA-27-2523-01A  
 GNG4 
 chr1 
 235731500 
 LYST 
 chr1 
 235840390 
  11  
  7  
 14 
 313.57  
   TCGA-27-2523-01A  
 GNG4 
 chr1 
 235746312 
 LYST 
 chr1 
 235840390 
  8  
  5  
 8 
 407.23  
 
    
362. chr12-chr12 rr 
 
   TCGA-06-0129-01A  
 PITPNM2 
 chr12 
 123561896 
 C12orf65 
 chr12 
 123718077 
  14  
  4  
 11 
 406.28  
 
    
363. chr12-chr12 fr 
 
   TCGA-06-0129-01A  
 LOH12CR1 
 chr12 
 12588718 
 DRAM1 
 chr12 
 102302162 
  8  
  2  
 11 
 404.09  
 
    
364. chr2-chr2 fr 
 
   TCGA-06-2561-01A  
 MFSD6 
 chr2 
 191302286 
 CALCRL 
 chr2 
 188228228 
  6  
  3  
 4 
 403.11  
 
    
365. chr11-chr7 fr 
 
   TCGA-12-0619-01A  
 CAPRIN1 
 chr11 
 34093296 
 RNF216 
 chr7 
 5684900 
  10  
  3  
 11 
 403.27  
 
    
366. chr16-chr16 rf 
 
   TCGA-06-5856-01A  
 LUC7L 
 chr16 
 249060 
 IFT140 
 chr16 
 1629275 
  10  
  4  
 9 
 402.69  
 
    
367. chr19-chr19 rr 
 
   TCGA-26-5136-01B  
 FBXW9 
 chr19 
 12805407 
 PRDX2 
 chr19 
 12912083 
  8  
  11  
 7 
 401.83  
 
    
368. chr7-chr15 rr 
 
   TCGA-76-4926-01B  
 ZYX 
 chr7 
 143078652 
 KIAA1199 
 chr15 
 81224379 
  6  
  10  
 7 
 401.08  
   TCGA-76-4926-01B  
 ZYX 
 chr7 
 143085311 
 KIAA1199 
 chr15 
 81224379 
  9  
  10  
 10 
 383.49  
 
    
369. chr9-chr9 rr 
 
   TCGA-28-5204-01A  
 FAM102A 
 chr9 
 130742270 
 PTGES2 
 chr9 
 130887719 
  8  
  2  
 4 
 399.10  
 
    
370. chr12-chr12 ff 
 
   TCGA-06-0129-01A  
 GNB3 
 chr12 
 6954965 
 METAP2 
 chr12 
 95869846 
  4  
  3  
 9 
 398.99  
 
    
371. chr4-chr4 ff 
 
   TCGA-26-5135-01A  
 PDGFRA 
 chr4 
 55141139 
 KDR 
 chr4 
 55976038 
  9  
  4  
 17 
 398.87  
 
    
372. chr22-chr22 ff 
 
   TCGA-06-0649-01B  
 DGCR5 
 chr22 
 18964206 
 SLC25A1 
 chr22 
 19165163 
  13  
  6  
 14 
 397.07  
 
    
373. chr7-chr19 ff 
 
   TCGA-19-0957-02A  
 EXOC4 
 chr7 
 133002143 
 MIA-RAB4B 
 chr19 
 41285923 
  4  
  3  
 6 
 397.07  
 
    
374. chr21-chr22 ff 
 
   TCGA-32-2634-01A  
 HUNK 
 chr21 
 33260208 
 C22orf13 
 chr22 
 24950916 
  2  
  6  
 6 
 92.27  
   TCGA-32-2634-01A  
 HUNK 
 chr21 
 33260208 
 ENSG00000138867 
 chr22 
 24951582 
  4  
  6  
 5 
 397.46  
 
    
375. chr15-chr15 rr 
 
   TCGA-06-0152-02A  
 PLEKHO2 
 chr15 
 65152092 
 ANKDD1A 
 chr15 
 65234761 
  7  
  6  
 5 
 396.15  
 
    
376. chr12-chr12 rf 
 
   TCGA-32-1980-01A  
 DBX2 
 chr12 
 45420551 
 HDAC7 
 chr12 
 48191166 
  5  
  5  
 4 
 190.37  
   TCGA-32-1980-01A  
 DBX2 
 chr12 
 45437254 
 HDAC7 
 chr12 
 48191166 
  11  
  2  
 10 
 396.79  
 
    
377. chr11-chr15 ff 
 
   TCGA-06-0174  
 ZNF143 
 chr11 
 9501092 
 C15orf41 
 chr15 
 36936478 
  8  
  2  
 8 
 395.86  
   TCGA-06-0174-01A  
 ZNF143 
 chr11 
 9501092 
 C15orf41 
 chr15 
 36936478 
  8  
  2  
 8 
 395.86  
 
    
378. chr1-chr1 ff 
 
   TCGA-06-0125-02A  
 ENSG00000235545 
 chr1 
 63154322 
 ATG4C 
 chr1 
 63269389 
  4  
  6  
 5 
 260.98  
   TCGA-06-0649-01B  
 ENSG00000235545 
 chr1 
 63154322 
 ATG4C 
 chr1 
 63269389 
  4  
  4  
 6 
 194.35  
   TCGA-28-2509-01A  
 ENSG00000235545 
 chr1 
 63154322 
 ATG4C 
 chr1 
 63269389 
  3  
  5  
 4 
 289.55  
   TCGA-06-0125-02A  
 ENSG00000235545 
 chr1 
 63154394 
 ATG4C 
 chr1 
 63269389 
  4  
  6  
 4 
 369.14  
   TCGA-06-0649-01B  
 ENSG00000235545 
 chr1 
 63154394 
 ATG4C 
 chr1 
 63269389 
  4  
  4  
 7 
 328.71  
   TCGA-14-1034-01A  
 ENSG00000235545 
 chr1 
 63154394 
 ATG4C 
 chr1 
 63269389 
  5  
  7  
 6 
 269.06  
   TCGA-32-2634-01A  
 ENSG00000235545 
 chr1 
 63154394 
 ATG4C 
 chr1 
 63269389 
  3  
  7  
 3 
 393.49  
 
    
379. chr3-chr22 rr 
 
   TCGA-19-1390-01A  
 LSAMP 
 chr3 
 115738361 
 CELSR1 
 chr22 
 46835307 
  8  
  3  
 10 
 393.82  
 
    
380. chr12-chr12 rr 
 
   TCGA-26-5132-01A  
 ENSG00000127324 
 chr12 
 71603759 
 TBC1D15 
 chr12 
 72233590 
  7  
  5  
 9 
 -1200.23  
   TCGA-26-5132-01A  
 ENSG00000127324 
 chr12 
 71603759 
 TBC1D15 
 chr12 
 72235010 
  6  
  5  
 5 
 393.67  
 
    
381. chr22-chr22 rr 
 
   TCGA-06-0745-01A  
 CHEK2 
 chr22 
 29137554 
 PPP6R2 
 chr22 
 50878477 
  9  
  2  
 13 
 392.92  
 
    
382. chr10-chr10 fr 
 
   TCGA-12-0619-01A  
 ENSG00000204740 
 chr10 
 19546699 
 SKIDA1 
 chr10 
 21812799 
  7  
  2  
 11 
 392.58  
 
    
383. chr16-chr16 fr 
 
   TCGA-41-5651-01A  
 CLEC16A 
 chr16 
 11076847 
 TXNDC11 
 chr16 
 11778100 
  13  
  3  
 16 
 392.95  
 
    
384. chr7-chr7 ff 
 
   TCGA-06-5859-01A  
 ENSG00000106066 
 chr7 
 29186574 
 CHN2 
 chr7 
 29394239 
  6  
  4  
 9 
 391.52  
 
    
385. chr9-chr9 fr 
 
   TCGA-27-2521-01A  
 FOCAD 
 chr9 
 20867010 
 MOB3B 
 chr9 
 27359233 
  5  
  5  
 5 
 390.51  
 
    
386. chr7-chr7 ff 
 
   TCGA-32-2634-01A  
 PHTF2 
 chr7 
 77542929 
 MAGI2 
 chr7 
 78119077 
  23  
  3  
 28 
 390.66  
 
    
387. chr12-chr12 fr 
 
   TCGA-06-0129-01A  
 ENSG00000188906 
 chr12 
 40601933 
 TCTN1 
 chr12 
 111085697 
  7  
  7  
 8 
 389.41  
   TCGA-06-0129-01A  
 ENSG00000188906 
 chr12 
 40604044 
 TCTN1 
 chr12 
 111085782 
  3  
  7  
 4 
 176.13  
 
    
388. chr12-chr12 fr 
 
   TCGA-06-0129-01A  
 SLC4A8 
 chr12 
 51901656 
 PPP1R12A 
 chr12 
 80304755 
  7  
  5  
 9 
 388.06  
 
    
389. chr4-chr4 ff 
 
   TCGA-27-1830-01A  
 DCUN1D4 
 chr4 
 52765543 
 NR3C2 
 chr4 
 149342591 
  14  
  3  
 17 
 388.69  
 
    
390. chr14-chr14 ff 
 
   TCGA-12-3653-01A  
 FAM179B 
 chr14 
 45501578 
 RTN1 
 chr14 
 60163212 
  10  
  3  
 8 
 385.39  
 
    
391. chr7-chr7 fr 
 
   TCGA-06-2557-01A  
 FOXK1 
 chr7 
 4722498 
 IKZF1 
 chr7 
 50456276 
  15  
  7  
 13 
 383.29  
 
    
392. chr12-chr12 ff 
 
   TCGA-06-5856-01A  
 TSFM 
 chr12 
 58180073 
 IFNG 
 chr12 
 68549194 
  312  
  146  
 462 
 329.39  
   TCGA-06-5856-01A  
 TSFM 
 chr12 
 58180073 
 IFNG 
 chr12 
 68549767 
  3  
  146  
 1 
 -117.52  
   TCGA-06-5856-01A  
 TSFM 
 chr12 
 58180073 
 IFNG 
 chr12 
 68553493 
  22  
  150  
 29 
 381.01  
 
    
393. chr4-chr12 fr 
 
   TCGA-06-2559-01A  
 PAICS 
 chr4 
 57302493 
 ENSG00000257541 
 chr12 
 58483380 
  25  
  9  
 23 
 380.49  
 
    
394. chr6-chr6 rf 
 
   TCGA-06-0129-01A  
 USP49 
 chr6 
 41839301 
 KCTD20 
 chr6 
 36437828 
  10  
  5  
 5 
 378.99  
 
    
395. chr10-chr10 ff 
 
   TCGA-06-2563-01A  
 EXOC6 
 chr10 
 94729199 
 ABLIM1 
 chr10 
 116304763 
  9  
  3  
 12 
 378.58  
 
    
396. chr2-chr3 ff 
 
   TCGA-06-5414-01A  
 TMEM131 
 chr2 
 98584861 
 PLA1A 
 chr3 
 119340850 
  18  
  7  
 16 
 378.87  
 
    
397. chr9-chr4 rf 
 
   TCGA-06-0129-01A  
 STRBP 
 chr9 
 125909133 
 ENSG00000249618 
 chr4 
 130783722 
  4  
  4  
 4 
 377.60  
 
    
398. chr12-chr12 rr 
 
   TCGA-26-5135-01A  
 NUP107 
 chr12 
 69114897 
 BEST3 
 chr12 
 70084571 
  6  
  6  
 5 
 377.29  
 
    
399. chr12-chr12 rr 
 
   TCGA-06-0138-01A  
 ENSG00000174099 
 chr12 
 65874758 
 FRS2 
 chr12 
 69864309 
  23  
  405  
 14 
 -6.87  
   TCGA-06-0138-01A  
 ENSG00000174099 
 chr12 
 65881778 
 FRS2 
 chr12 
 69864311 
  16  
  407  
 1 
 376.05  
   TCGA-06-0138-01A  
 ENSG00000250748 
 chr12 
 65898157 
 FRS2 
 chr12 
 69864309 
  7  
  11  
 10 
 244.85  
 
    
400. chr19-chr19 rr 
 
   TCGA-06-1804-01A  
 RAVER1 
 chr19 
 10439317 
 DOCK6 
 chr19 
 11356553 
  10  
  3  
 3 
 376.13  
 
    
401. chr9-chr9 ff 
 
   TCGA-19-2625-01A  
 FAM189A2 
 chr9 
 71990779 
 LOC158435 
 chr9 
 98851748 
  7  
  4  
 6 
 376.34  
 
    
402. chr2-chr2 rf 
 
   TCGA-14-0871-01A  
 SNRNP200 
 chr2 
 96948938 
 CIAO1 
 chr2 
 96935005 
  6  
  3  
 6 
 374.97  
 
    
403. chr12-chr12 fr 
 
   TCGA-19-5960-01A  
 TSFM 
 chr12 
 58180944 
 ENSG00000133773 
 chr12 
 82617472 
  6  
  2  
 4 
 374.07  
 
    
404. chr16-chr16 rr 
 
   TCGA-06-5856-01A  
 AXIN1 
 chr16 
 359972 
 ENSG00000182831 
 chr16 
 9215444 
  5  
  3  
 7 
 373.87  
 
    
405. chr16-chr16 rr 
 
   TCGA-27-2519-01A  
 GLYR1 
 chr16 
 4881979 
 SEC14L5 
 chr16 
 5029110 
  9  
  27  
 12 
 373.65  
 
    
406. chr10-chr10 ff 
 
   TCGA-12-0619-01A  
 GATA3 
 chr10 
 8096701 
 NRP1 
 chr10 
 33619635 
  84  
  32  
 97 
 140.67  
   TCGA-12-0619-01A  
 GATA3 
 chr10 
 8101920 
 NRP1 
 chr10 
 33619635 
  10  
  32  
 15 
 -85.03  
   TCGA-12-0619-01A  
 GATA3 
 chr10 
 8106100 
 NRP1 
 chr10 
 33578357 
  5  
  5  
 8 
 372.09  
 
    
407. chr19-chr19 rf 
 
   TCGA-19-1390-01A  
 MLLT1 
 chr19 
 6271749 
 C3 
 chr19 
 6686011 
  17  
  10  
 12 
 369.61  
 
    
408. chr17-chr10 rf 
 
   TCGA-06-2563-01A  
 DHRS13 
 chr17 
 27228007 
 ENSG00000237949 
 chr10 
 60760988 
  47  
  10  
 36 
 365.37  
 
    
409. chr21-chr21 rf 
 
   TCGA-06-5856-01A  
 HLCS 
 chr21 
 38334335 
 LINC00478 
 chr21 
 17859809 
  16  
  4  
 7 
 364.50  
 
    
410. chr12-chr12 fr 
 
   TCGA-06-0129-01A  
 DIP2B 
 chr12 
 51065279 
 ENSG00000130787 
 chr12 
 123319552 
  7  
  14  
 8 
 363.04  
   TCGA-06-0129-01A  
 DIP2B 
 chr12 
 51065279 
 HIP1R 
 chr12 
 123320189 
  33  
  14  
 29 
 241.85  
   TCGA-06-0129-01A  
 DIP2B 
 chr12 
 51065279 
 HIP1R 
 chr12 
 123323766 
  2  
  18  
 2 
 269.31  
   TCGA-06-0129-01A  
 DIP2B 
 chr12 
 51066246 
 HIP1R 
 chr12 
 123320189 
  1  
  14  
 1 
 80.37  
 
    
411. chr3-chr3 rr 
 
   TCGA-12-3652-01A  
 XXYLT1 
 chr3 
 194947437 
 ENSG00000230102 
 chr3 
 193733423 
  12  
  4  
 15 
 361.87  
 
    
412. chr7-chr7 ff 
 
   TCGA-06-0211-01B  
 LANCL2 
 chr7 
 55433921 
 ENSG00000224947 
 chr7 
 56627365 
  42  
  30  
 41 
 281.45  
   TCGA-06-0211-01A  
 LANCL2 
 chr7 
 55433921 
 ENSG00000224947 
 chr7 
 56627365 
  25  
  11  
 31 
 359.61  
 
    
413. chr7-chr7 rf 
 
   TCGA-12-0616-01A  
 TOMM7 
 chr7 
 22857618 
 PLXNA4 
 chr7 
 132236011 
  28  
  6  
 18 
 359.97  
 
    
414. chr5-chr12 ff 
 
   TCGA-26-5139-01A  
 APC 
 chr5 
 112043578 
 KRT6A 
 chr12 
 52885317 
  10  
  4  
 15 
 359.80  
   TCGA-26-5139-01A  
 APC 
 chr5 
 112073621 
 KRT6A 
 chr12 
 52885317 
  4  
  2  
 10 
 -808.66  
 
    
415. chr7-chr12 fr 
 
   TCGA-28-1747-01C  
 VWC2 
 chr7 
 49940810 
 SLCO1C1 
 chr12 
 20876187 
  8  
  2  
 6 
 359.19  
 
    
416. chr1-chr15 rr 
 
   TCGA-06-5415-01A  
 DAB1 
 chr1 
 57984916 
 RYR3 
 chr15 
 33603296 
  22  
  3  
 25 
 355.78  
   TCGA-06-5415-01A  
 DAB1 
 chr1 
 58117888 
 RYR3 
 chr15 
 33603296 
  12  
  2  
 14 
 310.31  
 
    
417. chr21-chr21 ff 
 
   TCGA-08-0386-01A  
 IGSF5 
 chr21 
 41165539 
 PCP4 
 chr21 
 41270400 
  5  
  3  
 4 
 354.19  
 
    
418. chr3-chr3 ff 
 
   TCGA-14-1829-01A  
 ZIC4 
 chr3 
 147111728 
 ENSG00000152977 
 chr3 
 147225727 
  6  
  2  
 13 
 354.18  
   TCGA-27-1831-01A  
 ZIC4 
 chr3 
 147111728 
 ENSG00000152977 
 chr3 
 147225727 
  3  
  5  
 5 
 353.84  
 
    
419. chr2-chr2 rr 
 
   TCGA-16-1045-01B  
 DNER 
 chr2 
 230271947 
 ENSG00000232023 
 chr2 
 229353508 
  4  
  4  
 8 
 353.97  
 
    
420. chr7-chr7 rf 
 
   TCGA-28-1747-01C  
 SEPT14 
 chr7 
 55874883 
 PSPH 
 chr7 
 56099621 
  43  
  8  
 32 
 161.00  
   TCGA-28-1747-01C  
 SEPT14 
 chr7 
 55896455 
 PSPH 
 chr7 
 56099621 
  14  
  2  
 8 
 352.65  
 
    
421. chrX-chrX rr 
 
   TCGA-19-1787-01B  
 MTMR8 
 chrX 
 63525445 
 MSN 
 chrX 
 64887719 
  70  
  21  
 24 
 349.37  
   TCGA-19-1787-01B  
 MTMR8 
 chrX 
 63525448 
 MSN 
 chrX 
 64887719 
  17  
  21  
 5 
 278.04  
 
    
422. chr12-chr12 rr 
 
   TCGA-06-0238-01A  
 FOXM1 
 chr12 
 2977728 
 SLC2A14 
 chr12 
 7970629 
  6  
  4  
 4 
 347.52  
 
    
423. chr7-chr12 fr 
 
   TCGA-19-2624-01A  
 EGFR 
 chr7 
 55087057 
 PPM1H 
 chr12 
 63195939 
  141  
  42  
 153 
 347.51  
 
    
424. chr15-chr15 rf 
 
   TCGA-76-4925-01A  
 SLC12A6 
 chr15 
 34536174 
 PEAK1 
 chr15 
 77545013 
  11  
  5  
 7 
 347.23  
 
    
425. chr17-chr17 rr 
 
   TCGA-41-5651-01A  
 PMP22 
 chr17 
 15141597 
 ALDH3A2 
 chr17 
 19555944 
  5  
  5  
 4 
 345.40  
 
    
426. chr6-chrX rr 
 
   TCGA-06-2569-01A  
 SSR1 
 chr6 
 7310149 
 DMD 
 chrX 
 32328392 
  55  
  12  
 74 
 342.85  
   TCGA-06-2569-01A  
 SSR1 
 chr6 
 7310149 
 DMD 
 chrX 
 32328397 
  1  
  12  
 1 
 251.45  
 
    
427. chr6-chr6 ff 
 
   TCGA-06-0190-01A  
 UTRN 
 chr6 
 144750874 
 LOC100507557 
 chr6 
 146126347 
  13  
  2  
 21 
 341.21  
 
    
428. chr13-chr13 rr 
 
   TCGA-06-0210-02A  
 DLEU2 
 chr13 
 50642232 
 ENSG00000176124 
 chr13 
 51014920 
  7  
  2  
 3 
 341.39  
 
    
429. chr18-chr22 fr 
 
   TCGA-06-0129-01A  
 TWSG1 
 chr18 
 9360068 
 SGSM1 
 chr22 
 25301296 
  9  
  2  
 10 
 338.39  
 
    
430. chr7-chr7 rr 
 
   TCGA-06-0750-01A  
 LANCL2 
 chr7 
 55459485 
 ENSG00000249773 
 chr7 
 55974071 
  22  
  8  
 23 
 336.43  
 
    
431. chr12-chr12 fr 
 
   TCGA-41-2571-01A  
 TMEM19 
 chr12 
 72083483 
 TSPAN8 
 chr12 
 71551566 
  5  
  4  
 8 
 332.24  
   TCGA-41-2571-01A  
 TMEM19 
 chr12 
 72083483 
 TSPAN8 
 chr12 
 71551566 
  5  
  4  
 8 
 332.24  
 
    
432. chr5-chr5 ff 
 
   TCGA-06-0125-02A  
 ENSG00000037749 
 chr5 
 153569748 
 GALNT10 
 chr5 
 153674375 
  6  
  26  
 8 
 279.26  
   TCGA-06-0171-02A  
 ENSG00000037749 
 chr5 
 153569748 
 GALNT10 
 chr5 
 153674375 
  1  
  8  
 3 
 -277.31  
   TCGA-19-2629-01A  
 ENSG00000037749 
 chr5 
 153569748 
 GALNT10 
 chr5 
 153674375 
  2  
  11  
 1 
 331.53  
 
    
433. chr12-chr12 ff 
 
   TCGA-06-0187-01A  
 HMGA2 
 chr12 
 66232348 
 NUP107 
 chr12 
 69109406 
  6  
  3  
 10 
 329.48  
 
    
434. chr1-chr19 ff 
 
   TCGA-06-0190-02A  
 ELAVL4 
 chr1 
 50530628 
 PTPRS 
 chr19 
 5238929 
  3  
  15  
 6 
 329.68  
 
    
435. chr7-chr7 fr 
 
   TCGA-06-2557-01A  
 AKAP9 
 chr7 
 91722629 
 PON2 
 chr7 
 95045608 
  7  
  4  
 6 
 329.34  
 
    
436. chr14-chr6 fr 
 
   TCGA-27-1835-01A  
 RHOJ 
 chr14 
 63671764 
 C6orf52 
 chr6 
 10671830 
  3  
  17  
 2 
 329.16  
 
    
437. chr2-chr2 fr 
 
   TCGA-06-0141-01A  
 GIGYF2 
 chr2 
 233562101 
 ECEL1 
 chr2 
 233345522 
  8  
  5  
 6 
 -475.04  
   TCGA-06-0141-01A  
 GIGYF2 
 chr2 
 233562101 
 ECEL1 
 chr2 
 233345865 
  96  
  5  
 76 
 -139.99  
   TCGA-06-2562-01A  
 GIGYF2 
 chr2 
 233621043 
 NGEF 
 chr2 
 233750078 
  10  
  3  
 11 
 328.49  
 
    
438. chr1-chr1 ff 
 
   TCGA-19-2619-01A  
 PIP5K1A 
 chr1 
 151209238 
 NPR1 
 chr1 
 153655210 
  11  
  5  
 16 
 326.59  
 
    
439. chr9-chr9 rr 
 
   TCGA-14-1829-01A  
 PTPRD 
 chr9 
 9842697 
 ACER2 
 chr9 
 19424838 
  22  
  5  
 17 
 325.57  
 
    
440. chr12-chr12 fr 
 
   TCGA-06-5856-01A  
 RNFT2 
 chr12 
 117217435 
 TMEM120B 
 chr12 
 122150869 
  22  
  31  
 16 
 153.17  
   TCGA-06-5856-01A  
 RNFT2 
 chr12 
 117218428 
 TMEM120B 
 chr12 
 122150869 
  20  
  31  
 20 
 322.40  
   TCGA-06-5856-01A  
 RNFT2 
 chr12 
 117225168 
 TMEM120B 
 chr12 
 122150869 
  3  
  37  
 3 
 218.64  
 
    
441. chr1-chr1 ff 
 
   TCGA-06-2565-01A  
 PRKCZ 
 chr1 
 2005367 
 MTOR 
 chr1 
 11204482 
  10  
  4  
 15 
 319.26  
   TCGA-06-2565-01A  
 PRKCZ 
 chr1 
 2005367 
 MTOR 
 chr1 
 11205209 
  12  
  4  
 11 
 287.50  
 
    
442. chr2-chr21 fr 
 
   TCGA-06-0178-01A  
 ACP1 
 chr2 
 272149 
 ENSG00000237664 
 chr21 
 46758792 
  4  
  4  
 4 
 318.18  
 
    
443. chr2-chr2 rr 
 
   TCGA-19-2629-01A  
 ADAM17 
 chr2 
 9633875 
 NHEJ1 
 chr2 
 220023083 
  79  
  11  
 20 
 318.24  
 
    
444. chr10-chr10 fr 
 
   TCGA-06-0645-01A  
 RBM20 
 chr10 
 112433398 
 KCNMA1 
 chr10 
 79102804 
  6  
  4  
 7 
 313.35  
 
    
445. chr8-chr9 rf 
 
   TCGA-28-5204-01A  
 ZFAND1 
 chr8 
 82627221 
 CDKN2B-AS1 
 chr9 
 22029431 
  9  
  8  
 6 
 312.41  
 
    
446. chr12-chr12 rf 
 
   TCGA-41-2571-01A  
 FAM19A2 
 chr12 
 62585039 
 PCBP2 
 chr12 
 53848509 
  6  
  6  
 7 
 311.74  
   TCGA-41-2571-01A  
 FAM19A2 
 chr12 
 62585039 
 PCBP2 
 chr12 
 53848509 
  6  
  6  
 7 
 311.74  
   TCGA-41-2571-01A  
 FAM19A2 
 chr12 
 62585692 
 PCBP2 
 chr12 
 53848509 
  5  
  6  
 2 
 300.70  
   TCGA-41-2571-01A  
 FAM19A2 
 chr12 
 62585692 
 PCBP2 
 chr12 
 53848509 
  5  
  6  
 2 
 300.70  
 
    
447. chr12-chr16 rf 
 
   TCGA-06-0238-01A  
 ENSG00000245651 
 chr12 
 58284095 
 TCEB2 
 chr16 
 2825451 
  5  
  4  
 7 
 310.78  
 
    
448. chr17-chr17 rr 
 
   TCGA-14-0789-01A  
 ENSG00000249451 
 chr17 
 48581025 
 ABCC3 
 chr17 
 48757259 
  2  
  12  
 0 
 290.68  
   TCGA-14-0789-01A  
 ENSG00000249451 
 chr17 
 48583172 
 ABCC3 
 chr17 
 48755579 
  18  
  12  
 12 
 310.04  
   TCGA-14-0789-01A  
 ENSG00000249451 
 chr17 
 48583172 
 ABCC3 
 chr17 
 48757259 
  8  
  12  
 7 
 220.98  
 
    
449. chr3-chr19 ff 
 
   TCGA-32-1970-01A  
 ENSG00000169760 
 chr3 
 173115852 
 CCDC155 
 chr19 
 49916224 
  8  
  4  
 11 
 310.97  
 
    
450. chr8-chr19 rr 
 
   TCGA-06-2570-01A  
 SNTG1 
 chr8 
 51085120 
 KDM4B 
 chr19 
 5047679 
  6  
  3  
 6 
 309.39  
 
    
451. chr1-chr1 rr 
 
   TCGA-41-5651-01A  
 NEGR1 
 chr1 
 72748001 
 ZRANB2 
 chr1 
 71544390 
  2  
  3  
 12 
 307.60  
 
    
452. chr10-chr10 ff 
 
   TCGA-06-0130-01A  
 TET1 
 chr10 
 70446463 
 CCAR1 
 chr10 
 70549466 
  7  
  3  
 12 
 303.53  
 
    
453. chr7-chr19 rr 
 
   TCGA-32-1970-01A  
 RALA 
 chr7 
 39745721 
 ZNF610 
 chr19 
 52839758 
  44  
  10  
 53 
 300.34  
   TCGA-32-1970-01A  
 RALA 
 chr7 
 39745724 
 ZNF610 
 chr19 
 52839760 
  1  
  10  
 1 
 197.09  
 
    
454. chr17-chr17 fr 
 
   TCGA-14-1034-02B  
 EIF4A1 
 chr17 
 7478766 
 PHOSPHO1 
 chr17 
 47302508 
  3  
  88  
 1 
 291.18  
   TCGA-14-1034-01A  
 EIF4A1 
 chr17 
 7478766 
 PHOSPHO1 
 chr17 
 47302508 
  2  
  42  
 4 
 64.75  
 
    
455. chr1-chr1 rr 
 
   TCGA-27-2523-01A  
 LPGAT1 
 chr1 
 211952259 
 PGBD2 
 chr1 
 249210787 
  8  
  4  
 5 
 291.49  
 
    
456. chr1-chr1 rr 
 
   TCGA-32-1980-01A  
 TRMT1L 
 chr1 
 185097800 
 SWT1 
 chr1 
 185128211 
  10  
  8  
 3 
 288.37  
 
    
457. chr1-chr1 rr 
 
   TCGA-19-2619-01A  
 DNM3 
 chr1 
 172368958 
 TNFSF4 
 chr1 
 173157707 
  7  
  2  
 2 
 218.61  
   TCGA-19-2619-01A  
 DNM3 
 chr1 
 172368958 
 TNFSF4 
 chr1 
 173176323 
  9  
  3  
 0 
 280.84  
 
    
458. chr22-chr22 rr 
 
   TCGA-19-2625-01A  
 RBFOX2 
 chr22 
 36423098 
 LGALS2 
 chr22 
 37967936 
  3  
  11  
 3 
 280.58  
   TCGA-19-2625-01A  
 RBFOX2 
 chr22 
 36424287 
 LGALS2 
 chr22 
 37966741 
  5  
  11  
 0 
 32.09  
   TCGA-19-2625-01A  
 RBFOX2 
 chr22 
 36424287 
 LGALS2 
 chr22 
 37967937 
  30  
  11  
 6 
 5.41  
 
    
459. chr6-chr6 fr 
 
   TCGA-27-1835-01A  
 ENSG00000181355 
 chr6 
 10141948 
 C6orf52 
 chr6 
 10671830 
  140  
  36  
 115 
 279.00  
 
    
460. chr17-chr1 rr 
 
   TCGA-06-2569-01A  
 PSMD12 
 chr17 
 65344665 
 ENSG00000228826 
 chr1 
 121236392 
  10  
  3  
 14 
 277.59  
 
    
461. chr11-chr16 rf 
 
   TCGA-06-0125-02A  
 FAM168A 
 chr11 
 73308967 
 SNX29 
 chr16 
 12571575 
  8  
  4  
 10 
 276.77  
 
    
462. chr11-chr11 ff 
 
   TCGA-06-5415-01A  
 C11orf48 
 chr11 
 62433417 
 DPF2 
 chr11 
 65119153 
  7  
  5  
 6 
 275.84  
 
    
463. chr2-chr5 rr 
 
   TCGA-06-5415-01A  
 ARPC2 
 chr2 
 219103386 
 TXNDC15 
 chr5 
 134210219 
  81  
  47  
 111 
 274.86  
 
    
464. chr9-chr4 rr 
 
   TCGA-06-0129-01A  
 TMEM245 
 chr9 
 111868787 
 MAML3 
 chr4 
 140759907 
  8  
  5  
 15 
 271.35  
 
    
465. chr22-chr22 rr 
 
   TCGA-19-2625-01A  
 TTC28 
 chr22 
 28402253 
 DEPDC5 
 chr22 
 32154618 
  21  
  8  
 10 
 270.38  
 
    
466. chr16-chr16 fr 
 
   TCGA-06-5856-01A  
 RBFOX1 
 chr16 
 6476216 
 JMJD8 
 chr16 
 733773 
  15  
  2  
 10 
 267.76  
 
    
467. chr9-chr9 rr 
 
   TCGA-14-1402-02A  
 ABCA2 
 chr9 
 139914785 
 ENTPD2 
 chr9 
 139946799 
  1  
  10  
 0 
 254.00  
   TCGA-14-1402-02A  
 ABCA2 
 chr9 
 139914787 
 ENTPD2 
 chr9 
 139946799 
  1  
  10  
 0 
 267.97  
 
    
468. chr3-chr16 rr 
 
   TCGA-14-1034-02B  
 PPARG 
 chr3 
 12366368 
 MAPK8IP3 
 chr16 
 1810646 
  29  
  9  
 24 
 266.84  
 
    
469. chr13-chr13 ff 
 
   TCGA-06-2559-01A  
 TPT1-AS1 
 chr13 
 45944316 
 DIAPH3 
 chr13 
 60695420 
  3  
  7  
 4 
 207.96  
   TCGA-06-2559-01A  
 TPT1-AS1 
 chr13 
 45944316 
 DIAPH3 
 chr13 
 60695455 
  2  
  7  
 3 
 265.82  
 
    
470. chr7-chr11 fr 
 
   TCGA-26-5135-01A  
 CALU 
 chr7 
 128395737 
 C11orf30 
 chr11 
 76213146 
  17  
  2  
 9 
 264.61  
 
    
471. chr6-chr6 rr 
 
   TCGA-19-5960-01A  
 TIAM2 
 chr6 
 155411422 
 ARID1B 
 chr6 
 157100604 
  57  
  12  
 12 
 261.89  
 
    
472. chr3-chr3 ff 
 
   TCGA-06-0210-02A  
 ATG7 
 chr3 
 11468399 
 VGLL4 
 chr3 
 11605732 
  1  
  26  
 0 
 260.68  
 
    
473. chr12-chr12 rr 
 
   TCGA-06-0138-01A  
 LYZ 
 chr12 
 69743887 
 FRS2 
 chr12 
 69864309 
  5  
  3  
 5 
 259.98  
   TCGA-41-2571-01A  
 LYZ 
 chr12 
 69743887 
 FRS2 
 chr12 
 69864309 
  6  
  3  
 5 
 57.85  
   TCGA-41-2571-01A  
 LYZ 
 chr12 
 69743887 
 FRS2 
 chr12 
 69864309 
  6  
  3  
 5 
 57.85  
 
    
474. chr4-chr4 rf 
 
   TCGA-19-2620-01A  
 PRMT10 
 chr4 
 148593017 
 TMEM192 
 chr4 
 166021779 
  23  
  13  
 20 
 258.43  
 
    
475. chr16-chr16 rr 
 
   TCGA-06-0141-01A  
 LOC595101 
 chr16 
 30346295 
 DCTPP1 
 chr16 
 30435853 
  8  
  8  
 1 
 250.46  
 
    
476. chr2-chr2 rr 
 
   TCGA-06-0178-01A  
 WDFY1 
 chr2 
 224809864 
 LRRFIP1 
 chr2 
 238667445 
  9  
  4  
 3 
 250.99  
   TCGA-06-0178-01A  
 WDFY1 
 chr2 
 224809864 
 ENSG00000124831 
 chr2 
 238698426 
  27  
  18  
 9 
 208.05  
 
    
477. chr14-chr14 ff 
 
   TCGA-27-1835-01A  
 ENSG00000139865 
 chr14 
 38095591 
 DCAF5 
 chr14 
 69583082 
  3  
  5  
 4 
 250.02  
 
    
478. chr12-chr12 rf 
 
   TCGA-19-5960-01A  
 ENSG00000123411 
 chr12 
 56408411 
 OS9 
 chr12 
 58088532 
  3  
  40  
 2 
 -652.01  
   TCGA-19-5960-01A  
 IKZF4 
 chr12 
 56415164 
 OS9 
 chr12 
 58088532 
  5  
  43  
 2 
 249.56  
   TCGA-19-5960-01A  
 IKZF4 
 chr12 
 56415346 
 OS9 
 chr12 
 58088488 
  1  
  43  
 0 
 113.71  
   TCGA-19-5960-01A  
 IKZF4 
 chr12 
 56415346 
 OS9 
 chr12 
 58088532 
  2  
  43  
 3 
 218.52  
   TCGA-19-5960-01A  
 IKZF4 
 chr12 
 56423358 
 OS9 
 chr12 
 58088532 
  1  
  41  
 1 
 -560.92  
 
    
479. chr10-chr10 rf 
 
   TCGA-06-0221-02A  
 SLIT1 
 chr10 
 98861107 
 SH3PXD2A 
 chr10 
 105489027 
  8  
  6  
 3 
 245.08  
 
    
480. chr1-chr1 rf 
 
   TCGA-19-2619-01A  
 AKT3 
 chr1 
 244006426 
 GCSAML-AS1 
 chr1 
 247690241 
  14  
  3  
 15 
 243.63  
 
    
481. chr1-chr1 fr 
 
   TCGA-28-2514-01A  
 ARID1A 
 chr1 
 27022590 
 AGBL4 
 chr1 
 49202123 
  13  
  7  
 10 
 -1433.70  
   TCGA-28-2514-01A  
 ARID1A 
 chr1 
 27024030 
 AGBL4 
 chr1 
 49202123 
  30  
  7  
 33 
 242.16  
 
    
482. chr6-chr6 rr 
 
   TCGA-14-2554-01A  
 SEC63 
 chr6 
 108279089 
 LACE1 
 chr6 
 108801426 
  7  
  4  
 8 
 240.65  
 
    
483. chr16-chr16 fr 
 
   TCGA-06-0210-01A  
 ZC3H18 
 chr16 
 88636973 
 CHMP1A 
 chr16 
 89713738 
  40  
  18  
 40 
 201.19  
   TCGA-06-0210-02A  
 ZC3H18 
 chr16 
 88636973 
 CHMP1A 
 chr16 
 89713738 
  52  
  15  
 52 
 238.73  
 
    
484. chr7-chr7 ff 
 
   TCGA-12-0616-01A  
 INHBA-AS1 
 chr7 
 41794705 
 SMURF1 
 chr7 
 98741348 
  90  
  11  
 71 
 238.39  
 
    
485. chr13-chr13 fr 
 
   TCGA-06-1804-01A  
 TDRD3 
 chr13 
 61109366 
 ESD 
 chr13 
 47345630 
  14  
  8  
 11 
 234.54  
 
    
486. chr12-chr12 rf 
 
   TCGA-19-2624-01A  
 AVIL 
 chr12 
 58200272 
 PPM1H 
 chr12 
 63171827 
  73  
  5  
 47 
 234.76  
 
    
487. chr17-chr17 rr 
 
   TCGA-14-1829-01A  
 SUMO2 
 chr17 
 73178462 
 UNK 
 chr17 
 73812841 
  1  
  38  
 1 
 226.39  
 
    
488. chr19-chr19 rr 
 
   TCGA-06-0211-01B  
 KLHL26 
 chr19 
 18775070 
 CRTC1 
 chr19 
 18794637 
  18  
  13  
 10 
 224.78  
   TCGA-06-0211-01A  
 KLHL26 
 chr19 
 18775070 
 CRTC1 
 chr19 
 18794637 
  7  
  9  
 8 
 161.79  
   TCGA-06-0211-02A  
 KLHL26 
 chr19 
 18775070 
 CRTC1 
 chr19 
 18794637 
  19  
  5  
 10 
 103.13  
 
    
489. chr6-chr6 fr 
 
   TCGA-06-5412-01A  
 RREB1 
 chr6 
 7108292 
 EEF1E1 
 chr6 
 8097699 
  7  
  4  
 7 
 223.48  
 
    
490. chr1-chr20 rr 
 
   TCGA-06-5859-01A  
 PDE4DIP 
 chr1 
 145042448 
 C20orf43 
 chr20 
 55059244 
  17  
  4  
 16 
 219.80  
 
    
491. chr6-chr6 fr 
 
   TCGA-28-5207-01A  
 ZNF292 
 chr6 
 87865476 
 ENSG00000231776 
 chr6 
 85154168 
  6  
  3  
 3 
 219.30  
 
    sample list 
 
  sample name 
 fragment length 
 read length 
 # of fragments  
 
    table description 
1. Sample name in which a fusion is identified  
2. Gene on the "left" side of the fusion  
3. Chromosome ID on the left  
4. Coordinates on the left  
5. Gene on the "right" side  
6. Chromosome ID on the right  
7. Coordinates on the right  
8. Number of spanning reads  
9. Number of spanning mate pairs  
10. Number of spanning mate pairs where one end spans a fusion  
If you follow the the 9th column, it shows coordinates "number1:number2" where one end is located at a distance of "number1" bases from the left genomic coordinate of a fusion and "number2" is similarly defined
     TCGA-02-0055-01A chr4-chr4 185765275 186231969 rr 
 
9 3 16 10 54 47
CAGCCATGATGCATACTTGTTACAGAAAGAAACGCCCACGTTGAGCTCTC CTTCTGGCTTTGAGGCCCTTCCCCCTCATCCAGGGCCCCATCCTCTTGCT
55677777777777777888899999999999999999999999999999 99999999999999999999997775443222222222222221111000
LOC731424 exon1(185764448-185767687) SNX25 exon7(186231787-186231969)
 
 blast search - human genome  
 left 
 >ref|NW_001838921.1| Homo sapiens chromosome 4 genomic contig, alternate assembly 
HuRef SCAF_1103279188415, whole genome shotgun sequence
 gb|DS486019.1| Homo sapiens SCAF_1103279188415 genomic scaffold, whole genome 
shotgun sequence
Length=50948600

 Score = 93.5 bits (50),  Expect = 5e-17
 Identities = 50/50 (100%), Gaps = 0/50 (0%)
 Strand=Plus/Minus

Query  1         CAGCCATGATGCATACTTGTTACAGAAAGAAACGCCCACGTTGAGCTCTC  50
                 ||||||||||||||||||||||||||||||||||||||||||||||||||
Sbjct  46001011  CAGCCATGATGCATACTTGTTACAGAAAGAAACGCCCACGTTGAGCTCTC  46000962


>gb|CH471056.2| Homo sapiens 211000035843078 genomic scaffold, whole genome shotgun 
sequence
Length=70496567

 Score = 93.5 bits (50),  Expect = 5e-17
 Identities = 50/50 (100%), Gaps = 0/50 (0%)
 Strand=Plus/Minus

Query  1         CAGCCATGATGCATACTTGTTACAGAAAGAAACGCCCACGTTGAGCTCTC  50
                 ||||||||||||||||||||||||||||||||||||||||||||||||||
Sbjct  65351331  CAGCCATGATGCATACTTGTTACAGAAAGAAACGCCCACGTTGAGCTCTC  65351282


>ref|NT_016354.19| Homo sapiens chromosome 4 genomic contig, GRCh37.p9 Primary Assembly
 gb|GL000046.1| Homo sapiens chromosome 4 genomic contig, GRCh37 reference primary 
assembly
Length=115591997

 Score = 93.5 bits (50),  Expect = 5e-17
 Identities = 50/50 (100%), Gaps = 0/50 (0%)
 Strand=Plus/Minus

Query  1          CAGCCATGATGCATACTTGTTACAGAAAGAAACGCCCACGTTGAGCTCTC  50
                  ||||||||||||||||||||||||||||||||||||||||||||||||||
Sbjct  110313046  CAGCCATGATGCATACTTGTTACAGAAAGAAACGCCCACGTTGAGCTCTC  110312997


>ref|NC_000004.11| Homo sapiens chromosome 4, GRCh37.p9 Primary Assembly
 gb|CM000666.1| Homo sapiens chromosome 4, GRCh37 primary reference assembly
Length=191154276

 Score = 93.5 bits (50),  Expect = 5e-17
 Identities = 50/50 (100%), Gaps = 0/50 (0%)
 Strand=Plus/Minus

Query  1          CAGCCATGATGCATACTTGTTACAGAAAGAAACGCCCACGTTGAGCTCTC  50
                  ||||||||||||||||||||||||||||||||||||||||||||||||||
Sbjct  185765325  CAGCCATGATGCATACTTGTTACAGAAAGAAACGCCCACGTTGAGCTCTC  185765276


>ref|AC_000136.1| Homo sapiens chromosome 4, alternate assembly HuRef, whole genome 
shotgun sequence
 gb|CM000465.1| Homo sapiens chromosome 4, whole genome shotgun sequence
Length=186704410

 Score = 93.5 bits (50),  Expect = 5e-17
 Identities = 50/50 (100%), Gaps = 0/50 (0%)
 Strand=Plus/Minus

Query  1          CAGCCATGATGCATACTTGTTACAGAAAGAAACGCCCACGTTGAGCTCTC  50
                  ||||||||||||||||||||||||||||||||||||||||||||||||||
Sbjct  181518772  CAGCCATGATGCATACTTGTTACAGAAAGAAACGCCCACGTTGAGCTCTC  181518723


>gb|CM000255.1| Homo sapiens chromosome 4, whole genome shotgun sequence
Length=189777617

 Score = 93.5 bits (50),  Expect = 5e-17
 Identities = 50/50 (100%), Gaps = 0/50 (0%)
 Strand=Plus/Minus

Query  1          CAGCCATGATGCATACTTGTTACAGAAAGAAACGCCCACGTTGAGCTCTC  50
                  ||||||||||||||||||||||||||||||||||||||||||||||||||
Sbjct  183093852  CAGCCATGATGCATACTTGTTACAGAAAGAAACGCCCACGTTGAGCTCTC  183093803


Lambda     K      H
    1.33    0.621     1.12 

Gapped
Lambda     K      H
    1.28    0.460    0.850 

Effective search space used: 681780354108
 
 right 
 >ref|NW_001838921.1| Homo sapiens chromosome 4 genomic contig, alternate assembly 
HuRef SCAF_1103279188415, whole genome shotgun sequence
 gb|DS486019.1| Homo sapiens SCAF_1103279188415 genomic scaffold, whole genome 
shotgun sequence
Length=50948600

 Score = 93.5 bits (50),  Expect = 5e-17
 Identities = 50/50 (100%), Gaps = 0/50 (0%)
 Strand=Plus/Minus

Query  1         CTTCTGGCTTTGAGGCCCTTCCCCCTCATCCAGGGCCCCATCCTCTTGCT  50
                 ||||||||||||||||||||||||||||||||||||||||||||||||||
Sbjct  46467705  CTTCTGGCTTTGAGGCCCTTCCCCCTCATCCAGGGCCCCATCCTCTTGCT  46467656


>gb|CH471056.2| Homo sapiens 211000035843078 genomic scaffold, whole genome shotgun 
sequence
Length=70496567

 Score = 93.5 bits (50),  Expect = 5e-17
 Identities = 50/50 (100%), Gaps = 0/50 (0%)
 Strand=Plus/Minus

Query  1         CTTCTGGCTTTGAGGCCCTTCCCCCTCATCCAGGGCCCCATCCTCTTGCT  50
                 ||||||||||||||||||||||||||||||||||||||||||||||||||
Sbjct  65815613  CTTCTGGCTTTGAGGCCCTTCCCCCTCATCCAGGGCCCCATCCTCTTGCT  65815564


>ref|NT_016354.19| Homo sapiens chromosome 4 genomic contig, GRCh37.p9 Primary Assembly
 gb|GL000046.1| Homo sapiens chromosome 4 genomic contig, GRCh37 reference primary 
assembly
Length=115591997

 Score = 93.5 bits (50),  Expect = 5e-17
 Identities = 50/50 (100%), Gaps = 0/50 (0%)
 Strand=Plus/Minus

Query  1          CTTCTGGCTTTGAGGCCCTTCCCCCTCATCCAGGGCCCCATCCTCTTGCT  50
                  ||||||||||||||||||||||||||||||||||||||||||||||||||
Sbjct  110779691  CTTCTGGCTTTGAGGCCCTTCCCCCTCATCCAGGGCCCCATCCTCTTGCT  110779642


>ref|NC_000004.11| Homo sapiens chromosome 4, GRCh37.p9 Primary Assembly
 gb|CM000666.1| Homo sapiens chromosome 4, GRCh37 primary reference assembly
Length=191154276

 Score = 93.5 bits (50),  Expect = 5e-17
 Identities = 50/50 (100%), Gaps = 0/50 (0%)
 Strand=Plus/Minus

Query  1          CTTCTGGCTTTGAGGCCCTTCCCCCTCATCCAGGGCCCCATCCTCTTGCT  50
                  ||||||||||||||||||||||||||||||||||||||||||||||||||
Sbjct  186231970  CTTCTGGCTTTGAGGCCCTTCCCCCTCATCCAGGGCCCCATCCTCTTGCT  186231921


>ref|AC_000136.1| Homo sapiens chromosome 4, alternate assembly HuRef, whole genome 
shotgun sequence
 gb|CM000465.1| Homo sapiens chromosome 4, whole genome shotgun sequence
Length=186704410

 Score = 93.5 bits (50),  Expect = 5e-17
 Identities = 50/50 (100%), Gaps = 0/50 (0%)
 Strand=Plus/Minus

Query  1          CTTCTGGCTTTGAGGCCCTTCCCCCTCATCCAGGGCCCCATCCTCTTGCT  50
                  ||||||||||||||||||||||||||||||||||||||||||||||||||
Sbjct  181985466  CTTCTGGCTTTGAGGCCCTTCCCCCTCATCCAGGGCCCCATCCTCTTGCT  181985417


>gb|CM000255.1| Homo sapiens chromosome 4, whole genome shotgun sequence
Length=189777617

 Score = 93.5 bits (50),  Expect = 5e-17
 Identities = 50/50 (100%), Gaps = 0/50 (0%)
 Strand=Plus/Minus

Query  1          CTTCTGGCTTTGAGGCCCTTCCCCCTCATCCAGGGCCCCATCCTCTTGCT  50
                  ||||||||||||||||||||||||||||||||||||||||||||||||||
Sbjct  183558134  CTTCTGGCTTTGAGGCCCTTCCCCCTCATCCAGGGCCCCATCCTCTTGCT  183558085


>gb|CM000491.1| Homo sapiens chromosome 1, whole genome shotgun sequence
Length=219474137

 Score = 82.4 bits (44),  Expect = 1e-13
 Identities = 48/50 (96%), Gaps = 0/50 (0%)
 Strand=Plus/Minus

Query  1          CTTCTGGCTTTGAGGCCCTTCCCCCTCATCCAGGGCCCCATCCTCTTGCT  50
                  |||||||||||||||||||||||||||||||| ||||||| |||||||||
Sbjct  182267373  CTTCTGGCTTTGAGGCCCTTCCCCCTCATCCACGGCCCCAACCTCTTGCT  182267324


>gb|GL583053.1| Homo sapiens unplaced genomic scaffold scaffold_73, whole genome 
shotgun sequence
Length=11389693

 Score = 82.4 bits (44),  Expect = 1e-13
 Identities = 48/50 (96%), Gaps = 0/50 (0%)
 Strand=Plus/Minus

Query  1       CTTCTGGCTTTGAGGCCCTTCCCCCTCATCCAGGGCCCCATCCTCTTGCT  50
               |||||||||||||||||||||||||||||||| ||||||| |||||||||
Sbjct  438796  CTTCTGGCTTTGAGGCCCTTCCCCCTCATCCACGGCCCCAACCTCTTGCT  438747


>gb|DS990706.1| Homo sapiens SCAF_1112675837268 genomic scaffold, whole genome 
shotgun sequence
Length=16132794

 Score = 82.4 bits (44),  Expect = 1e-13
 Identities = 48/50 (96%), Gaps = 0/50 (0%)
 Strand=Plus/Plus

Query  1         CTTCTGGCTTTGAGGCCCTTCCCCCTCATCCAGGGCCCCATCCTCTTGCT  50
                 |||||||||||||||||||||||||||||||| ||||||| |||||||||
Sbjct  11660316  CTTCTGGCTTTGAGGCCCTTCCCCCTCATCCACGGCCCCAACCTCTTGCT  11660365


>gb|CH003496.1| Homo sapiens chromosome 1, whole genome shotgun sequence
Length=223056964

 Score = 82.4 bits (44),  Expect = 1e-13
 Identities = 48/50 (96%), Gaps = 0/50 (0%)
 Strand=Plus/Minus

Query  1          CTTCTGGCTTTGAGGCCCTTCCCCCTCATCCAGGGCCCCATCCTCTTGCT  50
                  |||||||||||||||||||||||||||||||| ||||||| |||||||||
Sbjct  186252121  CTTCTGGCTTTGAGGCCCTTCCCCCTCATCCACGGCCCCAACCTCTTGCT  186252072


>gb|CH003448.1| Homo sapiens chromosome 1, whole genome shotgun sequence
Length=221336263

 Score = 82.4 bits (44),  Expect = 1e-13
 Identities = 48/50 (96%), Gaps = 0/50 (0%)
 Strand=Plus/Minus

Query  1          CTTCTGGCTTTGAGGCCCTTCCCCCTCATCCAGGGCCCCATCCTCTTGCT  50
                  |||||||||||||||||||||||||||||||| ||||||| |||||||||
Sbjct  184326798  CTTCTGGCTTTGAGGCCCTTCCCCCTCATCCACGGCCCCAACCTCTTGCT  184326749


>gb|CH471100.2| Homo sapiens 211000035839546 genomic scaffold, whole genome shotgun 
sequence
Length=17411188

 Score = 82.4 bits (44),  Expect = 1e-13
 Identities = 48/50 (96%), Gaps = 0/50 (0%)
 Strand=Plus/Minus

Query  1        CTTCTGGCTTTGAGGCCCTTCCCCCTCATCCAGGGCCCCATCCTCTTGCT  50
                |||||||||||||||||||||||||||||||| ||||||| |||||||||
Sbjct  4983624  CTTCTGGCTTTGAGGCCCTTCCCCCTCATCCACGGCCCCAACCTCTTGCT  4983575


>ref|NW_001838537.2| Homo sapiens chromosome 1 genomic contig, alternate assembly 
HuRef SCAF_1103279188323, whole genome shotgun sequence
 gb|DS486068.1| Homo sapiens SCAF_1103279188323 genomic scaffold, whole genome 
shotgun sequence
Length=16133001

 Score = 82.4 bits (44),  Expect = 1e-13
 Identities = 48/50 (96%), Gaps = 0/50 (0%)
 Strand=Plus/Plus

Query  1         CTTCTGGCTTTGAGGCCCTTCCCCCTCATCCAGGGCCCCATCCTCTTGCT  50
                 |||||||||||||||||||||||||||||||| ||||||| |||||||||
Sbjct  11660462  CTTCTGGCTTTGAGGCCCTTCCCCCTCATCCACGGCCCCAACCTCTTGCT  11660511


>ref|NT_167186.1| Homo sapiens chromosome 1 genomic contig, GRCh37.p9 Primary Assembly
 gb|GL000018.1| Homo sapiens chromosome 1 genomic contig, GRCh37 reference primary 
assembly
Length=42425989

 Score = 82.4 bits (44),  Expect = 1e-13
 Identities = 48/50 (96%), Gaps = 0/50 (0%)
 Strand=Plus/Minus

Query  1        CTTCTGGCTTTGAGGCCCTTCCCCCTCATCCAGGGCCCCATCCTCTTGCT  50
                |||||||||||||||||||||||||||||||| ||||||| |||||||||
Sbjct  5109055  CTTCTGGCTTTGAGGCCCTTCCCCCTCATCCACGGCCCCAACCTCTTGCT  5109006


>ref|NC_000001.10| Homo sapiens chromosome 1, GRCh37.p9 Primary Assembly
 gb|CM000663.1| Homo sapiens chromosome 1, GRCh37 primary reference assembly
Length=249250621

 Score = 82.4 bits (44),  Expect = 1e-13
 Identities = 48/50 (96%), Gaps = 0/50 (0%)
 Strand=Plus/Minus

Query  1          CTTCTGGCTTTGAGGCCCTTCCCCCTCATCCAGGGCCCCATCCTCTTGCT  50
                  |||||||||||||||||||||||||||||||| ||||||| |||||||||
Sbjct  211591276  CTTCTGGCTTTGAGGCCCTTCCCCCTCATCCACGGCCCCAACCTCTTGCT  211591227


>ref|AC_000133.1| Homo sapiens chromosome 1, alternate assembly HuRef, whole genome 
shotgun sequence
 gb|CM000462.1| Homo sapiens chromosome 1, whole genome shotgun sequence
Length=219475005

 Score = 82.4 bits (44),  Expect = 1e-13
 Identities = 48/50 (96%), Gaps = 0/50 (0%)
 Strand=Plus/Minus

Query  1          CTTCTGGCTTTGAGGCCCTTCCCCCTCATCCAGGGCCCCATCCTCTTGCT  50
                  |||||||||||||||||||||||||||||||| ||||||| |||||||||
Sbjct  182268222  CTTCTGGCTTTGAGGCCCTTCCCCCTCATCCACGGCCCCAACCTCTTGCT  182268173


>gb|CM000252.1| Homo sapiens chromosome 1, whole genome shotgun sequence
Length=222388987

 Score = 82.4 bits (44),  Expect = 1e-13
 Identities = 48/50 (96%), Gaps = 0/50 (0%)
 Strand=Plus/Minus

Query  1          CTTCTGGCTTTGAGGCCCTTCCCCCTCATCCAGGGCCCCATCCTCTTGCT  50
                  |||||||||||||||||||||||||||||||| ||||||| |||||||||
Sbjct  184817864  CTTCTGGCTTTGAGGCCCTTCCCCCTCATCCACGGCCCCAACCTCTTGCT  184817815


Lambda     K      H
    1.33    0.621     1.12 

Gapped
Lambda     K      H
    1.28    0.460    0.850 

Effective search space used: 681780354108
 
 blast search - nt  
 left 
 >ref|XR_151064.1| PREDICTED: Pongo abelii uncharacterized LOC100936760 (LOC100936760), 
misc_RNA
Length=3368

 Score = 93.5 bits (50),  Expect = 6e-17
 Identities = 50/50 (100%), Gaps = 0/50 (0%)
 Strand=Plus/Plus

Query  1     CAGCCATGATGCATACTTGTTACAGAAAGAAACGCCCACGTTGAGCTCTC  50
             ||||||||||||||||||||||||||||||||||||||||||||||||||
Sbjct  2491  CAGCCATGATGCATACTTGTTACAGAAAGAAACGCCCACGTTGAGCTCTC  2540


>ref|NR_037867.1| Homo sapiens uncharacterized LOC731424 (LOC731424), non-coding 
RNA
Length=3390

 Score = 93.5 bits (50),  Expect = 6e-17
 Identities = 50/50 (100%), Gaps = 0/50 (0%)
 Strand=Plus/Plus

Query  1     CAGCCATGATGCATACTTGTTACAGAAAGAAACGCCCACGTTGAGCTCTC  50
             ||||||||||||||||||||||||||||||||||||||||||||||||||
Sbjct  2515  CAGCCATGATGCATACTTGTTACAGAAAGAAACGCCCACGTTGAGCTCTC  2564


>gb|AC084871.4| Homo sapiens BAC clone RP11-701P16 from 4, complete sequence
Length=203726

 Score = 93.5 bits (50),  Expect = 6e-17
 Identities = 50/50 (100%), Gaps = 0/50 (0%)
 Strand=Plus/Minus

Query  1      CAGCCATGATGCATACTTGTTACAGAAAGAAACGCCCACGTTGAGCTCTC  50
              ||||||||||||||||||||||||||||||||||||||||||||||||||
Sbjct  86566  CAGCCATGATGCATACTTGTTACAGAAAGAAACGCCCACGTTGAGCTCTC  86517


Lambda     K      H
    1.33    0.621     1.12 

Gapped
Lambda     K      H
    1.28    0.460    0.850 

Effective search space used: 874911716973
 
 right 
 >ref|XM_003823472.1| PREDICTED: Pan paniscus sorting nexin 25 (SNX25), mRNA
Length=3323

 Score = 93.5 bits (50),  Expect = 6e-17
 Identities = 50/50 (100%), Gaps = 0/50 (0%)
 Strand=Plus/Minus

Query  1     CTTCTGGCTTTGAGGCCCTTCCCCCTCATCCAGGGCCCCATCCTCTTGCT  50
             ||||||||||||||||||||||||||||||||||||||||||||||||||
Sbjct  1146  CTTCTGGCTTTGAGGCCCTTCCCCCTCATCCAGGGCCCCATCCTCTTGCT  1097


>ref|XM_002815344.2| PREDICTED: Pongo abelii sorting nexin 25 (SNX25), mRNA
Length=2669

 Score = 93.5 bits (50),  Expect = 6e-17
 Identities = 50/50 (100%), Gaps = 0/50 (0%)
 Strand=Plus/Minus

Query  1     CTTCTGGCTTTGAGGCCCTTCCCCCTCATCCAGGGCCCCATCCTCTTGCT  50
             ||||||||||||||||||||||||||||||||||||||||||||||||||
Sbjct  1086  CTTCTGGCTTTGAGGCCCTTCCCCCTCATCCAGGGCCCCATCCTCTTGCT  1037


>ref|XM_517558.3| PREDICTED: Pan troglodytes sorting nexin 25, transcript variant 
2 (SNX25), mRNA
Length=3323

 Score = 93.5 bits (50),  Expect = 6e-17
 Identities = 50/50 (100%), Gaps = 0/50 (0%)
 Strand=Plus/Minus

Query  1     CTTCTGGCTTTGAGGCCCTTCCCCCTCATCCAGGGCCCCATCCTCTTGCT  50
             ||||||||||||||||||||||||||||||||||||||||||||||||||
Sbjct  1146  CTTCTGGCTTTGAGGCCCTTCCCCCTCATCCAGGGCCCCATCCTCTTGCT  1097


>ref|NM_031953.2| Homo sapiens sorting nexin 25 (SNX25), mRNA
Length=3237

 Score = 93.5 bits (50),  Expect = 6e-17
 Identities = 50/50 (100%), Gaps = 0/50 (0%)
 Strand=Plus/Minus

Query  1     CTTCTGGCTTTGAGGCCCTTCCCCCTCATCCAGGGCCCCATCCTCTTGCT  50
             ||||||||||||||||||||||||||||||||||||||||||||||||||
Sbjct  1041  CTTCTGGCTTTGAGGCCCTTCCCCCTCATCCAGGGCCCCATCCTCTTGCT  992


>gb|BC029868.1| Homo sapiens sorting nexin 25, mRNA (cDNA clone MGC:34107 IMAGE:5171892), 
complete cds
Length=1964

 Score = 93.5 bits (50),  Expect = 6e-17
 Identities = 50/50 (100%), Gaps = 0/50 (0%)
 Strand=Plus/Minus

Query  1    CTTCTGGCTTTGAGGCCCTTCCCCCTCATCCAGGGCCCCATCCTCTTGCT  50
            ||||||||||||||||||||||||||||||||||||||||||||||||||
Sbjct  386  CTTCTGGCTTTGAGGCCCTTCCCCCTCATCCAGGGCCCCATCCTCTTGCT  337


>gb|AY601647.1| Homo sapiens SNX25 (SNX25) mRNA, complete cds
Length=3316

 Score = 93.5 bits (50),  Expect = 6e-17
 Identities = 50/50 (100%), Gaps = 0/50 (0%)
 Strand=Plus/Minus

Query  1     CTTCTGGCTTTGAGGCCCTTCCCCCTCATCCAGGGCCCCATCCTCTTGCT  50
             ||||||||||||||||||||||||||||||||||||||||||||||||||
Sbjct  1146  CTTCTGGCTTTGAGGCCCTTCCCCCTCATCCAGGGCCCCATCCTCTTGCT  1097


>dbj|AK095661.1| Homo sapiens cDNA FLJ38342 fis, clone FCBBF3028188, highly similar 
to Homo sapiens sorting nexin 25 (SNX25), mRNA
Length=2720

 Score = 93.5 bits (50),  Expect = 6e-17
 Identities = 50/50 (100%), Gaps = 0/50 (0%)
 Strand=Plus/Minus

Query  1    CTTCTGGCTTTGAGGCCCTTCCCCCTCATCCAGGGCCCCATCCTCTTGCT  50
            ||||||||||||||||||||||||||||||||||||||||||||||||||
Sbjct  550  CTTCTGGCTTTGAGGCCCTTCCCCCTCATCCAGGGCCCCATCCTCTTGCT  501


>gb|AC073344.7| Homo sapiens BAC clone RP11-628B17 from 4, complete sequence
Length=192573

 Score = 93.5 bits (50),  Expect = 6e-17
 Identities = 50/50 (100%), Gaps = 0/50 (0%)
 Strand=Plus/Minus

Query  1       CTTCTGGCTTTGAGGCCCTTCCCCCTCATCCAGGGCCCCATCCTCTTGCT  50
               ||||||||||||||||||||||||||||||||||||||||||||||||||
Sbjct  121415  CTTCTGGCTTTGAGGCCCTTCCCCCTCATCCAGGGCCCCATCCTCTTGCT  121366


>gb|AC093592.3| Homo sapiens BAC clone RP11-50L21 from 4, complete sequence
Length=184533

 Score = 93.5 bits (50),  Expect = 6e-17
 Identities = 50/50 (100%), Gaps = 0/50 (0%)
 Strand=Plus/Minus

Query  1       CTTCTGGCTTTGAGGCCCTTCCCCCTCATCCAGGGCCCCATCCTCTTGCT  50
               ||||||||||||||||||||||||||||||||||||||||||||||||||
Sbjct  160573  CTTCTGGCTTTGAGGCCCTTCCCCCTCATCCAGGGCCCCATCCTCTTGCT  160524


>ref|XM_003483618.1| PREDICTED: Sus scrofa sorting nexin 25, transcript variant 2 
(SNX25), mRNA
Length=3189

 Score = 82.4 bits (44),  Expect = 1e-13
 Identities = 48/50 (96%), Gaps = 0/50 (0%)
 Strand=Plus/Minus

Query  1     CTTCTGGCTTTGAGGCCCTTCCCCCTCATCCAGGGCCCCATCCTCTTGCT  50
             |||||||||||||||||||||||||||||| | |||||||||||||||||
Sbjct  1029  CTTCTGGCTTTGAGGCCCTTCCCCCTCATCTAAGGCCCCATCCTCTTGCT  980


>ref|XM_001926109.4| PREDICTED: Sus scrofa sorting nexin 25, transcript variant 1 
(SNX25), mRNA
Length=2985

 Score = 82.4 bits (44),  Expect = 1e-13
 Identities = 48/50 (96%), Gaps = 0/50 (0%)
 Strand=Plus/Minus

Query  1    CTTCTGGCTTTGAGGCCCTTCCCCCTCATCCAGGGCCCCATCCTCTTGCT  50
            |||||||||||||||||||||||||||||| | |||||||||||||||||
Sbjct  825  CTTCTGGCTTTGAGGCCCTTCCCCCTCATCTAAGGCCCCATCCTCTTGCT  776


>ref|XM_003412443.1| PREDICTED: Loxodonta africana sorting nexin 25 (SNX25), mRNA
Length=2379

 Score = 82.4 bits (44),  Expect = 1e-13
 Identities = 48/50 (96%), Gaps = 0/50 (0%)
 Strand=Plus/Minus

Query  1    CTTCTGGCTTTGAGGCCCTTCCCCCTCATCCAGGGCCCCATCCTCTTGCT  50
            ||| |||||||||||||||||||||||||||| |||||||||||||||||
Sbjct  708  CTTTTGGCTTTGAGGCCCTTCCCCCTCATCCAAGGCCCCATCCTCTTGCT  659


>ref|NG_027204.1| Homo sapiens sorting nexin 25 pseudogene 1 (SNX25P1) on chromosome 
1
Length=1113

 Score = 82.4 bits (44),  Expect = 1e-13
 Identities = 48/50 (96%), Gaps = 0/50 (0%)
 Strand=Plus/Minus

Query  1     CTTCTGGCTTTGAGGCCCTTCCCCCTCATCCAGGGCCCCATCCTCTTGCT  50
             |||||||||||||||||||||||||||||||| ||||||| |||||||||
Sbjct  1010  CTTCTGGCTTTGAGGCCCTTCCCCCTCATCCACGGCCCCAACCTCTTGCT  961


>emb|AL590101.9| Human DNA sequence from clone RP11-318L16 on chromosome 1 Contains 
the 3' end of a novel gene (FLJ10876), the TRAF5 gene 
for TNF receptor-associated factor 5, a novel gene (MGC14801), 
a sorting nexin 25 (SNX25) pseudogene and a actin related 
protein 2/3 complex, subunit 3 21kDa (ARPC3) pseudogene, complete 
sequence
Length=180169

 Score = 82.4 bits (44),  Expect = 1e-13
 Identities = 48/50 (96%), Gaps = 0/50 (0%)
 Strand=Plus/Minus

Query  1       CTTCTGGCTTTGAGGCCCTTCCCCCTCATCCAGGGCCCCATCCTCTTGCT  50
               |||||||||||||||||||||||||||||||| ||||||| |||||||||
Sbjct  139201  CTTCTGGCTTTGAGGCCCTTCCCCCTCATCCACGGCCCCAACCTCTTGCT  139152


Lambda     K      H
    1.33    0.621     1.12 

Gapped
Lambda     K      H
    1.28    0.460    0.850 

Effective search space used: 874911716973
 
    reads 
 chr4 chr4 185771557 185765304 24m6177n52m                    |||||||||||||||||||||||||||||||||||||||||||||||||||||||||||||||||||||||||||||||||||||||||||||||||||||||||||||||||||||||||||||||||||||||||||||||||||||||||||||||||||||||||||||||||||||||||||||||||||||||||||||||||||||||||||CTTTAAACTGCTTGTTATGGGGAGGAGTCAGCCAGCCATGATGCATACTTGT
chr4 chr4 185771552 185765299 19m6177n57m                    |||||||||||||||||||||||||||||||||||||||||||||||||||||||||||||||||||||||||||||||||||||||||||||||||||||||||||||||||||||||||||||||||||||||||||||||||||||||||||||||||||||||||||||||||||||||||||||||||||||||||||||||||||||||||||CTTTAAACTGCTTGTTATGGGGAGGAGTCAGCCAGCCATGATGCATACTTGTTACAG
chr4 chr4 185765647 185765571 76m                            AGGA
chr4 chr4 185765643 185765567 76m                            AGGAATCT
chr4 chr4 185765639 185765563 76m                            AGGAATCTTCTG
chr4 chr4 185765628 185765552 76m                            AGGAATCTTCTGGTGGAAAGTTA
chr4 chr4 185765615 185765539 76m                            AGGAATCTTCTGGTGGAAAGTTAGGCAGGGAAACGG
chr4 chr4 185765608 185765532 76m                            AGGAATCTTCTGGTGGAAAGTTAGGCAGGGAAACGGACACTGC
chr4 chr4 185765599 185765523 76m                            AGGAATCTTCTGGTGGAAAGTTAGGCAGGGAAACGGACACTGCTATGAGCAT
chr4 chr4 185765595 185765519 76m                            AGGAATCTTCTGGTGGAAAGTTAGGCAGGGAAACGGACACTGCTATGAGCATGTCT
chr4 chr4 185765591 185765515 76m                            AGGAATCTTCTGGTGGAAAGTTAGGCAGGGAAACGGACACTGCTATGAGCATGTCTTCCT
chr4 chr4 185765584 185765508 76m                            AGGAATCTTCTGGTGGAAAGTTAGGCAGGGAAACGGACACTGCTATGAGCATGTCTTCCTGGGCAGC
chr4 chr4 185765578 185765502 76m                            AGGAATCTTCTGGTGGAAAGTTAGGCAGGGAAACGGACACTGCTATGAGCATGTCTTCCTGGGCAGCTTGCGA
chr4 chr4 185765571 185765495 76m                                ATCTTCTGGTGGAAAGTTAGGCAGGGAAACGGACACTGCTATGAGCATGTCTTCCTGGGCAGCTTGCGATGGTCAG
chr4 chr4 185765568 185765492 76m                                   TTCTGGTGGAAAGTTAGGCATGGAAACGGACACTGCTATGAGCATGTCTTCCTGGGCAGCTTGCGATGGTCCGGAG
chr4 chr4 185765564 185765488 76m                                       GGTGGAAAGTTAGGCAGGGAAACGGACACTGCTATGAGCATGTCTTCCTGGGCAGCTTGCGATGGTCAGGAGGAGG
chr4 chr4 185765561 185765485 76m                                          GGAAAGTTAGGCAGGGAAACGGACACTGCTATGAGCATGTCTTCCTGGGCAGCTTGCGATGGTCAGGAGGAGGTGG
chr4 chr4 185765554 185765478 76m                                                 TAGGCAGGGAACCGGACACTGCTATGAGCATGTCTTCCTGGGCAGCTTGCGATGGTCAGGAGGAGGTGGTTGTTCC
chr4 chr4 185765551 185765475 76m                                                    GCAGGGAAACGGACACTGCTATGAGCATGTCTTCCTGGGCAGCTTGCGATGGTCAGGAGGAGGTGGTTGTTCCTCA
chr4 chr4 185765548 185765472 76m                                                       GGGAAACGGACACTGCTATGAGCATGTCTTCCTGGGCAGCTTGCGATGGTCAGGAGGAGGTGGTTGTTCCTCAGCA
chr4 chr4 185765542 185765466 76m                                                             CGGACACTGCTATGAGCATGTCTTCCTGGGCAGCTTGCGATGGTCAGGAGGAGGTGGTTGTTCCTCAGCAGCGGTG
chr4 chr4 185765537 185765461 76m                                                                  ACTGCTATGAGCATGTCTTCCTGGGCAGCTTGCGATGGTCAGGAGGAGGTGGTTGTTCATCAGCAGCGGTGCTTCC
chr4 chr4 185765528 185765452 76m                                                                           AGCATGTCTTCCTGGGCAGCTTGCGATGGTCAGGAGGAGGTGGTTGTTCCTCAGCAGCGGTGCTTCCGATGAAGCC
chr4 chr4 185765525 185765449 76m                                                                              ATGTCTTCCTGGGCAGCTTGCGATGGTCAGGAGGAGGTGGTTGTTCCTCAGCAGCGGTGCTTCCGATGANGCCCCA
chr4 chr4 185765518 185765442 76m                                                                                     CCTGGGCAGCTTGCGATGGTCAGGAGGAGGTGGTTGTTCCTCAGCAGCGGTGCTTCCGATGAAGCCCCATGTTGTA
chr4 chr4 185765509 185765433 76m                                                                                              CTTGCGATGGTCAGGAGGAGGTGGTTGTTCCTCAGCAGCGGTGCTTCCGATGAAGCCCCATGTTGTAGTGGAAGAC
chr4 chr4 185765506 185765430 76m                                                                                                 GCGATGGTCAGGAGGAGGTGGTTGTTCCTCAGCAGCGGTGCTTCCGATGAAGCCCCATGTTGTAGTGGAAGACAAG
chr4 chr4 185765498 185765422 76m                                                                                                         CAGGAGGAGGTGGTTGTTCCTCAGCAGCGGTGCTTCCGATGAAGCCCCATGTTGTAGTGGAAGACAAGCCATTCCT
chr4 chr4 185765487 185765411 76m                                                                                                                    GGTTGTTCCTCAGCAGCGGTGCTTCCGATGAAGCCCCATGTTGTAGTGGAAGACAAGCCATTCCTGTCTGACCTTC
chr4 chr4 185765483 185765407 76m                                                                                                                        GTTCCTCAGCAGCGGTGCTTCCGATGAAGCCCCATGTTGTAGTGGAAGACAAGCCATTCCTGTCTGACCTTCTGTG
chr4 chr4 185765479 185765403 76m                                                                                                                            CTCAGCAGCGGTGCTTCCGATGAAGCCCCATGTTGTAGTGGAAGACAAGCCATTCCTGTCTGACCTTCTGTGCCGC
chr4 chr4 185765474 185765398 76m                                                                                                                                 CAGCGGTGCTTCCGATGAAGCCCCATGTTGTAGTGGAAGACAAGCCATTCCTGTCTGACCTTCTGTGCCGCGCCTA
chr4 chr4 185765470 185765394 76m                                                                                                                                     GGTGCTTCCGATGAAGCCCCATGTTGTAGTGGAAGACAAGCCATTCCTGTCTGACCTTCTGTGCCGCGCCTAGGCT
chr4 chr4 185765463 185765387 76m                                                                                                                                            CCGATGAAGCCCCATGTTGTAGTGAAAGAAAAGCCATTCCTGTCTGACCTTCTGTGCCGCGCCTAGGCTTCCAAAA
chr4 chr4 185765458 185765382 76m                                                                                                                                                 GAAGCCCCATGTTGTAGTAGAAGACAAGCCATTCCTGTCTGACCTTCTGTGCCGCGCCTAGGCTTCCAAAAGCTGC
chr4 chr4 185765455 185765379 76m                                                                                                                                                    TCCCCATGTTGTAGTGGAAGACAAGCCATTCCTGTCTGACCTTCTGTGCCGCGCCTAGGCTTCCAAAAGCTGCTAT
chr4 chr4 185765442 185765366 76m                                                                                                                                                                 GTGGAAGACAAGCCATTCCTGTCTGACCTTCTGTGCCGCGCCTAGGCTTCCAAAAGCTGCTATCGCCAAGATCCAT
chr4 chr4 185765425 185765349 76m                                                                                                                                                                                  CCTGTCTGACCTTCTGTGCCGCGCCTAGGCTTCCAAAAGCTGCTATCGCCAAGATCCATTTCCGTTCACCTTTAAA
chr4 chr4 185765420 185765344 76m                                                                                                                                                                                       CTGACCTTCTGCGCCGCGCCTAGGCTTCCAAAAGCTGCTATCGCCAAGATCCATTTCCGTTCACCTTTAAACTGCT
chr4 chr4 185765417 185765341 76m                                                                                                                                                                                          ACCTTCTGTGCCGCGCCTAGGCTTCCAAAAGCTGCTATCGCCAAGATCCATTTCCGTTCACCTTTAAACTGCTTGT
chr4 chr4 185765412 185765336 76m                                                                                                                                                                                               CTGTGCCGCGCCTAGGCTTCCAAAAGCTGCTATCGCCAAGATCCATTTCCGTTCACCTTTAAACTGCTTGTTATGG
chr4 chr4 185765408 185765332 76m                                                                                                                                                                                                   GCCGCGCCTAGGCTTCCAAAAGCTGCTATCGCCAAGATCCATTTCCGTTCACCTTTAAACTGCTTGTTATGGGGAG
chr4 chr4 185765400 185765324 76m                                                                                                                                                                                                           TAGGCTTCCAAAAGCTGCTATCGCCAAGATCCATTTCCGTTCACCTTTAAACTGCTTGTTATGGGGAGGAGTCAGC
chr4 chr4 185765397 185765321 76m                                                                                                                                                                                                              GCTTCCAAAAGCTGCTATCGCCAAGATCCATTTCCGTTCACCTTTAAACTGCTTGTTATGGGGAGGAGTCAGCCAG
chr4 chr4 185765379 185765303 76m                                                                                                                                                                                                                                CGCCAAGATCCATTTCCGTTCACCTTTAAACTGCTTGTTATGGGGAGGAGTCAGCCAGCCATGATGCATACTTGTT
chr4 chr4 185765374 185765298 76m                                                                                                                                                                                                                                     AGATCCATTTCCGTTCTCCTTTAAACTGCTTGTTATGGGGAGGAGTCAGCCAGCCATGATGCATACTTGTTACAGA
chr4 chr4 185765368 185765292 76m                                                                                                                                                                                                                                           ATTTCCGTTCACCTTTAAACTGCTTGTTATGGGGAGGAGTCAGCCAGCCATGATGCATACTTGTTACAGAAAGAAA
chr4 chr4 185765364 185765288 76m                                                                                                                                                                                                                                               CCGTTCACCTTTAAACTGCTTGTTATGGGGAGGAGTCAGCCAGCCATGATGCATACTTGTTACAGAAAGAAACGCC
chr4 chr4 185765357 185765281 76m                                                                                                                                                                                                                                                      CTTTTAAACTGCTTGTTATGGGGAGGAGTCAGCCAGCTATGATGCATACTTGTTACAGAAAGAAACGCCCACGTTG
chr4 chr4 185765348 185765272 76m                                                                                                                                                                                                                                                               CGCTTGTTATGGGGAGGAGTCAGCCAGCCATGATGCATACTTGTTACAGAAAGAAACGCCCACGTTGAGCTCTCCT
chr4 chr4 185765344 186231963 70m186231970F6m                                                                                                                                                                                                                                                       TGTTATGGGGAGGAGTCAGCCAGCCATGATGCATACTTGTTACAGAAAGAAACGCCCACGTTGAGCTCTC CTTCTG
chr4 chr4 185765344 186231963 70m186231970F6m                                                                                                                                                                                                                                                       TGTTATGGGGAGGAGTCAGCCAGCCATGATGCATACTTGTTACAGAAAGAAACGCCCACGTTGAGCTCTC CTTCTG
chr4 chr4 185765344 186231963 70m186231970F6m                                                                                                                                                                                                                                                       TGTTAGGGGGAGGAGTCAGCCAGCCATGATGCATACTTGTTACAGAAAGAAACGCCCACGTTGAGCTCTC CTTCTG
chr4 chr4 185765340 186231959 66m186231970F10m                                                                                                                                                                                                                                                          ATGGGGAGGAGCCAGCCAGCCATGATGCATACTTGTTACAGAAAGAAACGCCCACGTTGAGCTCTC CTTCTGGCTT
chr4 chr4 185765339 186231958 65m186231970F11m                                                                                                                                                                                                                                                           TGGGGAGGAGTCAGCCAGCCATGATGCATACTTGTTACAGAAAGAAACGCCCACGTTGAGCTCTC CTTCTGGCTTT
chr4 chr4 185765337 186231956 63m186231970F13m                                                                                                                                                                                                                                                             GGGAGGAGTCAGCCAGCCATGATGCATACTTGTTACAGAAAGAAACGCCCACGTTGAGCTCTC CTTCTGGCTTTGA
chr4 chr4 185765336 186231955 62m186231970F14m                                                                                                                                                                                                                                                              GGAGGAGTCAGCCAGCCATGATGCATACTTGTTACAGAAAGAAACGCCCACGTTGAGCTCTC CTTCTGGCTTTGAG
chr4 chr4 185765336 186231955 62m186231970F14m                                                                                                                                                                                                                                                              GGAGGAGTCAGCCAGCCATGATGCATACTTGTTACAGAAAGAAACGCCCACGTTGAGCTCTC CTTCTGGCTTTGAG
chr4 chr4 185765328 186231947 54m186231970F22m                                                                                                                                                                                                                                                                      CAGCCAGCCATGATGCATACTTGTTACAGAAAGAAACGCCCACGTTGAGCTCTC CTTCTGGCTTTGAGGCCCTTCC
chr4 chr4 185765328 186231947 54m186231970F22m                                                                                                                                                                                                                                                                      CACCCAGCCATGATGCATACTTGTTACAGAAAGAAACGCCCACGTTGAGCTCTC CTTCTGGCTTTGAGGCCCTTCC
chr4 chr4 185765325 186231944 51m186231970F25m                                                                                                                                                                                                                                                                         CCAGCCATGATGCATACTTGTTACAGAAAGAAACGCCCACGTTGAGCTCTC CTTCTGGCTTTGAGGCCCTTCCCCC
chr4 chr4 185765325 186231944 51m186231970F25m                                                                                                                                                                                                                                                                         CCAGCCATGATGCATACTTGTTACAGAAAGAAACGCCCACGTTGAGCTCTC CTTCTGGCTTTGAGGCCCTTCCCCC
chr4 chr4 185765324 186231943 50m186231970F26m                                                                                                                                                                                                                                                                          CGGCCATGATGCATACTTGTTACAGAAAGAAACGCCCACGTTGAGCTCTC CTTCTGGCTTTGAGGCCCTTCCCCCT
chr4 chr4 185765322 186231941 48m186231970F28m                                                                                                                                                                                                                                                                            GCCATGATGCATACTTGTTACAGAAAGAAACGCCCACGTTGAGCTCTC CTTCTGGCTTTGAGGCCCTTCCCCCTCA
chr4 chr4 185765321 186231940 47m186231970F29m                                                                                                                                                                                                                                                                             CCATGATGCATACTTGTTACAGAAAGAAACGCCCACGTTGAGCTCTC CTTCTGGCTTTGAGGCCCTTCCCCCTCAT
chr4 chr4 185765307 186231926 33m186231970F43m                                                                                                                                                                                                                                                                                           TGTTACAGAAAGAAACGCCCACGTTGAGCTCTC CTTCTGGCTTTGAGGCCCTTCCCCCTCATCCAGGGCCCCATCC
chr4 chr4 185765303 186231922 29m186231970F47m                                                                                                                                                                                                                                                                                               ACAGAAAGAAACGCCCACGTTGAGCTCTC CTTCTGGCTTTGAGGCCCTTCCCCCTCATCCAGGGCCCCATCCTCTT
chr4 chr4 185765291 186231910 17m186231970F59m                                                                                                                                                                                                                                                                                                           GCCCACGTTGAGCTCTC CTTCTGGCTTTGAGGCCCTTCCCCCTCATCCAGGGCCCCATCCTCTTGCTGGTCATAGG
chr4 chr4 185765290 186231909 16m186231970F60m                                                                                                                                                                                                                                                                                                            CCCACGTTGAGCTCTC CTTCTGGCTTTGAGGCCCTTCCCCCTCATCCAGGGCCCCATCCTCTTGCTGGTCATAGGC
chr4 chr4 185765289 186231908 15m186231970F61m                                                                                                                                                                                                                                                                                                             CCACGTTGAGCTCTC CTTCTGGCTTTGAGGCCCTTCCCCCTCATCCAGGGCCCCATCCTCTTGCTGGTCATAGGCA
chr4 chr4 185765289 186231908 15m186231970F61m                                                                                                                                                                                                                                                                                                             CCACGTTGAGCTCTC CTTCTGGCTTTGAGGCCCTTCCCCCTCATCCAGGGCCCCATCCTCTTGCTGGTCATAGGCA
chr4 chr4 185765281 186231900 7m186231970F69m                                                                                                                                                                                                                                                                                                                      AGCTCTC CTTCTGGCTTTGAGGCCCTTCCCCCTCATCCAGGGCCCCATCCTCTTGCTGGTCATAGGCAGGGCCTCC
chr4 chr4 186231969 186231893 76m                                                                                                                                                                                                                                                                                                                                          CTTCTGGCTTTGAGGCCCTTCCCCCTCATCCAGGGCCCCATCCTCTTGCTGGTCATAGGCAGGGCCTCCCAGGATT
chr4 chr4 186231966 186231890 76m                                                                                                                                                                                                                                                                                                                                             CTGGCTTTGAGGCCCTTCCCCCTCATCCAGGTCCCCATCCTCTTGCTGGTCATAGGCAGGGCCTCCCAGGATTCGG
chr4 chr4 186231963 186231887 76m                                                                                                                                                                                                                                                                                                                                                GCTTTGAGGCCCTTCCCCCTCATCCAGGGCCCCATCCTCTTGCTGGTCATAGGCAGGGCCTCCCAGGATTCGGATT
chr4 chr4 186231954 186231878 76m                                                                                                                                                                                                                                                                                                                                                         CCCTTCCCCCTCATCCAGGGCCCCATCCTCTTGCTGGTCATAGGCAGGGCCTCCCAGGATTCGGATTCTCTTCTCA
chr4 chr4 186231951 186231875 76m                                                                                                                                                                                                                                                                                                                                                            TTCCCCCTCATCCAGGGCCCCATCCTCTTGCTGGTCATAGGCAGGGCCTCCCAGGATTCGGATTCTCTTCTCACAC
chr4 chr4 186231947 186231871 76m                                                                                                                                                                                                                                                                                                                                                                CCCTCATCCAGGGCCCCATCCTCTTGCTGGTCATAGGCAGGGCCTCCCAGGATTCGGATTCTCTTCTCACACTGCT
chr4 chr4 186231942 186231866 76m                                                                                                                                                                                                                                                                                                                                                                     ATCCAGGGCCCCATCCTCTTGCTGGTCATAGGCAGGGCCTCCCAGGATTCGGATTCTCTTCTCACACTGCTTCTTT
chr4 chr4 186231938 186231862 76m                                                                                                                                                                                                                                                                                                                                                                         AGGGCCCCATCCTCTTGCTGGTCATAGGCAGGGCCTCCCAGGATTCGGATTCTCTTCTCACACTGCTTCTTTGCCA
chr4 chr4 186231934 186231858 76m                                                                                                                                                                                                                                                                                                                                                                             CCCCATCCTCTTGCTGGTCATAGGCAGGGCCTCCCAGGATTCGGATTCTCTTCTCACACTGCTTCTTTGCCACAGT
chr4 chr4 186231931 186231855 76m                                                                                                                                                                                                                                                                                                                                                                                CCTCCTCTTGCTGGTCATAGGCAGGGCCTCCCAGGATTCGGATTCTCTTCTCACACTGCTTCTTTGCCACAGTCAG
chr4 chr4 186231928 186231852 76m                                                                                                                                                                                                                                                                                                                                                                                   CCTCTTGCTGGTCATAGGCAGGGCCTCCCAGGATTCGGATTCTCTTCTCACACTGCTTCTTTGCCACAGTCAGTTG
chr4 chr4 186231925 186231849 76m                                                                                                                                                                                                                                                                                                                                                                                      CTTGCTGGTCATAGGCAGGGCCTCCCAGGATTCGGATTCTCTTCTCACACTGCTTCTTTGCCACAGTCAGTTGGTT
chr4 chr4 186231921 186231845 76m                                                                                                                                                                                                                                                                                                                                                                                          CTGGTCATAGGCAGGGCCTCCCAGGATTCGGATTCTCTTCTCACACTGCTTCTTTGCCACAGTCAGTTGGTTGATG
chr4 chr4 186231918 186231842 76m                                                                                                                                                                                                                                                                                                                                                                                             GTCATAGGCAGGGCCTCCCAGGATTCGGATTCTCTTCTCACACTGCTTCTTTGCCACAGTCAGTTGGTTGATGTAC
chr4 chr4 186231912 186231836 76m                                                                                                                                                                                                                                                                                                                                                                                                   GGCAGGGCCTCCCAGGATTCGGATTCTCTTCTCACACTGCTTCTTTGCCACAGTCAGTTGGTTGATGTACCTCTTC
chr4 chr4 186231909 186231833 76m                                                                                                                                                                                                                                                                                                                                                                                                      AGGGCCTCCCAGGATTCGGATTCTCTTCTCACACTGCTTCTTTGCCACAGTCAGTTGGTTGATGTACCTCTTCATG
chr4 chr4 186231903 186231827 76m                                                                                                                                                                                                                                                                                                                                                                                                            TCCCAGGATTCGGATTCTCTTCTCACACTGCTTCTTTGCCACAGTCAGTTGGTTGATGTACCTCTTCATGTTCCTG
chr4 chr4 186231900 186231824 76m                                                                                                                                                                                                                                                                                                                                                                                                               CAGGATTCGGATTCTCTTCTCACACTGCTTCTTTGCCACAGTCAGTTGGTTGATGTACCTCTTCATGTTCCTGGCC
chr4 chr4 186231895 186231819 76m                                                                                                                                                                                                                                                                                                                                                                                                                    TTCGGATTCTCTTCTCACACTGCTTCTTTGCCACAGTCAGTTGGTTGATGTACCTCTTCATGTTCCTGGCCCTCAG
chr4 chr4 186231892 186231816 76m                                                                                                                                                                                                                                                                                                                                                                                                                       GGATTCTCTTCTCACACTGCTTCTTTGCCACAGTCAGTTGGTTGATGTACCTCTTCATGTTCCTGGCCCTCAGGAG
chr4 chr4 186231889 186231813 76m                                                                                                                                                                                                                                                                                                                                                                                                                          TTCTCTTCTCACACTGCTTCTTTGCCACAGTCAGTTGGTTGATGTACCTCTTCATGTTCCTGGCCCTCAGGAGATC
chr4 chr4 186231885 186231809 76m                                                                                                                                                                                                                                                                                                                                                                                                                              CTTCTCACACTGCTTCTTTGCCACAGTCAGTTGGTTGATGTACCTCTTCATGTTCCTGGCCCTCAGGAGATCAGCT
chr4 chr4 186231882 186231806 76m                                                                                                                                                                                                                                                                                                                                                                                                                                 CTCACACTGCTTCTTTGCCACAGTCAGTTGGTTGATGTACCTCTTCATGTTCCTGGCCCTCAGGAGATCAGCTTTC
chr4 chr4 186231876 186231800 76m                                                                                                                                                                                                                                                                                                                                                                                                                                       CTGCTTCTTTGCCACAGTCAGTTGGTTGATGTACCTCTTCATGTTCCTGGCCCTCAGGAGATCAGCTTTNATTGCC
chr4 chr4 186231873 186231797 76m                                                                                                                                                                                                                                                                                                                                                                                                                                          CTTCTTTGCCACAGTCAGTTGGTTGATGTACCTCTTCATGTTCCTGGCCCTCAGGAGATCAGCTTTCATTACCGCA
chr4 chr4 186231870 186231794 76m                                                                                                                                                                                                                                                                                                                                                                                                                                             CTTTGCCACAGTCAGTTGGTTGATGTACCTCTTCATGTTCCTGGCCCTCAGGAGATCAGCTTTCATTGCCGCAGTT
chr4 chr4 186231866 186231790 76m                                                                                                                                                                                                                                                                                                                                                                                                                                                 GCCACAGTCAGTTGGTTGATGTACCTCTTCATGTTCCTGGCCCTCAGGAGATCAGCTTTCATTGCCGCAGTTTCTT
chr4 chr4 186231860 186209232 73m22552n3m                                                                                                                                                                                                                                                                                                                                                                                                                                               GTCAGTTGGTTGATGTACCTCTTCATGTTCCTGGCCCTCAGGAGATCAGCTTTCATTGCCGCAGTTTCTTTAC||||||||||||||||||||||||||||||||||||||||||||||||||||||||||||||||||||||||||||||||||||||||||||||||||||||||||||||||||||
chr4 chr4 186231856 186209228 69m22552n7m                                                                                                                                                                                                                                                                                                                                                                                                                                                   GTTGGTTGATGTACCTCTTCATGTTCCTGGCCCTCAGGAGATCAGCTTTCATTGCCGCAGTTTCTTTAC||||||||||||||||||||||||||||||||||||||||||||||||||||||||||||||||||||||||||||||||||||||||||||||||||||||||||||||||||||
chr4 chr4 186231853 186209225 66m22552n10m                                                                                                                                                                                                                                                                                                                                                                                                                                                     GGTTGATGTACCTCTTCATGTTCCTGGCCCTCAGGAGATCAGCTTTCATTGCCGCAGTTTCTTTAC||||||||||||||||||||||||||||||||||||||||||||||||||||||||||||||||||||||||||||||||||||||||||||||||||||||||||||||||||||
chr4 chr4 186231848 186209220 61m22552n15m                                                                                                                                                                                                                                                                                                                                                                                                                                                          ATGTACCTCTTCATGTTCCTGGCCCTCAGGAGATCAGCTTTCATTGCCGCAGTTTCTTTAC||||||||||||||||||||||||||||||||||||||||||||||||||||||||||||||||||||||||||||||||||||||||||||||||||||||||||||||||||||
chr4 chr4 186231843 186209215 56m22552n20m                                                                                                                                                                                                                                                                                                                                                                                                                                                               CCTCTTCATGTTCCTGGCCCTCAGGAGATCAGCTTTCATTGCCGCAGTTTCTTTAC||||||||||||||||||||||||||||||||||||||||||||||||||||||||||||||||||||||||||||||||||||||||||||||||||||||||||||||||||||
chr4 chr4 186231840 186209212 53m22552n23m                                                                                                                                                                                                                                                                                                                                                                                                                                                                  CTTCATGTTCCTGGCCCTCAGGAGATCAGCTTTCATTGCCGCAGTTTCTTTAC||||||||||||||||||||||||||||||||||||||||||||||||||||||||||||||||||||||||||||||||||||||||||||||||||||||||||||||||||||
chr4 chr4 186231836 186209208 49m22552n27m                                                                                                                                                                                                                                                                                                                                                                                                                                                                      ATGTTCCTGGCCCTCAGGAGATCAGCTTTCATTGCCGCAGTTTCTTTAC||||||||||||||||||||||||||||||||||||||||||||||||||||||||||||||||||||||||||||||||||||||||||||||||||||||||||||||||||||
chr4 chr4 186231833 186209205 46m22552n30m                                                                                                                                                                                                                                                                                                                                                                                                                                                                         GTCCTGGCCCTCAGGAGATCAGCTTTCATTGCCGCAGTTTCTTTAC||||||||||||||||||||||||||||||||||||||||||||||||||||||||||||||||||||||||||||||||||||||||||||||||||||||||||||||||||||
chr4 chr4 186231830 186209202 43m22552n33m                                                                                                                                                                                                                                                                                                                                                                                                                                                                            CTGGCCCTCAGGAGATCAGCTTTCATTGCCGCAGTTTCTTTAC||||||||||||||||||||||||||||||||||||||||||||||||||||||||||||||||||||||||||||||||||||||||||||||||||||||||||||||||||||
chr4 chr4 186231827 186209199 40m22552n36m                                                                                                                                                                                                                                                                                                                                                                                                                                                                               GCCCTCAGGAGATCAGCTTTCATTGCCGCAGTTTCTTTAC||||||||||||||||||||||||||||||||||||||||||||||||||||||||||||||||||||||||||||||||||||||||||||||||||||||||||||||||||||
chr4 chr4 186231822 186209194 35m22552n41m                                                                                                                                                                                                                                                                                                                                                                                                                                                                                    CAGGAGATCAGCTTTCATTGCCGCCGTTTCTTTAC||||||||||||||||||||||||||||||||||||||||||||||||||||||||||||||||||||||||||||||||||||||||||||||||||||||||||||||||||||
chr4 chr4 186231819 186209191 32m22552n44m                                                                                                                                                                                                                                                                                                                                                                                                                                                                                       GAGATCAGCTTTCATTGCCGCAGTTTCTTTAC||||||||||||||||||||||||||||||||||||||||||||||||||||||||||||||||||||||||||||||||||||||||||||||||||||||||||||||||||||
chr4 chr4 186231815 186209187 28m22552n48m                                                                                                                                                                                                                                                                                                                                                                                                                                                                                           TCAGCTTTCATTGCCGCAGTTTCTTTAC||||||||||||||||||||||||||||||||||||||||||||||||||||||||||||||||||||||||||||||||||||||||||||||||||||||||||||||||||||
chr4 chr4 186231812 186209184 25m22552n51m                                                                                                                                                                                                                                                                                                                                                                                                                                                                                              GCTTTCATTGCCGCAGTTTCTTTAC||||||||||||||||||||||||||||||||||||||||||||||||||||||||||||||||||||||||||||||||||||||||||||||||||||||||||||||||||||
chr4 chr4 186231809 186209181 22m22552n54m                                                                                                                                                                                                                                                                                                                                                                                                                                                                                                 TTCATTGCCGCAGTTTCTTTAC||||||||||||||||||||||||||||||||||||||||||||||||||||||||||||||||||||||||||||||||||||||||||||||||||||||||||||||||||||
chr4 chr4 186231801 186209173 14m22552n62m                                                                                                                                                                                                                                                                                                                                                                                                                                                                                                         CGCAGTTTCTTTAC||||||||||||||||||||||||||||||||||||||||||||||||||||||||||||||||||||||||||||||||||||||||||||||||||||||||||||||||||||
chr4 chr4 186231792 186209164 5m22552n71m                                                                                                                                                                                                                                                                                                                                                                                                                                                                                                                   TTTAC||||||||||||||||||||||||||||||||||||||||||||||||||||||||||||||||||||||||||||||||||||||||||||||||||||||||||||||||||||
chr4 chr4 186231789 186209161 2m22552n74m                                                                                                                                                                                                                                                                                                                                                                                                                                                                                                                      AC|||||||||||||||||||||||||||||||||||||||||||||||||||||||||||||||||||||||||||||||||||||||||||||||||||||||||||||||||||| 
    3 pairs 
 29:6
50:3
27:83 
     TCGA-02-2483-01A chr6-chr6 152607923 155138912 rr 
 
10 5 7 0 56 53
CTATTAGACCATGATAAAATTAAGAACTTCTTTCCATCAACAGATATTAT GAGAAACTCAAAAGGCATTCTATCATTTTATTTTCACTCTATACCTAATA
33333344444444455677888999999999999999999999999999 99999999999999999999999887777777666666666554332221
SYNE1 intron51(152605307-152614720) SCAF8 intron13(155136950-155139610)
 
 blast search - human genome  
 left 
 >ref|NW_001838990.2| Homo sapiens chromosome 6 genomic contig, alternate assembly 
HuRef SCAF_1103279188145, whole genome shotgun sequence
 gb|DS486018.1| Homo sapiens SCAF_1103279188145 genomic scaffold, whole genome 
shotgun sequence
Length=51215202

 Score = 93.5 bits (50),  Expect = 5e-17
 Identities = 50/50 (100%), Gaps = 0/50 (0%)
 Strand=Plus/Plus

Query  1        CTATTAGACCATGATAAAATTAAGAACTTCTTTCCATCAACAGATATTAT  50
                ||||||||||||||||||||||||||||||||||||||||||||||||||
Sbjct  4952743  CTATTAGACCATGATAAAATTAAGAACTTCTTTCCATCAACAGATATTAT  4952792


>gb|CH471051.2| Homo sapiens 181000117649897 genomic scaffold, whole genome shotgun 
sequence
Length=103786604

 Score = 93.5 bits (50),  Expect = 5e-17
 Identities = 50/50 (100%), Gaps = 0/50 (0%)
 Strand=Plus/Minus

Query  1         CTATTAGACCATGATAAAATTAAGAACTTCTTTCCATCAACAGATATTAT  50
                 ||||||||||||||||||||||||||||||||||||||||||||||||||
Sbjct  85410394  CTATTAGACCATGATAAAATTAAGAACTTCTTTCCATCAACAGATATTAT  85410345


>ref|NT_025741.15| Homo sapiens chromosome 6 genomic contig, GRCh37.p9 Primary Assembly
 gb|GL000054.1| Homo sapiens chromosome 6 genomic contig, GRCh37 reference primary 
assembly
Length=75224524

 Score = 93.5 bits (50),  Expect = 5e-17
 Identities = 50/50 (100%), Gaps = 0/50 (0%)
 Strand=Plus/Minus

Query  1         CTATTAGACCATGATAAAATTAAGAACTTCTTTCCATCAACAGATATTAT  50
                 ||||||||||||||||||||||||||||||||||||||||||||||||||
Sbjct  56777430  CTATTAGACCATGATAAAATTAAGAACTTCTTTCCATCAACAGATATTAT  56777381


>ref|NC_000006.11| Homo sapiens chromosome 6, GRCh37.p9 Primary Assembly
 gb|CM000668.1| Homo sapiens chromosome 6, GRCh37 primary reference assembly
Length=171115067

 Score = 93.5 bits (50),  Expect = 5e-17
 Identities = 50/50 (100%), Gaps = 0/50 (0%)
 Strand=Plus/Minus

Query  1          CTATTAGACCATGATAAAATTAAGAACTTCTTTCCATCAACAGATATTAT  50
                  ||||||||||||||||||||||||||||||||||||||||||||||||||
Sbjct  152607973  CTATTAGACCATGATAAAATTAAGAACTTCTTTCCATCAACAGATATTAT  152607924


>ref|AC_000138.1| Homo sapiens chromosome 6, alternate assembly HuRef, whole genome 
shotgun sequence
 gb|CM000467.1| Homo sapiens chromosome 6, whole genome shotgun sequence
Length=168433673

 Score = 93.5 bits (50),  Expect = 5e-17
 Identities = 50/50 (100%), Gaps = 0/50 (0%)
 Strand=Plus/Minus

Query  1          CTATTAGACCATGATAAAATTAAGAACTTCTTTCCATCAACAGATATTAT  50
                  ||||||||||||||||||||||||||||||||||||||||||||||||||
Sbjct  150170155  CTATTAGACCATGATAAAATTAAGAACTTCTTTCCATCAACAGATATTAT  150170106


>gb|CM000257.1| Homo sapiens chromosome 6, whole genome shotgun sequence
Length=171718000

 Score = 93.5 bits (50),  Expect = 5e-17
 Identities = 50/50 (100%), Gaps = 0/50 (0%)
 Strand=Plus/Minus

Query  1          CTATTAGACCATGATAAAATTAAGAACTTCTTTCCATCAACAGATATTAT  50
                  ||||||||||||||||||||||||||||||||||||||||||||||||||
Sbjct  153341790  CTATTAGACCATGATAAAATTAAGAACTTCTTTCCATCAACAGATATTAT  153341741


Lambda     K      H
    1.33    0.621     1.12 

Gapped
Lambda     K      H
    1.28    0.460    0.850 

Effective search space used: 681780354108
 
 right 
 >ref|NW_001838990.2| Homo sapiens chromosome 6 genomic contig, alternate assembly 
HuRef SCAF_1103279188145, whole genome shotgun sequence
 gb|DS486018.1| Homo sapiens SCAF_1103279188145 genomic scaffold, whole genome 
shotgun sequence
Length=51215202

 Score = 93.5 bits (50),  Expect = 5e-17
 Identities = 50/50 (100%), Gaps = 0/50 (0%)
 Strand=Plus/Plus

Query  1        GAGAAACTCAAAAGGCATTCTATCATTTTATTTTCACTCTATACCTAATA  50
                ||||||||||||||||||||||||||||||||||||||||||||||||||
Sbjct  2421243  GAGAAACTCAAAAGGCATTCTATCATTTTATTTTCACTCTATACCTAATA  2421292


>gb|CH471051.2| Homo sapiens 181000117649897 genomic scaffold, whole genome shotgun 
sequence
Length=103786604

 Score = 93.5 bits (50),  Expect = 5e-17
 Identities = 50/50 (100%), Gaps = 0/50 (0%)
 Strand=Plus/Minus

Query  1         GAGAAACTCAAAAGGCATTCTATCATTTTATTTTCACTCTATACCTAATA  50
                 ||||||||||||||||||||||||||||||||||||||||||||||||||
Sbjct  87941740  GAGAAACTCAAAAGGCATTCTATCATTTTATTTTCACTCTATACCTAATA  87941691


>ref|NT_025741.15| Homo sapiens chromosome 6 genomic contig, GRCh37.p9 Primary Assembly
 gb|GL000054.1| Homo sapiens chromosome 6 genomic contig, GRCh37 reference primary 
assembly
Length=75224524

 Score = 93.5 bits (50),  Expect = 5e-17
 Identities = 50/50 (100%), Gaps = 0/50 (0%)
 Strand=Plus/Minus

Query  1         GAGAAACTCAAAAGGCATTCTATCATTTTATTTTCACTCTATACCTAATA  50
                 ||||||||||||||||||||||||||||||||||||||||||||||||||
Sbjct  59308370  GAGAAACTCAAAAGGCATTCTATCATTTTATTTTCACTCTATACCTAATA  59308321


>ref|NC_000006.11| Homo sapiens chromosome 6, GRCh37.p9 Primary Assembly
 gb|CM000668.1| Homo sapiens chromosome 6, GRCh37 primary reference assembly
Length=171115067

 Score = 93.5 bits (50),  Expect = 5e-17
 Identities = 50/50 (100%), Gaps = 0/50 (0%)
 Strand=Plus/Minus

Query  1          GAGAAACTCAAAAGGCATTCTATCATTTTATTTTCACTCTATACCTAATA  50
                  ||||||||||||||||||||||||||||||||||||||||||||||||||
Sbjct  155138913  GAGAAACTCAAAAGGCATTCTATCATTTTATTTTCACTCTATACCTAATA  155138864


>ref|AC_000138.1| Homo sapiens chromosome 6, alternate assembly HuRef, whole genome 
shotgun sequence
 gb|CM000467.1| Homo sapiens chromosome 6, whole genome shotgun sequence
Length=168433673

 Score = 93.5 bits (50),  Expect = 5e-17
 Identities = 50/50 (100%), Gaps = 0/50 (0%)
 Strand=Plus/Minus

Query  1          GAGAAACTCAAAAGGCATTCTATCATTTTATTTTCACTCTATACCTAATA  50
                  ||||||||||||||||||||||||||||||||||||||||||||||||||
Sbjct  152701655  GAGAAACTCAAAAGGCATTCTATCATTTTATTTTCACTCTATACCTAATA  152701606


>gb|CM000257.1| Homo sapiens chromosome 6, whole genome shotgun sequence
Length=171718000

 Score = 93.5 bits (50),  Expect = 5e-17
 Identities = 50/50 (100%), Gaps = 0/50 (0%)
 Strand=Plus/Minus

Query  1          GAGAAACTCAAAAGGCATTCTATCATTTTATTTTCACTCTATACCTAATA  50
                  ||||||||||||||||||||||||||||||||||||||||||||||||||
Sbjct  155873136  GAGAAACTCAAAAGGCATTCTATCATTTTATTTTCACTCTATACCTAATA  155873087


Lambda     K      H
    1.33    0.621     1.12 

Gapped
Lambda     K      H
    1.28    0.460    0.850 

Effective search space used: 681780354108
 
 blast search - nt  
 left 
 >ref|NG_012855.1| Homo sapiens spectrin repeat containing, nuclear envelope 1 (SYNE1), 
RefSeqGene on chromosome 6
Length=522713

 Score = 93.5 bits (50),  Expect = 6e-17
 Identities = 50/50 (100%), Gaps = 0/50 (0%)
 Strand=Plus/Plus

Query  1       CTATTAGACCATGATAAAATTAAGAACTTCTTTCCATCAACAGATATTAT  50
               ||||||||||||||||||||||||||||||||||||||||||||||||||
Sbjct  355562  CTATTAGACCATGATAAAATTAAGAACTTCTTTCCATCAACAGATATTAT  355611


>emb|AL138832.10| Human DNA sequence from clone RP3-445H2 on chromosome 6q25.1-26 
Contains part of the synaptic nuclei expressed gene 1b (SYNE-1) 
(nesprin-1 beta, MYNE1, SYNE1, KIAA0796) and the gene 
for a novel protein, complete sequence
Length=139011

 Score = 93.5 bits (50),  Expect = 6e-17
 Identities = 50/50 (100%), Gaps = 0/50 (0%)
 Strand=Plus/Minus

Query  1       CTATTAGACCATGATAAAATTAAGAACTTCTTTCCATCAACAGATATTAT  50
               ||||||||||||||||||||||||||||||||||||||||||||||||||
Sbjct  120345  CTATTAGACCATGATAAAATTAAGAACTTCTTTCCATCAACAGATATTAT  120296


Lambda     K      H
    1.33    0.621     1.12 

Gapped
Lambda     K      H
    1.28    0.460    0.850 

Effective search space used: 874911716973
 
 right 
 
 
    reads 
 chr6 chr6 152608293 152608217 76m                            AAATGC
chr6 chr6 152608282 152608206 76m                            AAATGCTGCTTGGATGT
chr6 chr6 152608258 152608182 76m                            AAATGCTGCTTGGATGTCATAACTGTCAAATTACTATAAAA
chr6 chr6 152608253 152608177 76m                            AAATGCTGCTTGGATGTCATAACTGTCAAATTACTATAAAAGTTTC
chr6 chr6 152608249 152608173 76m                            AAATGCTGCTTGGATGTCATAACTGTCAAATTACTATAAAAGTTTCTTAG
chr6 chr6 152608246 152608170 76m                            AAATGCTGCTTGGATGTCATAACTGTCAAATTACTATAAAAGTTTCTTAGCAC
chr6 chr6 152608241 152608165 76m                            AAATGCTGCTTGGATGTCATAACTGTCAAATTACTATAAAAGTTTCTTAGCACTTGCT
chr6 chr6 152608237 152608161 76m                            AAATGCTGCTTGGATGTCATAACTGTCAAATTACTATAAAAGTTTCTTAGCACTTGCTCTCA
chr6 chr6 152608230 152608154 76m                            AAATGCTGCTTGGATGTCATAACTGTCAAATTACTATAAAAGTTTCTTAGCACTTGCTCTCAATATGTT
chr6 chr6 152608220 152608144 76m                               TGCTGCTTGGATGTCATAACTGTCAAATTACTATAAAAGTTTCTTAGCACTTGCTCTCAATATGTTTATCTCATCA
chr6 chr6 152608217 152608141 76m                                  CGCTTGGATGTCATAACTGTCAAATTACTATAAAAGTTTCTTAGCACTTGCTCTCAATATGTTTATCTCATCATAA
chr6 chr6 152608205 152608129 76m                                              ATAACTGTCAAATTACTATAAAAGTTTCTTAGCACTTGCTCTCAATATGTTTATCTCATCATAAGCCAGTAACAAA
chr6 chr6 152608200 152608124 76m                                                   TGTCAAATTACTATAAAAGTTTCTTAGCACTTGCTCTCAATATGTTTATCTCATCATAAGCCAGTAACAAACATTG
chr6 chr6 152608194 152608118 76m                                                         ATTACTATAAAAGTTTCTTAGCACTTGCTCTCAATATGTTTATCTCATCATAAGCCAGTAACAAACATTGTGCAGA
chr6 chr6 152608188 152608112 76m                                                               ATAAAAGTTTCTTAGCACTTGCTCTCAATATGTTTATCTCATCATAAGCCAGTAACAAACATTGTGCAGACTGTTA
chr6 chr6 152608184 152608108 76m                                                                   AAGTTTCTTAGCACTTGCTCTCAATATGTTTATCTCATCATAAGCCAGTAACAAACATTGTGCAGACTGTTATTAT
chr6 chr6 152608180 152608104 76m                                                                       TTCTTAGCACTTGCTCTCAATATGTTTATCTCATCATAAGCCAGTAACAAACATTGTGCAGACTGTTATTATTCTG
chr6 chr6 152608177 152608101 76m                                                                          TTAGCACTTGCTCTCAATATGTTTATCTCATCATAAGCCAGTAACAAACATTGTGCAGACTGTTATTATTCTGTGG
chr6 chr6 152608171 152608095 76m                                                                                CTTGCTCTCAATATGTTTATCTCATCATAAGCCAGTAACAAACATTGTGCAGACTGTTATTATTCTGTGGACCACG
chr6 chr6 152608168 152608092 76m                                                                                   GCTCTCAATATGTTTATCTCATCATAAGCCAGTAACAAACATTGTGCAGACTGTTATTATTCTGTGGACCACGCTT
chr6 chr6 152608164 152608088 76m                                                                                       TCAATATGTTTATCTCATCATAAGCCAGTAACAAACATTGTGCAGACTGTTATTATTCTGTGGACCACGCTTTGAG
chr6 chr6 152608158 152608082 76m                                                                                             TGTTTATCTCATCATAAGCCAGTAACAAAGATTGTGCAGACTGTTATTATTCTGTGGACCACGCTTTGAGTAGCAT
chr6 chr6 152608153 152608077 76m                                                                                                  ATCTCATCATAAGCCAGTAACAAACATTGTGCAGACTGTTATTATTCTGTGGACCACGCTTTGAGTAGCATTCTTA
chr6 chr6 152608147 152608071 76m                                                                                                        TCATAAGCCAGTAACAAACATTGTGCAGACTGTTATTATTCTGTGGACCACGCTTTGAGTAGCATTCTTACAGAAG
chr6 chr6 152608142 152608066 76m                                                                                                             CGCCAGTAACAAACATTGTGCAGACTGTTATTATTCTGTGGACCACGCTTTGAGTAGCATTCTTACAGAAGATAAC
chr6 chr6 152608139 152608063 76m                                                                                                                CAGTAACAAACATTGTGCAGACTGTTATTATTCTGTGGACCACGCTTTGAGTAGCATTCTTACAGAAGATAACGTA
chr6 chr6 152608135 152608059 76m                                                                                                                    AACAAACATTGTGCAGACTGTTATTATTCTGTGGACCACGCTTTGAGTAGCATTCTTACAGAAGATAACGTATGCG
chr6 chr6 152608132 152608056 76m                                                                                                                       AAACATTGTGCAGACTGTTATTATTCTGTGGACCACGCTTTGAGTAGCATTCTTACAGAAGATAACGTATGCGAAT
chr6 chr6 152608129 152608053 76m                                                                                                                          CATTGTGCAGACTGTTATTATTCTGTGGACCACGCTTTGAGTAGCATTCTTACAGAAGATAACGTATGCGAATGTA
chr6 chr6 152608126 152608050 76m                                                                                                                             TGTGCAGACTGTTATTATTCTGTGGACCACGCTTTGAGTAGCATTCTTACAGAAGATAACGTATGCGAATGTATTC
chr6 chr6 152608123 152608047 76m                                                                                                                                GCAGACTGTTATTATTCTGTGGACCACGCTTTGAGTAGCATTCTTACAGAAGATAACGTATGCGAATGTATTCTAT
chr6 chr6 152608112 152608036 76m                                                                                                                                           TTATTCTGTGGACCACGCTTTGAGTAGCATTCTTACAGAAGATAACGTATGCGAATGTATTCTATTTATGACCTTG
chr6 chr6 152608108 152608032 76m                                                                                                                                               TCTGTGGACCACGCTTTGAGTAGCATTCTTACAGAAGATAACGTATGCGAATGTATTCTATTTATGACCTTGGATT
chr6 chr6 152608099 152608023 76m                                                                                                                                                        CACGCTTTGAGTAGCATTCTTACAGAAGATAACATATGCGAATGTATTCTATTTATGACCTTGGATTAGGCAAAAG
chr6 chr6 152608096 152608020 76m                                                                                                                                                           GCTTTGAGTAGCATTCTTACAGAAGATAACGTATGCGAATGTATTCTATTTATGACCTTGGATTAGGCAAAAGATT
chr6 chr6 152608076 152608000 76m                                                                                                                                                                               AGAAGATAACGTATGCGAATGTATTCTATTTATGACCTTGGATTAGGCAAAAGATTTCCTATATAGTATACCAAAA
chr6 chr6 152608064 152607988 76m                                                                                                                                                                                           ATGCGAATGTATTCTATTTATGACCTTGGATTAGGCAAAAGATTTCCTATATAGTATACCAAAAGCCCTAACAATA
chr6 chr6 152608061 152607985 76m                                                                                                                                                                                              CGAATGTATTCTATTTATGACCTTGGATTAGGCAAAAGATTTCCTATATAGTATACCAAAAGCCCTAACAATAAAG
chr6 chr6 152608049 152607973 76m                                                                                                                                                                                                          ATTTATGACCTTGGATTAGGCAAAAGATTTCCTATATAGTATACCAAAAGCCCTAACAATAAAGGGAAATATTGAT
chr6 chr6 152608041 152607965 76m                                                                                                                                                                                                                  CCTTGGATTAGGCAAAAGATTTCCTATATAGTATACCAAAAGCCCTAACAATAAAGGGAAATATTGATTCTATTAG
chr6 chr6 152608036 152607960 76m                                                                                                                                                                                                                       GATTAGGCAAAAGATTTCCTATATAGTATACCAAAAGCCCTAACAATAAAGGGAAATATTGATTCTATTAGACCAT
chr6 chr6 152608028 152607952 76m                                                                                                                                                                                                                               AAAAGATTTCCTATATAGTATACCAAAAGCCCTAACAATAAAGGGAAATATTGATTCTATTAGACCATGATAAAAT
chr6 chr6 152608025 152607949 76m                                                                                                                                                                                                                                  AGATTTCCTATATAGTATACCAAAAGCCCTAACAATAAAGGGAAATATTGATTCTATTAGACCATGATAAAATTAA
chr6 chr6 152608015 152607939 76m                                                                                                                                                                                                                                            TATAGTATACCAAAAGCCCTAACAATAAAGGGAAATATTGATTCTATTAGACCATGATAAAATTAAGAACTTCTTT
chr6 chr6 152608004 152607928 76m                                                                                                                                                                                                                                                       AAAGGCCCTAACAATAAAGGGAAATATTGATTCTATTAGACCATGATAAAATTAAGAACTTCTTTCCATCAACAGA
chr6 chr6 152607995 155138909 73m155138913F3m                                                                                                                                                                                                                                                    AACAATAAAGGGAAATATTGATTCTATTAGACCATGATAAAATTAAGAACTTCTTTCCATCAACAGATATGAA GAG
chr6 chr6 152607985 155138899 63m155138913F13m                                                                                                                                                                                                                                                             GGAAATATTGATTCTATTAGACCATGATAAAATTAAGAACTTCTTTCCATCAACAGATATGAA GAGAAACTCAAAA
chr6 chr6 152607978 155138892 56m155138913F20m                                                                                                                                                                                                                                                                    TTGATTCTATTAGACCATGATAAAATTAAGAACTTCTTTCCATCAACAGATATGAA GAGAAACTCAAAAGGCATTC
chr6 chr6 152607975 155138889 53m155138913F23m                                                                                                                                                                                                                                                                       ATTCTATTAGACCATGATAAAATTAAGAACTTCTTTCCATCAACAGATATGAA GAGAAACTCAAAAGGCATTCTAT
chr6 chr6 152607973 155138887 51m155138913F25m                                                                                                                                                                                                                                                                         TCTATTAGACCATGATAAAATTAAGAACTTCTTTCCATCAACAGATATGAA GAGAAACTCAAAAGGCATTCTATCA
chr6 chr6 152607966 155138880 44m155138913F32m                                                                                                                                                                                                                                                                                GACCATGATAAAATTAAGAACTTCTTTCCATCAACAGATATGAA GAGAAACTCAAAAGGCATTCTATCATTTTATT
chr6 chr6 152607957 155138871 35m155138913F41m                                                                                                                                                                                                                                                                                         AAAATTAAGAACTTCTTTCCATCAACAGATATGAA GAGAAACTCAAAAGGCATTCTATCATTTTATTTTCACTCTA
chr6 chr6 152607955 155138869 33m155138913F43m                                                                                                                                                                                                                                                                                           AATTAAGAACTTCTTTCCATCAACAGATATGAA GAGAAACTCAAAAGGCATTCTATCATTTTATTTTCACTCTATA
chr6 chr6 152607954 155138868 32m155138913F44m                                                                                                                                                                                                                                                                                            ATTAAGAACTTCTTTCCATCAACAGATATGAA GAGAAACTCAAAAGGCATTCTATCATTTTATTTTCACTCTATAC
chr6 chr6 152607952 155138866 30m155138913F46m                                                                                                                                                                                                                                                                                              TAAGAACTTCTTTCCATCAACAGATATGAA GAGAAACTCAAAAGGCATTCTATCATTTTATTTTCACTCTATACCT
chr6 chr6 152607949 155138863 27m155138913F49m                                                                                                                                                                                                                                                                                                 GAACTTCTTTCCATCAACAGATATGAA GAGAAACTCAAAAGGCATTCTATCATTTTATTTTCACTCTATACCTAAT
chr6 chr6 152607945 155138859 23m155138913F53m                                                                                                                                                                                                                                                                                                     TTCTTTCCATCAACAGATATGAA GAGAAACTCAAAAGGCATTCTATCATTTTATTTTCACTCTATACCTAATAAGC
chr6 chr6 155138913 155138837 76m                                                                                                                                                                                                                                                                                                                                         AGAGAAACTCAAAAGGCATTCTATCATTTTATTTTCACTCTATACCTAATAAGCCATATCTGTTATTAAAGCTTAC
chr6 chr6 155138906 155138830 76m                                                                                                                                                                                                                                                                                                                                                CTCAAAAGGCATTCTATCATTTTATTTTCACTCTATACCTAATAAGCCATATCTGTTATTAAAGCTTACATAGAAA
chr6 chr6 155138900 155138824 76m                                                                                                                                                                                                                                                                                                                                                      AGGCATTCTATCATTTTATTTTCACTCTATACCTAATAAGCCATATCTGTTATTAAAGCTTACATAGAAACCTTTT
chr6 chr6 155138888 155138812 76m                                                                                                                                                                                                                                                                                                                                                                  ATTTTATTTTCACTCTATACCTAATAAGCCATATCTGTTATTAAAGCTTACATAGAAACCTTTTCCTCAGCCTTGC
chr6 chr6 155138883 155138807 76m                                                                                                                                                                                                                                                                                                                                                                       ATTTTCACTCTATACCTAATAAGCCATATCTGTTATTAAAGCTTACATAGAAACCTTTTCCTCAGCCTTGCTAACA
chr6 chr6 155138870 155138794 76m                                                                                                                                                                                                                                                                                                                                                                                    ACCTAATAAGCCATATCTGTTATTAAAGCTTACATAGAAACCTTTTCCTCAGCCTTGCTAACACTTCAAGCTACTG
chr6 chr6 155138865 155138789 76m                                                                                                                                                                                                                                                                                                                                                                                         ATAAGCCATATCTGTTATTAAAGCTTACATAGAAACCTTTTCCTCAGCCTTGCTAACACTTCAAGCTACTGCCTCT
chr6 chr6 155138848 155138772 76m                                                                                                                                                                                                                                                                                                                                                                                                          TTAAAGCTTACATAGAAACCTTTTCCTCAGCCTTGCTAACACTTCAAGCTACTGCCTCTTTAATATTAATATCAAA
chr6 chr6 155138837 155138761 76m                                                                                                                                                                                                                                                                                                                                                                                                                     ATAGAAACCTTTTCCTCAGCCTTGCTAACACTTCAAGCTACTGCCTCTTTAATATTAATATCAAACTTATTAAAAG
chr6 chr6 155138824 155138748 76m                                                                                                                                                                                                                                                                                                                                                                                                                                  CCTCAGCCTTGCTAACACTTCAAGCTACTGCCTCTTTAATATTAATATCAAACTTATTAAAAGAGAAATATATCTA
chr6 chr6 155138820 155138744 76m                                                                                                                                                                                                                                                                                                                                                                                                                                      CGCCTTGCTAACACTTCAAGCTACTGCCTCTTTAATATTAATATCAAACTTATTAAAAGAGAAATATATCTAAACC
chr6 chr6 155138817 155138741 76m                                                                                                                                                                                                                                                                                                                                                                                                                                         CTTGCTAACACTTCAAGCTACTGCCTCTTTAATATTAATATCAAACTTATTAAAAGAGAAATATATCTAAACCTTG
chr6 chr6 155138813 155138737 76m                                                                                                                                                                                                                                                                                                                                                                                                                                             CTAACACTTCAAGCTACTGCCTCTTTAATATTAATATCAAACTTATTAAAAGAGAAATATATCTAAACCTTGACAT
chr6 chr6 155138797 155138721 76m                                                                                                                                                                                                                                                                                                                                                                                                                                                             CTGCCTCTTTAATATTAATATCAAACTTATTAAAAGAGAAATATATCTAAACCTTGACATTCTTCTTTATCTATTA
chr6 chr6 155138792 155138716 76m                                                                                                                                                                                                                                                                                                                                                                                                                                                                  TCTTTAATATTAATATCAAACTTATTAAAAGAGAAATATATCTAAACCTTGACATTCTTCTTTATCTATTACAATC
chr6 chr6 155138789 155138713 76m                                                                                                                                                                                                                                                                                                                                                                                                                                                                     TTAATATTAATATCAAACTTATTAAAAGAGAAATATATCTAAACCTTGACATTCTTCTTTATCTATTACAATCTAG
chr6 chr6 155138782 155138706 76m                                                                                                                                                                                                                                                                                                                                                                                                                                                                            TAATATCAAACTTATTAAAAGAGAAATATATCTAAACCTTGACATTCTTCTTTATCTATTACAATCTAGCTCTAAT
chr6 chr6 155138778 155138702 76m                                                                                                                                                                                                                                                                                                                                                                                                                                                                                ATCAAACTTATTAAAAGAGAAATATATCTAAACCTTGACATTCTTCTTTATCTATTACAATCTAGCTCTAATTCCT
chr6 chr6 155138766 155138690 76m                                                                                                                                                                                                                                                                                                                                                                                                                                                                                            AAAAGAGAAATATATCTAAACCTTGACATTCTTCTTTATCTATTACAATCTAGCTCTAATTCCTCACCAACCCACT
chr6 chr6 155138757 155138681 76m                                                                                                                                                                                                                                                                                                                                                                                                                                                                                                     ATATATCTAAACCTTGACATTCTTCTTTATCTATTACAATCTAGCTCTAATTCCTCACCAACCCACTGGAAAATAC
chr6 chr6 155138746 155138670 76m                                                                                                                                                                                                                                                                                                                                                                                                                                                                                                                CCTTGACATTCTTCTTTATCTATTACAATCTAGCTCTAATTCCTCACCAACCCACTGGAAAATACTTTACTCTCTT
chr6 chr6 155138743 155138667 76m                                                                                                                                                                                                                                                                                                                                                                                                                                                                                                                   TGACATTCTTCTTTATCTATTACAATCTAGCTCTAATTCCTCACCAACCCACTGGAAAATACTTTACTCTCTTCAA
chr6 chr6 155138739 155138663 76m                                                                                                                                                                                                                                                                                                                                                                                                                                                                                                                       ATTCCTCTTTATCTATTACAATCTAGCTCTAATTCCTCACCAACCCACTGGAAAATACTTTACTCTCTTCAAGCTN
chr6 chr6 155138736 155138660 76m                                                                                                                                                                                                                                                                                                                                                                                                                                                                                                                          CTTCTTTATCTATTACAATCTAGCTCTAATTCCTCACCAACCCACTGGAAAATACTTTACTCTCTTCAAGCTCACC
chr6 chr6 155138713 155138637 76m                                                                                                                                                                                                                                                                                                                                                                                                                                                                                                                                                 CTCTAATTCCTCACCAACCCACTGGAAAATACTTTACTCTCTTCAAGCTCACCAATGACCTACTAAATGCCAAACT
chr6 chr6 155138707 155138631 76m                                                                                                                                                                                                                                                                                                                                                                                                                                                                                                                                                       TTCCTCACCAACCCACTGGAAAATACTTTACTCTCTTCAAGCTCACCAATGACCTACTAAATGCCAAACTGAATGG
chr6 chr6 155138697 155138621 76m                                                                                                                                                                                                                                                                                                                                                                                                                                                                                                                                                                 ACCCACTGGAAAATACTTTACTATCTTCAAGCTCACCAATGACCTACTAAATGCCAAACTGAATGGACTATGTCTT
chr6 chr6 155138688 155138612 76m                                                                                                                                                                                                                                                                                                                                                                                                                                                                                                                                                                          AAAATTCTTTACTCTCTTCAAGCTCACCAATGACCTACTAAATGCCAAACTGAATGGACTTTGTCTTTGAAGTC
chr6 chr6 155138685 155138609 76m                                                                                                                                                                                                                                                                                                                                                                                                                                                                                                                                                                             ATACTTTACTCTCTTCAAGCTCACCAATGACCTACTAAATGCCAAACTGAATGGACTTTGTCTTTGAAGTC
chr6 chr6 155138677 155138601 76m                                                                                                                                                                                                                                                                                                                                                                                                                                                                                                                                                                                     CTCTCTTCAAGCTCACCAATGACCTACTAAATGCCAAACTGAATGGACTTTGTCTTTGAAGTC
chr6 chr6 155138667 155138591 76m                                                                                                                                                                                                                                                                                                                                                                                                                                                                                                                                                                                               GCTCACCAATGACCTACTAAATGCCAAACTGAATGGACTTTGTCTTTGAAGTC
chr6 chr6 155138661 155138585 76m                                                                                                                                                                                                                                                                                                                                                                                                                                                                                                                                                                                                     CAATGACCTACTAAATGCCAAACTGAATGGACTTTGTCTTTGAAGTC
chr6 chr6 155138655 155138579 76m                                                                                                                                                                                                                                                                                                                                                                                                                                                                                                                                                                                                           CCTACTAAATGCCAAACTGAATGGACTTTGTCTTTGAAGTC
chr6 chr6 155138651 155138575 76m                                                                                                                                                                                                                                                                                                                                                                                                                                                                                                                                                                                                               CTAAATGCCAAACTGAATGGACTTTGTCTTTGAAGTC
chr6 chr6 155138645 155138569 76m                                                                                                                                                                                                                                                                                                                                                                                                                                                                                                                                                                                                                     GCCAGACTGAATGGACTTTGTCTTTGAAGTC
chr6 chr6 155138629 155138553 76m                                                                                                                                                                                                                                                                                                                                                                                                                                                                                                                                                                                                                                     TTTGTCTTTGAAGTC 
    5 pairs 
 6:12
38:-2
110:5
43:123
101:74 
     TCGA-02-2483-01A chr9-chr16 119976636 331745 rf 
 
12 24 17 0 52 51
GGACAGTCTGGGACATCCAGGGGAAGAGAAGGTGGACTTTGAGAAGAAAG TCCTCCTGGCTGACAAGGAGGGTGGGCCGCCGGCAGTGGACGAGGTGTTG
13355589999999999999999999999999999999999999999999 99999999999999999999999999999777433222222222222111
ASTN2 exon20(119976635-119977020) ARHGDIG exon2(331744-331924)
 
 blast search - human genome  
 left 
 >ref|NW_001839237.2| Homo sapiens chromosome 9 genomic contig, alternate assembly 
HuRef SCAF_1103279187522, whole genome shotgun sequence
 gb|DS486153.1| Homo sapiens SCAF_1103279187522 genomic scaffold, whole genome 
shotgun sequence
Length=4804840

 Score = 93.5 bits (50),  Expect = 5e-17
 Identities = 50/50 (100%), Gaps = 0/50 (0%)
 Strand=Plus/Plus

Query  1        GGACAGTCTGGGACATCCAGGGGAAGAGAAGGTGGACTTTGAGAAGAAAG  50
                ||||||||||||||||||||||||||||||||||||||||||||||||||
Sbjct  4589607  GGACAGTCTGGGACATCCAGGGGAAGAGAAGGTGGACTTTGAGAAGAAAG  4589656


>gb|CH471090.1| Homo sapiens 211000035837865 genomic scaffold, whole genome shotgun 
sequence
Length=25070985

 Score = 93.5 bits (50),  Expect = 5e-17
 Identities = 50/50 (100%), Gaps = 0/50 (0%)
 Strand=Plus/Minus

Query  1        GGACAGTCTGGGACATCCAGGGGAAGAGAAGGTGGACTTTGAGAAGAAAG  50
                ||||||||||||||||||||||||||||||||||||||||||||||||||
Sbjct  4112220  GGACAGTCTGGGACATCCAGGGGAAGAGAAGGTGGACTTTGAGAAGAAAG  4112171


>ref|NT_008470.19| Homo sapiens chromosome 9 genomic contig, GRCh37.p9 Primary Assembly
 gb|GL000089.1| Homo sapiens chromosome 9 genomic contig, GRCh37 reference primary 
assembly
Length=62237592

 Score = 93.5 bits (50),  Expect = 5e-17
 Identities = 50/50 (100%), Gaps = 0/50 (0%)
 Strand=Plus/Minus

Query  1         GGACAGTCTGGGACATCCAGGGGAAGAGAAGGTGGACTTTGAGAAGAAAG  50
                 ||||||||||||||||||||||||||||||||||||||||||||||||||
Sbjct  49141218  GGACAGTCTGGGACATCCAGGGGAAGAGAAGGTGGACTTTGAGAAGAAAG  49141169


>ref|NC_000009.11| Homo sapiens chromosome 9, GRCh37.p9 Primary Assembly
 gb|CM000671.1| Homo sapiens chromosome 9, GRCh37 primary reference assembly
Length=141213431

 Score = 93.5 bits (50),  Expect = 5e-17
 Identities = 50/50 (100%), Gaps = 0/50 (0%)
 Strand=Plus/Minus

Query  1          GGACAGTCTGGGACATCCAGGGGAAGAGAAGGTGGACTTTGAGAAGAAAG  50
                  ||||||||||||||||||||||||||||||||||||||||||||||||||
Sbjct  119976686  GGACAGTCTGGGACATCCAGGGGAAGAGAAGGTGGACTTTGAGAAGAAAG  119976637


>ref|AC_000141.1| Homo sapiens chromosome 9, alternate assembly HuRef, whole genome 
shotgun sequence
 gb|CM000470.1| Homo sapiens chromosome 9, whole genome shotgun sequence
Length=110559325

 Score = 93.5 bits (50),  Expect = 5e-17
 Identities = 50/50 (100%), Gaps = 0/50 (0%)
 Strand=Plus/Minus

Query  1         GGACAGTCTGGGACATCCAGGGGAAGAGAAGGTGGACTTTGAGAAGAAAG  50
                 ||||||||||||||||||||||||||||||||||||||||||||||||||
Sbjct  89595074  GGACAGTCTGGGACATCCAGGGGAAGAGAAGGTGGACTTTGAGAAGAAAG  89595025


>gb|CM000260.1| Homo sapiens chromosome 9, whole genome shotgun sequence
Length=111583154

 Score = 93.5 bits (50),  Expect = 5e-17
 Identities = 50/50 (100%), Gaps = 0/50 (0%)
 Strand=Plus/Minus

Query  1         GGACAGTCTGGGACATCCAGGGGAAGAGAAGGTGGACTTTGAGAAGAAAG  50
                 ||||||||||||||||||||||||||||||||||||||||||||||||||
Sbjct  90624389  GGACAGTCTGGGACATCCAGGGGAAGAGAAGGTGGACTTTGAGAAGAAAG  90624340


>gb|CH003504.1| Homo sapiens chromosome 9, whole genome shotgun sequence
Length=116547660

 Score = 86.1 bits (46),  Expect = 8e-15
 Identities = 49/50 (98%), Gaps = 1/50 (2%)
 Strand=Plus/Minus

Query  1         GGACAGTCTGGGACATCCAGGGGAAGAGAAGGTGGACTTTGAGAAGAAAG  50
                 |||||||||||||||||||||||||||||||||||| |||||||||||||
Sbjct  95205951  GGACAGTCTGGGACATCCAGGGGAAGAGAAGGTGGA-TTTGAGAAGAAAG  95205903


Lambda     K      H
    1.33    0.621     1.12 

Gapped
Lambda     K      H
    1.28    0.460    0.850 

Effective search space used: 681780354108
 
 right 
 >ref|NW_001838339.2| Homo sapiens chromosome 16 genomic contig, alternate assembly 
HuRef SCAF_1103279188181, whole genome shotgun sequence
 gb|DS486217.1| Homo sapiens SCAF_1103279188181 genomic scaffold, whole genome 
shotgun sequence
Length=2624016

 Score = 93.5 bits (50),  Expect = 5e-17
 Identities = 50/50 (100%), Gaps = 0/50 (0%)
 Strand=Plus/Minus

Query  1        TCCTCCTGGCTGACAAGGAGGGTGGGCCGCCGGCAGTGGACGAGGTGTTG  50
                ||||||||||||||||||||||||||||||||||||||||||||||||||
Sbjct  2374025  TCCTCCTGGCTGACAAGGAGGGTGGGCCGCCGGCAGTGGACGAGGTGTTG  2373976


>ref|NT_010393.16| Homo sapiens chromosome 16 genomic contig, GRCh37.p9 Primary 
Assembly
 gb|GL000124.1| Homo sapiens chromosome 16 genomic contig, GRCh37 reference primary 
assembly
Length=33963150

 Score = 93.5 bits (50),  Expect = 5e-17
 Identities = 50/50 (100%), Gaps = 0/50 (0%)
 Strand=Plus/Plus

Query  1       TCCTCCTGGCTGACAAGGAGGGTGGGCCGCCGGCAGTGGACGAGGTGTTG  50
               ||||||||||||||||||||||||||||||||||||||||||||||||||
Sbjct  271746  TCCTCCTGGCTGACAAGGAGGGTGGGCCGCCGGCAGTGGACGAGGTGTTG  271795


>ref|NC_000016.9| Homo sapiens chromosome 16, GRCh37.p9 Primary Assembly
 gb|CM000678.1| Homo sapiens chromosome 16, GRCh37 primary reference assembly
Length=90354753

 Score = 93.5 bits (50),  Expect = 5e-17
 Identities = 50/50 (100%), Gaps = 0/50 (0%)
 Strand=Plus/Plus

Query  1       TCCTCCTGGCTGACAAGGAGGGTGGGCCGCCGGCAGTGGACGAGGTGTTG  50
               ||||||||||||||||||||||||||||||||||||||||||||||||||
Sbjct  331746  TCCTCCTGGCTGACAAGGAGGGTGGGCCGCCGGCAGTGGACGAGGTGTTG  331795


>ref|AC_000148.1| Homo sapiens chromosome 16, alternate assembly HuRef, whole genome 
shotgun sequence
 gb|CM000477.1| Homo sapiens chromosome 16, whole genome shotgun sequence
Length=75877710

 Score = 93.5 bits (50),  Expect = 5e-17
 Identities = 50/50 (100%), Gaps = 0/50 (0%)
 Strand=Plus/Plus

Query  1       TCCTCCTGGCTGACAAGGAGGGTGGGCCGCCGGCAGTGGACGAGGTGTTG  50
               ||||||||||||||||||||||||||||||||||||||||||||||||||
Sbjct  249992  TCCTCCTGGCTGACAAGGAGGGTGGGCCGCCGGCAGTGGACGAGGTGTTG  250041


>gb|CM000267.1| Homo sapiens chromosome 16, whole genome shotgun sequence
Length=75226909

 Score = 93.5 bits (50),  Expect = 5e-17
 Identities = 50/50 (100%), Gaps = 0/50 (0%)
 Strand=Plus/Plus

Query  1       TCCTCCTGGCTGACAAGGAGGGTGGGCCGCCGGCAGTGGACGAGGTGTTG  50
               ||||||||||||||||||||||||||||||||||||||||||||||||||
Sbjct  531648  TCCTCCTGGCTGACAAGGAGGGTGGGCCGCCGGCAGTGGACGAGGTGTTG  531697


Lambda     K      H
    1.33    0.621     1.12 

Gapped
Lambda     K      H
    1.28    0.460    0.850 

Effective search space used: 681780354108
 
 blast search - nt  
 left 
 >ref|XM_003833082.1| PREDICTED: Pan paniscus astrotactin 2 (ASTN2), mRNA
Length=4388

 Score = 93.5 bits (50),  Expect = 6e-17
 Identities = 50/50 (100%), Gaps = 0/50 (0%)
 Strand=Plus/Plus

Query  1    GGACAGTCTGGGACATCCAGGGGAAGAGAAGGTGGACTTTGAGAAGAAAG  50
            ||||||||||||||||||||||||||||||||||||||||||||||||||
Sbjct  720  GGACAGTCTGGGACATCCAGGGGAAGAGAAGGTGGACTTTGAGAAGAAAG  769


>ref|XM_002743253.2| PREDICTED: Callithrix jacchus astrotactin 2 (ASTN2), mRNA
Length=4562

 Score = 93.5 bits (50),  Expect = 6e-17
 Identities = 50/50 (100%), Gaps = 0/50 (0%)
 Strand=Plus/Plus

Query  1     GGACAGTCTGGGACATCCAGGGGAAGAGAAGGTGGACTTTGAGAAGAAAG  50
             ||||||||||||||||||||||||||||||||||||||||||||||||||
Sbjct  1053  GGACAGTCTGGGACATCCAGGGGAAGAGAAGGTGGACTTTGAGAAGAAAG  1102


>ref|XM_003312265.1| PREDICTED: Pan troglodytes astrotactin-2-like (LOC100609975), 
mRNA
Length=1665

 Score = 93.5 bits (50),  Expect = 6e-17
 Identities = 50/50 (100%), Gaps = 0/50 (0%)
 Strand=Plus/Plus

Query  1     GGACAGTCTGGGACATCCAGGGGAAGAGAAGGTGGACTTTGAGAAGAAAG  50
             ||||||||||||||||||||||||||||||||||||||||||||||||||
Sbjct  1062  GGACAGTCTGGGACATCCAGGGGAAGAGAAGGTGGACTTTGAGAAGAAAG  1111


>ref|XM_002820154.1| PREDICTED: Pongo abelii astrotactin-2-like (LOC100456865), partial 
mRNA
Length=2180

 Score = 93.5 bits (50),  Expect = 6e-17
 Identities = 50/50 (100%), Gaps = 0/50 (0%)
 Strand=Plus/Plus

Query  1    GGACAGTCTGGGACATCCAGGGGAAGAGAAGGTGGACTTTGAGAAGAAAG  50
            ||||||||||||||||||||||||||||||||||||||||||||||||||
Sbjct  750  GGACAGTCTGGGACATCCAGGGGAAGAGAAGGTGGACTTTGAGAAGAAAG  799


>ref|NG_021409.1| Homo sapiens astrotactin 2 (ASTN2), RefSeqGene on chromosome 
9
Length=996814

 Score = 93.5 bits (50),  Expect = 6e-17
 Identities = 50/50 (100%), Gaps = 0/50 (0%)
 Strand=Plus/Plus

Query  1       GGACAGTCTGGGACATCCAGGGGAAGAGAAGGTGGACTTTGAGAAGAAAG  50
               ||||||||||||||||||||||||||||||||||||||||||||||||||
Sbjct  205632  GGACAGTCTGGGACATCCAGGGGAAGAGAAGGTGGACTTTGAGAAGAAAG  205681


>ref|NM_014010.4| Homo sapiens astrotactin 2 (ASTN2), transcript variant 1, mRNA
Length=4604

 Score = 93.5 bits (50),  Expect = 6e-17
 Identities = 50/50 (100%), Gaps = 0/50 (0%)
 Strand=Plus/Plus

Query  1     GGACAGTCTGGGACATCCAGGGGAAGAGAAGGTGGACTTTGAGAAGAAAG  50
             ||||||||||||||||||||||||||||||||||||||||||||||||||
Sbjct  1067  GGACAGTCTGGGACATCCAGGGGAAGAGAAGGTGGACTTTGAGAAGAAAG  1116


>gb|AC240434.1| Homo sapiens FOSMID clone ABC16-1870K4 from chromosome 9, complete 
sequence
Length=33505

 Score = 93.5 bits (50),  Expect = 6e-17
 Identities = 50/50 (100%), Gaps = 0/50 (0%)
 Strand=Plus/Plus

Query  1      GGACAGTCTGGGACATCCAGGGGAAGAGAAGGTGGACTTTGAGAAGAAAG  50
              ||||||||||||||||||||||||||||||||||||||||||||||||||
Sbjct  15145  GGACAGTCTGGGACATCCAGGGGAAGAGAAGGTGGACTTTGAGAAGAAAG  15194


>dbj|AB383928.1| Synthetic construct DNA, clone: pF1KSDA0634, Homo sapiens ASTN2 
gene for astrotactin 2 isoform a, complete cds, without stop 
codon, in Flexi system
Length=3905

 Score = 93.5 bits (50),  Expect = 6e-17
 Identities = 50/50 (100%), Gaps = 0/50 (0%)
 Strand=Plus/Plus

Query  1     GGACAGTCTGGGACATCCAGGGGAAGAGAAGGTGGACTTTGAGAAGAAAG  50
             ||||||||||||||||||||||||||||||||||||||||||||||||||
Sbjct  993   GGACAGTCTGGGACATCCAGGGGAAGAGAAGGTGGACTTTGAGAAGAAAG  1042


>dbj|AK315025.1| Homo sapiens cDNA, FLJ95964, Homo sapiens astrotactin 2 (ASTN2), 
mRNA
Length=3032

 Score = 93.5 bits (50),  Expect = 6e-17
 Identities = 50/50 (100%), Gaps = 0/50 (0%)
 Strand=Plus/Plus

Query  1    GGACAGTCTGGGACATCCAGGGGAAGAGAAGGTGGACTTTGAGAAGAAAG  50
            ||||||||||||||||||||||||||||||||||||||||||||||||||
Sbjct  131  GGACAGTCTGGGACATCCAGGGGAAGAGAAGGTGGACTTTGAGAAGAAAG  180


>gb|BC146756.1| Homo sapiens astrotactin 2, mRNA (cDNA clone MGC:166822 IMAGE:9007192), 
complete cds
Length=4607

 Score = 93.5 bits (50),  Expect = 6e-17
 Identities = 50/50 (100%), Gaps = 0/50 (0%)
 Strand=Plus/Plus

Query  1     GGACAGTCTGGGACATCCAGGGGAAGAGAAGGTGGACTTTGAGAAGAAAG  50
             ||||||||||||||||||||||||||||||||||||||||||||||||||
Sbjct  1067  GGACAGTCTGGGACATCCAGGGGAAGAGAAGGTGGACTTTGAGAAGAAAG  1116


>dbj|AK127350.1| Homo sapiens cDNA FLJ45423 fis, clone BRHIP3036936, highly similar 
to Homo sapiens astrotactin 2 (ASTN2), transcript variant 
2, mRNA
Length=3812

 Score = 93.5 bits (50),  Expect = 6e-17
 Identities = 50/50 (100%), Gaps = 0/50 (0%)
 Strand=Plus/Plus

Query  1    GGACAGTCTGGGACATCCAGGGGAAGAGAAGGTGGACTTTGAGAAGAAAG  50
            ||||||||||||||||||||||||||||||||||||||||||||||||||
Sbjct  147  GGACAGTCTGGGACATCCAGGGGAAGAGAAGGTGGACTTTGAGAAGAAAG  196


>emb|AL355608.11| Human DNA sequence from clone RP11-567J2 on chromosome 9 Contains 
part of the ASTN2 gene for astrotactin 2 (KIAA0634) and 
a novel gene similar to ribosomal protein L10 (RPL10), complete 
sequence
Length=132590

 Score = 93.5 bits (50),  Expect = 6e-17
 Identities = 50/50 (100%), Gaps = 0/50 (0%)
 Strand=Plus/Minus

Query  1      GGACAGTCTGGGACATCCAGGGGAAGAGAAGGTGGACTTTGAGAAGAAAG  50
              ||||||||||||||||||||||||||||||||||||||||||||||||||
Sbjct  56945  GGACAGTCTGGGACATCCAGGGGAAGAGAAGGTGGACTTTGAGAAGAAAG  56896


>emb|BX649176.1| Homo sapiens mRNA; cDNA DKFZp686P1898 (from clone DKFZp686P1898)
Length=3950

 Score = 93.5 bits (50),  Expect = 6e-17
 Identities = 50/50 (100%), Gaps = 0/50 (0%)
 Strand=Plus/Plus

Query  1    GGACAGTCTGGGACATCCAGGGGAAGAGAAGGTGGACTTTGAGAAGAAAG  50
            ||||||||||||||||||||||||||||||||||||||||||||||||||
Sbjct  249  GGACAGTCTGGGACATCCAGGGGAAGAGAAGGTGGACTTTGAGAAGAAAG  298


>gb|AF116574.1|AF116574 Homo sapiens astrotactin2 (ASTN2) mRNA, complete cds
Length=3837

 Score = 93.5 bits (50),  Expect = 6e-17
 Identities = 50/50 (100%), Gaps = 0/50 (0%)
 Strand=Plus/Plus

Query  1    GGACAGTCTGGGACATCCAGGGGAAGAGAAGGTGGACTTTGAGAAGAAAG  50
            ||||||||||||||||||||||||||||||||||||||||||||||||||
Sbjct  288  GGACAGTCTGGGACATCCAGGGGAAGAGAAGGTGGACTTTGAGAAGAAAG  337


>dbj|AB014534.1| Homo sapiens mRNA for KIAA0634 protein, partial cds
Length=4591

 Score = 93.5 bits (50),  Expect = 6e-17
 Identities = 50/50 (100%), Gaps = 0/50 (0%)
 Strand=Plus/Plus

Query  1     GGACAGTCTGGGACATCCAGGGGAAGAGAAGGTGGACTTTGAGAAGAAAG  50
             ||||||||||||||||||||||||||||||||||||||||||||||||||
Sbjct  1067  GGACAGTCTGGGACATCCAGGGGAAGAGAAGGTGGACTTTGAGAAGAAAG  1116


>ref|XM_001098403.2| PREDICTED: Macaca mulatta astrotactin 2 (ASTN2), mRNA
Length=4720

 Score = 87.9 bits (47),  Expect = 3e-15
 Identities = 49/50 (98%), Gaps = 0/50 (0%)
 Strand=Plus/Plus

Query  1     GGACAGTCTGGGACATCCAGGGGAAGAGAAGGTGGACTTTGAGAAGAAAG  50
             |||||||||||||| |||||||||||||||||||||||||||||||||||
Sbjct  1049  GGACAGTCTGGGACGTCCAGGGGAAGAGAAGGTGGACTTTGAGAAGAAAG  1098


>ref|XM_003263952.1| PREDICTED: Nomascus leucogenys astrotactin-2-like (LOC100589612), 
mRNA
Length=2470

 Score = 86.1 bits (46),  Expect = 1e-14
 Identities = 48/49 (97%), Gaps = 0/49 (0%)
 Strand=Plus/Plus

Query  2    GACAGTCTGGGACATCCAGGGGAAGAGAAGGTGGACTTTGAGAAGAAAG  50
            ||||||||||||| |||||||||||||||||||||||||||||||||||
Sbjct  602  GACAGTCTGGGACGTCCAGGGGAAGAGAAGGTGGACTTTGAGAAGAAAG  650


Lambda     K      H
    1.33    0.621     1.12 

Gapped
Lambda     K      H
    1.28    0.460    0.850 

Effective search space used: 874911716973
 
 right 
 >ref|NM_001176.3| Homo sapiens Rho GDP dissociation inhibitor (GDI) gamma (ARHGDIG), 
mRNA
Length=942

 Score = 93.5 bits (50),  Expect = 6e-17
 Identities = 50/50 (100%), Gaps = 0/50 (0%)
 Strand=Plus/Plus

Query  1    TCCTCCTGGCTGACAAGGAGGGTGGGCCGCCGGCAGTGGACGAGGTGTTG  50
            ||||||||||||||||||||||||||||||||||||||||||||||||||
Sbjct  149  TCCTCCTGGCTGACAAGGAGGGTGGGCCGCCGGCAGTGGACGAGGTGTTG  198


>emb|CU690464.1| Synthetic construct Homo sapiens gateway clone IMAGE:100021341 
5' read ARHGDIG mRNA
Length=1249

 Score = 93.5 bits (50),  Expect = 6e-17
 Identities = 50/50 (100%), Gaps = 0/50 (0%)
 Strand=Plus/Plus

Query  1    TCCTCCTGGCTGACAAGGAGGGTGGGCCGCCGGCAGTGGACGAGGTGTTG  50
            ||||||||||||||||||||||||||||||||||||||||||||||||||
Sbjct  90   TCCTCCTGGCTGACAAGGAGGGTGGGCCGCCGGCAGTGGACGAGGTGTTG  139


>gb|DQ656001.2| Homo sapiens clone Affy08250F05, mRNA sequence
Length=520

 Score = 93.5 bits (50),  Expect = 6e-17
 Identities = 50/50 (100%), Gaps = 0/50 (0%)
 Strand=Plus/Minus

Query  1    TCCTCCTGGCTGACAAGGAGGGTGGGCCGCCGGCAGTGGACGAGGTGTTG  50
            ||||||||||||||||||||||||||||||||||||||||||||||||||
Sbjct  240  TCCTCCTGGCTGACAAGGAGGGTGGGCCGCCGGCAGTGGACGAGGTGTTG  191


>gb|EF450770.1| Homo sapiens protein disulfide isomerase family A, member 2 variant 
(PDIA2) gene, complete cds
Length=7706

 Score = 93.5 bits (50),  Expect = 6e-17
 Identities = 50/50 (100%), Gaps = 0/50 (0%)
 Strand=Plus/Plus

Query  1    TCCTCCTGGCTGACAAGGAGGGTGGGCCGCCGGCAGTGGACGAGGTGTTG  50
            ||||||||||||||||||||||||||||||||||||||||||||||||||
Sbjct  388  TCCTCCTGGCTGACAAGGAGGGTGGGCCGCCGGCAGTGGACGAGGTGTTG  437


>dbj|AB127078.1| Homo sapiens ARHGDIG, PDIP polycistronic mRNA for Rho GDP dissociation 
inhibitor gamma, pancreatic protein disulfide isomerase, 
complete cds
Length=2626

 Score = 93.5 bits (50),  Expect = 6e-17
 Identities = 50/50 (100%), Gaps = 0/50 (0%)
 Strand=Plus/Plus

Query  1    TCCTCCTGGCTGACAAGGAGGGTGGGCCGCCGGCAGTGGACGAGGTGTTG  50
            ||||||||||||||||||||||||||||||||||||||||||||||||||
Sbjct  74   TCCTCCTGGCTGACAAGGAGGGTGGGCCGCCGGCAGTGGACGAGGTGTTG  123


>gb|AF498928.1| Homo sapiens Rho GDP dissociation inhibitor gamma (ARHGDIG) mRNA, 
complete cds
Length=678

 Score = 93.5 bits (50),  Expect = 6e-17
 Identities = 50/50 (100%), Gaps = 0/50 (0%)
 Strand=Plus/Plus

Query  1    TCCTCCTGGCTGACAAGGAGGGTGGGCCGCCGGCAGTGGACGAGGTGTTG  50
            ||||||||||||||||||||||||||||||||||||||||||||||||||
Sbjct  74   TCCTCCTGGCTGACAAGGAGGGTGGGCCGCCGGCAGTGGACGAGGTGTTG  123


>gb|AE006463.1| Homo sapiens 16p13.3 sequence section 2 of 8
Length=260418

 Score = 93.5 bits (50),  Expect = 6e-17
 Identities = 50/50 (100%), Gaps = 0/50 (0%)
 Strand=Plus/Plus

Query  1      TCCTCCTGGCTGACAAGGAGGGTGGGCCGCCGGCAGTGGACGAGGTGTTG  50
              ||||||||||||||||||||||||||||||||||||||||||||||||||
Sbjct  14342  TCCTCCTGGCTGACAAGGAGGGTGGGCCGCCGGCAGTGGACGAGGTGTTG  14391


>emb|Z69667.1| Human DNA sequence from clone LA16c-314G4 on chromosome 16 Contains 
the 3' end of the C16orf9 gene for chromosome 16 open 
reading frame 9, the RGS11 gene for regulator of G-protein 
signalling 11, a novel gene, the ARHGDIG gene for Rho GDP dissociation 
inhibitor (GDI) gamma, the PDIP gene for protein 
disulfide isomerase, pancreatic, the 3' end of the AXIN1 gene 
for axin 1 and two CpG islands, complete sequence
Length=31557

 Score = 93.5 bits (50),  Expect = 6e-17
 Identities = 50/50 (100%), Gaps = 0/50 (0%)
 Strand=Plus/Plus

Query  1      TCCTCCTGGCTGACAAGGAGGGTGGGCCGCCGGCAGTGGACGAGGTGTTG  50
              ||||||||||||||||||||||||||||||||||||||||||||||||||
Sbjct  19346  TCCTCCTGGCTGACAAGGAGGGTGGGCCGCCGGCAGTGGACGAGGTGTTG  19395


>gb|BC047699.1| Homo sapiens Rho GDP dissociation inhibitor (GDI) gamma, mRNA 
(cDNA clone MGC:51949 IMAGE:5581679), complete cds
Length=983

 Score = 93.5 bits (50),  Expect = 6e-17
 Identities = 50/50 (100%), Gaps = 0/50 (0%)
 Strand=Plus/Plus

Query  1    TCCTCCTGGCTGACAAGGAGGGTGGGCCGCCGGCAGTGGACGAGGTGTTG  50
            ||||||||||||||||||||||||||||||||||||||||||||||||||
Sbjct  150  TCCTCCTGGCTGACAAGGAGGGTGGGCCGCCGGCAGTGGACGAGGTGTTG  199


>gb|AF080237.1|AF080237 Homo sapiens Rho GDP-dissociation inhibitor gamma (ARHGDIG) gene, 
complete cds
Length=4300

 Score = 93.5 bits (50),  Expect = 6e-17
 Identities = 50/50 (100%), Gaps = 0/50 (0%)
 Strand=Plus/Plus

Query  1     TCCTCCTGGCTGACAAGGAGGGTGGGCCGCCGGCAGTGGACGAGGTGTTG  50
             ||||||||||||||||||||||||||||||||||||||||||||||||||
Sbjct  2346  TCCTCCTGGCTGACAAGGAGGGTGGGCCGCCGGCAGTGGACGAGGTGTTG  2395


>gb|U82532.1|HSU82532 Homo sapiens GDI-dissociation inhibitor RhoGDIgamma mRNA, complete 
cds
Length=802

 Score = 93.5 bits (50),  Expect = 6e-17
 Identities = 50/50 (100%), Gaps = 0/50 (0%)
 Strand=Plus/Plus

Query  1    TCCTCCTGGCTGACAAGGAGGGTGGGCCGCCGGCAGTGGACGAGGTGTTG  50
            ||||||||||||||||||||||||||||||||||||||||||||||||||
Sbjct  112  TCCTCCTGGCTGACAAGGAGGGTGGGCCGCCGGCAGTGGACGAGGTGTTG  161


>ref|XM_003809402.1| PREDICTED: Pan paniscus protein disulfide isomerase family A, 
member 2 (PDIA2), mRNA
Length=2623

 Score = 87.9 bits (47),  Expect = 3e-15
 Identities = 49/50 (98%), Gaps = 0/50 (0%)
 Strand=Plus/Plus

Query  1   TCCTCCTGGCTGACAAGGAGGGTGGGCCGCCGGCAGTGGACGAGGTGTTG  50
           ||||||||||||||||||||||||||||||| ||||||||||||||||||
Sbjct  7   TCCTCCTGGCTGACAAGGAGGGTGGGCCGCCAGCAGTGGACGAGGTGTTG  56


>ref|XM_002755740.1| PREDICTED: Callithrix jacchus Rho GDP dissociation inhibitor 
(GDI) gamma (ARHGDIG), mRNA
Length=798

 Score = 87.9 bits (47),  Expect = 3e-15
 Identities = 47/47 (100%), Gaps = 0/47 (0%)
 Strand=Plus/Plus

Query  1    TCCTCCTGGCTGACAAGGAGGGTGGGCCGCCGGCAGTGGACGAGGTG  47
            |||||||||||||||||||||||||||||||||||||||||||||||
Sbjct  194  TCCTCCTGGCTGACAAGGAGGGTGGGCCGCCGGCAGTGGACGAGGTG  240


>ref|XM_001171621.1| PREDICTED: Pan troglodytes Rho GDP dissociation inhibitor (GDI) 
gamma (ARHGDIG), mRNA
Length=905

 Score = 87.9 bits (47),  Expect = 3e-15
 Identities = 49/50 (98%), Gaps = 0/50 (0%)
 Strand=Plus/Plus

Query  1    TCCTCCTGGCTGACAAGGAGGGTGGGCCGCCGGCAGTGGACGAGGTGTTG  50
            ||||||||||||||||||||||||||||||| ||||||||||||||||||
Sbjct  125  TCCTCCTGGCTGACAAGGAGGGTGGGCCGCCAGCAGTGGACGAGGTGTTG  174


>ref|XM_002825909.2| PREDICTED: Pongo abelii uncharacterized LOC100433846 (LOC100433846), 
mRNA
Length=1387

 Score = 82.4 bits (44),  Expect = 1e-13
 Identities = 46/47 (97%), Gaps = 0/47 (0%)
 Strand=Plus/Plus

Query  1    TCCTCCTGGCTGACAAGGAGGGTGGGCCGCCGGCAGTGGACGAGGTG  47
            |||||||||||||||||||||||||||| ||||||||||||||||||
Sbjct  698  TCCTCCTGGCTGACAAGGAGGGTGGGCCACCGGCAGTGGACGAGGTG  744


>gb|AC197388.4| Pongo abelii BAC clone CH276-522N14 from chromosome 16, complete 
sequence
Length=211635

 Score = 82.4 bits (44),  Expect = 1e-13
 Identities = 46/47 (97%), Gaps = 0/47 (0%)
 Strand=Plus/Plus

Query  1      TCCTCCTGGCTGACAAGGAGGGTGGGCCGCCGGCAGTGGACGAGGTG  47
              |||||||||||||||||||||||||||| ||||||||||||||||||
Sbjct  78916  TCCTCCTGGCTGACAAGGAGGGTGGGCCACCGGCAGTGGACGAGGTG  78962


>ref|XM_001118456.2| PREDICTED: Macaca mulatta rho GDP-dissociation inhibitor 3-like 
(LOC722292), mRNA
Length=874

 Score = 76.8 bits (41),  Expect = 7e-12
 Identities = 45/47 (95%), Gaps = 0/47 (0%)
 Strand=Plus/Plus

Query  1    TCCTCCTGGCTGACAAGGAGGGTGGGCCGCCGGCAGTGGACGAGGTG  47
            |||||||||||||||||||||||||||| || |||||||||||||||
Sbjct  185  TCCTCCTGGCTGACAAGGAGGGTGGGCCACCAGCAGTGGACGAGGTG  231


>ref|NM_001168819.1| Papio anubis Rho GDP dissociation inhibitor (GDI) gamma (ARHGDIG), 
mRNA
Length=678

 Score = 76.8 bits (41),  Expect = 7e-12
 Identities = 45/47 (95%), Gaps = 0/47 (0%)
 Strand=Plus/Plus

Query  1    TCCTCCTGGCTGACAAGGAGGGTGGGCCGCCGGCAGTGGACGAGGTG  47
            |||||||||||||||||||||||||||| || |||||||||||||||
Sbjct  74   TCCTCCTGGCTGACAAGGAGGGTGGGCCACCAGCAGTGGACGAGGTG  120


Lambda     K      H
    1.33    0.621     1.12 

Gapped
Lambda     K      H
    1.28    0.460    0.850 

Effective search space used: 874911716973
 
    reads 
 chr9 chr9 119976874 119976798 76m                                    GCTGGGTCCCCAGGCGCGGGAGAGCTTCCGTTCATCCCGGCTGCAAACCCACAATTCCGTCATTGGCGTGCCCATC
chr9 chr9 119976870 119976794 76m                                        GGTCCCCAGGCGCGGGAGAGCTTCCGTTCATCCCGGCTGCAAACCCACAATTCCGTCATTGGCGTGCCCATCCGGG
chr9 chr9 119976866 119976790 76m                                            CCCAGGCGCGGGAGAGCTTCCGTTCATCCCGGCTGCAAACCCACAATTCCGTCATTGGCGTGCCCATCCGGGAGAC
chr9 chr9 119976863 119976787 76m                                               AGGCGCGGGAGAGCTTCCGTTCATCCCGGCTGCAAACCCACAATTCCGTCATTGGCGTGCCCATCCGGGAGACTCC
chr9 chr9 119976860 119976784 76m                                                  CGCGGGAGAGCTTCCGTTCATCCCGGCTGCAAACCCACAATTCCGTCATTGGCGTGCCCATCCGGGAGACTCCCAT
chr9 chr9 119976857 119976781 76m                                                     GGGAGAGCTTCCGTTCATCCCGGCTGCAAACCCACAATTCCGTCATTGGCGTGCCCATCCGGGAGACTCCCATCCT
chr9 chr9 119976854 119976778 76m                                                        AGAGCTTCCGTTCATCCCGGCTGCAAACCCACAATTCCGTCATTGGCGTGCCCATCCGGGAGACTCCCATCCTGGA
chr9 chr9 119976851 119976775 76m                                                           GCTTCCGTTCATCCCGGCTGCAAACCCACAATTCCGTCATTGGCGTGCCCATCCGGGAGACTCCCATCCTGGATGA
chr9 chr9 119976848 119976772 76m                                                              TCCGTTCATCCCGGCTGCAAACCCATAATTCCGTCATTGGCGTGCCCATCCGGGAGACTCCCATCCTGGATGACTA
chr9 chr9 119976845 119976769 76m                                                                 GTTCATCCCGGCTGCAAACCCACAATTCCGTCATTGGCGTGCCCATCCGGGAGACTCCCATCCTGGATGACTATGA
chr9 chr9 119976840 119976764 76m                                                                      TCCCGGCTGCAAACCCACAATTCCGTCATTGGCGTGCCCATCCGGGAGACTCCCATCCTGGATGACTATGACTGCG
chr9 chr9 119976837 119976761 76m                                                                         CGGCTGCAAACCCACAATTCCGTCATTGGCGTGCCCATCCGGGAGACCCCCATCCTGGATGACTATGACTGTGAGG
chr9 chr9 119976833 119976757 76m                                                                             TGCAAACCCACAATTCCGTCATTGGCGTGCCCATCCGGGAGACTCCCATCCTGGATGACTATGACTGTGAGGAGGG
chr9 chr9 119976829 119976753 76m                                                                                 AACCCACAATTCCGTCATTGGCGTGCCCATCCGGGAGACTCCCATCCTGGATGACTATGACTGTGAGGAGGATGAG
chr9 chr9 119976826 119976750 76m                                                                                    CCACAATTCCGTCATTGGCGTGCCCATCCGGGAGACTCCCATCCTGGATGACTATGACTGTGAGGAGGATGAGGAG
chr9 chr9 119976823 119976747 76m                                                                                       CAATTCCGTCATTGGCGTGCCCATCCGGGAGACTCCCATCCTGGATGACTATGACTGTGAGGAGGATGAGGAGCCA
chr9 chr9 119976820 119976744 76m                                                                                          TTCCGTCATTGGCGTGCCCATCCGGGAGACTCCCATCCTGGATGACTATGACTGTGAGGAGGATGAGGAGCCACCT
chr9 chr9 119976817 119976741 76m                                                                                             CGTCATTGGCGTGCCCATCCGGGAGACTCCCATCCTGGATGACTATGACTGTGAGGAGGATGAGGAGCCACCTAGG
chr9 chr9 119976814 119976738 76m                                                                                                CATTGGCGTGCCCATCCGGGAGACTCCCATCCTGGATGACTATGACTGTGAGGAGGATGAGGAGCCACCTAGGCGG
chr9 chr9 119976811 119976735 76m                                                                                                   TGGCGTGCCCATCCGGGAGACTCCCATCCTGGATGACTATGACTGTGAGGAGGATGAGGAGCCACCTAGGCGGGCC
chr9 chr9 119976808 119976732 76m                                                                                                      CGTGCCCATCCGGGAGACTCCCATCCTGGATGACTATGACTGTGAGGAGGATGAGGAGCCACCTAGGCGGGCCAAC
chr9 chr9 119976805 119976729 76m                                                                                                         GCCCATCCGGGAGACTCCCATCCTGGATGACTATGACTGTGAGGAGGATGAGGAGCCACCTAGGCGGGCCAACCAT
chr9 chr9 119976802 119976726 76m                                                                                                            CATCCGGGAGACTCCCATCCTGGATGACTATGACTGTGAGGAGGATGAGGAGCCACCTAGGCGGGCCAACCATGTC
chr9 chr9 119976799 119976723 76m                                                                                                               CCGGGAGACTCCCATCCTGGATGACTATGACTGTGAGGAGGATCAGGAGCCACCTAGGCGGGCCAACCATGCCTCC
chr9 chr9 119976796 119976720 76m                                                                                                                  GGAGACTCCCATCCTGGATGACTATGACTGTGAGGAGGATGAGGAGCCACCTAGGCGGGCCAACCATGTCTCCCGC
chr9 chr9 119976793 119976717 76m                                                                                                                     GACTCCCATCCTGGATGACTATGACTGTGAGGAGGATGAGGAGCCACCTAGGCGGGCCAACCATGTCTCCCGCGAG
chr9 chr9 119976789 119976713 76m                                                                                                                         CCCATCCTGGATGACTATGACTGTGAGGAGGATGAGGAGCCACCTAGGCGGGCCAACCATGTCTCCCGCGAGGACG
chr9 chr9 119976785 119976709 76m                                                                                                                             TCCTGGATGACTATGACTGTGAGGAGGATGAGGAGCCACCTAGGCGGGCCAACCATGTCTCCCGCGAGGACGAGTT
chr9 chr9 119976782 119976706 76m                                                                                                                                TGGATGACTATGACTGTGAGGAGGATGAGGAGCCACCTAGGCGGGCCAACCATGTCTCCCGCGAGGACGAGTTTGG
chr9 chr9 119976779 119976703 76m                                                                                                                                   ATGACTATGACTGTGAGGAGGATGAGGAGCCACCTAGGCGGGCCAACCATGTCTCCCGCGAGGACGAGTTTGGCAG
chr9 chr9 119976776 119976700 76m                                                                                                                                      ACTATGACTGTGAGGAGGATGAGGAGCCACCTAGGCGGGCCAACCATGTCTCCCGCGAGGACGAGTTTGGCAGCCG
chr9 chr9 119976773 119976697 76m                                                                                                                                         ATGACTGTGAGGAGGATGAGGAGCCACCTAGGAGGGCCAACCATGTCTCCCGCGAGGACGAGTTTGGCAGCCAGGT
chr9 chr9 119976770 119976694 76m                                                                                                                                            ACTGTGAGGAGGATGAGGAGCCACCTAGGCGGGCCAACCATGTCTCCCGCGAGGACGAGTTTGGCAGCCAGGTGAC
chr9 chr9 119976767 119976691 76m                                                                                                                                               GTGAGGAGGATGAGGAGCCACCTAGGCGGGCCAACCATGTCTCCCGCGAGGACGAGTTTGGCAGCCAGGTGACCCA
chr9 chr9 119976764 119976688 76m                                                                                                                                                  AGGAGGATGAGGAGCCACCTAGGCGGGCCAACCATGTCTCCCGCGAGGACGAGTTTGGCAGCCAGGTGACCCACAC
chr9 chr9 119976760 119976684 76m                                                                                                                                                      GGATGAGGAGCCACCTAGGCGGGCCAACCATGTCTCCCGCGAGGACGAGTTTGGCAGCCAGGTGACCCACACTCTG
chr9 chr9 119976757 119976681 76m                                                                                                                                                         TGAGGAGCCACCTAGGCGGGCCAACCATGTCTCCCGCGAGGACGAGTTTGGCAGCCAGGTGACCCACACTCTGGAC
chr9 chr9 119976753 119976677 76m                                                                                                                                                             GAGCCACCTAGGCGGGCCAACCATGTCTCCCGCGAGGACGAGCTTGGCAGCCAGGAGACCCACACTCTGGACAGTC
chr9 chr9 119976744 119976668 76m                                                                                                                                                                      AGGCGGGCCAACCATGTCTCCCGCGAGGACGAGTTTGGCAGCCAGGTGACCCACACTCTGGACAGGCTGGGACATC
chr9 chr9 119976741 119976665 76m                                                                                                                                                                         CGGGCCAACCATGTCTCCCGCGAGGACGAGTTTGGCAGCCAGGTGACCCACACTCTGGACAGTCTGGGACATCCAG
chr9 chr9 119976738 119976662 76m                                                                                                                                                                            GCCAACCATGTCTCCCGCGAGGACGAGTTTGGCAGCCAGGTGACCCACACTCTGGACAGTCTGGGACATCCAGGGG
chr9 chr9 119976735 119976659 76m                                                                                                                                                                               AACCATGTCTCCCGCGAGGACGAGTTTGGCAGCCAGGTGACCCACACTCTGGACAGTCTGGGACATCCAGGGGAAG
chr9 chr9 119976732 119976656 76m                                                                                                                                                                                  CATGTCTCCCGCGAGGACGAGTTTGGCAGCCAGGTGACCCACACTCTGGACAGTCTGGGACATCCAGGGGAAGAGA
chr9 chr9 119976729 119976653 76m                                                                                                                                                                                     GTCTCCCGCGAGGACGAGTTTGGCAGCCAGGTGACCCACACTCTGGACAGTCTGGGACATCCAGGGGAAGAGAAGG
chr9 chr9 119976725 119976649 76m                                                                                                                                                                                         CCCGCGAGGACGAGTTTGGCAGCCAGGTGACCCACACTCTGGACAGTCTGGGACATCCAGGGGAAGAGAAGGTGGA
chr9 chr9 119976722 119976646 76m                                                                                                                                                                                            GCGAGGACGAGTTTGGCAGCCAGGTGACCCACACTCTGGACAGTCTGGGACATCCAGGGGAAGAGAAGGTGGACTT
chr9 chr9 119976719 119976643 76m                                                                                                                                                                                               AGGACGAGTTTGGCAGCCAGGTGACCCACACTCTGGACAGTCTGGGACATCCAGGGGAAGAGAAGGTGGACTTTGA
chr9 chr9 119976716 119976640 76m                                                                                                                                                                                                  ACGAGTTTGGCAGCCAGGTGACCCACACTCTGGACAGTCTGGGACATCCAGGGGAAGAGAAGGTGGACTTTGAGAA
chr9 chr9 119976713 119976637 76m                                                                                                                                                                                                     AGTTTGGCAGCCAGGTGACCCACACTCTGGACAGTCTGGGACATCCAGGGGAAGAGAAGGTGGACTTTGAGAAGAA
chr9 chr9 119976710 119976634 76m                                                                                                                                                                                                        TTGGCAGCCAGGTGACCCACACTCTGGACAGTCTGGGACATCCAGGGGAAGAGAAGGTGGACTTTGAGAAGAAAGC
chr9 chr16 119976708 331748 73m331746F3M                                                                                                                                                                                                   TGCAGCCAGGTGACCCACACTCTGGACAGTCTGGGACATCCAGGGGAAGAGAAGGTGGACTTTGAGAAGAAAG TCC
chr9 chr16 119976706 331750 71m331746F5M                                                                                                                                                                                                     CAGCCAGGTGACCCACACTCTGGACAGTCTGGGACATCCAGGGGAAGAGAAGGTGGACTTTGAGAAGAAAG TCCTC
chr9 chr16 119976705 331751 70m331746F6M                                                                                                                                                                                                      AGCCAGGTGACCCACACTCTGGACAGTCTGGGACATCCAGGGGAAGAGAAGGTGGACTTTGAGAAGAAAG TCCTCC
chr9 chr16 119976699 331757 64m331746F12M                                                                                                                                                                                                           CAGCCAGGAGGACTTTCTTCTCAAAGTCCACCTTCTCTTCCCCTGGATGTCCCAGACTGTCCAG AGTGTGGGTCAC
chr9 chr16 119976697 331759 62m331746F14M                                                                                                                                                                                                             GTCAGCCAGGAGGACTTTCTTCTCAAAGTCCACCTTCTCTTCCCCTGGATGTCCCAGACTGT CCAGAGTGTGGGTC
chr9 chr16 119976697 331759 62m331746F14M                                                                                                                                                                                                             GTCAGCCAGGAGGACTTTCTTCTCAAAGTCCACCTTCTCTTCCCCTGGATGTCCCAGACTGT CCAGAGTGTGGGTC
chr9 chr16 119976697 331759 62m331746F14M                                                                                                                                                                                                             GTCAGCCAGGAGGACTTTCTTCTCAAAGTCCACCTTCTCTTCCCCTGGATGTCCCAGACTGT CCAGAGTGTGGGTC
chr9 chr16 119976697 331759 62m331746F14M                                                                                                                                                                                                             GTCAGCCAGGAGGACTTTCTTCTCAAAGTCCACCTTCTCTTCCCCTGGATGTCCCAGACTGT CCAGAGTGTGGGTC
chr9 chr16 119976695 331761 60m331746F16M                                                                                                                                                                                                               CCCACACTCTGGACAGTCTGGGACATCCAGGGGAAGAGAAGGTGGACTTTGAGAAGAAAG TCCTCCTGGCTGACAA
chr9 chr16 119976695 331761 60m331746F16M                                                                                                                                                                                                               CCCACACTCTGGACAGTCTGGGACATCCAGGGGAAGAGAAGGTGGACCTTGAGAAGAAAG TCCTCCTGGCTGACAG
chr9 chr16 119976694 331762 59m331746F17M                                                                                                                                                                                                                CCACACTCTGGACAGTCTGGGACATCCAGGGGAAGAGAAGGTGGACTTTGAGAAGAAAG TCCTCCTGGCTGACAAG
chr9 chr16 119976693 331763 58m331746F18M                                                                                                                                                                                                                 CCTTGTCAGCCAGGAGGACTTTCTTCTCAAAGTCCACCTTCTCTTCCCCTGGATGTCC CAGACTGTCCAGAGTGTG
chr9 chr16 119976687 331769 52m331746F24M                                                                                                                                                                                                                       CTGGACAGTCTGGGACATCCAGGGGAAGAGAAGGTGGACTTTGAGAAGAAAG TCCTCCTGGCTGACAAGGAGGGTG
chr9 chr16 119976684 331772 49m331746F27M                                                                                                                                                                                                                          GACAGTCTGGGACATCCAGGGGAAGAGAAGGTTGACTTTGAGAAGAAAG TCCTCCTGGCTGACAAGGAGGGTGGGC
chr9 chr16 119976684 331772 49m331746F27M                                                                                                                                                                                                                          GACAGTCTGGGACATCCAGGGGAAGAGAAGGTGGACTTTGAGAAGAAAG TCCTCCTGGCTGACAAGGAGGGTGGGT
chr9 chr16 119976682 331774 47m331746F29M                                                                                                                                                                                                                            CAGTCTGGGACATCCAGGGGAAGAGAAGGTGGACTTTGAGAAGAAAG TCCTCCTGGCTGACAAGGAGGGTGGGCCG
chr9 chr16 119976682 331774 47m331746F29M                                                                                                                                                                                                                            CAGTCTGGGACATCCAGGGGAAGAGAAGGTGGACTTTGAGAAGAAAG TCCTCCTGGCTGACAAGGAGGGTGGGCCG
chr9 chr16 119976679 331777 44m331746F32M                                                                                                                                                                                                                               TCTGGGACATCCAGGGGAAGAGAAGGTGGACTTTGAGAAGAAAG TCCTCCTGGCTGACAAGGAGGGTGGGCCGCCG
chr9 chr16 119976679 331777 44m331746F32M                                                                                                                                                                                                                               TCTGGGACATCCAGGGGAAGAGAAGGTGGACTTTGAGAAGAAAG TCCTCCTGGCTGACAAGGAGGGTGGGCCGCCG
chr9 chr16 119976679 331777 44m331746F32M                                                                                                                                                                                                                               TCTGGGACATCCAGGGGAAGAGAAGGTGGACTTTGAGAAGAAAG TCCTCCTGGCTGACAAGGAGGGTGGGCCGCCG
chr9 chr16 119976678 331778 43m331746F33M                                                                                                                                                                                                                                CTGGGACATCCAGGGGAAGAGAAGGTGGACTTTGAGAAGCAAG TCCTCCTGGCTGACAAGGAGGGTGGGCCGCCGG
chr9 chr16 119976676 331780 41m331746F35M                                                                                                                                                                                                                                  GGGACATCCAGGGGAAGAGAAGGTGGACTTTGAGAAGAAAG TCCTCCTGGCTGACAAGGAGGGTGGGCCGCCGGCA
chr9 chr16 119976664 331792 29m331746F47M                                                                                                                                                                                                                                              GGAAGAGAAGGTGGACTTTGAGAAGAAAG TCCTCCTGGCTGACAAGGAGGGTGGGCCGCCGGCAGTGGACGAGGTG
chr9 chr16 119976660 331796 25m331746F51M                                                                                                                                                                                                                                                  GAGAAGGTGGACTTTGAGAAGAAAG TCCTCCTGGCTGACAAGGAGGGTGGGCCGCCGGCAGTGGACGAGGTGTTGG
chr9 chr16 119976654 331802 19m331746F57M                                                                                                                                                                                                                                                        GTGGACTTTGAGAAGAAAG TCCTCCTGGCTGACAAGGAGGGTGGGCCGCCGGCAGTGGACGAGGTGTTGGATGAGG
chr9 chr16 119976654 331802 19m331746F57M                                                                                                                                                                                                                                                        CCTCATCCAACACCTCGTC CACTGCCGGCGGCCCACCCTCCTTGTCAGCCAGGAGGACTTTCTTCTCAAAGTCCAC
chr9 chr16 119976654 331802 19m331746F57M                                                                                                                                                                                                                                                        CCTCATCCAACACCTCGTC CACTGCCGGCGGCCCACCCTCCTTGTCAGCCAGGAGGACTTTCTTCTCAAAGTCCAC
chr9 chr16 119976653 331803 18m331746F58M                                                                                                                                                                                                                                                         TGGACTTTGAGAAGAAAG TCCTCCTGGCTGACAAGGAGGGTGGGCCGCCGGCAGTGGACGAGGTGTTGGACGAGGC
chr9 chr16 119976653 331803 18m331746F58M                                                                                                                                                                                                                                                         TGGACTTTGAGAAGAAAG TCCTCCTGGCTGACAAGGAGGGTGGGCCGCCGGCAGTGGACGAGGTGTTGGATGAGGA
chr9 chr16 119976653 331803 18m331746F58M                                                                                                                                                                                                                                                         TGAACTTTGAGAAGAAAG TCCTCCTGGCTGACAAGGAGGGTGGGCCGCCGGCAGTGGACGAGGTGTTGGATGAGGC
chr9 chr16 119976652 331804 17m331746F59M                                                                                                                                                                                                                                                          AGCCTCATCCAACACCT CGTCCACTGCCGGCGGCCCACCCTCCTTGTCAGCCAGGAGGACTTTCTTCTCAAAGTCC
chr9 chr16 119976649 331807 14m331746F62M                                                                                                                                                                                                                                                             CACAGCCTCATCCA ACACCTCGTCCACTGCCGGCGGCCCACCCTCCTTGTCAGCCAGGAGGACTTTCTTCTCAAAG
chr9 chr16 119976649 331807 14m331746F62M                                                                                                                                                                                                                                                             CTTTGAGAAGAAAG TCCTCCTGGCTGACAAGGAGGGTGGGCCGCCGGCAGTGGACGAGGTGTTGGATGAGGCTGTG
chr9 chr16 119976646 331810 11m331746F65M                                                                                                                                                                                                                                                                TGAGAAGAAAG TCCTCCTGGCTGACAAGGAGGGTGGGCCGCCGGCAGTGGACGAGGTGTTGGATGAGGCTGTGCCC
chr9 chr16 119976644 331812 9m331746F67M                                                                                                                                                                                                                                                                   AGAAGAAAG TCCTCCTGGCTGACAAGGAGGGTGGGCCGCCGGCAGTGGACGAGGTGTTGGATGAGGCTGTGCCCGC
chr9 chr16 119976643 331813 8m331746F68M                                                                                                                                                                                                                                                                    CTCGGGCA CAGCCTCATCCAACACCTCGTCCACTGCCGGCGGCCCACCCTCCTTGTCAGCCAGGAGGACTTTCTTC
chr16 chr16 331743 331819 76M                                                                                                                                                                                                                                                                                      AGTCCTCCTGGCTGACAAGGAGGGTGGGCCGCCGGCAGTGGACGAGGTGTTGGATGAGGCTGTGCCCGAGTACCGG
chr16 chr16 331746 331822 76M                                                                                                                                                                                                                                                                                         CCTCCTGGCTGACAAGGAGGGTGGGCCGCCGGCAGTGGACGAGGTGTTGGATGAGGCTGTGCCCGAGTACCGGGCG
chr16 chr16 331751 331827 76M                                                                                                                                                                                                                                                                                              TGGCTGACAAGGAGGGTGCGCCGCCGGCAGTGGACGAGGTGTTGGATGAGGCTGTGCCCGAGTACCGGGCGCCGGG
chr16 chr16 331755 331831 76M                                                                                                                                                                                                                                                                                                  TGACAAGGAGGGTGGGCCGCCGGCAGTGGACGAGGTGTTGGATGAGGCTGTGCCCGAGTACCGGGCGCCGGGGAGG
chr16 chr16 331758 331834 76M                                                                                                                                                                                                                                                                                                     CAAGGAGGGTGGGCCGCCGGCAGTGGACGAGGTGTTGGATGAGGCTGTGCCCGAGTACCGGGCGCCGGGGAGGAAG
chr16 chr16 331761 331837 76M                                                                                                                                                                                                                                                                                                        GGAGGGTGGGCCGCCGGCAGTGGACGAGGTGTTGGATGAGGCTGTGCCCGAGTACCGGGCGCCGGGGAGGAAGAGC
chr16 chr16 331764 331840 76M                                                                                                                                                                                                                                                                                                           GGGTGGGCCGCCGGCAGTGGACGAGGTGTTGGATGAGGCTGTGCCCGAGTACCGGGCGCCGGGGAGGAAGAGCCTC
chr16 chr16 331768 331844 76M                                                                                                                                                                                                                                                                                                               GGGCCGCCGGCAGTGGACGAGGTGTTGGATGAGGCTGTGCCCGAGTACCGGGCGCCGGGGAGGAAGAGCCTCTTGG
chr16 chr16 331772 331848 76M                                                                                                                                                                                                                                                                                                                   CGCCGGCAGTGGACGAGGTGTTGGATGAGGCTGTGCCCGAGTACCGGGCGCCGGGGAGGAAGAGCCTCTTGGAGAT
chr16 chr16 331775 331851 76M                                                                                                                                                                                                                                                                                                                      CGGCAGTGGACGAGGTGTTGGATGAGGCTGTGCCCGAGTACCGGGCGCCGGGGAGGAAGAGCCTCTTGGAGATCCG
chr16 chr16 331778 331854 76M                                                                                                                                                                                                                                                                                                                         CAGTGGACGAGGTGTTGGATGAGGCTGTGCCCGAGTACCGGGCGCCGGGGAGGAAGAGCCTCTTGGAGATCCGGCA
chr16 chr16 331784 331860 76M                                                                                                                                                                                                                                                                                                                               ACGAGGTGTTGGATGAGGCTGTGCCCGAGTACCGGGCGCCGGGGAGGAAGAGCCTCTTGGAGATCCGGCAGCTGGA
chr16 chr16 331787 331863 76M                                                                                                                                                                                                                                                                                                                                  AGGCGTTGGATGAGGCTGTGCCCGAGTACCGGGCGCCGGGGAGGAAGAGCCTCTTGGAGATCCGGCAGCTGGACCC
chr16 chr16 331790 331866 76M                                                                                                                                                                                                                                                                                                                                     TGTTGGATGAGGCTGTGCCCGAGTACCGGGCGCCGGGGAGGAAGAGCCTCTTGGAGATCCGGCAGCTGGACCCGGA
chr16 chr16 331794 331870 76M                                                                                                                                                                                                                                                                                                                                         GGATGAGGCTGTGCCCGAGTACCGGGCGCCGGGGAGGAAGAGCCTCTTGGAGATCCGGCAGCTGGACCCGGACGAC
chr16 chr16 331797 331873 76M                                                                                                                                                                                                                                                                                                                                            TGAGGCTGTGCCCGAGTACCGGGCGCCGGGGAGGAAGAGCCTCTTGGAGATCCGGCAGCTGGACCCGGACGACAGG
chr16 chr16 331800 331876 76M                                                                                                                                                                                                                                                                                                                                               GGCTGGGCCCGAGTACCGGGCGCCGGGGAGGAAGAGCCTGTTGGAGATCCGGCAGCTGGACCCGGACGACAGGAGC
chr16 chr16 331803 331879 76M                                                                                                                                                                                                                                                                                                                                                  TGTGCCCGAGTACCGGGCGCCGGGGAGGAAGAGCCTCTTGGAGATCCGGCAGCTGGACCCGGACGACAGGAGCCTG
chr16 chr16 331807 331883 76M                                                                                                                                                                                                                                                                                                                                                      ACCGAGTACCGGGCGCCGGGGAGGAAGAGCCTCTTGGAGATCCGGCAGCTGGACCCGGACGACAGGAGCCTGGCCA
chr16 chr16 331814 331890 76M                                                                                                                                                                                                                                                                                                                                                             ACCGGGCGCCGGGGAGGAAGAGCCTCTTGGAGATCCGGCAGCTGGACCCGGACGACAGGAGCCTGGCCAAGTACAA
chr16 chr16 331817 331893 76M                                                                                                                                                                                                                                                                                                                                                                GGGCGCCGGGGAGGAAGAGCCTCTTGGAGATCCGGCAGCTGGACCCGGACGACAGGAGCCTGGCCAAGTACAAGCG
chr16 chr16 331820 331896 76M                                                                                                                                                                                                                                                                                                                                                                   CGCCGGGGAGGAAGAGCCTCTTGGAGATCCGGCAGCTGGACCCGGACGACAGGAGCCTGGCCAAGTACAAGCGGGT
chr16 chr16 331823 331899 76M                                                                                                                                                                                                                                                                                                                                                                      CGGGGAGGAAGAGCCTCTTGGAGATCCGGCAGCTGGACCCGGACGACAGGAGCCTGGCCAAGTACAAGCGGGTGCT
chr16 chr16 331826 331902 76M                                                                                                                                                                                                                                                                                                                                                                         GGAGGAAGAGCCTCTTGGAGATCCGGCAGCTGGACCCGGACGACAGGAGCCTGGCCAAGTACAAGCGGGTGCTGCT
chr16 chr16 331829 331905 76M                                                                                                                                                                                                                                                                                                                                                                            GGAAGAGCCTCTTGGAGATCCGGCAGCTGGACCCGGACGACAGGAGCCTGGCCAAGTACAAGCGGGTGCTGCTGGG
chr16 chr16 331832 331908 76M                                                                                                                                                                                                                                                                                                                                                                               AGAGCCTCTTGGAGATCCGGCAGCTGGACCCGGACGACAGGAGCCTGGCCAAGTACAAGCGGGTGCTGCTGGGGCC
chr16 chr16 331835 331911 76M                                                                                                                                                                                                                                                                                                                                                                                  GCCTCTTGGAGATCCGGCAGCTGGACCCGGACGACAGGAGCCTGGCCAAGTACAAGCGGGTGCTGCTGGGGCCCCT
chr16 chr16 331838 331914 76M                                                                                                                                                                                                                                                                                                                                                                                     TCTTGGAGATCCGGCAGCTGGACCCGGACGACAGGAGCCTGGCCAAGTACAAGCGGGTGCTGCTGGGGCCCCTGCC
chr16 chr16 331841 331917 76M                                                                                                                                                                                                                                                                                                                                                                                        TGGAGATCCGGCAGCTGGACCCGGACGACAGGAGCCTGGCCAAGTACAAGCGGGTGCTGCTGGGGCCCCTGCCACC
chr16 chr16 331845 331921 76M                                                                                                                                                                                                                                                                                                                                                                                            GATCCGGCAGCTGGACCCGGACGACAGGAGCCTGGCCAAGTACAAGCGGGTGCTGCTGGGGCCCCTGCCACCGGCC
chr16 chr16 331848 331924 76M                                                                                                                                                                                                                                                                                                                                                                                               CCGGCAGCTGGACCCGGACGACAGGAGCCTGGCCAAGTACAAGCGGGTGCTGCTGGGGCCCCTGCCACCGGCCGTG
chr16 chr16 331851 332026 74M99N2M                                                                                                                                                                                                                                                                                                                                                                                             GCAGCTGGACCCGGACGACAGGAGCCTGGCCAAGTACAAGCGGGTGCTGCTGGGGCCCCTGCCACCGGCCGTGG|||||||||||||||||||||||||||||||||||||||||||||||||||||||||||||||||||||||||||||||||||||||||||||||||||AC
chr16 chr16 331854 332029 71M99N5M                                                                                                                                                                                                                                                                                                                                                                                                GCTGGACCCGGACGACAGGAGCCTGGCCAAGTACAAGCGGGTGCTGCTGGGGCCCCTGCCACCGGCCGTGG|||||||||||||||||||||||||||||||||||||||||||||||||||||||||||||||||||||||||||||||||||||||||||||||||||ACCCA
chr16 chr16 331860 332035 65M99N11M                                                                                                                                                                                                                                                                                                                                                                                                     CCCGGACGACAGGAGCCTGGCCAAGTACAAGCGGGTGCTGCTGGGGCCCCTGCCACCGGCCGTGG|||||||||||||||||||||||||||||||||||||||||||||||||||||||||||||||||||||||||||||||||||||||||||||||||||ACCCAAGCCTG
chr16 chr16 331863 332038 62M99N14M                                                                                                                                                                                                                                                                                                                                                                                                        GGACGACAGGAGCCTGGCCAAGTACAAGCGGGTGCTGCTGGGGCCCCTGCCACCGGCCGTGG|||||||||||||||||||||||||||||||||||||||||||||||||||||||||||||||||||||||||||||||||||||||||||||||||||ACCCAAGCCTGCCC
chr16 chr16 331866 332041 59M99N17M                                                                                                                                                                                                                                                                                                                                                                                                           CGACAGGAGCCTGGCCAAGTACAAGCGGGTGCTGCTGGGGCCCCTGCCACCGGCCGTGG|||||||||||||||||||||||||||||||||||||||||||||||||||||||||||||||||||||||||||||||||||||||||||||||||||ACCCCAGCCTGCCCAAT
chr16 chr16 331869 332044 56M99N20M                                                                                                                                                                                                                                                                                                                                                                                                              CCGGAGCCTGGCCAAGTACAAGCGGGTGCTGCTGGGGCCCCTGCCACCGGCCGTGG|||||||||||||||||||||||||||||||||||||||||||||||||||||||||||||||||||||||||||||||||||||||||||||||||||ACCCAAGCCTGCCCAATGT
chr16 chr16 331872 332047 53M99N23M                                                                                                                                                                                                                                                                                                                                                                                                                 GAGCCTGGCCAAGTACAAGCGGGTGCTGCTGGGGCCCCTGCCACCGGCCGTGG|||||||||||||||||||||||||||||||||||||||||||||||||||||||||||||||||||||||||||||||||||||||||||||||||||ACCCAAGCCTGCCCAATGT
chr16 chr16 331876 332051 49M99N27M                                                                                                                                                                                                                                                                                                                                                                                                                     CTGGCCAAGTACAAGCGGGTGCTGCTGGGGCCCCTGCCACCGGCCGTGG|||||||||||||||||||||||||||||||||||||||||||||||||||||||||||||||||||||||||||||||||||||||||||||||||||ACCCAAGCCTGCCCAATGT
chr16 chr16 331879 332054 46M99N30M                                                                                                                                                                                                                                                                                                                                                                                                                        GCCAAGTACAAGCGGGTGCTGCTGGGGCCCCTGCCACCGGCCGTGG|||||||||||||||||||||||||||||||||||||||||||||||||||||||||||||||||||||||||||||||||||||||||||||||||||ACCCAAGCCTGCCCAATGT
chr16 chr16 331884 332059 41M99N35M                                                                                                                                                                                                                                                                                                                                                                                                                             GTACAAGCGGGTGCTGCTGGGGCCCCTGCCACCGGCCGTGG|||||||||||||||||||||||||||||||||||||||||||||||||||||||||||||||||||||||||||||||||||||||||||||||||||ACCCAAGCCTGCCCAATGT
chr16 chr16 331887 332062 38M99N38M                                                                                                                                                                                                                                                                                                                                                                                                                                CAAGCGGGTGCTGCTGGGGCCCCTGCCACCGGCCGTGG|||||||||||||||||||||||||||||||||||||||||||||||||||||||||||||||||||||||||||||||||||||||||||||||||||ACCCAAGCCTGCCCAATGT
chr16 chr16 331890 332065 35M99N41M                                                                                                                                                                                                                                                                                                                                                                                                                                   GCGGGTGCTGCTGGGGCCCCTGCCACCGGCCGTGG|||||||||||||||||||||||||||||||||||||||||||||||||||||||||||||||||||||||||||||||||||||||||||||||||||ACCCAAGCCTGCCCAATGT
chr16 chr16 331893 332068 32M99N44M                                                                                                                                                                                                                                                                                                                                                                                                                                      GGTGCTGCTGGGGCCCCTGCCACCGGCCGTGG|||||||||||||||||||||||||||||||||||||||||||||||||||||||||||||||||||||||||||||||||||||||||||||||||||ACCCAAGCCTGCCCAATGT
chr16 chr16 331896 332071 29M99N47M                                                                                                                                                                                                                                                                                                                                                                                                                                         GCTGCTGGGGCCCCTGCCACCGGCCGTGG|||||||||||||||||||||||||||||||||||||||||||||||||||||||||||||||||||||||||||||||||||||||||||||||||||ACCCAAGCCTGCCCAATGT
chr16 chr16 331899 332074 26M99N50M                                                                                                                                                                                                                                                                                                                                                                                                                                            GCTGGGGCCCCTGCCACCGGCCGTGG|||||||||||||||||||||||||||||||||||||||||||||||||||||||||||||||||||||||||||||||||||||||||||||||||||ACCCAAGCCTGCCCAATGT
chr16 chr16 331902 332077 23M99N53M                                                                                                                                                                                                                                                                                                                                                                                                                                               GGGGCCCCTGCCACCGGCCGTGG|||||||||||||||||||||||||||||||||||||||||||||||||||||||||||||||||||||||||||||||||||||||||||||||||||ACCCAAGCCTGCCCAATGT
chr16 chr16 331907 332082 18M99N58M                                                                                                                                                                                                                                                                                                                                                                                                                                                    CCCTGCCACCGGCCGTGG|||||||||||||||||||||||||||||||||||||||||||||||||||||||||||||||||||||||||||||||||||||||||||||||||||ACCCAAGCCTGCCCAATGT
chr16 chr16 331910 332085 15M99N61M                                                                                                                                                                                                                                                                                                                                                                                                                                                       GGCCACCGGCCGTGG|||||||||||||||||||||||||||||||||||||||||||||||||||||||||||||||||||||||||||||||||||||||||||||||||||ACCCAAGCCTGCCCAATGT 
    24 pairs 
 1:6
13:44
31:45
114:70
216:30
-1:281
-2:302
45:279
46:285
46:285
46:289
46:297
28:323
6:346
28:340
86:287
80:297
84:298
60:345
46:579
78:547
87:546
146:607
95:779 
     TCGA-02-2483-01A chr9-chr16 119976636 332024 rf 
 
39 24 38 0 52 56
GGACAGTCTGGGACATCCAGGGGAAGAGAAGGTGGACTTTGAGAAGAAAG ACCCAAGCCTGCCCAATGTGCAGGTGACCAGGCTGACACTCCTGTCGGAA
55688999999999999999999999999999999999999999999999 99999999999999999999999999999999999999999999998554
ASTN2 exon20(119976635-119977020) ARHGDIG exon3(332023-332107)
 
 blast search - human genome  
 left 
 >ref|NW_001839237.2| Homo sapiens chromosome 9 genomic contig, alternate assembly 
HuRef SCAF_1103279187522, whole genome shotgun sequence
 gb|DS486153.1| Homo sapiens SCAF_1103279187522 genomic scaffold, whole genome 
shotgun sequence
Length=4804840

 Score = 93.5 bits (50),  Expect = 5e-17
 Identities = 50/50 (100%), Gaps = 0/50 (0%)
 Strand=Plus/Plus

Query  1        GGACAGTCTGGGACATCCAGGGGAAGAGAAGGTGGACTTTGAGAAGAAAG  50
                ||||||||||||||||||||||||||||||||||||||||||||||||||
Sbjct  4589607  GGACAGTCTGGGACATCCAGGGGAAGAGAAGGTGGACTTTGAGAAGAAAG  4589656


>gb|CH471090.1| Homo sapiens 211000035837865 genomic scaffold, whole genome shotgun 
sequence
Length=25070985

 Score = 93.5 bits (50),  Expect = 5e-17
 Identities = 50/50 (100%), Gaps = 0/50 (0%)
 Strand=Plus/Minus

Query  1        GGACAGTCTGGGACATCCAGGGGAAGAGAAGGTGGACTTTGAGAAGAAAG  50
                ||||||||||||||||||||||||||||||||||||||||||||||||||
Sbjct  4112220  GGACAGTCTGGGACATCCAGGGGAAGAGAAGGTGGACTTTGAGAAGAAAG  4112171


>ref|NT_008470.19| Homo sapiens chromosome 9 genomic contig, GRCh37.p9 Primary Assembly
 gb|GL000089.1| Homo sapiens chromosome 9 genomic contig, GRCh37 reference primary 
assembly
Length=62237592

 Score = 93.5 bits (50),  Expect = 5e-17
 Identities = 50/50 (100%), Gaps = 0/50 (0%)
 Strand=Plus/Minus

Query  1         GGACAGTCTGGGACATCCAGGGGAAGAGAAGGTGGACTTTGAGAAGAAAG  50
                 ||||||||||||||||||||||||||||||||||||||||||||||||||
Sbjct  49141218  GGACAGTCTGGGACATCCAGGGGAAGAGAAGGTGGACTTTGAGAAGAAAG  49141169


>ref|NC_000009.11| Homo sapiens chromosome 9, GRCh37.p9 Primary Assembly
 gb|CM000671.1| Homo sapiens chromosome 9, GRCh37 primary reference assembly
Length=141213431

 Score = 93.5 bits (50),  Expect = 5e-17
 Identities = 50/50 (100%), Gaps = 0/50 (0%)
 Strand=Plus/Minus

Query  1          GGACAGTCTGGGACATCCAGGGGAAGAGAAGGTGGACTTTGAGAAGAAAG  50
                  ||||||||||||||||||||||||||||||||||||||||||||||||||
Sbjct  119976686  GGACAGTCTGGGACATCCAGGGGAAGAGAAGGTGGACTTTGAGAAGAAAG  119976637


>ref|AC_000141.1| Homo sapiens chromosome 9, alternate assembly HuRef, whole genome 
shotgun sequence
 gb|CM000470.1| Homo sapiens chromosome 9, whole genome shotgun sequence
Length=110559325

 Score = 93.5 bits (50),  Expect = 5e-17
 Identities = 50/50 (100%), Gaps = 0/50 (0%)
 Strand=Plus/Minus

Query  1         GGACAGTCTGGGACATCCAGGGGAAGAGAAGGTGGACTTTGAGAAGAAAG  50
                 ||||||||||||||||||||||||||||||||||||||||||||||||||
Sbjct  89595074  GGACAGTCTGGGACATCCAGGGGAAGAGAAGGTGGACTTTGAGAAGAAAG  89595025


>gb|CM000260.1| Homo sapiens chromosome 9, whole genome shotgun sequence
Length=111583154

 Score = 93.5 bits (50),  Expect = 5e-17
 Identities = 50/50 (100%), Gaps = 0/50 (0%)
 Strand=Plus/Minus

Query  1         GGACAGTCTGGGACATCCAGGGGAAGAGAAGGTGGACTTTGAGAAGAAAG  50
                 ||||||||||||||||||||||||||||||||||||||||||||||||||
Sbjct  90624389  GGACAGTCTGGGACATCCAGGGGAAGAGAAGGTGGACTTTGAGAAGAAAG  90624340


>gb|CH003504.1| Homo sapiens chromosome 9, whole genome shotgun sequence
Length=116547660

 Score = 86.1 bits (46),  Expect = 8e-15
 Identities = 49/50 (98%), Gaps = 1/50 (2%)
 Strand=Plus/Minus

Query  1         GGACAGTCTGGGACATCCAGGGGAAGAGAAGGTGGACTTTGAGAAGAAAG  50
                 |||||||||||||||||||||||||||||||||||| |||||||||||||
Sbjct  95205951  GGACAGTCTGGGACATCCAGGGGAAGAGAAGGTGGA-TTTGAGAAGAAAG  95205903


Lambda     K      H
    1.33    0.621     1.12 

Gapped
Lambda     K      H
    1.28    0.460    0.850 

Effective search space used: 681780354108
 
 right 
 >ref|NW_001838339.2| Homo sapiens chromosome 16 genomic contig, alternate assembly 
HuRef SCAF_1103279188181, whole genome shotgun sequence
 gb|DS486217.1| Homo sapiens SCAF_1103279188181 genomic scaffold, whole genome 
shotgun sequence
Length=2624016

 Score = 93.5 bits (50),  Expect = 5e-17
 Identities = 50/50 (100%), Gaps = 0/50 (0%)
 Strand=Plus/Minus

Query  1        ACCCAAGCCTGCCCAATGTGCAGGTGACCAGGCTGACACTCCTGTCGGAA  50
                ||||||||||||||||||||||||||||||||||||||||||||||||||
Sbjct  2373746  ACCCAAGCCTGCCCAATGTGCAGGTGACCAGGCTGACACTCCTGTCGGAA  2373697


>ref|NT_010393.16| Homo sapiens chromosome 16 genomic contig, GRCh37.p9 Primary 
Assembly
 gb|GL000124.1| Homo sapiens chromosome 16 genomic contig, GRCh37 reference primary 
assembly
Length=33963150

 Score = 93.5 bits (50),  Expect = 5e-17
 Identities = 50/50 (100%), Gaps = 0/50 (0%)
 Strand=Plus/Plus

Query  1       ACCCAAGCCTGCCCAATGTGCAGGTGACCAGGCTGACACTCCTGTCGGAA  50
               ||||||||||||||||||||||||||||||||||||||||||||||||||
Sbjct  272025  ACCCAAGCCTGCCCAATGTGCAGGTGACCAGGCTGACACTCCTGTCGGAA  272074


>ref|NC_000016.9| Homo sapiens chromosome 16, GRCh37.p9 Primary Assembly
 gb|CM000678.1| Homo sapiens chromosome 16, GRCh37 primary reference assembly
Length=90354753

 Score = 93.5 bits (50),  Expect = 5e-17
 Identities = 50/50 (100%), Gaps = 0/50 (0%)
 Strand=Plus/Plus

Query  1       ACCCAAGCCTGCCCAATGTGCAGGTGACCAGGCTGACACTCCTGTCGGAA  50
               ||||||||||||||||||||||||||||||||||||||||||||||||||
Sbjct  332025  ACCCAAGCCTGCCCAATGTGCAGGTGACCAGGCTGACACTCCTGTCGGAA  332074


>ref|AC_000148.1| Homo sapiens chromosome 16, alternate assembly HuRef, whole genome 
shotgun sequence
 gb|CM000477.1| Homo sapiens chromosome 16, whole genome shotgun sequence
Length=75877710

 Score = 93.5 bits (50),  Expect = 5e-17
 Identities = 50/50 (100%), Gaps = 0/50 (0%)
 Strand=Plus/Plus

Query  1       ACCCAAGCCTGCCCAATGTGCAGGTGACCAGGCTGACACTCCTGTCGGAA  50
               ||||||||||||||||||||||||||||||||||||||||||||||||||
Sbjct  250271  ACCCAAGCCTGCCCAATGTGCAGGTGACCAGGCTGACACTCCTGTCGGAA  250320


>gb|CM000267.1| Homo sapiens chromosome 16, whole genome shotgun sequence
Length=75226909

 Score = 93.5 bits (50),  Expect = 5e-17
 Identities = 50/50 (100%), Gaps = 0/50 (0%)
 Strand=Plus/Plus

Query  1       ACCCAAGCCTGCCCAATGTGCAGGTGACCAGGCTGACACTCCTGTCGGAA  50
               ||||||||||||||||||||||||||||||||||||||||||||||||||
Sbjct  531927  ACCCAAGCCTGCCCAATGTGCAGGTGACCAGGCTGACACTCCTGTCGGAA  531976


Lambda     K      H
    1.33    0.621     1.12 

Gapped
Lambda     K      H
    1.28    0.460    0.850 

Effective search space used: 681780354108
 
 blast search - nt  
 left 
 >ref|XM_003833082.1| PREDICTED: Pan paniscus astrotactin 2 (ASTN2), mRNA
Length=4388

 Score = 93.5 bits (50),  Expect = 6e-17
 Identities = 50/50 (100%), Gaps = 0/50 (0%)
 Strand=Plus/Plus

Query  1    GGACAGTCTGGGACATCCAGGGGAAGAGAAGGTGGACTTTGAGAAGAAAG  50
            ||||||||||||||||||||||||||||||||||||||||||||||||||
Sbjct  720  GGACAGTCTGGGACATCCAGGGGAAGAGAAGGTGGACTTTGAGAAGAAAG  769


>ref|XM_002743253.2| PREDICTED: Callithrix jacchus astrotactin 2 (ASTN2), mRNA
Length=4562

 Score = 93.5 bits (50),  Expect = 6e-17
 Identities = 50/50 (100%), Gaps = 0/50 (0%)
 Strand=Plus/Plus

Query  1     GGACAGTCTGGGACATCCAGGGGAAGAGAAGGTGGACTTTGAGAAGAAAG  50
             ||||||||||||||||||||||||||||||||||||||||||||||||||
Sbjct  1053  GGACAGTCTGGGACATCCAGGGGAAGAGAAGGTGGACTTTGAGAAGAAAG  1102


>ref|XM_003312265.1| PREDICTED: Pan troglodytes astrotactin-2-like (LOC100609975), 
mRNA
Length=1665

 Score = 93.5 bits (50),  Expect = 6e-17
 Identities = 50/50 (100%), Gaps = 0/50 (0%)
 Strand=Plus/Plus

Query  1     GGACAGTCTGGGACATCCAGGGGAAGAGAAGGTGGACTTTGAGAAGAAAG  50
             ||||||||||||||||||||||||||||||||||||||||||||||||||
Sbjct  1062  GGACAGTCTGGGACATCCAGGGGAAGAGAAGGTGGACTTTGAGAAGAAAG  1111


>ref|XM_002820154.1| PREDICTED: Pongo abelii astrotactin-2-like (LOC100456865), partial 
mRNA
Length=2180

 Score = 93.5 bits (50),  Expect = 6e-17
 Identities = 50/50 (100%), Gaps = 0/50 (0%)
 Strand=Plus/Plus

Query  1    GGACAGTCTGGGACATCCAGGGGAAGAGAAGGTGGACTTTGAGAAGAAAG  50
            ||||||||||||||||||||||||||||||||||||||||||||||||||
Sbjct  750  GGACAGTCTGGGACATCCAGGGGAAGAGAAGGTGGACTTTGAGAAGAAAG  799


>ref|NG_021409.1| Homo sapiens astrotactin 2 (ASTN2), RefSeqGene on chromosome 
9
Length=996814

 Score = 93.5 bits (50),  Expect = 6e-17
 Identities = 50/50 (100%), Gaps = 0/50 (0%)
 Strand=Plus/Plus

Query  1       GGACAGTCTGGGACATCCAGGGGAAGAGAAGGTGGACTTTGAGAAGAAAG  50
               ||||||||||||||||||||||||||||||||||||||||||||||||||
Sbjct  205632  GGACAGTCTGGGACATCCAGGGGAAGAGAAGGTGGACTTTGAGAAGAAAG  205681


>ref|NM_014010.4| Homo sapiens astrotactin 2 (ASTN2), transcript variant 1, mRNA
Length=4604

 Score = 93.5 bits (50),  Expect = 6e-17
 Identities = 50/50 (100%), Gaps = 0/50 (0%)
 Strand=Plus/Plus

Query  1     GGACAGTCTGGGACATCCAGGGGAAGAGAAGGTGGACTTTGAGAAGAAAG  50
             ||||||||||||||||||||||||||||||||||||||||||||||||||
Sbjct  1067  GGACAGTCTGGGACATCCAGGGGAAGAGAAGGTGGACTTTGAGAAGAAAG  1116


>gb|AC240434.1| Homo sapiens FOSMID clone ABC16-1870K4 from chromosome 9, complete 
sequence
Length=33505

 Score = 93.5 bits (50),  Expect = 6e-17
 Identities = 50/50 (100%), Gaps = 0/50 (0%)
 Strand=Plus/Plus

Query  1      GGACAGTCTGGGACATCCAGGGGAAGAGAAGGTGGACTTTGAGAAGAAAG  50
              ||||||||||||||||||||||||||||||||||||||||||||||||||
Sbjct  15145  GGACAGTCTGGGACATCCAGGGGAAGAGAAGGTGGACTTTGAGAAGAAAG  15194


>dbj|AB383928.1| Synthetic construct DNA, clone: pF1KSDA0634, Homo sapiens ASTN2 
gene for astrotactin 2 isoform a, complete cds, without stop 
codon, in Flexi system
Length=3905

 Score = 93.5 bits (50),  Expect = 6e-17
 Identities = 50/50 (100%), Gaps = 0/50 (0%)
 Strand=Plus/Plus

Query  1     GGACAGTCTGGGACATCCAGGGGAAGAGAAGGTGGACTTTGAGAAGAAAG  50
             ||||||||||||||||||||||||||||||||||||||||||||||||||
Sbjct  993   GGACAGTCTGGGACATCCAGGGGAAGAGAAGGTGGACTTTGAGAAGAAAG  1042


>dbj|AK315025.1| Homo sapiens cDNA, FLJ95964, Homo sapiens astrotactin 2 (ASTN2), 
mRNA
Length=3032

 Score = 93.5 bits (50),  Expect = 6e-17
 Identities = 50/50 (100%), Gaps = 0/50 (0%)
 Strand=Plus/Plus

Query  1    GGACAGTCTGGGACATCCAGGGGAAGAGAAGGTGGACTTTGAGAAGAAAG  50
            ||||||||||||||||||||||||||||||||||||||||||||||||||
Sbjct  131  GGACAGTCTGGGACATCCAGGGGAAGAGAAGGTGGACTTTGAGAAGAAAG  180


>gb|BC146756.1| Homo sapiens astrotactin 2, mRNA (cDNA clone MGC:166822 IMAGE:9007192), 
complete cds
Length=4607

 Score = 93.5 bits (50),  Expect = 6e-17
 Identities = 50/50 (100%), Gaps = 0/50 (0%)
 Strand=Plus/Plus

Query  1     GGACAGTCTGGGACATCCAGGGGAAGAGAAGGTGGACTTTGAGAAGAAAG  50
             ||||||||||||||||||||||||||||||||||||||||||||||||||
Sbjct  1067  GGACAGTCTGGGACATCCAGGGGAAGAGAAGGTGGACTTTGAGAAGAAAG  1116


>dbj|AK127350.1| Homo sapiens cDNA FLJ45423 fis, clone BRHIP3036936, highly similar 
to Homo sapiens astrotactin 2 (ASTN2), transcript variant 
2, mRNA
Length=3812

 Score = 93.5 bits (50),  Expect = 6e-17
 Identities = 50/50 (100%), Gaps = 0/50 (0%)
 Strand=Plus/Plus

Query  1    GGACAGTCTGGGACATCCAGGGGAAGAGAAGGTGGACTTTGAGAAGAAAG  50
            ||||||||||||||||||||||||||||||||||||||||||||||||||
Sbjct  147  GGACAGTCTGGGACATCCAGGGGAAGAGAAGGTGGACTTTGAGAAGAAAG  196


>emb|AL355608.11| Human DNA sequence from clone RP11-567J2 on chromosome 9 Contains 
part of the ASTN2 gene for astrotactin 2 (KIAA0634) and 
a novel gene similar to ribosomal protein L10 (RPL10), complete 
sequence
Length=132590

 Score = 93.5 bits (50),  Expect = 6e-17
 Identities = 50/50 (100%), Gaps = 0/50 (0%)
 Strand=Plus/Minus

Query  1      GGACAGTCTGGGACATCCAGGGGAAGAGAAGGTGGACTTTGAGAAGAAAG  50
              ||||||||||||||||||||||||||||||||||||||||||||||||||
Sbjct  56945  GGACAGTCTGGGACATCCAGGGGAAGAGAAGGTGGACTTTGAGAAGAAAG  56896


>emb|BX649176.1| Homo sapiens mRNA; cDNA DKFZp686P1898 (from clone DKFZp686P1898)
Length=3950

 Score = 93.5 bits (50),  Expect = 6e-17
 Identities = 50/50 (100%), Gaps = 0/50 (0%)
 Strand=Plus/Plus

Query  1    GGACAGTCTGGGACATCCAGGGGAAGAGAAGGTGGACTTTGAGAAGAAAG  50
            ||||||||||||||||||||||||||||||||||||||||||||||||||
Sbjct  249  GGACAGTCTGGGACATCCAGGGGAAGAGAAGGTGGACTTTGAGAAGAAAG  298


>gb|AF116574.1|AF116574 Homo sapiens astrotactin2 (ASTN2) mRNA, complete cds
Length=3837

 Score = 93.5 bits (50),  Expect = 6e-17
 Identities = 50/50 (100%), Gaps = 0/50 (0%)
 Strand=Plus/Plus

Query  1    GGACAGTCTGGGACATCCAGGGGAAGAGAAGGTGGACTTTGAGAAGAAAG  50
            ||||||||||||||||||||||||||||||||||||||||||||||||||
Sbjct  288  GGACAGTCTGGGACATCCAGGGGAAGAGAAGGTGGACTTTGAGAAGAAAG  337


>dbj|AB014534.1| Homo sapiens mRNA for KIAA0634 protein, partial cds
Length=4591

 Score = 93.5 bits (50),  Expect = 6e-17
 Identities = 50/50 (100%), Gaps = 0/50 (0%)
 Strand=Plus/Plus

Query  1     GGACAGTCTGGGACATCCAGGGGAAGAGAAGGTGGACTTTGAGAAGAAAG  50
             ||||||||||||||||||||||||||||||||||||||||||||||||||
Sbjct  1067  GGACAGTCTGGGACATCCAGGGGAAGAGAAGGTGGACTTTGAGAAGAAAG  1116


>ref|XM_001098403.2| PREDICTED: Macaca mulatta astrotactin 2 (ASTN2), mRNA
Length=4720

 Score = 87.9 bits (47),  Expect = 3e-15
 Identities = 49/50 (98%), Gaps = 0/50 (0%)
 Strand=Plus/Plus

Query  1     GGACAGTCTGGGACATCCAGGGGAAGAGAAGGTGGACTTTGAGAAGAAAG  50
             |||||||||||||| |||||||||||||||||||||||||||||||||||
Sbjct  1049  GGACAGTCTGGGACGTCCAGGGGAAGAGAAGGTGGACTTTGAGAAGAAAG  1098


>ref|XM_003263952.1| PREDICTED: Nomascus leucogenys astrotactin-2-like (LOC100589612), 
mRNA
Length=2470

 Score = 86.1 bits (46),  Expect = 1e-14
 Identities = 48/49 (97%), Gaps = 0/49 (0%)
 Strand=Plus/Plus

Query  2    GACAGTCTGGGACATCCAGGGGAAGAGAAGGTGGACTTTGAGAAGAAAG  50
            ||||||||||||| |||||||||||||||||||||||||||||||||||
Sbjct  602  GACAGTCTGGGACGTCCAGGGGAAGAGAAGGTGGACTTTGAGAAGAAAG  650


Lambda     K      H
    1.33    0.621     1.12 

Gapped
Lambda     K      H
    1.28    0.460    0.850 

Effective search space used: 874911716973
 
 right 
 >ref|XM_003809402.1| PREDICTED: Pan paniscus protein disulfide isomerase family A, 
member 2 (PDIA2), mRNA
Length=2623

 Score = 93.5 bits (50),  Expect = 6e-17
 Identities = 50/50 (100%), Gaps = 0/50 (0%)
 Strand=Plus/Plus

Query  1    ACCCAAGCCTGCCCAATGTGCAGGTGACCAGGCTGACACTCCTGTCGGAA  50
            ||||||||||||||||||||||||||||||||||||||||||||||||||
Sbjct  187  ACCCAAGCCTGCCCAATGTGCAGGTGACCAGGCTGACACTCCTGTCGGAA  236


>dbj|AB593111.1| Homo sapiens ARHGDIG mRNA for rho GDP-dissociation inhibitor 
3, complete cds, clone: HP06965-RBd09D08
Length=1106

 Score = 93.5 bits (50),  Expect = 6e-17
 Identities = 50/50 (100%), Gaps = 0/50 (0%)
 Strand=Plus/Plus

Query  1    ACCCAAGCCTGCCCAATGTGCAGGTGACCAGGCTGACACTCCTGTCGGAA  50
            ||||||||||||||||||||||||||||||||||||||||||||||||||
Sbjct  488  ACCCAAGCCTGCCCAATGTGCAGGTGACCAGGCTGACACTCCTGTCGGAA  537


>dbj|AB590832.1| Synthetic construct DNA, clone: pFN21AE0909, Homo sapiens ARHGDIG 
gene for Rho GDP dissociation inhibitor (GDI) gamma, without 
stop codon, in Flexi system
Length=692

 Score = 93.5 bits (50),  Expect = 6e-17
 Identities = 50/50 (100%), Gaps = 0/50 (0%)
 Strand=Plus/Plus

Query  1    ACCCAAGCCTGCCCAATGTGCAGGTGACCAGGCTGACACTCCTGTCGGAA  50
            ||||||||||||||||||||||||||||||||||||||||||||||||||
Sbjct  263  ACCCAAGCCTGCCCAATGTGCAGGTGACCAGGCTGACACTCCTGTCGGAA  312


>ref|NM_001176.3| Homo sapiens Rho GDP dissociation inhibitor (GDI) gamma (ARHGDIG), 
mRNA
Length=942

 Score = 93.5 bits (50),  Expect = 6e-17
 Identities = 50/50 (100%), Gaps = 0/50 (0%)
 Strand=Plus/Plus

Query  1    ACCCAAGCCTGCCCAATGTGCAGGTGACCAGGCTGACACTCCTGTCGGAA  50
            ||||||||||||||||||||||||||||||||||||||||||||||||||
Sbjct  329  ACCCAAGCCTGCCCAATGTGCAGGTGACCAGGCTGACACTCCTGTCGGAA  378


>emb|CU690464.1| Synthetic construct Homo sapiens gateway clone IMAGE:100021341 
5' read ARHGDIG mRNA
Length=1249

 Score = 93.5 bits (50),  Expect = 6e-17
 Identities = 50/50 (100%), Gaps = 0/50 (0%)
 Strand=Plus/Plus

Query  1    ACCCAAGCCTGCCCAATGTGCAGGTGACCAGGCTGACACTCCTGTCGGAA  50
            ||||||||||||||||||||||||||||||||||||||||||||||||||
Sbjct  270  ACCCAAGCCTGCCCAATGTGCAGGTGACCAGGCTGACACTCCTGTCGGAA  319


>gb|EF450770.1| Homo sapiens protein disulfide isomerase family A, member 2 variant 
(PDIA2) gene, complete cds
Length=7706

 Score = 93.5 bits (50),  Expect = 6e-17
 Identities = 50/50 (100%), Gaps = 0/50 (0%)
 Strand=Plus/Plus

Query  1    ACCCAAGCCTGCCCAATGTGCAGGTGACCAGGCTGACACTCCTGTCGGAA  50
            ||||||||||||||||||||||||||||||||||||||||||||||||||
Sbjct  667  ACCCAAGCCTGCCCAATGTGCAGGTGACCAGGCTGACACTCCTGTCGGAA  716


>ref|XM_001171621.1| PREDICTED: Pan troglodytes Rho GDP dissociation inhibitor (GDI) 
gamma (ARHGDIG), mRNA
Length=905

 Score = 93.5 bits (50),  Expect = 6e-17
 Identities = 50/50 (100%), Gaps = 0/50 (0%)
 Strand=Plus/Plus

Query  1    ACCCAAGCCTGCCCAATGTGCAGGTGACCAGGCTGACACTCCTGTCGGAA  50
            ||||||||||||||||||||||||||||||||||||||||||||||||||
Sbjct  305  ACCCAAGCCTGCCCAATGTGCAGGTGACCAGGCTGACACTCCTGTCGGAA  354


>dbj|AB127078.1| Homo sapiens ARHGDIG, PDIP polycistronic mRNA for Rho GDP dissociation 
inhibitor gamma, pancreatic protein disulfide isomerase, 
complete cds
Length=2626

 Score = 93.5 bits (50),  Expect = 6e-17
 Identities = 50/50 (100%), Gaps = 0/50 (0%)
 Strand=Plus/Plus

Query  1    ACCCAAGCCTGCCCAATGTGCAGGTGACCAGGCTGACACTCCTGTCGGAA  50
            ||||||||||||||||||||||||||||||||||||||||||||||||||
Sbjct  254  ACCCAAGCCTGCCCAATGTGCAGGTGACCAGGCTGACACTCCTGTCGGAA  303


>gb|AF498928.1| Homo sapiens Rho GDP dissociation inhibitor gamma (ARHGDIG) mRNA, 
complete cds
Length=678

 Score = 93.5 bits (50),  Expect = 6e-17
 Identities = 50/50 (100%), Gaps = 0/50 (0%)
 Strand=Plus/Plus

Query  1    ACCCAAGCCTGCCCAATGTGCAGGTGACCAGGCTGACACTCCTGTCGGAA  50
            ||||||||||||||||||||||||||||||||||||||||||||||||||
Sbjct  254  ACCCAAGCCTGCCCAATGTGCAGGTGACCAGGCTGACACTCCTGTCGGAA  303


>gb|AE006463.1| Homo sapiens 16p13.3 sequence section 2 of 8
Length=260418

 Score = 93.5 bits (50),  Expect = 6e-17
 Identities = 50/50 (100%), Gaps = 0/50 (0%)
 Strand=Plus/Plus

Query  1      ACCCAAGCCTGCCCAATGTGCAGGTGACCAGGCTGACACTCCTGTCGGAA  50
              ||||||||||||||||||||||||||||||||||||||||||||||||||
Sbjct  14621  ACCCAAGCCTGCCCAATGTGCAGGTGACCAGGCTGACACTCCTGTCGGAA  14670


>emb|Z69667.1| Human DNA sequence from clone LA16c-314G4 on chromosome 16 Contains 
the 3' end of the C16orf9 gene for chromosome 16 open 
reading frame 9, the RGS11 gene for regulator of G-protein 
signalling 11, a novel gene, the ARHGDIG gene for Rho GDP dissociation 
inhibitor (GDI) gamma, the PDIP gene for protein 
disulfide isomerase, pancreatic, the 3' end of the AXIN1 gene 
for axin 1 and two CpG islands, complete sequence
Length=31557

 Score = 93.5 bits (50),  Expect = 6e-17
 Identities = 50/50 (100%), Gaps = 0/50 (0%)
 Strand=Plus/Plus

Query  1      ACCCAAGCCTGCCCAATGTGCAGGTGACCAGGCTGACACTCCTGTCGGAA  50
              ||||||||||||||||||||||||||||||||||||||||||||||||||
Sbjct  19625  ACCCAAGCCTGCCCAATGTGCAGGTGACCAGGCTGACACTCCTGTCGGAA  19674


>gb|BC047699.1| Homo sapiens Rho GDP dissociation inhibitor (GDI) gamma, mRNA 
(cDNA clone MGC:51949 IMAGE:5581679), complete cds
Length=983

 Score = 93.5 bits (50),  Expect = 6e-17
 Identities = 50/50 (100%), Gaps = 0/50 (0%)
 Strand=Plus/Plus

Query  1    ACCCAAGCCTGCCCAATGTGCAGGTGACCAGGCTGACACTCCTGTCGGAA  50
            ||||||||||||||||||||||||||||||||||||||||||||||||||
Sbjct  330  ACCCAAGCCTGCCCAATGTGCAGGTGACCAGGCTGACACTCCTGTCGGAA  379


>gb|AF080237.1|AF080237 Homo sapiens Rho GDP-dissociation inhibitor gamma (ARHGDIG) gene, 
complete cds
Length=4300

 Score = 93.5 bits (50),  Expect = 6e-17
 Identities = 50/50 (100%), Gaps = 0/50 (0%)
 Strand=Plus/Plus

Query  1     ACCCAAGCCTGCCCAATGTGCAGGTGACCAGGCTGACACTCCTGTCGGAA  50
             ||||||||||||||||||||||||||||||||||||||||||||||||||
Sbjct  2625  ACCCAAGCCTGCCCAATGTGCAGGTGACCAGGCTGACACTCCTGTCGGAA  2674


>gb|U82532.1|HSU82532 Homo sapiens GDI-dissociation inhibitor RhoGDIgamma mRNA, complete 
cds
Length=802

 Score = 93.5 bits (50),  Expect = 6e-17
 Identities = 50/50 (100%), Gaps = 0/50 (0%)
 Strand=Plus/Plus

Query  1    ACCCAAGCCTGCCCAATGTGCAGGTGACCAGGCTGACACTCCTGTCGGAA  50
            ||||||||||||||||||||||||||||||||||||||||||||||||||
Sbjct  292  ACCCAAGCCTGCCCAATGTGCAGGTGACCAGGCTGACACTCCTGTCGGAA  341


>gb|DQ656001.2| Homo sapiens clone Affy08250F05, mRNA sequence
Length=520

 Score = 87.9 bits (47),  Expect = 3e-15
 Identities = 49/50 (98%), Gaps = 0/50 (0%)
 Strand=Plus/Minus

Query  1   ACCCAAGCCTGCCCAATGTGCAGGTGACCAGGCTGACACTCCTGTCGGAA  50
           |||||||||||||||||||||||||||||| |||||||||||||||||||
Sbjct  60  ACCCAAGCCTGCCCAATGTGCAGGTGACCATGCTGACACTCCTGTCGGAA  11


>gb|DQ656000.2| Homo sapiens clone Affy08250B05, mRNA sequence
Length=338

 Score = 87.9 bits (47),  Expect = 3e-15
 Identities = 49/50 (98%), Gaps = 0/50 (0%)
 Strand=Plus/Minus

Query  1   ACCCAAGCCTGCCCAATGTGCAGGTGACCAGGCTGACACTCCTGTCGGAA  50
           ||||||||||||||||||||||||||||||||||||| ||||||||||||
Sbjct  60  ACCCAAGCCTGCCCAATGTGCAGGTGACCAGGCTGACGCTCCTGTCGGAA  11


>ref|XM_002825909.2| PREDICTED: Pongo abelii uncharacterized LOC100433846 (LOC100433846), 
mRNA
Length=1387

 Score = 86.1 bits (46),  Expect = 1e-14
 Identities = 46/46 (100%), Gaps = 0/46 (0%)
 Strand=Plus/Plus

Query  1    ACCCAAGCCTGCCCAATGTGCAGGTGACCAGGCTGACACTCCTGTC  46
            ||||||||||||||||||||||||||||||||||||||||||||||
Sbjct  878  ACCCAAGCCTGCCCAATGTGCAGGTGACCAGGCTGACACTCCTGTC  923


>gb|AC197388.4| Pongo abelii BAC clone CH276-522N14 from chromosome 16, complete 
sequence
Length=211635

 Score = 86.1 bits (46),  Expect = 1e-14
 Identities = 46/46 (100%), Gaps = 0/46 (0%)
 Strand=Plus/Plus

Query  1      ACCCAAGCCTGCCCAATGTGCAGGTGACCAGGCTGACACTCCTGTC  46
              ||||||||||||||||||||||||||||||||||||||||||||||
Sbjct  79194  ACCCAAGCCTGCCCAATGTGCAGGTGACCAGGCTGACACTCCTGTC  79239


>ref|XM_001118456.2| PREDICTED: Macaca mulatta rho GDP-dissociation inhibitor 3-like 
(LOC722292), mRNA
Length=874

 Score = 80.5 bits (43),  Expect = 5e-13
 Identities = 47/49 (95%), Gaps = 0/49 (0%)
 Strand=Plus/Plus

Query  1    ACCCAAGCCTGCCCAATGTGCAGGTGACCAGGCTGACACTCCTGTCGGA  49
            |||||||||||||||||||||||||||||||||| |||||| |||||||
Sbjct  365  ACCCAAGCCTGCCCAATGTGCAGGTGACCAGGCTAACACTCTTGTCGGA  413


>ref|NM_001168819.1| Papio anubis Rho GDP dissociation inhibitor (GDI) gamma (ARHGDIG), 
mRNA
Length=678

 Score = 80.5 bits (43),  Expect = 5e-13
 Identities = 47/49 (95%), Gaps = 0/49 (0%)
 Strand=Plus/Plus

Query  1    ACCCAAGCCTGCCCAATGTGCAGGTGACCAGGCTGACACTCCTGTCGGA  49
            |||||||||||||||||||||||||||||||||| |||||| |||||||
Sbjct  254  ACCCAAGCCTGCCCAATGTGCAGGTGACCAGGCTAACACTCTTGTCGGA  302


Lambda     K      H
    1.33    0.621     1.12 

Gapped
Lambda     K      H
    1.28    0.460    0.850 

Effective search space used: 874911716973
 
    reads 
 chr9 chr9 119976874 119976798 76m                                    GCTGGGTCCCCAGGCGCGGGAGAGCTTCCGTTCATCCCGGCTGCAAACCCACAATTCCGTCATTGGCGTGCCCATC
chr9 chr9 119976870 119976794 76m                                        GGTCCCCAGGCGCGGGAGAGCTTCCGTTCATCCCGGCTGCAAACCCACAATTCCGTCATTGGCGTGCCCATCCGGG
chr9 chr9 119976866 119976790 76m                                            CCCAGGCGCGGGAGAGCTTCCGTTCATCCCGGCTGCAAACCCACAATTCCGTCATTGGCGTGCCCATCCGGGAGAC
chr9 chr9 119976863 119976787 76m                                               AGGCGCGGGAGAGCTTCCGTTCATCCCGGCTGCAAACCCACAATTCCGTCATTGGCGTGCCCATCCGGGAGACTCC
chr9 chr9 119976860 119976784 76m                                                  CGCGGGAGAGCTTCCGTTCATCCCGGCTGCAAACCCACAATTCCGTCATTGGCGTGCCCATCCGGGAGACTCCCAT
chr9 chr9 119976857 119976781 76m                                                     GGGAGAGCTTCCGTTCATCCCGGCTGCAAACCCACAATTCCGTCATTGGCGTGCCCATCCGGGAGACTCCCATCCT
chr9 chr9 119976854 119976778 76m                                                        AGAGCTTCCGTTCATCCCGGCTGCAAACCCACAATTCCGTCATTGGCGTGCCCATCCGGGAGACTCCCATCCTGGA
chr9 chr9 119976851 119976775 76m                                                           GCTTCCGTTCATCCCGGCTGCAAACCCACAATTCCGTCATTGGCGTGCCCATCCGGGAGACTCCCATCCTGGATGA
chr9 chr9 119976848 119976772 76m                                                              TCCGTTCATCCCGGCTGCAAACCCATAATTCCGTCATTGGCGTGCCCATCCGGGAGACTCCCATCCTGGATGACTA
chr9 chr9 119976845 119976769 76m                                                                 GTTCATCCCGGCTGCAAACCCACAATTCCGTCATTGGCGTGCCCATCCGGGAGACTCCCATCCTGGATGACTATGA
chr9 chr9 119976840 119976764 76m                                                                      TCCCGGCTGCAAACCCACAATTCCGTCATTGGCGTGCCCATCCGGGAGACTCCCATCCTGGATGACTATGACTGCG
chr9 chr9 119976837 119976761 76m                                                                         CGGCTGCAAACCCACAATTCCGTCATTGGCGTGCCCATCCGGGAGACCCCCATCCTGGATGACTATGACTGTGAGG
chr9 chr9 119976833 119976757 76m                                                                             TGCAAACCCACAATTCCGTCATTGGCGTGCCCATCCGGGAGACTCCCATCCTGGATGACTATGACTGTGAGGAGGG
chr9 chr9 119976829 119976753 76m                                                                                 AACCCACAATTCCGTCATTGGCGTGCCCATCCGGGAGACTCCCATCCTGGATGACTATGACTGTGAGGAGGATGAG
chr9 chr9 119976826 119976750 76m                                                                                    CCACAATTCCGTCATTGGCGTGCCCATCCGGGAGACTCCCATCCTGGATGACTATGACTGTGAGGAGGATGAGGAG
chr9 chr9 119976823 119976747 76m                                                                                       CAATTCCGTCATTGGCGTGCCCATCCGGGAGACTCCCATCCTGGATGACTATGACTGTGAGGAGGATGAGGAGCCA
chr9 chr9 119976820 119976744 76m                                                                                          TTCCGTCATTGGCGTGCCCATCCGGGAGACTCCCATCCTGGATGACTATGACTGTGAGGAGGATGAGGAGCCACCT
chr9 chr9 119976817 119976741 76m                                                                                             CGTCATTGGCGTGCCCATCCGGGAGACTCCCATCCTGGATGACTATGACTGTGAGGAGGATGAGGAGCCACCTAGG
chr9 chr9 119976814 119976738 76m                                                                                                CATTGGCGTGCCCATCCGGGAGACTCCCATCCTGGATGACTATGACTGTGAGGAGGATGAGGAGCCACCTAGGCGG
chr9 chr9 119976811 119976735 76m                                                                                                   TGGCGTGCCCATCCGGGAGACTCCCATCCTGGATGACTATGACTGTGAGGAGGATGAGGAGCCACCTAGGCGGGCC
chr9 chr9 119976808 119976732 76m                                                                                                      CGTGCCCATCCGGGAGACTCCCATCCTGGATGACTATGACTGTGAGGAGGATGAGGAGCCACCTAGGCGGGCCAAC
chr9 chr9 119976805 119976729 76m                                                                                                         GCCCATCCGGGAGACTCCCATCCTGGATGACTATGACTGTGAGGAGGATGAGGAGCCACCTAGGCGGGCCAACCAT
chr9 chr9 119976802 119976726 76m                                                                                                            CATCCGGGAGACTCCCATCCTGGATGACTATGACTGTGAGGAGGATGAGGAGCCACCTAGGCGGGCCAACCATGTC
chr9 chr9 119976799 119976723 76m                                                                                                               CCGGGAGACTCCCATCCTGGATGACTATGACTGTGAGGAGGATCAGGAGCCACCTAGGCGGGCCAACCATGCCTCC
chr9 chr9 119976796 119976720 76m                                                                                                                  GGAGACTCCCATCCTGGATGACTATGACTGTGAGGAGGATGAGGAGCCACCTAGGCGGGCCAACCATGTCTCCCGC
chr9 chr9 119976793 119976717 76m                                                                                                                     GACTCCCATCCTGGATGACTATGACTGTGAGGAGGATGAGGAGCCACCTAGGCGGGCCAACCATGTCTCCCGCGAG
chr9 chr9 119976789 119976713 76m                                                                                                                         CCCATCCTGGATGACTATGACTGTGAGGAGGATGAGGAGCCACCTAGGCGGGCCAACCATGTCTCCCGCGAGGACG
chr9 chr9 119976785 119976709 76m                                                                                                                             TCCTGGATGACTATGACTGTGAGGAGGATGAGGAGCCACCTAGGCGGGCCAACCATGTCTCCCGCGAGGACGAGTT
chr9 chr9 119976782 119976706 76m                                                                                                                                TGGATGACTATGACTGTGAGGAGGATGAGGAGCCACCTAGGCGGGCCAACCATGTCTCCCGCGAGGACGAGTTTGG
chr9 chr9 119976779 119976703 76m                                                                                                                                   ATGACTATGACTGTGAGGAGGATGAGGAGCCACCTAGGCGGGCCAACCATGTCTCCCGCGAGGACGAGTTTGGCAG
chr9 chr9 119976776 119976700 76m                                                                                                                                      ACTATGACTGTGAGGAGGATGAGGAGCCACCTAGGCGGGCCAACCATGTCTCCCGCGAGGACGAGTTTGGCAGCCG
chr9 chr9 119976773 119976697 76m                                                                                                                                         ATGACTGTGAGGAGGATGAGGAGCCACCTAGGAGGGCCAACCATGTCTCCCGCGAGGACGAGTTTGGCAGCCAGGT
chr9 chr9 119976770 119976694 76m                                                                                                                                            ACTGTGAGGAGGATGAGGAGCCACCTAGGCGGGCCAACCATGTCTCCCGCGAGGACGAGTTTGGCAGCCAGGTGAC
chr9 chr9 119976767 119976691 76m                                                                                                                                               GTGAGGAGGATGAGGAGCCACCTAGGCGGGCCAACCATGTCTCCCGCGAGGACGAGTTTGGCAGCCAGGTGACCCA
chr9 chr9 119976764 119976688 76m                                                                                                                                                  AGGAGGATGAGGAGCCACCTAGGCGGGCCAACCATGTCTCCCGCGAGGACGAGTTTGGCAGCCAGGTGACCCACAC
chr9 chr9 119976760 119976684 76m                                                                                                                                                      GGATGAGGAGCCACCTAGGCGGGCCAACCATGTCTCCCGCGAGGACGAGTTTGGCAGCCAGGTGACCCACACTCTG
chr9 chr9 119976757 119976681 76m                                                                                                                                                         TGAGGAGCCACCTAGGCGGGCCAACCATGTCTCCCGCGAGGACGAGTTTGGCAGCCAGGTGACCCACACTCTGGAC
chr9 chr9 119976753 119976677 76m                                                                                                                                                             GAGCCACCTAGGCGGGCCAACCATGTCTCCCGCGAGGACGAGCTTGGCAGCCAGGAGACCCACACTCTGGACAGTC
chr9 chr9 119976744 119976668 76m                                                                                                                                                                      AGGCGGGCCAACCATGTCTCCCGCGAGGACGAGTTTGGCAGCCAGGTGACCCACACTCTGGACAGGCTGGGACATC
chr9 chr9 119976741 119976665 76m                                                                                                                                                                         CGGGCCAACCATGTCTCCCGCGAGGACGAGTTTGGCAGCCAGGTGACCCACACTCTGGACAGTCTGGGACATCCAG
chr9 chr9 119976738 119976662 76m                                                                                                                                                                            GCCAACCATGTCTCCCGCGAGGACGAGTTTGGCAGCCAGGTGACCCACACTCTGGACAGTCTGGGACATCCAGGGG
chr9 chr9 119976735 119976659 76m                                                                                                                                                                               AACCATGTCTCCCGCGAGGACGAGTTTGGCAGCCAGGTGACCCACACTCTGGACAGTCTGGGACATCCAGGGGAAG
chr9 chr9 119976732 119976656 76m                                                                                                                                                                                  CATGTCTCCCGCGAGGACGAGTTTGGCAGCCAGGTGACCCACACTCTGGACAGTCTGGGACATCCAGGGGAAGAGA
chr9 chr9 119976729 119976653 76m                                                                                                                                                                                     GTCTCCCGCGAGGACGAGTTTGGCAGCCAGGTGACCCACACTCTGGACAGTCTGGGACATCCAGGGGAAGAGAAGG
chr9 chr9 119976725 119976649 76m                                                                                                                                                                                         CCCGCGAGGACGAGTTTGGCAGCCAGGTGACCCACACTCTGGACAGTCTGGGACATCCAGGGGAAGAGAAGGTGGA
chr9 chr9 119976722 119976646 76m                                                                                                                                                                                            GCGAGGACGAGTTTGGCAGCCAGGTGACCCACACTCTGGACAGTCTGGGACATCCAGGGGAAGAGAAGGTGGACTT
chr9 chr9 119976719 119976643 76m                                                                                                                                                                                               AGGACGAGTTTGGCAGCCAGGTGACCCACACTCTGGACAGTCTGGGACATCCAGGGGAAGAGAAGGTGGACTTTGA
chr9 chr9 119976716 119976640 76m                                                                                                                                                                                                  ACGAGTTTGGCAGCCAGGTGACCCACACTCTGGACAGTCTGGGACATCCAGGGGAAGAGAAGGTGGACTTTGAGAA
chr9 chr9 119976713 119976637 76m                                                                                                                                                                                                     AGTTTGGCAGCCAGGTGACCCACACTCTGGACAGTCTGGGACATCCAGGGGAAGAGAAGGTGGACTTTGAGAAGAA
chr9 chr9 119976710 119976634 76m                                                                                                                                                                                                        TTGGCAGCCAGGTGACCCACACTCTGGACAGTCTGGGACATCCAGGGGAAGAGAAGGTGGACTTTGAGAAGAAAGC
chr9 chr16 119976706 332029 71m332025F5M                                                                                                                                                                                                     TGGGTCTTTCTTCTCAAAGTCCACCTTCTCTTCCCCTGGATGTCCCAGACTGTCCAGAGTGTGGGTCACCT GGCTG
chr9 chr16 119976704 332031 69m332025F7M                                                                                                                                                                                                       CTTGGGTCTTTCTTCTCAAAGTCCACCTTCTCTTCCCCTGGATGTCCCAGACTGTCCAGAGTGTGGGTC ACCTAGA
chr9 chr16 119976704 332031 69m332025F7M                                                                                                                                                                                                       CTTGGGTCTTTCTTCTCAAAGTCCACCTTCTCTTCCCCTGGATGTCCCAGACTGTCCAGAGTGTGGGTC ACCTGGC
chr9 chr16 119976704 332031 69m332025F7M                                                                                                                                                                                                       GCCAGGTGACCCACACTCTGGACAGTCTGGGACATCCAGGGGAAGAGAAGGTGGACTTTGAGAAGAAAG ACCCAAG
chr9 chr16 119976704 332031 69m332025F7M                                                                                                                                                                                                       GCCAGGTGACCCACACTCTGGACAGTCTGGGACATCCAGGGGAAGAGAAGGTGGACTTTGAGAAGAAAG ACCCAAG
chr9 chr16 119976702 332033 67m332025F9M                                                                                                                                                                                                         GGCTTGGGTCTTTCTTCTCAAAGTCCACCTTCTCTTCCCCTGGATGTCCCAGACTGTCCAGAGTGTG GGACACCTG
chr9 chr16 119976702 332033 67m332025F9M                                                                                                                                                                                                         CAGGTGACCCACACTCTGGACAGTCTGGGACATCCAGGGGAAGAGAAGGTGGACTTTGAGAAGAAAG ACCCAAGCC
chr9 chr16 119976701 332034 66m332025F10M                                                                                                                                                                                                         AGGTGACCCACACTCTGGACAGTCTGGGACATCCAGGGGAAGAGAAGGTGGACTTTGAGAAGAAAG ACCCAAGCCT
chr9 chr16 119976701 332034 66m332025F10M                                                                                                                                                                                                         AGGTGACCCACACTCTGGACAGTCTGGGACATCCAGGGGAAGAGAAGGTGGACTTTGAGAAGAAAG ACCCAAGCCT
chr9 chr16 119976699 332036 64m332025F12M                                                                                                                                                                                                           GTGACCCACACTCTGGACAGTCTGGGACATCCAGGGGAAGAGAAGGTGGACTTTGAGAAGAAAG ACCCAAGCCTGC
chr9 chr16 119976699 332036 64m332025F12M                                                                                                                                                                                                           GCAGGCTTGGGTCTTTCTTCTCAAAGTCCACCTTCTCTTCCCCTGGATGTCCCAGACTGTCCAG AGTGTGGGTCAC
chr9 chr16 119976699 332036 64m332025F12M                                                                                                                                                                                                           GTGACCCACACTCTGGACAGTCTGGGACATCCAGGGGAAGAGAAGGTGGACTTTGAGAAGAAAG ACCCAAGCCTGC
chr9 chr16 119976697 332038 62m332025F14M                                                                                                                                                                                                             GGGCAGGCTTGGGTCTTTCTTCTCAAAGTCCACCTTCTCTTCCCCTGGATGTCCCAGACTGT CCAGAGTGTGGGTC
chr9 chr16 119976696 332039 61m332025F15M                                                                                                                                                                                                              TGGGCAGGCTTGGGTCTTTCTTCTCAAAGTCCACCTTCTCTTCCCCTGGATGTCCCAGACT GTCCAGAGTGTGGGT
chr9 chr16 119976695 332040 60m332025F16M                                                                                                                                                                                                               TTGGGCAGGCTTGGGTCTTTCTTCTCAAAGTCCACCTTCTCTTCCCCTGGATGTCCCAGA CTGTCCAGAGTGTGGG
chr9 chr16 119976694 332041 59m332025F17M                                                                                                                                                                                                                CTACACTCTGGACAGTCTGGGACCTCCAGGGGAAGAGAAGGTGGACTTTGAGAAGAAAG ACCCAAGCCTGCCCAAT
chr9 chr16 119976687 332048 52m332025F24M                                                                                                                                                                                                                       CTGGACAGTCTGGGACATCCAGGGGAAGAGAAGGTGGACTTTGAGAAGAAAG ACCCAAGCCTGCCCAATGTGCAGG
chr9 chr16 119976687 332048 52m332025F24M                                                                                                                                                                                                                       CTGGACAGTCTGGGACATCCAGGGGAAGAGAAGGTGGACTTTGAGAAGAAAG ACCCAAGCCTGCCCAATGTGCAGG
chr9 chr16 119976686 332049 51m332025F25M                                                                                                                                                                                                                        TGGACAGTCTGGGACATCCAGGGGAAGAGAAGGTGGACTTTGAGAAGAAAG ACCCAAGCCTGCCCAATGTGCAGGT
chr9 chr16 119976685 332050 50m332025F26M                                                                                                                                                                                                                         GGACAGTCTGGGACATCCAGGGGAAGAGAAGGTGGACTTTGAGAAGAAAG ACCCAAGCCTGCCCAATGTGCAGGTG
chr9 chr16 119976685 332050 50m332025F26M                                                                                                                                                                                                                         GGACAGTCTGGGACATCCAGGGGAAGAGAAGGGGGACTTTGAGAAGAAAG ACCCAAGCCTGCCCAATGTGCAGGTG
chr9 chr16 119976683 332052 48m332025F28M                                                                                                                                                                                                                           ACAGTCTGGGACATCCAGGGGAAGAGAAGGTGGACTTTGAGAAGAAAG ACCCAAGCCTGCCCAATGTGCAGGTGAC
chr9 chr16 119976682 332053 47m332025F29M                                                                                                                                                                                                                            CAGTCTGGGACATCCAGGGGAAGAGAAGGTGGACTTTGAGAAGAAAG ACCCAAGCCTGCCCAATGTGCAGGTGACC
chr9 chr16 119976682 332053 47m332025F29M                                                                                                                                                                                                                            CAGTCTGGGACATCCAGGGGAAGAGAAGGTGGACTTTGAGAAGAAAG ACCCAAGCCTGCCCAATGTGCAGGTGACC
chr9 chr16 119976680 332055 45m332025F31M                                                                                                                                                                                                                              GTCTGGGACATCCAGGGGAAGAGAAGGTGGACTTTGAGAAGAAAG ACCCAAGCCTGCCCAATGTGCAGGTGACCAG
chr9 chr16 119976680 332055 45m332025F31M                                                                                                                                                                                                                              GTCTGGGACATCCAGGGGAAGAGAAGGTGGACTTTGAGAAGAAAG ACCCAAGCCTGCCCAATGTGCAGGTGACCAG
chr9 chr16 119976678 332057 43m332025F33M                                                                                                                                                                                                                                TTGGGACATCCAGGGGAAGAGAAGGTGGACTTTGAGAAGAAAG ACCCAAGCCTGCCCAATGTGCAGGTGACCAGGC
chr9 chr16 119976678 332057 43m332025F33M                                                                                                                                                                                                                                CTGGGACATCCAGGGGAAGAGAAGGTGGACTTTGAGAAGAAAG ACCCAAGCCTGCCCAATGTGCAGGTGACCAGGC
chr9 chr16 119976678 332057 43m332025F33M                                                                                                                                                                                                                                CTGGGACATCCAGGGGAAGAGAAGGTGGACTTTGAGAAGAAAG ACCCAAGCCTGCCCAATGTGCAGGTGACCAGGC
chr9 chr16 119976678 332057 43m332025F33M                                                                                                                                                                                                                                CTGGGACATCCAGGGGAAGAGAAGGTGGACTTTGAGAAGAAAG ACCCAAGCCTGCCCAATGTGCAGGTGACCAGGC
chr9 chr16 119976678 332057 43m332025F33M                                                                                                                                                                                                                                CTGGGACATCCAGGGGAAGAGAAGGTGGACTTTGAGAAGAAAG ACCCAAGCCTGCCCAATGTGCAGGTGACCAGGC
chr9 chr16 119976678 332057 43m332025F33M                                                                                                                                                                                                                                CTGGGACATCCAGGGGAAGAGAAGGTGGGCTTTGAGAAGAAAG ACCCAAGCCTGCCCAATGTGCAGGTGACCAGGC
chr9 chr16 119976677 332058 42m332025F34M                                                                                                                                                                                                                                 TGGGACATCCAGGGGAAGAGAAGGTGGACTTTGAGAAGAAAG ACCCAAGCCTGCCCAATGTGCAGGTGACCAGGCG
chr9 chr16 119976676 332059 41m332025F35M                                                                                                                                                                                                                                  GGGACATCCAGGGGAAGAGAAGGTGGACTTTGAGAAGAAAG ACCCAAGCCTTCCCAATGTGCAGGTGACCAGGCTG
chr9 chr16 119976676 332059 41m332025F35M                                                                                                                                                                                                                                  GGGACATCCAGGGGAAGAGAAGGTGGACTTTGAGAAGAAAG ACCCAAGCCTGCCCAATGTGCAGGTGACCAGGCTG
chr9 chr16 119976676 332059 41m332025F35M                                                                                                                                                                                                                                  GGGACATCCAGGGGAAGAGAAGGTGGACTTTGAGAAGAAAG ACCCAAGCCTGCCCAATGTGCAGGTGACCAGGCTG
chr9 chr16 119976676 332059 41m332025F35M                                                                                                                                                                                                                                  GGGACATCCAGGGGAATAGAAGGTGGACTTTGAGAAGAAAG ACCCAAGCCTGCCCAATGTGCAGGTGACCAGGCTG
chr9 chr16 119976676 332059 41m332025F35M                                                                                                                                                                                                                                  GGGACATCCAGGGGAAGAGAAGGTGGACTTTGAGAAGAAAG ACCCAAGCCTGCCCAATGTGCAGGTGACCAGGCTG
chr9 chr16 119976675 332060 40m332025F36M                                                                                                                                                                                                                                   GGACATCCAGGGGAAGAGAAGGTGGACTTTGAGAAGAAAG ACCCAAGCCTGCCCAATGTGCAGGTGACCAGGCTGA
chr9 chr16 119976674 332061 39m332025F37M                                                                                                                                                                                                                                    GACATCCAGGGGAAGAGAAGGTGGACTTTGAGAAGAAAG ACCCAAGCCTGCCCAATGTGCAGGTGACCAGGCTGAC
chr9 chr16 119976672 332063 37m332025F39M                                                                                                                                                                                                                                      CATCCAGGGGAAGAGAAGGTGGACTTTGAGAAGAAAG ACCCAAGCCTGCCCAATGTGCAGGTGACCAGGCTGACAC
chr9 chr16 119976669 332066 34m332025F42M                                                                                                                                                                                                                                         CCAGGGGAAGAGAAGGTGGACTTTGAGAAGAAAG ACCCAAGCCTGCCCAATGTGCAGGTGACCAGGCTGACACTCC
chr9 chr16 119976668 332067 33m332025F43M                                                                                                                                                                                                                                          CAGGGAAAGAGAAGGTGGACTTTGAGAAGAAAG CCCCAAGCCTGCCCAATGTGCAGGTGACCAGGCTGACACTCCT
chr9 chr16 119976667 332068 32m332025F44M                                                                                                                                                                                                                                           AGGGGAAGAGAAGGTGGACTTTGAGAAGAAAG ACCCAAGCCTGCCCAATGTGCAGGTGACCAGGCTGACACTCCTG
chr9 chr16 119976666 332069 31m332025F45M                                                                                                                                                                                                                                            GGGGAAGAGAAGGTGGACTTTGAGAAGAAAG ACCCAAGCCTGCCCAATGTGCAGGTGACCAGGCTGACACTCCTGT
chr9 chr16 119976665 332070 30m332025F46M                                                                                                                                                                                                                                             GGGAAGAGAAGGTGGACTTTGAGAAGAAAG ACCCAAGCCTGCCCAATGTGCAGGTGACCAGGCTGACACTCCTGTC
chr9 chr16 119976665 332070 30m332025F46M                                                                                                                                                                                                                                             GGGAAGAGAAGGTGGACTTTGAGAAGAAAG ACCCAAGCCTGCCCAATGTGCAGGTGACCAGGCTGACACTCCTGTC
chr9 chr16 119976664 332071 29m332025F47M                                                                                                                                                                                                                                              GGAAGAGAAGGTGGACTTTGAGAAGAAAG ACCCAAGCCTGCCCAATGTGCAGGTGACCAGGCTGACACTCCTGTCG
chr9 chr16 119976664 332071 29m332025F47M                                                                                                                                                                                                                                              GGAAGAGAAGGTGGACTTTGAGAAGAAAG ACCCAAGCCTGCCCAATGTGCAGGTGACCAGGCTGACACTCCTGTCG
chr9 chr16 119976664 332071 29m332025F47M                                                                                                                                                                                                                                              GGAAGAGAAGGTGGACTTTGAGAAGAAAG ACCCAAGCCTGCCCAATGTGCAGGTGACCAGGCTGACACTCCTGTCG
chr9 chr16 119976662 332073 27m332025F49M                                                                                                                                                                                                                                                AAGAGAAGGTGGACTTTGAGAAGAAAG ACCCAAGCCTGCCCAATGTGCAGGTGACCAGGCTGACACTCCTGTCGGA
chr9 chr16 119976659 332076 24m332025F52M                                                                                                                                                                                                                                                   AGAAGGTGGACTTTGAGAAGAAAG ACCCAAGCCTGCGCAATGTGCAGGTGACCAGGCTGACACTCCTGTCGGAACA
chr9 chr16 119976659 332076 24m332025F52M                                                                                                                                                                                                                                                   AGAAGGTGGACTTTGAGAAGAAAG ACCCAAGCCTGCCCAATGTGCAGGTGACCAGGCTGACACTCCTGTCGGAACA
chr9 chr16 119976658 332077 23m332025F53M                                                                                                                                                                                                                                                    GAAGGTGGACTTTGAGAAGAAAC ACCCAAGCCTGCCCAATGTGCAGGTGACCAGGCTGACACCCCTGTCGGAACAG
chr9 chr16 119976655 332080 20m332025F56M                                                                                                                                                                                                                                                       AGCCTGTTCCGACAGGAGTG TCAGCCTGGTCACCTGCACATTGGGCAGGCTTGGGTCTTTCTTCTCAAAGTCCACC
chr9 chr16 119976654 332081 19m332025F57M                                                                                                                                                                                                                                                        GAGCCTGTTCCGACAGGAG TGTCAGCCTGGTCACCTGCACATTGGGCAGGCTTGGGTCTTTCTTCTCAAAGTCCAC
chr9 chr16 119976654 332081 19m332025F57M                                                                                                                                                                                                                                                        GAGCCTGTTCCGACAGGAG TGTCAGCCTGGTCACCTGCACATTGGGCAGGCTTGGGTCTTTCTTCTCAAAGTCCAC
chr9 chr16 119976654 332081 19m332025F57M                                                                                                                                                                                                                                                        GAGCCTGTTCCGACAGGAG TGTCAGCCTGGTCACCTGCACATTGGGCAGGCTTGGGTCTTTCTTCTCAAAGTCCAC
chr9 chr16 119976654 332081 19m332025F57M                                                                                                                                                                                                                                                        GAGCCTGTTCCGACAGGAG TGTCAGCCTGGTCACCTGCACATTGGGCAGGCTTGGGTCTTTCTTCTCAAAGTCCAC
chr9 chr16 119976654 332081 19m332025F57M                                                                                                                                                                                                                                                        GAGCCTGTTCCGACAGGAG TGTCAGCCTGGTCACCTGCACATTGGGCAGGCTTGGGTCTTTCTTCTCAAAGTCCAC
chr9 chr16 119976653 332082 18m332025F58M                                                                                                                                                                                                                                                         GGAGCCTGTTCCGACAGG AGTGTCAGCCTGGTCACCTGCACATTGGGCAGGCTTGGGTCTTTCTTCTCAAAGTCCA
chr9 chr16 119976653 332082 18m332025F58M                                                                                                                                                                                                                                                         TGGACTTTGAGAAGAAAG ACCCAAGCCTGCCCAATGTGCAGGTGACCAGGCTGACACTCCTGTCGGAACAGGCTCC
chr9 chr16 119976653 332082 18m332025F58M                                                                                                                                                                                                                                                         GGGACTTTGAGAAGAAAG CCCCAAGCCTGCCCAATGTGCAGGTGACCAGGCTGACACTCCTGTCGGAACAGGCTCC
chr9 chr16 119976653 332082 18m332025F58M                                                                                                                                                                                                                                                         TGGACTTTGAGAAGAAAG ACCCAAGCCTGCCCAATGTGCAGGTGACCAGGCTGACACTCCTGTCGGAACAGGCTCC
chr9 chr16 119976652 332083 17m332025F59M                                                                                                                                                                                                                                                          CGGAGCCTGTTCCGACA GGAGTGTCAGCCTGGTCACCTGCACATTGGGCAGGCTTGGGTCTTTCTTCTCAAAGTCC
chr9 chr16 119976651 332084 16m332025F60M                                                                                                                                                                                                                                                           GACTTTGAGAAGAAAG ACCCAAGCCTGCCCAATGTGCAGGTGACCAGGCTGACACTCCTGTCGGAACAGGCCCCGG
chr9 chr16 119976650 332085 15m332025F61M                                                                                                                                                                                                                                                            ACTTTGAGAAGAAAG ACCCAAGCCTGCCCAATGTGCAGGTGACCAGGCTGACACTCCTGTCGGAACAGGCTCCGGG
chr9 chr16 119976649 332086 14m332025F62M                                                                                                                                                                                                                                                             CCCCGGAGCCTGTT CCGACAGGAGTGTCAGCCTGGTCACCTGCACATTGGGCAGGCTTGGGTCTTTCTTCTCAAAG
chr9 chr16 119976649 332086 14m332025F62M                                                                                                                                                                                                                                                             CTTTGAGAAGAAAG ACCCAAGCCTGCCCAATGTGCAGGTGACCAGGCTGACACTCCTGTCGGAACAGGCTCCGGGG
chr9 chr16 119976649 332086 14m332025F62M                                                                                                                                                                                                                                                             CCCCGGAGCCTGTT CCGACAGGAGTGTCAGCCTGGTCACCTGCACATTGGGCAGGCTTGGGTCTTTCTTCTCAAAG
chr9 chr16 119976648 332087 13m332025F63M                                                                                                                                                                                                                                                              GCCCCGGAGCCTG TTCCGACAGGAGTGTCAGCCTGGTCACCTGCACATTGGGCAGGCTTGGGTCTTTCTTCTCAAA
chr9 chr16 119976648 332087 13m332025F63M                                                                                                                                                                                                                                                              GCCCCGGAGCCTG TTCCGACAGGAGTGTCAGCCTGGTCACCTGCACATTGGGCAGGCTTGGGTCTTTCTTCTCAAA
chr9 chr16 119976646 332089 11m332025F65M                                                                                                                                                                                                                                                                GGGCCCCGGAG CCTGTTCCGACAGGAGTGTCAGCCTGGTCACCTGCACATTGGGCAGGCTTGGGTCTTTCTTCTCA
chr9 chr16 119976645 332090 10m332025F66M                                                                                                                                                                                                                                                                 GAGAAGAAAG ACCCAAGCCTGCCCAATGTGCAGGTGACCAGGCTGACACTCCTGTCGGAACAGGCTCCGGGGCCCG
chr9 chr16 119976643 332092 8m332025F68M                                                                                                                                                                                                                                                                    GACGGGCC CCGGAGCCTGTTCCGACAGGAGTGTCAGCCTGGTCACCTGCACATTGGGCAGGCTTGGGTCTTTCTTC
chr9 chr16 119976642 332093 7m332025F69M                                                                                                                                                                                                                                                                     AAGAAAG ACCCAAGCCTGCCCAATGTGCAGGTGACCAGGCTGACACTCCTGTCGGAACAGGCTCCGGGGCCCGTCG
chr9 chr16 119976642 332093 7m332025F69M                                                                                                                                                                                                                                                                     CGACGGG CCCCGGAGCCTGTTCCGACAGGAGTGTCAGCCTGGTCACCTGCACATTGGGCAGGCTTGGGTCTTTCTT
chr9 chr16 119976640 332095 5m332025F71M                                                                                                                                                                                                                                                                       GACGA CGGGCCCCGGAGCCTGTTCCGACAGGAGTGTCAGCCTGGTCACCTGCACATTGGGCAGGCTTGGGTCTTTC
chr9 chr16 119976640 332095 5m332025F71M                                                                                                                                                                                                                                                                       GACGA CGGGCCCCGGAGCCTGTTCCGACAGGAGTGTCAGCCTGGTCACCTGCACATTGGGCAGGCTTGGGTCTTTC
chr9 chr16 119976640 332095 5m332025F71M                                                                                                                                                                                                                                                                       GACGA CGGGCCCCGGAGCCTGTTCCGACAGGAGTGTCAGCCTGGTCACCTGCACATTGGGCAGGCTTGGGTCTTTC
chr9 chr16 119976640 332095 5m332025F71M                                                                                                                                                                                                                                                                       GACGA CGGGCCCCGGAGCCTGTTCCGACAGGAGTGTCAGCCTGGTCACCTGCACATTGGGCAGGCTTGGGTCTTTC
chr9 chr16 119976640 332095 5m332025F71M                                                                                                                                                                                                                                                                       GAAAG ACCCAAGCCTGCCCAATGTGCAGGTGACCAGGCTGACACTCCTGTCGGAACAGGCTCCGGGGCCCGTCGTC
chr16 chr16 332021 332097 76M                                                                                                                                                                                                                                                                                     AAGACCCAAGCCTGCCCAATGTGCAGGGGACCAGGCTGACACTCCTGTCGGAACAGGCTCCGGGGCCCGTCGTCAT
chr16 chr16 332024 332100 76M                                                                                                                                                                                                                                                                                        ACCCAAGCCTGCCCAATGTGCAGGTGACCAGGCTGACACTCCTGTCGGAACAGGCTCCGGGGCCCGTCGTCATGGA
chr16 chr16 332027 332103 76M                                                                                                                                                                                                                                                                                           CAAGCCTGCCCAATGTGCAGGTGACCAGGCTGACACTCCTGTCGGAACAGGCTCCGGGGCCCGTCGTCATGGATCT
chr16 chr16 332030 332106 76M                                                                                                                                                                                                                                                                                              GCCTGCCCAATGTGCAGGTGACCAGGCTGACACTCCTGTCGGAACAGGCTCCGGGGCCCGTCGTCATGGATCTCAA
chr16 chr16 332033 332109 76M                                                                                                                                                                                                                                                                                                 TGCCCAATGTGCAGGTGACCAGGCTGACACTCCTGTCGGAACAGGCTCCGGGGCCCGTCGTCATGGATCTCACAGA
chr16 chr16 332036 332112 76M                                                                                                                                                                                                                                                                                                    CCAATGTGCAGGTGACCAGGCTGACACTCCTGTCGGAACAGGCTCCGGGGCCCGTCGTCATGGATCTCACAGGGGA
chr16 chr16 332040 332304 68M188N8M                                                                                                                                                                                                                                                                                                  TGTGCAGGTGACCAGGCTGACACTCCTGTCGGAACAGGCTCCGGGGCCCGTCGTCATGGATCTCACAG||||||||||||||||||||||||||||||||||||||||||||||||||||||||||||||||||||||||||||||||||||||||||||||||||||||||||||||||||||||||||||||||||||||||||||||||||||||||||||||||||||||||||||||||||||||||||||GGGACCTG
chr16 chr16 332043 332307 65M188N11M                                                                                                                                                                                                                                                                                                    GCAGGTGACCAGGCTGACACTCCTGTCGGAACAGGCTCCGGGGCCCGTCGTCATGGATCTCACAG||||||||||||||||||||||||||||||||||||||||||||||||||||||||||||||||||||||||||||||||||||||||||||||||||||||||||||||||||||||||||||||||||||||||||||||||||||||||||||||||||||||||||||||||||||||||||||GGGACCTGGCA
chr16 chr16 332046 332310 62M188N14M                                                                                                                                                                                                                                                                                                       GGTGACCAGGCTGACACTCCTGTCGGAACAGGCTCCGGGGCCCGTCGTCATGGATCTCACAG||||||||||||||||||||||||||||||||||||||||||||||||||||||||||||||||||||||||||||||||||||||||||||||||||||||||||||||||||||||||||||||||||||||||||||||||||||||||||||||||||||||||||||||||||||||||||||GGGACCTGGCTGTT
chr16 chr16 332049 332313 59M188N17M                                                                                                                                                                                                                                                                                                          GACCAGGCTGACACTCCTGTCGGAACAGGCTCCGGGGCCCGTCGTCATGGATCTCACAG||||||||||||||||||||||||||||||||||||||||||||||||||||||||||||||||||||||||||||||||||||||||||||||||||||||||||||||||||||||||||||||||||||||||||||||||||||||||||||||||||||||||||||||||||||||||||||GGGACCTGGCTGTTCTG
chr16 chr16 332052 332316 56M188N20M                                                                                                                                                                                                                                                                                                             CAGGCTGACACTCCTGTCGGAACAGGCTCCGGGGCCCGTCGTCATGGATCTCACAG||||||||||||||||||||||||||||||||||||||||||||||||||||||||||||||||||||||||||||||||||||||||||||||||||||||||||||||||||||||||||||||||||||||||||||||||||||||||||||||||||||||||||||||||||||||||||||GGGACCTGGCTGTTCTGAAG
chr16 chr16 332055 332319 53M188N23M                                                                                                                                                                                                                                                                                                                GCTGACACTCCTGTCGGAACAGGCTCCGGGGCCCGTCGTCATGGATCTCACAG||||||||||||||||||||||||||||||||||||||||||||||||||||||||||||||||||||||||||||||||||||||||||||||||||||||||||||||||||||||||||||||||||||||||||||||||||||||||||||||||||||||||||||||||||||||||||||GGGACCTGGCTGTTCTGAAGGAC
chr16 chr16 332058 332322 50M188N26M                                                                                                                                                                                                                                                                                                                   GACACTCCTGTCGGAACAGGCTCCGGGGCCCGTCGTCATGGATCTCACAG||||||||||||||||||||||||||||||||||||||||||||||||||||||||||||||||||||||||||||||||||||||||||||||||||||||||||||||||||||||||||||||||||||||||||||||||||||||||||||||||||||||||||||||||||||||||||||GGGACCTGGCTGTTCTGAAGGACCAG
chr16 chr16 332062 332326 46M188N30M                                                                                                                                                                                                                                                                                                                       CTCCTGTCGGAACAGGCTCCGGGGCCCGTCGTCATGGATCTCACAG||||||||||||||||||||||||||||||||||||||||||||||||||||||||||||||||||||||||||||||||||||||||||||||||||||||||||||||||||||||||||||||||||||||||||||||||||||||||||||||||||||||||||||||||||||||||||||GGGACCTGGCTGTTCTGAAGGACCAG
chr16 chr16 332065 332329 43M188N33M                                                                                                                                                                                                                                                                                                                          CTGTCGGAACAGGCTCCGGGGCCCGTCGTCATGGATCTCACAG||||||||||||||||||||||||||||||||||||||||||||||||||||||||||||||||||||||||||||||||||||||||||||||||||||||||||||||||||||||||||||||||||||||||||||||||||||||||||||||||||||||||||||||||||||||||||||GGGACCTGGCTGTTCTGAAGGACCAG
chr16 chr16 332068 332332 40M188N36M                                                                                                                                                                                                                                                                                                                             TCGGAACAGGCTCCGGGGCCCGTCGTCATGGATCTCACAG||||||||||||||||||||||||||||||||||||||||||||||||||||||||||||||||||||||||||||||||||||||||||||||||||||||||||||||||||||||||||||||||||||||||||||||||||||||||||||||||||||||||||||||||||||||||||||GGGACCTGGCTGTTCTGAAGGACCAG
chr16 chr16 332072 332336 36M188N40M                                                                                                                                                                                                                                                                                                                                 AACAGGCTCCGGGGCCCGTCGTCATGGATCTCACAG||||||||||||||||||||||||||||||||||||||||||||||||||||||||||||||||||||||||||||||||||||||||||||||||||||||||||||||||||||||||||||||||||||||||||||||||||||||||||||||||||||||||||||||||||||||||||||GGGACCTGGCTGTTCTGAAGGACCAG
chr16 chr16 332076 332340 32M188N44M                                                                                                                                                                                                                                                                                                                                     GGCTCCGGGGCCCGTCGTCATGGATCTCACAG||||||||||||||||||||||||||||||||||||||||||||||||||||||||||||||||||||||||||||||||||||||||||||||||||||||||||||||||||||||||||||||||||||||||||||||||||||||||||||||||||||||||||||||||||||||||||||GGGACCTGGCTGTTCTGAAGGACCAG
chr16 chr16 332079 332343 29M188N47M                                                                                                                                                                                                                                                                                                                                        TCCGGGGCCCGTCGTCATGGATCTCACAG||||||||||||||||||||||||||||||||||||||||||||||||||||||||||||||||||||||||||||||||||||||||||||||||||||||||||||||||||||||||||||||||||||||||||||||||||||||||||||||||||||||||||||||||||||||||||||GGGACCTGGCTGTTCTGAAGGACCAG
chr16 chr16 332082 332346 26M188N50M                                                                                                                                                                                                                                                                                                                                           GGGGCCCGTCGTCATGGATCTCACAG||||||||||||||||||||||||||||||||||||||||||||||||||||||||||||||||||||||||||||||||||||||||||||||||||||||||||||||||||||||||||||||||||||||||||||||||||||||||||||||||||||||||||||||||||||||||||||GGGACCTGGCTGTTCTGAAGGACCAG
chr16 chr16 332085 332349 23M188N53M                                                                                                                                                                                                                                                                                                                                              GCCCGTCGTCATGGATCTCACAG||||||||||||||||||||||||||||||||||||||||||||||||||||||||||||||||||||||||||||||||||||||||||||||||||||||||||||||||||||||||||||||||||||||||||||||||||||||||||||||||||||||||||||||||||||||||||||GGGATCTGGCTGTTCTGAAGGACCAG
chr16 chr16 332088 332352 20M188N56M                                                                                                                                                                                                                                                                                                                                                 CGTCGTCATGGATCTCACAG||||||||||||||||||||||||||||||||||||||||||||||||||||||||||||||||||||||||||||||||||||||||||||||||||||||||||||||||||||||||||||||||||||||||||||||||||||||||||||||||||||||||||||||||||||||||||||GGGACCTGGCTGTTCTGAAGGACCAG
chr16 chr16 332091 332355 17M188N59M                                                                                                                                                                                                                                                                                                                                                    CGTCATGGATCTCACAG||||||||||||||||||||||||||||||||||||||||||||||||||||||||||||||||||||||||||||||||||||||||||||||||||||||||||||||||||||||||||||||||||||||||||||||||||||||||||||||||||||||||||||||||||||||||||||GGGACCTGGCTGTTCTGAATGACCAG
chr16 chr16 332094 332358 14M188N62M                                                                                                                                                                                                                                                                                                                                                       CATGGATCTCACAG||||||||||||||||||||||||||||||||||||||||||||||||||||||||||||||||||||||||||||||||||||||||||||||||||||||||||||||||||||||||||||||||||||||||||||||||||||||||||||||||||||||||||||||||||||||||||||GGGACCTGGCTGTTCTGAAGGACCAG
chr16 chr16 332097 332361 11M188N65M                                                                                                                                                                                                                                                                                                                                                          GGATCTCACAG||||||||||||||||||||||||||||||||||||||||||||||||||||||||||||||||||||||||||||||||||||||||||||||||||||||||||||||||||||||||||||||||||||||||||||||||||||||||||||||||||||||||||||||||||||||||||||GGGACCTGGCTGTTCTGAAGGACCAG
chr16 chr16 332100 332364 8M188N68M                                                                                                                                                                                                                                                                                                                                                              TCTCACAG||||||||||||||||||||||||||||||||||||||||||||||||||||||||||||||||||||||||||||||||||||||||||||||||||||||||||||||||||||||||||||||||||||||||||||||||||||||||||||||||||||||||||||||||||||||||||||GGGACCTGGCTGTTCTGAAGGACCAG
chr16 chr16 332103 332367 5M188N71M                                                                                                                                                                                                                                                                                                                                                                 CACAG||||||||||||||||||||||||||||||||||||||||||||||||||||||||||||||||||||||||||||||||||||||||||||||||||||||||||||||||||||||||||||||||||||||||||||||||||||||||||||||||||||||||||||||||||||||||||||GGGACCTGGCTGTTCGGAAGGACCAG
chr16 chr16 332223 332299 76M                                                                                                                                                                                                                                                                                                                                                                                                                                                                                               CCTACGTGGGGGCTCCTGGAGCAGGTCTCAGCCTGTAGGGGAGTTCCGACCCTAGCTGAGGTTTCCCCAATAGGGG
chr16 chr16 332291 332367 76M                                                                                                                                                                                                                                                                                                                                                                                                                                                                                                                                                                   CACAGGGGACCTGGCTGTTCTGAAGGACCAG
chr16 chr16 332294 332370 76M                                                                                                                                                                                                                                                                                                                                                                                                                                                                                                                                                                      AGGGGACCTGGCTGTTCTGAAGGACCAG
chr16 chr16 332297 332373 76M                                                                                                                                                                                                                                                                                                                                                                                                                                                                                                                                                                         GGATCTGGCTGTTCTGAAGGACCAG
chr16 chr16 332300 332376 76M                                                                                                                                                                                                                                                                                                                                                                                                                                                                                                                                                                            CCTGGCTGTTCTGAAGGACCAG
chr16 chr16 332303 332472 70M93N6M                                                                                                                                                                                                                                                                                                                                                                                                                                                                                                                                                                          GGCTGTTCTGAAGGACCAG
chr16 chr16 332307 332476 66M93N10M                                                                                                                                                                                                                                                                                                                                                                                                                                                                                                                                                                             GTTCTGAAGGACCAG
chr16 chr16 332310 332479 63M93N13M                                                                                                                                                                                                                                                                                                                                                                                                                                                                                                                                                                                CTGAAGGACCAG
chr16 chr16 332313 332482 60M93N16M                                                                                                                                                                                                                                                                                                                                                                                                                                                                                                                                                                                   AAGGACCAG
chr16 chr16 332316 332485 57M93N19M                                                                                                                                                                                                                                                                                                                                                                                                                                                                                                                                                                                      GACCAG
chr16 chr16 332319 332488 54M93N22M                                                                                                                                                                                                                                                                                                                                                                                                                                                                                                                                                                                         CAG
chr16 chr16 332322 332491 51M93N25M                                                                                                                                                                                                                                                                                                                                                                                                                                                                                                                                                                                             
    24 pairs 
 -1:2
-2:23
45:0
46:6
46:6
46:10
46:18
28:44
6:67
28:61
86:8
80:18
84:19
60:66
13:-235
31:-234
1:-273
114:-209
46:300
78:268
87:267
216:-249
146:328
95:500 
     TCGA-02-2483-01A chr10-chr10 103336915 128799871 rr 
 
5 27 4 15 55 54
AGCTAATGGAGGGATTTCTGAGTCTTGAGAAAGGCTGCCCAGTTTGGAGG CGGAGCACAGAGGACTTTTAGGATGGTGAAGCTACCCAGTGAGAGACTAC
11222222223333333333444444445555555555555555555555 55555555555555555555544444443333333322222222221111
ENSG00000166171 intron1(103330506-103339814) DOCK1 intron10(128798571-128806993)
 
 blast search - human genome  
 left 
 >ref|NT_030059.13| Homo sapiens chromosome 10 genomic contig, GRCh37.p9 Primary 
Assembly
 gb|GL000099.1| Homo sapiens chromosome 10 genomic contig, GRCh37 reference primary 
assembly
Length=79420533

 Score = 93.5 bits (50),  Expect = 5e-17
 Identities = 50/50 (100%), Gaps = 0/50 (0%)
 Strand=Plus/Minus

Query  1         AGCTAATGGAGGGATTTCTGAGTCTTGAGAAAGGCTGCCCAGTTTGGAGG  50
                 ||||||||||||||||||||||||||||||||||||||||||||||||||
Sbjct  54141429  AGCTAATGGAGGGATTTCTGAGTCTTGAGAAAGGCTGCCCAGTTTGGAGG  54141380


>ref|NW_001838006.2| Homo sapiens chromosome 10 genomic contig, alternate assembly 
HuRef SCAF_1103279188397, whole genome shotgun sequence
 gb|DS486042.1| Homo sapiens SCAF_1103279188397 genomic scaffold, whole genome 
shotgun sequence
Length=24511762

 Score = 93.5 bits (50),  Expect = 5e-17
 Identities = 50/50 (100%), Gaps = 0/50 (0%)
 Strand=Plus/Plus

Query  1         AGCTAATGGAGGGATTTCTGAGTCTTGAGAAAGGCTGCCCAGTTTGGAGG  50
                 ||||||||||||||||||||||||||||||||||||||||||||||||||
Sbjct  21664122  AGCTAATGGAGGGATTTCTGAGTCTTGAGAAAGGCTGCCCAGTTTGGAGG  21664171


>ref|NC_000010.10| Homo sapiens chromosome 10, GRCh37.p9 Primary Assembly
 gb|CM000672.1| Homo sapiens chromosome 10, GRCh37 primary reference assembly
Length=135534747

 Score = 93.5 bits (50),  Expect = 5e-17
 Identities = 50/50 (100%), Gaps = 0/50 (0%)
 Strand=Plus/Minus

Query  1          AGCTAATGGAGGGATTTCTGAGTCTTGAGAAAGGCTGCCCAGTTTGGAGG  50
                  ||||||||||||||||||||||||||||||||||||||||||||||||||
Sbjct  103336965  AGCTAATGGAGGGATTTCTGAGTCTTGAGAAAGGCTGCCCAGTTTGGAGG  103336916


>ref|AC_000142.1| Homo sapiens chromosome 10, alternate assembly HuRef, whole genome 
shotgun sequence
 gb|CM000471.1| Homo sapiens chromosome 10, whole genome shotgun sequence
Length=128985118

 Score = 93.5 bits (50),  Expect = 5e-17
 Identities = 50/50 (100%), Gaps = 0/50 (0%)
 Strand=Plus/Minus

Query  1         AGCTAATGGAGGGATTTCTGAGTCTTGAGAAAGGCTGCCCAGTTTGGAGG  50
                 ||||||||||||||||||||||||||||||||||||||||||||||||||
Sbjct  96967562  AGCTAATGGAGGGATTTCTGAGTCTTGAGAAAGGCTGCCCAGTTTGGAGG  96967513


>gb|CM000261.1| Homo sapiens chromosome 10, whole genome shotgun sequence
Length=129189614

 Score = 93.5 bits (50),  Expect = 5e-17
 Identities = 50/50 (100%), Gaps = 0/50 (0%)
 Strand=Plus/Minus

Query  1         AGCTAATGGAGGGATTTCTGAGTCTTGAGAAAGGCTGCCCAGTTTGGAGG  50
                 ||||||||||||||||||||||||||||||||||||||||||||||||||
Sbjct  97075751  AGCTAATGGAGGGATTTCTGAGTCTTGAGAAAGGCTGCCCAGTTTGGAGG  97075702


Lambda     K      H
    1.33    0.621     1.12 

Gapped
Lambda     K      H
    1.28    0.460    0.850 

Effective search space used: 681780354108
 
 right 
 >ref|NT_008818.16| Homo sapiens chromosome 10 genomic contig, GRCh37.p9 Primary 
Assembly
 gb|GL000100.1| Homo sapiens chromosome 10 genomic contig, GRCh37 reference primary 
assembly
Length=6758678

 Score = 93.5 bits (50),  Expect = 5e-17
 Identities = 50/50 (100%), Gaps = 0/50 (0%)
 Strand=Plus/Minus

Query  1      CGGAGCACAGAGGACTTTTAGGATGGTGAAGCTACCCAGTGAGAGACTAC  50
              ||||||||||||||||||||||||||||||||||||||||||||||||||
Sbjct  33803  CGGAGCACAGAGGACTTTTAGGATGGTGAAGCTACCCAGTGAGAGACTAC  33754


>ref|NW_001838011.1| Homo sapiens chromosome 10 genomic contig, alternate assembly 
HuRef SCAF_1103279183978, whole genome shotgun sequence
 gb|DS486180.1| Homo sapiens SCAF_1103279183978 genomic scaffold, whole genome 
shotgun sequence
Length=3840288

 Score = 93.5 bits (50),  Expect = 5e-17
 Identities = 50/50 (100%), Gaps = 0/50 (0%)
 Strand=Plus/Minus

Query  1      CGGAGCACAGAGGACTTTTAGGATGGTGAAGCTACCCAGTGAGAGACTAC  50
              ||||||||||||||||||||||||||||||||||||||||||||||||||
Sbjct  48056  CGGAGCACAGAGGACTTTTAGGATGGTGAAGCTACCCAGTGAGAGACTAC  48007


>ref|NC_000010.10| Homo sapiens chromosome 10, GRCh37.p9 Primary Assembly
 gb|CM000672.1| Homo sapiens chromosome 10, GRCh37 primary reference assembly
Length=135534747

 Score = 93.5 bits (50),  Expect = 5e-17
 Identities = 50/50 (100%), Gaps = 0/50 (0%)
 Strand=Plus/Minus

Query  1          CGGAGCACAGAGGACTTTTAGGATGGTGAAGCTACCCAGTGAGAGACTAC  50
                  ||||||||||||||||||||||||||||||||||||||||||||||||||
Sbjct  128799872  CGGAGCACAGAGGACTTTTAGGATGGTGAAGCTACCCAGTGAGAGACTAC  128799823


>ref|AC_000142.1| Homo sapiens chromosome 10, alternate assembly HuRef, whole genome 
shotgun sequence
 gb|CM000471.1| Homo sapiens chromosome 10, whole genome shotgun sequence
Length=128985118

 Score = 93.5 bits (50),  Expect = 5e-17
 Identities = 50/50 (100%), Gaps = 0/50 (0%)
 Strand=Plus/Minus

Query  1          CGGAGCACAGAGGACTTTTAGGATGGTGAAGCTACCCAGTGAGAGACTAC  50
                  ||||||||||||||||||||||||||||||||||||||||||||||||||
Sbjct  122371665  CGGAGCACAGAGGACTTTTAGGATGGTGAAGCTACCCAGTGAGAGACTAC  122371616


>gb|CM000261.1| Homo sapiens chromosome 10, whole genome shotgun sequence
Length=129189614

 Score = 93.5 bits (50),  Expect = 5e-17
 Identities = 50/50 (100%), Gaps = 0/50 (0%)
 Strand=Plus/Minus

Query  1          CGGAGCACAGAGGACTTTTAGGATGGTGAAGCTACCCAGTGAGAGACTAC  50
                  ||||||||||||||||||||||||||||||||||||||||||||||||||
Sbjct  122470049  CGGAGCACAGAGGACTTTTAGGATGGTGAAGCTACCCAGTGAGAGACTAC  122470000


Lambda     K      H
    1.33    0.621     1.12 

Gapped
Lambda     K      H
    1.28    0.460    0.850 

Effective search space used: 681780354108
 
 blast search - nt  
 left 
 >ref|NG_017063.1| Homo sapiens polymerase (DNA directed), lambda (POLL), RefSeqGene 
on chromosome 10
Length=16389

 Score = 93.5 bits (50),  Expect = 6e-17
 Identities = 50/50 (100%), Gaps = 0/50 (0%)
 Strand=Plus/Plus

Query  1      AGCTAATGGAGGGATTTCTGAGTCTTGAGAAAGGCTGCCCAGTTTGGAGG  50
              ||||||||||||||||||||||||||||||||||||||||||||||||||
Sbjct  16063  AGCTAATGGAGGGATTTCTGAGTCTTGAGAAAGGCTGCCCAGTTTGGAGG  16112


>dbj|AK128521.1| Homo sapiens cDNA FLJ46679 fis, clone TRACH3010331, highly  similar 
to Homo sapiens polymerase (DNA directed), lambda (POLL)
Length=4128

 Score = 93.5 bits (50),  Expect = 6e-17
 Identities = 50/50 (100%), Gaps = 0/50 (0%)
 Strand=Plus/Plus

Query  1     AGCTAATGGAGGGATTTCTGAGTCTTGAGAAAGGCTGCCCAGTTTGGAGG  50
             ||||||||||||||||||||||||||||||||||||||||||||||||||
Sbjct  3252  AGCTAATGGAGGGATTTCTGAGTCTTGAGAAAGGCTGCCCAGTTTGGAGG  3301


>emb|AL627424.8| Human DNA sequence from clone RP11-529I10 on chromosome 10 Contains 
the 3' end of the BTRC gene for beta-transducin repeat 
containing, the POLL gene for polymerase (DNA directed) lambda, 
a novel gene (DKFZP566F084), the 3' end of the SHFM3 gene 
for split hand/foot malformation (ectrodactyly) type 3 and 
three CpG islands, complete sequence
Length=108503

 Score = 93.5 bits (50),  Expect = 6e-17
 Identities = 50/50 (100%), Gaps = 0/50 (0%)
 Strand=Plus/Minus

Query  1      AGCTAATGGAGGGATTTCTGAGTCTTGAGAAAGGCTGCCCAGTTTGGAGG  50
              ||||||||||||||||||||||||||||||||||||||||||||||||||
Sbjct  46865  AGCTAATGGAGGGATTTCTGAGTCTTGAGAAAGGCTGCCCAGTTTGGAGG  46816


Lambda     K      H
    1.33    0.621     1.12 

Gapped
Lambda     K      H
    1.28    0.460    0.850 

Effective search space used: 874911716973
 
 right 
 
 
    reads 
 chr10 chr10 103340063 103337131 60m2856n16m                  ||||||||||||||||||||||||||||||||||||||||||||||||||||||||||||||||||||GAGACTCAGAATTCAG
chr10 chr10 103340056 103337124 53m2856n23m                  ||||||||||||||||||||||||||||||||||||||||||||||||||||||||||||||||||||GAGATTCAGAATTCAGAGACTGC
chr10 chr10 103340010 103337078 7m2856n69m                   ||||||||||||||||||||||||||||||||||||||||||||||||||||||||||||||||||||GAGACTCAGAATTCAGAGACTGCCTTGAAACCATGCAGACTCTTGGTCCCACCTCCTGGCCAGGGCAAC
chr10 chr10 103339789 103337002 35m2711n41m                  ||||||||||||||||||||||||||||||||||||||||||||||||||||||||||||||||||||||||||||||||||||||||||||||||||||||||||||||||||||||||||||||||||||||||||||||||||||||||||||||||||||||||||||CTCCAGTGACCCATCTGGGTGTCCCTTCAGCTGCCCAGTCT
chr10 chr10 103339338 103337105 34m2157n42m                  ||||||||||||||||||||||||||||||||||||||||||||||||||||||||||||||||||||GAGACTCAGAATTCAGAGACTGCCTTGAAACCATGCAGACTN
chr10 chr10 103337272 103337196 76m                          TGTCTGAAATCAGGGTCTG
chr10 chr10 103337259 103337183 76m                          TGTCTGAAATCAGGGTCTGACCTCCATTCTTG
chr10 chr10 103337248 103337172 76m                          TGTCTGAAATCAGGGTCTGACCTCCATTCTTGACATCCCTCCC
chr10 chr10 103337232 103337156 76m                          TGTCTGAAATCAGGGTCTGACCTCCATTCTTGACATCCCTCCCCATCTGCCTTAACTCC
chr10 chr10 103337227 103337151 76m                          TGTCTGAAATCAGGGTCTGACCTCCATTCTTGACATCCCTCCCCATCTGCCTTAACTCCTTCCC
chr10 chr10 103337224 103337148 76m                          TGTCTGAAATCAGGGTCTGACCTCCATTCTTGACATCCCTCCCCATCTGCCTTAACTCCTTCCCCCA
chr10 chr10 103337220 103337144 76m                          TGTCTGAAATCAGGGTCTGACCTCCATTCTTGACATCCCTCCCCATCTGCCTTAACTCCTTCCCCCAGGAG
chr10 chr10 103337193 103337117 76m                                                TCCATTCTTGACATCCCTCCCCATCTGCCTTAACTCCTTCCCCCAGGAGACTCAGAATTCAGAGACTGCCTTGAAA
chr10 chr10 103337176 103337100 76m                                                                 TCCCCATCTGCCTTAACTCCTTCCCCCAGGAGACTCAGAATTCAGAGACTGCCTTGAAACCATGCAGACTCTTGGT
chr10 chr10 103337167 103337091 76m                                                                          GCCTTAACTCCTTCCCCCAGGAGACTCAGAATTCAGAGACTGCCTTGAAACCATGCAGACTCTTGGTCCCACCTCC
chr10 chr10 103337141 103337065 76m                                                                                                    CAGAATTCAGAGACTGCCTTGAAACCATGCAGACTCTTGGTCCCACCTCCTGGCCAGGGCAACACAAAGTCGTGGG
chr10 chr10 103337128 103337052 76m                                                                                                                 CTGCCTTGAAACCATGCAGACTCTTGGTCCCACCTCCTGGCCAGGGCAACACAAAGTCGTGGGATAAAGAGGGAGC
chr10 chr10 103337120 103337044 76m                                                                                                                         AAACCATGCAGACTCTTGGTCCCACCTCCTGGCCAGGGCAACACAAAGTCGTGGGATAAAGAGGGAGCATGCCCCA
chr10 chr10 103337113 103337037 76m                                                                                                                                GCAGACTCTTGGTCCCACCTCCTGGCCAGGGCAACACAAAGTCGTGGGATAAAGAGCGAGCATGCCCCACCTCCAG
chr10 chr10 103337100 103337024 76m                                                                                                                                             CCCACCTCCTGGCCAGGGCAACACAAAGTCGTGGGATAAAGAGGGAGCATGCCCCACCTCCAGTGACCCATCTGGG
chr10 chr10 103337095 103337019 76m                                                                                                                                                  CTCCTGGCCAGGGCAACACAAAGTCGTGGGATAAAGAGGGAGCATGCCCCACCTCCAGTGACCCATCTGGGTGTCC
chr10 chr10 103337092 103337016 76m                                                                                                                                                     CTGGCCAGGGCAACACAAAGTCGTGGGATAAAGAGGGAGCATGCCCCACCTCCAGTGACCCATCTGGGTGTCCCTT
chr10 chr10 103337083 103337007 76m                                                                                                                                                              GCAACACAAAGTCGTGGGATAAAGAGGGAGCATGCCCCACCTCCAGTGACCCATCTGGGTGTCCCTTCAGCTGCCC
chr10 chr10 103337079 103337003 76m                                                                                                                                                                  CACAAAGTCGTGGGATAAAGAGGGAGCATGCCCCACCTCCAGTGACCCATCTGGGTGTCCCTTCAGCTGCCCAGTC
chr10 chr10 103337075 103336999 76m                                                                                                                                                                      AAGTCGTGGGATAAAGAGGGAGCATGCCCCACCTCCTGTGACCCATCTGGGTGTCCCTTCAGCTGCCCAGTCTGAC
chr10 chr10 103337068 103336992 76m                                                                                                                                                                             GGGATAAAGAGGGAGCATGCCCCACCTCCAGTGACACATCTGGGTGTCCCTTCAGCTGCCCAGTCTGACACAGAAC
chr10 chr10 103337064 103336988 76m                                                                                                                                                                                 TAAAGAGGGAGCATGCCCCACCTCCAGTGACCCATCTGGGTGTCCCTTCAGCTGCCCAGTCTGACACAGAAGCCCA
chr10 chr10 103337052 103336976 76m                                                                                                                                                                                             ATGCCCCACCTCCAGTGACCCATCTGGGTGTCCCTTCAGCTGCCCAGTCTGACACAGAAGCCCAGGAGAAGAGCCG
chr10 chr10 103337038 103336962 76m                                                                                                                                                                                                           GTGACCCATCTGGGTGTCCCTTCAGCTGCCCAGTCTGACACAGAAGCCCAGGAGAAGAGCCACTGAGGCAGAGGAG
chr10 chr10 103337029 103336953 76m                                                                                                                                                                                                                    CTGGGTGTCCCTTCAGCTGCCCAGTCTGACACAGAAGCCCAGGAGAAGAGCCACTGAGGCAGAGGAGCTAATGGAG
chr10 chr10 103337018 103336942 76m                                                                                                                                                                                                                               TTCAGCTGCCCAGTCTGACACAGAAGCCCAGGAGAAGAGCCACTGAGGCAGAGGAGCTAATGGAGGGATTTCTGAG
chr10 chr10 103336998 103336922 76m                                                                                                                                                                                                                                                   CAGAAGCCCAGGAGAAGAGCCACTGAGGCAGAGGAGCTAATGGAGGGATTTCTGAGTCTTGAGAAAGGCTGCCCAG
chr10 chr10 103336989 103336913 76m                                                                                                                                                                                                                                                            AGGAGAAGAGCCACTGAGGCAGAGGAGCTAATGGAGGGATTTCTGAGTCTTGAGAAAGGCTGCCCAGTTTGGAGGC
chr10 chr10 103336973 128799854 59m128799872F17m                                                                                                                                                                                                                                                               AGGCAGAGGAGCTAATGGAGGGATTTCTGAGTCTTGAGAAAGGCTGCCCAGTTTGGAGG CGGAGCACAGAGGACTT
chr10 chr10 103336969 128799850 55m128799872F21m                                                                                                                                                                                                                                                                   AGAGGAGCTAATGGAGGGATTTCTGAGTCTTGAGAAAGGCTGCCCAGTTTGGAGG CGGAGCACAGAGGACTTTTAG
chr10 chr10 103336962 128799843 48m128799872F28m                                                                                                                                                                                                                                                                          CTAATGGAGGGATTTCTGAGTCTTGAGAAAGGCTGCCCAGTTTGGAGG CGGAGCACAGAGGACTTTTAGGATGGTG
chr10 chr10 103336954 128799835 40m128799872F36m                                                                                                                                                                                                                                                                                  GGGATTTCTGAGTCTTGAGAAAGGCTGCCCAGTTTGGAGG CGGAGCACAGAGGACTTTTAGGATGGTGAAGCTACC
chr10 chr10 103336944 128799825 30m128799872F46m                                                                                                                                                                                                                                                                                            AGTCTTGAGAAAGGCTGCCCAGTTTGGAGG CGGAGCACAGAGGACTTTTAGGATGGTGAAGCTACCCAGTGAGAGA
chr10 chr10 103336936 128799817 22m128799872F54m                                                                                                                                                                                                                                                                                                    GAAAGGCTGCCCAGTTTGGAGG CGGAGCACAGAGGACTTTTAGGATGGTGAAGCTACCCAGTGAGAGACTACAATG
chr10 chr10 103336927 128799808 13m128799872F63m                                                                                                                                                                                                                                                                                                             CCCAGTTTGGAGG CGGAGCACAGAGGACTTTTAGGATGGTGAAGCTACCCAGTGAGAGACTACAATGGTGAATATA
chr10 chr10 103336925 128799806 11m128799872F65m                                                                                                                                                                                                                                                                                                               CAGTTTGGAGG CGGAGCACAGAGGACTTTTAGGATGGTGAAGCTACCCAGTGAGAGACTACAATGGTGAATATACG
chr10 chr10 128799877 128799801 76m                                                                                                                                                                                                                                                                                                                                  CCCAGGCGGAGCACAGAGGACTTTTAGGATGGTGAAGCTACCCAGTGAGAGACTACAATGGTGAATATACGGTATT
chr10 chr10 128799874 128799798 76m                                                                                                                                                                                                                                                                                                                                     TGGCGGAGCACAGAGGACTTTTAGGATGGTGAAGCTACCCAGTGAGAGACTACAATGGTGAATATACGGTATTATT
chr10 chr10 128799871 128799233 54m562n22m                                                                                                                                                                                                                                                                                                                                 CGGAGCACAGAGGACTTTTAGGATGGTGAAGCTACCCAGTGAGAGACTACAATG||||||||||||||||||||||||||||||||||||||||||||||||||||||||||||||||||||||||||||||||||||||||||||||||||||||||||||||||||||||||||||||||||||||||||||||||||||||||||||||||||||||||||||||||||||||||||||||||||||||||||||||||||||||||||||||||||||||||||||||||||||
chr10 chr10 128799867 128799229 50m562n26m                                                                                                                                                                                                                                                                                                                                     GCACAGAGGACTTTTAGGATGGTGAAGCTACCCAGTGAGAGACTACAATG||||||||||||||||||||||||||||||||||||||||||||||||||||||||||||||||||||||||||||||||||||||||||||||||||||||||||||||||||||||||||||||||||||||||||||||||||||||||||||||||||||||||||||||||||||||||||||||||||||||||||||||||||||||||||||||||||||||||||||||||||||
chr10 chr10 128799863 128799787 76m                                                                                                                                                                                                                                                                                                                                                AGAGGACTTTTAGGATGGTGAAGCTACCCAGTGAGAGACTACAATGGTGAATATACGGTATTATTCATTTGTCGAA
chr10 chr10 128799860 128799222 43m562n33m                                                                                                                                                                                                                                                                                                                                            GGACTTTTAGGATGGTGAAGCTACCCAGTGAGAGACTACAATG||||||||||||||||||||||||||||||||||||||||||||||||||||||||||||||||||||||||||||||||||||||||||||||||||||||||||||||||||||||||||||||||||||||||||||||||||||||||||||||||||||||||||||||||||||||||||||||||||||||||||||||||||||||||||||||||||||||||||||||||||||
chr10 chr10 128799857 128799219 40m562n36m                                                                                                                                                                                                                                                                                                                                               CTTTTAGGATGGTGAAGCTACCCAGTGAGAGACTACAATG||||||||||||||||||||||||||||||||||||||||||||||||||||||||||||||||||||||||||||||||||||||||||||||||||||||||||||||||||||||||||||||||||||||||||||||||||||||||||||||||||||||||||||||||||||||||||||||||||||||||||||||||||||||||||||||||||||||||||||||||||||
chr10 chr10 128799854 128799216 37m562n39m                                                                                                                                                                                                                                                                                                                                                  TTAGGATGGTGAAGCTACCCAGTGAGAGACTACAATG||||||||||||||||||||||||||||||||||||||||||||||||||||||||||||||||||||||||||||||||||||||||||||||||||||||||||||||||||||||||||||||||||||||||||||||||||||||||||||||||||||||||||||||||||||||||||||||||||||||||||||||||||||||||||||||||||||||||||||||||||||
chr10 chr10 128799851 128799775 76m                                                                                                                                                                                                                                                                                                                                                            GGATGGTGAAGCTACCCAGTGAGAGACTACAATGGTGAATATACGGTATTATTCATTTGTCGAAACCCACAGAAGG
chr10 chr10 128799848 128799210 31m562n45m                                                                                                                                                                                                                                                                                                                                                        TGGTGAAGCTACCCAGTGAGAGACTACAATG||||||||||||||||||||||||||||||||||||||||||||||||||||||||||||||||||||||||||||||||||||||||||||||||||||||||||||||||||||||||||||||||||||||||||||||||||||||||||||||||||||||||||||||||||||||||||||||||||||||||||||||||||||||||||||||||||||||||||||||||||||
chr10 chr10 128799845 128799769 76m                                                                                                                                                                                                                                                                                                                                                                  TGAAGCTACCCAGTGAGAGACTACAATGGTGAATATACGGTATTATTCATTTGTCGAAACCCACAGAAGGCACAAC
chr10 chr10 128799841 128799203 24m562n52m                                                                                                                                                                                                                                                                                                                                                               GCTACCCAGTGAGAGACTACAATG||||||||||||||||||||||||||||||||||||||||||||||||||||||||||||||||||||||||||||||||||||||||||||||||||||||||||||||||||||||||||||||||||||||||||||||||||||||||||||||||||||||||||||||||||||||||||||||||||||||||||||||||||||||||||||||||||||||||||||||||||||
chr10 chr10 128799838 128799762 76m                                                                                                                                                                                                                                                                                                                                                                         ACCCAGTGAGAGACTACAATGGTGAATATACGGTATTATTCATTTGTCGAAACCCACAGAAGGCACAACATCAAGA
chr10 chr10 128799835 128799197 18m562n58m                                                                                                                                                                                                                                                                                                                                                                     CAGTGAGAGACTACAATG||||||||||||||||||||||||||||||||||||||||||||||||||||||||||||||||||||||||||||||||||||||||||||||||||||||||||||||||||||||||||||||||||||||||||||||||||||||||||||||||||||||||||||||||||||||||||||||||||||||||||||||||||||||||||||||||||||||||||||||||||||
chr10 chr10 128799832 128799756 76m                                                                                                                                                                                                                                                                                                                                                                               TGAGAGACTACAATGGTGAATATACGGTATTATTCATTTGTCGAAACCCACAGAAGGCACAACATCAAGAATGAAC
chr10 chr10 128799829 128799753 76m                                                                                                                                                                                                                                                                                                                                                                                  GAGACTACAATGGTGAATATACGGTATTATTCATTTGTCGAAACCCACAGAAGGCACAACATCAAGAATGAACGCG
chr10 chr10 128799826 128799188 9m562n67m                                                                                                                                                                                                                                                                                                                                                                               ACTGCAATG||||||||||||||||||||||||||||||||||||||||||||||||||||||||||||||||||||||||||||||||||||||||||||||||||||||||||||||||||||||||||||||||||||||||||||||||||||||||||||||||||||||||||||||||||||||||||||||||||||||||||||||||||||||||||||||||||||||||||||||||||||
chr10 chr10 128799823 128799185 6m562n70m                                                                                                                                                                                                                                                                                                                                                                                  ACAATG||||||||||||||||||||||||||||||||||||||||||||||||||||||||||||||||||||||||||||||||||||||||||||||||||||||||||||||||||||||||||||||||||||||||||||||||||||||||||||||||||||||||||||||||||||||||||||||||||||||||||||||||||||||||||||||||||||||||||||||||||||
chr10 chr10 128799820 128799744 76m                                                                                                                                                                                                                                                                                                                                                                                           ATGGTGAATATACGGTATTATTCATTTGTCGAAACCCACAGAAGGCACAACATCAAGAATGAACGCTAATGTAAAC
chr10 chr10 128799817 128799741 76m                                                                                                                                                                                                                                                                                                                                                                                              GTGAATATACGGTATTATTCATTTGTCGAAACCCACAGAAGGCACAACATCAAGAATGAACGCTAATGTAAACTAC
chr10 chr10 128799813 128799737 76m                                                                                                                                                                                                                                                                                                                                                                                                  ATATACGGTATTATTCATTTGTCGAAACCCACAGAAGGCACAACATCAAGAATGAACGCTAATGTAAACTACAGAC
chr10 chr10 128799810 128799734 76m                                                                                                                                                                                                                                                                                                                                                                                                     CACGGTATTATTCATTTGTCGAAACCCACAGAAGGCACAACATCAAGAATGAACGCTAATGTAAACTACAGACTTC
chr10 chr10 128799807 128799731 76m                                                                                                                                                                                                                                                                                                                                                                                                        GGTATTATTCATTTGTCGAAACCCACAGAAGGCACAACATCAAGAATGAACGCTAATGTAAACTACAGACTTCACA
chr10 chr10 128799803 128799727 76m                                                                                                                                                                                                                                                                                                                                                                                                            TTATTCATTTGTCGAAACCCACAGAAGGCACAACATCAAGAATGAACGCTAATGTAAACTACAGACTTCACAGGGC
chr10 chr10 128799800 128799724 76m                                                                                                                                                                                                                                                                                                                                                                                                               TTCATTTGTCGAAACCCACAGAAGGCACAACATCAAGAATGAACGCTAATGTAAACTACAGACTTCACAGGGCGAC
chr10 chr10 128799797 128799721 76m                                                                                                                                                                                                                                                                                                                                                                                                                  ATTTGTCGAAACCCACAGAAGGCACAACATCAAGAATGAACGCTAATGTAAACTACAGACTTCACAGGGCGACGAT
chr10 chr10 128799793 128799717 76m                                                                                                                                                                                                                                                                                                                                                                                                                      GTCGAAACCCACAGAAGGCACAACATCAAGAATGAACGCTAATGTAAACTACAGACTTCACAGGGCGACGATGTGT
chr10 chr10 128799789 128799713 76m                                                                                                                                                                                                                                                                                                                                                                                                                          AAACCAACAGAAGGCACAACATCAAGAATGAACGCTAATGTAAACTACAGACTTCACAGGGCGACGATGTGTCAAT
chr10 chr10 128799786 128799710 76m                                                                                                                                                                                                                                                                                                                                                                                                                             CCCACAGAAGGCACAACATCAAGAATGAACGCTAATGTAAACTACAGACTTCACAGGGCGACGATGTGTCAATGCG
chr10 chr10 128799783 128799707 76m                                                                                                                                                                                                                                                                                                                                                                                                                                ACAGAAGGCACAACATCAAGAATGAACGCTAATGTAAACTACAGACTTCACAGGGCGACGATGTGTCAATGCGGTG
chr10 chr10 128799780 128799704 76m                                                                                                                                                                                                                                                                                                                                                                                                                                   GAAGGCACAACATCAAGAATGAACGCTAATGTAAACTACAGACTTCACAGGGCGACGATGTGTCAATGCGGTTCAC
chr10 chr10 128799777 128799701 76m                                                                                                                                                                                                                                                                                                                                                                                                                                      GGCACAACATCAAGAATGAACGCTAATGTAAACTACAGACTTCACAGGGCGACGATGTGTCAATGCGGTTCACCAA
chr10 chr10 128799774 128799698 76m                                                                                                                                                                                                                                                                                                                                                                                                                                         ACAACATCAAGAATGAACGCTAATGTAAACTACAGACTTCACAGGGCGACGATGTGTCAATGCGGTTCACCAACTG
chr10 chr10 128799771 128799695 76m                                                                                                                                                                                                                                                                                                                                                                                                                                            ACATCAAGAATGAACGCTAATGTAAACTACAGACTTCACAGGGCGACGATGTGTCAATGCGGTTCACCAACTGTAA
chr10 chr10 128799768 128799692 76m                                                                                                                                                                                                                                                                                                                                                                                                                                               TCAAGAATGAACGCTAATGTAAACTACAGACTTCACAGGGCGACGATGTGTCAATGCGGTTCACCAACTGTAATCA
chr10 chr10 128799764 128799688 76m                                                                                                                                                                                                                                                                                                                                                                                                                                                   GAATGAACGCTAATGTAAACTACAGACTTCACAGGGCGACGATGTGTCAATGCGGTTCACCAACTGTAATCAATGT
chr10 chr10 128799761 128799685 76m                                                                                                                                                                                                                                                                                                                                                                                                                                                      TGAACGCTAATGTAAACTACAGACTTCACAGGGCGACGATGTGTCAATGCGGTTCACCAACTGTAATCAATGTACC
chr10 chr10 128799758 128799682 76m                                                                                                                                                                                                                                                                                                                                                                                                                                                         ACGCTAATGTAAACTACAGACTTCACAGGGCGACGATGTGTCAATGCGGTTCACCAACTGTAATCAATGTACCAAT
chr10 chr10 128799755 128799679 76m                                                                                                                                                                                                                                                                                                                                                                                                                                                            CTAATGTAAACTACAGACTTCACAGGGCGACGATGTGTCAATGCGGTTCACCAACTGTAATCAATGTACCAATCTA
chr10 chr10 128799752 128799676 76m                                                                                                                                                                                                                                                                                                                                                                                                                                                               ATGTAAACTACAGACTTCACAGGGCGACGATGTGTCAATGCGGTTCACCAACTGTAATCAATGTACCAATCTATGG
chr10 chr10 128799748 128799672 76m                                                                                                                                                                                                                                                                                                                                                                                                                                                                   AAACTACAGACTTCACAGGGCGACGATGTGTCAATGCGGTTCACCAACTGTAATCAATGTACCAATCTATGGTGGA
chr10 chr10 128799744 128799668 76m                                                                                                                                                                                                                                                                                                                                                                                                                                                                       TACAGACTTCACAGGGCGACGATGTGTCAATGCGGTTCACCAACTGTAATCAATGTACCAATCTATGGTGGATACT
chr10 chr10 128799741 128799665 76m                                                                                                                                                                                                                                                                                                                                                                                                                                                                          AGACTTCACAGGGCGACGATGTGTCAATGCGGTTCACCAACTGTAATCAATGTACCAATCTATGGTGGATACTGAT
chr10 chr10 128799738 128799662 76m                                                                                                                                                                                                                                                                                                                                                                                                                                                                             CTTCACAGGGCGACGATGTGTCAATGCGGTTCACCAACTGTAATCAATGTACCAATCTATGGTGGATACTGATAAT
chr10 chr10 128799734 128799658 76m                                                                                                                                                                                                                                                                                                                                                                                                                                                                                 ACAGGGCGACGATGTGTCAATGCGGTTCACCAACTGTAATCAATGTACCAATCTATGGTGGATACTGATAATGGGG
chr10 chr10 128799731 128799655 76m                                                                                                                                                                                                                                                                                                                                                                                                                                                                                    CGGCGACGATGTGTCAATGCGGTTCACCAACTGTAATCAATGTACCAATCTATGGTGGATACTGATAATGGGGAGG
chr10 chr10 128799728 128799652 76m                                                                                                                                                                                                                                                                                                                                                                                                                                                                                       CGACGATGTGTCAATGCGGTTCACCAACTGTAATCAATGTACCAATCTATGGTGGATACTGATAATGGGGAGGCAG
chr10 chr10 128799725 128799649 76m                                                                                                                                                                                                                                                                                                                                                                                                                                                                                          CGATGTGTCAATGCGGTTCACCAACTGTAATCAATGTACCAATCTATGGTGGATACTGATAATGGGGAGGCAGAGG
chr10 chr10 128799721 128799645 76m                                                                                                                                                                                                                                                                                                                                                                                                                                                                                              GTGTCAATGCGGTTCACCAACTGTAATCAATGTACCAATCTATGGTGGATACTGATAATGGGGAGGCAGGGGGAAG 
    27 pairs 
 105:631
-789:-715
-484:1310
2387:17
3089:5
3111:12
3110:17
3111:24
3105:31
3117:20
3095:43
3116:24
3116:32
3135:18
3130:27
3111:92
3199:17
3122:109
3111:644
3111:644
3089:668
3111:756
3006:1305
3048:1321
3005:1403
3000:1412
2996:1421 
     TCGA-02-2483-01A chr10-chr10 103340004 128799872 rr 
 
65 33 30 37 55 618
GTCCCACCGGGGTATCTTCAGCCGCCTCCTTGACAGTCTTCGGCAGGAAG GCGGAGCACAGAGGACTTTTAGGATGGTGAAGCTACCCAGTGAGAGACTA
99999999999999999999999999999999999999999999999999 99999999999999999999999999999999999999999999997766
POLL exon2(103340003-103340172) DOCK1 intron10(128798571-128806993)
 
 blast search - human genome  
 left 
 >ref|NT_030059.13| Homo sapiens chromosome 10 genomic contig, GRCh37.p9 Primary 
Assembly
 gb|GL000099.1| Homo sapiens chromosome 10 genomic contig, GRCh37 reference primary 
assembly
Length=79420533

 Score = 93.5 bits (50),  Expect = 5e-17
 Identities = 50/50 (100%), Gaps = 0/50 (0%)
 Strand=Plus/Minus

Query  1         GTCCCACCGGGGTATCTTCAGCCGCCTCCTTGACAGTCTTCGGCAGGAAG  50
                 ||||||||||||||||||||||||||||||||||||||||||||||||||
Sbjct  54144518  GTCCCACCGGGGTATCTTCAGCCGCCTCCTTGACAGTCTTCGGCAGGAAG  54144469


>ref|NW_001838006.2| Homo sapiens chromosome 10 genomic contig, alternate assembly 
HuRef SCAF_1103279188397, whole genome shotgun sequence
 gb|DS486042.1| Homo sapiens SCAF_1103279188397 genomic scaffold, whole genome 
shotgun sequence
Length=24511762

 Score = 93.5 bits (50),  Expect = 5e-17
 Identities = 50/50 (100%), Gaps = 0/50 (0%)
 Strand=Plus/Plus

Query  1         GTCCCACCGGGGTATCTTCAGCCGCCTCCTTGACAGTCTTCGGCAGGAAG  50
                 ||||||||||||||||||||||||||||||||||||||||||||||||||
Sbjct  21661033  GTCCCACCGGGGTATCTTCAGCCGCCTCCTTGACAGTCTTCGGCAGGAAG  21661082


>ref|NC_000010.10| Homo sapiens chromosome 10, GRCh37.p9 Primary Assembly
 gb|CM000672.1| Homo sapiens chromosome 10, GRCh37 primary reference assembly
Length=135534747

 Score = 93.5 bits (50),  Expect = 5e-17
 Identities = 50/50 (100%), Gaps = 0/50 (0%)
 Strand=Plus/Minus

Query  1          GTCCCACCGGGGTATCTTCAGCCGCCTCCTTGACAGTCTTCGGCAGGAAG  50
                  ||||||||||||||||||||||||||||||||||||||||||||||||||
Sbjct  103340054  GTCCCACCGGGGTATCTTCAGCCGCCTCCTTGACAGTCTTCGGCAGGAAG  103340005


>ref|AC_000142.1| Homo sapiens chromosome 10, alternate assembly HuRef, whole genome 
shotgun sequence
 gb|CM000471.1| Homo sapiens chromosome 10, whole genome shotgun sequence
Length=128985118

 Score = 93.5 bits (50),  Expect = 5e-17
 Identities = 50/50 (100%), Gaps = 0/50 (0%)
 Strand=Plus/Minus

Query  1         GTCCCACCGGGGTATCTTCAGCCGCCTCCTTGACAGTCTTCGGCAGGAAG  50
                 ||||||||||||||||||||||||||||||||||||||||||||||||||
Sbjct  96970651  GTCCCACCGGGGTATCTTCAGCCGCCTCCTTGACAGTCTTCGGCAGGAAG  96970602


>gb|CM000261.1| Homo sapiens chromosome 10, whole genome shotgun sequence
Length=129189614

 Score = 93.5 bits (50),  Expect = 5e-17
 Identities = 50/50 (100%), Gaps = 0/50 (0%)
 Strand=Plus/Minus

Query  1         GTCCCACCGGGGTATCTTCAGCCGCCTCCTTGACAGTCTTCGGCAGGAAG  50
                 ||||||||||||||||||||||||||||||||||||||||||||||||||
Sbjct  97078840  GTCCCACCGGGGTATCTTCAGCCGCCTCCTTGACAGTCTTCGGCAGGAAG  97078791


Lambda     K      H
    1.33    0.621     1.12 

Gapped
Lambda     K      H
    1.28    0.460    0.850 

Effective search space used: 681780354108
 
 right 
 >ref|NT_008818.16| Homo sapiens chromosome 10 genomic contig, GRCh37.p9 Primary 
Assembly
 gb|GL000100.1| Homo sapiens chromosome 10 genomic contig, GRCh37 reference primary 
assembly
Length=6758678

 Score = 93.5 bits (50),  Expect = 5e-17
 Identities = 50/50 (100%), Gaps = 0/50 (0%)
 Strand=Plus/Minus

Query  1      GCGGAGCACAGAGGACTTTTAGGATGGTGAAGCTACCCAGTGAGAGACTA  50
              ||||||||||||||||||||||||||||||||||||||||||||||||||
Sbjct  33804  GCGGAGCACAGAGGACTTTTAGGATGGTGAAGCTACCCAGTGAGAGACTA  33755


>ref|NW_001838011.1| Homo sapiens chromosome 10 genomic contig, alternate assembly 
HuRef SCAF_1103279183978, whole genome shotgun sequence
 gb|DS486180.1| Homo sapiens SCAF_1103279183978 genomic scaffold, whole genome 
shotgun sequence
Length=3840288

 Score = 93.5 bits (50),  Expect = 5e-17
 Identities = 50/50 (100%), Gaps = 0/50 (0%)
 Strand=Plus/Minus

Query  1      GCGGAGCACAGAGGACTTTTAGGATGGTGAAGCTACCCAGTGAGAGACTA  50
              ||||||||||||||||||||||||||||||||||||||||||||||||||
Sbjct  48057  GCGGAGCACAGAGGACTTTTAGGATGGTGAAGCTACCCAGTGAGAGACTA  48008


>ref|NC_000010.10| Homo sapiens chromosome 10, GRCh37.p9 Primary Assembly
 gb|CM000672.1| Homo sapiens chromosome 10, GRCh37 primary reference assembly
Length=135534747

 Score = 93.5 bits (50),  Expect = 5e-17
 Identities = 50/50 (100%), Gaps = 0/50 (0%)
 Strand=Plus/Minus

Query  1          GCGGAGCACAGAGGACTTTTAGGATGGTGAAGCTACCCAGTGAGAGACTA  50
                  ||||||||||||||||||||||||||||||||||||||||||||||||||
Sbjct  128799873  GCGGAGCACAGAGGACTTTTAGGATGGTGAAGCTACCCAGTGAGAGACTA  128799824


>ref|AC_000142.1| Homo sapiens chromosome 10, alternate assembly HuRef, whole genome 
shotgun sequence
 gb|CM000471.1| Homo sapiens chromosome 10, whole genome shotgun sequence
Length=128985118

 Score = 93.5 bits (50),  Expect = 5e-17
 Identities = 50/50 (100%), Gaps = 0/50 (0%)
 Strand=Plus/Minus

Query  1          GCGGAGCACAGAGGACTTTTAGGATGGTGAAGCTACCCAGTGAGAGACTA  50
                  ||||||||||||||||||||||||||||||||||||||||||||||||||
Sbjct  122371666  GCGGAGCACAGAGGACTTTTAGGATGGTGAAGCTACCCAGTGAGAGACTA  122371617


>gb|CM000261.1| Homo sapiens chromosome 10, whole genome shotgun sequence
Length=129189614

 Score = 93.5 bits (50),  Expect = 5e-17
 Identities = 50/50 (100%), Gaps = 0/50 (0%)
 Strand=Plus/Minus

Query  1          GCGGAGCACAGAGGACTTTTAGGATGGTGAAGCTACCCAGTGAGAGACTA  50
                  ||||||||||||||||||||||||||||||||||||||||||||||||||
Sbjct  122470050  GCGGAGCACAGAGGACTTTTAGGATGGTGAAGCTACCCAGTGAGAGACTA  122470001


Lambda     K      H
    1.33    0.621     1.12 

Gapped
Lambda     K      H
    1.28    0.460    0.850 

Effective search space used: 681780354108
 
 blast search - nt  
 left 
 >ref|XM_003825496.1| PREDICTED: Pan paniscus polymerase (DNA directed), lambda, transcript 
variant 5 (POLL), mRNA
Length=2324

 Score = 93.5 bits (50),  Expect = 6e-17
 Identities = 50/50 (100%), Gaps = 0/50 (0%)
 Strand=Plus/Plus

Query  1     GTCCCACCGGGGTATCTTCAGCCGCCTCCTTGACAGTCTTCGGCAGGAAG  50
             ||||||||||||||||||||||||||||||||||||||||||||||||||
Sbjct  1339  GTCCCACCGGGGTATCTTCAGCCGCCTCCTTGACAGTCTTCGGCAGGAAG  1388


>ref|XM_003825495.1| PREDICTED: Pan paniscus polymerase (DNA directed), lambda, transcript 
variant 4 (POLL), mRNA
Length=2455

 Score = 93.5 bits (50),  Expect = 6e-17
 Identities = 50/50 (100%), Gaps = 0/50 (0%)
 Strand=Plus/Plus

Query  1     GTCCCACCGGGGTATCTTCAGCCGCCTCCTTGACAGTCTTCGGCAGGAAG  50
             ||||||||||||||||||||||||||||||||||||||||||||||||||
Sbjct  1470  GTCCCACCGGGGTATCTTCAGCCGCCTCCTTGACAGTCTTCGGCAGGAAG  1519


>ref|XM_003825494.1| PREDICTED: Pan paniscus polymerase (DNA directed), lambda, transcript 
variant 3 (POLL), mRNA
Length=2329

 Score = 93.5 bits (50),  Expect = 6e-17
 Identities = 50/50 (100%), Gaps = 0/50 (0%)
 Strand=Plus/Plus

Query  1     GTCCCACCGGGGTATCTTCAGCCGCCTCCTTGACAGTCTTCGGCAGGAAG  50
             ||||||||||||||||||||||||||||||||||||||||||||||||||
Sbjct  1344  GTCCCACCGGGGTATCTTCAGCCGCCTCCTTGACAGTCTTCGGCAGGAAG  1393


>ref|XM_003825493.1| PREDICTED: Pan paniscus polymerase (DNA directed), lambda, transcript 
variant 2 (POLL), mRNA
Length=2801

 Score = 93.5 bits (50),  Expect = 6e-17
 Identities = 50/50 (100%), Gaps = 0/50 (0%)
 Strand=Plus/Plus

Query  1     GTCCCACCGGGGTATCTTCAGCCGCCTCCTTGACAGTCTTCGGCAGGAAG  50
             ||||||||||||||||||||||||||||||||||||||||||||||||||
Sbjct  1816  GTCCCACCGGGGTATCTTCAGCCGCCTCCTTGACAGTCTTCGGCAGGAAG  1865


>ref|XM_003825492.1| PREDICTED: Pan paniscus polymerase (DNA directed), lambda, transcript 
variant 1 (POLL), mRNA
Length=2731

 Score = 93.5 bits (50),  Expect = 6e-17
 Identities = 50/50 (100%), Gaps = 0/50 (0%)
 Strand=Plus/Plus

Query  1     GTCCCACCGGGGTATCTTCAGCCGCCTCCTTGACAGTCTTCGGCAGGAAG  50
             ||||||||||||||||||||||||||||||||||||||||||||||||||
Sbjct  1746  GTCCCACCGGGGTATCTTCAGCCGCCTCCTTGACAGTCTTCGGCAGGAAG  1795


>dbj|AK306684.1| Pan troglodytes mRNA for DNA polymerase lambda, complete cds, 
clone: PtsC-28-5_D09
Length=2383

 Score = 93.5 bits (50),  Expect = 6e-17
 Identities = 50/50 (100%), Gaps = 0/50 (0%)
 Strand=Plus/Plus

Query  1     GTCCCACCGGGGTATCTTCAGCCGCCTCCTTGACAGTCTTCGGCAGGAAG  50
             ||||||||||||||||||||||||||||||||||||||||||||||||||
Sbjct  1382  GTCCCACCGGGGTATCTTCAGCCGCCTCCTTGACAGTCTTCGGCAGGAAG  1431


>ref|XM_001169987.2| PREDICTED: Pan troglodytes polymerase (DNA directed), lambda, 
transcript variant 9 (POLL), mRNA
Length=2326

 Score = 93.5 bits (50),  Expect = 6e-17
 Identities = 50/50 (100%), Gaps = 0/50 (0%)
 Strand=Plus/Plus

Query  1     GTCCCACCGGGGTATCTTCAGCCGCCTCCTTGACAGTCTTCGGCAGGAAG  50
             ||||||||||||||||||||||||||||||||||||||||||||||||||
Sbjct  1339  GTCCCACCGGGGTATCTTCAGCCGCCTCCTTGACAGTCTTCGGCAGGAAG  1388


>ref|XM_001170157.2| PREDICTED: Pan troglodytes polymerase (DNA directed), lambda, 
transcript variant 17 (POLL), mRNA
Length=2457

 Score = 93.5 bits (50),  Expect = 6e-17
 Identities = 50/50 (100%), Gaps = 0/50 (0%)
 Strand=Plus/Plus

Query  1     GTCCCACCGGGGTATCTTCAGCCGCCTCCTTGACAGTCTTCGGCAGGAAG  50
             ||||||||||||||||||||||||||||||||||||||||||||||||||
Sbjct  1470  GTCCCACCGGGGTATCTTCAGCCGCCTCCTTGACAGTCTTCGGCAGGAAG  1519


>ref|XM_001170138.2| PREDICTED: Pan troglodytes polymerase (DNA directed), lambda, 
transcript variant 16 (POLL), mRNA
Length=2803

 Score = 93.5 bits (50),  Expect = 6e-17
 Identities = 50/50 (100%), Gaps = 0/50 (0%)
 Strand=Plus/Plus

Query  1     GTCCCACCGGGGTATCTTCAGCCGCCTCCTTGACAGTCTTCGGCAGGAAG  50
             ||||||||||||||||||||||||||||||||||||||||||||||||||
Sbjct  1816  GTCCCACCGGGGTATCTTCAGCCGCCTCCTTGACAGTCTTCGGCAGGAAG  1865


>ref|XM_001170074.2| PREDICTED: Pan troglodytes polymerase (DNA directed), lambda, 
transcript variant 13 (POLL), mRNA
Length=2733

 Score = 93.5 bits (50),  Expect = 6e-17
 Identities = 50/50 (100%), Gaps = 0/50 (0%)
 Strand=Plus/Plus

Query  1     GTCCCACCGGGGTATCTTCAGCCGCCTCCTTGACAGTCTTCGGCAGGAAG  50
             ||||||||||||||||||||||||||||||||||||||||||||||||||
Sbjct  1746  GTCCCACCGGGGTATCTTCAGCCGCCTCCTTGACAGTCTTCGGCAGGAAG  1795


>ref|NG_017063.1| Homo sapiens polymerase (DNA directed), lambda (POLL), RefSeqGene 
on chromosome 10
Length=16389

 Score = 93.5 bits (50),  Expect = 6e-17
 Identities = 50/50 (100%), Gaps = 0/50 (0%)
 Strand=Plus/Plus

Query  1      GTCCCACCGGGGTATCTTCAGCCGCCTCCTTGACAGTCTTCGGCAGGAAG  50
              ||||||||||||||||||||||||||||||||||||||||||||||||||
Sbjct  12974  GTCCCACCGGGGTATCTTCAGCCGCCTCCTTGACAGTCTTCGGCAGGAAG  13023


>ref|NR_033406.1| Homo sapiens polymerase (DNA directed), lambda (POLL), transcript 
variant 4, non-coding RNA
Length=2340

 Score = 93.5 bits (50),  Expect = 6e-17
 Identities = 50/50 (100%), Gaps = 0/50 (0%)
 Strand=Plus/Plus

Query  1     GTCCCACCGGGGTATCTTCAGCCGCCTCCTTGACAGTCTTCGGCAGGAAG  50
             ||||||||||||||||||||||||||||||||||||||||||||||||||
Sbjct  1345  GTCCCACCGGGGTATCTTCAGCCGCCTCCTTGACAGTCTTCGGCAGGAAG  1394


>ref|NM_013274.3| Homo sapiens polymerase (DNA directed), lambda (POLL), transcript 
variant 2, mRNA
Length=2741

 Score = 93.5 bits (50),  Expect = 6e-17
 Identities = 50/50 (100%), Gaps = 0/50 (0%)
 Strand=Plus/Plus

Query  1     GTCCCACCGGGGTATCTTCAGCCGCCTCCTTGACAGTCTTCGGCAGGAAG  50
             ||||||||||||||||||||||||||||||||||||||||||||||||||
Sbjct  1746  GTCCCACCGGGGTATCTTCAGCCGCCTCCTTGACAGTCTTCGGCAGGAAG  1795


>ref|NM_001174085.1| Homo sapiens polymerase (DNA directed), lambda (POLL), transcript 
variant 3, mRNA
Length=2465

 Score = 93.5 bits (50),  Expect = 6e-17
 Identities = 50/50 (100%), Gaps = 0/50 (0%)
 Strand=Plus/Plus

Query  1     GTCCCACCGGGGTATCTTCAGCCGCCTCCTTGACAGTCTTCGGCAGGAAG  50
             ||||||||||||||||||||||||||||||||||||||||||||||||||
Sbjct  1470  GTCCCACCGGGGTATCTTCAGCCGCCTCCTTGACAGTCTTCGGCAGGAAG  1519


>ref|NM_001174084.1| Homo sapiens polymerase (DNA directed), lambda (POLL), transcript 
variant 1, mRNA
Length=2804

 Score = 93.5 bits (50),  Expect = 6e-17
 Identities = 50/50 (100%), Gaps = 0/50 (0%)
 Strand=Plus/Plus

Query  1     GTCCCACCGGGGTATCTTCAGCCGCCTCCTTGACAGTCTTCGGCAGGAAG  50
             ||||||||||||||||||||||||||||||||||||||||||||||||||
Sbjct  1809  GTCCCACCGGGGTATCTTCAGCCGCCTCCTTGACAGTCTTCGGCAGGAAG  1858


>dbj|AK296220.1| Homo sapiens cDNA FLJ57512 complete cds
Length=1499

 Score = 93.5 bits (50),  Expect = 6e-17
 Identities = 50/50 (100%), Gaps = 0/50 (0%)
 Strand=Plus/Minus

Query  1    GTCCCACCGGGGTATCTTCAGCCGCCTCCTTGACAGTCTTCGGCAGGAAG  50
            ||||||||||||||||||||||||||||||||||||||||||||||||||
Sbjct  241  GTCCCACCGGGGTATCTTCAGCCGCCTCCTTGACAGTCTTCGGCAGGAAG  192


>dbj|AK300862.1| Homo sapiens cDNA FLJ54038 complete cds, highly similar to DNA 
polymerase lambda (EC 2.7.7.7)
Length=1406

 Score = 93.5 bits (50),  Expect = 6e-17
 Identities = 50/50 (100%), Gaps = 0/50 (0%)
 Strand=Plus/Plus

Query  1    GTCCCACCGGGGTATCTTCAGCCGCCTCCTTGACAGTCTTCGGCAGGAAG  50
            ||||||||||||||||||||||||||||||||||||||||||||||||||
Sbjct  773  GTCCCACCGGGGTATCTTCAGCCGCCTCCTTGACAGTCTTCGGCAGGAAG  822


>dbj|AK293602.1| Homo sapiens cDNA FLJ55191 complete cds, highly similar to DNA 
polymerase lambda (EC 2.7.7.7)
Length=1533

 Score = 93.5 bits (50),  Expect = 6e-17
 Identities = 50/50 (100%), Gaps = 0/50 (0%)
 Strand=Plus/Plus

Query  1    GTCCCACCGGGGTATCTTCAGCCGCCTCCTTGACAGTCTTCGGCAGGAAG  50
            ||||||||||||||||||||||||||||||||||||||||||||||||||
Sbjct  642  GTCCCACCGGGGTATCTTCAGCCGCCTCCTTGACAGTCTTCGGCAGGAAG  691


>dbj|AK293421.1| Homo sapiens cDNA FLJ53301 complete cds, highly similar to DNA 
polymerase lambda (EC 2.7.7.7)
Length=1686

 Score = 93.5 bits (50),  Expect = 6e-17
 Identities = 50/50 (100%), Gaps = 0/50 (0%)
 Strand=Plus/Plus

Query  1     GTCCCACCGGGGTATCTTCAGCCGCCTCCTTGACAGTCTTCGGCAGGAAG  50
             ||||||||||||||||||||||||||||||||||||||||||||||||||
Sbjct  967   GTCCCACCGGGGTATCTTCAGCCGCCTCCTTGACAGTCTTCGGCAGGAAG  1016


>dbj|AB384717.1| Synthetic construct DNA, clone: pF1KB3002, Homo sapiens POLL 
gene for DNA polymerase lambda, complete cds, without stop codon, 
in Flexi system
Length=1742

 Score = 93.5 bits (50),  Expect = 6e-17
 Identities = 50/50 (100%), Gaps = 0/50 (0%)
 Strand=Plus/Plus

Query  1     GTCCCACCGGGGTATCTTCAGCCGCCTCCTTGACAGTCTTCGGCAGGAAG  50
             ||||||||||||||||||||||||||||||||||||||||||||||||||
Sbjct  1323  GTCCCACCGGGGTATCTTCAGCCGCCTCCTTGACAGTCTTCGGCAGGAAG  1372


>dbj|AK292225.1| Homo sapiens cDNA FLJ77175 complete cds, highly similar to Homo 
sapiens DNA polymerase lamda2 mRNA
Length=2161

 Score = 93.5 bits (50),  Expect = 6e-17
 Identities = 50/50 (100%), Gaps = 0/50 (0%)
 Strand=Plus/Plus

Query  1     GTCCCACCGGGGTATCTTCAGCCGCCTCCTTGACAGTCTTCGGCAGGAAG  50
             ||||||||||||||||||||||||||||||||||||||||||||||||||
Sbjct  1392  GTCCCACCGGGGTATCTTCAGCCGCCTCCTTGACAGTCTTCGGCAGGAAG  1441


>gb|AY302442.1| Homo sapiens DNA polymerase lamda2 mRNA, complete cds
Length=1452

 Score = 93.5 bits (50),  Expect = 6e-17
 Identities = 50/50 (100%), Gaps = 0/50 (0%)
 Strand=Plus/Plus

Query  1     GTCCCACCGGGGTATCTTCAGCCGCCTCCTTGACAGTCTTCGGCAGGAAG  50
             ||||||||||||||||||||||||||||||||||||||||||||||||||
Sbjct  1038  GTCCCACCGGGGTATCTTCAGCCGCCTCCTTGACAGTCTTCGGCAGGAAG  1087


>gb|BC003548.1| Homo sapiens polymerase (DNA directed), lambda, mRNA (cDNA clone 
MGC:1149 IMAGE:3536366), complete cds
Length=2380

 Score = 93.5 bits (50),  Expect = 6e-17
 Identities = 50/50 (100%), Gaps = 0/50 (0%)
 Strand=Plus/Plus

Query  1     GTCCCACCGGGGTATCTTCAGCCGCCTCCTTGACAGTCTTCGGCAGGAAG  50
             ||||||||||||||||||||||||||||||||||||||||||||||||||
Sbjct  1304  GTCCCACCGGGGTATCTTCAGCCGCCTCCTTGACAGTCTTCGGCAGGAAG  1353


>dbj|AK094956.1| Homo sapiens cDNA FLJ37637 fis, clone BRCOC2018877, highly similar 
to Homo sapiens mRNA for DNA polymerase lambda (POLL gene)
Length=1871

 Score = 93.5 bits (50),  Expect = 6e-17
 Identities = 50/50 (100%), Gaps = 0/50 (0%)
 Strand=Plus/Plus

Query  1    GTCCCACCGGGGTATCTTCAGCCGCCTCCTTGACAGTCTTCGGCAGGAAG  50
            ||||||||||||||||||||||||||||||||||||||||||||||||||
Sbjct  888  GTCCCACCGGGGTATCTTCAGCCGCCTCCTTGACAGTCTTCGGCAGGAAG  937


>dbj|AK128521.1| Homo sapiens cDNA FLJ46679 fis, clone TRACH3010331, highly  similar 
to Homo sapiens polymerase (DNA directed), lambda (POLL)
Length=4128

 Score = 93.5 bits (50),  Expect = 6e-17
 Identities = 50/50 (100%), Gaps = 0/50 (0%)
 Strand=Plus/Plus

Query  1    GTCCCACCGGGGTATCTTCAGCCGCCTCCTTGACAGTCTTCGGCAGGAAG  50
            ||||||||||||||||||||||||||||||||||||||||||||||||||
Sbjct  163  GTCCCACCGGGGTATCTTCAGCCGCCTCCTTGACAGTCTTCGGCAGGAAG  212


>dbj|AK127896.1| Homo sapiens cDNA FLJ46002 fis, clone SMINT2011509, highly similar 
to DNA polymerase lambda (EC 2.7.7.7)
Length=2064

 Score = 93.5 bits (50),  Expect = 6e-17
 Identities = 50/50 (100%), Gaps = 0/50 (0%)
 Strand=Plus/Plus

Query  1     GTCCCACCGGGGTATCTTCAGCCGCCTCCTTGACAGTCTTCGGCAGGAAG  50
             ||||||||||||||||||||||||||||||||||||||||||||||||||
Sbjct  1081  GTCCCACCGGGGTATCTTCAGCCGCCTCCTTGACAGTCTTCGGCAGGAAG  1130


>dbj|AK091106.1| Homo sapiens cDNA FLJ33787 fis, clone BRSSN2009119
Length=2912

 Score = 93.5 bits (50),  Expect = 6e-17
 Identities = 50/50 (100%), Gaps = 0/50 (0%)
 Strand=Plus/Plus

Query  1     GTCCCACCGGGGTATCTTCAGCCGCCTCCTTGACAGTCTTCGGCAGGAAG  50
             ||||||||||||||||||||||||||||||||||||||||||||||||||
Sbjct  1929  GTCCCACCGGGGTATCTTCAGCCGCCTCCTTGACAGTCTTCGGCAGGAAG  1978


>dbj|AK022476.1| Homo sapiens cDNA FLJ12414 fis, clone MAMMA1003013, weakly similar 
to DNA POLYMERASE BETA (EC 2.7.7.7)
Length=2306

 Score = 93.5 bits (50),  Expect = 6e-17
 Identities = 50/50 (100%), Gaps = 0/50 (0%)
 Strand=Plus/Plus

Query  1     GTCCCACCGGGGTATCTTCAGCCGCCTCCTTGACAGTCTTCGGCAGGAAG  50
             ||||||||||||||||||||||||||||||||||||||||||||||||||
Sbjct  1323  GTCCCACCGGGGTATCTTCAGCCGCCTCCTTGACAGTCTTCGGCAGGAAG  1372


>dbj|AK021600.1| Homo sapiens cDNA FLJ11538 fis, clone HEMBA1002746, weakly similar 
to DNA POLYMERASE BETA (EC 2.7.7.7)
Length=2281

 Score = 93.5 bits (50),  Expect = 6e-17
 Identities = 50/50 (100%), Gaps = 0/50 (0%)
 Strand=Plus/Plus

Query  1     GTCCCACCGGGGTATCTTCAGCCGCCTCCTTGACAGTCTTCGGCAGGAAG  50
             ||||||||||||||||||||||||||||||||||||||||||||||||||
Sbjct  1417  GTCCCACCGGGGTATCTTCAGCCGCCTCCTTGACAGTCTTCGGCAGGAAG  1466


>gb|BC068529.1| Homo sapiens polymerase (DNA directed), lambda, mRNA (cDNA clone 
MGC:87359 IMAGE:5267859), complete cds
Length=2696

 Score = 93.5 bits (50),  Expect = 6e-17
 Identities = 50/50 (100%), Gaps = 0/50 (0%)
 Strand=Plus/Plus

Query  1     GTCCCACCGGGGTATCTTCAGCCGCCTCCTTGACAGTCTTCGGCAGGAAG  50
             ||||||||||||||||||||||||||||||||||||||||||||||||||
Sbjct  1698  GTCCCACCGGGGTATCTTCAGCCGCCTCCTTGACAGTCTTCGGCAGGAAG  1747


>gb|AF525924.1| Homo sapiens polymerase (DNA directed), lambda (POLL) gene, complete 
cds
Length=11263

 Score = 93.5 bits (50),  Expect = 6e-17
 Identities = 50/50 (100%), Gaps = 0/50 (0%)
 Strand=Plus/Plus

Query  1     GTCCCACCGGGGTATCTTCAGCCGCCTCCTTGACAGTCTTCGGCAGGAAG  50
             ||||||||||||||||||||||||||||||||||||||||||||||||||
Sbjct  8855  GTCCCACCGGGGTATCTTCAGCCGCCTCCTTGACAGTCTTCGGCAGGAAG  8904


>emb|AL627424.8| Human DNA sequence from clone RP11-529I10 on chromosome 10 Contains 
the 3' end of the BTRC gene for beta-transducin repeat 
containing, the POLL gene for polymerase (DNA directed) lambda, 
a novel gene (DKFZP566F084), the 3' end of the SHFM3 gene 
for split hand/foot malformation (ectrodactyly) type 3 and 
three CpG islands, complete sequence
Length=108503

 Score = 93.5 bits (50),  Expect = 6e-17
 Identities = 50/50 (100%), Gaps = 0/50 (0%)
 Strand=Plus/Minus

Query  1      GTCCCACCGGGGTATCTTCAGCCGCCTCCTTGACAGTCTTCGGCAGGAAG  50
              ||||||||||||||||||||||||||||||||||||||||||||||||||
Sbjct  49954  GTCCCACCGGGGTATCTTCAGCCGCCTCCTTGACAGTCTTCGGCAGGAAG  49905


>emb|AJ844642.1| Homo sapiens partial mRNA for DNA polymerase lambda, shorter 
clone (POLL gene)
Length=502

 Score = 93.5 bits (50),  Expect = 6e-17
 Identities = 50/50 (100%), Gaps = 0/50 (0%)
 Strand=Plus/Plus

Query  1    GTCCCACCGGGGTATCTTCAGCCGCCTCCTTGACAGTCTTCGGCAGGAAG  50
            ||||||||||||||||||||||||||||||||||||||||||||||||||
Sbjct  120  GTCCCACCGGGGTATCTTCAGCCGCCTCCTTGACAGTCTTCGGCAGGAAG  169


>emb|AJ844641.1| Homo sapiens partial mRNA for DNA polymerase lambda (POLL gene)
Length=634

 Score = 93.5 bits (50),  Expect = 6e-17
 Identities = 50/50 (100%), Gaps = 0/50 (0%)
 Strand=Plus/Plus

Query  1    GTCCCACCGGGGTATCTTCAGCCGCCTCCTTGACAGTCTTCGGCAGGAAG  50
            ||||||||||||||||||||||||||||||||||||||||||||||||||
Sbjct  252  GTCCCACCGGGGTATCTTCAGCCGCCTCCTTGACAGTCTTCGGCAGGAAG  301


>gb|AF283478.1|AF283478 Homo sapiens DNA polymerase beta2 mRNA, complete cds
Length=1728

 Score = 93.5 bits (50),  Expect = 6e-17
 Identities = 50/50 (100%), Gaps = 0/50 (0%)
 Strand=Plus/Plus

Query  1     GTCCCACCGGGGTATCTTCAGCCGCCTCCTTGACAGTCTTCGGCAGGAAG  50
             ||||||||||||||||||||||||||||||||||||||||||||||||||
Sbjct  1314  GTCCCACCGGGGTATCTTCAGCCGCCTCCTTGACAGTCTTCGGCAGGAAG  1363


>gb|AF218027.1|AF218027 Homo sapiens clone PP712 unknown mRNA
Length=1553

 Score = 93.5 bits (50),  Expect = 6e-17
 Identities = 50/50 (100%), Gaps = 0/50 (0%)
 Strand=Plus/Plus

Query  1    GTCCCACCGGGGTATCTTCAGCCGCCTCCTTGACAGTCTTCGGCAGGAAG  50
            ||||||||||||||||||||||||||||||||||||||||||||||||||
Sbjct  551  GTCCCACCGGGGTATCTTCAGCCGCCTCCTTGACAGTCTTCGGCAGGAAG  600


>gb|AF161019.1|AF161019 Homo sapiens DNA polymerase lambda (Pol lambda) mRNA, complete 
cds
Length=2368

 Score = 93.5 bits (50),  Expect = 6e-17
 Identities = 50/50 (100%), Gaps = 0/50 (0%)
 Strand=Plus/Plus

Query  1     GTCCCACCGGGGTATCTTCAGCCGCCTCCTTGACAGTCTTCGGCAGGAAG  50
             ||||||||||||||||||||||||||||||||||||||||||||||||||
Sbjct  1375  GTCCCACCGGGGTATCTTCAGCCGCCTCCTTGACAGTCTTCGGCAGGAAG  1424


>emb|AJ131890.1| Homo sapiens mRNA for DNA polymerase lambda (POLL gene)
Length=2678

 Score = 93.5 bits (50),  Expect = 6e-17
 Identities = 50/50 (100%), Gaps = 0/50 (0%)
 Strand=Plus/Plus

Query  1     GTCCCACCGGGGTATCTTCAGCCGCCTCCTTGACAGTCTTCGGCAGGAAG  50
             ||||||||||||||||||||||||||||||||||||||||||||||||||
Sbjct  1685  GTCCCACCGGGGTATCTTCAGCCGCCTCCTTGACAGTCTTCGGCAGGAAG  1734


>ref|XM_002821081.2| PREDICTED: Pongo abelii polymerase (DNA directed), lambda, transcript 
variant 2 (POLL), mRNA
Length=2285

 Score = 87.9 bits (47),  Expect = 3e-15
 Identities = 49/50 (98%), Gaps = 0/50 (0%)
 Strand=Plus/Plus

Query  1     GTCCCACCGGGGTATCTTCAGCCGCCTCCTTGACAGTCTTCGGCAGGAAG  50
             |||||||||||||||||||||||||||||||||||||||||||||| |||
Sbjct  1318  GTCCCACCGGGGTATCTTCAGCCGCCTCCTTGACAGTCTTCGGCAGCAAG  1367


>ref|XM_003777618.1| PREDICTED: Pongo abelii polymerase (DNA directed), lambda (POLL), 
mRNA
Length=2417

 Score = 87.9 bits (47),  Expect = 3e-15
 Identities = 49/50 (98%), Gaps = 0/50 (0%)
 Strand=Plus/Plus

Query  1     GTCCCACCGGGGTATCTTCAGCCGCCTCCTTGACAGTCTTCGGCAGGAAG  50
             |||||||||||||||||||||||||||||||||||||||||||||| |||
Sbjct  1450  GTCCCACCGGGGTATCTTCAGCCGCCTCCTTGACAGTCTTCGGCAGCAAG  1499


>ref|XM_003777617.1| PREDICTED: Pongo abelii polymerase (DNA directed), lambda (POLL), 
mRNA
Length=2763

 Score = 87.9 bits (47),  Expect = 3e-15
 Identities = 49/50 (98%), Gaps = 0/50 (0%)
 Strand=Plus/Plus

Query  1     GTCCCACCGGGGTATCTTCAGCCGCCTCCTTGACAGTCTTCGGCAGGAAG  50
             |||||||||||||||||||||||||||||||||||||||||||||| |||
Sbjct  1796  GTCCCACCGGGGTATCTTCAGCCGCCTCCTTGACAGTCTTCGGCAGCAAG  1845


>ref|XM_002821080.2| PREDICTED: Pongo abelii polymerase (DNA directed), lambda, transcript 
variant 1 (POLL), mRNA
Length=2693

 Score = 87.9 bits (47),  Expect = 3e-15
 Identities = 49/50 (98%), Gaps = 0/50 (0%)
 Strand=Plus/Plus

Query  1     GTCCCACCGGGGTATCTTCAGCCGCCTCCTTGACAGTCTTCGGCAGGAAG  50
             |||||||||||||||||||||||||||||||||||||||||||||| |||
Sbjct  1726  GTCCCACCGGGGTATCTTCAGCCGCCTCCTTGACAGTCTTCGGCAGCAAG  1775


>ref|XM_002821082.2| PREDICTED: Pongo abelii polymerase (DNA directed), lambda, transcript 
variant 3 (POLL), mRNA
Length=2358

 Score = 87.9 bits (47),  Expect = 3e-15
 Identities = 49/50 (98%), Gaps = 0/50 (0%)
 Strand=Plus/Plus

Query  1     GTCCCACCGGGGTATCTTCAGCCGCCTCCTTGACAGTCTTCGGCAGGAAG  50
             |||||||||||||||||||||||||||||||||||||||||||||| |||
Sbjct  1391  GTCCCACCGGGGTATCTTCAGCCGCCTCCTTGACAGTCTTCGGCAGCAAG  1440


>gb|HM486797.1| Gorilla gorilla DNA polymerase lambda (POLL) mRNA, partial cds
Length=1725

 Score = 87.9 bits (47),  Expect = 3e-15
 Identities = 49/50 (98%), Gaps = 0/50 (0%)
 Strand=Plus/Plus

Query  1     GTCCCACCGGGGTATCTTCAGCCGCCTCCTTGACAGTCTTCGGCAGGAAG  50
             |||||||||||||||||||||||||||||||||||||||||||||| |||
Sbjct  1314  GTCCCACCGGGGTATCTTCAGCCGCCTCCTTGACAGTCTTCGGCAGCAAG  1363


>gb|HM486801.1| Pongo abelii DNA polymerase lambda (POLL) mRNA, partial cds
Length=1704

 Score = 87.9 bits (47),  Expect = 3e-15
 Identities = 49/50 (98%), Gaps = 0/50 (0%)
 Strand=Plus/Plus

Query  1     GTCCCACCGGGGTATCTTCAGCCGCCTCCTTGACAGTCTTCGGCAGGAAG  50
             |||||||||||||||||||||||||||||||||||||||||||||| |||
Sbjct  1293  GTCCCACCGGGGTATCTTCAGCCGCCTCCTTGACAGTCTTCGGCAGCAAG  1342


>gb|HM486794.1| Pongo pygmaeus DNA polymerase lambda (POLL) mRNA, partial cds
Length=1704

 Score = 87.9 bits (47),  Expect = 3e-15
 Identities = 49/50 (98%), Gaps = 0/50 (0%)
 Strand=Plus/Plus

Query  1     GTCCCACCGGGGTATCTTCAGCCGCCTCCTTGACAGTCTTCGGCAGGAAG  50
             |||||||||||||||||||||||||||||||||||||||||||||| |||
Sbjct  1293  GTCCCACCGGGGTATCTTCAGCCGCCTCCTTGACAGTCTTCGGCAGCAAG  1342


>ref|NM_001266906.2| Macaca mulatta polymerase (DNA directed), lambda (POLL), mRNA
Length=2282

 Score = 82.4 bits (44),  Expect = 1e-13
 Identities = 48/50 (96%), Gaps = 0/50 (0%)
 Strand=Plus/Plus

Query  1     GTCCCACCGGGGTATCTTCAGCCGCCTCCTTGACAGTCTTCGGCAGGAAG  50
             |||||||||||||||||||||||||||||||||||| ||||||||| |||
Sbjct  1351  GTCCCACCGGGGTATCTTCAGCCGCCTCCTTGACAGCCTTCGGCAGCAAG  1400


>ref|XM_003255372.1| PREDICTED: Nomascus leucogenys polymerase (DNA directed), lambda, 
transcript variant 4 (POLL), mRNA
Length=2307

 Score = 82.4 bits (44),  Expect = 1e-13
 Identities = 48/50 (96%), Gaps = 0/50 (0%)
 Strand=Plus/Plus

Query  1     GTCCCACCGGGGTATCTTCAGCCGCCTCCTTGACAGTCTTCGGCAGGAAG  50
             |||||| ||||||||||||||||||||||||||||||||||||||| |||
Sbjct  1339  GTCCCATCGGGGTATCTTCAGCCGCCTCCTTGACAGTCTTCGGCAGCAAG  1388


>ref|XM_003255371.1| PREDICTED: Nomascus leucogenys polymerase (DNA directed), lambda, 
transcript variant 3 (POLL), mRNA
Length=2437

 Score = 82.4 bits (44),  Expect = 1e-13
 Identities = 48/50 (96%), Gaps = 0/50 (0%)
 Strand=Plus/Plus

Query  1     GTCCCACCGGGGTATCTTCAGCCGCCTCCTTGACAGTCTTCGGCAGGAAG  50
             |||||| ||||||||||||||||||||||||||||||||||||||| |||
Sbjct  1469  GTCCCATCGGGGTATCTTCAGCCGCCTCCTTGACAGTCTTCGGCAGCAAG  1518


>ref|XM_003255370.1| PREDICTED: Nomascus leucogenys polymerase (DNA directed), lambda, 
transcript variant 2 (POLL), mRNA
Length=2783

 Score = 82.4 bits (44),  Expect = 1e-13
 Identities = 48/50 (96%), Gaps = 0/50 (0%)
 Strand=Plus/Plus

Query  1     GTCCCACCGGGGTATCTTCAGCCGCCTCCTTGACAGTCTTCGGCAGGAAG  50
             |||||| ||||||||||||||||||||||||||||||||||||||| |||
Sbjct  1815  GTCCCATCGGGGTATCTTCAGCCGCCTCCTTGACAGTCTTCGGCAGCAAG  1864


>ref|XM_003255369.1| PREDICTED: Nomascus leucogenys polymerase (DNA directed), lambda, 
transcript variant 1 (POLL), mRNA
Length=2713

 Score = 82.4 bits (44),  Expect = 1e-13
 Identities = 48/50 (96%), Gaps = 0/50 (0%)
 Strand=Plus/Plus

Query  1     GTCCCACCGGGGTATCTTCAGCCGCCTCCTTGACAGTCTTCGGCAGGAAG  50
             |||||| ||||||||||||||||||||||||||||||||||||||| |||
Sbjct  1745  GTCCCATCGGGGTATCTTCAGCCGCCTCCTTGACAGTCTTCGGCAGCAAG  1794


>gb|HM486807.1| Cercopithecus wolfi DNA polymerase lambda (POLL) mRNA, partial 
cds
Length=1725

 Score = 82.4 bits (44),  Expect = 1e-13
 Identities = 48/50 (96%), Gaps = 0/50 (0%)
 Strand=Plus/Plus

Query  1     GTCCCACCGGGGTATCTTCAGCCGCCTCCTTGACAGTCTTCGGCAGGAAG  50
             |||||||||||||||||||||||||||||||||||| ||||||||| |||
Sbjct  1314  GTCCCACCGGGGTATCTTCAGCCGCCTCCTTGACAGCCTTCGGCAGCAAG  1363


>gb|HM486806.1| Nomascus leucogenys DNA polymerase lambda (POLL) mRNA, partial 
cds
Length=1722

 Score = 82.4 bits (44),  Expect = 1e-13
 Identities = 48/50 (96%), Gaps = 0/50 (0%)
 Strand=Plus/Plus

Query  1     GTCCCACCGGGGTATCTTCAGCCGCCTCCTTGACAGTCTTCGGCAGGAAG  50
             |||||| ||||||||||||||||||||||||||||||||||||||| |||
Sbjct  1311  GTCCCATCGGGGTATCTTCAGCCGCCTCCTTGACAGTCTTCGGCAGCAAG  1360


>gb|HM486804.1| Miopithecus talapoin DNA polymerase lambda (POLL) mRNA, partial 
cds
Length=1722

 Score = 82.4 bits (44),  Expect = 1e-13
 Identities = 48/50 (96%), Gaps = 0/50 (0%)
 Strand=Plus/Plus

Query  1     GTCCCACCGGGGTATCTTCAGCCGCCTCCTTGACAGTCTTCGGCAGGAAG  50
             |||||||||||||||||||||||||||||||||||| ||||||||| |||
Sbjct  1311  GTCCCACCGGGGTATCTTCAGCCGCCTCCTTGACAGCCTTCGGCAGCAAG  1360


>gb|HM486802.1| Symphalangus syndactylus DNA polymerase lambda (POLL) mRNA, partial 
cds
Length=1722

 Score = 82.4 bits (44),  Expect = 1e-13
 Identities = 48/50 (96%), Gaps = 0/50 (0%)
 Strand=Plus/Plus

Query  1     GTCCCACCGGGGTATCTTCAGCCGCCTCCTTGACAGTCTTCGGCAGGAAG  50
             |||||| ||||||||||||||||||||||||||||||||||||||| |||
Sbjct  1311  GTCCCATCGGGGTATCTTCAGCCGCCTCCTTGACAGTCTTCGGCAGCAAG  1360


>gb|HM486800.1| Papio anubis DNA polymerase lambda (POLL) mRNA, partial cds
Length=1725

 Score = 82.4 bits (44),  Expect = 1e-13
 Identities = 48/50 (96%), Gaps = 0/50 (0%)
 Strand=Plus/Plus

Query  1     GTCCCACCGGGGTATCTTCAGCCGCCTCCTTGACAGTCTTCGGCAGGAAG  50
             |||||||||||||||||||||||||||||||||||| ||||||||| |||
Sbjct  1314  GTCCCACCGGGGTATCTTCAGCCGCCTCCTTGACAGCCTTCGGCAGCAAG  1363


>gb|HM486799.1| Trachypithecus francoisi DNA polymerase lambda (POLL) mRNA, partial 
cds
Length=1722

 Score = 82.4 bits (44),  Expect = 1e-13
 Identities = 48/50 (96%), Gaps = 0/50 (0%)
 Strand=Plus/Plus

Query  1     GTCCCACCGGGGTATCTTCAGCCGCCTCCTTGACAGTCTTCGGCAGGAAG  50
             |||||||||||||||||||||||||||||||||||| ||||||||| |||
Sbjct  1311  GTCCCACCGGGGTATCTTCAGCCGCCTCCTTGACAGCCTTCGGCAGCAAG  1360


>gb|HM486795.1| Macaca fascicularis DNA polymerase lambda (POLL) mRNA, partial 
cds
Length=1725

 Score = 82.4 bits (44),  Expect = 1e-13
 Identities = 48/50 (96%), Gaps = 0/50 (0%)
 Strand=Plus/Plus

Query  1     GTCCCACCGGGGTATCTTCAGCCGCCTCCTTGACAGTCTTCGGCAGGAAG  50
             |||||||||||||||||||||||||||||||||||| ||||||||| |||
Sbjct  1314  GTCCCACCGGGGTATCTTCAGCCGCCTCCTTGACAGCCTTCGGCAGCAAG  1363


>gb|HM486793.1| Lophocebus albigena DNA polymerase lambda (POLL) mRNA, partial 
cds
Length=1725

 Score = 82.4 bits (44),  Expect = 1e-13
 Identities = 48/50 (96%), Gaps = 0/50 (0%)
 Strand=Plus/Plus

Query  1     GTCCCACCGGGGTATCTTCAGCCGCCTCCTTGACAGTCTTCGGCAGGAAG  50
             |||||||||||||||||||||||||||||||||||| ||||||||| |||
Sbjct  1314  GTCCCACCGGGGTATCTTCAGCCGCCTCCTTGACAGCCTTCGGCAGCAAG  1363


>gb|HM486792.1| Hylobates agilis DNA polymerase lambda (POLL) mRNA, partial cds
Length=1722

 Score = 82.4 bits (44),  Expect = 1e-13
 Identities = 48/50 (96%), Gaps = 0/50 (0%)
 Strand=Plus/Plus

Query  1     GTCCCACCGGGGTATCTTCAGCCGCCTCCTTGACAGTCTTCGGCAGGAAG  50
             |||||| ||||||||||||||||||||||||||||||||||||||| |||
Sbjct  1311  GTCCCATCGGGGTATCTTCAGCCGCCTCCTTGACAGTCTTCGGCAGCAAG  1360


>dbj|AB179386.1| Macaca fascicularis testis cDNA clone: QtsA-18827, similar to 
human polymerase (DNA directed), lambda (POLL), mRNA, RefSeq: 
NM_013274.2
Length=2431

 Score = 82.4 bits (44),  Expect = 1e-13
 Identities = 48/50 (96%), Gaps = 0/50 (0%)
 Strand=Plus/Plus

Query  1     GTCCCACCGGGGTATCTTCAGCCGCCTCCTTGACAGTCTTCGGCAGGAAG  50
             |||||||||||||||||||||||||||||||||||| ||||||||| |||
Sbjct  1373  GTCCCACCGGGGTATCTTCAGCCGCCTCCTTGACAGCCTTCGGCAGCAAG  1422


Lambda     K      H
    1.33    0.621     1.12 

Gapped
Lambda     K      H
    1.28    0.460    0.850 

Effective search space used: 874911716973
 
 right 
 
 
    reads 
 chr10 chr10 103347104 103340061 73m6967n3m                   ||||||||||||||||||||||||||||||||||||||||||||||||||||||||||||||||||||||||||||||||||||||||||||||||||||||||||||||||||||||||||||||||||||||||||||||||||||||||||||||||||||||||||||||||||||||||||||||||||||||||||||||||||||||||||||||||||||||||||||||||CAG
chr10 chr10 103347101 103340058 70m6967n6m                   ||||||||||||||||||||||||||||||||||||||||||||||||||||||||||||||||||||||||||||||||||||||||||||||||||||||||||||||||||||||||||||||||||||||||||||||||||||||||||||||||||||||||||||||||||||||||||||||||||||||||||||||||||||||||||||||||||||||||||||||||CCAGAT
chr10 chr10 103347098 103340055 67m6967n9m                   ||||||||||||||||||||||||||||||||||||||||||||||||||||||||||||||||||||||||||||||||||||||||||||||||||||||||||||||||||||||||||||||||||||||||||||||||||||||||||||||||||||||||||||||||||||||||||||||||||||||||||||||||||||||||||||||||||||||||||||||||CCAGATGGC
chr10 chr10 103347093 103340050 62m6967n14m                  ||||||||||||||||||||||||||||||||||||||||||||||||||||||||||||||||||||||||||||||||||||||||||||||||||||||||||||||||||||||||||||||||||||||||||||||||||||||||||||||||||||||||||||||||||||||||||||||||||||||||||||||||||||||||||||||||||||||||||||||||CCAGATGGCTGGTC
chr10 chr10 103347082 103340039 51m6967n25m                  ||||||||||||||||||||||||||||||||||||||||||||||||||||||||||||||||||||||||||||||||||||||||||||||||||||||||||||||||||||||||||||||||||||||||||||||||||||||||||||||||||||||||||||||||||||||||||||||||||||||||||||||||||||||||||||||||||||||||||||||||CCAGATGGCTGGTCCCACCGGGGTA
chr10 chr10 103347077 103340034 46m6967n30m                  ||||||||||||||||||||||||||||||||||||||||||||||||||||||||||||||||||||||||||||||||||||||||||||||||||||||||||||||||||||||||||||||||||||||||||||||||||||||||||||||||||||||||||||||||||||||||||||||||||||||||||||||||||||||||||||||||||||||||||||||||CCAGATGGCTGGTCCCACCGGGGTATCTTC
chr10 chr10 103347074 103340031 43m6967n33m                  ||||||||||||||||||||||||||||||||||||||||||||||||||||||||||||||||||||||||||||||||||||||||||||||||||||||||||||||||||||||||||||||||||||||||||||||||||||||||||||||||||||||||||||||||||||||||||||||||||||||||||||||||||||||||||||||||||||||||||||||||CCAGATGGCTGGTCCCACCGGGGTATCTTCAGC
chr10 chr10 103347071 103340166 70m6829n6m                   ||||||||||||||||||||||||||||||||||||||||||||||||||||||||||||||||||||||||||||||||||||||||||||||||||||||||||||||||||||||||||||||||||||GTCCAG
chr10 chr10 103347063 103340158 62m6829n14m                  ||||||||||||||||||||||||||||||||||||||||||||||||||||||||||||||||||||||||||||||||||||||||||||||||||||||||||||||||||||||||||||||||||||GTCCAGAAAGCGGC
chr10 chr10 103347059 103340016 28m6967n48m                  ||||||||||||||||||||||||||||||||||||||||||||||||||||||||||||||||||||||||||||||||||||||||||||||||||||||||||||||||||||||||||||||||||||||||||||||||||||||||||||||||||||||||||||||||||||||||||||||||||||||||||||||||||||||||||||||||||||||||||||||||CCAGATGGCTGGTCCCACCGGGGTATCTTCAGCCGCCTCCTTGACAGT
chr10 chr10 103347056 103340151 55m6829n21m                  ||||||||||||||||||||||||||||||||||||||||||||||||||||||||||||||||||||||||||||||||||||||||||||||||||||||||||||||||||||||||||||||||||||GTCCAGAAAGCAGCCCAGGCC
chr10 chr10 103347051 103340146 50m6829n26m                  ||||||||||||||||||||||||||||||||||||||||||||||||||||||||||||||||||||||||||||||||||||||||||||||||||||||||||||||||||||||||||||||||||||GTCCAGAAAGCAGCCCAGGCCTTTAA
chr10 chr10 103347047 103340004 16m6967n60m                  ||||||||||||||||||||||||||||||||||||||||||||||||||||||||||||||||||||||||||||||||||||||||||||||||||||||||||||||||||||||||||||||||||||||||||||||||||||||||||||||||||||||||||||||||||||||||||||||||||||||||||||||||||||||||||||||||||||||||||||||||CCAGATGGCTGGTCCCACCGGGGTATCTTCAGCCGCCTCCTTGACAGTCTTCGGCAGGAA
chr10 chr10 103347021 103340116 20m6829n56m                  ||||||||||||||||||||||||||||||||||||||||||||||||||||||||||||||||||||||||||||||||||||||||||||||||||||||||||||||||||||||||||||||||||||GTCCAGAAAGCAGCCCAGGCCTTTAACTCTGGGCTGCTGTGTGTGGCATGTGGTTC
chr10 chr10 103347018 103340113 17m6829n59m                  ||||||||||||||||||||||||||||||||||||||||||||||||||||||||||||||||||||||||||||||||||||||||||||||||||||||||||||||||||||||||||||||||||||GTCCAGAAAGCAGCCCAGGCCTTTAACTCCGGGCTGCTGTGTGTGGCATGTGGTTCATA
chr10 chr10 103347013 103340108 12m6829n64m                  ||||||||||||||||||||||||||||||||||||||||||||||||||||||||||||||||||||||||||||||||||||||||||||||||||||||||||||||||||||||||||||||||||||GTCCAGAAAGCAGCCCAGGCCTTTAACTCCGGGCTGCTGTGTGTGGCATGTGGTTCATACCGAC
chr10 chr10 103347010 103340105 9m6829n67m                   ||||||||||||||||||||||||||||||||||||||||||||||||||||||||||||||||||||||||||||||||||||||||||||||||||||||||||||||||||||||||||||||||||||GTCCAGAAAGCAGCCCAGGCCTTTAACTCCGGGCTGCTGTGTGTGGCATGTGGTTCATACCGACGGG
chr10 chr10 103347005 103340100 4m6829n72m                   ||||||||||||||||||||||||||||||||||||||||||||||||||||||||||||||||||||||||||||||||||||||||||||||||||||||||||||||||||||||||||||||||||||GTCCAGAAAGCAGCCCAGGCCTTTAACTCCGGGCTGCTGTGTGTGGCATGTGGTTCATACCGACGGGGAAAG
chr10 chr10 103346946 103340165 69m6705n7m                   ||||||||||||||||||||||||||||||||||||||||||||||||||||||||||||||||||||||||||||||||||||||||||||||||||||||||||||||||||||||||||||||||||||GTCCAGA
chr10 chr10 103346931 103340150 54m6705n22m                  ||||||||||||||||||||||||||||||||||||||||||||||||||||||||||||||||||||||||||||||||||||||||||||||||||||||||||||||||||||||||||||||||||||GTCCAGAAAGCAGCCCAGGCCT
chr10 chr10 103345099 103340124 28m4899n48m                  ||||||||||||||||||||||||||||||||||||||||||||||||||||||||||||||||||||||||||||||||||||||||||||||||||||||||||||||||||||||||||||||||||||GTCCAGAAAGCAGCCCAGGCCTTTAACTCCGGGCTGCTGTGTGTGGCA
chr10 chr10 103342593 103340171 75m2346n1m                   ||||||||||||||||||||||||||||||||||||||||||||||||||||||||||||||||||||||||||||||||||||||||||||||||||||||||||||||||||||||||||||||||||||G
chr10 chr10 103342590 103340168 72m2346n4m                   ||||||||||||||||||||||||||||||||||||||||||||||||||||||||||||||||||||||||||||||||||||||||||||||||||||||||||||||||||||||||||||||||||||GTCC
chr10 chr10 103342587 103340165 69m2346n7m                   ||||||||||||||||||||||||||||||||||||||||||||||||||||||||||||||||||||||||||||||||||||||||||||||||||||||||||||||||||||||||||||||||||||GTCCAGA
chr10 chr10 103342584 103340162 66m2346n10m                  ||||||||||||||||||||||||||||||||||||||||||||||||||||||||||||||||||||||||||||||||||||||||||||||||||||||||||||||||||||||||||||||||||||GTCCAGAAAG
chr10 chr10 103342581 103340159 63m2346n13m                  ||||||||||||||||||||||||||||||||||||||||||||||||||||||||||||||||||||||||||||||||||||||||||||||||||||||||||||||||||||||||||||||||||||GTCCAGAAAGCAG
chr10 chr10 103342578 103340156 60m2346n16m                  ||||||||||||||||||||||||||||||||||||||||||||||||||||||||||||||||||||||||||||||||||||||||||||||||||||||||||||||||||||||||||||||||||||GTCCAGAAAGCAGCCC
chr10 chr10 103342575 103340153 57m2346n19m                  ||||||||||||||||||||||||||||||||||||||||||||||||||||||||||||||||||||||||||||||||||||||||||||||||||||||||||||||||||||||||||||||||||||GTCCAGAAAGCAGCCCAGG
chr10 chr10 103342572 103340150 54m2346n22m                  ||||||||||||||||||||||||||||||||||||||||||||||||||||||||||||||||||||||||||||||||||||||||||||||||||||||||||||||||||||||||||||||||||||GTCCAGAAAGCAGCCCAGGCCT
chr10 chr10 103342569 103340147 51m2346n25m                  ||||||||||||||||||||||||||||||||||||||||||||||||||||||||||||||||||||||||||||||||||||||||||||||||||||||||||||||||||||||||||||||||||||GTCCAGAAAGCAGCCAAGGCCTTTA
chr10 chr10 103342564 103340142 46m2346n30m                  ||||||||||||||||||||||||||||||||||||||||||||||||||||||||||||||||||||||||||||||||||||||||||||||||||||||||||||||||||||||||||||||||||||GTCCAGAAAGCAGCCCAGGCCTTTAACTCC
chr10 chr10 103342561 103340139 43m2346n33m                  ||||||||||||||||||||||||||||||||||||||||||||||||||||||||||||||||||||||||||||||||||||||||||||||||||||||||||||||||||||||||||||||||||||GTCCAGAAAGCAGCCCAGGCCTTTAACTCTGGG
chr10 chr10 103342557 103340135 39m2346n37m                  ||||||||||||||||||||||||||||||||||||||||||||||||||||||||||||||||||||||||||||||||||||||||||||||||||||||||||||||||||||||||||||||||||||GTCCAGAAAGCAGCCCAGGCCTTTAACTCCGGGCTGC
chr10 chr10 103342553 103340131 35m2346n41m                  ||||||||||||||||||||||||||||||||||||||||||||||||||||||||||||||||||||||||||||||||||||||||||||||||||||||||||||||||||||||||||||||||||||GTCCAGAAAGCAGCCCAGGCCTTTAACTCCGGGCTGCTGTG
chr10 chr10 103342549 103340127 31m2346n45m                  ||||||||||||||||||||||||||||||||||||||||||||||||||||||||||||||||||||||||||||||||||||||||||||||||||||||||||||||||||||||||||||||||||||GTCCAGAAAGCAGCCCAGGCCTTTAACTCCGGGCTGCTGTGTGTG
chr10 chr10 103342546 103340124 28m2346n48m                  ||||||||||||||||||||||||||||||||||||||||||||||||||||||||||||||||||||||||||||||||||||||||||||||||||||||||||||||||||||||||||||||||||||GTCCAGAAAGCAGCCCAGGCCTTTAACTCCGGGCTGCTGTGTGTGGCA
chr10 chr10 103342541 103340119 23m2346n53m                  ||||||||||||||||||||||||||||||||||||||||||||||||||||||||||||||||||||||||||||||||||||||||||||||||||||||||||||||||||||||||||||||||||||GTCCAGAAAGCAGCCCAGGCCTTTAACTCTGGGCTGCTGTGTGTGGCATGTGG
chr10 chr10 103342538 103340116 20m2346n56m                  ||||||||||||||||||||||||||||||||||||||||||||||||||||||||||||||||||||||||||||||||||||||||||||||||||||||||||||||||||||||||||||||||||||GTCCAGAAAGCAGCCCAGGCCTTTAACTCCGGGCTGCTGTGTGTGGCATGTGGTTC
chr10 chr10 103342535 103340113 17m2346n59m                  ||||||||||||||||||||||||||||||||||||||||||||||||||||||||||||||||||||||||||||||||||||||||||||||||||||||||||||||||||||||||||||||||||||GTCCAGAAAGCAGCCCAGGCCTTTAACTCCGGGCTGCTGTGTGTGGCATGTGGTTCATA
chr10 chr10 103342532 103340110 14m2346n62m                  ||||||||||||||||||||||||||||||||||||||||||||||||||||||||||||||||||||||||||||||||||||||||||||||||||||||||||||||||||||||||||||||||||||GTCCAGAAAGCAGCCCAGGCCTTTAACTCCGGGCTGCTGTGTGTGGCATGTGGTTCATACCG
chr10 chr10 103342528 103340106 10m2346n66m                  ||||||||||||||||||||||||||||||||||||||||||||||||||||||||||||||||||||||||||||||||||||||||||||||||||||||||||||||||||||||||||||||||||||GTCCAGAAAGCAGCCCAGGCCTTTAACTCCGGGCTGCTGTGTGTGGCATGTGGTTCATACCGACGG
chr10 chr10 103342525 103340103 7m2346n69m                   ||||||||||||||||||||||||||||||||||||||||||||||||||||||||||||||||||||||||||||||||||||||||||||||||||||||||||||||||||||||||||||||||||||GTCCAGAAAGCAGCCCAGGCCTTTAACTCCGGGCTGCTGTGTGTGGCATGTGGTTCATACCGACGGGGA
chr10 chr10 103342522 103340100 4m2346n72m                   ||||||||||||||||||||||||||||||||||||||||||||||||||||||||||||||||||||||||||||||||||||||||||||||||||||||||||||||||||||||||||||||||||||GTCCAGAAAGCAGCCCAGGCCTTTAACTCCGGGCTGCTGTGTGTGGCATGTGGTTCATACCGACGGGGAAAG
chr10 chr10 103342519 103340097 1m2346n75m                   ||||||||||||||||||||||||||||||||||||||||||||||||||||||||||||||||||||||||||||||||||||||||||||||||||||||||||||||||||||||||||||||||||||GTCCAGAAAGCAGCCCAGGCCTTTAACTCCGGGCTGCTGTGTGTGGCATGTGGTTCATACCGACGGGGAAAGACG
chr10 chr10 103340957 103340060 72m821n4m                    ||||||||||||||||||||||||||||||||||||||||||||||||||||||||||||||||||||||||||||||||||||||||||||||||||||||||||||||||||||||||||||||||||||||||||||||||||||||||||||||||||||||||||||||||||||||||||||||||||||||||||||||||||||||||||||||||||||||||||||||||CCAG
chr10 chr10 103340950 103340053 65m821n11m                   ||||||||||||||||||||||||||||||||||||||||||||||||||||||||||||||||||||||||||||||||||||||||||||||||||||||||||||||||||||||||||||||||||||||||||||||||||||||||||||||||||||||||||||||||||||||||||||||||||||||||||||||||||||||||||||||||||||||||||||||||CCAGATGGCTG
chr10 chr10 103340928 103340031 43m821n33m                   ||||||||||||||||||||||||||||||||||||||||||||||||||||||||||||||||||||||||||||||||||||||||||||||||||||||||||||||||||||||||||||||||||||||||||||||||||||||||||||||||||||||||||||||||||||||||||||||||||||||||||||||||||||||||||||||||||||||||||||||||CCAGATGGCTGGTCCCACCGGGGTATCTTCAGC
chr10 chr10 103340908 103340011 23m821n53m                   ||||||||||||||||||||||||||||||||||||||||||||||||||||||||||||||||||||||||||||||||||||||||||||||||||||||||||||||||||||||||||||||||||||||||||||||||||||||||||||||||||||||||||||||||||||||||||||||||||||||||||||||||||||||||||||||||||||||||||||||||CCAGATGGCTGGTCCCACCGGGGTATCTTCAGCCGCCTCCTTGACAGTCTTCG
chr10 chr10 103340392 103340033 45m283n31m                   ||||||||||||||||||||||||||||||||||||||||||||||||||||||||||||||||||||||||||||||||||||||||||||||||||||||||||||||||||||||||||||||||||||||||||||||||||||||||||||||||||||||||||||||||||||||||||||||||||||||||||||||||||||||||||||||||||||||||||||||||CCAGATGGCTGGTCCCACCGGGGTATCTTCA
chr10 chr10 103340348 128799858 1m283n61m128799873F14m       ||||||||||||||||||||||||||||||||||||||||||||||||||||||||||||||||||||||||||||||||||||||||||||||||||||||||||||||||||||||||||||||||||||||||||||||||||||||||||||||||||||||||||||||||||||||||||||||||||||||||||||||||||||||||||||||||||||||||||||||||CCAGATGGCTGGTCCCACCGGGGTATCTTCAGCCGCCTCCTTGACAGTCTTCGGCAGGAAG GCGGAGCACAGAGG
chr10 chr10 103340348 128799858 1m283n61m128799873F14m       ||||||||||||||||||||||||||||||||||||||||||||||||||||||||||||||||||||||||||||||||||||||||||||||||||||||||||||||||||||||||||||||||||||||||||||||||||||||||||||||||||||||||||||||||||||||||||||||||||||||||||||||||||||||||||||||||||||||||||||||||CCAGATGGCTGGTCCCACCGGGGTATCTTCAGCCGCCTCCTTGACAGTCTTCGGCAGGAAG GCGGAGCACAGAGG
chr10 chr10 103340343 103340267 76m                          TGGGCTTGCAGCAGGCATCTGGAATGGGCATAAGCTG
chr10 chr10 103340327 103340251 76m                          TGGGCTTGCAGCAGGCATCTGGAATGGGCATAAGCTGAGGGTGTGGGCCCGAG
chr10 chr10 103340250 103340174 76m                                                                                CTGGGAAGGCCCTTGTGCTGCTGTGGGAGAGGAGCTCCCCACGCACAGCCCCTCACCTGTCACCGCTCTCTTCCCC
chr10 chr10 103340212 103340136 76m                                                                                                                      CCACGCACAGCCCCTCACCTGTCACCGCTCTCTTCCCCAGGTCCAGAAAGCAGCCCAGGCCTTTAACTCCGGGCTG
chr10 chr10 103340199 103340123 76m                                                                                                                                   CTCACCTGTCACCGCTCTCTTCCCCAGGTCCAGAAAGCAGCCCAGGCCTTTAACTCCGGGCTGCTGTGTGTGGCAT
chr10 chr10 103340188 103340112 76m                                                                                                                                              CCGCTCTCTTCCCCAGGTCCAGAAAGCAGCCCAGGCCTTTAACTCCGGGCTGCTGTGTGTGGCATGTGGTTCATAC
chr10 chr10 103340177 103340101 76m                                                                                                                                                         CCCAGGTCCAGAAAGCAGCCCAGGCCTTTAACTCCGGGCTGCTGTGTGTGGCATGTGGTTCATACCGACGGGGAAA
chr10 chr10 103340174 103340098 76m                                                                                                                                                            TGGTCCAGAAAGCAGCCCAGGCCTTTAACTCCGGGCTGCTGTGTGTGGCATGTGGTTCATACCGACGGGGAAAGGC
chr10 chr10 103340171 103340095 76m                                                                                                                                                               TCCAGAAAGCAGCCCAGGCCTTTAACTCCGGGCTGCTGTGTGTGGCATGTGGTTCATACCGACGGGGAAAGGCGAC
chr10 chr10 103340168 103340092 76m                                                                                                                                                                  AGAAAGCAGCCCAGGCCTTTAACTCCGGGCTGCTGTGTGTGGCATGTGGTTCATACCGACGGGGAAAGGCGACCTG
chr10 chr10 103340165 103340089 76m                                                                                                                                                                     AAGCAGCCCAGGCCTTTAACTCCGGGCTGCTGTGTGTGGCATGTGGTTCATACCGACGGGGAAAGGCGACCTGTGG
chr10 chr10 103340160 103340084 76m                                                                                                                                                                          GCCCAGGCCTTTAACTCCGGGCTGCTGTGTGTGGCATGTGGTTCATACCGACGGGGAAAGGCGACCTGTGGTGATG
chr10 chr10 103340157 103340081 76m                                                                                                                                                                             CAGGCCTTTAACTCCGGGCTGCTGTGTGTGGCATGTGGTTCATACCGACGGGGAAAGGCGCCCTGTGGTGATGTCG
chr10 chr10 103340154 103340078 76m                                                                                                                                                                                GCCTTTAACTCCGGGCTGCTGTGTGTGGCATGTGGTTCATACCTACGGGGAAAGGCGACCTGTGGTGATGTCGACG
chr10 chr10 103340151 103340075 76m                                                                                                                                                                                   TTTAACTCCGGGCTGCTGTGTGTGGCATGTGGTTCATACCGACGGGGAAAGGCGACCTGTGGTGATGTCGACGTGC
chr10 chr10 103340148 103340072 76m                                                                                                                                                                                      AACTCCGGGCTGCTGTGTGTGGCATGTGGTTCATACCGACGGGGAAAGGCGACCTGTGGTGATGTCGACGTGCTCA
chr10 chr10 103340144 103340068 76m                                                                                                                                                                                          CTGGGCTGCTGTGTGTGGCATGTGGTTCATACCGACGGGGAAAGGCGACCTGTGGTGATGTCGACGTGCTCATCAC
chr10 chr10 103340139 103340063 76m                                                                                                                                                                                               CTGCTGTGTGTGGCATGTGGTTCATACCGACGGGGAAAGGCGACCTGTGGTGATGTCGACGTGCTCATCACTCACC
chr10 chr10 103340136 103340060 76m                                                                                                                                                                                                  CTGTGTGTGGCATGTGGTTCATACCGACGGGGAAAGGCGACCTGTGGTGATGTCGACGTGCTCATCTCTCACCCAG
chr10 chr10 103340132 103340056 76m                                                                                                                                                                                                      GTGTGGCATGTGGTTCATACCGACGGGGAAAGGCGACCTGTGGGGATGTCGACGTGCTCATCACTCACCCAGATGG
chr10 chr10 103340129 103340053 76m                                                                                                                                                                                                         TGGCATGTGGTTCATACCGACGGGGAAAGGCGACCTGTGGTGATGTCGACGTGCTCATCACTCACCCAGATGGCCG
chr10 chr10 103340126 103340050 76m                                                                                                                                                                                                            CATGTGGTTCATACCGACGGGGAAAGGCGACCTGTGGTGATGTCGACGTGCTCATCACTCACCCAGATGGCTGGTC
chr10 chr10 103340123 103340047 76m                                                                                                                                                                                                               GTGGTTCATACCGACGGGGAAAGGCGACCTGTGGTGATGTCGACGTGCTCATCACTCACCCAGATGGCTGGTCCCA
chr10 chr10 103340120 103340044 76m                                                                                                                                                                                                                  GTTCATACCGACGGGGAAAGGCGACCTGTGGTGATGTCGACGTGCTCATCACTCACCCAGATGGCTGGTCCCACCG
chr10 chr10 103340117 103340041 76m                                                                                                                                                                                                                     CATACCGACGGGGAAAGGCGACCTGTGGTGATGTCGACGTGCTCATCACTCACCCAGATGGCTGGTCCCACCGGGG
chr10 chr10 103340113 103340037 76m                                                                                                                                                                                                                         CCGACGGGGAAAGGCGACCTGTGGTGATGTCGACGTGCTCATCACTCACCCAGATGGCCGGTCCCACCGGGGTATC
chr10 chr10 103340110 103340034 76m                                                                                                                                                                                                                            ACGGGGAAAGGCGACCTGTGGTGATGTCGACGTGCTCATCACTCACCCAGATGGCTGGTCCCACCGGGGTATCTTC
chr10 chr10 103340107 103340031 76m                                                                                                                                                                                                                               GGGAAAGGCGACCTGTGGTGATGTCGACGTGCTCATCACTCACCCAGATGGCTGGTCCCACCGGGGTATCTTCAGC
chr10 chr10 103340104 103340028 76m                                                                                                                                                                                                                                  AAAGGCGACCTGTGGTGATGTCGACGTGCTCATCACTCACCCAGATGGCTGGTCCCACCGGGGTATCTTCAGCCGC
chr10 chr10 103340101 103340025 76m                                                                                                                                                                                                                                     GGCGACCTGTGGTGATGTCGACGTGCTCATCACTCACCCAGATGGCTGGTCCCACCGGGGTATCTTCAGCCGCCTC
chr10 chr10 103340097 103340021 76m                                                                                                                                                                                                                                         ACCTGTGGTGATGTCGACGTGCTCATCACTCACCCAGATGGCTGGTCCCACCGGGGTATCTTCAGCCGCCTCCTTG
chr10 chr10 103340091 103340015 76m                                                                                                                                                                                                                                               GGTGATGTCGACGTGCTCATCACTCACCCAGATGGCTGGTCCCACCGGGGTATCTTCAGCCGCCTCCTTGACAGTA
chr10 chr10 103340088 103340012 76m                                                                                                                                                                                                                                                  GATGTCGACGTGCTCATCACTCACCCAGATGGCTGGTCCCACCGGGGTATCTTCAGCCGCCTCCTTGACAGTCTTC
chr10 chr10 103340085 103340009 76m                                                                                                                                                                                                                                                     GTCGACGTGCTCATCACTCACCCAGATGGCTGGTCCCACCGGGGTATCTTCAGCCGCCTCCTTGACAGTCTTCGGC
chr10 chr10 103340082 103340006 76m                                                                                                                                                                                                                                                        GACGTGCTCATCACTCACCCAGATGGCCGGTCCCACCGGGGTATCTTCAGCCGCCTCCTTGACAGTCTTCGGCAGG
chr10 chr10 103340079 103340003 76m                                                                                                                                                                                                                                                           GTGCTCATCACTCACCCAGATGGCTGGTCCCACCGGGGAATCTTCAGCCGCCTCCTTGACAGTCTTCGGCAGGAAG
chr10 chr10 103340076 103340000 76m                                                                                                                                                                                                                                                              CTCATCACTCACCCAGATGGCCGGTCCCACCGGGGTATCTTCAGCCGCCTCCTTGACAGTCTTCGGCAGGAAGGGT
chr10 chr10 103340072 103339996 76m                                                                                                                                                                                                                                                                  TCACTCACCCAGATGGCTGGTCCCACCGGGGTATCTTCAGCCGCCTCCTTGACAGTCTTCGGCAGGAAGGTATAAG
chr10 chr10 103340063 128799856 60m128799873F16m                                                                                                                                                                                                                                                              CAGATGGCTGGTCCCACCGGGGTATCTTCAGCCGCCTCCTTGACAGACTTCGGCAGGAAG GCGGAGCACAGAGGAC
chr10 chr10 103340063 128799856 60m128799873F16m                                                                                                                                                                                                                                                              CAGATGGCTGGTCCCACCGGGGTATCTTCAGCCGCCTCCTTGACAGTCTTCGGCAGGAAG GCGGAGCACAGAGGAC
chr10 chr10 103340061 128799854 58m128799873F18m                                                                                                                                                                                                                                                                GATGGCTGGTCCCACCGGGGTATCTTCAGCCGCCTCCTTGACAGTCTTCGGCAGGAAG GCGGAGTACAGAGGACTT
chr10 chr10 103340061 128799854 58m128799873F18m                                                                                                                                                                                                                                                                GATGGCTGGTCCCACCGGGGTATCTTCAGCCGCCTCCTTGACAGTCTTCGGCAGGAAG GCGGAGTACAGAGGACTT
chr10 chr10 103340058 128799851 55m128799873F21m                                                                                                                                                                                                                                                                   GGCTGGTCCCACCGGGGTATCTTCAGCCGCCTCCTTGACAGTCTTCGGCAGGAAG GCGGAGCACAGAGGACTTTTA
chr10 chr10 103340058 128799851 55m128799873F21m                                                                                                                                                                                                                                                                   GGCTGGTCCCACCGGGGTATCTTCAGCCGCCTCCTTGACAGTCTTCGGCAGGAAG GCGGAGCACAGAGGACTTTTA
chr10 chr10 103340058 128799851 55m128799873F21m                                                                                                                                                                                                                                                                   GGCTGGTCCCACCGGGGTATCTTCAGCCGCCTCCTTGACAGTCTTCGGCAGGAAG GCGGAGCACAGAGGACTTTTA
chr10 chr10 103340057 128799850 54m128799873F22m                                                                                                                                                                                                                                                                    GCTGGTCCCACCGGGGTATCTTCAGCCGCCTCCTTGACAGTCTTCGGCAGGAAG GCGGACCACAGAGGACTTTTAG
chr10 chr10 103340057 128799850 54m128799873F22m                                                                                                                                                                                                                                                                    GCTGGTCCCACCGGGGTATCTTCAGCCGCCTCCTTGACAGTCTTCGGCAGGAAG GCGGAGCACAGAGGACTTTTAG
chr10 chr10 103340057 128799850 54m128799873F22m                                                                                                                                                                                                                                                                    GCTGGTCCCACCGGGGTATCTTCAGCCGCCTCCTTGACAGTCTTCGGCAGGAAG GCGGAGCACAGAGGACTTTTAG
chr10 chr10 103340056 128799849 53m128799873F23m                                                                                                                                                                                                                                                                     CTGGTCCCACCGGGGTATCTTCAGCCGCCTCCTTGACAGTCTTCGGCAGGAAG GCGGAGCACAGAGGACTTTTAGG
chr10 chr10 103340053 128799846 50m128799873F26m                                                                                                                                                                                                                                                                        GTCCCACCGGGGTATCTTCAGCCGCCTCCTTGACAGTCTTCGGCAGGAAG GCGGAGCACAGAGGACTTTTAGGATG
chr10 chr10 103340053 128799846 50m128799873F26m                                                                                                                                                                                                                                                                        GTCCCACCGGGGTATCTTCAGCCGCCTCCTTGACAGTCTTCGGCAGGAAG GCGGAGCACAGAGGACTTTTAGGATG
chr10 chr10 103340052 128799845 49m128799873F27m                                                                                                                                                                                                                                                                         TCCCACCGGGGTATCTTCAGCCGCCTCCTTGACAGTCTTCGGCAGGAAG GCGGAGCACAGAGGACTTTTAGGATGG
chr10 chr10 103340052 128799845 49m128799873F27m                                                                                                                                                                                                                                                                         TCCCACCGGGGTATCTTCAGCCGCCTCCTTGACAGTCTTCGGCNGGAAG GCGGAGCACAGAGGACTTTTANGATGG
chr10 chr10 103340052 128799845 49m128799873F27m                                                                                                                                                                                                                                                                         TCCCACCGGGGTATCTTCAGCCGCCTCCTTGACAGTCTTCGGCAGGAAG GCGGAGCACAGAGGACTTTTAGGATGG
chr10 chr10 103340050 128799843 47m128799873F29m                                                                                                                                                                                                                                                                           CCACCGGGGTATCTTCAGCCGCCTCCTTGACAGTCTTCGGCAGGAAG GCGGAGCACAGAGGACTTTTAGGATGGTG
chr10 chr10 103340050 128799843 47m128799873F29m                                                                                                                                                                                                                                                                           CCACCGGGGTATCTTCAGCCGCCTCCTTGACAGTCTTCGGCAGGAAG GCGGGGCACAGAGGACTTTTAGGATGGTG
chr10 chr10 103340049 128799842 46m128799873F30m                                                                                                                                                                                                                                                                            CACCGGGGTATCTTCAGCCGCCTCCTTGACAGTCTTCGGCAGGAAG GCGGAGCACGGAGGACTTTTAGGATGGTGA
chr10 chr10 103340048 128799841 45m128799873F31m                                                                                                                                                                                                                                                                             ACCGGGGTATCTTCAGCCGCCTCCTTGACAGTCTTCGGCAGGAAG GCGGAGCACAGAGGACTTTTAGGATGGTGAA
chr10 chr10 103340047 128799840 44m128799873F32m                                                                                                                                                                                                                                                                              CCGGGGTATCTTCAGCCGCCTCCTTGACAGTCTTCGGCAGGAAG GCGGAGCACAGAGGACTTTTAGGATGGTGAAG
chr10 chr10 103340047 128799840 44m128799873F32m                                                                                                                                                                                                                                                                              CCGGGGTATCTTCAGCCGCCTCCTTGACAGTCTTCGGCAGGAAG GCGGAGCACAGAGGACTTTTAGGATGGTGAAG
chr10 chr10 103340047 128799840 44m128799873F32m                                                                                                                                                                                                                                                                              CCGGGGTATCTTCAGCCGCCTCCTTGACAGTCTTCGGCAGGAAG GCGGAGCACAGAGGACTTTTAGGATGGTGAAG
chr10 chr10 103340047 128799840 44m128799873F32m                                                                                                                                                                                                                                                                              CCGGGGTATCTTCAGCCGCCTCCTTGACAGTCTTCGGCAGGAAG GCGGAGCACAGAGGACTTTTAGGATGGTGAAG
chr10 chr10 103340047 128799840 44m128799873F32m                                                                                                                                                                                                                                                                              CCGGGGTATCTTCAGCCGCCTCCTTGACAGTCTTCGGCAGGAAG GCGGAGCACAGAGGACTTTTAGGATGGTGAAG
chr10 chr10 103340047 128799840 44m128799873F32m                                                                                                                                                                                                                                                                              CCGGGGTATCTTCAGCCGCCTCCTTGACAGTCTTCGGCAGGAAG GCGGAGCACAGAGGACTTTTAGGATGGTGAAG
chr10 chr10 103340047 128799840 44m128799873F32m                                                                                                                                                                                                                                                                              CCGGGGTATCTTCAGCCGCCTCCTTGACAGTCTTCGGCAGGAAG GCGGAGCACAGAGGACTTTTAGGATGGTGAAG
chr10 chr10 103340047 128799840 44m128799873F32m                                                                                                                                                                                                                                                                              CCGGGGTATCTTCAGCCGCCTCCTTGACAGTCTTCGGCAGGAAG GCGGAGCACAGAGGACTTTTAGGATGGTGAAG
chr10 chr10 103340047 128799840 44m128799873F32m                                                                                                                                                                                                                                                                              CCGGGGTATCTTCAGCCGCCTCCTTGACAGTCTTCGGCAGGAAG GCGGAGCACAGAGGACTTTTAGGATGGTGAAG
chr10 chr10 103340047 128799840 44m128799873F32m                                                                                                                                                                                                                                                                              CCGGGGTATCTTCAGCCGCCTCCTTGACAGTCTTCGGCAGGAAG GCGGAGCACAGAGGACTTTTAGGATGGTGAAG
chr10 chr10 103340046 128799839 43m128799873F33m                                                                                                                                                                                                                                                                               CGGGGTATCTTCAGCCGCCTCCTTGACAGTCTTCGGCAGGAAG GCGGAGCACAGAGGACTTTTAGGATGGTGAAGC
chr10 chr10 103340046 128799839 43m128799873F33m                                                                                                                                                                                                                                                                               CGGGGTATCTTCAGCCGCCTCCTTGACAGTCTTCGGCAGGAAG GCGGAGCACAGAGGACTTTTAGGATGGTGAAGC
chr10 chr10 103340046 128799839 43m128799873F33m                                                                                                                                                                                                                                                                               CGGGGTATCTTCAGCCGCCTCCTTGACAGTCTTCGGCAGGAAG GCGGAGCACAGAGGACTTTTAGGATGGTGAAGC
chr10 chr10 103340043 128799836 40m128799873F36m                                                                                                                                                                                                                                                                                  GGTATCTTCAGCCGCCTCCTTGACAGTCTTCGGCAGGAAG GCGGAGCACAGAGGACTTTTAGGATGGTGAAGCTAC
chr10 chr10 103340043 128799836 40m128799873F36m                                                                                                                                                                                                                                                                                  GGTATCTTCAGCCGCCTCCTTGACAGTCTTCGTCAGGAAG GCGTAGCACAGAGGACTTTTAGGATGGTGAAGCTAC
chr10 chr10 103340043 128799836 40m128799873F36m                                                                                                                                                                                                                                                                                  GGTATCTTCAGCCGCCTCCTTGACAGTCTTCGTCAGGAAG GCGTAGCACAGAGGACTTTTAGGATGGTGAAGCTAC
chr10 chr10 103340043 128799836 40m128799873F36m                                                                                                                                                                                                                                                                                  GGTATCTTCAGCCGCCTCCTTGACAGTCTTCGGCAGGAAG GCGGAGCACAGAGGACTTTTAGGATGGTGAAGCTAC
chr10 chr10 103340042 128799835 39m128799873F37m                                                                                                                                                                                                                                                                                   GTATCTTCAGCCGCCTCCTTGACAGTCTTCGGCAGGAAG GCGGAGCACAGAGGACTTTTAGGATGGTGAAGCTACC
chr10 chr10 103340042 128799835 39m128799873F37m                                                                                                                                                                                                                                                                                   GTATCTTCAGCCGCCTCCTTGACAGTCTTCGGCAGGAAG GCGGAGCACAGAGGACTTTTAGGATGGTGAAGCTACC
chr10 chr10 103340042 128799835 39m128799873F37m                                                                                                                                                                                                                                                                                   GTATCTTCAGCCGCCTCCTTGACAGTCTTCGGCAGGAAG GCGGAGCACAGAGGACTTTTAGGATGGTGAAGCTACC
chr10 chr10 103340039 128799832 36m128799873F40m                                                                                                                                                                                                                                                                                      TCTTCAGCCGCCTCCTTGACAGTCTTCGGCAGGAAG GCGGAGCACAGAGGACTTTTAGGATGGTGAAGCTAACCAG
chr10 chr10 103340039 128799832 36m128799873F40m                                                                                                                                                                                                                                                                                      TCTTCAGCCGCCTCCTTGACAGTCTTCGGCAGGAAG GCGGAGCACAGAGGACTTTTAGGATGGTGAAGCTACCCAG
chr10 chr10 103340039 128799832 36m128799873F40m                                                                                                                                                                                                                                                                                      TCTTCAGCCGCCTCCTTGACAGTCTTCGGCAGGAAG GCGGAGCACAGAGGACTTTTAGGATGGTGAAGCTACCCAG
chr10 chr10 103340039 128799832 36m128799873F40m                                                                                                                                                                                                                                                                                      TCTTCAGCCGCCTCCTTGACAGTCTTCGGCAGGAAG GCGGAGCACAGAGGACTTTTAGGATGGTGAAGCTACCCAG
chr10 chr10 103340039 128799832 36m128799873F40m                                                                                                                                                                                                                                                                                      TCTTCAGCCGCCTCCTTGACAGTCTTCGGCAGGAAG GCGGAGCACAGAGGACTTTTAGGATTGTGAAGCTACCCAG
chr10 chr10 103340038 128799831 35m128799873F41m                                                                                                                                                                                                                                                                                       CTTCAGCCGCCTCCTTGACAGTCTTCGGCAGGAAG GCGGAGCCCAGAGGACTTTTAGGATGGTGAAGCTACCCAGG
chr10 chr10 103340038 128799831 35m128799873F41m                                                                                                                                                                                                                                                                                       CTTCAGCCGCCTCCTTGACAGTCTTCGGCAGGAAG GCGGAGCACAGAGGACTTTTAGGATGGTGAAGCTACCCAGT
chr10 chr10 103340038 128799831 35m128799873F41m                                                                                                                                                                                                                                                                                       CTTCAGCCGCCTCCTTGACAGTCTTCGGCAGGAAG GCGGAGCACAGAGGACTTTTAGGATGGTGAAGCTACCCAGT
chr10 chr10 103340038 128799831 35m128799873F41m                                                                                                                                                                                                                                                                                       CTTCAGCCGCCTCCTTGACAGTCTTCGGCAGGAAG GCGGAGCACAGAGGACTTTTAGGATGGTGAAGCTACCCAGT
chr10 chr10 103340037 128799830 34m128799873F42m                                                                                                                                                                                                                                                                                        TTCAGCCGCCTCCTTGACAGTCTTCGGCAGGAAG GCGGAGCACAGAGGACTTTTAGGATGGTGAAGCTACCCAGTG
chr10 chr10 103340037 128799830 34m128799873F42m                                                                                                                                                                                                                                                                                        TTCAGCCGCCTCCTTGACAGTCTTCGGCAGGAAG GCGGAGCACAGAGGACTTTTAGGATGGTGAAGCTACCCAGTG
chr10 chr10 103340035 128799828 32m128799873F44m                                                                                                                                                                                                                                                                                          CAGCCGCCTCCTTGACAGTCTTCGGCAGGAAG GCGGAGCACAGAGGACTTTTAGGATGGTGAAGCTACCCAGTGAG
chr10 chr10 103340035 128799828 32m128799873F44m                                                                                                                                                                                                                                                                                          CAGCCGCCTCCTTGACAGTCTTCGGCAGGAAG GCGGAGCACAGAGGACTTTTAGGATGGTGAAGCTACCCAGTGAG
chr10 chr10 103340033 128799826 30m128799873F46m                                                                                                                                                                                                                                                                                            GCCGCCTCCTTGACAGTCTTCGGCAGGAAG GCGGAGCACAGAGGACTTTTAGGATGGTGAAGCTACCCAGTGAGAG
chr10 chr10 103340033 128799826 30m128799873F46m                                                                                                                                                                                                                                                                                            GCCGCCTCCTTGACAGTCTTCGGCAGGAAG GCGGAGCACAGAGGACTTTTAGGATGGTGAAGCTACCCAGTGAGAG
chr10 chr10 103340033 128799826 30m128799873F46m                                                                                                                                                                                                                                                                                            GCCGCCTCCTTGACAGTCTTCGGCAGGAAG GCGGAGCACAGAGGACTTTTAGGATGGTGAAGCTACCCAGTGAGAG
chr10 chr10 103340033 128799826 30m128799873F46m                                                                                                                                                                                                                                                                                            GCCGCCTCCTTGACAGTCTTCGGCAGGAAG GCGGAGCACAGAGGACTTTTAGGATGGTGAAGCTACACAGTGAGAG
chr10 chr10 103340033 128799826 30m128799873F46m                                                                                                                                                                                                                                                                                            GCCGCCTCCTTGACAGTCTTCGGCAGGAAG GCGGAGCACAGAGGACTTTTAGGATGGTGAAGCTACCCAGTGAGAG
chr10 chr10 103340033 128799826 30m128799873F46m                                                                                                                                                                                                                                                                                            GCCGCCTCCTTGACAGTCTTCGGCAGGAAG GCGGAGCACAGAGGACTTTTAGGATGGTGAAGCTACCCAGTGAGAG
chr10 chr10 103340033 128799826 30m128799873F46m                                                                                                                                                                                                                                                                                            GCCGCCTCCTTGACAGTCTTCGGCAGGAAG GCGGAGCACAGAGGACTTTTAGGATGGTGAAGCTACCCAGTGAGAG
chr10 chr10 103340033 128799826 30m128799873F46m                                                                                                                                                                                                                                                                                            GCCGCCTCCTTGACAGTCTTCGGCAGGAAG GCGGAGCACAGCGGACTTTTAGGATGGTGAAGCTACCCAGTGAGAG
chr10 chr10 103340033 128799826 30m128799873F46m                                                                                                                                                                                                                                                                                            GCCGCCTCCTTGACAGTCTTCGGCAGGAAG GCGGAGCACAGAGGACTTTTAGGATGGTGAAGCGACCCAGTGAGAG
chr10 chr10 103340031 128799824 28m128799873F48m                                                                                                                                                                                                                                                                                              CGCCTCCTTGACAGTCTTCGGCAGGAAG GCGGAGCACAGAGGACTCTTAGGATGGTGAAGCTACCCAGTGATAGAC
chr10 chr10 103340026 128799819 23m128799873F53m                                                                                                                                                                                                                                                                                                   CCTTGACAGTCTTCGGCAGGAAG GCGGAGCACAGAGGACTTTTAGGATGGTGAAGATACCCAGTGAGAGACTACAA
chr10 chr10 103340026 128799819 23m128799873F53m                                                                                                                                                                                                                                                                                                   CCTTGACAGTCTTCGGCAGGAAG GCGGAGCACAGAGGACTTTTAGGATGGTGAAGCTACCCAGTGAGAGACTACAA
chr10 chr10 103340023 128799816 20m128799873F56m                                                                                                                                                                                                                                                                                                      TGACAGTCTTCGGCAGGAAG GCGGAGCACAGAGGACTTTTAGGATGGTGAAGCTACCCAGTGAGAGACTACAATGG
chr10 chr10 103340023 128799254 20m128799873F55m562n1m                                                                                                                                                                                                                                                                                                TGACAGTCTTCGGCAGGAAG GCGGAGCACAGAGGACTTTTAGGATGGTGAAGCTACCCAGTGAGAGACTACAATG|||||||||||||||||||||||||||||||||||||||||||||||||||||||||||||||||||||||||||||||||||||||||||||||||||||||||||||||||||||||||||||||||||||||||||||||||||||||||||||||||||||||||||||||||||||||||||||||||||||||||||||||||||||||||||||||||||||||||||||||||||
chr10 chr10 103340023 128799254 20m128799873F55m562n1m                                                                                                                                                                                                                                                                                                TGACAGTCTTCGGCAGGAAG GCGGAGCACAGAGGACTTTTAGGATGGTGAAGCTACCCAGTGAGAGACTACAATG|||||||||||||||||||||||||||||||||||||||||||||||||||||||||||||||||||||||||||||||||||||||||||||||||||||||||||||||||||||||||||||||||||||||||||||||||||||||||||||||||||||||||||||||||||||||||||||||||||||||||||||||||||||||||||||||||||||||||||||||||||
chr10 chr10 103340023 128799816 20m128799873F56m                                                                                                                                                                                                                                                                                                      TGACAGTCTTCGGCAGGAAG GCGGAGCACAGAGGACTTTTAGGATGGTGAAGCTACCCAGTGAGAGACTACAATGG
chr10 chr10 103340022 128799253 19m128799873F55m562n2m                                                                                                                                                                                                                                                                                                 GACAGTCTTCGGCAGGAAG GCGGAGCACAGAGGACTTTTAGGATGGTGAAGCTACCCAGTGAGAGACTACAATG|||||||||||||||||||||||||||||||||||||||||||||||||||||||||||||||||||||||||||||||||||||||||||||||||||||||||||||||||||||||||||||||||||||||||||||||||||||||||||||||||||||||||||||||||||||||||||||||||||||||||||||||||||||||||||||||||||||||||||||||||||
chr10 chr10 103340021 128799814 18m128799873F58m                                                                                                                                                                                                                                                                                                        ACAGTCTTCGGCAGGAAG GCGGAGCACAGAGGACTTTTAGGATGGTGAAGCTACCCAGTGAGAGACTACAATGGTG
chr10 chr10 103340021 128799814 18m128799873F58m                                                                                                                                                                                                                                                                                                        ACAGTCTTCGGCAGGAAG GCGGAGCACAGAGGACTTTTAGGATGGTGAAGCTACCCAGTGAGAGACTACAATGGTG
chr10 chr10 103340021 128799252 18m128799873F55m562n3m                                                                                                                                                                                                                                                                                                  ACAGTCTTCGGCAGGAAG GCGGAGCACAGAGGACTTTTAGGATGGTGAAGCTACCCAGTGAGAGACTACAATG|||||||||||||||||||||||||||||||||||||||||||||||||||||||||||||||||||||||||||||||||||||||||||||||||||||||||||||||||||||||||||||||||||||||||||||||||||||||||||||||||||||||||||||||||||||||||||||||||||||||||||||||||||||||||||||||||||||||||||||||||||
chr10 chr10 103340020 128799251 17m128799873F55m562n4m                                                                                                                                                                                                                                                                                                   CAGTCTTCGGCAGGAAG GCGGAGCACAGAGGACTTTTAGGATGGTGAAGCTACCCAGTGAGAGACTACAATG|||||||||||||||||||||||||||||||||||||||||||||||||||||||||||||||||||||||||||||||||||||||||||||||||||||||||||||||||||||||||||||||||||||||||||||||||||||||||||||||||||||||||||||||||||||||||||||||||||||||||||||||||||||||||||||||||||||||||||||||||||
chr10 chr10 103340020 128799251 17m128799873F55m562n4m                                                                                                                                                                                                                                                                                                   CAGTCTTCGGCAGGAAG GCGGAGCACAGAGGACTTTTAGGATGGTGAAGCTACCCAGTGAGAGACTACAATG|||||||||||||||||||||||||||||||||||||||||||||||||||||||||||||||||||||||||||||||||||||||||||||||||||||||||||||||||||||||||||||||||||||||||||||||||||||||||||||||||||||||||||||||||||||||||||||||||||||||||||||||||||||||||||||||||||||||||||||||||||
chr10 chr10 103340020 128799251 17m128799873F55m562n4m                                                                                                                                                                                                                                                                                                   CAGTCTTCGGCAGGAAG GCGGAGCACAGAGGACTTTTAGGATGGTGAAGCTACCCAGCCAGAGACTACAATG|||||||||||||||||||||||||||||||||||||||||||||||||||||||||||||||||||||||||||||||||||||||||||||||||||||||||||||||||||||||||||||||||||||||||||||||||||||||||||||||||||||||||||||||||||||||||||||||||||||||||||||||||||||||||||||||||||||||||||||||||||
chr10 chr10 103340020 128799251 17m128799873F55m562n4m                                                                                                                                                                                                                                                                                                   CAGTCTTCGGCAGGAAG GCGGAGCACAGAGGACTTTTAGGATGGTGAAGCTACCCAGCCAGAGACTACAATG|||||||||||||||||||||||||||||||||||||||||||||||||||||||||||||||||||||||||||||||||||||||||||||||||||||||||||||||||||||||||||||||||||||||||||||||||||||||||||||||||||||||||||||||||||||||||||||||||||||||||||||||||||||||||||||||||||||||||||||||||||
chr10 chr10 103340020 128799251 17m128799873F55m562n4m                                                                                                                                                                                                                                                                                                   CTGTCTTCGGCAGGAAG GCGGAGCACAGAGGACTTTTAGGATGGTGAAGCTACCCAGTGAGAGACTACAATG|||||||||||||||||||||||||||||||||||||||||||||||||||||||||||||||||||||||||||||||||||||||||||||||||||||||||||||||||||||||||||||||||||||||||||||||||||||||||||||||||||||||||||||||||||||||||||||||||||||||||||||||||||||||||||||||||||||||||||||||||||
chr10 chr10 103340018 128799811 15m128799873F61m                                                                                                                                                                                                                                                                                                           GTCTTCGGCAGGAAG GCGGAGCACAGAGGACTTTTAGGATGGTGAAGCTACCCAGTGAGAGACTACAATGGTGAAT
chr10 chr10 103340018 128799249 15m128799873F55m562n6m                                                                                                                                                                                                                                                                                                     GTCTTCGGCAGGAAG GCGGAGCACAGAGGACTTTTAGGATGGTGAAGCTACCCAGTGAGAGACTACAATG|||||||||||||||||||||||||||||||||||||||||||||||||||||||||||||||||||||||||||||||||||||||||||||||||||||||||||||||||||||||||||||||||||||||||||||||||||||||||||||||||||||||||||||||||||||||||||||||||||||||||||||||||||||||||||||||||||||||||||||||||||
chr10 chr10 103340018 128799811 15m128799873F61m                                                                                                                                                                                                                                                                                                           GTCTTCGGCAGGAAG GCGGAGCACAGAGGACTTTTAGGATGGTGAAGCTACCCAGTGAGAGACTACAATGGTGAAT
chr10 chr10 103340014 128799245 11m128799873F55m562n10m                                                                                                                                                                                                                                                                                                        TCGGCAGGAAG GCGGAGCACAGAGGACTTTTAGGATGGTGAAGCTACCCAGTGAGAGACTACAATG|||||||||||||||||||||||||||||||||||||||||||||||||||||||||||||||||||||||||||||||||||||||||||||||||||||||||||||||||||||||||||||||||||||||||||||||||||||||||||||||||||||||||||||||||||||||||||||||||||||||||||||||||||||||||||||||||||||||||||||||||||
chr10 chr10 103340013 128799806 10m128799873F66m                                                                                                                                                                                                                                                                                                                CGGCAGGAAG GCGGAGCACAGAGGACTTTTAGGATGGTGAAGCTACCCAGTGAGAGACTACAATGGTGAATATACG
chr10 chr10 103340012 128799805 9m128799873F67m                                                                                                                                                                                                                                                                                                                  GGCAGGAAG GCGGAGCACAGAGGACTTTTAGGATGGTGAAGCTACCCAGTGAGAGACTACAATGGTGAATATACGG
chr10 chr10 103340007 128799238 4m128799873F55m562n17m                                                                                                                                                                                                                                                                                                                GAAG GCGGAGCACAGAGGACTTTTAGGATGGTGAAGCTACCCAGTGAGAGACTACAATG|||||||||||||||||||||||||||||||||||||||||||||||||||||||||||||||||||||||||||||||||||||||||||||||||||||||||||||||||||||||||||||||||||||||||||||||||||||||||||||||||||||||||||||||||||||||||||||||||||||||||||||||||||||||||||||||||||||||||||||||||||
chr10 chr10 128799877 128799801 76m                                                                                                                                                                                                                                                                                                                                   CCCAGGCGGAGCACAGAGGACTTTTAGGATGGTGAAGCTACCCAGTGAGAGACTACAATGGTGAATATACGGTATT
chr10 chr10 128799874 128799798 76m                                                                                                                                                                                                                                                                                                                                      TGGCGGAGCACAGAGGACTTTTAGGATGGTGAAGCTACCCAGTGAGAGACTACAATGGTGAATATACGGTATTATT
chr10 chr10 128799871 128799233 22m562n54m                                                                                                                                                                                                                                                                                                                                  CGGAGCACAGAGGACTTTTAGG|||||||||||||||||||||||||||||||||||||||||||||||||||||||||||||||||||||||||||||||||||||||||||||||||||||||||||||||||||||||||||||||||||||||||||||||||||||||||||||||||||||||||||||||||||||||||||||||||||||||||||||||||||||||||||||||||||||||||||||||||||||||||||||||||||||||||||||||||||
chr10 chr10 128799867 128799229 26m562n50m                                                                                                                                                                                                                                                                                                                                      GCACAGAGGACTTTTAGGATGGTGAA|||||||||||||||||||||||||||||||||||||||||||||||||||||||||||||||||||||||||||||||||||||||||||||||||||||||||||||||||||||||||||||||||||||||||||||||||||||||||||||||||||||||||||||||||||||||||||||||||||||||||||||||||||||||||||||||||||||||||||||||||||||||||||||||||||||||||||
chr10 chr10 128799863 128799787 76m                                                                                                                                                                                                                                                                                                                                                 AGAGGACTTTTAGGATGGTGAAGCTACCCAGTGAGAGACTACAATGGTGAATATACGGTATTATTCATTTGTCGAA
chr10 chr10 128799860 128799222 33m562n43m                                                                                                                                                                                                                                                                                                                                             GGACTTTTAGGATGGTGAAGCTACCCAGTGAGA|||||||||||||||||||||||||||||||||||||||||||||||||||||||||||||||||||||||||||||||||||||||||||||||||||||||||||||||||||||||||||||||||||||||||||||||||||||||||||||||||||||||||||||||||||||||||||||||||||||||||||||||||||||||||||||||||||||||||||||||||||||||||||||
chr10 chr10 128799857 128799219 36m562n40m                                                                                                                                                                                                                                                                                                                                                CTTTTAGGATGGTGAAGCTACCCAGTGAGAGACTAC|||||||||||||||||||||||||||||||||||||||||||||||||||||||||||||||||||||||||||||||||||||||||||||||||||||||||||||||||||||||||||||||||||||||||||||||||||||||||||||||||||||||||||||||||||||||||||||||||||||||||||||||||||||||||||||||||||||||||||||||||||||||
chr10 chr10 128799854 128799216 39m562n37m                                                                                                                                                                                                                                                                                                                                                   TTAGGATGGTGAAGCTACCCAGTGAGAGACTACAATGAG|||||||||||||||||||||||||||||||||||||||||||||||||||||||||||||||||||||||||||||||||||||||||||||||||||||||||||||||||||||||||||||||||||||||||||||||||||||||||||||||||||||||||||||||||||||||||||||||||||||||||||||||||||||||||||||||||||||||||||||||||
chr10 chr10 128799851 128799775 76m                                                                                                                                                                                                                                                                                                                                                             GGATGGTGAAGCTACCCAGTGAGAGACTACAATGGTGAATATACGGTATTATTCATTTGTCGAAACCCACAGAAGG
chr10 chr10 128799848 128799210 45m562n31m                                                                                                                                                                                                                                                                                                                                                         TGGTGAAGCTACCCAGTGAGAGACTACAATGAGATAATTGGTCTT|||||||||||||||||||||||||||||||||||||||||||||||||||||||||||||||||||||||||||||||||||||||||||||||||||||||||||||||||||||||||||||||||||||||||||||||||||||||||||||||||||||||||||||||||||||||||||||||||||||||||||||||||||||||||||||||||||||
chr10 chr10 128799845 128799769 76m                                                                                                                                                                                                                                                                                                                                                                   TGAAGCTACCCAGTGAGAGACTACAATGGTGAATATACGGTATTATTCATTTGTCGAAACCCACAGAAGGCACAAC
chr10 chr10 128799841 128799203 52m562n24m                                                                                                                                                                                                                                                                                                                                                                GCTACCCAGTGAGAGACTACAATGAGATAATTGGTCTTTCACATTCCTCGTG|||||||||||||||||||||||||||||||||||||||||||||||||||||||||||||||||||||||||||||||||||||||||||||||||||||||||||||||||||||||||||||||||||||||||||||||||||||||||||||||||||||||||||||||||||||||||||||||||||||||||||||||||||||||
chr10 chr10 128799838 128799762 76m                                                                                                                                                                                                                                                                                                                                                                          ACCCAGTGAGAGACTACAATGGTGAATATACGGTATTATTCATTTGTCGAAACCCACAGAAGGCACAACATCAAGA
chr10 chr10 128799835 128799197 58m562n18m                                                                                                                                                                                                                                                                                                                                                                      CAGTGAGAGACTACAATGAGATAATTGGTCTTTCACATTCCTCGTGCTGTCACCAAAA|||||||||||||||||||||||||||||||||||||||||||||||||||||||||||||||||||||||||||||||||||||||||||||||||||||||||||||||||||||||||||||||||||||||||||||||||||||||||||||||||||||||||||||||||||||||||||||||||||||||||||
chr10 chr10 128799832 128799756 76m                                                                                                                                                                                                                                                                                                                                                                                TGAGAGACTACAATGGTGAATATACGGTATTATTCATTTGTCGAAACCCACAGAAGGCACAACATCAAGAATGAAC
chr10 chr10 128799829 128799753 76m                                                                                                                                                                                                                                                                                                                                                                                   GAGACTACAATGGTGAATATACGGTATTATTCATTTGTCGAAACCCACAGAAGGCACAACATCAAGAATGAACGCG
chr10 chr10 128799826 128799188 67m562n9m                                                                                                                                                                                                                                                                                                                                                                                ACTGCAATGAGATAATTGGTCTTTCACATTCCTCGTGCTGTCACCAAAAATAACAGAGCAACAGCAG|||||||||||||||||||||||||||||||||||||||||||||||||||||||||||||||||||||||||||||||||||||||||||||||||||||||||||||||||||||||||||||||||||||||||||||||||||||||||||||||||||||||||||||||||||||||||
chr10 chr10 128799823 128799185 70m562n6m                                                                                                                                                                                                                                                                                                                                                                                   ACAATGAGATAATTGGTCTTTCACATTCCTCGTGCTGTCACCAAAAATAACAGAGCAACAGCAGGGTGGA|||||||||||||||||||||||||||||||||||||||||||||||||||||||||||||||||||||||||||||||||||||||||||||||||||||||||||||||||||||||||||||||||||||||||||||||||||||||||||||||||||||||||||||||||||
chr10 chr10 128799820 128799744 76m                                                                                                                                                                                                                                                                                                                                                                                            ATGGTGAATATACGGTATTATTCATTTGTCGAAACCCACAGAAGGCACAACATCAAGAATGAACGCTAATGTAAAC
chr10 chr10 128799817 128799741 76m                                                                                                                                                                                                                                                                                                                                                                                               GTGAATATACGGTATTATTCATTTGTCGAAACCCACAGAAGGCACAACATCAAGAATGAACGCTAATGTAAACTAC
chr10 chr10 128799813 128799737 76m                                                                                                                                                                                                                                                                                                                                                                                                   ATATACGGTATTATTCATTTGTCGAAACCCACAGAAGGCACAACATCAAGAATGAACGCTAATGTAAACTACAGAC
chr10 chr10 128799810 128799734 76m                                                                                                                                                                                                                                                                                                                                                                                                      CACGGTATTATTCATTTGTCGAAACCCACAGAAGGCACAACATCAAGAATGAACGCTAATGTAAACTACAGACTTC
chr10 chr10 128799807 128799731 76m                                                                                                                                                                                                                                                                                                                                                                                                         GGTATTATTCATTTGTCGAAACCCACAGAAGGCACAACATCAAGAATGAACGCTAATGTAAACTACAGACTTCACA
chr10 chr10 128799803 128799727 76m                                                                                                                                                                                                                                                                                                                                                                                                             TTATTCATTTGTCGAAACCCACAGAAGGCACAACATCAAGAATGAACGCTAATGTAAACTACAGACTTCACAGGGC
chr10 chr10 128799800 128799724 76m                                                                                                                                                                                                                                                                                                                                                                                                                TTCATTTGTCGAAACCCACAGAAGGCACAACATCAAGAATGAACGCTAATGTAAACTACAGACTTCACAGGGCGAC
chr10 chr10 128799797 128799721 76m                                                                                                                                                                                                                                                                                                                                                                                                                   ATTTGTCGAAACCCACAGAAGGCACAACATCAAGAATGAACGCTAATGTAAACTACAGACTTCACAGGGCGACGAT
chr10 chr10 128799793 128799717 76m                                                                                                                                                                                                                                                                                                                                                                                                                       GTCGAAACCCACAGAAGGCACAACATCAAGAATGAACGCTAATGTAAACTACAGACTTCACAGGGCGACGATGTGT
chr10 chr10 128799789 128799713 76m                                                                                                                                                                                                                                                                                                                                                                                                                           AAACCAACAGAAGGCACAACATCAAGAATGAACGCTAATGTAAACTACAGACTTCACAGGGCGACGATGTGTCAAT
chr10 chr10 128799786 128799710 76m                                                                                                                                                                                                                                                                                                                                                                                                                              CCCACAGAAGGCACAACATCAAGAATGAACGCTAATGTAAACTACAGACTTCACAGGGCGACGATGTGTCAATGCG
chr10 chr10 128799783 128799707 76m                                                                                                                                                                                                                                                                                                                                                                                                                                 ACAGAAGGCACAACATCAAGAATGAACGCTAATGTAAACTACAGACTTCACAGGGCGACGATGTGTCAATGCGGTG
chr10 chr10 128799780 128799704 76m                                                                                                                                                                                                                                                                                                                                                                                                                                    GAAGGCACAACATCAAGAATGAACGCTAATGTAAACTACAGACTTCACAGGGCGACGATGTGTCAATGCGGTTCAC
chr10 chr10 128799777 128799701 76m                                                                                                                                                                                                                                                                                                                                                                                                                                       GGCACAACATCAAGAATGAACGCTAATGTAAACTACAGACTTCACAGGGCGACGATGTGTCAATGCGGTTCACCAA
chr10 chr10 128799774 128799698 76m                                                                                                                                                                                                                                                                                                                                                                                                                                          ACAACATCAAGAATGAACGCTAATGTAAACTACAGACTTCACAGGGCGACGATGTGTCAATGCGGTTCACCAACTG
chr10 chr10 128799771 128799695 76m                                                                                                                                                                                                                                                                                                                                                                                                                                             ACATCAAGAATGAACGCTAATGTAAACTACAGACTTCACAGGGCGACGATGTGTCAATGCGGTTCACCAACTGTAA
chr10 chr10 128799768 128799692 76m                                                                                                                                                                                                                                                                                                                                                                                                                                                TCAAGAATGAACGCTAATGTAAACTACAGACTTCACAGGGCGACGATGTGTCAATGCGGTTCACCAACTGTAATCA
chr10 chr10 128799764 128799688 76m                                                                                                                                                                                                                                                                                                                                                                                                                                                    GAATGAACGCTAATGTAAACTACAGACTTCACAGGGCGACGATGTGTCAATGCGGTTCACCAACTGTAATCAATGT
chr10 chr10 128799761 128799685 76m                                                                                                                                                                                                                                                                                                                                                                                                                                                       TGAACGCTAATGTAAACTACAGACTTCACAGGGCGACGATGTGTCAATGCGGTTCACCAACTGTAATCAATGTACC
chr10 chr10 128799758 128799682 76m                                                                                                                                                                                                                                                                                                                                                                                                                                                          ACGCTAATGTAAACTACAGACTTCACAGGGCGACGATGTGTCAATGCGGTTCACCAACTGTAATCAATGTACCAAT
chr10 chr10 128799755 128799679 76m                                                                                                                                                                                                                                                                                                                                                                                                                                                             CTAATGTAAACTACAGACTTCACAGGGCGACGATGTGTCAATGCGGTTCACCAACTGTAATCAATGTACCAATCTA
chr10 chr10 128799752 128799676 76m                                                                                                                                                                                                                                                                                                                                                                                                                                                                ATGTAAACTACAGACTTCACAGGGCGACGATGTGTCAATGCGGTTCACCAACTGTAATCAATGTACCAATCTATGG
chr10 chr10 128799748 128799672 76m                                                                                                                                                                                                                                                                                                                                                                                                                                                                    AAACTACAGACTTCACAGGGCGACGATGTGTCAATGCGGTTCACCAACTGTAATCAATGTACCAATCTATGGTGGA
chr10 chr10 128799744 128799668 76m                                                                                                                                                                                                                                                                                                                                                                                                                                                                        TACAGACTTCACAGGGCGACGATGTGTCAATGCGGTTCACCAACTGTAATCAATGTACCAATCTATGGTGGATACT
chr10 chr10 128799741 128799665 76m                                                                                                                                                                                                                                                                                                                                                                                                                                                                           AGACTTCACAGGGCGACGATGTGTCAATGCGGTTCACCAACTGTAATCAATGTACCAATCTATGGTGGATACTGAT
chr10 chr10 128799738 128799662 76m                                                                                                                                                                                                                                                                                                                                                                                                                                                                              CTTCACAGGGCGACGATGTGTCAATGCGGTTCACCAACTGTAATCAATGTACCAATCTATGGTGGATACTGATAAT
chr10 chr10 128799734 128799658 76m                                                                                                                                                                                                                                                                                                                                                                                                                                                                                  ACAGGGCGACGATGTGTCAATGCGGTTCACCAACTGTAATCAATGTACCAATCTATGGTGGATACTGATAATGGGG
chr10 chr10 128799731 128799655 76m                                                                                                                                                                                                                                                                                                                                                                                                                                                                                     CGGCGACGATGTGTCAATGCGGTTCACCAACTGTAATCAATGTACCAATCTATGGTGGATACTGATAATGGGGAGG
chr10 chr10 128799728 128799652 76m                                                                                                                                                                                                                                                                                                                                                                                                                                                                                        CGACGATGTGTCAATGCGGTTCACCAACTGTAATCAATGTACCAATCTATGGTGGATACTGATAATGGGGAGGCAG
chr10 chr10 128799725 128799649 76m                                                                                                                                                                                                                                                                                                                                                                                                                                                                                           CGATGTGTCAATGCGGTTCACCAACTGTAATCAATGTACCAATCTATGGTGGATACTGATAATGGGGAGGCAGAGG
chr10 chr10 128799721 128799645 76m                                                                                                                                                                                                                                                                                                                                                                                                                                                                                               GTGTCAATGCGGTTCACCAACTGTAATCAATGTACCAATCTATGGTGGATACTGATAATGGGGAGGCAGGGGGAAG 
    33 pairs 
 0:6
22:13
21:18
22:25
16:32
28:21
6:44
27:25
27:33
46:19
41:28
22:93
110:18
33:110
22:645
22:645
0:669
-702:18
22:757
-41:1322
-83:1306
-84:1404
-89:1413
-93:1422
-2984:632
-3878:-714
-3573:1311
7027:1320
7027:1320
7106:1326
7145:1334
7199:1405
8149:1433 
     TCGA-02-2483-01A chr10-chr10 126186696 127668729 ff 
 
4 5 5 7 55 50
CTGAGTTTTTCAAGTCTGCCCTGCAAGCGATAGGAGTGGAAGCCCACCAG AAATCATGCCACCCTCAAAGCCTCATCCACCTGTCGTGGGCAAAGTGACT
11112222222222222222222344444444444444444444444444 44444444444444444444433333333322222222222222222221
LHPP exon5(126186603-126186696) FANK1 exon2(127668728-127668906)
 
 blast search - human genome  
 left 
 >ref|NT_030059.13| Homo sapiens chromosome 10 genomic contig, GRCh37.p9 Primary 
Assembly
 gb|GL000099.1| Homo sapiens chromosome 10 genomic contig, GRCh37 reference primary 
assembly
Length=79420533

 Score = 93.5 bits (50),  Expect = 5e-17
 Identities = 50/50 (100%), Gaps = 0/50 (0%)
 Strand=Plus/Plus

Query  1         CTGAGTTTTTCAAGTCTGCCCTGCAAGCGATAGGAGTGGAAGCCCACCAG  50
                 ||||||||||||||||||||||||||||||||||||||||||||||||||
Sbjct  76991112  CTGAGTTTTTCAAGTCTGCCCTGCAAGCGATAGGAGTGGAAGCCCACCAG  76991161


>ref|NW_001838010.1| Homo sapiens chromosome 10 genomic contig, alternate assembly 
HuRef SCAF_1103279188229, whole genome shotgun sequence
 gb|DS486216.1| Homo sapiens SCAF_1103279188229 genomic scaffold, whole genome 
shotgun sequence
Length=2644411

 Score = 93.5 bits (50),  Expect = 5e-17
 Identities = 50/50 (100%), Gaps = 0/50 (0%)
 Strand=Plus/Plus

Query  1       CTGAGTTTTTCAAGTCTGCCCTGCAAGCGATAGGAGTGGAAGCCCACCAG  50
               ||||||||||||||||||||||||||||||||||||||||||||||||||
Sbjct  193704  CTGAGTTTTTCAAGTCTGCCCTGCAAGCGATAGGAGTGGAAGCCCACCAG  193753


>ref|NC_000010.10| Homo sapiens chromosome 10, GRCh37.p9 Primary Assembly
 gb|CM000672.1| Homo sapiens chromosome 10, GRCh37 primary reference assembly
Length=135534747

 Score = 93.5 bits (50),  Expect = 5e-17
 Identities = 50/50 (100%), Gaps = 0/50 (0%)
 Strand=Plus/Plus

Query  1          CTGAGTTTTTCAAGTCTGCCCTGCAAGCGATAGGAGTGGAAGCCCACCAG  50
                  ||||||||||||||||||||||||||||||||||||||||||||||||||
Sbjct  126186648  CTGAGTTTTTCAAGTCTGCCCTGCAAGCGATAGGAGTGGAAGCCCACCAG  126186697


>ref|AC_000142.1| Homo sapiens chromosome 10, alternate assembly HuRef, whole genome 
shotgun sequence
 gb|CM000471.1| Homo sapiens chromosome 10, whole genome shotgun sequence
Length=128985118

 Score = 93.5 bits (50),  Expect = 5e-17
 Identities = 50/50 (100%), Gaps = 0/50 (0%)
 Strand=Plus/Plus

Query  1          CTGAGTTTTTCAAGTCTGCCCTGCAAGCGATAGGAGTGGAAGCCCACCAG  50
                  ||||||||||||||||||||||||||||||||||||||||||||||||||
Sbjct  119872802  CTGAGTTTTTCAAGTCTGCCCTGCAAGCGATAGGAGTGGAAGCCCACCAG  119872851


>gb|CM000261.1| Homo sapiens chromosome 10, whole genome shotgun sequence
Length=129189614

 Score = 93.5 bits (50),  Expect = 5e-17
 Identities = 50/50 (100%), Gaps = 0/50 (0%)
 Strand=Plus/Plus

Query  1          CTGAGTTTTTCAAGTCTGCCCTGCAAGCGATAGGAGTGGAAGCCCACCAG  50
                  ||||||||||||||||||||||||||||||||||||||||||||||||||
Sbjct  119960521  CTGAGTTTTTCAAGTCTGCCCTGCAAGCGATAGGAGTGGAAGCCCACCAG  119960570


Lambda     K      H
    1.33    0.621     1.12 

Gapped
Lambda     K      H
    1.28    0.460    0.850 

Effective search space used: 681780354108
 
 right 
 >ref|NT_030059.13| Homo sapiens chromosome 10 genomic contig, GRCh37.p9 Primary 
Assembly
 gb|GL000099.1| Homo sapiens chromosome 10 genomic contig, GRCh37 reference primary 
assembly
Length=79420533

 Score = 93.5 bits (50),  Expect = 5e-17
 Identities = 50/50 (100%), Gaps = 0/50 (0%)
 Strand=Plus/Plus

Query  1         AAATCATGCCACCCTCAAAGCCTCATCCACCTGTCGTGGGCAAAGTGACT  50
                 ||||||||||||||||||||||||||||||||||||||||||||||||||
Sbjct  78473194  AAATCATGCCACCCTCAAAGCCTCATCCACCTGTCGTGGGCAAAGTGACT  78473243


>ref|NW_001838010.1| Homo sapiens chromosome 10 genomic contig, alternate assembly 
HuRef SCAF_1103279188229, whole genome shotgun sequence
 gb|DS486216.1| Homo sapiens SCAF_1103279188229 genomic scaffold, whole genome 
shotgun sequence
Length=2644411

 Score = 93.5 bits (50),  Expect = 5e-17
 Identities = 50/50 (100%), Gaps = 0/50 (0%)
 Strand=Plus/Plus

Query  1        AAATCATGCCACCCTCAAAGCCTCATCCACCTGTCGTGGGCAAAGTGACT  50
                ||||||||||||||||||||||||||||||||||||||||||||||||||
Sbjct  1678799  AAATCATGCCACCCTCAAAGCCTCATCCACCTGTCGTGGGCAAAGTGACT  1678848


>ref|NC_000010.10| Homo sapiens chromosome 10, GRCh37.p9 Primary Assembly
 gb|CM000672.1| Homo sapiens chromosome 10, GRCh37 primary reference assembly
Length=135534747

 Score = 93.5 bits (50),  Expect = 5e-17
 Identities = 50/50 (100%), Gaps = 0/50 (0%)
 Strand=Plus/Plus

Query  1          AAATCATGCCACCCTCAAAGCCTCATCCACCTGTCGTGGGCAAAGTGACT  50
                  ||||||||||||||||||||||||||||||||||||||||||||||||||
Sbjct  127668730  AAATCATGCCACCCTCAAAGCCTCATCCACCTGTCGTGGGCAAAGTGACT  127668779


>ref|AC_000142.1| Homo sapiens chromosome 10, alternate assembly HuRef, whole genome 
shotgun sequence
 gb|CM000471.1| Homo sapiens chromosome 10, whole genome shotgun sequence
Length=128985118

 Score = 93.5 bits (50),  Expect = 5e-17
 Identities = 50/50 (100%), Gaps = 0/50 (0%)
 Strand=Plus/Plus

Query  1          AAATCATGCCACCCTCAAAGCCTCATCCACCTGTCGTGGGCAAAGTGACT  50
                  ||||||||||||||||||||||||||||||||||||||||||||||||||
Sbjct  121357897  AAATCATGCCACCCTCAAAGCCTCATCCACCTGTCGTGGGCAAAGTGACT  121357946


>gb|CM000261.1| Homo sapiens chromosome 10, whole genome shotgun sequence
Length=129189614

 Score = 93.5 bits (50),  Expect = 5e-17
 Identities = 50/50 (100%), Gaps = 0/50 (0%)
 Strand=Plus/Plus

Query  1          AAATCATGCCACCCTCAAAGCCTCATCCACCTGTCGTGGGCAAAGTGACT  50
                  ||||||||||||||||||||||||||||||||||||||||||||||||||
Sbjct  121444157  AAATCATGCCACCCTCAAAGCCTCATCCACCTGTCGTGGGCAAAGTGACT  121444206


>gb|CM000500.1| Homo sapiens chromosome 10, whole genome shotgun sequence
Length=128985077

 Score = 87.9 bits (47),  Expect = 2e-15
 Identities = 49/50 (98%), Gaps = 0/50 (0%)
 Strand=Plus/Plus

Query  1          AAATCATGCCACCCTCAAAGCCTCATCCACCTGTCGTGGGCAAAGTGACT  50
                  ||||||||||||||||||||| ||||||||||||||||||||||||||||
Sbjct  121357942  AAATCATGCCACCCTCAAAGCTTCATCCACCTGTCGTGGGCAAAGTGACT  121357991


>gb|DS990854.1| Homo sapiens SCAF_1112675837174 genomic scaffold, whole genome 
shotgun sequence
Length=2644434

 Score = 87.9 bits (47),  Expect = 2e-15
 Identities = 49/50 (98%), Gaps = 0/50 (0%)
 Strand=Plus/Plus

Query  1        AAATCATGCCACCCTCAAAGCCTCATCCACCTGTCGTGGGCAAAGTGACT  50
                ||||||||||||||||||||| ||||||||||||||||||||||||||||
Sbjct  1678839  AAATCATGCCACCCTCAAAGCTTCATCCACCTGTCGTGGGCAAAGTGACT  1678888


Lambda     K      H
    1.33    0.621     1.12 

Gapped
Lambda     K      H
    1.28    0.460    0.850 

Effective search space used: 681780354108
 
 blast search - nt  
 left 
 >ref|XM_003816293.1| PREDICTED: Pan paniscus phospholysine phosphohistidine inorganic 
pyrophosphate phosphatase (LHPP), mRNA
Length=1079

 Score = 93.5 bits (50),  Expect = 6e-17
 Identities = 50/50 (100%), Gaps = 0/50 (0%)
 Strand=Plus/Plus

Query  1    CTGAGTTTTTCAAGTCTGCCCTGCAAGCGATAGGAGTGGAAGCCCACCAG  50
            ||||||||||||||||||||||||||||||||||||||||||||||||||
Sbjct  726  CTGAGTTTTTCAAGTCTGCCCTGCAAGCGATAGGAGTGGAAGCCCACCAG  775


>ref|XM_508095.3| PREDICTED: Pan troglodytes phospholysine phosphohistidine inorganic 
pyrophosphate phosphatase-like (LHPP), mRNA
Length=862

 Score = 93.5 bits (50),  Expect = 6e-17
 Identities = 50/50 (100%), Gaps = 0/50 (0%)
 Strand=Plus/Plus

Query  1    CTGAGTTTTTCAAGTCTGCCCTGCAAGCGATAGGAGTGGAAGCCCACCAG  50
            ||||||||||||||||||||||||||||||||||||||||||||||||||
Sbjct  626  CTGAGTTTTTCAAGTCTGCCCTGCAAGCGATAGGAGTGGAAGCCCACCAG  675


>ref|XM_003277768.1| PREDICTED: Nomascus leucogenys phospholysine phosphohistidine 
inorganic pyrophosphate phosphatase-like, transcript variant 
2 (LOC100594200), mRNA
Length=864

 Score = 93.5 bits (50),  Expect = 6e-17
 Identities = 50/50 (100%), Gaps = 0/50 (0%)
 Strand=Plus/Plus

Query  1    CTGAGTTTTTCAAGTCTGCCCTGCAAGCGATAGGAGTGGAAGCCCACCAG  50
            ||||||||||||||||||||||||||||||||||||||||||||||||||
Sbjct  628  CTGAGTTTTTCAAGTCTGCCCTGCAAGCGATAGGAGTGGAAGCCCACCAG  677


>ref|XM_003277767.1| PREDICTED: Nomascus leucogenys phospholysine phosphohistidine 
inorganic pyrophosphate phosphatase-like, transcript variant 
1 (LOC100594200), mRNA
Length=1602

 Score = 93.5 bits (50),  Expect = 6e-17
 Identities = 50/50 (100%), Gaps = 0/50 (0%)
 Strand=Plus/Plus

Query  1    CTGAGTTTTTCAAGTCTGCCCTGCAAGCGATAGGAGTGGAAGCCCACCAG  50
            ||||||||||||||||||||||||||||||||||||||||||||||||||
Sbjct  660  CTGAGTTTTTCAAGTCTGCCCTGCAAGCGATAGGAGTGGAAGCCCACCAG  709


>gb|HQ258160.1| Synthetic construct Homo sapiens clone IMAGE:100072469 phospholysine 
phosphohistidine inorganic pyrophosphate phosphatase 
(LHPP) gene, encodes complete protein
Length=847

 Score = 93.5 bits (50),  Expect = 6e-17
 Identities = 50/50 (100%), Gaps = 0/50 (0%)
 Strand=Plus/Plus

Query  1    CTGAGTTTTTCAAGTCTGCCCTGCAAGCGATAGGAGTGGAAGCCCACCAG  50
            ||||||||||||||||||||||||||||||||||||||||||||||||||
Sbjct  594  CTGAGTTTTTCAAGTCTGCCCTGCAAGCGATAGGAGTGGAAGCCCACCAG  643


>ref|XM_002821242.1| PREDICTED: Pongo abelii phospholysine phosphohistidine inorganic 
pyrophosphate phosphatase (LHPP), mRNA
Length=1629

 Score = 93.5 bits (50),  Expect = 6e-17
 Identities = 50/50 (100%), Gaps = 0/50 (0%)
 Strand=Plus/Plus

Query  1    CTGAGTTTTTCAAGTCTGCCCTGCAAGCGATAGGAGTGGAAGCCCACCAG  50
            ||||||||||||||||||||||||||||||||||||||||||||||||||
Sbjct  666  CTGAGTTTTTCAAGTCTGCCCTGCAAGCGATAGGAGTGGAAGCCCACCAG  715


>ref|NM_001167880.1| Homo sapiens phospholysine phosphohistidine inorganic pyrophosphate 
phosphatase (LHPP), transcript variant 2, mRNA
Length=1611

 Score = 93.5 bits (50),  Expect = 6e-17
 Identities = 50/50 (100%), Gaps = 0/50 (0%)
 Strand=Plus/Plus

Query  1    CTGAGTTTTTCAAGTCTGCCCTGCAAGCGATAGGAGTGGAAGCCCACCAG  50
            ||||||||||||||||||||||||||||||||||||||||||||||||||
Sbjct  666  CTGAGTTTTTCAAGTCTGCCCTGCAAGCGATAGGAGTGGAAGCCCACCAG  715


>ref|NM_022126.3| Homo sapiens phospholysine phosphohistidine inorganic pyrophosphate 
phosphatase (LHPP), transcript variant 1, mRNA
Length=1703

 Score = 93.5 bits (50),  Expect = 6e-17
 Identities = 50/50 (100%), Gaps = 0/50 (0%)
 Strand=Plus/Plus

Query  1    CTGAGTTTTTCAAGTCTGCCCTGCAAGCGATAGGAGTGGAAGCCCACCAG  50
            ||||||||||||||||||||||||||||||||||||||||||||||||||
Sbjct  666  CTGAGTTTTTCAAGTCTGCCCTGCAAGCGATAGGAGTGGAAGCCCACCAG  715


>emb|CU683965.1| Synthetic construct Homo sapiens gateway clone IMAGE:100020193 
3' read mRNA
Length=1113

 Score = 93.5 bits (50),  Expect = 6e-17
 Identities = 50/50 (100%), Gaps = 0/50 (0%)
 Strand=Plus/Minus

Query  1   CTGAGTTTTTCAAGTCTGCCCTGCAAGCGATAGGAGTGGAAGCCCACCAG  50
           ||||||||||||||||||||||||||||||||||||||||||||||||||
Sbjct  74  CTGAGTTTTTCAAGTCTGCCCTGCAAGCGATAGGAGTGGAAGCCCACCAG  25


>gb|BC113629.1| Homo sapiens phospholysine phosphohistidine inorganic pyrophosphate 
phosphatase, mRNA (cDNA clone MGC:142189 IMAGE:8322681), 
complete cds
Length=1591

 Score = 93.5 bits (50),  Expect = 6e-17
 Identities = 50/50 (100%), Gaps = 0/50 (0%)
 Strand=Plus/Plus

Query  1    CTGAGTTTTTCAAGTCTGCCCTGCAAGCGATAGGAGTGGAAGCCCACCAG  50
            ||||||||||||||||||||||||||||||||||||||||||||||||||
Sbjct  666  CTGAGTTTTTCAAGTCTGCCCTGCAAGCGATAGGAGTGGAAGCCCACCAG  715


>gb|BC113631.1| Homo sapiens phospholysine phosphohistidine inorganic pyrophosphate 
phosphatase, mRNA (cDNA clone MGC:142191 IMAGE:8322683), 
complete cds
Length=1574

 Score = 93.5 bits (50),  Expect = 6e-17
 Identities = 50/50 (100%), Gaps = 0/50 (0%)
 Strand=Plus/Plus

Query  1    CTGAGTTTTTCAAGTCTGCCCTGCAAGCGATAGGAGTGGAAGCCCACCAG  50
            ||||||||||||||||||||||||||||||||||||||||||||||||||
Sbjct  666  CTGAGTTTTTCAAGTCTGCCCTGCAAGCGATAGGAGTGGAAGCCCACCAG  715


>gb|BC007324.2| Homo sapiens phospholysine phosphohistidine inorganic pyrophosphate 
phosphatase, mRNA (cDNA clone IMAGE:3640409), partial 
cds
Length=1540

 Score = 93.5 bits (50),  Expect = 6e-17
 Identities = 50/50 (100%), Gaps = 0/50 (0%)
 Strand=Plus/Plus

Query  1    CTGAGTTTTTCAAGTCTGCCCTGCAAGCGATAGGAGTGGAAGCCCACCAG  50
            ||||||||||||||||||||||||||||||||||||||||||||||||||
Sbjct  595  CTGAGTTTTTCAAGTCTGCCCTGCAAGCGATAGGAGTGGAAGCCCACCAG  644


>dbj|AK055532.1| Homo sapiens cDNA FLJ30970 fis, clone HEART2000444, highly similar 
to Homo sapiens phospholysine phosphohistidine inorganic 
pyrophosphate phosphatase (LHPP), mRNA
Length=1613

 Score = 93.5 bits (50),  Expect = 6e-17
 Identities = 50/50 (100%), Gaps = 0/50 (0%)
 Strand=Plus/Plus

Query  1    CTGAGTTTTTCAAGTCTGCCCTGCAAGCGATAGGAGTGGAAGCCCACCAG  50
            ||||||||||||||||||||||||||||||||||||||||||||||||||
Sbjct  595  CTGAGTTTTTCAAGTCTGCCCTGCAAGCGATAGGAGTGGAAGCCCACCAG  644


>emb|AL445237.16| Human DNA sequence from clone RP13-238F13 on chromosome 10 Contains 
the OAT gene for ornithine aminotransferase (gyrate atrophy), 
a homeobox protein pseudogene, the 5' end of the LHPP 
gene for phospholysine phosphohistidine inorganic pyrophosphate 
phosphatase, a RPS10 (40S ribosomal protein 10) pseudogene 
and four CpG islands, complete sequence
Length=182955

 Score = 93.5 bits (50),  Expect = 6e-17
 Identities = 50/50 (100%), Gaps = 0/50 (0%)
 Strand=Plus/Plus

Query  1       CTGAGTTTTTCAAGTCTGCCCTGCAAGCGATAGGAGTGGAAGCCCACCAG  50
               ||||||||||||||||||||||||||||||||||||||||||||||||||
Sbjct  169199  CTGAGTTTTTCAAGTCTGCCCTGCAAGCGATAGGAGTGGAAGCCCACCAG  169248


>dbj|AB049629.1| Homo sapiens lhpp mRNA for phospholysine phosphohistidine inorganic 
pyrophosphate phosphatase, complete cds
Length=1814

 Score = 93.5 bits (50),  Expect = 6e-17
 Identities = 50/50 (100%), Gaps = 0/50 (0%)
 Strand=Plus/Plus

Query  1    CTGAGTTTTTCAAGTCTGCCCTGCAAGCGATAGGAGTGGAAGCCCACCAG  50
            ||||||||||||||||||||||||||||||||||||||||||||||||||
Sbjct  740  CTGAGTTTTTCAAGTCTGCCCTGCAAGCGATAGGAGTGGAAGCCCACCAG  789


>gb|BC110344.1| Homo sapiens phospholysine phosphohistidine inorganic pyrophosphate 
phosphatase, mRNA (cDNA clone MGC:117251 IMAGE:5762731), 
complete cds
Length=1645

 Score = 93.5 bits (50),  Expect = 6e-17
 Identities = 50/50 (100%), Gaps = 0/50 (0%)
 Strand=Plus/Plus

Query  1    CTGAGTTTTTCAAGTCTGCCCTGCAAGCGATAGGAGTGGAAGCCCACCAG  50
            ||||||||||||||||||||||||||||||||||||||||||||||||||
Sbjct  620  CTGAGTTTTTCAAGTCTGCCCTGCAAGCGATAGGAGTGGAAGCCCACCAG  669


>ref|XM_001083060.2| PREDICTED: Macaca mulatta phospholysine phosphohistidine inorganic 
pyrophosphate phosphatase (LHPP), mRNA
Length=1500

 Score = 87.9 bits (47),  Expect = 3e-15
 Identities = 49/50 (98%), Gaps = 0/50 (0%)
 Strand=Plus/Plus

Query  1    CTGAGTTTTTCAAGTCTGCCCTGCAAGCGATAGGAGTGGAAGCCCACCAG  50
            |||||||||||||||||||||||||||| |||||||||||||||||||||
Sbjct  638  CTGAGTTTTTCAAGTCTGCCCTGCAAGCCATAGGAGTGGAAGCCCACCAG  687


>gb|AC210119.5| Macaca mulatta BAC CH250-198D1 (Children's Hospital Oakland Research 
Institute Rhesus macaque Adult Male BAC Library) complete 
sequence
Length=172090

 Score = 87.9 bits (47),  Expect = 3e-15
 Identities = 49/50 (98%), Gaps = 0/50 (0%)
 Strand=Plus/Minus

Query  1      CTGAGTTTTTCAAGTCTGCCCTGCAAGCGATAGGAGTGGAAGCCCACCAG  50
              |||||||||||||||||||||||||||| |||||||||||||||||||||
Sbjct  70799  CTGAGTTTTTCAAGTCTGCCCTGCAAGCCATAGGAGTGGAAGCCCACCAG  70750


>ref|XM_002756691.2| PREDICTED: Callithrix jacchus phospholysine phosphohistidine 
inorganic pyrophosphate phosphatase (LHPP), mRNA
Length=836

 Score = 82.4 bits (44),  Expect = 1e-13
 Identities = 48/50 (96%), Gaps = 0/50 (0%)
 Strand=Plus/Plus

Query  1    CTGAGTTTTTCAAGTCTGCCCTGCAAGCGATAGGAGTGGAAGCCCACCAG  50
            |||||||||||||||||||||||||||| ||||| |||||||||||||||
Sbjct  577  CTGAGTTTTTCAAGTCTGCCCTGCAAGCAATAGGTGTGGAAGCCCACCAG  626


Lambda     K      H
    1.33    0.621     1.12 

Gapped
Lambda     K      H
    1.28    0.460    0.850 

Effective search space used: 874911716973
 
 right 
 >ref|XM_003816282.1| PREDICTED: Pan paniscus fibronectin type III and ankyrin repeat 
domains 1 (FANK1), mRNA
Length=1404

 Score = 93.5 bits (50),  Expect = 6e-17
 Identities = 50/50 (100%), Gaps = 0/50 (0%)
 Strand=Plus/Plus

Query  1    AAATCATGCCACCCTCAAAGCCTCATCCACCTGTCGTGGGCAAAGTGACT  50
            ||||||||||||||||||||||||||||||||||||||||||||||||||
Sbjct  121  AAATCATGCCACCCTCAAAGCCTCATCCACCTGTCGTGGGCAAAGTGACT  170


>ref|XM_003312821.1| PREDICTED: Pan troglodytes fibronectin type III and ankyrin repeat 
domains 1, transcript variant 2 (FANK1), mRNA
Length=1348

 Score = 93.5 bits (50),  Expect = 6e-17
 Identities = 50/50 (100%), Gaps = 0/50 (0%)
 Strand=Plus/Plus

Query  1    AAATCATGCCACCCTCAAAGCCTCATCCACCTGTCGTGGGCAAAGTGACT  50
            ||||||||||||||||||||||||||||||||||||||||||||||||||
Sbjct  90   AAATCATGCCACCCTCAAAGCCTCATCCACCTGTCGTGGGCAAAGTGACT  139


>ref|XM_003312820.1| PREDICTED: Pan troglodytes fibronectin type III and ankyrin repeat 
domains 1, transcript variant 1 (FANK1), mRNA
Length=1292

 Score = 93.5 bits (50),  Expect = 6e-17
 Identities = 50/50 (100%), Gaps = 0/50 (0%)
 Strand=Plus/Plus

Query  1    AAATCATGCCACCCTCAAAGCCTCATCCACCTGTCGTGGGCAAAGTGACT  50
            ||||||||||||||||||||||||||||||||||||||||||||||||||
Sbjct  112  AAATCATGCCACCCTCAAAGCCTCATCCACCTGTCGTGGGCAAAGTGACT  161


>dbj|AK304407.1| Homo sapiens cDNA FLJ52987 complete cds, highly similar to Fibronectin 
type 3 and ankyrin repeat domains protein 1
Length=1283

 Score = 93.5 bits (50),  Expect = 6e-17
 Identities = 50/50 (100%), Gaps = 0/50 (0%)
 Strand=Plus/Plus

Query  1    AAATCATGCCACCCTCAAAGCCTCATCCACCTGTCGTGGGCAAAGTGACT  50
            ||||||||||||||||||||||||||||||||||||||||||||||||||
Sbjct  169  AAATCATGCCACCCTCAAAGCCTCATCCACCTGTCGTGGGCAAAGTGACT  218


>ref|NM_145235.3| Homo sapiens fibronectin type III and ankyrin repeat domains 
1 (FANK1), mRNA
Length=1395

 Score = 93.5 bits (50),  Expect = 6e-17
 Identities = 50/50 (100%), Gaps = 0/50 (0%)
 Strand=Plus/Plus

Query  1    AAATCATGCCACCCTCAAAGCCTCATCCACCTGTCGTGGGCAAAGTGACT  50
            ||||||||||||||||||||||||||||||||||||||||||||||||||
Sbjct  118  AAATCATGCCACCCTCAAAGCCTCATCCACCTGTCGTGGGCAAAGTGACT  167


>gb|AY358154.1| Homo sapiens clone DNA176837 CG3104 hlg (UNQ6504) mRNA, complete 
cds
Length=1373

 Score = 93.5 bits (50),  Expect = 6e-17
 Identities = 50/50 (100%), Gaps = 0/50 (0%)
 Strand=Plus/Plus

Query  1    AAATCATGCCACCCTCAAAGCCTCATCCACCTGTCGTGGGCAAAGTGACT  50
            ||||||||||||||||||||||||||||||||||||||||||||||||||
Sbjct  96   AAATCATGCCACCCTCAAAGCCTCATCCACCTGTCGTGGGCAAAGTGACT  145


>gb|AC063963.11| Homo sapiens chromosome 10 clone RP11-295J3, complete sequence
Length=179104

 Score = 93.5 bits (50),  Expect = 6e-17
 Identities = 50/50 (100%), Gaps = 0/50 (0%)
 Strand=Plus/Plus

Query  1      AAATCATGCCACCCTCAAAGCCTCATCCACCTGTCGTGGGCAAAGTGACT  50
              ||||||||||||||||||||||||||||||||||||||||||||||||||
Sbjct  61132  AAATCATGCCACCCTCAAAGCCTCATCCACCTGTCGTGGGCAAAGTGACT  61181


>emb|CR627249.1| Homo sapiens mRNA; cDNA DKFZp781G1012 (from clone DKFZp781G1012)
Length=8002

 Score = 93.5 bits (50),  Expect = 6e-17
 Identities = 50/50 (100%), Gaps = 0/50 (0%)
 Strand=Plus/Plus

Query  1    AAATCATGCCACCCTCAAAGCCTCATCCACCTGTCGTGGGCAAAGTGACT  50
            ||||||||||||||||||||||||||||||||||||||||||||||||||
Sbjct  102  AAATCATGCCACCCTCAAAGCCTCATCCACCTGTCGTGGGCAAAGTGACT  151


>gb|BC024189.2| Homo sapiens fibronectin type III and ankyrin repeat domains 
1, mRNA (cDNA clone MGC:26734 IMAGE:4826296), complete cds
Length=1395

 Score = 91.6 bits (49),  Expect = 2e-16
 Identities = 49/49 (100%), Gaps = 0/49 (0%)
 Strand=Plus/Plus

Query  2    AATCATGCCACCCTCAAAGCCTCATCCACCTGTCGTGGGCAAAGTGACT  50
            |||||||||||||||||||||||||||||||||||||||||||||||||
Sbjct  119  AATCATGCCACCCTCAAAGCCTCATCCACCTGTCGTGGGCAAAGTGACT  167


>gb|AY251163.1| Homo sapiens HSD13 mRNA, complete cds
Length=1220

 Score = 89.8 bits (48),  Expect = 8e-16
 Identities = 48/48 (100%), Gaps = 0/48 (0%)
 Strand=Plus/Plus

Query  3   ATCATGCCACCCTCAAAGCCTCATCCACCTGTCGTGGGCAAAGTGACT  50
           ||||||||||||||||||||||||||||||||||||||||||||||||
Sbjct  20  ATCATGCCACCCTCAAAGCCTCATCCACCTGTCGTGGGCAAAGTGACT  67


>ref|XM_002821261.2| PREDICTED: Pongo abelii fibronectin type III and ankyrin repeat 
domains 1, transcript variant 1 (FANK1), mRNA
Length=1380

 Score = 87.9 bits (47),  Expect = 3e-15
 Identities = 49/50 (98%), Gaps = 0/50 (0%)
 Strand=Plus/Plus

Query  1    AAATCATGCCACCCTCAAAGCCTCATCCACCTGTCGTGGGCAAAGTGACT  50
            |||||| |||||||||||||||||||||||||||||||||||||||||||
Sbjct  122  AAATCACGCCACCCTCAAAGCCTCATCCACCTGTCGTGGGCAAAGTGACT  171


>ref|XM_002821262.2| PREDICTED: Pongo abelii fibronectin type III and ankyrin repeat 
domains 1, transcript variant 2 (FANK1), mRNA
Length=1302

 Score = 87.9 bits (47),  Expect = 3e-15
 Identities = 49/50 (98%), Gaps = 0/50 (0%)
 Strand=Plus/Plus

Query  1    AAATCATGCCACCCTCAAAGCCTCATCCACCTGTCGTGGGCAAAGTGACT  50
            |||||| |||||||||||||||||||||||||||||||||||||||||||
Sbjct  122  AAATCACGCCACCCTCAAAGCCTCATCCACCTGTCGTGGGCAAAGTGACT  171


>ref|XM_001087857.2| PREDICTED: Macaca mulatta fibronectin type III and ankyrin repeat 
domains 1, transcript variant 6 (FANK1), mRNA
Length=1172

 Score = 87.9 bits (47),  Expect = 3e-15
 Identities = 49/50 (98%), Gaps = 0/50 (0%)
 Strand=Plus/Plus

Query  1    AAATCATGCCACCCTCAAAGCCTCATCCACCTGTCGTGGGCAAAGTGACT  50
            |||||| |||||||||||||||||||||||||||||||||||||||||||
Sbjct  118  AAATCACGCCACCCTCAAAGCCTCATCCACCTGTCGTGGGCAAAGTGACT  167


>ref|XM_001087611.2| PREDICTED: Macaca mulatta fibronectin type III and ankyrin repeat 
domains 1, transcript variant 4 (FANK1), mRNA
Length=1297

 Score = 87.9 bits (47),  Expect = 3e-15
 Identities = 49/50 (98%), Gaps = 0/50 (0%)
 Strand=Plus/Plus

Query  1    AAATCATGCCACCCTCAAAGCCTCATCCACCTGTCGTGGGCAAAGTGACT  50
            |||||| |||||||||||||||||||||||||||||||||||||||||||
Sbjct  118  AAATCACGCCACCCTCAAAGCCTCATCCACCTGTCGTGGGCAAAGTGACT  167


>ref|XM_001086789.2| PREDICTED: Macaca mulatta fibronectin type III and ankyrin repeat 
domains 1, transcript variant 1 (FANK1), mRNA
Length=1353

 Score = 87.9 bits (47),  Expect = 3e-15
 Identities = 49/50 (98%), Gaps = 0/50 (0%)
 Strand=Plus/Plus

Query  1    AAATCATGCCACCCTCAAAGCCTCATCCACCTGTCGTGGGCAAAGTGACT  50
            |||||| |||||||||||||||||||||||||||||||||||||||||||
Sbjct  96   AAATCACGCCACCCTCAAAGCCTCATCCACCTGTCGTGGGCAAAGTGACT  145


>ref|XM_002756706.1| PREDICTED: Callithrix jacchus fibronectin type III and ankyrin 
repeat domains 1 (FANK1), mRNA
Length=1759

 Score = 87.9 bits (47),  Expect = 3e-15
 Identities = 49/50 (98%), Gaps = 0/50 (0%)
 Strand=Plus/Plus

Query  1    AAATCATGCCACCCTCAAAGCCTCATCCACCTGTCGTGGGCAAAGTGACT  50
            ||||||||||| ||||||||||||||||||||||||||||||||||||||
Sbjct  317  AAATCATGCCATCCTCAAAGCCTCATCCACCTGTCGTGGGCAAAGTGACT  366


>dbj|AB179439.1| Macaca fascicularis testis cDNA clone: QtsA-20030, similar to 
human fibronectin type 3 and ankyrin repeat domains 1(FANK1), 
mRNA, RefSeq: NM_145235.2
Length=1201

 Score = 87.9 bits (47),  Expect = 3e-15
 Identities = 49/50 (98%), Gaps = 0/50 (0%)
 Strand=Plus/Plus

Query  1    AAATCATGCCACCCTCAAAGCCTCATCCACCTGTCGTGGGCAAAGTGACT  50
            |||||| |||||||||||||||||||||||||||||||||||||||||||
Sbjct  102  AAATCACGCCACCCTCAAAGCCTCATCCACCTGTCGTGGGCAAAGTGACT  151


>ref|XM_003479614.1| PREDICTED: Cavia porcellus fibronectin type 3 and ankyrin repeat 
domains 1 protein-like (LOC100735426), mRNA
Length=1104

 Score = 76.8 bits (41),  Expect = 7e-12
 Identities = 47/50 (94%), Gaps = 0/50 (0%)
 Strand=Plus/Plus

Query  1    AAATCATGCCACCCTCAAAGCCTCATCCACCTGTCGTGGGCAAAGTGACT  50
            |||||| |||  ||||||||||||||||||||||||||||||||||||||
Sbjct  80   AAATCACGCCGTCCTCAAAGCCTCATCCACCTGTCGTGGGCAAAGTGACT  129


Lambda     K      H
    1.33    0.621     1.12 

Gapped
Lambda     K      H
    1.28    0.460    0.850 

Effective search space used: 874911716973
 
    reads 
 chr10 chr10 126177087 126186623 57M9460N19M                  ||||||||||||||||||||||||||||||||||||||||||||||||||||||||||||||||||||||||||||||||||||||||||||||||||||||||||||||||||||||||||||||||||||||||||||||||||||||||||||||||||||||||||||||||||||||||||||||||||||||||||||||||TATGCCTGTGGCATCAAAG
chr10 chr10 126177098 126186634 46M9460N30M                  ||||||||||||||||||||||||||||||||||||||||||||||||||||||||||||||||||||||||||||||||||||||||||||||||||||||||||||||||||||||||||||||||||||||||||||||||||||||||||||||||||||||||||||||||||||||||||||||||||||||||||||||||TATGCCTGTGGCATCAAAGCCGAGGTGGTG
chr10 chr10 126177111 126186647 33M9460N43M                  ||||||||||||||||||||||||||||||||||||||||||||||||||||||||||||||||||||||||||||||||||||||||||||||||||||||||||||||||||||||||||||||||||||||||||||||||||||||||||||||||||||||||||||||||||||||||||||||||||||||||||||||||TATGCCTGTGGCATCAAAGCCGAGGTGGTGGGGAAGCCTTCTC
chr10 chr10 126177118 126186654 26M9460N50M                  ||||||||||||||||||||||||||||||||||||||||||||||||||||||||||||||||||||||||||||||||||||||||||||||||||||||||||||||||||||||||||||||||||||||||||||||||||||||||||||||||||||||||||||||||||||||||||||||||||||||||||||||||TATGCCTGTGGCATCAAAGCCGAGGTGGTGGGGAAGCCTTCTCCTGAGTT
chr10 chr10 126177130 126186666 14M9460N62M                  ||||||||||||||||||||||||||||||||||||||||||||||||||||||||||||||||||||||||||||||||||||||||||||||||||||||||||||||||||||||||||||||||||||||||||||||||||||||||||||||||||||||||||||||||||||||||||||||||||||||||||||||||TATGCCTGTGGCATCAAAGCCGAGGTGGTGGGGAAGCCTTCTCCTGAGTTTTTCAAGTCTGC
chr10 chr10 126177133 126186605 11M8385N64M1011N1M           ||||||||||||||||||||||||||||||||||||||||||||||||||||||||||||||||||||||||||||||||||||||||||||||||||||||||||||||||||||||||||||||||||||||||||||||||||||||||||||||||||||||||||||||||||||||||||||||||||||||||||||||||T
chr10 chr10 126177136 126186672 8M9460N68M                   ||||||||||||||||||||||||||||||||||||||||||||||||||||||||||||||||||||||||||||||||||||||||||||||||||||||||||||||||||||||||||||||||||||||||||||||||||||||||||||||||||||||||||||||||||||||||||||||||||||||||||||||||TATGCCTGTGGCATCAAAGCCGAGGTGGTGGGGAAGCCTTCTCCTGAGTTTTTCAAGTCTGCCCTGCA
chr10 chr10 126177140 126186612 4M8385N64M1011N8M            ||||||||||||||||||||||||||||||||||||||||||||||||||||||||||||||||||||||||||||||||||||||||||||||||||||||||||||||||||||||||||||||||||||||||||||||||||||||||||||||||||||||||||||||||||||||||||||||||||||||||||||||||TATGCCTG
chr10 chr10 126177143 126186615 1M8385N64M1011N11M           ||||||||||||||||||||||||||||||||||||||||||||||||||||||||||||||||||||||||||||||||||||||||||||||||||||||||||||||||||||||||||||||||||||||||||||||||||||||||||||||||||||||||||||||||||||||||||||||||||||||||||||||||TATGCCTGTGG
chr10 chr10 126185530 126186617 63M1011N13M                  ||||||||||||||||||||||||||||||||||||||||||||||||||||||||||||||||||||||||||||||||||||||||||||||||||||||||||||||||||||||||||||||||||||||||||||||||||||||||||||||||||||||||||||||||||||||||||||||||||||||||||||||||AATGCCTGTGGCA
chr10 chr10 126185534 126186621 59M1011N17M                  ||||||||||||||||||||||||||||||||||||||||||||||||||||||||||||||||||||||||||||||||||||||||||||||||||||||||||||||||||||||||||||||||||||||||||||||||||||||||||||||||||||||||||||||||||||||||||||||||||||||||||||||||TATGCCTGTGGCATCAA
chr10 chr10 126185537 126186624 56M1011N20M                  ||||||||||||||||||||||||||||||||||||||||||||||||||||||||||||||||||||||||||||||||||||||||||||||||||||||||||||||||||||||||||||||||||||||||||||||||||||||||||||||||||||||||||||||||||||||||||||||||||||||||||||||||TATGCCTGTGGCATCAAAGC
chr10 chr10 126185540 126186627 53M1011N23M                  ||||||||||||||||||||||||||||||||||||||||||||||||||||||||||||||||||||||||||||||||||||||||||||||||||||||||||||||||||||||||||||||||||||||||||||||||||||||||||||||||||||||||||||||||||||||||||||||||||||||||||||||||TATGCCTGTGGCATCAAAGCCGA
chr10 chr10 126185543 126186630 50M1011N26M                  ||||||||||||||||||||||||||||||||||||||||||||||||||||||||||||||||||||||||||||||||||||||||||||||||||||||||||||||||||||||||||||||||||||||||||||||||||||||||||||||||||||||||||||||||||||||||||||||||||||||||||||||||TATGCCTGTGGCATCAAAGCCGAGAT
chr10 chr10 126185546 126186633 47M1011N29M                  ||||||||||||||||||||||||||||||||||||||||||||||||||||||||||||||||||||||||||||||||||||||||||||||||||||||||||||||||||||||||||||||||||||||||||||||||||||||||||||||||||||||||||||||||||||||||||||||||||||||||||||||||TATGCCTGTGGCATCAAAGCCGAGGTGGT
chr10 chr10 126185549 126186636 44M1011N32M                  ||||||||||||||||||||||||||||||||||||||||||||||||||||||||||||||||||||||||||||||||||||||||||||||||||||||||||||||||||||||||||||||||||||||||||||||||||||||||||||||||||||||||||||||||||||||||||||||||||||||||||||||||TATGCCTGTGGCATCAAAGCCGAGGTGGTGGG
chr10 chr10 126185552 126186639 41M1011N35M                  ||||||||||||||||||||||||||||||||||||||||||||||||||||||||||||||||||||||||||||||||||||||||||||||||||||||||||||||||||||||||||||||||||||||||||||||||||||||||||||||||||||||||||||||||||||||||||||||||||||||||||||||||TATGCCTGTGGCATCAAAGCCGAGGTGGTGGGGAG
chr10 chr10 126185555 126186642 38M1011N38M                  ||||||||||||||||||||||||||||||||||||||||||||||||||||||||||||||||||||||||||||||||||||||||||||||||||||||||||||||||||||||||||||||||||||||||||||||||||||||||||||||||||||||||||||||||||||||||||||||||||||||||||||||||TATGCCTGTGGCATCAAAGCCGAGGTGGTGGGGAAAGC
chr10 chr10 126185559 126186646 34M1011N42M                  ||||||||||||||||||||||||||||||||||||||||||||||||||||||||||||||||||||||||||||||||||||||||||||||||||||||||||||||||||||||||||||||||||||||||||||||||||||||||||||||||||||||||||||||||||||||||||||||||||||||||||||||||TATGCCTGTGGCATCAAAGCCGAGGTGGTGGGGAAGCCTTCT
chr10 chr10 126185562 126186649 31M1011N45M                  ||||||||||||||||||||||||||||||||||||||||||||||||||||||||||||||||||||||||||||||||||||||||||||||||||||||||||||||||||||||||||||||||||||||||||||||||||||||||||||||||||||||||||||||||||||||||||||||||||||||||||||||||TATGCCTGTGGCATCAAAGCCGAGGTGGTGGGGAAGCCTTCTCCT
chr10 chr10 126185565 126186652 28M1011N48M                  ||||||||||||||||||||||||||||||||||||||||||||||||||||||||||||||||||||||||||||||||||||||||||||||||||||||||||||||||||||||||||||||||||||||||||||||||||||||||||||||||||||||||||||||||||||||||||||||||||||||||||||||||TATGCCTGTGGCATCAAAGCCGAGGTGGTGGGGAAGCCTTCTCCTGAG
chr10 chr10 126185569 126186656 24M1011N52M                  ||||||||||||||||||||||||||||||||||||||||||||||||||||||||||||||||||||||||||||||||||||||||||||||||||||||||||||||||||||||||||||||||||||||||||||||||||||||||||||||||||||||||||||||||||||||||||||||||||||||||||||||||TATGCCTGTGGCATCAAAGCCGAGGTGGTGGGGAAGCCTTCTCCTGAGTTTT
chr10 chr10 126185573 126186660 20M1011N56M                  ||||||||||||||||||||||||||||||||||||||||||||||||||||||||||||||||||||||||||||||||||||||||||||||||||||||||||||||||||||||||||||||||||||||||||||||||||||||||||||||||||||||||||||||||||||||||||||||||||||||||||||||||TATGCCTGTGGCATCAAAGCCGAGGTGGTGGGGAAGCCTTCTCCTGAGTTTTTCAA
chr10 chr10 126185577 126186664 16M1011N60M                  ||||||||||||||||||||||||||||||||||||||||||||||||||||||||||||||||||||||||||||||||||||||||||||||||||||||||||||||||||||||||||||||||||||||||||||||||||||||||||||||||||||||||||||||||||||||||||||||||||||||||||||||||TATGCCTGTGGCATCAAAGCCGAGGTGGTGGGGAAGCCTTCTCCTGAGTTTTTCAAGTCA
chr10 chr10 126185580 126186667 13M1011N63M                  ||||||||||||||||||||||||||||||||||||||||||||||||||||||||||||||||||||||||||||||||||||||||||||||||||||||||||||||||||||||||||||||||||||||||||||||||||||||||||||||||||||||||||||||||||||||||||||||||||||||||||||||||TATGCCTGTGGCATCAAAGCCGAGGTGGTGGGGAAGCCTTCTCCTGAGTTTTTCAAGTCTGCC
chr10 chr10 126185583 126186670 10M1011N66M                  ||||||||||||||||||||||||||||||||||||||||||||||||||||||||||||||||||||||||||||||||||||||||||||||||||||||||||||||||||||||||||||||||||||||||||||||||||||||||||||||||||||||||||||||||||||||||||||||||||||||||||||||||TATGCCTGTGGCATCAAAGCCGAGGTGGTGGGGAAGCCTTCTCCTGAGTTTTTCAAGTCTGCCCTG
chr10 chr10 126185586 126186673 7M1011N69M                   ||||||||||||||||||||||||||||||||||||||||||||||||||||||||||||||||||||||||||||||||||||||||||||||||||||||||||||||||||||||||||||||||||||||||||||||||||||||||||||||||||||||||||||||||||||||||||||||||||||||||||||||||TATGCCTGTGGCATCAAAGCCGAGGTGGTGGGGAAGCCTTCTCCTGAGTTTTTCAAGTCTGCCCTGCAA
chr10 chr10 126185589 126186676 4M1011N72M                   ||||||||||||||||||||||||||||||||||||||||||||||||||||||||||||||||||||||||||||||||||||||||||||||||||||||||||||||||||||||||||||||||||||||||||||||||||||||||||||||||||||||||||||||||||||||||||||||||||||||||||||||||TATGCCTGTGGCATCAAAGCCGAGGTGGTGGGGAAGCCTTCTCCTGAGTTTTTCAAGTCTGCCCTGCAAGCG
chr10 chr10 126185592 126186679 1M1011N75M                   ||||||||||||||||||||||||||||||||||||||||||||||||||||||||||||||||||||||||||||||||||||||||||||||||||||||||||||||||||||||||||||||||||||||||||||||||||||||||||||||||||||||||||||||||||||||||||||||||||||||||||||||||TATGCCTGTGGCATCAAAGCCGAGGTGGTGGGGAAGCCTTCTCCTGAGTTTTTCAAGTCTGCCCTGCAAGCGATA
chr10 chr10 126186482 126186558 76M                                                                                                                AGCATCCTGCTTCTTCAAGGCCCTTGACTCTTGGGGGCACATTTGGGACAGTGTTTCCGTGGCTGGGCATACACCT
chr10 chr10 126186551 126186627 76M                                                                                                                                                                                     TACACCTGTTCCTTGATCCCAAACTTATGCCATGTCCCTCTCTCTTTTCCCAGTATGCCTGTGGCATCAAAGCCGA
chr10 chr10 126186602 126186678 76M                                                                                                                                                                                                                                        AGTATGCCTGTGGCATCAAAGCCGAGGTGGTGGGGAAGCCTTCTCCTGAGTTTTTCAAGTCTGCCCTGCAAGCGAT
chr10 chr10 126186605 126186681 76M                                                                                                                                                                                                                                           ATGCCTGTGGCATCAAAGCCGAGGTGGTGGGGAAGCCTTCTCCTGAGTTTTTCAAGTCTGCCCTGCAAGCGATAGG
chr10 chr10 126186609 126186685 76M                                                                                                                                                                                                                                               CTGTGGCATCAAGGCCGAGGTGGTGGGGAAGCCTTCTCCTGAGTTTTTCAAGTCTGCCCTGCAAGCGATAGGAGTG
chr10 chr10 126186612 126186688 76M                                                                                                                                                                                                                                                  TGGCATCAAAGCCGAGGTGGTGGGGAAGCCTTCTCCTGAGTTTTTCAAGTCTGCCCTGCAAGCGATAGGAGTGGAA
chr10 chr10 126186615 126186691 76M                                                                                                                                                                                                                                                     CATCAAAGCCGAGGTGGTGGGGAAGCCTTCTCCTGAGTTTTTCAAGTCTGCCCTGCAAGCGATAGGAGTGGAAGCC
chr10 chr10 126186618 126186694 76M                                                                                                                                                                                                                                                        CAAAGCCGAGGTGGTGGGGAAGCCTTCTCCTGAGTTTTTCAAGTCTGCCCTGCAAGCGATAGGAGTGGAAGCCCAC
chr10 chr10 126186621 126186697 76M                                                                                                                                                                                                                                                           AGCCGAGGTGGTGGGGAAGCGTTCTCCTGAGTTTTTCAAGTCTGCCCTGCAAGCGATAGGAGTGGAAGCCCACCAG
chr10 chr10 126186624 126186700 76M                                                                                                                                                                                                                                                              CGAGGTGGTGGGGAAGCCTTCTCCTGAGTTTTTCAAGTCTGCCCTGCAAGCGATAGGAGTGGAAGCCCACCAGAAA
chr10 chr10 126186626 127668734 71M127668730F5M                                                                                                                                                                                                                                                    AGGTGGTGGGTAAGCCTTCTCCTGAGTTTTTCAAGTCTGCCCTGCAAGCGATAGGAGTGGAAGCCCACTAG AAATC
chr10 chr10 126186642 127668750 55M127668730F21M                                                                                                                                                                                                                                                                   TTCTCCTGAGTTTTTCAAGTCTGCCCTGCAAGCGATAGGAGTGGAAGCCCACCAG AAATCATGCCACCCTCAAAGC
chr10 chr10 126186651 127668759 46M127668730F30M                                                                                                                                                                                                                                                                            GTTTTTCAAGTCTGCCCTGCAAGCGATAGGAGTGGAAGCCCACCAG AAATCATGCCACCCTCAAAGCCTCATCCAC
chr10 chr10 126186670 127668778 27M127668730F49M                                                                                                                                                                                                                                                                                               CAAGCGATAGGAGTGGAAGCCCACCAG AAATCATGCCACCCTCAAAGCCTCATCCACCTGTCGTGGGCAAAGTGAC
chr10 chr10 126186671 127668779 26M127668730F50M                                                                                                                                                                                                                                                                                                AAGCGATAGGAGTGGAAGCCCACCAG AAATCATGCCACCCTCAAAGCCTCATCCTCCTGTCGTGGGCAAAGTGACT
chr10 chr10 127668724 127668800 76M                                                                                                                                                                                                                                                                                                                                   TTCAGAAATCATGCCACCCTCAAAGCCTCATCCACCTGTCGTGGGCAAAGTGACTCATCACAGCATTGAATTATAC
chr10 chr10 127668727 127668803 76M                                                                                                                                                                                                                                                                                                                                      AGAAATCATGCCACCCTCAAAGCCTCATCCACCTGTCGTGGGCAAAGTGACTCATCACAGCATTGAATTATACTGG
chr10 chr10 127668730 127668806 76M                                                                                                                                                                                                                                                                                                                                         AATCATGCCACCCTCAAAGCCTCATCCACCTGTCGTGGGCAAAGTGACTCATCACAGCATTGAATTATACTGGGAT
chr10 chr10 127668735 127668811 76M                                                                                                                                                                                                                                                                                                                                              TGCCACCCTCAAAGCCTCATCCACCTGTCGTGGGCAAAGTGACTCATCACAGCATTGAATTATACTGGGATCTGGA
chr10 chr10 127668738 127668814 76M                                                                                                                                                                                                                                                                                                                                                 CACCCTCAAAGCCTCATCCACCTGTCGTGGGCAAAGTGACTCATCACAGCATTGAATTATACTGGGATCTGGAAAA
chr10 chr10 127668741 127668817 76M                                                                                                                                                                                                                                                                                                                                                    CCTCAAAGCCTCATCCACCTGTCGTGGGCAAAGTGACTCATCACAGCATTGAATTATACTGGGATCTGGAAAAGAA
chr10 chr10 127668744 127668820 76M                                                                                                                                                                                                                                                                                                                                                       CAAAGCCTCATCCACCTGTCGTGGGCAAAGTGACTCATCACAGCATTGAATTATACTGGGATCTGGAAAAGAAAGC
chr10 chr10 127668747 127668823 76M                                                                                                                                                                                                                                                                                                                                                          AGCCTCATCCACCTGTCGTGGGCAAAGTGACTCATCACAGCATTGAATTATACTGGGATCTGGAAAAGAAAGCCAA
chr10 chr10 127668750 127668826 76M                                                                                                                                                                                                                                                                                                                                                             CTCATCCACCTGTCGTGGGCAAAGTGACTCATCACAGCATTGAATTATACTGGGATCTGGAAAAGAAAGCCAAACG
chr10 chr10 127668753 127668829 76M                                                                                                                                                                                                                                                                                                                                                                ATCCACCTGTCGTGGGCAAAGTGACTCATCACAGCATTGAATTATACTGGGATCTGGAAAAGAAAGCAAAACGCCA
chr10 chr10 127668756 127668832 76M                                                                                                                                                                                                                                                                                                                                                                   CACCTGTCGTGGGCAAAGTGACTCATCACAGCATTGAATTATACTGGGATCTGGAAAAGAAAGCCAAACGCCAAGG
chr10 chr10 127668762 127668838 76M                                                                                                                                                                                                                                                                                                                                                                         TCGTGGGCAAAGTGACTCATCACAGCATTGAATTATACTGGGATCTGGAAAAGAAAGCCAAACGCCAAGGACCTCG
chr10 chr10 127668765 127668841 76M                                                                                                                                                                                                                                                                                                                                                                            TGGGCAAAGTGACTCATCACAGCATTGAATTATACTGGGATCTGGAAAAGAAAGCCAAACGCCAAGGACCTCAAGA
chr10 chr10 127668768 127668844 76M                                                                                                                                                                                                                                                                                                                                                                               GCAAAGTGACTCATCACAGCATTGAATTATACTGGGATCTGGAAAAGAAAGCCAAACGCCAGGGACCTCAAGAGCA
chr10 chr10 127668771 127668847 76M                                                                                                                                                                                                                                                                                                                                                                                  AAGTGACTCATCACAGCATTGAATTATACTGGGATCTGGAAAAGAAAGCCAAACGCCAAGGACCTCAAGAGCAGTG
chr10 chr10 127668775 127668851 76M                                                                                                                                                                                                                                                                                                                                                                                      GACTCATCACAGCATTGAATTATACTGGGATCTGGAAAAGAAAGCCAAACGCCAAGGACCTCAAGAGCAGTGGTTC
chr10 chr10 127668779 127668855 76M                                                                                                                                                                                                                                                                                                                                                                                          CATCACAGCATTGAATTATACTGGGATCTGGAAAAGAAAGCCAAACGCCAAGGACCTCAAGAGCAGTGGTTCAGGT
chr10 chr10 127668782 127668858 76M                                                                                                                                                                                                                                                                                                                                                                                             CACAGCATTGAATTATACTGGGATCTGGAAAAGAAAGCCAAACGCCAAGGACCTCAAGAGCAGTGGTTCAGGTTCT
chr10 chr10 127668785 127668861 76M                                                                                                                                                                                                                                                                                                                                                                                                AGCATTGAATTATACTGGGATCTGGAAAAGAAAGCCAAACGCCAAGGACCTCAAGAGCAGTGGTTCAGGTTCTCGG
chr10 chr10 127668788 127668864 76M                                                                                                                                                                                                                                                                                                                                                                                                   ATTGAATTATACTGGGATCTGGAAAAGAAAGCCAAACGCCAAGGACCTCAAGAGCAGTGGTTCAGGTTCTCGATTG
chr10 chr10 127668791 127668867 76M                                                                                                                                                                                                                                                                                                                                                                                                      GAATTATACTGGGATCTGGAAAAGAAAGCCAAACGCCAAGGACCTCAAGAGCAGTGGTTCAGGTTCTCGATTGAAG
chr10 chr10 127668794 127668870 76M                                                                                                                                                                                                                                                                                                                                                                                                         TTATACTGGGATCTGGAAAAGAAAGCCAAACGCCAAGGACCTCAAGAGCAGTGGTTCAGGTTCTCGATTGAAGAAG
chr10 chr10 127668797 127668873 76M                                                                                                                                                                                                                                                                                                                                                                                                            TACTGGGATCTGGAAAAGAAAGCCAAACGCCAAGGACCTCAAGAGCAGTGGTTCAGGTTCTCGATTGAAGAAGAAG
chr10 chr10 127668800 127668876 76M                                                                                                                                                                                                                                                                                                                                                                                                               TGGGATCTGGAAAAGAAAGCCAAACGCCAAGGACCTCAAGAGCAGTGGTTCAGGTTCTCGATTGAAGAAGAAGACC
chr10 chr10 127668803 127668879 76M                                                                                                                                                                                                                                                                                                                                                                                                                  GATCTGGAAAAGAAAGCCAAACGCCAAGGACCTCAAGAGCAGTGGTTCAGGTTCTCGATTGAAGAAGAAGACCCCA
chr10 chr10 127668806 127668882 76M                                                                                                                                                                                                                                                                                                                                                                                                                     CTGGAAAAGAAAGCCAAACGCCAAGGACCTCAAGAGCAGTGGTTCAGGTTCTCGATTGAAGAAGAAGACCCCAAAA
chr10 chr10 127668809 127668885 76M                                                                                                                                                                                                                                                                                                                                                                                                                        GAAAAGAAAGCCAAACGCCAAGGACCTCAAGAGCAGTGGTTCAGGTTCTCGATTGAAGAAGAAGACCCCAAAATGC
chr10 chr10 127668812 127668888 76M                                                                                                                                                                                                                                                                                                                                                                                                                           AAGAAAGCCAAACGCCAAGGACCTCAAGAGCAGTGGTTCAGGTTCTCGATTGAAGAACAAGACCCCAAAATGCACA
chr10 chr10 127668818 127668894 76M                                                                                                                                                                                                                                                                                                                                                                                                                                 GCCAAACGCCAAGGACCTCAAGAGCAGTGGTTCAGGTTCTCGATTGAAGAAGAAGACCCCAAAATGCACACTTATG
chr10 chr10 127668821 127668897 76M                                                                                                                                                                                                                                                                                                                                                                                                                                    AAACGCCAAGGACCTCAAGAGCAGTGGTTCAGGTTCTCGATTGAAGAAGAAGACCCCAAAATGCACACTTATGGTA
chr10 chr10 127668824 127668900 76M                                                                                                                                                                                                                                                                                                                                                                                                                                       CGCCAAGGACCTCAAGAGCAGTGGTTCAGGTTCTCGATTGAAGAAGAAGACCCCAAAATGCACACTTATGGTATCA
chr10 chr10 127668827 127668903 76M                                                                                                                                                                                                                                                                                                                                                                                                                                          CAAGGACCTCAAGAGCAGTGGTTCAGGTTCTCGATTGAAGAAGAAGACCCCAAAATGCACACTTATGGTATCATTT
chr10 chr10 127668830 127668906 76M                                                                                                                                                                                                                                                                                                                                                                                                                                             GGACCTCAAGAGCAGTGGTTCAGGTTCTCGATTGAAGAAGAAGACCCCAAAATGCACACTTATGGTATCATTTATA
chr10 chr10 127668834 127677122 73M8212N3M                                                                                                                                                                                                                                                                                                                                                                                                                                          CTCAAGAGCAGTGGTTCAGGTTCTCGATTGAAGAAGAAGACCCCAAAATGCACACTTATGGTATCATTTATAC||||||||||||||||||||||||||||||||||||||||||||||||||||||||||||||||||||||||||||||||||||||||||||||||||||||||||||||||||||||||
chr10 chr10 127668837 127677125 70M8212N6M                                                                                                                                                                                                                                                                                                                                                                                                                                             AAGAGCAGTGGTTCAGGTTCTCGATTGAAGAAGAAGACCCCAAAATGCACACTTATGGTATCATTTATAC||||||||||||||||||||||||||||||||||||||||||||||||||||||||||||||||||||||||||||||||||||||||||||||||||||||||||||||||||||||||
chr10 chr10 127668841 127677129 66M8212N10M                                                                                                                                                                                                                                                                                                                                                                                                                                                GCAGTGGTTCAGGTTCTCGATTGAAGAAGAAGACCCCAAAATGCACACTTATGGTATCATTTATAC||||||||||||||||||||||||||||||||||||||||||||||||||||||||||||||||||||||||||||||||||||||||||||||||||||||||||||||||||||||||
chr10 chr10 127668847 127677135 60M8212N16M                                                                                                                                                                                                                                                                                                                                                                                                                                                      GTTCAGGTTCTCGATTGAAGAAGAAGACCCCAAAATGCACACTTATGGTATCATTTATAC||||||||||||||||||||||||||||||||||||||||||||||||||||||||||||||||||||||||||||||||||||||||||||||||||||||||||||||||||||||||
chr10 chr10 127668850 127668926 76M                                                                                                                                                                                                                                                                                                                                                                                                                                                                 CAGGTTCTCGATTGAAGAAGAAGACCCCAAAATGCACACTTATGGTATCATTTATACGTAGGTGCAGTGTTGAATT
chr10 chr10 127668853 127668929 76M                                                                                                                                                                                                                                                                                                                                                                                                                                                                    GTTCTCGATTGAAGAAGAAGACCCCAAAATGCACACTTATGGTATCATTTATACGTAGGTGCAGTGTTGAATTGCT
chr10 chr10 127668856 127677144 51M8212N25M                                                                                                                                                                                                                                                                                                                                                                                                                                                               CTCGATTGAAGAAGAAGACCCCAAAATGCACACTTATGGTATCATTTATAC||||||||||||||||||||||||||||||||||||||||||||||||||||||||||||||||||||||||||||||||||||||||||||||||||||||||||||||||||||||||
chr10 chr10 127668860 127677148 47M8212N29M                                                                                                                                                                                                                                                                                                                                                                                                                                                                   ATTGAAGAAGAAGACCCCAAAATGCACACTTATGGTATCATTTATAC||||||||||||||||||||||||||||||||||||||||||||||||||||||||||||||||||||||||||||||||||||||||||||||||||||||||||||||||||||||||
chr10 chr10 127668864 127668940 76M                                                                                                                                                                                                                                                                                                                                                                                                                                                                               AAGAAGAAGACCCCAAAATGCACACTTATGGTATCATTTATACGTAGGTGCAGTGTTGAATTGCTTGCCACTTTGC
chr10 chr10 127668867 127668943 76M                                                                                                                                                                                                                                                                                                                                                                                                                                                                                  AAGAAGACCCCAAAATGCACACTTATGGTATCATTTATACGTAGGTGCAGTGTTGAATTGCTTGCCACTTTGCCTT
chr10 chr10 127668870 127677158 37M8212N39M                                                                                                                                                                                                                                                                                                                                                                                                                                                                             AAGACCCCAAAATGCACACTTATGGTATCTTTTATAC||||||||||||||||||||||||||||||||||||||||||||||||||||||||||||||||||||||||||||||||||||||||||||||||||||||||||||||||||||||||
chr10 chr10 127668873 127677161 34M8212N42M                                                                                                                                                                                                                                                                                                                                                                                                                                                                                ACCCCAAAATGCACACTTATGGTATCATTTATAC||||||||||||||||||||||||||||||||||||||||||||||||||||||||||||||||||||||||||||||||||||||||||||||||||||||||||||||||||||||||
chr10 chr10 127668876 127677164 31M8212N45M                                                                                                                                                                                                                                                                                                                                                                                                                                                                                   CCAAAATGCACACTTATGGTATCATTTATAC||||||||||||||||||||||||||||||||||||||||||||||||||||||||||||||||||||||||||||||||||||||||||||||||||||||||||||||||||||||||
chr10 chr10 127668879 127668955 76M                                                                                                                                                                                                                                                                                                                                                                                                                                                                                              AAATGCACACTTATGGTATCATTTATACGTAGGTGCAGTGTTGAATTGCTTGCCACTTTGCCTTACTTCCTGATTA
chr10 chr10 127668883 127677171 24M8212N52M                                                                                                                                                                                                                                                                                                                                                                                                                                                                                          GCACACTTATGGTATCATTTATAC||||||||||||||||||||||||||||||||||||||||||||||||||||||||||||||||||||||||||||||||||||||||||||||||||||||||||||||||||||||||
chr10 chr10 127668887 127668963 76M                                                                                                                                                                                                                                                                                                                                                                                                                                                                                                      ACTTATGGTATCATTTATACGTAGGTGCAGTGTTGAATTGCTTGCCACTTTGCCTTACTTCCTGATTAGCTGGAAT 
    5 pairs 
 -2:26
44:-1
49:33
36:68
36:68 
     TCGA-02-2483-01A chr10-chr10 126205839 127668729 ff 
 
27 14 51 2 56 56
GCCCAGCGGTGTGGAATGAGAGCGCTGCAGGTGCGCACCGGGAAGTTCAG AAATCATGCCACCCTCAAAGCCTCATCCACCTGTCGTGGGCAAAGTGACT
22233444445677899999999999999999999999999999999999 99999999999999999999999999999999999999999999999998
LHPP exon6(126205747-126205839) FANK1 exon2(127668728-127668906)
 
 blast search - human genome  
 left 
 >ref|NT_030059.13| Homo sapiens chromosome 10 genomic contig, GRCh37.p9 Primary 
Assembly
 gb|GL000099.1| Homo sapiens chromosome 10 genomic contig, GRCh37 reference primary 
assembly
Length=79420533

 Score = 93.5 bits (50),  Expect = 5e-17
 Identities = 50/50 (100%), Gaps = 0/50 (0%)
 Strand=Plus/Plus

Query  1         GCCCAGCGGTGTGGAATGAGAGCGCTGCAGGTGCGCACCGGGAAGTTCAG  50
                 ||||||||||||||||||||||||||||||||||||||||||||||||||
Sbjct  77010255  GCCCAGCGGTGTGGAATGAGAGCGCTGCAGGTGCGCACCGGGAAGTTCAG  77010304


>ref|NW_001838010.1| Homo sapiens chromosome 10 genomic contig, alternate assembly 
HuRef SCAF_1103279188229, whole genome shotgun sequence
 gb|DS486216.1| Homo sapiens SCAF_1103279188229 genomic scaffold, whole genome 
shotgun sequence
Length=2644411

 Score = 93.5 bits (50),  Expect = 5e-17
 Identities = 50/50 (100%), Gaps = 0/50 (0%)
 Strand=Plus/Plus

Query  1       GCCCAGCGGTGTGGAATGAGAGCGCTGCAGGTGCGCACCGGGAAGTTCAG  50
               ||||||||||||||||||||||||||||||||||||||||||||||||||
Sbjct  213431  GCCCAGCGGTGTGGAATGAGAGCGCTGCAGGTGCGCACCGGGAAGTTCAG  213480


>ref|NC_000010.10| Homo sapiens chromosome 10, GRCh37.p9 Primary Assembly
 gb|CM000672.1| Homo sapiens chromosome 10, GRCh37 primary reference assembly
Length=135534747

 Score = 93.5 bits (50),  Expect = 5e-17
 Identities = 50/50 (100%), Gaps = 0/50 (0%)
 Strand=Plus/Plus

Query  1          GCCCAGCGGTGTGGAATGAGAGCGCTGCAGGTGCGCACCGGGAAGTTCAG  50
                  ||||||||||||||||||||||||||||||||||||||||||||||||||
Sbjct  126205791  GCCCAGCGGTGTGGAATGAGAGCGCTGCAGGTGCGCACCGGGAAGTTCAG  126205840


>ref|AC_000142.1| Homo sapiens chromosome 10, alternate assembly HuRef, whole genome 
shotgun sequence
 gb|CM000471.1| Homo sapiens chromosome 10, whole genome shotgun sequence
Length=128985118

 Score = 93.5 bits (50),  Expect = 5e-17
 Identities = 50/50 (100%), Gaps = 0/50 (0%)
 Strand=Plus/Plus

Query  1          GCCCAGCGGTGTGGAATGAGAGCGCTGCAGGTGCGCACCGGGAAGTTCAG  50
                  ||||||||||||||||||||||||||||||||||||||||||||||||||
Sbjct  119892529  GCCCAGCGGTGTGGAATGAGAGCGCTGCAGGTGCGCACCGGGAAGTTCAG  119892578


>gb|CM000261.1| Homo sapiens chromosome 10, whole genome shotgun sequence
Length=129189614

 Score = 93.5 bits (50),  Expect = 5e-17
 Identities = 50/50 (100%), Gaps = 0/50 (0%)
 Strand=Plus/Plus

Query  1          GCCCAGCGGTGTGGAATGAGAGCGCTGCAGGTGCGCACCGGGAAGTTCAG  50
                  ||||||||||||||||||||||||||||||||||||||||||||||||||
Sbjct  119979684  GCCCAGCGGTGTGGAATGAGAGCGCTGCAGGTGCGCACCGGGAAGTTCAG  119979733


Lambda     K      H
    1.33    0.621     1.12 

Gapped
Lambda     K      H
    1.28    0.460    0.850 

Effective search space used: 681780354108
 
 right 
 >ref|NT_030059.13| Homo sapiens chromosome 10 genomic contig, GRCh37.p9 Primary 
Assembly
 gb|GL000099.1| Homo sapiens chromosome 10 genomic contig, GRCh37 reference primary 
assembly
Length=79420533

 Score = 93.5 bits (50),  Expect = 5e-17
 Identities = 50/50 (100%), Gaps = 0/50 (0%)
 Strand=Plus/Plus

Query  1         AAATCATGCCACCCTCAAAGCCTCATCCACCTGTCGTGGGCAAAGTGACT  50
                 ||||||||||||||||||||||||||||||||||||||||||||||||||
Sbjct  78473194  AAATCATGCCACCCTCAAAGCCTCATCCACCTGTCGTGGGCAAAGTGACT  78473243


>ref|NW_001838010.1| Homo sapiens chromosome 10 genomic contig, alternate assembly 
HuRef SCAF_1103279188229, whole genome shotgun sequence
 gb|DS486216.1| Homo sapiens SCAF_1103279188229 genomic scaffold, whole genome 
shotgun sequence
Length=2644411

 Score = 93.5 bits (50),  Expect = 5e-17
 Identities = 50/50 (100%), Gaps = 0/50 (0%)
 Strand=Plus/Plus

Query  1        AAATCATGCCACCCTCAAAGCCTCATCCACCTGTCGTGGGCAAAGTGACT  50
                ||||||||||||||||||||||||||||||||||||||||||||||||||
Sbjct  1678799  AAATCATGCCACCCTCAAAGCCTCATCCACCTGTCGTGGGCAAAGTGACT  1678848


>ref|NC_000010.10| Homo sapiens chromosome 10, GRCh37.p9 Primary Assembly
 gb|CM000672.1| Homo sapiens chromosome 10, GRCh37 primary reference assembly
Length=135534747

 Score = 93.5 bits (50),  Expect = 5e-17
 Identities = 50/50 (100%), Gaps = 0/50 (0%)
 Strand=Plus/Plus

Query  1          AAATCATGCCACCCTCAAAGCCTCATCCACCTGTCGTGGGCAAAGTGACT  50
                  ||||||||||||||||||||||||||||||||||||||||||||||||||
Sbjct  127668730  AAATCATGCCACCCTCAAAGCCTCATCCACCTGTCGTGGGCAAAGTGACT  127668779


>ref|AC_000142.1| Homo sapiens chromosome 10, alternate assembly HuRef, whole genome 
shotgun sequence
 gb|CM000471.1| Homo sapiens chromosome 10, whole genome shotgun sequence
Length=128985118

 Score = 93.5 bits (50),  Expect = 5e-17
 Identities = 50/50 (100%), Gaps = 0/50 (0%)
 Strand=Plus/Plus

Query  1          AAATCATGCCACCCTCAAAGCCTCATCCACCTGTCGTGGGCAAAGTGACT  50
                  ||||||||||||||||||||||||||||||||||||||||||||||||||
Sbjct  121357897  AAATCATGCCACCCTCAAAGCCTCATCCACCTGTCGTGGGCAAAGTGACT  121357946


>gb|CM000261.1| Homo sapiens chromosome 10, whole genome shotgun sequence
Length=129189614

 Score = 93.5 bits (50),  Expect = 5e-17
 Identities = 50/50 (100%), Gaps = 0/50 (0%)
 Strand=Plus/Plus

Query  1          AAATCATGCCACCCTCAAAGCCTCATCCACCTGTCGTGGGCAAAGTGACT  50
                  ||||||||||||||||||||||||||||||||||||||||||||||||||
Sbjct  121444157  AAATCATGCCACCCTCAAAGCCTCATCCACCTGTCGTGGGCAAAGTGACT  121444206


>gb|CM000500.1| Homo sapiens chromosome 10, whole genome shotgun sequence
Length=128985077

 Score = 87.9 bits (47),  Expect = 2e-15
 Identities = 49/50 (98%), Gaps = 0/50 (0%)
 Strand=Plus/Plus

Query  1          AAATCATGCCACCCTCAAAGCCTCATCCACCTGTCGTGGGCAAAGTGACT  50
                  ||||||||||||||||||||| ||||||||||||||||||||||||||||
Sbjct  121357942  AAATCATGCCACCCTCAAAGCTTCATCCACCTGTCGTGGGCAAAGTGACT  121357991


>gb|DS990854.1| Homo sapiens SCAF_1112675837174 genomic scaffold, whole genome 
shotgun sequence
Length=2644434

 Score = 87.9 bits (47),  Expect = 2e-15
 Identities = 49/50 (98%), Gaps = 0/50 (0%)
 Strand=Plus/Plus

Query  1        AAATCATGCCACCCTCAAAGCCTCATCCACCTGTCGTGGGCAAAGTGACT  50
                ||||||||||||||||||||| ||||||||||||||||||||||||||||
Sbjct  1678839  AAATCATGCCACCCTCAAAGCTTCATCCACCTGTCGTGGGCAAAGTGACT  1678888


Lambda     K      H
    1.33    0.621     1.12 

Gapped
Lambda     K      H
    1.28    0.460    0.850 

Effective search space used: 681780354108
 
 blast search - nt  
 left 
 >ref|XM_003816293.1| PREDICTED: Pan paniscus phospholysine phosphohistidine inorganic 
pyrophosphate phosphatase (LHPP), mRNA
Length=1079

 Score = 93.5 bits (50),  Expect = 6e-17
 Identities = 50/50 (100%), Gaps = 0/50 (0%)
 Strand=Plus/Plus

Query  1    GCCCAGCGGTGTGGAATGAGAGCGCTGCAGGTGCGCACCGGGAAGTTCAG  50
            ||||||||||||||||||||||||||||||||||||||||||||||||||
Sbjct  818  GCCCAGCGGTGTGGAATGAGAGCGCTGCAGGTGCGCACCGGGAAGTTCAG  867


>gb|HQ258160.1| Synthetic construct Homo sapiens clone IMAGE:100072469 phospholysine 
phosphohistidine inorganic pyrophosphate phosphatase 
(LHPP) gene, encodes complete protein
Length=847

 Score = 93.5 bits (50),  Expect = 6e-17
 Identities = 50/50 (100%), Gaps = 0/50 (0%)
 Strand=Plus/Plus

Query  1    GCCCAGCGGTGTGGAATGAGAGCGCTGCAGGTGCGCACCGGGAAGTTCAG  50
            ||||||||||||||||||||||||||||||||||||||||||||||||||
Sbjct  686  GCCCAGCGGTGTGGAATGAGAGCGCTGCAGGTGCGCACCGGGAAGTTCAG  735


>ref|NM_022126.3| Homo sapiens phospholysine phosphohistidine inorganic pyrophosphate 
phosphatase (LHPP), transcript variant 1, mRNA
Length=1703

 Score = 93.5 bits (50),  Expect = 6e-17
 Identities = 50/50 (100%), Gaps = 0/50 (0%)
 Strand=Plus/Plus

Query  1    GCCCAGCGGTGTGGAATGAGAGCGCTGCAGGTGCGCACCGGGAAGTTCAG  50
            ||||||||||||||||||||||||||||||||||||||||||||||||||
Sbjct  758  GCCCAGCGGTGTGGAATGAGAGCGCTGCAGGTGCGCACCGGGAAGTTCAG  807


>gb|BC113629.1| Homo sapiens phospholysine phosphohistidine inorganic pyrophosphate 
phosphatase, mRNA (cDNA clone MGC:142189 IMAGE:8322681), 
complete cds
Length=1591

 Score = 93.5 bits (50),  Expect = 6e-17
 Identities = 50/50 (100%), Gaps = 0/50 (0%)
 Strand=Plus/Plus

Query  1    GCCCAGCGGTGTGGAATGAGAGCGCTGCAGGTGCGCACCGGGAAGTTCAG  50
            ||||||||||||||||||||||||||||||||||||||||||||||||||
Sbjct  758  GCCCAGCGGTGTGGAATGAGAGCGCTGCAGGTGCGCACCGGGAAGTTCAG  807


>gb|BC113631.1| Homo sapiens phospholysine phosphohistidine inorganic pyrophosphate 
phosphatase, mRNA (cDNA clone MGC:142191 IMAGE:8322683), 
complete cds
Length=1574

 Score = 93.5 bits (50),  Expect = 6e-17
 Identities = 50/50 (100%), Gaps = 0/50 (0%)
 Strand=Plus/Plus

Query  1    GCCCAGCGGTGTGGAATGAGAGCGCTGCAGGTGCGCACCGGGAAGTTCAG  50
            ||||||||||||||||||||||||||||||||||||||||||||||||||
Sbjct  758  GCCCAGCGGTGTGGAATGAGAGCGCTGCAGGTGCGCACCGGGAAGTTCAG  807


>dbj|AK055532.1| Homo sapiens cDNA FLJ30970 fis, clone HEART2000444, highly similar 
to Homo sapiens phospholysine phosphohistidine inorganic 
pyrophosphate phosphatase (LHPP), mRNA
Length=1613

 Score = 93.5 bits (50),  Expect = 6e-17
 Identities = 50/50 (100%), Gaps = 0/50 (0%)
 Strand=Plus/Plus

Query  1    GCCCAGCGGTGTGGAATGAGAGCGCTGCAGGTGCGCACCGGGAAGTTCAG  50
            ||||||||||||||||||||||||||||||||||||||||||||||||||
Sbjct  687  GCCCAGCGGTGTGGAATGAGAGCGCTGCAGGTGCGCACCGGGAAGTTCAG  736


>emb|AL391708.8| Human DNA sequence from clone RP11-45O7 on chromosome 10 Contains 
part of the LHPP gene for phospholysine phosphohistidine 
inorganic pyrophosphate phosphatase, complete sequence
Length=53675

 Score = 93.5 bits (50),  Expect = 6e-17
 Identities = 50/50 (100%), Gaps = 0/50 (0%)
 Strand=Plus/Plus

Query  1     GCCCAGCGGTGTGGAATGAGAGCGCTGCAGGTGCGCACCGGGAAGTTCAG  50
             ||||||||||||||||||||||||||||||||||||||||||||||||||
Sbjct  5487  GCCCAGCGGTGTGGAATGAGAGCGCTGCAGGTGCGCACCGGGAAGTTCAG  5536


>dbj|AB049629.1| Homo sapiens lhpp mRNA for phospholysine phosphohistidine inorganic 
pyrophosphate phosphatase, complete cds
Length=1814

 Score = 93.5 bits (50),  Expect = 6e-17
 Identities = 50/50 (100%), Gaps = 0/50 (0%)
 Strand=Plus/Plus

Query  1    GCCCAGCGGTGTGGAATGAGAGCGCTGCAGGTGCGCACCGGGAAGTTCAG  50
            ||||||||||||||||||||||||||||||||||||||||||||||||||
Sbjct  832  GCCCAGCGGTGTGGAATGAGAGCGCTGCAGGTGCGCACCGGGAAGTTCAG  881


>ref|XM_003794074.1| PREDICTED: Otolemur garnettii phospholysine phosphohistidine 
inorganic pyrophosphate phosphatase, transcript variant 2 (LHPP), 
mRNA
Length=813

 Score = 87.9 bits (47),  Expect = 3e-15
 Identities = 49/50 (98%), Gaps = 0/50 (0%)
 Strand=Plus/Plus

Query  1    GCCCAGCGGTGTGGAATGAGAGCGCTGCAGGTGCGCACCGGGAAGTTCAG  50
            ||||||||||||||||||||||||||||||||||| ||||||||||||||
Sbjct  667  GCCCAGCGGTGTGGAATGAGAGCGCTGCAGGTGCGGACCGGGAAGTTCAG  716


>ref|XM_003794073.1| PREDICTED: Otolemur garnettii phospholysine phosphohistidine 
inorganic pyrophosphate phosphatase, transcript variant 1 (LHPP), 
mRNA
Length=813

 Score = 87.9 bits (47),  Expect = 3e-15
 Identities = 49/50 (98%), Gaps = 0/50 (0%)
 Strand=Plus/Plus

Query  1    GCCCAGCGGTGTGGAATGAGAGCGCTGCAGGTGCGCACCGGGAAGTTCAG  50
            ||||||||||||||||||||||||||||||||||| ||||||||||||||
Sbjct  667  GCCCAGCGGTGTGGAATGAGAGCGCTGCAGGTGCGGACCGGGAAGTTCAG  716


>ref|XM_003277767.1| PREDICTED: Nomascus leucogenys phospholysine phosphohistidine 
inorganic pyrophosphate phosphatase-like, transcript variant 
1 (LOC100594200), mRNA
Length=1602

 Score = 87.9 bits (47),  Expect = 3e-15
 Identities = 49/50 (98%), Gaps = 0/50 (0%)
 Strand=Plus/Plus

Query  1    GCCCAGCGGTGTGGAATGAGAGCGCTGCAGGTGCGCACCGGGAAGTTCAG  50
            ||||||||||||||||||||||||||||||||||| ||||||||||||||
Sbjct  752  GCCCAGCGGTGTGGAATGAGAGCGCTGCAGGTGCGGACCGGGAAGTTCAG  801


>ref|XM_002821242.1| PREDICTED: Pongo abelii phospholysine phosphohistidine inorganic 
pyrophosphate phosphatase (LHPP), mRNA
Length=1629

 Score = 87.9 bits (47),  Expect = 3e-15
 Identities = 49/50 (98%), Gaps = 0/50 (0%)
 Strand=Plus/Plus

Query  1    GCCCAGCGGTGTGGAATGAGAGCGCTGCAGGTGCGCACCGGGAAGTTCAG  50
            ||||||||||||||||||||||||||||||||||| ||||||||||||||
Sbjct  758  GCCCAGCGGTGTGGAATGAGAGCGCTGCAGGTGCGGACCGGGAAGTTCAG  807


>ref|XM_001083060.2| PREDICTED: Macaca mulatta phospholysine phosphohistidine inorganic 
pyrophosphate phosphatase (LHPP), mRNA
Length=1500

 Score = 82.4 bits (44),  Expect = 1e-13
 Identities = 48/50 (96%), Gaps = 0/50 (0%)
 Strand=Plus/Plus

Query  1    GCCCAGCGGTGTGGAATGAGAGCGCTGCAGGTGCGCACCGGGAAGTTCAG  50
            ||||||||||||||||||||||||||||||||||| || |||||||||||
Sbjct  730  GCCCAGCGGTGTGGAATGAGAGCGCTGCAGGTGCGGACTGGGAAGTTCAG  779


>gb|AC210119.5| Macaca mulatta BAC CH250-198D1 (Children's Hospital Oakland Research 
Institute Rhesus macaque Adult Male BAC Library) complete 
sequence
Length=172090

 Score = 82.4 bits (44),  Expect = 1e-13
 Identities = 48/50 (96%), Gaps = 0/50 (0%)
 Strand=Plus/Minus

Query  1      GCCCAGCGGTGTGGAATGAGAGCGCTGCAGGTGCGCACCGGGAAGTTCAG  50
              ||||||||||||||||||||||||||||||||||| || |||||||||||
Sbjct  53080  GCCCAGCGGTGTGGAATGAGAGCGCTGCAGGTGCGGACTGGGAAGTTCAG  53031


Lambda     K      H
    1.33    0.621     1.12 

Gapped
Lambda     K      H
    1.28    0.460    0.850 

Effective search space used: 874911716973
 
 right 
 >ref|XM_003816282.1| PREDICTED: Pan paniscus fibronectin type III and ankyrin repeat 
domains 1 (FANK1), mRNA
Length=1404

 Score = 93.5 bits (50),  Expect = 6e-17
 Identities = 50/50 (100%), Gaps = 0/50 (0%)
 Strand=Plus/Plus

Query  1    AAATCATGCCACCCTCAAAGCCTCATCCACCTGTCGTGGGCAAAGTGACT  50
            ||||||||||||||||||||||||||||||||||||||||||||||||||
Sbjct  121  AAATCATGCCACCCTCAAAGCCTCATCCACCTGTCGTGGGCAAAGTGACT  170


>ref|XM_003312821.1| PREDICTED: Pan troglodytes fibronectin type III and ankyrin repeat 
domains 1, transcript variant 2 (FANK1), mRNA
Length=1348

 Score = 93.5 bits (50),  Expect = 6e-17
 Identities = 50/50 (100%), Gaps = 0/50 (0%)
 Strand=Plus/Plus

Query  1    AAATCATGCCACCCTCAAAGCCTCATCCACCTGTCGTGGGCAAAGTGACT  50
            ||||||||||||||||||||||||||||||||||||||||||||||||||
Sbjct  90   AAATCATGCCACCCTCAAAGCCTCATCCACCTGTCGTGGGCAAAGTGACT  139


>ref|XM_003312820.1| PREDICTED: Pan troglodytes fibronectin type III and ankyrin repeat 
domains 1, transcript variant 1 (FANK1), mRNA
Length=1292

 Score = 93.5 bits (50),  Expect = 6e-17
 Identities = 50/50 (100%), Gaps = 0/50 (0%)
 Strand=Plus/Plus

Query  1    AAATCATGCCACCCTCAAAGCCTCATCCACCTGTCGTGGGCAAAGTGACT  50
            ||||||||||||||||||||||||||||||||||||||||||||||||||
Sbjct  112  AAATCATGCCACCCTCAAAGCCTCATCCACCTGTCGTGGGCAAAGTGACT  161


>dbj|AK304407.1| Homo sapiens cDNA FLJ52987 complete cds, highly similar to Fibronectin 
type 3 and ankyrin repeat domains protein 1
Length=1283

 Score = 93.5 bits (50),  Expect = 6e-17
 Identities = 50/50 (100%), Gaps = 0/50 (0%)
 Strand=Plus/Plus

Query  1    AAATCATGCCACCCTCAAAGCCTCATCCACCTGTCGTGGGCAAAGTGACT  50
            ||||||||||||||||||||||||||||||||||||||||||||||||||
Sbjct  169  AAATCATGCCACCCTCAAAGCCTCATCCACCTGTCGTGGGCAAAGTGACT  218


>ref|NM_145235.3| Homo sapiens fibronectin type III and ankyrin repeat domains 
1 (FANK1), mRNA
Length=1395

 Score = 93.5 bits (50),  Expect = 6e-17
 Identities = 50/50 (100%), Gaps = 0/50 (0%)
 Strand=Plus/Plus

Query  1    AAATCATGCCACCCTCAAAGCCTCATCCACCTGTCGTGGGCAAAGTGACT  50
            ||||||||||||||||||||||||||||||||||||||||||||||||||
Sbjct  118  AAATCATGCCACCCTCAAAGCCTCATCCACCTGTCGTGGGCAAAGTGACT  167


>gb|AY358154.1| Homo sapiens clone DNA176837 CG3104 hlg (UNQ6504) mRNA, complete 
cds
Length=1373

 Score = 93.5 bits (50),  Expect = 6e-17
 Identities = 50/50 (100%), Gaps = 0/50 (0%)
 Strand=Plus/Plus

Query  1    AAATCATGCCACCCTCAAAGCCTCATCCACCTGTCGTGGGCAAAGTGACT  50
            ||||||||||||||||||||||||||||||||||||||||||||||||||
Sbjct  96   AAATCATGCCACCCTCAAAGCCTCATCCACCTGTCGTGGGCAAAGTGACT  145


>gb|AC063963.11| Homo sapiens chromosome 10 clone RP11-295J3, complete sequence
Length=179104

 Score = 93.5 bits (50),  Expect = 6e-17
 Identities = 50/50 (100%), Gaps = 0/50 (0%)
 Strand=Plus/Plus

Query  1      AAATCATGCCACCCTCAAAGCCTCATCCACCTGTCGTGGGCAAAGTGACT  50
              ||||||||||||||||||||||||||||||||||||||||||||||||||
Sbjct  61132  AAATCATGCCACCCTCAAAGCCTCATCCACCTGTCGTGGGCAAAGTGACT  61181


>emb|CR627249.1| Homo sapiens mRNA; cDNA DKFZp781G1012 (from clone DKFZp781G1012)
Length=8002

 Score = 93.5 bits (50),  Expect = 6e-17
 Identities = 50/50 (100%), Gaps = 0/50 (0%)
 Strand=Plus/Plus

Query  1    AAATCATGCCACCCTCAAAGCCTCATCCACCTGTCGTGGGCAAAGTGACT  50
            ||||||||||||||||||||||||||||||||||||||||||||||||||
Sbjct  102  AAATCATGCCACCCTCAAAGCCTCATCCACCTGTCGTGGGCAAAGTGACT  151


>gb|BC024189.2| Homo sapiens fibronectin type III and ankyrin repeat domains 
1, mRNA (cDNA clone MGC:26734 IMAGE:4826296), complete cds
Length=1395

 Score = 91.6 bits (49),  Expect = 2e-16
 Identities = 49/49 (100%), Gaps = 0/49 (0%)
 Strand=Plus/Plus

Query  2    AATCATGCCACCCTCAAAGCCTCATCCACCTGTCGTGGGCAAAGTGACT  50
            |||||||||||||||||||||||||||||||||||||||||||||||||
Sbjct  119  AATCATGCCACCCTCAAAGCCTCATCCACCTGTCGTGGGCAAAGTGACT  167


>gb|AY251163.1| Homo sapiens HSD13 mRNA, complete cds
Length=1220

 Score = 89.8 bits (48),  Expect = 8e-16
 Identities = 48/48 (100%), Gaps = 0/48 (0%)
 Strand=Plus/Plus

Query  3   ATCATGCCACCCTCAAAGCCTCATCCACCTGTCGTGGGCAAAGTGACT  50
           ||||||||||||||||||||||||||||||||||||||||||||||||
Sbjct  20  ATCATGCCACCCTCAAAGCCTCATCCACCTGTCGTGGGCAAAGTGACT  67


>ref|XM_002821261.2| PREDICTED: Pongo abelii fibronectin type III and ankyrin repeat 
domains 1, transcript variant 1 (FANK1), mRNA
Length=1380

 Score = 87.9 bits (47),  Expect = 3e-15
 Identities = 49/50 (98%), Gaps = 0/50 (0%)
 Strand=Plus/Plus

Query  1    AAATCATGCCACCCTCAAAGCCTCATCCACCTGTCGTGGGCAAAGTGACT  50
            |||||| |||||||||||||||||||||||||||||||||||||||||||
Sbjct  122  AAATCACGCCACCCTCAAAGCCTCATCCACCTGTCGTGGGCAAAGTGACT  171


>ref|XM_002821262.2| PREDICTED: Pongo abelii fibronectin type III and ankyrin repeat 
domains 1, transcript variant 2 (FANK1), mRNA
Length=1302

 Score = 87.9 bits (47),  Expect = 3e-15
 Identities = 49/50 (98%), Gaps = 0/50 (0%)
 Strand=Plus/Plus

Query  1    AAATCATGCCACCCTCAAAGCCTCATCCACCTGTCGTGGGCAAAGTGACT  50
            |||||| |||||||||||||||||||||||||||||||||||||||||||
Sbjct  122  AAATCACGCCACCCTCAAAGCCTCATCCACCTGTCGTGGGCAAAGTGACT  171


>ref|XM_001087857.2| PREDICTED: Macaca mulatta fibronectin type III and ankyrin repeat 
domains 1, transcript variant 6 (FANK1), mRNA
Length=1172

 Score = 87.9 bits (47),  Expect = 3e-15
 Identities = 49/50 (98%), Gaps = 0/50 (0%)
 Strand=Plus/Plus

Query  1    AAATCATGCCACCCTCAAAGCCTCATCCACCTGTCGTGGGCAAAGTGACT  50
            |||||| |||||||||||||||||||||||||||||||||||||||||||
Sbjct  118  AAATCACGCCACCCTCAAAGCCTCATCCACCTGTCGTGGGCAAAGTGACT  167


>ref|XM_001087611.2| PREDICTED: Macaca mulatta fibronectin type III and ankyrin repeat 
domains 1, transcript variant 4 (FANK1), mRNA
Length=1297

 Score = 87.9 bits (47),  Expect = 3e-15
 Identities = 49/50 (98%), Gaps = 0/50 (0%)
 Strand=Plus/Plus

Query  1    AAATCATGCCACCCTCAAAGCCTCATCCACCTGTCGTGGGCAAAGTGACT  50
            |||||| |||||||||||||||||||||||||||||||||||||||||||
Sbjct  118  AAATCACGCCACCCTCAAAGCCTCATCCACCTGTCGTGGGCAAAGTGACT  167


>ref|XM_001086789.2| PREDICTED: Macaca mulatta fibronectin type III and ankyrin repeat 
domains 1, transcript variant 1 (FANK1), mRNA
Length=1353

 Score = 87.9 bits (47),  Expect = 3e-15
 Identities = 49/50 (98%), Gaps = 0/50 (0%)
 Strand=Plus/Plus

Query  1    AAATCATGCCACCCTCAAAGCCTCATCCACCTGTCGTGGGCAAAGTGACT  50
            |||||| |||||||||||||||||||||||||||||||||||||||||||
Sbjct  96   AAATCACGCCACCCTCAAAGCCTCATCCACCTGTCGTGGGCAAAGTGACT  145


>ref|XM_002756706.1| PREDICTED: Callithrix jacchus fibronectin type III and ankyrin 
repeat domains 1 (FANK1), mRNA
Length=1759

 Score = 87.9 bits (47),  Expect = 3e-15
 Identities = 49/50 (98%), Gaps = 0/50 (0%)
 Strand=Plus/Plus

Query  1    AAATCATGCCACCCTCAAAGCCTCATCCACCTGTCGTGGGCAAAGTGACT  50
            ||||||||||| ||||||||||||||||||||||||||||||||||||||
Sbjct  317  AAATCATGCCATCCTCAAAGCCTCATCCACCTGTCGTGGGCAAAGTGACT  366


>dbj|AB179439.1| Macaca fascicularis testis cDNA clone: QtsA-20030, similar to 
human fibronectin type 3 and ankyrin repeat domains 1(FANK1), 
mRNA, RefSeq: NM_145235.2
Length=1201

 Score = 87.9 bits (47),  Expect = 3e-15
 Identities = 49/50 (98%), Gaps = 0/50 (0%)
 Strand=Plus/Plus

Query  1    AAATCATGCCACCCTCAAAGCCTCATCCACCTGTCGTGGGCAAAGTGACT  50
            |||||| |||||||||||||||||||||||||||||||||||||||||||
Sbjct  102  AAATCACGCCACCCTCAAAGCCTCATCCACCTGTCGTGGGCAAAGTGACT  151


>ref|XM_003479614.1| PREDICTED: Cavia porcellus fibronectin type 3 and ankyrin repeat 
domains 1 protein-like (LOC100735426), mRNA
Length=1104

 Score = 76.8 bits (41),  Expect = 7e-12
 Identities = 47/50 (94%), Gaps = 0/50 (0%)
 Strand=Plus/Plus

Query  1    AAATCATGCCACCCTCAAAGCCTCATCCACCTGTCGTGGGCAAAGTGACT  50
            |||||| |||  ||||||||||||||||||||||||||||||||||||||
Sbjct  80   AAATCACGCCGTCCTCAAAGCCTCATCCACCTGTCGTGGGCAAAGTGACT  129


Lambda     K      H
    1.33    0.621     1.12 

Gapped
Lambda     K      H
    1.28    0.460    0.850 

Effective search space used: 874911716973
 
    reads 
 chr10 chr10 126186628 126205755 69M19051N7M                  |||||||||||||||||||||||||||||||||||||||||||||||||||||||||||||||||||||||||||||||||||||||||||||||||||||||||||||||||||||||||||||||||||||||||||||||||||||||||||||||||||||||||||||||||||||||||||||||||||||||||||||||||GCCGTCA
chr10 chr10 126186631 126205758 66M19051N10M                 |||||||||||||||||||||||||||||||||||||||||||||||||||||||||||||||||||||||||||||||||||||||||||||||||||||||||||||||||||||||||||||||||||||||||||||||||||||||||||||||||||||||||||||||||||||||||||||||||||||||||||||||||GCCGTCATGA
chr10 chr10 126186634 126205761 63M19051N13M                 |||||||||||||||||||||||||||||||||||||||||||||||||||||||||||||||||||||||||||||||||||||||||||||||||||||||||||||||||||||||||||||||||||||||||||||||||||||||||||||||||||||||||||||||||||||||||||||||||||||||||||||||||GCCGTCATGATTG
chr10 chr10 126186637 126205764 60M19051N16M                 |||||||||||||||||||||||||||||||||||||||||||||||||||||||||||||||||||||||||||||||||||||||||||||||||||||||||||||||||||||||||||||||||||||||||||||||||||||||||||||||||||||||||||||||||||||||||||||||||||||||||||||||||GCCGTCATGATAGGGG
chr10 chr10 126186640 126205767 57M19051N19M                 |||||||||||||||||||||||||||||||||||||||||||||||||||||||||||||||||||||||||||||||||||||||||||||||||||||||||||||||||||||||||||||||||||||||||||||||||||||||||||||||||||||||||||||||||||||||||||||||||||||||||||||||||GCCGTCATGATTGGGGACG
chr10 chr10 126186644 126205771 53M19051N23M                 |||||||||||||||||||||||||||||||||||||||||||||||||||||||||||||||||||||||||||||||||||||||||||||||||||||||||||||||||||||||||||||||||||||||||||||||||||||||||||||||||||||||||||||||||||||||||||||||||||||||||||||||||GCCGTCATGATTGGGGACGATAT
chr10 chr10 126186647 126205774 50M19051N26M                 |||||||||||||||||||||||||||||||||||||||||||||||||||||||||||||||||||||||||||||||||||||||||||||||||||||||||||||||||||||||||||||||||||||||||||||||||||||||||||||||||||||||||||||||||||||||||||||||||||||||||||||||||GCCGTCATGATTGGGGACGATATCGT
chr10 chr10 126186650 126205777 47M19051N29M                 |||||||||||||||||||||||||||||||||||||||||||||||||||||||||||||||||||||||||||||||||||||||||||||||||||||||||||||||||||||||||||||||||||||||||||||||||||||||||||||||||||||||||||||||||||||||||||||||||||||||||||||||||GCCGTCATGATTGGGGACGATATCGTGGG
chr10 chr10 126186654 126205781 43M19051N33M                 |||||||||||||||||||||||||||||||||||||||||||||||||||||||||||||||||||||||||||||||||||||||||||||||||||||||||||||||||||||||||||||||||||||||||||||||||||||||||||||||||||||||||||||||||||||||||||||||||||||||||||||||||GCCGTCATGATTGGGGACGATATCGTGGGCGAC
chr10 chr10 126186657 126205784 40M19051N36M                 |||||||||||||||||||||||||||||||||||||||||||||||||||||||||||||||||||||||||||||||||||||||||||||||||||||||||||||||||||||||||||||||||||||||||||||||||||||||||||||||||||||||||||||||||||||||||||||||||||||||||||||||||GCCGTCATGATTGGGGACGATATCGTGGGCGACGTG
chr10 chr10 126186660 126205787 37M19051N39M                 |||||||||||||||||||||||||||||||||||||||||||||||||||||||||||||||||||||||||||||||||||||||||||||||||||||||||||||||||||||||||||||||||||||||||||||||||||||||||||||||||||||||||||||||||||||||||||||||||||||||||||||||||GCCGTCATGATTGGGGACGATATCGTGGGCGACGTCGGC
chr10 chr10 126186663 126205790 34M19051N42M                 |||||||||||||||||||||||||||||||||||||||||||||||||||||||||||||||||||||||||||||||||||||||||||||||||||||||||||||||||||||||||||||||||||||||||||||||||||||||||||||||||||||||||||||||||||||||||||||||||||||||||||||||||GCCGTCATGATTGGGGACGATATCGTGGGCGACGTCGGCGGA
chr10 chr10 126186666 126205793 31M19051N45M                 |||||||||||||||||||||||||||||||||||||||||||||||||||||||||||||||||||||||||||||||||||||||||||||||||||||||||||||||||||||||||||||||||||||||||||||||||||||||||||||||||||||||||||||||||||||||||||||||||||||||||||||||||GCCGTCATGATTGGGGACGATATCGTGGGCGACGTCGGCGGTGCC
chr10 chr10 126186669 126205796 28M19051N48M                 |||||||||||||||||||||||||||||||||||||||||||||||||||||||||||||||||||||||||||||||||||||||||||||||||||||||||||||||||||||||||||||||||||||||||||||||||||||||||||||||||||||||||||||||||||||||||||||||||||||||||||||||||GCCGTCATGATTGGGGACGATATCGTGGGCGACGTCGGCGGTGCCCAG
chr10 chr10 126186673 126205800 24M19051N52M                 |||||||||||||||||||||||||||||||||||||||||||||||||||||||||||||||||||||||||||||||||||||||||||||||||||||||||||||||||||||||||||||||||||||||||||||||||||||||||||||||||||||||||||||||||||||||||||||||||||||||||||||||||GCCGTCATGATTGGGGACGATATCGTGGGCGACGTCGGCGGTGCCCAGCGGT
chr10 chr10 126186676 126205803 21M19051N55M                 |||||||||||||||||||||||||||||||||||||||||||||||||||||||||||||||||||||||||||||||||||||||||||||||||||||||||||||||||||||||||||||||||||||||||||||||||||||||||||||||||||||||||||||||||||||||||||||||||||||||||||||||||GCCGTCATGATTGGGGACGATATCGTGGGCGACGTCGGCGGTGCCCAGCGGTGTG
chr10 chr10 126186679 126205806 18M19051N58M                 |||||||||||||||||||||||||||||||||||||||||||||||||||||||||||||||||||||||||||||||||||||||||||||||||||||||||||||||||||||||||||||||||||||||||||||||||||||||||||||||||||||||||||||||||||||||||||||||||||||||||||||||||GCCGTCATGATTGGGGACGATATCGTGGGCGACGTCGGCGGTGCCCAGCGGTGTGGAA
chr10 chr10 126186682 126205809 15M19051N61M                 |||||||||||||||||||||||||||||||||||||||||||||||||||||||||||||||||||||||||||||||||||||||||||||||||||||||||||||||||||||||||||||||||||||||||||||||||||||||||||||||||||||||||||||||||||||||||||||||||||||||||||||||||GCCGTCATGATTGGGGACGATATCGTGGGCGACGTCGGCGGTGCCCAGCGGTGTGGAATGA
chr10 chr10 126186685 126205812 12M19051N64M                 |||||||||||||||||||||||||||||||||||||||||||||||||||||||||||||||||||||||||||||||||||||||||||||||||||||||||||||||||||||||||||||||||||||||||||||||||||||||||||||||||||||||||||||||||||||||||||||||||||||||||||||||||GCCGTCATGATTGGGGACGATATCGTGGGCGACGTCGGCGGTGCCCAGCGGTGTGGAATGAGAG
chr10 chr10 126186688 126205815 9M19051N67M                  |||||||||||||||||||||||||||||||||||||||||||||||||||||||||||||||||||||||||||||||||||||||||||||||||||||||||||||||||||||||||||||||||||||||||||||||||||||||||||||||||||||||||||||||||||||||||||||||||||||||||||||||||GCCGTCATGATTGGGGACGATATCGTGGGCGACGTCGGCGGTGCCCAGCGGTGTGGAATGAGAGCGC
chr10 chr10 126186691 126205818 6M19051N70M                  |||||||||||||||||||||||||||||||||||||||||||||||||||||||||||||||||||||||||||||||||||||||||||||||||||||||||||||||||||||||||||||||||||||||||||||||||||||||||||||||||||||||||||||||||||||||||||||||||||||||||||||||||GCCGTCATGATTGGGGACGATATCGTGGGCGACGTCGGCGGTGCCCAGCGGTGTGGAATGAGAGCGCTGC
chr10 chr10 126186694 126205821 3M19051N73M                  |||||||||||||||||||||||||||||||||||||||||||||||||||||||||||||||||||||||||||||||||||||||||||||||||||||||||||||||||||||||||||||||||||||||||||||||||||||||||||||||||||||||||||||||||||||||||||||||||||||||||||||||||GCCGTCATGATTGGGGACGATATCGTGGGCGACGTCGGCGGTGCCCAGCGGTGTGGAATGAGAGCGCTGCAGG
chr10 chr10 126205571 126205647 76M                                                          TATATTCAAGGTGGGTTGACTTTTAATCAATACGATGACAATATTATCTTGTTTAATTTGTATGATGGTGGTTGTA
chr10 chr10 126205624 126205700 76M                                                                                                               TAATTTGTATGATGGTGGTTGTAAACATCAAATCAAGCCATTTATTATGTCAAAAAAAGATGAAGGAGCCCGGGAA
chr10 chr10 126205749 126205825 76M                                                                                                                                                                                                                                            CCGTCATGATTGGGGACGATATCGTGGGCGACGTCGGCGGTGCCCAGCGGTGTGGAATGAGAGCGCTGCAGGTGCG
chr10 chr10 126205754 126205830 76M                                                                                                                                                                                                                                                 ATGATTGGGGACGATATCGTGGGCGACGTCGGCGGTGCCCAGCGGTGTGGAATGAGAGCGCTGCAGGTGCGCACCG
chr10 chr10 126205757 126205833 76M                                                                                                                                                                                                                                                    ATTGGGGACGATATCGTGGGCGACGTCGGCGGTGCCCAGCGGTGTGGAATGAGAGCGCTGCAGGTGCGCACCGGGG
chr10 chr10 126205760 126205836 76M                                                                                                                                                                                                                                                       GGGGACGATATCGTGGGCGACGTCGGCGGTGCCCAGCGGTGTGGAATGAGAGCGCTGCAGGTGCGCACCGGGAAGT
chr10 chr10 126205763 126205839 76M                                                                                                                                                                                                                                                          GACGATATCGTGGGCGACGTCGGCGGTGCCCAGCGGTGTGGAATGAGAGCGCTGCAGGTGCGCACCGGGAAGATCA
chr10 chr10 126205766 126205842 76M                                                                                                                                                                                                                                                             GATATCGTGGGCGACGTCGGCGGTGCCCAGCGGTGTGGAATGAGAGCGCTGCAGGTGCGCACCGGGAAGTTCAGGC
chr10 chr10 126205769 127668734 71M127668730F5M                                                                                                                                                                                                                                                    ATCGTGGGCGACGTCGGCGGTGCCCAGCGGTGTGGAATGAGAGCGCTGCAGGTGCGCACCGGGAAGTTCAG AAATC
chr10 chr10 126205769 127668734 71M127668730F5M                                                                                                                                                                                                                                                    ATCGTGGGCGACGTCGGCGGAGCCCAGCGGTGTGGAATGAGAGCGCTGCAGGTGCGCACCGGGAAGTTCAG AAATC
chr10 chr10 126205772 127668737 68M127668730F8M                                                                                                                                                                                                                                                       GTGGGCGACGTCGGCGGTGCCCAGCGGTGTGGAATGAGAGCGCTGCAGGTGCGCACCGGGAAGTTCAG AAATCATG
chr10 chr10 126205774 127668739 66M127668730F10M                                                                                                                                                                                                                                                        GGGCGACGTCGGCGGTGCCCAGCGGTGTGGAATGAGAGCGCTGCAGGTGCGCACCGGGAAGTTCAG AAATCATGCC
chr10 chr10 126205775 127668740 65M127668730F11M                                                                                                                                                                                                                                                         GGCGACGTCGGCGGTGCCCAGCGGTGTGGAATGAGAGCGCTGCAGGTGCGCACCGGGAAGTTCAG AAATCATGCCA
chr10 chr10 126205776 127668741 64M127668730F12M                                                                                                                                                                                                                                                          GCGACGTCGGCGGTGCCCAGCGGTGTGGAATGAGAGCGCTGCAGGTGCGCACCGGGAAGTTCAG AAATCATGCCAC
chr10 chr10 126205777 127668742 63M127668730F13M                                                                                                                                                                                                                                                           CGACGTCGGCGGTGCCCAGCGGTGTGGAATGAGAGCGCTGCAGGTGCGCACCGGGAAGTTCAG AAATCATGCCACC
chr10 chr10 126205777 127668742 63M127668730F13M                                                                                                                                                                                                                                                           CGACGTCGGCGGTGCCCAGCGGGGTGGAATGAGAGCGCTGCAGGTGCGCACCGGGAAGTTCAG AAATCATGCCACC
chr10 chr10 126205781 127668746 59M127668730F17M                                                                                                                                                                                                                                                               GTCGGCGGTGCCCAGCGGTGTGGAATGAGAGCGCTGCAGGTGCGCACCGGGAAGTTCAG AAATCATGCCACACTCA
chr10 chr10 126205781 127668746 59M127668730F17M                                                                                                                                                                                                                                                               GTCGGCGGTGCCCAGCGGTGTGGAATGAGAGCGCTGCAGGTGCGCACCGGGAAGTTCAG AAATCATGCCACCCTCA
chr10 chr10 126205783 127668748 57M127668730F19M                                                                                                                                                                                                                                                                 CGGCGGTGCCCAGCGGTGTGGAATGAGAGCGCTGCAGGTGCGCACCGGGAAGTTCAG AAATCATGCCACCCTCAAA
chr10 chr10 126205783 127668748 57M127668730F19M                                                                                                                                                                                                                                                                 CGGCGGTGCCCAGCGGTGTGGAATGAGAGCGCTGCAGGTGCGCACCGGGGAGTTCAG AAATCATGCCACCCTCAAA
chr10 chr10 126205783 127668748 57M127668730F19M                                                                                                                                                                                                                                                                 CGGCGGTGCCCAGCGGTGTGGAATGAGAGCGCTGCAGGTGCGCACCGGGAAGTTCAG AAATCATGCCACCCTCAAA
chr10 chr10 126205783 127668748 57M127668730F19M                                                                                                                                                                                                                                                                 CGGCGGTGCCCAGCGGTGTGGAATGAGAGCGCTGCAGGTGCGCACCGGGAAGTTCAG AAATCATGCCACCCTCAAA
chr10 chr10 126205783 127668748 57M127668730F19M                                                                                                                                                                                                                                                                 CGGCGGTGCCCAGCGGTGTGGAATGAGAGCGCTGCAGGTGCGCACCGGGAAGTTCAG AAATCATGCCACCCTCAAA
chr10 chr10 126205783 127668748 57M127668730F19M                                                                                                                                                                                                                                                                 CGGCGGTGCCCAGCGGTGTGGAATGAGAGCGCTGCAGGTGCGCACCGGGAAGTTCAG AAATCATGCCACCCTCAAA
chr10 chr10 126205783 127668748 57M127668730F19M                                                                                                                                                                                                                                                                 CGGCGGTGCCCAGCGGGGTGGAATGAGAGCGCTGCAGGTGCGCACCGGGGAGTTCAG AAATCATGCCACCCTCAAA
chr10 chr10 126205783 127668748 57M127668730F19M                                                                                                                                                                                                                                                                 CGGCGGTGCCCAGCGGTGTGGAATGAGAGCGCTGCAGGTGCGCACCGGGAAGTTCAG AAATCATGCCACCCTCAAA
chr10 chr10 126205784 127668749 56M127668730F20M                                                                                                                                                                                                                                                                  GGCGGTGCCCAGCGGTGTGGAATGAGAGCGCTGCAGGTGCGCACTGGGAAGTTCAG AAATCATGCCACCCTCAAAG
chr10 chr10 126205788 127668753 52M127668730F24M                                                                                                                                                                                                                                                                      GTGCCCAGCGGTGTGGAATGAGAGCGCTGCAGGTGCGCACCGGGAAGTTCAG AAATCATGCCACCCTCAAAGCCTC
chr10 chr10 126205793 127668758 47M127668730F29M                                                                                                                                                                                                                                                                           CAGCGGTGTGGAATGAGAGCGCTGCAGGTGCGCACCGGGAAGTTCAG AAATCATGCCACCCTCAAAGCCTCATCCA
chr10 chr10 126205795 127668760 45M127668730F31M                                                                                                                                                                                                                                                                             GCGGTGTGGAATGAGAGCGCTGCAGGTGCGCACCGGGAAGTTCAG AAATCATGCCACCCTCAAAGCCTCATCCACC
chr10 chr10 126205800 127668765 40M127668730F36M                                                                                                                                                                                                                                                                                  GTGGAATGAGAGCGCTGCAGGTGCGCACCGGGAAGTTCAG AAATCATGCCACCCTCAAAGCCTCATCCACCTGTCG
chr10 chr10 126205801 127668766 39M127668730F37M                                                                                                                                                                                                                                                                                   TGGAATGAGAGCGCTGCAGGTGCGCACCGGGAAGTTCAG AAATCATGCCACCCTCAAAGCCTCATCCACCTGTCGT
chr10 chr10 126205802 127668767 38M127668730F38M                                                                                                                                                                                                                                                                                    GGAATGAGAGCGCTGCAGGTGCGCACCGGGAAGTTCAG AAATCATGCCACCCTCAAAGCCTCATCCACCTGTCGTG
chr10 chr10 126205804 127668769 36M127668730F40M                                                                                                                                                                                                                                                                                      AATGAGAGCGCTGCAGGTGCGCACCGGGAAGTTCAG AAATCATGCCACCCTCAAAGCCTCATCCACCTGTCGTGGG
chr10 chr10 126205805 127668770 35M127668730F41M                                                                                                                                                                                                                                                                                       ATGAGAGCGCTGCAGGTGCGCACCGGGAAGTTCAG AAATCATGCCACCCTCAAAGCCTCATCCACCTGTCGTGGGC
chr10 chr10 126205806 127668771 34M127668730F42M                                                                                                                                                                                                                                                                                        TGAGAGCGCTGCAGGTGCGCACCGGGAAGTTCAG AAATCATGCCACCCTCAAAGCCTCATCCACCTGTCGTGGGCA
chr10 chr10 126205806 127668771 34M127668730F42M                                                                                                                                                                                                                                                                                        TGAGAGCGCTGCAGGTGCGCACCGGGAAGTTCAG AAATCATGCCACCCTCAAAGCCTCATCCACCTGTCGTGGGCA
chr10 chr10 126205807 127668772 33M127668730F43M                                                                                                                                                                                                                                                                                         GAGAGCGCTGCAGGTGCGCACCGGGAAGTTCAG AAATCATGCCACCCTCAAAGCCTCATCCACCTGTCGTGGGCAA
chr10 chr10 126205809 127668774 31M127668730F45M                                                                                                                                                                                                                                                                                           GAGCGCTGCAGGTGCGCACCGGGAAGTTCAG AAATCATGCCACCCTCAAAGCCTCATCCACCTGTCGTGGGCAAAG
chr10 chr10 126205809 127668774 31M127668730F45M                                                                                                                                                                                                                                                                                           GAGCGCTGCAGGTGCGCACCGGGAAGTTCAG AAATCATGCCACCCTCAAAGCCTCATCCACCTGTCGTGGGCAAAG
chr10 chr10 126205809 127668774 31M127668730F45M                                                                                                                                                                                                                                                                                           GAGCGCTGCAGGTGCGCACCGGGAAGTTCAG AAATCATGCCACCCTCAAAGCCTCATCCACCTGTCGTGGGCAAAG
chr10 chr10 126205809 127668774 31M127668730F45M                                                                                                                                                                                                                                                                                           GAGCGCTGCAGGTGCGCACCGGGAAGTTCAG AAATCATGCCACCCTCAAAGCCTCATCCACCTGTCGTGGGCAAAG
chr10 chr10 126205813 127668778 27M127668730F49M                                                                                                                                                                                                                                                                                               GCTGCAGGTGCGCACCGGGAAGTTCAG AAATCATGCCACCCTCAAAGCCTCATCCACCTGTCGTGGGCAAAGTGAC
chr10 chr10 126205813 127668778 27M127668730F49M                                                                                                                                                                                                                                                                                               GCTGCAGGTGCGCACCGGGAAGTTCAG AAATCATGCCACCCTCAAAGCCTCATCCACCTGTCGTGGGCAAAGTGAC
chr10 chr10 126205813 127668778 27M127668730F49M                                                                                                                                                                                                                                                                                               GCTGCAGGTGCGCACCGGGAAGTTCAG AAATCATGCCACCCTCAAAGCCTCATCCACCTGTCGTGGGCAAAGTGAC
chr10 chr10 126205814 127668779 26M127668730F50M                                                                                                                                                                                                                                                                                                CTGCAGGTGCGCACCGGGAAGTTCAG AAATCATGCCACCCTCAAAGCCTCATCCACCTGTCGTGGGCAAAGTGACT
chr10 chr10 126205818 127668783 22M127668730F54M                                                                                                                                                                                                                                                                                                    AGGTGCGCACCGGGAAGTTCAG AAATCATGCCACCCTCAAAGCCTCATCCACCTGTCGTGGGCAAAGTGACTCATC
chr10 chr10 126205819 127668784 21M127668730F55M                                                                                                                                                                                                                                                                                                     GGTGCGCACCGGGAAGTTCAG AAATCATGCCACCCTCAAAGCCTCATCCACCTGTCGTGGGCAAAGTGACTCATCA
chr10 chr10 126205819 127668784 21M127668730F55M                                                                                                                                                                                                                                                                                                     GGTGCGCACCGGGAAGTTCAG AAATCATGCCACCCTCAAAGCCTCATCCACCTGTCGTGGGCAAAGTGACTCATCA
chr10 chr10 126205819 127668784 21M127668730F55M                                                                                                                                                                                                                                                                                                     GGTGCGCACCGGGAAGTTCAG AAATCATGCCACCCTCAAAGCCTCATCCACCTGTCGTGGGCAAAGTGACTCATCA
chr10 chr10 126205820 127668785 20M127668730F56M                                                                                                                                                                                                                                                                                                      GTGCGCACCGGGAAGTTCAG AAATCCTGCCACCCTCAAAGCCTCATCCACCTGTCGTGGGCAAAGTGACTCATCAC
chr10 chr10 126205820 127668785 20M127668730F56M                                                                                                                                                                                                                                                                                                      GTGCGCACCGGGAAGTTCAG AAATCATGCCACCGTCAAAGCCTCATCCACCTGTCGTGGGCAAAGTGACTCATCAC
chr10 chr10 126205820 127668785 20M127668730F56M                                                                                                                                                                                                                                                                                                      GTGCGCACCGGGAAGTTCAG AAATCATGCCTCCCTCAAAGCCTCATCCACCTGTCGTGGGCAAAGTGACTCATCAC
chr10 chr10 126205822 127668787 18M127668730F58M                                                                                                                                                                                                                                                                                                        GCGCACCGGGAAGTTCAG AAATCATGCCACCCTCAAAGCCTCATCCACCTGTCGTGGGCAAAGTGACTCATCACAG
chr10 chr10 126205824 127668789 16M127668730F60M                                                                                                                                                                                                                                                                                                          GCACCGGGAAGTTCAG AAATCATGCCACCCTCAAAGCCTCATCCACCTGTCGTGGGCAAAGTGACTCATCACAGCA
chr10 chr10 126205825 127668790 15M127668730F61M                                                                                                                                                                                                                                                                                                           CACCGGGAAGTTCAG AAATCATGCCACCCTCAAAGCCTCATCCACCTGTCGTGGGCAAAGTGACTCATCACAGCAT
chr10 chr10 126205827 127668792 13M127668730F63M                                                                                                                                                                                                                                                                                                             CCGGGAAGTTCAG AAATCATGCCACCCTCAAAGCCTCATCCACCTGTCGTGGGCAAAGTGACTCATCACAGCATTG
chr10 chr10 126205827 127668792 13M127668730F63M                                                                                                                                                                                                                                                                                                             CCGGGAAGTTCAG AAATCATGCCACCCTCAAAGCCTCATCCACCTGTCGTGGGCAAAGTGACTCATCACAGCATTG
chr10 chr10 126205827 127668792 13M127668730F63M                                                                                                                                                                                                                                                                                                             CCGGGAAGTTCAG AAATCATGCCACCCTCAAAGCCTCATCCACCTGTCGTGGGCAAAGTGACTCATCACAGCATTG
chr10 chr10 126205828 127668793 12M127668730F64M                                                                                                                                                                                                                                                                                                              CGGGAAGTTCAG AAATCATGCCACCCTCAAAGCCTCATCCACCTGTCGTGGGCAAAGTGACTCATCACAGCATTGA
chr10 chr10 126205828 127668793 12M127668730F64M                                                                                                                                                                                                                                                                                                              CGGGAAGTTCAG AAATCATGCCACCCTCAAAGCCTCATCCACCTGTCGTGGGCAAAGTGACTCATCACAGCATTGA
chr10 chr10 126205828 127668793 12M127668730F64M                                                                                                                                                                                                                                                                                                              CGGGAAGTTCAG AAATCATGCCACCCTCAAAGCCTCATCCACCTGTCGTGGGCAAAGTGACTCATCACAGCATTGA
chr10 chr10 126205828 127668793 12M127668730F64M                                                                                                                                                                                                                                                                                                              CGGGAAGTTCAG AAATCATGCCACCCTCAAAGCCTCATCCACCTGTCGTGGGCAAAGTGACTCATCACAGCATTGA
chr10 chr10 126205829 127668794 11M127668730F65M                                                                                                                                                                                                                                                                                                               GGGAAGTTCAG AAATCATGCCACCCTCAAAGCCTCATCCACCTGTCGTGGGCAAAGTGACTCATCACAGCATTGAA
chr10 chr10 126205829 127668794 11M127668730F65M                                                                                                                                                                                                                                                                                                               GGGAAGTTCAG AAATCATGCCACCCTCAAAGCCTCATCCACCTGTCGTGGGCAAAGTGACTCATCACAGCATTGAA
chr10 chr10 126205829 127668794 11M127668730F65M                                                                                                                                                                                                                                                                                                               GGGAAGTTCAG AAATCATGCCACCCTCAAAGCCTCATCCACCTGTCGTGGGCAAAGTGACTCATCACAGCATTGAA
chr10 chr10 126205830 127668795 10M127668730F66M                                                                                                                                                                                                                                                                                                                GGAAGTTCAG AAATCATGCCACCCTCAAAGCCTCATCCACCTGTCGTGGGCAAAGTGACTCATCACAGCATTGAAT
chr10 chr10 126205830 127668795 10M127668730F66M                                                                                                                                                                                                                                                                                                                GGAAGTTCAG AAATCATGCCACCCTCAAAGCCTCATCCACCTGTCGTGGGCAAAGTGACTCATCACAGCATTGAAT
chr10 chr10 126205830 127668795 10M127668730F66M                                                                                                                                                                                                                                                                                                                GGAAGTTCAG AAATCATGCCACCCTCAAAGCCTCATCCACCTGTCGTGGGCAAAGTGACTCATCACAGCATTGAAT
chr10 chr10 126205830 127668795 10M127668730F66M                                                                                                                                                                                                                                                                                                                GGAAGTTCAG AAATCATGCCACCCTCAAAGCCTCATCCACCTGTCGTGGGCAAAGTGACTCATCACAGCATTGAAT
chr10 chr10 126205830 127668795 10M127668730F66M                                                                                                                                                                                                                                                                                                                GGAAGTTCAG AAATCATGCCACCCTCAAAGCCTCATCCACCTGTCGTGGGCAAAGTGACTCATCACAGCATTGAAT
chr10 chr10 126205831 127668796 9M127668730F67M                                                                                                                                                                                                                                                                                                                  GAAGTTCAG AAATCATGCCACCCTCAAAGCCTCATCCACCTGTCGTGGGCAAAGTGACTCATCACAGCATTGAATT
chr10 chr10 126205834 127668799 6M127668730F70M                                                                                                                                                                                                                                                                                                                     GTTCAG AAATCATGCCACCCTCAAAGCCTCATCCACCTGTCGTGGGCAAAGTGACTCATCACAGCATTGAGTTATA
chr10 chr10 127668724 127668800 76M                                                                                                                                                                                                                                                                                                                                   TTCAGAAATCATGCCACCCTCAAAGCCTCATCCACCTGTCGTGGGCAAAGTGACTCATCACAGCATTGAATTATAC
chr10 chr10 127668727 127668803 76M                                                                                                                                                                                                                                                                                                                                      AGAAATCATGCCACCCTCAAAGCCTCATCCACCTGTCGTGGGCAAAGTGACTCATCACAGCATTGAATTATACTGG
chr10 chr10 127668730 127668806 76M                                                                                                                                                                                                                                                                                                                                         AATCATGCCACCCTCAAAGCCTCATCCACCTGTCGTGGGCAAAGTGACTCATCACAGCATTGAATTATACTGGGAT
chr10 chr10 127668735 127668811 76M                                                                                                                                                                                                                                                                                                                                              TGCCACCCTCAAAGCCTCATCCACCTGTCGTGGGCAAAGTGACTCATCACAGCATTGAATTATACTGGGATCTGGA
chr10 chr10 127668738 127668814 76M                                                                                                                                                                                                                                                                                                                                                 CACCCTCAAAGCCTCATCCACCTGTCGTGGGCAAAGTGACTCATCACAGCATTGAATTATACTGGGATCTGGAAAA
chr10 chr10 127668741 127668817 76M                                                                                                                                                                                                                                                                                                                                                    CCTCAAAGCCTCATCCACCTGTCGTGGGCAAAGTGACTCATCACAGCATTGAATTATACTGGGATCTGGAAAAGAA
chr10 chr10 127668744 127668820 76M                                                                                                                                                                                                                                                                                                                                                       CAAAGCCTCATCCACCTGTCGTGGGCAAAGTGACTCATCACAGCATTGAATTATACTGGGATCTGGAAAAGAAAGC
chr10 chr10 127668747 127668823 76M                                                                                                                                                                                                                                                                                                                                                          AGCCTCATCCACCTGTCGTGGGCAAAGTGACTCATCACAGCATTGAATTATACTGGGATCTGGAAAAGAAAGCCAA
chr10 chr10 127668750 127668826 76M                                                                                                                                                                                                                                                                                                                                                             CTCATCCACCTGTCGTGGGCAAAGTGACTCATCACAGCATTGAATTATACTGGGATCTGGAAAAGAAAGCCAAACG
chr10 chr10 127668753 127668829 76M                                                                                                                                                                                                                                                                                                                                                                ATCCACCTGTCGTGGGCAAAGTGACTCATCACAGCATTGAATTATACTGGGATCTGGAAAAGAAAGCAAAACGCCA
chr10 chr10 127668756 127668832 76M                                                                                                                                                                                                                                                                                                                                                                   CACCTGTCGTGGGCAAAGTGACTCATCACAGCATTGAATTATACTGGGATCTGGAAAAGAAAGCCAAACGCCAAGG
chr10 chr10 127668762 127668838 76M                                                                                                                                                                                                                                                                                                                                                                         TCGTGGGCAAAGTGACTCATCACAGCATTGAATTATACTGGGATCTGGAAAAGAAAGCCAAACGCCAAGGACCTCG
chr10 chr10 127668765 127668841 76M                                                                                                                                                                                                                                                                                                                                                                            TGGGCAAAGTGACTCATCACAGCATTGAATTATACTGGGATCTGGAAAAGAAAGCCAAACGCCAAGGACCTCAAGA
chr10 chr10 127668768 127668844 76M                                                                                                                                                                                                                                                                                                                                                                               GCAAAGTGACTCATCACAGCATTGAATTATACTGGGATCTGGAAAAGAAAGCCAAACGCCAGGGACCTCAAGAGCA
chr10 chr10 127668771 127668847 76M                                                                                                                                                                                                                                                                                                                                                                                  AAGTGACTCATCACAGCATTGAATTATACTGGGATCTGGAAAAGAAAGCCAAACGCCAAGGACCTCAAGAGCAGTG
chr10 chr10 127668775 127668851 76M                                                                                                                                                                                                                                                                                                                                                                                      GACTCATCACAGCATTGAATTATACTGGGATCTGGAAAAGAAAGCCAAACGCCAAGGACCTCAAGAGCAGTGGTTC
chr10 chr10 127668779 127668855 76M                                                                                                                                                                                                                                                                                                                                                                                          CATCACAGCATTGAATTATACTGGGATCTGGAAAAGAAAGCCAAACGCCAAGGACCTCAAGAGCAGTGGTTCAGGT
chr10 chr10 127668782 127668858 76M                                                                                                                                                                                                                                                                                                                                                                                             CACAGCATTGAATTATACTGGGATCTGGAAAAGAAAGCCAAACGCCAAGGACCTCAAGAGCAGTGGTTCAGGTTCT
chr10 chr10 127668785 127668861 76M                                                                                                                                                                                                                                                                                                                                                                                                AGCATTGAATTATACTGGGATCTGGAAAAGAAAGCCAAACGCCAAGGACCTCAAGAGCAGTGGTTCAGGTTCTCGG
chr10 chr10 127668788 127668864 76M                                                                                                                                                                                                                                                                                                                                                                                                   ATTGAATTATACTGGGATCTGGAAAAGAAAGCCAAACGCCAAGGACCTCAAGAGCAGTGGTTCAGGTTCTCGATTG
chr10 chr10 127668791 127668867 76M                                                                                                                                                                                                                                                                                                                                                                                                      GAATTATACTGGGATCTGGAAAAGAAAGCCAAACGCCAAGGACCTCAAGAGCAGTGGTTCAGGTTCTCGATTGAAG
chr10 chr10 127668794 127668870 76M                                                                                                                                                                                                                                                                                                                                                                                                         TTATACTGGGATCTGGAAAAGAAAGCCAAACGCCAAGGACCTCAAGAGCAGTGGTTCAGGTTCTCGATTGAAGAAG
chr10 chr10 127668797 127668873 76M                                                                                                                                                                                                                                                                                                                                                                                                            TACTGGGATCTGGAAAAGAAAGCCAAACGCCAAGGACCTCAAGAGCAGTGGTTCAGGTTCTCGATTGAAGAAGAAG
chr10 chr10 127668800 127668876 76M                                                                                                                                                                                                                                                                                                                                                                                                               TGGGATCTGGAAAAGAAAGCCAAACGCCAAGGACCTCAAGAGCAGTGGTTCAGGTTCTCGATTGAAGAAGAAGACC
chr10 chr10 127668803 127668879 76M                                                                                                                                                                                                                                                                                                                                                                                                                  GATCTGGAAAAGAAAGCCAAACGCCAAGGACCTCAAGAGCAGTGGTTCAGGTTCTCGATTGAAGAAGAAGACCCCA
chr10 chr10 127668806 127668882 76M                                                                                                                                                                                                                                                                                                                                                                                                                     CTGGAAAAGAAAGCCAAACGCCAAGGACCTCAAGAGCAGTGGTTCAGGTTCTCGATTGAAGAAGAAGACCCCAAAA
chr10 chr10 127668809 127668885 76M                                                                                                                                                                                                                                                                                                                                                                                                                        GAAAAGAAAGCCAAACGCCAAGGACCTCAAGAGCAGTGGTTCAGGTTCTCGATTGAAGAAGAAGACCCCAAAATGC
chr10 chr10 127668812 127668888 76M                                                                                                                                                                                                                                                                                                                                                                                                                           AAGAAAGCCAAACGCCAAGGACCTCAAGAGCAGTGGTTCAGGTTCTCGATTGAAGAACAAGACCCCAAAATGCACA
chr10 chr10 127668818 127668894 76M                                                                                                                                                                                                                                                                                                                                                                                                                                 GCCAAACGCCAAGGACCTCAAGAGCAGTGGTTCAGGTTCTCGATTGAAGAAGAAGACCCCAAAATGCACACTTATG
chr10 chr10 127668821 127668897 76M                                                                                                                                                                                                                                                                                                                                                                                                                                    AAACGCCAAGGACCTCAAGAGCAGTGGTTCAGGTTCTCGATTGAAGAAGAAGACCCCAAAATGCACACTTATGGTA
chr10 chr10 127668824 127668900 76M                                                                                                                                                                                                                                                                                                                                                                                                                                       CGCCAAGGACCTCAAGAGCAGTGGTTCAGGTTCTCGATTGAAGAAGAAGACCCCAAAATGCACACTTATGGTATCA
chr10 chr10 127668827 127668903 76M                                                                                                                                                                                                                                                                                                                                                                                                                                          CAAGGACCTCAAGAGCAGTGGTTCAGGTTCTCGATTGAAGAAGAAGACCCCAAAATGCACACTTATGGTATCATTT
chr10 chr10 127668830 127668906 76M                                                                                                                                                                                                                                                                                                                                                                                                                                             GGACCTCAAGAGCAGTGGTTCAGGTTCTCGATTGAAGAAGAAGACCCCAAAATGCACACTTATGGTATCATTTATA
chr10 chr10 127668834 127677122 73M8212N3M                                                                                                                                                                                                                                                                                                                                                                                                                                          CTCAAGAGCAGTGGTTCAGGTTCTCGATTGAAGAAGAAGACCCCAAAATGCACACTTATGGTATCATTTATAC||||||||||||||||||||||||||||||||||||||||||||||||||||||||||||||||||||||||||||||||||||||||||||||||||||||||||||||||||||||||
chr10 chr10 127668837 127677125 70M8212N6M                                                                                                                                                                                                                                                                                                                                                                                                                                             AAGAGCAGTGGTTCAGGTTCTCGATTGAAGAAGAAGACCCCAAAATGCACACTTATGGTATCATTTATAC||||||||||||||||||||||||||||||||||||||||||||||||||||||||||||||||||||||||||||||||||||||||||||||||||||||||||||||||||||||||
chr10 chr10 127668841 127677129 66M8212N10M                                                                                                                                                                                                                                                                                                                                                                                                                                                GCAGTGGTTCAGGTTCTCGATTGAAGAAGAAGACCCCAAAATGCACACTTATGGTATCATTTATAC||||||||||||||||||||||||||||||||||||||||||||||||||||||||||||||||||||||||||||||||||||||||||||||||||||||||||||||||||||||||
chr10 chr10 127668847 127677135 60M8212N16M                                                                                                                                                                                                                                                                                                                                                                                                                                                      GTTCAGGTTCTCGATTGAAGAAGAAGACCCCAAAATGCACACTTATGGTATCATTTATAC||||||||||||||||||||||||||||||||||||||||||||||||||||||||||||||||||||||||||||||||||||||||||||||||||||||||||||||||||||||||
chr10 chr10 127668850 127668926 76M                                                                                                                                                                                                                                                                                                                                                                                                                                                                 CAGGTTCTCGATTGAAGAAGAAGACCCCAAAATGCACACTTATGGTATCATTTATACGTAGGTGCAGTGTTGAATT
chr10 chr10 127668853 127668929 76M                                                                                                                                                                                                                                                                                                                                                                                                                                                                    GTTCTCGATTGAAGAAGAAGACCCCAAAATGCACACTTATGGTATCATTTATACGTAGGTGCAGTGTTGAATTGCT
chr10 chr10 127668856 127677144 51M8212N25M                                                                                                                                                                                                                                                                                                                                                                                                                                                               CTCGATTGAAGAAGAAGACCCCAAAATGCACACTTATGGTATCATTTATAC||||||||||||||||||||||||||||||||||||||||||||||||||||||||||||||||||||||||||||||||||||||||||||||||||||||||||||||||||||||||
chr10 chr10 127668860 127677148 47M8212N29M                                                                                                                                                                                                                                                                                                                                                                                                                                                                   ATTGAAGAAGAAGACCCCAAAATGCACACTTATGGTATCATTTATAC||||||||||||||||||||||||||||||||||||||||||||||||||||||||||||||||||||||||||||||||||||||||||||||||||||||||||||||||||||||||
chr10 chr10 127668864 127668940 76M                                                                                                                                                                                                                                                                                                                                                                                                                                                                               AAGAAGAAGACCCCAAAATGCACACTTATGGTATCATTTATACGTAGGTGCAGTGTTGAATTGCTTGCCACTTTGC
chr10 chr10 127668867 127668943 76M                                                                                                                                                                                                                                                                                                                                                                                                                                                                                  AAGAAGACCCCAAAATGCACACTTATGGTATCATTTATACGTAGGTGCAGTGTTGAATTGCTTGCCACTTTGCCTT
chr10 chr10 127668870 127677158 37M8212N39M                                                                                                                                                                                                                                                                                                                                                                                                                                                                             AAGACCCCAAAATGCACACTTATGGTATCTTTTATAC||||||||||||||||||||||||||||||||||||||||||||||||||||||||||||||||||||||||||||||||||||||||||||||||||||||||||||||||||||||||
chr10 chr10 127668873 127677161 34M8212N42M                                                                                                                                                                                                                                                                                                                                                                                                                                                                                ACCCCAAAATGCACACTTATGGTATCATTTATAC||||||||||||||||||||||||||||||||||||||||||||||||||||||||||||||||||||||||||||||||||||||||||||||||||||||||||||||||||||||||
chr10 chr10 127668876 127677164 31M8212N45M                                                                                                                                                                                                                                                                                                                                                                                                                                                                                   CCAAAATGCACACTTATGGTATCATTTATAC||||||||||||||||||||||||||||||||||||||||||||||||||||||||||||||||||||||||||||||||||||||||||||||||||||||||||||||||||||||||
chr10 chr10 127668879 127668955 76M                                                                                                                                                                                                                                                                                                                                                                                                                                                                                              AAATGCACACTTATGGTATCATTTATACGTAGGTGCAGTGTTGAATTGCTTGCCACTTTGCCTTACTTCCTGATTA
chr10 chr10 127668883 127677171 24M8212N52M                                                                                                                                                                                                                                                                                                                                                                                                                                                                                          GCACACTTATGGTATCATTTATAC||||||||||||||||||||||||||||||||||||||||||||||||||||||||||||||||||||||||||||||||||||||||||||||||||||||||||||||||||||||||
chr10 chr10 127668887 127668963 76M                                                                                                                                                                                                                                                                                                                                                                                                                                                                                                      ACTTATGGTATCATTTATACGTAGGTGCAGTGTTGAATTGCTTGCCACTTTGCCTTACTTCCTGATTAGCTGGAAT 
    14 pairs 
 22:0
12:10
1:26
29:-2
1:36
29:15
24:24
29:24
73:7
21:73
12:125
38:126
1:203
-8979:40 
     TCGA-02-2483-01A chr10-chr10 126214832 127668729 ff 
 
1 14 1 0 42 34
AGACACTCCCTGAAGCGCGTTCCCCAGGAGCTGGAAGGGGGATAACGCAG AAATCATGCCACCCTCAAAGCCTCATCCACCTGTCGTGGGCAAAGTGACT
00000000111111111111111111111111111111111111111111 11111111111111111111111111111111110000000000000000
LHPP intron6(126205840-126301830) FANK1 exon2(127668728-127668906)
 
 blast search - human genome  
 left 
 >ref|NT_030059.13| Homo sapiens chromosome 10 genomic contig, GRCh37.p9 Primary 
Assembly
 gb|GL000099.1| Homo sapiens chromosome 10 genomic contig, GRCh37 reference primary 
assembly
Length=79420533

 Score = 93.5 bits (50),  Expect = 5e-17
 Identities = 50/50 (100%), Gaps = 0/50 (0%)
 Strand=Plus/Plus

Query  1         AGACACTCCCTGAAGCGCGTTCCCCAGGAGCTGGAAGGGGGATAACGCAG  50
                 ||||||||||||||||||||||||||||||||||||||||||||||||||
Sbjct  77019248  AGACACTCCCTGAAGCGCGTTCCCCAGGAGCTGGAAGGGGGATAACGCAG  77019297


>ref|NW_001838010.1| Homo sapiens chromosome 10 genomic contig, alternate assembly 
HuRef SCAF_1103279188229, whole genome shotgun sequence
 gb|DS486216.1| Homo sapiens SCAF_1103279188229 genomic scaffold, whole genome 
shotgun sequence
Length=2644411

 Score = 93.5 bits (50),  Expect = 5e-17
 Identities = 50/50 (100%), Gaps = 0/50 (0%)
 Strand=Plus/Plus

Query  1       AGACACTCCCTGAAGCGCGTTCCCCAGGAGCTGGAAGGGGGATAACGCAG  50
               ||||||||||||||||||||||||||||||||||||||||||||||||||
Sbjct  222425  AGACACTCCCTGAAGCGCGTTCCCCAGGAGCTGGAAGGGGGATAACGCAG  222474


>ref|NC_000010.10| Homo sapiens chromosome 10, GRCh37.p9 Primary Assembly
 gb|CM000672.1| Homo sapiens chromosome 10, GRCh37 primary reference assembly
Length=135534747

 Score = 93.5 bits (50),  Expect = 5e-17
 Identities = 50/50 (100%), Gaps = 0/50 (0%)
 Strand=Plus/Plus

Query  1          AGACACTCCCTGAAGCGCGTTCCCCAGGAGCTGGAAGGGGGATAACGCAG  50
                  ||||||||||||||||||||||||||||||||||||||||||||||||||
Sbjct  126214784  AGACACTCCCTGAAGCGCGTTCCCCAGGAGCTGGAAGGGGGATAACGCAG  126214833


>ref|AC_000142.1| Homo sapiens chromosome 10, alternate assembly HuRef, whole genome 
shotgun sequence
 gb|CM000471.1| Homo sapiens chromosome 10, whole genome shotgun sequence
Length=128985118

 Score = 93.5 bits (50),  Expect = 5e-17
 Identities = 50/50 (100%), Gaps = 0/50 (0%)
 Strand=Plus/Plus

Query  1          AGACACTCCCTGAAGCGCGTTCCCCAGGAGCTGGAAGGGGGATAACGCAG  50
                  ||||||||||||||||||||||||||||||||||||||||||||||||||
Sbjct  119901523  AGACACTCCCTGAAGCGCGTTCCCCAGGAGCTGGAAGGGGGATAACGCAG  119901572


>gb|CM000261.1| Homo sapiens chromosome 10, whole genome shotgun sequence
Length=129189614

 Score = 93.5 bits (50),  Expect = 5e-17
 Identities = 50/50 (100%), Gaps = 0/50 (0%)
 Strand=Plus/Plus

Query  1          AGACACTCCCTGAAGCGCGTTCCCCAGGAGCTGGAAGGGGGATAACGCAG  50
                  ||||||||||||||||||||||||||||||||||||||||||||||||||
Sbjct  119988677  AGACACTCCCTGAAGCGCGTTCCCCAGGAGCTGGAAGGGGGATAACGCAG  119988726


Lambda     K      H
    1.33    0.621     1.12 

Gapped
Lambda     K      H
    1.28    0.460    0.850 

Effective search space used: 681780354108
 
 right 
 >ref|NT_030059.13| Homo sapiens chromosome 10 genomic contig, GRCh37.p9 Primary 
Assembly
 gb|GL000099.1| Homo sapiens chromosome 10 genomic contig, GRCh37 reference primary 
assembly
Length=79420533

 Score = 93.5 bits (50),  Expect = 5e-17
 Identities = 50/50 (100%), Gaps = 0/50 (0%)
 Strand=Plus/Plus

Query  1         AAATCATGCCACCCTCAAAGCCTCATCCACCTGTCGTGGGCAAAGTGACT  50
                 ||||||||||||||||||||||||||||||||||||||||||||||||||
Sbjct  78473194  AAATCATGCCACCCTCAAAGCCTCATCCACCTGTCGTGGGCAAAGTGACT  78473243


>ref|NW_001838010.1| Homo sapiens chromosome 10 genomic contig, alternate assembly 
HuRef SCAF_1103279188229, whole genome shotgun sequence
 gb|DS486216.1| Homo sapiens SCAF_1103279188229 genomic scaffold, whole genome 
shotgun sequence
Length=2644411

 Score = 93.5 bits (50),  Expect = 5e-17
 Identities = 50/50 (100%), Gaps = 0/50 (0%)
 Strand=Plus/Plus

Query  1        AAATCATGCCACCCTCAAAGCCTCATCCACCTGTCGTGGGCAAAGTGACT  50
                ||||||||||||||||||||||||||||||||||||||||||||||||||
Sbjct  1678799  AAATCATGCCACCCTCAAAGCCTCATCCACCTGTCGTGGGCAAAGTGACT  1678848


>ref|NC_000010.10| Homo sapiens chromosome 10, GRCh37.p9 Primary Assembly
 gb|CM000672.1| Homo sapiens chromosome 10, GRCh37 primary reference assembly
Length=135534747

 Score = 93.5 bits (50),  Expect = 5e-17
 Identities = 50/50 (100%), Gaps = 0/50 (0%)
 Strand=Plus/Plus

Query  1          AAATCATGCCACCCTCAAAGCCTCATCCACCTGTCGTGGGCAAAGTGACT  50
                  ||||||||||||||||||||||||||||||||||||||||||||||||||
Sbjct  127668730  AAATCATGCCACCCTCAAAGCCTCATCCACCTGTCGTGGGCAAAGTGACT  127668779


>ref|AC_000142.1| Homo sapiens chromosome 10, alternate assembly HuRef, whole genome 
shotgun sequence
 gb|CM000471.1| Homo sapiens chromosome 10, whole genome shotgun sequence
Length=128985118

 Score = 93.5 bits (50),  Expect = 5e-17
 Identities = 50/50 (100%), Gaps = 0/50 (0%)
 Strand=Plus/Plus

Query  1          AAATCATGCCACCCTCAAAGCCTCATCCACCTGTCGTGGGCAAAGTGACT  50
                  ||||||||||||||||||||||||||||||||||||||||||||||||||
Sbjct  121357897  AAATCATGCCACCCTCAAAGCCTCATCCACCTGTCGTGGGCAAAGTGACT  121357946


>gb|CM000261.1| Homo sapiens chromosome 10, whole genome shotgun sequence
Length=129189614

 Score = 93.5 bits (50),  Expect = 5e-17
 Identities = 50/50 (100%), Gaps = 0/50 (0%)
 Strand=Plus/Plus

Query  1          AAATCATGCCACCCTCAAAGCCTCATCCACCTGTCGTGGGCAAAGTGACT  50
                  ||||||||||||||||||||||||||||||||||||||||||||||||||
Sbjct  121444157  AAATCATGCCACCCTCAAAGCCTCATCCACCTGTCGTGGGCAAAGTGACT  121444206


>gb|CM000500.1| Homo sapiens chromosome 10, whole genome shotgun sequence
Length=128985077

 Score = 87.9 bits (47),  Expect = 2e-15
 Identities = 49/50 (98%), Gaps = 0/50 (0%)
 Strand=Plus/Plus

Query  1          AAATCATGCCACCCTCAAAGCCTCATCCACCTGTCGTGGGCAAAGTGACT  50
                  ||||||||||||||||||||| ||||||||||||||||||||||||||||
Sbjct  121357942  AAATCATGCCACCCTCAAAGCTTCATCCACCTGTCGTGGGCAAAGTGACT  121357991


>gb|DS990854.1| Homo sapiens SCAF_1112675837174 genomic scaffold, whole genome 
shotgun sequence
Length=2644434

 Score = 87.9 bits (47),  Expect = 2e-15
 Identities = 49/50 (98%), Gaps = 0/50 (0%)
 Strand=Plus/Plus

Query  1        AAATCATGCCACCCTCAAAGCCTCATCCACCTGTCGTGGGCAAAGTGACT  50
                ||||||||||||||||||||| ||||||||||||||||||||||||||||
Sbjct  1678839  AAATCATGCCACCCTCAAAGCTTCATCCACCTGTCGTGGGCAAAGTGACT  1678888


Lambda     K      H
    1.33    0.621     1.12 

Gapped
Lambda     K      H
    1.28    0.460    0.850 

Effective search space used: 681780354108
 
 blast search - nt  
 left 
 
 
 right 
 >ref|XM_003816282.1| PREDICTED: Pan paniscus fibronectin type III and ankyrin repeat 
domains 1 (FANK1), mRNA
Length=1404

 Score = 93.5 bits (50),  Expect = 6e-17
 Identities = 50/50 (100%), Gaps = 0/50 (0%)
 Strand=Plus/Plus

Query  1    AAATCATGCCACCCTCAAAGCCTCATCCACCTGTCGTGGGCAAAGTGACT  50
            ||||||||||||||||||||||||||||||||||||||||||||||||||
Sbjct  121  AAATCATGCCACCCTCAAAGCCTCATCCACCTGTCGTGGGCAAAGTGACT  170


>ref|XM_003312821.1| PREDICTED: Pan troglodytes fibronectin type III and ankyrin repeat 
domains 1, transcript variant 2 (FANK1), mRNA
Length=1348

 Score = 93.5 bits (50),  Expect = 6e-17
 Identities = 50/50 (100%), Gaps = 0/50 (0%)
 Strand=Plus/Plus

Query  1    AAATCATGCCACCCTCAAAGCCTCATCCACCTGTCGTGGGCAAAGTGACT  50
            ||||||||||||||||||||||||||||||||||||||||||||||||||
Sbjct  90   AAATCATGCCACCCTCAAAGCCTCATCCACCTGTCGTGGGCAAAGTGACT  139


>ref|XM_003312820.1| PREDICTED: Pan troglodytes fibronectin type III and ankyrin repeat 
domains 1, transcript variant 1 (FANK1), mRNA
Length=1292

 Score = 93.5 bits (50),  Expect = 6e-17
 Identities = 50/50 (100%), Gaps = 0/50 (0%)
 Strand=Plus/Plus

Query  1    AAATCATGCCACCCTCAAAGCCTCATCCACCTGTCGTGGGCAAAGTGACT  50
            ||||||||||||||||||||||||||||||||||||||||||||||||||
Sbjct  112  AAATCATGCCACCCTCAAAGCCTCATCCACCTGTCGTGGGCAAAGTGACT  161


>dbj|AK304407.1| Homo sapiens cDNA FLJ52987 complete cds, highly similar to Fibronectin 
type 3 and ankyrin repeat domains protein 1
Length=1283

 Score = 93.5 bits (50),  Expect = 6e-17
 Identities = 50/50 (100%), Gaps = 0/50 (0%)
 Strand=Plus/Plus

Query  1    AAATCATGCCACCCTCAAAGCCTCATCCACCTGTCGTGGGCAAAGTGACT  50
            ||||||||||||||||||||||||||||||||||||||||||||||||||
Sbjct  169  AAATCATGCCACCCTCAAAGCCTCATCCACCTGTCGTGGGCAAAGTGACT  218


>ref|NM_145235.3| Homo sapiens fibronectin type III and ankyrin repeat domains 
1 (FANK1), mRNA
Length=1395

 Score = 93.5 bits (50),  Expect = 6e-17
 Identities = 50/50 (100%), Gaps = 0/50 (0%)
 Strand=Plus/Plus

Query  1    AAATCATGCCACCCTCAAAGCCTCATCCACCTGTCGTGGGCAAAGTGACT  50
            ||||||||||||||||||||||||||||||||||||||||||||||||||
Sbjct  118  AAATCATGCCACCCTCAAAGCCTCATCCACCTGTCGTGGGCAAAGTGACT  167


>gb|AY358154.1| Homo sapiens clone DNA176837 CG3104 hlg (UNQ6504) mRNA, complete 
cds
Length=1373

 Score = 93.5 bits (50),  Expect = 6e-17
 Identities = 50/50 (100%), Gaps = 0/50 (0%)
 Strand=Plus/Plus

Query  1    AAATCATGCCACCCTCAAAGCCTCATCCACCTGTCGTGGGCAAAGTGACT  50
            ||||||||||||||||||||||||||||||||||||||||||||||||||
Sbjct  96   AAATCATGCCACCCTCAAAGCCTCATCCACCTGTCGTGGGCAAAGTGACT  145


>gb|AC063963.11| Homo sapiens chromosome 10 clone RP11-295J3, complete sequence
Length=179104

 Score = 93.5 bits (50),  Expect = 6e-17
 Identities = 50/50 (100%), Gaps = 0/50 (0%)
 Strand=Plus/Plus

Query  1      AAATCATGCCACCCTCAAAGCCTCATCCACCTGTCGTGGGCAAAGTGACT  50
              ||||||||||||||||||||||||||||||||||||||||||||||||||
Sbjct  61132  AAATCATGCCACCCTCAAAGCCTCATCCACCTGTCGTGGGCAAAGTGACT  61181


>emb|CR627249.1| Homo sapiens mRNA; cDNA DKFZp781G1012 (from clone DKFZp781G1012)
Length=8002

 Score = 93.5 bits (50),  Expect = 6e-17
 Identities = 50/50 (100%), Gaps = 0/50 (0%)
 Strand=Plus/Plus

Query  1    AAATCATGCCACCCTCAAAGCCTCATCCACCTGTCGTGGGCAAAGTGACT  50
            ||||||||||||||||||||||||||||||||||||||||||||||||||
Sbjct  102  AAATCATGCCACCCTCAAAGCCTCATCCACCTGTCGTGGGCAAAGTGACT  151


>gb|BC024189.2| Homo sapiens fibronectin type III and ankyrin repeat domains 
1, mRNA (cDNA clone MGC:26734 IMAGE:4826296), complete cds
Length=1395

 Score = 91.6 bits (49),  Expect = 2e-16
 Identities = 49/49 (100%), Gaps = 0/49 (0%)
 Strand=Plus/Plus

Query  2    AATCATGCCACCCTCAAAGCCTCATCCACCTGTCGTGGGCAAAGTGACT  50
            |||||||||||||||||||||||||||||||||||||||||||||||||
Sbjct  119  AATCATGCCACCCTCAAAGCCTCATCCACCTGTCGTGGGCAAAGTGACT  167


>gb|AY251163.1| Homo sapiens HSD13 mRNA, complete cds
Length=1220

 Score = 89.8 bits (48),  Expect = 8e-16
 Identities = 48/48 (100%), Gaps = 0/48 (0%)
 Strand=Plus/Plus

Query  3   ATCATGCCACCCTCAAAGCCTCATCCACCTGTCGTGGGCAAAGTGACT  50
           ||||||||||||||||||||||||||||||||||||||||||||||||
Sbjct  20  ATCATGCCACCCTCAAAGCCTCATCCACCTGTCGTGGGCAAAGTGACT  67


>ref|XM_002821261.2| PREDICTED: Pongo abelii fibronectin type III and ankyrin repeat 
domains 1, transcript variant 1 (FANK1), mRNA
Length=1380

 Score = 87.9 bits (47),  Expect = 3e-15
 Identities = 49/50 (98%), Gaps = 0/50 (0%)
 Strand=Plus/Plus

Query  1    AAATCATGCCACCCTCAAAGCCTCATCCACCTGTCGTGGGCAAAGTGACT  50
            |||||| |||||||||||||||||||||||||||||||||||||||||||
Sbjct  122  AAATCACGCCACCCTCAAAGCCTCATCCACCTGTCGTGGGCAAAGTGACT  171


>ref|XM_002821262.2| PREDICTED: Pongo abelii fibronectin type III and ankyrin repeat 
domains 1, transcript variant 2 (FANK1), mRNA
Length=1302

 Score = 87.9 bits (47),  Expect = 3e-15
 Identities = 49/50 (98%), Gaps = 0/50 (0%)
 Strand=Plus/Plus

Query  1    AAATCATGCCACCCTCAAAGCCTCATCCACCTGTCGTGGGCAAAGTGACT  50
            |||||| |||||||||||||||||||||||||||||||||||||||||||
Sbjct  122  AAATCACGCCACCCTCAAAGCCTCATCCACCTGTCGTGGGCAAAGTGACT  171


>ref|XM_001087857.2| PREDICTED: Macaca mulatta fibronectin type III and ankyrin repeat 
domains 1, transcript variant 6 (FANK1), mRNA
Length=1172

 Score = 87.9 bits (47),  Expect = 3e-15
 Identities = 49/50 (98%), Gaps = 0/50 (0%)
 Strand=Plus/Plus

Query  1    AAATCATGCCACCCTCAAAGCCTCATCCACCTGTCGTGGGCAAAGTGACT  50
            |||||| |||||||||||||||||||||||||||||||||||||||||||
Sbjct  118  AAATCACGCCACCCTCAAAGCCTCATCCACCTGTCGTGGGCAAAGTGACT  167


>ref|XM_001087611.2| PREDICTED: Macaca mulatta fibronectin type III and ankyrin repeat 
domains 1, transcript variant 4 (FANK1), mRNA
Length=1297

 Score = 87.9 bits (47),  Expect = 3e-15
 Identities = 49/50 (98%), Gaps = 0/50 (0%)
 Strand=Plus/Plus

Query  1    AAATCATGCCACCCTCAAAGCCTCATCCACCTGTCGTGGGCAAAGTGACT  50
            |||||| |||||||||||||||||||||||||||||||||||||||||||
Sbjct  118  AAATCACGCCACCCTCAAAGCCTCATCCACCTGTCGTGGGCAAAGTGACT  167


>ref|XM_001086789.2| PREDICTED: Macaca mulatta fibronectin type III and ankyrin repeat 
domains 1, transcript variant 1 (FANK1), mRNA
Length=1353

 Score = 87.9 bits (47),  Expect = 3e-15
 Identities = 49/50 (98%), Gaps = 0/50 (0%)
 Strand=Plus/Plus

Query  1    AAATCATGCCACCCTCAAAGCCTCATCCACCTGTCGTGGGCAAAGTGACT  50
            |||||| |||||||||||||||||||||||||||||||||||||||||||
Sbjct  96   AAATCACGCCACCCTCAAAGCCTCATCCACCTGTCGTGGGCAAAGTGACT  145


>ref|XM_002756706.1| PREDICTED: Callithrix jacchus fibronectin type III and ankyrin 
repeat domains 1 (FANK1), mRNA
Length=1759

 Score = 87.9 bits (47),  Expect = 3e-15
 Identities = 49/50 (98%), Gaps = 0/50 (0%)
 Strand=Plus/Plus

Query  1    AAATCATGCCACCCTCAAAGCCTCATCCACCTGTCGTGGGCAAAGTGACT  50
            ||||||||||| ||||||||||||||||||||||||||||||||||||||
Sbjct  317  AAATCATGCCATCCTCAAAGCCTCATCCACCTGTCGTGGGCAAAGTGACT  366


>dbj|AB179439.1| Macaca fascicularis testis cDNA clone: QtsA-20030, similar to 
human fibronectin type 3 and ankyrin repeat domains 1(FANK1), 
mRNA, RefSeq: NM_145235.2
Length=1201

 Score = 87.9 bits (47),  Expect = 3e-15
 Identities = 49/50 (98%), Gaps = 0/50 (0%)
 Strand=Plus/Plus

Query  1    AAATCATGCCACCCTCAAAGCCTCATCCACCTGTCGTGGGCAAAGTGACT  50
            |||||| |||||||||||||||||||||||||||||||||||||||||||
Sbjct  102  AAATCACGCCACCCTCAAAGCCTCATCCACCTGTCGTGGGCAAAGTGACT  151


>ref|XM_003479614.1| PREDICTED: Cavia porcellus fibronectin type 3 and ankyrin repeat 
domains 1 protein-like (LOC100735426), mRNA
Length=1104

 Score = 76.8 bits (41),  Expect = 7e-12
 Identities = 47/50 (94%), Gaps = 0/50 (0%)
 Strand=Plus/Plus

Query  1    AAATCATGCCACCCTCAAAGCCTCATCCACCTGTCGTGGGCAAAGTGACT  50
            |||||| |||  ||||||||||||||||||||||||||||||||||||||
Sbjct  80   AAATCACGCCGTCCTCAAAGCCTCATCCACCTGTCGTGGGCAAAGTGACT  129


Lambda     K      H
    1.33    0.621     1.12 

Gapped
Lambda     K      H
    1.28    0.460    0.850 

Effective search space used: 874911716973
 
    reads 
 chr10 chr10 126205770 126214724 70M8878N6M                   ||||||||||||||||||||||||||||||||||||||||||||||||||||||||||||||||||||||||||||||||||||||||||||||||||||||||||||||||||||||||||||||||||||||||||||||||||||||||||||||||||||||||||||||||||||||||||AGCCGC
chr10 chr10 126205809 126214763 31M8878N45M                  ||||||||||||||||||||||||||||||||||||||||||||||||||||||||||||||||||||||||||||||||||||||||||||||||||||||||||||||||||||||||||||||||||||||||||||||||||||||||||||||||||||||||||||||||||||||||||AGCCGCTCAACACGTGTTGCAAGGCACGTGCTCTGGACCTGGTGC
chr10 chr10 126214742 126214818 76M                                                                                                                                                                                                                                            CACGTGCTCTGGACCTGGTGCTCACACGTGCTCTTCTCCCAAGACACTCCCTGAAGCGCGTTCCCCAGGAGCTGGG
chr10 chr10 126214791 127668763 42M127668730F34M                                                                                                                                                                                                                                                                                CCTGAAGCGCGTTCCCCAGGAGCTGGAAGGGGGATAACGCAG AAATCATGCCACCCTCAAAGCCTCATCCACCTGT
chr10 chr10 127668724 127668800 76M                                                                                                                                                                                                                                                                                                                                   TTCAGAAATCATGCCACCCTCAAAGCCTCATCCACCTGTCGTGGGCAAAGTGACTCATCACAGCATTGAATTATAC
chr10 chr10 127668727 127668803 76M                                                                                                                                                                                                                                                                                                                                      AGAAATCATGCCACCCTCAAAGCCTCATCCACCTGTCGTGGGCAAAGTGACTCATCACAGCATTGAATTATACTGG
chr10 chr10 127668730 127668806 76M                                                                                                                                                                                                                                                                                                                                         AATCATGCCACCCTCAAAGCCTCATCCACCTGTCGTGGGCAAAGTGACTCATCACAGCATTGAATTATACTGGGAT
chr10 chr10 127668735 127668811 76M                                                                                                                                                                                                                                                                                                                                              TGCCACCCTCAAAGCCTCATCCACCTGTCGTGGGCAAAGTGACTCATCACAGCATTGAATTATACTGGGATCTGGA
chr10 chr10 127668738 127668814 76M                                                                                                                                                                                                                                                                                                                                                 CACCCTCAAAGCCTCATCCACCTGTCGTGGGCAAAGTGACTCATCACAGCATTGAATTATACTGGGATCTGGAAAA
chr10 chr10 127668741 127668817 76M                                                                                                                                                                                                                                                                                                                                                    CCTCAAAGCCTCATCCACCTGTCGTGGGCAAAGTGACTCATCACAGCATTGAATTATACTGGGATCTGGAAAAGAA
chr10 chr10 127668744 127668820 76M                                                                                                                                                                                                                                                                                                                                                       CAAAGCCTCATCCACCTGTCGTGGGCAAAGTGACTCATCACAGCATTGAATTATACTGGGATCTGGAAAAGAAAGC
chr10 chr10 127668747 127668823 76M                                                                                                                                                                                                                                                                                                                                                          AGCCTCATCCACCTGTCGTGGGCAAAGTGACTCATCACAGCATTGAATTATACTGGGATCTGGAAAAGAAAGCCAA
chr10 chr10 127668750 127668826 76M                                                                                                                                                                                                                                                                                                                                                             CTCATCCACCTGTCGTGGGCAAAGTGACTCATCACAGCATTGAATTATACTGGGATCTGGAAAAGAAAGCCAAACG
chr10 chr10 127668753 127668829 76M                                                                                                                                                                                                                                                                                                                                                                ATCCACCTGTCGTGGGCAAAGTGACTCATCACAGCATTGAATTATACTGGGATCTGGAAAAGAAAGCAAAACGCCA
chr10 chr10 127668756 127668832 76M                                                                                                                                                                                                                                                                                                                                                                   CACCTGTCGTGGGCAAAGTGACTCATCACAGCATTGAATTATACTGGGATCTGGAAAAGAAAGCCAAACGCCAAGG
chr10 chr10 127668762 127668838 76M                                                                                                                                                                                                                                                                                                                                                                         TCGTGGGCAAAGTGACTCATCACAGCATTGAATTATACTGGGATCTGGAAAAGAAAGCCAAACGCCAAGGACCTCG
chr10 chr10 127668765 127668841 76M                                                                                                                                                                                                                                                                                                                                                                            TGGGCAAAGTGACTCATCACAGCATTGAATTATACTGGGATCTGGAAAAGAAAGCCAAACGCCAAGGACCTCAAGA
chr10 chr10 127668768 127668844 76M                                                                                                                                                                                                                                                                                                                                                                               GCAAAGTGACTCATCACAGCATTGAATTATACTGGGATCTGGAAAAGAAAGCCAAACGCCAGGGACCTCAAGAGCA
chr10 chr10 127668771 127668847 76M                                                                                                                                                                                                                                                                                                                                                                                  AAGTGACTCATCACAGCATTGAATTATACTGGGATCTGGAAAAGAAAGCCAAACGCCAAGGACCTCAAGAGCAGTG
chr10 chr10 127668775 127668851 76M                                                                                                                                                                                                                                                                                                                                                                                      GACTCATCACAGCATTGAATTATACTGGGATCTGGAAAAGAAAGCCAAACGCCAAGGACCTCAAGAGCAGTGGTTC
chr10 chr10 127668779 127668855 76M                                                                                                                                                                                                                                                                                                                                                                                          CATCACAGCATTGAATTATACTGGGATCTGGAAAAGAAAGCCAAACGCCAAGGACCTCAAGAGCAGTGGTTCAGGT
chr10 chr10 127668782 127668858 76M                                                                                                                                                                                                                                                                                                                                                                                             CACAGCATTGAATTATACTGGGATCTGGAAAAGAAAGCCAAACGCCAAGGACCTCAAGAGCAGTGGTTCAGGTTCT
chr10 chr10 127668785 127668861 76M                                                                                                                                                                                                                                                                                                                                                                                                AGCATTGAATTATACTGGGATCTGGAAAAGAAAGCCAAACGCCAAGGACCTCAAGAGCAGTGGTTCAGGTTCTCGG
chr10 chr10 127668788 127668864 76M                                                                                                                                                                                                                                                                                                                                                                                                   ATTGAATTATACTGGGATCTGGAAAAGAAAGCCAAACGCCAAGGACCTCAAGAGCAGTGGTTCAGGTTCTCGATTG
chr10 chr10 127668791 127668867 76M                                                                                                                                                                                                                                                                                                                                                                                                      GAATTATACTGGGATCTGGAAAAGAAAGCCAAACGCCAAGGACCTCAAGAGCAGTGGTTCAGGTTCTCGATTGAAG
chr10 chr10 127668794 127668870 76M                                                                                                                                                                                                                                                                                                                                                                                                         TTATACTGGGATCTGGAAAAGAAAGCCAAACGCCAAGGACCTCAAGAGCAGTGGTTCAGGTTCTCGATTGAAGAAG
chr10 chr10 127668797 127668873 76M                                                                                                                                                                                                                                                                                                                                                                                                            TACTGGGATCTGGAAAAGAAAGCCAAACGCCAAGGACCTCAAGAGCAGTGGTTCAGGTTCTCGATTGAAGAAGAAG
chr10 chr10 127668800 127668876 76M                                                                                                                                                                                                                                                                                                                                                                                                               TGGGATCTGGAAAAGAAAGCCAAACGCCAAGGACCTCAAGAGCAGTGGTTCAGGTTCTCGATTGAAGAAGAAGACC
chr10 chr10 127668803 127668879 76M                                                                                                                                                                                                                                                                                                                                                                                                                  GATCTGGAAAAGAAAGCCAAACGCCAAGGACCTCAAGAGCAGTGGTTCAGGTTCTCGATTGAAGAAGAAGACCCCA
chr10 chr10 127668806 127668882 76M                                                                                                                                                                                                                                                                                                                                                                                                                     CTGGAAAAGAAAGCCAAACGCCAAGGACCTCAAGAGCAGTGGTTCAGGTTCTCGATTGAAGAAGAAGACCCCAAAA
chr10 chr10 127668809 127668885 76M                                                                                                                                                                                                                                                                                                                                                                                                                        GAAAAGAAAGCCAAACGCCAAGGACCTCAAGAGCAGTGGTTCAGGTTCTCGATTGAAGAAGAAGACCCCAAAATGC
chr10 chr10 127668812 127668888 76M                                                                                                                                                                                                                                                                                                                                                                                                                           AAGAAAGCCAAACGCCAAGGACCTCAAGAGCAGTGGTTCAGGTTCTCGATTGAAGAACAAGACCCCAAAATGCACA
chr10 chr10 127668818 127668894 76M                                                                                                                                                                                                                                                                                                                                                                                                                                 GCCAAACGCCAAGGACCTCAAGAGCAGTGGTTCAGGTTCTCGATTGAAGAAGAAGACCCCAAAATGCACACTTATG
chr10 chr10 127668821 127668897 76M                                                                                                                                                                                                                                                                                                                                                                                                                                    AAACGCCAAGGACCTCAAGAGCAGTGGTTCAGGTTCTCGATTGAAGAAGAAGACCCCAAAATGCACACTTATGGTA
chr10 chr10 127668824 127668900 76M                                                                                                                                                                                                                                                                                                                                                                                                                                       CGCCAAGGACCTCAAGAGCAGTGGTTCAGGTTCTCGATTGAAGAAGAAGACCCCAAAATGCACACTTATGGTATCA
chr10 chr10 127668827 127668903 76M                                                                                                                                                                                                                                                                                                                                                                                                                                          CAAGGACCTCAAGAGCAGTGGTTCAGGTTCTCGATTGAAGAAGAAGACCCCAAAATGCACACTTATGGTATCATTT
chr10 chr10 127668830 127668906 76M                                                                                                                                                                                                                                                                                                                                                                                                                                             GGACCTCAAGAGCAGTGGTTCAGGTTCTCGATTGAAGAAGAAGACCCCAAAATGCACACTTATGGTATCATTTATA
chr10 chr10 127668834 127677122 73M8212N3M                                                                                                                                                                                                                                                                                                                                                                                                                                          CTCAAGAGCAGTGGTTCAGGTTCTCGATTGAAGAAGAAGACCCCAAAATGCACACTTATGGTATCATTTATAC||||||||||||||||||||||||||||||||||||||||||||||||||||||||||||||||||||||||||||||||||||||||||||||||||||||||||||||||||||||||
chr10 chr10 127668837 127677125 70M8212N6M                                                                                                                                                                                                                                                                                                                                                                                                                                             AAGAGCAGTGGTTCAGGTTCTCGATTGAAGAAGAAGACCCCAAAATGCACACTTATGGTATCATTTATAC||||||||||||||||||||||||||||||||||||||||||||||||||||||||||||||||||||||||||||||||||||||||||||||||||||||||||||||||||||||||
chr10 chr10 127668841 127677129 66M8212N10M                                                                                                                                                                                                                                                                                                                                                                                                                                                GCAGTGGTTCAGGTTCTCGATTGAAGAAGAAGACCCCAAAATGCACACTTATGGTATCATTTATAC||||||||||||||||||||||||||||||||||||||||||||||||||||||||||||||||||||||||||||||||||||||||||||||||||||||||||||||||||||||||
chr10 chr10 127668847 127677135 60M8212N16M                                                                                                                                                                                                                                                                                                                                                                                                                                                      GTTCAGGTTCTCGATTGAAGAAGAAGACCCCAAAATGCACACTTATGGTATCATTTATAC||||||||||||||||||||||||||||||||||||||||||||||||||||||||||||||||||||||||||||||||||||||||||||||||||||||||||||||||||||||||
chr10 chr10 127668850 127668926 76M                                                                                                                                                                                                                                                                                                                                                                                                                                                                 CAGGTTCTCGATTGAAGAAGAAGACCCCAAAATGCACACTTATGGTATCATTTATACGTAGGTGCAGTGTTGAATT
chr10 chr10 127668853 127668929 76M                                                                                                                                                                                                                                                                                                                                                                                                                                                                    GTTCTCGATTGAAGAAGAAGACCCCAAAATGCACACTTATGGTATCATTTATACGTAGGTGCAGTGTTGAATTGCT
chr10 chr10 127668856 127677144 51M8212N25M                                                                                                                                                                                                                                                                                                                                                                                                                                                               CTCGATTGAAGAAGAAGACCCCAAAATGCACACTTATGGTATCATTTATAC||||||||||||||||||||||||||||||||||||||||||||||||||||||||||||||||||||||||||||||||||||||||||||||||||||||||||||||||||||||||
chr10 chr10 127668860 127677148 47M8212N29M                                                                                                                                                                                                                                                                                                                                                                                                                                                                   ATTGAAGAAGAAGACCCCAAAATGCACACTTATGGTATCATTTATAC||||||||||||||||||||||||||||||||||||||||||||||||||||||||||||||||||||||||||||||||||||||||||||||||||||||||||||||||||||||||
chr10 chr10 127668864 127668940 76M                                                                                                                                                                                                                                                                                                                                                                                                                                                                               AAGAAGAAGACCCCAAAATGCACACTTATGGTATCATTTATACGTAGGTGCAGTGTTGAATTGCTTGCCACTTTGC
chr10 chr10 127668867 127668943 76M                                                                                                                                                                                                                                                                                                                                                                                                                                                                                  AAGAAGACCCCAAAATGCACACTTATGGTATCATTTATACGTAGGTGCAGTGTTGAATTGCTTGCCACTTTGCCTT
chr10 chr10 127668870 127677158 37M8212N39M                                                                                                                                                                                                                                                                                                                                                                                                                                                                             AAGACCCCAAAATGCACACTTATGGTATCTTTTATAC||||||||||||||||||||||||||||||||||||||||||||||||||||||||||||||||||||||||||||||||||||||||||||||||||||||||||||||||||||||||
chr10 chr10 127668873 127677161 34M8212N42M                                                                                                                                                                                                                                                                                                                                                                                                                                                                                ACCCCAAAATGCACACTTATGGTATCATTTATAC||||||||||||||||||||||||||||||||||||||||||||||||||||||||||||||||||||||||||||||||||||||||||||||||||||||||||||||||||||||||
chr10 chr10 127668876 127677164 31M8212N45M                                                                                                                                                                                                                                                                                                                                                                                                                                                                                   CCAAAATGCACACTTATGGTATCATTTATAC||||||||||||||||||||||||||||||||||||||||||||||||||||||||||||||||||||||||||||||||||||||||||||||||||||||||||||||||||||||||
chr10 chr10 127668879 127668955 76M                                                                                                                                                                                                                                                                                                                                                                                                                                                                                              AAATGCACACTTATGGTATCATTTATACGTAGGTGCAGTGTTGAATTGCTTGCCACTTTGCCTTACTTCCTGATTA
chr10 chr10 127668883 127677171 24M8212N52M                                                                                                                                                                                                                                                                                                                                                                                                                                                                                          GCACACTTATGGTATCATTTATAC||||||||||||||||||||||||||||||||||||||||||||||||||||||||||||||||||||||||||||||||||||||||||||||||||||||||||||||||||||||||
chr10 chr10 127668887 127668963 76M                                                                                                                                                                                                                                                                                                                                                                                                                                                                                                      ACTTATGGTATCATTTATACGTAGGTGCAGTGTTGAATTGCTTGCCACTTTGCCTTACTTCCTGATTAGCTGGAAT 
    14 pairs 
 14:40
9015:0
9005:10
8994:26
9022:-2
8994:36
9022:15
9017:24
9022:24
9066:7
9014:73
9005:125
9031:126
8994:203 
     TCGA-02-2483-01A chr10-chr10 126234757 127668729 ff 
 
27 22 20 16 56 55
GCCCCAGACCCAAGAATCGCCCGCCTCTCCAAGACCATCCCTGGCTGCTG AAATCATGCCACCCTCAAAGCCTCATCCACCTGTCGTGGGCAAAGTGACT
67999999999999999999999999999999999999999999999999 99999999999999999999999999999999999999999999988766
LHPP intron6(126205840-126301830) FANK1 exon2(127668728-127668906)
 
 blast search - human genome  
 left 
 >ref|NT_030059.13| Homo sapiens chromosome 10 genomic contig, GRCh37.p9 Primary 
Assembly
 gb|GL000099.1| Homo sapiens chromosome 10 genomic contig, GRCh37 reference primary 
assembly
Length=79420533

 Score = 93.5 bits (50),  Expect = 5e-17
 Identities = 50/50 (100%), Gaps = 0/50 (0%)
 Strand=Plus/Plus

Query  1         GCCCCAGACCCAAGAATCGCCCGCCTCTCCAAGACCATCCCTGGCTGCTG  50
                 ||||||||||||||||||||||||||||||||||||||||||||||||||
Sbjct  77039173  GCCCCAGACCCAAGAATCGCCCGCCTCTCCAAGACCATCCCTGGCTGCTG  77039222


>ref|NC_000010.10| Homo sapiens chromosome 10, GRCh37.p9 Primary Assembly
 gb|CM000672.1| Homo sapiens chromosome 10, GRCh37 primary reference assembly
Length=135534747

 Score = 93.5 bits (50),  Expect = 5e-17
 Identities = 50/50 (100%), Gaps = 0/50 (0%)
 Strand=Plus/Plus

Query  1          GCCCCAGACCCAAGAATCGCCCGCCTCTCCAAGACCATCCCTGGCTGCTG  50
                  ||||||||||||||||||||||||||||||||||||||||||||||||||
Sbjct  126234709  GCCCCAGACCCAAGAATCGCCCGCCTCTCCAAGACCATCCCTGGCTGCTG  126234758


>gb|CM000261.1| Homo sapiens chromosome 10, whole genome shotgun sequence
Length=129189614

 Score = 93.5 bits (50),  Expect = 5e-17
 Identities = 50/50 (100%), Gaps = 0/50 (0%)
 Strand=Plus/Plus

Query  1          GCCCCAGACCCAAGAATCGCCCGCCTCTCCAAGACCATCCCTGGCTGCTG  50
                  ||||||||||||||||||||||||||||||||||||||||||||||||||
Sbjct  120008611  GCCCCAGACCCAAGAATCGCCCGCCTCTCCAAGACCATCCCTGGCTGCTG  120008660


>gb|GL583189.1| Homo sapiens unplaced genomic scaffold scaffold_209, whole genome 
shotgun sequence
Length=3658509

 Score = 89.8 bits (48),  Expect = 7e-16
 Identities = 49/50 (98%), Gaps = 0/50 (0%)
 Strand=Plus/Minus

Query  1        GCCCCAGACCCAAGAATCGCCCGCCTCTCCAAGACCATCCCTGGCTGCTG  50
                |||||||||||||||||||||||||||||| |||||||||||||||||||
Sbjct  2379346  GCCCCAGACCCAAGAATCGCCCGCCTCTCCRAGACCATCCCTGGCTGCTG  2379297


>gb|GL297060.1| Homo sapiens unplaced genomic scaffold scaffold3403, whole genome 
shotgun sequence
Length=1179847

 Score = 87.9 bits (47),  Expect = 2e-15
 Identities = 49/50 (98%), Gaps = 0/50 (0%)
 Strand=Plus/Minus

Query  1       GCCCCAGACCCAAGAATCGCCCGCCTCTCCAAGACCATCCCTGGCTGCTG  50
               |||||||||||||||||||||||||||||| |||||||||||||||||||
Sbjct  777771  GCCCCAGACCCAAGAATCGCCCGCCTCTCCGAGACCATCCCTGGCTGCTG  777722


>ref|NW_001838010.1| Homo sapiens chromosome 10 genomic contig, alternate assembly 
HuRef SCAF_1103279188229, whole genome shotgun sequence
 gb|DS486216.1| Homo sapiens SCAF_1103279188229 genomic scaffold, whole genome 
shotgun sequence
Length=2644411

 Score = 87.9 bits (47),  Expect = 2e-15
 Identities = 49/50 (98%), Gaps = 0/50 (0%)
 Strand=Plus/Plus

Query  1       GCCCCAGACCCAAGAATCGCCCGCCTCTCCAAGACCATCCCTGGCTGCTG  50
               |||||||||||||||||||||||||||||| |||||||||||||||||||
Sbjct  242350  GCCCCAGACCCAAGAATCGCCCGCCTCTCCGAGACCATCCCTGGCTGCTG  242399


>ref|AC_000142.1| Homo sapiens chromosome 10, alternate assembly HuRef, whole genome 
shotgun sequence
 gb|CM000471.1| Homo sapiens chromosome 10, whole genome shotgun sequence
Length=128985118

 Score = 87.9 bits (47),  Expect = 2e-15
 Identities = 49/50 (98%), Gaps = 0/50 (0%)
 Strand=Plus/Plus

Query  1          GCCCCAGACCCAAGAATCGCCCGCCTCTCCAAGACCATCCCTGGCTGCTG  50
                  |||||||||||||||||||||||||||||| |||||||||||||||||||
Sbjct  119921448  GCCCCAGACCCAAGAATCGCCCGCCTCTCCGAGACCATCCCTGGCTGCTG  119921497


Lambda     K      H
    1.33    0.621     1.12 

Gapped
Lambda     K      H
    1.28    0.460    0.850 

Effective search space used: 681780354108
 
 right 
 >ref|NT_030059.13| Homo sapiens chromosome 10 genomic contig, GRCh37.p9 Primary 
Assembly
 gb|GL000099.1| Homo sapiens chromosome 10 genomic contig, GRCh37 reference primary 
assembly
Length=79420533

 Score = 93.5 bits (50),  Expect = 5e-17
 Identities = 50/50 (100%), Gaps = 0/50 (0%)
 Strand=Plus/Plus

Query  1         AAATCATGCCACCCTCAAAGCCTCATCCACCTGTCGTGGGCAAAGTGACT  50
                 ||||||||||||||||||||||||||||||||||||||||||||||||||
Sbjct  78473194  AAATCATGCCACCCTCAAAGCCTCATCCACCTGTCGTGGGCAAAGTGACT  78473243


>ref|NW_001838010.1| Homo sapiens chromosome 10 genomic contig, alternate assembly 
HuRef SCAF_1103279188229, whole genome shotgun sequence
 gb|DS486216.1| Homo sapiens SCAF_1103279188229 genomic scaffold, whole genome 
shotgun sequence
Length=2644411

 Score = 93.5 bits (50),  Expect = 5e-17
 Identities = 50/50 (100%), Gaps = 0/50 (0%)
 Strand=Plus/Plus

Query  1        AAATCATGCCACCCTCAAAGCCTCATCCACCTGTCGTGGGCAAAGTGACT  50
                ||||||||||||||||||||||||||||||||||||||||||||||||||
Sbjct  1678799  AAATCATGCCACCCTCAAAGCCTCATCCACCTGTCGTGGGCAAAGTGACT  1678848


>ref|NC_000010.10| Homo sapiens chromosome 10, GRCh37.p9 Primary Assembly
 gb|CM000672.1| Homo sapiens chromosome 10, GRCh37 primary reference assembly
Length=135534747

 Score = 93.5 bits (50),  Expect = 5e-17
 Identities = 50/50 (100%), Gaps = 0/50 (0%)
 Strand=Plus/Plus

Query  1          AAATCATGCCACCCTCAAAGCCTCATCCACCTGTCGTGGGCAAAGTGACT  50
                  ||||||||||||||||||||||||||||||||||||||||||||||||||
Sbjct  127668730  AAATCATGCCACCCTCAAAGCCTCATCCACCTGTCGTGGGCAAAGTGACT  127668779


>ref|AC_000142.1| Homo sapiens chromosome 10, alternate assembly HuRef, whole genome 
shotgun sequence
 gb|CM000471.1| Homo sapiens chromosome 10, whole genome shotgun sequence
Length=128985118

 Score = 93.5 bits (50),  Expect = 5e-17
 Identities = 50/50 (100%), Gaps = 0/50 (0%)
 Strand=Plus/Plus

Query  1          AAATCATGCCACCCTCAAAGCCTCATCCACCTGTCGTGGGCAAAGTGACT  50
                  ||||||||||||||||||||||||||||||||||||||||||||||||||
Sbjct  121357897  AAATCATGCCACCCTCAAAGCCTCATCCACCTGTCGTGGGCAAAGTGACT  121357946


>gb|CM000261.1| Homo sapiens chromosome 10, whole genome shotgun sequence
Length=129189614

 Score = 93.5 bits (50),  Expect = 5e-17
 Identities = 50/50 (100%), Gaps = 0/50 (0%)
 Strand=Plus/Plus

Query  1          AAATCATGCCACCCTCAAAGCCTCATCCACCTGTCGTGGGCAAAGTGACT  50
                  ||||||||||||||||||||||||||||||||||||||||||||||||||
Sbjct  121444157  AAATCATGCCACCCTCAAAGCCTCATCCACCTGTCGTGGGCAAAGTGACT  121444206


>gb|CM000500.1| Homo sapiens chromosome 10, whole genome shotgun sequence
Length=128985077

 Score = 87.9 bits (47),  Expect = 2e-15
 Identities = 49/50 (98%), Gaps = 0/50 (0%)
 Strand=Plus/Plus

Query  1          AAATCATGCCACCCTCAAAGCCTCATCCACCTGTCGTGGGCAAAGTGACT  50
                  ||||||||||||||||||||| ||||||||||||||||||||||||||||
Sbjct  121357942  AAATCATGCCACCCTCAAAGCTTCATCCACCTGTCGTGGGCAAAGTGACT  121357991


>gb|DS990854.1| Homo sapiens SCAF_1112675837174 genomic scaffold, whole genome 
shotgun sequence
Length=2644434

 Score = 87.9 bits (47),  Expect = 2e-15
 Identities = 49/50 (98%), Gaps = 0/50 (0%)
 Strand=Plus/Plus

Query  1        AAATCATGCCACCCTCAAAGCCTCATCCACCTGTCGTGGGCAAAGTGACT  50
                ||||||||||||||||||||| ||||||||||||||||||||||||||||
Sbjct  1678839  AAATCATGCCACCCTCAAAGCTTCATCCACCTGTCGTGGGCAAAGTGACT  1678888


Lambda     K      H
    1.33    0.621     1.12 

Gapped
Lambda     K      H
    1.28    0.460    0.850 

Effective search space used: 681780354108
 
 blast search - nt  
 left 
 
 
 right 
 >ref|XM_003816282.1| PREDICTED: Pan paniscus fibronectin type III and ankyrin repeat 
domains 1 (FANK1), mRNA
Length=1404

 Score = 93.5 bits (50),  Expect = 6e-17
 Identities = 50/50 (100%), Gaps = 0/50 (0%)
 Strand=Plus/Plus

Query  1    AAATCATGCCACCCTCAAAGCCTCATCCACCTGTCGTGGGCAAAGTGACT  50
            ||||||||||||||||||||||||||||||||||||||||||||||||||
Sbjct  121  AAATCATGCCACCCTCAAAGCCTCATCCACCTGTCGTGGGCAAAGTGACT  170


>ref|XM_003312821.1| PREDICTED: Pan troglodytes fibronectin type III and ankyrin repeat 
domains 1, transcript variant 2 (FANK1), mRNA
Length=1348

 Score = 93.5 bits (50),  Expect = 6e-17
 Identities = 50/50 (100%), Gaps = 0/50 (0%)
 Strand=Plus/Plus

Query  1    AAATCATGCCACCCTCAAAGCCTCATCCACCTGTCGTGGGCAAAGTGACT  50
            ||||||||||||||||||||||||||||||||||||||||||||||||||
Sbjct  90   AAATCATGCCACCCTCAAAGCCTCATCCACCTGTCGTGGGCAAAGTGACT  139


>ref|XM_003312820.1| PREDICTED: Pan troglodytes fibronectin type III and ankyrin repeat 
domains 1, transcript variant 1 (FANK1), mRNA
Length=1292

 Score = 93.5 bits (50),  Expect = 6e-17
 Identities = 50/50 (100%), Gaps = 0/50 (0%)
 Strand=Plus/Plus

Query  1    AAATCATGCCACCCTCAAAGCCTCATCCACCTGTCGTGGGCAAAGTGACT  50
            ||||||||||||||||||||||||||||||||||||||||||||||||||
Sbjct  112  AAATCATGCCACCCTCAAAGCCTCATCCACCTGTCGTGGGCAAAGTGACT  161


>dbj|AK304407.1| Homo sapiens cDNA FLJ52987 complete cds, highly similar to Fibronectin 
type 3 and ankyrin repeat domains protein 1
Length=1283

 Score = 93.5 bits (50),  Expect = 6e-17
 Identities = 50/50 (100%), Gaps = 0/50 (0%)
 Strand=Plus/Plus

Query  1    AAATCATGCCACCCTCAAAGCCTCATCCACCTGTCGTGGGCAAAGTGACT  50
            ||||||||||||||||||||||||||||||||||||||||||||||||||
Sbjct  169  AAATCATGCCACCCTCAAAGCCTCATCCACCTGTCGTGGGCAAAGTGACT  218


>ref|NM_145235.3| Homo sapiens fibronectin type III and ankyrin repeat domains 
1 (FANK1), mRNA
Length=1395

 Score = 93.5 bits (50),  Expect = 6e-17
 Identities = 50/50 (100%), Gaps = 0/50 (0%)
 Strand=Plus/Plus

Query  1    AAATCATGCCACCCTCAAAGCCTCATCCACCTGTCGTGGGCAAAGTGACT  50
            ||||||||||||||||||||||||||||||||||||||||||||||||||
Sbjct  118  AAATCATGCCACCCTCAAAGCCTCATCCACCTGTCGTGGGCAAAGTGACT  167


>gb|AY358154.1| Homo sapiens clone DNA176837 CG3104 hlg (UNQ6504) mRNA, complete 
cds
Length=1373

 Score = 93.5 bits (50),  Expect = 6e-17
 Identities = 50/50 (100%), Gaps = 0/50 (0%)
 Strand=Plus/Plus

Query  1    AAATCATGCCACCCTCAAAGCCTCATCCACCTGTCGTGGGCAAAGTGACT  50
            ||||||||||||||||||||||||||||||||||||||||||||||||||
Sbjct  96   AAATCATGCCACCCTCAAAGCCTCATCCACCTGTCGTGGGCAAAGTGACT  145


>gb|AC063963.11| Homo sapiens chromosome 10 clone RP11-295J3, complete sequence
Length=179104

 Score = 93.5 bits (50),  Expect = 6e-17
 Identities = 50/50 (100%), Gaps = 0/50 (0%)
 Strand=Plus/Plus

Query  1      AAATCATGCCACCCTCAAAGCCTCATCCACCTGTCGTGGGCAAAGTGACT  50
              ||||||||||||||||||||||||||||||||||||||||||||||||||
Sbjct  61132  AAATCATGCCACCCTCAAAGCCTCATCCACCTGTCGTGGGCAAAGTGACT  61181


>emb|CR627249.1| Homo sapiens mRNA; cDNA DKFZp781G1012 (from clone DKFZp781G1012)
Length=8002

 Score = 93.5 bits (50),  Expect = 6e-17
 Identities = 50/50 (100%), Gaps = 0/50 (0%)
 Strand=Plus/Plus

Query  1    AAATCATGCCACCCTCAAAGCCTCATCCACCTGTCGTGGGCAAAGTGACT  50
            ||||||||||||||||||||||||||||||||||||||||||||||||||
Sbjct  102  AAATCATGCCACCCTCAAAGCCTCATCCACCTGTCGTGGGCAAAGTGACT  151


>gb|BC024189.2| Homo sapiens fibronectin type III and ankyrin repeat domains 
1, mRNA (cDNA clone MGC:26734 IMAGE:4826296), complete cds
Length=1395

 Score = 91.6 bits (49),  Expect = 2e-16
 Identities = 49/49 (100%), Gaps = 0/49 (0%)
 Strand=Plus/Plus

Query  2    AATCATGCCACCCTCAAAGCCTCATCCACCTGTCGTGGGCAAAGTGACT  50
            |||||||||||||||||||||||||||||||||||||||||||||||||
Sbjct  119  AATCATGCCACCCTCAAAGCCTCATCCACCTGTCGTGGGCAAAGTGACT  167


>gb|AY251163.1| Homo sapiens HSD13 mRNA, complete cds
Length=1220

 Score = 89.8 bits (48),  Expect = 8e-16
 Identities = 48/48 (100%), Gaps = 0/48 (0%)
 Strand=Plus/Plus

Query  3   ATCATGCCACCCTCAAAGCCTCATCCACCTGTCGTGGGCAAAGTGACT  50
           ||||||||||||||||||||||||||||||||||||||||||||||||
Sbjct  20  ATCATGCCACCCTCAAAGCCTCATCCACCTGTCGTGGGCAAAGTGACT  67


>ref|XM_002821261.2| PREDICTED: Pongo abelii fibronectin type III and ankyrin repeat 
domains 1, transcript variant 1 (FANK1), mRNA
Length=1380

 Score = 87.9 bits (47),  Expect = 3e-15
 Identities = 49/50 (98%), Gaps = 0/50 (0%)
 Strand=Plus/Plus

Query  1    AAATCATGCCACCCTCAAAGCCTCATCCACCTGTCGTGGGCAAAGTGACT  50
            |||||| |||||||||||||||||||||||||||||||||||||||||||
Sbjct  122  AAATCACGCCACCCTCAAAGCCTCATCCACCTGTCGTGGGCAAAGTGACT  171


>ref|XM_002821262.2| PREDICTED: Pongo abelii fibronectin type III and ankyrin repeat 
domains 1, transcript variant 2 (FANK1), mRNA
Length=1302

 Score = 87.9 bits (47),  Expect = 3e-15
 Identities = 49/50 (98%), Gaps = 0/50 (0%)
 Strand=Plus/Plus

Query  1    AAATCATGCCACCCTCAAAGCCTCATCCACCTGTCGTGGGCAAAGTGACT  50
            |||||| |||||||||||||||||||||||||||||||||||||||||||
Sbjct  122  AAATCACGCCACCCTCAAAGCCTCATCCACCTGTCGTGGGCAAAGTGACT  171


>ref|XM_001087857.2| PREDICTED: Macaca mulatta fibronectin type III and ankyrin repeat 
domains 1, transcript variant 6 (FANK1), mRNA
Length=1172

 Score = 87.9 bits (47),  Expect = 3e-15
 Identities = 49/50 (98%), Gaps = 0/50 (0%)
 Strand=Plus/Plus

Query  1    AAATCATGCCACCCTCAAAGCCTCATCCACCTGTCGTGGGCAAAGTGACT  50
            |||||| |||||||||||||||||||||||||||||||||||||||||||
Sbjct  118  AAATCACGCCACCCTCAAAGCCTCATCCACCTGTCGTGGGCAAAGTGACT  167


>ref|XM_001087611.2| PREDICTED: Macaca mulatta fibronectin type III and ankyrin repeat 
domains 1, transcript variant 4 (FANK1), mRNA
Length=1297

 Score = 87.9 bits (47),  Expect = 3e-15
 Identities = 49/50 (98%), Gaps = 0/50 (0%)
 Strand=Plus/Plus

Query  1    AAATCATGCCACCCTCAAAGCCTCATCCACCTGTCGTGGGCAAAGTGACT  50
            |||||| |||||||||||||||||||||||||||||||||||||||||||
Sbjct  118  AAATCACGCCACCCTCAAAGCCTCATCCACCTGTCGTGGGCAAAGTGACT  167


>ref|XM_001086789.2| PREDICTED: Macaca mulatta fibronectin type III and ankyrin repeat 
domains 1, transcript variant 1 (FANK1), mRNA
Length=1353

 Score = 87.9 bits (47),  Expect = 3e-15
 Identities = 49/50 (98%), Gaps = 0/50 (0%)
 Strand=Plus/Plus

Query  1    AAATCATGCCACCCTCAAAGCCTCATCCACCTGTCGTGGGCAAAGTGACT  50
            |||||| |||||||||||||||||||||||||||||||||||||||||||
Sbjct  96   AAATCACGCCACCCTCAAAGCCTCATCCACCTGTCGTGGGCAAAGTGACT  145


>ref|XM_002756706.1| PREDICTED: Callithrix jacchus fibronectin type III and ankyrin 
repeat domains 1 (FANK1), mRNA
Length=1759

 Score = 87.9 bits (47),  Expect = 3e-15
 Identities = 49/50 (98%), Gaps = 0/50 (0%)
 Strand=Plus/Plus

Query  1    AAATCATGCCACCCTCAAAGCCTCATCCACCTGTCGTGGGCAAAGTGACT  50
            ||||||||||| ||||||||||||||||||||||||||||||||||||||
Sbjct  317  AAATCATGCCATCCTCAAAGCCTCATCCACCTGTCGTGGGCAAAGTGACT  366


>dbj|AB179439.1| Macaca fascicularis testis cDNA clone: QtsA-20030, similar to 
human fibronectin type 3 and ankyrin repeat domains 1(FANK1), 
mRNA, RefSeq: NM_145235.2
Length=1201

 Score = 87.9 bits (47),  Expect = 3e-15
 Identities = 49/50 (98%), Gaps = 0/50 (0%)
 Strand=Plus/Plus

Query  1    AAATCATGCCACCCTCAAAGCCTCATCCACCTGTCGTGGGCAAAGTGACT  50
            |||||| |||||||||||||||||||||||||||||||||||||||||||
Sbjct  102  AAATCACGCCACCCTCAAAGCCTCATCCACCTGTCGTGGGCAAAGTGACT  151


>ref|XM_003479614.1| PREDICTED: Cavia porcellus fibronectin type 3 and ankyrin repeat 
domains 1 protein-like (LOC100735426), mRNA
Length=1104

 Score = 76.8 bits (41),  Expect = 7e-12
 Identities = 47/50 (94%), Gaps = 0/50 (0%)
 Strand=Plus/Plus

Query  1    AAATCATGCCACCCTCAAAGCCTCATCCACCTGTCGTGGGCAAAGTGACT  50
            |||||| |||  ||||||||||||||||||||||||||||||||||||||
Sbjct  80   AAATCACGCCGTCCTCAAAGCCTCATCCACCTGTCGTGGGCAAAGTGACT  129


Lambda     K      H
    1.33    0.621     1.12 

Gapped
Lambda     K      H
    1.28    0.460    0.850 

Effective search space used: 874911716973
 
    reads 
 chr10 chr10 126234569 126234645 76M                          TGGGATGCTGTCCTCCCTCCCTCCCTCCCTCTGCCAAGCTGGTGGGAAGGCCCTTGCCAAAGCGCACAAGACGCCT
chr10 chr10 126234582 126234658 76M                                       TCCCTCCCTCCCTCCCTCTGCCAAGCTGGTGGGAAGGCCCTTGCCAAAGCGCACAAGACCCCTTCACACAGCAGGG
chr10 chr10 126234585 126234661 76M                                          CTCCCTCCCTCCCTCTGCCAAGCTGGTGGGAAGGCCCTTGCCAAAGCGCACAAGACCCCTTCACACAGCAGGGGCA
chr10 chr10 126234599 126234675 76M                                                        CTGCCAAGCTGGTGGGAAGGCCCTTGCCAAAGCGCACAAGACCCCTTCACACAGCAGAGGCACAAGCTCTTCGAGG
chr10 chr10 126234607 126234683 76M                                                                CTGGTGGGAAGGCCCTTGCCAAAGCGCACAAGACCCCTTCACACAGCAGGGGCACAAGCTCTTCGAGGCAGAGTCG
chr10 chr10 126234610 126234686 76M                                                                   GTGGGAAGGCCCTTGCCAAAGCGCACAAGACCCCTTCACACAGCAGGGGCACAAGCTCTTCGAGGCAGAGTCGCCT
chr10 chr10 126234613 126234689 76M                                                                      GGAAGGCCCTTGCCAAAGCGCACAAGACCCCTTCACACAGCAGGGGCACAAGCTCTTCGAGGCAGAGTCGCCTGCA
chr10 chr10 126234617 126234693 76M                                                                          GGCCCTTGCCAAAGCGCACAAGACCCCTTCACACAGCAGGGGCACAAGCTCTTCGAGGCAGAGTCGCCTGCAGGTG
chr10 chr10 126234620 126234696 76M                                                                             CCTTGCCAAAGCGCACAAGACCCCTTCACACAGCAGGGGCACAAGCTCTTCGAGGCAGAGTCGCCTGCAGGTGGGG
chr10 chr10 126234625 126234701 76M                                                                                  CCAAAGCGCACAAGACCCCTTCACACAGCAGAGGCACAAGCTCTTCGAGGCAGAGTCGCCTGCAGGTGGGGTGGAA
chr10 chr10 126234632 126234708 76M                                                                                         GCACAAGACCCCTTCACACAGCAGGGGCACAAGCTCTTCGAGGCAGAGTCGCCTGCAGGTGGGGTGGAAGGGGTGC
chr10 chr10 126234635 126234711 76M                                                                                            CAAGACCCCTTCACACAGCAGGGGCACAAGCTCTTCGAGGCAGAGTCGCCTGCAGGTGGGGTGGAAGGGGTGCGCC
chr10 chr10 126234638 126234714 76M                                                                                               GACCCCTTCACACAGCAGGGGCACAAGCTCTTCGAGGCAGAGTCGCCTGCAGGTGGGGTGGAAGGGGTGCGCCCCA
chr10 chr10 126234643 126234719 76M                                                                                                    CTTCACACAGCAGGGGCACAAGCTCTTCGAGGCAGAGTCGCCTGCAGGTGGGGTGGAAGGGGTGCGACCCAGACCC
chr10 chr10 126234646 126234722 76M                                                                                                       CACACAGCAGGGGCACAAGCTCTTCGAGGCAGAGTCGCCTGCAGGTGGGGTGGAAGGGGTGCGCCCCAGACCCAAG
chr10 chr10 126234650 126234726 76M                                                                                                           CAGCAGGGGCACAAGCTCTTCGAGGCAGAGTCGCCTGCAGGTGGGGTGGAAGGGGTGCGCCCCAGACCCAAGAATC
chr10 chr10 126234653 126234729 76M                                                                                                              CAGGGGCACAAGCTCTTCGAGGCAGAGTCGCCTGCAGGTGGGGTGGAAGGGGTGCGCCCCAGACCCAAGAATCGCC
chr10 chr10 126234657 126234733 76M                                                                                                                  GGCACAAGCTCTTCGAGGCAGAGTCGCCTGCAGGTGGGGTGGAAGGGGTGCGCCCCAGACCCAAGAATCGCCCGCC
chr10 chr10 126234661 126234737 76M                                                                                                                      CAAGCTCTTCGAGGCAGAGTCGCCTGCAGGTGGGGTGGAAGGGGTGCGCCCCAGACCCAAGAATCGCCCGCCTCTC
chr10 chr10 126234664 126234740 76M                                                                                                                         GCTCTTCGAGGCAGAGTCGCCTGCAGGTGGGGTGGAAGGGGTGCGCCCCAGACCCAAGAATCGCCCGCCTCTCCGA
chr10 chr10 126234667 126234743 76M                                                                                                                            CTTCGAGGCAGAGTCGCCTGCAGGTGGGGTGGAAGGGGTGCGCCCCAGACCCAAGAATCGCCCGCCTCTCCGAGAC
chr10 chr10 126234671 126234747 76M                                                                                                                                GAGGCAGAGTCGCCTGCAGGTGGGGTGGAAGGGGTGCGCCCCAGACCCAAGAATCGCCCGCCTCTCCGAGACCATC
chr10 chr10 126234674 126234750 76M                                                                                                                                   GCAGAGTCGCCTGCAGGTGGGGTGGAAGGGGTGCGCCCCAGACCCAAGAATCGCCCGCCTCTCCGAGACCATCCCT
chr10 chr10 126234679 126234755 76M                                                                                                                                        GTCGCCTGCAGGTGGGGTGGAAGGGGTGCGCCCCAGACCCAAGAATCGCCCGCCTCTCCGAGACCATCCCTGGCTG
chr10 chr10 126234684 126234760 76M                                                                                                                                             CTGCAGGTGGGGGGGAAGGGGTGCGCCCCAGACCCAAGAATCGCCCGCCTCTCCGAGACCATCCCTGGCTGCTGGT
chr10 chr10 126234697 127668744 61M127668730F15M                                                                                                                                             GGAAGGGGTGCGCCCCAGACCCAAGAATCGCCCGCCTCTCCGAGACCATCCCTGGCTGCTG AAATCATGCCACCCT
chr10 chr10 126234697 127668744 61M127668730F15M                                                                                                                                             GGAAGGGGTGCGCCCCAGACCCAAGAATCGCCCGCCTCTCCGAGACCATCCCTGGCTGCTG AAATCATGCCACCCT
chr10 chr10 126234702 127668749 56M127668730F20M                                                                                                                                                  GGGTGCGCCCCAGCCCCAAGAATCGCCCGCCTCTCCGAGACCATCCCTGGCTGCTG AAATCATGCCACCCTCAAAG
chr10 chr10 126234702 127668749 56M127668730F20M                                                                                                                                                  GGGTGCGCCCCAGCCCCAAGAATCGCCCGCCTCTCCGAGACCATCCCTGGCTGCTG AAATCATGCCACCCTCAAAG
chr10 chr10 126234704 127668751 54M127668730F22M                                                                                                                                                    GTGCGCCCCAGACCCAAGAATCGCCCGCCTCTCCGAGACCATCCCTGGCTGCTG AAATCATGCCACCCTCAAAGCC
chr10 chr10 126234705 127668752 53M127668730F23M                                                                                                                                                     TGCGCCCCAGACCCAAGAATCGCCCGCCTCTCCGAGACCATCCCTGGCTGCTG AAATCATGCCACCCTCAAAGCCT
chr10 chr10 126234705 127668752 53M127668730F23M                                                                                                                                                     TGCGCCCCAGACCCAAGAATCGCCCGCCTCTCCGAGACCATCCCTGGCTGCTG AAATCATGCCACCCTCAAAGCCT
chr10 chr10 126234706 127668753 52M127668730F24M                                                                                                                                                      GCGCCCCAGACCCAAGAATCGCCCGCCTCTCCGAGACCATCCCTGGCTGCTG AAATCATGCCACCCTCAAAGCCTC
chr10 chr10 126234709 127668756 49M127668730F27M                                                                                                                                                         CCCCAGACCCAAGAATCGCCCGCCTCTCCGAGACCATCCCTGGCTGCTG AAATCATGCCACCCTCAAAGCCTCATC
chr10 chr10 126234710 127668757 48M127668730F28M                                                                                                                                                          CCCAGACCCAAGAATCGCCCGCCTCTCCGAGACCATCCCTGGCTGCTG AAATCATGCCACCCTCAAAGCCTCATCC
chr10 chr10 126234710 127668757 48M127668730F28M                                                                                                                                                          CCCAGACCCAAGAATCGCCCGCCTCTCCGAGACCATCCCTGGCTGCTG AAATCATGCCACCCTCAAAGCCTCATCC
chr10 chr10 126234710 127668757 48M127668730F28M                                                                                                                                                          CCCAGACCCAAGAATCGCCCGCCTCTCCGAGACCATCCCTGGCTGCTG AAATCATGCCACCCTCAAAGCCTCATCC
chr10 chr10 126234710 127668757 48M127668730F28M                                                                                                                                                          CCCAGACCCAAGAATCGCCCGCCTCTCCGAGACCATCCCTGGCTGCTG AAATCATGCCACCCTCAAAGCCTCATCC
chr10 chr10 126234710 127668757 48M127668730F28M                                                                                                                                                          CCCAGACCCAAGAATCGCCCGCCTCTCCGAGACCATCCCTGGCTGCTG AAATCATGCCACCCTCAAAGCCTCATCC
chr10 chr10 126234711 127668758 47M127668730F29M                                                                                                                                                           CCAGACCCAAGAATCGCCCGCCTCTCCGAGACCATCCCTGGCTGCTG AAATCATGCCACCCTCAAAGCCTCATCCA
chr10 chr10 126234711 127668758 47M127668730F29M                                                                                                                                                           CCAGACCCAAGAATCGCCCGCCTCTCCGAGACCATCCCTGGCTGCTG AAATCATGCCACCCTCAAAGCCTCATCCA
chr10 chr10 126234718 127668765 40M127668730F36M                                                                                                                                                                  CAAGAATCGACCGCCTCTCCGAGACCATCCCTGGCTGCTG AAATCATGCCACCCTCAAAGCCTCATCCACCTGTCG
chr10 chr10 126234727 127668774 31M127668730F45M                                                                                                                                                                           CCCGCCTCTCCGAGACCATCCCTGGCTGCTG AAATCATGCCACCCTCAAAGCCTCATCCACCTGTCGTGGGCAAAG
chr10 chr10 126234727 127668774 31M127668730F45M                                                                                                                                                                           CCCGCCTCTCCGAGACCATCCCTGGCTGCTG AAATCATGCCACCCTCAAAGCCTCATCCACCTGTCGTGGGCAAAG
chr10 chr10 126234727 127668774 31M127668730F45M                                                                                                                                                                           CCCGCCTCTCCGAGACCATCCCTGGCTGCTG AAATCATGCCACCCTCAAAGCCTCATCCACCTGTCGTGGGCAAAG
chr10 chr10 126234727 127668774 31M127668730F45M                                                                                                                                                                           CCCGCCTCTCCGAGACCATCCCTGGCTGCTG AAATCATGCCACCCTCAAAGACTCATCCACCTGTCGTGGGCAAAG
chr10 chr10 126234729 127668776 29M127668730F47M                                                                                                                                                                             CGCCTCTCCGAGACCATCCCTGGCTGCTG AAATCATGCCACCCTCAAAGCCTCATCCACCTGTCGTGGGCAAAGTG
chr10 chr10 126234730 127668777 28M127668730F48M                                                                                                                                                                              GCCTCTCCGAGACCATCCCTGGCTGCTG AAATCATGCCACCCTCAAAGCCTCATCCACCTGTCGTGGGCAAAGTGA
chr10 chr10 126234734 127668781 24M127668730F52M                                                                                                                                                                                  CTCCGAGACCATCCCTGGCTGCTG AAATCATGCCACCCTCAAAGCCTCATCCACCTGTCGTGGGCAAAGTGACTCA
chr10 chr10 126234735 127668782 23M127668730F53M                                                                                                                                                                                   TCCGAGACCATCCCTGGCTGCTG AAATCATGCCACCCTCAAAGCCTCATCCACCTGTCGTGGGCAAAGTGACTCAT
chr10 chr10 126234736 127668783 22M127668730F54M                                                                                                                                                                                    CCGAGACCATCCCTGGCTGCTG AAATCATGCCACCCTCAAAGCCTCATCCACCTGTCGTGGGCAAAGTGACTCATC
chr10 chr10 126234736 127668783 22M127668730F54M                                                                                                                                                                                    CCGAGACCATCCCTGGCTGCTG AAATCATGCCACCCTCAAAGCCTCATCCACCTGTCGTGGGCAAAGTGACTCATC
chr10 chr10 126234737 127668784 21M127668730F55M                                                                                                                                                                                     CGAGACCATCCCTGGCTGCTG AAATCATGCCACCCTCAAAGCCTCATCCACCTGTCGTGGGCAAAGTGACTCATCA
chr10 chr10 126234737 127668784 21M127668730F55M                                                                                                                                                                                     CGAGACCATCCCTGGCTGCTG AAATCATGCCACCCTCAAAGCCTCATCCACCTGTCGTGGGCAAAGTGACTCATCA
chr10 chr10 126234747 127668794 11M127668730F65M                                                                                                                                                                                               CCTGGCTGCTG AAATCATGCCACCCTCAAAGCCTCATCCACCTGTCGTGGGCAAAGTGACTCATCACAGCATTGAA
chr10 chr10 126234748 127668795 10M127668730F66M                                                                                                                                                                                                CTGGCTGCTG AAATCATGCCACCCTCAAAGCCTCATCCACCTGTCGTGGGCAAAGTGACTCATCACAGCATTGAAT
chr10 chr10 126234753 127668800 5M127668730F71M                                                                                                                                                                                                      TGCTG AAATCATGCCACCCTCAAAGCCTCATCCACCTGTCGTGGGCAAAGTGACTCATCACAGCATTGAATTATAC
chr10 chr10 126234753 127668800 5M127668730F71M                                                                                                                                                                                                      TGCTG AAATCATGCCACCCTCAAAGCCTCATCCACCTGTCGTGGGCAAAGTGACTCATCACAGCATTGAATTATAC
chr10 chr10 126234753 127668800 5M127668730F71M                                                                                                                                                                                                      TGCTG AAATCATGCCACCCTCAAAGCCTCATCCACCTGTCGTGGGCAAAGTGACTCATCACAGCATTGAATTATAC
chr10 chr10 127668724 127668800 76M                                                                                                                                                                                                                   TTCAGAAATCATGCCACCCTCAAAGCCTCATCCACCTGTCGTGGGCAAAGTGACTCATCACAGCATTGAATTATAC
chr10 chr10 127668727 127668803 76M                                                                                                                                                                                                                      AGAAATCATGCCACCCTCAAAGCCTCATCCACCTGTCGTGGGCAAAGTGACTCATCACAGCATTGAATTATACTGG
chr10 chr10 127668730 127668806 76M                                                                                                                                                                                                                         AATCATGCCACCCTCAAAGCCTCATCCACCTGTCGTGGGCAAAGTGACTCATCACAGCATTGAATTATACTGGGAT
chr10 chr10 127668735 127668811 76M                                                                                                                                                                                                                              TGCCACCCTCAAAGCCTCATCCACCTGTCGTGGGCAAAGTGACTCATCACAGCATTGAATTATACTGGGATCTGGA
chr10 chr10 127668738 127668814 76M                                                                                                                                                                                                                                 CACCCTCAAAGCCTCATCCACCTGTCGTGGGCAAAGTGACTCATCACAGCATTGAATTATACTGGGATCTGGAAAA
chr10 chr10 127668741 127668817 76M                                                                                                                                                                                                                                    CCTCAAAGCCTCATCCACCTGTCGTGGGCAAAGTGACTCATCACAGCATTGAATTATACTGGGATCTGGAAAAGAA
chr10 chr10 127668744 127668820 76M                                                                                                                                                                                                                                       CAAAGCCTCATCCACCTGTCGTGGGCAAAGTGACTCATCACAGCATTGAATTATACTGGGATCTGGAAAAGAAAGC
chr10 chr10 127668747 127668823 76M                                                                                                                                                                                                                                          AGCCTCATCCACCTGTCGTGGGCAAAGTGACTCATCACAGCATTGAATTATACTGGGATCTGGAAAAGAAAGCCAA
chr10 chr10 127668750 127668826 76M                                                                                                                                                                                                                                             CTCATCCACCTGTCGTGGGCAAAGTGACTCATCACAGCATTGAATTATACTGGGATCTGGAAAAGAAAGCCAAACG
chr10 chr10 127668753 127668829 76M                                                                                                                                                                                                                                                ATCCACCTGTCGTGGGCAAAGTGACTCATCACAGCATTGAATTATACTGGGATCTGGAAAAGAAAGCAAAACGCCA
chr10 chr10 127668756 127668832 76M                                                                                                                                                                                                                                                   CACCTGTCGTGGGCAAAGTGACTCATCACAGCATTGAATTATACTGGGATCTGGAAAAGAAAGCCAAACGCCAAGG
chr10 chr10 127668762 127668838 76M                                                                                                                                                                                                                                                         TCGTGGGCAAAGTGACTCATCACAGCATTGAATTATACTGGGATCTGGAAAAGAAAGCCAAACGCCAAGGACCTCG
chr10 chr10 127668765 127668841 76M                                                                                                                                                                                                                                                            TGGGCAAAGTGACTCATCACAGCATTGAATTATACTGGGATCTGGAAAAGAAAGCCAAACGCCAAGGACCTCAAGA
chr10 chr10 127668768 127668844 76M                                                                                                                                                                                                                                                               GCAAAGTGACTCATCACAGCATTGAATTATACTGGGATCTGGAAAAGAAAGCCAAACGCCAGGGACCTCAAGAGCA
chr10 chr10 127668771 127668847 76M                                                                                                                                                                                                                                                                  AAGTGACTCATCACAGCATTGAATTATACTGGGATCTGGAAAAGAAAGCCAAACGCCAAGGACCTCAAGAGCAGTG
chr10 chr10 127668775 127668851 76M                                                                                                                                                                                                                                                                      GACTCATCACAGCATTGAATTATACTGGGATCTGGAAAAGAAAGCCAAACGCCAAGGACCTCAAGAGCAGTGGTTC
chr10 chr10 127668779 127668855 76M                                                                                                                                                                                                                                                                          CATCACAGCATTGAATTATACTGGGATCTGGAAAAGAAAGCCAAACGCCAAGGACCTCAAGAGCAGTGGTTCAGGT
chr10 chr10 127668782 127668858 76M                                                                                                                                                                                                                                                                             CACAGCATTGAATTATACTGGGATCTGGAAAAGAAAGCCAAACGCCAAGGACCTCAAGAGCAGTGGTTCAGGTTCT
chr10 chr10 127668785 127668861 76M                                                                                                                                                                                                                                                                                AGCATTGAATTATACTGGGATCTGGAAAAGAAAGCCAAACGCCAAGGACCTCAAGAGCAGTGGTTCAGGTTCTCGG
chr10 chr10 127668788 127668864 76M                                                                                                                                                                                                                                                                                   ATTGAATTATACTGGGATCTGGAAAAGAAAGCCAAACGCCAAGGACCTCAAGAGCAGTGGTTCAGGTTCTCGATTG
chr10 chr10 127668791 127668867 76M                                                                                                                                                                                                                                                                                      GAATTATACTGGGATCTGGAAAAGAAAGCCAAACGCCAAGGACCTCAAGAGCAGTGGTTCAGGTTCTCGATTGAAG
chr10 chr10 127668794 127668870 76M                                                                                                                                                                                                                                                                                         TTATACTGGGATCTGGAAAAGAAAGCCAAACGCCAAGGACCTCAAGAGCAGTGGTTCAGGTTCTCGATTGAAGAAG
chr10 chr10 127668797 127668873 76M                                                                                                                                                                                                                                                                                            TACTGGGATCTGGAAAAGAAAGCCAAACGCCAAGGACCTCAAGAGCAGTGGTTCAGGTTCTCGATTGAAGAAGAAG
chr10 chr10 127668800 127668876 76M                                                                                                                                                                                                                                                                                               TGGGATCTGGAAAAGAAAGCCAAACGCCAAGGACCTCAAGAGCAGTGGTTCAGGTTCTCGATTGAAGAAGAAGACC
chr10 chr10 127668803 127668879 76M                                                                                                                                                                                                                                                                                                  GATCTGGAAAAGAAAGCCAAACGCCAAGGACCTCAAGAGCAGTGGTTCAGGTTCTCGATTGAAGAAGAAGACCCCA
chr10 chr10 127668806 127668882 76M                                                                                                                                                                                                                                                                                                     CTGGAAAAGAAAGCCAAACGCCAAGGACCTCAAGAGCAGTGGTTCAGGTTCTCGATTGAAGAAGAAGACCCCAAAA
chr10 chr10 127668809 127668885 76M                                                                                                                                                                                                                                                                                                        GAAAAGAAAGCCAAACGCCAAGGACCTCAAGAGCAGTGGTTCAGGTTCTCGATTGAAGAAGAAGACCCCAAAATGC
chr10 chr10 127668812 127668888 76M                                                                                                                                                                                                                                                                                                           AAGAAAGCCAAACGCCAAGGACCTCAAGAGCAGTGGTTCAGGTTCTCGATTGAAGAACAAGACCCCAAAATGCACA
chr10 chr10 127668818 127668894 76M                                                                                                                                                                                                                                                                                                                 GCCAAACGCCAAGGACCTCAAGAGCAGTGGTTCAGGTTCTCGATTGAAGAAGAAGACCCCAAAATGCACACTTATG
chr10 chr10 127668821 127668897 76M                                                                                                                                                                                                                                                                                                                    AAACGCCAAGGACCTCAAGAGCAGTGGTTCAGGTTCTCGATTGAAGAAGAAGACCCCAAAATGCACACTTATGGTA
chr10 chr10 127668824 127668900 76M                                                                                                                                                                                                                                                                                                                       CGCCAAGGACCTCAAGAGCAGTGGTTCAGGTTCTCGATTGAAGAAGAAGACCCCAAAATGCACACTTATGGTATCA
chr10 chr10 127668827 127668903 76M                                                                                                                                                                                                                                                                                                                          CAAGGACCTCAAGAGCAGTGGTTCAGGTTCTCGATTGAAGAAGAAGACCCCAAAATGCACACTTATGGTATCATTT
chr10 chr10 127668830 127668906 76M                                                                                                                                                                                                                                                                                                                             GGACCTCAAGAGCAGTGGTTCAGGTTCTCGATTGAAGAAGAAGACCCCAAAATGCACACTTATGGTATCATTTATA
chr10 chr10 127668834 127677122 73M8212N3M                                                                                                                                                                                                                                                                                                                          CTCAAGAGCAGTGGTTCAGGTTCTCGATTGAAGAAGAAGACCCCAAAATGCACACTTATGGTATCATTTATAC||||||||||||||||||||||||||||||||||||||||||||||||||||||||||||||||||||||||||||||||||||||||||||||||||||||||||||||||||||||||
chr10 chr10 127668837 127677125 70M8212N6M                                                                                                                                                                                                                                                                                                                             AAGAGCAGTGGTTCAGGTTCTCGATTGAAGAAGAAGACCCCAAAATGCACACTTATGGTATCATTTATAC||||||||||||||||||||||||||||||||||||||||||||||||||||||||||||||||||||||||||||||||||||||||||||||||||||||||||||||||||||||||
chr10 chr10 127668841 127677129 66M8212N10M                                                                                                                                                                                                                                                                                                                                GCAGTGGTTCAGGTTCTCGATTGAAGAAGAAGACCCCAAAATGCACACTTATGGTATCATTTATAC||||||||||||||||||||||||||||||||||||||||||||||||||||||||||||||||||||||||||||||||||||||||||||||||||||||||||||||||||||||||
chr10 chr10 127668847 127677135 60M8212N16M                                                                                                                                                                                                                                                                                                                                      GTTCAGGTTCTCGATTGAAGAAGAAGACCCCAAAATGCACACTTATGGTATCATTTATAC||||||||||||||||||||||||||||||||||||||||||||||||||||||||||||||||||||||||||||||||||||||||||||||||||||||||||||||||||||||||
chr10 chr10 127668850 127668926 76M                                                                                                                                                                                                                                                                                                                                                 CAGGTTCTCGATTGAAGAAGAAGACCCCAAAATGCACACTTATGGTATCATTTATACGTAGGTGCAGTGTTGAATT
chr10 chr10 127668853 127668929 76M                                                                                                                                                                                                                                                                                                                                                    GTTCTCGATTGAAGAAGAAGACCCCAAAATGCACACTTATGGTATCATTTATACGTAGGTGCAGTGTTGAATTGCT
chr10 chr10 127668856 127677144 51M8212N25M                                                                                                                                                                                                                                                                                                                                               CTCGATTGAAGAAGAAGACCCCAAAATGCACACTTATGGTATCATTTATAC||||||||||||||||||||||||||||||||||||||||||||||||||||||||||||||||||||||||||||||||||||||||||||||||||||||||||||||||||||||||
chr10 chr10 127668860 127677148 47M8212N29M                                                                                                                                                                                                                                                                                                                                                   ATTGAAGAAGAAGACCCCAAAATGCACACTTATGGTATCATTTATAC||||||||||||||||||||||||||||||||||||||||||||||||||||||||||||||||||||||||||||||||||||||||||||||||||||||||||||||||||||||||
chr10 chr10 127668864 127668940 76M                                                                                                                                                                                                                                                                                                                                                               AAGAAGAAGACCCCAAAATGCACACTTATGGTATCATTTATACGTAGGTGCAGTGTTGAATTGCTTGCCACTTTGC
chr10 chr10 127668867 127668943 76M                                                                                                                                                                                                                                                                                                                                                                  AAGAAGACCCCAAAATGCACACTTATGGTATCATTTATACGTAGGTGCAGTGTTGAATTGCTTGCCACTTTGCCTT
chr10 chr10 127668870 127677158 37M8212N39M                                                                                                                                                                                                                                                                                                                                                             AAGACCCCAAAATGCACACTTATGGTATCTTTTATAC||||||||||||||||||||||||||||||||||||||||||||||||||||||||||||||||||||||||||||||||||||||||||||||||||||||||||||||||||||||||
chr10 chr10 127668873 127677161 34M8212N42M                                                                                                                                                                                                                                                                                                                                                                ACCCCAAAATGCACACTTATGGTATCATTTATAC||||||||||||||||||||||||||||||||||||||||||||||||||||||||||||||||||||||||||||||||||||||||||||||||||||||||||||||||||||||||
chr10 chr10 127668876 127677164 31M8212N45M                                                                                                                                                                                                                                                                                                                                                                   CCAAAATGCACACTTATGGTATCATTTATAC||||||||||||||||||||||||||||||||||||||||||||||||||||||||||||||||||||||||||||||||||||||||||||||||||||||||||||||||||||||||
chr10 chr10 127668879 127668955 76M                                                                                                                                                                                                                                                                                                                                                                              AAATGCACACTTATGGTATCATTTATACGTAGGTGCAGTGTTGAATTGCTTGCCACTTTGCCTTACTTCCTGATTA
chr10 chr10 127668883 127677171 24M8212N52M                                                                                                                                                                                                                                                                                                                                                                          GCACACTTATGGTATCATTTATAC||||||||||||||||||||||||||||||||||||||||||||||||||||||||||||||||||||||||||||||||||||||||||||||||||||||||||||||||||||||||
chr10 chr10 127668887 127668963 76M                                                                                                                                                                                                                                                                                                                                                                                      ACTTATGGTATCATTTATACGTAGGTGCAGTGTTGAATTGCTTGCCACTTTGCCTTACTTCCTGATTAGCTGGAAT 
    22 pairs 
 -3:7
2:13
8:8
2:21
-28:0
64:17
49:40
10:80
18:80
14:98
23:136
28:164
2:8392
28:8388
30:8394
46:8411
20:8442
-23:8442
17:8449
27:8451
46:8502
70:9180 
     TCGA-02-2483-01A chr10-chr10 126234757 127677119 ff 
 
40 23 31 16 56 56
GCCCCAGACCCAAGAATCGCCCGCCTCTCCAAGACCATCCCTGGCTGCTG GGGATATGCAACGAAGCATGTTGTTGAAGGTCTGGAACCAAGGACGCTGT
99999999999999999999999999999999999999999999999999 99999999999999999999999999999999999999999999999852
LHPP intron6(126205840-126301830) FANK1 exon3(127677118-127677243)
 
 blast search - human genome  
 left 
 >ref|NT_030059.13| Homo sapiens chromosome 10 genomic contig, GRCh37.p9 Primary 
Assembly
 gb|GL000099.1| Homo sapiens chromosome 10 genomic contig, GRCh37 reference primary 
assembly
Length=79420533

 Score = 93.5 bits (50),  Expect = 5e-17
 Identities = 50/50 (100%), Gaps = 0/50 (0%)
 Strand=Plus/Plus

Query  1         GCCCCAGACCCAAGAATCGCCCGCCTCTCCAAGACCATCCCTGGCTGCTG  50
                 ||||||||||||||||||||||||||||||||||||||||||||||||||
Sbjct  77039173  GCCCCAGACCCAAGAATCGCCCGCCTCTCCAAGACCATCCCTGGCTGCTG  77039222


>ref|NC_000010.10| Homo sapiens chromosome 10, GRCh37.p9 Primary Assembly
 gb|CM000672.1| Homo sapiens chromosome 10, GRCh37 primary reference assembly
Length=135534747

 Score = 93.5 bits (50),  Expect = 5e-17
 Identities = 50/50 (100%), Gaps = 0/50 (0%)
 Strand=Plus/Plus

Query  1          GCCCCAGACCCAAGAATCGCCCGCCTCTCCAAGACCATCCCTGGCTGCTG  50
                  ||||||||||||||||||||||||||||||||||||||||||||||||||
Sbjct  126234709  GCCCCAGACCCAAGAATCGCCCGCCTCTCCAAGACCATCCCTGGCTGCTG  126234758


>gb|CM000261.1| Homo sapiens chromosome 10, whole genome shotgun sequence
Length=129189614

 Score = 93.5 bits (50),  Expect = 5e-17
 Identities = 50/50 (100%), Gaps = 0/50 (0%)
 Strand=Plus/Plus

Query  1          GCCCCAGACCCAAGAATCGCCCGCCTCTCCAAGACCATCCCTGGCTGCTG  50
                  ||||||||||||||||||||||||||||||||||||||||||||||||||
Sbjct  120008611  GCCCCAGACCCAAGAATCGCCCGCCTCTCCAAGACCATCCCTGGCTGCTG  120008660


>gb|GL583189.1| Homo sapiens unplaced genomic scaffold scaffold_209, whole genome 
shotgun sequence
Length=3658509

 Score = 89.8 bits (48),  Expect = 7e-16
 Identities = 49/50 (98%), Gaps = 0/50 (0%)
 Strand=Plus/Minus

Query  1        GCCCCAGACCCAAGAATCGCCCGCCTCTCCAAGACCATCCCTGGCTGCTG  50
                |||||||||||||||||||||||||||||| |||||||||||||||||||
Sbjct  2379346  GCCCCAGACCCAAGAATCGCCCGCCTCTCCRAGACCATCCCTGGCTGCTG  2379297


>gb|GL297060.1| Homo sapiens unplaced genomic scaffold scaffold3403, whole genome 
shotgun sequence
Length=1179847

 Score = 87.9 bits (47),  Expect = 2e-15
 Identities = 49/50 (98%), Gaps = 0/50 (0%)
 Strand=Plus/Minus

Query  1       GCCCCAGACCCAAGAATCGCCCGCCTCTCCAAGACCATCCCTGGCTGCTG  50
               |||||||||||||||||||||||||||||| |||||||||||||||||||
Sbjct  777771  GCCCCAGACCCAAGAATCGCCCGCCTCTCCGAGACCATCCCTGGCTGCTG  777722


>ref|NW_001838010.1| Homo sapiens chromosome 10 genomic contig, alternate assembly 
HuRef SCAF_1103279188229, whole genome shotgun sequence
 gb|DS486216.1| Homo sapiens SCAF_1103279188229 genomic scaffold, whole genome 
shotgun sequence
Length=2644411

 Score = 87.9 bits (47),  Expect = 2e-15
 Identities = 49/50 (98%), Gaps = 0/50 (0%)
 Strand=Plus/Plus

Query  1       GCCCCAGACCCAAGAATCGCCCGCCTCTCCAAGACCATCCCTGGCTGCTG  50
               |||||||||||||||||||||||||||||| |||||||||||||||||||
Sbjct  242350  GCCCCAGACCCAAGAATCGCCCGCCTCTCCGAGACCATCCCTGGCTGCTG  242399


>ref|AC_000142.1| Homo sapiens chromosome 10, alternate assembly HuRef, whole genome 
shotgun sequence
 gb|CM000471.1| Homo sapiens chromosome 10, whole genome shotgun sequence
Length=128985118

 Score = 87.9 bits (47),  Expect = 2e-15
 Identities = 49/50 (98%), Gaps = 0/50 (0%)
 Strand=Plus/Plus

Query  1          GCCCCAGACCCAAGAATCGCCCGCCTCTCCAAGACCATCCCTGGCTGCTG  50
                  |||||||||||||||||||||||||||||| |||||||||||||||||||
Sbjct  119921448  GCCCCAGACCCAAGAATCGCCCGCCTCTCCGAGACCATCCCTGGCTGCTG  119921497


Lambda     K      H
    1.33    0.621     1.12 

Gapped
Lambda     K      H
    1.28    0.460    0.850 

Effective search space used: 681780354108
 
 right 
 >ref|NT_030059.13| Homo sapiens chromosome 10 genomic contig, GRCh37.p9 Primary 
Assembly
 gb|GL000099.1| Homo sapiens chromosome 10 genomic contig, GRCh37 reference primary 
assembly
Length=79420533

 Score = 93.5 bits (50),  Expect = 5e-17
 Identities = 50/50 (100%), Gaps = 0/50 (0%)
 Strand=Plus/Plus

Query  1         GGGATATGCAACGAAGCATGTTGTTGAAGGTCTGGAACCAAGGACGCTGT  50
                 ||||||||||||||||||||||||||||||||||||||||||||||||||
Sbjct  78481584  GGGATATGCAACGAAGCATGTTGTTGAAGGTCTGGAACCAAGGACGCTGT  78481633


>ref|NW_001838010.1| Homo sapiens chromosome 10 genomic contig, alternate assembly 
HuRef SCAF_1103279188229, whole genome shotgun sequence
 gb|DS486216.1| Homo sapiens SCAF_1103279188229 genomic scaffold, whole genome 
shotgun sequence
Length=2644411

 Score = 93.5 bits (50),  Expect = 5e-17
 Identities = 50/50 (100%), Gaps = 0/50 (0%)
 Strand=Plus/Plus

Query  1        GGGATATGCAACGAAGCATGTTGTTGAAGGTCTGGAACCAAGGACGCTGT  50
                ||||||||||||||||||||||||||||||||||||||||||||||||||
Sbjct  1687190  GGGATATGCAACGAAGCATGTTGTTGAAGGTCTGGAACCAAGGACGCTGT  1687239


>ref|NC_000010.10| Homo sapiens chromosome 10, GRCh37.p9 Primary Assembly
 gb|CM000672.1| Homo sapiens chromosome 10, GRCh37 primary reference assembly
Length=135534747

 Score = 93.5 bits (50),  Expect = 5e-17
 Identities = 50/50 (100%), Gaps = 0/50 (0%)
 Strand=Plus/Plus

Query  1          GGGATATGCAACGAAGCATGTTGTTGAAGGTCTGGAACCAAGGACGCTGT  50
                  ||||||||||||||||||||||||||||||||||||||||||||||||||
Sbjct  127677120  GGGATATGCAACGAAGCATGTTGTTGAAGGTCTGGAACCAAGGACGCTGT  127677169


>ref|AC_000142.1| Homo sapiens chromosome 10, alternate assembly HuRef, whole genome 
shotgun sequence
 gb|CM000471.1| Homo sapiens chromosome 10, whole genome shotgun sequence
Length=128985118

 Score = 93.5 bits (50),  Expect = 5e-17
 Identities = 50/50 (100%), Gaps = 0/50 (0%)
 Strand=Plus/Plus

Query  1          GGGATATGCAACGAAGCATGTTGTTGAAGGTCTGGAACCAAGGACGCTGT  50
                  ||||||||||||||||||||||||||||||||||||||||||||||||||
Sbjct  121366288  GGGATATGCAACGAAGCATGTTGTTGAAGGTCTGGAACCAAGGACGCTGT  121366337


>gb|CM000261.1| Homo sapiens chromosome 10, whole genome shotgun sequence
Length=129189614

 Score = 93.5 bits (50),  Expect = 5e-17
 Identities = 50/50 (100%), Gaps = 0/50 (0%)
 Strand=Plus/Plus

Query  1          GGGATATGCAACGAAGCATGTTGTTGAAGGTCTGGAACCAAGGACGCTGT  50
                  ||||||||||||||||||||||||||||||||||||||||||||||||||
Sbjct  121452548  GGGATATGCAACGAAGCATGTTGTTGAAGGTCTGGAACCAAGGACGCTGT  121452597


Lambda     K      H
    1.33    0.621     1.12 

Gapped
Lambda     K      H
    1.28    0.460    0.850 

Effective search space used: 681780354108
 
 blast search - nt  
 left 
 
 
 right 
 >ref|XM_003816282.1| PREDICTED: Pan paniscus fibronectin type III and ankyrin repeat 
domains 1 (FANK1), mRNA
Length=1404

 Score = 93.5 bits (50),  Expect = 6e-17
 Identities = 50/50 (100%), Gaps = 0/50 (0%)
 Strand=Plus/Plus

Query  1    GGGATATGCAACGAAGCATGTTGTTGAAGGTCTGGAACCAAGGACGCTGT  50
            ||||||||||||||||||||||||||||||||||||||||||||||||||
Sbjct  299  GGGATATGCAACGAAGCATGTTGTTGAAGGTCTGGAACCAAGGACGCTGT  348


>ref|XM_003312821.1| PREDICTED: Pan troglodytes fibronectin type III and ankyrin repeat 
domains 1, transcript variant 2 (FANK1), mRNA
Length=1348

 Score = 93.5 bits (50),  Expect = 6e-17
 Identities = 50/50 (100%), Gaps = 0/50 (0%)
 Strand=Plus/Plus

Query  1    GGGATATGCAACGAAGCATGTTGTTGAAGGTCTGGAACCAAGGACGCTGT  50
            ||||||||||||||||||||||||||||||||||||||||||||||||||
Sbjct  268  GGGATATGCAACGAAGCATGTTGTTGAAGGTCTGGAACCAAGGACGCTGT  317


>ref|XM_003312820.1| PREDICTED: Pan troglodytes fibronectin type III and ankyrin repeat 
domains 1, transcript variant 1 (FANK1), mRNA
Length=1292

 Score = 93.5 bits (50),  Expect = 6e-17
 Identities = 50/50 (100%), Gaps = 0/50 (0%)
 Strand=Plus/Plus

Query  1    GGGATATGCAACGAAGCATGTTGTTGAAGGTCTGGAACCAAGGACGCTGT  50
            ||||||||||||||||||||||||||||||||||||||||||||||||||
Sbjct  290  GGGATATGCAACGAAGCATGTTGTTGAAGGTCTGGAACCAAGGACGCTGT  339


>ref|XM_001087857.2| PREDICTED: Macaca mulatta fibronectin type III and ankyrin repeat 
domains 1, transcript variant 6 (FANK1), mRNA
Length=1172

 Score = 93.5 bits (50),  Expect = 6e-17
 Identities = 50/50 (100%), Gaps = 0/50 (0%)
 Strand=Plus/Plus

Query  1    GGGATATGCAACGAAGCATGTTGTTGAAGGTCTGGAACCAAGGACGCTGT  50
            ||||||||||||||||||||||||||||||||||||||||||||||||||
Sbjct  296  GGGATATGCAACGAAGCATGTTGTTGAAGGTCTGGAACCAAGGACGCTGT  345


>ref|XM_001087611.2| PREDICTED: Macaca mulatta fibronectin type III and ankyrin repeat 
domains 1, transcript variant 4 (FANK1), mRNA
Length=1297

 Score = 93.5 bits (50),  Expect = 6e-17
 Identities = 50/50 (100%), Gaps = 0/50 (0%)
 Strand=Plus/Plus

Query  1    GGGATATGCAACGAAGCATGTTGTTGAAGGTCTGGAACCAAGGACGCTGT  50
            ||||||||||||||||||||||||||||||||||||||||||||||||||
Sbjct  296  GGGATATGCAACGAAGCATGTTGTTGAAGGTCTGGAACCAAGGACGCTGT  345


>ref|XM_001086789.2| PREDICTED: Macaca mulatta fibronectin type III and ankyrin repeat 
domains 1, transcript variant 1 (FANK1), mRNA
Length=1353

 Score = 93.5 bits (50),  Expect = 6e-17
 Identities = 50/50 (100%), Gaps = 0/50 (0%)
 Strand=Plus/Plus

Query  1    GGGATATGCAACGAAGCATGTTGTTGAAGGTCTGGAACCAAGGACGCTGT  50
            ||||||||||||||||||||||||||||||||||||||||||||||||||
Sbjct  274  GGGATATGCAACGAAGCATGTTGTTGAAGGTCTGGAACCAAGGACGCTGT  323


>dbj|AK304407.1| Homo sapiens cDNA FLJ52987 complete cds, highly similar to Fibronectin 
type 3 and ankyrin repeat domains protein 1
Length=1283

 Score = 93.5 bits (50),  Expect = 6e-17
 Identities = 50/50 (100%), Gaps = 0/50 (0%)
 Strand=Plus/Plus

Query  1    GGGATATGCAACGAAGCATGTTGTTGAAGGTCTGGAACCAAGGACGCTGT  50
            ||||||||||||||||||||||||||||||||||||||||||||||||||
Sbjct  347  GGGATATGCAACGAAGCATGTTGTTGAAGGTCTGGAACCAAGGACGCTGT  396


>ref|NM_145235.3| Homo sapiens fibronectin type III and ankyrin repeat domains 
1 (FANK1), mRNA
Length=1395

 Score = 93.5 bits (50),  Expect = 6e-17
 Identities = 50/50 (100%), Gaps = 0/50 (0%)
 Strand=Plus/Plus

Query  1    GGGATATGCAACGAAGCATGTTGTTGAAGGTCTGGAACCAAGGACGCTGT  50
            ||||||||||||||||||||||||||||||||||||||||||||||||||
Sbjct  296  GGGATATGCAACGAAGCATGTTGTTGAAGGTCTGGAACCAAGGACGCTGT  345


>gb|AY251163.1| Homo sapiens HSD13 mRNA, complete cds
Length=1220

 Score = 93.5 bits (50),  Expect = 6e-17
 Identities = 50/50 (100%), Gaps = 0/50 (0%)
 Strand=Plus/Plus

Query  1    GGGATATGCAACGAAGCATGTTGTTGAAGGTCTGGAACCAAGGACGCTGT  50
            ||||||||||||||||||||||||||||||||||||||||||||||||||
Sbjct  196  GGGATATGCAACGAAGCATGTTGTTGAAGGTCTGGAACCAAGGACGCTGT  245


>gb|AY358154.1| Homo sapiens clone DNA176837 CG3104 hlg (UNQ6504) mRNA, complete 
cds
Length=1373

 Score = 93.5 bits (50),  Expect = 6e-17
 Identities = 50/50 (100%), Gaps = 0/50 (0%)
 Strand=Plus/Plus

Query  1    GGGATATGCAACGAAGCATGTTGTTGAAGGTCTGGAACCAAGGACGCTGT  50
            ||||||||||||||||||||||||||||||||||||||||||||||||||
Sbjct  274  GGGATATGCAACGAAGCATGTTGTTGAAGGTCTGGAACCAAGGACGCTGT  323


>gb|BC024189.2| Homo sapiens fibronectin type III and ankyrin repeat domains 
1, mRNA (cDNA clone MGC:26734 IMAGE:4826296), complete cds
Length=1395

 Score = 93.5 bits (50),  Expect = 6e-17
 Identities = 50/50 (100%), Gaps = 0/50 (0%)
 Strand=Plus/Plus

Query  1    GGGATATGCAACGAAGCATGTTGTTGAAGGTCTGGAACCAAGGACGCTGT  50
            ||||||||||||||||||||||||||||||||||||||||||||||||||
Sbjct  296  GGGATATGCAACGAAGCATGTTGTTGAAGGTCTGGAACCAAGGACGCTGT  345


>dbj|AB179439.1| Macaca fascicularis testis cDNA clone: QtsA-20030, similar to 
human fibronectin type 3 and ankyrin repeat domains 1(FANK1), 
mRNA, RefSeq: NM_145235.2
Length=1201

 Score = 93.5 bits (50),  Expect = 6e-17
 Identities = 50/50 (100%), Gaps = 0/50 (0%)
 Strand=Plus/Plus

Query  1    GGGATATGCAACGAAGCATGTTGTTGAAGGTCTGGAACCAAGGACGCTGT  50
            ||||||||||||||||||||||||||||||||||||||||||||||||||
Sbjct  280  GGGATATGCAACGAAGCATGTTGTTGAAGGTCTGGAACCAAGGACGCTGT  329


>gb|AC063963.11| Homo sapiens chromosome 10 clone RP11-295J3, complete sequence
Length=179104

 Score = 93.5 bits (50),  Expect = 6e-17
 Identities = 50/50 (100%), Gaps = 0/50 (0%)
 Strand=Plus/Plus

Query  1      GGGATATGCAACGAAGCATGTTGTTGAAGGTCTGGAACCAAGGACGCTGT  50
              ||||||||||||||||||||||||||||||||||||||||||||||||||
Sbjct  69522  GGGATATGCAACGAAGCATGTTGTTGAAGGTCTGGAACCAAGGACGCTGT  69571


>emb|CR627249.1| Homo sapiens mRNA; cDNA DKFZp781G1012 (from clone DKFZp781G1012)
Length=8002

 Score = 93.5 bits (50),  Expect = 6e-17
 Identities = 50/50 (100%), Gaps = 0/50 (0%)
 Strand=Plus/Plus

Query  1    GGGATATGCAACGAAGCATGTTGTTGAAGGTCTGGAACCAAGGACGCTGT  50
            ||||||||||||||||||||||||||||||||||||||||||||||||||
Sbjct  267  GGGATATGCAACGAAGCATGTTGTTGAAGGTCTGGAACCAAGGACGCTGT  316


>ref|XM_002821261.2| PREDICTED: Pongo abelii fibronectin type III and ankyrin repeat 
domains 1, transcript variant 1 (FANK1), mRNA
Length=1380

 Score = 87.9 bits (47),  Expect = 3e-15
 Identities = 49/50 (98%), Gaps = 0/50 (0%)
 Strand=Plus/Plus

Query  1    GGGATATGCAACGAAGCATGTTGTTGAAGGTCTGGAACCAAGGACGCTGT  50
            |||||| |||||||||||||||||||||||||||||||||||||||||||
Sbjct  300  GGGATACGCAACGAAGCATGTTGTTGAAGGTCTGGAACCAAGGACGCTGT  349


>ref|XM_002821262.2| PREDICTED: Pongo abelii fibronectin type III and ankyrin repeat 
domains 1, transcript variant 2 (FANK1), mRNA
Length=1302

 Score = 87.9 bits (47),  Expect = 3e-15
 Identities = 49/50 (98%), Gaps = 0/50 (0%)
 Strand=Plus/Plus

Query  1    GGGATATGCAACGAAGCATGTTGTTGAAGGTCTGGAACCAAGGACGCTGT  50
            |||||| |||||||||||||||||||||||||||||||||||||||||||
Sbjct  300  GGGATACGCAACGAAGCATGTTGTTGAAGGTCTGGAACCAAGGACGCTGT  349


>ref|XM_003277780.1| PREDICTED: Nomascus leucogenys fibronectin type 3 and ankyrin 
repeat domains protein 1-like (LOC100599088), mRNA
Length=1298

 Score = 87.9 bits (47),  Expect = 3e-15
 Identities = 47/47 (100%), Gaps = 0/47 (0%)
 Strand=Plus/Plus

Query  1    GGGATATGCAACGAAGCATGTTGTTGAAGGTCTGGAACCAAGGACGC  47
            |||||||||||||||||||||||||||||||||||||||||||||||
Sbjct  296  GGGATATGCAACGAAGCATGTTGTTGAAGGTCTGGAACCAAGGACGC  342


Lambda     K      H
    1.33    0.621     1.12 

Gapped
Lambda     K      H
    1.28    0.460    0.850 

Effective search space used: 874911716973
 
    reads 
 chr10 chr10 126234569 126234645 76M                          TGGGATGCTGTCCTCCCTCCCTCCCTCCCTCTGCCAAGCTGGTGGGAAGGCCCTTGCCAAAGCGCACAAGACGCCT
chr10 chr10 126234582 126234658 76M                                       TCCCTCCCTCCCTCCCTCTGCCAAGCTGGTGGGAAGGCCCTTGCCAAAGCGCACAAGACCCCTTCACACAGCAGGG
chr10 chr10 126234585 126234661 76M                                          CTCCCTCCCTCCCTCTGCCAAGCTGGTGGGAAGGCCCTTGCCAAAGCGCACAAGACCCCTTCACACAGCAGGGGCA
chr10 chr10 126234599 126234675 76M                                                        CTGCCAAGCTGGTGGGAAGGCCCTTGCCAAAGCGCACAAGACCCCTTCACACAGCAGAGGCACAAGCTCTTCGAGG
chr10 chr10 126234607 126234683 76M                                                                CTGGTGGGAAGGCCCTTGCCAAAGCGCACAAGACCCCTTCACACAGCAGGGGCACAAGCTCTTCGAGGCAGAGTCG
chr10 chr10 126234610 126234686 76M                                                                   GTGGGAAGGCCCTTGCCAAAGCGCACAAGACCCCTTCACACAGCAGGGGCACAAGCTCTTCGAGGCAGAGTCGCCT
chr10 chr10 126234613 126234689 76M                                                                      GGAAGGCCCTTGCCAAAGCGCACAAGACCCCTTCACACAGCAGGGGCACAAGCTCTTCGAGGCAGAGTCGCCTGCA
chr10 chr10 126234617 126234693 76M                                                                          GGCCCTTGCCAAAGCGCACAAGACCCCTTCACACAGCAGGGGCACAAGCTCTTCGAGGCAGAGTCGCCTGCAGGTG
chr10 chr10 126234620 126234696 76M                                                                             CCTTGCCAAAGCGCACAAGACCCCTTCACACAGCAGGGGCACAAGCTCTTCGAGGCAGAGTCGCCTGCAGGTGGGG
chr10 chr10 126234625 126234701 76M                                                                                  CCAAAGCGCACAAGACCCCTTCACACAGCAGAGGCACAAGCTCTTCGAGGCAGAGTCGCCTGCAGGTGGGGTGGAA
chr10 chr10 126234632 126234708 76M                                                                                         GCACAAGACCCCTTCACACAGCAGGGGCACAAGCTCTTCGAGGCAGAGTCGCCTGCAGGTGGGGTGGAAGGGGTGC
chr10 chr10 126234635 126234711 76M                                                                                            CAAGACCCCTTCACACAGCAGGGGCACAAGCTCTTCGAGGCAGAGTCGCCTGCAGGTGGGGTGGAAGGGGTGCGCC
chr10 chr10 126234638 126234714 76M                                                                                               GACCCCTTCACACAGCAGGGGCACAAGCTCTTCGAGGCAGAGTCGCCTGCAGGTGGGGTGGAAGGGGTGCGCCCCA
chr10 chr10 126234643 126234719 76M                                                                                                    CTTCACACAGCAGGGGCACAAGCTCTTCGAGGCAGAGTCGCCTGCAGGTGGGGTGGAAGGGGTGCGACCCAGACCC
chr10 chr10 126234646 126234722 76M                                                                                                       CACACAGCAGGGGCACAAGCTCTTCGAGGCAGAGTCGCCTGCAGGTGGGGTGGAAGGGGTGCGCCCCAGACCCAAG
chr10 chr10 126234650 126234726 76M                                                                                                           CAGCAGGGGCACAAGCTCTTCGAGGCAGAGTCGCCTGCAGGTGGGGTGGAAGGGGTGCGCCCCAGACCCAAGAATC
chr10 chr10 126234653 126234729 76M                                                                                                              CAGGGGCACAAGCTCTTCGAGGCAGAGTCGCCTGCAGGTGGGGTGGAAGGGGTGCGCCCCAGACCCAAGAATCGCC
chr10 chr10 126234657 126234733 76M                                                                                                                  GGCACAAGCTCTTCGAGGCAGAGTCGCCTGCAGGTGGGGTGGAAGGGGTGCGCCCCAGACCCAAGAATCGCCCGCC
chr10 chr10 126234661 126234737 76M                                                                                                                      CAAGCTCTTCGAGGCAGAGTCGCCTGCAGGTGGGGTGGAAGGGGTGCGCCCCAGACCCAAGAATCGCCCGCCTCTC
chr10 chr10 126234664 126234740 76M                                                                                                                         GCTCTTCGAGGCAGAGTCGCCTGCAGGTGGGGTGGAAGGGGTGCGCCCCAGACCCAAGAATCGCCCGCCTCTCCGA
chr10 chr10 126234667 126234743 76M                                                                                                                            CTTCGAGGCAGAGTCGCCTGCAGGTGGGGTGGAAGGGGTGCGCCCCAGACCCAAGAATCGCCCGCCTCTCCGAGAC
chr10 chr10 126234671 126234747 76M                                                                                                                                GAGGCAGAGTCGCCTGCAGGTGGGGTGGAAGGGGTGCGCCCCAGACCCAAGAATCGCCCGCCTCTCCGAGACCATC
chr10 chr10 126234674 126234750 76M                                                                                                                                   GCAGAGTCGCCTGCAGGTGGGGTGGAAGGGGTGCGCCCCAGACCCAAGAATCGCCCGCCTCTCCGAGACCATCCCT
chr10 chr10 126234679 126234755 76M                                                                                                                                        GTCGCCTGCAGGTGGGGTGGAAGGGGTGCGCCCCAGACCCAAGAATCGCCCGCCTCTCCGAGACCATCCCTGGCTG
chr10 chr10 126234684 126234760 76M                                                                                                                                             CTGCAGGTGGGGGGGAAGGGGTGCGCCCCAGACCCAAGAATCGCCCGCCTCTCCGAGACCATCCCTGGCTGCTGGT
chr10 chr10 126234687 127677124 71M127677120F5M                                                                                                                                    CAGGTGGGGTGGAAGGGGTGCGCCCCAGACCCAAGAATCGCCCGCCTCTCCGAGACCATCCCTGGCTGCTG GGGAT
chr10 chr10 126234694 127677131 64M127677120F12M                                                                                                                                          GGTGGAAGGGGTGCGCCCCAGACCCAAGAATCGCCCGCCTCTCCGAGACCATCCCTGGCTGCTG GGGATATGCAAC
chr10 chr10 126234695 127677132 63M127677120F13M                                                                                                                                           GTGGAAGGGGTGCGCCCCAGACCCAAGAATCGCCCGCCTCTCCGAGACGATCCCTGGCTGCTG GGGATATGCAACG
chr10 chr10 126234697 127677134 61M127677120F15M                                                                                                                                             GGAAGGGGTGCGCCCCAGACCCAAGAATCGCCCGCCTCTCCGAGACCATCCCTGGCTGCTG GGGATATGCAACGAA
chr10 chr10 126234697 127677134 61M127677120F15M                                                                                                                                             GGAAGGGGTGCGCCCCAGACCCAAGAATCGCCCGCCTCTCCGAGACCATCCCTGGCTGCTG GGGATATGCAACGAA
chr10 chr10 126234697 127677134 61M127677120F15M                                                                                                                                             GGAAGGGGTGCGCCCCAGACCCAAGAATCGCCCGCCTCTCCGAGACCATCCCTGGCTGCTG GGGATATGCAACGAA
chr10 chr10 126234697 127677134 61M127677120F15M                                                                                                                                             GGAAGGGGTGCGCCCCAGACCCAAGAATCGCCCGCCTCTCCGAGACCATCCCTGGCTGCTG GGGATATGCAACGAA
chr10 chr10 126234698 127677135 60M127677120F16M                                                                                                                                              GAAGGGGTGCGCCCCAGACCCAAGAATCGCCCGCCTCTCCGAGACCATCCCTGGCTGCTG GGGATATGCAACGAAG
chr10 chr10 126234702 127677139 56M127677120F20M                                                                                                                                                  GGGTGCGCCCCAGACCCAAGAATCGCCCGCCTCTCCGAGACCATCCCTGGCTGCTG GGGATATGCAACGAAGCATG
chr10 chr10 126234703 127677140 55M127677120F21M                                                                                                                                                   GGTGCGCCCCAGACCCAAGAATCGCCCGCCTCTCCGAGACCATCCCTGGCTGCTG GGGATATGCAACGAAGCATGT
chr10 chr10 126234704 127677141 54M127677120F22M                                                                                                                                                    GTGCGCCCCAGACCCAAGAATCGCCCGCCTCTCCGAGACCATCCCTGGCTGCTG GGGATATGCAACGAAGCATGTT
chr10 chr10 126234704 127677141 54M127677120F22M                                                                                                                                                    GTGCGCCCCAGACCCAAGAATCGCCCGCCTCTCCGAGACCATCCCTGGCTGCTG GGGATATGCAACGAAGCATGTT
chr10 chr10 126234704 127677141 54M127677120F22M                                                                                                                                                    GTGCGCCCCAGACCCAAGAATCGCCCGCCTCTCCGAGACCATCCCTGGCTGCTG GGGATATGCAACGACGCATGTT
chr10 chr10 126234704 127677141 54M127677120F22M                                                                                                                                                    GTGCGCCCCAGACCCAAGAATCGCCCGCCTCTCCGAGACCATCCCTGGCTGCTG GGGATATGCAACGAAGCATGTT
chr10 chr10 126234704 127677141 54M127677120F22M                                                                                                                                                    GTGCGCCCCAGACCCAAGAATCGCCCGCCTCTCCGAGACCATCCCTGGCTGCTG GGGATATGCAACGAAGCATGTT
chr10 chr10 126234705 127677142 53M127677120F23M                                                                                                                                                     TGCGCCCCAGACCCAAGAATCGCCCGCCTCTCCGAGACCATCCCTGGCTGCTG GGGATATGCAACGAAGCATGTTG
chr10 chr10 126234708 127677145 50M127677120F26M                                                                                                                                                        GCCCCAGACCCAAGAATCGCCCGCCTCTCCGAGACCATCCCTGGCTCCTG GGGATATGCAACGAAGCATGTTGTTG
chr10 chr10 126234710 127677147 48M127677120F28M                                                                                                                                                          CCCAGACCCAAGAATCGCCCGCCTCTCCGAGACCATCCCTGGCTGCTG GGGATATGCAACGAAGCATGTTGTTGAA
chr10 chr10 126234710 127677147 48M127677120F28M                                                                                                                                                          CCCAGACCCAAGAATCGCCCGCCTCTCCGAGACCATCCCTGGCTGCTG GGGATATGCAACGAAGCATGTTGTTGAA
chr10 chr10 126234710 127677147 48M127677120F28M                                                                                                                                                          CCCAGACCCAAGAATCGCCCGCCTCTCCGAGACCATCCCTGGCTGCTG GGGATATGCAACGAAGCATGTTGTTGAA
chr10 chr10 126234710 127677147 48M127677120F28M                                                                                                                                                          CCCAGACCCAAGAATNGCCCGCCTCTCCGAGACCATCCCTGGCTGCTG GGGATATGCAACGAAGCATGTTGTTGAA
chr10 chr10 126234712 127677149 46M127677120F30M                                                                                                                                                            CAGACCCAAGAATCGCCCGCCTCTCCGAGACCATCCCTGGCTGCTG GGGATATGCAACGAAGCATGTTGTTGAAGG
chr10 chr10 126234717 127677154 41M127677120F35M                                                                                                                                                                 CCAAGAATCGCCCGCCTCTCCGAGACCATCCCTGGCTGCTG GGGATATGCAACGAAGCATGTTGTTGAAGGTCTGG
chr10 chr10 126234718 127677155 40M127677120F36M                                                                                                                                                                  CAAGAATCGCCCGCCTCTCCGAGACCATCCCTGGCTGCTG GGGATATGCAACGAAGCATGTTGTTGAAGGTCTGGA
chr10 chr10 126234719 127677156 39M127677120F37M                                                                                                                                                                   AAGAATCGCCCGCCTCTCCGAGACCATCCCTGGCTGCTG GGGATATGCAACGAAGCATGTTGTTGAAGGTCTGGAA
chr10 chr10 126234722 127677159 36M127677120F40M                                                                                                                                                                      AATCGCCCGCCTCTCCGAGACCATCCCTGGCTGCCG GGGATATGCAACGAAGCATGTTGTTGAAGGTCTGGAACCA
chr10 chr10 126234723 127677160 35M127677120F41M                                                                                                                                                                       ATCGCCCGCCTCTCCGAGACCATCCCTGGCTGCTG GGGATATGCAACGAAGCATGTTGTTGAAGGTCTGGAACCAA
chr10 chr10 126234723 127677160 35M127677120F41M                                                                                                                                                                       ATCGCCCGCCTCTCCGAGACCATCCCTGGCTGCTG GGGATATGCAACGAAGCATGTTGTTGAAGGTCTGGAACCAA
chr10 chr10 126234724 127677161 34M127677120F42M                                                                                                                                                                        TCGCCCGCCTCTCCGAGACCATCCCTGGCTGCTG GGGATATGCAACGAAGCATGTTGTTGAAGGTCTGGAACCAAG
chr10 chr10 126234724 127677161 34M127677120F42M                                                                                                                                                                        TCGCCCGCCTCTCCGAGACCATCCCTGGCTGCTG GGGATATGCAACGAAGCATGTTGTTGAAGGTCTGGAACCAAG
chr10 chr10 126234724 127677161 34M127677120F42M                                                                                                                                                                        CCGCCCGCCTCTCCGAGACCATCCCTGGCTGCTG GGGATATGCAACGAAGCATGTTGTTGAAGGTCTGGAACCAAG
chr10 chr10 126234724 127677161 34M127677120F42M                                                                                                                                                                        CCGCCCGCCTCTCCGAGACCATCCCTGGCTGCTG GGGATATGCAACGAAGCATGTTGTTGAAGGTCTGGAACCAAG
chr10 chr10 126234724 127677161 34M127677120F42M                                                                                                                                                                        TCGCCCGCCTCTCCGAGACCATCCCTGGCTGCTG GGGATATGCAACGAAGCATGTTGTTGAAGGTCTGGAACCAAG
chr10 chr10 126234725 127677162 33M127677120F43M                                                                                                                                                                         CGCCCGCCTCTCCGAGACCATCCCTGGCTGCTG GGGATATGCAACGAAGCATGTTGTTGAAGGTCTGGAACCAAGG
chr10 chr10 126234725 127677162 33M127677120F43M                                                                                                                                                                         CGCCCGCCTCTCCGAGACCATCCCTGGCTGCTG GGGATATGCAACGAAGCATGTTGTTGAAGGTCTGGAACCAAGG
chr10 chr10 126234725 127677162 33M127677120F43M                                                                                                                                                                         CCCCCGCCTCTCCGAGACCATCCCTGGCTGCTG GGGATATGCAACGAAGCATGTTGTTGAAGGTCTGGAACCAAGG
chr10 chr10 126234727 127677164 31M127677120F45M                                                                                                                                                                           CCCGCCTCTCCGAGACCATCCCTGGCTGCTG GGGATATGCAACGAAGCATGTTGTTGAAGGTCTGGAACCAAGGAC
chr10 chr10 126234728 127677165 30M127677120F46M                                                                                                                                                                            CCGCCTCTCCGAGACCATCCCTGGCTGCTG GGGATATGCAACGAAGCATGTTGTTGAAGGTCTGGAACCAAGGACG
chr10 chr10 126234729 127677166 29M127677120F47M                                                                                                                                                                             CGCCTCTCCGAGACCATCCCTGGCTGCTG GGGATATGCAACGAAGCATGTTGTTGAAGGTCTGGAACCAAGGACGC
chr10 chr10 126234729 127677166 29M127677120F47M                                                                                                                                                                             CGCCTCTCCGAGACCATCCCTGGCTGCTG GGGATATGCAACGAAGCATGTTGTTGAAGGTCTGGAACCAAGGACGC
chr10 chr10 126234730 127677167 28M127677120F48M                                                                                                                                                                              GCCTCTCCGAGACCATCCCTGGCTGCTG GGGATATGCAACGAAGCATGTTGTTGAAGGTCTGGAACCAAGGACGCT
chr10 chr10 126234730 127677167 28M127677120F48M                                                                                                                                                                              GCCTCTCCGAGACCATCCCTGGCTGCTG GGGATATGCAACGAAGCATGTTGTTGAAGGTCTGGAACCAAGGACGCT
chr10 chr10 126234730 127677167 28M127677120F48M                                                                                                                                                                              GCCTCTCCGAGACCATCCCTGGCTGCTG GGGATATGCAACGAAGCATGTTGTTGAAGGTCTGGAACCAAGGACGCT
chr10 chr10 126234731 127677168 27M127677120F49M                                                                                                                                                                               CCTCTCCGAGACCATCCCTGGCTGCTG GGGATATGCAACGAAGCATGTTGTTGAAGGTCTGGAACCAAGGACGCTG
chr10 chr10 126234731 127677168 27M127677120F49M                                                                                                                                                                               CCTCTCCGAGACCATCCCTGGCTGCTG GGGATATGCAACGAAGCATGTTGTTGAAGGTCTGGAACCAAGGACGCTG
chr10 chr10 126234731 127677168 27M127677120F49M                                                                                                                                                                               CCTCTCCGAGACCATCCCTGGCTGCTG GGGATATGCAACGAAGCATGTTGTTGAAGGTCTGGAACCAAGGACGCTG
chr10 chr10 126234737 127677174 21M127677120F55M                                                                                                                                                                                     CGAGACCATCCCTGGCTGCTG GGGATATGCAACGAAGCATGTTGTTGAAGGTCTGGAACCAAGGACGCTGTACAGA
chr10 chr10 126234738 127677175 20M127677120F56M                                                                                                                                                                                      GAGACCATCCCTGGCTGCTG GGGATATGCAACGAAGCATGTTGTTGAAGGTCTGGAACCAAGGACGCTGTACAGAG
chr10 chr10 126234743 127677180 15M127677120F61M                                                                                                                                                                                           CATCCCTGGCTGCTG GGGATATGCAACGAAGCATGTTGTTGAAGGTCTGGAACCAAGGACGCTGTACAGATTTCGC
chr10 chr10 126234743 127677180 15M127677120F61M                                                                                                                                                                                           CATCCCTGGCTGCTG GGGATATGCAACGAAGCATGTTGTTGAAGGTCTGGAACCAAGGACGCTGTACAGATTTCGC
chr10 chr10 126234744 127677181 14M127677120F62M                                                                                                                                                                                            ATCCCTGGCTGCTG GGGATATGCAACGAAGCATGTTGTTGAAGGTCTGGAACCAAGGACGCTGTACAGATTTCGCC
chr10 chr10 126234744 127677181 14M127677120F62M                                                                                                                                                                                            ATCCCTGGCTGCTG GGGATATGCAACGAAGCATGTTGTTGAAGGTCTGGAACCAAGGACGCTGTACAGATTTCGCC
chr10 chr10 126234745 127677182 13M127677120F63M                                                                                                                                                                                             TCCCTGGCTGCTG GGGATATGCAACGAAGCATGTTGTTGAAGGTCTGGAACCAAGGACGCTGTACAGATTTCGCCA
chr10 chr10 126234747 127677184 11M127677120F65M                                                                                                                                                                                               CCTGGCTGCTG GGGATATGCAACGAAGCATGTTGTTGAAGGTCTGGAACCAAGGACGCTGTACAGATTTCGCCTGA
chr10 chr10 126234747 127677184 11M127677120F65M                                                                                                                                                                                               CCTGGCTGCTG GGGATATGCAACGAAGCATGTTGTTGAAGGTCTGGAACCAAGGACGCTGTACAGATTTCGCCTGA
chr10 chr10 126234749 127677186 9M127677120F67M                                                                                                                                                                                                  TGGCTGCTT GGGATATGCAACGAAGCATGTTGTTGAAGGTCTGGAACCAAGGACGCTGTACAGATTTCGCCTGAAG
chr10 chr10 126234753 127677190 5M127677120F71M                                                                                                                                                                                                      TGCTG GGGATATGCAACGAAGCATGTTGTTGAAGGTCTGGAACCAAGGACGCTGTACAGATTTCGCCTGAAGGTCA
chr10 chr10 126234754 127677191 4M127677120F72M                                                                                                                                                                                                       GCTG GGGATATGCAACGAAGCATGTTGTTGAAGGTCTGGAACCAAGGACGCTGTACAGATTTCGCCTGAAGGTCAC
chr10 chr10 126234754 127677191 4M127677120F72M                                                                                                                                                                                                       GCTG GGGATATGCAACGAAGCATGTTGTTGAAGGTCTGGAACCAAGGACGCTGTACAGATTTCGCCTGAAGGTCAC
chr10 chr10 126234754 127677191 4M127677120F72M                                                                                                                                                                                                       GCTG GGGATATGCAACGAAGCATGTTGTTGAAGGTCTGGAACCAAGGACGCTGTACAGATTTCGACTGAAGGTCAC
chr10 chr10 127677115 127677191 76M                                                                                                                                                                                                                    CCAGGGGATATGCAACGAAGCATGTTGTTGAAGGTCTGGAACCAAGGACGCTGTACAGATTTCGCCTGAAGGTCAC
chr10 chr10 127677118 127677194 76M                                                                                                                                                                                                                       GGGGATATGCAACGAAGCATGTTGTTGAAGGTCTGGAACCAAGGACGCTGTACAGATTTCGCCTGAAGGTCACCAG
chr10 chr10 127677121 127677197 76M                                                                                                                                                                                                                          GATATGCAACGAAGCATGTTGTTGAAGGTCTGGAACCAAGGACGCTGTACAGATTTCGCCTGAAGGTCACCAGCCC
chr10 chr10 127677124 127677200 76M                                                                                                                                                                                                                             ATGCAACGAAGCATGTTGTTGAAGGTCTGGAACCAAGGACGCTGTACAGATTTCGCCTGAAGGTCACCAGCCCCTC
chr10 chr10 127677127 127677203 76M                                                                                                                                                                                                                                CAACGAAGCATGTTGTTGAAGGTCTGGAACCAAGGACGCTGTACAGATTTCGCCTGAAGGTCACCAGCCCCTCTGG
chr10 chr10 127677130 127677206 76M                                                                                                                                                                                                                                   CGAAGCATGTTGTTGAAGGTCTGGAACCAAGGACGCTGTACAGATTTCGCCTGAAGGTCACCAGCCCCTCTGGGGA
chr10 chr10 127677133 127677209 76M                                                                                                                                                                                                                                      AGCATGTTGTTGAAGGTCTGGAACCAAGGACGCTGTACAGATTTCGCCTGAAGGTCACCAGCCCCTCTGGGGAGTG
chr10 chr10 127677136 127677212 76M                                                                                                                                                                                                                                         ATGTTGTTGAAGGTCTGGAACCAAGGACGCTGTACAGATTTCGCCTGAAGGTCACCAGCCCCTCTGGGGAGTGTGA
chr10 chr10 127677139 127677215 76M                                                                                                                                                                                                                                            TTGTTGAAGGTCTGGAACCAAGGACGCTGTACAGATTTCGCCTGAAGGTCACCAGCCCCTCTGGGGAGTGTGAGTA
chr10 chr10 127677143 127677219 76M                                                                                                                                                                                                                                                TGAAGGTCTGGAACCAAGGACGCTGTACAGATTTCGCCTGAAGGTCACCAGCCCCTCTGGGGAGTGTGAGTACAGC
chr10 chr10 127677146 127677222 76M                                                                                                                                                                                                                                                   AGGTCTGGAACCAAGGACGCTGTACAGATTTCGCCTGAAGGTCACCAGCCCCTCTGGGGAGTGTGAGTACAGCCGG
chr10 chr10 127677149 127677225 76M                                                                                                                                                                                                                                                      TCTGGAACCAAGGACGCTGTACAGATTTCGCCTGAAGGTCACCAGCCCCTCTGGGGAGTGTGAGTACAGCCCACTC
chr10 chr10 127677153 127677229 76M                                                                                                                                                                                                                                                          GAACCAAGGACGCTGTACAGATTTCGCCTGAAGGTCACCAGCCCCTCTGGGGAGTGTGAGTACAGCCCACTCGTCT
chr10 chr10 127677157 127677233 76M                                                                                                                                                                                                                                                              CAAGGACGCTGTACAGATTTCGCCTGAAGGTCACCAGCCCCTCTGGGGAGTGTGAGTACAGCCCACTCGTCTCAGT
chr10 chr10 127677160 127677236 76M                                                                                                                                                                                                                                                                 GGACGCTGTACAGATTTCGCCTGAAGGTCACCAGCCCCTCTGGGGAGTGTGAGTACAGCCCACTCGTCTCAGTGTC
chr10 chr10 127677163 127677239 76M                                                                                                                                                                                                                                                                    CGCTGTACAGATTTCGCCTGAAGGTCTCCAGCCCCTCTGGGGAGTGTGAGTACAGCCCACTCGTCTCAGTGTCTAC
chr10 chr10 127677167 127677243 76M                                                                                                                                                                                                                                                                        GTACAGATTTCGCCTGAAGGTCACCAGCCCCTCTGGGGAGTGTGAGTACAGCCCACTCGTCTCAGTGTCTACAACC
chr10 chr10 127677170 127677246 76M                                                                                                                                                                                                                                                                           CAGATTTCGCCTGAAGGTCACCAGCCCCTCTGGGGAGTGTGAGTACAGCCCACTCGTCTCAGTGTCTACAACCAGA
chr10 chr10 127677173 127683990 71M6741N5M                                                                                                                                                                                                                                                                       ATTTCGCCTGAAGGTCACCAGCCCCTCTGGGGAGCGTGAGTACAGCCCACTCGTCTCAGTGTCTACAACCA|||||||||||||||||||||||||||||||||||||||||||||||||||||||||||||||||||||||||||||||||||||||||||||||||||||||||||||||||||||||||||||||||||||||||||||||||||||||||||||||||||||||||||||
chr10 chr10 127677176 127683993 68M6741N8M                                                                                                                                                                                                                                                                          TCGCCTGAAGGTCACCAGCCCCTCTGGGGAGTGTGAGTACAGCCCACTCGTCTCAGTGTCTACAACCA|||||||||||||||||||||||||||||||||||||||||||||||||||||||||||||||||||||||||||||||||||||||||||||||||||||||||||||||||||||||||||||||||||||||||||||||||||||||||||||||||||||||||||||
chr10 chr10 127677179 127678009 65M754N11M                                                                                                                                                                                                                                                                             CCTGAAGGTCACCAGCCCCTCTGGGGAGTGTGAGTACAGCCCACTCGTCTCAGTGTCTACAACCA|||||||||||||||||||||||||||||||||||||||||||||||||||||||||||||||||||||||||||||||||||||||||||||||||||||||||||||||||||||||||||||||||||||||||||||||||||||||||||||||||||||||||||||
chr10 chr10 127677184 127684001 60M6741N16M                                                                                                                                                                                                                                                                                 AGGTCACCAGCCCCTCTGGGGAGTGTGAGTACAGCCCACTCGTCTCAGTGTCTACAACCA|||||||||||||||||||||||||||||||||||||||||||||||||||||||||||||||||||||||||||||||||||||||||||||||||||||||||||||||||||||||||||||||||||||||||||||||||||||||||||||||||||||||||||||
chr10 chr10 127677188 127684005 56M6741N20M                                                                                                                                                                                                                                                                                     CACCAGCCCCTCTGGGGAGTGTGAGTACAGCCCACTCGTCTCAGTGTCTACAACCA|||||||||||||||||||||||||||||||||||||||||||||||||||||||||||||||||||||||||||||||||||||||||||||||||||||||||||||||||||||||||||||||||||||||||||||||||||||||||||||||||||||||||||||
chr10 chr10 127677191 127684008 53M6741N23M                                                                                                                                                                                                                                                                                        CAGCCCCTCTGGGGAGTGTGAGTACAGCCCACTCGTCTCAGTGTCTACAACCA|||||||||||||||||||||||||||||||||||||||||||||||||||||||||||||||||||||||||||||||||||||||||||||||||||||||||||||||||||||||||||||||||||||||||||||||||||||||||||||||||||||||||||||
chr10 chr10 127677194 127684011 50M6741N26M                                                                                                                                                                                                                                                                                           CCCCTCTGGGGAGTGTGAGTACAGCCCACTCGTCTCAGTGTCTACAACCA|||||||||||||||||||||||||||||||||||||||||||||||||||||||||||||||||||||||||||||||||||||||||||||||||||||||||||||||||||||||||||||||||||||||||||||||||||||||||||||||||||||||||||||
chr10 chr10 127677197 127684014 47M6741N29M                                                                                                                                                                                                                                                                                              CTCTGGGGAGTGTGAGTACAGCCCACTCGTCTCAGTGTCTACAACCA|||||||||||||||||||||||||||||||||||||||||||||||||||||||||||||||||||||||||||||||||||||||||||||||||||||||||||||||||||||||||||||||||||||||||||||||||||||||||||||||||||||||||||||
chr10 chr10 127677200 127684017 44M6741N32M                                                                                                                                                                                                                                                                                                 TGGGGAGTGTGAGTACAGCCCACTCGTCTCAGTGTCTACAACCA|||||||||||||||||||||||||||||||||||||||||||||||||||||||||||||||||||||||||||||||||||||||||||||||||||||||||||||||||||||||||||||||||||||||||||||||||||||||||||||||||||||||||||||
chr10 chr10 127677203 127684020 41M6741N35M                                                                                                                                                                                                                                                                                                    GGAGTGTGAGTACAGCCCACTCGTCTCAGTGTCTACAACCA|||||||||||||||||||||||||||||||||||||||||||||||||||||||||||||||||||||||||||||||||||||||||||||||||||||||||||||||||||||||||||||||||||||||||||||||||||||||||||||||||||||||||||||
chr10 chr10 127677206 127684023 38M6741N38M                                                                                                                                                                                                                                                                                                       GTGTGAGTACAGCCCCCTCGTCTCAGTGTCTACAACCA|||||||||||||||||||||||||||||||||||||||||||||||||||||||||||||||||||||||||||||||||||||||||||||||||||||||||||||||||||||||||||||||||||||||||||||||||||||||||||||||||||||||||||||
chr10 chr10 127677209 127678039 35M754N41M                                                                                                                                                                                                                                                                                                           TGAGTACAGCCCACTCGTCTCAGTGTCTACAACCA|||||||||||||||||||||||||||||||||||||||||||||||||||||||||||||||||||||||||||||||||||||||||||||||||||||||||||||||||||||||||||||||||||||||||||||||||||||||||||||||||||||||||||||
chr10 chr10 127677212 127684029 32M6741N44M                                                                                                                                                                                                                                                                                                             GTACAGCCCACTCGTCTCAGTGTCTACAACCA|||||||||||||||||||||||||||||||||||||||||||||||||||||||||||||||||||||||||||||||||||||||||||||||||||||||||||||||||||||||||||||||||||||||||||||||||||||||||||||||||||||||||||||
chr10 chr10 127677215 127684032 29M6741N47M                                                                                                                                                                                                                                                                                                                CAGCCCACTCGTCTCAGTGTCTACAACCA|||||||||||||||||||||||||||||||||||||||||||||||||||||||||||||||||||||||||||||||||||||||||||||||||||||||||||||||||||||||||||||||||||||||||||||||||||||||||||||||||||||||||||||
chr10 chr10 127677218 127684035 26M6741N50M                                                                                                                                                                                                                                                                                                                   CCCACTCGTCTCAGTGTCTACAACCA|||||||||||||||||||||||||||||||||||||||||||||||||||||||||||||||||||||||||||||||||||||||||||||||||||||||||||||||||||||||||||||||||||||||||||||||||||||||||||||||||||||||||||||
chr10 chr10 127677221 127684038 23M6741N53M                                                                                                                                                                                                                                                                                                                      ACTCGTCTCAGTGTCTACAACCA|||||||||||||||||||||||||||||||||||||||||||||||||||||||||||||||||||||||||||||||||||||||||||||||||||||||||||||||||||||||||||||||||||||||||||||||||||||||||||||||||||||||||||||
chr10 chr10 127677224 127684041 20M6741N56M                                                                                                                                                                                                                                                                                                                         CGTCTCAGTGTCTACAACCA|||||||||||||||||||||||||||||||||||||||||||||||||||||||||||||||||||||||||||||||||||||||||||||||||||||||||||||||||||||||||||||||||||||||||||||||||||||||||||||||||||||||||||||
chr10 chr10 127677227 127684044 17M6741N59M                                                                                                                                                                                                                                                                                                                            CTCAGTGTCTACAACCA|||||||||||||||||||||||||||||||||||||||||||||||||||||||||||||||||||||||||||||||||||||||||||||||||||||||||||||||||||||||||||||||||||||||||||||||||||||||||||||||||||||||||||||
chr10 chr10 127677231 127684048 13M6741N63M                                                                                                                                                                                                                                                                                                                                GTGTCTACAACCA|||||||||||||||||||||||||||||||||||||||||||||||||||||||||||||||||||||||||||||||||||||||||||||||||||||||||||||||||||||||||||||||||||||||||||||||||||||||||||||||||||||||||||||
chr10 chr10 127677234 127684051 10M6741N66M                                                                                                                                                                                                                                                                                                                                   TCTACAACCA|||||||||||||||||||||||||||||||||||||||||||||||||||||||||||||||||||||||||||||||||||||||||||||||||||||||||||||||||||||||||||||||||||||||||||||||||||||||||||||||||||||||||||||
chr10 chr10 127677238 127684055 6M6741N70M                                                                                                                                                                                                                                                                                                                                        CAACCA|||||||||||||||||||||||||||||||||||||||||||||||||||||||||||||||||||||||||||||||||||||||||||||||||||||||||||||||||||||||||||||||||||||||||||||||||||||||||||||||||||||||||||||
chr10 chr10 127677241 127677921 3M604N73M                                                                                                                                                                                                                                                                                                                                            CCA||||||||||||||||||||||||||||||||||||||||||||||||||||||||||||||||||||||||||||||||||||||||||||||||||||||||||||||||||||||||||||||||||||||||||||||||||||||||||||||||||||||||||||| 
    23 pairs 
 2:2
28:-2
30:4
46:21
20:52
-23:52
17:59
27:61
46:112
70:790
2:6868
28:-8226
23:-8254
14:-8292
10:-8310
18:-8310
2:-8369
2:-8377
-3:-8383
8:-8382
49:-8350
-28:-8390
64:-8373 
     TCGA-02-2483-01A chr10-chr10 126234809 127668729 ff 
 
8 22 10 0 55 56
GAGCAGGGTAGGAGGGTTGTTGGCACCTGCTGTGGCCGGGGCCGTGCCAG AAATCATGCCACCCTCAAAGCCTCATCCACCTGTCGTGGGCAAAGTGACT
23333445555555555555566677777788888888888888888888 88888888888888888888877666655554433333333333333222
LHPP intron6(126205840-126301830) FANK1 exon2(127668728-127668906)
 
 blast search - human genome  
 left 
 >ref|NT_030059.13| Homo sapiens chromosome 10 genomic contig, GRCh37.p9 Primary 
Assembly
 gb|GL000099.1| Homo sapiens chromosome 10 genomic contig, GRCh37 reference primary 
assembly
Length=79420533

 Score = 93.5 bits (50),  Expect = 5e-17
 Identities = 50/50 (100%), Gaps = 0/50 (0%)
 Strand=Plus/Plus

Query  1         GAGCAGGGTAGGAGGGTTGTTGGCACCTGCTGTGGCCGGGGCCGTGCCAG  50
                 ||||||||||||||||||||||||||||||||||||||||||||||||||
Sbjct  77039225  GAGCAGGGTAGGAGGGTTGTTGGCACCTGCTGTGGCCGGGGCCGTGCCAG  77039274


>ref|NW_001838010.1| Homo sapiens chromosome 10 genomic contig, alternate assembly 
HuRef SCAF_1103279188229, whole genome shotgun sequence
 gb|DS486216.1| Homo sapiens SCAF_1103279188229 genomic scaffold, whole genome 
shotgun sequence
Length=2644411

 Score = 93.5 bits (50),  Expect = 5e-17
 Identities = 50/50 (100%), Gaps = 0/50 (0%)
 Strand=Plus/Plus

Query  1       GAGCAGGGTAGGAGGGTTGTTGGCACCTGCTGTGGCCGGGGCCGTGCCAG  50
               ||||||||||||||||||||||||||||||||||||||||||||||||||
Sbjct  242402  GAGCAGGGTAGGAGGGTTGTTGGCACCTGCTGTGGCCGGGGCCGTGCCAG  242451


>ref|NC_000010.10| Homo sapiens chromosome 10, GRCh37.p9 Primary Assembly
 gb|CM000672.1| Homo sapiens chromosome 10, GRCh37 primary reference assembly
Length=135534747

 Score = 93.5 bits (50),  Expect = 5e-17
 Identities = 50/50 (100%), Gaps = 0/50 (0%)
 Strand=Plus/Plus

Query  1          GAGCAGGGTAGGAGGGTTGTTGGCACCTGCTGTGGCCGGGGCCGTGCCAG  50
                  ||||||||||||||||||||||||||||||||||||||||||||||||||
Sbjct  126234761  GAGCAGGGTAGGAGGGTTGTTGGCACCTGCTGTGGCCGGGGCCGTGCCAG  126234810


>ref|AC_000142.1| Homo sapiens chromosome 10, alternate assembly HuRef, whole genome 
shotgun sequence
 gb|CM000471.1| Homo sapiens chromosome 10, whole genome shotgun sequence
Length=128985118

 Score = 93.5 bits (50),  Expect = 5e-17
 Identities = 50/50 (100%), Gaps = 0/50 (0%)
 Strand=Plus/Plus

Query  1          GAGCAGGGTAGGAGGGTTGTTGGCACCTGCTGTGGCCGGGGCCGTGCCAG  50
                  ||||||||||||||||||||||||||||||||||||||||||||||||||
Sbjct  119921500  GAGCAGGGTAGGAGGGTTGTTGGCACCTGCTGTGGCCGGGGCCGTGCCAG  119921549


>gb|CM000261.1| Homo sapiens chromosome 10, whole genome shotgun sequence
Length=129189614

 Score = 93.5 bits (50),  Expect = 5e-17
 Identities = 50/50 (100%), Gaps = 0/50 (0%)
 Strand=Plus/Plus

Query  1          GAGCAGGGTAGGAGGGTTGTTGGCACCTGCTGTGGCCGGGGCCGTGCCAG  50
                  ||||||||||||||||||||||||||||||||||||||||||||||||||
Sbjct  120008663  GAGCAGGGTAGGAGGGTTGTTGGCACCTGCTGTGGCCGGGGCCGTGCCAG  120008712


Lambda     K      H
    1.33    0.621     1.12 

Gapped
Lambda     K      H
    1.28    0.460    0.850 

Effective search space used: 681780354108
 
 right 
 >ref|NT_030059.13| Homo sapiens chromosome 10 genomic contig, GRCh37.p9 Primary 
Assembly
 gb|GL000099.1| Homo sapiens chromosome 10 genomic contig, GRCh37 reference primary 
assembly
Length=79420533

 Score = 93.5 bits (50),  Expect = 5e-17
 Identities = 50/50 (100%), Gaps = 0/50 (0%)
 Strand=Plus/Plus

Query  1         AAATCATGCCACCCTCAAAGCCTCATCCACCTGTCGTGGGCAAAGTGACT  50
                 ||||||||||||||||||||||||||||||||||||||||||||||||||
Sbjct  78473194  AAATCATGCCACCCTCAAAGCCTCATCCACCTGTCGTGGGCAAAGTGACT  78473243


>ref|NW_001838010.1| Homo sapiens chromosome 10 genomic contig, alternate assembly 
HuRef SCAF_1103279188229, whole genome shotgun sequence
 gb|DS486216.1| Homo sapiens SCAF_1103279188229 genomic scaffold, whole genome 
shotgun sequence
Length=2644411

 Score = 93.5 bits (50),  Expect = 5e-17
 Identities = 50/50 (100%), Gaps = 0/50 (0%)
 Strand=Plus/Plus

Query  1        AAATCATGCCACCCTCAAAGCCTCATCCACCTGTCGTGGGCAAAGTGACT  50
                ||||||||||||||||||||||||||||||||||||||||||||||||||
Sbjct  1678799  AAATCATGCCACCCTCAAAGCCTCATCCACCTGTCGTGGGCAAAGTGACT  1678848


>ref|NC_000010.10| Homo sapiens chromosome 10, GRCh37.p9 Primary Assembly
 gb|CM000672.1| Homo sapiens chromosome 10, GRCh37 primary reference assembly
Length=135534747

 Score = 93.5 bits (50),  Expect = 5e-17
 Identities = 50/50 (100%), Gaps = 0/50 (0%)
 Strand=Plus/Plus

Query  1          AAATCATGCCACCCTCAAAGCCTCATCCACCTGTCGTGGGCAAAGTGACT  50
                  ||||||||||||||||||||||||||||||||||||||||||||||||||
Sbjct  127668730  AAATCATGCCACCCTCAAAGCCTCATCCACCTGTCGTGGGCAAAGTGACT  127668779


>ref|AC_000142.1| Homo sapiens chromosome 10, alternate assembly HuRef, whole genome 
shotgun sequence
 gb|CM000471.1| Homo sapiens chromosome 10, whole genome shotgun sequence
Length=128985118

 Score = 93.5 bits (50),  Expect = 5e-17
 Identities = 50/50 (100%), Gaps = 0/50 (0%)
 Strand=Plus/Plus

Query  1          AAATCATGCCACCCTCAAAGCCTCATCCACCTGTCGTGGGCAAAGTGACT  50
                  ||||||||||||||||||||||||||||||||||||||||||||||||||
Sbjct  121357897  AAATCATGCCACCCTCAAAGCCTCATCCACCTGTCGTGGGCAAAGTGACT  121357946


>gb|CM000261.1| Homo sapiens chromosome 10, whole genome shotgun sequence
Length=129189614

 Score = 93.5 bits (50),  Expect = 5e-17
 Identities = 50/50 (100%), Gaps = 0/50 (0%)
 Strand=Plus/Plus

Query  1          AAATCATGCCACCCTCAAAGCCTCATCCACCTGTCGTGGGCAAAGTGACT  50
                  ||||||||||||||||||||||||||||||||||||||||||||||||||
Sbjct  121444157  AAATCATGCCACCCTCAAAGCCTCATCCACCTGTCGTGGGCAAAGTGACT  121444206


>gb|CM000500.1| Homo sapiens chromosome 10, whole genome shotgun sequence
Length=128985077

 Score = 87.9 bits (47),  Expect = 2e-15
 Identities = 49/50 (98%), Gaps = 0/50 (0%)
 Strand=Plus/Plus

Query  1          AAATCATGCCACCCTCAAAGCCTCATCCACCTGTCGTGGGCAAAGTGACT  50
                  ||||||||||||||||||||| ||||||||||||||||||||||||||||
Sbjct  121357942  AAATCATGCCACCCTCAAAGCTTCATCCACCTGTCGTGGGCAAAGTGACT  121357991


>gb|DS990854.1| Homo sapiens SCAF_1112675837174 genomic scaffold, whole genome 
shotgun sequence
Length=2644434

 Score = 87.9 bits (47),  Expect = 2e-15
 Identities = 49/50 (98%), Gaps = 0/50 (0%)
 Strand=Plus/Plus

Query  1        AAATCATGCCACCCTCAAAGCCTCATCCACCTGTCGTGGGCAAAGTGACT  50
                ||||||||||||||||||||| ||||||||||||||||||||||||||||
Sbjct  1678839  AAATCATGCCACCCTCAAAGCTTCATCCACCTGTCGTGGGCAAAGTGACT  1678888


Lambda     K      H
    1.33    0.621     1.12 

Gapped
Lambda     K      H
    1.28    0.460    0.850 

Effective search space used: 681780354108
 
 blast search - nt  
 left 
 
 
 right 
 >ref|XM_003816282.1| PREDICTED: Pan paniscus fibronectin type III and ankyrin repeat 
domains 1 (FANK1), mRNA
Length=1404

 Score = 93.5 bits (50),  Expect = 6e-17
 Identities = 50/50 (100%), Gaps = 0/50 (0%)
 Strand=Plus/Plus

Query  1    AAATCATGCCACCCTCAAAGCCTCATCCACCTGTCGTGGGCAAAGTGACT  50
            ||||||||||||||||||||||||||||||||||||||||||||||||||
Sbjct  121  AAATCATGCCACCCTCAAAGCCTCATCCACCTGTCGTGGGCAAAGTGACT  170


>ref|XM_003312821.1| PREDICTED: Pan troglodytes fibronectin type III and ankyrin repeat 
domains 1, transcript variant 2 (FANK1), mRNA
Length=1348

 Score = 93.5 bits (50),  Expect = 6e-17
 Identities = 50/50 (100%), Gaps = 0/50 (0%)
 Strand=Plus/Plus

Query  1    AAATCATGCCACCCTCAAAGCCTCATCCACCTGTCGTGGGCAAAGTGACT  50
            ||||||||||||||||||||||||||||||||||||||||||||||||||
Sbjct  90   AAATCATGCCACCCTCAAAGCCTCATCCACCTGTCGTGGGCAAAGTGACT  139


>ref|XM_003312820.1| PREDICTED: Pan troglodytes fibronectin type III and ankyrin repeat 
domains 1, transcript variant 1 (FANK1), mRNA
Length=1292

 Score = 93.5 bits (50),  Expect = 6e-17
 Identities = 50/50 (100%), Gaps = 0/50 (0%)
 Strand=Plus/Plus

Query  1    AAATCATGCCACCCTCAAAGCCTCATCCACCTGTCGTGGGCAAAGTGACT  50
            ||||||||||||||||||||||||||||||||||||||||||||||||||
Sbjct  112  AAATCATGCCACCCTCAAAGCCTCATCCACCTGTCGTGGGCAAAGTGACT  161


>dbj|AK304407.1| Homo sapiens cDNA FLJ52987 complete cds, highly similar to Fibronectin 
type 3 and ankyrin repeat domains protein 1
Length=1283

 Score = 93.5 bits (50),  Expect = 6e-17
 Identities = 50/50 (100%), Gaps = 0/50 (0%)
 Strand=Plus/Plus

Query  1    AAATCATGCCACCCTCAAAGCCTCATCCACCTGTCGTGGGCAAAGTGACT  50
            ||||||||||||||||||||||||||||||||||||||||||||||||||
Sbjct  169  AAATCATGCCACCCTCAAAGCCTCATCCACCTGTCGTGGGCAAAGTGACT  218


>ref|NM_145235.3| Homo sapiens fibronectin type III and ankyrin repeat domains 
1 (FANK1), mRNA
Length=1395

 Score = 93.5 bits (50),  Expect = 6e-17
 Identities = 50/50 (100%), Gaps = 0/50 (0%)
 Strand=Plus/Plus

Query  1    AAATCATGCCACCCTCAAAGCCTCATCCACCTGTCGTGGGCAAAGTGACT  50
            ||||||||||||||||||||||||||||||||||||||||||||||||||
Sbjct  118  AAATCATGCCACCCTCAAAGCCTCATCCACCTGTCGTGGGCAAAGTGACT  167


>gb|AY358154.1| Homo sapiens clone DNA176837 CG3104 hlg (UNQ6504) mRNA, complete 
cds
Length=1373

 Score = 93.5 bits (50),  Expect = 6e-17
 Identities = 50/50 (100%), Gaps = 0/50 (0%)
 Strand=Plus/Plus

Query  1    AAATCATGCCACCCTCAAAGCCTCATCCACCTGTCGTGGGCAAAGTGACT  50
            ||||||||||||||||||||||||||||||||||||||||||||||||||
Sbjct  96   AAATCATGCCACCCTCAAAGCCTCATCCACCTGTCGTGGGCAAAGTGACT  145


>gb|AC063963.11| Homo sapiens chromosome 10 clone RP11-295J3, complete sequence
Length=179104

 Score = 93.5 bits (50),  Expect = 6e-17
 Identities = 50/50 (100%), Gaps = 0/50 (0%)
 Strand=Plus/Plus

Query  1      AAATCATGCCACCCTCAAAGCCTCATCCACCTGTCGTGGGCAAAGTGACT  50
              ||||||||||||||||||||||||||||||||||||||||||||||||||
Sbjct  61132  AAATCATGCCACCCTCAAAGCCTCATCCACCTGTCGTGGGCAAAGTGACT  61181


>emb|CR627249.1| Homo sapiens mRNA; cDNA DKFZp781G1012 (from clone DKFZp781G1012)
Length=8002

 Score = 93.5 bits (50),  Expect = 6e-17
 Identities = 50/50 (100%), Gaps = 0/50 (0%)
 Strand=Plus/Plus

Query  1    AAATCATGCCACCCTCAAAGCCTCATCCACCTGTCGTGGGCAAAGTGACT  50
            ||||||||||||||||||||||||||||||||||||||||||||||||||
Sbjct  102  AAATCATGCCACCCTCAAAGCCTCATCCACCTGTCGTGGGCAAAGTGACT  151


>gb|BC024189.2| Homo sapiens fibronectin type III and ankyrin repeat domains 
1, mRNA (cDNA clone MGC:26734 IMAGE:4826296), complete cds
Length=1395

 Score = 91.6 bits (49),  Expect = 2e-16
 Identities = 49/49 (100%), Gaps = 0/49 (0%)
 Strand=Plus/Plus

Query  2    AATCATGCCACCCTCAAAGCCTCATCCACCTGTCGTGGGCAAAGTGACT  50
            |||||||||||||||||||||||||||||||||||||||||||||||||
Sbjct  119  AATCATGCCACCCTCAAAGCCTCATCCACCTGTCGTGGGCAAAGTGACT  167


>gb|AY251163.1| Homo sapiens HSD13 mRNA, complete cds
Length=1220

 Score = 89.8 bits (48),  Expect = 8e-16
 Identities = 48/48 (100%), Gaps = 0/48 (0%)
 Strand=Plus/Plus

Query  3   ATCATGCCACCCTCAAAGCCTCATCCACCTGTCGTGGGCAAAGTGACT  50
           ||||||||||||||||||||||||||||||||||||||||||||||||
Sbjct  20  ATCATGCCACCCTCAAAGCCTCATCCACCTGTCGTGGGCAAAGTGACT  67


>ref|XM_002821261.2| PREDICTED: Pongo abelii fibronectin type III and ankyrin repeat 
domains 1, transcript variant 1 (FANK1), mRNA
Length=1380

 Score = 87.9 bits (47),  Expect = 3e-15
 Identities = 49/50 (98%), Gaps = 0/50 (0%)
 Strand=Plus/Plus

Query  1    AAATCATGCCACCCTCAAAGCCTCATCCACCTGTCGTGGGCAAAGTGACT  50
            |||||| |||||||||||||||||||||||||||||||||||||||||||
Sbjct  122  AAATCACGCCACCCTCAAAGCCTCATCCACCTGTCGTGGGCAAAGTGACT  171


>ref|XM_002821262.2| PREDICTED: Pongo abelii fibronectin type III and ankyrin repeat 
domains 1, transcript variant 2 (FANK1), mRNA
Length=1302

 Score = 87.9 bits (47),  Expect = 3e-15
 Identities = 49/50 (98%), Gaps = 0/50 (0%)
 Strand=Plus/Plus

Query  1    AAATCATGCCACCCTCAAAGCCTCATCCACCTGTCGTGGGCAAAGTGACT  50
            |||||| |||||||||||||||||||||||||||||||||||||||||||
Sbjct  122  AAATCACGCCACCCTCAAAGCCTCATCCACCTGTCGTGGGCAAAGTGACT  171


>ref|XM_001087857.2| PREDICTED: Macaca mulatta fibronectin type III and ankyrin repeat 
domains 1, transcript variant 6 (FANK1), mRNA
Length=1172

 Score = 87.9 bits (47),  Expect = 3e-15
 Identities = 49/50 (98%), Gaps = 0/50 (0%)
 Strand=Plus/Plus

Query  1    AAATCATGCCACCCTCAAAGCCTCATCCACCTGTCGTGGGCAAAGTGACT  50
            |||||| |||||||||||||||||||||||||||||||||||||||||||
Sbjct  118  AAATCACGCCACCCTCAAAGCCTCATCCACCTGTCGTGGGCAAAGTGACT  167


>ref|XM_001087611.2| PREDICTED: Macaca mulatta fibronectin type III and ankyrin repeat 
domains 1, transcript variant 4 (FANK1), mRNA
Length=1297

 Score = 87.9 bits (47),  Expect = 3e-15
 Identities = 49/50 (98%), Gaps = 0/50 (0%)
 Strand=Plus/Plus

Query  1    AAATCATGCCACCCTCAAAGCCTCATCCACCTGTCGTGGGCAAAGTGACT  50
            |||||| |||||||||||||||||||||||||||||||||||||||||||
Sbjct  118  AAATCACGCCACCCTCAAAGCCTCATCCACCTGTCGTGGGCAAAGTGACT  167


>ref|XM_001086789.2| PREDICTED: Macaca mulatta fibronectin type III and ankyrin repeat 
domains 1, transcript variant 1 (FANK1), mRNA
Length=1353

 Score = 87.9 bits (47),  Expect = 3e-15
 Identities = 49/50 (98%), Gaps = 0/50 (0%)
 Strand=Plus/Plus

Query  1    AAATCATGCCACCCTCAAAGCCTCATCCACCTGTCGTGGGCAAAGTGACT  50
            |||||| |||||||||||||||||||||||||||||||||||||||||||
Sbjct  96   AAATCACGCCACCCTCAAAGCCTCATCCACCTGTCGTGGGCAAAGTGACT  145


>ref|XM_002756706.1| PREDICTED: Callithrix jacchus fibronectin type III and ankyrin 
repeat domains 1 (FANK1), mRNA
Length=1759

 Score = 87.9 bits (47),  Expect = 3e-15
 Identities = 49/50 (98%), Gaps = 0/50 (0%)
 Strand=Plus/Plus

Query  1    AAATCATGCCACCCTCAAAGCCTCATCCACCTGTCGTGGGCAAAGTGACT  50
            ||||||||||| ||||||||||||||||||||||||||||||||||||||
Sbjct  317  AAATCATGCCATCCTCAAAGCCTCATCCACCTGTCGTGGGCAAAGTGACT  366


>dbj|AB179439.1| Macaca fascicularis testis cDNA clone: QtsA-20030, similar to 
human fibronectin type 3 and ankyrin repeat domains 1(FANK1), 
mRNA, RefSeq: NM_145235.2
Length=1201

 Score = 87.9 bits (47),  Expect = 3e-15
 Identities = 49/50 (98%), Gaps = 0/50 (0%)
 Strand=Plus/Plus

Query  1    AAATCATGCCACCCTCAAAGCCTCATCCACCTGTCGTGGGCAAAGTGACT  50
            |||||| |||||||||||||||||||||||||||||||||||||||||||
Sbjct  102  AAATCACGCCACCCTCAAAGCCTCATCCACCTGTCGTGGGCAAAGTGACT  151


>ref|XM_003479614.1| PREDICTED: Cavia porcellus fibronectin type 3 and ankyrin repeat 
domains 1 protein-like (LOC100735426), mRNA
Length=1104

 Score = 76.8 bits (41),  Expect = 7e-12
 Identities = 47/50 (94%), Gaps = 0/50 (0%)
 Strand=Plus/Plus

Query  1    AAATCATGCCACCCTCAAAGCCTCATCCACCTGTCGTGGGCAAAGTGACT  50
            |||||| |||  ||||||||||||||||||||||||||||||||||||||
Sbjct  80   AAATCACGCCGTCCTCAAAGCCTCATCCACCTGTCGTGGGCAAAGTGACT  129


Lambda     K      H
    1.33    0.621     1.12 

Gapped
Lambda     K      H
    1.28    0.460    0.850 

Effective search space used: 874911716973
 
    reads 
 chr10 chr10 126234569 126234645 76M                          TGGGATGCTGTCCTCCCTCCCTCCCTCCCTCTGCCAAGCTGGTGGGAAGGCCCTTGCCAAAGCGCACAAGACGCCT
chr10 chr10 126234582 126234658 76M                                       TCCCTCCCTCCCTCCCTCTGCCAAGCTGGTGGGAAGGCCCTTGCCAAAGCGCACAAGACCCCTTCACACAGCAGGG
chr10 chr10 126234585 126234661 76M                                          CTCCCTCCCTCCCTCTGCCAAGCTGGTGGGAAGGCCCTTGCCAAAGCGCACAAGACCCCTTCACACAGCAGGGGCA
chr10 chr10 126234599 126234675 76M                                                        CTGCCAAGCTGGTGGGAAGGCCCTTGCCAAAGCGCACAAGACCCCTTCACACAGCAGAGGCACAAGCTCTTCGAGG
chr10 chr10 126234607 126234683 76M                                                                CTGGTGGGAAGGCCCTTGCCAAAGCGCACAAGACCCCTTCACACAGCAGGGGCACAAGCTCTTCGAGGCAGAGTCG
chr10 chr10 126234610 126234686 76M                                                                   GTGGGAAGGCCCTTGCCAAAGCGCACAAGACCCCTTCACACAGCAGGGGCACAAGCTCTTCGAGGCAGAGTCGCCT
chr10 chr10 126234613 126234689 76M                                                                      GGAAGGCCCTTGCCAAAGCGCACAAGACCCCTTCACACAGCAGGGGCACAAGCTCTTCGAGGCAGAGTCGCCTGCA
chr10 chr10 126234617 126234693 76M                                                                          GGCCCTTGCCAAAGCGCACAAGACCCCTTCACACAGCAGGGGCACAAGCTCTTCGAGGCAGAGTCGCCTGCAGGTG
chr10 chr10 126234620 126234696 76M                                                                             CCTTGCCAAAGCGCACAAGACCCCTTCACACAGCAGGGGCACAAGCTCTTCGAGGCAGAGTCGCCTGCAGGTGGGG
chr10 chr10 126234625 126234701 76M                                                                                  CCAAAGCGCACAAGACCCCTTCACACAGCAGAGGCACAAGCTCTTCGAGGCAGAGTCGCCTGCAGGTGGGGTGGAA
chr10 chr10 126234632 126234708 76M                                                                                         GCACAAGACCCCTTCACACAGCAGGGGCACAAGCTCTTCGAGGCAGAGTCGCCTGCAGGTGGGGTGGAAGGGGTGC
chr10 chr10 126234635 126234711 76M                                                                                            CAAGACCCCTTCACACAGCAGGGGCACAAGCTCTTCGAGGCAGAGTCGCCTGCAGGTGGGGTGGAAGGGGTGCGCC
chr10 chr10 126234638 126234714 76M                                                                                               GACCCCTTCACACAGCAGGGGCACAAGCTCTTCGAGGCAGAGTCGCCTGCAGGTGGGGTGGAAGGGGTGCGCCCCA
chr10 chr10 126234643 126234719 76M                                                                                                    CTTCACACAGCAGGGGCACAAGCTCTTCGAGGCAGAGTCGCCTGCAGGTGGGGTGGAAGGGGTGCGACCCAGACCC
chr10 chr10 126234646 126234722 76M                                                                                                       CACACAGCAGGGGCACAAGCTCTTCGAGGCAGAGTCGCCTGCAGGTGGGGTGGAAGGGGTGCGCCCCAGACCCAAG
chr10 chr10 126234650 126234726 76M                                                                                                           CAGCAGGGGCACAAGCTCTTCGAGGCAGAGTCGCCTGCAGGTGGGGTGGAAGGGGTGCGCCCCAGACCCAAGAATC
chr10 chr10 126234653 126234729 76M                                                                                                              CAGGGGCACAAGCTCTTCGAGGCAGAGTCGCCTGCAGGTGGGGTGGAAGGGGTGCGCCCCAGACCCAAGAATCGCC
chr10 chr10 126234657 126234733 76M                                                                                                                  GGCACAAGCTCTTCGAGGCAGAGTCGCCTGCAGGTGGGGTGGAAGGGGTGCGCCCCAGACCCAAGAATCGCCCGCC
chr10 chr10 126234661 126234737 76M                                                                                                                      CAAGCTCTTCGAGGCAGAGTCGCCTGCAGGTGGGGTGGAAGGGGTGCGCCCCAGACCCAAGAATCGCCCGCCTCTC
chr10 chr10 126234664 126234740 76M                                                                                                                         GCTCTTCGAGGCAGAGTCGCCTGCAGGTGGGGTGGAAGGGGTGCGCCCCAGACCCAAGAATCGCCCGCCTCTCCGA
chr10 chr10 126234667 126234743 76M                                                                                                                            CTTCGAGGCAGAGTCGCCTGCAGGTGGGGTGGAAGGGGTGCGCCCCAGACCCAAGAATCGCCCGCCTCTCCGAGAC
chr10 chr10 126234671 126234747 76M                                                                                                                                GAGGCAGAGTCGCCTGCAGGTGGGGTGGAAGGGGTGCGCCCCAGACCCAAGAATCGCCCGCCTCTCCGAGACCATC
chr10 chr10 126234674 126234750 76M                                                                                                                                   GCAGAGTCGCCTGCAGGTGGGGTGGAAGGGGTGCGCCCCAGACCCAAGAATCGCCCGCCTCTCCGAGACCATCCCT
chr10 chr10 126234679 126234755 76M                                                                                                                                        GTCGCCTGCAGGTGGGGTGGAAGGGGTGCGCCCCAGACCCAAGAATCGCCCGCCTCTCCGAGACCATCCCTGGCTG
chr10 chr10 126234684 126234760 76M                                                                                                                                             CTGCAGGTGGGGGGGAAGGGGTGCGCCCCAGACCCAAGAATCGCCCGCCTCTCCGAGACCATCCCTGGCTGCTGGT
chr10 chr10 126234697 126234773 76M                                                                                                                                                          GGAAGGGGTGCGCCCCAGACCCAAGAATCGCCCGCCTCTCCGAGACCATCCCTGGCTGCTGGTGAGCAGGGTAGGA
chr10 chr10 126234701 126234777 76M                                                                                                                                                              GGGGTGCGCCCCAGACCCAAGAATCGCCCGCCTCTCCAAGACCATCCCTGGCTGCTGGTGAGCAGGGTAGGAGGGT
chr10 chr10 126234704 126234780 76M                                                                                                                                                                 GTGCGCCCCAGACCCAAGAATCGCCCGCCTCTCCGAGACCATCCCTGGCTGCTGGTGAGCAGGGTAGGAGGGTTGT
chr10 chr10 126234708 126234784 76M                                                                                                                                                                     GCCCCAGACCCAAGAATCGCCCGCCTCTCCGAGACCATCCCTGGCTGCTGGTGAGCAGGGTAGGAGGGTTGTTGGC
chr10 chr10 126234711 126234787 76M                                                                                                                                                                        CCAGACCCAAGAATCGCCCGCCTCTCCGAGACCATCCCTGGCTGCTGGTGAGCAGGGGAGGAGGGTTGTTGGCACC
chr10 chr10 126234723 126234799 76M                                                                                                                                                                                    ATCGCCCGCCTCTCCAAGACCATCCCTGGCTGCTGGTGAGCAGGGTAGGAGGGTTGTTGGCACCTGCTGTGGCCGG
chr10 chr10 126234727 126234803 76M                                                                                                                                                                                        CCCGCCTCTCCGAGACCATCCCTGGCTGCTGGTGAGCAGGGTAGGAGGGTTGTTGGCACCTGCTGTGGCCGGGGCC
chr10 chr10 126234731 126234807 76M                                                                                                                                                                                            CCTCTCCGAGACCATCCCTGGCTGCTGGTGAGCAGGGTAGGAGGGTTGTTGGCACCTGCTGTGGCCGGGGCCGTGC
chr10 chr10 126234734 126234810 76M                                                                                                                                                                                               CTCCGAGACCATCCCTGGCTGCTGGTGAGCAGGGTAGGAGGGTTGTTGGCACCTGCTGTGGCCGGGGCCGTGCCAG
chr10 chr10 126234755 127668750 55M127668730F21M                                                                                                                                                                                                       CTGGTGAGCAGGGTAGGAGGGTTGTTGGCACCTGCTGTGGCCGGGGCCGTGCCAG AAATCATGCCACCCTCAAAGC
chr10 chr10 126234757 127668752 53M127668730F23M                                                                                                                                                                                                         GGTGAGCAGGGTAGGAGGGTTGTTGGCACCTGCTGTGGCCGGGGCCGTGCCAG AAATCATGCCACCCTCAAAGCCT
chr10 chr10 126234761 127668756 49M127668730F27M                                                                                                                                                                                                             AGCAGGGTAGGAGGGTTGTTGGCACCTGCTGTGGCCGGGGCCGTGCCAG AAATCATGCCACCCTCAAAGCCTCATC
chr10 chr10 126234765 127668760 45M127668730F31M                                                                                                                                                                                                                 GGGTAGGAGGGTTGTTGGCACCTGCTGTGGCCGGGGCCGTGCCAG AAATCATGCCACCCTCAAAGCCTCATCCACC
chr10 chr10 126234767 127668762 43M127668730F33M                                                                                                                                                                                                                   GTAGGAGGGTTGTTGGCACCTGCTGTGGCCGGGGCCGTGCCAG AAATCATGCCACCCTCAAAGCCTCATCCACCTG
chr10 chr10 126234781 127668776 29M127668730F47M                                                                                                                                                                                                                                 GGCACCTGCTGTGGCCGGGGCCGTGCCAG AAATCATGCCACCCTCAAAGCCTCATCCACCTGTCGTGGGCAAAGTG
chr10 chr10 126234784 127668779 26M127668730F50M                                                                                                                                                                                                                                    ACCTGCTGTGGCCGGGGCCGTGCCAG AAATCATGCCACCCTCAAAGCCTCATCCACCTGTCGTGGGCAAAGTGACT
chr10 chr10 126234790 127668785 20M127668730F56M                                                                                                                                                                                                                                          TGTGGCCGGGGCCGTGCCAG AAATCATGCCACCCTCAAAGCCTCATCCACCTGTCGTGGGCAAAGTGACTCATCAC
chr10 chr10 126234801 127668796 9M127668730F67M                                                                                                                                                                                                                                                      CCGTGCCAG AAATCATGCCACCCTCAAAGCCTCATCCACCTGTCGTGGGCAAAGTGACTCATCACAGCATTGAATT
chr10 chr10 126234805 127668800 5M127668730F71M                                                                                                                                                                                                                                                          GCCAG AAATCATGCCACCCTCAAAGCCTCATCCACCTGTCGTGGGCAAAGTGACTCATCACAGCATTGAATTATAC
chr10 chr10 127668724 127668800 76M                                                                                                                                                                                                                                                                       TTCAGAAATCATGCCACCCTCAAAGCCTCATCCACCTGTCGTGGGCAAAGTGACTCATCACAGCATTGAATTATAC
chr10 chr10 127668727 127668803 76M                                                                                                                                                                                                                                                                          AGAAATCATGCCACCCTCAAAGCCTCATCCACCTGTCGTGGGCAAAGTGACTCATCACAGCATTGAATTATACTGG
chr10 chr10 127668730 127668806 76M                                                                                                                                                                                                                                                                             AATCATGCCACCCTCAAAGCCTCATCCACCTGTCGTGGGCAAAGTGACTCATCACAGCATTGAATTATACTGGGAT
chr10 chr10 127668735 127668811 76M                                                                                                                                                                                                                                                                                  TGCCACCCTCAAAGCCTCATCCACCTGTCGTGGGCAAAGTGACTCATCACAGCATTGAATTATACTGGGATCTGGA
chr10 chr10 127668738 127668814 76M                                                                                                                                                                                                                                                                                     CACCCTCAAAGCCTCATCCACCTGTCGTGGGCAAAGTGACTCATCACAGCATTGAATTATACTGGGATCTGGAAAA
chr10 chr10 127668741 127668817 76M                                                                                                                                                                                                                                                                                        CCTCAAAGCCTCATCCACCTGTCGTGGGCAAAGTGACTCATCACAGCATTGAATTATACTGGGATCTGGAAAAGAA
chr10 chr10 127668744 127668820 76M                                                                                                                                                                                                                                                                                           CAAAGCCTCATCCACCTGTCGTGGGCAAAGTGACTCATCACAGCATTGAATTATACTGGGATCTGGAAAAGAAAGC
chr10 chr10 127668747 127668823 76M                                                                                                                                                                                                                                                                                              AGCCTCATCCACCTGTCGTGGGCAAAGTGACTCATCACAGCATTGAATTATACTGGGATCTGGAAAAGAAAGCCAA
chr10 chr10 127668750 127668826 76M                                                                                                                                                                                                                                                                                                 CTCATCCACCTGTCGTGGGCAAAGTGACTCATCACAGCATTGAATTATACTGGGATCTGGAAAAGAAAGCCAAACG
chr10 chr10 127668753 127668829 76M                                                                                                                                                                                                                                                                                                    ATCCACCTGTCGTGGGCAAAGTGACTCATCACAGCATTGAATTATACTGGGATCTGGAAAAGAAAGCAAAACGCCA
chr10 chr10 127668756 127668832 76M                                                                                                                                                                                                                                                                                                       CACCTGTCGTGGGCAAAGTGACTCATCACAGCATTGAATTATACTGGGATCTGGAAAAGAAAGCCAAACGCCAAGG
chr10 chr10 127668762 127668838 76M                                                                                                                                                                                                                                                                                                             TCGTGGGCAAAGTGACTCATCACAGCATTGAATTATACTGGGATCTGGAAAAGAAAGCCAAACGCCAAGGACCTCG
chr10 chr10 127668765 127668841 76M                                                                                                                                                                                                                                                                                                                TGGGCAAAGTGACTCATCACAGCATTGAATTATACTGGGATCTGGAAAAGAAAGCCAAACGCCAAGGACCTCAAGA
chr10 chr10 127668768 127668844 76M                                                                                                                                                                                                                                                                                                                   GCAAAGTGACTCATCACAGCATTGAATTATACTGGGATCTGGAAAAGAAAGCCAAACGCCAGGGACCTCAAGAGCA
chr10 chr10 127668771 127668847 76M                                                                                                                                                                                                                                                                                                                      AAGTGACTCATCACAGCATTGAATTATACTGGGATCTGGAAAAGAAAGCCAAACGCCAAGGACCTCAAGAGCAGTG
chr10 chr10 127668775 127668851 76M                                                                                                                                                                                                                                                                                                                          GACTCATCACAGCATTGAATTATACTGGGATCTGGAAAAGAAAGCCAAACGCCAAGGACCTCAAGAGCAGTGGTTC
chr10 chr10 127668779 127668855 76M                                                                                                                                                                                                                                                                                                                              CATCACAGCATTGAATTATACTGGGATCTGGAAAAGAAAGCCAAACGCCAAGGACCTCAAGAGCAGTGGTTCAGGT
chr10 chr10 127668782 127668858 76M                                                                                                                                                                                                                                                                                                                                 CACAGCATTGAATTATACTGGGATCTGGAAAAGAAAGCCAAACGCCAAGGACCTCAAGAGCAGTGGTTCAGGTTCT
chr10 chr10 127668785 127668861 76M                                                                                                                                                                                                                                                                                                                                    AGCATTGAATTATACTGGGATCTGGAAAAGAAAGCCAAACGCCAAGGACCTCAAGAGCAGTGGTTCAGGTTCTCGG
chr10 chr10 127668788 127668864 76M                                                                                                                                                                                                                                                                                                                                       ATTGAATTATACTGGGATCTGGAAAAGAAAGCCAAACGCCAAGGACCTCAAGAGCAGTGGTTCAGGTTCTCGATTG
chr10 chr10 127668791 127668867 76M                                                                                                                                                                                                                                                                                                                                          GAATTATACTGGGATCTGGAAAAGAAAGCCAAACGCCAAGGACCTCAAGAGCAGTGGTTCAGGTTCTCGATTGAAG
chr10 chr10 127668794 127668870 76M                                                                                                                                                                                                                                                                                                                                             TTATACTGGGATCTGGAAAAGAAAGCCAAACGCCAAGGACCTCAAGAGCAGTGGTTCAGGTTCTCGATTGAAGAAG
chr10 chr10 127668797 127668873 76M                                                                                                                                                                                                                                                                                                                                                TACTGGGATCTGGAAAAGAAAGCCAAACGCCAAGGACCTCAAGAGCAGTGGTTCAGGTTCTCGATTGAAGAAGAAG
chr10 chr10 127668800 127668876 76M                                                                                                                                                                                                                                                                                                                                                   TGGGATCTGGAAAAGAAAGCCAAACGCCAAGGACCTCAAGAGCAGTGGTTCAGGTTCTCGATTGAAGAAGAAGACC
chr10 chr10 127668803 127668879 76M                                                                                                                                                                                                                                                                                                                                                      GATCTGGAAAAGAAAGCCAAACGCCAAGGACCTCAAGAGCAGTGGTTCAGGTTCTCGATTGAAGAAGAAGACCCCA
chr10 chr10 127668806 127668882 76M                                                                                                                                                                                                                                                                                                                                                         CTGGAAAAGAAAGCCAAACGCCAAGGACCTCAAGAGCAGTGGTTCAGGTTCTCGATTGAAGAAGAAGACCCCAAAA
chr10 chr10 127668809 127668885 76M                                                                                                                                                                                                                                                                                                                                                            GAAAAGAAAGCCAAACGCCAAGGACCTCAAGAGCAGTGGTTCAGGTTCTCGATTGAAGAAGAAGACCCCAAAATGC
chr10 chr10 127668812 127668888 76M                                                                                                                                                                                                                                                                                                                                                               AAGAAAGCCAAACGCCAAGGACCTCAAGAGCAGTGGTTCAGGTTCTCGATTGAAGAACAAGACCCCAAAATGCACA
chr10 chr10 127668818 127668894 76M                                                                                                                                                                                                                                                                                                                                                                     GCCAAACGCCAAGGACCTCAAGAGCAGTGGTTCAGGTTCTCGATTGAAGAAGAAGACCCCAAAATGCACACTTATG
chr10 chr10 127668821 127668897 76M                                                                                                                                                                                                                                                                                                                                                                        AAACGCCAAGGACCTCAAGAGCAGTGGTTCAGGTTCTCGATTGAAGAAGAAGACCCCAAAATGCACACTTATGGTA
chr10 chr10 127668824 127668900 76M                                                                                                                                                                                                                                                                                                                                                                           CGCCAAGGACCTCAAGAGCAGTGGTTCAGGTTCTCGATTGAAGAAGAAGACCCCAAAATGCACACTTATGGTATCA
chr10 chr10 127668827 127668903 76M                                                                                                                                                                                                                                                                                                                                                                              CAAGGACCTCAAGAGCAGTGGTTCAGGTTCTCGATTGAAGAAGAAGACCCCAAAATGCACACTTATGGTATCATTT
chr10 chr10 127668830 127668906 76M                                                                                                                                                                                                                                                                                                                                                                                 GGACCTCAAGAGCAGTGGTTCAGGTTCTCGATTGAAGAAGAAGACCCCAAAATGCACACTTATGGTATCATTTATA
chr10 chr10 127668834 127677122 73M8212N3M                                                                                                                                                                                                                                                                                                                                                                              CTCAAGAGCAGTGGTTCAGGTTCTCGATTGAAGAAGAAGACCCCAAAATGCACACTTATGGTATCATTTATAC||||||||||||||||||||||||||||||||||||||||||||||||||||||||||||||||||||||||||||||||||||||||||||||||||||||||||||||||||||||||
chr10 chr10 127668837 127677125 70M8212N6M                                                                                                                                                                                                                                                                                                                                                                                 AAGAGCAGTGGTTCAGGTTCTCGATTGAAGAAGAAGACCCCAAAATGCACACTTATGGTATCATTTATAC||||||||||||||||||||||||||||||||||||||||||||||||||||||||||||||||||||||||||||||||||||||||||||||||||||||||||||||||||||||||
chr10 chr10 127668841 127677129 66M8212N10M                                                                                                                                                                                                                                                                                                                                                                                    GCAGTGGTTCAGGTTCTCGATTGAAGAAGAAGACCCCAAAATGCACACTTATGGTATCATTTATAC||||||||||||||||||||||||||||||||||||||||||||||||||||||||||||||||||||||||||||||||||||||||||||||||||||||||||||||||||||||||
chr10 chr10 127668847 127677135 60M8212N16M                                                                                                                                                                                                                                                                                                                                                                                          GTTCAGGTTCTCGATTGAAGAAGAAGACCCCAAAATGCACACTTATGGTATCATTTATAC||||||||||||||||||||||||||||||||||||||||||||||||||||||||||||||||||||||||||||||||||||||||||||||||||||||||||||||||||||||||
chr10 chr10 127668850 127668926 76M                                                                                                                                                                                                                                                                                                                                                                                                     CAGGTTCTCGATTGAAGAAGAAGACCCCAAAATGCACACTTATGGTATCATTTATACGTAGGTGCAGTGTTGAATT
chr10 chr10 127668853 127668929 76M                                                                                                                                                                                                                                                                                                                                                                                                        GTTCTCGATTGAAGAAGAAGACCCCAAAATGCACACTTATGGTATCATTTATACGTAGGTGCAGTGTTGAATTGCT
chr10 chr10 127668856 127677144 51M8212N25M                                                                                                                                                                                                                                                                                                                                                                                                   CTCGATTGAAGAAGAAGACCCCAAAATGCACACTTATGGTATCATTTATAC||||||||||||||||||||||||||||||||||||||||||||||||||||||||||||||||||||||||||||||||||||||||||||||||||||||||||||||||||||||||
chr10 chr10 127668860 127677148 47M8212N29M                                                                                                                                                                                                                                                                                                                                                                                                       ATTGAAGAAGAAGACCCCAAAATGCACACTTATGGTATCATTTATAC||||||||||||||||||||||||||||||||||||||||||||||||||||||||||||||||||||||||||||||||||||||||||||||||||||||||||||||||||||||||
chr10 chr10 127668864 127668940 76M                                                                                                                                                                                                                                                                                                                                                                                                                   AAGAAGAAGACCCCAAAATGCACACTTATGGTATCATTTATACGTAGGTGCAGTGTTGAATTGCTTGCCACTTTGC
chr10 chr10 127668867 127668943 76M                                                                                                                                                                                                                                                                                                                                                                                                                      AAGAAGACCCCAAAATGCACACTTATGGTATCATTTATACGTAGGTGCAGTGTTGAATTGCTTGCCACTTTGCCTT
chr10 chr10 127668870 127677158 37M8212N39M                                                                                                                                                                                                                                                                                                                                                                                                                 AAGACCCCAAAATGCACACTTATGGTATCTTTTATAC||||||||||||||||||||||||||||||||||||||||||||||||||||||||||||||||||||||||||||||||||||||||||||||||||||||||||||||||||||||||
chr10 chr10 127668873 127677161 34M8212N42M                                                                                                                                                                                                                                                                                                                                                                                                                    ACCCCAAAATGCACACTTATGGTATCATTTATAC||||||||||||||||||||||||||||||||||||||||||||||||||||||||||||||||||||||||||||||||||||||||||||||||||||||||||||||||||||||||
chr10 chr10 127668876 127677164 31M8212N45M                                                                                                                                                                                                                                                                                                                                                                                                                       CCAAAATGCACACTTATGGTATCATTTATAC||||||||||||||||||||||||||||||||||||||||||||||||||||||||||||||||||||||||||||||||||||||||||||||||||||||||||||||||||||||||
chr10 chr10 127668879 127668955 76M                                                                                                                                                                                                                                                                                                                                                                                                                                  AAATGCACACTTATGGTATCATTTATACGTAGGTGCAGTGTTGAATTGCTTGCCACTTTGCCTTACTTCCTGATTA
chr10 chr10 127668883 127677171 24M8212N52M                                                                                                                                                                                                                                                                                                                                                                                                                              GCACACTTATGGTATCATTTATAC||||||||||||||||||||||||||||||||||||||||||||||||||||||||||||||||||||||||||||||||||||||||||||||||||||||||||||||||||||||||
chr10 chr10 127668887 127668963 76M                                                                                                                                                                                                                                                                                                                                                                                                                                          ACTTATGGTATCATTTATACGTAGGTGCAGTGTTGAATTGCTTGCCACTTTGCCTTACTTCCTGATTAGCTGGAAT 
    22 pairs 
 24:0
49:7
54:13
60:8
54:21
116:17
101:40
62:80
70:80
66:98
75:136
80:164
54:8392
80:8388
29:8442
82:8394
98:8411
72:8442
69:8449
79:8451
98:8502
122:9180 
     TCGA-02-2483-01A chr10-chr10 126234809 127677119 ff 
 
19 23 17 0 56 55
GAGCAGGGTAGGAGGGTTGTTGGCACCTGCTGTGGCCGGGGCCGTGCCAG GGGATATGCAACGAAGCATGTTGTTGAAGGTCTGGAACCAAGGACGCTGT
44455666667779999999999999999999999999999999999999 99999999999999999999999999999999999999999655444331
LHPP intron6(126205840-126301830) FANK1 exon3(127677118-127677243)
 
 blast search - human genome  
 left 
 >ref|NT_030059.13| Homo sapiens chromosome 10 genomic contig, GRCh37.p9 Primary 
Assembly
 gb|GL000099.1| Homo sapiens chromosome 10 genomic contig, GRCh37 reference primary 
assembly
Length=79420533

 Score = 93.5 bits (50),  Expect = 5e-17
 Identities = 50/50 (100%), Gaps = 0/50 (0%)
 Strand=Plus/Plus

Query  1         GAGCAGGGTAGGAGGGTTGTTGGCACCTGCTGTGGCCGGGGCCGTGCCAG  50
                 ||||||||||||||||||||||||||||||||||||||||||||||||||
Sbjct  77039225  GAGCAGGGTAGGAGGGTTGTTGGCACCTGCTGTGGCCGGGGCCGTGCCAG  77039274


>ref|NW_001838010.1| Homo sapiens chromosome 10 genomic contig, alternate assembly 
HuRef SCAF_1103279188229, whole genome shotgun sequence
 gb|DS486216.1| Homo sapiens SCAF_1103279188229 genomic scaffold, whole genome 
shotgun sequence
Length=2644411

 Score = 93.5 bits (50),  Expect = 5e-17
 Identities = 50/50 (100%), Gaps = 0/50 (0%)
 Strand=Plus/Plus

Query  1       GAGCAGGGTAGGAGGGTTGTTGGCACCTGCTGTGGCCGGGGCCGTGCCAG  50
               ||||||||||||||||||||||||||||||||||||||||||||||||||
Sbjct  242402  GAGCAGGGTAGGAGGGTTGTTGGCACCTGCTGTGGCCGGGGCCGTGCCAG  242451


>ref|NC_000010.10| Homo sapiens chromosome 10, GRCh37.p9 Primary Assembly
 gb|CM000672.1| Homo sapiens chromosome 10, GRCh37 primary reference assembly
Length=135534747

 Score = 93.5 bits (50),  Expect = 5e-17
 Identities = 50/50 (100%), Gaps = 0/50 (0%)
 Strand=Plus/Plus

Query  1          GAGCAGGGTAGGAGGGTTGTTGGCACCTGCTGTGGCCGGGGCCGTGCCAG  50
                  ||||||||||||||||||||||||||||||||||||||||||||||||||
Sbjct  126234761  GAGCAGGGTAGGAGGGTTGTTGGCACCTGCTGTGGCCGGGGCCGTGCCAG  126234810


>ref|AC_000142.1| Homo sapiens chromosome 10, alternate assembly HuRef, whole genome 
shotgun sequence
 gb|CM000471.1| Homo sapiens chromosome 10, whole genome shotgun sequence
Length=128985118

 Score = 93.5 bits (50),  Expect = 5e-17
 Identities = 50/50 (100%), Gaps = 0/50 (0%)
 Strand=Plus/Plus

Query  1          GAGCAGGGTAGGAGGGTTGTTGGCACCTGCTGTGGCCGGGGCCGTGCCAG  50
                  ||||||||||||||||||||||||||||||||||||||||||||||||||
Sbjct  119921500  GAGCAGGGTAGGAGGGTTGTTGGCACCTGCTGTGGCCGGGGCCGTGCCAG  119921549


>gb|CM000261.1| Homo sapiens chromosome 10, whole genome shotgun sequence
Length=129189614

 Score = 93.5 bits (50),  Expect = 5e-17
 Identities = 50/50 (100%), Gaps = 0/50 (0%)
 Strand=Plus/Plus

Query  1          GAGCAGGGTAGGAGGGTTGTTGGCACCTGCTGTGGCCGGGGCCGTGCCAG  50
                  ||||||||||||||||||||||||||||||||||||||||||||||||||
Sbjct  120008663  GAGCAGGGTAGGAGGGTTGTTGGCACCTGCTGTGGCCGGGGCCGTGCCAG  120008712


Lambda     K      H
    1.33    0.621     1.12 

Gapped
Lambda     K      H
    1.28    0.460    0.850 

Effective search space used: 681780354108
 
 right 
 >ref|NT_030059.13| Homo sapiens chromosome 10 genomic contig, GRCh37.p9 Primary 
Assembly
 gb|GL000099.1| Homo sapiens chromosome 10 genomic contig, GRCh37 reference primary 
assembly
Length=79420533

 Score = 93.5 bits (50),  Expect = 5e-17
 Identities = 50/50 (100%), Gaps = 0/50 (0%)
 Strand=Plus/Plus

Query  1         GGGATATGCAACGAAGCATGTTGTTGAAGGTCTGGAACCAAGGACGCTGT  50
                 ||||||||||||||||||||||||||||||||||||||||||||||||||
Sbjct  78481584  GGGATATGCAACGAAGCATGTTGTTGAAGGTCTGGAACCAAGGACGCTGT  78481633


>ref|NW_001838010.1| Homo sapiens chromosome 10 genomic contig, alternate assembly 
HuRef SCAF_1103279188229, whole genome shotgun sequence
 gb|DS486216.1| Homo sapiens SCAF_1103279188229 genomic scaffold, whole genome 
shotgun sequence
Length=2644411

 Score = 93.5 bits (50),  Expect = 5e-17
 Identities = 50/50 (100%), Gaps = 0/50 (0%)
 Strand=Plus/Plus

Query  1        GGGATATGCAACGAAGCATGTTGTTGAAGGTCTGGAACCAAGGACGCTGT  50
                ||||||||||||||||||||||||||||||||||||||||||||||||||
Sbjct  1687190  GGGATATGCAACGAAGCATGTTGTTGAAGGTCTGGAACCAAGGACGCTGT  1687239


>ref|NC_000010.10| Homo sapiens chromosome 10, GRCh37.p9 Primary Assembly
 gb|CM000672.1| Homo sapiens chromosome 10, GRCh37 primary reference assembly
Length=135534747

 Score = 93.5 bits (50),  Expect = 5e-17
 Identities = 50/50 (100%), Gaps = 0/50 (0%)
 Strand=Plus/Plus

Query  1          GGGATATGCAACGAAGCATGTTGTTGAAGGTCTGGAACCAAGGACGCTGT  50
                  ||||||||||||||||||||||||||||||||||||||||||||||||||
Sbjct  127677120  GGGATATGCAACGAAGCATGTTGTTGAAGGTCTGGAACCAAGGACGCTGT  127677169


>ref|AC_000142.1| Homo sapiens chromosome 10, alternate assembly HuRef, whole genome 
shotgun sequence
 gb|CM000471.1| Homo sapiens chromosome 10, whole genome shotgun sequence
Length=128985118

 Score = 93.5 bits (50),  Expect = 5e-17
 Identities = 50/50 (100%), Gaps = 0/50 (0%)
 Strand=Plus/Plus

Query  1          GGGATATGCAACGAAGCATGTTGTTGAAGGTCTGGAACCAAGGACGCTGT  50
                  ||||||||||||||||||||||||||||||||||||||||||||||||||
Sbjct  121366288  GGGATATGCAACGAAGCATGTTGTTGAAGGTCTGGAACCAAGGACGCTGT  121366337


>gb|CM000261.1| Homo sapiens chromosome 10, whole genome shotgun sequence
Length=129189614

 Score = 93.5 bits (50),  Expect = 5e-17
 Identities = 50/50 (100%), Gaps = 0/50 (0%)
 Strand=Plus/Plus

Query  1          GGGATATGCAACGAAGCATGTTGTTGAAGGTCTGGAACCAAGGACGCTGT  50
                  ||||||||||||||||||||||||||||||||||||||||||||||||||
Sbjct  121452548  GGGATATGCAACGAAGCATGTTGTTGAAGGTCTGGAACCAAGGACGCTGT  121452597


Lambda     K      H
    1.33    0.621     1.12 

Gapped
Lambda     K      H
    1.28    0.460    0.850 

Effective search space used: 681780354108
 
 blast search - nt  
 left 
 
 
 right 
 >ref|XM_003816282.1| PREDICTED: Pan paniscus fibronectin type III and ankyrin repeat 
domains 1 (FANK1), mRNA
Length=1404

 Score = 93.5 bits (50),  Expect = 6e-17
 Identities = 50/50 (100%), Gaps = 0/50 (0%)
 Strand=Plus/Plus

Query  1    GGGATATGCAACGAAGCATGTTGTTGAAGGTCTGGAACCAAGGACGCTGT  50
            ||||||||||||||||||||||||||||||||||||||||||||||||||
Sbjct  299  GGGATATGCAACGAAGCATGTTGTTGAAGGTCTGGAACCAAGGACGCTGT  348


>ref|XM_003312821.1| PREDICTED: Pan troglodytes fibronectin type III and ankyrin repeat 
domains 1, transcript variant 2 (FANK1), mRNA
Length=1348

 Score = 93.5 bits (50),  Expect = 6e-17
 Identities = 50/50 (100%), Gaps = 0/50 (0%)
 Strand=Plus/Plus

Query  1    GGGATATGCAACGAAGCATGTTGTTGAAGGTCTGGAACCAAGGACGCTGT  50
            ||||||||||||||||||||||||||||||||||||||||||||||||||
Sbjct  268  GGGATATGCAACGAAGCATGTTGTTGAAGGTCTGGAACCAAGGACGCTGT  317


>ref|XM_003312820.1| PREDICTED: Pan troglodytes fibronectin type III and ankyrin repeat 
domains 1, transcript variant 1 (FANK1), mRNA
Length=1292

 Score = 93.5 bits (50),  Expect = 6e-17
 Identities = 50/50 (100%), Gaps = 0/50 (0%)
 Strand=Plus/Plus

Query  1    GGGATATGCAACGAAGCATGTTGTTGAAGGTCTGGAACCAAGGACGCTGT  50
            ||||||||||||||||||||||||||||||||||||||||||||||||||
Sbjct  290  GGGATATGCAACGAAGCATGTTGTTGAAGGTCTGGAACCAAGGACGCTGT  339


>ref|XM_001087857.2| PREDICTED: Macaca mulatta fibronectin type III and ankyrin repeat 
domains 1, transcript variant 6 (FANK1), mRNA
Length=1172

 Score = 93.5 bits (50),  Expect = 6e-17
 Identities = 50/50 (100%), Gaps = 0/50 (0%)
 Strand=Plus/Plus

Query  1    GGGATATGCAACGAAGCATGTTGTTGAAGGTCTGGAACCAAGGACGCTGT  50
            ||||||||||||||||||||||||||||||||||||||||||||||||||
Sbjct  296  GGGATATGCAACGAAGCATGTTGTTGAAGGTCTGGAACCAAGGACGCTGT  345


>ref|XM_001087611.2| PREDICTED: Macaca mulatta fibronectin type III and ankyrin repeat 
domains 1, transcript variant 4 (FANK1), mRNA
Length=1297

 Score = 93.5 bits (50),  Expect = 6e-17
 Identities = 50/50 (100%), Gaps = 0/50 (0%)
 Strand=Plus/Plus

Query  1    GGGATATGCAACGAAGCATGTTGTTGAAGGTCTGGAACCAAGGACGCTGT  50
            ||||||||||||||||||||||||||||||||||||||||||||||||||
Sbjct  296  GGGATATGCAACGAAGCATGTTGTTGAAGGTCTGGAACCAAGGACGCTGT  345


>ref|XM_001086789.2| PREDICTED: Macaca mulatta fibronectin type III and ankyrin repeat 
domains 1, transcript variant 1 (FANK1), mRNA
Length=1353

 Score = 93.5 bits (50),  Expect = 6e-17
 Identities = 50/50 (100%), Gaps = 0/50 (0%)
 Strand=Plus/Plus

Query  1    GGGATATGCAACGAAGCATGTTGTTGAAGGTCTGGAACCAAGGACGCTGT  50
            ||||||||||||||||||||||||||||||||||||||||||||||||||
Sbjct  274  GGGATATGCAACGAAGCATGTTGTTGAAGGTCTGGAACCAAGGACGCTGT  323


>dbj|AK304407.1| Homo sapiens cDNA FLJ52987 complete cds, highly similar to Fibronectin 
type 3 and ankyrin repeat domains protein 1
Length=1283

 Score = 93.5 bits (50),  Expect = 6e-17
 Identities = 50/50 (100%), Gaps = 0/50 (0%)
 Strand=Plus/Plus

Query  1    GGGATATGCAACGAAGCATGTTGTTGAAGGTCTGGAACCAAGGACGCTGT  50
            ||||||||||||||||||||||||||||||||||||||||||||||||||
Sbjct  347  GGGATATGCAACGAAGCATGTTGTTGAAGGTCTGGAACCAAGGACGCTGT  396


>ref|NM_145235.3| Homo sapiens fibronectin type III and ankyrin repeat domains 
1 (FANK1), mRNA
Length=1395

 Score = 93.5 bits (50),  Expect = 6e-17
 Identities = 50/50 (100%), Gaps = 0/50 (0%)
 Strand=Plus/Plus

Query  1    GGGATATGCAACGAAGCATGTTGTTGAAGGTCTGGAACCAAGGACGCTGT  50
            ||||||||||||||||||||||||||||||||||||||||||||||||||
Sbjct  296  GGGATATGCAACGAAGCATGTTGTTGAAGGTCTGGAACCAAGGACGCTGT  345


>gb|AY251163.1| Homo sapiens HSD13 mRNA, complete cds
Length=1220

 Score = 93.5 bits (50),  Expect = 6e-17
 Identities = 50/50 (100%), Gaps = 0/50 (0%)
 Strand=Plus/Plus

Query  1    GGGATATGCAACGAAGCATGTTGTTGAAGGTCTGGAACCAAGGACGCTGT  50
            ||||||||||||||||||||||||||||||||||||||||||||||||||
Sbjct  196  GGGATATGCAACGAAGCATGTTGTTGAAGGTCTGGAACCAAGGACGCTGT  245


>gb|AY358154.1| Homo sapiens clone DNA176837 CG3104 hlg (UNQ6504) mRNA, complete 
cds
Length=1373

 Score = 93.5 bits (50),  Expect = 6e-17
 Identities = 50/50 (100%), Gaps = 0/50 (0%)
 Strand=Plus/Plus

Query  1    GGGATATGCAACGAAGCATGTTGTTGAAGGTCTGGAACCAAGGACGCTGT  50
            ||||||||||||||||||||||||||||||||||||||||||||||||||
Sbjct  274  GGGATATGCAACGAAGCATGTTGTTGAAGGTCTGGAACCAAGGACGCTGT  323


>gb|BC024189.2| Homo sapiens fibronectin type III and ankyrin repeat domains 
1, mRNA (cDNA clone MGC:26734 IMAGE:4826296), complete cds
Length=1395

 Score = 93.5 bits (50),  Expect = 6e-17
 Identities = 50/50 (100%), Gaps = 0/50 (0%)
 Strand=Plus/Plus

Query  1    GGGATATGCAACGAAGCATGTTGTTGAAGGTCTGGAACCAAGGACGCTGT  50
            ||||||||||||||||||||||||||||||||||||||||||||||||||
Sbjct  296  GGGATATGCAACGAAGCATGTTGTTGAAGGTCTGGAACCAAGGACGCTGT  345


>dbj|AB179439.1| Macaca fascicularis testis cDNA clone: QtsA-20030, similar to 
human fibronectin type 3 and ankyrin repeat domains 1(FANK1), 
mRNA, RefSeq: NM_145235.2
Length=1201

 Score = 93.5 bits (50),  Expect = 6e-17
 Identities = 50/50 (100%), Gaps = 0/50 (0%)
 Strand=Plus/Plus

Query  1    GGGATATGCAACGAAGCATGTTGTTGAAGGTCTGGAACCAAGGACGCTGT  50
            ||||||||||||||||||||||||||||||||||||||||||||||||||
Sbjct  280  GGGATATGCAACGAAGCATGTTGTTGAAGGTCTGGAACCAAGGACGCTGT  329


>gb|AC063963.11| Homo sapiens chromosome 10 clone RP11-295J3, complete sequence
Length=179104

 Score = 93.5 bits (50),  Expect = 6e-17
 Identities = 50/50 (100%), Gaps = 0/50 (0%)
 Strand=Plus/Plus

Query  1      GGGATATGCAACGAAGCATGTTGTTGAAGGTCTGGAACCAAGGACGCTGT  50
              ||||||||||||||||||||||||||||||||||||||||||||||||||
Sbjct  69522  GGGATATGCAACGAAGCATGTTGTTGAAGGTCTGGAACCAAGGACGCTGT  69571


>emb|CR627249.1| Homo sapiens mRNA; cDNA DKFZp781G1012 (from clone DKFZp781G1012)
Length=8002

 Score = 93.5 bits (50),  Expect = 6e-17
 Identities = 50/50 (100%), Gaps = 0/50 (0%)
 Strand=Plus/Plus

Query  1    GGGATATGCAACGAAGCATGTTGTTGAAGGTCTGGAACCAAGGACGCTGT  50
            ||||||||||||||||||||||||||||||||||||||||||||||||||
Sbjct  267  GGGATATGCAACGAAGCATGTTGTTGAAGGTCTGGAACCAAGGACGCTGT  316


>ref|XM_002821261.2| PREDICTED: Pongo abelii fibronectin type III and ankyrin repeat 
domains 1, transcript variant 1 (FANK1), mRNA
Length=1380

 Score = 87.9 bits (47),  Expect = 3e-15
 Identities = 49/50 (98%), Gaps = 0/50 (0%)
 Strand=Plus/Plus

Query  1    GGGATATGCAACGAAGCATGTTGTTGAAGGTCTGGAACCAAGGACGCTGT  50
            |||||| |||||||||||||||||||||||||||||||||||||||||||
Sbjct  300  GGGATACGCAACGAAGCATGTTGTTGAAGGTCTGGAACCAAGGACGCTGT  349


>ref|XM_002821262.2| PREDICTED: Pongo abelii fibronectin type III and ankyrin repeat 
domains 1, transcript variant 2 (FANK1), mRNA
Length=1302

 Score = 87.9 bits (47),  Expect = 3e-15
 Identities = 49/50 (98%), Gaps = 0/50 (0%)
 Strand=Plus/Plus

Query  1    GGGATATGCAACGAAGCATGTTGTTGAAGGTCTGGAACCAAGGACGCTGT  50
            |||||| |||||||||||||||||||||||||||||||||||||||||||
Sbjct  300  GGGATACGCAACGAAGCATGTTGTTGAAGGTCTGGAACCAAGGACGCTGT  349


>ref|XM_003277780.1| PREDICTED: Nomascus leucogenys fibronectin type 3 and ankyrin 
repeat domains protein 1-like (LOC100599088), mRNA
Length=1298

 Score = 87.9 bits (47),  Expect = 3e-15
 Identities = 47/47 (100%), Gaps = 0/47 (0%)
 Strand=Plus/Plus

Query  1    GGGATATGCAACGAAGCATGTTGTTGAAGGTCTGGAACCAAGGACGC  47
            |||||||||||||||||||||||||||||||||||||||||||||||
Sbjct  296  GGGATATGCAACGAAGCATGTTGTTGAAGGTCTGGAACCAAGGACGC  342


Lambda     K      H
    1.33    0.621     1.12 

Gapped
Lambda     K      H
    1.28    0.460    0.850 

Effective search space used: 874911716973
 
    reads 
 chr10 chr10 126234569 126234645 76M                          TGGGATGCTGTCCTCCCTCCCTCCCTCCCTCTGCCAAGCTGGTGGGAAGGCCCTTGCCAAAGCGCACAAGACGCCT
chr10 chr10 126234582 126234658 76M                                       TCCCTCCCTCCCTCCCTCTGCCAAGCTGGTGGGAAGGCCCTTGCCAAAGCGCACAAGACCCCTTCACACAGCAGGG
chr10 chr10 126234585 126234661 76M                                          CTCCCTCCCTCCCTCTGCCAAGCTGGTGGGAAGGCCCTTGCCAAAGCGCACAAGACCCCTTCACACAGCAGGGGCA
chr10 chr10 126234599 126234675 76M                                                        CTGCCAAGCTGGTGGGAAGGCCCTTGCCAAAGCGCACAAGACCCCTTCACACAGCAGAGGCACAAGCTCTTCGAGG
chr10 chr10 126234607 126234683 76M                                                                CTGGTGGGAAGGCCCTTGCCAAAGCGCACAAGACCCCTTCACACAGCAGGGGCACAAGCTCTTCGAGGCAGAGTCG
chr10 chr10 126234610 126234686 76M                                                                   GTGGGAAGGCCCTTGCCAAAGCGCACAAGACCCCTTCACACAGCAGGGGCACAAGCTCTTCGAGGCAGAGTCGCCT
chr10 chr10 126234613 126234689 76M                                                                      GGAAGGCCCTTGCCAAAGCGCACAAGACCCCTTCACACAGCAGGGGCACAAGCTCTTCGAGGCAGAGTCGCCTGCA
chr10 chr10 126234617 126234693 76M                                                                          GGCCCTTGCCAAAGCGCACAAGACCCCTTCACACAGCAGGGGCACAAGCTCTTCGAGGCAGAGTCGCCTGCAGGTG
chr10 chr10 126234620 126234696 76M                                                                             CCTTGCCAAAGCGCACAAGACCCCTTCACACAGCAGGGGCACAAGCTCTTCGAGGCAGAGTCGCCTGCAGGTGGGG
chr10 chr10 126234625 126234701 76M                                                                                  CCAAAGCGCACAAGACCCCTTCACACAGCAGAGGCACAAGCTCTTCGAGGCAGAGTCGCCTGCAGGTGGGGTGGAA
chr10 chr10 126234632 126234708 76M                                                                                         GCACAAGACCCCTTCACACAGCAGGGGCACAAGCTCTTCGAGGCAGAGTCGCCTGCAGGTGGGGTGGAAGGGGTGC
chr10 chr10 126234635 126234711 76M                                                                                            CAAGACCCCTTCACACAGCAGGGGCACAAGCTCTTCGAGGCAGAGTCGCCTGCAGGTGGGGTGGAAGGGGTGCGCC
chr10 chr10 126234638 126234714 76M                                                                                               GACCCCTTCACACAGCAGGGGCACAAGCTCTTCGAGGCAGAGTCGCCTGCAGGTGGGGTGGAAGGGGTGCGCCCCA
chr10 chr10 126234643 126234719 76M                                                                                                    CTTCACACAGCAGGGGCACAAGCTCTTCGAGGCAGAGTCGCCTGCAGGTGGGGTGGAAGGGGTGCGACCCAGACCC
chr10 chr10 126234646 126234722 76M                                                                                                       CACACAGCAGGGGCACAAGCTCTTCGAGGCAGAGTCGCCTGCAGGTGGGGTGGAAGGGGTGCGCCCCAGACCCAAG
chr10 chr10 126234650 126234726 76M                                                                                                           CAGCAGGGGCACAAGCTCTTCGAGGCAGAGTCGCCTGCAGGTGGGGTGGAAGGGGTGCGCCCCAGACCCAAGAATC
chr10 chr10 126234653 126234729 76M                                                                                                              CAGGGGCACAAGCTCTTCGAGGCAGAGTCGCCTGCAGGTGGGGTGGAAGGGGTGCGCCCCAGACCCAAGAATCGCC
chr10 chr10 126234657 126234733 76M                                                                                                                  GGCACAAGCTCTTCGAGGCAGAGTCGCCTGCAGGTGGGGTGGAAGGGGTGCGCCCCAGACCCAAGAATCGCCCGCC
chr10 chr10 126234661 126234737 76M                                                                                                                      CAAGCTCTTCGAGGCAGAGTCGCCTGCAGGTGGGGTGGAAGGGGTGCGCCCCAGACCCAAGAATCGCCCGCCTCTC
chr10 chr10 126234664 126234740 76M                                                                                                                         GCTCTTCGAGGCAGAGTCGCCTGCAGGTGGGGTGGAAGGGGTGCGCCCCAGACCCAAGAATCGCCCGCCTCTCCGA
chr10 chr10 126234667 126234743 76M                                                                                                                            CTTCGAGGCAGAGTCGCCTGCAGGTGGGGTGGAAGGGGTGCGCCCCAGACCCAAGAATCGCCCGCCTCTCCGAGAC
chr10 chr10 126234671 126234747 76M                                                                                                                                GAGGCAGAGTCGCCTGCAGGTGGGGTGGAAGGGGTGCGCCCCAGACCCAAGAATCGCCCGCCTCTCCGAGACCATC
chr10 chr10 126234674 126234750 76M                                                                                                                                   GCAGAGTCGCCTGCAGGTGGGGTGGAAGGGGTGCGCCCCAGACCCAAGAATCGCCCGCCTCTCCGAGACCATCCCT
chr10 chr10 126234679 126234755 76M                                                                                                                                        GTCGCCTGCAGGTGGGGTGGAAGGGGTGCGCCCCAGACCCAAGAATCGCCCGCCTCTCCGAGACCATCCCTGGCTG
chr10 chr10 126234684 126234760 76M                                                                                                                                             CTGCAGGTGGGGGGGAAGGGGTGCGCCCCAGACCCAAGAATCGCCCGCCTCTCCGAGACCATCCCTGGCTGCTGGT
chr10 chr10 126234697 126234773 76M                                                                                                                                                          GGAAGGGGTGCGCCCCAGACCCAAGAATCGCCCGCCTCTCCGAGACCATCCCTGGCTGCTGGTGAGCAGGGTAGGA
chr10 chr10 126234701 126234777 76M                                                                                                                                                              GGGGTGCGCCCCAGACCCAAGAATCGCCCGCCTCTCCAAGACCATCCCTGGCTGCTGGTGAGCAGGGTAGGAGGGT
chr10 chr10 126234704 126234780 76M                                                                                                                                                                 GTGCGCCCCAGACCCAAGAATCGCCCGCCTCTCCGAGACCATCCCTGGCTGCTGGTGAGCAGGGTAGGAGGGTTGT
chr10 chr10 126234708 126234784 76M                                                                                                                                                                     GCCCCAGACCCAAGAATCGCCCGCCTCTCCGAGACCATCCCTGGCTGCTGGTGAGCAGGGTAGGAGGGTTGTTGGC
chr10 chr10 126234711 126234787 76M                                                                                                                                                                        CCAGACCCAAGAATCGCCCGCCTCTCCGAGACCATCCCTGGCTGCTGGTGAGCAGGGGAGGAGGGTTGTTGGCACC
chr10 chr10 126234723 126234799 76M                                                                                                                                                                                    ATCGCCCGCCTCTCCAAGACCATCCCTGGCTGCTGGTGAGCAGGGTAGGAGGGTTGTTGGCACCTGCTGTGGCCGG
chr10 chr10 126234727 126234803 76M                                                                                                                                                                                        CCCGCCTCTCCGAGACCATCCCTGGCTGCTGGTGAGCAGGGTAGGAGGGTTGTTGGCACCTGCTGTGGCCGGGGCC
chr10 chr10 126234731 126234807 76M                                                                                                                                                                                            CCTCTCCGAGACCATCCCTGGCTGCTGGTGAGCAGGGTAGGAGGGTTGTTGGCACCTGCTGTGGCCGGGGCCGTGC
chr10 chr10 126234734 126234810 76M                                                                                                                                                                                               CTCCGAGACCATCCCTGGCTGCTGGTGAGCAGGGTAGGAGGGTTGTTGGCACCTGCTGTGGCCGGGGCCGTGCCAG
chr10 chr10 126234748 127677133 62M127677120F14M                                                                                                                                                                                                CTGGCTGCTGGTGAGCAGGGTAGGAGGGTTGTTGGCACCTGCTGTGGCCGGGGCCGTGCCAG GGGATATGCAACGA
chr10 chr10 126234754 127677139 56M127677120F20M                                                                                                                                                                                                      GCTGGTGAGCAGGGTAGGAGGGTTGTTGGCACCTGCTGTGGCCGGGGCCGTTCCAG GGGATATGCAACGAAGCATG
chr10 chr10 126234755 127677140 55M127677120F21M                                                                                                                                                                                                       CTGGTGAGCAGGGTAGGAGGGTTGTTGGCACCTGCTGTGGCCGGGGCCGTGCCAG GGGATATGCAACGAAGCATGG
chr10 chr10 126234756 127677141 54M127677120F22M                                                                                                                                                                                                        TGGTGAGCAGGGTAGGAGGGTTGTTGGCACCTGCTGTGGCCGGGGCCGTGCCAG GGGATATGCAACGAAGCATGTT
chr10 chr10 126234756 127677141 54M127677120F22M                                                                                                                                                                                                        TGGTGAGCAGGGTAGGAGGGTTGTTGGCACCTGCTGTGGCCGGGGCCGTGCCAG GGGATATGCAACGAAGCATGTT
chr10 chr10 126234763 127677148 47M127677120F29M                                                                                                                                                                                                               CAGGGTAGGAGGGTTGTTGGCACCTGCTGTGGCCGGGGCCGTGCCAG GGGATATGCAACGAAGCATGTTGTTGAAG
chr10 chr10 126234765 127677150 45M127677120F31M                                                                                                                                                                                                                 GGGTAGGAGGGTTGTTGGCACCTGCTGTGGCCGGGGCCGTGCCAG GGGATATGCAACGAAGCATGTTGTTGAAGGT
chr10 chr10 126234770 127677155 40M127677120F36M                                                                                                                                                                                                                      GGAGGGTTGTTGGCACCTGCTGTGGCCGGGGCCGTGCCAG GGGATATGCAACGAAGCATGTTGTTGAAGGTCTGGA
chr10 chr10 126234773 127677158 37M127677120F39M                                                                                                                                                                                                                         GGGTTGTTGGCACCTGCTGTGGCCGGGGCCGTGCCAG GGGATATGCAACGAAGCATGTTGTTGAAGGTCTGGAACC
chr10 chr10 126234773 127677158 37M127677120F39M                                                                                                                                                                                                                         GGGTTGTTGGCACCTGCTGTGGCCGGGGCCGTGCCAG GGGATATGCAACGAAGCATGTTGTTGAAGGTCTGGAACC
chr10 chr10 126234773 127677158 37M127677120F39M                                                                                                                                                                                                                         GGGTTGTTGGCACCTGCTGTGGCCGGGGCCGTGCCAG GGGATATGCAACGAAGCATGTTGTTGAAGGTCTGGAACC
chr10 chr10 126234775 127677160 35M127677120F41M                                                                                                                                                                                                                           GTTGTTGGCACCTGCTGTGGCCGGGGCCGTGCCAG GGGATATGCAACGAAGCATGTTGTTGAAGGTCTGGAACCAA
chr10 chr10 126234775 127677160 35M127677120F41M                                                                                                                                                                                                                           GTTGTTGGCACCTGCTGTGGCCGGGGCCGTGCCAG GGGATATGCAACGAAGCATGTTGTTGAAGGTCTGGAACCAA
chr10 chr10 126234775 127677160 35M127677120F41M                                                                                                                                                                                                                           GTTGTTGGCACCTGCTGTGGCCGGGGCCGTGCCAG GGGATATGCAACGAAGCATGTTGTTGAAGGTCTGGAACCAA
chr10 chr10 126234776 127677161 34M127677120F42M                                                                                                                                                                                                                            TTGTTGGCACCTGCTGTGGCCGGGGCCGTGCCAG GGTATATGCAACGAAGCATGTTGTTGAAGGTCTGGAACCAAG
chr10 chr10 126234778 127677163 32M127677120F44M                                                                                                                                                                                                                              GTTGGCACCTGCTGTGGCCGGGGCCGTGCCAG GGGATATGCAACGAAGCATGTTGTTGAAGGTCTGGAACCAAGGA
chr10 chr10 126234781 127677166 29M127677120F47M                                                                                                                                                                                                                                 GGCACCTGCTGTGGCCGGGGCCGTGCCAG GGGATATGCAACGAAGCATGGTGTTGAAGGGCTGGAACCAAGGACGC
chr10 chr10 126234783 127677168 27M127677120F49M                                                                                                                                                                                                                                   CACCTGCTGTGGCCGGGGCCGTGCCAG GGGATATGCAACGAAGCATGTTGTTGAAGGTCTGGAACCAAGGACGCTG
chr10 chr10 126234783 127677168 27M127677120F49M                                                                                                                                                                                                                                   CACCTGCTGTGGCCGGGGCCGTGCCAG GGGATATGCAACGAAGCATGTTGTTGAAGGTCTGGAACCAAGGACGCTG
chr10 chr10 126234789 127677174 21M127677120F55M                                                                                                                                                                                                                                         CTGTGGCCGGGGCCGTGCCAG GGGATATGCAACGAAGCATGTTGTTGAAGGTCTGGAACCAAGGACGCTGTACAGA
chr10 chr10 126234795 127677180 15M127677120F61M                                                                                                                                                                                                                                               CCGGGGCCGTGCCAG GGGATATGCAACGAAGCATGTTGTTGAAGGTCTGGAACCAAGGACGCTGTACAGATTTCGC
chr10 chr10 126234801 127677186 9M127677120F67M                                                                                                                                                                                                                                                      CCGTGCCAG GGGATATGCAACGAAGCATGTTGTTGAAGGTCTGGAACCAAGGACGCTGTACAGATTTCGCCTGAAG
chr10 chr10 126234801 127677186 9M127677120F67M                                                                                                                                                                                                                                                      CCGTGCCAG GGGATATGCAACGAAGCATGTTGTTGAAGGTCTGGAACCAAGGACGCTGTACAGATTTCGCCTGAAG
chr10 chr10 127677115 127677191 76M                                                                                                                                                                                                                                                                        CCAGGGGATATGCAACGAAGCATGTTGTTGAAGGTCTGGAACCAAGGACGCTGTACAGATTTCGCCTGAAGGTCAC
chr10 chr10 127677118 127677194 76M                                                                                                                                                                                                                                                                           GGGGATATGCAACGAAGCATGTTGTTGAAGGTCTGGAACCAAGGACGCTGTACAGATTTCGCCTGAAGGTCACCAG
chr10 chr10 127677121 127677197 76M                                                                                                                                                                                                                                                                              GATATGCAACGAAGCATGTTGTTGAAGGTCTGGAACCAAGGACGCTGTACAGATTTCGCCTGAAGGTCACCAGCCC
chr10 chr10 127677124 127677200 76M                                                                                                                                                                                                                                                                                 ATGCAACGAAGCATGTTGTTGAAGGTCTGGAACCAAGGACGCTGTACAGATTTCGCCTGAAGGTCACCAGCCCCTC
chr10 chr10 127677127 127677203 76M                                                                                                                                                                                                                                                                                    CAACGAAGCATGTTGTTGAAGGTCTGGAACCAAGGACGCTGTACAGATTTCGCCTGAAGGTCACCAGCCCCTCTGG
chr10 chr10 127677130 127677206 76M                                                                                                                                                                                                                                                                                       CGAAGCATGTTGTTGAAGGTCTGGAACCAAGGACGCTGTACAGATTTCGCCTGAAGGTCACCAGCCCCTCTGGGGA
chr10 chr10 127677133 127677209 76M                                                                                                                                                                                                                                                                                          AGCATGTTGTTGAAGGTCTGGAACCAAGGACGCTGTACAGATTTCGCCTGAAGGTCACCAGCCCCTCTGGGGAGTG
chr10 chr10 127677136 127677212 76M                                                                                                                                                                                                                                                                                             ATGTTGTTGAAGGTCTGGAACCAAGGACGCTGTACAGATTTCGCCTGAAGGTCACCAGCCCCTCTGGGGAGTGTGA
chr10 chr10 127677139 127677215 76M                                                                                                                                                                                                                                                                                                TTGTTGAAGGTCTGGAACCAAGGACGCTGTACAGATTTCGCCTGAAGGTCACCAGCCCCTCTGGGGAGTGTGAGTA
chr10 chr10 127677143 127677219 76M                                                                                                                                                                                                                                                                                                    TGAAGGTCTGGAACCAAGGACGCTGTACAGATTTCGCCTGAAGGTCACCAGCCCCTCTGGGGAGTGTGAGTACAGC
chr10 chr10 127677146 127677222 76M                                                                                                                                                                                                                                                                                                       AGGTCTGGAACCAAGGACGCTGTACAGATTTCGCCTGAAGGTCACCAGCCCCTCTGGGGAGTGTGAGTACAGCCGG
chr10 chr10 127677149 127677225 76M                                                                                                                                                                                                                                                                                                          TCTGGAACCAAGGACGCTGTACAGATTTCGCCTGAAGGTCACCAGCCCCTCTGGGGAGTGTGAGTACAGCCCACTC
chr10 chr10 127677153 127677229 76M                                                                                                                                                                                                                                                                                                              GAACCAAGGACGCTGTACAGATTTCGCCTGAAGGTCACCAGCCCCTCTGGGGAGTGTGAGTACAGCCCACTCGTCT
chr10 chr10 127677157 127677233 76M                                                                                                                                                                                                                                                                                                                  CAAGGACGCTGTACAGATTTCGCCTGAAGGTCACCAGCCCCTCTGGGGAGTGTGAGTACAGCCCACTCGTCTCAGT
chr10 chr10 127677160 127677236 76M                                                                                                                                                                                                                                                                                                                     GGACGCTGTACAGATTTCGCCTGAAGGTCACCAGCCCCTCTGGGGAGTGTGAGTACAGCCCACTCGTCTCAGTGTC
chr10 chr10 127677163 127677239 76M                                                                                                                                                                                                                                                                                                                        CGCTGTACAGATTTCGCCTGAAGGTCTCCAGCCCCTCTGGGGAGTGTGAGTACAGCCCACTCGTCTCAGTGTCTAC
chr10 chr10 127677167 127677243 76M                                                                                                                                                                                                                                                                                                                            GTACAGATTTCGCCTGAAGGTCACCAGCCCCTCTGGGGAGTGTGAGTACAGCCCACTCGTCTCAGTGTCTACAACC
chr10 chr10 127677170 127677246 76M                                                                                                                                                                                                                                                                                                                               CAGATTTCGCCTGAAGGTCACCAGCCCCTCTGGGGAGTGTGAGTACAGCCCACTCGTCTCAGTGTCTACAACCAGA
chr10 chr10 127677173 127683990 71M6741N5M                                                                                                                                                                                                                                                                                                                           ATTTCGCCTGAAGGTCACCAGCCCCTCTGGGGAGCGTGAGTACAGCCCACTCGTCTCAGTGTCTACAACCA|||||||||||||||||||||||||||||||||||||||||||||||||||||||||||||||||||||||||||||||||||||||||||||||||||||||||||||||||||||||||||||||||||||||||||||||||||||||||||||||||||||||||||||
chr10 chr10 127677176 127683993 68M6741N8M                                                                                                                                                                                                                                                                                                                              TCGCCTGAAGGTCACCAGCCCCTCTGGGGAGTGTGAGTACAGCCCACTCGTCTCAGTGTCTACAACCA|||||||||||||||||||||||||||||||||||||||||||||||||||||||||||||||||||||||||||||||||||||||||||||||||||||||||||||||||||||||||||||||||||||||||||||||||||||||||||||||||||||||||||||
chr10 chr10 127677179 127678009 65M754N11M                                                                                                                                                                                                                                                                                                                                 CCTGAAGGTCACCAGCCCCTCTGGGGAGTGTGAGTACAGCCCACTCGTCTCAGTGTCTACAACCA|||||||||||||||||||||||||||||||||||||||||||||||||||||||||||||||||||||||||||||||||||||||||||||||||||||||||||||||||||||||||||||||||||||||||||||||||||||||||||||||||||||||||||||
chr10 chr10 127677184 127684001 60M6741N16M                                                                                                                                                                                                                                                                                                                                     AGGTCACCAGCCCCTCTGGGGAGTGTGAGTACAGCCCACTCGTCTCAGTGTCTACAACCA|||||||||||||||||||||||||||||||||||||||||||||||||||||||||||||||||||||||||||||||||||||||||||||||||||||||||||||||||||||||||||||||||||||||||||||||||||||||||||||||||||||||||||||
chr10 chr10 127677188 127684005 56M6741N20M                                                                                                                                                                                                                                                                                                                                         CACCAGCCCCTCTGGGGAGTGTGAGTACAGCCCACTCGTCTCAGTGTCTACAACCA|||||||||||||||||||||||||||||||||||||||||||||||||||||||||||||||||||||||||||||||||||||||||||||||||||||||||||||||||||||||||||||||||||||||||||||||||||||||||||||||||||||||||||||
chr10 chr10 127677191 127684008 53M6741N23M                                                                                                                                                                                                                                                                                                                                            CAGCCCCTCTGGGGAGTGTGAGTACAGCCCACTCGTCTCAGTGTCTACAACCA|||||||||||||||||||||||||||||||||||||||||||||||||||||||||||||||||||||||||||||||||||||||||||||||||||||||||||||||||||||||||||||||||||||||||||||||||||||||||||||||||||||||||||||
chr10 chr10 127677194 127684011 50M6741N26M                                                                                                                                                                                                                                                                                                                                               CCCCTCTGGGGAGTGTGAGTACAGCCCACTCGTCTCAGTGTCTACAACCA|||||||||||||||||||||||||||||||||||||||||||||||||||||||||||||||||||||||||||||||||||||||||||||||||||||||||||||||||||||||||||||||||||||||||||||||||||||||||||||||||||||||||||||
chr10 chr10 127677197 127684014 47M6741N29M                                                                                                                                                                                                                                                                                                                                                  CTCTGGGGAGTGTGAGTACAGCCCACTCGTCTCAGTGTCTACAACCA|||||||||||||||||||||||||||||||||||||||||||||||||||||||||||||||||||||||||||||||||||||||||||||||||||||||||||||||||||||||||||||||||||||||||||||||||||||||||||||||||||||||||||||
chr10 chr10 127677200 127684017 44M6741N32M                                                                                                                                                                                                                                                                                                                                                     TGGGGAGTGTGAGTACAGCCCACTCGTCTCAGTGTCTACAACCA|||||||||||||||||||||||||||||||||||||||||||||||||||||||||||||||||||||||||||||||||||||||||||||||||||||||||||||||||||||||||||||||||||||||||||||||||||||||||||||||||||||||||||||
chr10 chr10 127677203 127684020 41M6741N35M                                                                                                                                                                                                                                                                                                                                                        GGAGTGTGAGTACAGCCCACTCGTCTCAGTGTCTACAACCA|||||||||||||||||||||||||||||||||||||||||||||||||||||||||||||||||||||||||||||||||||||||||||||||||||||||||||||||||||||||||||||||||||||||||||||||||||||||||||||||||||||||||||||
chr10 chr10 127677206 127684023 38M6741N38M                                                                                                                                                                                                                                                                                                                                                           GTGTGAGTACAGCCCCCTCGTCTCAGTGTCTACAACCA|||||||||||||||||||||||||||||||||||||||||||||||||||||||||||||||||||||||||||||||||||||||||||||||||||||||||||||||||||||||||||||||||||||||||||||||||||||||||||||||||||||||||||||
chr10 chr10 127677209 127678039 35M754N41M                                                                                                                                                                                                                                                                                                                                                               TGAGTACAGCCCACTCGTCTCAGTGTCTACAACCA|||||||||||||||||||||||||||||||||||||||||||||||||||||||||||||||||||||||||||||||||||||||||||||||||||||||||||||||||||||||||||||||||||||||||||||||||||||||||||||||||||||||||||||
chr10 chr10 127677212 127684029 32M6741N44M                                                                                                                                                                                                                                                                                                                                                                 GTACAGCCCACTCGTCTCAGTGTCTACAACCA|||||||||||||||||||||||||||||||||||||||||||||||||||||||||||||||||||||||||||||||||||||||||||||||||||||||||||||||||||||||||||||||||||||||||||||||||||||||||||||||||||||||||||||
chr10 chr10 127677215 127684032 29M6741N47M                                                                                                                                                                                                                                                                                                                                                                    CAGCCCACTCGTCTCAGTGTCTACAACCA|||||||||||||||||||||||||||||||||||||||||||||||||||||||||||||||||||||||||||||||||||||||||||||||||||||||||||||||||||||||||||||||||||||||||||||||||||||||||||||||||||||||||||||
chr10 chr10 127677218 127684035 26M6741N50M                                                                                                                                                                                                                                                                                                                                                                       CCCACTCGTCTCAGTGTCTACAACCA|||||||||||||||||||||||||||||||||||||||||||||||||||||||||||||||||||||||||||||||||||||||||||||||||||||||||||||||||||||||||||||||||||||||||||||||||||||||||||||||||||||||||||||
chr10 chr10 127677221 127684038 23M6741N53M                                                                                                                                                                                                                                                                                                                                                                          ACTCGTCTCAGTGTCTACAACCA|||||||||||||||||||||||||||||||||||||||||||||||||||||||||||||||||||||||||||||||||||||||||||||||||||||||||||||||||||||||||||||||||||||||||||||||||||||||||||||||||||||||||||||
chr10 chr10 127677224 127684041 20M6741N56M                                                                                                                                                                                                                                                                                                                                                                             CGTCTCAGTGTCTACAACCA|||||||||||||||||||||||||||||||||||||||||||||||||||||||||||||||||||||||||||||||||||||||||||||||||||||||||||||||||||||||||||||||||||||||||||||||||||||||||||||||||||||||||||||
chr10 chr10 127677227 127684044 17M6741N59M                                                                                                                                                                                                                                                                                                                                                                                CTCAGTGTCTACAACCA|||||||||||||||||||||||||||||||||||||||||||||||||||||||||||||||||||||||||||||||||||||||||||||||||||||||||||||||||||||||||||||||||||||||||||||||||||||||||||||||||||||||||||||
chr10 chr10 127677231 127684048 13M6741N63M                                                                                                                                                                                                                                                                                                                                                                                    GTGTCTACAACCA|||||||||||||||||||||||||||||||||||||||||||||||||||||||||||||||||||||||||||||||||||||||||||||||||||||||||||||||||||||||||||||||||||||||||||||||||||||||||||||||||||||||||||||
chr10 chr10 127677234 127684051 10M6741N66M                                                                                                                                                                                                                                                                                                                                                                                       TCTACAACCA|||||||||||||||||||||||||||||||||||||||||||||||||||||||||||||||||||||||||||||||||||||||||||||||||||||||||||||||||||||||||||||||||||||||||||||||||||||||||||||||||||||||||||||
chr10 chr10 127677238 127684055 6M6741N70M                                                                                                                                                                                                                                                                                                                                                                                            CAACCA|||||||||||||||||||||||||||||||||||||||||||||||||||||||||||||||||||||||||||||||||||||||||||||||||||||||||||||||||||||||||||||||||||||||||||||||||||||||||||||||||||||||||||||
chr10 chr10 127677241 127677921 3M604N73M                                                                                                                                                                                                                                                                                                                                                                                                CCA||||||||||||||||||||||||||||||||||||||||||||||||||||||||||||||||||||||||||||||||||||||||||||||||||||||||||||||||||||||||||||||||||||||||||||||||||||||||||||||||||||||||||||| 
    23 pairs 
 54:2
29:52
80:-2
82:4
98:21
72:52
69:59
79:61
98:112
122:790
54:6868
80:-8226
75:-8254
66:-8292
62:-8310
70:-8310
24:-8390
54:-8369
54:-8377
49:-8383
60:-8382
101:-8350
116:-8373 
     TCGA-02-2485-01A chr12-chr12 57660513 58339410 rf 
 
10 5 9 0 55 1374
TCAGTCTCCCTCTCCCTGCAGTCCACCACAGATGCCACAGCAGTACTCAG ATCCATATGTCAAACTTCTGCTTGATGCTATGAAACACTCAGGTTGGTAA
22222233333333345555666788999999999999999999999999 99999999999999999999988888888888777777777655554444
R3HDM2 exon6(57660512-57660613) XRCC6BP1 exon2(58339409-58339455)
 
 blast search - human genome  
 left 
 >ref|NT_029419.12| Homo sapiens chromosome 12 genomic contig, GRCh37.p9 Primary 
Assembly
 gb|GL000107.1| Homo sapiens chromosome 12 genomic contig, GRCh37 reference primary 
assembly
Length=71516776

 Score = 93.5 bits (50),  Expect = 5e-17
 Identities = 50/50 (100%), Gaps = 0/50 (0%)
 Strand=Plus/Minus

Query  1         TCAGTCTCCCTCTCCCTGCAGTCCACCACAGATGCCACAGCAGTACTCAG  50
                 ||||||||||||||||||||||||||||||||||||||||||||||||||
Sbjct  19803869  TCAGTCTCCCTCTCCCTGCAGTCCACCACAGATGCCACAGCAGTACTCAG  19803820


>ref|NW_001838060.2| Homo sapiens chromosome 12 genomic contig, alternate assembly 
HuRef SCAF_1103279188147, whole genome shotgun sequence
 gb|DS486061.1| Homo sapiens SCAF_1103279188147 genomic scaffold, whole genome 
shotgun sequence
Length=18942049

 Score = 93.5 bits (50),  Expect = 5e-17
 Identities = 50/50 (100%), Gaps = 0/50 (0%)
 Strand=Plus/Plus

Query  1         TCAGTCTCCCTCTCCCTGCAGTCCACCACAGATGCCACAGCAGTACTCAG  50
                 ||||||||||||||||||||||||||||||||||||||||||||||||||
Sbjct  18867142  TCAGTCTCCCTCTCCCTGCAGTCCACCACAGATGCCACAGCAGTACTCAG  18867191


>ref|NC_000012.11| Homo sapiens chromosome 12, GRCh37.p9 Primary Assembly
 gb|CM000674.1| Homo sapiens chromosome 12, GRCh37 primary reference assembly
Length=133851895

 Score = 93.5 bits (50),  Expect = 5e-17
 Identities = 50/50 (100%), Gaps = 0/50 (0%)
 Strand=Plus/Minus

Query  1         TCAGTCTCCCTCTCCCTGCAGTCCACCACAGATGCCACAGCAGTACTCAG  50
                 ||||||||||||||||||||||||||||||||||||||||||||||||||
Sbjct  57660563  TCAGTCTCCCTCTCCCTGCAGTCCACCACAGATGCCACAGCAGTACTCAG  57660514


>ref|AC_000144.1| Homo sapiens chromosome 12, alternate assembly HuRef, whole genome 
shotgun sequence
 gb|CM000473.1| Homo sapiens chromosome 12, whole genome shotgun sequence
Length=130617856

 Score = 93.5 bits (50),  Expect = 5e-17
 Identities = 50/50 (100%), Gaps = 0/50 (0%)
 Strand=Plus/Minus

Query  1         TCAGTCTCCCTCTCCCTGCAGTCCACCACAGATGCCACAGCAGTACTCAG  50
                 ||||||||||||||||||||||||||||||||||||||||||||||||||
Sbjct  54699435  TCAGTCTCCCTCTCCCTGCAGTCCACCACAGATGCCACAGCAGTACTCAG  54699386


>gb|CM000263.1| Homo sapiens chromosome 12, whole genome shotgun sequence
Length=133516800

 Score = 93.5 bits (50),  Expect = 5e-17
 Identities = 50/50 (100%), Gaps = 0/50 (0%)
 Strand=Plus/Minus

Query  1         TCAGTCTCCCTCTCCCTGCAGTCCACCACAGATGCCACAGCAGTACTCAG  50
                 ||||||||||||||||||||||||||||||||||||||||||||||||||
Sbjct  57314758  TCAGTCTCCCTCTCCCTGCAGTCCACCACAGATGCCACAGCAGTACTCAG  57314709


Lambda     K      H
    1.33    0.621     1.12 

Gapped
Lambda     K      H
    1.28    0.460    0.850 

Effective search space used: 681780354108
 
 right 
 >ref|NT_029419.12| Homo sapiens chromosome 12 genomic contig, GRCh37.p9 Primary 
Assembly
 gb|GL000107.1| Homo sapiens chromosome 12 genomic contig, GRCh37 reference primary 
assembly
Length=71516776

 Score = 93.5 bits (50),  Expect = 5e-17
 Identities = 50/50 (100%), Gaps = 0/50 (0%)
 Strand=Plus/Plus

Query  1         ATCCATATGTCAAACTTCTGCTTGATGCTATGAAACACTCAGGTTGGTAA  50
                 ||||||||||||||||||||||||||||||||||||||||||||||||||
Sbjct  20482717  ATCCATATGTCAAACTTCTGCTTGATGCTATGAAACACTCAGGTTGGTAA  20482766


>ref|NW_001838060.2| Homo sapiens chromosome 12 genomic contig, alternate assembly 
HuRef SCAF_1103279188147, whole genome shotgun sequence
 gb|DS486061.1| Homo sapiens SCAF_1103279188147 genomic scaffold, whole genome 
shotgun sequence
Length=18942049

 Score = 93.5 bits (50),  Expect = 5e-17
 Identities = 50/50 (100%), Gaps = 0/50 (0%)
 Strand=Plus/Minus

Query  1         ATCCATATGTCAAACTTCTGCTTGATGCTATGAAACACTCAGGTTGGTAA  50
                 ||||||||||||||||||||||||||||||||||||||||||||||||||
Sbjct  18190843  ATCCATATGTCAAACTTCTGCTTGATGCTATGAAACACTCAGGTTGGTAA  18190794


>ref|NC_000012.11| Homo sapiens chromosome 12, GRCh37.p9 Primary Assembly
 gb|CM000674.1| Homo sapiens chromosome 12, GRCh37 primary reference assembly
Length=133851895

 Score = 93.5 bits (50),  Expect = 5e-17
 Identities = 50/50 (100%), Gaps = 0/50 (0%)
 Strand=Plus/Plus

Query  1         ATCCATATGTCAAACTTCTGCTTGATGCTATGAAACACTCAGGTTGGTAA  50
                 ||||||||||||||||||||||||||||||||||||||||||||||||||
Sbjct  58339411  ATCCATATGTCAAACTTCTGCTTGATGCTATGAAACACTCAGGTTGGTAA  58339460


>ref|AC_000144.1| Homo sapiens chromosome 12, alternate assembly HuRef, whole genome 
shotgun sequence
 gb|CM000473.1| Homo sapiens chromosome 12, whole genome shotgun sequence
Length=130617856

 Score = 93.5 bits (50),  Expect = 5e-17
 Identities = 50/50 (100%), Gaps = 0/50 (0%)
 Strand=Plus/Plus

Query  1         ATCCATATGTCAAACTTCTGCTTGATGCTATGAAACACTCAGGTTGGTAA  50
                 ||||||||||||||||||||||||||||||||||||||||||||||||||
Sbjct  55375734  ATCCATATGTCAAACTTCTGCTTGATGCTATGAAACACTCAGGTTGGTAA  55375783


>gb|CM000263.1| Homo sapiens chromosome 12, whole genome shotgun sequence
Length=133516800

 Score = 93.5 bits (50),  Expect = 5e-17
 Identities = 50/50 (100%), Gaps = 0/50 (0%)
 Strand=Plus/Plus

Query  1         ATCCATATGTCAAACTTCTGCTTGATGCTATGAAACACTCAGGTTGGTAA  50
                 ||||||||||||||||||||||||||||||||||||||||||||||||||
Sbjct  57998016  ATCCATATGTCAAACTTCTGCTTGATGCTATGAAACACTCAGGTTGGTAA  57998065


Lambda     K      H
    1.33    0.621     1.12 

Gapped
Lambda     K      H
    1.28    0.460    0.850 

Effective search space used: 681780354108
 
 blast search - nt  
 left 
 >ref|XM_003824871.1| PREDICTED: Pan paniscus R3H domain containing 2, transcript variant 
5 (R3HDM2), mRNA
Length=3264

 Score = 93.5 bits (50),  Expect = 6e-17
 Identities = 50/50 (100%), Gaps = 0/50 (0%)
 Strand=Plus/Plus

Query  1     TCAGTCTCCCTCTCCCTGCAGTCCACCACAGATGCCACAGCAGTACTCAG  50
             ||||||||||||||||||||||||||||||||||||||||||||||||||
Sbjct  2132  TCAGTCTCCCTCTCCCTGCAGTCCACCACAGATGCCACAGCAGTACTCAG  2181


>ref|XM_003824870.1| PREDICTED: Pan paniscus R3H domain containing 2, transcript variant 
4 (R3HDM2), mRNA
Length=3303

 Score = 93.5 bits (50),  Expect = 6e-17
 Identities = 50/50 (100%), Gaps = 0/50 (0%)
 Strand=Plus/Plus

Query  1     TCAGTCTCCCTCTCCCTGCAGTCCACCACAGATGCCACAGCAGTACTCAG  50
             ||||||||||||||||||||||||||||||||||||||||||||||||||
Sbjct  2171  TCAGTCTCCCTCTCCCTGCAGTCCACCACAGATGCCACAGCAGTACTCAG  2220


>ref|XM_003824869.1| PREDICTED: Pan paniscus R3H domain containing 2, transcript variant 
3 (R3HDM2), mRNA
Length=3399

 Score = 93.5 bits (50),  Expect = 6e-17
 Identities = 50/50 (100%), Gaps = 0/50 (0%)
 Strand=Plus/Plus

Query  1     TCAGTCTCCCTCTCCCTGCAGTCCACCACAGATGCCACAGCAGTACTCAG  50
             ||||||||||||||||||||||||||||||||||||||||||||||||||
Sbjct  2267  TCAGTCTCCCTCTCCCTGCAGTCCACCACAGATGCCACAGCAGTACTCAG  2316


>ref|XM_003824868.1| PREDICTED: Pan paniscus R3H domain containing 2, transcript variant 
2 (R3HDM2), mRNA
Length=3437

 Score = 93.5 bits (50),  Expect = 6e-17
 Identities = 50/50 (100%), Gaps = 0/50 (0%)
 Strand=Plus/Plus

Query  1     TCAGTCTCCCTCTCCCTGCAGTCCACCACAGATGCCACAGCAGTACTCAG  50
             ||||||||||||||||||||||||||||||||||||||||||||||||||
Sbjct  2305  TCAGTCTCCCTCTCCCTGCAGTCCACCACAGATGCCACAGCAGTACTCAG  2354


>ref|XM_003824867.1| PREDICTED: Pan paniscus R3H domain containing 2, transcript variant 
1 (R3HDM2), mRNA
Length=3201

 Score = 93.5 bits (50),  Expect = 6e-17
 Identities = 50/50 (100%), Gaps = 0/50 (0%)
 Strand=Plus/Plus

Query  1     TCAGTCTCCCTCTCCCTGCAGTCCACCACAGATGCCACAGCAGTACTCAG  50
             ||||||||||||||||||||||||||||||||||||||||||||||||||
Sbjct  2069  TCAGTCTCCCTCTCCCTGCAGTCCACCACAGATGCCACAGCAGTACTCAG  2118


>ref|XM_003476155.1| PREDICTED: Cavia porcellus R3H domain containing 2, transcript 
variant 5 (R3hdm2), mRNA
Length=3024

 Score = 93.5 bits (50),  Expect = 6e-17
 Identities = 50/50 (100%), Gaps = 0/50 (0%)
 Strand=Plus/Plus

Query  1     TCAGTCTCCCTCTCCCTGCAGTCCACCACAGATGCCACAGCAGTACTCAG  50
             ||||||||||||||||||||||||||||||||||||||||||||||||||
Sbjct  2133  TCAGTCTCCCTCTCCCTGCAGTCCACCACAGATGCCACAGCAGTACTCAG  2182


>ref|XM_003476154.1| PREDICTED: Cavia porcellus R3H domain containing 2, transcript 
variant 4 (R3hdm2), mRNA
Length=2985

 Score = 93.5 bits (50),  Expect = 6e-17
 Identities = 50/50 (100%), Gaps = 0/50 (0%)
 Strand=Plus/Plus

Query  1     TCAGTCTCCCTCTCCCTGCAGTCCACCACAGATGCCACAGCAGTACTCAG  50
             ||||||||||||||||||||||||||||||||||||||||||||||||||
Sbjct  2094  TCAGTCTCCCTCTCCCTGCAGTCCACCACAGATGCCACAGCAGTACTCAG  2143


>ref|XM_003476153.1| PREDICTED: Cavia porcellus R3H domain containing 2, transcript 
variant 3 (R3hdm2), mRNA
Length=2964

 Score = 93.5 bits (50),  Expect = 6e-17
 Identities = 50/50 (100%), Gaps = 0/50 (0%)
 Strand=Plus/Plus

Query  1     TCAGTCTCCCTCTCCCTGCAGTCCACCACAGATGCCACAGCAGTACTCAG  50
             ||||||||||||||||||||||||||||||||||||||||||||||||||
Sbjct  2073  TCAGTCTCCCTCTCCCTGCAGTCCACCACAGATGCCACAGCAGTACTCAG  2122


>ref|XM_003476152.1| PREDICTED: Cavia porcellus R3H domain containing 2, transcript 
variant 2 (R3hdm2), mRNA
Length=2922

 Score = 93.5 bits (50),  Expect = 6e-17
 Identities = 50/50 (100%), Gaps = 0/50 (0%)
 Strand=Plus/Plus

Query  1     TCAGTCTCCCTCTCCCTGCAGTCCACCACAGATGCCACAGCAGTACTCAG  50
             ||||||||||||||||||||||||||||||||||||||||||||||||||
Sbjct  2031  TCAGTCTCCCTCTCCCTGCAGTCCACCACAGATGCCACAGCAGTACTCAG  2080


>ref|XM_003476151.1| PREDICTED: Cavia porcellus R3H domain containing 2, transcript 
variant 1 (R3hdm2), mRNA
Length=3120

 Score = 93.5 bits (50),  Expect = 6e-17
 Identities = 50/50 (100%), Gaps = 0/50 (0%)
 Strand=Plus/Plus

Query  1     TCAGTCTCCCTCTCCCTGCAGTCCACCACAGATGCCACAGCAGTACTCAG  50
             ||||||||||||||||||||||||||||||||||||||||||||||||||
Sbjct  2229  TCAGTCTCCCTCTCCCTGCAGTCCACCACAGATGCCACAGCAGTACTCAG  2278


>ref|XM_003365274.1| PREDICTED: Equus caballus R3H domain containing 2, transcript 
variant 2 (R3HDM2), mRNA
Length=3013

 Score = 93.5 bits (50),  Expect = 6e-17
 Identities = 50/50 (100%), Gaps = 0/50 (0%)
 Strand=Plus/Plus

Query  1     TCAGTCTCCCTCTCCCTGCAGTCCACCACAGATGCCACAGCAGTACTCAG  50
             ||||||||||||||||||||||||||||||||||||||||||||||||||
Sbjct  2076  TCAGTCTCCCTCTCCCTGCAGTCCACCACAGATGCCACAGCAGTACTCAG  2125


>ref|XM_001488652.3| PREDICTED: Equus caballus R3H domain containing 2, transcript 
variant 1 (R3HDM2), mRNA
Length=2971

 Score = 93.5 bits (50),  Expect = 6e-17
 Identities = 50/50 (100%), Gaps = 0/50 (0%)
 Strand=Plus/Plus

Query  1     TCAGTCTCCCTCTCCCTGCAGTCCACCACAGATGCCACAGCAGTACTCAG  50
             ||||||||||||||||||||||||||||||||||||||||||||||||||
Sbjct  2034  TCAGTCTCCCTCTCCCTGCAGTCCACCACAGATGCCACAGCAGTACTCAG  2083


>ref|XM_003313565.1| PREDICTED: Pan troglodytes r3H domain-containing protein 2-like 
(LOC452011), mRNA
Length=3918

 Score = 93.5 bits (50),  Expect = 6e-17
 Identities = 50/50 (100%), Gaps = 0/50 (0%)
 Strand=Plus/Plus

Query  1     TCAGTCTCCCTCTCCCTGCAGTCCACCACAGATGCCACAGCAGTACTCAG  50
             ||||||||||||||||||||||||||||||||||||||||||||||||||
Sbjct  2013  TCAGTCTCCCTCTCCCTGCAGTCCACCACAGATGCCACAGCAGTACTCAG  2062


>ref|XM_003252774.1| PREDICTED: Nomascus leucogenys R3H domain containing 2 (R3HDM2), 
mRNA
Length=4332

 Score = 93.5 bits (50),  Expect = 6e-17
 Identities = 50/50 (100%), Gaps = 0/50 (0%)
 Strand=Plus/Plus

Query  1     TCAGTCTCCCTCTCCCTGCAGTCCACCACAGATGCCACAGCAGTACTCAG  50
             ||||||||||||||||||||||||||||||||||||||||||||||||||
Sbjct  2429  TCAGTCTCCCTCTCCCTGCAGTCCACCACAGATGCCACAGCAGTACTCAG  2478


>ref|XM_002916009.1| PREDICTED: Ailuropoda melanoleuca R3H domain containing 2, transcript 
variant 2 (R3HDM2), mRNA
Length=3619

 Score = 93.5 bits (50),  Expect = 6e-17
 Identities = 50/50 (100%), Gaps = 0/50 (0%)
 Strand=Plus/Plus

Query  1     TCAGTCTCCCTCTCCCTGCAGTCCACCACAGATGCCACAGCAGTACTCAG  50
             ||||||||||||||||||||||||||||||||||||||||||||||||||
Sbjct  2043  TCAGTCTCCCTCTCCCTGCAGTCCACCACAGATGCCACAGCAGTACTCAG  2092


>ref|XM_002916008.1| PREDICTED: Ailuropoda melanoleuca R3H domain containing 2, transcript 
variant 1 (R3HDM2), mRNA
Length=3661

 Score = 93.5 bits (50),  Expect = 6e-17
 Identities = 50/50 (100%), Gaps = 0/50 (0%)
 Strand=Plus/Plus

Query  1     TCAGTCTCCCTCTCCCTGCAGTCCACCACAGATGCCACAGCAGTACTCAG  50
             ||||||||||||||||||||||||||||||||||||||||||||||||||
Sbjct  2085  TCAGTCTCCCTCTCCCTGCAGTCCACCACAGATGCCACAGCAGTACTCAG  2134


>ref|XM_001100250.2| PREDICTED: Macaca mulatta r3H domain-containing protein 2-like 
(LOC711448), mRNA
Length=3937

 Score = 93.5 bits (50),  Expect = 6e-17
 Identities = 50/50 (100%), Gaps = 0/50 (0%)
 Strand=Plus/Plus

Query  1     TCAGTCTCCCTCTCCCTGCAGTCCACCACAGATGCCACAGCAGTACTCAG  50
             ||||||||||||||||||||||||||||||||||||||||||||||||||
Sbjct  2029  TCAGTCTCCCTCTCCCTGCAGTCCACCACAGATGCCACAGCAGTACTCAG  2078


>gb|BC143605.1| Homo sapiens R3H domain containing 2, mRNA (cDNA clone MGC:177135 
IMAGE:9052118), complete cds
Length=2123

 Score = 93.5 bits (50),  Expect = 6e-17
 Identities = 50/50 (100%), Gaps = 0/50 (0%)
 Strand=Plus/Plus

Query  1     TCAGTCTCCCTCTCCCTGCAGTCCACCACAGATGCCACAGCAGTACTCAG  50
             ||||||||||||||||||||||||||||||||||||||||||||||||||
Sbjct  1184  TCAGTCTCCCTCTCCCTGCAGTCCACCACAGATGCCACAGCAGTACTCAG  1233


>dbj|AK293394.1| Homo sapiens cDNA FLJ60419 complete cds, highly similar to R3H 
domain-containing protein 2
Length=2107

 Score = 93.5 bits (50),  Expect = 6e-17
 Identities = 50/50 (100%), Gaps = 0/50 (0%)
 Strand=Plus/Plus

Query  1     TCAGTCTCCCTCTCCCTGCAGTCCACCACAGATGCCACAGCAGTACTCAG  50
             ||||||||||||||||||||||||||||||||||||||||||||||||||
Sbjct  1327  TCAGTCTCCCTCTCCCTGCAGTCCACCACAGATGCCACAGCAGTACTCAG  1376


>dbj|AB384552.1| Synthetic construct DNA, clone: pF1KA1002, Homo sapiens R3HDM2 
gene for R3H domain-containing protein 2, complete cds, without 
stop codon, in Flexi system
Length=2537

 Score = 93.5 bits (50),  Expect = 6e-17
 Identities = 50/50 (100%), Gaps = 0/50 (0%)
 Strand=Plus/Plus

Query  1     TCAGTCTCCCTCTCCCTGCAGTCCACCACAGATGCCACAGCAGTACTCAG  50
             ||||||||||||||||||||||||||||||||||||||||||||||||||
Sbjct  1641  TCAGTCTCCCTCTCCCTGCAGTCCACCACAGATGCCACAGCAGTACTCAG  1690


>ref|NM_014925.3| Homo sapiens R3H domain containing 2 (R3HDM2), mRNA
Length=3974

 Score = 93.5 bits (50),  Expect = 6e-17
 Identities = 50/50 (100%), Gaps = 0/50 (0%)
 Strand=Plus/Plus

Query  1     TCAGTCTCCCTCTCCCTGCAGTCCACCACAGATGCCACAGCAGTACTCAG  50
             ||||||||||||||||||||||||||||||||||||||||||||||||||
Sbjct  2075  TCAGTCTCCCTCTCCCTGCAGTCCACCACAGATGCCACAGCAGTACTCAG  2124


>ref|NM_001078034.1| Bos taurus R3H domain containing 2 (R3HDM2), mRNA
 gb|BC126608.1| Bos taurus R3H domain containing 2, mRNA (cDNA clone MGC:142849 
IMAGE:8217364), complete cds
Length=3771

 Score = 93.5 bits (50),  Expect = 6e-17
 Identities = 50/50 (100%), Gaps = 0/50 (0%)
 Strand=Plus/Plus

Query  1     TCAGTCTCCCTCTCCCTGCAGTCCACCACAGATGCCACAGCAGTACTCAG  50
             ||||||||||||||||||||||||||||||||||||||||||||||||||
Sbjct  2145  TCAGTCTCCCTCTCCCTGCAGTCCACCACAGATGCCACAGCAGTACTCAG  2194


>gb|BC112226.1| Homo sapiens R3H domain containing 2, mRNA (cDNA clone MGC:138431 
IMAGE:8327694), complete cds
Length=2014

 Score = 93.5 bits (50),  Expect = 6e-17
 Identities = 50/50 (100%), Gaps = 0/50 (0%)
 Strand=Plus/Plus

Query  1     TCAGTCTCCCTCTCCCTGCAGTCCACCACAGATGCCACAGCAGTACTCAG  50
             ||||||||||||||||||||||||||||||||||||||||||||||||||
Sbjct  1082  TCAGTCTCCCTCTCCCTGCAGTCCACCACAGATGCCACAGCAGTACTCAG  1131


>gb|BC104995.1| Homo sapiens R3H domain containing 2, mRNA (cDNA clone MGC:132655 
IMAGE:8143998), complete cds
Length=2020

 Score = 93.5 bits (50),  Expect = 6e-17
 Identities = 50/50 (100%), Gaps = 0/50 (0%)
 Strand=Plus/Plus

Query  1     TCAGTCTCCCTCTCCCTGCAGTCCACCACAGATGCCACAGCAGTACTCAG  50
             ||||||||||||||||||||||||||||||||||||||||||||||||||
Sbjct  1082  TCAGTCTCCCTCTCCCTGCAGTCCACCACAGATGCCACAGCAGTACTCAG  1131


>gb|BC041857.1| Homo sapiens R3H domain containing 2, mRNA (cDNA clone MGC:43641 
IMAGE:5270511), complete cds
Length=3800

 Score = 93.5 bits (50),  Expect = 6e-17
 Identities = 50/50 (100%), Gaps = 0/50 (0%)
 Strand=Plus/Plus

Query  1     TCAGTCTCCCTCTCCCTGCAGTCCACCACAGATGCCACAGCAGTACTCAG  50
             ||||||||||||||||||||||||||||||||||||||||||||||||||
Sbjct  1887  TCAGTCTCCCTCTCCCTGCAGTCCACCACAGATGCCACAGCAGTACTCAG  1936


>gb|AC137834.9| Homo sapiens 12 BAC RP11-123K3 (Roswell Park Cancer Institute 
Human BAC Library) complete sequence
Length=109523

 Score = 93.5 bits (50),  Expect = 6e-17
 Identities = 50/50 (100%), Gaps = 0/50 (0%)
 Strand=Plus/Plus

Query  1      TCAGTCTCCCTCTCCCTGCAGTCCACCACAGATGCCACAGCAGTACTCAG  50
              ||||||||||||||||||||||||||||||||||||||||||||||||||
Sbjct  25629  TCAGTCTCCCTCTCCCTGCAGTCCACCACAGATGCCACAGCAGTACTCAG  25678


>dbj|AB023219.1| Homo sapiens mRNA for KIAA1002 protein, partial cds
Length=4331

 Score = 93.5 bits (50),  Expect = 6e-17
 Identities = 50/50 (100%), Gaps = 0/50 (0%)
 Strand=Plus/Plus

Query  1     TCAGTCTCCCTCTCCCTGCAGTCCACCACAGATGCCACAGCAGTACTCAG  50
             ||||||||||||||||||||||||||||||||||||||||||||||||||
Sbjct  2432  TCAGTCTCCCTCTCCCTGCAGTCCACCACAGATGCCACAGCAGTACTCAG  2481


>ref|XM_003790560.1| PREDICTED: Otolemur garnettii R3H domain containing 2, transcript 
variant 4 (R3HDM2), mRNA
Length=3088

 Score = 87.9 bits (47),  Expect = 3e-15
 Identities = 49/50 (98%), Gaps = 0/50 (0%)
 Strand=Plus/Plus

Query  1     TCAGTCTCCCTCTCCCTGCAGTCCACCACAGATGCCACAGCAGTACTCAG  50
             ||||||||||||||| ||||||||||||||||||||||||||||||||||
Sbjct  2123  TCAGTCTCCCTCTCCTTGCAGTCCACCACAGATGCCACAGCAGTACTCAG  2172


>ref|XM_003790559.1| PREDICTED: Otolemur garnettii R3H domain containing 2, transcript 
variant 3 (R3HDM2), mRNA
Length=3127

 Score = 87.9 bits (47),  Expect = 3e-15
 Identities = 49/50 (98%), Gaps = 0/50 (0%)
 Strand=Plus/Plus

Query  1     TCAGTCTCCCTCTCCCTGCAGTCCACCACAGATGCCACAGCAGTACTCAG  50
             ||||||||||||||| ||||||||||||||||||||||||||||||||||
Sbjct  2162  TCAGTCTCCCTCTCCTTGCAGTCCACCACAGATGCCACAGCAGTACTCAG  2211


>ref|XM_003790558.1| PREDICTED: Otolemur garnettii R3H domain containing 2, transcript 
variant 2 (R3HDM2), mRNA
Length=3223

 Score = 87.9 bits (47),  Expect = 3e-15
 Identities = 49/50 (98%), Gaps = 0/50 (0%)
 Strand=Plus/Plus

Query  1     TCAGTCTCCCTCTCCCTGCAGTCCACCACAGATGCCACAGCAGTACTCAG  50
             ||||||||||||||| ||||||||||||||||||||||||||||||||||
Sbjct  2258  TCAGTCTCCCTCTCCTTGCAGTCCACCACAGATGCCACAGCAGTACTCAG  2307


>ref|XM_003790557.1| PREDICTED: Otolemur garnettii R3H domain containing 2, transcript 
variant 1 (R3HDM2), mRNA
Length=3025

 Score = 87.9 bits (47),  Expect = 3e-15
 Identities = 49/50 (98%), Gaps = 0/50 (0%)
 Strand=Plus/Plus

Query  1     TCAGTCTCCCTCTCCCTGCAGTCCACCACAGATGCCACAGCAGTACTCAG  50
             ||||||||||||||| ||||||||||||||||||||||||||||||||||
Sbjct  2060  TCAGTCTCCCTCTCCTTGCAGTCCACCACAGATGCCACAGCAGTACTCAG  2109


>ref|XR_091221.2| PREDICTED: Callithrix jacchus R3H domain-containing protein 2-like 
(LOC100404885), misc_RNA
Length=2877

 Score = 87.9 bits (47),  Expect = 3e-15
 Identities = 49/50 (98%), Gaps = 0/50 (0%)
 Strand=Plus/Plus

Query  1     TCAGTCTCCCTCTCCCTGCAGTCCACCACAGATGCCACAGCAGTACTCAG  50
             |||||||||||||||||| |||||||||||||||||||||||||||||||
Sbjct  1986  TCAGTCTCCCTCTCCCTGTAGTCCACCACAGATGCCACAGCAGTACTCAG  2035


>ref|XM_003733788.1| PREDICTED: Callithrix jacchus R3H domain containing 2, transcript 
variant 3 (R3HDM2), mRNA
Length=3089

 Score = 87.9 bits (47),  Expect = 3e-15
 Identities = 49/50 (98%), Gaps = 0/50 (0%)
 Strand=Plus/Plus

Query  1     TCAGTCTCCCTCTCCCTGCAGTCCACCACAGATGCCACAGCAGTACTCAG  50
             |||||||||||||||||| |||||||||||||||||||||||||||||||
Sbjct  2116  TCAGTCTCCCTCTCCCTGTAGTCCACCACAGATGCCACAGCAGTACTCAG  2165


>ref|XM_002752642.2| PREDICTED: Callithrix jacchus R3H domain containing 2, transcript 
variant 1 (R3HDM2), mRNA
Length=3128

 Score = 87.9 bits (47),  Expect = 3e-15
 Identities = 49/50 (98%), Gaps = 0/50 (0%)
 Strand=Plus/Plus

Query  1     TCAGTCTCCCTCTCCCTGCAGTCCACCACAGATGCCACAGCAGTACTCAG  50
             |||||||||||||||||| |||||||||||||||||||||||||||||||
Sbjct  2155  TCAGTCTCCCTCTCCCTGTAGTCCACCACAGATGCCACAGCAGTACTCAG  2204


>ref|XM_003733787.1| PREDICTED: Callithrix jacchus R3H domain containing 2, transcript 
variant 2 (R3HDM2), mRNA
Length=3026

 Score = 87.9 bits (47),  Expect = 3e-15
 Identities = 49/50 (98%), Gaps = 0/50 (0%)
 Strand=Plus/Plus

Query  1     TCAGTCTCCCTCTCCCTGCAGTCCACCACAGATGCCACAGCAGTACTCAG  50
             |||||||||||||||||| |||||||||||||||||||||||||||||||
Sbjct  2053  TCAGTCTCCCTCTCCCTGTAGTCCACCACAGATGCCACAGCAGTACTCAG  2102


>ref|XM_003355486.2| PREDICTED: Sus scrofa R3H domain containing 2 (R3HDM2), partial 
mRNA
Length=2590

 Score = 87.9 bits (47),  Expect = 3e-15
 Identities = 49/50 (98%), Gaps = 0/50 (0%)
 Strand=Plus/Plus

Query  1     TCAGTCTCCCTCTCCCTGCAGTCCACCACAGATGCCACAGCAGTACTCAG  50
             ||||||||||||||||||||||||||||||||||||||| ||||||||||
Sbjct  1007  TCAGTCTCCCTCTCCCTGCAGTCCACCACAGATGCCACAACAGTACTCAG  1056


>ref|XM_853562.2| PREDICTED: Canis lupus familiaris R3H domain containing 2, transcript 
variant 6 (R3HDM2), mRNA
Length=3625

 Score = 87.9 bits (47),  Expect = 3e-15
 Identities = 49/50 (98%), Gaps = 0/50 (0%)
 Strand=Plus/Plus

Query  1     TCAGTCTCCCTCTCCCTGCAGTCCACCACAGATGCCACAGCAGTACTCAG  50
             ||||||||||||||||||||| ||||||||||||||||||||||||||||
Sbjct  2043  TCAGTCTCCCTCTCCCTGCAGCCCACCACAGATGCCACAGCAGTACTCAG  2092


>dbj|AK240306.1| Sus scrofa mRNA, clone:UTR010052F09, expressed in uterus
Length=2035

 Score = 87.9 bits (47),  Expect = 3e-15
 Identities = 49/50 (98%), Gaps = 0/50 (0%)
 Strand=Plus/Plus

Query  1    TCAGTCTCCCTCTCCCTGCAGTCCACCACAGATGCCACAGCAGTACTCAG  50
            ||||||||||||||||||||||||||||||||||||||| ||||||||||
Sbjct  435  TCAGTCTCCCTCTCCCTGCAGTCCACCACAGATGCCACAACAGTACTCAG  484


>ref|NM_001132581.1| Pongo abelii R3H domain containing 2 (R3HDM2), mRNA
 emb|CR859659.1| Pongo abelii mRNA; cDNA DKFZp469E2113 (from clone DKFZp469E2113)
Length=4076

 Score = 87.9 bits (47),  Expect = 3e-15
 Identities = 49/50 (98%), Gaps = 0/50 (0%)
 Strand=Plus/Plus

Query  1     TCAGTCTCCCTCTCCCTGCAGTCCACCACAGATGCCACAGCAGTACTCAG  50
             |||||||||||||||||||||||||||||||||||||||||||| |||||
Sbjct  2191  TCAGTCTCCCTCTCCCTGCAGTCCACCACAGATGCCACAGCAGTGCTCAG  2240


>gb|AY609747.1| Sus scrofa clone Clu_29164.scr.msk.p1.Contig1, mRNA sequence
Length=1790

 Score = 87.9 bits (47),  Expect = 3e-15
 Identities = 49/50 (98%), Gaps = 0/50 (0%)
 Strand=Plus/Plus

Query  1    TCAGTCTCCCTCTCCCTGCAGTCCACCACAGATGCCACAGCAGTACTCAG  50
            ||||||||||||||||||||||||||||||||||||||| ||||||||||
Sbjct  190  TCAGTCTCCCTCTCCCTGCAGTCCACCACAGATGCCACAACAGTACTCAG  239


>ref|XM_002720907.1| PREDICTED: Oryctolagus cuniculus R3H domain containing 2, transcript 
variant 3 (LOC100357871), mRNA
Length=3012

 Score = 82.4 bits (44),  Expect = 1e-13
 Identities = 46/47 (97%), Gaps = 0/47 (0%)
 Strand=Plus/Plus

Query  4     GTCTCCCTCTCCCTGCAGTCCACCACAGATGCCACAGCAGTACTCAG  50
             |||||||||||||||||| ||||||||||||||||||||||||||||
Sbjct  2124  GTCTCCCTCTCCCTGCAGCCCACCACAGATGCCACAGCAGTACTCAG  2170


>ref|XM_002720906.1| PREDICTED: Oryctolagus cuniculus R3H domain containing 2, transcript 
variant 2 (LOC100357871), mRNA
Length=3051

 Score = 82.4 bits (44),  Expect = 1e-13
 Identities = 46/47 (97%), Gaps = 0/47 (0%)
 Strand=Plus/Plus

Query  4     GTCTCCCTCTCCCTGCAGTCCACCACAGATGCCACAGCAGTACTCAG  50
             |||||||||||||||||| ||||||||||||||||||||||||||||
Sbjct  2163  GTCTCCCTCTCCCTGCAGCCCACCACAGATGCCACAGCAGTACTCAG  2209


>ref|XM_002720905.1| PREDICTED: Oryctolagus cuniculus R3H domain containing 2, transcript 
variant 1 (LOC100357871), mRNA
Length=2949

 Score = 82.4 bits (44),  Expect = 1e-13
 Identities = 46/47 (97%), Gaps = 0/47 (0%)
 Strand=Plus/Plus

Query  4     GTCTCCCTCTCCCTGCAGTCCACCACAGATGCCACAGCAGTACTCAG  50
             |||||||||||||||||| ||||||||||||||||||||||||||||
Sbjct  2061  GTCTCCCTCTCCCTGCAGCCCACCACAGATGCCACAGCAGTACTCAG  2107


Lambda     K      H
    1.33    0.621     1.12 

Gapped
Lambda     K      H
    1.28    0.460    0.850 

Effective search space used: 874911716973
 
 right 
 >ref|XM_003824981.1| PREDICTED: Pan paniscus XRCC6 binding protein 1 (XRCC6BP1), mRNA
Length=1561

 Score = 86.1 bits (46),  Expect = 1e-14
 Identities = 46/46 (100%), Gaps = 0/46 (0%)
 Strand=Plus/Plus

Query  1    ATCCATATGTCAAACTTCTGCTTGATGCTATGAAACACTCAGGTTG  46
            ||||||||||||||||||||||||||||||||||||||||||||||
Sbjct  629  ATCCATATGTCAAACTTCTGCTTGATGCTATGAAACACTCAGGTTG  674


>ref|XM_003780310.1| PREDICTED: Pongo abelii XRCC6 binding protein 1 (XRCC6BP1), mRNA
Length=378

 Score = 86.1 bits (46),  Expect = 1e-14
 Identities = 46/46 (100%), Gaps = 0/46 (0%)
 Strand=Plus/Plus

Query  1    ATCCATATGTCAAACTTCTGCTTGATGCTATGAAACACTCAGGTTG  46
            ||||||||||||||||||||||||||||||||||||||||||||||
Sbjct  215  ATCCATATGTCAAACTTCTGCTTGATGCTATGAAACACTCAGGTTG  260


>ref|XM_002807125.2| PREDICTED: Callithrix jacchus mitochondrial inner membrane protease 
ATP23 homolog (LOC100390044), mRNA
Length=1523

 Score = 86.1 bits (46),  Expect = 1e-14
 Identities = 46/46 (100%), Gaps = 0/46 (0%)
 Strand=Plus/Plus

Query  1    ATCCATATGTCAAACTTCTGCTTGATGCTATGAAACACTCAGGTTG  46
            ||||||||||||||||||||||||||||||||||||||||||||||
Sbjct  581  ATCCATATGTCAAACTTCTGCTTGATGCTATGAAACACTCAGGTTG  626


>ref|XM_509179.3| PREDICTED: Pan troglodytes XRCC6 binding protein 1 (XRCC6BP1), 
mRNA
Length=1280

 Score = 86.1 bits (46),  Expect = 1e-14
 Identities = 46/46 (100%), Gaps = 0/46 (0%)
 Strand=Plus/Plus

Query  1    ATCCATATGTCAAACTTCTGCTTGATGCTATGAAACACTCAGGTTG  46
            ||||||||||||||||||||||||||||||||||||||||||||||
Sbjct  348  ATCCATATGTCAAACTTCTGCTTGATGCTATGAAACACTCAGGTTG  393


>gb|BC115381.1| Homo sapiens XRCC6 binding protein 1, mRNA (cDNA clone MGC:134817 
IMAGE:40036852), complete cds
Length=1098

 Score = 86.1 bits (46),  Expect = 1e-14
 Identities = 46/46 (100%), Gaps = 0/46 (0%)
 Strand=Plus/Plus

Query  1    ATCCATATGTCAAACTTCTGCTTGATGCTATGAAACACTCAGGTTG  46
            ||||||||||||||||||||||||||||||||||||||||||||||
Sbjct  227  ATCCATATGTCAAACTTCTGCTTGATGCTATGAAACACTCAGGTTG  272


>gb|BC115382.1| Homo sapiens XRCC6 binding protein 1, mRNA (cDNA clone MGC:134818 
IMAGE:40036859), complete cds
Length=1097

 Score = 86.1 bits (46),  Expect = 1e-14
 Identities = 46/46 (100%), Gaps = 0/46 (0%)
 Strand=Plus/Plus

Query  1    ATCCATATGTCAAACTTCTGCTTGATGCTATGAAACACTCAGGTTG  46
            ||||||||||||||||||||||||||||||||||||||||||||||
Sbjct  226  ATCCATATGTCAAACTTCTGCTTGATGCTATGAAACACTCAGGTTG  271


>ref|NM_033276.2| Homo sapiens XRCC6 binding protein 1 (XRCC6BP1), mRNA
 gb|BC012776.2| Homo sapiens XRCC6 binding protein 1, mRNA (cDNA clone MGC:16380 
IMAGE:3936722), complete cds
Length=1182

 Score = 86.1 bits (46),  Expect = 1e-14
 Identities = 46/46 (100%), Gaps = 0/46 (0%)
 Strand=Plus/Plus

Query  1    ATCCATATGTCAAACTTCTGCTTGATGCTATGAAACACTCAGGTTG  46
            ||||||||||||||||||||||||||||||||||||||||||||||
Sbjct  228  ATCCATATGTCAAACTTCTGCTTGATGCTATGAAACACTCAGGTTG  273


>gb|AY890450.1| Synthetic construct Homo sapiens clone FLH141483.01X Ku70-binding 
protein 3 (KUB3) mRNA, complete cds
Length=741

 Score = 86.1 bits (46),  Expect = 1e-14
 Identities = 46/46 (100%), Gaps = 0/46 (0%)
 Strand=Plus/Plus

Query  1    ATCCATATGTCAAACTTCTGCTTGATGCTATGAAACACTCAGGTTG  46
            ||||||||||||||||||||||||||||||||||||||||||||||
Sbjct  188  ATCCATATGTCAAACTTCTGCTTGATGCTATGAAACACTCAGGTTG  233


>gb|AY892908.1| Synthetic construct Homo sapiens clone FLH141479.01L Ku70-binding 
protein 3 (KUB3) mRNA, partial cds
Length=741

 Score = 86.1 bits (46),  Expect = 1e-14
 Identities = 46/46 (100%), Gaps = 0/46 (0%)
 Strand=Plus/Plus

Query  1    ATCCATATGTCAAACTTCTGCTTGATGCTATGAAACACTCAGGTTG  46
            ||||||||||||||||||||||||||||||||||||||||||||||
Sbjct  188  ATCCATATGTCAAACTTCTGCTTGATGCTATGAAACACTCAGGTTG  233


>gb|AF078164.2|AF078164 Homo sapiens Ku70-binding protein (KUB3) mRNA, partial cds
Length=1259

 Score = 86.1 bits (46),  Expect = 1e-14
 Identities = 46/46 (100%), Gaps = 0/46 (0%)
 Strand=Plus/Plus

Query  1    ATCCATATGTCAAACTTCTGCTTGATGCTATGAAACACTCAGGTTG  46
            ||||||||||||||||||||||||||||||||||||||||||||||
Sbjct  316  ATCCATATGTCAAACTTCTGCTTGATGCTATGAAACACTCAGGTTG  361


>ref|XM_003790597.1| PREDICTED: Otolemur garnettii XRCC6 binding protein 1 (XRCC6BP1), 
mRNA
Length=1176

 Score = 80.5 bits (43),  Expect = 5e-13
 Identities = 45/46 (97%), Gaps = 0/46 (0%)
 Strand=Plus/Plus

Query  1    ATCCATATGTCAAACTTCTGCTTGATGCTATGAAACACTCAGGTTG  46
            ||||||||||||||||||||||||||||||||||| ||||||||||
Sbjct  371  ATCCATATGTCAAACTTCTGCTTGATGCTATGAAATACTCAGGTTG  416


>ref|XM_003252400.1| PREDICTED: Nomascus leucogenys mitochondrial inner membrane protease 
ATP23 homolog (LOC100605009), partial mRNA
Length=1342

 Score = 80.5 bits (43),  Expect = 5e-13
 Identities = 45/46 (97%), Gaps = 0/46 (0%)
 Strand=Plus/Plus

Query  1    ATCCATATGTCAAACTTCTGCTTGATGCTATGAAACACTCAGGTTG  46
            |||| |||||||||||||||||||||||||||||||||||||||||
Sbjct  410  ATCCGTATGTCAAACTTCTGCTTGATGCTATGAAACACTCAGGTTG  455


>ref|XM_001102089.1| PREDICTED: Macaca mulatta mitochondrial inner membrane protease 
ATP23 homolog (LOC712932), mRNA
Length=1186

 Score = 80.5 bits (43),  Expect = 5e-13
 Identities = 45/46 (97%), Gaps = 0/46 (0%)
 Strand=Plus/Plus

Query  1    ATCCATATGTCAAACTTCTGCTTGATGCTATGAAACACTCAGGTTG  46
            |||| |||||||||||||||||||||||||||||||||||||||||
Sbjct  257  ATCCGTATGTCAAACTTCTGCTTGATGCTATGAAACACTCAGGTTG  302


>ref|XM_003476121.1| PREDICTED: Cavia porcellus mitochondrial inner membrane protease 
ATP23 homolog (LOC100716008), mRNA
Length=747

 Score = 75.0 bits (40),  Expect = 2e-11
 Identities = 44/46 (95%), Gaps = 0/46 (0%)
 Strand=Plus/Plus

Query  1    ATCCATATGTCAAACTTCTGCTTGATGCTATGAAACACTCAGGTTG  46
            |||| ||||||||||||||||||||||||||||| |||||||||||
Sbjct  194  ATCCCTATGTCAAACTTCTGCTTGATGCTATGAAGCACTCAGGTTG  239


>ref|XM_002916030.1| PREDICTED: Ailuropoda melanoleuca mitochondrial inner membrane 
protease ATP23 homolog (LOC100482991), mRNA
Length=1075

 Score = 75.0 bits (40),  Expect = 2e-11
 Identities = 44/46 (95%), Gaps = 0/46 (0%)
 Strand=Plus/Plus

Query  1    ATCCATATGTCAAACTTCTGCTTGATGCTATGAAACACTCAGGTTG  46
            |||||||||||||||||||||||||||| ||||| |||||||||||
Sbjct  188  ATCCATATGTCAAACTTCTGCTTGATGCCATGAAGCACTCAGGTTG  233


Lambda     K      H
    1.33    0.621     1.12 

Gapped
Lambda     K      H
    1.28    0.460    0.850 

Effective search space used: 874911716973
 
    reads 
 chr12 chr12 57662164 57660538 73m1550n3m                     ||||||||||||||||||||||||||||||||||||||||||||||||||||||||||||||||||||||||||||||||||||||||||||||||||||||||||||||||||||||||||||||||||||||||||||||||||||||||||||||||||||||||||||||||||||||||||||||||||||||||||||||||||||||||||||||||||||||||||||||||||||||||||||||||||||||||||||||||GCG
chr12 chr12 57662159 57660612 75m1471n1m                     ||||||||||||||||||||||||||||||||||||||||||||||||||||||||||||||||||||||||||||||||||||||||||||||||||||||||||||||||||||||||||||||||||||||||||||||||||||||||||||||||||||||||||||||||||||||||||||||||||||||||C
chr12 chr12 57662156 57660609 72m1471n4m                     ||||||||||||||||||||||||||||||||||||||||||||||||||||||||||||||||||||||||||||||||||||||||||||||||||||||||||||||||||||||||||||||||||||||||||||||||||||||||||||||||||||||||||||||||||||||||||||||||||||||||CCCT
chr12 chr12 57662152 57660605 68m1471n8m                     ||||||||||||||||||||||||||||||||||||||||||||||||||||||||||||||||||||||||||||||||||||||||||||||||||||||||||||||||||||||||||||||||||||||||||||||||||||||||||||||||||||||||||||||||||||||||||||||||||||||||CCCTTCTG
chr12 chr12 57662149 57660602 65m1471n11m                    ||||||||||||||||||||||||||||||||||||||||||||||||||||||||||||||||||||||||||||||||||||||||||||||||||||||||||||||||||||||||||||||||||||||||||||||||||||||||||||||||||||||||||||||||||||||||||||||||||||||||CCCTTCTGTAG
chr12 chr12 57662146 57660599 62m1471n14m                    ||||||||||||||||||||||||||||||||||||||||||||||||||||||||||||||||||||||||||||||||||||||||||||||||||||||||||||||||||||||||||||||||||||||||||||||||||||||||||||||||||||||||||||||||||||||||||||||||||||||||CCCTTCTGTAGGGT
chr12 chr12 57662143 57660517 52m1550n24m                    ||||||||||||||||||||||||||||||||||||||||||||||||||||||||||||||||||||||||||||||||||||||||||||||||||||||||||||||||||||||||||||||||||||||||||||||||||||||||||||||||||||||||||||||||||||||||||||||||||||||||||||||||||||||||||||||||||||||||||||||||||||||||||||||||||||||||||||||||TCCACCACAGATGCCACAGCAGTA
chr12 chr12 57662140 57660593 56m1471n20m                    ||||||||||||||||||||||||||||||||||||||||||||||||||||||||||||||||||||||||||||||||||||||||||||||||||||||||||||||||||||||||||||||||||||||||||||||||||||||||||||||||||||||||||||||||||||||||||||||||||||||||CCCTTCTGTAGGGTTTCTGC
chr12 chr12 57662137 57660590 53m1471n23m                    ||||||||||||||||||||||||||||||||||||||||||||||||||||||||||||||||||||||||||||||||||||||||||||||||||||||||||||||||||||||||||||||||||||||||||||||||||||||||||||||||||||||||||||||||||||||||||||||||||||||||CCCTTCTGTAGGGTTTCTGCAAC
chr12 chr12 57662134 57660587 50m1471n26m                    ||||||||||||||||||||||||||||||||||||||||||||||||||||||||||||||||||||||||||||||||||||||||||||||||||||||||||||||||||||||||||||||||||||||||||||||||||||||||||||||||||||||||||||||||||||||||||||||||||||||||CCCTTCTGTAGGGTTTCTGCAACCCC
chr12 chr12 57662131 57660584 47m1471n29m                    ||||||||||||||||||||||||||||||||||||||||||||||||||||||||||||||||||||||||||||||||||||||||||||||||||||||||||||||||||||||||||||||||||||||||||||||||||||||||||||||||||||||||||||||||||||||||||||||||||||||||CCCTTCTGTAGGGTTTCTGCAACCCCCTG
chr12 chr12 57662128 57660581 44m1471n32m                    ||||||||||||||||||||||||||||||||||||||||||||||||||||||||||||||||||||||||||||||||||||||||||||||||||||||||||||||||||||||||||||||||||||||||||||||||||||||||||||||||||||||||||||||||||||||||||||||||||||||||CCCTTCTGTAGGGTTTCTGCAACCCCCTGGCT
chr12 chr12 57662125 57660578 41m1471n35m                    ||||||||||||||||||||||||||||||||||||||||||||||||||||||||||||||||||||||||||||||||||||||||||||||||||||||||||||||||||||||||||||||||||||||||||||||||||||||||||||||||||||||||||||||||||||||||||||||||||||||||CCCTTCTGTAGGGTTTCTGCAACCCCCTGGCTCTG
chr12 chr12 57662122 57660575 38m1471n38m                    ||||||||||||||||||||||||||||||||||||||||||||||||||||||||||||||||||||||||||||||||||||||||||||||||||||||||||||||||||||||||||||||||||||||||||||||||||||||||||||||||||||||||||||||||||||||||||||||||||||||||CCCTTCTGTAGGGTTTCTGCAACCCCCTGGCTCTGAGC
chr12 chr12 57662119 57660572 35m1471n41m                    ||||||||||||||||||||||||||||||||||||||||||||||||||||||||||||||||||||||||||||||||||||||||||||||||||||||||||||||||||||||||||||||||||||||||||||||||||||||||||||||||||||||||||||||||||||||||||||||||||||||||CCCTTCTGTAGGGTTTCTGCAACCCCCTGGCTCTGAGCAGT
chr12 chr12 57662116 57660569 32m1471n44m                    ||||||||||||||||||||||||||||||||||||||||||||||||||||||||||||||||||||||||||||||||||||||||||||||||||||||||||||||||||||||||||||||||||||||||||||||||||||||||||||||||||||||||||||||||||||||||||||||||||||||||CCCTTCTGTAGGGTTTCTGCAACCCCCTGGCTCTGAGCAGTACC
chr12 chr12 57662113 57660566 29m1471n47m                    ||||||||||||||||||||||||||||||||||||||||||||||||||||||||||||||||||||||||||||||||||||||||||||||||||||||||||||||||||||||||||||||||||||||||||||||||||||||||||||||||||||||||||||||||||||||||||||||||||||||||CCCTTCTGTAGGGTTTCTGCAACCCCCTGGCTCTGAGCAGTACCAGA
chr12 chr12 57662110 57660563 26m1471n50m                    ||||||||||||||||||||||||||||||||||||||||||||||||||||||||||||||||||||||||||||||||||||||||||||||||||||||||||||||||||||||||||||||||||||||||||||||||||||||||||||||||||||||||||||||||||||||||||||||||||||||||CCCTTCTGTAGGGTTTCTGCAACCCCCTGGCTCTGAGCAGTACCAGATGC
chr12 chr12 57662107 57660560 23m1471n53m                    ||||||||||||||||||||||||||||||||||||||||||||||||||||||||||||||||||||||||||||||||||||||||||||||||||||||||||||||||||||||||||||||||||||||||||||||||||||||||||||||||||||||||||||||||||||||||||||||||||||||||CCCTTCTGTAGGGTTTCTGCAACCCCCTGGCTCTGAGCAGTACCAGATGCCTC
chr12 chr12 57662104 57660557 20m1471n56m                    ||||||||||||||||||||||||||||||||||||||||||||||||||||||||||||||||||||||||||||||||||||||||||||||||||||||||||||||||||||||||||||||||||||||||||||||||||||||||||||||||||||||||||||||||||||||||||||||||||||||||CCCTTCTGTAGGGTTTCTGCAACCCCCTGGCTCTGAGCAGTACCAGATGCCTCAGT
chr12 chr12 57662101 57660554 17m1471n59m                    ||||||||||||||||||||||||||||||||||||||||||||||||||||||||||||||||||||||||||||||||||||||||||||||||||||||||||||||||||||||||||||||||||||||||||||||||||||||||||||||||||||||||||||||||||||||||||||||||||||||||CCCTTCTGTAGGGTTTCTGCAACCCCCTGGCTCTGAGCAGTACCAGATGCCTCAGTCTC
chr12 chr12 57662098 57660551 14m1471n62m                    ||||||||||||||||||||||||||||||||||||||||||||||||||||||||||||||||||||||||||||||||||||||||||||||||||||||||||||||||||||||||||||||||||||||||||||||||||||||||||||||||||||||||||||||||||||||||||||||||||||||||CCCTTCTGTAGGGTTTCTGCAACCCCCTGGCTCTGAGCAGTACCAGATGCCTCAGTCTCCCT
chr12 chr12 57662095 57660548 11m1471n65m                    ||||||||||||||||||||||||||||||||||||||||||||||||||||||||||||||||||||||||||||||||||||||||||||||||||||||||||||||||||||||||||||||||||||||||||||||||||||||||||||||||||||||||||||||||||||||||||||||||||||||||CCCTTCTGTAGGGTTTCTGCAACCCCCTGGCTCTGAGCAGTACCAGATGCCTCAGTCTCCCTCTC
chr12 chr12 57662092 57660545 8m1471n68m                     ||||||||||||||||||||||||||||||||||||||||||||||||||||||||||||||||||||||||||||||||||||||||||||||||||||||||||||||||||||||||||||||||||||||||||||||||||||||||||||||||||||||||||||||||||||||||||||||||||||||||CCCTTCTGTAGGGTTTCTGCAACCCCCTGGCTCTGAGCAGTACCAGATGCCTCAGTCTCCCTCTCCCT
chr12 chr12 57662089 57660542 5m1471n71m                     ||||||||||||||||||||||||||||||||||||||||||||||||||||||||||||||||||||||||||||||||||||||||||||||||||||||||||||||||||||||||||||||||||||||||||||||||||||||||||||||||||||||||||||||||||||||||||||||||||||||||CCCTTCTGTAGGGTTTCTGCAACCCCCTGGCTCTGAGCAGTACCAGATGCCTCAGTCTCCATCTCCCTGCA
chr12 chr12 57660859 57660783 76m                            GAAAGATGATCAAGTACAAATACATATAAT
chr12 chr12 57660689 57660613 76m                                                                                                                                                        GAAGCAGCAGCTGTTTGCCAGGAGCTGTGTTCATCCCTCTACTGCTTCACCACTCCTCTCTTCTCTGTCTTCCCAG
chr12 chr12 57660619 57660543 76m                                                                                                                                                                                                                              TACGAGCCCTTCTGTAGGGTTTCTGCAACCCCCTGGCTCTGAGCAGTACCAGATGCCTCAGTCTCCCTCTCCCTGC
chr12 chr12 57660616 57660540 76m                                                                                                                                                                                                                                 GCGCCCTTCTGTAGGGTTTCTGCAACCCCCTGGCTCTGAGCAGTACCAGATGCCTCAGTCTCCCTCTCCCTGCAGT
chr12 chr12 57660613 57660537 76m                                                                                                                                                                                                                                    CCCTTCTGTAGGGTTTCTGCAACCCCCTGGCTCTGAGCAGTACCAGATGCCTCAGTCTCCCTCTCCCTGCAGTCCA
chr12 chr12 57660610 57660534 76m                                                                                                                                                                                                                                       TTCTGTAGGGTTTCTGCAACCCCCTGGCTCTGAGCAGTACCAGATGCCTCAGTCTCCCTCTCCCTGCAGTCCACCA
chr12 chr12 57660607 57660531 76m                                                                                                                                                                                                                                          TGTAGGGTTTCTGCAACCCCCTGGCTCTGAGCAGTACCAGATGCCTCAGTCTCCCTCTCCCTGCAGTCCACCACAG
chr12 chr12 57660604 57660528 76m                                                                                                                                                                                                                                             AGGGTTTCTGCAACCCCCTGGCTCTGAGCAGTACCAGATGCCTCAGTCTCCCTCTCCCTGCAGTCCACCACAGATG
chr12 chr12 57660601 57660525 76m                                                                                                                                                                                                                                                GTTTCTGCAACCCCCTGGCTCTGAGCAGTACCAGATGCCTCAGTCTCCCTCTCCCTGCAGTCCACCACAGATGCCA
chr12 chr12 57660598 57660522 76m                                                                                                                                                                                                                                                   TCTGCAACCCCCTGGCTCTGAGCAGTACCAGATGCCTCAGTCTCCCTCTCCCTGCAGTCCACCACAGATGCCACAG
chr12 chr12 57660595 57660519 76m                                                                                                                                                                                                                                                      GCAACCCCCTGGCTCTGAGCAGTACCAGATGCCTCAGTCTCCCTCTCCCTGCAGTCCACCACAGATGCCACAGCAG
chr12 chr12 57660592 57660516 76m                                                                                                                                                                                                                                                         ACCTCCTGGCTCTGAGCAGTACCAGATGCCTCAGTCTCCCTCTCCCTGCAGTCCACCACAGATGCCACAGCAGTAC
chr12 chr12 57660589 57660513 76m                                                                                                                                                                                                                                                            CCCTGGCTCTGAGCAGTACCAGATGCCTCAGTCTCCCTCTCCCTGCAGTCCACCACAGATGCCACAGCAGTACTCA
chr12 chr12 57660586 57660510 76m                                                                                                                                                                                                                                                               TGGCTCTGAGCAGTACCAGATGCCTCAGTCTCCCTCTCCCTGCAGTCCACCACAGATGCCACAGCAGTACTCAGGA
chr12 chr12 57660581 58339417 69m58339411F7M                                                                                                                                                                                                                                                         CTGAGCAGTACCAGATGCCTCAGTCTCCCTCTCCCTGCAGTCCACCACAGATGCCACAGCAGTACTCAG ATCCATA
chr12 chr12 57660570 58339428 58m58339411F18M                                                                                                                                                                                                                                                                   CAGATGCCTCAGTCTCCCTCTCCCTGCAGTCCACCACAGATGCCACAGCAGTACTCAG ATCCATATGTCAAACTTC
chr12 chr12 57660570 58339428 58m58339411F18M                                                                                                                                                                                                                                                                   CAGATGCCTCAGTCTCCCTCTCCCTGCAGTCCACCACAGATGCCACAGCAGTACTCAG ATCCATATGTCAAACTTC
chr12 chr12 57660569 58339429 57m58339411F19M                                                                                                                                                                                                                                                                    AGATGCCTCAGTCTCCCTCTCCCTGCAGTCCACCACAGATGCCACAGCAGTACTCAG ATCCATATGTCAAACTTCT
chr12 chr12 57660567 58339431 55m58339411F21M                                                                                                                                                                                                                                                                      GCAGAAGTTTGACATATGGATCTGAGTACTGCTGTGGCATCTGTGGTGGACTGCA GGGAGAGGGAGACTGAGGCAT
chr12 chr12 57660567 58339431 55m58339411F21M                                                                                                                                                                                                                                                                      ATGCCTCAGTCTCCCTCTCCCTGCAGTCCACCACAGATGCCACAGCAGTACTCAG ATCCATATGTCAAACTTCTGC
chr12 chr12 57660556 58339442 44m58339411F32M                                                                                                                                                                                                                                                                                 TCCCTCTCCCTGCAGTCCACCACAGATGCCACAGCAGTACTCAG ATCCATATGTCAAACTTCTGCTTGATGCTATG
chr12 chr12 57660547 58339451 35m58339411F41M                                                                                                                                                                                                                                                                                          CTGCAGTCCACCACAGATGCCACAGCAGTACTCAG ATCCATATGTCAAACTTCTGCTTGATGCTATGAAACACTCA
chr12 chr12 57660546 58339452 34m58339411F42M                                                                                                                                                                                                                                                                                           TGCAGTCCACCACAGATGCCACAGCAGTACTCAG ATCCATATGTCAAACTTCTGCTTGATGCTATGAAACACTCAG
chr12 chr12 57660542 58339456 30m58339411F46M                                                                                                                                                                                                                                                                                               GTCCACCACAGATGCCACAGCAGTACTCAG ATCCATATGTCAAACTTCTGCTTGATGCTATGAAACACTCAGGTTG
chr12 chr12 57660539 58340780 27m58339411F46M1321N3M                                                                                                                                                                                                                                                                                           CACCACAGATGCCACAGCAGTACTCAG ATCCATATGTCAAACTTCTGCTTGATGCTATGAAGCACTCAGGTTG||||||||||||||||||||||||||||||||||||||||||||||||||||||||||||||||||||||||||||||||||||||||||||||||||||||||||||||||||||||||||||||||||||||||||||||||||||||||||||||||||||||||||||||||||||||||||||||||||||||||||||||||||||||||||||||||||||||||||||||||||||||||||||
chr12 chr12 57660538 58340781 26m58339411F46M1321N4M                                                                                                                                                                                                                                                                                            ACCACAGATGCCACAGCAGTACTCAG ATCCATATGTCAAACTTCTGCTTGATGCTATGAAACACTCAGGTTG||||||||||||||||||||||||||||||||||||||||||||||||||||||||||||||||||||||||||||||||||||||||||||||||||||||||||||||||||||||||||||||||||||||||||||||||||||||||||||||||||||||||||||||||||||||||||||||||||||||||||||||||||||||||||||||||||||||||||||||||||||||||||||
chr12 chr12 57660536 58340783 24m58339411F46M1321N6M                                                                                                                                                                                                                                                                                              CACAGATGCCACAGCAGTACTCAG ATCCATATGTCAAACTTCTGCTTGCTGCTATGAAACACTCAGGTTG||||||||||||||||||||||||||||||||||||||||||||||||||||||||||||||||||||||||||||||||||||||||||||||||||||||||||||||||||||||||||||||||||||||||||||||||||||||||||||||||||||||||||||||||||||||||||||||||||||||||||||||||||||||||||||||||||||||||||||||||||||||||||||
chr12 chr12 57660535 58340784 23m58339411F46M1321N7M                                                                                                                                                                                                                                                                                               ACAGATGCCACAGCAGTACTCAG ATCCATATGTCAAACTTCTGCTTGATGCTATGAAACACTCAGGTTG||||||||||||||||||||||||||||||||||||||||||||||||||||||||||||||||||||||||||||||||||||||||||||||||||||||||||||||||||||||||||||||||||||||||||||||||||||||||||||||||||||||||||||||||||||||||||||||||||||||||||||||||||||||||||||||||||||||||||||||||||||||||||||
chr12 chr12 57660521 58340798 9m58339411F46M1321N21M                                                                                                                                                                                                                                                                                                             AGTACTCAG ATCCATATGTCAAACTTCTGCTTGATGCTATGAAACACTCAGGTTG||||||||||||||||||||||||||||||||||||||||||||||||||||||||||||||||||||||||||||||||||||||||||||||||||||||||||||||||||||||||||||||||||||||||||||||||||||||||||||||||||||||||||||||||||||||||||||||||||||||||||||||||||||||||||||||||||||||||||||||||||||||||||||
chr12 chr12 57660521 58340798 9m58339411F46M1321N21M                                                                                                                                                                                                                                                                                                             AGTACTCAG ATCCATATGTCAAACTTCTGCTTGATGCTATGAAACACTCAGGTTG||||||||||||||||||||||||||||||||||||||||||||||||||||||||||||||||||||||||||||||||||||||||||||||||||||||||||||||||||||||||||||||||||||||||||||||||||||||||||||||||||||||||||||||||||||||||||||||||||||||||||||||||||||||||||||||||||||||||||||||||||||||||||||
chr12 chr12 58339401 58339477 76M                                                                                                                                                                                                                                                                                                                                 CTTTTTTAGATCCATATGTCAAACTTCTGCTTGATGCTATGAAACACTCAGGTTGGTAAGTAACTCTTTTTGAAGT
chr12 chr12 58339407 58340804 49M1321N27M                                                                                                                                                                                                                                                                                                                               TAGATCCATATGTCAAACTTCTGCTTGATGCTATGAAACACTCAGGTTG||||||||||||||||||||||||||||||||||||||||||||||||||||||||||||||||||||||||||||||||||||||||||||||||||||||||||||||||||||||||||||||||||||||||||||||||||||||||||||||||||||||||||||||||||||||||||||||||||||||||||||||||||||||||||||||||||||||||||||||||||||||||||||
chr12 chr12 58339410 58340807 46M1321N30M                                                                                                                                                                                                                                                                                                                                  ATCCATATGTCAAACTTCTGCTTGATGCTATGAAACACTCAGGTTG||||||||||||||||||||||||||||||||||||||||||||||||||||||||||||||||||||||||||||||||||||||||||||||||||||||||||||||||||||||||||||||||||||||||||||||||||||||||||||||||||||||||||||||||||||||||||||||||||||||||||||||||||||||||||||||||||||||||||||||||||||||||||||
chr12 chr12 58339413 58340810 43M1321N33M                                                                                                                                                                                                                                                                                                                                     CATATGTCAAACTTCTGCTTGATGCTATGAAACACTCAGGTTG||||||||||||||||||||||||||||||||||||||||||||||||||||||||||||||||||||||||||||||||||||||||||||||||||||||||||||||||||||||||||||||||||||||||||||||||||||||||||||||||||||||||||||||||||||||||||||||||||||||||||||||||||||||||||||||||||||||||||||||||||||||||||||
chr12 chr12 58339417 58340814 39M1321N37M                                                                                                                                                                                                                                                                                                                                         TGTCAAACTTCTGCTTGATGCTATGAAACACTCAGGTTG||||||||||||||||||||||||||||||||||||||||||||||||||||||||||||||||||||||||||||||||||||||||||||||||||||||||||||||||||||||||||||||||||||||||||||||||||||||||||||||||||||||||||||||||||||||||||||||||||||||||||||||||||||||||||||||||||||||||||||||||||||||||||||
chr12 chr12 58339420 58340817 36M1321N40M                                                                                                                                                                                                                                                                                                                                            CAAACTTCGGCTTGATGCTATGAAACACTCAGGTTC||||||||||||||||||||||||||||||||||||||||||||||||||||||||||||||||||||||||||||||||||||||||||||||||||||||||||||||||||||||||||||||||||||||||||||||||||||||||||||||||||||||||||||||||||||||||||||||||||||||||||||||||||||||||||||||||||||||||||||||||||||||||||||
chr12 chr12 58339423 58340820 33M1321N43M                                                                                                                                                                                                                                                                                                                                               ACTTCTGCTTGATGCTATGAAACACTCAGGTTG||||||||||||||||||||||||||||||||||||||||||||||||||||||||||||||||||||||||||||||||||||||||||||||||||||||||||||||||||||||||||||||||||||||||||||||||||||||||||||||||||||||||||||||||||||||||||||||||||||||||||||||||||||||||||||||||||||||||||||||||||||||||||||
chr12 chr12 58339427 58340824 29M1321N47M                                                                                                                                                                                                                                                                                                                                                   CTGCTTGATGCTATGAAACACTCAGGTTG||||||||||||||||||||||||||||||||||||||||||||||||||||||||||||||||||||||||||||||||||||||||||||||||||||||||||||||||||||||||||||||||||||||||||||||||||||||||||||||||||||||||||||||||||||||||||||||||||||||||||||||||||||||||||||||||||||||||||||||||||||||||||||
chr12 chr12 58339430 58340827 26M1321N50M                                                                                                                                                                                                                                                                                                                                                      CTTGATGCTATGAAACACTCAGGTTG||||||||||||||||||||||||||||||||||||||||||||||||||||||||||||||||||||||||||||||||||||||||||||||||||||||||||||||||||||||||||||||||||||||||||||||||||||||||||||||||||||||||||||||||||||||||||||||||||||||||||||||||||||||||||||||||||||||||||||||||||||||||||||
chr12 chr12 58339433 58340830 23M1321N53M                                                                                                                                                                                                                                                                                                                                                         GATGCTATGAAACACTCAGGTTG||||||||||||||||||||||||||||||||||||||||||||||||||||||||||||||||||||||||||||||||||||||||||||||||||||||||||||||||||||||||||||||||||||||||||||||||||||||||||||||||||||||||||||||||||||||||||||||||||||||||||||||||||||||||||||||||||||||||||||||||||||||||||||
chr12 chr12 58339437 58340834 19M1321N57M                                                                                                                                                                                                                                                                                                                                                             CTATGAAACACTCAGGTTG||||||||||||||||||||||||||||||||||||||||||||||||||||||||||||||||||||||||||||||||||||||||||||||||||||||||||||||||||||||||||||||||||||||||||||||||||||||||||||||||||||||||||||||||||||||||||||||||||||||||||||||||||||||||||||||||||||||||||||||||||||||||||||
chr12 chr12 58339440 58340837 16M1321N60M                                                                                                                                                                                                                                                                                                                                                                TGAAACACTCAGGTTG||||||||||||||||||||||||||||||||||||||||||||||||||||||||||||||||||||||||||||||||||||||||||||||||||||||||||||||||||||||||||||||||||||||||||||||||||||||||||||||||||||||||||||||||||||||||||||||||||||||||||||||||||||||||||||||||||||||||||||||||||||||||||||
chr12 chr12 58339444 58340841 12M1321N64M                                                                                                                                                                                                                                                                                                                                                                    ACACTCAGGTTG||||||||||||||||||||||||||||||||||||||||||||||||||||||||||||||||||||||||||||||||||||||||||||||||||||||||||||||||||||||||||||||||||||||||||||||||||||||||||||||||||||||||||||||||||||||||||||||||||||||||||||||||||||||||||||||||||||||||||||||||||||||||||||
chr12 chr12 58339447 58340844 9M1321N67M                                                                                                                                                                                                                                                                                                                                                                        CTCAGGTTG||||||||||||||||||||||||||||||||||||||||||||||||||||||||||||||||||||||||||||||||||||||||||||||||||||||||||||||||||||||||||||||||||||||||||||||||||||||||||||||||||||||||||||||||||||||||||||||||||||||||||||||||||||||||||||||||||||||||||||||||||||||||||||
chr12 chr12 58339451 58340848 5M1321N71M                                                                                                                                                                                                                                                                                                                                                                            GGTTG||||||||||||||||||||||||||||||||||||||||||||||||||||||||||||||||||||||||||||||||||||||||||||||||||||||||||||||||||||||||||||||||||||||||||||||||||||||||||||||||||||||||||||||||||||||||||||||||||||||||||||||||||||||||||||||||||||||||||||||||||||||||||||
chr12 chr12 58339454 58340851 2M1321N74M                                                                                                                                                                                                                                                                                                                                                                               TG||||||||||||||||||||||||||||||||||||||||||||||||||||||||||||||||||||||||||||||||||||||||||||||||||||||||||||||||||||||||||||||||||||||||||||||||||||||||||||||||||||||||||||||||||||||||||||||||||||||||||||||||||||||||||||||||||||||||||||||||||||||||||||
chr12 chr12 58339467 58339543 76M                                                                                                                                                                                                                                                                                                                                                                                                   TTTTTGAAGTGCTGCTCTGAGGTCTGGCTGCTGAAAACAAAAACCAAGTTCTCTTCTTAAGAGAACTGCTTAAGAT
chr12 chr12 58339481 58339557 76M                                                                                                                                                                                                                                                                                                                                                                                                                 CTCTGAGGTCTGGCTGCTGAAAACAAAAACCAAGTTCTCTTCTTAAGAGAACTGCTTAAGATTTCTCTGAGGTCGA
chr12 chr12 58339501 58339577 76M                                                                                                                                                                                                                                                                                                                                                                                                                                     AAACAAAAACCAAGTTCTCTTCTTAAGAGAACTGCTTAAGATTTCTCTGAGGTCACAAAGTCTTTTCCATAGGGTA
chr12 chr12 58339504 58339580 76M                                                                                                                                                                                                                                                                                                                                                                                                                                        CAAAAACCAAGTTCTCTTCTTAAGAGAACTGCTTAAGATTTCTCTGAGGTCACAAAGTCTTTTCCATAGGGTAAAT
chr12 chr12 58339508 58339584 76M                                                                                                                                                                                                                                                                                                                                                                                                                                            AACCAAGTTCTCTTCTTAAGAGAACTGCTTAAGATTTCTCTGAGGTCACAAAGTCTTTTCCATAGGGTAAATAGAG
chr12 chr12 58339521 58339597 76M                                                                                                                                                                                                                                                                                                                                                                                                                                                         TCTTAAGAGAACTGCTTAAGATTTCTCTGAGGTCACAAAGTCTTTTCCATAGGGTAAATAGAGGTAATTGGCTGCA
chr12 chr12 58339548 58339624 76M                                                                                                                                                                                                                                                                                                                                                                                                                                                                                    TGAGGTCACAAAGTCTTTTCCATAGGGTAAATAGAGGTAATTGGCTGCATTGTCTCCGGGCTGGCCTTGTTCTCTT
chr12 chr12 58339562 58339638 76M                                                                                                                                                                                                                                                                                                                                                                                                                                                                                                  CTTTTCCATAGGGTAAATAGAGGTAATTGGCTGCATTGTCTCCGGGCTGGCCTTGTTCTCTTTGGACATTTTCTGC
chr12 chr12 58339580 58339656 76M                                                                                                                                                                                                                                                                                                                                                                                                                                                                                                                    AGAGGTAATTGGCTGCATTGTCTCCGGGCTGGCCTTGTTCTCTTTGGACATTTTCTGCCCTAACTCATGTTTTTTT
chr12 chr12 58339697 58339773 76M                                                                                                                                                                                                                                                                                                                                                                                                                                                                                                                                                                                                                                         GAGTGCGTTGG 
    5 pairs 
 9:24
97:24
14:1379
1662:11
23:-9778 
     TCGA-02-2485-01A chr12-chr12 57824502 57898007 rf 
 
271 2 2 4 56 56
CGGGCGGCGGGCGGAGACGTGGGCGCCCTCCCCGCCAAGGCCCGTGCGAG AAGCCTCAGTGTAAAGTCTGCCGATCATGCCCTGTGGTGCAGTCGAGCCA
99999999999999999999999999999999999999999999999999 99999999999999999999999999999999999999999999999999
ENSG00000179912 exon2(57824501-57824572) MARS exon11(57898006-57898081)
 
 blast search - human genome  
 left 
 >ref|NT_029419.12| Homo sapiens chromosome 12 genomic contig, GRCh37.p9 Primary 
Assembly
 gb|GL000107.1| Homo sapiens chromosome 12 genomic contig, GRCh37 reference primary 
assembly
Length=71516776

 Score = 93.5 bits (50),  Expect = 5e-17
 Identities = 50/50 (100%), Gaps = 0/50 (0%)
 Strand=Plus/Minus

Query  1         CGGGCGGCGGGCGGAGACGTGGGCGCCCTCCCCGCCAAGGCCCGTGCGAG  50
                 ||||||||||||||||||||||||||||||||||||||||||||||||||
Sbjct  19967858  CGGGCGGCGGGCGGAGACGTGGGCGCCCTCCCCGCCAAGGCCCGTGCGAG  19967809


>ref|NW_001838060.2| Homo sapiens chromosome 12 genomic contig, alternate assembly 
HuRef SCAF_1103279188147, whole genome shotgun sequence
 gb|DS486061.1| Homo sapiens SCAF_1103279188147 genomic scaffold, whole genome 
shotgun sequence
Length=18942049

 Score = 93.5 bits (50),  Expect = 5e-17
 Identities = 50/50 (100%), Gaps = 0/50 (0%)
 Strand=Plus/Plus

Query  1         CGGGCGGCGGGCGGAGACGTGGGCGCCCTCCCCGCCAAGGCCCGTGCGAG  50
                 ||||||||||||||||||||||||||||||||||||||||||||||||||
Sbjct  18704272  CGGGCGGCGGGCGGAGACGTGGGCGCCCTCCCCGCCAAGGCCCGTGCGAG  18704321


>ref|NC_000012.11| Homo sapiens chromosome 12, GRCh37.p9 Primary Assembly
 gb|CM000674.1| Homo sapiens chromosome 12, GRCh37 primary reference assembly
Length=133851895

 Score = 93.5 bits (50),  Expect = 5e-17
 Identities = 50/50 (100%), Gaps = 0/50 (0%)
 Strand=Plus/Minus

Query  1         CGGGCGGCGGGCGGAGACGTGGGCGCCCTCCCCGCCAAGGCCCGTGCGAG  50
                 ||||||||||||||||||||||||||||||||||||||||||||||||||
Sbjct  57824552  CGGGCGGCGGGCGGAGACGTGGGCGCCCTCCCCGCCAAGGCCCGTGCGAG  57824503


>ref|AC_000144.1| Homo sapiens chromosome 12, alternate assembly HuRef, whole genome 
shotgun sequence
 gb|CM000473.1| Homo sapiens chromosome 12, whole genome shotgun sequence
Length=130617856

 Score = 93.5 bits (50),  Expect = 5e-17
 Identities = 50/50 (100%), Gaps = 0/50 (0%)
 Strand=Plus/Minus

Query  1         CGGGCGGCGGGCGGAGACGTGGGCGCCCTCCCCGCCAAGGCCCGTGCGAG  50
                 ||||||||||||||||||||||||||||||||||||||||||||||||||
Sbjct  54862305  CGGGCGGCGGGCGGAGACGTGGGCGCCCTCCCCGCCAAGGCCCGTGCGAG  54862256


Lambda     K      H
    1.33    0.621     1.12 

Gapped
Lambda     K      H
    1.28    0.460    0.850 

Effective search space used: 681780354108
 
 right 
 >ref|NT_029419.12| Homo sapiens chromosome 12 genomic contig, GRCh37.p9 Primary 
Assembly
 gb|GL000107.1| Homo sapiens chromosome 12 genomic contig, GRCh37 reference primary 
assembly
Length=71516776

 Score = 93.5 bits (50),  Expect = 5e-17
 Identities = 50/50 (100%), Gaps = 0/50 (0%)
 Strand=Plus/Plus

Query  1         AAGCCTCAGTGTAAAGTCTGCCGATCATGCCCTGTGGTGCAGTCGAGCCA  50
                 ||||||||||||||||||||||||||||||||||||||||||||||||||
Sbjct  20041314  AAGCCTCAGTGTAAAGTCTGCCGATCATGCCCTGTGGTGCAGTCGAGCCA  20041363


>ref|NW_001838060.2| Homo sapiens chromosome 12 genomic contig, alternate assembly 
HuRef SCAF_1103279188147, whole genome shotgun sequence
 gb|DS486061.1| Homo sapiens SCAF_1103279188147 genomic scaffold, whole genome 
shotgun sequence
Length=18942049

 Score = 93.5 bits (50),  Expect = 5e-17
 Identities = 50/50 (100%), Gaps = 0/50 (0%)
 Strand=Plus/Minus

Query  1         AAGCCTCAGTGTAAAGTCTGCCGATCATGCCCTGTGGTGCAGTCGAGCCA  50
                 ||||||||||||||||||||||||||||||||||||||||||||||||||
Sbjct  18630492  AAGCCTCAGTGTAAAGTCTGCCGATCATGCCCTGTGGTGCAGTCGAGCCA  18630443


>ref|NC_000012.11| Homo sapiens chromosome 12, GRCh37.p9 Primary Assembly
 gb|CM000674.1| Homo sapiens chromosome 12, GRCh37 primary reference assembly
Length=133851895

 Score = 93.5 bits (50),  Expect = 5e-17
 Identities = 50/50 (100%), Gaps = 0/50 (0%)
 Strand=Plus/Plus

Query  1         AAGCCTCAGTGTAAAGTCTGCCGATCATGCCCTGTGGTGCAGTCGAGCCA  50
                 ||||||||||||||||||||||||||||||||||||||||||||||||||
Sbjct  57898008  AAGCCTCAGTGTAAAGTCTGCCGATCATGCCCTGTGGTGCAGTCGAGCCA  57898057


>ref|AC_000144.1| Homo sapiens chromosome 12, alternate assembly HuRef, whole genome 
shotgun sequence
 gb|CM000473.1| Homo sapiens chromosome 12, whole genome shotgun sequence
Length=130617856

 Score = 93.5 bits (50),  Expect = 5e-17
 Identities = 50/50 (100%), Gaps = 0/50 (0%)
 Strand=Plus/Plus

Query  1         AAGCCTCAGTGTAAAGTCTGCCGATCATGCCCTGTGGTGCAGTCGAGCCA  50
                 ||||||||||||||||||||||||||||||||||||||||||||||||||
Sbjct  54936085  AAGCCTCAGTGTAAAGTCTGCCGATCATGCCCTGTGGTGCAGTCGAGCCA  54936134


>gb|CM000263.1| Homo sapiens chromosome 12, whole genome shotgun sequence
Length=133516800

 Score = 93.5 bits (50),  Expect = 5e-17
 Identities = 50/50 (100%), Gaps = 0/50 (0%)
 Strand=Plus/Plus

Query  1         AAGCCTCAGTGTAAAGTCTGCCGATCATGCCCTGTGGTGCAGTCGAGCCA  50
                 ||||||||||||||||||||||||||||||||||||||||||||||||||
Sbjct  57553844  AAGCCTCAGTGTAAAGTCTGCCGATCATGCCCTGTGGTGCAGTCGAGCCA  57553893


Lambda     K      H
    1.33    0.621     1.12 

Gapped
Lambda     K      H
    1.28    0.460    0.850 

Effective search space used: 681780354108
 
 blast search - nt  
 left 
 >ref|NM_001132581.1| Pongo abelii R3H domain containing 2 (R3HDM2), mRNA
 emb|CR859659.1| Pongo abelii mRNA; cDNA DKFZp469E2113 (from clone DKFZp469E2113)
Length=4076

 Score = 76.8 bits (41),  Expect = 7e-12
 Identities = 47/50 (94%), Gaps = 0/50 (0%)
 Strand=Plus/Plus

Query  1   CGGGCGGCGGGCGGAGACGTGGGCGCCCTCCCCGCCAAGGCCCGTGCGAG  50
           ||||||||||||||||||||||||||||||||||| | ||| ||||||||
Sbjct  23  CGGGCGGCGGGCGGAGACGTGGGCGCCCTCCCCGCTACGGCTCGTGCGAG  72


Lambda     K      H
    1.33    0.621     1.12 

Gapped
Lambda     K      H
    1.28    0.460    0.850 

Effective search space used: 874911716973
 
 right 
 >ref|XM_002823424.2| PREDICTED: Pongo abelii methionyl-tRNA synthetase (MARS), mRNA
Length=2917

 Score = 93.5 bits (50),  Expect = 6e-17
 Identities = 50/50 (100%), Gaps = 0/50 (0%)
 Strand=Plus/Plus

Query  1     AAGCCTCAGTGTAAAGTCTGCCGATCATGCCCTGTGGTGCAGTCGAGCCA  50
             ||||||||||||||||||||||||||||||||||||||||||||||||||
Sbjct  1432  AAGCCTCAGTGTAAAGTCTGCCGATCATGCCCTGTGGTGCAGTCGAGCCA  1481


>ref|NM_004990.3| Homo sapiens methionyl-tRNA synthetase (MARS), mRNA
Length=2932

 Score = 93.5 bits (50),  Expect = 6e-17
 Identities = 50/50 (100%), Gaps = 0/50 (0%)
 Strand=Plus/Plus

Query  1     AAGCCTCAGTGTAAAGTCTGCCGATCATGCCCTGTGGTGCAGTCGAGCCA  50
             ||||||||||||||||||||||||||||||||||||||||||||||||||
Sbjct  1432  AAGCCTCAGTGTAAAGTCTGCCGATCATGCCCTGTGGTGCAGTCGAGCCA  1481


>dbj|AB590802.1| Synthetic construct DNA, clone: pFN21AB8542, Homo sapiens MARS 
gene for methionyl-tRNA synthetase, without stop codon, in 
Flexi system
Length=2717

 Score = 93.5 bits (50),  Expect = 6e-17
 Identities = 50/50 (100%), Gaps = 0/50 (0%)
 Strand=Plus/Plus

Query  1     AAGCCTCAGTGTAAAGTCTGCCGATCATGCCCTGTGGTGCAGTCGAGCCA  50
             ||||||||||||||||||||||||||||||||||||||||||||||||||
Sbjct  1303  AAGCCTCAGTGTAAAGTCTGCCGATCATGCCCTGTGGTGCAGTCGAGCCA  1352


>dbj|AK293945.1| Homo sapiens cDNA FLJ53309 complete cds, highly similar to Methionyl-tRNA 
synthetase (EC 6.1.1.10)
Length=2702

 Score = 93.5 bits (50),  Expect = 6e-17
 Identities = 50/50 (100%), Gaps = 0/50 (0%)
 Strand=Plus/Plus

Query  1     AAGCCTCAGTGTAAAGTCTGCCGATCATGCCCTGTGGTGCAGTCGAGCCA  50
             ||||||||||||||||||||||||||||||||||||||||||||||||||
Sbjct  1243  AAGCCTCAGTGTAAAGTCTGCCGATCATGCCCTGTGGTGCAGTCGAGCCA  1292


>dbj|AK290857.1| Homo sapiens cDNA FLJ76789 complete cds, highly similar to Homo 
sapiens methionine-tRNA synthetase (MARS), mRNA
Length=2780

 Score = 93.5 bits (50),  Expect = 6e-17
 Identities = 50/50 (100%), Gaps = 0/50 (0%)
 Strand=Plus/Plus

Query  1     AAGCCTCAGTGTAAAGTCTGCCGATCATGCCCTGTGGTGCAGTCGAGCCA  50
             ||||||||||||||||||||||||||||||||||||||||||||||||||
Sbjct  1303  AAGCCTCAGTGTAAAGTCTGCCGATCATGCCCTGTGGTGCAGTCGAGCCA  1352


>gb|BC065187.1| Homo sapiens methionine-tRNA synthetase, mRNA (cDNA clone IMAGE:5021529), 
partial cds
Length=2830

 Score = 93.5 bits (50),  Expect = 6e-17
 Identities = 50/50 (100%), Gaps = 0/50 (0%)
 Strand=Plus/Plus

Query  1     AAGCCTCAGTGTAAAGTCTGCCGATCATGCCCTGTGGTGCAGTCGAGCCA  50
             ||||||||||||||||||||||||||||||||||||||||||||||||||
Sbjct  1329  AAGCCTCAGTGTAAAGTCTGCCGATCATGCCCTGTGGTGCAGTCGAGCCA  1378


>dbj|AK122956.1| Homo sapiens cDNA FLJ16674 fis, clone THYMU3008136, highly  similar 
to Methionyl-tRNA synthetase (EC 6.1.1.10)
Length=3574

 Score = 93.5 bits (50),  Expect = 6e-17
 Identities = 50/50 (100%), Gaps = 0/50 (0%)
 Strand=Plus/Plus

Query  1     AAGCCTCAGTGTAAAGTCTGCCGATCATGCCCTGTGGTGCAGTCGAGCCA  50
             ||||||||||||||||||||||||||||||||||||||||||||||||||
Sbjct  1980  AAGCCTCAGTGTAAAGTCTGCCGATCATGCCCTGTGGTGCAGTCGAGCCA  2029


>gb|BT007338.1| Homo sapiens methionine-tRNA synthetase mRNA, complete cds
Length=2703

 Score = 93.5 bits (50),  Expect = 6e-17
 Identities = 50/50 (100%), Gaps = 0/50 (0%)
 Strand=Plus/Plus

Query  1     AAGCCTCAGTGTAAAGTCTGCCGATCATGCCCTGTGGTGCAGTCGAGCCA  50
             ||||||||||||||||||||||||||||||||||||||||||||||||||
Sbjct  1294  AAGCCTCAGTGTAAAGTCTGCCGATCATGCCCTGTGGTGCAGTCGAGCCA  1343


>dbj|AK222767.1| Homo sapiens mRNA for methionine-tRNA synthetase variant, clone: 
HEP00371
Length=2738

 Score = 93.5 bits (50),  Expect = 6e-17
 Identities = 50/50 (100%), Gaps = 0/50 (0%)
 Strand=Plus/Plus

Query  1     AAGCCTCAGTGTAAAGTCTGCCGATCATGCCCTGTGGTGCAGTCGAGCCA  50
             ||||||||||||||||||||||||||||||||||||||||||||||||||
Sbjct  1314  AAGCCTCAGTGTAAAGTCTGCCGATCATGCCCTGTGGTGCAGTCGAGCCA  1363


>gb|BC015011.1| Homo sapiens methionyl-tRNA synthetase, mRNA (cDNA clone IMAGE:4864644), 
partial cds
Length=2790

 Score = 93.5 bits (50),  Expect = 6e-17
 Identities = 50/50 (100%), Gaps = 0/50 (0%)
 Strand=Plus/Plus

Query  1     AAGCCTCAGTGTAAAGTCTGCCGATCATGCCCTGTGGTGCAGTCGAGCCA  50
             ||||||||||||||||||||||||||||||||||||||||||||||||||
Sbjct  1292  AAGCCTCAGTGTAAAGTCTGCCGATCATGCCCTGTGGTGCAGTCGAGCCA  1341


>gb|AC022506.38| Homo sapiens 12 BAC RP11-181L23 (Roswell Park Cancer Institute 
Human BAC Library) complete sequence
Length=172136

 Score = 93.5 bits (50),  Expect = 6e-17
 Identities = 50/50 (100%), Gaps = 0/50 (0%)
 Strand=Plus/Minus

Query  1       AAGCCTCAGTGTAAAGTCTGCCGATCATGCCCTGTGGTGCAGTCGAGCCA  50
               ||||||||||||||||||||||||||||||||||||||||||||||||||
Sbjct  103861  AAGCCTCAGTGTAAAGTCTGCCGATCATGCCCTGTGGTGCAGTCGAGCCA  103812


>emb|X94754.1| H.sapiens mRNA for yeast methionyl-tRNA synthetase homologue
Length=2779

 Score = 93.5 bits (50),  Expect = 6e-17
 Identities = 50/50 (100%), Gaps = 0/50 (0%)
 Strand=Plus/Plus

Query  1     AAGCCTCAGTGTAAAGTCTGCCGATCATGCCCTGTGGTGCAGTCGAGCCA  50
             ||||||||||||||||||||||||||||||||||||||||||||||||||
Sbjct  1301  AAGCCTCAGTGTAAAGTCTGCCGATCATGCCCTGTGGTGCAGTCGAGCCA  1350


>emb|Z49216.1| H.sapiens mitoxantrone-resistance associated mRNA
Length=1550

 Score = 93.5 bits (50),  Expect = 6e-17
 Identities = 50/50 (100%), Gaps = 0/50 (0%)
 Strand=Plus/Plus

Query  1   AAGCCTCAGTGTAAAGTCTGCCGATCATGCCCTGTGGTGCAGTCGAGCCA  50
           ||||||||||||||||||||||||||||||||||||||||||||||||||
Sbjct  28  AAGCCTCAGTGTAAAGTCTGCCGATCATGCCCTGTGGTGCAGTCGAGCCA  77


>gb|AY889906.1| Synthetic construct Homo sapiens clone FLH022775.01X methionine-tRNA 
synthetase (MARS) mRNA, complete cds
Length=2703

 Score = 93.5 bits (50),  Expect = 6e-17
 Identities = 50/50 (100%), Gaps = 0/50 (0%)
 Strand=Plus/Plus

Query  1     AAGCCTCAGTGTAAAGTCTGCCGATCATGCCCTGTGGTGCAGTCGAGCCA  50
             ||||||||||||||||||||||||||||||||||||||||||||||||||
Sbjct  1294  AAGCCTCAGTGTAAAGTCTGCCGATCATGCCCTGTGGTGCAGTCGAGCCA  1343


>gb|BC011849.2| Homo sapiens methionyl-tRNA synthetase, mRNA (cDNA clone MGC:20313 
IMAGE:4135154), complete cds
Length=2800

 Score = 93.5 bits (50),  Expect = 6e-17
 Identities = 50/50 (100%), Gaps = 0/50 (0%)
 Strand=Plus/Plus

Query  1     AAGCCTCAGTGTAAAGTCTGCCGATCATGCCCTGTGGTGCAGTCGAGCCA  50
             ||||||||||||||||||||||||||||||||||||||||||||||||||
Sbjct  1300  AAGCCTCAGTGTAAAGTCTGCCGATCATGCCCTGTGGTGCAGTCGAGCCA  1349


>gb|BC011639.2| Homo sapiens methionyl-tRNA synthetase, mRNA (cDNA clone MGC:12828 
IMAGE:4120680), complete cds
Length=2799

 Score = 93.5 bits (50),  Expect = 6e-17
 Identities = 50/50 (100%), Gaps = 0/50 (0%)
 Strand=Plus/Plus

Query  1     AAGCCTCAGTGTAAAGTCTGCCGATCATGCCCTGTGGTGCAGTCGAGCCA  50
             ||||||||||||||||||||||||||||||||||||||||||||||||||
Sbjct  1302  AAGCCTCAGTGTAAAGTCTGCCGATCATGCCCTGTGGTGCAGTCGAGCCA  1351


>gb|BC006328.2| Homo sapiens methionyl-tRNA synthetase, mRNA (cDNA clone MGC:12889 
IMAGE:4139330), complete cds
Length=2795

 Score = 93.5 bits (50),  Expect = 6e-17
 Identities = 50/50 (100%), Gaps = 0/50 (0%)
 Strand=Plus/Plus

Query  1     AAGCCTCAGTGTAAAGTCTGCCGATCATGCCCTGTGGTGCAGTCGAGCCA  50
             ||||||||||||||||||||||||||||||||||||||||||||||||||
Sbjct  1300  AAGCCTCAGTGTAAAGTCTGCCGATCATGCCCTGTGGTGCAGTCGAGCCA  1349


>gb|BC002384.2| Homo sapiens methionyl-tRNA synthetase, mRNA (cDNA clone MGC:8646 
IMAGE:2961615), complete cds
Length=2799

 Score = 93.5 bits (50),  Expect = 6e-17
 Identities = 50/50 (100%), Gaps = 0/50 (0%)
 Strand=Plus/Plus

Query  1     AAGCCTCAGTGTAAAGTCTGCCGATCATGCCCTGTGGTGCAGTCGAGCCA  50
             ||||||||||||||||||||||||||||||||||||||||||||||||||
Sbjct  1300  AAGCCTCAGTGTAAAGTCTGCCGATCATGCCCTGTGGTGCAGTCGAGCCA  1349


>dbj|D84224.1| Homo sapiens mRNA for methionyl tRNA synthetase, complete cds
Length=2800

 Score = 93.5 bits (50),  Expect = 6e-17
 Identities = 50/50 (100%), Gaps = 0/50 (0%)
 Strand=Plus/Plus

Query  1     AAGCCTCAGTGTAAAGTCTGCCGATCATGCCCTGTGGTGCAGTCGAGCCA  50
             ||||||||||||||||||||||||||||||||||||||||||||||||||
Sbjct  1317  AAGCCTCAGTGTAAAGTCTGCCGATCATGCCCTGTGGTGCAGTCGAGCCA  1366


>ref|XM_003824853.1| PREDICTED: Pan paniscus methionyl-tRNA synthetase (MARS), mRNA
Length=3665

 Score = 87.9 bits (47),  Expect = 3e-15
 Identities = 49/50 (98%), Gaps = 0/50 (0%)
 Strand=Plus/Plus

Query  1     AAGCCTCAGTGTAAAGTCTGCCGATCATGCCCTGTGGTGCAGTCGAGCCA  50
             |||||||||||||||||||||||||||||||||||||||||||||| |||
Sbjct  1985  AAGCCTCAGTGTAAAGTCTGCCGATCATGCCCTGTGGTGCAGTCGACCCA  2034


>ref|XM_509164.3| PREDICTED: Pan troglodytes methionyl-tRNA synthetase (MARS), 
mRNA
Length=3469

 Score = 87.9 bits (47),  Expect = 3e-15
 Identities = 49/50 (98%), Gaps = 0/50 (0%)
 Strand=Plus/Plus

Query  1     AAGCCTCAGTGTAAAGTCTGCCGATCATGCCCTGTGGTGCAGTCGAGCCA  50
             |||||||||||||||||||||||||||||||||||||||||||||| |||
Sbjct  1985  AAGCCTCAGTGTAAAGTCTGCCGATCATGCCCTGTGGTGCAGTCGACCCA  2034


>dbj|AK303349.1| Homo sapiens cDNA FLJ55046 complete cds, highly similar to Methionyl-tRNA 
synthetase (EC 6.1.1.10)
Length=1328

 Score = 87.9 bits (47),  Expect = 3e-15
 Identities = 49/50 (98%), Gaps = 0/50 (0%)
 Strand=Plus/Plus

Query  1    AAGCCTCAGTGTAAAGTCTGCCGATCATGCCCTGTGGTGCAGTCGAGCCA  50
            |||||||||||| |||||||||||||||||||||||||||||||||||||
Sbjct  936  AAGCCTCAGTGTGAAGTCTGCCGATCATGCCCTGTGGTGCAGTCGAGCCA  985


>gb|AC191734.3| Pan troglodytes BAC clone CH251-555H2 from chromosome 12, complete 
sequence
Length=223618

 Score = 87.9 bits (47),  Expect = 3e-15
 Identities = 49/50 (98%), Gaps = 0/50 (0%)
 Strand=Plus/Minus

Query  1      AAGCCTCAGTGTAAAGTCTGCCGATCATGCCCTGTGGTGCAGTCGAGCCA  50
              |||||||||||||||||||||||||||||||||||||||||||||| |||
Sbjct  76106  AAGCCTCAGTGTAAAGTCTGCCGATCATGCCCTGTGGTGCAGTCGACCCA  76057


>ref|NM_001266948.1| Macaca mulatta methionyl-tRNA synthetase (MARS), mRNA
Length=2786

 Score = 82.4 bits (44),  Expect = 1e-13
 Identities = 48/50 (96%), Gaps = 0/50 (0%)
 Strand=Plus/Plus

Query  1     AAGCCTCAGTGTAAAGTCTGCCGATCATGCCCTGTGGTGCAGTCGAGCCA  50
             |||||||||||||||||||||||||||||||| ||||||||||||| |||
Sbjct  1326  AAGCCTCAGTGTAAAGTCTGCCGATCATGCCCCGTGGTGCAGTCGACCCA  1375


>ref|XM_003252767.1| PREDICTED: Nomascus leucogenys methionyl-tRNA synthetase (MARS), 
mRNA
Length=2916

 Score = 82.4 bits (44),  Expect = 1e-13
 Identities = 48/50 (96%), Gaps = 0/50 (0%)
 Strand=Plus/Plus

Query  1     AAGCCTCAGTGTAAAGTCTGCCGATCATGCCCTGTGGTGCAGTCGAGCCA  50
             ||||||||||||||||||||||||||||| |||||||||||||||| |||
Sbjct  1432  AAGCCTCAGTGTAAAGTCTGCCGATCATGTCCTGTGGTGCAGTCGACCCA  1481


>ref|XM_002752687.2| PREDICTED: Callithrix jacchus methionyl-tRNA synthetase (MARS), 
mRNA
Length=2658

 Score = 78.7 bits (42),  Expect = 2e-12
 Identities = 44/45 (97%), Gaps = 0/45 (0%)
 Strand=Plus/Plus

Query  1     AAGCCTCAGTGTAAAGTCTGCCGATCATGCCCTGTGGTGCAGTCG  45
             ||||||||||||||||||||||||||||||||||||||| |||||
Sbjct  1150  AAGCCTCAGTGTAAAGTCTGCCGATCATGCCCTGTGGTGAAGTCG  1194


Lambda     K      H
    1.33    0.621     1.12 

Gapped
Lambda     K      H
    1.28    0.460    0.850 

Effective search space used: 874911716973
 
    reads 
 chr12 chr12 57824816 57824740 76m                            GGGGGGCGGACTCTGGGCTTTTCTTCCCCTTCTGATTTTCCCCTCCCCCCTTTTCTCCCTTT
chr12 chr12 57824806 57824730 76m                            GGGGGGCGGACTCTGGGCTTTTCTTCCCCTTCTGATTTTCCCCTCCCCCCTTTTCTCCCTTTCCGGGTTTGG
chr12 chr12 57824803 57824727 76m                            GGGGGGCGGACTCTGGGCTTTTCTTCCCCTTCTGATTTTCCCCTCCCCCCTTTTCTCCCTCTCCGGGCTTGGTTC
chr12 chr12 57824797 57824721 76m                                 GCGGACTCTGGGCTTTTCTTCCCCTTCTGATTTTCCCCTCCCCCATTTTCTCCCTTTCCGGGTTTGGTTCCCCCCT
chr12 chr12 57824790 57824714 76m                                        CTGGGCTTTTCTTCCCCTTCTGATTTTCCCCTCCCCCCTTTTCTCCCTTTCCGGGTTTGGTTCCCCCCTTTTCTTT
chr12 chr12 57824787 57824711 76m                                           TGCTTTTCTTCCCCTTCTGATTTTCCCCTCCCCCCTTTTCTCCCTTTCCGGGTTTGGTTCCCCCCTTTTCTTCCCC
chr12 chr12 57824784 57824708 76m                                              TTTTCTTCCCCTTCTGATTTTCCCCTCCCCCCTTTTCTCCCTTTCCGGGTTTGGTTCCCTCCTTTTCTTTCCCTCT
chr12 chr12 57824780 57824704 76m                                                  CTTCCCCTTCTGATTTTCCCCTCCCCCCTTTTATCCCTTTCCGGGTTTGGTTCCCCCCTTTTTTTCCCCTCTCCCC
chr12 chr12 57824772 57824696 76m                                                          TCTGATTTTCCCCTCCCCCCTTTTCTCCCTTTCCGGGTTTGGTTCCCCCCTTTTCTTCCCCTCTCCCCCCTCCTTC
chr12 chr12 57824769 57824693 76m                                                             CTTTTTCCCCTCCCCCCTTTTCTCCCTTTCCGGGTTTGGTTCCCCCCTTTTCTTCCCCTCTCCCCCCTCCTTCCCA
chr12 chr12 57824766 57824690 76m                                                                TTTCCCCTCCCCCCTTTTCTCCCTTTCCGGGTTTGGTTCCCCCCTTTTCTTCCCCTCTCCCCCCTCCTTCCCAACC
chr12 chr12 57824762 57824686 76m                                                                    CCCTCCCCCCTTTTCTCCCTTTCCGGGTTTGGTTCCCCCCTTTTCTTCCCCTCTCCCCCCTCCTTCCCCAGCCCCT
chr12 chr12 57824759 57824683 76m                                                                       TCCCCCCTTTTCTCCCTTTCCGGGTTTGGTTCCCCCCTTTTCTTCCCCTCGCCCCCCTCCTTCCCAAGCCCCTTTC
chr12 chr12 57824756 57824680 76m                                                                          CCCCTTTTCTCCCTTTCCGGGTTTGGTTCCCCCCTTTTCTTCCCCTCTCCCCCCTCCTTCCCAAGCCCCTTTCCCC
chr12 chr12 57824753 57824677 76m                                                                             CTTTTCTCCCTTTCCGGGTTTGGTTCCCCCCTTTTCTTCCCCTCTCCCCCCTCCTTCCCCAGCCCCTTTCCCCTCC
chr12 chr12 57824749 57824673 76m                                                                                 TCTCCCTTTCCGGGTTTGGTTCCCCCCTTTTCTTCCCCTCTCCCCCCTCCTTCCCAAGCCCCTTTCCCCTCCCCAG
chr12 chr12 57824745 57824669 76m                                                                                     CCTTTCCGGGTTTGGTTCCCCCCTTTTCTTCCCCTCTCCCCCCTCCTTCCCAAGCCCCTTTCCCCTCCCCCGTCCC
chr12 chr12 57824742 57824666 76m                                                                                        TTCCGGGTTTGGTTCCCCCCTTTTCTTCCCCTCTCCCCCCTCCTTCCCAAGCCCCTTTCCCCTCCCCCGTCCCCGT
chr12 chr12 57824739 57824663 76m                                                                                           CGGGTTTGGTTCCCCCCTTTTCTTCCCCTCTCCCCCCTCCTTCCCAAGCCCCTTTCCCCTCCCCCGTCCCAGTCCC
chr12 chr12 57824736 57824660 76m                                                                                              GTTTGGTTCCCCCCTTTTCTTCCCCTCTCCCCCCTCCTTCCCAAGCCCCTTTCCCCTCCCCCGTCCCAGTCCCCCC
chr12 chr12 57824733 57824657 76m                                                                                                 TGGTTCCCCCCTTTTCTTCCCCTCTCCCCCCCCCTTCCCAAGCCCCTTTCCCCTCCCCCGTCCCAGTCCCCCCTGC
chr12 chr12 57824730 57824654 76m                                                                                                    TTCCCCCCTTTTCTTCCCCTCTCCCCCCTCCTTCCCAAGCCCCTTTCCCCTCCCCCGTCCCAGTCCCCCCTGCTTC
chr12 chr12 57824727 57824651 76m                                                                                                       CCCCCTTTTCTTCCCCTCTCCCCCCTCCTTCCCAAGCCCCTTTCCCCTCCCCCGTCCCAGTCCCCCCTGCTTCCCA
chr12 chr12 57824723 57824647 76m                                                                                                           CTTTTCTTCCCCTCTCCCCCCTCCTTCCCAAGCCCCTTTCCCCTCCCCCGTCCCCGTCCCCCCTGCTTCCCAGCCC
chr12 chr12 57824719 57824643 76m                                                                                                               TCTTCCCCTCTCCCCCCTCCTTCCCAAGCCCCTTTCCCCTCCCCCGTCCCAGTCCCCCCTGCTTCCCAGCCCTCGG
chr12 chr12 57824711 57824635 76m                                                                                                                       TCTCCCCCCTCCTTCCCAAGCCCCTTTCCCCTCCCCCGTCCCAGTCCCCCCCGCTTCCCAGCCCTCGGCCCCGGGG
chr12 chr12 57824704 57824628 76m                                                                                                                              CCTCCTTCCCAAGCCCCTTTCCCCTCCCCCGTCCCAGTCCCCCCTGCTTCCCAGCCCTCGGCCCCGGGGAAGCCCC
chr12 chr12 57824701 57824625 76m                                                                                                                                 CCTTCCCAAGCCCCTTTCCCCTCCCCCGTCCCAGTCCCCCCTGCTTCCCAGCCCTCTGCACCGGGGAAGCCCCGGC
chr12 chr12 57824698 57824622 76m                                                                                                                                    TCCCAAGCCCCTTTCCCCTCCCCCGTCCCAGTCCCCCCTGCTTCCCAGCCCTCGGCCCCGGGGAAGCCCCGGCCGA
chr12 chr12 57824695 57824619 76m                                                                                                                                       CAAGCCCCTTTCCCCTCCCCCGTCCCAGTCCCCCCTGCTTCCCAGCCCTCGGCCCCGGGGAAGCCCCGGCCGAGAA
chr12 chr12 57824692 57824616 76m                                                                                                                                          GCCCCTTTCCCCTCCCCCGTCCCAGTCCCCCCTGCTTCCCAGCCCTCGGCCCCGGGGAAGCCCCGGCCGAGAAAAG
chr12 chr12 57824689 57824613 76m                                                                                                                                             CCTTTCCCCTCCCCCGTCCCAGTCCCCCCTGCTTCCCAGCCCTCGGCCCCGGGGAAGCCCCGGCCGAGAAAAGCCC
chr12 chr12 57824686 57824610 76m                                                                                                                                                TTCCCCTCCCCCGTCCCAGTCCCCCCTGCTTCCCAGCCCTCGGCCCCGGGGAAGCCCCGGCCGAGAAAAGCCCGCC
chr12 chr12 57824683 57824607 76m                                                                                                                                                   CCCTCCCCCGTCCCAGTCCCCCCTGCTTCCCAGCCCTCGGCCCCGGGGAAGCCCCGGCCGAGAAAAGCCCGCCCCC
chr12 chr12 57824680 57824604 76m                                                                                                                                                      TCCCCCGTCCCAGTCCCCCCTGCTTCCCAGCCCTCGGCCCCGGGGAAGCCCCGGCCGAGAAAAGCCCGCCCCCAGA
chr12 chr12 57824676 57824600 76m                                                                                                                                                          CCGTCCCAGTCCCCCCTGCTTCCCAGCCCTCGGCCCCGGGGAAGCCCCGGCCGAGAAAAGCCCGCCCCCAGAAGCG
chr12 chr12 57824673 57824597 76m                                                                                                                                                             TCCCAGTCCCCCCTGCTTCCCAGCCCTCGGCCCCGGGGAAGCCCCGGCCGAGAAAAGCCCGCCCCCAGAAGCGCGC
chr12 chr12 57824668 57824592 76m                                                                                                                                                                  GTCCCCCCTGCTTCCCAGCCCTCGGCCCCGGGGAAGCCCCGGCCGAGAAAAGCCCGCCCCCAGAAGCGCGCCGCCG
chr12 chr12 57824664 57824588 76m                                                                                                                                                                      CCCCTGCTTCCCAGCCCTCGGCCCCGGGGAAGCCCCGGCCGAGAAAAGCCCGCCCCCAGAAGCGCGCCGCCGCCGG
chr12 chr12 57824661 57824585 76m                                                                                                                                                                         CTGCTTCCCAGCCCTCGGCCCCGGGGAAGCCCCGGCCGAGAAAAGCCCGCCCCCAGAAGCGCGCCGCCGCCGGCCG
chr12 chr12 57824658 57824582 76m                                                                                                                                                                            CTTCCCAGCCCTCGGCCCCGGGGAAGCCCCGGCCGAGAAAAGCCCGCCCCCAGAAGCGCGCCGCCGCCGGCCGTCG
chr12 chr12 57824655 57824579 76m                                                                                                                                                                               CCCAGCCCTCGGCCCCGGGGAAGCCCCGGCCGAGAAAAGCCCGCCCCCAGAAGCGCGCCGCCGCCGGCCGTCGGGG
chr12 chr12 57824652 57824576 76m                                                                                                                                                                                  AGCCCTCGGCCCCGGGGAAGCCCCGGCCGAGAAAAGCCCGCCCCCAGAAGCGCGCCGCCGCCGGCCGTCGGGGCCG
chr12 chr12 57824648 57824572 76m                                                                                                                                                                                      CTCGGCCCCGGGGAAGCCCCGGCCGAGAAAAGCCCGCCCCCAGAAGCGCGCCGCCGCCGGCCGTCGGGGCCGAGCC
chr12 chr12 57824581 57824505 76m                                                                                                                                                                                                                                                         GGCCGAGCCGCAGCCGAGCGGCCGTGGGCCCGGGCGGCGGGCGGAGACGTGGGCGCCCTCCCCGCCAAGGCCCGTG
chr12 chr12 57824578 57824502 76m                                                                                                                                                                                                                                                            CGAGCCGCAGCCGAGCGGCCGTGGGCCCGGGCGGCGGGCGGAGACGTGGGCGCCCTCCCCGCCAAGGCCCGTGCGA
chr12 chr12 57824575 57824499 76m                                                                                                                                                                                                                                                               GCCGCAGCCGAGCGGCCGTGGGCCCGGGCGGCGGGCGGAGACGTGGGCGCCCTCCCCGCCAAGGCCCGTGCGAGAG
chr12 chr12 57824573 57898011 72m57898008F4M                                                                                                                                                                                                                                                      CGCAGCCGAGCGGCCGTGGGCCCGGGCGGCGGGCGGAGACGTGGGCGCCCTCCCCGCCAAGGCCCGTGCGAG AAGC
chr12 chr12 57824573 57898011 72m57898008F4M                                                                                                                                                                                                                                                      CGCAGCCGAGCGGCCGTGGGCCCGGGCGGCGGGCGGAGACGTGGGCGCCCTCCCCGCCAAGGCCCGTGCGAG AAGC
chr12 chr12 57824573 57898011 72m57898008F4M                                                                                                                                                                                                                                                      CGCAGCCGAGCGGCCGTGGGGCCGGGCGGCGGGCGGAGACGTGGGCGCCCTCCCCGCCAAGGCCCGTGCGAG AAGC
chr12 chr12 57824571 57898013 70m57898008F6M                                                                                                                                                                                                                                                        CAGCCGAGCGGCCGTGGGCCCGGGCGGCGGGCGGAGACGTGGGCGCCCTCCCCGCCAAGGCCCGTGCGAG AAGCCT
chr12 chr12 57824571 57898013 70m57898008F6M                                                                                                                                                                                                                                                        CAGCCGAGCGGCCGTGGGCCCGGGCGGCGGGCGGAGACGTGGGCGCCCTCCCCGCCAAGGCCCGTGCGAG AAGCCT
chr12 chr12 57824570 57898014 69m57898008F7M                                                                                                                                                                                                                                                         AGCCGAGCGGCCGTGGGCCCGGGCGGCGGGCGGAGACGTGGGCGCCCTCCCCGCCAAGGCCCGTGCGAG AAGCCTC
chr12 chr12 57824570 57898014 69m57898008F7M                                                                                                                                                                                                                                                         AGCCGAGCGGCCGTGGGCCCGGGCGGCGGGCGGAGCCGTGGGCGCCCTCCCCGCCAAGGCCCGTGCGAG AAGCCTC
chr12 chr12 57824569 57898015 68m57898008F8M                                                                                                                                                                                                                                                          GCCGAGCGGCCGTGGGCCCGGGCGGCGGGCGGAGACGTGGGCGCCCTCCCCGCCAAGGCCCGTGCGAG AAGCCTCA
chr12 chr12 57824569 57898015 68m57898008F8M                                                                                                                                                                                                                                                          GCCGAGCGGCCGTGGGCCCGGGCGGCGGGCGGAGACGTGGGCGCCCTCCCCGCCAAGGCCCGTGCGAG AAGCCTCA
chr12 chr12 57824568 57898016 67m57898008F9M                                                                                                                                                                                                                                                           CCGAGCGGCCGTGGGCCCGGGCGGCGGGCGGAGACGTGGGCGCCCTCCCCGCCAAGGCCCGTGCGAG AAGCCTCAG
chr12 chr12 57824568 57898016 67m57898008F9M                                                                                                                                                                                                                                                           CCGAGCGGCCGTGGGCCCGGGCGGCGGGCGGAGACGTGGGCGCCCTCCCCGCCAAGGCCCGTGCGAG AAGCCTCAG
chr12 chr12 57824568 57898016 67m57898008F9M                                                                                                                                                                                                                                                           CCGAGCGGCCGTGGGCCCGGGCGGCGGGCGGAGACGTGGGCGCCCTCCCCGCCAAGGTCCGGGCGAG AAGCCTCAG
chr12 chr12 57824568 57898016 67m57898008F9M                                                                                                                                                                                                                                                           CCGAGCGGCCGTGGGCCCGGGCGGCGGGCGGAGACGTGGGCGCCCTCCCCGCCAAGGCCCGTGCGAG AAGCCTCAG
chr12 chr12 57824568 57898016 67m57898008F9M                                                                                                                                                                                                                                                           CCGAGCGGCCGTGGGCCCGGGCGGCGGGCGGAGACGTGGGCGCCCTCCCCGCCAAGGCCCGTGCGAG AAGCCTCAG
chr12 chr12 57824567 57898017 66m57898008F10M                                                                                                                                                                                                                                                           CGAGCGGCCGTGGGCCCGGGCGGCGGGCGGAGACGTGGGCGCCCTCCCCGCCAAGGCCCGTGCGAG AAGCCTCAGT
chr12 chr12 57824567 57898017 66m57898008F10M                                                                                                                                                                                                                                                           CGAGCGGCCGTGGGCCCGGGCGGCGGGCGGAGACGTGGGCGCCCTCCCCGCCAAGGCCCGTGCGAG AAGCCTCAGT
chr12 chr12 57824567 57898017 66m57898008F10M                                                                                                                                                                                                                                                           CGAGCGGCCGTGGGCCCGGGCGGCGGGCGGAGACGTGGGCGCCCTCCCCGCCAAGGCCCGTGCGAG AAGCCTCAGT
chr12 chr12 57824567 57898017 66m57898008F10M                                                                                                                                                                                                                                                           CGAGCGGCCGTGGGCCCGGGCGGCGGGCGGAGACGTGGGCGCCCTCCCCGCCAAGGCCCGTGCGAG AAGCCTCAGG
chr12 chr12 57824567 57898017 66m57898008F10M                                                                                                                                                                                                                                                           CGAGCGGCCGTGGGCCCGGGCGGCGGGCGGAGACGTGGGCGCCCTCCCCGCCAAGGCCCGTGCGAG AAGCCTCAGT
chr12 chr12 57824567 57898017 66m57898008F10M                                                                                                                                                                                                                                                           CGAGCGGCCGTGGGCCCGGGCGGCGGGCGGAGACGTGGGCGCCCTCCCCGCCAAGGCCCGTGCGAG AAGCCTCAGT
chr12 chr12 57824566 57898018 65m57898008F11M                                                                                                                                                                                                                                                            GAGCGGCCGTGGGCCCGGGCGGCGGGCGGAGACGTGGGCGCCCTCCCCGCCAAGGCCCGTGCGAG AAGCCTCAGTG
chr12 chr12 57824566 57898018 65m57898008F11M                                                                                                                                                                                                                                                            GAGCGGCCGTGGGCCCGGGCGGCGGGCGGAGACGTGGGCGCCCTCCCCGCCAAGGCCCGTGCGAG AAGCCTCAGTG
chr12 chr12 57824566 57898018 65m57898008F11M                                                                                                                                                                                                                                                            GAGCGGCCGTGGGCCCGGGCGGCGGGCGGAGACGTGGGCGCCCTCCCCGCCAAGGCCCGTGCGAG AAGCCTCAGTG
chr12 chr12 57824564 57898020 63m57898008F13M                                                                                                                                                                                                                                                              GCGGCCGTGGGCCCGGGCGGCGGGCGGAGACGTGGGCGCCCTCCCCGCCAAGGCCCGTGCGAG AAGCCTCAGTGTA
chr12 chr12 57824564 57898020 63m57898008F13M                                                                                                                                                                                                                                                              GCGGCCGTGGGCCCGGGCGGCGGGCGGAGACGTGGGCGCCCTCCCCGCCAAGGCCCGTGCGAG AAGCCTCAGTGTA
chr12 chr12 57824564 57898020 63m57898008F13M                                                                                                                                                                                                                                                              GCGGCCGTGGGCCCGGGCGGCGGGCGGAGACGTGGGCGCCCTCCCCGCCAAGGCCCGTGCGAG AAGCCTCAGTGTA
chr12 chr12 57824564 57898020 63m57898008F13M                                                                                                                                                                                                                                                              GCGGCCGTGGGCCCGGGCGGCGGGCGGAGACGTGGGCGCCCTCCCCGCCAAGGCCCGTGCGAG AAGCCTCAGTGTA
chr12 chr12 57824564 57898020 63m57898008F13M                                                                                                                                                                                                                                                              GCGGCCGTGGGCCCGGGCGGCGGGCGGAGACGTGGGCGCCCTCCCCGCCAAGGCCCGTGCGAG AAGCCTCAGTGTA
chr12 chr12 57824564 57898020 63m57898008F13M                                                                                                                                                                                                                                                              GCGGCCGTGGGCCCGGGCGGCGGGCGGAGACGTGGGCGCCCTCCCCGCCAAGGCCCGTGCGAG AAGCCTCAGTGTA
chr12 chr12 57824564 57898020 63m57898008F13M                                                                                                                                                                                                                                                              GCGGCCGTGGGCCCGGGCGGCGGGCGGAGACGTGGGCGCCCTCCCCGCCAAGGCCCGTGCGAG AAGCCTCAGTGTA
chr12 chr12 57824563 57898021 62m57898008F14M                                                                                                                                                                                                                                                               CGGCCGTGGGCCCGGGCGGCGGGCGGAGACGTGGGCGCCCTCCCCGCCAAGGCCCGTGCGAG AAGCCTCAGTGTAA
chr12 chr12 57824563 57898021 62m57898008F14M                                                                                                                                                                                                                                                               CGGCCGTGGGCCCGGGCGGCGGGCGGAGACGTGGGCGCCCTCCCCGCCAAGGCCCGTGCGAG AAGCCTCAGTGTAA
chr12 chr12 57824563 57898021 62m57898008F14M                                                                                                                                                                                                                                                               CGGCCGTGGGCCCGGGCGGCGGGCGGAGACGTGGGCGCCCTCCCCGCCAAGGCCCGTGCGAG AAGCCTCAGTGTAA
chr12 chr12 57824563 57898021 62m57898008F14M                                                                                                                                                                                                                                                               CGGCCGTGGGCCCGGGCGGCGGGCGGAGACGTGGGCGCCCTCCCCGCCCAGGCCCGTGCGAG AAGCCTCAGTGTAA
chr12 chr12 57824563 57898021 62m57898008F14M                                                                                                                                                                                                                                                               CGGCCGTGGGCCCGGGCGGCGGGCGGAGACGTGGGCGCCCTCCCCGCCAAGGCCCGTGCGAG AAGCCTCAGTGTAA
chr12 chr12 57824563 57898021 62m57898008F14M                                                                                                                                                                                                                                                               CGGCCGTGGGCCCGGGCGGCGGGCGGAGACGTGGGCGCCCTCCCCGCCAAGGCCCGTGCGAG AAGCCTCAGTGTAA
chr12 chr12 57824563 57898021 62m57898008F14M                                                                                                                                                                                                                                                               CGGCCGTGGGCCCGGGCGGCGGGCGGAGACGTGGGCGCCCTCCCCGCCAAGGCCCGTGCGAG AAGCCTCAGTGTAA
chr12 chr12 57824563 57898021 62m57898008F14M                                                                                                                                                                                                                                                               CGGCCGTGGGCCCGGGCGGCGGGCGGAGACGTGGGCGCCCTCCCCGCCAAGGCCCGTGCGAG AAGCCTCAGTGTAA
chr12 chr12 57824562 57898022 61m57898008F15M                                                                                                                                                                                                                                                                GGCCGTGGGCCCGGGCGGCGGGCGGAGACGTGGGCGCCCTCCCCGCCAAGGCCCGTGCGAG AAGCCTCAGTGTAAA
chr12 chr12 57824561 57898023 60m57898008F16M                                                                                                                                                                                                                                                                 GCCGTGGGCCCGGGCGGCGGGCGGAGACGTGGGCGCCCTCCCCGCCAAGGCCCGTGCGAG AAGCCTCAGTGTAAAG
chr12 chr12 57824561 57898023 60m57898008F16M                                                                                                                                                                                                                                                                 GCCGTGGGCCCGGGCGGCGGGCGGAGACGTGGGCGCCCTCCCCGCCAAGGCCCGTGCGAG AAGCCTCAGTGTAAAG
chr12 chr12 57824561 57898023 60m57898008F16M                                                                                                                                                                                                                                                                 GCCGTGGGCCCGGGCGGCGGGCGGAGACGTGGGCGCCCTCCCCGCCCAGGCCCGTGCGAG AAGCCTCAGTGTAAAG
chr12 chr12 57824560 57898024 59m57898008F17M                                                                                                                                                                                                                                                                  CCGTGGGCCCGGGCGGCGGGCGGAGACGTGGGCGACCTCCCCGCCAAGGCCCGTGCGAG AAGCCTCAGTGTAAAGT
chr12 chr12 57824559 57898025 58m57898008F18M                                                                                                                                                                                                                                                                   CGTGGGCCCGGGCGGCGGGCGGAGACGTGGGCGCCCTCCCCGCCAAGGCCCGGGCGAG AAGCCTCAGTGTAAAGTC
chr12 chr12 57824559 57898025 58m57898008F18M                                                                                                                                                                                                                                                                   CGTGGGCCCGGGCGGCGGGCGGAGACGTGGGCGCCCTCCCCGCCAAGGCCCGTGCGAG AAGCCTCAGTGTAAAGTC
chr12 chr12 57824559 57898025 58m57898008F18M                                                                                                                                                                                                                                                                   CGTGGGCCCGGGCGGCGGGCGGAGACGTGGGCGCCCTCCCCGCCAAGGCCCGTGCGAG AAGCCTCAGGGTAAAGGC
chr12 chr12 57824559 57898025 58m57898008F18M                                                                                                                                                                                                                                                                   CGTGGGCCCGGGCGGCGGGCGGAGACGTGGGCGCCCTCCCCGCCAAGGCCCGTGCGAG AAGCCTCAGTGTAAAGTC
chr12 chr12 57824559 57898025 58m57898008F18M                                                                                                                                                                                                                                                                   CGTGGGCCCGGGCGGCGGGCGGAGACGTGGGCGCCCTCCCCGCCAAGGCCCGTGCGAG AAGCCTCAGTGTAAAGTC
chr12 chr12 57824559 57898025 58m57898008F18M                                                                                                                                                                                                                                                                   CGTGGGCCCGGGCGGCGGGCGGAGACGTGGGCGCCCTCCCCGCCAAGGCCCGTGCGAG AAGCCTCAGTGTAAAGTC
chr12 chr12 57824559 57898025 58m57898008F18M                                                                                                                                                                                                                                                                   CGTGGGCCCGGGCGGCGGGCGGAGACGTGGGCGCCCTCCCCGCCAAGGCCCGTGCGAG AAGCCTCAGTGTAAAGTC
chr12 chr12 57824558 57898026 57m57898008F19M                                                                                                                                                                                                                                                                    AGACTTTACACTGAGGCTTCTCGCACGGGCCTTGGCGGGGAGGGCGCCCACGTCTCC GCCCGCCGCCCGGGCCCAC
chr12 chr12 57824558 57898026 57m57898008F19M                                                                                                                                                                                                                                                                    GTGGGCCCGGGCGGCGGGCGGAGACGTGGGCGCCCTCCCCGCCAAGGCCCGTGCGAG AAGCCTCAGTGTAAAGTCT
chr12 chr12 57824558 57898026 57m57898008F19M                                                                                                                                                                                                                                                                    GTGGGCCCGGGCGGCGGGCGGAGACGTGGGCGCCCTCCCCGCCAAGGCCCGTGCGAG GAGCCTCAGTGTAAAGTCT
chr12 chr12 57824558 57898026 57m57898008F19M                                                                                                                                                                                                                                                                    GTGGGCCCGGGCGGCGGGCGGAGACGTGGGCGCCCTCCCCGCCAAGGCCCGTGCGAG AAGCCTCAGTGTAAAGTCT
chr12 chr12 57824558 57898026 57m57898008F19M                                                                                                                                                                                                                                                                    GTGGGCCCGGGCGGCGGGGGGAGACGTGGGGGCCCTCCCCGCCAAGGCCCGTGCGAG AAGCCTCAGTGTAAAGTCT
chr12 chr12 57824558 57898026 57m57898008F19M                                                                                                                                                                                                                                                                    GTGGGCCCGGGCGGCGGGCGGAGACGTGGGCGCCCTCCCCGCCAAGGCCCGTGCGAG AAGCCTCAGTGTAAAGTCT
chr12 chr12 57824558 57898026 57m57898008F19M                                                                                                                                                                                                                                                                    GTGGGCCCGGGCGGCGGGCGGAGACGTGGGCGCCCTCCCCGCCAAGGCCCGTGCGAG AAGCCTCAGTGTAAAGTCT
chr12 chr12 57824557 57898027 56m57898008F20M                                                                                                                                                                                                                                                                     TGGGCCCGGGCGGCGGGCGGAGACGTGGGCGCCCTCCCCGCCAAGGCCCGTGCGAG AAGCCTCAGTGTAAAGTCTG
chr12 chr12 57824556 57898028 55m57898008F21M                                                                                                                                                                                                                                                                      GGGCCCGGGCGGCGGGCGGAGACGTGGGCGCCCTCCCCGCCAAGGCCCGTGCGAG AAGCCTCAGTGTAAAGCCTGC
chr12 chr12 57824555 57898029 54m57898008F22M                                                                                                                                                                                                                                                                       GGCCCGGGCGGCGGGCGGAGACGTGGGGGCCCTCCCCGCCAAGGCCCGTGCGCG AAGCCTCAGTGTAAAGTCTGCC
chr12 chr12 57824555 57898029 54m57898008F22M                                                                                                                                                                                                                                                                       GGCAGACTTTACGCTGAGGCTTCTCGCACGGGCCTTGGCGGGGAGGGCGCCCAG GTCTCCGCCCGCCGCCCGGGCC
chr12 chr12 57824555 57898029 54m57898008F22M                                                                                                                                                                                                                                                                       GGCCCGGGCGGCGGGCGGAGACCTGGGCGCCCTCCCCGCCAAGGCCCGTGCGAG AAGCCTCAGCGTAAAGTCTGCC
chr12 chr12 57824555 57898029 54m57898008F22M                                                                                                                                                                                                                                                                       GGCCCGGGCGGCGGGCGGAGACGTGGGCGCCCTCCCCGCCAAGGCCCGTGCGAG AAGCCTCAGTGTAAAGTCTGCC
chr12 chr12 57824554 57898030 53m57898008F23M                                                                                                                                                                                                                                                                        GCCCGGGCGGCGGGCGGAGACGTGGGCGCCCTCCCCGCCAAGGCCCGTGCGAG AAGCCTCAGTGTAAAGTCTGCCG
chr12 chr12 57824554 57898030 53m57898008F23M                                                                                                                                                                                                                                                                        GCCCGGGCGGCGGGCGGAGACGTGGGCGCCCTCCCCGCCAAGGCCCGTGCGAG AAGCCTCAGTGTAAAGTCTGCCG
chr12 chr12 57824554 57898030 53m57898008F23M                                                                                                                                                                                                                                                                        GCCCGGTCGGCGGGCGGAGACGGGGGCGCCCTCCCCGCCAAGGCCCGTGCGAG AAGCCTCAGTGTAAAGTCTGCCG
chr12 chr12 57824554 57898030 53m57898008F23M                                                                                                                                                                                                                                                                        GCCCGGGCGGCGGGCGGAGACGTGGGCGCCCTCCCCGCCAAGGCCCGTGCGAG AAGCCTCAGTGTAAAGTCTGCCG
chr12 chr12 57824554 57898030 53m57898008F23M                                                                                                                                                                                                                                                                        GCCCGGGCGGCGGGCGGAGACGTGGGCGCCCTCCCCGCCAAGGCCCGTGCGAG AAGCCTCAGTGTAAAGTCTGCCG
chr12 chr12 57824554 57898030 53m57898008F23M                                                                                                                                                                                                                                                                        GCCCGGGCGGCGGGCGGAGACGTGGGCGCCCTCCCCGCCAAGGCCCGTGCGAG AAGCCTCAGTGTAAAGTCTGCCG
chr12 chr12 57824554 57898030 53m57898008F23M                                                                                                                                                                                                                                                                        GCCCGGGCGGCGGGCGGAGACGTGGGCGCCCTCCCCGCCAAGGCCCGTGCGAG AAGCCTCAGTGTAAAGTCTGCCG
chr12 chr12 57824553 57898031 52m57898008F24M                                                                                                                                                                                                                                                                         CCCGGGCGGCGGGCGGAGACGTGGGCGCCCTCCCCGCCAAGGCCCGTGCGAG AAGCCTCAGTGTAAAGTCTGCCGA
chr12 chr12 57824553 57898031 52m57898008F24M                                                                                                                                                                                                                                                                         CCCGGGCGGCGGGCGGAGACGTGGGCGCCCTCCCCGCCAAGGCCCGTGCGAG AAGCCTCAGTGTAAAGTCTGCCGA
chr12 chr12 57824553 57898031 52m57898008F24M                                                                                                                                                                                                                                                                         CCCGGGCGGCGGGCGGAGACGTGGGCGCCCTCCCCGCCAAGGCCCGTGCGAG AAGCCTCAGTGTAAAGTCTGCCGA
chr12 chr12 57824553 57898031 52m57898008F24M                                                                                                                                                                                                                                                                         CCCGGGCGGCGGGCGGAGACGTGGGCGCCCTCCCCGCCAAGGCCCGTGCGAG AAGCCTCAGTGTAAAGTCTGCCGA
chr12 chr12 57824553 57898031 52m57898008F24M                                                                                                                                                                                                                                                                         CCCGGGCGGCGGGCGGAGACGTGGGCGCCCTCCCCGCCGAGGCCCGTGCGAG AAGCCTCCGTGTAAAGTCTGCCGA
chr12 chr12 57824553 57898031 52m57898008F24M                                                                                                                                                                                                                                                                         CCCGGGCGGCGGGCGGAGACGTGGGCGCCCTCCCCGCCAAGGCCCGTGCGAG AAGCCTCAGTGTAAAGTCTGCCGA
chr12 chr12 57824553 57898031 52m57898008F24M                                                                                                                                                                                                                                                                         CCCGGGCGGCGGGCGGAGACGTGGGCGCCCTCCCCGCCAAGGCCCGTGCGAG AAGCCTCAGTGTAAAGTCTGCCGA
chr12 chr12 57824552 57898032 51m57898008F25M                                                                                                                                                                                                                                                                          CCGGGCGGCGGGCGGAGACGTGGGCGCCCTCCCCGCCAAGGCCCGTGCGAG AAGCCTCAGTGTAAAGTCTGCCGAT
chr12 chr12 57824552 57898032 51m57898008F25M                                                                                                                                                                                                                                                                          CCGGGCGGCGGGCGGAGACGTGGGCGCCCTCCCCGCCAAGGCCCGTGCGAG AAGCCTCAGTGTAAAGTCTGCCGAT
chr12 chr12 57824552 57898032 51m57898008F25M                                                                                                                                                                                                                                                                          CCGGGCGGCGGGCGGAGACGTGGGCGCCCTCCCCGCCAAGGCCCGTGCGAG AAGCCTCAGTGTAAAGTCTGCCGAT
chr12 chr12 57824552 57898032 51m57898008F25M                                                                                                                                                                                                                                                                          CCGGGCGGCGGGCGGAGACGTGGGCGCCCTCCCCGCCAAGGCCCGTGCGAG AAGCCTCAGTGTAAAGTCTGCCGAT
chr12 chr12 57824552 57898032 51m57898008F25M                                                                                                                                                                                                                                                                          CCGGGCGGCGGGCGGAGACGTGGGCGCCCTCCCCGCCAAGGCCCGTGCGAG AAGCCTCAGTGTAAAGTCTGCCGAT
chr12 chr12 57824552 57898032 51m57898008F25M                                                                                                                                                                                                                                                                          CCGGGCGGCGGGCGGAGACGTGGGCGCCCTCCCCGCCAAGGCCCGTGCGAG AAGCCTCAGTGTAAAGTCTGCCGAT
chr12 chr12 57824552 57898032 51m57898008F25M                                                                                                                                                                                                                                                                          CCGGGCGGCGGGCGGAGACGTGGGCGCCCTCCCCGCCAAGGCCCGGGCGAG AAGCCTCAGTGTAAAGTATGCCGAT
chr12 chr12 57824552 57898032 51m57898008F25M                                                                                                                                                                                                                                                                          CCGGGCGGCGGGCGGAGCCGTGGGCGCCCTCCCCGCCAAGGCCCGTGCGAG AAGCCTCAGTGTAAAGTATGCCGAT
chr12 chr12 57824552 57898032 51m57898008F25M                                                                                                                                                                                                                                                                          CCGGGCGGCGGGCGGAGACGTGGGCGCCCTCCCCGCCAAGGCCCGTGCGAG AAGCCTCAGTGTAAAGTCTGCCGAT
chr12 chr12 57824552 57898032 51m57898008F25M                                                                                                                                                                                                                                                                          CCGGGCGGCGGGCGGAGACGTGGGCGCCCTCCCCGCCAAGGCCCGTGCGAG AAGCCTCAGTGTAAAGTCTGCCGAT
chr12 chr12 57824552 57898032 51m57898008F25M                                                                                                                                                                                                                                                                          CCGGGCGGCGGGCGGAGACGTGGGCGCCCTCCCCGCCAAGGCCCGTGCGAG AAGCCTCAGTGTAAAGTCTGCCGAT
chr12 chr12 57824551 57898033 50m57898008F26M                                                                                                                                                                                                                                                                           CGGGCGGCGGGCGGAGACGTGGGCGCCCTCCCCGCCAAGGCCCGTGCGAG AAGCCTCAGTGTAAAGTCTGCCGATC
chr12 chr12 57824551 57898033 50m57898008F26M                                                                                                                                                                                                                                                                           CGGGCGGCGGGCGGAGACGTGGGCGCCCTCCCCGCCAAGGCCCGTGCGAG AAGCCTCAGTGTAAAGTCTGCCGATC
chr12 chr12 57824551 57898033 50m57898008F26M                                                                                                                                                                                                                                                                           CGGGCGGCGGGCGGAGACGTGGGCGCCCTCCCCGCCAAGGCCCGTGCGAG AAGCCTCAGTGTAAAGTCTGCCGATC
chr12 chr12 57824551 57898033 50m57898008F26M                                                                                                                                                                                                                                                                           CGGGCGGCGGGCGGAGACGTGGGCGCCCTCCCCGCCAAGGCCCGTGCGAG AAGCCTCAGTGTAAAGTCTGCCGATC
chr12 chr12 57824551 57898033 50m57898008F26M                                                                                                                                                                                                                                                                           CGGGCGGCGGGCGGAGACGTGGGCGCCCTCCCCGCCAAGGCCCGTGCGAG AAGCCTCAGTGTAAAGTCTGCCGATC
chr12 chr12 57824551 57898033 50m57898008F26M                                                                                                                                                                                                                                                                           CGGGCGGCGGGCGGAGACGTGGGCGCCCTCCCCGCCAAGGCCCGTGCGAG AAGCCTCAGTGTAAAGTCTGCCGATC
chr12 chr12 57824551 57898033 50m57898008F26M                                                                                                                                                                                                                                                                           CGGGCGGCGGGCGGAGACGTGGGCGCCCTCCCCGCCAAGGCCCGTGCGAG AAGCCTCAGTGTAAAGTCTGCCGATC
chr12 chr12 57824551 57898033 50m57898008F26M                                                                                                                                                                                                                                                                           AGGGCGGCGGGCGGAGACGTGGGCGCCCTCCCCGCCAAGGCCCGTGCGAG AAGCCTCAGTGTAAAGTCTGCCGATC
chr12 chr12 57824551 57898033 50m57898008F26M                                                                                                                                                                                                                                                                           CGGGCGGCGGGCGGAGACGTGGGCGCCCTCCCCGCCAAGGCCCGTGCGAG AAGCCTCAGTGTAAAGTCTGCCGATC
chr12 chr12 57824551 57898033 50m57898008F26M                                                                                                                                                                                                                                                                           CGGGCGGCGGGCGGAGACGTGGGCGCCCTCCCCGCCAAGGCCCGTGCGAG AAGCCTCAGTGTAAAGTCTGCCGATC
chr12 chr12 57824550 57898034 49m57898008F27M                                                                                                                                                                                                                                                                            GGGCGGCGGGCGGAGACGTGGGCGCCCTCCCCGCCAAGGCCCGTGCGAG AAGCCTCAGTGTAAAGTCTGCCGATCA
chr12 chr12 57824550 57898034 49m57898008F27M                                                                                                                                                                                                                                                                            GGGCGGCGGGCGGAGACGTGGGCGCCCTCCCCGCCAAGGCCCGTGCGAG AAGCCTCAGTGTAAAGTCTGCCGATCA
chr12 chr12 57824550 57898034 49m57898008F27M                                                                                                                                                                                                                                                                            GGGCGGCGGGCGGAGACGTGGGCGCCCTCCCCGCCAAGGCCCGTGCGAG AAGCCTCAGTGTAAAGTCTGCCGATCA
chr12 chr12 57824550 57898034 49m57898008F27M                                                                                                                                                                                                                                                                            GGGCGGCGGGCGGAGACGTGGGCGCCCTCCCCGCCAAGGCCCGTGCGAG AAGCCTCAGTGTAAAGTCTGCCGATCA
chr12 chr12 57824550 57898034 49m57898008F27M                                                                                                                                                                                                                                                                            GGGCGGCGGGCGGAGACGTGGGCGCCCTCCCCGCCAAGGCCCGTGCGAG AAGCCTCAGTGTAAAGTCTGCCGATCA
chr12 chr12 57824550 57898034 49m57898008F27M                                                                                                                                                                                                                                                                            GGGCGGCGGGCGGAGACGTGGGCGCCCTCCCCGCCAAGGCCCGTGCGAG AAGCCTCAGTGTAAAGTCTGCCGATCA
chr12 chr12 57824550 57898034 49m57898008F27M                                                                                                                                                                                                                                                                            GGGCGGCGGGCGGAGACGTGGGCGCCCTCCCCGCCAAGGCCCGTGCGAG AAGCCTCAGTGTAAAGTCTGCCGATCA
chr12 chr12 57824550 57898034 49m57898008F27M                                                                                                                                                                                                                                                                            GGGCGGCGGGCGGAGACGTGGGCGCCCTCCCCGCCAAGGCCCGTGCGAG AAGCCTCAGTGTAAAGTCTGCCGATCA
chr12 chr12 57824550 57898034 49m57898008F27M                                                                                                                                                                                                                                                                            GGGCGGCGGGCGGAGACGTGGGCGCCCTCCCCGCCAAGGCCCGTGCGAG AAGCCTCAGTGTAAAGTCTGCCGATCA
chr12 chr12 57824549 57898035 48m57898008F28M                                                                                                                                                                                                                                                                             GGCGGCGGGCGGAGACGTGGGCGCCCTCCCCGCCAAGGCCCGTGCGAG AAGCCTCAGTGTAAAGTCTGCCGATCAT
chr12 chr12 57824549 57898035 48m57898008F28M                                                                                                                                                                                                                                                                             GGCGGCGGGCGGAGACGTGGGCGCCCTCCCCGCCAAGGCCCGTGCGAG AAGCCTCAGTGTAAAGTCTGCCGATCAT
chr12 chr12 57824549 57898035 48m57898008F28M                                                                                                                                                                                                                                                                             GGCGGCGGGCGGAGACGTGGGCGCCCTCCCCGCCAAGGCCCGTGCGAG AAGCCTCAGTGTAAAGTCTGCCGATCAT
chr12 chr12 57824547 57898037 46m57898008F30M                                                                                                                                                                                                                                                                               CGGCGGGCGGAGACGTGGGCGCCCTCCCCCCCAAGGCCCGTGCGAG AAGCCTCCGTGTAAAGTCTGCCGATCATGC
chr12 chr12 57824546 57898038 45m57898008F31M                                                                                                                                                                                                                                                                                GGCGGGCGGAGACGTGGGCGCCCTCCCCGCCAAGGCCCGTGCGAG AAGCCTCAGTGTAAAGTCTGCCGATCATGCC
chr12 chr12 57824546 57898038 45m57898008F31M                                                                                                                                                                                                                                                                                GGCGGGCGGAGACGTGGGCGCCCTCCCCGCCAAGGCCCGTGCGAG AAGCCTCAGTGTAAAGTCTGCCGATCATGCC
chr12 chr12 57824546 57898038 45m57898008F31M                                                                                                                                                                                                                                                                                GGCGGGCGGAGACGTGGGCGCCCTCCCCGCCAAGGCCCGTGCGAG AAGCCTCAGTGTAAAGTCTGCCGATCATGCC
chr12 chr12 57824545 57898039 44m57898008F32M                                                                                                                                                                                                                                                                                 GCGGGCGGAGACGTGGGCGCCCTCCCCGCCAAGGCCCGTGCGAG AAGCCTCAGTGTAAAGTCTGCCGATCATGCCC
chr12 chr12 57824545 57898039 44m57898008F32M                                                                                                                                                                                                                                                                                 GCGGGCGGAGACGTGGGCGCCCTCCCCGCCAAGGCCCGTGCGAG AAGCCTCAGTGTAAAGTCTGCCGATCATGCCC
chr12 chr12 57824545 57898039 44m57898008F32M                                                                                                                                                                                                                                                                                 GCGGGCGGAGACGTGGGCGCCCTCCCCGCCAAGGCCCGTGCGAG AAGCCTCAGTGTAAAGTCTGCCGATCATGCCC
chr12 chr12 57824545 57898039 44m57898008F32M                                                                                                                                                                                                                                                                                 GCGGGCGGAGACGTGGGCGCCCTCCCCGCCAAGGCCCGTGCGAG AAGCCTCAGTGTAAAGTCTGCCGATCATGCCC
chr12 chr12 57824544 57898040 43m57898008F33M                                                                                                                                                                                                                                                                                  CGGGCGGAGACGTGGGCGCCCTCCCCGCCAAGGCCCGTGCGAG AAGCCTCAGTGTAAAGTCTGCCGATCATGCCCT
chr12 chr12 57824544 57898040 43m57898008F33M                                                                                                                                                                                                                                                                                  CGGGCGGAGACGTGGGCGCCCTCCCCGCCAAGGCCCGTGCGAG AAGCCTCAGTGTAAAGTCTGCCGATCATGCCCT
chr12 chr12 57824544 57898040 43m57898008F33M                                                                                                                                                                                                                                                                                  CGGGCGGAGACGTGGGCGCCCTCCCCGCCAAGGCCCGTGCGAG AAGCCTCAGTGTAAAGTCTGCCGATCATGCCCT
chr12 chr12 57824544 57898040 43m57898008F33M                                                                                                                                                                                                                                                                                  CGGGCGGAGACGTGGGCGCCCTCCCCGCCAAGGCCCGTGCGAG AAGCCTCAGTGTAAAGTCTGCCGATCATGCCCT
chr12 chr12 57824544 57898040 43m57898008F33M                                                                                                                                                                                                                                                                                  CGGGCGGAGACGTGGGCGCCCTCCCCGCCAAGGCCCGTGCGAG AAGCCTCAGTGTAAAGTCTGCCGATCATGCCCT
chr12 chr12 57824544 57898040 43m57898008F33M                                                                                                                                                                                                                                                                                  CGGGCGGAGACGTGGGCGCCCTCCCCGCCAAGGCCCGTGCGAG AAGCCTCAGTGCAAAGTCTGACGATCATGCCCT
chr12 chr12 57824544 57898040 43m57898008F33M                                                                                                                                                                                                                                                                                  CGGGCGGAGACGTGGGCGCCCTCCCCGCCAAGGCCCGTGCGAG AAGCCTCAGTGTAAAGTCTGCCGATCATGCCCT
chr12 chr12 57824544 57898040 43m57898008F33M                                                                                                                                                                                                                                                                                  CGGGCGGAGACGTGGGCGCCCTCCCCGCCAAGGCCCGTGCGAG AAGCCTCAGTGTAAAGTCTGCCGATCATGCCCT
chr12 chr12 57824544 57898040 43m57898008F33M                                                                                                                                                                                                                                                                                  CGGGCGGAGACGTGGGCGCCCTCCCCGCCAAGGCCCGTGCGAG AAGCCTCAGTGTAAAGTCTGCCGATCATGCCCT
chr12 chr12 57824544 57898040 43m57898008F33M                                                                                                                                                                                                                                                                                  CGGGCGGAGACGTGGGCGCCCTCCCCGCCAAGGCCCGTGCGAG AAGCCTCAGTGTAAAGTCTGCCGATCATGCCCT
chr12 chr12 57824544 57898040 43m57898008F33M                                                                                                                                                                                                                                                                                  CGGGCGGAGACGTGGGCGCCCTCCCCGCCAAGGCCCGTGCGAG AAGCCTCAGTGTAAAGTCTGCCGATCATGCCCT
chr12 chr12 57824544 57898040 43m57898008F33M                                                                                                                                                                                                                                                                                  CGGGCGGAGAAGTGGGCGCCCTCCCCGCCAAGGCCCGTGCGAG AAGCCTCAGTGTAAAGTCTGCCGATCATGCCCT
chr12 chr12 57824544 57898040 43m57898008F33M                                                                                                                                                                                                                                                                                  CGGGCGGAGACGTGGGCGCCCTCCCCGCCAAGGCCCGTGCGAG AAGCCTCAGTGTAAAGTCTGCCGATCATGCCCT
chr12 chr12 57824544 57898040 43m57898008F33M                                                                                                                                                                                                                                                                                  CGGGCGGAGACGTGGGCGCCCTCCCCGCCAAGGCCCGTGCGAG AAGCCTCAGTGTAAAGTCTGCCGATCATGCCCT
chr12 chr12 57824544 57898040 43m57898008F33M                                                                                                                                                                                                                                                                                  CGGGCGGAGACGTGGGCGCCCTCCCCGCCAAGGCCCGTGCGAG AAGCCTCAGTGTAAAGTCTGCCGATCATGCCCT
chr12 chr12 57824544 57898040 43m57898008F33M                                                                                                                                                                                                                                                                                  CGGGCGGAGACGTGGGCGCCCTCCCCGCCAAGGCCCGTGCGAG AAGCCTCAGTGTAAAGTCTGCCGATCATGCCCT
chr12 chr12 57824544 57898040 43m57898008F33M                                                                                                                                                                                                                                                                                  CGGGCGGAGACGTGGGCGCCCTCCCCGCCAAGGCCCGTGCGAG AAGCCTCAGTGTAAAGTCTGCCGATCATGCCCT
chr12 chr12 57824544 57898040 43m57898008F33M                                                                                                                                                                                                                                                                                  CGGGCGGAGACGTGGGCGCCCTCCCCGCCAAGGCCCGTGCGAG AAGCCTCAGTGTAAAGTCTGCCGATCATGCCCT
chr12 chr12 57824543 57898041 42m57898008F34M                                                                                                                                                                                                                                                                                   GGGCGGAGACGTGGGCGCCCTCCCCGCCAAGGCCCGTGCGAG AAGCCTCAGTGTAAAGTCTGCCGATCATGCCCTG
chr12 chr12 57824543 57898041 42m57898008F34M                                                                                                                                                                                                                                                                                   GGGCGGAGACGTGGGCGCCCTCCCCGCCAAGGCCCGTGCGAG AAGCCTCAGTGTAAAGTCTGCCGATCATGCCCTG
chr12 chr12 57824543 57898041 42m57898008F34M                                                                                                                                                                                                                                                                                   GGGCGGAGACGTGGGCGCCCTCCCCGCCAAGGCCCGTGCGAG AAGCCTCAGTGTAAAGTCTGCCGATCATGCCCTG
chr12 chr12 57824543 57898041 42m57898008F34M                                                                                                                                                                                                                                                                                   GGGCGGAGACGAGGGCGCCCTCCCCGCCAAGGCCCGTGCGAG AAGCCTCAGTGTAAAGTCTGCCGATCATGCCCTG
chr12 chr12 57824543 57898041 42m57898008F34M                                                                                                                                                                                                                                                                                   GGGCGGAGACGTGGGCGCCCTCCCCGCCAAGGCCCGTGCGAG AAGCCTCAGTGTAAAGTCTGCCGATCATGCCCTG
chr12 chr12 57824543 57898041 42m57898008F34M                                                                                                                                                                                                                                                                                   GGGCGGAGACGTGGGCGCCCTCCCCGCCAAGGCCCGTGCGAG AAGCCTCAGTGTAAAGTCTGCCGATCATGCCCTG
chr12 chr12 57824543 57898041 42m57898008F34M                                                                                                                                                                                                                                                                                   GGGCGGAGACGTGGGCGCCCTCCCCGCCAAGGCCCGTGCGAG AAGCCTCAGTGTAAAGTCTGCCGATCATGCCCTG
chr12 chr12 57824542 57898042 41m57898008F35M                                                                                                                                                                                                                                                                                    GGCGGAGACGTGGGCGCCCTCCCCGCCAAGGCCCGTGCGAG AAGCCTCAGTGTAAAGTCTGCCGATCATGCCCTGT
chr12 chr12 57824542 57898042 41m57898008F35M                                                                                                                                                                                                                                                                                    GGCGGAGACGTGGGCGCCCTCCCCGCCAAGGCCCGTGCGAG AAGCCTCAGTGTAAAGTCTGCCGATCATGCCCTGT
chr12 chr12 57824542 57898042 41m57898008F35M                                                                                                                                                                                                                                                                                    GGCGGAGACGTGGGCGCCCTCCCCGCCAAGGCCCGTGCGAG AAGCCTCAGAGTAAAGTCTGCCGATCATGCCCTGC
chr12 chr12 57824542 57898042 41m57898008F35M                                                                                                                                                                                                                                                                                    GGCGGAGACGTGGGCGCCCTCCCCGCCAAGGCCCGTGCGAG AAGCCTCAGTGTAAAGTCTGCCGATCATGCCCTGT
chr12 chr12 57824541 57898043 40m57898008F36M                                                                                                                                                                                                                                                                                     GCGGAGACGTGGGCGCCCTCCCCGCCAAGGCCCGTGCGAG AAGCCTCAGTGTAAAGTCTGCCGATCATGCCCTGTG
chr12 chr12 57824541 57898043 40m57898008F36M                                                                                                                                                                                                                                                                                     GCGGAGACGTGGGCGCCCTCCCCGCCAAGGCCCGTGCGAG AAGCCTCAGTGTAAAGTCTGCCGATCATGCCCTGTG
chr12 chr12 57824541 57898043 40m57898008F36M                                                                                                                                                                                                                                                                                     GCGGAGACGTGGGCGCCCTCCCCGCCAAGGCCCGTGCGAG AAGCCTCAGTGTAAAGTCTGCCGATCATGCCCTGTG
chr12 chr12 57824541 57898043 40m57898008F36M                                                                                                                                                                                                                                                                                     GCGGAGACGTGGGCGCCCTCCCCGCCAAGGCCCGTGCGAG AAGCCTCAGTGTAAAGTCTGCCGATCATGCCCTGTG
chr12 chr12 57824541 57898043 40m57898008F36M                                                                                                                                                                                                                                                                                     GCGGAGACGTGGGCGCCCTCCCCGCCAAGGCCCGTGCGAG AAGCCTCAGTGTAAAGTCTGCCGATCATGCCCTGTG
chr12 chr12 57824540 57898044 39m57898008F37M                                                                                                                                                                                                                                                                                      CGGAGACGTGGGCGCCCTCCCCGCCAAGGCCCGTGCGAG AAGCCTCAGTGTAAAGTCTGCCGATCATGCCCTGTGG
chr12 chr12 57824540 57898044 39m57898008F37M                                                                                                                                                                                                                                                                                      CGGAGACGTGGGCGCCCTCCCCGCCAAGGCCCGTGCGAG AAGCCTCAGTGTAAAGTCTGCCGATCATGCCCTGTGG
chr12 chr12 57824540 57898044 39m57898008F37M                                                                                                                                                                                                                                                                                      CGGAGACGTGGGCGCCCTCCCCGCCAAGGCCCGTGCGAG AAGCCTCAGTGTAAAGTCTGCCGATCATGCCCTGTGG
chr12 chr12 57824540 57898044 39m57898008F37M                                                                                                                                                                                                                                                                                      CGGAGACGTGGGCGCCCTCCCCGCCAAGGCCCGTGCGAG AAGCCTCAGTGTAAAGTCTGCCGATCATGCCCTGTGG
chr12 chr12 57824540 57898044 39m57898008F37M                                                                                                                                                                                                                                                                                      CGGAGACGTGGGCGCCCTCCCCGCCAAGGCCCGTGCGAG AAGCCTCAGTGTAAAGTCTGCCGATCATGCCCTGTGG
chr12 chr12 57824540 57898044 39m57898008F37M                                                                                                                                                                                                                                                                                      CGGAGACGTGGGCGCCCTCCCCGGCAAGGCCCGTGCGAG AAGCCTCAGTGTAAAGTCTGCCGATCATGCCCTGTGG
chr12 chr12 57824540 57898044 39m57898008F37M                                                                                                                                                                                                                                                                                      CGGAGACGTGGGCGCCCTCCCCGCCAAGGCCCGTGCGAG AAGCCTCAGTGTAAAGTCTGCCGATCATGCCCTGTGG
chr12 chr12 57824540 57898044 39m57898008F37M                                                                                                                                                                                                                                                                                      CGGAGACGTGGGCGCCCTCCCCGCCAAGGCCCGTGCGAG AAGCCTCAGTGTAAAGTCTGCCGATCATGCCCTGTGG
chr12 chr12 57824540 57898044 39m57898008F37M                                                                                                                                                                                                                                                                                      CGGAGACGTGGGCGCCCTCCCCGCCAAGGCCCGCGCGAG AAGCCTCAGTGTAAAGTCTGCCGATCATGCCCTGTGG
chr12 chr12 57824540 57898044 39m57898008F37M                                                                                                                                                                                                                                                                                      CGGAGACGTGGGCGCCCTCCCCGCCAAGGCCCGTGCGAG AAGCCTCAGTGTAAAGTCTGCCGATCATGCCCTGTGG
chr12 chr12 57824540 57898044 39m57898008F37M                                                                                                                                                                                                                                                                                      CGGAGACGTGGGCGCCCTCCCCGCCAAGGCCCGTGCGAG AAGCCTCAGTGTAAAGTCTGCCGATCATGCCCTGTGG
chr12 chr12 57824540 57898044 39m57898008F37M                                                                                                                                                                                                                                                                                      CGGAGACGTGGGCGCCCTCCCCGCCAAGGCCCGTGCGAG AAGCCTCAGTGTAAAGTCTGCCGATCATGCCCTGTGG
chr12 chr12 57824540 57898044 39m57898008F37M                                                                                                                                                                                                                                                                                      CGGAGACGTGGGCGCCCTCCCCGCCAAGGCCCGTGCGAG AAGCCTCAGTGTAAAGTCTGCCGATCATGCCCTGTGG
chr12 chr12 57824540 57898044 39m57898008F37M                                                                                                                                                                                                                                                                                      CGGAGACGTGGGCGCCCTCCCCGCCAAGGCCCGTGCGAG AAGCCTCAGTGTAAAGTCTGCCGATCATGCCCTGTGG
chr12 chr12 57824540 57898044 39m57898008F37M                                                                                                                                                                                                                                                                                      CGGAGACGTGGGCGCCCTCCCCGCCAAGGCCCGTGCGAG AAGCCTCAGTGTAAAGTCTGCCGATCATGCCCTGTGG
chr12 chr12 57824540 57898044 39m57898008F37M                                                                                                                                                                                                                                                                                      CGGAGACGTGGGCGCCCTCCCCGCCAAGGCCCGTGCGAG AAGCCTCAGTGTAAAGTCTGCCGATCATGCCCTGTGG
chr12 chr12 57824540 57898044 39m57898008F37M                                                                                                                                                                                                                                                                                      CGGAGACGTGGGCGCCCTCCCCGCCAAGGCCCGTGCGAG AAGCCTCAGTGTAAAGTCTGCCGATCATGCCCTGTGG
chr12 chr12 57824540 57898044 39m57898008F37M                                                                                                                                                                                                                                                                                      CGGAGACGTGGGCGCCCTCCCCGCCAAGGCCCGTGCGAG AAGCCTCAGGGTAAAGTCTGCCGATCATGCCCTGTGG
chr12 chr12 57824540 57898044 39m57898008F37M                                                                                                                                                                                                                                                                                      CGGAGACGTGGGCGCCCTCCCCGCCAAGGCCCGTGCGAG AAGCCTCGGTGTAAAGTCTGCCGATCATGCCCTGTGG
chr12 chr12 57824540 57898044 39m57898008F37M                                                                                                                                                                                                                                                                                      CGGAGACGTGGGCGCCCTCCCCGCCAAGGCCCGTGCGAG AAGCCTCAGTGTAAAGTCTGCCGATCATGCCCTGTGG
chr12 chr12 57824540 57898044 39m57898008F37M                                                                                                                                                                                                                                                                                      CGGAGACGTGGGCGCCCTCCCCGCCAAGGCCCGTGCGAG AAGCCTCAGTGTAAAGTCTGCCGATCATGCCCTGTGG
chr12 chr12 57824540 57898044 39m57898008F37M                                                                                                                                                                                                                                                                                      CGGAGACGTGGGCGCCCTCCCCGCCAAGGCCCGTGCGAG AAGCCTCAGTGTAAAGTCTGCCGATCATGCCCTGTGG
chr12 chr12 57824540 57898044 39m57898008F37M                                                                                                                                                                                                                                                                                      CGGAGACGTGGGCGCCCTCCCCGCCAAGGCCCGTGCGAG AAGCCTCAGTGTAAAGTCTGCCGATCATGCCCTGTGG
chr12 chr12 57824540 57898044 39m57898008F37M                                                                                                                                                                                                                                                                                      CGGAGACGTGGGCGCCCTCCCCGCCAAGGCCCGTGCGAG AAGCCTCAGTGTAAAGTCTGCCGATCATGCCCTGTGG
chr12 chr12 57824540 57898044 39m57898008F37M                                                                                                                                                                                                                                                                                      CGGAGACGTGGGCGCCCTCCCCGCCAAGGCCCGTGCGAG AAGCCTCAGTGTAAAGTCTGCCGATCATGCCCTGTGG
chr12 chr12 57824539 57898045 38m57898008F38M                                                                                                                                                                                                                                                                                       GGAGACGTGGGCGCCCTCCCCGCCAAGGCCCGTGCGAG AAGCCTCAGTGTAAAGTCTGCCGATCATGCCCTGTGGT
chr12 chr12 57824539 57898045 38m57898008F38M                                                                                                                                                                                                                                                                                       GGAGACGTGGGCGCCCTCCCCGCCAAGGCCCGTGCGAG AAGCCTCAGTGTAAAGTCTGCCGATCATGCCCTGTGGT
chr12 chr12 57824539 57898045 38m57898008F38M                                                                                                                                                                                                                                                                                       GGAGACGTGGGCGCCCTCCCCGCCAAGGCCCGTGCGAG AAGCCTCAGTGTAAAGTCTGCCGATCATGCCCTGTGGT
chr12 chr12 57824539 57898045 38m57898008F38M                                                                                                                                                                                                                                                                                       GGAGACGTGGGCGCCCTCCCCGCCAAGGCCCGTGCGAG AAGCCTCAGTGTAAAGTCTGCCGATCATGCCCTGTGGT
chr12 chr12 57824539 57898045 38m57898008F38M                                                                                                                                                                                                                                                                                       GGAGACGTGGGCGCCCTCCCCGCCAAGGCCCGTGCGAG AAGCCTCAGTGTAAAGTCTGCCGATCATGCCCTGTGGT
chr12 chr12 57824539 57898045 38m57898008F38M                                                                                                                                                                                                                                                                                       GGAGACGTGGGCGCCCTCCCCGCCAAGGCCCGTGCGAG AAGCCTCAGTGTAAAGTCTGCCGATCATGCCCTGTGGT
chr12 chr12 57824539 57898045 38m57898008F38M                                                                                                                                                                                                                                                                                       GGAGACGTGGGCGCCCTCCCCGCCAAGGCCCGTGCGAG AAGCCTCAGTGTAAAGTCTGCCGATCATGCCCTGTGGT
chr12 chr12 57824539 57898045 38m57898008F38M                                                                                                                                                                                                                                                                                       GGAGACGTGGGCGCCCTCCCCGCCAAGGCCCGTGCGAG AAGCCTCAGTGTAAAGTCTGCCGATCATGCCCTGTGGT
chr12 chr12 57824538 57898046 37m57898008F39M                                                                                                                                                                                                                                                                                        GAGACGTGGGCGCCCTCCCCGCCAAGGCCCGTGCGAG AAGCCTCAGTGTAAAGTCTGCCGATCATGCCCTGTGGTG
chr12 chr12 57824538 57898046 37m57898008F39M                                                                                                                                                                                                                                                                                        GAGACGTGGGCGCCCTCCCCGCCAAGGCCCGTGCGAG AAGCCTCAGTGTAAAGTCTGCCGATCATGCCCTGTGGTG
chr12 chr12 57824538 57898046 37m57898008F39M                                                                                                                                                                                                                                                                                        GAGACGTGGGCGCCCTCCCCGCCAAGGCCCGTGCGAG AAGCCTCAGTGTAAAGTCTGCCGATCATGCCCTGTGGTG
chr12 chr12 57824537 57898047 36m57898008F40M                                                                                                                                                                                                                                                                                         AGACGTGGGCGCCCTCCCCGCCAAGGCCCGTGCGAG AAGCCTCAGTGTAAAGTCTGCCGATCATGCCCTGTGGTGC
chr12 chr12 57824536 57898048 35m57898008F41M                                                                                                                                                                                                                                                                                          GACGTGGGCGCCCTCCCCGCCAAGGCCCGTGCGAG AAGCCTCAGTGTAAAGTCTGCCGATCATGCCCTGTGGTGCA
chr12 chr12 57824536 57898048 35m57898008F41M                                                                                                                                                                                                                                                                                          GACGTGGGCGCCCTCCCCGCCAAGGCCCGTGCGAG AAGCCTCAGTGTAAAGTCTGCCGATCATGCCCTGTGGTGCA
chr12 chr12 57824536 57898048 35m57898008F41M                                                                                                                                                                                                                                                                                          GACGTGGGCGCCCTCCCCGCCAAGGCCCGTGCGAG AAGCCTCAGTGTAAAGTCTGCCGATCATGCCCTGTGGTGCA
chr12 chr12 57824536 57898048 35m57898008F41M                                                                                                                                                                                                                                                                                          GACGTGGGCGCCCTCCCCGCCAAGGCCCGTGCGAG AAGCCTCAGTGTAAAGTCTGCCGATCATGCCCTGTGGTGCA
chr12 chr12 57824536 57898048 35m57898008F41M                                                                                                                                                                                                                                                                                          GACGTGGGCGCCCTCCCCGCCAAGGCCCGTGCGAG AAGCCTCAGTGTAAAGTCTGCCGATCATGCCCTGTGGTGCA
chr12 chr12 57824536 57898048 35m57898008F41M                                                                                                                                                                                                                                                                                          GACGTGGGCGCCCTCCCCGCCAAGGCCCGTGCGAG AAGCCTCAGTGTAAAGTCTGCCGATCATGCCCTGTGGTGCA
chr12 chr12 57824534 57898050 33m57898008F43M                                                                                                                                                                                                                                                                                            CGTGGGCGCCCTCCCCGCCAAGGCCCGTGCGAG AAGCCTCAGTGTAAAGTCTGCCGATCATGCCCTGTGGTGCAGT
chr12 chr12 57824534 57898050 33m57898008F43M                                                                                                                                                                                                                                                                                            CGTGGGCGCCCTCCCCGCCAAGGCCCGTGCGGG AAGCCTCAGTGTAAAGTCTGCCGATCATGCCCTGTGGTGCAGT
chr12 chr12 57824534 57898050 33m57898008F43M                                                                                                                                                                                                                                                                                            CGTGGGCGCCCTCCCCGCCAAGGCCCGTGCGAG AAGCCTCAGTGTAAAGTCTGCCGATCATGCCCTGTGGTGCAGT
chr12 chr12 57824534 57898050 33m57898008F43M                                                                                                                                                                                                                                                                                            CGTGGGCGCCCTCCCCGCCAAGGCCCGTGCGAG AAGCCTCAGTGTAAAGTCTGCCGATCATGCCCTGTGGTGCAGT
chr12 chr12 57824534 57898050 33m57898008F43M                                                                                                                                                                                                                                                                                            CGTGGGCGCCCTCCCCGCCAAGGCCCGTGCGAG AAGCCTCAGTGTAAAGTCTGCCGATCATGCCCTGTGGTGCAGT
chr12 chr12 57824534 57898050 33m57898008F43M                                                                                                                                                                                                                                                                                            CGTGGGCGCCCTCCCCGCCAAGGCCCGTGCGAG AAGCCTCAGTGTAAAGTCTGCCGATCATGCCCTGTGGTGCAGT
chr12 chr12 57824534 57898050 33m57898008F43M                                                                                                                                                                                                                                                                                            CGTGGGCGCCCTCCCCGCCAAGGCCCGTGCGAG AAGCCTCAGTGTAAAGTCTGCCGATCATGCCCTGTGGTGCAGT
chr12 chr12 57824534 57898050 33m57898008F43M                                                                                                                                                                                                                                                                                            CGTGGGCGCCCTCCCCGCCAAGGCCCGTGCGAG AAGCCTCAGTGTAAAGTCTGCCGATCATGCCCTGTGGTGCAGT
chr12 chr12 57824534 57898050 33m57898008F43M                                                                                                                                                                                                                                                                                            CGTGGGCGCCCTCCCCGCCAAGGCCCGTGCGAG AAGCCTCAGTGTAAAGTCTGCCGATCATGCCCTGTGGTGCAGT
chr12 chr12 57824534 57898050 33m57898008F43M                                                                                                                                                                                                                                                                                            CGTGGGCGCCCTCCCCGCCAAGGCCCGTGCGAG AAGCCTCAGTGTAAAGTCTGCCGATCATGCCCTGTGGTGCAGT
chr12 chr12 57824534 57898050 33m57898008F43M                                                                                                                                                                                                                                                                                            CGTGGGCGCCCTCCCCGCCAAGGCCCGTGCGAG AAGCCTCAGTGTAAAGTCTGCCGATCATGCCCTGTGGTGCAGT
chr12 chr12 57824533 57898051 32m57898008F44M                                                                                                                                                                                                                                                                                             GTGGGCGCCCTCCCCGCCAAGGCCCGTGCGAG AAGCCTCAGTGTAAAGTCTGCCGATCATGCCCTGTGGTGCAGTC
chr12 chr12 57824533 57898051 32m57898008F44M                                                                                                                                                                                                                                                                                             GTGGGCGCCCTCCCCGCCAAGGCCCGTGCGAG AAGCCTCAGTGTAAAGTCTGCCGATCATGCCCTGTGGTGCAGTC
chr12 chr12 57824533 57898051 32m57898008F44M                                                                                                                                                                                                                                                                                             GTGGGCGCCCTCCCCGCCAAGGCCCGTGCGAG AAGCCTCAGTGTAAAGTCTGCCGATCATGCCCTGTGGTGCAGTC
chr12 chr12 57824533 57898051 32m57898008F44M                                                                                                                                                                                                                                                                                             GTGGGCGCCCTCCCCGCCAAGGCCCGTGCGAG AAGCCTCAGTGTAAAGTCTGCCGATCATGCCCTGTGGTGCAGTC
chr12 chr12 57824533 57898051 32m57898008F44M                                                                                                                                                                                                                                                                                             GTGGGCGCCCTCCCCGCCAAGGCCCGTGCGAG AAGCCTCAGTGTAAAGTCTGCCGATCATGCCCTGTGGTGCAGTC
chr12 chr12 57824533 57898051 32m57898008F44M                                                                                                                                                                                                                                                                                             GTGGGCGCCCTCCCCGCCAAGGCCCGTGCGAG AAGCCTCAGTGTAAAGTCTGCCGATCATGCCCTGTGGTGCAGTC
chr12 chr12 57824533 57898051 32m57898008F44M                                                                                                                                                                                                                                                                                             GTGGGCGCCCTCCCCGCCAAGGCCCGTGCGAG AAGCCTCAGTGTAAAGTCTGCCGATCATGCCCTGTGGTGCAGTC
chr12 chr12 57824533 57898051 32m57898008F44M                                                                                                                                                                                                                                                                                             GTGGGCGCCCTCCCCGCCAAGGCCCGTGCGAG AAGCCTCAGTGTAAAGTCTGCCGATCATGCCCTGTGGTGCAGTC
chr12 chr12 57824532 57898052 31m57898008F45M                                                                                                                                                                                                                                                                                              TGGGCGCCCTCCCCGCCAAGGCCCGTGCGAG AAGCCTCAGTGTAAAGTCTGCCGATCATGCCCTGTGGTGCAGTCG
chr12 chr12 57824532 57898052 31m57898008F45M                                                                                                                                                                                                                                                                                              TGGGCGCCCTCCCCGCCAAGGCCCGTGCGAG AAGCCTCAGTGTAAAGTCTGCCGATCATGCCCTGTGGTGCAGTCG
chr12 chr12 57824532 57898052 31m57898008F45M                                                                                                                                                                                                                                                                                              TGGGCGCCCTCCCCGCCAAGGCCCGTGCGAG AAGCCTCAGTGTAAAGTCTGCCGATCATGCCCTGTGGTGCAGTCG
chr12 chr12 57824531 57898053 30m57898008F46M                                                                                                                                                                                                                                                                                               GGGCGCCCTCCCCGCCAAGGCCCGTGCGAG AAGCCTCAGTGTAAAGTCTGCCGATCATGCCCTGTGGTGCAGTCGA
chr12 chr12 57824531 57898053 30m57898008F46M                                                                                                                                                                                                                                                                                               GGGCGCCCTCCCCGCCAAGGCCCGTGCGAG AAGCCTCAGTGTAAATTCTGCCGATCATGCCCTGTGGTGCAGTCGA
chr12 chr12 57824531 57898053 30m57898008F46M                                                                                                                                                                                                                                                                                               GGGCGCCCTCCCCGCCAAGGCCCGTGCGAG AAGCCTCAGTGTAAAGTCTGCCGATCATGCCCTGTGGTGCAGTCGA
chr12 chr12 57824531 57898053 30m57898008F46M                                                                                                                                                                                                                                                                                               GGGCGCCCTCCCCGCCAAGGCCCGTGCGAG AAGCCTCAGTGTAAAGTCTGCCGATCATGCCCTGTGGTGCAGTCGA
chr12 chr12 57824531 57898053 30m57898008F46M                                                                                                                                                                                                                                                                                               GGGCGCCCTCCCCGCCAAGGCCCGTGCGAG AAGCCTCAGTGTAAAGTCTGCCGATCATGCCCTGTGGTGCAGTCGA
chr12 chr12 57824531 57898053 30m57898008F46M                                                                                                                                                                                                                                                                                               GGGCGCCCTCCCCGCCAAGGCCCGTGCGAG AAGCCTCAGTGTAAAGTCTACCGATCATGCCCTGTGGTGCAGTCGA
chr12 chr12 57824531 57898053 30m57898008F46M                                                                                                                                                                                                                                                                                               GGGCGCCCTCCCCGCCAAGGCCCGTGCGAG AAGCCTCAGTGTAAAGTCTGCCGATCATGCCCTGTGGTGCAGTCGA
chr12 chr12 57824531 57898053 30m57898008F46M                                                                                                                                                                                                                                                                                               GGGCGCCCTCCCCGCCAAGGCCCGTGCGAG AAGCCTCAGTGTAAAGTCTGCCGATCATGCCCTGTGGTGCAGTCGA
chr12 chr12 57824531 57898053 30m57898008F46M                                                                                                                                                                                                                                                                                               GGGCGCCCTCCCCGCCAAGGCCCGTGCGAG AAGCCTCAGTGTAAAGTCTGCCGATCATGCCCTGTGGTGCAGTCGA
chr12 chr12 57824531 57898053 30m57898008F46M                                                                                                                                                                                                                                                                                               GGGCGCCCTCCCCGCCAAGGCCCGTGCGAG AAGCCTCAGTGTAAAGTCTGCCGATCATGCCCTGTGGTGCAGTCGA
chr12 chr12 57824531 57898053 30m57898008F46M                                                                                                                                                                                                                                                                                               GGGCGCCCTCCCCGCCAAGGCCCGTGCGAG AAGCCTCAGTGTAAAGTCTGCCGATCATGCCCTGTGGTGCAGTCGA
chr12 chr12 57824531 57898053 30m57898008F46M                                                                                                                                                                                                                                                                                               ATGCGCCCTCCCCGCCAAGGCCCGTGCGAG AAGCCTCAGTGTAAAGTCTGCCGATCATGCCCTGTGGTGCAGTCGA
chr12 chr12 57824531 57898053 30m57898008F46M                                                                                                                                                                                                                                                                                               GGGCGCCCTCCCCGCCAAGGCCCGTGCGAG AAGCCTCAGTGTAAAGTCTGCCGATCATGCCCTGTGGTGCAGTCGA
chr12 chr12 57824531 57898053 30m57898008F46M                                                                                                                                                                                                                                                                                               GGGCGCCCTCCCCGCCAAGGCCCGTGCGAG AAGCCTCAGTGTAAAGTCTGCCGATCATGCCCTGTGGTGCAGTCGA
chr12 chr12 57824531 57898053 30m57898008F46M                                                                                                                                                                                                                                                                                               GGGCGCCCTCCCCGCCAAGGCCCGTGCGAG AAGCCTCAGTGTAAAGTCTGCCGATCATGCCCTGTGGTGCAGTCGA
chr12 chr12 57824531 57898053 30m57898008F46M                                                                                                                                                                                                                                                                                               GGGCGCCCTCCCCGCCAAGGCCCGTGCGAG AAGCCTCAGTGTAAATTCTGCCGATCATGCCCTGTGGTGCAGTCGA
chr12 chr12 57824531 57898053 30m57898008F46M                                                                                                                                                                                                                                                                                               GGGCGCCCTCCCCGCCAAGGCCCGTGCGAG AAGCCTCAGTGTAAAGTCTGCCGATCATGCCCTGTGGTGCAGTCGA
chr12 chr12 57824531 57898053 30m57898008F46M                                                                                                                                                                                                                                                                                               GGGCGCCCTCCCCGCCAAGGCCCGTGCGAG AAGCCTCAGTGTAAAGTCCGCCGATCATGCCCTGTGGTGCAGTCGA
chr12 chr12 57824531 57898053 30m57898008F46M                                                                                                                                                                                                                                                                                               GGGCGCCCTCCCCGCCAAGGCCCGTGCGAG AAGCCTCAGTGTAAAGTCTGCCGATCATGCCCTGTGGTGCAGTCGA
chr12 chr12 57824531 57898053 30m57898008F46M                                                                                                                                                                                                                                                                                               GGGCGCCCTCCCCGCCAAGGCCCGTGCGAG AAGCCTCAGTGTAAATTCTGCCGATCATGCACTGTGGTGCAGTCGA
chr12 chr12 57824531 57898053 30m57898008F46M                                                                                                                                                                                                                                                                                               GGGCGCCCTCCCCGCCAAGGCCCGTGCGAG AAGCCTCAGTGTAAAGTCTGCCGATCATGCCCTGTGGTGCAGGCGA
chr12 chr12 57824531 57898053 30m57898008F46M                                                                                                                                                                                                                                                                                               GGGCGCCCTCCCCGCCAAGGCCCGTGCGAG AAGCCTCAGTGTAAAGTCTGCCGATCATGCCCTGTGGTGCAGTCGA
chr12 chr12 57824531 57898053 30m57898008F46M                                                                                                                                                                                                                                                                                               GGGCGCCCTCCCCGCCAAGGCCCGTGCGAG AAGCCTCAGTGTAAAGTCTGCCGATCATGCCCTGTGGTGCAGTCGA
chr12 chr12 57824531 57898053 30m57898008F46M                                                                                                                                                                                                                                                                                               GGGCGCCCTCCCCGCCAAGGCCCATGCGAG AAGCCTCAGTGTAAAGTCTGCCGATCATGCCCTGTGGTGCAGTCGA
chr12 chr12 57824531 57898053 30m57898008F46M                                                                                                                                                                                                                                                                                               GGGCGCCCTCCCCGCCAAGGCCCGTGCGAG AAGCCTCAGTGTAAAGTCTGCCGATCATGCCCTGTGGTGCAGTCGA
chr12 chr12 57824531 57898053 30m57898008F46M                                                                                                                                                                                                                                                                                               GGGCGCCCTCCCCGCCAAGGCCCGTGCGAG AAGCCTCAGTGTAAAGTCTGCCGATCATGCCCTGTGGTGCAGTCGA
chr12 chr12 57824531 57898053 30m57898008F46M                                                                                                                                                                                                                                                                                               GGGCGCCCTCCCCGCCAAGGCCCGTGCGAG AAGCCTCAGTGTAAAGCCTGCCGATCATGCCCTGTGGTGCAGTCGA
chr12 chr12 57824530 57898054 29m57898008F47M                                                                                                                                                                                                                                                                                                GGCGCCCTCCCCGCCAAGGCCCGTGCGAG AAGCCTCAGTGTAAAGTATGCCGATCATGCCCTGTGGTGCAGTCGAG
chr12 chr12 57824530 57898054 29m57898008F47M                                                                                                                                                                                                                                                                                                GGCGCCCTCCCCGCCAAGGCCCGTGCGAG AAGCCTCAGTGTAAAGTCTGCCGATCATGCCCTGTGGTGCAGTCGAG
chr12 chr12 57824530 57898054 29m57898008F47M                                                                                                                                                                                                                                                                                                GGCGCCCTCCCCGCCAAGGCCCGTGCGAG AAGCCTCAGTGTAAAGTCTGCCGATCATGCCCTGTGGTGCAGTCGAG
chr12 chr12 57824530 57898054 29m57898008F47M                                                                                                                                                                                                                                                                                                GGCGCCCTCCCCGCCAAGGCCCGTGCGAG AAGCCTCAGTGTAAAGTCTGCCGATCATGCCCTGTGGTGCAGTCGAG
chr12 chr12 57824530 57898054 29m57898008F47M                                                                                                                                                                                                                                                                                                GGCGCCCTCCCCGCCAAGGCCCGTGCGAG AAGCCTCAGTGTAAAGTCTGCCGATCATGCCCTGTGGTGCAGTCGAG
chr12 chr12 57824530 57898054 29m57898008F47M                                                                                                                                                                                                                                                                                                GGCGCCCTCCCCGCCAAGGCCCGTGCGAG AAGCCTCAGTGTAAAGTCTGCCGATCATGCCCTGTGGTGCAGTCGAG
chr12 chr12 57824530 57898054 29m57898008F47M                                                                                                                                                                                                                                                                                                GGCGCCCTCCCCGCCAAGGCCCGTGCGAG AAGCCTCAGTGTAAAGTCTGCCGATCATGCCCTGTGGTGCAGTCGAG
chr12 chr12 57824530 57898054 29m57898008F47M                                                                                                                                                                                                                                                                                                GGCGCCCTCCCCGCCAAGGCCCGTGCGAG AAGCCTCAGTGTAAAGTCTGCCGATCATGCCCTGTGGTGCAGTCGAG
chr12 chr12 57824530 57898054 29m57898008F47M                                                                                                                                                                                                                                                                                                GGCGCCCTCCCCGCCAAGGCCCGTGCGAG AAGCCTCAGTGTAAAGTCTGCCGATCATGCCCTGTGGTGCAGTCGAG
chr12 chr12 57824530 57898054 29m57898008F47M                                                                                                                                                                                                                                                                                                GGCGCCCTCCCCGCCAAGGCCCGTGCGAG AAGCCTCAGTGTAAAGTCTGCCGATCATGCCCTGTGGTGCAGTCGAG
chr12 chr12 57824530 57898054 29m57898008F47M                                                                                                                                                                                                                                                                                                GGCGCCCTCCCCGCCAAGGCCCGTGCGAG AAGCCTCAGTGTAAAGTCTGCCGATCATGCCCTGTGGTGCAGTCGAG
chr12 chr12 57824530 57898054 29m57898008F47M                                                                                                                                                                                                                                                                                                GGCGCCCTCCCCGCCAAGGCCCGTGCGAG AAGCCTCAGTGTAAAGTCTGCCGATCATGCCCTGTGGTGCAGTCGAG
chr12 chr12 57824529 57898055 28m57898008F48M                                                                                                                                                                                                                                                                                                 GCGCCCTCCCCGCCAAGGCCCGTGCGAG AAGCCTCAGTGTAAAGTCTGCCGATCATGCCCTGTGGTGCAGTCGAGC
chr12 chr12 57824529 57898055 28m57898008F48M                                                                                                                                                                                                                                                                                                 GCGCCCTCCCCGCCAAGGCCCGTGCGAG AAGCCTCAGTGTAAAGTCTGCCGATCATGCCCTGTGGTGCAGTCGAGC
chr12 chr12 57824529 57898055 28m57898008F48M                                                                                                                                                                                                                                                                                                 GCGCCCTCCCCGCCAAGGCACGGGCGAG AAGCCTCAGTGTAAAGTCTGCCGATCATGCCCTGTGGTGCAGTCGAGC
chr12 chr12 57824529 57898055 28m57898008F48M                                                                                                                                                                                                                                                                                                 GCGCCCTCCCCGCCAAGGCCCGTGCGAG AAGCCTCAGTGTAAAGTCTGCCGATCATGCCCTGTGGTGCAGTCGAGC
chr12 chr12 57824529 57898055 28m57898008F48M                                                                                                                                                                                                                                                                                                 GCGCCCTCCCCGCCAAGGCCCGTGCGAG AAGCCTCAGTGTAAAGTCTGCCGATCATGCCCTGTGGTGCAGTCGACC
chr12 chr12 57824529 57898055 28m57898008F48M                                                                                                                                                                                                                                                                                                 GCGCCCTCCCCGCCAAGGCCCGTGCGAG AAGCCTCAGTGTAAAGTCTGCCGATCATGCCCTGTGGTGCAGTCGAGC
chr12 chr12 57824529 57898055 28m57898008F48M                                                                                                                                                                                                                                                                                                 GCGCCCTCCCCGCCAAGGCCCGTGCGAG AAGCCTCAGTGTAAAGTCTGCCGATCATGCCCTGTGGTGCAGTCGAGC
chr12 chr12 57824528 57898056 27m57898008F49M                                                                                                                                                                                                                                                                                                  CGCCCTCCCCGCCAAGGCCCGTGCGAG AAGCCTCAGTGTAAAGTCTGCCGATCATGCCCTGTGGTGCAGTCGAGCC
chr12 chr12 57824528 57898056 27m57898008F49M                                                                                                                                                                                                                                                                                                  CGCCCTCCCCGCCAAGGCCCGTGCGAG AAGCCTCAGTGTAAAGTCTGCCGATCATGCCCTGTGGTGCAGTCGAGCC
chr12 chr12 57824528 57898056 27m57898008F49M                                                                                                                                                                                                                                                                                                  AGCCCTCCCCGCCAAGGCCCGTGCGAG AAGCCTCAGTGTAAAGTCTGCCGATCATGCCCTGTGGTGCAGTCGAGCC
chr12 chr12 57824528 57898056 27m57898008F49M                                                                                                                                                                                                                                                                                                  CGCCCTCCCCGCCAAGGCCCGTGCGAG AAGCCTCAGTGTAAAGTCTGCCGATCATGCCCTGTGGTGCAGTCGAGCC
chr12 chr12 57824528 57898056 27m57898008F49M                                                                                                                                                                                                                                                                                                  CGCCCTCCCCGCCAAGGCCCGTGCGAG AAGCCTCAGTGTAAAGTCTGCCGATCATGCCCTGTGGTGCAGTCGAGCC
chr12 chr12 57824527 57898057 26m57898008F50M                                                                                                                                                                                                                                                                                                   GCCCTCCCCGCCAAGGCCCGTGCGAG AAGCCTCAGTGTAAAGTCTGCCGATCATGCCCTGTGGTGCAGTCGAGCCA
chr12 chr12 57824527 57898057 26m57898008F50M                                                                                                                                                                                                                                                                                                   GCCCTCCCCGCCAAGGCCCGTGCGAG AAGCCTCAGTGTAAAGTCTGCCGATCATGCCCTGTGGTGCAGTCGAGCCA
chr12 chr12 57824527 57898057 26m57898008F50M                                                                                                                                                                                                                                                                                                   GCCCTCCCCGCCAAGGCCCGTGCGAG AAGCCTCAGTGTAAAGTCTGCCGATCATGCCCTGTGGTGCAGTCGAGCCA
chr12 chr12 57824527 57898057 26m57898008F50M                                                                                                                                                                                                                                                                                                   GCCCTCCCCGCCAAGGCCCGTGCGAG AAGCCTCAGTGTAAAGTCTGCCGATCATGCCCTGTGGTGCAGTCGAGCCA
chr12 chr12 57824527 57898057 26m57898008F50M                                                                                                                                                                                                                                                                                                   GCCCTCCCCGCCAAGGCCCGTGCGAG AAGCCTCAGTGTAAAGTCTGCCGATCATGCCCTGTGGTGCAGTCGAGCCA
chr12 chr12 57824527 57898057 26m57898008F50M                                                                                                                                                                                                                                                                                                   GCCCTCCCCGCCAAGGCCCGTGCGAG AAGCCTCAGTGTAAAGTCTGCCGATCATGCCCTGTGGTGCAGTCGAGCCA
chr12 chr12 57824527 57898057 26m57898008F50M                                                                                                                                                                                                                                                                                                   GCCCTCCCCGCCAAGGCCCGTGCGAG AAGCCTCAGTGTAAAGTCTGCCGATCATGCCCTGTGGTGCAGTCGAGCCA
chr12 chr12 57824527 57898057 26m57898008F50M                                                                                                                                                                                                                                                                                                   GCCCTCCCCGCCAAGGCCCGTGCGAG AAGCCTCAGTGTAAAGTCTGCCGATCATTCCCTGTGGTGCAGTCGAGCCA
chr12 chr12 57824527 57898057 26m57898008F50M                                                                                                                                                                                                                                                                                                   GCCCTCCCCGCCAAGGCCCGTGCGAG AAGCCTCAGTGTAAAGTCTGCCGATCATCCCCTGTGGTGCAGTCGAGCCA
chr12 chr12 57824527 57898057 26m57898008F50M                                                                                                                                                                                                                                                                                                   GCCCTCCCCGCCAAGGCCCGTGCGAG AAGCCTCAGTGTAAAGTCTGCCGATCATGCCCTGTGGTGCAGTCGAGCCA
chr12 chr12 57824527 57898057 26m57898008F50M                                                                                                                                                                                                                                                                                                   GCCCTCCCCGCCAAGGCCCGTGCGAG AAGCCTCAGTGTAAAGTCTGCCGATCATGCCCTGTGGTGCAGTCGAGCCA
chr12 chr12 57824527 57898057 26m57898008F50M                                                                                                                                                                                                                                                                                                   GCCCTCCCCGCCAAGGCCCGTGCGAG AAGCCTCAGTGTAAAGTCTGCCGATCATGCCCTGTGGTGCAGTCGAGCCA
chr12 chr12 57824527 57898057 26m57898008F50M                                                                                                                                                                                                                                                                                                   GCCCTCCCCGCCAAGGCCCGTGCGAG AAGCCTCAGTGTAAAGTCTGCCGATCATGCCCTGTGGTGCAGTCGAGCCA
chr12 chr12 57824527 57898057 26m57898008F50M                                                                                                                                                                                                                                                                                                   GCCCTCCCCGCCAAGGCCCGTGCGAG AAGCCTCAGTGTAAAGTCTGCCGATCATGCCCTGTGGTGCAGTCGAGCCA
chr12 chr12 57824527 57898057 26m57898008F50M                                                                                                                                                                                                                                                                                                   GCCCTCCCCGCCAAGGCCCGTGCGAG AAGCCTCAGTGTAAAGTCTGCCGATCATGCCCTGTGGTGCAGTCGAGCCA
chr12 chr12 57824527 57898057 26m57898008F50M                                                                                                                                                                                                                                                                                                   GCCCTCCCCGCCAAGGCCCGTGCGAG AAGCCTCAGTGTAAAGTCTGCCGATCATGCCCTGTGGTGCAGTCGAGCCA
chr12 chr12 57824527 57898057 26m57898008F50M                                                                                                                                                                                                                                                                                                   GCCCTCCCCGCCAAGGCCCGTGCGAG AAGCCTCAGTGTAAAGTCTGCCGATCATGCCCTGTGGTGCAGTCGAGCCA
chr12 chr12 57824527 57898057 26m57898008F50M                                                                                                                                                                                                                                                                                                   GCCCTCCCCGCCAAGGCCCGTGCGAG AAGCCTCAGTGTAAAGTCTGCCGATCATGCCCTGTGGTGCAGTCGAGCCA
chr12 chr12 57824527 57898057 26m57898008F50M                                                                                                                                                                                                                                                                                                   GCCCTCCCCGCCAAGGCCCGTGCGAG AAGCCTCAGTGTAAAGTCTGCCGATCATGCCCTGTGGTGCAGTCGAGCCA
chr12 chr12 57824526 57898058 25m57898008F51M                                                                                                                                                                                                                                                                                                    CCCTCCCCGCCAAGGCCCGTGCGAG AAGCCTCAGTGTAAAGTCTGCCGATCATGCCCTGTGGTGCAGTCGAGCCAG
chr12 chr12 57824526 57898058 25m57898008F51M                                                                                                                                                                                                                                                                                                    CCTTCCCCGCCAAGGCCCGTGCGAG AAGCCTCAGTGTAAAGTCTGCCGATCATGCCCTGTGGTGCAGTCGAGCCAG
chr12 chr12 57824526 57898058 25m57898008F51M                                                                                                                                                                                                                                                                                                    CCCTCCCCGCCAAGGCCCGTGCGAG AAGCCTCAGTGTAAAGTCTGCCGATCATGCCCTGTGGTGCAGTCGAGCCAG
chr12 chr12 57824525 57898059 24m57898008F52M                                                                                                                                                                                                                                                                                                     CCTCCCCGCCAAGGCCCGTGCGAG AAGCCTCAGTGTAAAGTCTGCCGATCATGCCCTGTGGTGCAGTCGAGCCAGC
chr12 chr12 57824525 57898059 24m57898008F52M                                                                                                                                                                                                                                                                                                     CCTCCCCGCCAAGGCCCGTGCGAG AAGCCTCAGTGTAAAGTCTGCCGATCATGCCCTGTGGTGCAGTCGAGCCAGG
chr12 chr12 57824524 57898060 23m57898008F53M                                                                                                                                                                                                                                                                                                      CTCCCCGCCAAGGCCCGTGCGAG AAGCCTCAGTGTAAAGTCTGCCGATCATGCCCTGTGGTGCAGTCGAGCCAGCA
chr12 chr12 57824524 57898060 23m57898008F53M                                                                                                                                                                                                                                                                                                      CTCCCCGCCAAGGCCCGTGCGAG AAGCCTCAGTGTAAAGTCTGCCGATCATGCCCTGTGGTGCAGTCGAGCCAGCA
chr12 chr12 57824524 57898060 23m57898008F53M                                                                                                                                                                                                                                                                                                      TGCTGGCTCGACTGCACCACAGG GCATGATCGGCAGACTTTACACTGAGGCTTCTCGCACGGGCCTTGGCGGGGAG
chr12 chr12 57824524 57898060 23m57898008F53M                                                                                                                                                                                                                                                                                                      CTCCCCGCCAAGGCCCGTGCGAG AAGCCTCAGTGTAAAGTCTGCCGATCATGCCCTGTGGTGCAGTCGAGCCAGCA
chr12 chr12 57824524 57898060 23m57898008F53M                                                                                                                                                                                                                                                                                                      TGCTGGCTCGACTGCACCACAGG GCATGATCGGCAGACTTTACACTGAGGCTTCTCGCACGGGCCTTGGCGGGGAG
chr12 chr12 57824524 57898060 23m57898008F53M                                                                                                                                                                                                                                                                                                      CTCCCCGCCAAGGCCCGTGCGAG AAGCCTCAGTGTAAAGTCTGCCGATCATGCCCTGTGGTGCAGTCGAGCCAGCA
chr12 chr12 57824524 57898060 23m57898008F53M                                                                                                                                                                                                                                                                                                      TGCTGGCTCGACTGCACCACAGG GCATGATCGGCAGACTTTACACTGAGGCTTCTCGCACGGGCCTTGGCGGGGAG
chr12 chr12 57824524 57898060 23m57898008F53M                                                                                                                                                                                                                                                                                                      CTCCCCGCCAAGGCCCGTGCGAG AAGCCTCAGTGTAAAGTCTGCCGATCATGCCCTGTGGTGCAGTCGAGCCAGCA
chr12 chr12 57824524 57898060 23m57898008F53M                                                                                                                                                                                                                                                                                                      TGCTGGCTCGACTGCACCACAGG GCATGATCGGCAGACTTTACACTGAGGCTTCTCGCACGGGCCTTGGCGGGGAG
chr12 chr12 57824524 57898060 23m57898008F53M                                                                                                                                                                                                                                                                                                      TGCTGGCTCGACTGCACCACAGG GCATGATCGGCAGACTTTACACTGAGGCTTCTCGCACGGGCCTTGGCGGGGAG
chr12 chr12 57824524 57898060 23m57898008F53M                                                                                                                                                                                                                                                                                                      CTCCCCGCCAAGGCCCGTGCGAG AAGCCTCAGTGTAAAGTCTGCCGATCATGCCCTGTGGTGCAGTCGAGCCAGCA
chr12 chr12 57824524 57898060 23m57898008F53M                                                                                                                                                                                                                                                                                                      TGCTGGCTCGACTGCACCACAGG GCATGATCGGCAGACTTTACACTGAGGCTTCTCGCACGGGCCTTGGCGGGGAG
chr12 chr12 57824524 57898060 23m57898008F53M                                                                                                                                                                                                                                                                                                      CTCCCCGCCAAGGCCCGTGCGAG AAGCCTCAGTGTAAAGTCTGCCGATCATGCCCTGTGGTGCAGTCGAGCCAGCA
chr12 chr12 57824524 57898060 23m57898008F53M                                                                                                                                                                                                                                                                                                      TGCTGGCTCGACTGCACCACAGG GCATGATCGGCAGACTTTACACTGAGGCTTCTCGCACGGGCCTTGGCGGGGAG
chr12 chr12 57824524 57898060 23m57898008F53M                                                                                                                                                                                                                                                                                                      CTCCCCGCCAAGGCCCGTGCGAG AAGCCTCAGTGTAAAGTCTGCCGATCATGCCCTGTGGTGCAGTCGAGCCAGCA
chr12 chr12 57824524 57898060 23m57898008F53M                                                                                                                                                                                                                                                                                                      TGCTGGCTCGACTGCACCACAGG GCATGATCGGCAGACTTTACACTGAGGCTTCTCGCACGGGCCTTGGCGGGGAG
chr12 chr12 57824522 57898062 21m57898008F55M                                                                                                                                                                                                                                                                                                        GGTGCTGGCTCGACTGCACCA CAGGGCATGATCGGCAGACTTTACACTGAGGCTTCTCGCACGGGCCTTGGCGGGG
chr12 chr12 57824522 57898062 21m57898008F55M                                                                                                                                                                                                                                                                                                        GGTGCTGGCTCGACTGCACCA CAGGGCATGATCGGCAGACTTTACACTGAGGCTTCTCGCACGGGCCTTGGCGGGG
chr12 chr12 57824522 57898062 21m57898008F55M                                                                                                                                                                                                                                                                                                        GGTGCTGGCTCGACTGCACCA CAGGGCATGATCGGCAGACTTTACACTGAGGCTTCTCGCACGGGCCTTGGCGGGG
chr12 chr12 57824522 57898062 21m57898008F55M                                                                                                                                                                                                                                                                                                        GGTGCTGGCTCGACTGCACCA CAGGGCATGATCGGCAGACTTTACACTGAGGCTTCTCGCACGGGCCTTGGCGGGG
chr12 chr12 57824522 57898062 21m57898008F55M                                                                                                                                                                                                                                                                                                        GTTGCTGGCTCGACTGCACCA CAGGGCATGATCGGCAGACTTTACACTGAGGCTTCTCGCACGGGCCTTGGCGGGG
chr12 chr12 57824522 57898062 21m57898008F55M                                                                                                                                                                                                                                                                                                        GGTGCTGGCTCGACTGCACCA CAGGGCATGATCGGCAGACTTTACACTGAGGCTTCTCGCACGGGCCTTGGCGGGG
chr12 chr12 57824522 57898062 21m57898008F55M                                                                                                                                                                                                                                                                                                        GGTGCTGGCTCGACTGCACCA CAGGGCATGATCGGCAGACTTTACACTGAGGCTTCTCGCACGGGCCTTGGCGGGG
chr12 chr12 57824522 57898062 21m57898008F55M                                                                                                                                                                                                                                                                                                        GGTGCTGGCTCGACTGCACCA CAGGGCATGATCGGCAGACTTTACACTGAGGCTTCTCGCACGGGCCTTGGCGGGG
chr12 chr12 57824522 57898062 21m57898008F55M                                                                                                                                                                                                                                                                                                        GGTGCTGGCTCGACTGCACCA CAGGGCATGATCGGCAGACTTTACACTGAGGCTTCTCGCACGGGCCTTGGCGGGG
chr12 chr12 57824522 57898062 21m57898008F55M                                                                                                                                                                                                                                                                                                        GGTGCTGGCTCGACTGCACCA CAGGGCATGATCGGCAGACTTTACACTGAGGCTTCTCGCACGGGCCTTGGCGGGG
chr12 chr12 57824522 57898062 21m57898008F55M                                                                                                                                                                                                                                                                                                        GGTGCTGGCTCGACTGCACCA CAGGGCATGATCGGCAGACTTTACACTGAGGCTTCTCGCACGGGCCTTGGCGGGG
chr12 chr12 57824522 57898062 21m57898008F55M                                                                                                                                                                                                                                                                                                        GGTGCTGGCTCGACTGCACCA CAGGGCATGATCGGCAGACTTTACACTGAGGCTTCTCGCACGGGCCTTGGCGGGG
chr12 chr12 57824522 57898062 21m57898008F55M                                                                                                                                                                                                                                                                                                        GGTGCTGGCTCGACTGCACCA CAGGGCATGATCGGCAGACTTTACACTGAGGCTTCTCGCACGGGCCTTGGCGGGG
chr12 chr12 57824522 57898062 21m57898008F55M                                                                                                                                                                                                                                                                                                        GGTGCTGGCTCGACTGCACCA CAGGGCATGATCGGCAGACTTTACACTGAGGCTTCTCGCACGGGCCTTGGCGGGG
chr12 chr12 57824522 57898062 21m57898008F55M                                                                                                                                                                                                                                                                                                        GGTGCTGGCTCGACTGCACCA CAGGGCATGATCGGCAGACTTTACACTGAGGCTTCTCGCACGGGCCTTGGCGGGG
chr12 chr12 57824521 57898063 20m57898008F56M                                                                                                                                                                                                                                                                                                         AGGTGCTGGCTCGACTGCAC CACAGGGCATGATCGGCAGACTTTACACTGAGGCTTCTCGCACGGGCCTTGGCGGG
chr12 chr12 57824521 57898063 20m57898008F56M                                                                                                                                                                                                                                                                                                         AGGTGCTGGCTCGACTGCAC CACAGGGCATGATCGGCAGACTTTACACTGAGGCTTCTCGCACGGGCCTTGGCGGG
chr12 chr12 57824521 57898063 20m57898008F56M                                                                                                                                                                                                                                                                                                         AGGTGCTGGCTCGACTGCAC CACAGGGCATGATCGGCAGCCTTTACACTGAGGCTTCTCTCACGGGCCTTGGCGGG
chr12 chr12 57824521 57898063 20m57898008F56M                                                                                                                                                                                                                                                                                                         AGGTGCTGGCTCGACTGCAC CACAGGGCATGATCGGCAGACTTTACACTGAGGCTTCTCGCACGGGCCTTGGCGGG
chr12 chr12 57824521 57898063 20m57898008F56M                                                                                                                                                                                                                                                                                                         AGGTGCTGGCTCGACTGCAC CACAGGGCATGATCGGCAGACTTTACACTGAGGCTTCTCGCACGGGCCTTGGCGGG
chr12 chr12 57824520 57898064 19m57898008F57M                                                                                                                                                                                                                                                                                                          CAGGTGCTGGCTCGACTGC ACCACAGGGCATGATCGGCAGACTTTACACTGAGGCTTCTCGCACGGGCCTTGGCGG
chr12 chr12 57824520 57898064 19m57898008F57M                                                                                                                                                                                                                                                                                                          CAGGTGCTGGCTCGACTGC ACCACAGGGCATGATCGGCAGACTTTACACTGAGGCTTCTCGCACGGGCCTTGGCGG
chr12 chr12 57824520 57898064 19m57898008F57M                                                                                                                                                                                                                                                                                                          CAGGTGCTGGCTCGACTGC ACCACAGGGCATGATCGGCAGACTTTACACTGAGGCTTCTCGCACGGGCCTTGGCGG
chr12 chr12 57824520 57898064 19m57898008F57M                                                                                                                                                                                                                                                                                                          CAGGTGCTGGCTCGACTGC ACCACAGGGCATGATCGGCAGACTTTACACTGAGGCTTCTCGCACGGGCCTTGGCGG
chr12 chr12 57824520 57898064 19m57898008F57M                                                                                                                                                                                                                                                                                                          CAGGTGCTGGCTCGACTGC ACCACAGGGCATGATCGGCAGACTTTACACTGAGGCTTCTCGCACGGGCCTTGGCGG
chr12 chr12 57824520 57898064 19m57898008F57M                                                                                                                                                                                                                                                                                                          CAGGTGCTGGCTCGACTGC ACCACAGGGCATGATCGGCAGACTTTACACTGAGGCTTCTCGCACGGGCCTTGGCGG
chr12 chr12 57824520 57898064 19m57898008F57M                                                                                                                                                                                                                                                                                                          CAGGTGCTGGCTCGACTGC ACCACAGGGCATGATCGGCAGACTTTACACTGAGGCTTCTCGCACGGGCCTTGGCGG
chr12 chr12 57824520 57898064 19m57898008F57M                                                                                                                                                                                                                                                                                                          CAGGTGCTGGCTCGACTGC ACCACAGGGCATGATCGGCAGACTTTACACTGAGGCTTCTCGCACGGGCCTTGGCGG
chr12 chr12 57824519 57898065 18m57898008F58M                                                                                                                                                                                                                                                                                                           ACAGGTGCTGGCTCGACT GCACCACAGGGCATGATCGGCAGACTTTACACTGAGGCTTCTCGCACGGGCCTTGGCG
chr12 chr12 57824519 57898065 18m57898008F58M                                                                                                                                                                                                                                                                                                           ACAGGTGCTGGCTCGACT GCACCACAGGGCATGATCGGCAGACTTTACACTGAGGCTTCTCGCACGGGCCTTGGCG
chr12 chr12 57824519 57898065 18m57898008F58M                                                                                                                                                                                                                                                                                                           ACAGGTGCTGGCTCGACT GCACCACAGGGCATGATCGGCAGACTTTACACTGAGGCTTCTCGCACGGGCCTTGGCG
chr12 chr12 57824519 57898065 18m57898008F58M                                                                                                                                                                                                                                                                                                           ACAGGTGCTGGCTCGACT GCACCACAGGGCATGATCGGCAGACTTTACACTGAGGCTTCTCGCACGGGCCTTGGCG
chr12 chr12 57824519 57898065 18m57898008F58M                                                                                                                                                                                                                                                                                                           ACAGGTGCTGGCTCGACT GCACCACAGGGCATGATCGGCAGACTTTACACTGAGGCTTCTCGCACGGGCCTTGGCG
chr12 chr12 57824519 57898065 18m57898008F58M                                                                                                                                                                                                                                                                                                           ACAGGTGCTGGCTCGACT GCACCACAGGGCATTATCGGCAGACTTTACACTGAGGCTTCTCGCACGGGCCTTGGCG
chr12 chr12 57824519 57898065 18m57898008F58M                                                                                                                                                                                                                                                                                                           ACAGGTGCTGGCTCGACT GCACCACAGGGCATGATCGGCAGACTTTACACTGAGGCTTCTCGCACGGGCCTTGGCG
chr12 chr12 57824519 57898065 18m57898008F58M                                                                                                                                                                                                                                                                                                           ACAGGTGCTGGCTCGACT GCACCACAGGGCATGATCGGCAGACTTTACACTGAGGCTTCTCGCACGGGCCTTGGCG
chr12 chr12 57824519 57898065 18m57898008F58M                                                                                                                                                                                                                                                                                                           ACAGGTGCTGGCTCGACT GCACCACAGGGCATGATCGGCAGACTTTACACTGAGGCTTCTCGCACGGGCCTTGGCG
chr12 chr12 57824519 57898065 18m57898008F58M                                                                                                                                                                                                                                                                                                           ACAGGTGCTGGCTCGACT GCACCACAGGGCATGATCGGCAGACTTTACACTGAGGCTTCTCGCACGGGCCTTGGCG
chr12 chr12 57824519 57898065 18m57898008F58M                                                                                                                                                                                                                                                                                                           ACAGGTGCTGGCTCGACT GCACCACAGGGCATGATCGGCAGACTTTACACTGAGGCTTCTCGCACGGGCCTTGGCG
chr12 chr12 57824519 57898065 18m57898008F58M                                                                                                                                                                                                                                                                                                           ACAGGTGCTGGCTCGACT GCACCACAGCGCATGATCGGCAGACTTTACACTGAGGCTTCTCGCACGGGCCTTGCCG
chr12 chr12 57824519 57898065 18m57898008F58M                                                                                                                                                                                                                                                                                                           ACAGGTGCTGGCTCGACT GCACCACAGGGCATGATCGGCAGACTTTACACTGAGGCTTCTCGCACGGGCCTTGGCG
chr12 chr12 57824519 57898065 18m57898008F58M                                                                                                                                                                                                                                                                                                           ACAGGTGCTGGCTCGACT GCACCACAGGGCATGATCGGCAGACTTTACACTGAGGCTTCTCGCACGGGCCTTGGCG
chr12 chr12 57824519 57898065 18m57898008F58M                                                                                                                                                                                                                                                                                                           ACAGGTGCTGGCTCGACT GCACCACAGGGCATGATCGGCAGACTTTACACTGAGGCTTCTCGCACGGGCCTTGGCG
chr12 chr12 57824519 57898065 18m57898008F58M                                                                                                                                                                                                                                                                                                           ACAGGTGCTGGCTCGACT GCACCACAGGGCATGATCGGCAGACTTTACACTGAGGCTTCTCGCACGGGCCTTGGCG
chr12 chr12 57824519 57898065 18m57898008F58M                                                                                                                                                                                                                                                                                                           ACAGGTGCTGGCTCGACT GCACCACAGGGCATGATCGGCAGACTTTACACTGAGGCTTCTCGCACGGGCCTTGGCG
chr12 chr12 57824519 57898065 18m57898008F58M                                                                                                                                                                                                                                                                                                           ACAGGTGCTGGCTCGACT GCACCACAGGGCATGATCGGCAGACTTTACACTGAGGCTTCTCGCACGGGCCTTGGCG
chr12 chr12 57824519 57898065 18m57898008F58M                                                                                                                                                                                                                                                                                                           ACAGGTGCTGGCTCGACT GCACCACAGGGCATGATCGGCAGACTTTACACTGAGGCTTCTCGCGCGGGCCTTGGCG
chr12 chr12 57824519 57898065 18m57898008F58M                                                                                                                                                                                                                                                                                                           ACAGGTGCTGGCTCGACT GCACCACAGGGCATGATCGGCAGACTTTACACTGAGGCTTCTCGCACGGGCCTTGGCG
chr12 chr12 57824519 57898065 18m57898008F58M                                                                                                                                                                                                                                                                                                           ACAGGTGCTGGCTCGACT GCACCACAGGGCATGATCGGCAGACTTTACACTGAGGCTTCTCGCACGGGCCTTGGCG
chr12 chr12 57824519 57898065 18m57898008F58M                                                                                                                                                                                                                                                                                                           ACAGGTGCTGGCTCGACT GCACCACAGGGCATGATCGGCAGACTTTACACTGAGGCTTCTCGCACGGGCCTTGGCG
chr12 chr12 57824519 57898065 18m57898008F58M                                                                                                                                                                                                                                                                                                           ACAGGTGCTGGCTCGACT GCACCACAGGGCATGATCGGCAGACTTTACACTGAGGCTTCTCGCACGGGCCTTGGCG
chr12 chr12 57824519 57898065 18m57898008F58M                                                                                                                                                                                                                                                                                                           ACAGGTGCTGGCTCGACT GCACCACAGGGCATGATCGGCAGACTTTACACTGAGGCTTCTCGCACGGGCCTTGGCG
chr12 chr12 57824519 57898065 18m57898008F58M                                                                                                                                                                                                                                                                                                           ACAGGTGCTGGCTCGACT GCACCACAGGGCATGATCGGCAGACTTTACACTGAGGCTTCTCGCACGGGCCTTGGCG
chr12 chr12 57824519 57898065 18m57898008F58M                                                                                                                                                                                                                                                                                                           ACAGGTGCTGGCTCGACT GCACCACAGGGCATGATCGGCAGACTTTACACTGAGGCTTCTCGCACGGGCCTTGGCG
chr12 chr12 57824519 57898065 18m57898008F58M                                                                                                                                                                                                                                                                                                           ACAGGTGCTGGCTCGACT GCACCACAGGGCATGATCGGCAGACTTTACACTGAGGCTTCTCGCACGGGCCTTGGCG
chr12 chr12 57824519 57898065 18m57898008F58M                                                                                                                                                                                                                                                                                                           ACAGGTGCTGGCTCGACT GCACCACAGGGCATGATCGGCAGACTTTACACTGAGGCTTCTCGCACGGGCCTTGGCG
chr12 chr12 57824519 57898065 18m57898008F58M                                                                                                                                                                                                                                                                                                           ACAGGTGCTGGCTCGACT GCACCACAGGGCATGATCGGCAGTCTTTACACTGAGGCTTCTCGCACGGGCCTTGGCG
chr12 chr12 57824518 57898066 17m57898008F59M                                                                                                                                                                                                                                                                                                            AACAGGTGCTGGCTCGA CTGCACCACAGGGCATGATCGGCAGACTTTACACTGAGGCTTCTCGCACGGGCCTTGGC
chr12 chr12 57824518 57898066 17m57898008F59M                                                                                                                                                                                                                                                                                                            AACAGGTGCTGGCTCGA CTGCACCACAGGGCATGATCGGCAGACTTTACACTGAGGCTTCTCGCACGGGCCTTGGC
chr12 chr12 57824518 57898066 17m57898008F59M                                                                                                                                                                                                                                                                                                            AACAGGTGCTGGCTCGA CTGCACCACAGGGCATGATCGGCAGACTTTACACTGAGGCTTCTCGCACGGGCCTTGGC
chr12 chr12 57824518 57898066 17m57898008F59M                                                                                                                                                                                                                                                                                                            AACAGGTGCTGGCTCGA CTGCACCACAGGGCATGATCGGCAGACTTTACACTGAGGCTTCTCGCACGGGCCTTGGC
chr12 chr12 57824518 57898066 17m57898008F59M                                                                                                                                                                                                                                                                                                            AACAGGTGCTGGCTCGA CTGCACCACAGGGCATGATCGGCAGACTTTACACTGAGGCTTCTCGCACGGGCCTTGGC
chr12 chr12 57824518 57898066 17m57898008F59M                                                                                                                                                                                                                                                                                                            AACAGGTGCTGGCTCGA CTGCACCACAGGGCATGATCGGCAGACTTTACACTGAGGCTTCTCGCACGGGCCTTGGC
chr12 chr12 57824518 57898066 17m57898008F59M                                                                                                                                                                                                                                                                                                            AACAGGTGCTGGCACGA CTGCACCACAGGGCATGATCGGCAGACTTTACACTGAGGCTTCTCGCACGGGCCTTGGC
chr12 chr12 57824518 57898066 17m57898008F59M                                                                                                                                                                                                                                                                                                            AACAGGTGCTGGCTCGA CTGCACCACAGGGCATGATCGGCAGACTTTACACTGAGGCTTCTCGCACGGGCCTTGGC
chr12 chr12 57824518 57898066 17m57898008F59M                                                                                                                                                                                                                                                                                                            GCCAAGGCCCGTGCGAG AAGCCTCAGTGTAAAGTCTGCCGATCATGCCCTGTGGTGCAGTCGAGCCAGCACCTGTT
chr12 chr12 57824518 57898066 17m57898008F59M                                                                                                                                                                                                                                                                                                            GCCAAGGCCCGTGCGAG AAGCCTCAGTGTAAAGTCTGCCGATCATGCCCTGTGGTGCAGTCGAGCCAGCACCTGTT
chr12 chr12 57824518 57898066 17m57898008F59M                                                                                                                                                                                                                                                                                                            AACAGGTGCTGGCTCGA CTGCACCACAGGGCATGATCGGCAGACTTTACACTGAGGCTTCTCGCACGGGCCTTGGC
chr12 chr12 57824518 57898066 17m57898008F59M                                                                                                                                                                                                                                                                                                            AACAGGTGCTGGCTCGC CTGCACCACAGGGCATGATCGGCAGACTTTACACTGAGGCTTCTCGCACGGGCCTTGGC
chr12 chr12 57824518 57898066 17m57898008F59M                                                                                                                                                                                                                                                                                                            GCCAAGGCCCGGGCGAG AAGCCTCAGTGTAAAGTCTGCCGATCATGCCCTGTGGTGCAGTCGAGCCAGCACCTGTT
chr12 chr12 57824518 57898066 17m57898008F59M                                                                                                                                                                                                                                                                                                            AACAGGTGCTGGCTCGA CTGCACCACAGGGCATGATCGGCAGACTTTACACTTAGGCTTCTCGCACGGGCCTTGGC
chr12 chr12 57824518 57898066 17m57898008F59M                                                                                                                                                                                                                                                                                                            AACAGGTGCTGGCTCGA CTGCACCACAGGGCATGATCGGCAGACTTTACACTGAGGCTTCTCGCACGGGCCTTGGC
chr12 chr12 57824518 57898066 17m57898008F59M                                                                                                                                                                                                                                                                                                            AACAGGTGCTGGCTCGA CTGCACCACAGGGCATGATCGGCAGACTTTACACTGAGGCTTCTCGCACGGGCCTTGGC
chr12 chr12 57824518 57898066 17m57898008F59M                                                                                                                                                                                                                                                                                                            AACAGGTGCTGGCTCGA CTGCACCACAGGGCATGATCGGCAGACTTTACACTGAGGCTTCTCGCACGGGCCTTGGC
chr12 chr12 57824518 57898066 17m57898008F59M                                                                                                                                                                                                                                                                                                            AACAGGTGCTGGCTCGA CTGCACCACAGGGCATGATCGGCAGACTTTACACTGAGGCTTCTCGCACGGGCCTTGGC
chr12 chr12 57824518 57898066 17m57898008F59M                                                                                                                                                                                                                                                                                                            AACAGGTGCTGGCTCGA CTGCACCACAGGGCATGATCGGCAGACTTTACACTGAGGCTTCTCGCACGGGCCTTGGC
chr12 chr12 57824518 57898066 17m57898008F59M                                                                                                                                                                                                                                                                                                            AACAGGTGCTGGCTCGA CTGCACCACAGGGCATGATCGGCAGACTTTACACTGAGGCTTCTCGCACGGGCCTTGGC
chr12 chr12 57824518 57898066 17m57898008F59M                                                                                                                                                                                                                                                                                                            GCCAAGGCCCGTGCGAG AAGCCTCAGTGTAAAGTCTGCCGATCATGCCCTGTGGTGCAGTCGAGCCAGCACCTGTT
chr12 chr12 57824518 57898066 17m57898008F59M                                                                                                                                                                                                                                                                                                            AACAGGTGCTGGCTCGA CTGCACCACAGGGCATGATCGGCAGACTTTACACTGAGGCTTCTCGCACGGGCCTTGGC
chr12 chr12 57824517 57898067 16m57898008F60M                                                                                                                                                                                                                                                                                                             AAACAGGTGCTGGCTC GACTGCACCACAGGGCATGATCGGCAGCCTTTACACTGAGGCTTCTCGCACGGGCCTTGG
chr12 chr12 57824517 57898067 16m57898008F60M                                                                                                                                                                                                                                                                                                             AAACAGGTGCTGGCTC GACTGCACCACAGGGCATGATCGGCAGACTTTACACTGAGGCTTCTCGCACGGGCCTTGG
chr12 chr12 57824517 57898067 16m57898008F60M                                                                                                                                                                                                                                                                                                             AAACAGGTGCTGGCTC GACTGCACCACAGGGCATGATCGGCAGACTTTACACTGAGGCTTCTCGCACGGGCCTTGG
chr12 chr12 57824516 57898068 15m57898008F61M                                                                                                                                                                                                                                                                                                              CAAGGCCCGTGCGAG AAGCCTCAGTGTAAAGTCTGCCGATCATGCCCTGTGGTGCAGTCGAGCCAGCACCTGTTTC
chr12 chr12 57824516 57898068 15m57898008F61M                                                                                                                                                                                                                                                                                                              CAAGGCCCGTGCGAG AAGCCTCAGTGTAAAGTCTGCCGATCATGCCCTGTGGTGCAGTCGAGCCAGCACCTGTTTC
chr12 chr12 57824516 57898068 15m57898008F61M                                                                                                                                                                                                                                                                                                              GAAACAGGTGCTGGC TCGACTGCACCACAGGGCATGATCGGCAGACTTTACACTGAGGCTTCTCGCACGGGCCTTG
chr12 chr12 57824516 57898068 15m57898008F61M                                                                                                                                                                                                                                                                                                              GAAACAGGTGCTGGC TCGACTGCACCACAGGGCATGATCGGCAGACTTTACACTGAGGCTTCTCGCACGGGCCTTG
chr12 chr12 57824516 57898068 15m57898008F61M                                                                                                                                                                                                                                                                                                              GAAACAGGTGCTGGC TCGACTGCACCACAGGGCATGATCGGCAGACTTTACACTGAGGCTTCTCGCACGGGCCTTG
chr12 chr12 57824516 57898068 15m57898008F61M                                                                                                                                                                                                                                                                                                              GAAACAGGTGCTGGC TCGACTGCACCACAGGGCATGATCGGCAGACTTTACACTGAGGCTTCTCGCACGGGCCTTG
chr12 chr12 57824516 57898068 15m57898008F61M                                                                                                                                                                                                                                                                                                              GAAACAGGTGCTGGC TCGACTGCACCACAGGGCATGATCGGCAGACTTTACACTGAGGCTTCTCGCACGGGCCTTG
chr12 chr12 57824516 57898068 15m57898008F61M                                                                                                                                                                                                                                                                                                              GAAACAGGTGCTGGC TCGACTGCACCACAGGGCATGATCGGCAGACTTTACACTGAGGCTTCTCGCACGGGCCTTG
chr12 chr12 57824516 57898068 15m57898008F61M                                                                                                                                                                                                                                                                                                              GAAACAGGTGCTGGC TCGACTGCACCACAGGGCATGATCGGCAGACTTTACACTGAGGCTTCTCGCACGGGCCTTG
chr12 chr12 57824516 57898068 15m57898008F61M                                                                                                                                                                                                                                                                                                              GAAACAGGTGCTGGC TCGACTGCACCACAGGGCATGATCGGCAGACTTTACACTGAGGCTTCTCGCACGGGCCTTG
chr12 chr12 57824516 57898068 15m57898008F61M                                                                                                                                                                                                                                                                                                              CAAGGCCCGTGCGAG AAGCCTCAGTGTAAAGTCTGCCGATCATGCCCTGTGGTGCAGTCGAGCCAGCACCTGTTTC
chr12 chr12 57824516 57898068 15m57898008F61M                                                                                                                                                                                                                                                                                                              GAAACAGGTGCTGGC TCGACTGCACCACAGGGCATGATCGGCAGACTTTACACTGAGGCTTCTCGCACGGGCCTTG
chr12 chr12 57824515 57898069 14m57898008F62M                                                                                                                                                                                                                                                                                                               AGAAACAGGTGCTG GCTCGACTGCACCACAGGGCATGATCGGCAGACTTTACACTGAGGCTTCTCGCACGGGCCTT
chr12 chr12 57824515 57898069 14m57898008F62M                                                                                                                                                                                                                                                                                                               AGAAACAGGTGCTG GCTCGACTGCACCACAGGGCATGATCGGCAGACTTTACACTGAGGCTTCTCGCACGGGCCTT
chr12 chr12 57824515 57898069 14m57898008F62M                                                                                                                                                                                                                                                                                                               AGAAACAGGTGCTG GCTCGACTGCACCACAGGGCATGATCGGCAGACTTTACACTGAGGCTTCTCGCACGGGCCTT
chr12 chr12 57824515 57898069 14m57898008F62M                                                                                                                                                                                                                                                                                                               AAGGCCCGTGCGAG AAGCCTCAGTGTAAAGTCTGCCGATCATGCCCTGTGGTGCAGTCGAGCCAGCACCTGTTTCT
chr12 chr12 57824514 57898070 13m57898008F63M                                                                                                                                                                                                                                                                                                                AGGCCCGTGCGAG AAGCCTCAGTGTAAAGTCTGCCGATCATGCCCTGTGGTGCAGTCGAGCCAGCACCTGTTTCTG
chr12 chr12 57824514 57898070 13m57898008F63M                                                                                                                                                                                                                                                                                                                AGGCCCGTGCGAG AAGCCTCAGTGTAAAGTCTGCCGATCATGCCCTGTGGTGCAGTCGAGCCAGCACCTGTTTCTG
chr12 chr12 57824514 57898070 13m57898008F63M                                                                                                                                                                                                                                                                                                                AGGCCCGTGCGAG AAGCCTCAGTGTAAAGTCTGCCGATCATGCCCTGTGGTGCAGTCGAGCCAGCACCTGTTTCTG
chr12 chr12 57824514 57898070 13m57898008F63M                                                                                                                                                                                                                                                                                                                AGGCCCGTGCGAG AAGCCTCAGTGTAAAGTCTGCCGATCATGCCCTGTGGTGCAGTCGAGCCAGCACCTGTTTCTG
chr12 chr12 57824514 57898070 13m57898008F63M                                                                                                                                                                                                                                                                                                                AGGCCCGTGCGAG AAGCCTCAGTGTAAAGTCTGCCGATCATGCCCTGTGGTGCAGTCGAGCCAGCACCTGTTTCTG
chr12 chr12 57824514 57898070 13m57898008F63M                                                                                                                                                                                                                                                                                                                AGGCCCGTGCGAG AAGCCTCAGTGTAAAGTCTGCCGATCATGCCCTGTGGTGCAGTCGAGCCAGCACCTGTTTCTG
chr12 chr12 57824514 57898070 13m57898008F63M                                                                                                                                                                                                                                                                                                                AGGCCCGTGCGAG AAGCCTCAGTGTAAAGTCTGCCGATCATGCCCTGTGGTGCAGTCGAGCCAGCACCTGTTTCTG
chr12 chr12 57824514 57898070 13m57898008F63M                                                                                                                                                                                                                                                                                                                AGGCCCGTGCGAG AAGCCTCAGTGTAAAGTCTGCCGATCATGCCCTGTGGTGCAGTCGAGCCAGCACCTGTTTCTG
chr12 chr12 57824514 57898070 13m57898008F63M                                                                                                                                                                                                                                                                                                                AGGCCCGTGCGAG AAGCCTCAGTGTAAAGTCTGCCGATCATGCCCTGTGGTGCAGTCGAGCCAGCACCTGTTTCTG
chr12 chr12 57824514 57898070 13m57898008F63M                                                                                                                                                                                                                                                                                                                AGGCCCGTGCGAG AAGCCTCAGTGTAAAGTCTGCCGATCATGCCCTGTGGTGCAGTCGAGCCAGCACCTGTTTCTG
chr12 chr12 57824514 57898070 13m57898008F63M                                                                                                                                                                                                                                                                                                                AGGCCCGTGCGAG AAGCCTCAGTGTAAAGTCTGCCGATCATGCCCTGTGGTGCAGTCGAGCCAGCACCTGTTTCTG
chr12 chr12 57824514 57898070 13m57898008F63M                                                                                                                                                                                                                                                                                                                AGGCCCGTGCGAG AAGCCTCAGTGTAAAGTCTGCCGATCATGCCCTGTGGTGCAGTCGAGCCAGCACCTGTTTCTG
chr12 chr12 57824513 57898071 12m57898008F64M                                                                                                                                                                                                                                                                                                                 CCAGAAACAGGT GCTGGCTCGACTGCACCACAGGGCATGATCGGCAGACTTTACACTGAGGCTTCTCGCACGGGCC
chr12 chr12 57824513 57898071 12m57898008F64M                                                                                                                                                                                                                                                                                                                 CCAGAAACAGGT GCTGGCTCGACTGCACCACAGGGCATGATCGGCAGACTTTACACTGAGGCTTCTCGCACGGGCC
chr12 chr12 57824513 57898071 12m57898008F64M                                                                                                                                                                                                                                                                                                                 CCAGAAACAGGT GCTGGCTCGACTGCACCACAGGGCATGATCGGCAGACTTTACACTGAGGCTTCTCGCACGGGCC
chr12 chr12 57824513 57898071 12m57898008F64M                                                                                                                                                                                                                                                                                                                 CCAGAAACAGGT GCTGGCTCGACTGCACCACAGGGCATGATCGGCAGACTTTACACTGAGGCTTCTCGCACGGGCC
chr12 chr12 57824513 57898071 12m57898008F64M                                                                                                                                                                                                                                                                                                                 CCAGAAACAGGT GCTGGCTCGACTGCACCACAGGGCATGATCGGCAGACTTTACACTGAGGCTTCTCGCACGGGCC
chr12 chr12 57824513 57898071 12m57898008F64M                                                                                                                                                                                                                                                                                                                 GGCCCGTGCGAG AAGCCTCAGTGTAAAGTCTGCCGATCATGCCCTGTGGTGCAGTCGAGCCAGCACCTGTTTCTGG
chr12 chr12 57824512 57898072 11m57898008F65M                                                                                                                                                                                                                                                                                                                  TCCAGAAACAG GTGCTGGCTCGACTGCACCACAGGGCATGATCGGCAGACTTTACACTGAGGCTTCTCGCACGGGC
chr12 chr12 57824512 57898072 11m57898008F65M                                                                                                                                                                                                                                                                                                                  TCCAGAAACAG GTGCTGGCTCGACTGCACCACAGGGCATGATCGGCAGACTTTACACTGAGGCTTCTCGCACGGGC
chr12 chr12 57824512 57898072 11m57898008F65M                                                                                                                                                                                                                                                                                                                  TCCAGAAACAG GTGCTGGCTCGACTGCACCACAGGGCATGATCGGCAGACTTTACACTGAGGCTTCTCGCACGGGC
chr12 chr12 57824512 57898072 11m57898008F65M                                                                                                                                                                                                                                                                                                                  TCCAGAAACAG GTGCTGGCTCGACTGCACCACAGGGCATGATCGGCAGACTTTACACTGAGGCTTCTCGCACGGGC
chr12 chr12 57824511 57898073 10m57898008F66M                                                                                                                                                                                                                                                                                                                   GTCCAGAAAC AGGTGCTGGCTCGACTGCACCACAGGGCATGATCGGCAGACTTTACACTGAGGCTTCTCGCACGGG
chr12 chr12 57824511 57898073 10m57898008F66M                                                                                                                                                                                                                                                                                                                   GTCCAGAAAC AGGTGCTGGCTCGACTGCACCACAGGGCATGATCGGCAGACTTTACACTGAGGCTTCTCGCACGGG
chr12 chr12 57824511 57898073 10m57898008F66M                                                                                                                                                                                                                                                                                                                   CCCGTGCGAG AAGCCTCAGTGTAAAGTCTGCCGATCATGCCCTGTGGTGCAGTCGAGCCAGCACCTGTTTCTGGAC
chr12 chr12 57824510 57898074 9m57898008F67M                                                                                                                                                                                                                                                                                                                     GGTCCAGAA ACAGGTGCTGGCTCGACTGCACCACAGGGCATGATCGGCAGACTTTACACTGAGGCTTCTCGCACGG
chr12 chr12 57824510 57898074 9m57898008F67M                                                                                                                                                                                                                                                                                                                     GGTCCAGAA ACAGGTGCTGGCTCGACTGCACCACAGGGCATGATCGGCAGACTTTACACTGAGGCTTCTCGCACGG
chr12 chr12 57824509 57898075 8m57898008F68M                                                                                                                                                                                                                                                                                                                      AGGTCCAG AAACAGGTGCTGGCTCGACTGCACCACAGGGCATGATCGGCAGACTTTACACTGAGGCTTCTCGCACG
chr12 chr12 57824509 57898075 8m57898008F68M                                                                                                                                                                                                                                                                                                                      AGGTCCAG AAACAGGTGCTGGCTCGACTGCACCACAGGGCATGATCGGCAGACTTTACACTGAGGCTTCTCGCACG
chr12 chr12 57824509 57898075 8m57898008F68M                                                                                                                                                                                                                                                                                                                      AGGTCCAG AAACAGGTGCTGGCTCGACTGCACCACAGGGCATGATCGGCAGACTTTACACTGAGGCTTCTCGCACG
chr12 chr12 57824509 57898075 8m57898008F68M                                                                                                                                                                                                                                                                                                                      AGGTCCAG AAACAGGTGCTGGCTCGACTGCACCACAGGGCATGATCGGCAGACTTTACACTGAGGCTTCTCGCACG
chr12 chr12 57824508 57898076 7m57898008F69M                                                                                                                                                                                                                                                                                                                       CAGGTCC AGAAACAGGTGCTGGCTCGACTGCACCACAGGGCATGATCGGCAGACTTTACACTGAGGCTTCTCGCAC
chr12 chr12 57824508 57898076 7m57898008F69M                                                                                                                                                                                                                                                                                                                       CAGGTCC AGAAACAGGTGCTGGCTCGACTGCACCACAGGGCATGATCGGCAGACTTTACACTGAGGCTTCTCGCAC
chr12 chr12 57824508 57898076 7m57898008F69M                                                                                                                                                                                                                                                                                                                       CAGGTCC AGAAACAGGTGCTGGCTCGACTGCACCACAGGGCATGATCGGCAGACTTTACACTGAGGCTTCTCGCAC
chr12 chr12 57824508 57898076 7m57898008F69M                                                                                                                                                                                                                                                                                                                       CAGGTCC AGAAACAGGTGCTGGCTCGACTGCACCACAGGGCATGATCGGCAGACTTTACACTGAGGCTTCTCGCAC
chr12 chr12 57824508 57898076 7m57898008F69M                                                                                                                                                                                                                                                                                                                       CAGGTCC AGAAACAGGTGCTGGCTCGACTGCACCACAGGGCATGATCGGCAGACTTTACACTGAGGCTTCTCGCAC
chr12 chr12 57824508 57898076 7m57898008F69M                                                                                                                                                                                                                                                                                                                       CAGGTCC AGAAACAGGTGCTGGCTCGACTGCACCACAGGGCATGATCGGCAGACTTTACACTGAGGCTTCTCGCAC
chr12 chr12 57824508 57898076 7m57898008F69M                                                                                                                                                                                                                                                                                                                       CAGGTCC AGAAACAGGTGCTGGCTCGACTGCACCACAGGGCATGATCGGCAGACTTTACACTGAGGCTTCTCGCAC
chr12 chr12 57824508 57898076 7m57898008F69M                                                                                                                                                                                                                                                                                                                       CAGGTCC AGAAACAGGTGCTGGCTCGACTGCACCACAGGGCATGATCGGCAGACTTTACACTGAGGCTTCTCGCAC
chr12 chr12 57824508 57898076 7m57898008F69M                                                                                                                                                                                                                                                                                                                       CAGGTCC AGAAACAGGTGCTGGCTCGACTGCACCACAGGGCATGATCGGCAGACTTTACACTGAGGCTTCTCGCAC
chr12 chr12 57824508 57898076 7m57898008F69M                                                                                                                                                                                                                                                                                                                       CAGGTCC AGAAACAGGTGCTGGCTCGACTGCACCACAGGGCATGATCGGCAGACTTTACACTGAGGCTTCTCGCAC
chr12 chr12 57824508 57898076 7m57898008F69M                                                                                                                                                                                                                                                                                                                       CAGGTCC AGAAACAGGTGCTGGCTCGACTGCACCACAGGGCATGATCGGCAGACTTTACACTGAGGCTTCTCGCAC
chr12 chr12 57824508 57898076 7m57898008F69M                                                                                                                                                                                                                                                                                                                       GTGCGAG AAGCCTCAGTGTAAAGTCTGCCGATCATGCCCTGTGGTGCAGTCGAGCCAGCACCTGTTTCTGGACCTG
chr12 chr12 57824508 57898076 7m57898008F69M                                                                                                                                                                                                                                                                                                                       CAGGTCC AGAAACAGGTGCTGGCTCGACTGCACCACAGGGCATGATCGGCAGACTTTACACTGAGGCTTCTCGCAC
chr12 chr12 57824508 57898076 7m57898008F69M                                                                                                                                                                                                                                                                                                                       GTGCGAG AAGCCTCAGTGTAAAGTCTGCCGATCATGCCCTGTGGTGCAGTCGAGCCAGCACCTGTTTCTGGACCTG
chr12 chr12 57824508 57898076 7m57898008F69M                                                                                                                                                                                                                                                                                                                       GTGCGAG AAGCCTCAGTGTAAAGTCTGCCGATCATGCCCTGTGGTGCAGTCGAGCCAGCACCTGTTTCTGGACCTG
chr12 chr12 57824508 57898076 7m57898008F69M                                                                                                                                                                                                                                                                                                                       CAGGTCC AGAAACAGGTGCTGGCTCGACTGCACCACAGGGCATGATCGGCAGACTTTACACTGAGGCTTCTCGCAC
chr12 chr12 57824508 57898076 7m57898008F69M                                                                                                                                                                                                                                                                                                                       CAGGTCC AGAAACAGGTGCTGGCTCGACTGCACCACAGGGCATGATCGGCAGACTTTACACTGAGGCTTCTCGCAC
chr12 chr12 57824506 57898078 5m57898008F71M                                                                                                                                                                                                                                                                                                                         GGCAG GTCCAGAAACAGGTGCTGGCTCGACTGCACCACAGGGCATGATCGGCAGACTTTACACTGAGGCTTCTCGC
chr12 chr12 57824506 57898078 5m57898008F71M                                                                                                                                                                                                                                                                                                                         GGCAG GTCCAGAAACAGGTGCTGGCTCGACTGCACCACAGGGCATGATCGGCAGACTTTACACTGAGGCTTCTCGC
chr12 chr12 57824506 57898078 5m57898008F71M                                                                                                                                                                                                                                                                                                                         GGCAG GTCCAGAAACAGGTGCTGGCTCGACTGCACCACAGGGCATGATCGGCAGACTTTACACTGAGGCTTCTCGC
chr12 chr12 57824506 57898078 5m57898008F71M                                                                                                                                                                                                                                                                                                                         GGCAG GTCCAGAAACATGTGCTGGCTCGACTGCACCACAGGGCATGATCGGCAGACTTTACACTGAGGCTTCTCGC
chr12 chr12 57824506 57898078 5m57898008F71M                                                                                                                                                                                                                                                                                                                         GGCAG GTCCAGAAACAGGTGCTGGCTCGACTGCACCACAGGGCATGATCGGCAGACTTTACACTGAGGCTTCTCGC
chr12 chr12 57824506 57898078 5m57898008F71M                                                                                                                                                                                                                                                                                                                         GGCAG GTCCAGAAACAGGTGCTGGCTCGACTGCACCACAGGGCATGATCGGCAGACTTTACACTGAGGCTTCTCGC
chr12 chr12 57824506 57898078 5m57898008F71M                                                                                                                                                                                                                                                                                                                         GGCAG GTCCAGAAACAGGTGCTGGCTCGACTGCACCACAGGGCATGATCGGCAGACTTTACACTGAGGCTTCTCGC
chr12 chr12 57824506 57898078 5m57898008F71M                                                                                                                                                                                                                                                                                                                         GGCAG GTCCAGAAACAGGTGCTGGCTCGACTGCACCACAGGGCATGATCGGCAGACTTTACACTGAGGCTTCTCGC
chr12 chr12 57824506 57898078 5m57898008F71M                                                                                                                                                                                                                                                                                                                         GGCAG GTCCAGAAACAGGTGCTGGCTCGACTGCACCACAGGGCATGATCGGCAGACTTTACACTGAGGCTTCTCGC
chr12 chr12 57824506 57898078 5m57898008F71M                                                                                                                                                                                                                                                                                                                         GGCAG GTCCAGAAACAGGTGCTGGCTCGACTGCACCACAGGGCATGATCGGCAGACTTTACACTGAGGCTTCTCGC
chr12 chr12 57824506 57898078 5m57898008F71M                                                                                                                                                                                                                                                                                                                         GGCAG GTCCAGAAACAGGTGCTGGCTCGACTGCACCACAGGGCATGATCGGCAGACTTTACACTGAGGCTTCTCGC
chr12 chr12 57824506 57898078 5m57898008F71M                                                                                                                                                                                                                                                                                                                         GGCAG GTCCAGAAACAGGTGCTGGCTCGACTGCACCACAGGGCATGATCGGCAGACTTTACACTGAGGCTTCTCGC
chr12 chr12 57824506 57898078 5m57898008F71M                                                                                                                                                                                                                                                                                                                         GGCAG GTCCAGAAACAGGTGCTGGCTCGACTGCACCACAGGGCATGATCGGCAGACTTTACACTGAGGCTTCTCGC
chr12 chr12 57824506 57898078 5m57898008F71M                                                                                                                                                                                                                                                                                                                         GGCAG GTCCAGAAACAGGTGCTGGCTCGACTGCACCACAGGGCATGATCGGCAGACTTTACACTGAGGCTTCTCGC
chr12 chr12 57824506 57898078 5m57898008F71M                                                                                                                                                                                                                                                                                                                         GGCAG GTCCAGAAACAGGTGCTGGCTCGACTGCACCACAGGGCATGATCGGCAGACTTTACACTGAGGCTTCTCGC
chr12 chr12 57824506 57898078 5m57898008F71M                                                                                                                                                                                                                                                                                                                         GGCAG GTCCAGAAACAGGTGCTGGCTCGACTGCACCACAGGGCATGATCGGCAGACTTTACACTGAGGCTTCTCGC
chr12 chr12 57824506 57898078 5m57898008F71M                                                                                                                                                                                                                                                                                                                         GGCAG GTCCAGAAACAGGTGCTGGCTCGACTGCACCACAGGGCATGATCGGCAGACTTTACACTGAGGCTTCTCGC
chr12 chr12 57824506 57898078 5m57898008F71M                                                                                                                                                                                                                                                                                                                         GGCAG GTCCAGAAACAGGTGCTGGCTCGACTGCACCACAGGGCATGATCGGCAGACTTTACACTGAGGCTTCTCGC
chr12 chr12 57824506 57898078 5m57898008F71M                                                                                                                                                                                                                                                                                                                         GGCAG GTCCAGAAACAGGTGCTGGCTCGACTGCACCACAGGGCATGATCGGCAGACTTTACACTGAGGCTTCTCGC
chr12 chr12 57824506 57898078 5m57898008F71M                                                                                                                                                                                                                                                                                                                         GGCAG GTCCAGAAACAGGTGCTGGCTCGACTGCACCACAGGGCATGATCGGCAGACTTTACACTGAGGCTTCTCGC
chr12 chr12 57824506 57898078 5m57898008F71M                                                                                                                                                                                                                                                                                                                         GGCAG GTCCAGAAACAGGTGCTGGCTCGACTGCACCACAGGGCATGATTGGCAGACTTTACACTGAGGCTTCTCGC
chr12 chr12 57824506 57898078 5m57898008F71M                                                                                                                                                                                                                                                                                                                         GGCAG GTCCAGAAACAGGTGCTGGCTCGACTGCACCACAGGGCATGATCGGCAGACTTTACACTGAGGCTTCTCGC
chr12 chr12 57824506 57898078 5m57898008F71M                                                                                                                                                                                                                                                                                                                         GGCAG GTCCAGAAACAGGTGCTGGCTCGACTGCACCACAGGGCATGATCGGCAGACTTTACACTGAGGCTTCTCGC
chr12 chr12 57824506 57898078 5m57898008F71M                                                                                                                                                                                                                                                                                                                         GGCAG GTCCAGAAACAGGTGCTGGCTCGACTGCACCACAGGGCATGCTCGGCAGACTTTACACTGAGGCTTCTCGC
chr12 chr12 57824506 57898078 5m57898008F71M                                                                                                                                                                                                                                                                                                                         GGCAG GTCCAGAAACAGGTGCTGGCTCGACTGCACCGCAGGGCATGATCGGCAGACTTTACACTGAGGCTTCTCGC
chr12 chr12 57824506 57898078 5m57898008F71M                                                                                                                                                                                                                                                                                                                         GGCAG GTCCAGAAACAGGTGCTGGCTCGACTGCACCACAGGGCATGATCGGCAGACTTTACACTGAGGCTTCTCGC
chr12 chr12 57824505 57898079 4m57898008F72M                                                                                                                                                                                                                                                                                                                          AGGC AGGTCCAGAAACAGGTGCTGGCTCGACTGCACCACAGGGCATGATCGGCAGACTTTACACTGAGGCTTCACG
chr12 chr12 57824505 57898079 4m57898008F72M                                                                                                                                                                                                                                                                                                                          CGAG AAGCCTCAGTGTAAAGTCTGCCGATCATGCCCTGTGGTGCAGTCGAGCCAGCACCTGTTTCTGGACCTGCCA
chr12 chr12 57824505 57898079 4m57898008F72M                                                                                                                                                                                                                                                                                                                          AGGC AGGTCCAGAAACAGGTGCTGGCTCGACTGCGCCACAGGGCATGATCGGCAGACTTTACACTGAGGCTTCTCG
chr12 chr12 57824505 57898079 4m57898008F72M                                                                                                                                                                                                                                                                                                                          AGGC AGGTCCAGAAACAGGTGCTGGCTCGACTCCACCACAGGGCATGATCGGCAGACTTTACACTGCGGCTTCTCG
chr12 chr12 57824505 57898079 4m57898008F72M                                                                                                                                                                                                                                                                                                                          CGAG AAGCCTCAGTGTAAAGTCTGCCGATCATGCCCTGTGGTGCAGTCGAGCCAGCACCTGTTTCTGGACCTGCCA
chr12 chr12 57824505 57898079 4m57898008F72M                                                                                                                                                                                                                                                                                                                          CGAG AAGCCTCAGTGTAAAGTCTGCCGATCATGCCCTGTGGTGCAGTCGAGCCAGCACCTGTTTCTGGACCTGCCA
chr12 chr12 57824505 57898079 4m57898008F72M                                                                                                                                                                                                                                                                                                                          CGAG AAGCCTCAGTGTAAAGTCTGCCGATCATGCCCTGTGGTGCAGTCGAGCCAGCACCTGTTTCTGGACCTGCCA
chr12 chr12 57824505 57898079 4m57898008F72M                                                                                                                                                                                                                                                                                                                          CGAG AAGCCTCAGTGTAAAGTCTGCCGATCATGCCCTGTGGTGCAGTCGAGCCAGCACCTGTTTCTGGACCTGCCA
chr12 chr12 57824505 57898079 4m57898008F72M                                                                                                                                                                                                                                                                                                                          CTAG AAGCCTCAGTGTAAAGTCTGCCGATCATGCCCTGTGGTGCAGTCGAGCCAGCACCTGTTTCTGGACCTGCCA
chr12 chr12 57824505 57898079 4m57898008F72M                                                                                                                                                                                                                                                                                                                          AGGC AGGTCCAGAGACAGGTGCTGGCTCGACTGCACCACAGGGCATGATCGGCAGACTTTACACTGAGGCTTCTCG
chr12 chr12 57898003 57898079 76M                                                                                                                                                                                                                                                                                                                                      TAAGAAGCCTCAGTGTAAAGTCTGCCGATCATGCCCTGTGGTGCAGTCGAGCCAGCACCTGTTTCTGGACCTGCCT
chr12 chr12 57898006 57898082 76M                                                                                                                                                                                                                                                                                                                                         GAAGCCTCAGTGTAAAGTCTCCCGATCATGCCCTGTGGTGCAGTCGAGCCAGCACCTGTTTCTGGACCTGCCTAAG
chr12 chr12 57898009 57898085 76M                                                                                                                                                                                                                                                                                                                                            GCCTCAGTGTAAAGTCTGCCGATCATGCCCTGTGGTGCAGTCGAGCCAGCACCTGTTTCTGGACCTGCCTAAGCTG
chr12 chr12 57898012 57905486 70M7398N6M                                                                                                                                                                                                                                                                                                                                        TCAGTGTAAAGTCTGCCGATCATGCCCTGTGGTGCAGTCGAGCCAGCACCTGTTTCTGGACCTGCCTAAG|||||||||||||||||||||||||||||||||||||||||||||||||||||||||||||||||||||||||||||||||||||||||||||||||||||||||||||||||||||||||||||||||||||||||||||||||||||||||||||||||||||||||||||||||||||||||||||||||||||||||||||||||||||||||||||||
chr12 chr12 57898015 57905489 67M7398N9M                                                                                                                                                                                                                                                                                                                                           GTGTAAAGTCTGCCGATCATGCCCTGAGGTGCAGTCGAGCCAGCACCTGTTTCTGGACCTGCCTAAG|||||||||||||||||||||||||||||||||||||||||||||||||||||||||||||||||||||||||||||||||||||||||||||||||||||||||||||||||||||||||||||||||||||||||||||||||||||||||||||||||||||||||||||||||||||||||||||||||||||||||||||||||||||||||||||||
chr12 chr12 57898018 57905492 64M7398N12M                                                                                                                                                                                                                                                                                                                                             TAAAGTCTGCCGATCATCCCCTGTGGTGCAGTCGAGCCAGCACCTGTTTCTGGACCTGCCTAAG|||||||||||||||||||||||||||||||||||||||||||||||||||||||||||||||||||||||||||||||||||||||||||||||||||||||||||||||||||||||||||||||||||||||||||||||||||||||||||||||||||||||||||||||||||||||||||||||||||||||||||||||||||||||||||||||
chr12 chr12 57898021 57905495 61M7398N15M                                                                                                                                                                                                                                                                                                                                                AGTCTGCCGATCATGCCCTGTGGTGCAGTCGAGCCAGCACCTGTTTCTGGACCTGCCTAAG|||||||||||||||||||||||||||||||||||||||||||||||||||||||||||||||||||||||||||||||||||||||||||||||||||||||||||||||||||||||||||||||||||||||||||||||||||||||||||||||||||||||||||||||||||||||||||||||||||||||||||||||||||||||||||||||
chr12 chr12 57898024 57905498 58M7398N18M                                                                                                                                                                                                                                                                                                                                                   CTGCCGATCATGCCCTGTGGGGCAGTCGAGGCAGCACCTGTTTCTGGACCTGCCTAAG|||||||||||||||||||||||||||||||||||||||||||||||||||||||||||||||||||||||||||||||||||||||||||||||||||||||||||||||||||||||||||||||||||||||||||||||||||||||||||||||||||||||||||||||||||||||||||||||||||||||||||||||||||||||||||||||
chr12 chr12 57898027 57899820 55M1717N21M                                                                                                                                                                                                                                                                                                                                                      CCGATCATGCCCTGTGGTGCAGTCGAGCCAGCACATGTTTCTGGACCTGCCTAAG|||||||||||||||||||||||||||||||||||||||||||||||||||||||||||||||||||||||||||||||||||||||||||||||||||||||||||||||||||||||||||||||||||||||||||||||||||||||||||||||||||||||||||||||||||||||||||||||||||||||||||||||||||||||||||||||
chr12 chr12 57898030 57905504 52M7398N24M                                                                                                                                                                                                                                                                                                                                                         ATCATGCCCTGTGGTGCAGTCGAGCCAGCACCTGTTTCTGGACCTGCCTAAG|||||||||||||||||||||||||||||||||||||||||||||||||||||||||||||||||||||||||||||||||||||||||||||||||||||||||||||||||||||||||||||||||||||||||||||||||||||||||||||||||||||||||||||||||||||||||||||||||||||||||||||||||||||||||||||||
chr12 chr12 57898033 57905507 49M7398N27M                                                                                                                                                                                                                                                                                                                                                            ATGCCCTGTGGTGCAGTCGAGCCAGCACCTGTCTCTGGACCTGCCTAAG|||||||||||||||||||||||||||||||||||||||||||||||||||||||||||||||||||||||||||||||||||||||||||||||||||||||||||||||||||||||||||||||||||||||||||||||||||||||||||||||||||||||||||||||||||||||||||||||||||||||||||||||||||||||||||||||
chr12 chr12 57898036 57905510 46M7398N30M                                                                                                                                                                                                                                                                                                                                                               CCTTGTGGTGCAGTCGAGCCAGCACCTGTTTCTGGACCTGCCTAAG|||||||||||||||||||||||||||||||||||||||||||||||||||||||||||||||||||||||||||||||||||||||||||||||||||||||||||||||||||||||||||||||||||||||||||||||||||||||||||||||||||||||||||||||||||||||||||||||||||||||||||||||||||||||||||||||
chr12 chr12 57898039 57905513 43M7398N33M                                                                                                                                                                                                                                                                                                                                                                  GGTGGTGCAGTCGAGCCAGCACCTGTTTCTGGACCTGCCTAAG|||||||||||||||||||||||||||||||||||||||||||||||||||||||||||||||||||||||||||||||||||||||||||||||||||||||||||||||||||||||||||||||||||||||||||||||||||||||||||||||||||||||||||||||||||||||||||||||||||||||||||||||||||||||||||||||
chr12 chr12 57898042 57905516 40M7398N36M                                                                                                                                                                                                                                                                                                                                                                     GGTGCAGTCGAGCCAGCACCTGTTTCTGGACCTGCCTAAG|||||||||||||||||||||||||||||||||||||||||||||||||||||||||||||||||||||||||||||||||||||||||||||||||||||||||||||||||||||||||||||||||||||||||||||||||||||||||||||||||||||||||||||||||||||||||||||||||||||||||||||||||||||||||||||||
chr12 chr12 57898045 57905519 37M7398N39M                                                                                                                                                                                                                                                                                                                                                                        GCAGTCGAGCCAGCACCTGTTTCTGGACCTGCCTAAG|||||||||||||||||||||||||||||||||||||||||||||||||||||||||||||||||||||||||||||||||||||||||||||||||||||||||||||||||||||||||||||||||||||||||||||||||||||||||||||||||||||||||||||||||||||||||||||||||||||||||||||||||||||||||||||||
chr12 chr12 57898048 57905522 34M7398N42M                                                                                                                                                                                                                                                                                                                                                                           GTCGAGCCAGCACCTGTTTCTGGACCTGCCTAAG|||||||||||||||||||||||||||||||||||||||||||||||||||||||||||||||||||||||||||||||||||||||||||||||||||||||||||||||||||||||||||||||||||||||||||||||||||||||||||||||||||||||||||||||||||||||||||||||||||||||||||||||||||||||||||||||
chr12 chr12 57898051 57905525 31M7398N45M                                                                                                                                                                                                                                                                                                                                                                              GAGCCAGCACCTGTTTCTGGACCTGCCTAAG|||||||||||||||||||||||||||||||||||||||||||||||||||||||||||||||||||||||||||||||||||||||||||||||||||||||||||||||||||||||||||||||||||||||||||||||||||||||||||||||||||||||||||||||||||||||||||||||||||||||||||||||||||||||||||||||
chr12 chr12 57898054 57905528 28M7398N48M                                                                                                                                                                                                                                                                                                                                                                                 CCAGCACCTGTTTCTGGACCTGCCTAAG|||||||||||||||||||||||||||||||||||||||||||||||||||||||||||||||||||||||||||||||||||||||||||||||||||||||||||||||||||||||||||||||||||||||||||||||||||||||||||||||||||||||||||||||||||||||||||||||||||||||||||||||||||||||||||||||
chr12 chr12 57898057 57905531 25M7398N51M                                                                                                                                                                                                                                                                                                                                                                                    GCACCTGTTTCTGGACCTGCCTAAG|||||||||||||||||||||||||||||||||||||||||||||||||||||||||||||||||||||||||||||||||||||||||||||||||||||||||||||||||||||||||||||||||||||||||||||||||||||||||||||||||||||||||||||||||||||||||||||||||||||||||||||||||||||||||||||||
chr12 chr12 57898060 57905534 22M7398N54M                                                                                                                                                                                                                                                                                                                                                                                       CCTGTTTCTGGACCTGCCTAAG|||||||||||||||||||||||||||||||||||||||||||||||||||||||||||||||||||||||||||||||||||||||||||||||||||||||||||||||||||||||||||||||||||||||||||||||||||||||||||||||||||||||||||||||||||||||||||||||||||||||||||||||||||||||||||||||
chr12 chr12 57898063 57905537 19M7398N57M                                                                                                                                                                                                                                                                                                                                                                                          GTTTCTGGACCTGCCTAAG|||||||||||||||||||||||||||||||||||||||||||||||||||||||||||||||||||||||||||||||||||||||||||||||||||||||||||||||||||||||||||||||||||||||||||||||||||||||||||||||||||||||||||||||||||||||||||||||||||||||||||||||||||||||||||||||
chr12 chr12 57898066 57905540 16M7398N60M                                                                                                                                                                                                                                                                                                                                                                                             TCTGGACCTACCTAAG|||||||||||||||||||||||||||||||||||||||||||||||||||||||||||||||||||||||||||||||||||||||||||||||||||||||||||||||||||||||||||||||||||||||||||||||||||||||||||||||||||||||||||||||||||||||||||||||||||||||||||||||||||||||||||||||
chr12 chr12 57898069 57905543 13M7398N63M                                                                                                                                                                                                                                                                                                                                                                                                GGACCTGCCTAAG|||||||||||||||||||||||||||||||||||||||||||||||||||||||||||||||||||||||||||||||||||||||||||||||||||||||||||||||||||||||||||||||||||||||||||||||||||||||||||||||||||||||||||||||||||||||||||||||||||||||||||||||||||||||||||||||
chr12 chr12 57898072 57905546 10M7398N66M                                                                                                                                                                                                                                                                                                                                                                                                   CCTGCCTAAG|||||||||||||||||||||||||||||||||||||||||||||||||||||||||||||||||||||||||||||||||||||||||||||||||||||||||||||||||||||||||||||||||||||||||||||||||||||||||||||||||||||||||||||||||||||||||||||||||||||||||||||||||||||||||||||||
chr12 chr12 57898075 57905549 7M7398N69M                                                                                                                                                                                                                                                                                                                                                                                                       GCCTAAG|||||||||||||||||||||||||||||||||||||||||||||||||||||||||||||||||||||||||||||||||||||||||||||||||||||||||||||||||||||||||||||||||||||||||||||||||||||||||||||||||||||||||||||||||||||||||||||||||||||||||||||||||||||||||||||||
chr12 chr12 57898078 57905552 4M7398N72M                                                                                                                                                                                                                                                                                                                                                                                                          TAAG|||||||||||||||||||||||||||||||||||||||||||||||||||||||||||||||||||||||||||||||||||||||||||||||||||||||||||||||||||||||||||||||||||||||||||||||||||||||||||||||||||||||||||||||||||||||||||||||||||||||||||||||||||||||||||||||
chr12 chr12 57898081 57905555 1M7398N75M                                                                                                                                                                                                                                                                                                                                                                                                             G|||||||||||||||||||||||||||||||||||||||||||||||||||||||||||||||||||||||||||||||||||||||||||||||||||||||||||||||||||||||||||||||||||||||||||||||||||||||||||||||||||||||||||||||||||||||||||||||||||||||||||||||||||||||||||||||
chr12 chr12 57898086 57898162 76M                                                                                                                                                                                                                                                                                                                                                                                                                         GTGAGCTTTTCTCTCAACCTAGTTTTCAGGAGGCCTCTTCTGTCCCCTCTGCCTTAGCCACAAATACTGAGAGCAG
chr12 chr12 57898098 57898174 76M                                                                                                                                                                                                                                                                                                                                                                                                                                     CTCAACCTAGTTTTCAGGAGGCCTCTTCTGTCCCCTCTGCCTTAGCCACAAATACTGAGAGCAGATTACTCTTAAA
chr12 chr12 57898109 57898185 76M                                                                                                                                                                                                                                                                                                                                                                                                                                                TTTCAGGAGGCCTCTTCTGTCCCCTCTGCCTTAGCCACAAATACTGAGAGCAGATTACTCTTAAAAGTTTTAAATT
chr12 chr12 57898119 57898195 76M                                                                                                                                                                                                                                                                                                                                                                                                                                                          CCTCTTCTGTCCCCTCTGCCTTAGCCACAAATACTGAGAGCAGATTACTCTTAAAAGTTTTAAATTTCCATTAAGA
chr12 chr12 57898135 57898211 76M                                                                                                                                                                                                                                                                                                                                                                                                                                                                          TGCCTTAGCCACAAATACTGAGAGCAGATTACTCTTAAAAGTTTTAAATTTCCATTAAGAATTGTACATACTATTG
chr12 chr12 57898146 57898222 76M                                                                                                                                                                                                                                                                                                                                                                                                                                                                                     CAAATACTGAGAGCAGATTACTCTTAAAAGTTTTAAATTTCCATTAAGAATTGTACATACTATTGTCAAGAGACGA
chr12 chr12 57898172 57898248 76M                                                                                                                                                                                                                                                                                                                                                                                                                                                                                                               AAAGTTTTAAATTTCCATTAAGAATTGTCCATACTATTGTCAAGAGATGAAATAGATTAAAGGGCACAAAGTGAAA
chr12 chr12 57898175 57898251 76M                                                                                                                                                                                                                                                                                                                                                                                                                                                                                                                  GTTTTAAATTTCCATTAAGAATTGTACATACTATTGTCAAGAGATGAAATAGATAAAAGGGCACAAAGTGAAAGCC
chr12 chr12 57898178 57898254 76M                                                                                                                                                                                                                                                                                                                                                                                                                                                                                                                     TTAAATTTCCATTAATAATTGTACATACTATTGTCAAGTGATGAAATAGATTAAAGGGCACAAAGTGAAAGCCCCA
chr12 chr12 57898190 57898266 76M                                                                                                                                                                                                                                                                                                                                                                                                                                                                                                                                 TAAGAATTGTACATACTATTGTCAAGAGATGAAATAGATTAAAGGGCACAAAGTGAAAGCCCCAGGCCCACTCGCG
chr12 chr12 57898195 57898271 76M                                                                                                                                                                                                                                                                                                                                                                                                                                                                                                                                      ATTGTACATACTATTGTCAAGAGATGAAATAGATTAAAGGGCACAAAGTGAAAGCCCCAGGCCCACTTGCGAGAGG
chr12 chr12 57898206 57898282 76M                                                                                                                                                                                                                                                                                                                                                                                                                                                                                                                                                 TATTGTCAAGAGATGAAATAGATTAAAGGGCACAAAGTGAAAGCCCCAGGCCCACTTGCGAGAGGTATTTACTACT
chr12 chr12 57898217 57898293 76M                                                                                                                                                                                                                                                                                                                                                                                                                                                                                                                                                            GATGAAATAGATTAAAGGGCACAAAGTGAAAGCCCCAGGCCCACTTGAGAGAGGTATTTACTACTTTTTCCTTTGT
chr12 chr12 57898223 57898299 76M                                                                                                                                                                                                                                                                                                                                                                                                                                                                                                                                                                  ATAGATTAAAGGGCACAAAGTGAAAGCCCCAGGCCCACTTGCGAGAGGTATTTACTACTTTTTCCTTTGTGTCTAT
chr12 chr12 57898228 57898304 76M                                                                                                                                                                                                                                                                                                                                                                                                                                                                                                                                                                       TTAAAGGGCACAAAGTGAAAGCCCCAGGCCCACTTGCGAGAGGGATTTACTACTTTTTCCTTTGTGTCTATTCAGA
chr12 chr12 57898234 57898310 76M                                                                                                                                                                                                                                                                                                                                                                                                                                                                                                                                                                             GGCACAAAGTGAAAGCCCCAGGCCCACTTGCGAGAGGTATTTACTACTTTTTCCTTTGTGTCTATTCAGAA
chr12 chr12 57898237 57898313 76M                                                                                                                                                                                                                                                                                                                                                                                                                                                                                                                                                                                ACAAAGTGAAAGCCCCAGGCCCACTTGCGAGAGGTATTTACTACTTTTTCCTTTGTGTCTATTCAGAA
chr12 chr12 57898249 57898325 76M                                                                                                                                                                                                                                                                                                                                                                                                                                                                                                                                                                                            CCCCAGGCCCACTTGCGAGAGGTATTTACTACTTTTTCCTTTGTGTCTATTCAGAA
chr12 chr12 57898263 57898339 76M                                                                                                                                                                                                                                                                                                                                                                                                                                                                                                                                                                                                          GCGAGAGGTATTTACTACTTTTTCCTTTGTGTCTATTCAGAA
chr12 chr12 57898270 57898346 76M                                                                                                                                                                                                                                                                                                                                                                                                                                                                                                                                                                                                                 GTATTTACTACTTTTTCCTTTGTGTCTATTCAGAA
chr12 chr12 57898273 57898349 76M                                                                                                                                                                                                                                                                                                                                                                                                                                                                                                                                                                                                                    TTTACTACTTTTTCCTTTGTGTCTATTCAGAA
chr12 chr12 57898297 57898373 76M                                                                                                                                                                                                                                                                                                                                                                                                                                                                                                                                                                                                                                            ATTCAGAA 
    2 pairs 
 -168:-1
-3:-5605 
     TCGA-02-2485-01A chr12-chr12 57849876 58166910 rr 
 
8 6 11 23 55 50
AGGAGTGGACAGGTGAAAAGTGAGCAGGGAGCTGTAGGCTGAAGTGGAGT CCTGCAGCGCTGCCAAGATCCCCACGATGCCTGTCCCCGCACCCAGTTCG
11111233334445666666666788888888888888888888888888 88888888888888888888877777777776555544432222222221
INHBE exon2(57849875-57851790) METTL21B exon2(58166798-58166910)
 
 blast search - human genome  
 left 
 >ref|NT_029419.12| Homo sapiens chromosome 12 genomic contig, GRCh37.p9 Primary 
Assembly
 gb|GL000107.1| Homo sapiens chromosome 12 genomic contig, GRCh37 reference primary 
assembly
Length=71516776

 Score = 93.5 bits (50),  Expect = 5e-17
 Identities = 50/50 (100%), Gaps = 0/50 (0%)
 Strand=Plus/Minus

Query  1         AGGAGTGGACAGGTGAAAAGTGAGCAGGGAGCTGTAGGCTGAAGTGGAGT  50
                 ||||||||||||||||||||||||||||||||||||||||||||||||||
Sbjct  19993232  AGGAGTGGACAGGTGAAAAGTGAGCAGGGAGCTGTAGGCTGAAGTGGAGT  19993183


>ref|NW_001838060.2| Homo sapiens chromosome 12 genomic contig, alternate assembly 
HuRef SCAF_1103279188147, whole genome shotgun sequence
 gb|DS486061.1| Homo sapiens SCAF_1103279188147 genomic scaffold, whole genome 
shotgun sequence
Length=18942049

 Score = 93.5 bits (50),  Expect = 5e-17
 Identities = 50/50 (100%), Gaps = 0/50 (0%)
 Strand=Plus/Plus

Query  1         AGGAGTGGACAGGTGAAAAGTGAGCAGGGAGCTGTAGGCTGAAGTGGAGT  50
                 ||||||||||||||||||||||||||||||||||||||||||||||||||
Sbjct  18678778  AGGAGTGGACAGGTGAAAAGTGAGCAGGGAGCTGTAGGCTGAAGTGGAGT  18678827


>ref|NC_000012.11| Homo sapiens chromosome 12, GRCh37.p9 Primary Assembly
 gb|CM000674.1| Homo sapiens chromosome 12, GRCh37 primary reference assembly
Length=133851895

 Score = 93.5 bits (50),  Expect = 5e-17
 Identities = 50/50 (100%), Gaps = 0/50 (0%)
 Strand=Plus/Minus

Query  1         AGGAGTGGACAGGTGAAAAGTGAGCAGGGAGCTGTAGGCTGAAGTGGAGT  50
                 ||||||||||||||||||||||||||||||||||||||||||||||||||
Sbjct  57849926  AGGAGTGGACAGGTGAAAAGTGAGCAGGGAGCTGTAGGCTGAAGTGGAGT  57849877


>ref|AC_000144.1| Homo sapiens chromosome 12, alternate assembly HuRef, whole genome 
shotgun sequence
 gb|CM000473.1| Homo sapiens chromosome 12, whole genome shotgun sequence
Length=130617856

 Score = 93.5 bits (50),  Expect = 5e-17
 Identities = 50/50 (100%), Gaps = 0/50 (0%)
 Strand=Plus/Minus

Query  1         AGGAGTGGACAGGTGAAAAGTGAGCAGGGAGCTGTAGGCTGAAGTGGAGT  50
                 ||||||||||||||||||||||||||||||||||||||||||||||||||
Sbjct  54887799  AGGAGTGGACAGGTGAAAAGTGAGCAGGGAGCTGTAGGCTGAAGTGGAGT  54887750


>gb|CM000263.1| Homo sapiens chromosome 12, whole genome shotgun sequence
Length=133516800

 Score = 93.5 bits (50),  Expect = 5e-17
 Identities = 50/50 (100%), Gaps = 0/50 (0%)
 Strand=Plus/Minus

Query  1         AGGAGTGGACAGGTGAAAAGTGAGCAGGGAGCTGTAGGCTGAAGTGGAGT  50
                 ||||||||||||||||||||||||||||||||||||||||||||||||||
Sbjct  57503913  AGGAGTGGACAGGTGAAAAGTGAGCAGGGAGCTGTAGGCTGAAGTGGAGT  57503864


Lambda     K      H
    1.33    0.621     1.12 

Gapped
Lambda     K      H
    1.28    0.460    0.850 

Effective search space used: 681780354108
 
 right 
 >ref|NT_029419.12| Homo sapiens chromosome 12 genomic contig, GRCh37.p9 Primary 
Assembly
 gb|GL000107.1| Homo sapiens chromosome 12 genomic contig, GRCh37 reference primary 
assembly
Length=71516776

 Score = 93.5 bits (50),  Expect = 5e-17
 Identities = 50/50 (100%), Gaps = 0/50 (0%)
 Strand=Plus/Minus

Query  1         CCTGCAGCGCTGCCAAGATCCCCACGATGCCTGTCCCCGCACCCAGTTCG  50
                 ||||||||||||||||||||||||||||||||||||||||||||||||||
Sbjct  20310217  CCTGCAGCGCTGCCAAGATCCCCACGATGCCTGTCCCCGCACCCAGTTCG  20310168


>ref|NW_001838060.2| Homo sapiens chromosome 12 genomic contig, alternate assembly 
HuRef SCAF_1103279188147, whole genome shotgun sequence
 gb|DS486061.1| Homo sapiens SCAF_1103279188147 genomic scaffold, whole genome 
shotgun sequence
Length=18942049

 Score = 93.5 bits (50),  Expect = 5e-17
 Identities = 50/50 (100%), Gaps = 0/50 (0%)
 Strand=Plus/Plus

Query  1         CCTGCAGCGCTGCCAAGATCCCCACGATGCCTGTCCCCGCACCCAGTTCG  50
                 ||||||||||||||||||||||||||||||||||||||||||||||||||
Sbjct  18362827  CCTGCAGCGCTGCCAAGATCCCCACGATGCCTGTCCCCGCACCCAGTTCG  18362876


>ref|NC_000012.11| Homo sapiens chromosome 12, GRCh37.p9 Primary Assembly
 gb|CM000674.1| Homo sapiens chromosome 12, GRCh37 primary reference assembly
Length=133851895

 Score = 93.5 bits (50),  Expect = 5e-17
 Identities = 50/50 (100%), Gaps = 0/50 (0%)
 Strand=Plus/Minus

Query  1         CCTGCAGCGCTGCCAAGATCCCCACGATGCCTGTCCCCGCACCCAGTTCG  50
                 ||||||||||||||||||||||||||||||||||||||||||||||||||
Sbjct  58166911  CCTGCAGCGCTGCCAAGATCCCCACGATGCCTGTCCCCGCACCCAGTTCG  58166862


>ref|AC_000144.1| Homo sapiens chromosome 12, alternate assembly HuRef, whole genome 
shotgun sequence
 gb|CM000473.1| Homo sapiens chromosome 12, whole genome shotgun sequence
Length=130617856

 Score = 93.5 bits (50),  Expect = 5e-17
 Identities = 50/50 (100%), Gaps = 0/50 (0%)
 Strand=Plus/Minus

Query  1         CCTGCAGCGCTGCCAAGATCCCCACGATGCCTGTCCCCGCACCCAGTTCG  50
                 ||||||||||||||||||||||||||||||||||||||||||||||||||
Sbjct  55203750  CCTGCAGCGCTGCCAAGATCCCCACGATGCCTGTCCCCGCACCCAGTTCG  55203701


>gb|CM000263.1| Homo sapiens chromosome 12, whole genome shotgun sequence
Length=133516800

 Score = 93.5 bits (50),  Expect = 5e-17
 Identities = 50/50 (100%), Gaps = 0/50 (0%)
 Strand=Plus/Minus

Query  1         CCTGCAGCGCTGCCAAGATCCCCACGATGCCTGTCCCCGCACCCAGTTCG  50
                 ||||||||||||||||||||||||||||||||||||||||||||||||||
Sbjct  57824506  CCTGCAGCGCTGCCAAGATCCCCACGATGCCTGTCCCCGCACCCAGTTCG  57824457


Lambda     K      H
    1.33    0.621     1.12 

Gapped
Lambda     K      H
    1.28    0.460    0.850 

Effective search space used: 681780354108
 
 blast search - nt  
 left 
 >ref|XM_003824865.1| PREDICTED: Pan paniscus inhibin, beta E (INHBE), mRNA
Length=2611

 Score = 93.5 bits (50),  Expect = 6e-17
 Identities = 50/50 (100%), Gaps = 0/50 (0%)
 Strand=Plus/Minus

Query  1    AGGAGTGGACAGGTGAAAAGTGAGCAGGGAGCTGTAGGCTGAAGTGGAGT  50
            ||||||||||||||||||||||||||||||||||||||||||||||||||
Sbjct  733  AGGAGTGGACAGGTGAAAAGTGAGCAGGGAGCTGTAGGCTGAAGTGGAGT  684


>ref|NG_029564.1| Homo sapiens GLI family zinc finger 1 (GLI1), RefSeqGene on chromosome 
12
Length=19134

 Score = 93.5 bits (50),  Expect = 6e-17
 Identities = 50/50 (100%), Gaps = 0/50 (0%)
 Strand=Plus/Minus

Query  1     AGGAGTGGACAGGTGAAAAGTGAGCAGGGAGCTGTAGGCTGAAGTGGAGT  50
             ||||||||||||||||||||||||||||||||||||||||||||||||||
Sbjct  1009  AGGAGTGGACAGGTGAAAAGTGAGCAGGGAGCTGTAGGCTGAAGTGGAGT  960


>ref|XM_509161.3| PREDICTED: Pan troglodytes inhibin, beta E (INHBE), mRNA
Length=2610

 Score = 93.5 bits (50),  Expect = 6e-17
 Identities = 50/50 (100%), Gaps = 0/50 (0%)
 Strand=Plus/Minus

Query  1    AGGAGTGGACAGGTGAAAAGTGAGCAGGGAGCTGTAGGCTGAAGTGGAGT  50
            ||||||||||||||||||||||||||||||||||||||||||||||||||
Sbjct  733  AGGAGTGGACAGGTGAAAAGTGAGCAGGGAGCTGTAGGCTGAAGTGGAGT  684


>ref|XM_003252772.1| PREDICTED: Nomascus leucogenys inhibin, beta E (INHBE), mRNA
Length=2584

 Score = 93.5 bits (50),  Expect = 6e-17
 Identities = 50/50 (100%), Gaps = 0/50 (0%)
 Strand=Plus/Minus

Query  1    AGGAGTGGACAGGTGAAAAGTGAGCAGGGAGCTGTAGGCTGAAGTGGAGT  50
            ||||||||||||||||||||||||||||||||||||||||||||||||||
Sbjct  733  AGGAGTGGACAGGTGAAAAGTGAGCAGGGAGCTGTAGGCTGAAGTGGAGT  684


>gb|JF432681.1| Synthetic construct Homo sapiens clone IMAGE:100073919 inhibin, 
beta E (INHBE) gene, encodes complete protein
Length=1152

 Score = 93.5 bits (50),  Expect = 6e-17
 Identities = 50/50 (100%), Gaps = 0/50 (0%)
 Strand=Plus/Minus

Query  1    AGGAGTGGACAGGTGAAAAGTGAGCAGGGAGCTGTAGGCTGAAGTGGAGT  50
            ||||||||||||||||||||||||||||||||||||||||||||||||||
Sbjct  398  AGGAGTGGACAGGTGAAAAGTGAGCAGGGAGCTGTAGGCTGAAGTGGAGT  349


>ref|XM_002823423.1| PREDICTED: Pongo abelii inhibin, beta E (INHBE), mRNA
Length=2608

 Score = 93.5 bits (50),  Expect = 6e-17
 Identities = 50/50 (100%), Gaps = 0/50 (0%)
 Strand=Plus/Minus

Query  1    AGGAGTGGACAGGTGAAAAGTGAGCAGGGAGCTGTAGGCTGAAGTGGAGT  50
            ||||||||||||||||||||||||||||||||||||||||||||||||||
Sbjct  733  AGGAGTGGACAGGTGAAAAGTGAGCAGGGAGCTGTAGGCTGAAGTGGAGT  684


>gb|EU831419.1| Synthetic construct Homo sapiens clone HAIB:100066448; DKFZo008B0617 
inhibin, beta E protein (INHBE) gene, encodes complete 
protein
Length=1096

 Score = 93.5 bits (50),  Expect = 6e-17
 Identities = 50/50 (100%), Gaps = 0/50 (0%)
 Strand=Plus/Minus

Query  1    AGGAGTGGACAGGTGAAAAGTGAGCAGGGAGCTGTAGGCTGAAGTGGAGT  50
            ||||||||||||||||||||||||||||||||||||||||||||||||||
Sbjct  370  AGGAGTGGACAGGTGAAAAGTGAGCAGGGAGCTGTAGGCTGAAGTGGAGT  321


>gb|EU831512.1| Synthetic construct Homo sapiens clone HAIB:100066541; DKFZo004B0618 
inhibin, beta E protein (INHBE) gene, encodes complete 
protein
Length=1096

 Score = 93.5 bits (50),  Expect = 6e-17
 Identities = 50/50 (100%), Gaps = 0/50 (0%)
 Strand=Plus/Minus

Query  1    AGGAGTGGACAGGTGAAAAGTGAGCAGGGAGCTGTAGGCTGAAGTGGAGT  50
            ||||||||||||||||||||||||||||||||||||||||||||||||||
Sbjct  370  AGGAGTGGACAGGTGAAAAGTGAGCAGGGAGCTGTAGGCTGAAGTGGAGT  321


>dbj|AK308647.1| Homo sapiens cDNA, FLJ98688
Length=1228

 Score = 93.5 bits (50),  Expect = 6e-17
 Identities = 50/50 (100%), Gaps = 0/50 (0%)
 Strand=Plus/Minus

Query  1    AGGAGTGGACAGGTGAAAAGTGAGCAGGGAGCTGTAGGCTGAAGTGGAGT  50
            ||||||||||||||||||||||||||||||||||||||||||||||||||
Sbjct  267  AGGAGTGGACAGGTGAAAAGTGAGCAGGGAGCTGTAGGCTGAAGTGGAGT  218


>gb|AC191734.3| Pan troglodytes BAC clone CH251-555H2 from chromosome 12, complete 
sequence
Length=223618

 Score = 93.5 bits (50),  Expect = 6e-17
 Identities = 50/50 (100%), Gaps = 0/50 (0%)
 Strand=Plus/Plus

Query  1       AGGAGTGGACAGGTGAAAAGTGAGCAGGGAGCTGTAGGCTGAAGTGGAGT  50
               ||||||||||||||||||||||||||||||||||||||||||||||||||
Sbjct  123422  AGGAGTGGACAGGTGAAAAGTGAGCAGGGAGCTGTAGGCTGAAGTGGAGT  123471


>gb|AF412024.1| Homo sapiens activin beta E subunit mRNA, complete cds
Length=2459

 Score = 93.5 bits (50),  Expect = 6e-17
 Identities = 50/50 (100%), Gaps = 0/50 (0%)
 Strand=Plus/Minus

Query  1    AGGAGTGGACAGGTGAAAAGTGAGCAGGGAGCTGTAGGCTGAAGTGGAGT  50
            ||||||||||||||||||||||||||||||||||||||||||||||||||
Sbjct  556  AGGAGTGGACAGGTGAAAAGTGAGCAGGGAGCTGTAGGCTGAAGTGGAGT  507


>dbj|AK075285.1| Homo sapiens cDNA FLJ90804 fis, clone Y79AA1000426, highly similar 
to Inhibin beta E chain precursor
Length=1616

 Score = 93.5 bits (50),  Expect = 6e-17
 Identities = 50/50 (100%), Gaps = 0/50 (0%)
 Strand=Plus/Minus

Query  1    AGGAGTGGACAGGTGAAAAGTGAGCAGGGAGCTGTAGGCTGAAGTGGAGT  50
            ||||||||||||||||||||||||||||||||||||||||||||||||||
Sbjct  567  AGGAGTGGACAGGTGAAAAGTGAGCAGGGAGCTGTAGGCTGAAGTGGAGT  518


>gb|BC005161.2| Homo sapiens inhibin, beta E, mRNA (cDNA clone MGC:4638 IMAGE:3529092), 
complete cds
Length=2445

 Score = 93.5 bits (50),  Expect = 6e-17
 Identities = 50/50 (100%), Gaps = 0/50 (0%)
 Strand=Plus/Minus

Query  1    AGGAGTGGACAGGTGAAAAGTGAGCAGGGAGCTGTAGGCTGAAGTGGAGT  50
            ||||||||||||||||||||||||||||||||||||||||||||||||||
Sbjct  564  AGGAGTGGACAGGTGAAAAGTGAGCAGGGAGCTGTAGGCTGAAGTGGAGT  515


>ref|NM_031479.3| Homo sapiens inhibin, beta E (INHBE), mRNA
Length=2453

 Score = 93.5 bits (50),  Expect = 6e-17
 Identities = 50/50 (100%), Gaps = 0/50 (0%)
 Strand=Plus/Minus

Query  1    AGGAGTGGACAGGTGAAAAGTGAGCAGGGAGCTGTAGGCTGAAGTGGAGT  50
            ||||||||||||||||||||||||||||||||||||||||||||||||||
Sbjct  572  AGGAGTGGACAGGTGAAAAGTGAGCAGGGAGCTGTAGGCTGAAGTGGAGT  523


>gb|AC022506.38| Homo sapiens 12 BAC RP11-181L23 (Roswell Park Cancer Institute 
Human BAC Library) complete sequence
Length=172136

 Score = 93.5 bits (50),  Expect = 6e-17
 Identities = 50/50 (100%), Gaps = 0/50 (0%)
 Strand=Plus/Plus

Query  1       AGGAGTGGACAGGTGAAAAGTGAGCAGGGAGCTGTAGGCTGAAGTGGAGT  50
               ||||||||||||||||||||||||||||||||||||||||||||||||||
Sbjct  151943  AGGAGTGGACAGGTGAAAAGTGAGCAGGGAGCTGTAGGCTGAAGTGGAGT  151992


>ref|XM_001115958.1| PREDICTED: Macaca mulatta inhibin, beta E, transcript variant 
2 (INHBE), mRNA
Length=1779

 Score = 82.4 bits (44),  Expect = 1e-13
 Identities = 48/50 (96%), Gaps = 0/50 (0%)
 Strand=Plus/Minus

Query  1    AGGAGTGGACAGGTGAAAAGTGAGCAGGGAGCTGTAGGCTGAAGTGGAGT  50
            |||||||||||||||||| || ||||||||||||||||||||||||||||
Sbjct  733  AGGAGTGGACAGGTGAAAGGTAAGCAGGGAGCTGTAGGCTGAAGTGGAGT  684


Lambda     K      H
    1.33    0.621     1.12 

Gapped
Lambda     K      H
    1.28    0.460    0.850 

Effective search space used: 874911716973
 
 right 
 >ref|XM_003778077.1| PREDICTED: Pongo abelii methyltransferase like 21B, transcript 
variant 2 (METTL21B), mRNA
Length=2870

 Score = 93.5 bits (50),  Expect = 6e-17
 Identities = 50/50 (100%), Gaps = 0/50 (0%)
 Strand=Plus/Minus

Query  1    CCTGCAGCGCTGCCAAGATCCCCACGATGCCTGTCCCCGCACCCAGTTCG  50
            ||||||||||||||||||||||||||||||||||||||||||||||||||
Sbjct  449  CCTGCAGCGCTGCCAAGATCCCCACGATGCCTGTCCCCGCACCCAGTTCG  400


>ref|XM_002823443.2| PREDICTED: Pongo abelii methyltransferase like 21B, transcript 
variant 1 (METTL21B), mRNA
Length=2731

 Score = 93.5 bits (50),  Expect = 6e-17
 Identities = 50/50 (100%), Gaps = 0/50 (0%)
 Strand=Plus/Minus

Query  1    CCTGCAGCGCTGCCAAGATCCCCACGATGCCTGTCCCCGCACCCAGTTCG  50
            ||||||||||||||||||||||||||||||||||||||||||||||||||
Sbjct  449  CCTGCAGCGCTGCCAAGATCCCCACGATGCCTGTCCCCGCACCCAGTTCG  400


>ref|XM_003252747.1| PREDICTED: Nomascus leucogenys protein FAM119B-like (LOC100592795), 
mRNA
Length=2881

 Score = 93.5 bits (50),  Expect = 6e-17
 Identities = 50/50 (100%), Gaps = 0/50 (0%)
 Strand=Plus/Minus

Query  1    CCTGCAGCGCTGCCAAGATCCCCACGATGCCTGTCCCCGCACCCAGTTCG  50
            ||||||||||||||||||||||||||||||||||||||||||||||||||
Sbjct  582  CCTGCAGCGCTGCCAAGATCCCCACGATGCCTGTCCCCGCACCCAGTTCG  533


>ref|NM_001194759.1| Macaca mulatta methyltransferase like 21B (METTL21B), mRNA
Length=1191

 Score = 93.5 bits (50),  Expect = 6e-17
 Identities = 50/50 (100%), Gaps = 0/50 (0%)
 Strand=Plus/Minus

Query  1    CCTGCAGCGCTGCCAAGATCCCCACGATGCCTGTCCCCGCACCCAGTTCG  50
            ||||||||||||||||||||||||||||||||||||||||||||||||||
Sbjct  414  CCTGCAGCGCTGCCAAGATCCCCACGATGCCTGTCCCCGCACCCAGTTCG  365


>dbj|AB528834.1| Synthetic construct DNA, clone: pF1KE0458, Homo sapiens FAM119B 
gene for family with sequence similarity 119, member B, without 
stop codon, in Flexi system
Length=695

 Score = 93.5 bits (50),  Expect = 6e-17
 Identities = 50/50 (100%), Gaps = 0/50 (0%)
 Strand=Plus/Minus

Query  1    CCTGCAGCGCTGCCAAGATCCCCACGATGCCTGTCCCCGCACCCAGTTCG  50
            ||||||||||||||||||||||||||||||||||||||||||||||||||
Sbjct  298  CCTGCAGCGCTGCCAAGATCCCCACGATGCCTGTCCCCGCACCCAGTTCG  249


>emb|CU677624.1| Synthetic construct Homo sapiens gateway clone IMAGE:100016821 
3' read FAM119B mRNA
Length=1236

 Score = 93.5 bits (50),  Expect = 6e-17
 Identities = 50/50 (100%), Gaps = 0/50 (0%)
 Strand=Plus/Plus

Query  1    CCTGCAGCGCTGCCAAGATCCCCACGATGCCTGTCCCCGCACCCAGTTCG  50
            ||||||||||||||||||||||||||||||||||||||||||||||||||
Sbjct  408  CCTGCAGCGCTGCCAAGATCCCCACGATGCCTGTCCCCGCACCCAGTTCG  457


>emb|CU677623.1| Synthetic construct Homo sapiens gateway clone IMAGE:100016821 
5' read FAM119B mRNA
Length=906

 Score = 93.5 bits (50),  Expect = 6e-17
 Identities = 50/50 (100%), Gaps = 0/50 (0%)
 Strand=Plus/Minus

Query  1    CCTGCAGCGCTGCCAAGATCCCCACGATGCCTGTCCCCGCACCCAGTTCG  50
            ||||||||||||||||||||||||||||||||||||||||||||||||||
Sbjct  305  CCTGCAGCGCTGCCAAGATCCCCACGATGCCTGTCCCCGCACCCAGTTCG  256


>dbj|AK292276.1| Homo sapiens cDNA FLJ77367 complete cds, highly similar to Homo 
sapiens hepatocellularcarcinoma-associated antigen HCA557a, 
transcript variant 1, mRNA
Length=1186

 Score = 93.5 bits (50),  Expect = 6e-17
 Identities = 50/50 (100%), Gaps = 0/50 (0%)
 Strand=Plus/Minus

Query  1    CCTGCAGCGCTGCCAAGATCCCCACGATGCCTGTCCCCGCACCCAGTTCG  50
            ||||||||||||||||||||||||||||||||||||||||||||||||||
Sbjct  358  CCTGCAGCGCTGCCAAGATCCCCACGATGCCTGTCCCCGCACCCAGTTCG  309


>emb|AM393762.1| Synthetic construct Homo sapiens clone IMAGE:100002384 for hypothetical 
protein (FAM119B gene)
Length=720

 Score = 93.5 bits (50),  Expect = 6e-17
 Identities = 50/50 (100%), Gaps = 0/50 (0%)
 Strand=Plus/Minus

Query  1    CCTGCAGCGCTGCCAAGATCCCCACGATGCCTGTCCCCGCACCCAGTTCG  50
            ||||||||||||||||||||||||||||||||||||||||||||||||||
Sbjct  309  CCTGCAGCGCTGCCAAGATCCCCACGATGCCTGTCCCCGCACCCAGTTCG  260


>emb|AM393242.1| Synthetic construct Homo sapiens clone IMAGE:100002383 for hypothetical 
protein (FAM119B gene)
Length=720

 Score = 93.5 bits (50),  Expect = 6e-17
 Identities = 50/50 (100%), Gaps = 0/50 (0%)
 Strand=Plus/Minus

Query  1    CCTGCAGCGCTGCCAAGATCCCCACGATGCCTGTCCCCGCACCCAGTTCG  50
            ||||||||||||||||||||||||||||||||||||||||||||||||||
Sbjct  309  CCTGCAGCGCTGCCAAGATCCCCACGATGCCTGTCCCCGCACCCAGTTCG  260


>gb|AC025165.27| Homo sapiens 12 BAC RP11-571M6 (Roswell Park Cancer Institute 
Human BAC Library) complete sequence
Length=211544

 Score = 93.5 bits (50),  Expect = 6e-17
 Identities = 50/50 (100%), Gaps = 0/50 (0%)
 Strand=Plus/Minus

Query  1       CCTGCAGCGCTGCCAAGATCCCCACGATGCCTGTCCCCGCACCCAGTTCG  50
               ||||||||||||||||||||||||||||||||||||||||||||||||||
Sbjct  167047  CCTGCAGCGCTGCCAAGATCCCCACGATGCCTGTCCCCGCACCCAGTTCG  166998


>gb|AF455816.1|AF455816 Homo sapiens hepatocellular carcinoma-associated antigen HCA557a 
mRNA, complete cds
 gb|BC016395.1| Homo sapiens family with sequence similarity 119, member B, mRNA 
(cDNA clone MGC:16818 IMAGE:3882414), complete cds
Length=2624

 Score = 93.5 bits (50),  Expect = 6e-17
 Identities = 50/50 (100%), Gaps = 0/50 (0%)
 Strand=Plus/Minus

Query  1    CCTGCAGCGCTGCCAAGATCCCCACGATGCCTGTCCCCGCACCCAGTTCG  50
            ||||||||||||||||||||||||||||||||||||||||||||||||||
Sbjct  414  CCTGCAGCGCTGCCAAGATCCCCACGATGCCTGTCCCCGCACCCAGTTCG  365


>gb|BC103501.1| Homo sapiens family with sequence similarity 119, member B, transcript 
variant 1, mRNA (cDNA clone MGC:119656 IMAGE:40010152), 
complete cds
Length=1999

 Score = 93.5 bits (50),  Expect = 6e-17
 Identities = 50/50 (100%), Gaps = 0/50 (0%)
 Strand=Plus/Minus

Query  1    CCTGCAGCGCTGCCAAGATCCCCACGATGCCTGTCCCCGCACCCAGTTCG  50
            ||||||||||||||||||||||||||||||||||||||||||||||||||
Sbjct  389  CCTGCAGCGCTGCCAAGATCCCCACGATGCCTGTCCCCGCACCCAGTTCG  340


>gb|BC099841.1| Homo sapiens family with sequence similarity 119, member B, mRNA 
(cDNA clone MGC:119654 IMAGE:40010149), complete cds
Length=2002

 Score = 93.5 bits (50),  Expect = 6e-17
 Identities = 50/50 (100%), Gaps = 0/50 (0%)
 Strand=Plus/Minus

Query  1    CCTGCAGCGCTGCCAAGATCCCCACGATGCCTGTCCCCGCACCCAGTTCG  50
            ||||||||||||||||||||||||||||||||||||||||||||||||||
Sbjct  389  CCTGCAGCGCTGCCAAGATCCCCACGATGCCTGTCCCCGCACCCAGTTCG  340


>ref|NM_015433.2| Homo sapiens methyltransferase like 21B (METTL21B), transcript 
variant 1, mRNA
Length=2717

 Score = 93.5 bits (50),  Expect = 6e-17
 Identities = 50/50 (100%), Gaps = 0/50 (0%)
 Strand=Plus/Minus

Query  1    CCTGCAGCGCTGCCAAGATCCCCACGATGCCTGTCCCCGCACCCAGTTCG  50
            ||||||||||||||||||||||||||||||||||||||||||||||||||
Sbjct  414  CCTGCAGCGCTGCCAAGATCCCCACGATGCCTGTCCCCGCACCCAGTTCG  365


>emb|AL050100.1| Homo sapiens mRNA; cDNA DKFZp586D0919 (from clone DKFZp586D0919)
Length=2777

 Score = 93.5 bits (50),  Expect = 6e-17
 Identities = 50/50 (100%), Gaps = 0/50 (0%)
 Strand=Plus/Minus

Query  1    CCTGCAGCGCTGCCAAGATCCCCACGATGCCTGTCCCCGCACCCAGTTCG  50
            ||||||||||||||||||||||||||||||||||||||||||||||||||
Sbjct  336  CCTGCAGCGCTGCCAAGATCCCCACGATGCCTGTCCCCGCACCCAGTTCG  287


>ref|NM_206914.1| Homo sapiens methyltransferase like 21B (METTL21B), transcript 
variant 2, mRNA
Length=2856

 Score = 93.5 bits (50),  Expect = 6e-17
 Identities = 50/50 (100%), Gaps = 0/50 (0%)
 Strand=Plus/Minus

Query  1    CCTGCAGCGCTGCCAAGATCCCCACGATGCCTGTCCCCGCACCCAGTTCG  50
            ||||||||||||||||||||||||||||||||||||||||||||||||||
Sbjct  414  CCTGCAGCGCTGCCAAGATCCCCACGATGCCTGTCCCCGCACCCAGTTCG  365


>ref|XM_003733791.1| PREDICTED: Callithrix jacchus methyltransferase like 21B, transcript 
variant 2 (METTL21B), mRNA
Length=837

 Score = 91.6 bits (49),  Expect = 2e-16
 Identities = 49/49 (100%), Gaps = 0/49 (0%)
 Strand=Plus/Minus

Query  1    CCTGCAGCGCTGCCAAGATCCCCACGATGCCTGTCCCCGCACCCAGTTC  49
            |||||||||||||||||||||||||||||||||||||||||||||||||
Sbjct  373  CCTGCAGCGCTGCCAAGATCCCCACGATGCCTGTCCCCGCACCCAGTTC  325


>ref|XM_002752670.1| PREDICTED: Callithrix jacchus methyltransferase like 21B, transcript 
variant 1 (METTL21B), mRNA
Length=880

 Score = 91.6 bits (49),  Expect = 2e-16
 Identities = 49/49 (100%), Gaps = 0/49 (0%)
 Strand=Plus/Minus

Query  1    CCTGCAGCGCTGCCAAGATCCCCACGATGCCTGTCCCCGCACCCAGTTC  49
            |||||||||||||||||||||||||||||||||||||||||||||||||
Sbjct  416  CCTGCAGCGCTGCCAAGATCCCCACGATGCCTGTCCCCGCACCCAGTTC  368


>ref|XM_003824831.1| PREDICTED: Pan paniscus methyltransferase like 21B, transcript 
variant 2 (METTL21B), mRNA
Length=3007

 Score = 87.9 bits (47),  Expect = 3e-15
 Identities = 49/50 (98%), Gaps = 0/50 (0%)
 Strand=Plus/Minus

Query  1    CCTGCAGCGCTGCCAAGATCCCCACGATGCCTGTCCCCGCACCCAGTTCG  50
            ||||||||||||||||||| ||||||||||||||||||||||||||||||
Sbjct  582  CCTGCAGCGCTGCCAAGATTCCCACGATGCCTGTCCCCGCACCCAGTTCG  533


>ref|XM_003824830.1| PREDICTED: Pan paniscus methyltransferase like 21B, transcript 
variant 1 (METTL21B), mRNA
Length=2868

 Score = 87.9 bits (47),  Expect = 3e-15
 Identities = 49/50 (98%), Gaps = 0/50 (0%)
 Strand=Plus/Minus

Query  1    CCTGCAGCGCTGCCAAGATCCCCACGATGCCTGTCCCCGCACCCAGTTCG  50
            ||||||||||||||||||| ||||||||||||||||||||||||||||||
Sbjct  582  CCTGCAGCGCTGCCAAGATTCCCACGATGCCTGTCCCCGCACCCAGTTCG  533


>ref|XM_001167287.2| PREDICTED: Pan troglodytes methyltransferase-like protein 21B-like, 
transcript variant 1 (LOC746092), mRNA
Length=2836

 Score = 87.9 bits (47),  Expect = 3e-15
 Identities = 49/50 (98%), Gaps = 0/50 (0%)
 Strand=Plus/Minus

Query  1    CCTGCAGCGCTGCCAAGATCCCCACGATGCCTGTCCCCGCACCCAGTTCG  50
            ||||||||||||||||||| ||||||||||||||||||||||||||||||
Sbjct  408  CCTGCAGCGCTGCCAAGATTCCCACGATGCCTGTCCCCGCACCCAGTTCG  359


>ref|XM_003313545.1| PREDICTED: Pan troglodytes methyltransferase-like protein 21B-like 
(LOC746092), mRNA
Length=2712

 Score = 87.9 bits (47),  Expect = 3e-15
 Identities = 49/50 (98%), Gaps = 0/50 (0%)
 Strand=Plus/Minus

Query  1    CCTGCAGCGCTGCCAAGATCCCCACGATGCCTGTCCCCGCACCCAGTTCG  50
            ||||||||||||||||||| ||||||||||||||||||||||||||||||
Sbjct  423  CCTGCAGCGCTGCCAAGATTCCCACGATGCCTGTCCCCGCACCCAGTTCG  374


Lambda     K      H
    1.33    0.621     1.12 

Gapped
Lambda     K      H
    1.28    0.460    0.850 

Effective search space used: 874911716973
 
    reads 
 chr12 chr12 57850157 57850081 76m                                    GTCTGCAGTCTAGTTGCAGTTTCAGGACACCAGACTTCTCACCCCTCAAGCCACTAGAGGGCAGAGTTAAGGTATG
chr12 chr12 57850153 57850077 76m                                        GCAGTCTAGTTGCAGTTTCAGGACACCAGACTTCTCACCCCTCAAGCCACTAGAGGGCAGAGTTAAGGTATGCCAG
chr12 chr12 57850144 57850068 76m                                                 TTGCAGTTTCAGGACACCAGACTTCTCACCCCTCAAGCCACTAGAGGGCAGAGTTAAGGTATGCCAGCCCAGGTTG
chr12 chr12 57850139 57850063 76m                                                      GTTTCAGGACACCAGACTTCTCACCCCTCAAGCCACTAGAGGGCAGAGTTAAGGTATGCCAGCCCAGGTTGGTGAT
chr12 chr12 57850133 57850057 76m                                                            GGACACCAGACTTCTCCCCCCTCAAGCCACTAGAGGGCAGAGTTAAGGTATGCCAGCCCAGGTTGGTGATGTGGTG
chr12 chr12 57850123 57850047 76m                                                                      CTTCTCACCCCTCAAGCCACTAGAGGGCAGAGTTAAGGTATGCCAGCCCAGGTTGGTGATGTGGTGCTCAGCCAGG
chr12 chr12 57850120 57850044 76m                                                                         CTCACCCCTCAAGCCACTAGAGGGCAGAGTTAAGGTATGCCAGCCCAGGTTGGTGATGTGGTGCTCAGCCAGGAGA
chr12 chr12 57850116 57850040 76m                                                                             CCCCTCAAGCCACTAGAGGGCAGAGTTAAGGTATGCCAGCCCAGGTTGGTGATGTGGTGCTCAGCCAGGAGAGTGC
chr12 chr12 57850113 57850037 76m                                                                                CTCAAGCCACTAGAGGGCAGAGTTAAGGTATGCCAGCCCAGGTTGGTGATGTGGTGCTCAGCCAGGAGAGTGCGGG
chr12 chr12 57850107 57850031 76m                                                                                      CCACTAGAGGGCAGAGTTAAGGTATGCCAGCCCAGGTTGGTGATGTGGTGCTCAGCCAGGAGAGTGCGGGACCCTT
chr12 chr12 57850104 57850028 76m                                                                                         CTAGAGGGCAGAGTTAAGGTATGCCAGCCCAGGTTGGTGATGTGGTGCTCAGCCAGGAGAGTGCGGGACCCTTGGC
chr12 chr12 57850100 57850024 76m                                                                                             AGGGCAGAGTTAAGGTATGCCAGCCCAGGTTGGTGATGTGGTGCTCAGCCAGGAGAGTGCGGGACCCTTGGCGCCT
chr12 chr12 57850097 57850021 76m                                                                                                GCAGAGTTAAGGTATGCCAGCCCAGGTTGGTGATGTGGTGCTCAGCCAGGAGAGTGCGGGACCCTTGGCGCCTCCT
chr12 chr12 57850094 57850018 76m                                                                                                   GAGTTAAGGTATGCCAGCCCAGGTTGGTGATGTGGTGCTCAGCCAGGAGAGTGCGGGACCCTTGGCGCCTCCTCCT
chr12 chr12 57850091 57850015 76m                                                                                                      TTAAGGTATGCCAGCCCAGGTTGGTGATGTGGTGCTCAGCCAGGAGAGTGCGGGACCCTTGGCGCCTCCTCCTTGG
chr12 chr12 57850087 57850011 76m                                                                                                          GGTATGCCAGCCCAGGTTGGTGATGTGGTGCTCAGCCAGGAGAGTGCGGGACCCTTGGCGCCTCCTCCTTGGTCCC
chr12 chr12 57850082 57850006 76m                                                                                                               GCCGGCCCAGGTTGGTGATGTGGTGCTCAGCCAGGAGAGTGCGGGACCCTTGGCGCCTCCTGCTTGGTCCCCATCG
chr12 chr12 57850078 57850002 76m                                                                                                                   GTCCAGGTTGGTGATGTGGTGCTCAGCCAGGAGAGTGCGGGACCCTTGGCGCCTCCTCCTTGGTCCCCATCGGAAG
chr12 chr12 57850074 57849998 76m                                                                                                                       AGGTTGGTGATGTGGTGCTCAGCCAGGAGAGTGCGGGACCCTTGGCGCCTCCTCCTTGGTCCCCATCGGAAGATCC
chr12 chr12 57850070 57849994 76m                                                                                                                           TGGTGATGTGGTGCTCAGCCAGGAGAGTGCGGGACCCTTGGCGCCTCCTCCTTGGTCCCCATCGGAAGATCCTCAA
chr12 chr12 57850065 57849989 76m                                                                                                                                ATGTGGTGCTCAGCCAGGAGAGTGCGGGACCCTTGGCGCCTCCTCCTTGGTCCCCATCGGAAGATCCTCAAGCAAA
chr12 chr12 57850062 57849986 76m                                                                                                                                   TGGTGCTCAGCCAGGAGAGGGCGGGACCCTTGGCGCCTCGTCCTTGGTCCCCATCGGAAGATCCTCAAGCAAAGAG
chr12 chr12 57850059 57849983 76m                                                                                                                                      TGCTCAGCCAGGAGAGTGCGGGACCCTTGGCGCCTCCTCCTTGGTCCCCATCGGAAGATCCTCAAGCAAAGAGTGC
chr12 chr12 57850056 57849980 76m                                                                                                                                         TCAGCCAGGAGAGTGCGGGACCCTTGGCGCCTCCTCCTTGGTCCCCATCGGAAGATCCTCAAGCAAAGAGTGCCAG
chr12 chr12 57850053 57849977 76m                                                                                                                                            GCCAGGAGAGTGCGGGACCCTTGGCGCCTCCTCCTTGGTCCCCATCGGAAGATCCTCAAGCAAAGAGTGCCAGGAA
chr12 chr12 57850046 57849970 76m                                                                                                                                                   GAGTGCGGGACCCTTGGCGCCTCCTCCTTGGTCCCCATCGGAAGATCCTCAAGCAAAGAGTGCCAGGAAGGGAGGG
chr12 chr12 57850042 57849966 76m                                                                                                                                                       GCGGGACCCTTGGCGCCTCCTCCTTGGTCCCCATCGGAAGATCCTCAAGCAAAGAGTGCCAGGAAGGGAGGGGAGC
chr12 chr12 57850039 57849963 76m                                                                                                                                                          GGACCCTTGGCGCCTCCTCCTTGGTCCCCATCGGAAGATCCTCAAGCAAAGAGTGCCAGGAAGGGTGGGGAGCACG
chr12 chr12 57850035 57849959 76m                                                                                                                                                              CCTTGGCGCCTCCTCCTTGGTCCCCATCGGAAGATCCTCAAGCAAAGAGTGCCAGGAAGGGTGGGGAGCACGTGCA
chr12 chr12 57850032 57849956 76m                                                                                                                                                                 CGGCGCCTCCTCCTTGGTCCCCATCGGAAGATCCTCAAGCAAAGAGTGCCAGGAAGGGTGGGGAGCACGTGCAGCC
chr12 chr12 57850029 57849952 39m1d37m                                                                                                                                                               CGCCTCCTCCTTGGTCCCCATCGGAAGATCCTCAAGCAADGAGTGCCAGGAAGGGTGGGGAGCACGTGCAGCCACAG
chr12 chr12 57850026 57849950 76m                                                                                                                                                                       CTCCTCCTTGGTCCCCATCGGAAGATCCTCAAGCAAAGAGTGCCAGGAAGGGTGGGGAGCACGTGCAGCCACAGGC
chr12 chr12 57850023 57849947 76m                                                                                                                                                                          CTCCTTGGTCCCCATCGGAAGATCCTCAAGCAAAGAGTGCCAGGAAGGGTGGGGAGCACGTGCAGCCACAGGCGGG
chr12 chr12 57850017 57849941 76m                                                                                                                                                                                GGTCCCCATCGGAAGATCCTCAAGCAAAGAGTGCCAGGAAGGGTGGGGAGCACGTGCAGCCACAGGCGGGCATGGT
chr12 chr12 57850013 57849937 76m                                                                                                                                                                                    CCCATCGGAAGATCCTCAAGCAAAGAGTGCCAGGAAGTGTGGGGAGCACGTGCAGCCACAGGCGGGCATGGTACAG
chr12 chr12 57850010 57849934 76m                                                                                                                                                                                       ATCGGAAGATCCTCAAGCAAAGAGTGCCAGGAAGGGTGGGGAGCACGTGCAGCCACAGGCGGGCATGGTACAGGTG
chr12 chr12 57850004 57849928 76m                                                                                                                                                                                             AGATCCTCAAGCAAAGAGTGCCAGGAAGGGTGGGGAGCACGTGCAGCCACAGGCGGGCATGGTACAGGTGGGGGGA
chr12 chr12 57850000 57849924 76m                                                                                                                                                                                                 CCTCAAGCAAAGAGTGCCAGGAAGGGTGGGGAGCACGTGCAGCCACAGGCGGGCATGGCACAGGTGGTGGGACCGG
chr12 chr12 57849991 57849915 76m                                                                                                                                                                                                          AAGAGTGCCAGGAAGGGTGGGGAGCACGTGCAGCCACAGGCGGGCATGGTACAGGTGGTGGGACCGAGGAGGGGAC
chr12 chr12 57849988 57849912 76m                                                                                                                                                                                                             AGTGCCAGGAAGGGTGGGGAGCACGTGCAGCCACAGGCGGGCATGGTACAGGTGGTGGGACCGAGGAGTGGACAGG
chr12 chr12 57849982 57849906 76m                                                                                                                                                                                                                   AGGAAGGGTGGGGAGCACGTGCAGCCACAGGCGGGCATGGTACAGGTGGTGGGACCGAGGAGGGGACAGGTGAAAA
chr12 chr12 57849979 57849903 76m                                                                                                                                                                                                                      AAGGGTGGGGAGCACGTGCAGCCACAGGCGGGCATGGTACAGGTGGTGGGACCGAGGAGTGGACAGGTGAAAAGTG
chr12 chr12 57849975 57849899 76m                                                                                                                                                                                                                          GTGGGGAGCACGTGCAGCCACAGGCGGGCATGGTACAGGTGGTGGGACCGAGGAGTGGACAGGTGAAAAGTGAGCA
chr12 chr12 57849972 57849896 76m                                                                                                                                                                                                                             GGGAGCACGTGCAGCCACAGGCGGGCATGGTACAGGTGGTGGGACCGAGGAGTGGACAGGTGAAAAGTGAGCAGGG
chr12 chr12 57849968 57849892 76m                                                                                                                                                                                                                                 GCACGTGCAGCCACAGGCGGGCATGGTACAGGTGGTGGGACCGAGGAGTGGACAGGTGAAAAGTGAGCAGGGAGCT
chr12 chr12 57849963 57849887 76m                                                                                                                                                                                                                                      TGCAGCCACAGGCGGGCATGGGACAGGTGGTGGGACCGAGGAGTGGACAGGTGAAAAGTGAGCAGGGAGCTGTAGG
chr12 chr12 57849960 57849884 76m                                                                                                                                                                                                                                         AGCCACAGGCGGGCATGGTACAGGTGGTGGGACCGAGGAGTGGACAGGTGAAAAGTGAGCAGGGAGCTGTAGGCTG
chr12 chr12 57849957 57849881 76m                                                                                                                                                                                                                                            CACAGGCGGGCATGGTACAGGTGGTGGGACCGAGGAGTGGACAGGTGAAAAGTGAGCAGGGAGCTGTAGGCTGAAG
chr12 chr12 57849953 57849877 76m                                                                                                                                                                                                                                                GGCGGGCATGGTACAGGTGGTGGGACCGAGGAGTGGACAGGTGAAAAGCGAGCAGGGAGCTGTAGGCTGAAGAGGA
chr12 chr12 57849949 57849873 76m                                                                                                                                                                                                                                                    GGCATGGTACAGGTGGTGGGACCGAGGAGTGGACAGGTGAAAAGTGAGCAGGGAGCTGTAGGCTGAAGGGGAGTCT
chr12 chr12 57849947 58166906 72m58166911F4m                                                                                                                                                                                                                                           CATGGTACAGGTGGTGGGACCGAGGAGTGGACAGGTGAAAAGTGAGCAGGGAGCTGTAGGCTGAAGTGGAGT CCTG
chr12 chr12 57849946 58166905 71m58166911F5m                                                                                                                                                                                                                                            ATGGTACAGGTGGTGGGACCGAGGAGTGGACAGGTGAAAAGTGAGCAGGGAGCTGTAGGCTGAAGTGGAGT CCAGC
chr12 chr12 57849941 58166900 66m58166911F10m                                                                                                                                                                                                                                                ACAGGTGGTGGGACCGAGGAGTGGACAGGTGAAAAGTGAGCAGGGAGCTGTAGGCTGAAGTGGAGT CCTGCAGCGC
chr12 chr12 57849941 58166900 66m58166911F10m                                                                                                                                                                                                                                                ACAGGTGGTGGGACCGAGGAGTGGACAGGTGAAAAGTGAGCAGGGAGCTGTAGGCTGAAGTGGAGT CCTGCAGCGC
chr12 chr12 57849934 58166893 59m58166911F17m                                                                                                                                                                                                                                                       GTGGGACCGAGGAGTGGACAGGTGAAAAGTGAGCAGGGAGCTGTAGGCTGAAGTGGAGT CCTGCAGCGCTGCCAAG
chr12 chr12 57849930 58166889 55m58166911F21m                                                                                                                                                                                                                                                           GACCGAGGAGTGGACAGGTGAAAAGTGAGCAGGGAGCTGTAGGCTGAAGTGGAGT CCTGCAGCGCTGCCAAGATCA
chr12 chr12 57849920 58166879 45m58166911F31m                                                                                                                                                                                                                                                                     TGGACAGGTGAAAAGTGAGCAGGGAGCTGTAGGCTGAAGTGGAGT CCTGCAGCGCTGCCAAGATCCCCACGATGCC
chr12 chr12 57849919 58166878 44m58166911F32m                                                                                                                                                                                                                                                                      GGACAGGTGAAAAGTGAGCAGGGAGCTGTAGGCTGAAGTGGAGT CCTGCAGCGCTGCCAAGATCCCCACGATGCCT
chr12 chr12 57849915 58166874 40m58166911F36m                                                                                                                                                                                                                                                                          AGGTGAAAAGTGAGCAGGGAGCTGTAGGCTGAAGTGGAGT CCTGCAGCGCTGCCAAGATCCCCACGATGCCTGTCC
chr12 chr12 57849912 58166871 37m58166911F39m                                                                                                                                                                                                                                                                             TGAAAAGTGAGCAGGGAGCTGTAGGCTGAAGTGGAGT CCTGCAGCGCTGCCAAGATCCCCACGATGCCTGTCCCCG
chr12 chr12 57849911 58166870 36m58166911F40m                                                                                                                                                                                                                                                                              GAAAAGTGAGCAGGGAGCTGTAGGCTGAAGTGGAGT CCTGCAGCGCTGCCAAGATCCCCACGATGCCTGTCCCCGC
chr12 chr12 57849902 58166861 27m58166911F49m                                                                                                                                                                                                                                                                                       GCAGGGAGCTGTAGGCTGAAGTGGAGT CCTGCAGCGCTGCCAAGATCCCCACGATGCCTGTCCCCGCACCCAGTTC
chr12 chr12 57849901 58166860 26m58166911F50m                                                                                                                                                                                                                                                                                        CAGGGAGCTCTAGGCTGAAGTGGAGG CCTGCAGCGCTGCCAAGATCCCCACGATGCCTGTCCCCGCACCCAGTTCG
chr12 chr12 57849889 58166848 14m58166911F62m                                                                                                                                                                                                                                                                                                    GGCTGAAGTGGAGT CCTGCAGCGCTGCCAAGATCCCCACGATGCCTGTCCCCGCACCCAGTTCGATCACCTTCTTG
chr12 chr12 57849883 58166842 8m58166911F68m                                                                                                                                                                                                                                                                                                           AGTGGAGT CCTGCAGCGCTGCCAAGATCCCCACGATGCCTGTCCCCGCACCCAGTTCGATCACCTTCTTGCCTCGG
chr12 chr12 58166914 58166838 76m                                                                                                                                                                                                                                                                                                                           GCTCCCTGCAGCGCTGCCAAGATCCCCACGATGCCTGTCCCCGCACCCAGTTCGATCACCTTCTTGCCTCGGAAAT
chr12 chr12 58166911 58166835 76m                                                                                                                                                                                                                                                                                                                              GCCTGCAGCGCTGCCAAGATCCCCACGATGCCTGTCCCCGCACCCAGTTCGATCACCTTCTTGCCTCGGAAATCCA
chr12 chr12 58166908 58166832 76m                                                                                                                                                                                                                                                                                                                                 TGCAGCGCTGCCAAGATCCCCACGATGCCTGTCCCCGCACCCAGTTCGATCACCTTCTTGCCTCGGAAATCCACAT
chr12 chr12 58166905 58166829 76m                                                                                                                                                                                                                                                                                                                                    AGCGCTGCCAAGATCCCCACGATGCCTGTCCCCGCACCCAGTTCGATCACCTTCTTGCCTCGGAAATCCACATTTT
chr12 chr12 58166902 58166826 76m                                                                                                                                                                                                                                                                                                                                       GCTGCCAAGATCCCCACGATGCCTGTCCCCGCACCCAGTTCGATCACCTTCTTGCCTCGGAAATCCACATTTTGAC
chr12 chr12 58166899 58166823 76m                                                                                                                                                                                                                                                                                                                                          GCCAAGATCCCCACGATGCCTGTCCCCGCACCCAGTTCGATCACCTTCTTGCCTCGGAAATCCACATTTTGACTCT
chr12 chr12 58166896 58166820 76m                                                                                                                                                                                                                                                                                                                                             AAGATCCCCACGATGCCTGTCCCCGCACCCAGTTCGATCACCTTCTTGCCTCGGAAATCCACATTTTGACTCTCGA
chr12 chr12 58166893 58166817 76m                                                                                                                                                                                                                                                                                                                                                ATCCCCACGATGCCTGTCCCCGCACCCAGTTCGATCACCTTCTTGCCTCGGAAATCCACATTTTGACTCTCGAAAT
chr12 chr12 58166890 58166814 76m                                                                                                                                                                                                                                                                                                                                                   CCCACGATGCCTGTCCCCGCACCCAGTTCGATCACCTTCTTGCCTCGGAAATCCACATTTTGACTCTCGAAATAAT
chr12 chr12 58166886 58166810 76m                                                                                                                                                                                                                                                                                                                                                       CGATGCCTGTCCCCGCACCCAGTTCGATCACCTTCTTGCCTCGGAAATCCACATTTTGACTCTCGAAATAATTGCA
chr12 chr12 58166883 58166807 76m                                                                                                                                                                                                                                                                                                                                                          TGCCTGTCCCCGCACCCAGTTCGATCACCTTCTTGCCTCGGAAATCCACATTTTGACTCTCGAAATAATTGCACAG
chr12 chr12 58166880 58166804 76m                                                                                                                                                                                                                                                                                                                                                             CTGTCCCCGCACCCAGTTCGATCACCTTCTTGCCTCGGAAATCCACATTTTGACTCTCGAAATAATTGCACAGGCT
chr12 chr12 58166877 58166801 76m                                                                                                                                                                                                                                                                                                                                                                TCCCCGCACCCAGTTCGATCACCTTCTTGCCTCGGAAATCCACATTTTGACTCTCGAAATAATTGCACAGGCTCAG
chr12 chr12 58166874 58166798 76m                                                                                                                                                                                                                                                                                                                                                                   CCGCACCCAGTTCGATCACCTTCTTGCCTCGGAAATCCACATTTTGACTCTCGAAATAATTGCACAGGCTCAGGGC
chr12 chr12 58166871 58166795 76m                                                                                                                                                                                                                                                                                                                                                                      CACCCAGTTCGATCACCTTCTTGCCTCGGAAATCCACATTTTGACTCTCGAAATAATTGCACAGGCTCAGGGCCTA
chr12 chr12 58166868 58166677 70m115n6m                                                                                                                                                                                                                                                                                                                                                                   CCAGTTCGATCACCTTCTTGCCTCGGAAATCCACATTTTGACTCTCGAAATAATTGCACAGGCTCAGGGC|||||||||||||||||||||||||||||||||||||||||||||||||||||||||||||||||||||||||||||||||||||||||||||||||||||||||||||||||||CGCGTC
chr12 chr12 58166865 58166170 67m619n9m                                                                                                                                                                                                                                                                                                                                                                      GTTCGATCACCTTCTTGCCTCGGAAATCCACATTTTGACTCTCGAAATAATTGCACAGGCTCAGGGC||||||||||||||||||||||||||||||||||||||||||||||||||||||||||||||||||||||||||||||||||||||||||||||||||||||||||||||||||||||||||||||||||||||||||||||||||||||||||||||||||||||||||||||||||||||||||
chr12 chr12 58166862 58166167 64m619n12m                                                                                                                                                                                                                                                                                                                                                                        CGATCACCTTCTTGCCTCGGAAATCCACATTTTGACTCTCGAAACAATTGCACAGGCTCAGGGC||||||||||||||||||||||||||||||||||||||||||||||||||||||||||||||||||||||||||||||||||||||||||||||||||||||||||||||||||||||||||||||||||||||||||||||||||||||||||||||||||||||||||||||||||||||||||
chr12 chr12 58166859 58166668 61m115n15m                                                                                                                                                                                                                                                                                                                                                                           TCACCTTCTTGCCTCGGAAATCCACATTTTGACTCTCGAAATAATTGCACAGGCTCAGGGC|||||||||||||||||||||||||||||||||||||||||||||||||||||||||||||||||||||||||||||||||||||||||||||||||||||||||||||||||||CGCGTCCCACACGCG
chr12 chr12 58166856 58166161 58m619n18m                                                                                                                                                                                                                                                                                                                                                                              CCTTCTTGCCTCGGAAATCCACATTTTGACTCTCGAAATAATTGCACAGGCTCAGGGC||||||||||||||||||||||||||||||||||||||||||||||||||||||||||||||||||||||||||||||||||||||||||||||||||||||||||||||||||||||||||||||||||||||||||||||||||||||||||||||||||||||||||||||||||||||||||
chr12 chr12 58166853 58166662 55m115n21m                                                                                                                                                                                                                                                                                                                                                                                 TCTTGCCTCGGAAATCCACATTTTGACTCTCGAAATAATTGCACAGGCTCAGGGC|||||||||||||||||||||||||||||||||||||||||||||||||||||||||||||||||||||||||||||||||||||||||||||||||||||||||||||||||||CGCGTCCCACACGCGCGCCGC
chr12 chr12 58166850 58166155 52m619n24m                                                                                                                                                                                                                                                                                                                                                                                    TGCCTCGGAAATCCACATTTTGACTCTCGAAATAATTGCACAGGCTCAGGGC||||||||||||||||||||||||||||||||||||||||||||||||||||||||||||||||||||||||||||||||||||||||||||||||||||||||||||||||||||||||||||||||||||||||||||||||||||||||||||||||||||||||||||||||||||||||||
chr12 chr12 58166847 58166152 49m619n27m                                                                                                                                                                                                                                                                                                                                                                                       CTCGGAAATCCACATTTTGGCTCTCGAAATAATTGCATAGGCTCAGGGC||||||||||||||||||||||||||||||||||||||||||||||||||||||||||||||||||||||||||||||||||||||||||||||||||||||||||||||||||||||||||||||||||||||||||||||||||||||||||||||||||||||||||||||||||||||||||
chr12 chr12 58166844 58166149 46m619n30m                                                                                                                                                                                                                                                                                                                                                                                          GGAAATCCACATTTTGACTCTCGAAATAATTGCACAGGCTCAGGGC||||||||||||||||||||||||||||||||||||||||||||||||||||||||||||||||||||||||||||||||||||||||||||||||||||||||||||||||||||||||||||||||||||||||||||||||||||||||||||||||||||||||||||||||||||||||||
chr12 chr12 58166841 58166146 43m619n33m                                                                                                                                                                                                                                                                                                                                                                                             AATCCACATTTTGACTCTCGAAATAATTGCACAGGCTCAGGGC||||||||||||||||||||||||||||||||||||||||||||||||||||||||||||||||||||||||||||||||||||||||||||||||||||||||||||||||||||||||||||||||||||||||||||||||||||||||||||||||||||||||||||||||||||||||||
chr12 chr12 58166838 58166647 40m115n36m                                                                                                                                                                                                                                                                                                                                                                                                CCACATTTTGACTCTCGAAATAATTGCACAGGCTCAGGGC|||||||||||||||||||||||||||||||||||||||||||||||||||||||||||||||||||||||||||||||||||||||||||||||||||||||||||||||||||CGCGTCCCACACGCGCGCCGCCACCCCGAGGCGGGA
chr12 chr12 58166835 58166140 37m619n39m                                                                                                                                                                                                                                                                                                                                                                                                   CATTTTGACTCTCGAAATAATTGCACAGGCTCAGGGC||||||||||||||||||||||||||||||||||||||||||||||||||||||||||||||||||||||||||||||||||||||||||||||||||||||||||||||||||||||||||||||||||||||||||||||||||||||||||||||||||||||||||||||||||||||||||
chr12 chr12 58166832 58150824 34m15932n42m                                                                                                                                                                                                                                                                                                                                                                                                    TCTGACTCTCGAAATAATTGCACAGGCTCAGGGC||||||||||||||||||||||||||||||||||||||||||||||||||||||||||||||||||||||||||||||||||||||||||||||||||||||||||||||||||||||||||||||||||||||||||||||||||||||||||||||||||||||||||||||||||||||||||
chr12 chr12 58166829 58166753 76m                                                                                                                                                                                                                                                                                                                                                                                                                GACTCTCGAAATAATTGCACAGGCTCAGGGCGTACGGGAGAAAAGACAGAGAAAAAGACAGTCGTGAGGATGGCTC
chr12 chr12 58166826 58166131 28m619n48m                                                                                                                                                                                                                                                                                                                                                                                                            CCTCGAAATAATTGCACAGGCTCAGGGC||||||||||||||||||||||||||||||||||||||||||||||||||||||||||||||||||||||||||||||||||||||||||||||||||||||||||||||||||||||||||||||||||||||||||||||||||||||||||||||||||||||||||||||||||||||||||
chr12 chr12 58166823 58166632 25m115n51m                                                                                                                                                                                                                                                                                                                                                                                                               CGAAATAATTGCACAGGCTCAGGGC|||||||||||||||||||||||||||||||||||||||||||||||||||||||||||||||||||||||||||||||||||||||||||||||||||||||||||||||||||CGCGTCCCACACGCGCGCCGCCACCCCGAGGCGGGACCCAAAGTTCTGCGT
chr12 chr12 58166820 58166125 22m619n54m                                                                                                                                                                                                                                                                                                                                                                                                                  AATAATTGCACAGGCTCAGGGC||||||||||||||||||||||||||||||||||||||||||||||||||||||||||||||||||||||||||||||||||||||||||||||||||||||||||||||||||||||||||||||||||||||||||||||||||||||||||||||||||||||||||||||||||||||||||
chr12 chr12 58166816 58166121 18m619n58m                                                                                                                                                                                                                                                                                                                                                                                                                      ATTGCACAGGCTCAGGGC||||||||||||||||||||||||||||||||||||||||||||||||||||||||||||||||||||||||||||||||||||||||||||||||||||||||||||||||||||||||||||||||||||||||||||||||||||||||||||||||||||||||||||||||||||||||||
chr12 chr12 58166813 58166622 15m115n61m                                                                                                                                                                                                                                                                                                                                                                                                                         GCACAGGCTCAGGGC|||||||||||||||||||||||||||||||||||||||||||||||||||||||||||||||||||||||||||||||||||||||||||||||||||||||||||||||||||CGCGTCCCACACGCGCGCCGCCACCCCGAGGCGGGACCCAAAGTTCTGCGTGATGGTCAGC
chr12 chr12 58166810 58166115 12m619n64m                                                                                                                                                                                                                                                                                                                                                                                                                            CAGGCTCAGGGC||||||||||||||||||||||||||||||||||||||||||||||||||||||||||||||||||||||||||||||||||||||||||||||||||||||||||||||||||||||||||||||||||||||||||||||||||||||||||||||||||||||||||||||||||||||||||
chr12 chr12 58166806 58166111 8m619n68m                                                                                                                                                                                                                                                                                                                                                                                                                                 CTCAGGGC||||||||||||||||||||||||||||||||||||||||||||||||||||||||||||||||||||||||||||||||||||||||||||||||||||||||||||||||||||||||||||||||||||||||||||||||||||||||||||||||||||||||||||||||||||||||||
chr12 chr12 58166803 58166612 5m115n71m                                                                                                                                                                                                                                                                                                                                                                                                                                    AGGGC|||||||||||||||||||||||||||||||||||||||||||||||||||||||||||||||||||||||||||||||||||||||||||||||||||||||||||||||||||CGCGTCCCACACGCGCGCCGCCACCCCGAGGCGGGACCCAAAGTTCTGCGTGATGGTCAGCACATGCCCAC
chr12 chr12 58166800 58166105 2m619n74m                                                                                                                                                                                                                                                                                                                                                                                                                                       GC||||||||||||||||||||||||||||||||||||||||||||||||||||||||||||||||||||||||||||||||||||||||||||||||||||||||||||||||||||||||||||||||||||||||||||||||||||||||||||||||||||||||||||||||||||||||||
chr12 chr12 58166761 58166685 76m                                                                                                                                                                                                                                                                                                                                                                                                                                                                                    GATGGCTCCATATGGGAGAGGCGGAGTCCGGGCTCTGGACATCCCACGGACCCTCACCCGGCGCAGCCCATTCCTC
chr12 chr12 58166686 58166610 76m                                                                                                                                                                                                                                                                                                                                                                                                                                                                                                                                                               CACCGCGTCCCACACGCGCGCCGCCACCCCGAGGCGGGACCCAAAGTTCTGCGTGATGGTCAGCACATGCCCAC
chr12 chr12 58166682 58166606 76m                                                                                                                                                                                                                                                                                                                                                                                                                                                                                                                                                                   GCGTCCCACACGCGCGCCGCCACCCCGAGGCGGGACCCAAAGTTCTGCGTGATGGTCAGCACATGCCCAC
chr12 chr12 58166679 58166603 76m                                                                                                                                                                                                                                                                                                                                                                                                                                                                                                                                                                      TCCCACACGCGCGCCGCCACCCCGAGGCGGGACCCAAAGTTCTGCGTGATGGTCAGCACATGCCCAC
chr12 chr12 58166675 58166599 76m                                                                                                                                                                                                                                                                                                                                                                                                                                                                                                                                                                          ACACGCGCGCCGCCACCCCGAGGCGGGACCCAAAGTTCTGCGTGATGGTCAGCACATGCCCAC
chr12 chr12 58166672 58166596 76m                                                                                                                                                                                                                                                                                                                                                                                                                                                                                                                                                                             CGCGCGCCGCCACCCCGAGGCGGGACCCAAAGTTCTGCGTGATGGTCAGCACATGCCCAC
chr12 chr12 58166668 58166592 76m                                                                                                                                                                                                                                                                                                                                                                                                                                                                                                                                                                                 CGCCGCCACCCCGAGGCGGGACCCAAAGTTCTGCGTGATGGTCAGCACATGCCCAC
chr12 chr12 58166665 58166589 76m                                                                                                                                                                                                                                                                                                                                                                                                                                                                                                                                                                                    CGCCACCCCGAGGCGGGACCCAAAGTTCTGCGTGATGGTCAGCACATGCCCAC
chr12 chr12 58166662 58166586 76m                                                                                                                                                                                                                                                                                                                                                                                                                                                                                                                                                                                       CACCCCGAGGCGGGACCCAAAGTTCTGCGTGATGGTCAGCACATGCCCAC
chr12 chr12 58166659 58166583 76m                                                                                                                                                                                                                                                                                                                                                                                                                                                                                                                                                                                          CCCGAGGCGGGACCCAAAGTTCTGCGTGATGGTCAGCACATGCCCAC
chr12 chr12 58166655 58166579 76m                                                                                                                                                                                                                                                                                                                                                                                                                                                                                                                                                                                              AGGCGGGACCCAAAGTTCTGCGTGATGGTCAGCACATGCCCAC 
    6 pairs 
 17:7
0:42
31:64
22:106
-286:26
-666:19 
     TCGA-02-2485-01A chr12-chr12 57856658 57892405 fr 
 
12 46 19 61 54 53
GGCTAGGGCTCCCTCTGCTGGACACTGCAGAGATTGTACACGGGCTGTCC TTGGTCTGCTGTGGAGTGGTGGTGCGACCAAAAATATCAAACGAAATGTT
23444566666677777777777788999999999999999999999999 99999999999999999999999999998887666666555555555555
GLI1 intron1(57853984-57858453) MARS exon9(57892201-57892405)
 
 blast search - human genome  
 left 
 >ref|NT_029419.12| Homo sapiens chromosome 12 genomic contig, GRCh37.p9 Primary 
Assembly
 gb|GL000107.1| Homo sapiens chromosome 12 genomic contig, GRCh37 reference primary 
assembly
Length=71516776

 Score = 93.5 bits (50),  Expect = 5e-17
 Identities = 50/50 (100%), Gaps = 0/50 (0%)
 Strand=Plus/Plus

Query  1         GGCTAGGGCTCCCTCTGCTGGACACTGCAGAGATTGTACACGGGCTGTCC  50
                 ||||||||||||||||||||||||||||||||||||||||||||||||||
Sbjct  19999916  GGCTAGGGCTCCCTCTGCTGGACACTGCAGAGATTGTACACGGGCTGTCC  19999965


>ref|NW_001838060.2| Homo sapiens chromosome 12 genomic contig, alternate assembly 
HuRef SCAF_1103279188147, whole genome shotgun sequence
 gb|DS486061.1| Homo sapiens SCAF_1103279188147 genomic scaffold, whole genome 
shotgun sequence
Length=18942049

 Score = 93.5 bits (50),  Expect = 5e-17
 Identities = 50/50 (100%), Gaps = 0/50 (0%)
 Strand=Plus/Minus

Query  1         GGCTAGGGCTCCCTCTGCTGGACACTGCAGAGATTGTACACGGGCTGTCC  50
                 ||||||||||||||||||||||||||||||||||||||||||||||||||
Sbjct  18672093  GGCTAGGGCTCCCTCTGCTGGACACTGCAGAGATTGTACACGGGCTGTCC  18672044


>ref|NC_000012.11| Homo sapiens chromosome 12, GRCh37.p9 Primary Assembly
 gb|CM000674.1| Homo sapiens chromosome 12, GRCh37 primary reference assembly
Length=133851895

 Score = 93.5 bits (50),  Expect = 5e-17
 Identities = 50/50 (100%), Gaps = 0/50 (0%)
 Strand=Plus/Plus

Query  1         GGCTAGGGCTCCCTCTGCTGGACACTGCAGAGATTGTACACGGGCTGTCC  50
                 ||||||||||||||||||||||||||||||||||||||||||||||||||
Sbjct  57856610  GGCTAGGGCTCCCTCTGCTGGACACTGCAGAGATTGTACACGGGCTGTCC  57856659


>ref|AC_000144.1| Homo sapiens chromosome 12, alternate assembly HuRef, whole genome 
shotgun sequence
 gb|CM000473.1| Homo sapiens chromosome 12, whole genome shotgun sequence
Length=130617856

 Score = 93.5 bits (50),  Expect = 5e-17
 Identities = 50/50 (100%), Gaps = 0/50 (0%)
 Strand=Plus/Plus

Query  1         GGCTAGGGCTCCCTCTGCTGGACACTGCAGAGATTGTACACGGGCTGTCC  50
                 ||||||||||||||||||||||||||||||||||||||||||||||||||
Sbjct  54894484  GGCTAGGGCTCCCTCTGCTGGACACTGCAGAGATTGTACACGGGCTGTCC  54894533


>gb|CM000263.1| Homo sapiens chromosome 12, whole genome shotgun sequence
Length=133516800

 Score = 93.5 bits (50),  Expect = 5e-17
 Identities = 50/50 (100%), Gaps = 0/50 (0%)
 Strand=Plus/Plus

Query  1         GGCTAGGGCTCCCTCTGCTGGACACTGCAGAGATTGTACACGGGCTGTCC  50
                 ||||||||||||||||||||||||||||||||||||||||||||||||||
Sbjct  57510597  GGCTAGGGCTCCCTCTGCTGGACACTGCAGAGATTGTACACGGGCTGTCC  57510646


Lambda     K      H
    1.33    0.621     1.12 

Gapped
Lambda     K      H
    1.28    0.460    0.850 

Effective search space used: 681780354108
 
 right 
 >ref|NT_029419.12| Homo sapiens chromosome 12 genomic contig, GRCh37.p9 Primary 
Assembly
 gb|GL000107.1| Homo sapiens chromosome 12 genomic contig, GRCh37 reference primary 
assembly
Length=71516776

 Score = 93.5 bits (50),  Expect = 5e-17
 Identities = 50/50 (100%), Gaps = 0/50 (0%)
 Strand=Plus/Minus

Query  1         TTGGTCTGCTGTGGAGTGGTGGTGCGACCAAAAATATCAAACGAAATGTT  50
                 ||||||||||||||||||||||||||||||||||||||||||||||||||
Sbjct  20035712  TTGGTCTGCTGTGGAGTGGTGGTGCGACCAAAAATATCAAACGAAATGTT  20035663


>ref|NW_001838060.2| Homo sapiens chromosome 12 genomic contig, alternate assembly 
HuRef SCAF_1103279188147, whole genome shotgun sequence
 gb|DS486061.1| Homo sapiens SCAF_1103279188147 genomic scaffold, whole genome 
shotgun sequence
Length=18942049

 Score = 93.5 bits (50),  Expect = 5e-17
 Identities = 50/50 (100%), Gaps = 0/50 (0%)
 Strand=Plus/Plus

Query  1         TTGGTCTGCTGTGGAGTGGTGGTGCGACCAAAAATATCAAACGAAATGTT  50
                 ||||||||||||||||||||||||||||||||||||||||||||||||||
Sbjct  18636095  TTGGTCTGCTGTGGAGTGGTGGTGCGACCAAAAATATCAAACGAAATGTT  18636144


>ref|NC_000012.11| Homo sapiens chromosome 12, GRCh37.p9 Primary Assembly
 gb|CM000674.1| Homo sapiens chromosome 12, GRCh37 primary reference assembly
Length=133851895

 Score = 93.5 bits (50),  Expect = 5e-17
 Identities = 50/50 (100%), Gaps = 0/50 (0%)
 Strand=Plus/Minus

Query  1         TTGGTCTGCTGTGGAGTGGTGGTGCGACCAAAAATATCAAACGAAATGTT  50
                 ||||||||||||||||||||||||||||||||||||||||||||||||||
Sbjct  57892406  TTGGTCTGCTGTGGAGTGGTGGTGCGACCAAAAATATCAAACGAAATGTT  57892357


>ref|AC_000144.1| Homo sapiens chromosome 12, alternate assembly HuRef, whole genome 
shotgun sequence
 gb|CM000473.1| Homo sapiens chromosome 12, whole genome shotgun sequence
Length=130617856

 Score = 93.5 bits (50),  Expect = 5e-17
 Identities = 50/50 (100%), Gaps = 0/50 (0%)
 Strand=Plus/Minus

Query  1         TTGGTCTGCTGTGGAGTGGTGGTGCGACCAAAAATATCAAACGAAATGTT  50
                 ||||||||||||||||||||||||||||||||||||||||||||||||||
Sbjct  54930482  TTGGTCTGCTGTGGAGTGGTGGTGCGACCAAAAATATCAAACGAAATGTT  54930433


>gb|CM000263.1| Homo sapiens chromosome 12, whole genome shotgun sequence
Length=133516800

 Score = 93.5 bits (50),  Expect = 5e-17
 Identities = 50/50 (100%), Gaps = 0/50 (0%)
 Strand=Plus/Minus

Query  1         TTGGTCTGCTGTGGAGTGGTGGTGCGACCAAAAATATCAAACGAAATGTT  50
                 ||||||||||||||||||||||||||||||||||||||||||||||||||
Sbjct  57548187  TTGGTCTGCTGTGGAGTGGTGGTGCGACCAAAAATATCAAACGAAATGTT  57548138


Lambda     K      H
    1.33    0.621     1.12 

Gapped
Lambda     K      H
    1.28    0.460    0.850 

Effective search space used: 681780354108
 
 blast search - nt  
 left 
 >ref|NG_029564.1| Homo sapiens GLI family zinc finger 1 (GLI1), RefSeqGene on chromosome 
12
Length=19134

 Score = 93.5 bits (50),  Expect = 6e-17
 Identities = 50/50 (100%), Gaps = 0/50 (0%)
 Strand=Plus/Plus

Query  1     GGCTAGGGCTCCCTCTGCTGGACACTGCAGAGATTGTACACGGGCTGTCC  50
             ||||||||||||||||||||||||||||||||||||||||||||||||||
Sbjct  7693  GGCTAGGGCTCCCTCTGCTGGACACTGCAGAGATTGTACACGGGCTGTCC  7742


>gb|AC191734.3| Pan troglodytes BAC clone CH251-555H2 from chromosome 12, complete 
sequence
Length=223618

 Score = 93.5 bits (50),  Expect = 6e-17
 Identities = 50/50 (100%), Gaps = 0/50 (0%)
 Strand=Plus/Minus

Query  1       GGCTAGGGCTCCCTCTGCTGGACACTGCAGAGATTGTACACGGGCTGTCC  50
               ||||||||||||||||||||||||||||||||||||||||||||||||||
Sbjct  116738  GGCTAGGGCTCCCTCTGCTGGACACTGCAGAGATTGTACACGGGCTGTCC  116689


>gb|AF316573.1| Homo sapiens GLI1 gene, exons 2-12 and complete cds
Length=10163

 Score = 93.5 bits (50),  Expect = 6e-17
 Identities = 50/50 (100%), Gaps = 0/50 (0%)
 Strand=Plus/Plus

Query  1    GGCTAGGGCTCCCTCTGCTGGACACTGCAGAGATTGTACACGGGCTGTCC  50
            ||||||||||||||||||||||||||||||||||||||||||||||||||
Sbjct  739  GGCTAGGGCTCCCTCTGCTGGACACTGCAGAGATTGTACACGGGCTGTCC  788


>gb|AC022506.38| Homo sapiens 12 BAC RP11-181L23 (Roswell Park Cancer Institute 
Human BAC Library) complete sequence
Length=172136

 Score = 93.5 bits (50),  Expect = 6e-17
 Identities = 50/50 (100%), Gaps = 0/50 (0%)
 Strand=Plus/Minus

Query  1       GGCTAGGGCTCCCTCTGCTGGACACTGCAGAGATTGTACACGGGCTGTCC  50
               ||||||||||||||||||||||||||||||||||||||||||||||||||
Sbjct  145259  GGCTAGGGCTCCCTCTGCTGGACACTGCAGAGATTGTACACGGGCTGTCC  145210


Lambda     K      H
    1.33    0.621     1.12 

Gapped
Lambda     K      H
    1.28    0.460    0.850 

Effective search space used: 874911716973
 
 right 
 >ref|XM_509164.3| PREDICTED: Pan troglodytes methionyl-tRNA synthetase (MARS), 
mRNA
Length=3469

 Score = 93.5 bits (50),  Expect = 6e-17
 Identities = 50/50 (100%), Gaps = 0/50 (0%)
 Strand=Plus/Minus

Query  1     TTGGTCTGCTGTGGAGTGGTGGTGCGACCAAAAATATCAAACGAAATGTT  50
             ||||||||||||||||||||||||||||||||||||||||||||||||||
Sbjct  1782  TTGGTCTGCTGTGGAGTGGTGGTGCGACCAAAAATATCAAACGAAATGTT  1733


>ref|XM_003252767.1| PREDICTED: Nomascus leucogenys methionyl-tRNA synthetase (MARS), 
mRNA
Length=2916

 Score = 93.5 bits (50),  Expect = 6e-17
 Identities = 50/50 (100%), Gaps = 0/50 (0%)
 Strand=Plus/Minus

Query  1     TTGGTCTGCTGTGGAGTGGTGGTGCGACCAAAAATATCAAACGAAATGTT  50
             ||||||||||||||||||||||||||||||||||||||||||||||||||
Sbjct  1229  TTGGTCTGCTGTGGAGTGGTGGTGCGACCAAAAATATCAAACGAAATGTT  1180


>ref|NM_004990.3| Homo sapiens methionyl-tRNA synthetase (MARS), mRNA
Length=2932

 Score = 93.5 bits (50),  Expect = 6e-17
 Identities = 50/50 (100%), Gaps = 0/50 (0%)
 Strand=Plus/Minus

Query  1     TTGGTCTGCTGTGGAGTGGTGGTGCGACCAAAAATATCAAACGAAATGTT  50
             ||||||||||||||||||||||||||||||||||||||||||||||||||
Sbjct  1229  TTGGTCTGCTGTGGAGTGGTGGTGCGACCAAAAATATCAAACGAAATGTT  1180


>dbj|AB590802.1| Synthetic construct DNA, clone: pFN21AB8542, Homo sapiens MARS 
gene for methionyl-tRNA synthetase, without stop codon, in 
Flexi system
Length=2717

 Score = 93.5 bits (50),  Expect = 6e-17
 Identities = 50/50 (100%), Gaps = 0/50 (0%)
 Strand=Plus/Minus

Query  1     TTGGTCTGCTGTGGAGTGGTGGTGCGACCAAAAATATCAAACGAAATGTT  50
             ||||||||||||||||||||||||||||||||||||||||||||||||||
Sbjct  1100  TTGGTCTGCTGTGGAGTGGTGGTGCGACCAAAAATATCAAACGAAATGTT  1051


>dbj|AK303349.1| Homo sapiens cDNA FLJ55046 complete cds, highly similar to Methionyl-tRNA 
synthetase (EC 6.1.1.10)
Length=1328

 Score = 93.5 bits (50),  Expect = 6e-17
 Identities = 50/50 (100%), Gaps = 0/50 (0%)
 Strand=Plus/Minus

Query  1    TTGGTCTGCTGTGGAGTGGTGGTGCGACCAAAAATATCAAACGAAATGTT  50
            ||||||||||||||||||||||||||||||||||||||||||||||||||
Sbjct  733  TTGGTCTGCTGTGGAGTGGTGGTGCGACCAAAAATATCAAACGAAATGTT  684


>dbj|AK293945.1| Homo sapiens cDNA FLJ53309 complete cds, highly similar to Methionyl-tRNA 
synthetase (EC 6.1.1.10)
Length=2702

 Score = 93.5 bits (50),  Expect = 6e-17
 Identities = 50/50 (100%), Gaps = 0/50 (0%)
 Strand=Plus/Minus

Query  1     TTGGTCTGCTGTGGAGTGGTGGTGCGACCAAAAATATCAAACGAAATGTT  50
             ||||||||||||||||||||||||||||||||||||||||||||||||||
Sbjct  1040  TTGGTCTGCTGTGGAGTGGTGGTGCGACCAAAAATATCAAACGAAATGTT  991


>dbj|AK290857.1| Homo sapiens cDNA FLJ76789 complete cds, highly similar to Homo 
sapiens methionine-tRNA synthetase (MARS), mRNA
Length=2780

 Score = 93.5 bits (50),  Expect = 6e-17
 Identities = 50/50 (100%), Gaps = 0/50 (0%)
 Strand=Plus/Minus

Query  1     TTGGTCTGCTGTGGAGTGGTGGTGCGACCAAAAATATCAAACGAAATGTT  50
             ||||||||||||||||||||||||||||||||||||||||||||||||||
Sbjct  1100  TTGGTCTGCTGTGGAGTGGTGGTGCGACCAAAAATATCAAACGAAATGTT  1051


>gb|AC191734.3| Pan troglodytes BAC clone CH251-555H2 from chromosome 12, complete 
sequence
Length=223618

 Score = 93.5 bits (50),  Expect = 6e-17
 Identities = 50/50 (100%), Gaps = 0/50 (0%)
 Strand=Plus/Plus

Query  1      TTGGTCTGCTGTGGAGTGGTGGTGCGACCAAAAATATCAAACGAAATGTT  50
              ||||||||||||||||||||||||||||||||||||||||||||||||||
Sbjct  81704  TTGGTCTGCTGTGGAGTGGTGGTGCGACCAAAAATATCAAACGAAATGTT  81753


>gb|BC004197.1| Homo sapiens mRNA similar to advillin (cDNA clone IMAGE:2961396)
Length=3147

 Score = 93.5 bits (50),  Expect = 6e-17
 Identities = 50/50 (100%), Gaps = 0/50 (0%)
 Strand=Plus/Minus

Query  1     TTGGTCTGCTGTGGAGTGGTGGTGCGACCAAAAATATCAAACGAAATGTT  50
             ||||||||||||||||||||||||||||||||||||||||||||||||||
Sbjct  1118  TTGGTCTGCTGTGGAGTGGTGGTGCGACCAAAAATATCAAACGAAATGTT  1069


>gb|BC004134.1| Homo sapiens mRNA similar to advillin (cDNA clone IMAGE:2961396)
Length=3147

 Score = 93.5 bits (50),  Expect = 6e-17
 Identities = 50/50 (100%), Gaps = 0/50 (0%)
 Strand=Plus/Minus

Query  1     TTGGTCTGCTGTGGAGTGGTGGTGCGACCAAAAATATCAAACGAAATGTT  50
             ||||||||||||||||||||||||||||||||||||||||||||||||||
Sbjct  1118  TTGGTCTGCTGTGGAGTGGTGGTGCGACCAAAAATATCAAACGAAATGTT  1069


>gb|BC065187.1| Homo sapiens methionine-tRNA synthetase, mRNA (cDNA clone IMAGE:5021529), 
partial cds
Length=2830

 Score = 93.5 bits (50),  Expect = 6e-17
 Identities = 50/50 (100%), Gaps = 0/50 (0%)
 Strand=Plus/Minus

Query  1     TTGGTCTGCTGTGGAGTGGTGGTGCGACCAAAAATATCAAACGAAATGTT  50
             ||||||||||||||||||||||||||||||||||||||||||||||||||
Sbjct  1126  TTGGTCTGCTGTGGAGTGGTGGTGCGACCAAAAATATCAAACGAAATGTT  1077


>dbj|AK122956.1| Homo sapiens cDNA FLJ16674 fis, clone THYMU3008136, highly  similar 
to Methionyl-tRNA synthetase (EC 6.1.1.10)
Length=3574

 Score = 93.5 bits (50),  Expect = 6e-17
 Identities = 50/50 (100%), Gaps = 0/50 (0%)
 Strand=Plus/Minus

Query  1     TTGGTCTGCTGTGGAGTGGTGGTGCGACCAAAAATATCAAACGAAATGTT  50
             ||||||||||||||||||||||||||||||||||||||||||||||||||
Sbjct  1777  TTGGTCTGCTGTGGAGTGGTGGTGCGACCAAAAATATCAAACGAAATGTT  1728


>gb|BT007338.1| Homo sapiens methionine-tRNA synthetase mRNA, complete cds
Length=2703

 Score = 93.5 bits (50),  Expect = 6e-17
 Identities = 50/50 (100%), Gaps = 0/50 (0%)
 Strand=Plus/Minus

Query  1     TTGGTCTGCTGTGGAGTGGTGGTGCGACCAAAAATATCAAACGAAATGTT  50
             ||||||||||||||||||||||||||||||||||||||||||||||||||
Sbjct  1091  TTGGTCTGCTGTGGAGTGGTGGTGCGACCAAAAATATCAAACGAAATGTT  1042


>dbj|AK222767.1| Homo sapiens mRNA for methionine-tRNA synthetase variant, clone: 
HEP00371
Length=2738

 Score = 93.5 bits (50),  Expect = 6e-17
 Identities = 50/50 (100%), Gaps = 0/50 (0%)
 Strand=Plus/Minus

Query  1     TTGGTCTGCTGTGGAGTGGTGGTGCGACCAAAAATATCAAACGAAATGTT  50
             ||||||||||||||||||||||||||||||||||||||||||||||||||
Sbjct  1111  TTGGTCTGCTGTGGAGTGGTGGTGCGACCAAAAATATCAAACGAAATGTT  1062


>gb|BC015011.1| Homo sapiens methionyl-tRNA synthetase, mRNA (cDNA clone IMAGE:4864644), 
partial cds
Length=2790

 Score = 93.5 bits (50),  Expect = 6e-17
 Identities = 50/50 (100%), Gaps = 0/50 (0%)
 Strand=Plus/Minus

Query  1     TTGGTCTGCTGTGGAGTGGTGGTGCGACCAAAAATATCAAACGAAATGTT  50
             ||||||||||||||||||||||||||||||||||||||||||||||||||
Sbjct  1089  TTGGTCTGCTGTGGAGTGGTGGTGCGACCAAAAATATCAAACGAAATGTT  1040


>gb|AC022506.38| Homo sapiens 12 BAC RP11-181L23 (Roswell Park Cancer Institute 
Human BAC Library) complete sequence
Length=172136

 Score = 93.5 bits (50),  Expect = 6e-17
 Identities = 50/50 (100%), Gaps = 0/50 (0%)
 Strand=Plus/Plus

Query  1       TTGGTCTGCTGTGGAGTGGTGGTGCGACCAAAAATATCAAACGAAATGTT  50
               ||||||||||||||||||||||||||||||||||||||||||||||||||
Sbjct  109463  TTGGTCTGCTGTGGAGTGGTGGTGCGACCAAAAATATCAAACGAAATGTT  109512


>emb|X94754.1| H.sapiens mRNA for yeast methionyl-tRNA synthetase homologue
Length=2779

 Score = 93.5 bits (50),  Expect = 6e-17
 Identities = 50/50 (100%), Gaps = 0/50 (0%)
 Strand=Plus/Minus

Query  1     TTGGTCTGCTGTGGAGTGGTGGTGCGACCAAAAATATCAAACGAAATGTT  50
             ||||||||||||||||||||||||||||||||||||||||||||||||||
Sbjct  1098  TTGGTCTGCTGTGGAGTGGTGGTGCGACCAAAAATATCAAACGAAATGTT  1049


>gb|AY889906.1| Synthetic construct Homo sapiens clone FLH022775.01X methionine-tRNA 
synthetase (MARS) mRNA, complete cds
Length=2703

 Score = 93.5 bits (50),  Expect = 6e-17
 Identities = 50/50 (100%), Gaps = 0/50 (0%)
 Strand=Plus/Minus

Query  1     TTGGTCTGCTGTGGAGTGGTGGTGCGACCAAAAATATCAAACGAAATGTT  50
             ||||||||||||||||||||||||||||||||||||||||||||||||||
Sbjct  1091  TTGGTCTGCTGTGGAGTGGTGGTGCGACCAAAAATATCAAACGAAATGTT  1042


>gb|BC011849.2| Homo sapiens methionyl-tRNA synthetase, mRNA (cDNA clone MGC:20313 
IMAGE:4135154), complete cds
Length=2800

 Score = 93.5 bits (50),  Expect = 6e-17
 Identities = 50/50 (100%), Gaps = 0/50 (0%)
 Strand=Plus/Minus

Query  1     TTGGTCTGCTGTGGAGTGGTGGTGCGACCAAAAATATCAAACGAAATGTT  50
             ||||||||||||||||||||||||||||||||||||||||||||||||||
Sbjct  1097  TTGGTCTGCTGTGGAGTGGTGGTGCGACCAAAAATATCAAACGAAATGTT  1048


>gb|BC011639.2| Homo sapiens methionyl-tRNA synthetase, mRNA (cDNA clone MGC:12828 
IMAGE:4120680), complete cds
Length=2799

 Score = 93.5 bits (50),  Expect = 6e-17
 Identities = 50/50 (100%), Gaps = 0/50 (0%)
 Strand=Plus/Minus

Query  1     TTGGTCTGCTGTGGAGTGGTGGTGCGACCAAAAATATCAAACGAAATGTT  50
             ||||||||||||||||||||||||||||||||||||||||||||||||||
Sbjct  1099  TTGGTCTGCTGTGGAGTGGTGGTGCGACCAAAAATATCAAACGAAATGTT  1050


>gb|BC006328.2| Homo sapiens methionyl-tRNA synthetase, mRNA (cDNA clone MGC:12889 
IMAGE:4139330), complete cds
Length=2795

 Score = 93.5 bits (50),  Expect = 6e-17
 Identities = 50/50 (100%), Gaps = 0/50 (0%)
 Strand=Plus/Minus

Query  1     TTGGTCTGCTGTGGAGTGGTGGTGCGACCAAAAATATCAAACGAAATGTT  50
             ||||||||||||||||||||||||||||||||||||||||||||||||||
Sbjct  1097  TTGGTCTGCTGTGGAGTGGTGGTGCGACCAAAAATATCAAACGAAATGTT  1048


>gb|BC002384.2| Homo sapiens methionyl-tRNA synthetase, mRNA (cDNA clone MGC:8646 
IMAGE:2961615), complete cds
Length=2799

 Score = 93.5 bits (50),  Expect = 6e-17
 Identities = 50/50 (100%), Gaps = 0/50 (0%)
 Strand=Plus/Minus

Query  1     TTGGTCTGCTGTGGAGTGGTGGTGCGACCAAAAATATCAAACGAAATGTT  50
             ||||||||||||||||||||||||||||||||||||||||||||||||||
Sbjct  1097  TTGGTCTGCTGTGGAGTGGTGGTGCGACCAAAAATATCAAACGAAATGTT  1048


>dbj|D84224.1| Homo sapiens mRNA for methionyl tRNA synthetase, complete cds
Length=2800

 Score = 93.5 bits (50),  Expect = 6e-17
 Identities = 50/50 (100%), Gaps = 0/50 (0%)
 Strand=Plus/Minus

Query  1     TTGGTCTGCTGTGGAGTGGTGGTGCGACCAAAAATATCAAACGAAATGTT  50
             ||||||||||||||||||||||||||||||||||||||||||||||||||
Sbjct  1114  TTGGTCTGCTGTGGAGTGGTGGTGCGACCAAAAATATCAAACGAAATGTT  1065


>ref|XM_003824853.1| PREDICTED: Pan paniscus methionyl-tRNA synthetase (MARS), mRNA
Length=3665

 Score = 87.9 bits (47),  Expect = 3e-15
 Identities = 47/47 (100%), Gaps = 0/47 (0%)
 Strand=Plus/Minus

Query  4     GTCTGCTGTGGAGTGGTGGTGCGACCAAAAATATCAAACGAAATGTT  50
             |||||||||||||||||||||||||||||||||||||||||||||||
Sbjct  1779  GTCTGCTGTGGAGTGGTGGTGCGACCAAAAATATCAAACGAAATGTT  1733


>ref|NM_001266948.1| Macaca mulatta methionyl-tRNA synthetase (MARS), mRNA
Length=2786

 Score = 82.4 bits (44),  Expect = 1e-13
 Identities = 48/50 (96%), Gaps = 0/50 (0%)
 Strand=Plus/Minus

Query  1     TTGGTCTGCTGTGGAGTGGTGGTGCGACCAAAAATATCAAACGAAATGTT  50
             ||||||||||||||||||||||||||||||||| ||||||| ||||||||
Sbjct  1123  TTGGTCTGCTGTGGAGTGGTGGTGCGACCAAAAGTATCAAATGAAATGTT  1074


>ref|XM_003476145.1| PREDICTED: Cavia porcellus methionyl-tRNA synthetase, cytoplasmic-like, 
transcript variant 2 (LOC100721918), mRNA
Length=2727

 Score = 76.8 bits (41),  Expect = 7e-12
 Identities = 46/48 (95%), Gaps = 2/48 (4%)
 Strand=Plus/Minus

Query  1     TTGGTCTGCTGTGGAGTGGTGGTGCGACCAAAAATA-TCAAACGAAAT  47
             |||||||||||||||||||||||||||||||||| | |||||||||||
Sbjct  1091  TTGGTCTGCTGTGGAGTGGTGGTGCGACCAAAAA-AGTCAAACGAAAT  1045


>ref|XM_003476144.1| PREDICTED: Cavia porcellus methionyl-tRNA synthetase, cytoplasmic-like, 
transcript variant 1 (LOC100721918), mRNA
Length=2703

 Score = 76.8 bits (41),  Expect = 7e-12
 Identities = 46/48 (95%), Gaps = 2/48 (4%)
 Strand=Plus/Minus

Query  1     TTGGTCTGCTGTGGAGTGGTGGTGCGACCAAAAATA-TCAAACGAAAT  47
             |||||||||||||||||||||||||||||||||| | |||||||||||
Sbjct  1091  TTGGTCTGCTGTGGAGTGGTGGTGCGACCAAAAA-AGTCAAACGAAAT  1045


>ref|XM_003405154.1| PREDICTED: Loxodonta africana methionyl-tRNA synthetase (MARS), 
mRNA
Length=2703

 Score = 73.1 bits (39),  Expect = 8e-11
 Identities = 41/42 (97%), Gaps = 0/42 (0%)
 Strand=Plus/Minus

Query  3     GGTCTGCTGTGGAGTGGTGGTGCGACCAAAAATATCAAACGA  44
             |||||||||| |||||||||||||||||||||||||||||||
Sbjct  1089  GGTCTGCTGTTGAGTGGTGGTGCGACCAAAAATATCAAACGA  1048


Lambda     K      H
    1.33    0.621     1.12 

Gapped
Lambda     K      H
    1.28    0.460    0.850 

Effective search space used: 874911716973
 
    reads 
 chr12 chr12 57856410 57856486 76M                                    CCCCCAAAACAGAGACCAAAAAGATTAGGTGGGCCTGATTTAAACTACCCCAGAATAACACCAGCTTTCACCTTTG
chr12 chr12 57856413 57856489 76M                                       CCAAAACAGAGACCAAAAAGATTAGGCGGGCCTGATTTAAACTACCCCAGAATAACACCAGCTTTAACCTTTGTGC
chr12 chr12 57856416 57856492 76M                                          AAACAGAGACCAAAAAGATTAGGTGGGCCTGATTTAAACTACCCCAGAATAACACCAGCTTTCACCTTTGTGCCCA
chr12 chr12 57856420 57856496 76M                                              AGAGACCAAAAAGATTAGGTGGGCCTGATTTAAACTACCCCAGAATAACACCAGCTTTCACCTTTGTGCCCAACAG
chr12 chr12 57856425 57856501 76M                                                   CCAAAAAGATTAGGTGGGCCTGATTTAAACTACCCCAGAATAACACCAGCTTTCACCTTTGTGCCCAACAGACCCA
chr12 chr12 57856428 57856504 76M                                                      AAAAGATTAGGTGGGCCTGATTTAAACTACCCCAGAATAACACCAGCTTTCACCTTTGTGCCCAACAGACCCAGAA
chr12 chr12 57856433 57856509 76M                                                           ATTAGGTGGGCCTGATTTAAACTACCCCAGAATAACACCAGCTTTCACCTTTGTGCCCAACAGACCCAGAAGACTC
chr12 chr12 57856436 57856512 76M                                                              AGGTGGGCCTGATTTAAACTACCCCAGAATAACACCAGCTTTCACCTTTGTGCCCAACAGACCCAGAAGACTCCCC
chr12 chr12 57856440 57856516 76M                                                                  GGGCCTGATTTAAACTACCCCAGAATAACACCAGCTTTCACCTTTGTGCCCAACAGACCCAGAAGCCCCCCCTCAG
chr12 chr12 57856443 57856519 76M                                                                     CCTGATTTAAACTACCCCAGAATAACACCAGCTTTCACCTTTGTGCCCAACAGACCCAGAAGACTCCCCTCAGGAA
chr12 chr12 57856446 57856522 76M                                                                        GATTTAAACTACCCCAGAATAACACCAGCTTTCACATTTGTGCCCAACAGACCCAGAAGACTCCCCTCAGGAACCT
chr12 chr12 57856449 57856525 76M                                                                           TTAAACTACCCCAGAATAACACCAGCTTTCACCTTTGTGCCCAACAGACCCAGAAGACTCCCCTCAGGAACCTTAC
chr12 chr12 57856453 57856529 76M                                                                               ACTACCCCAGAATAACACCAGCTTTCACCTTTGTGCCCAACAGACCCAGAAGACTCCCCTCAGGAACCTTACCTCC
chr12 chr12 57856456 57856532 76M                                                                                  ACCCCAGAATAACACCAGCTTTCACCTTTGTGCCCAACAGACCCAGAAGACTCCCCTCAGGAACCTTACCTCCTCC
chr12 chr12 57856459 57856535 76M                                                                                     CCAGAATAACACCAGCTTTCACCTTTGTGCCCAACAGACCCAGAAGACTCCCCTCAGGAACCTTACCTCCTCCTCC
chr12 chr12 57856463 57856539 76M                                                                                         AATAACACCAGCTTTCACCTTTGTGCCCAACAGACCCAGAAGACTCCCCTCAGGAACCTTACCTCCTCCTCCACAC
chr12 chr12 57856466 57856542 76M                                                                                            AACACCAGCTTTCACCTTTGTGCCCAACAGACCCAGAAGACTCCCCTCAGGAACCTTACCTCCTCCTCCACACTGT
chr12 chr12 57856469 57856545 76M                                                                                               ACCAGCTTTCACCTTTGTGCCCAACAGACCCAGAAGACTCCCCTCAGGAACCTTACCTCCTCCTCCACACTGTTTT
chr12 chr12 57856474 57856550 76M                                                                                                    CTTTCACCTTTGTGCCCAACAGACCCAAAAGACTCCCCTCAGGAACCTTACCTCCTCCTCCACACTGTTTTCTGTC
chr12 chr12 57856478 57856554 76M                                                                                                        CACCTTTGTGCCCAACAGACCCAGAAGACTCCCCTCAGGAACCTTACCTCCTCCTCCACACTGTTTTCTGTCTAAG
chr12 chr12 57856481 57856557 76M                                                                                                           CTTTGTGCCCAACAGACCCAGAAGACTCCCCTCAGGAACCTTACCTCCTCCTCCACACTGTTTTCTGTCTAAGAGG
chr12 chr12 57856484 57856560 76M                                                                                                              TGTGCCCAACAGACCCAGAAGACTCCCCTCAGGAACCTTACCTCCTCCTCCACACTGTTTTCTGTCTAAGAGGGCA
chr12 chr12 57856489 57856565 76M                                                                                                                   CCAACAGACCCAGAAGACTCCCCTCAGGAACCTTACCTCCTCCTCCACACTGTTTTCTGTCTAAGAGGGCAGATGG
chr12 chr12 57856492 57856568 76M                                                                                                                      ACAGACCCAGAAGACTCCCCTCAGGAACCTTACCTCCTCCTCCACACTGTTTTCTGTCTAAGAGGGCAGATGGCTT
chr12 chr12 57856495 57856571 76M                                                                                                                         GACCCAGAAGACTCCCCTCAGGAACCTTACCTCCTCCTCCACACTGTTTTCTGTCTAAGAGGGCAGATGGCTTGTG
chr12 chr12 57856499 57856575 76M                                                                                                                             CAGAAGACTCCCCTCAGGAACCTTACCTCCTCCTCCACACTGTTTTCTGTCTAAGAGGGCAGATGGCTTGTGCTCC
chr12 chr12 57856502 57856578 76M                                                                                                                                AAGACTCCCCTCAGGAACCTTACCTCCTCCTCCACACTGTTTTCTGTCTAAGAGGGCAGATGGCTTGTGCTCCCTC
chr12 chr12 57856505 57856581 76M                                                                                                                                   ACTCCCCTCAGGAACCTTACCTCCTCCTCCACACTGTTTTCTGTCTAAGAGGGCAGATGGCTTGTGCTCCCCCCTG
chr12 chr12 57856510 57856586 76M                                                                                                                                        CCTCAGGAACCTTACCTCCTCCTCCACACTGTTTTCTGTCTAAGAGGGCAGATGGCTTGTGCTCCCTCCTGGCCCC
chr12 chr12 57856515 57856591 76M                                                                                                                                             GGAACCTTACCTCCTCCTCCACACTGTTTTCTGTCTAAGAGGGCAGATGGCTTGTGCTCCCTCCTGGCCCCACCTG
chr12 chr12 57856520 57856596 76M                                                                                                                                                  CTTACCTCCTCCTCCACACTGTTTTCTGTCTAAGAGGGCAGATGGCTTGTGCTCCCTCCTGGCCCCACCTGGCTCT
chr12 chr12 57856523 57856599 76M                                                                                                                                                     ACCTCCTCCTCCACACTGTTTTCTGTCTAAGAGGGCAGATGGCTTGTGCTCCCTCCTGGCCCCACCTGGCTCTGTG
chr12 chr12 57856526 57856602 76M                                                                                                                                                        TCCTCCTCCACACTGTTTTCTGTCTAAGAGGGCAGATGGCTTGTGCTCCCTCCTGGCCCCCCCTGGCTCTGTGTCG
chr12 chr12 57856530 57856606 76M                                                                                                                                                            CCTCCACACTGTTTTCTGTCTAAGAGGGCAGATGGCTTGTGCTCCCTCCTGGCCCCACCTGGCTCTGTGTCGGGAC
chr12 chr12 57856533 57856609 76M                                                                                                                                                               CCACACTGTTTTCTGTCTAAGAGGGCAGATGGCTTGTGCTCCCTCCTGGCCCCACCTGGCTCTGTGTCGGGACTGT
chr12 chr12 57856536 57856612 76M                                                                                                                                                                  CACTGTTTTCTGTCTAAGAGGGCAGATGGCTTGTGCTCCCTCCTGGCCCCACCTGGCTCTGTGTCGGGACTGTGGC
chr12 chr12 57856539 57856615 76M                                                                                                                                                                     TGTTTTCTGTCTAAGAGGGCAGATGGCTTGTGCTCCCTCCTGGCCCCACCTGGCTCTGTGTCGGGACTGTGGCTAG
chr12 chr12 57856542 57856618 76M                                                                                                                                                                        TTTCTGTCTAAGAGGGCAGATGGCTTGTGCTCCCTCCTGGCCCCACCTGGCTCTGTGTCGGGACTGTGGCTAGGGC
chr12 chr12 57856545 57856621 76M                                                                                                                                                                           CTGTCTAAGAGGGCAGATGGCTTGTGCTCCCTCCTGGCCCCACCTGGCTCTGTGTCGGGACTGTGGCTAGGGCTCC
chr12 chr12 57856550 57856626 76M                                                                                                                                                                                TAAGAGGGCAGATGGCTTGTGCTCCCTCCTGGCCCCACCTGGCTCTGTGTCGGGACTGTGGCTAGGGCTCCCTCTG
chr12 chr12 57856553 57856629 76M                                                                                                                                                                                   GAGGGCAGATGGCTTGTGCTCCCTCCTGGCCCCACCTGGCTCTGTGTCGGGACTGTGGCTAGGGCTCCCTCTGCTG
chr12 chr12 57856556 57856632 76M                                                                                                                                                                                      GGCAGATGGCTTGTGCTCCCTCCTGGCCCCACCTGGCTCTGTGTCGGGACTGTGGCTAGGGCTCCCTCTGCTGGAC
chr12 chr12 57856561 57856637 76M                                                                                                                                                                                           ATGGCTTGTGCTCCCTCCTGGCCCCACCTGGCTCTGTGTCGGGACTGTGGCTAGGGCTCCCTCTGCTGGACACTGC
chr12 chr12 57856564 57856640 76M                                                                                                                                                                                              GCTTGTGCTCCCTCCTGGCCCCACCTGGCTCTGTGTCGGGACTGTGGCTAGGGCTCCCTCTGCTGGACACTGCAGA
chr12 chr12 57856568 57856644 76M                                                                                                                                                                                                  GTGCTCCCTCCTGGCCCCACCTGGCTCTGTGTCGGGACTGTGGCTAGGGCTCCCTCTGCTGGACACTGCAGAGATT
chr12 chr12 57856572 57856648 76M                                                                                                                                                                                                      TCCCTCCTGGCCCCACCTGGCTCTGTGTCGGGACTGTGGCTAGGGCTCCCTCTGCTGGACACTGCAGAGATTGTAC
chr12 chr12 57856575 57856651 76M                                                                                                                                                                                                         CTCCTGGCCCCACCTGGCTCTGTGTCGGGACTGTGGCTAGGGCTCCCTCTGCTGGACACCGCAGAGATTGAACACG
chr12 chr12 57856579 57856655 76M                                                                                                                                                                                                             TTGCCCCACCTGGCTCTGTGTCGGGACTGTGGCTAGGGCTCCCTCTGCTGGACACTGCAGAGATTGTACACGGGCT
chr12 chr12 57856582 57856658 76M                                                                                                                                                                                                                CCCCACCTGGCTCTGTGTCGGGACTGTGGCTAGGGCTCCCTCTGCTGGACACTGCAGAGATTGTACACGGGCTGTC
chr12 chr12 57856586 57856662 76M                                                                                                                                                                                                                    ACCTGGCTCTGTGTCGGGACTGTGGCTAGGGCTCCCTCTGCTGGACACTGCAGAGATTGTACACGGGCTGTCCCTG
chr12 chr12 57856587 57892401 72M57892406F4m                                                                                                                                                                                                          CCTGGCTCTTTGTCGGGACTGTGGCTAGTGCTCCCTCTGCTGGACACTGCAGAGATTGTACACGGGCTGTCC TTGG
chr12 chr12 57856597 57892391 62M57892406F14m                                                                                                                                                                                                                   CCACAGCAGACCAAGGACAGCCCGTGTACAATCTCTGCAGTGTCCAGCAGAGGGAGCCCTAG CCACAGTCCCGACA
chr12 chr12 57856598 57892390 61M57892406F15m                                                                                                                                                                                                                    GTCGGGACTGTGGCTAGGGCTCCCTCTGCTGGACACTGCAGAGATTGTACACGGGCTGTCC TTGGTCTGCTGTGGA
chr12 chr12 57856602 57892386 57M57892406F19m                                                                                                                                                                                                                        CCACTCCACAGCAGACCAAGGACAGCCCGTGTACAATCTCTGCAGTGTCCAGCAGAG GGAGCCCTAGCCACAGTCC
chr12 chr12 57856605 57892383 54M57892406F22m                                                                                                                                                                                                                           CTGTGGCTAGGGCTCCCTCTGCTGGACACTGCAGAGATTGTACACGGGCTGTCC TTGGTCTGCTGTGGAGTGGTGG
chr12 chr12 57856609 57892379 50M57892406F26m                                                                                                                                                                                                                               GGCTAGGGCTCCCTCTGCTGGACACTGCAGAGATTGTACACGGGCTGTCC TTGGTCTGCTGTGGAGTGGTGGTGCG
chr12 chr12 57856610 57892378 49M57892406F27m                                                                                                                                                                                                                                GCTAGGGCTCCCTCTGCTGGACACTGCAGAGATTGTACACGGGCTGTCC TTGGTCTGCTGTGGAGTGGTGGTGCGA
chr12 chr12 57856611 57892377 48M57892406F28m                                                                                                                                                                                                                                 CTAGGGCTCCCTCTGCTGGACACTGCAGAGATTGTACACGGGCTGTCC TTGGTCTGCTGTGGAGTGGTGGTGCGAC
chr12 chr12 57856614 57892374 45M57892406F31m                                                                                                                                                                                                                                    GGGCTCCCTCTGCTGGACACTGCAGAGATTGTACACGGGCTGTCC TTGGTCTGCTGTGGAGTGGTGGTGCGACCAA
chr12 chr12 57856615 57892373 44M57892406F32m                                                                                                                                                                                                                                     GGCTCCCTCTGCTGGACACTGCAGAGATTGTACACGGGCTGTCC TTGGTCTGCTGTGGAGTGGTGGTGCGACCAAA
chr12 chr12 57856621 57892367 38M57892406F38m                                                                                                                                                                                                                                           CTCTGCTGGACACTGCAGAGATTGTACACGGGCTGTCC TTGGTCTGCTGTGGAGTGGTGGTGCGACCAAAAATATC
chr12 chr12 57856633 57892355 26M57892406F50m                                                                                                                                                                                                                                                       CTGCAGAGATTGTACACGGGCTGTCC TTGGTCTGCTGTGGAGTGGTGGTGCGACCAAAAATATCAAACGAAATGTT
chr12 chr12 57856635 57892353 24M57892406F52m                                                                                                                                                                                                                                                         GCATAGATTGTACACGGGCTGTCC TTGGTCTGCTGTGGAGTGGTGGTGCGACCAAAAATATCAAACGAAATGTTAA
chr12 chr12 57856636 57892352 23M57892406F53m                                                                                                                                                                                                                                                          CAGAGATTGTACACGGGCTGTCC TTGGTCTGCTGTGGAGTGGTGGTGCGACCAAAAATATCAAACGAAATGTTAAA
chr12 chr12 57856636 57892352 23M57892406F53m                                                                                                                                                                                                                                                          AAGAGATTGTACACGGGCTGTCC TTGGTCTGCTGTGGAGTGGTGGTGCGACCAAAAATATCAAACGAAATGTTAAA
chr12 chr12 57856636 57892352 23M57892406F53m                                                                                                                                                                                                                                                          TTTAACATTTCGTTTGATATTTT TGGTCGCACCACCACTCCACAGCAGACCAAGGACAGCCCGTGTACAATCTCTT
chr12 chr12 57856640 57892348 19M57892406F57m                                                                                                                                                                                                                                                              GATTGTACACGGGCTGTCC TTGGTCTGCTGTGGAGTGGTGGTGCGACCAAAAATATCAAACGAAATGTTAAACCAG
chr12 chr12 57856640 57892348 19M57892406F57m                                                                                                                                                                                                                                                              GATTGTACACGGGCTGTCC TTGGTCTGCTGTGGAGTGGTGGTGCGACCAAAAATATCAAACGAAATGTTAAACCAG
chr12 chr12 57856640 57892348 19M57892406F57m                                                                                                                                                                                                                                                              GATTGTACACGGGCTGTCC TTGGTCTGCTGTGGAGTGGTGGTGCGACCAAAAATATCAAACGAAATGTTAAACCAG
chr12 chr12 57856642 57892346 17M57892406F59m                                                                                                                                                                                                                                                                TTGTACACGGTCTGTCC TTGGTCTGCTGTGGAGTGGTGGTGCGACCAAAAATATCAAACGAAATGTTAAACCAGCG
chr12 chr12 57856642 57892346 17M57892406F59m                                                                                                                                                                                                                                                                TTGTACACGGGCTGTCC TTGGTCTGCTGTGGAGTGGTGGTGCGACCAAAAATATCAAACGAAATGTTAAACCAGCG
chr12 chr12 57856645 57892343 14M57892406F62m                                                                                                                                                                                                                                                                   TACACGGGCTGTCC TTGGTCTGCTGTGGAGTGGTGGTGCGACCAAAAATATCAAACGAAATGTTAAACCAGCGGTA
chr12 chr12 57856650 57892338 9M57892406F67m                                                                                                                                                                                                                                                                         ACATCTACC GCTGGTTTAACATTTCGTTTGATATTTTTGGTCGCACCACCACTCCACAGCAGACCAAGGACAGCCC
chr12 chr12 57856650 57892338 9M57892406F67m                                                                                                                                                                                                                                                                         ACATCTACC GCTGGTTTAACATTTCGTTTGATATTTTTGGTCGCACCACCACTCCACAGCAGACCAAGGACAGCCA
chr12 chr12 57856653 57892335 6M57892406F70m                                                                                                                                                                                                                                                                            CTGTCC TTGGTCTGCTGTGGAGTGGTGGTGCGACCAAAAATATCAAACGAAATGTTAAACCAGCGGTAGATGTCAG
chr12 chr12 57892413 57892337 76m                                                                                                                                                                                                                                                                                      AAACTTACTTGGTCTGCTGTGGAGTGGTGGTGCGACCAAAAATATCAAACGAAATGTTAAACCAGCGGTAGATGTC
chr12 chr12 57892409 57892333 76m                                                                                                                                                                                                                                                                                          GTCCTTGGTCTGCTGTGGAGTGGTGGTGCGACCAAAAATATCAAACGAAATGTTAAACCAGCGGTAGATGTCAGCA
chr12 chr12 57892406 57892330 76m                                                                                                                                                                                                                                                                                             CTTGGTCTGCTGTGGAGTGGTAGTGCGACCAAAAATATCAAACGAAATGTTAAACCAGCGGTAGATGTCAGCATGG
chr12 chr12 57892403 57892327 76m                                                                                                                                                                                                                                                                                                GGTCTGCTGTGGAGTGGTGGTGCGACCAAAAATATCAAACGAAATGTTAAACCAGCGGTAGATGTCAGCATGGATG
chr12 chr12 57892400 57892324 76m                                                                                                                                                                                                                                                                                                   CTGCTGTGGAGTGGTGGTGCGACCAAAAATATCAAACGAAATGTTAAACCAGCGGTAGATGTCAGCATGGATGATG
chr12 chr12 57892397 57892321 76m                                                                                                                                                                                                                                                                                                      CTGTGGAGTGGTGGTGCGACCAAAAATATCAAACGAAATGTTAAACCAGCGGTAGATGTCAGCATGGATGATGTGG
chr12 chr12 57892394 57892318 76m                                                                                                                                                                                                                                                                                                         TGGAGTGGTGGTGCGACCAAAAATATCAAACGAAATGTTAAACCAGCGGTAGATGTCAGCATGGATGATGTGGTAC
chr12 chr12 57892391 57892315 76m                                                                                                                                                                                                                                                                                                            AGTGGTGGTGCGACCAAAAATATCAAACGAAATGTTAAACCAGCGGTAGATGTCAGCATGGATGATGTGGTACTTG
chr12 chr12 57892388 57892312 76m                                                                                                                                                                                                                                                                                                               GGTGGTGCGCCCAAAAATATCAATCGAAATGTTAAACCAGCGGTAGATGTCAGCATGGATGATGTGGTACTTGTCG
chr12 chr12 57892385 57892309 76m                                                                                                                                                                                                                                                                                                                  TGTGCGACCAAAAATATCAAACGAAATGTTAAACCAGCGGTAGATGTCAGCATGGATGATGTGGTACTTGTCGCAG
chr12 chr12 57892382 57892306 76m                                                                                                                                                                                                                                                                                                                     GCGACCAAAAATATCAAACGAAATGTTAAACCAGCGGTAGATGTCAGCATGGATGATGTGGTACTTGTCGCAGATC
chr12 chr12 57892379 57892302 7m1d69m                                                                                                                                                                                                                                                                                                                    ACCAAAADTATCAAACGAAATGTTAAACCAGCGTTAGATGTCAGCATGGATGATGCGGTACTTGTCGCAGATCTCCT
chr12 chr12 57892376 57892300 76m                                                                                                                                                                                                                                                                                                                           AAAAATATCAAACGAAATGTTAAACCAGCGGTAGATGTCAGCATGGATGATGTGGTACTTGTCGCAGATCTCCTGG
chr12 chr12 57892373 57892297 76m                                                                                                                                                                                                                                                                                                                              AATATCAAACGAAATGTTAAACCAGCGGTAGATGTCAGCATGGATGATGTGGTACTTGTCGCAGATCTCCTGGGGG
chr12 chr12 57892370 57892294 76m                                                                                                                                                                                                                                                                                                                                 ATCAAACGAAATGTTAAACCAGCGGTAGATGTCAGCATGGATGATGTGGTACTTGTCGCAGATCTCCTGGGGGGTT
chr12 chr12 57892367 57892291 76m                                                                                                                                                                                                                                                                                                                                    AAACGAAATGTTAAACCAGCGGTAGATGTCAGCATGGATGATGTGGTACTTGTCGCAGATCTCCTGGGGGGTTAGT
chr12 chr12 57892364 57892288 76m                                                                                                                                                                                                                                                                                                                                       CGAAATGTTAAACCAGCGGTAGATGTCAGCATGGATGATGTGGTACTTGTCGCAGATCTCCTGGGGGGTTAGTCCC
chr12 chr12 57892361 57892285 76m                                                                                                                                                                                                                                                                                                                                          AATGTTAAACCAGCGGTAGATGTCAGCATGGATGATGTGGTACTTGTCGCAGATCTCCTGGGGGGTTAGTCCCTCC
chr12 chr12 57892358 57892282 76m                                                                                                                                                                                                                                                                                                                                             GTTAAACCAGCGGTAGATGTCAGCATGGATGATGTGGTACTTGTCGCAGATCTCCTGGGGGGTTAGTCCCTCCTCC
chr12 chr12 57892355 57892279 76m                                                                                                                                                                                                                                                                                                                                                AAACCAGCGGTAGATGTCAGCATGGATGATGTGGTACTTGTCGCAGATCTCCTGGGGGGTTAGTCCCTCCTCCAGA
chr12 chr12 57892352 57892276 76m                                                                                                                                                                                                                                                                                                                                                   CCAGCGGTAGATGTCAGCATGGATGATGTGGTACTTGTCGCAGATCTCCTGGGGGGTTAGTCCCTCCTCCAGAGCC
chr12 chr12 57892349 57892273 76m                                                                                                                                                                                                                                                                                                                                                      GCGGTAGATGGCAGCATGGATGATGTGGTACTTGTCGCAGATCTCCTGGGGGGTTAGTCCCTCCTCCAGAGCCTTG
chr12 chr12 57892346 57892270 76m                                                                                                                                                                                                                                                                                                                                                         GTAGATGTCAGCATGGATGATGTGGTACTTGTCGCAGATCTCCTGGGGGGTTAGTCCCTCCTCCAGAGCCTTGGTC
chr12 chr12 57892343 57892267 76m                                                                                                                                                                                                                                                                                                                                                            GATGTCAGCATGGATGGTGTGGTACTTGTCGCAGCTCTCCTGGGGGGTTAGTCCCTCCTCCAGAGCCTTGGTCTCT
chr12 chr12 57892340 57892264 76m                                                                                                                                                                                                                                                                                                                                                               GTCTGCATGGATGATGTGGTACTTGTCGCAGATCTCGTGGGGGGTTAGTCCCTCCTCCAGAGCCTTGGTCTCTGTT
chr12 chr12 57892337 57892261 76m                                                                                                                                                                                                                                                                                                                                                                  AGCATGGATGATGTGGTACTTGTCTCAGATCTCCTGGGGGGTTAGTCCCTCCTCCAGAGCCTTGGTCTCTGTTGCT
chr12 chr12 57892334 57892258 76m                                                                                                                                                                                                                                                                                                                                                                     GTGGATGATGTGGTACTTGTCGCAGATCTCCTGGGGGGTTAGTCCCTCCTCCAGAGCCTTGGTCTCTGTTGCTGTA
chr12 chr12 57892331 57892255 76m                                                                                                                                                                                                                                                                                                                                                                        GATGATGTGGTACTTGTCGCAGATCTCCTGGGGGGTTAGTCCCTCCTCCAGAGCCTTGGTCTCTGTTGCTGTACCA
chr12 chr12 57892328 57892252 76m                                                                                                                                                                                                                                                                                                                                                                           GATGTGGTACTTGTCGCAGATCTCCTGGGGGGTTAGTCCCTCCTCCAGAGCCTTGGTCTCTGTTGCTGTACCATAC
chr12 chr12 57892325 57892249 76m                                                                                                                                                                                                                                                                                                                                                                              GTGGTACTTGTCGCAGATCTCCTGGGGGGTTAGTCCCTCCTCCAGAGCCTTGGTCTCTGTTGCTGTACCATACTCA
chr12 chr12 57892322 57892246 76m                                                                                                                                                                                                                                                                                                                                                                                 GTACTTGTCGCAGATCTCCTGGGGGGTTAGTCCCTCCTCCAGAGCCTTGGTCTCTGTTGCTGTACCATACTCATCT
chr12 chr12 57892319 57892243 76m                                                                                                                                                                                                                                                                                                                                                                                    CTTGTCGCAGATCTCCTGGGGGGTTAGTCCCTCCTCCAGAGCCTTGGTCTCTGTTGCTGTACCATACTCATCTGTC
chr12 chr12 57892316 57892240 76m                                                                                                                                                                                                                                                                                                                                                                                       GTCGCAGATCTCCTGGGGGGTTAGTCCCTCCTCCAGAGCCTTGGTCTCTGTTGCTGTACCATACTCATCTGTCCCA
chr12 chr12 57892313 57892237 76m                                                                                                                                                                                                                                                                                                                                                                                          GCAGATCTCCTGAGGGGTTAGTCCCTCCTCCAGAGCCTTGGTCTCTGTTGCTGTACCATACTCATCTGTCACACAC
chr12 chr12 57892310 57892234 76m                                                                                                                                                                                                                                                                                                                                                                                             GATCTCCTGGGGGGTTAGTCCCTCCTCCAGAGCCTTGGTCTCTGTTGCTGTACCATACTCATCTGTCCCACACAGA
chr12 chr12 57892307 57892231 76m                                                                                                                                                                                                                                                                                                                                                                                                CTCCTGGGGGGTTAGTCCCTCCTCCAGAGCCTTGGTCTCTGTTGCTGTACCATACTCATCTGTCCCACACAGATAG
chr12 chr12 57892304 57892227 45m1d31m                                                                                                                                                                                                                                                                                                                                                                                              CTGGGGGGTTAGTCCCTCCTCCAGAGCCTTGGTCTCTGTTGCTGTDCCATACTCATCTGTCCCACACAGATAGAGGG
chr12 chr12 57892301 57892224 73m1d3m                                                                                                                                                                                                                                                                                                                                                                                                  GGGGGTTAGTCCCTCCTCCAGAGCCTTGGTCTCTGTTGCTGTACCATACTCATCTGTCCCACACAGATAGAGGDTGT
chr12 chr12 57892298 57892222 76m                                                                                                                                                                                                                                                                                                                                                                                                         GGTTAGTCCCTCCTCCAGAGCCTTGGTCTCTGTTGCTGTACCATACTCATCTGTCCCCCACAGATAGAGGGTGTTC
chr12 chr12 57892295 57892219 76m                                                                                                                                                                                                                                                                                                                                                                                                            TAGTCCCTCCTCCAGAGCCTTGGTCTCTGTTGCTGTACCATTCTCATCTGGCCCACACAGATAGAGGGTGTTCCAC
chr12 chr12 57892292 57892216 76m                                                                                                                                                                                                                                                                                                                                                                                                               TCCCTCCTCCAGAGCGTTGGTCTCTGTTGCTGTACCATACTCATCTGTCCCACACAGATAGAGGGTGTTCCACTGG
chr12 chr12 57892289 57892213 76m                                                                                                                                                                                                                                                                                                                                                                                                                  CTCCTCCAGAGCCTTGGTCTCTGTTGCTGTACCATACTCATCTGTCCCACACAGATAGAGGGTGTTCCACTGGCGG
chr12 chr12 57892286 57892210 76m                                                                                                                                                                                                                                                                                                                                                                                                                     CTCCAGAGCCTTGGTCTCTGTTGCTGTACCATACTCATCTGTCCCACACAGATAGAGGGTGTTCCACTGGCGGAGG
chr12 chr12 57892283 57892207 76m                                                                                                                                                                                                                                                                                                                                                                                                                        CAGAGCCTTGGTCTCTGTTGCTGTACCATACTCATCTGTCCCACACAGATAGAGGGTGTTCCACTGGCGGAGGCGA
chr12 chr12 57892280 57892204 76m                                                                                                                                                                                                                                                                                                                                                                                                                           AGCCTTGGTCTCTGTTGCTGTACCATACTCATCTGTCCCACACAGATAGAGGGTGTTCCACTGGCGGAGGCGAGAG
chr12 chr12 57892277 57892054 49m1d26m146n1m                                                                                                                                                                                                                                                                                                                                                                                                                   CTTGGTCTCTGTTGCTGTACCATACTCATCTGTCCCACACAGATAGAGGDTGTTCCACTGGCGGAGGCGAGAGTAC||||||||||||||||||||||||||||||||||||||||||||||||||||||||||||||||||||||||||||||||||||||||||||||
chr12 chr12 57892274 57884423 73m7775n3m                                                                                                                                                                                                                                                                                                                                                                                                                          GGTCTCTGTTGCTGTACCATACTCATCTGTCCCACACAGATAGAGGGTGTTCCACTGGCGGAGGCGAGAGTAC||||||||||||||||||||||||||||||||||||||||||||||||||||||||||||||||||||||||||||||||||||||||||||||
chr12 chr12 57892271 57892049 70m146n6m                                                                                                                                                                                                                                                                                                                                                                                                                              CTCTGTTGCTGTACCATACTCATCTGTCCCACACAGATAGAGGGTGTTCCACTGGCGGAGGCGAGAGCAC||||||||||||||||||||||||||||||||||||||||||||||||||||||||||||||||||||||||||||||||||||||||||||||
chr12 chr12 57892268 57892046 67m146n9m                                                                                                                                                                                                                                                                                                                                                                                                                                 TGTTGCTGTACCATACTCATCTGTCCCACACAGATAGAGGGTGTTCCACTGGCGGAGGCGAGAGTAC|||||||||||||||||||||||||||||||||||||||||||||||||||||||||||||||||||||||||||||||||||||||||||||| 
    46 pairs 
 -6:4
9:26
6:39
77:26
-1943:86
3345:26
3345:26
5154:7
5154:19
5208:36
5192:-1867
5229:-1869
-7095:49
-7078:96
-7189:-1696
-7167:-1889
5144:-5614
5130:-5634
5139:-5633
5138:-5637
5130:-5648
5136:-5656
5170:-5623
5146:-5654
5129:-5671
5130:-5671
5146:-5656
5130:-5674
5152:-5659
5142:-5671
5144:-5672
5146:-5672
5166:-5666
5157:-5676
5193:-5666
5205:-5656
5218:-5647
5218:-5647
5258:-5611
5258:-5611
5197:-5675
5205:-5677
5259:-5639
5229:-5671
5229:-5676
5248:-5671 
     TCGA-02-2485-01A chr12-chr12 57898081 59195041 ff 
 
427 28 418 7 56 11178
CATGCCCTGTGGTGCAGTCGAGCCAGCACCTGTTTCTGGACCTGCCTAAG GGCCTGAATTTTCTGCTCCTGAATTCAAAGTAAGTTTCCATAACCCTGAT
99999999999999999999999999999999999999999999999999 99999999999999999999999999999999999999999999999999
MARS exon11(57898006-57898081) ENSG00000258231 exon3(59195040-59195069)
 
 blast search - human genome  
 left 
 >ref|NT_029419.12| Homo sapiens chromosome 12 genomic contig, GRCh37.p9 Primary 
Assembly
 gb|GL000107.1| Homo sapiens chromosome 12 genomic contig, GRCh37 reference primary 
assembly
Length=71516776

 Score = 93.5 bits (50),  Expect = 5e-17
 Identities = 50/50 (100%), Gaps = 0/50 (0%)
 Strand=Plus/Plus

Query  1         CATGCCCTGTGGTGCAGTCGAGCCAGCACCTGTTTCTGGACCTGCCTAAG  50
                 ||||||||||||||||||||||||||||||||||||||||||||||||||
Sbjct  20041339  CATGCCCTGTGGTGCAGTCGAGCCAGCACCTGTTTCTGGACCTGCCTAAG  20041388


>ref|NW_001838060.2| Homo sapiens chromosome 12 genomic contig, alternate assembly 
HuRef SCAF_1103279188147, whole genome shotgun sequence
 gb|DS486061.1| Homo sapiens SCAF_1103279188147 genomic scaffold, whole genome 
shotgun sequence
Length=18942049

 Score = 93.5 bits (50),  Expect = 5e-17
 Identities = 50/50 (100%), Gaps = 0/50 (0%)
 Strand=Plus/Minus

Query  1         CATGCCCTGTGGTGCAGTCGAGCCAGCACCTGTTTCTGGACCTGCCTAAG  50
                 ||||||||||||||||||||||||||||||||||||||||||||||||||
Sbjct  18630467  CATGCCCTGTGGTGCAGTCGAGCCAGCACCTGTTTCTGGACCTGCCTAAG  18630418


>ref|NC_000012.11| Homo sapiens chromosome 12, GRCh37.p9 Primary Assembly
 gb|CM000674.1| Homo sapiens chromosome 12, GRCh37 primary reference assembly
Length=133851895

 Score = 93.5 bits (50),  Expect = 5e-17
 Identities = 50/50 (100%), Gaps = 0/50 (0%)
 Strand=Plus/Plus

Query  1         CATGCCCTGTGGTGCAGTCGAGCCAGCACCTGTTTCTGGACCTGCCTAAG  50
                 ||||||||||||||||||||||||||||||||||||||||||||||||||
Sbjct  57898033  CATGCCCTGTGGTGCAGTCGAGCCAGCACCTGTTTCTGGACCTGCCTAAG  57898082


>ref|AC_000144.1| Homo sapiens chromosome 12, alternate assembly HuRef, whole genome 
shotgun sequence
 gb|CM000473.1| Homo sapiens chromosome 12, whole genome shotgun sequence
Length=130617856

 Score = 93.5 bits (50),  Expect = 5e-17
 Identities = 50/50 (100%), Gaps = 0/50 (0%)
 Strand=Plus/Plus

Query  1         CATGCCCTGTGGTGCAGTCGAGCCAGCACCTGTTTCTGGACCTGCCTAAG  50
                 ||||||||||||||||||||||||||||||||||||||||||||||||||
Sbjct  54936110  CATGCCCTGTGGTGCAGTCGAGCCAGCACCTGTTTCTGGACCTGCCTAAG  54936159


>gb|CM000263.1| Homo sapiens chromosome 12, whole genome shotgun sequence
Length=133516800

 Score = 93.5 bits (50),  Expect = 5e-17
 Identities = 50/50 (100%), Gaps = 0/50 (0%)
 Strand=Plus/Plus

Query  1         CATGCCCTGTGGTGCAGTCGAGCCAGCACCTGTTTCTGGACCTGCCTAAG  50
                 ||||||||||||||||||||||||||||||||||||||||||||||||||
Sbjct  57553869  CATGCCCTGTGGTGCAGTCGAGCCAGCACCTGTTTCTGGACCTGCCTAAG  57553918


Lambda     K      H
    1.33    0.621     1.12 

Gapped
Lambda     K      H
    1.28    0.460    0.850 

Effective search space used: 681780354108
 
 right 
 >ref|NT_029419.12| Homo sapiens chromosome 12 genomic contig, GRCh37.p9 Primary 
Assembly
 gb|GL000107.1| Homo sapiens chromosome 12 genomic contig, GRCh37 reference primary 
assembly
Length=71516776

 Score = 93.5 bits (50),  Expect = 5e-17
 Identities = 50/50 (100%), Gaps = 0/50 (0%)
 Strand=Plus/Plus

Query  1         GGCCTGAATTTTCTGCTCCTGAATTCAAAGTAAGTTTCCATAACCCTGAT  50
                 ||||||||||||||||||||||||||||||||||||||||||||||||||
Sbjct  21338348  GGCCTGAATTTTCTGCTCCTGAATTCAAAGTAAGTTTCCATAACCCTGAT  21338397


>ref|NC_000012.11| Homo sapiens chromosome 12, GRCh37.p9 Primary Assembly
 gb|CM000674.1| Homo sapiens chromosome 12, GRCh37 primary reference assembly
Length=133851895

 Score = 93.5 bits (50),  Expect = 5e-17
 Identities = 50/50 (100%), Gaps = 0/50 (0%)
 Strand=Plus/Plus

Query  1         GGCCTGAATTTTCTGCTCCTGAATTCAAAGTAAGTTTCCATAACCCTGAT  50
                 ||||||||||||||||||||||||||||||||||||||||||||||||||
Sbjct  59195042  GGCCTGAATTTTCTGCTCCTGAATTCAAAGTAAGTTTCCATAACCCTGAT  59195091


>gb|CM000263.1| Homo sapiens chromosome 12, whole genome shotgun sequence
Length=133516800

 Score = 93.5 bits (50),  Expect = 5e-17
 Identities = 50/50 (100%), Gaps = 0/50 (0%)
 Strand=Plus/Plus

Query  1         GGCCTGAATTTTCTGCTCCTGAATTCAAAGTAAGTTTCCATAACCCTGAT  50
                 ||||||||||||||||||||||||||||||||||||||||||||||||||
Sbjct  58856683  GGCCTGAATTTTCTGCTCCTGAATTCAAAGTAAGTTTCCATAACCCTGAT  58856732


>gb|GL583101.1| Homo sapiens unplaced genomic scaffold scaffold_121, whole genome 
shotgun sequence
Length=7049662

 Score = 89.8 bits (48),  Expect = 7e-16
 Identities = 48/48 (100%), Gaps = 0/48 (0%)
 Strand=Plus/Minus

Query  1        GGCCTGAATTTTCTGCTCCTGAATTCAAAGTAAGTTTCCATAACCCTG  48
                ||||||||||||||||||||||||||||||||||||||||||||||||
Sbjct  4693876  GGCCTGAATTTTCTGCTCCTGAATTCAAAGTAAGTTTCCATAACCCTG  4693829


>ref|NW_001838060.2| Homo sapiens chromosome 12 genomic contig, alternate assembly 
HuRef SCAF_1103279188147, whole genome shotgun sequence
 gb|DS486061.1| Homo sapiens SCAF_1103279188147 genomic scaffold, whole genome 
shotgun sequence
Length=18942049

 Score = 89.8 bits (48),  Expect = 7e-16
 Identities = 48/48 (100%), Gaps = 0/48 (0%)
 Strand=Plus/Minus

Query  1         GGCCTGAATTTTCTGCTCCTGAATTCAAAGTAAGTTTCCATAACCCTG  48
                 ||||||||||||||||||||||||||||||||||||||||||||||||
Sbjct  17322944  GGCCTGAATTTTCTGCTCCTGAATTCAAAGTAAGTTTCCATAACCCTG  17322897


>ref|AC_000144.1| Homo sapiens chromosome 12, alternate assembly HuRef, whole genome 
shotgun sequence
 gb|CM000473.1| Homo sapiens chromosome 12, whole genome shotgun sequence
Length=130617856

 Score = 89.8 bits (48),  Expect = 7e-16
 Identities = 48/48 (100%), Gaps = 0/48 (0%)
 Strand=Plus/Plus

Query  1         GGCCTGAATTTTCTGCTCCTGAATTCAAAGTAAGTTTCCATAACCCTG  48
                 ||||||||||||||||||||||||||||||||||||||||||||||||
Sbjct  56243633  GGCCTGAATTTTCTGCTCCTGAATTCAAAGTAAGTTTCCATAACCCTG  56243680


Lambda     K      H
    1.33    0.621     1.12 

Gapped
Lambda     K      H
    1.28    0.460    0.850 

Effective search space used: 681780354108
 
 blast search - nt  
 left 
 >ref|XM_002823424.2| PREDICTED: Pongo abelii methionyl-tRNA synthetase (MARS), mRNA
Length=2917

 Score = 93.5 bits (50),  Expect = 6e-17
 Identities = 50/50 (100%), Gaps = 0/50 (0%)
 Strand=Plus/Plus

Query  1     CATGCCCTGTGGTGCAGTCGAGCCAGCACCTGTTTCTGGACCTGCCTAAG  50
             ||||||||||||||||||||||||||||||||||||||||||||||||||
Sbjct  1457  CATGCCCTGTGGTGCAGTCGAGCCAGCACCTGTTTCTGGACCTGCCTAAG  1506


>ref|NM_004990.3| Homo sapiens methionyl-tRNA synthetase (MARS), mRNA
Length=2932

 Score = 93.5 bits (50),  Expect = 6e-17
 Identities = 50/50 (100%), Gaps = 0/50 (0%)
 Strand=Plus/Plus

Query  1     CATGCCCTGTGGTGCAGTCGAGCCAGCACCTGTTTCTGGACCTGCCTAAG  50
             ||||||||||||||||||||||||||||||||||||||||||||||||||
Sbjct  1457  CATGCCCTGTGGTGCAGTCGAGCCAGCACCTGTTTCTGGACCTGCCTAAG  1506


>dbj|AB590802.1| Synthetic construct DNA, clone: pFN21AB8542, Homo sapiens MARS 
gene for methionyl-tRNA synthetase, without stop codon, in 
Flexi system
Length=2717

 Score = 93.5 bits (50),  Expect = 6e-17
 Identities = 50/50 (100%), Gaps = 0/50 (0%)
 Strand=Plus/Plus

Query  1     CATGCCCTGTGGTGCAGTCGAGCCAGCACCTGTTTCTGGACCTGCCTAAG  50
             ||||||||||||||||||||||||||||||||||||||||||||||||||
Sbjct  1328  CATGCCCTGTGGTGCAGTCGAGCCAGCACCTGTTTCTGGACCTGCCTAAG  1377


>dbj|AK303349.1| Homo sapiens cDNA FLJ55046 complete cds, highly similar to Methionyl-tRNA 
synthetase (EC 6.1.1.10)
Length=1328

 Score = 93.5 bits (50),  Expect = 6e-17
 Identities = 50/50 (100%), Gaps = 0/50 (0%)
 Strand=Plus/Plus

Query  1     CATGCCCTGTGGTGCAGTCGAGCCAGCACCTGTTTCTGGACCTGCCTAAG  50
             ||||||||||||||||||||||||||||||||||||||||||||||||||
Sbjct  961   CATGCCCTGTGGTGCAGTCGAGCCAGCACCTGTTTCTGGACCTGCCTAAG  1010


>dbj|AK293945.1| Homo sapiens cDNA FLJ53309 complete cds, highly similar to Methionyl-tRNA 
synthetase (EC 6.1.1.10)
Length=2702

 Score = 93.5 bits (50),  Expect = 6e-17
 Identities = 50/50 (100%), Gaps = 0/50 (0%)
 Strand=Plus/Plus

Query  1     CATGCCCTGTGGTGCAGTCGAGCCAGCACCTGTTTCTGGACCTGCCTAAG  50
             ||||||||||||||||||||||||||||||||||||||||||||||||||
Sbjct  1268  CATGCCCTGTGGTGCAGTCGAGCCAGCACCTGTTTCTGGACCTGCCTAAG  1317


>dbj|AK290857.1| Homo sapiens cDNA FLJ76789 complete cds, highly similar to Homo 
sapiens methionine-tRNA synthetase (MARS), mRNA
Length=2780

 Score = 93.5 bits (50),  Expect = 6e-17
 Identities = 50/50 (100%), Gaps = 0/50 (0%)
 Strand=Plus/Plus

Query  1     CATGCCCTGTGGTGCAGTCGAGCCAGCACCTGTTTCTGGACCTGCCTAAG  50
             ||||||||||||||||||||||||||||||||||||||||||||||||||
Sbjct  1328  CATGCCCTGTGGTGCAGTCGAGCCAGCACCTGTTTCTGGACCTGCCTAAG  1377


>gb|BC065187.1| Homo sapiens methionine-tRNA synthetase, mRNA (cDNA clone IMAGE:5021529), 
partial cds
Length=2830

 Score = 93.5 bits (50),  Expect = 6e-17
 Identities = 50/50 (100%), Gaps = 0/50 (0%)
 Strand=Plus/Plus

Query  1     CATGCCCTGTGGTGCAGTCGAGCCAGCACCTGTTTCTGGACCTGCCTAAG  50
             ||||||||||||||||||||||||||||||||||||||||||||||||||
Sbjct  1354  CATGCCCTGTGGTGCAGTCGAGCCAGCACCTGTTTCTGGACCTGCCTAAG  1403


>dbj|AK122956.1| Homo sapiens cDNA FLJ16674 fis, clone THYMU3008136, highly  similar 
to Methionyl-tRNA synthetase (EC 6.1.1.10)
Length=3574

 Score = 93.5 bits (50),  Expect = 6e-17
 Identities = 50/50 (100%), Gaps = 0/50 (0%)
 Strand=Plus/Plus

Query  1     CATGCCCTGTGGTGCAGTCGAGCCAGCACCTGTTTCTGGACCTGCCTAAG  50
             ||||||||||||||||||||||||||||||||||||||||||||||||||
Sbjct  2005  CATGCCCTGTGGTGCAGTCGAGCCAGCACCTGTTTCTGGACCTGCCTAAG  2054


>gb|BT007338.1| Homo sapiens methionine-tRNA synthetase mRNA, complete cds
Length=2703

 Score = 93.5 bits (50),  Expect = 6e-17
 Identities = 50/50 (100%), Gaps = 0/50 (0%)
 Strand=Plus/Plus

Query  1     CATGCCCTGTGGTGCAGTCGAGCCAGCACCTGTTTCTGGACCTGCCTAAG  50
             ||||||||||||||||||||||||||||||||||||||||||||||||||
Sbjct  1319  CATGCCCTGTGGTGCAGTCGAGCCAGCACCTGTTTCTGGACCTGCCTAAG  1368


>dbj|AK222767.1| Homo sapiens mRNA for methionine-tRNA synthetase variant, clone: 
HEP00371
Length=2738

 Score = 93.5 bits (50),  Expect = 6e-17
 Identities = 50/50 (100%), Gaps = 0/50 (0%)
 Strand=Plus/Plus

Query  1     CATGCCCTGTGGTGCAGTCGAGCCAGCACCTGTTTCTGGACCTGCCTAAG  50
             ||||||||||||||||||||||||||||||||||||||||||||||||||
Sbjct  1339  CATGCCCTGTGGTGCAGTCGAGCCAGCACCTGTTTCTGGACCTGCCTAAG  1388


>gb|BC015011.1| Homo sapiens methionyl-tRNA synthetase, mRNA (cDNA clone IMAGE:4864644), 
partial cds
Length=2790

 Score = 93.5 bits (50),  Expect = 6e-17
 Identities = 50/50 (100%), Gaps = 0/50 (0%)
 Strand=Plus/Plus

Query  1     CATGCCCTGTGGTGCAGTCGAGCCAGCACCTGTTTCTGGACCTGCCTAAG  50
             ||||||||||||||||||||||||||||||||||||||||||||||||||
Sbjct  1317  CATGCCCTGTGGTGCAGTCGAGCCAGCACCTGTTTCTGGACCTGCCTAAG  1366


>gb|AC022506.38| Homo sapiens 12 BAC RP11-181L23 (Roswell Park Cancer Institute 
Human BAC Library) complete sequence
Length=172136

 Score = 93.5 bits (50),  Expect = 6e-17
 Identities = 50/50 (100%), Gaps = 0/50 (0%)
 Strand=Plus/Minus

Query  1       CATGCCCTGTGGTGCAGTCGAGCCAGCACCTGTTTCTGGACCTGCCTAAG  50
               ||||||||||||||||||||||||||||||||||||||||||||||||||
Sbjct  103836  CATGCCCTGTGGTGCAGTCGAGCCAGCACCTGTTTCTGGACCTGCCTAAG  103787


>emb|X94754.1| H.sapiens mRNA for yeast methionyl-tRNA synthetase homologue
Length=2779

 Score = 93.5 bits (50),  Expect = 6e-17
 Identities = 50/50 (100%), Gaps = 0/50 (0%)
 Strand=Plus/Plus

Query  1     CATGCCCTGTGGTGCAGTCGAGCCAGCACCTGTTTCTGGACCTGCCTAAG  50
             ||||||||||||||||||||||||||||||||||||||||||||||||||
Sbjct  1326  CATGCCCTGTGGTGCAGTCGAGCCAGCACCTGTTTCTGGACCTGCCTAAG  1375


>emb|Z49216.1| H.sapiens mitoxantrone-resistance associated mRNA
Length=1550

 Score = 93.5 bits (50),  Expect = 6e-17
 Identities = 50/50 (100%), Gaps = 0/50 (0%)
 Strand=Plus/Plus

Query  1    CATGCCCTGTGGTGCAGTCGAGCCAGCACCTGTTTCTGGACCTGCCTAAG  50
            ||||||||||||||||||||||||||||||||||||||||||||||||||
Sbjct  53   CATGCCCTGTGGTGCAGTCGAGCCAGCACCTGTTTCTGGACCTGCCTAAG  102


>gb|AY889906.1| Synthetic construct Homo sapiens clone FLH022775.01X methionine-tRNA 
synthetase (MARS) mRNA, complete cds
Length=2703

 Score = 93.5 bits (50),  Expect = 6e-17
 Identities = 50/50 (100%), Gaps = 0/50 (0%)
 Strand=Plus/Plus

Query  1     CATGCCCTGTGGTGCAGTCGAGCCAGCACCTGTTTCTGGACCTGCCTAAG  50
             ||||||||||||||||||||||||||||||||||||||||||||||||||
Sbjct  1319  CATGCCCTGTGGTGCAGTCGAGCCAGCACCTGTTTCTGGACCTGCCTAAG  1368


>gb|BC011849.2| Homo sapiens methionyl-tRNA synthetase, mRNA (cDNA clone MGC:20313 
IMAGE:4135154), complete cds
Length=2800

 Score = 93.5 bits (50),  Expect = 6e-17
 Identities = 50/50 (100%), Gaps = 0/50 (0%)
 Strand=Plus/Plus

Query  1     CATGCCCTGTGGTGCAGTCGAGCCAGCACCTGTTTCTGGACCTGCCTAAG  50
             ||||||||||||||||||||||||||||||||||||||||||||||||||
Sbjct  1325  CATGCCCTGTGGTGCAGTCGAGCCAGCACCTGTTTCTGGACCTGCCTAAG  1374


>gb|BC011639.2| Homo sapiens methionyl-tRNA synthetase, mRNA (cDNA clone MGC:12828 
IMAGE:4120680), complete cds
Length=2799

 Score = 93.5 bits (50),  Expect = 6e-17
 Identities = 50/50 (100%), Gaps = 0/50 (0%)
 Strand=Plus/Plus

Query  1     CATGCCCTGTGGTGCAGTCGAGCCAGCACCTGTTTCTGGACCTGCCTAAG  50
             ||||||||||||||||||||||||||||||||||||||||||||||||||
Sbjct  1327  CATGCCCTGTGGTGCAGTCGAGCCAGCACCTGTTTCTGGACCTGCCTAAG  1376


>gb|BC006328.2| Homo sapiens methionyl-tRNA synthetase, mRNA (cDNA clone MGC:12889 
IMAGE:4139330), complete cds
Length=2795

 Score = 93.5 bits (50),  Expect = 6e-17
 Identities = 50/50 (100%), Gaps = 0/50 (0%)
 Strand=Plus/Plus

Query  1     CATGCCCTGTGGTGCAGTCGAGCCAGCACCTGTTTCTGGACCTGCCTAAG  50
             ||||||||||||||||||||||||||||||||||||||||||||||||||
Sbjct  1325  CATGCCCTGTGGTGCAGTCGAGCCAGCACCTGTTTCTGGACCTGCCTAAG  1374


>gb|BC002384.2| Homo sapiens methionyl-tRNA synthetase, mRNA (cDNA clone MGC:8646 
IMAGE:2961615), complete cds
Length=2799

 Score = 93.5 bits (50),  Expect = 6e-17
 Identities = 50/50 (100%), Gaps = 0/50 (0%)
 Strand=Plus/Plus

Query  1     CATGCCCTGTGGTGCAGTCGAGCCAGCACCTGTTTCTGGACCTGCCTAAG  50
             ||||||||||||||||||||||||||||||||||||||||||||||||||
Sbjct  1325  CATGCCCTGTGGTGCAGTCGAGCCAGCACCTGTTTCTGGACCTGCCTAAG  1374


>dbj|D84224.1| Homo sapiens mRNA for methionyl tRNA synthetase, complete cds
Length=2800

 Score = 93.5 bits (50),  Expect = 6e-17
 Identities = 50/50 (100%), Gaps = 0/50 (0%)
 Strand=Plus/Plus

Query  1     CATGCCCTGTGGTGCAGTCGAGCCAGCACCTGTTTCTGGACCTGCCTAAG  50
             ||||||||||||||||||||||||||||||||||||||||||||||||||
Sbjct  1342  CATGCCCTGTGGTGCAGTCGAGCCAGCACCTGTTTCTGGACCTGCCTAAG  1391


>ref|XM_003824853.1| PREDICTED: Pan paniscus methionyl-tRNA synthetase (MARS), mRNA
Length=3665

 Score = 87.9 bits (47),  Expect = 3e-15
 Identities = 49/50 (98%), Gaps = 0/50 (0%)
 Strand=Plus/Plus

Query  1     CATGCCCTGTGGTGCAGTCGAGCCAGCACCTGTTTCTGGACCTGCCTAAG  50
             ||||||||||||||||||||| ||||||||||||||||||||||||||||
Sbjct  2010  CATGCCCTGTGGTGCAGTCGACCCAGCACCTGTTTCTGGACCTGCCTAAG  2059


>ref|XM_509164.3| PREDICTED: Pan troglodytes methionyl-tRNA synthetase (MARS), 
mRNA
Length=3469

 Score = 87.9 bits (47),  Expect = 3e-15
 Identities = 49/50 (98%), Gaps = 0/50 (0%)
 Strand=Plus/Plus

Query  1     CATGCCCTGTGGTGCAGTCGAGCCAGCACCTGTTTCTGGACCTGCCTAAG  50
             ||||||||||||||||||||| ||||||||||||||||||||||||||||
Sbjct  2010  CATGCCCTGTGGTGCAGTCGACCCAGCACCTGTTTCTGGACCTGCCTAAG  2059


>gb|AC191734.3| Pan troglodytes BAC clone CH251-555H2 from chromosome 12, complete 
sequence
Length=223618

 Score = 87.9 bits (47),  Expect = 3e-15
 Identities = 49/50 (98%), Gaps = 0/50 (0%)
 Strand=Plus/Minus

Query  1      CATGCCCTGTGGTGCAGTCGAGCCAGCACCTGTTTCTGGACCTGCCTAAG  50
              ||||||||||||||||||||| ||||||||||||||||||||||||||||
Sbjct  76081  CATGCCCTGTGGTGCAGTCGACCCAGCACCTGTTTCTGGACCTGCCTAAG  76032


>ref|XM_003252767.1| PREDICTED: Nomascus leucogenys methionyl-tRNA synthetase (MARS), 
mRNA
Length=2916

 Score = 82.4 bits (44),  Expect = 1e-13
 Identities = 48/50 (96%), Gaps = 0/50 (0%)
 Strand=Plus/Plus

Query  1     CATGCCCTGTGGTGCAGTCGAGCCAGCACCTGTTTCTGGACCTGCCTAAG  50
             |||| |||||||||||||||| ||||||||||||||||||||||||||||
Sbjct  1457  CATGTCCTGTGGTGCAGTCGACCCAGCACCTGTTTCTGGACCTGCCTAAG  1506


Lambda     K      H
    1.33    0.621     1.12 

Gapped
Lambda     K      H
    1.28    0.460    0.850 

Effective search space used: 874911716973
 
 right 
 
 
    reads 
 chr12 chr12 57894225 57897970 74M3669N2M                     |||||||||||||||||||||||||||||||||||||||||||||||||||||||||||||||||||||||||||||||||||||||||||||||||||||||||||||||||||||||||||||||||||||||||||||||||||||||||||||||||||||||||||||||||||||||||||CT
chr12 chr12 57894230 57898008 75M3702N1M                     ||||||||||||||||||||||||||||||||||||||||||||||||||||||||||||||||||||||||||||||||||||||||||||||||||||||||||||||||||||||||||||||||||||||||||||||||||||||||||||||||||||||||||||||||||||||||||||||||||||||||||||||||||||||||||||||||||A
chr12 chr12 57894233 57898011 72M3702N4M                     ||||||||||||||||||||||||||||||||||||||||||||||||||||||||||||||||||||||||||||||||||||||||||||||||||||||||||||||||||||||||||||||||||||||||||||||||||||||||||||||||||||||||||||||||||||||||||||||||||||||||||||||||||||||||||||||||||AAGC
chr12 chr12 57894236 57898014 69M3702N7M                     ||||||||||||||||||||||||||||||||||||||||||||||||||||||||||||||||||||||||||||||||||||||||||||||||||||||||||||||||||||||||||||||||||||||||||||||||||||||||||||||||||||||||||||||||||||||||||||||||||||||||||||||||||||||||||||||||||AAGCCTC
chr12 chr12 57894239 57898017 66M3702N10M                    ||||||||||||||||||||||||||||||||||||||||||||||||||||||||||||||||||||||||||||||||||||||||||||||||||||||||||||||||||||||||||||||||||||||||||||||||||||||||||||||||||||||||||||||||||||||||||||||||||||||||||||||||||||||||||||||||||AAGCCTCAGT
chr12 chr12 57894242 57898020 63M3702N13M                    ||||||||||||||||||||||||||||||||||||||||||||||||||||||||||||||||||||||||||||||||||||||||||||||||||||||||||||||||||||||||||||||||||||||||||||||||||||||||||||||||||||||||||||||||||||||||||||||||||||||||||||||||||||||||||||||||||AAGCCTCAGTGTA
chr12 chr12 57894245 57898023 60M3702N16M                    ||||||||||||||||||||||||||||||||||||||||||||||||||||||||||||||||||||||||||||||||||||||||||||||||||||||||||||||||||||||||||||||||||||||||||||||||||||||||||||||||||||||||||||||||||||||||||||||||||||||||||||||||||||||||||||||||||AAGCCTCAGTGTAAAG
chr12 chr12 57894248 57898026 57M3702N19M                    ||||||||||||||||||||||||||||||||||||||||||||||||||||||||||||||||||||||||||||||||||||||||||||||||||||||||||||||||||||||||||||||||||||||||||||||||||||||||||||||||||||||||||||||||||||||||||||||||||||||||||||||||||||||||||||||||||AAGCCTCAGTGTAAAGTCT
chr12 chr12 57894251 57898029 54M3702N22M                    ||||||||||||||||||||||||||||||||||||||||||||||||||||||||||||||||||||||||||||||||||||||||||||||||||||||||||||||||||||||||||||||||||||||||||||||||||||||||||||||||||||||||||||||||||||||||||||||||||||||||||||||||||||||||||||||||||AAGCCTCAGTGTAAAGTCTGCC
chr12 chr12 57894254 57898032 51M3702N25M                    ||||||||||||||||||||||||||||||||||||||||||||||||||||||||||||||||||||||||||||||||||||||||||||||||||||||||||||||||||||||||||||||||||||||||||||||||||||||||||||||||||||||||||||||||||||||||||||||||||||||||||||||||||||||||||||||||||AAGCCTCAGTGTAAAGTCTGCCGAT
chr12 chr12 57894257 57898035 48M3702N28M                    ||||||||||||||||||||||||||||||||||||||||||||||||||||||||||||||||||||||||||||||||||||||||||||||||||||||||||||||||||||||||||||||||||||||||||||||||||||||||||||||||||||||||||||||||||||||||||||||||||||||||||||||||||||||||||||||||||AAGCCTCAGTGTAAAGTCTGCCGATCAT
chr12 chr12 57894260 57898038 45M3702N31M                    ||||||||||||||||||||||||||||||||||||||||||||||||||||||||||||||||||||||||||||||||||||||||||||||||||||||||||||||||||||||||||||||||||||||||||||||||||||||||||||||||||||||||||||||||||||||||||||||||||||||||||||||||||||||||||||||||||AAGCCTCCGTGTAAAGGCTGCCGATCATGCC
chr12 chr12 57894263 57898041 42M3702N34M                    ||||||||||||||||||||||||||||||||||||||||||||||||||||||||||||||||||||||||||||||||||||||||||||||||||||||||||||||||||||||||||||||||||||||||||||||||||||||||||||||||||||||||||||||||||||||||||||||||||||||||||||||||||||||||||||||||||AAGCCTCAGTGTAAAGTCTGCCGATCATGCCCTG
chr12 chr12 57894266 57898044 39M3702N37M                    ||||||||||||||||||||||||||||||||||||||||||||||||||||||||||||||||||||||||||||||||||||||||||||||||||||||||||||||||||||||||||||||||||||||||||||||||||||||||||||||||||||||||||||||||||||||||||||||||||||||||||||||||||||||||||||||||||AAGCCTCAGTGTAAAGTCTGCCGATCATGCCCTGTGG
chr12 chr12 57894269 57898047 36M3702N40M                    ||||||||||||||||||||||||||||||||||||||||||||||||||||||||||||||||||||||||||||||||||||||||||||||||||||||||||||||||||||||||||||||||||||||||||||||||||||||||||||||||||||||||||||||||||||||||||||||||||||||||||||||||||||||||||||||||||AAGCCTCAGTGTAAAGTCTGCCGATCATGCCCTGTGGTGC
chr12 chr12 57894272 57898050 33M3702N43M                    ||||||||||||||||||||||||||||||||||||||||||||||||||||||||||||||||||||||||||||||||||||||||||||||||||||||||||||||||||||||||||||||||||||||||||||||||||||||||||||||||||||||||||||||||||||||||||||||||||||||||||||||||||||||||||||||||||AAGCCTCAGTGTAAAGTCTGCCGATCATGCCCTGTGGTGCAGA
chr12 chr12 57894275 57898053 30M3702N46M                    ||||||||||||||||||||||||||||||||||||||||||||||||||||||||||||||||||||||||||||||||||||||||||||||||||||||||||||||||||||||||||||||||||||||||||||||||||||||||||||||||||||||||||||||||||||||||||||||||||||||||||||||||||||||||||||||||||AAGCCTCAGTGTAAAGTCTGCCGATCATGCCCTGTGGTGCAGTCGA
chr12 chr12 57894278 57898056 27M3702N49M                    ||||||||||||||||||||||||||||||||||||||||||||||||||||||||||||||||||||||||||||||||||||||||||||||||||||||||||||||||||||||||||||||||||||||||||||||||||||||||||||||||||||||||||||||||||||||||||||||||||||||||||||||||||||||||||||||||||AAGCCTCAGTGTAAAGTCTGCCGATCATGCCCTGTGGTGCAGTCGAGCC
chr12 chr12 57894281 57898059 24M3702N52M                    ||||||||||||||||||||||||||||||||||||||||||||||||||||||||||||||||||||||||||||||||||||||||||||||||||||||||||||||||||||||||||||||||||||||||||||||||||||||||||||||||||||||||||||||||||||||||||||||||||||||||||||||||||||||||||||||||||AAGCCTCAGTGTAAAGTCTGCCGATCATGCCCTGTGGTGCAGTCGAGCCAGC
chr12 chr12 57894284 57898062 21M3702N55M                    ||||||||||||||||||||||||||||||||||||||||||||||||||||||||||||||||||||||||||||||||||||||||||||||||||||||||||||||||||||||||||||||||||||||||||||||||||||||||||||||||||||||||||||||||||||||||||||||||||||||||||||||||||||||||||||||||||AAGCCTCAGTGTAAAGTCTGCCGATCATGCCCTGTGGTGCAGTCGAGCCAGCACC
chr12 chr12 57894287 57898065 18M3702N58M                    ||||||||||||||||||||||||||||||||||||||||||||||||||||||||||||||||||||||||||||||||||||||||||||||||||||||||||||||||||||||||||||||||||||||||||||||||||||||||||||||||||||||||||||||||||||||||||||||||||||||||||||||||||||||||||||||||||AAGCCTCAGTGTAAAGTCTGCCGATCATGCCCTGTGGTGCAGTCGAGCCAGCACCTGT
chr12 chr12 57894290 57898068 15M3702N61M                    ||||||||||||||||||||||||||||||||||||||||||||||||||||||||||||||||||||||||||||||||||||||||||||||||||||||||||||||||||||||||||||||||||||||||||||||||||||||||||||||||||||||||||||||||||||||||||||||||||||||||||||||||||||||||||||||||||AAGCCTCAGTGTAAAGTCTGCCGATCATGCCCTGTGGTGCAGTCGAGCCAGCACCTGTTTC
chr12 chr12 57894293 57898071 12M3702N64M                    ||||||||||||||||||||||||||||||||||||||||||||||||||||||||||||||||||||||||||||||||||||||||||||||||||||||||||||||||||||||||||||||||||||||||||||||||||||||||||||||||||||||||||||||||||||||||||||||||||||||||||||||||||||||||||||||||||AAGCCTCAGTGTAAAGTCTGCCGATCATGCCCTGTGGTGCAGTCGAGCCAGCACCTGTTTCTGG
chr12 chr12 57894296 57898074 9M3702N67M                     ||||||||||||||||||||||||||||||||||||||||||||||||||||||||||||||||||||||||||||||||||||||||||||||||||||||||||||||||||||||||||||||||||||||||||||||||||||||||||||||||||||||||||||||||||||||||||||||||||||||||||||||||||||||||||||||||||AAGCCTCAGTTTAAAGTCTGCCGATCATGCCCTGTGGTGCAGTCGAGCCAGCACCTGTTTCTGGACC
chr12 chr12 57894299 57898077 6M3702N70M                     ||||||||||||||||||||||||||||||||||||||||||||||||||||||||||||||||||||||||||||||||||||||||||||||||||||||||||||||||||||||||||||||||||||||||||||||||||||||||||||||||||||||||||||||||||||||||||||||||||||||||||||||||||||||||||||||||||AAGCCTCAGTGTAAAGTCTGCCGATCATGCCCTGTGGTGCAGTCGAGCCAGCACCTGTTTCTGGACCTGC
chr12 chr12 57894302 57898080 3M3702N73M                     ||||||||||||||||||||||||||||||||||||||||||||||||||||||||||||||||||||||||||||||||||||||||||||||||||||||||||||||||||||||||||||||||||||||||||||||||||||||||||||||||||||||||||||||||||||||||||||||||||||||||||||||||||||||||||||||||||AGGCCTCAGTGTAAAGTCTGCCGATCATGCCCTGTGGTCCAGTCGAGCCAGCACCTGTTTCTGGACCTGCCTA
chr12 chr12 57897715 57897791 76M                            GGTGTGATGG
chr12 chr12 57897719 57897795 76M                            GGTGTGATGGGACA
chr12 chr12 57897742 57897818 76M                            GGTGTGATGGGACAGAGATCTGAAACTCTGAGAACAA
chr12 chr12 57897746 57897822 76M                            GGTGTGATGGGACAGAGATCTGAAACTCTGAGAACAAATGG
chr12 chr12 57897780 57897856 76M                            GGTGTGATGGGACAGAGATCTGAAACTCTGAGAACAAATGGAGTTATACTGAGAAGATTTGAGTCTCCATGTTTA
chr12 chr12 57897786 57897862 76M                                 GATGGGACAGAGATCTGAAACTCTGAGAACAAATGGAGTTATACTGAGAAGATTTGAGTCTCCATGTTTAGAGTAG
chr12 chr12 57897804 57897880 76M                                                   AACTCTGAGAACAAATGGAGTTATACTGAGAAGATTTGAGTCTCCATGTTTAGAGTAGGCCACATAGTAGAGTTAT
chr12 chr12 57897832 57897908 76M                                                                               AGAAGATTTGAGTCTCCATGTTTAGAGTAGGCCACATAGTAGAGTTATTGGAGTGTGAGCCTGAAGCTAAACTAGA
chr12 chr12 57897852 57897928 76M                                                                                                   TTTAGAGTAGGCCACATAGTAGAGTTATTGGAGTGTGAGCCTGAAGCTAAACTAGATGGCAGACTGAGGAGCTAAT
chr12 chr12 57897874 57897950 76M                                                                                                                         AGTTATTGGAGTGTGAGCCTGAAGCTAAACTAGATGGCAGACTGAGGAGCTAATCCTTCTCTGCTCCTCCCCTTGC
chr12 chr12 57897880 57897956 76M                                                                                                                               TGGAGTGTGAGCCTGAAGCTAAACTAGATGGCAGACTGAGGAGCTAATCCTTCTCTGCTCCTCCCCTTGCCTGGAC
chr12 chr12 57897893 57897969 76M                                                                                                                                            TGAAGCTAAACTAGATGGCAGACTGAGGAGCTAATCCTTCTCTGCTCCTCCCCTTGCCTGGACCCCTCCCCTGGCC
chr12 chr12 57897903 57897979 76M                                                                                                                                                      CTAGATGGCAGACTGAGGAGCTAATCCTTCTCTGCTCCTCCCCTTGCCTGGACCCCTCCCCTGGCCTGCAGGCCTG
chr12 chr12 57897909 57897985 76M                                                                                                                                                            GGCAGACTGAGGAGCTAATCCTTCTCTGCTCCTCCCCTTGCCTGGACCCCTCCCCTGGCCTGCAGGCCTGATCTGT
chr12 chr12 57897923 57897999 76M                                                                                                                                                                          CTAATCCTTCTCTGCTCCTCCCCTTGCCTGGACCCCTCCCCTGGCCTGCAGGCCTGATCTGTCCTCTGGAATTTTC
chr12 chr12 57897928 57898004 76M                                                                                                                                                                               CCTTCTCTGCTCCTCCCCTTGCCTGGACCCCTCCCCTGGCCTGCAGGCCTGATCTGTCCTCTGGAATTTTCCTTCG
chr12 chr12 57897953 57898029 76M                                                                                                                                                                                                        GACCCCTCCCCTGGCCTGCAGGCCTGATCTGTCCTCTGGAATTTTCCTTCGCAGAAGCCTCAGTGTAAAGTCTGCC
chr12 chr12 57897972 57898048 76M                                                                                                                                                                                                                           AGGCCTGATCTGTCCTCTGGAATTTTCCTTCGCAGAAGCCTCAGTGTAAAGTCTGCCGATCATGCCCTGTGGTGCA
chr12 chr12 57897976 57898052 76M                                                                                                                                                                                                                               CTGATCTGTCCTCTGGAATTTTCCTTCGCAGAAGCCTCAGTGTAAAGTCTGCCGATCATGCCCTGTGGTGCAGTCG
chr12 chr12 57897989 57898065 76M                                                                                                                                                                                                                                            TGGAATTTTCCTTCGCAGAAGCCTCAGTGTAAAGTCTGCCGATCATGCCCTGTGGTGCAGTCGAGCCAGCACCTGT
chr12 chr12 57897995 57898071 76M                                                                                                                                                                                                                                                  TTTCCTTCGCAGAAGCCTCAGTGTAAAGTCTGCCGATCATGCCCTGTGGTGCAGTCGAGCCAGCACCTGTTTCTGG
chr12 chr12 57898003 57898079 76M                                                                                                                                                                                                                                                          TAAGAAGCCTCAGTGTAAAGTCTGCCGATCATGCCCTGTGGTGCAGTCGAGCCAGCACCTGTTTCTGGACCTGCCT
chr12 chr12 57898006 57898082 76M                                                                                                                                                                                                                                                             GAAGCCTCAGTGTAAAGTCTCCCGATCATGCCCTGTGGTGCAGTCGAGCCAGCACCTGTTTCTGGACCTGCCTAAG
chr12 chr12 57898009 57898085 76M                                                                                                                                                                                                                                                                GCCTCAGTGTAAAGTCTGCCGATCATGCCCTGTGGTGCAGTCGAGCCAGCACCTGTTTCTGGACCTGCCTAAGCTG
chr12 chr12 57898010 59195045 72M59195042F4M                                                                                                                                                                                                                                                      CCTCAGTGTAAAGTCTGCCGATCATGCCCTGTGGTGCAGTCGAGCCAGCACCTGTTTCTGGACCTGCCTAAG GGCC
chr12 chr12 57898010 59195045 72M59195042F4M                                                                                                                                                                                                                                                      CCTCAGTGTAAAGTCTGCCGATCATGCCCTGTGGTGCAGTCGAGCCAGCACCTGTTTCTGGACCTGCCTAAG GGCC
chr12 chr12 57898010 59195045 72M59195042F4M                                                                                                                                                                                                                                                      CCTCAGTGTAAAGTCTGCGGATCATGCCCTGTGGTGCAGTCGAGCCAGCACCTGTTTCTGGACCTGCCTAAG GGCC
chr12 chr12 57898010 59195045 72M59195042F4M                                                                                                                                                                                                                                                      CCTCAGTGTAAAGTCTGCCGATCATGCCCTGTGGTGCAGTCGAGCCAGCACCTGTTTCTGGACCTGCCTAAG GGCC
chr12 chr12 57898010 59195045 72M59195042F4M                                                                                                                                                                                                                                                      CCTCAGTGTAAAGTCTGCCGATCATGCCCTGTGGTGCAGTCGAGCCAGCACCTGTTTCTGGACCTGCCTAAG GGCC
chr12 chr12 57898010 59195045 72M59195042F4M                                                                                                                                                                                                                                                      CCTCAGTGTAAAGTCTGCCGATCATGCCCTGTGGTGCAGTCGAGCCAGCACCTGTTTCTGGACCTGCCTAAG GGCC
chr12 chr12 57898010 59195045 72M59195042F4M                                                                                                                                                                                                                                                      CCTCAGTGTAAAGTCTGCCGATCATGCCCTGTGGTGCAGTCGAGCCAGCACCTGTTTCTGGACCTGCCTAAG GGCC
chr12 chr12 57898010 59195045 72M59195042F4M                                                                                                                                                                                                                                                      CCTCAGTGTAAAGTCTGCCGATCATGCCCTGTGGTGCAGTCGAGCCAGCACCTGTTTCTGGACCTGCCTAAG GGCC
chr12 chr12 57898010 59195045 72M59195042F4M                                                                                                                                                                                                                                                      CCTCAGTGTAAAGTCTGCCGATCATGCCCTGTGGTGCAGTCGAGCCAGCACCTGTTTCTGGACCTGCCTAAG GGCC
chr12 chr12 57898010 59195045 72M59195042F4M                                                                                                                                                                                                                                                      CCTCAGTGTAAAGTCTGCCGATCATGCCCTGTGGTGCAGTCGAGCCAGCACCTGTTTCTGGACCTGCCTAAG GGCC
chr12 chr12 57898010 59195045 72M59195042F4M                                                                                                                                                                                                                                                      CCTCAGTGTAAAGTCTGCCGATCATGCCCTGTGGTGCAGTCGAGCCAGCACCTGTTTCTGGACCTGCCTAAG GGCC
chr12 chr12 57898010 59195045 72M59195042F4M                                                                                                                                                                                                                                                      CCTCAGTGTAAAGTCTGCCGATCATGCCCTGTGGTGCAGTCGAGCCAGCACCTGTTTCTGGACCTGCCTAAG GGCC
chr12 chr12 57898010 59195045 72M59195042F4M                                                                                                                                                                                                                                                      CCTCAGTGTAAAGTCTGCCGATCATGCCCTGTGGTGCAGTCGAGCCAGCACCTGTTTCTGGACCTGCCTAAG GGCC
chr12 chr12 57898010 59195045 72M59195042F4M                                                                                                                                                                                                                                                      CCTCAGTGTAAAGTCTGCCGATCATGCCCTGTGGTGCAGTCGAGCCAGCACCTGTTTCTGGACCTGCCTAAG GGCC
chr12 chr12 57898011 59195046 71M59195042F5M                                                                                                                                                                                                                                                       CTCAGTGTAAAGTCTGCCGATCATGCCCTGTGGTGCAGTCGAGCCAGCACCTGTTTCTGGACCTGCCTAAG GGCCT
chr12 chr12 57898011 59195046 71M59195042F5M                                                                                                                                                                                                                                                       CTCAGTGTAAAGTCTGCCGATCATGCCCTGTGGTGCAGTCGAGCCAGCACCTGTTTCTGGACCTGCCTAAG GGCCT
chr12 chr12 57898011 59195046 71M59195042F5M                                                                                                                                                                                                                                                       CTCAGTGTAAAGTCTGCCGATCATGCCCTGTGGTGCAGTCGAGCCAGCACCTGTTTCTGGACCTGCCTAAG GGCCT
chr12 chr12 57898012 59195047 70M59195042F6M                                                                                                                                                                                                                                                        TCATTGTAAAGTCTGCCGATCATGCCCTGTGGTGCAGTCGAGCCAGCACCTGTTTCTGGACCTGCCTAAG GGCCTG
chr12 chr12 57898012 59195047 70M59195042F6M                                                                                                                                                                                                                                                        TCAGTGTAAAGTCTGCCGATCATGCCCTGTGGTGCAGTCGAGCCAGCACCTGTTTCTGGACCTGCCTAAG GGCCTG
chr12 chr12 57898012 59195047 70M59195042F6M                                                                                                                                                                                                                                                        TCAGTGTAAAGTCTGCCGATCATGCCCTGTGGTGCAGTCGAGCCAGCACCTGTTTCTGGACCTGCCTAAG GGCCTG
chr12 chr12 57898012 59195047 70M59195042F6M                                                                                                                                                                                                                                                        TCAGTGTAAAGTCTGCCGATTATGCCCTGTGGTGCAGTCGAGCCAGCACCTGTTTCTGGACCTGCCTAAG GGCCTG
chr12 chr12 57898013 59195048 69M59195042F7M                                                                                                                                                                                                                                                         CAGTGTAAAGTCTGCCGATCATGCCCTGTGGTGCAGTCGAGCCAGCACCTGTTTCTGGACCTGCCTAAG GGCCTGA
chr12 chr12 57898015 59195050 67M59195042F9M                                                                                                                                                                                                                                                           GTGTAAAGTCTGCCGATCATGCCCTGTGGTGCAGTCGAGCCAGCACCTGTTTCTGGACCTGCCTAAG GGCCTGAAT
chr12 chr12 57898015 59195050 67M59195042F9M                                                                                                                                                                                                                                                           GTGTAAAGTCTGCCGATCATGCCCTGTGGTGCAGTCGAGCCAGCACCTGTTTCTGGACCTGCCTAAG GGCCTGAAT
chr12 chr12 57898015 59195050 67M59195042F9M                                                                                                                                                                                                                                                           GTGTAAAGTCTGCCGATCATGCCCTGTGGTGCAGTCGAGCCAGCACCTGTTTCTGGACCTGCCTAAG GGCCTGAAT
chr12 chr12 57898015 59195050 67M59195042F9M                                                                                                                                                                                                                                                           GTGTAAAGTCTGCCGATCATGCCCTGTGGTGCAGTCGAGCCAGCACCTGTTTCTGGACCTGCCTAAG GGCCTGAAT
chr12 chr12 57898015 59195050 67M59195042F9M                                                                                                                                                                                                                                                           GTGTAAAGTCTGCCGATCATGCCCTGTGGTGCAGTCGAGCCAGCACCTGTTTCTGGACCTGCCTAAG GGCCTGAAT
chr12 chr12 57898015 59195050 67M59195042F9M                                                                                                                                                                                                                                                           GTGTAAAGTCCGCCGATCATGCCCTGTGGTGCAGTCGAGCCAGCACCTGTTTCTGGACCTGCCTAAG GGCCTGAAT
chr12 chr12 57898016 59195051 66M59195042F10M                                                                                                                                                                                                                                                           TGTAAAGTCTGCCGATCATGCCCTGTGGTGCAGTCGAGCCAGCACCTGTTTCTGGACCTGCCTAAG GGCCTGAATT
chr12 chr12 57898016 59195051 66M59195042F10M                                                                                                                                                                                                                                                           TGTAAAGTCTGCCGATCATGCCCTGTGGTGCAGTCGAGCCAGCACCTGTTTCTGGACCTGCCTAAG GGCCTGAATT
chr12 chr12 57898016 59195051 66M59195042F10M                                                                                                                                                                                                                                                           TGTAAAGCCTGCCGATCATGCCCTGTGGTGCGGTCGAGCCAGCACCTGTTTCTGGACCTGCCTAAG GGCCTGAATT
chr12 chr12 57898016 59195051 66M59195042F10M                                                                                                                                                                                                                                                           TGTAAAGTCTGCCGATCATGCCCTGTGGTGCAGTCGAGCCAGCACCTGTTTCTGGACCTGCCTAAG GGCCTGAATT
chr12 chr12 57898016 59195051 66M59195042F10M                                                                                                                                                                                                                                                           TGTAAAGTCTGCCGATTATGCCCTGTGGTGCAGTCGAGCCAGCACCTGTTTCTGGACCTGCCTAAG GGCCTGAATT
chr12 chr12 57898016 59195051 66M59195042F10M                                                                                                                                                                                                                                                           TGTAAAGTCTGCCGATAATCCCCTGTGGTGCAGTCGAGCCAGCACCTGTTTCTGGACCTGCCTAAG GGCCTGAATT
chr12 chr12 57898017 59195052 65M59195042F11M                                                                                                                                                                                                                                                            GTAAAGTCTGCCGATCATGCCCTGTGGTGCAGTCGAGCCAGCACCTGTTTCTGGACCTGCCTAAG GGCCTGAATTT
chr12 chr12 57898017 59195052 65M59195042F11M                                                                                                                                                                                                                                                            GTAAAGTCTGCCGATCATGCCCTGTGGTGCAGTCGAGCCAGCACCTGTTTCTGGACCTGCCTAAG GGCCTGAATTT
chr12 chr12 57898017 59195052 65M59195042F11M                                                                                                                                                                                                                                                            GTAAAGTCTGCCGATCATGCCCTGTGGTGCAGTCGAGCCAGCACCTGTTTCTGGACCTGCCTAAG GGCCTGAATTT
chr12 chr12 57898017 59195052 65M59195042F11M                                                                                                                                                                                                                                                            GTAAAGTCTGCCGATCATGCCCTGTGGTGCAGTCGAGCCAGCACCTGTTTCTGGACCTGCCTAAG GGCCTGAATTT
chr12 chr12 57898017 59195052 65M59195042F11M                                                                                                                                                                                                                                                            GTAAAGTCTGCCGATCATGCCCTGTGGTGCAGTCGAGCCAGCACCTGTTTCTGGACCTGCCTAAG GGCCTGAATTT
chr12 chr12 57898017 59195052 65M59195042F11M                                                                                                                                                                                                                                                            GTAAAGTCTGCCGATCATGCCCTGTGGTGCAGTCGAGCCAGCACCTGTTTCTGGACCTGCCTAAG GGCCTGAATTT
chr12 chr12 57898017 59195052 65M59195042F11M                                                                                                                                                                                                                                                            GTAAAGTCTGCCGATCATGCCCTGTGGCCCAGTCGAGCCAGCACCTGTTTCTGGACCTGCCTAAG GGCCTGAATTT
chr12 chr12 57898017 59195052 65M59195042F11M                                                                                                                                                                                                                                                            GTAAAGTCGGCCGATCATGCCCTGTGGTGCAGTCGAGCCAGCACCTGTTTCTGGACCTGCCTAAG GGCCTGAATTT
chr12 chr12 57898017 59195052 65M59195042F11M                                                                                                                                                                                                                                                            GTAAAGTCTGCCGATCATGCCCTGTGGTGCAGTCGAGCCAGCACCTGTTTCTGGACCTGCCTAAG GGCCTGAATTT
chr12 chr12 57898017 59195052 65M59195042F11M                                                                                                                                                                                                                                                            GTAAAGTCTGCCGATCATGCCCTGTGGTGCAGTCGAGCCAGCACCTGTTTCTGGACCTGCCTAAG GGCCTGAATTT
chr12 chr12 57898017 59195052 65M59195042F11M                                                                                                                                                                                                                                                            GTAAAGTCTGCCGATCATGCCCTGTGGTGCAGTCGAGCCAGCACCTGTTTCTGGACCTGCCTAAG GGCCTGAATTT
chr12 chr12 57898017 59195052 65M59195042F11M                                                                                                                                                                                                                                                            GTAAAGTCTGCCGATCATGCCCTGTGGTGCAGTCGAGCCAGCACCTGTTTCTGGACCTGCCTAAG GGCCTGAATTT
chr12 chr12 57898017 59195052 65M59195042F11M                                                                                                                                                                                                                                                            GTAAAGTCTGCCGATCATGCCCTGTGGTGCAGTCGAGCCAGCACCTGTTTCTGGACCTGCCTAAG GGCCTGAATTT
chr12 chr12 57898017 59195052 65M59195042F11M                                                                                                                                                                                                                                                            GTAAAGTCTGCCGATCATGCCCTGTGGTGCAGTCGAGCCAGCACCTGTTTCTGGACCTGCCTAAG GGCCTGAATTT
chr12 chr12 57898017 59195052 65M59195042F11M                                                                                                                                                                                                                                                            GTAAAGTCTGCCGATCATGCCCTGTGGTGCAGTCGAGCCAGCACCTGTTTCTGGACCTGCCTAAG GGCCTGAATTT
chr12 chr12 57898017 59195052 65M59195042F11M                                                                                                                                                                                                                                                            GTAAAGTCTGCCGATCATGCCCTGTGGTGCAGTCGAGCCAGCACCTGTTTCTGGACCTGCCTAAG GGCATGAATTT
chr12 chr12 57898017 59195052 65M59195042F11M                                                                                                                                                                                                                                                            GTAAAGTCTGCCGATCATGCCCTGTGGTGCAGTCGAGCCAGCACCTGTTTCTGGACCTGCCTAAG GGCCTGAATTT
chr12 chr12 57898017 59195052 65M59195042F11M                                                                                                                                                                                                                                                            GTAAAGTCTGCCGATCATGCCCTGTGGTGCAGTCGAGCCAGCACCTGTTTCTGGACCTGCCTAAG GGCCTGAATTT
chr12 chr12 57898017 59195052 65M59195042F11M                                                                                                                                                                                                                                                            GTAAAGTCTGCCGATCATGCCCTGTGGTGCAGTCGAGCCAGCACCTGTTTCTGGACCTGCCTAAG GGCCTGAATTT
chr12 chr12 57898017 59195052 65M59195042F11M                                                                                                                                                                                                                                                            GTAAAGTCTGCCGATCATGCCCTGTGGTGCAGTCGAGCCAGCACCTGTTTCTGGACCTGCCTAAG GGCCTGAATTT
chr12 chr12 57898017 59195052 65M59195042F11M                                                                                                                                                                                                                                                            GTAAAGTCTGCCGATCATGCCCTGTGGTGCAGTCGAGCCAGCACCTGTTTCTGGACCTGCCTAAG GGCCTGAATTT
chr12 chr12 57898018 59195053 64M59195042F12M                                                                                                                                                                                                                                                             TAAAGTCTGCCGATCATGCCCTGTGGTGCAGTCGAGCCAGCACCTGTTTCTGGACCTGCCTAAG GGCCTGAATTTT
chr12 chr12 57898018 59195053 64M59195042F12M                                                                                                                                                                                                                                                             TAAAGTCTGCCGATCATGCCCTGTGGTGCAGTCGAGCCAGCACCTGTTTCTGGACCTGCCTAAG GGCCTGAATTTT
chr12 chr12 57898018 59195053 64M59195042F12M                                                                                                                                                                                                                                                             AAAAGTCTGCCGATCATGCCCTGTGGTGCAGTCGAGCCAGCACCTGTTTCTGGACCTGCCTAAG GGCCTGAATTTT
chr12 chr12 57898019 59195054 63M59195042F13M                                                                                                                                                                                                                                                              AAAGTCTGCCGATCATGCCCTGTGGTGCAGTCGAGCCAGCACCTGTTTCTGGACCTGCCTAAG GGCCTGAATTTTC
chr12 chr12 57898019 59195054 63M59195042F13M                                                                                                                                                                                                                                                              AAAGTCTGCCGATCATGCCCTGTGGTGCAGTCGAGCCAGCACCTGTTTCTGGACCTGCCTTAG GGCCTGAATTTTC
chr12 chr12 57898019 59195054 63M59195042F13M                                                                                                                                                                                                                                                              AAAGTCTGCCGATCATGCCCTGTGGTGCAGTCGAGCCAGCACCTGTTTCTGGACCTGCCTAAG GGCCTGAATTTTC
chr12 chr12 57898019 59195054 63M59195042F13M                                                                                                                                                                                                                                                              AAAGTCTGCCGATCATGCCCTGTGGTGCAGTCGAGCCAGCACCTGTTTCTGGACCTGCCTAAG GGCCTGAATTTTC
chr12 chr12 57898019 59195054 63M59195042F13M                                                                                                                                                                                                                                                              AAAGTCTGCCGATCATGCCCTGGGGTGCAGTCGAGCCAGCACCTGTTTCTGGACCTGCCTAAG GGCCTGAATTTTC
chr12 chr12 57898019 59195054 63M59195042F13M                                                                                                                                                                                                                                                              AAAGTCTGCCGATCATGCCCGGTGGTGCAGTCGAGCCAGCACCTGTTTCTGGACCTGCCTAAG GGCCTGAATTTTC
chr12 chr12 57898019 59195054 63M59195042F13M                                                                                                                                                                                                                                                              AAAGTCTGCCGATCATGCCCTGTGGTGCAGTCGAGCCAGCACCTGTTTCTGGACCTGCCTAAG GGCCTGAATTTTC
chr12 chr12 57898019 59195054 63M59195042F13M                                                                                                                                                                                                                                                              AAAGTCTGCCGATCATGCCCTGTGGTGCAGTCGAGCCAGCACCTGTTTCTGGACCTGCCTAAG GGCCTGAATTTTC
chr12 chr12 57898019 59195054 63M59195042F13M                                                                                                                                                                                                                                                              AAAGTCTGCCGATCATGCCCTGTGGTGCAGTCGAGCCAGCACCTGTTTCTGGACCTGCCTAAG GGCCTGAATTTTC
chr12 chr12 57898019 59195054 63M59195042F13M                                                                                                                                                                                                                                                              AAAGTCTGCCGATCATGCCCTGTGGTGCAGTCGAGCCAGCACCTGTTTCTGGACCTGCCTAAG GGCCTGAATTTTC
chr12 chr12 57898019 59195054 63M59195042F13M                                                                                                                                                                                                                                                              AAAGTCTGCCGATCATGCCCTGTGGTGCAGTCGAGCCAGCACCTGTTTCTGGACCTGCCTAAG GGCCTGAATTTTC
chr12 chr12 57898019 59195054 63M59195042F13M                                                                                                                                                                                                                                                              AAAGTCTGCCGATCATGCCCTGTGGTGCATTCGAGCCAGCACCTGTTTCTGGACCTGCCTAAG GGCCTGAATTTTC
chr12 chr12 57898019 59195054 63M59195042F13M                                                                                                                                                                                                                                                              AAAGTCTGCCGATCATGCCCTGTGGTGCAGTCGAGCCAGCACCTGTTTCTGGACCTGCCTAAG GGCCTGAATTTTC
chr12 chr12 57898019 59195054 63M59195042F13M                                                                                                                                                                                                                                                              AAAGTCTGCCGATCATGCCCTGTGGTGCGGTGGAGCCAGCACCTGTTTCTGGACCTGCCTAAG GGCCTGAATTTTC
chr12 chr12 57898019 59195054 63M59195042F13M                                                                                                                                                                                                                                                              AAAGTCTGCCGATCATGCCCTGTGGTGCAGTCGAGCCAGCACCTGTTTCTGGACCTGCCTAAG GGCCTGAATTTTC
chr12 chr12 57898019 59195054 63M59195042F13M                                                                                                                                                                                                                                                              AAGGTCTGCCGATCATGCCCTGTGGTGCAGTCGAGCCAGCACCTGTTTCTGGACCTGCCTAAG GGCCTGAATTTTC
chr12 chr12 57898019 59195054 63M59195042F13M                                                                                                                                                                                                                                                              AAAGTCTGCCGATCATGCCCTGTGGTGCAGTGGAGCCAGCACCTGTTTCTGGACCTGCCTAAG GGCCTGAATTTTC
chr12 chr12 57898019 59195054 63M59195042F13M                                                                                                                                                                                                                                                              AAAGTCTGCCTATCATGCCCTGTGGTGCAGTCGAGCCAGCACCTGTTTCTGGACCTGCCTAAG GGCCTGAATTTTC
chr12 chr12 57898019 59195054 63M59195042F13M                                                                                                                                                                                                                                                              AAAGTCTGCCGATCATGCCCTGTGGTGCAGTCGAGCCAGCACCTGTTTCTGGACCTGCCTAAG GGCCTGAATTTTC
chr12 chr12 57898019 59195054 63M59195042F13M                                                                                                                                                                                                                                                              AAAGTCTGCCGATCATGCCCTGTGGTGCAGTCGAGCCAGCACCTGTTTCTGGACCTGCCTAAG GGCCTGAATTTTC
chr12 chr12 57898019 59195054 63M59195042F13M                                                                                                                                                                                                                                                              AAAGTCTGCCGATCATGCCCTGTGGTGCAGTCGAGCCAGCACCTGTTTCTGGACCTGCCTAAG GGCCTGAATTTTC
chr12 chr12 57898019 59195054 63M59195042F13M                                                                                                                                                                                                                                                              AAAGTCTGCCGATCATGCCCTGTGGTGCAGTCGAGCCAGCACCTGTTTCTGGACCTGCCTAAG GGCCTGAATTTTC
chr12 chr12 57898019 59195054 63M59195042F13M                                                                                                                                                                                                                                                              AAAGTCTGCCGATCATGCCCTGTGGTGCAGTCGAGCCAGCACCTGTTTCTGGACCTGCCTAAG GGCCTGAATTTTC
chr12 chr12 57898019 59195054 63M59195042F13M                                                                                                                                                                                                                                                              AAAGTCTGCCGATCATGCCCTGTGGTGCAGTCGAGCCAGCACCTGTTTCTGGACCTGCCTAAG GGCCTGAATTTTC
chr12 chr12 57898019 59195054 63M59195042F13M                                                                                                                                                                                                                                                              AAACTCTGCCGATCATGCCCTGTGGTGCAGTCGAGCCAGCACCTGTTTCTGGACCTGCCTAAG GGCCTGAATTTTC
chr12 chr12 57898019 59195054 63M59195042F13M                                                                                                                                                                                                                                                              AAGGTCTGCCGATCATGCCCTGTGGTGCAGTCGAGCCAGCACCTGTTTCTGGACCTGCCTAAG GGCCTGAATTTTC
chr12 chr12 57898019 59195054 63M59195042F13M                                                                                                                                                                                                                                                              AAAGTCTGCCGATCATGCCCTGTGGTGCAGTCGAGCCAGCACCTGTTTCTGGACCTGCCTAAG GGCCTGAATTTTC
chr12 chr12 57898019 59195054 63M59195042F13M                                                                                                                                                                                                                                                              AAAGTCTGCCGATCATGCCCTGTGGTGCAGTCGAGCCAGCACCTGTTTCTGGACCTGCCTAAG GGCCTGAATTTTC
chr12 chr12 57898019 59195054 63M59195042F13M                                                                                                                                                                                                                                                              AAAGTCTGCCGATCATGCCCTGTGGTGCAGTCGAGCCAGCACCTGTTTCTGGACCTGCCTAAG GGCCTGAATTTTC
chr12 chr12 57898019 59195054 63M59195042F13M                                                                                                                                                                                                                                                              AAAGTCTGCCGATCATGCCCTGTGGTGCAGTCGAGCCAGCACCTGTTTCTGGACCTGCCTAAG GGCCTGAATTTTC
chr12 chr12 57898019 59195054 63M59195042F13M                                                                                                                                                                                                                                                              AAAGTCTGCCGATCATGCCCTGTGGTGCAGTCGAGCCAGCACCTGTTTCTGGACCTGCCTAAG GGCCTGAATTTTC
chr12 chr12 57898019 59195054 63M59195042F13M                                                                                                                                                                                                                                                              AAAGTCTGCCGATCATGCCCTGTGGTGCAGTCGAGCCAGCACCTGTTTCTGGACCTGCCTAAG GGCCTGAATTTTC
chr12 chr12 57898020 59195055 62M59195042F14M                                                                                                                                                                                                                                                               AAGTCTGCCGATCATGCCCTGTGGTGCAGTCGAGCCAGCACCTGTTTCTGGACCTGCCTAAG GGCCTGAATTTTCT
chr12 chr12 57898020 59195055 62M59195042F14M                                                                                                                                                                                                                                                               AATTCTGCCGATCATGCCCTGTGGTGCAGTCGAGCCAGCACCTGTTTCTGGACCTGCCTAAG GGCCTGAATTTTCT
chr12 chr12 57898020 59195055 62M59195042F14M                                                                                                                                                                                                                                                               AAGTCTGCCGATCATGCCCTGTGGTGCAGTCGAGCCAGCACCTGTTTCTGGACCTGCCTAAG GGCCTGAATTTTTT
chr12 chr12 57898020 59195055 62M59195042F14M                                                                                                                                                                                                                                                               AACTCTGCCGATCATGCCCTGTGGTGCAGTCGAGCCAGCACCTGTTTCTGGACCTGCCTAAG GGCCTGAATTTTCT
chr12 chr12 57898020 59195055 62M59195042F14M                                                                                                                                                                                                                                                               AAATCTGCCGATCATGCCCTGTGGTGCAGTCGAGCCAGCACCTGTTTCTGGCCCTGCCTAAG GGCCTGAATTTTCT
chr12 chr12 57898020 59195055 62M59195042F14M                                                                                                                                                                                                                                                               AAGTCTGCCGATCATGCCCTGTGGTGCAGTCGAGCCAGCACCTGTTTCTGGACCTGCCTAAG GGCCTGAATTTTCT
chr12 chr12 57898020 59195055 62M59195042F14M                                                                                                                                                                                                                                                               AAGTCTGCCGATCATGCCCTGTGGTGCAGTCGAGCCAGCACCTGTTTCTGGACCTGCCTAAG GGCCTGAATTTTCT
chr12 chr12 57898021 59195056 61M59195042F15M                                                                                                                                                                                                                                                                AGTCTGCCGATCATGCCCTGTGGTGCAGTCGAGCCAGCACCTGTTTCTGGACCTGCCTAAG GGCCTGAATTTTCTG
chr12 chr12 57898021 59195056 61M59195042F15M                                                                                                                                                                                                                                                                AGTCTGCCGATCATGCCCTGTGGTGCAGTCGAGCCAGCACCTGTTTCTGGACCTGCCTAAG GGCCTGAATTTTCTG
chr12 chr12 57898021 59195056 61M59195042F15M                                                                                                                                                                                                                                                                AGTCTGCCGATCATGCCCTGTGGTGCAGTCGAGCCAGCACCTGTTTCTGGACCTGCCTAAG GGCCTGAATTTTCTG
chr12 chr12 57898022 59195057 60M59195042F16M                                                                                                                                                                                                                                                                 GTCTGCCGATCATGCCCTGTGGTGCAGTCGAGCCAGCACCTGTTTCTGGACCTGCCTAAG GGCCTGAATTTTCTGC
chr12 chr12 57898022 59195057 60M59195042F16M                                                                                                                                                                                                                                                                 GTCTGCCGATCATGCCCTGTGGTGCAGTCGAGCCAGCACCTGTTTCTGGACCTGCCTAAG GGCCTGAATTTTCTGC
chr12 chr12 57898022 59195057 60M59195042F16M                                                                                                                                                                                                                                                                 GTCTGCCGATCATGCCCTGTGGTGCAGTCGAGCCAGCACCTGTTTCTGGACCTGCCTAAG GGCCTGAATTTTCTGC
chr12 chr12 57898023 59195058 59M59195042F17M                                                                                                                                                                                                                                                                  TCTGCCGATCATGCCCTGTGGTGCAGTCGAGCCAGCACCTGTTTCTGGACCTGCCTAAG GGCCTGAATTTTCTGCT
chr12 chr12 57898024 59195059 58M59195042F18M                                                                                                                                                                                                                                                                   CTGCCGATCATGCCCTGTGGTGCAGTCGAGCCAGCACCTGTTTCTGGACCTGCCTAAG GGCCTGAATTTTCTGCTC
chr12 chr12 57898024 59195059 58M59195042F18M                                                                                                                                                                                                                                                                   CTGCCGATCATGCCCTGTGGTGCAGTCGAGCCAGCACCTGTTTCTGGACCTGCCTAAG GGCCTGAATTTTCTGCTC
chr12 chr12 57898024 59195059 58M59195042F18M                                                                                                                                                                                                                                                                   CTGCCGATCATGCCCTGTGGTGCAGTCGAGCCAGCACCTGTTTCTGGACCTGCCTAAG GGCCTGAATTTTCTGCTC
chr12 chr12 57898025 59195060 57M59195042F19M                                                                                                                                                                                                                                                                    TGCCGATCATGCCCTGTGGTGCAGTCGAGCCAGCACCTGGTTCTGGACCTGCCTAAG GGCCTGAATTTTCTGCTCC
chr12 chr12 57898025 59195060 57M59195042F19M                                                                                                                                                                                                                                                                    TGCCGATCATGCCCTGTGGTGCAGTCGAGCCAGCACCTGTTTCTGGACCTGCCTAAG GGCCTGAATTTTCTGCTCC
chr12 chr12 57898025 59195060 57M59195042F19M                                                                                                                                                                                                                                                                    TGCCGATCATGCCCTGTGGTGCAGTCGAGCCAGCACCTGTTTCTGGACCTGCCTAAG GGCCTGAATTTTCTGCTCC
chr12 chr12 57898025 59195060 57M59195042F19M                                                                                                                                                                                                                                                                    TGCCGATCATGCCCTGTGGTGCAGTCGAGCCAGCACCTGTTTCTGGACCTGCCTAAG GGCCTGAATTTTCTGCTCC
chr12 chr12 57898025 59195060 57M59195042F19M                                                                                                                                                                                                                                                                    TGCCGATCATGCCCTGTGGTGCAGTCGAGCCAGCACCTGTTTCTGGACCTGCCTAAG GGCCTGAATTTTCTGCTCC
chr12 chr12 57898025 59195060 57M59195042F19M                                                                                                                                                                                                                                                                    TGCCGATCATGCCCTGTGGTGCAGTCGAGCCAGCACCTGTTTCTGGACCTGCCTAAG GGCCTGAATTTTCTGCTCC
chr12 chr12 57898025 59195060 57M59195042F19M                                                                                                                                                                                                                                                                    TGCCGATCATGCCCTGTGGTGCAGTCGAGCCAGCACCTGTTTCTGGACCTGCCTAAG GGCCTGAATTTTCTGCTCC
chr12 chr12 57898025 59195060 57M59195042F19M                                                                                                                                                                                                                                                                    TGCCGATCATGCCCTGTGGTGCAGTCGAGCCAGCACCTGTTTCTGGACCTGCCTAAG GGCCTGAATTTTCTGCTCC
chr12 chr12 57898025 59195060 57M59195042F19M                                                                                                                                                                                                                                                                    TGCCGATCATGCCCTGTGGTGCAGTCGAGCCAGCACCTGTTTCTGGACCTGCCTAAG GGCCTGAATTTTCTGCTCC
chr12 chr12 57898025 59195060 57M59195042F19M                                                                                                                                                                                                                                                                    TGCCGATCATGCCCTGTGGTGCAGTCGAGCCAGCACCTGTTTCTGGACCTGCCTAAG GGCCTGAATTTTCTGCTCC
chr12 chr12 57898025 59195060 57M59195042F19M                                                                                                                                                                                                                                                                    TGCCGATCATGCCCTGTGGTGCAGTCGAGCCAGCACCTGTTTCTGGACCTGCCTAAG GGCCTGAATTTTCTGCTCC
chr12 chr12 57898025 59195060 57M59195042F19M                                                                                                                                                                                                                                                                    TGCCGTCCATGCCCTGTGGTGCAGTCGAGCCAGCACCTGTTTCTGGACCTGCCTAAG GGCCTGAATTTTCTGCTCC
chr12 chr12 57898025 59195060 57M59195042F19M                                                                                                                                                                                                                                                                    TGCCGATCATGCCCTGTGGTGCAGTCGAGCCAGCACCTGTTTCTGGACCTGCCTAAG GGCCTGAATTTTCTGCTCC
chr12 chr12 57898025 59195060 57M59195042F19M                                                                                                                                                                                                                                                                    TGCCGATCATGCCCTGTGGTGCAGTCGAGCCAGCACCTGTTTCTGGACCTGCCTAAG GGCCTGAATTTTCTGCTCC
chr12 chr12 57898025 59195060 57M59195042F19M                                                                                                                                                                                                                                                                    TGCCGATCATGCCCTGTGGTGCAGTCGAGCCAGCACCTGTTTCTGGACCTGCCTAAG GGCCTGAATTTTCTGCTCC
chr12 chr12 57898026 59195061 56M59195042F20M                                                                                                                                                                                                                                                                     GCCGATCATGCCCTGTGGTGCAGTCGAGCCAGCACCTGTTTCTGGACCTGCCTAAG GGCCTGAATTTTCTGCTCCT
chr12 chr12 57898026 59195061 56M59195042F20M                                                                                                                                                                                                                                                                     GCCGATCATGCCCTGTGGTGCAGTCGAGCCAGCACCTGTTTCTGGACCTGCCTAAG GGCCTGAATTTTCTGCTCCT
chr12 chr12 57898026 59195061 56M59195042F20M                                                                                                                                                                                                                                                                     GCCGATCATGCCCTGTGGTGCAGTCGAGCCAGCACCTGTTTCTGGACCTGCCTAAG GGCCTGAATTTTCTGCTCCT
chr12 chr12 57898026 59195061 56M59195042F20M                                                                                                                                                                                                                                                                     GCCGATCATGCCCTGTGGTGCAGTCGAGCCAGCACCTGTTTCTGGACCTGCCTAAG GGCCTGAATTTTCTGCTCCT
chr12 chr12 57898026 59195061 56M59195042F20M                                                                                                                                                                                                                                                                     GCCGAGCATGCCCTGTGGTGCAGTCGAGCCAGCACCTGTTTCTGGACCTGCCTAAG GGCCTGAATTTTCTGCTCCT
chr12 chr12 57898026 59195061 56M59195042F20M                                                                                                                                                                                                                                                                     GCCGATCATGCCCTGTGGTTCAGTCGAGCCAGCACCTGTTTCTGGACCTGCCTAAG GGCCTGAATTTTCTGCTCCT
chr12 chr12 57898026 59195061 56M59195042F20M                                                                                                                                                                                                                                                                     GCCGATCATGCCCTGTGGTGCAGTCGAGCCAGCACCTGTTTCTGGACCTGCCTAAG GGCCTGAATTTTCTGCTCCT
chr12 chr12 57898026 59195061 56M59195042F20M                                                                                                                                                                                                                                                                     GCCGATCATGCCCTGTGGTGCAGTCGAGCCAGCACCTGTTTCTGGACCTGCCTAAG GGCCTGAATTTTCTGCTCCT
chr12 chr12 57898026 59195061 56M59195042F20M                                                                                                                                                                                                                                                                     GCCGATCATGCCCTGTGGTGCAGTCGAGCCAGCACCTGTTTCTGGACCTGCCTAAG GGCCTGAATTTTCTGCTCCT
chr12 chr12 57898026 59195061 56M59195042F20M                                                                                                                                                                                                                                                                     GCCGATCATGCCCTGTGGTGCAGTCGAGCCAGCACCTGTTTCTGGACCTGCCTAAG GGCCTGAATTTTCTGCTCCT
chr12 chr12 57898026 59195061 56M59195042F20M                                                                                                                                                                                                                                                                     GCCGATCATGCCCTGTGGTGCAGTCGAGCCAGCACCTGTTTCTGGACCTGCCTAAG GGCCTGAATTTTCTGCTCCT
chr12 chr12 57898026 59195061 56M59195042F20M                                                                                                                                                                                                                                                                     GCCGATCATGCCCTGTGGTGCAGTCGAGCCAGCACCTGTTTCTGGACCTGCCTAAG GGCCTGAATTTTCTGCTCCT
chr12 chr12 57898026 59195061 56M59195042F20M                                                                                                                                                                                                                                                                     GCCGATCATGCCCTGTGGTGCAGTCGAGCCAGCACCTGTTTCTGGACCTGCCTAAG GGCCTGAATTTTCTGCTCCT
chr12 chr12 57898026 59195061 56M59195042F20M                                                                                                                                                                                                                                                                     GCCGATCATGCCCTGTGGTGCAGTCGAGCCAGCACCTGTTTCTGGACCTGCCTAAG GGCCTGAATTTTCTGCTCCT
chr12 chr12 57898026 59195061 56M59195042F20M                                                                                                                                                                                                                                                                     GCCGATCATGCCCTGTGGTGCAGTCGAGCCAGCACCTGTTTCTGGACCTGCCTAAG GGCCTGAATTTTCTGCTCCT
chr12 chr12 57898026 59195061 56M59195042F20M                                                                                                                                                                                                                                                                     GCCGATCATGCCCTGTGGTGCAGTCGAGCCAGCACCTGTTTCTGGACCTGCCTAAG GGCCTGAATTTTCTGCTCCT
chr12 chr12 57898026 59195061 56M59195042F20M                                                                                                                                                                                                                                                                     GCCGATCATGCCCTGTGGTGCAGTCGAGCCAGCACCTGTTTCTGGACCTGCCTAAG GGCCTGAATTTTCTGCTCCT
chr12 chr12 57898026 59195061 56M59195042F20M                                                                                                                                                                                                                                                                     GCCGATCATGCCCTGTGGTGCAGTCGAGCCAGCACCTGTTTCTGGACCTGCCTAAG GGCCTGAATTTTCTGCTCCT
chr12 chr12 57898026 59195061 56M59195042F20M                                                                                                                                                                                                                                                                     GCCGATCATGCCCTGTGGTGCAGTCGAGCCAGCACCTGTTTCTGGACCTGCCTAAG GGCCTGAATTTTCTGCTCCT
chr12 chr12 57898026 59195061 56M59195042F20M                                                                                                                                                                                                                                                                     GCCGATCATGCCCTGTGGTGCAGTCGAGCCAGCACCTGTTTCTGGACCTGCCTAAG GGCCTGAATTTTCTGCTCCT
chr12 chr12 57898026 59195061 56M59195042F20M                                                                                                                                                                                                                                                                     GCCGATCATGCCCTGTGGTGCAGTCGAGCCAGCACCTGTTTCTGGACCTGCCTAAG GGCCTGAATTTTCTGCTCCT
chr12 chr12 57898026 59195061 56M59195042F20M                                                                                                                                                                                                                                                                     GCCGATCATGCTCTGTGGTGCAGTCGAGCCAGCACCTGTTTCTGGACCTGCCTAAG GGCCTGAATTTTCTGCTCCT
chr12 chr12 57898026 59195061 56M59195042F20M                                                                                                                                                                                                                                                                     GCCGATCATGCCCTGTGGTGCAGTCGAGCCAGCACCTGTTTCTGGACCTGCCTAAG GGCCTGAATTTTCTGCTCCT
chr12 chr12 57898026 59195061 56M59195042F20M                                                                                                                                                                                                                                                                     GCCGATCATGCCCTGTGGTGCAGTCGAGCCAGCACCTGTTTCTGGACCTGCCTAAG GGCCTGAATTTTCTGCTCCT
chr12 chr12 57898026 59195061 56M59195042F20M                                                                                                                                                                                                                                                                     GCCGATCATGCCCTGTGGTGCAGTCGAGCCAGCACCTGTTTCTGGACCTGCCTAAG GGCCTGAATTTTCTGCTCCT
chr12 chr12 57898026 59195061 56M59195042F20M                                                                                                                                                                                                                                                                     GCCGATCATGCCCTGTGGTGCAGTCGAGCCAGCACCTGTTTCTGGACCTGCCTAAG GGCCTGAATTTTCTGCTCCT
chr12 chr12 57898026 59195061 56M59195042F20M                                                                                                                                                                                                                                                                     GCCGATCATGCCCTGTGGTGCAGTCGAGCCAGCACCTGTTTCTGGACCTGCCTAAG GGCCTGAATTTTCTGCTCCG
chr12 chr12 57898026 59195061 56M59195042F20M                                                                                                                                                                                                                                                                     GCCGATCATGCCCTGTGGTGCAGTCGAGCCAGCACCTGTTTCTGGACCTGCCTAAG GGCCTGAATTTTCTGCTCCT
chr12 chr12 57898026 59195061 56M59195042F20M                                                                                                                                                                                                                                                                     GCCGATCATGCCCTGTGGTGCAGTCGAGCCAGCACCTGTTTCTGGACCTGCCTAAG GGCCTGAATTTTCTGCTCCT
chr12 chr12 57898026 59195061 56M59195042F20M                                                                                                                                                                                                                                                                     GCCGATCATGCCCTGTGGTGCAGTCGAGCCAGCACCTGTTTCTGGACCTGCCTAAG GGCCTGAATTTTCTGCTCCT
chr12 chr12 57898026 59195061 56M59195042F20M                                                                                                                                                                                                                                                                     GCCGATCATGCCCTGTGGTGCAGTCGAGCCAGCACCTGTTTCTGGACCTGCCTAAG GGCCTGAATTTTCTGCTCCA
chr12 chr12 57898026 59195061 56M59195042F20M                                                                                                                                                                                                                                                                     GCCGATCATGCCCTGTGGCGCAGTCGAGCCAGCACCTGTTTCTGGACCTGCCTAAG GGCCTGAATTTTCTGCTCCT
chr12 chr12 57898026 59195061 56M59195042F20M                                                                                                                                                                                                                                                                     GCCGATCATGCCCTGTGGTGCAGTCGAGCCAGCACCTGTTTCTGGACCTGCCTAAG GGCCTGAATTTTCTGCTCCT
chr12 chr12 57898026 59195061 56M59195042F20M                                                                                                                                                                                                                                                                     GCCGATCATGCCCTGTGGTGCAGTCGAGCCAGCACCTGTTTCTGGACCTGCCTAAG GGCCTGAATTTTCTGCTCCT
chr12 chr12 57898026 59195061 56M59195042F20M                                                                                                                                                                                                                                                                     GCCGATCATGCCCTGTGGTGCAGTCGAGCCAGCACCTGTTTCTGGACCTGCCTAAG GGCCTGAATTTTCTGCTCCT
chr12 chr12 57898026 59195061 56M59195042F20M                                                                                                                                                                                                                                                                     GCCGATCATGCCCTGTGGTGCAGTCGAGCCAGCACCTGTTTCTGGACCTGCCTAAG GGCCTGAATTTTCTGCTCCT
chr12 chr12 57898026 59195061 56M59195042F20M                                                                                                                                                                                                                                                                     GCCGATCATGCCCTGTGGTGCAGTCGAGCCAGCACCTGTTTCTGGACCTGCCTAAG GGCCTGAATTTTCTGCTCCT
chr12 chr12 57898026 59195061 56M59195042F20M                                                                                                                                                                                                                                                                     GCCGATCATGCCCTGTGGTGCAGTCGAGCCAGCACCTGTTTCTGGACCTGCCTAAG GGCCTGAATTTTCTGCTCCT
chr12 chr12 57898026 59195061 56M59195042F20M                                                                                                                                                                                                                                                                     GCCGATCATGCCCTGTGGTGCAGTCGAGCCAGCACCTGTTTCTGGACCTGCCTAAG GGCCTGAATTTTCTGCTCCT
chr12 chr12 57898026 59195061 56M59195042F20M                                                                                                                                                                                                                                                                     GCCGATCATGCCCTGTGGTGCAGTCGAGCCAGCACCTGTTTCTGGACCTGCCTAAG GGCCTGAATTTTCTGCTCCT
chr12 chr12 57898026 59195061 56M59195042F20M                                                                                                                                                                                                                                                                     GCCGATCATGCCCTGTGGTGCAGTCGAGCCAGCACCTGTTTCTGGACCTGCCTAAG GGCCTGAATTTTCTGCTCCT
chr12 chr12 57898026 59195061 56M59195042F20M                                                                                                                                                                                                                                                                     GCCGATCATGCCCTGTGGTGCAGTCGAGCCAGCACCTGTTTCTGGACCTGCCTAAG GGCCTGAATTTTCTGCTCCT
chr12 chr12 57898026 59195061 56M59195042F20M                                                                                                                                                                                                                                                                     GCCGATCACGCCCTGTGGTGCAGTCGAGCCAGCACCTGTTTCTGGACCTGCCTAAG GGCCTGAATTTTCTGCTCCT
chr12 chr12 57898026 59195061 56M59195042F20M                                                                                                                                                                                                                                                                     GCCGATCATGCCCTGTGGTGCAGTCGAGCCAGCACCTGTTTCTGGACCTGCCTAAG GGCCTGAATTTTCTGCTCCT
chr12 chr12 57898027 59195062 55M59195042F21M                                                                                                                                                                                                                                                                      CCGATCATGCCCTGTGGTGCAGTCGAGCCAGCACCTGTTTCTGGACCTGCCTAAG GGCCTGAATTTTCTGCTCCTG
chr12 chr12 57898027 59195062 55M59195042F21M                                                                                                                                                                                                                                                                      CCGATCATGCCCTGTGGTGCAGTCGAGCCAGCACCTGTTTCTGGACCTGCCTAAG GGCCTGAATTTTCTGCTCCTG
chr12 chr12 57898027 59195062 55M59195042F21M                                                                                                                                                                                                                                                                      CCGATCATGCCCTGTGGTGCAGTCGAGCCAGCACCTGTTTCTGGACCTGCCTAAG GGCCTGAATTTTCTGCTCCTG
chr12 chr12 57898027 59195062 55M59195042F21M                                                                                                                                                                                                                                                                      CCGATCATGCCCTGTGGTGCAGTCGAGCCAGCACCTGTTTCTGGACCTGCCTAAG GGCCTGAATTTTCTGCTCCTG
chr12 chr12 57898028 59195063 54M59195042F22M                                                                                                                                                                                                                                                                       CGATCATGCCCTGTGGTGCAGTCGAGCCAGCACCTGTTTCTGGACCTGCCTAAG GGCCTGAATTTTCTGCTCCTGA
chr12 chr12 57898028 59195063 54M59195042F22M                                                                                                                                                                                                                                                                       CGATCATGCCCTGTGGTGCAGTCGAGCCAGCACCTGTTTCTGGACCTGCCTAAG GGCCTGAATTTTCTGCTCCTGA
chr12 chr12 57898028 59195063 54M59195042F22M                                                                                                                                                                                                                                                                       CGATCATGCCCTGTGGTGCAGTCGAGCCAGCACCTGTTTCTGGACCTGCCTAAG GGCCTGAATTTTCTGCTCCTGA
chr12 chr12 57898028 59195063 54M59195042F22M                                                                                                                                                                                                                                                                       CGATCATGCCCTGTGGTGCAGTCGAGCCAGCACCTGTTTCTGGACCTGCCTAAG GGCCTGAATTTTCTGCTCCTGA
chr12 chr12 57898028 59195063 54M59195042F22M                                                                                                                                                                                                                                                                       CGATCATGCCCTGTGGTGCAGTCGAGCCAGCACCTGTTTCTGGACCTGCCTAAG GGCCTGAATTTTCTGCTCCTGA
chr12 chr12 57898028 59195063 54M59195042F22M                                                                                                                                                                                                                                                                       CGATCATGCCCTGTGGTGCAGTCGAGCCAGCACCTGTTTCTGGACCTGCCTAAG GGCCTGAATTTTCTGCTCCTGA
chr12 chr12 57898028 59195063 54M59195042F22M                                                                                                                                                                                                                                                                       CGATCATGCCCTGTCGTGCAGTCGAGCCAGCACCTGTTTCTGGACCTGCCTAAG GGCCTGAATTTTCTGCTCCTGA
chr12 chr12 57898028 59195063 54M59195042F22M                                                                                                                                                                                                                                                                       CGATCATGCCCTGTGGTGCAGTCGAGCCAGCACCTGTTTCTGGACCTGCCTAAG GGCCTGAATTTTCTGCTCCTGA
chr12 chr12 57898028 59195063 54M59195042F22M                                                                                                                                                                                                                                                                       CGATCATGCCCTGTGGTGCAGTCGAGCCAGCACCTGTTTCTGGACCTGCCTAAG GGCCTGAATTTTCTGCTCCTGA
chr12 chr12 57898028 59195063 54M59195042F22M                                                                                                                                                                                                                                                                       CGATCATGCCCTGTGGGGCAGTCGAGCCAGCACCTGTTTCTGGACCTGCCTAAG GGCCTGAATTTTCTGCTCCTGA
chr12 chr12 57898028 59195063 54M59195042F22M                                                                                                                                                                                                                                                                       CGATCATGCCCTGTGGTGCAGTCGAGCCAGCACCTGTTTCTGGACCTGCCTAAG GGCCTGAATTTTCTGCTCCTGA
chr12 chr12 57898028 59195063 54M59195042F22M                                                                                                                                                                                                                                                                       CGATCATGCCCTGTGGTGCAGTCGAGCCAGCACCTGTTTCTGGACCTGCCTAAG GGCCTGAATTTTCTGCTCCTGA
chr12 chr12 57898028 59195063 54M59195042F22M                                                                                                                                                                                                                                                                       CGATCATGCCCTGTGGTGCAGTCGAGCCAGCACCTGTTTCTGGACCTGCCTAAG GGCCTGAATTTTCTGCTCCTGA
chr12 chr12 57898028 59195063 54M59195042F22M                                                                                                                                                                                                                                                                       CGATCATGCCCTGTGGTGCAGTCGAGCCAGCACCTGTTTCTGGACCTGCCTAAG GGCCTGAATTTTCTGCTCCTGA
chr12 chr12 57898028 59195063 54M59195042F22M                                                                                                                                                                                                                                                                       CGATCATGCCCTGTGGTGCAGTCGAGCCAGCACCTGTTTCTGGACCTGCCTAAG GGCCTGAATTTTCTGCTCCTGA
chr12 chr12 57898028 59195063 54M59195042F22M                                                                                                                                                                                                                                                                       CGATCATGCCCTGTGGTGCAGTCGAGCCAGCACCTGTTTCTGGACCTGCCTAAG GGCCTGAATTTTCTGCTCCTGA
chr12 chr12 57898028 59195063 54M59195042F22M                                                                                                                                                                                                                                                                       CGATCATGCCCTGTGGTGCAGTCGAGCCAGCACCTGTTTCTGGACCTGCCTAAG GGCCTGAATTTTCTGCTCCTGA
chr12 chr12 57898028 59195063 54M59195042F22M                                                                                                                                                                                                                                                                       CGATCATGCCCTGTGGTGCAGTCGAGCCAGCACCTGTTTCTGGACCTGCCTAAG GGCCTGAATTTTCTGCTCCTGA
chr12 chr12 57898028 59195063 54M59195042F22M                                                                                                                                                                                                                                                                       CGATCATGCCCTGTGGTGCAGTCGAGCCAGCACCTGTTTCTGGACCTGCCTAAA GGCCTGAATTTTATGCTCCTGA
chr12 chr12 57898028 59195063 54M59195042F22M                                                                                                                                                                                                                                                                       CGATCATGCCCTGTGGTGCAGTCGAGCCAGCACCTGTTTCTGGACCTGCCTAAG GGCCTGAATTTTCTGCTCCTGA
chr12 chr12 57898028 59195063 54M59195042F22M                                                                                                                                                                                                                                                                       CGATCATGCCCTGTGGTGCAGTCGAGCCAGCACCTGTTTCTGGACCTGCCTAAG GGCCTGAATTTTCTGCTCCTGA
chr12 chr12 57898028 59195063 54M59195042F22M                                                                                                                                                                                                                                                                       CGATCATGCCCTGTGGTGCAGTCGAGCCAGCACCTGTTTCTGGACCTGCCTAAG GGCCTGAATTTTCTGCTCCTGA
chr12 chr12 57898028 59195063 54M59195042F22M                                                                                                                                                                                                                                                                       CGATCATGCCCTGTGGTGCAGTCGAGCCAGCACCTGTTTCTGGACCTGCCTAAG GGCCTGAATTTTCTGCTCCTGA
chr12 chr12 57898028 59195063 54M59195042F22M                                                                                                                                                                                                                                                                       CGATCATGCCCTGTGGTGCAGTCGAGCCAGCACCTGTTTCTGGACCTGCCTAAG GGCCTGAATTTTCTGCTCCTGA
chr12 chr12 57898028 59195063 54M59195042F22M                                                                                                                                                                                                                                                                       CGATCATGCCCTGTGGTGCAGTCGAGCCAGCACCTGTTTCTGGACCTGCCTAAG GGCCTGAATTTTCTGCTCCTGA
chr12 chr12 57898028 59195063 54M59195042F22M                                                                                                                                                                                                                                                                       CGATCATGCCCTGTGGTGCAGTCGAGCCAGCACCTGTTTCTGGACCTGCCTAAG GGCCTGAATTTTCTGCTCCTGA
chr12 chr12 57898028 59195063 54M59195042F22M                                                                                                                                                                                                                                                                       CGATCATGCCCTGTGGTGCAGTCGAGCCAGCACCTGTTTCTGGACCTGCCTAAG GGCCTGAATTTTCTGCTCCTGA
chr12 chr12 57898028 59195063 54M59195042F22M                                                                                                                                                                                                                                                                       CGATCATGCCCTGTGGTGCAGTCGAGCCAGCACCTGTTTCTGGACCTGCCTAAG GGCCTGAATTTTCTGCTCCTGA
chr12 chr12 57898029 59195064 53M59195042F23M                                                                                                                                                                                                                                                                        GATCATGCCCTGTGGTGCAGTCGAGCCAGCACCTGTTTCTGGACCTGCCTAAG GGCCTGAATTTTCTGCTCCTGAA
chr12 chr12 57898029 59195064 53M59195042F23M                                                                                                                                                                                                                                                                        GATCATGCCCTGTGGTGCAGTCGAGCCAGCACCTGTTTCTGGACCTGCCTAAG GGCATGAATTTTCTGCTCCTGAA
chr12 chr12 57898029 59195064 53M59195042F23M                                                                                                                                                                                                                                                                        GATCATGCCCTGTGGTGCAGTCGAGCCAGCACCTGTTTCTGGACCTGCCTAAG GGCCTGAATTTTCTGCTCCTGAA
chr12 chr12 57898030 59195065 52M59195042F24M                                                                                                                                                                                                                                                                         ATCATGCCCTGTGGTGCAGTCGAGCCAGCACCTGTTTCTGGACCTGCCTAAG GGCCTGAATTTTCTGCTCCTGAAT
chr12 chr12 57898030 59195065 52M59195042F24M                                                                                                                                                                                                                                                                         ATCATGCCCTGTGGTGCAGTCGAGCCAGCACCTGTTTCTGGACCTGCCTAAG GGCCTGAATTTTCTGCTCCTGAAT
chr12 chr12 57898030 59195065 52M59195042F24M                                                                                                                                                                                                                                                                         ATCATGCCCTGTGGTGCAGTCGAGCCAGCACCTGTTTCTGGACCTGCCTAAG GGCCTGAATTTTCTGCTCCTGAAT
chr12 chr12 57898030 59195065 52M59195042F24M                                                                                                                                                                                                                                                                         ATCATGCCCTGTGGTGCAGTCGAGCCAGCACCTGTTTCTGGACCTGCCTAAG GGCCTGAATTTTCTGCTCCTGAAT
chr12 chr12 57898030 59195065 52M59195042F24M                                                                                                                                                                                                                                                                         ATCATGCCCTGTGGTGCAGTCGAGCCAGCACCTGTTTCTGGACCTGCCTAAG GGCCTGAATTTTCTGCTCCTGAAT
chr12 chr12 57898031 59195066 51M59195042F25M                                                                                                                                                                                                                                                                          TCATGCCCTGTGGTGCAGTCGAGCCAGCACCTGTTTCTGGACCTGCCTAAG GGCCTGAATTTTCTGCTCCTGAATT
chr12 chr12 57898031 59195066 51M59195042F25M                                                                                                                                                                                                                                                                          TCATGCCCTGTGGTGCAGTCGAGCCAGCACCTGTTTCTGGACCTGCCTAAG GGCCTGAATTTTCTGCTCCTGAATT
chr12 chr12 57898031 59195066 51M59195042F25M                                                                                                                                                                                                                                                                          TCATGCCCTGTGGTGCAGCCGAGCCACCACCTGTTTCTGGACCTGCCTAAG GGCCTGAATTTTCTGCTCCTGAATT
chr12 chr12 57898032 59195067 50M59195042F26M                                                                                                                                                                                                                                                                           CATGCCCTGTGGTGCAGTCGAGCCAGCACCTGTTTCTGGACCTGCCTAAG GGCCTGAATTTTCTGCTCCTGAATTC
chr12 chr12 57898032 59195067 50M59195042F26M                                                                                                                                                                                                                                                                           CATGCCCTGTGGTGCAGTCGAGCCAGCACCTGTTTCTGGACCTGCCTAAG GGCCTGAATTTTCTGCTCCTGAATTC
chr12 chr12 57898032 59195067 50M59195042F26M                                                                                                                                                                                                                                                                           CATGCCCTGTGGTGCAGTCGAGCCAGCACCTGTTTCTGGACCTGCCTAAG GGCCTGAATTTTCTGCTCCTGAATTC
chr12 chr12 57898033 59195068 49M59195042F27M                                                                                                                                                                                                                                                                            ATGCCCTGTGGTGCAGTCGAGCCAGCACCTGTTTCTGGACCTGCCTAAG GGCCTGAATTTTCTGCTCCTGAATTCA
chr12 chr12 57898034 59195069 48M59195042F28M                                                                                                                                                                                                                                                                             TGCCCTGTGGTGCAGTCGAGCCAGCACCTGTTTCTGGACCTGCCTAAG GGCCTGAATTTTCTGCTCCTGAATTCAA
chr12 chr12 57898034 59195069 48M59195042F28M                                                                                                                                                                                                                                                                             TCCCCTGTGGTGCATTCGAGCCAGCACCTGTTTCTGGACCTGCCTAAG GGCCTGAATTTTCTGCTCCTGAATTCAA
chr12 chr12 57898034 59195069 48M59195042F28M                                                                                                                                                                                                                                                                             TGCCCTGTGGTGCAGTCGAGCCAGCACCTGTTTCTGGACCTGCCTAAG GGCCTGAATTTTCTGCTCCTGAATTCAA
chr12 chr12 57898035 59195070 47M59195042F29M                                                                                                                                                                                                                                                                              GCCCTGTGGTGCAGTCGAGCCAGCACCTGTTTCTGGACCTGCCTAAG GGCCTGAATTTTCTGCTCCTGAATTCAAA
chr12 chr12 57898035 59195070 47M59195042F29M                                                                                                                                                                                                                                                                              GCCCTGTGGTGCAGTCGAGCCAGCACCTGTTTCTGGACCTGCCTAAG GGCCTGAATTTTCTGCTCCTGAATTCAAA
chr12 chr12 57898035 59195070 47M59195042F29M                                                                                                                                                                                                                                                                              GCCCTGTGGTGCAGTCGAGCCAGCACCTGTTTCTGGACCTGCCTAAG GGCCTGAATTTTCTGCTCCTGAATTCAAA
chr12 chr12 57898035 59195070 47M59195042F29M                                                                                                                                                                                                                                                                              GCCCTGTGGTGCAGTCGAGCCAGCACCTGTTTCTGGACCTGCCTAAG GGCCTGAATTTTCTGCTCCTGAATTCAAA
chr12 chr12 57898035 59195070 47M59195042F29M                                                                                                                                                                                                                                                                              GCCCTGTGGTGCAGTCGAGCCAGCACCTGTTTCTGGACCTGCCTAAG GGCCTGAATTTTCTGCTCCTGAATTCAAA
chr12 chr12 57898035 59195070 47M59195042F29M                                                                                                                                                                                                                                                                              GCCCTGTGGTGCAGTCGAGCCAGCACCTGTTTCTGGACCTGCCTAAG GGCCTGAATTTTCTGCTCCTGAATTCAAA
chr12 chr12 57898035 59195070 47M59195042F29M                                                                                                                                                                                                                                                                              GCCCTGTGGTGCAGTCGAGCCAGCACCTGTTTCTGGACCTGCCTAAG GGCCTGAATTTTCTGCTCCTGAATTCAAA
chr12 chr12 57898035 59195070 47M59195042F29M                                                                                                                                                                                                                                                                              GCCCTGTGGTGCAGTCGAGCCAGCACCTGTTTCTGGACCTGCCTAAG GGCCTGAATTTTCTGCTCCTGAATTCAAA
chr12 chr12 57898035 59195070 47M59195042F29M                                                                                                                                                                                                                                                                              GCCCTGTGGTGCAGTCGAGCCAGCACCTGTTTCTGGACCTGCCTAAG GGCCTGAATTTTCTGCTCCTGAATTCAAA
chr12 chr12 57898035 59195070 47M59195042F29M                                                                                                                                                                                                                                                                              GCCCGGTGGTGCAGTCGAGCCAGCACCTGTTTCTGGACCTGCCTAAG GGCCTGAATTTTCTGCTCCTGAATTCAAA
chr12 chr12 57898035 59195070 47M59195042F29M                                                                                                                                                                                                                                                                              CCCCTGTGGTGCAGTCGAGCCAGCACCTGTTTCTGGACCTGCCTAAG GGCCTGAATTTTCTGCTCCTGAATTCAAA
chr12 chr12 57898035 59195070 47M59195042F29M                                                                                                                                                                                                                                                                              GCCCTGTGGTGCAGTCGAGCCAGCACCTGTTTCTGGACCTGCCTAAG GGCCTGAATTTTCTGCTCCTGAATTCAAA
chr12 chr12 57898035 59195070 47M59195042F29M                                                                                                                                                                                                                                                                              GCCCTGTGGTGCAGTCGAGCCAGCACCTGTTTCTGGACCTGCCTAAG GGCCTGAATTTTCTGCTCCTGAATTCAAA
chr12 chr12 57898035 59195070 47M59195042F29M                                                                                                                                                                                                                                                                              CCCCTGTGGTGCAGTCGAGCCAGCACCTGTTTCTGGACCTGCCTAAG GGCCTGAATTTTCTGCTCCTGAATTCAAA
chr12 chr12 57898035 59195070 47M59195042F29M                                                                                                                                                                                                                                                                              GCCCTGTGGTGCAGTCGAGCCAGCACCTGTTTCTGGACCTGCCTAAG GGCCTGAATTTTCTGCTCCTGAATTCAAA
chr12 chr12 57898035 59195070 47M59195042F29M                                                                                                                                                                                                                                                                              GCCCTGTGGTGCAGTCGAGCCAGCACCTGTTTCTGGACCTGCCTAAG GGCCTGAATTTTCTGCTCCTGAATTCAAA
chr12 chr12 57898035 59195070 47M59195042F29M                                                                                                                                                                                                                                                                              GCCCTGTGGTGCAGTCGAGCCAGCACCTGTTTCTGGACCTGCCTAAG GGCCTGAATTTTCTGCTCCTGAATTCAAA
chr12 chr12 57898035 59195070 47M59195042F29M                                                                                                                                                                                                                                                                              GCCCTGTGGTGCAGTCGAGCCAGCACCTGTTTCTGGACCTGCCTAAG GGCCTGAATTTTCTGCTCCTGAATTCAAA
chr12 chr12 57898035 59195070 47M59195042F29M                                                                                                                                                                                                                                                                              GCCCTGTGGTGCAGTCGAGCCAGCACCTGTTTCTGGACCTGCCTAAG GGCCTGAATTTTCTGCTCCTGAATTCAAA
chr12 chr12 57898035 59195070 47M59195042F29M                                                                                                                                                                                                                                                                              GCCCTGTGGTGCAGTCGAGCCAGCACCTGTTTCTGGACCTGCCTAAG GGCCTGAATTTTCTGCTCCTGAATTCAAA
chr12 chr12 57898035 59195070 47M59195042F29M                                                                                                                                                                                                                                                                              GCCCTGTGGTGCAGTCGAGCCAGCTCCTGTTTCTGGACCTGCCTAAG GGCCTGAATTTTCTGCTCCTGAATTCAAA
chr12 chr12 57898035 59195070 47M59195042F29M                                                                                                                                                                                                                                                                              GCCCTGTGGTGCAGTCGAGCCAGCACCTGTTTCTGGACCTGCCTAAG GGCCTGAATTTTCTGCTCCTGAATTCAAA
chr12 chr12 57898036 59196713 46M59195042F29M1642N1M                                                                                                                                                                                                                                                                        CCCTGTGGTGCAGTCGAGCCAGCACCTGTTTCTGGACCTGCCTAAG GGCCTGAATTTTCTGCTCCTGAATTCAAA|||||||||||||||||||||||||||||||||||||||||||||||||||||||||||||||||||||||||||||||||||||||||||||||||||||||||||||||||||||||||||||||||||||||||||||||||||||||||||||||||||||||||||||||||||||||||||||||||||||||||||||||||||||||||||||||||||||||||||||||||||||||||||||||||||||||||||||
chr12 chr12 57898036 59199723 46M59195042F29M4652N1M                                                                                                                                                                                                                                                                        CCCTGTGGTGCAGTCGAGCCAGCACCTGTTTCTGGACCTGCCTAAG GGCCTGAATTTTCTGCTCCTGAATTCAAA|||||||||||||||||||||||||||||||||||||||||||||||||||||||||||||||||||||||||||||||||||||||||||||||||||||||||||||||||||||||||||||||||||||||||||||||||||||||||||||||||||||||||||||||||||||||||||||||||||||||||||||||||||||||||||||||||||||||||||||||||||||||||||||||||||||||||||||
chr12 chr12 57898036 59196608 46M59195042F29M1537N1M                                                                                                                                                                                                                                                                        CCCTGTGGTGCAGTCGAGCCAGCACCTGTTTCTGGACCTGCCTAAG GGCCTGAATTTTCTGCTCCTGAATTCAAA|||||||||||||||||||||||||||||||||||||||||||||||||||||||||||||||||||||||||||||||||||||||||||||||||||||||||||||||||||||||||||||||||||||||||||||||||||||||||||||||||||||||||||||||||||||||||||||||||||||||||||||||||||||||||||||||||||||||||||||||||||||||||||||||||||||||||||||
chr12 chr12 57898036 59196608 46M59195042F29M1537N1M                                                                                                                                                                                                                                                                        CCCTGTGGTGCAGTCGAGCCAGCACCTGTTTCTGGACCTGCCTAAG GGCCTGAATTTTCTGCTCCTGAATTCAAA|||||||||||||||||||||||||||||||||||||||||||||||||||||||||||||||||||||||||||||||||||||||||||||||||||||||||||||||||||||||||||||||||||||||||||||||||||||||||||||||||||||||||||||||||||||||||||||||||||||||||||||||||||||||||||||||||||||||||||||||||||||||||||||||||||||||||||||
chr12 chr12 57898036 59196713 46M59195042F29M1642N1M                                                                                                                                                                                                                                                                        CCCTGTGGTGCAGTCGAGCCAGCACCTGTTTCTGGACCTGCCTAAG GGCCTGAATTTTCTGCTCCTGAATTCAAA|||||||||||||||||||||||||||||||||||||||||||||||||||||||||||||||||||||||||||||||||||||||||||||||||||||||||||||||||||||||||||||||||||||||||||||||||||||||||||||||||||||||||||||||||||||||||||||||||||||||||||||||||||||||||||||||||||||||||||||||||||||||||||||||||||||||||||||
chr12 chr12 57898036 59199723 46M59195042F29M4652N1M                                                                                                                                                                                                                                                                        CCCTGTGGTGCAGTCGAGCCAGCACCTGTTTCTGGACCTGCCTAAG GGCCTGAATTTTCTGCTCCTGAATTCAAA|||||||||||||||||||||||||||||||||||||||||||||||||||||||||||||||||||||||||||||||||||||||||||||||||||||||||||||||||||||||||||||||||||||||||||||||||||||||||||||||||||||||||||||||||||||||||||||||||||||||||||||||||||||||||||||||||||||||||||||||||||||||||||||||||||||||||||||
chr12 chr12 57898036 59196713 46M59195042F29M1642N1M                                                                                                                                                                                                                                                                        CCCTTTGGTGCAGTCGAGCCAGCACCTGTTTCTGGACCTGGCTAAG GGCCTGAATTTTCTGCTCCTGAATTCAAA|||||||||||||||||||||||||||||||||||||||||||||||||||||||||||||||||||||||||||||||||||||||||||||||||||||||||||||||||||||||||||||||||||||||||||||||||||||||||||||||||||||||||||||||||||||||||||||||||||||||||||||||||||||||||||||||||||||||||||||||||||||||||||||||||||||||||||||
chr12 chr12 57898036 59199723 46M59195042F29M4652N1M                                                                                                                                                                                                                                                                        CCCTTTGGTGCAGTCGAGCCAGCACCTGTTTCTGGACCTGGCTAAG GGCCTGAATTTTCTGCTCCTGAATTCAAA|||||||||||||||||||||||||||||||||||||||||||||||||||||||||||||||||||||||||||||||||||||||||||||||||||||||||||||||||||||||||||||||||||||||||||||||||||||||||||||||||||||||||||||||||||||||||||||||||||||||||||||||||||||||||||||||||||||||||||||||||||||||||||||||||||||||||||||
chr12 chr12 57898036 59196713 46M59195042F29M1642N1M                                                                                                                                                                                                                                                                        CCATGTGGTGCATTCGAGCCAGCACCTGTTTCTGGACCTGCCTAAG GGCCTGAATTTTCTGCTCCTGAATTCAAA|||||||||||||||||||||||||||||||||||||||||||||||||||||||||||||||||||||||||||||||||||||||||||||||||||||||||||||||||||||||||||||||||||||||||||||||||||||||||||||||||||||||||||||||||||||||||||||||||||||||||||||||||||||||||||||||||||||||||||||||||||||||||||||||||||||||||||||
chr12 chr12 57898036 59199723 46M59195042F29M4652N1M                                                                                                                                                                                                                                                                        CCATGTGGTGCATTCGAGCCAGCACCTGTTTCTGGACCTGCCTAAG GGCCTGAATTTTCTGCTCCTGAATTCAAA|||||||||||||||||||||||||||||||||||||||||||||||||||||||||||||||||||||||||||||||||||||||||||||||||||||||||||||||||||||||||||||||||||||||||||||||||||||||||||||||||||||||||||||||||||||||||||||||||||||||||||||||||||||||||||||||||||||||||||||||||||||||||||||||||||||||||||||
chr12 chr12 57898037 59196609 45M59195042F29M1537N2M                                                                                                                                                                                                                                                                         CCTGTGGTGCAGTCGAGCCAGCACCTGTTTCTGGACCTGCCTAAG GGCCTGAATTTTCTGCTCCTGAATTCAAA|||||||||||||||||||||||||||||||||||||||||||||||||||||||||||||||||||||||||||||||||||||||||||||||||||||||||||||||||||||||||||||||||||||||||||||||||||||||||||||||||||||||||||||||||||||||||||||||||||||||||||||||||||||||||||||||||||||||||||||||||||||||||||||||||||||||||||||
chr12 chr12 57898038 59196610 44M59195042F29M1537N3M                                                                                                                                                                                                                                                                          CTGTGGTGCAGTCGAGCCAGCACCTGTTTCTGGACCTGCCTAAG GGCCTGAATTTTCTGCTCCTGAATTCAAA|||||||||||||||||||||||||||||||||||||||||||||||||||||||||||||||||||||||||||||||||||||||||||||||||||||||||||||||||||||||||||||||||||||||||||||||||||||||||||||||||||||||||||||||||||||||||||||||||||||||||||||||||||||||||||||||||||||||||||||||||||||||||||||||||||||||||||||
chr12 chr12 57898038 59196610 44M59195042F29M1537N3M                                                                                                                                                                                                                                                                          CTGTGGTGCAGTCGAGCCAGCACCTGTTTCTGGACCTGCCTAAG GGCCTGAATTTTCTGCTCCTGAATTCAAA|||||||||||||||||||||||||||||||||||||||||||||||||||||||||||||||||||||||||||||||||||||||||||||||||||||||||||||||||||||||||||||||||||||||||||||||||||||||||||||||||||||||||||||||||||||||||||||||||||||||||||||||||||||||||||||||||||||||||||||||||||||||||||||||||||||||||||||
chr12 chr12 57898038 59206198 44M59195042F29M11125N3M                                                                                                                                                                                                                                                                         CTGTGGTGCAGTCGAGCCAGCACCTGTTTCTGGACCTGCCTAAG GGCCTGAATTTTCTGCTCCTGAATTCAAA|||||||||||||||||||||||||||||||||||||||||||||||||||||||||||||||||||||||||||||||||||||||||||||||||||||||||||||||||||||||||||||||||||||||||||||||||||||||||||||||||||||||||||||||||||||||||||||||||||||||||||||||||||||||||||||||||||||||||||||||||||||||||||||||||||||||||||||
chr12 chr12 57898038 59199725 44M59195042F29M4652N3M                                                                                                                                                                                                                                                                          CTGTGGTGCAGTCGAGCCAGCACCTGTTTCTGGACCTGCCTAAG GGCCTGAATTTTCTGCTCCTGAATTCAAA|||||||||||||||||||||||||||||||||||||||||||||||||||||||||||||||||||||||||||||||||||||||||||||||||||||||||||||||||||||||||||||||||||||||||||||||||||||||||||||||||||||||||||||||||||||||||||||||||||||||||||||||||||||||||||||||||||||||||||||||||||||||||||||||||||||||||||||
chr12 chr12 57898038 59195073 44M59195042F32M                                                                                                                                                                                                                                                                                 CTGTGGTGCAGTCGAGCCAGCACCTGTTTCTGGACCTGCCTAAG GGCCTGAATTTTCTGCTCCTGAATTCAAAGTA
chr12 chr12 57898038 59196610 44M59195042F29M1537N3M                                                                                                                                                                                                                                                                          CTGTGGTGCAGTCGAGCCAGCACCTGTTTCTGGACCTGCCTAAG GGCCTGAATTTTCTGCTCCTGAATTCAAA|||||||||||||||||||||||||||||||||||||||||||||||||||||||||||||||||||||||||||||||||||||||||||||||||||||||||||||||||||||||||||||||||||||||||||||||||||||||||||||||||||||||||||||||||||||||||||||||||||||||||||||||||||||||||||||||||||||||||||||||||||||||||||||||||||||||||||||
chr12 chr12 57898038 59196610 44M59195042F29M1537N3M                                                                                                                                                                                                                                                                          CTGTGGTGCAGTCGAGCCAGCACCTGTTTCTGGACCTGCCTAAG GGCCTGAATTTTCTGCTCCTGAATTCAAA|||||||||||||||||||||||||||||||||||||||||||||||||||||||||||||||||||||||||||||||||||||||||||||||||||||||||||||||||||||||||||||||||||||||||||||||||||||||||||||||||||||||||||||||||||||||||||||||||||||||||||||||||||||||||||||||||||||||||||||||||||||||||||||||||||||||||||||
chr12 chr12 57898038 59196610 44M59195042F29M1537N3M                                                                                                                                                                                                                                                                          CTGTGGTGCAGTCGANCCAGCACCTGTTTCTGGACCTGCCTAAG GGCCTGAATTTTCTACTCCTGAATTCAAA|||||||||||||||||||||||||||||||||||||||||||||||||||||||||||||||||||||||||||||||||||||||||||||||||||||||||||||||||||||||||||||||||||||||||||||||||||||||||||||||||||||||||||||||||||||||||||||||||||||||||||||||||||||||||||||||||||||||||||||||||||||||||||||||||||||||||||||
chr12 chr12 57898038 59196610 44M59195042F29M1537N3M                                                                                                                                                                                                                                                                          CTGTGGTGCAGTCGAGCCAGCACCTGTTTCTGGACCTGCCTAAG GGCCTGAATTTTCTGCTCCTGAATTCAAA|||||||||||||||||||||||||||||||||||||||||||||||||||||||||||||||||||||||||||||||||||||||||||||||||||||||||||||||||||||||||||||||||||||||||||||||||||||||||||||||||||||||||||||||||||||||||||||||||||||||||||||||||||||||||||||||||||||||||||||||||||||||||||||||||||||||||||||
chr12 chr12 57898038 59196610 44M59195042F29M1537N3M                                                                                                                                                                                                                                                                          CTGTGGTGCAGTCGAGCCAGCACCTGTTTCTGGACCTGCCTAAG GGCCTGAATTTTCTGCTCCTGAATTCAAA|||||||||||||||||||||||||||||||||||||||||||||||||||||||||||||||||||||||||||||||||||||||||||||||||||||||||||||||||||||||||||||||||||||||||||||||||||||||||||||||||||||||||||||||||||||||||||||||||||||||||||||||||||||||||||||||||||||||||||||||||||||||||||||||||||||||||||||
chr12 chr12 57898038 59196610 44M59195042F29M1537N3M                                                                                                                                                                                                                                                                          CTGTGGTGCAGTCGAGCCAGCACCTGTTTCTGGACCTGCCTAAG GGCCTGAATTTTCTGCTCCTGAATTCAAA|||||||||||||||||||||||||||||||||||||||||||||||||||||||||||||||||||||||||||||||||||||||||||||||||||||||||||||||||||||||||||||||||||||||||||||||||||||||||||||||||||||||||||||||||||||||||||||||||||||||||||||||||||||||||||||||||||||||||||||||||||||||||||||||||||||||||||||
chr12 chr12 57898038 59196610 44M59195042F29M1537N3M                                                                                                                                                                                                                                                                          CTGTGGTGCAGTCGAGCCAGCACCTGTTTCTGGACCTGCCTAAG GGCCTGAATTTTCTGCTCCTGAATTCAAA|||||||||||||||||||||||||||||||||||||||||||||||||||||||||||||||||||||||||||||||||||||||||||||||||||||||||||||||||||||||||||||||||||||||||||||||||||||||||||||||||||||||||||||||||||||||||||||||||||||||||||||||||||||||||||||||||||||||||||||||||||||||||||||||||||||||||||||
chr12 chr12 57898038 59196610 44M59195042F29M1537N3M                                                                                                                                                                                                                                                                          CTGTGGTGCAGTCGAGCCAGCACCTGTTTCTGGACCTGCCTAAG GGCCTGAATTTTCTGCTCCTGAATTCAAA|||||||||||||||||||||||||||||||||||||||||||||||||||||||||||||||||||||||||||||||||||||||||||||||||||||||||||||||||||||||||||||||||||||||||||||||||||||||||||||||||||||||||||||||||||||||||||||||||||||||||||||||||||||||||||||||||||||||||||||||||||||||||||||||||||||||||||||
chr12 chr12 57898038 59196610 44M59195042F29M1537N3M                                                                                                                                                                                                                                                                          CTGTGGTGCAGTCGAGCCAGCACCTGTTTCTGGACCTGCCTAAG GGCCTGAATTTTCTGCTCCTGAATTCAAA|||||||||||||||||||||||||||||||||||||||||||||||||||||||||||||||||||||||||||||||||||||||||||||||||||||||||||||||||||||||||||||||||||||||||||||||||||||||||||||||||||||||||||||||||||||||||||||||||||||||||||||||||||||||||||||||||||||||||||||||||||||||||||||||||||||||||||||
chr12 chr12 57898038 59199725 44M59195042F29M4652N3M                                                                                                                                                                                                                                                                          CTGTGGTGCAGTCGAGCCAGCACCTGTTTCTGGACCTGCCTAAG GGCCTGAATTTTCTGCTCCTGAATTCAAA|||||||||||||||||||||||||||||||||||||||||||||||||||||||||||||||||||||||||||||||||||||||||||||||||||||||||||||||||||||||||||||||||||||||||||||||||||||||||||||||||||||||||||||||||||||||||||||||||||||||||||||||||||||||||||||||||||||||||||||||||||||||||||||||||||||||||||||
chr12 chr12 57898038 59199725 44M59195042F29M4652N3M                                                                                                                                                                                                                                                                          CTGGGGTGCAGTCGAGCCAGCACCTGTTTCTGGACCTGCCTAAG GGCCTGAATTTTCTGCTCCTGAATTCAAA|||||||||||||||||||||||||||||||||||||||||||||||||||||||||||||||||||||||||||||||||||||||||||||||||||||||||||||||||||||||||||||||||||||||||||||||||||||||||||||||||||||||||||||||||||||||||||||||||||||||||||||||||||||||||||||||||||||||||||||||||||||||||||||||||||||||||||||
chr12 chr12 57898038 59199725 44M59195042F29M4652N3M                                                                                                                                                                                                                                                                          CTGTGGTGCAGTCGAGCCAGCACCTGTTTCTGGACCTGCCTAAG GGCCTGAATTTTCTGCTCCTGAATTCAAA|||||||||||||||||||||||||||||||||||||||||||||||||||||||||||||||||||||||||||||||||||||||||||||||||||||||||||||||||||||||||||||||||||||||||||||||||||||||||||||||||||||||||||||||||||||||||||||||||||||||||||||||||||||||||||||||||||||||||||||||||||||||||||||||||||||||||||||
chr12 chr12 57898038 59199725 44M59195042F29M4652N3M                                                                                                                                                                                                                                                                          CTGTGGTGCAGTCGAGCCAGCACCTGTTTCTGGACCTGCCTAAG GGCCTGAATTTTCTGCTCCTGAATTCAAA|||||||||||||||||||||||||||||||||||||||||||||||||||||||||||||||||||||||||||||||||||||||||||||||||||||||||||||||||||||||||||||||||||||||||||||||||||||||||||||||||||||||||||||||||||||||||||||||||||||||||||||||||||||||||||||||||||||||||||||||||||||||||||||||||||||||||||||
chr12 chr12 57898038 59196610 44M59195042F29M1537N3M                                                                                                                                                                                                                                                                          CTGTGGTGCAGTCGAGCCAGCACCTGTTTCTGGACCTGCCTAAG GGCCTGAATTTTCTGCTCCTGAATTCAAA|||||||||||||||||||||||||||||||||||||||||||||||||||||||||||||||||||||||||||||||||||||||||||||||||||||||||||||||||||||||||||||||||||||||||||||||||||||||||||||||||||||||||||||||||||||||||||||||||||||||||||||||||||||||||||||||||||||||||||||||||||||||||||||||||||||||||||||
chr12 chr12 57898038 59199725 44M59195042F29M4652N3M                                                                                                                                                                                                                                                                          CTGTGAGGCAGTCGAGCCAGCACCTGTTTCTGGACCTGCCTAAG GGCCTGAATTTTCTGCTCCTGAATTCAAA|||||||||||||||||||||||||||||||||||||||||||||||||||||||||||||||||||||||||||||||||||||||||||||||||||||||||||||||||||||||||||||||||||||||||||||||||||||||||||||||||||||||||||||||||||||||||||||||||||||||||||||||||||||||||||||||||||||||||||||||||||||||||||||||||||||||||||||
chr12 chr12 57898038 59206198 44M59195042F29M11125N3M                                                                                                                                                                                                                                                                         CTGTGGCGCAGTCGAGCCAGCACCTGTTTCTGGACCTGCCTAAG GGCCTGAATTTTCTGCTCCTGAATTCAAA|||||||||||||||||||||||||||||||||||||||||||||||||||||||||||||||||||||||||||||||||||||||||||||||||||||||||||||||||||||||||||||||||||||||||||||||||||||||||||||||||||||||||||||||||||||||||||||||||||||||||||||||||||||||||||||||||||||||||||||||||||||||||||||||||||||||||||||
chr12 chr12 57898038 59199725 44M59195042F29M4652N3M                                                                                                                                                                                                                                                                          CTGTGGTGCAGTCGAGCCAGCACCTGTTTCTGGACCTGCCTAAG GGCCTGAATTTTCTGCTCCTGAATTCAAA|||||||||||||||||||||||||||||||||||||||||||||||||||||||||||||||||||||||||||||||||||||||||||||||||||||||||||||||||||||||||||||||||||||||||||||||||||||||||||||||||||||||||||||||||||||||||||||||||||||||||||||||||||||||||||||||||||||||||||||||||||||||||||||||||||||||||||||
chr12 chr12 57898038 59196610 44M59195042F29M1537N3M                                                                                                                                                                                                                                                                          CTGTGGTGCAGTCGAGCCAGCACCTGTTTCTGGACCTGCCTAAG GGCCTGAATTTTCTGCTCCTGAATTCAAA|||||||||||||||||||||||||||||||||||||||||||||||||||||||||||||||||||||||||||||||||||||||||||||||||||||||||||||||||||||||||||||||||||||||||||||||||||||||||||||||||||||||||||||||||||||||||||||||||||||||||||||||||||||||||||||||||||||||||||||||||||||||||||||||||||||||||||||
chr12 chr12 57898039 59196611 43M59195042F29M1537N4M                                                                                                                                                                                                                                                                           TGTGGTGCAGTCGAGCCAGCACCTGTTTCTGGACCTGCCTAAG GGCCTGAATTTTCTGCTCCTGAATTCAAA|||||||||||||||||||||||||||||||||||||||||||||||||||||||||||||||||||||||||||||||||||||||||||||||||||||||||||||||||||||||||||||||||||||||||||||||||||||||||||||||||||||||||||||||||||||||||||||||||||||||||||||||||||||||||||||||||||||||||||||||||||||||||||||||||||||||||||||
chr12 chr12 57898040 59199727 42M59195042F29M4652N5M                                                                                                                                                                                                                                                                            GTGGTGCAGTCGAGCCAGCACCTGTTTCTGGACCTGCCTAAG GGCCTGAATTTTCTGCTCCTGAATTCAAA|||||||||||||||||||||||||||||||||||||||||||||||||||||||||||||||||||||||||||||||||||||||||||||||||||||||||||||||||||||||||||||||||||||||||||||||||||||||||||||||||||||||||||||||||||||||||||||||||||||||||||||||||||||||||||||||||||||||||||||||||||||||||||||||||||||||||||||
chr12 chr12 57898040 59196612 42M59195042F29M1537N5M                                                                                                                                                                                                                                                                            GTGGTGCAGTCGAGCCAGCACCTGTTTCTGGACCTGCCTAAG GGCCTGAATTTTCTGCTCCTGAATTCAAA|||||||||||||||||||||||||||||||||||||||||||||||||||||||||||||||||||||||||||||||||||||||||||||||||||||||||||||||||||||||||||||||||||||||||||||||||||||||||||||||||||||||||||||||||||||||||||||||||||||||||||||||||||||||||||||||||||||||||||||||||||||||||||||||||||||||||||||
chr12 chr12 57898040 59196612 42M59195042F29M1537N5M                                                                                                                                                                                                                                                                            GTGGTGCAGTCGAGCCAGCACCTGTTTCTGGACCTGCCTAAG GGCCTGAATTTTCTGCTCCTGAATTCAAA|||||||||||||||||||||||||||||||||||||||||||||||||||||||||||||||||||||||||||||||||||||||||||||||||||||||||||||||||||||||||||||||||||||||||||||||||||||||||||||||||||||||||||||||||||||||||||||||||||||||||||||||||||||||||||||||||||||||||||||||||||||||||||||||||||||||||||||
chr12 chr12 57898040 59199727 42M59195042F29M4652N5M                                                                                                                                                                                                                                                                            GTGGTGCAGTCGAGCCAGCACCTGTTTCTGGACCTGCCTAAG GGCCTGAATTTTCTGCTCCTGAATTCAAA|||||||||||||||||||||||||||||||||||||||||||||||||||||||||||||||||||||||||||||||||||||||||||||||||||||||||||||||||||||||||||||||||||||||||||||||||||||||||||||||||||||||||||||||||||||||||||||||||||||||||||||||||||||||||||||||||||||||||||||||||||||||||||||||||||||||||||||
chr12 chr12 57898040 59199727 42M59195042F29M4652N5M                                                                                                                                                                                                                                                                            GTGGTGCAGTCGAGCCAGCACCTGTTTCTGGACCTGCCTAAG GGCCTGAATTTTCTGCTCCTGAATTCAAA|||||||||||||||||||||||||||||||||||||||||||||||||||||||||||||||||||||||||||||||||||||||||||||||||||||||||||||||||||||||||||||||||||||||||||||||||||||||||||||||||||||||||||||||||||||||||||||||||||||||||||||||||||||||||||||||||||||||||||||||||||||||||||||||||||||||||||||
chr12 chr12 57898042 59196614 40M59195042F29M1537N7M                                                                                                                                                                                                                                                                              GGTGCAGTCGAGCCAGCACCTGTTTCTGGACCTGCCTAAG GGCCTGAATTTTCTGCTCCTGAATTCAAA|||||||||||||||||||||||||||||||||||||||||||||||||||||||||||||||||||||||||||||||||||||||||||||||||||||||||||||||||||||||||||||||||||||||||||||||||||||||||||||||||||||||||||||||||||||||||||||||||||||||||||||||||||||||||||||||||||||||||||||||||||||||||||||||||||||||||||||
chr12 chr12 57898042 59196614 40M59195042F29M1537N7M                                                                                                                                                                                                                                                                              GGTGCAGTCGAGCCAGCACCTGTTTCTGGACCTGCCTAAG GGCCTGAATTTTCTGCTCCTGAATTCAAA|||||||||||||||||||||||||||||||||||||||||||||||||||||||||||||||||||||||||||||||||||||||||||||||||||||||||||||||||||||||||||||||||||||||||||||||||||||||||||||||||||||||||||||||||||||||||||||||||||||||||||||||||||||||||||||||||||||||||||||||||||||||||||||||||||||||||||||
chr12 chr12 57898043 59196615 39M59195042F29M1537N8M                                                                                                                                                                                                                                                                               GTGCAGTCGAGCCAGCACCTGTTTCTGGACCTGCCTAAG GGCCTGAATTTTCTGCTCCTGAATTCAAA|||||||||||||||||||||||||||||||||||||||||||||||||||||||||||||||||||||||||||||||||||||||||||||||||||||||||||||||||||||||||||||||||||||||||||||||||||||||||||||||||||||||||||||||||||||||||||||||||||||||||||||||||||||||||||||||||||||||||||||||||||||||||||||||||||||||||||||
chr12 chr12 57898043 59199730 39M59195042F29M4652N8M                                                                                                                                                                                                                                                                               GTGCAGTCGAGCCAGCACCTGTTTCTGGACCTGCCTAAG GGCCTGAATTTTCTGCTCCTGAATTCAAA|||||||||||||||||||||||||||||||||||||||||||||||||||||||||||||||||||||||||||||||||||||||||||||||||||||||||||||||||||||||||||||||||||||||||||||||||||||||||||||||||||||||||||||||||||||||||||||||||||||||||||||||||||||||||||||||||||||||||||||||||||||||||||||||||||||||||||||
chr12 chr12 57898043 59196615 39M59195042F29M1537N8M                                                                                                                                                                                                                                                                               GTGCAGTCGAGCCAGCACCTGTTTCTGGACCTGCCTAAG GGCCTGAATTTTCTGCTCCTGAATTCAAA|||||||||||||||||||||||||||||||||||||||||||||||||||||||||||||||||||||||||||||||||||||||||||||||||||||||||||||||||||||||||||||||||||||||||||||||||||||||||||||||||||||||||||||||||||||||||||||||||||||||||||||||||||||||||||||||||||||||||||||||||||||||||||||||||||||||||||||
chr12 chr12 57898043 59199730 39M59195042F29M4652N8M                                                                                                                                                                                                                                                                               GTGCAGTCGAGCCAGCACCTGTTTCTGGACCTGCCTAAG GGCCTGAATTTTCTGCTCCTGAATTCAAA|||||||||||||||||||||||||||||||||||||||||||||||||||||||||||||||||||||||||||||||||||||||||||||||||||||||||||||||||||||||||||||||||||||||||||||||||||||||||||||||||||||||||||||||||||||||||||||||||||||||||||||||||||||||||||||||||||||||||||||||||||||||||||||||||||||||||||||
chr12 chr12 57898043 59199730 39M59195042F29M4652N8M                                                                                                                                                                                                                                                                               GTGCAGTCGAGCCAGCACCTGTTTCTGGACCTGCCTAAG GGCCTGAATTTTCTGCTCCTGAATTCAAA|||||||||||||||||||||||||||||||||||||||||||||||||||||||||||||||||||||||||||||||||||||||||||||||||||||||||||||||||||||||||||||||||||||||||||||||||||||||||||||||||||||||||||||||||||||||||||||||||||||||||||||||||||||||||||||||||||||||||||||||||||||||||||||||||||||||||||||
chr12 chr12 57898043 59196720 39M59195042F29M1642N8M                                                                                                                                                                                                                                                                               GTGCAGTCGAGCCAGCACCTGGTTCTGGACCTGCCTAAG GGCCTGAATTTTCTGCTCCTGAATTCAAA|||||||||||||||||||||||||||||||||||||||||||||||||||||||||||||||||||||||||||||||||||||||||||||||||||||||||||||||||||||||||||||||||||||||||||||||||||||||||||||||||||||||||||||||||||||||||||||||||||||||||||||||||||||||||||||||||||||||||||||||||||||||||||||||||||||||||||||
chr12 chr12 57898043 59199730 39M59195042F29M4652N8M                                                                                                                                                                                                                                                                               GTGCAGTCGAGCCAGCACCTGTTTCTGGACCTGCCTAAG GGCCTGAATTTTCTGCTCCTGAATTCAAA|||||||||||||||||||||||||||||||||||||||||||||||||||||||||||||||||||||||||||||||||||||||||||||||||||||||||||||||||||||||||||||||||||||||||||||||||||||||||||||||||||||||||||||||||||||||||||||||||||||||||||||||||||||||||||||||||||||||||||||||||||||||||||||||||||||||||||||
chr12 chr12 57898043 59196615 39M59195042F29M1537N8M                                                                                                                                                                                                                                                                               GTGCAGTCGAGCCAGCACCTGTTTCTGGACCTGCCTAAG GGCCTGAATTTTCTGCTCCTGAATTCAAA|||||||||||||||||||||||||||||||||||||||||||||||||||||||||||||||||||||||||||||||||||||||||||||||||||||||||||||||||||||||||||||||||||||||||||||||||||||||||||||||||||||||||||||||||||||||||||||||||||||||||||||||||||||||||||||||||||||||||||||||||||||||||||||||||||||||||||||
chr12 chr12 57898043 59199730 39M59195042F29M4652N8M                                                                                                                                                                                                                                                                               GTGCAGTCGAGCCAGCACCTGTTTCTGGACCTGCCTAAG GGCCTGAATTTTCTGCTCCTGAATTCAAA|||||||||||||||||||||||||||||||||||||||||||||||||||||||||||||||||||||||||||||||||||||||||||||||||||||||||||||||||||||||||||||||||||||||||||||||||||||||||||||||||||||||||||||||||||||||||||||||||||||||||||||||||||||||||||||||||||||||||||||||||||||||||||||||||||||||||||||
chr12 chr12 57898043 59196615 39M59195042F29M1537N8M                                                                                                                                                                                                                                                                               GTGCAGTCGAGCCAGCACCTGTTTCTGGACCTGCCTAAG GGCCTGAATTTTCTGCTCCTGAATTCAAA|||||||||||||||||||||||||||||||||||||||||||||||||||||||||||||||||||||||||||||||||||||||||||||||||||||||||||||||||||||||||||||||||||||||||||||||||||||||||||||||||||||||||||||||||||||||||||||||||||||||||||||||||||||||||||||||||||||||||||||||||||||||||||||||||||||||||||||
chr12 chr12 57898043 59199730 39M59195042F29M4652N8M                                                                                                                                                                                                                                                                               GTGCAGTCGAGCCAGCACCTGTTTCTGGACCTGCCTAAG GGCCTGAATTTTCTGCTCCTGAATTCAAA|||||||||||||||||||||||||||||||||||||||||||||||||||||||||||||||||||||||||||||||||||||||||||||||||||||||||||||||||||||||||||||||||||||||||||||||||||||||||||||||||||||||||||||||||||||||||||||||||||||||||||||||||||||||||||||||||||||||||||||||||||||||||||||||||||||||||||||
chr12 chr12 57898043 59199730 39M59195042F29M4652N8M                                                                                                                                                                                                                                                                               GTGCAGTCGAGCCAGCACCTGTTTCTGGACCTGCCTAAG GGCCTGAATTTTCTGCTCCTGAATTCAAA|||||||||||||||||||||||||||||||||||||||||||||||||||||||||||||||||||||||||||||||||||||||||||||||||||||||||||||||||||||||||||||||||||||||||||||||||||||||||||||||||||||||||||||||||||||||||||||||||||||||||||||||||||||||||||||||||||||||||||||||||||||||||||||||||||||||||||||
chr12 chr12 57898043 59199730 39M59195042F29M4652N8M                                                                                                                                                                                                                                                                               GTGCAGTCGAGCCAGCACCTGTTTCTGGACCTGCCTAAG GGCCTGAATTTTCTGCTCCTGAATTCAAA|||||||||||||||||||||||||||||||||||||||||||||||||||||||||||||||||||||||||||||||||||||||||||||||||||||||||||||||||||||||||||||||||||||||||||||||||||||||||||||||||||||||||||||||||||||||||||||||||||||||||||||||||||||||||||||||||||||||||||||||||||||||||||||||||||||||||||||
chr12 chr12 57898043 59199730 39M59195042F29M4652N8M                                                                                                                                                                                                                                                                               GTGCAGTCGAGCCAGCACCTGTTTCTGGACCTGCCTAAG GGCCTGAATTTTCTGCTCCTGAATTCAAA|||||||||||||||||||||||||||||||||||||||||||||||||||||||||||||||||||||||||||||||||||||||||||||||||||||||||||||||||||||||||||||||||||||||||||||||||||||||||||||||||||||||||||||||||||||||||||||||||||||||||||||||||||||||||||||||||||||||||||||||||||||||||||||||||||||||||||||
chr12 chr12 57898043 59196615 39M59195042F29M1537N8M                                                                                                                                                                                                                                                                               GTGCAGTCGAGCCAGCACCTGTTTCTGGACCTGCCTAAG GGCCTGAATTTTCTGCTCCTGAATTCAAA|||||||||||||||||||||||||||||||||||||||||||||||||||||||||||||||||||||||||||||||||||||||||||||||||||||||||||||||||||||||||||||||||||||||||||||||||||||||||||||||||||||||||||||||||||||||||||||||||||||||||||||||||||||||||||||||||||||||||||||||||||||||||||||||||||||||||||||
chr12 chr12 57898043 59196615 39M59195042F29M1537N8M                                                                                                                                                                                                                                                                               GTGCAGTCGAGCCAGCACCTGTTTCTGGACCTGCCTAAG GGCCTGAATTTTCTGCTCCTGAATTCAAA|||||||||||||||||||||||||||||||||||||||||||||||||||||||||||||||||||||||||||||||||||||||||||||||||||||||||||||||||||||||||||||||||||||||||||||||||||||||||||||||||||||||||||||||||||||||||||||||||||||||||||||||||||||||||||||||||||||||||||||||||||||||||||||||||||||||||||||
chr12 chr12 57898043 59196615 39M59195042F29M1537N8M                                                                                                                                                                                                                                                                               GTGCAGTCGAGCCAGCACCTGTTTCTGGACCTGCCTAAG GGCCTGAATTTTCTGCTCCTGAATTCAAA|||||||||||||||||||||||||||||||||||||||||||||||||||||||||||||||||||||||||||||||||||||||||||||||||||||||||||||||||||||||||||||||||||||||||||||||||||||||||||||||||||||||||||||||||||||||||||||||||||||||||||||||||||||||||||||||||||||||||||||||||||||||||||||||||||||||||||||
chr12 chr12 57898043 59196615 39M59195042F29M1537N8M                                                                                                                                                                                                                                                                               GTGCAGTCGAGCCAGCACCTGTTTCTGGACCTGCCTAAG GGCCTGAATTTTCTGCTCCTGAATTCAAA|||||||||||||||||||||||||||||||||||||||||||||||||||||||||||||||||||||||||||||||||||||||||||||||||||||||||||||||||||||||||||||||||||||||||||||||||||||||||||||||||||||||||||||||||||||||||||||||||||||||||||||||||||||||||||||||||||||||||||||||||||||||||||||||||||||||||||||
chr12 chr12 57898043 59196615 39M59195042F29M1537N8M                                                                                                                                                                                                                                                                               GTGCAGTCGAGCCAGCACCTGTTTCTGGACCTGCCTAAG GGCCTGAATTTTCTGCTCCTGAATTCAAA|||||||||||||||||||||||||||||||||||||||||||||||||||||||||||||||||||||||||||||||||||||||||||||||||||||||||||||||||||||||||||||||||||||||||||||||||||||||||||||||||||||||||||||||||||||||||||||||||||||||||||||||||||||||||||||||||||||||||||||||||||||||||||||||||||||||||||||
chr12 chr12 57898043 59196615 39M59195042F29M1537N8M                                                                                                                                                                                                                                                                               GTGCAGTCGAGCCAGCACCTGTTTCTGGACCTGCCTAAG GGCCTGAATTTTCTGCTCCTGAATTCAAA|||||||||||||||||||||||||||||||||||||||||||||||||||||||||||||||||||||||||||||||||||||||||||||||||||||||||||||||||||||||||||||||||||||||||||||||||||||||||||||||||||||||||||||||||||||||||||||||||||||||||||||||||||||||||||||||||||||||||||||||||||||||||||||||||||||||||||||
chr12 chr12 57898043 59196615 39M59195042F29M1537N8M                                                                                                                                                                                                                                                                               GTGCAGTCGAGCCAGCACCTGTTTCTGGACCTGCCTAAG GGCCTGAATTTTCTGCTCCTGAATTCAAA|||||||||||||||||||||||||||||||||||||||||||||||||||||||||||||||||||||||||||||||||||||||||||||||||||||||||||||||||||||||||||||||||||||||||||||||||||||||||||||||||||||||||||||||||||||||||||||||||||||||||||||||||||||||||||||||||||||||||||||||||||||||||||||||||||||||||||||
chr12 chr12 57898043 59199730 39M59195042F29M4652N8M                                                                                                                                                                                                                                                                               GTGCAGTCGAGCCAGCACCTGTTTCTGGACCTGCCTAAG GGCCTGAATTTTCTGCTCCTGAATTCAAA|||||||||||||||||||||||||||||||||||||||||||||||||||||||||||||||||||||||||||||||||||||||||||||||||||||||||||||||||||||||||||||||||||||||||||||||||||||||||||||||||||||||||||||||||||||||||||||||||||||||||||||||||||||||||||||||||||||||||||||||||||||||||||||||||||||||||||||
chr12 chr12 57898043 59199730 39M59195042F29M4652N8M                                                                                                                                                                                                                                                                               GTGCAGTCGAGCCAGCACCTGTTTCTGGACCTGCCTAAG GGCCTGAATTTTCTGCTCCTGAATTCAAA|||||||||||||||||||||||||||||||||||||||||||||||||||||||||||||||||||||||||||||||||||||||||||||||||||||||||||||||||||||||||||||||||||||||||||||||||||||||||||||||||||||||||||||||||||||||||||||||||||||||||||||||||||||||||||||||||||||||||||||||||||||||||||||||||||||||||||||
chr12 chr12 57898043 59199730 39M59195042F29M4652N8M                                                                                                                                                                                                                                                                               GTGCAGTCGAGCCAGCACCTGTTTCTGGACCTGCCTAAG GGCCTGAATTTTCTGCTCCTGAATTCAAA|||||||||||||||||||||||||||||||||||||||||||||||||||||||||||||||||||||||||||||||||||||||||||||||||||||||||||||||||||||||||||||||||||||||||||||||||||||||||||||||||||||||||||||||||||||||||||||||||||||||||||||||||||||||||||||||||||||||||||||||||||||||||||||||||||||||||||||
chr12 chr12 57898043 59199730 39M59195042F29M4652N8M                                                                                                                                                                                                                                                                               GTGCAGTCGAGCCAGCACCTGTTTCTGGACCTGCCTAAG GGCCTGAATTTTCTGCTCCTGAATTCAAA|||||||||||||||||||||||||||||||||||||||||||||||||||||||||||||||||||||||||||||||||||||||||||||||||||||||||||||||||||||||||||||||||||||||||||||||||||||||||||||||||||||||||||||||||||||||||||||||||||||||||||||||||||||||||||||||||||||||||||||||||||||||||||||||||||||||||||||
chr12 chr12 57898043 59199730 39M59195042F29M4652N8M                                                                                                                                                                                                                                                                               GTGCAGTCGAGCCAGCACCTGTTTCTGGACCTGCCTAAG GGCCTGAATTTTCTGCTCCTGAATTCAAA|||||||||||||||||||||||||||||||||||||||||||||||||||||||||||||||||||||||||||||||||||||||||||||||||||||||||||||||||||||||||||||||||||||||||||||||||||||||||||||||||||||||||||||||||||||||||||||||||||||||||||||||||||||||||||||||||||||||||||||||||||||||||||||||||||||||||||||
chr12 chr12 57898043 59196615 39M59195042F29M1537N8M                                                                                                                                                                                                                                                                               GTGCAGTCGAGCCAGCACCTGTTTCTGGACCTGCCTAAG GGCCTGAATTTTCTGCTCCTGAATTCAAA|||||||||||||||||||||||||||||||||||||||||||||||||||||||||||||||||||||||||||||||||||||||||||||||||||||||||||||||||||||||||||||||||||||||||||||||||||||||||||||||||||||||||||||||||||||||||||||||||||||||||||||||||||||||||||||||||||||||||||||||||||||||||||||||||||||||||||||
chr12 chr12 57898043 59196615 39M59195042F29M1537N8M                                                                                                                                                                                                                                                                               GTGCAGTCGAGCCAGCACCTGTTTCTGGACCTGCCTAAG GGCCTGAATTTTCTGCTCCTGAATTCAAA|||||||||||||||||||||||||||||||||||||||||||||||||||||||||||||||||||||||||||||||||||||||||||||||||||||||||||||||||||||||||||||||||||||||||||||||||||||||||||||||||||||||||||||||||||||||||||||||||||||||||||||||||||||||||||||||||||||||||||||||||||||||||||||||||||||||||||||
chr12 chr12 57898043 59196615 39M59195042F29M1537N8M                                                                                                                                                                                                                                                                               GTGCAGTCGAGCCAGCACCTGTTTCTGGACCTGCCTAAG GGCCTGAATTTTCTGCTCCTGAATTCAAA|||||||||||||||||||||||||||||||||||||||||||||||||||||||||||||||||||||||||||||||||||||||||||||||||||||||||||||||||||||||||||||||||||||||||||||||||||||||||||||||||||||||||||||||||||||||||||||||||||||||||||||||||||||||||||||||||||||||||||||||||||||||||||||||||||||||||||||
chr12 chr12 57898043 59206203 39M59195042F29M11125N8M                                                                                                                                                                                                                                                                              GTGCAGTCGAGCCAGCACCTGTTTCTGGACCTGCCTAAG GGCCTGAATTTTCTGCTCCTGAATTCAAA|||||||||||||||||||||||||||||||||||||||||||||||||||||||||||||||||||||||||||||||||||||||||||||||||||||||||||||||||||||||||||||||||||||||||||||||||||||||||||||||||||||||||||||||||||||||||||||||||||||||||||||||||||||||||||||||||||||||||||||||||||||||||||||||||||||||||||||
chr12 chr12 57898043 59196615 39M59195042F29M1537N8M                                                                                                                                                                                                                                                                               GTGCAGTCGAGGCAGCACCTGTTTCTGGACCTGCCTAAG GGCCTGAATTTTCTGCTGCTGAATTCAAA|||||||||||||||||||||||||||||||||||||||||||||||||||||||||||||||||||||||||||||||||||||||||||||||||||||||||||||||||||||||||||||||||||||||||||||||||||||||||||||||||||||||||||||||||||||||||||||||||||||||||||||||||||||||||||||||||||||||||||||||||||||||||||||||||||||||||||||
chr12 chr12 57898043 59199730 39M59195042F29M4652N8M                                                                                                                                                                                                                                                                               GTGCAGTCGAGCCAGCACCTGTTTCTGGACCTGCCTAAG GGCCTGAATTTTCTGCTCCTGAATTCAAA|||||||||||||||||||||||||||||||||||||||||||||||||||||||||||||||||||||||||||||||||||||||||||||||||||||||||||||||||||||||||||||||||||||||||||||||||||||||||||||||||||||||||||||||||||||||||||||||||||||||||||||||||||||||||||||||||||||||||||||||||||||||||||||||||||||||||||||
chr12 chr12 57898043 59196615 39M59195042F29M1537N8M                                                                                                                                                                                                                                                                               GTGCAGTCGAGCCAGCACCTGTTTCTGGACCTGCCTAAG GGCCTGAATTTTCTGCTCCTGAATTCAAA|||||||||||||||||||||||||||||||||||||||||||||||||||||||||||||||||||||||||||||||||||||||||||||||||||||||||||||||||||||||||||||||||||||||||||||||||||||||||||||||||||||||||||||||||||||||||||||||||||||||||||||||||||||||||||||||||||||||||||||||||||||||||||||||||||||||||||||
chr12 chr12 57898043 59196615 39M59195042F29M1537N8M                                                                                                                                                                                                                                                                               GTGCAGTCGAGCCAGCACCTGTTTCTGGACCTGCCTAAG GGCCTGAATTTTCTGCTCCTGAATTCAAA|||||||||||||||||||||||||||||||||||||||||||||||||||||||||||||||||||||||||||||||||||||||||||||||||||||||||||||||||||||||||||||||||||||||||||||||||||||||||||||||||||||||||||||||||||||||||||||||||||||||||||||||||||||||||||||||||||||||||||||||||||||||||||||||||||||||||||||
chr12 chr12 57898043 59199730 39M59195042F29M4652N8M                                                                                                                                                                                                                                                                               GTGCAGTCGAGCCAGCACCTGTTTCTGGACCTGCCTAAG GGCCTGAATTTTCTGCTCCTGAATTCAAA|||||||||||||||||||||||||||||||||||||||||||||||||||||||||||||||||||||||||||||||||||||||||||||||||||||||||||||||||||||||||||||||||||||||||||||||||||||||||||||||||||||||||||||||||||||||||||||||||||||||||||||||||||||||||||||||||||||||||||||||||||||||||||||||||||||||||||||
chr12 chr12 57898043 59199730 39M59195042F29M4652N8M                                                                                                                                                                                                                                                                               GTGCAGTCGAGCCAGCACCTGTTTCTGGACCTGCCTAAG GGCCTGAATTTTCTGCTCCTGAATTCAAA|||||||||||||||||||||||||||||||||||||||||||||||||||||||||||||||||||||||||||||||||||||||||||||||||||||||||||||||||||||||||||||||||||||||||||||||||||||||||||||||||||||||||||||||||||||||||||||||||||||||||||||||||||||||||||||||||||||||||||||||||||||||||||||||||||||||||||||
chr12 chr12 57898044 59196616 38M59195042F29M1537N9M                                                                                                                                                                                                                                                                                TGCAGTCGAGCCAGCACCTGTTTCTGGACCTGCCTAAG GGCCTGAATTTTCTGCTCCTGAATTCAAA|||||||||||||||||||||||||||||||||||||||||||||||||||||||||||||||||||||||||||||||||||||||||||||||||||||||||||||||||||||||||||||||||||||||||||||||||||||||||||||||||||||||||||||||||||||||||||||||||||||||||||||||||||||||||||||||||||||||||||||||||||||||||||||||||||||||||||||
chr12 chr12 57898044 59196616 38M59195042F29M1537N9M                                                                                                                                                                                                                                                                                TGCAGTCGAGCCAGCACCTGTTTCTGGACCTGCCTAAG GGCCTGAATTTTCTGCTCCTGAATTCAAA|||||||||||||||||||||||||||||||||||||||||||||||||||||||||||||||||||||||||||||||||||||||||||||||||||||||||||||||||||||||||||||||||||||||||||||||||||||||||||||||||||||||||||||||||||||||||||||||||||||||||||||||||||||||||||||||||||||||||||||||||||||||||||||||||||||||||||||
chr12 chr12 57898044 59196616 38M59195042F29M1537N9M                                                                                                                                                                                                                                                                                TGCAGTCGAGCCAGCACCTGTTTCTGGACCTGCCTAAG GGCCTGAATTTTCTGCTCCTGAATTCAAA|||||||||||||||||||||||||||||||||||||||||||||||||||||||||||||||||||||||||||||||||||||||||||||||||||||||||||||||||||||||||||||||||||||||||||||||||||||||||||||||||||||||||||||||||||||||||||||||||||||||||||||||||||||||||||||||||||||||||||||||||||||||||||||||||||||||||||||
chr12 chr12 57898044 59196616 38M59195042F29M1537N9M                                                                                                                                                                                                                                                                                TGCAGAGGAGCCAGCACCTGTTTCTGGACCTGCCTAAG GGCCTGAATTTTCTGCTCCTGAATTCAAA|||||||||||||||||||||||||||||||||||||||||||||||||||||||||||||||||||||||||||||||||||||||||||||||||||||||||||||||||||||||||||||||||||||||||||||||||||||||||||||||||||||||||||||||||||||||||||||||||||||||||||||||||||||||||||||||||||||||||||||||||||||||||||||||||||||||||||||
chr12 chr12 57898044 59206204 38M59195042F29M11125N9M                                                                                                                                                                                                                                                                               TGCAGTCGAGCCAGCACCTGTTTCTGGACCTGCCTAAG GGCCTGAATTTTCTGCTCCTGAATTCAAA|||||||||||||||||||||||||||||||||||||||||||||||||||||||||||||||||||||||||||||||||||||||||||||||||||||||||||||||||||||||||||||||||||||||||||||||||||||||||||||||||||||||||||||||||||||||||||||||||||||||||||||||||||||||||||||||||||||||||||||||||||||||||||||||||||||||||||||
chr12 chr12 57898044 59196616 38M59195042F29M1537N9M                                                                                                                                                                                                                                                                                TGCAGTCGAGCCAGCACCTGTTTCTGGACCTGCCTAAG GGCCTGAATTTTCTGCTCCTGAATTCAAA|||||||||||||||||||||||||||||||||||||||||||||||||||||||||||||||||||||||||||||||||||||||||||||||||||||||||||||||||||||||||||||||||||||||||||||||||||||||||||||||||||||||||||||||||||||||||||||||||||||||||||||||||||||||||||||||||||||||||||||||||||||||||||||||||||||||||||||
chr12 chr12 57898044 59196616 38M59195042F29M1537N9M                                                                                                                                                                                                                                                                                TGCAGTCGAGCCAGCACCTGTTTCTGGACCTGCCTAAG GGCCTGAATTTTCTGCTCCTGAATTCAAA|||||||||||||||||||||||||||||||||||||||||||||||||||||||||||||||||||||||||||||||||||||||||||||||||||||||||||||||||||||||||||||||||||||||||||||||||||||||||||||||||||||||||||||||||||||||||||||||||||||||||||||||||||||||||||||||||||||||||||||||||||||||||||||||||||||||||||||
chr12 chr12 57898045 59196617 37M59195042F29M1537N10M                                                                                                                                                                                                                                                                                GCAGTCGAGCCAGCACCTGTTTCTGGACCTGCCTAAG GGCCTGAATTTTCTGCTCCTGAATTCAAA|||||||||||||||||||||||||||||||||||||||||||||||||||||||||||||||||||||||||||||||||||||||||||||||||||||||||||||||||||||||||||||||||||||||||||||||||||||||||||||||||||||||||||||||||||||||||||||||||||||||||||||||||||||||||||||||||||||||||||||||||||||||||||||||||||||||||||||
chr12 chr12 57898045 59196617 37M59195042F29M1537N10M                                                                                                                                                                                                                                                                                GCAGTCGAGCCAGCACCTGTTTCTGGACCTGCCTAAG GGCCTGAATTTTCTGCTCCTGAATTCAAA|||||||||||||||||||||||||||||||||||||||||||||||||||||||||||||||||||||||||||||||||||||||||||||||||||||||||||||||||||||||||||||||||||||||||||||||||||||||||||||||||||||||||||||||||||||||||||||||||||||||||||||||||||||||||||||||||||||||||||||||||||||||||||||||||||||||||||||
chr12 chr12 57898045 59196617 37M59195042F29M1537N10M                                                                                                                                                                                                                                                                                GCAGTCGAGCCAGCACCTGTTTCTGGACCTGCCTAAG GGCCTGAATTTTCTGCTCCTGAATTCAAA|||||||||||||||||||||||||||||||||||||||||||||||||||||||||||||||||||||||||||||||||||||||||||||||||||||||||||||||||||||||||||||||||||||||||||||||||||||||||||||||||||||||||||||||||||||||||||||||||||||||||||||||||||||||||||||||||||||||||||||||||||||||||||||||||||||||||||||
chr12 chr12 57898045 59196617 37M59195042F29M1537N10M                                                                                                                                                                                                                                                                                GCAGTCGAGCCAGCACCTGTTTCTGGACCTGCCTAAG GGCCTGAATTTTCTGCTCCTGAATTCAAA|||||||||||||||||||||||||||||||||||||||||||||||||||||||||||||||||||||||||||||||||||||||||||||||||||||||||||||||||||||||||||||||||||||||||||||||||||||||||||||||||||||||||||||||||||||||||||||||||||||||||||||||||||||||||||||||||||||||||||||||||||||||||||||||||||||||||||||
chr12 chr12 57898045 59196617 37M59195042F29M1537N10M                                                                                                                                                                                                                                                                                GCAGTCGAGCCAGCACCTGTTTCTGGACCTGCCTAAG GGCCTGAATTTTCTGCTCCTGAATTCAAA|||||||||||||||||||||||||||||||||||||||||||||||||||||||||||||||||||||||||||||||||||||||||||||||||||||||||||||||||||||||||||||||||||||||||||||||||||||||||||||||||||||||||||||||||||||||||||||||||||||||||||||||||||||||||||||||||||||||||||||||||||||||||||||||||||||||||||||
chr12 chr12 57898045 59196617 37M59195042F29M1537N10M                                                                                                                                                                                                                                                                                GCAGTCGAGCCAGCACCTGTTTCTGGACCTGCCTAAG GGCCTGAATTTTCTGCTCCTGAATTCAAA|||||||||||||||||||||||||||||||||||||||||||||||||||||||||||||||||||||||||||||||||||||||||||||||||||||||||||||||||||||||||||||||||||||||||||||||||||||||||||||||||||||||||||||||||||||||||||||||||||||||||||||||||||||||||||||||||||||||||||||||||||||||||||||||||||||||||||||
chr12 chr12 57898045 59196617 37M59195042F29M1537N10M                                                                                                                                                                                                                                                                                GCAGTCGAGCCAGCGCCTGTTTCTGGACCTGCCTAAG GGCCTGAATTTTCTGCTCCTGAATTCAAA|||||||||||||||||||||||||||||||||||||||||||||||||||||||||||||||||||||||||||||||||||||||||||||||||||||||||||||||||||||||||||||||||||||||||||||||||||||||||||||||||||||||||||||||||||||||||||||||||||||||||||||||||||||||||||||||||||||||||||||||||||||||||||||||||||||||||||||
chr12 chr12 57898045 59196617 37M59195042F29M1537N10M                                                                                                                                                                                                                                                                                GCAGTCGAGCCAGCACCTGTTTCTGGACCTGCCTAAG GGCCTGAATTTTCTGCTCCTGAATTCAAA|||||||||||||||||||||||||||||||||||||||||||||||||||||||||||||||||||||||||||||||||||||||||||||||||||||||||||||||||||||||||||||||||||||||||||||||||||||||||||||||||||||||||||||||||||||||||||||||||||||||||||||||||||||||||||||||||||||||||||||||||||||||||||||||||||||||||||||
chr12 chr12 57898046 59199733 36M59195042F29M4652N11M                                                                                                                                                                                                                                                                                 CAGTCGAGCCAGCACCTGTTTCTGGACCTGCCTAAG GGCCTGAATTTTCTGCTCCTGAATTCAAA|||||||||||||||||||||||||||||||||||||||||||||||||||||||||||||||||||||||||||||||||||||||||||||||||||||||||||||||||||||||||||||||||||||||||||||||||||||||||||||||||||||||||||||||||||||||||||||||||||||||||||||||||||||||||||||||||||||||||||||||||||||||||||||||||||||||||||||
chr12 chr12 57898046 59196618 36M59195042F29M1537N11M                                                                                                                                                                                                                                                                                 CAGTCGAGCCAGCACCTGTTTCTGGACCTGCCTAAG GGCCTGAATTTTCTGCTCCTGAATTCAAA|||||||||||||||||||||||||||||||||||||||||||||||||||||||||||||||||||||||||||||||||||||||||||||||||||||||||||||||||||||||||||||||||||||||||||||||||||||||||||||||||||||||||||||||||||||||||||||||||||||||||||||||||||||||||||||||||||||||||||||||||||||||||||||||||||||||||||||
chr12 chr12 57898047 59196619 35M59195042F29M1537N12M                                                                                                                                                                                                                                                                                  AGTCGAGCCAGCACCTGTTTCTGGACCTGCCTAAG GGCCTGAATTTTCTGCTCCTGAATTCAAA|||||||||||||||||||||||||||||||||||||||||||||||||||||||||||||||||||||||||||||||||||||||||||||||||||||||||||||||||||||||||||||||||||||||||||||||||||||||||||||||||||||||||||||||||||||||||||||||||||||||||||||||||||||||||||||||||||||||||||||||||||||||||||||||||||||||||||||
chr12 chr12 57898047 59196619 35M59195042F29M1537N12M                                                                                                                                                                                                                                                                                  AGTCGAGCCAGCACCTGTTTCTGGACCTGCCTAAG GGCCTGAATTTTCTGCTCCTGAATTCAAA|||||||||||||||||||||||||||||||||||||||||||||||||||||||||||||||||||||||||||||||||||||||||||||||||||||||||||||||||||||||||||||||||||||||||||||||||||||||||||||||||||||||||||||||||||||||||||||||||||||||||||||||||||||||||||||||||||||||||||||||||||||||||||||||||||||||||||||
chr12 chr12 57898047 59196619 35M59195042F29M1537N12M                                                                                                                                                                                                                                                                                  AGTCGAGCCAGCACCTGTTTCTGGACCTGCCTAAG GGCCTGAATTTTCTGCTCCTGAATTCAAA|||||||||||||||||||||||||||||||||||||||||||||||||||||||||||||||||||||||||||||||||||||||||||||||||||||||||||||||||||||||||||||||||||||||||||||||||||||||||||||||||||||||||||||||||||||||||||||||||||||||||||||||||||||||||||||||||||||||||||||||||||||||||||||||||||||||||||||
chr12 chr12 57898047 59199734 35M59195042F29M4652N12M                                                                                                                                                                                                                                                                                  AGTCGAGCCAGCACCTGTTTCTGGACCTGCCTAAG GGCCTGAATTTTCTGCTCCTGAATTCAAA|||||||||||||||||||||||||||||||||||||||||||||||||||||||||||||||||||||||||||||||||||||||||||||||||||||||||||||||||||||||||||||||||||||||||||||||||||||||||||||||||||||||||||||||||||||||||||||||||||||||||||||||||||||||||||||||||||||||||||||||||||||||||||||||||||||||||||||
chr12 chr12 57898047 59199734 35M59195042F29M4652N12M                                                                                                                                                                                                                                                                                  AGTCGAGCCAGCACCTGTTTCTGGACCTGCCTAAG GGCCTGAATTTTCTGCTCCTGAATTCAAA|||||||||||||||||||||||||||||||||||||||||||||||||||||||||||||||||||||||||||||||||||||||||||||||||||||||||||||||||||||||||||||||||||||||||||||||||||||||||||||||||||||||||||||||||||||||||||||||||||||||||||||||||||||||||||||||||||||||||||||||||||||||||||||||||||||||||||||
chr12 chr12 57898047 59196619 35M59195042F29M1537N12M                                                                                                                                                                                                                                                                                  AGTCGAGCCAGCACCTGTTTCTGGACCTGCCTAAG GGCCTGAATTTTCTGCTCCTGAATTCAAA|||||||||||||||||||||||||||||||||||||||||||||||||||||||||||||||||||||||||||||||||||||||||||||||||||||||||||||||||||||||||||||||||||||||||||||||||||||||||||||||||||||||||||||||||||||||||||||||||||||||||||||||||||||||||||||||||||||||||||||||||||||||||||||||||||||||||||||
chr12 chr12 57898047 59206207 35M59195042F29M11125N12M                                                                                                                                                                                                                                                                                 AGTCGAGCCAGCACCTGATTCTGGACCTGCCTAAG GGCCTGAATTTTCTGCTCCTGAATTCAAA|||||||||||||||||||||||||||||||||||||||||||||||||||||||||||||||||||||||||||||||||||||||||||||||||||||||||||||||||||||||||||||||||||||||||||||||||||||||||||||||||||||||||||||||||||||||||||||||||||||||||||||||||||||||||||||||||||||||||||||||||||||||||||||||||||||||||||||
chr12 chr12 57898047 59199734 35M59195042F29M4652N12M                                                                                                                                                                                                                                                                                  AGTCGAGCCAGCACCTGTTTCTGGACCTGCCTAAG GGCCTGAATTTTCTGCTCCTGAATTCAAA|||||||||||||||||||||||||||||||||||||||||||||||||||||||||||||||||||||||||||||||||||||||||||||||||||||||||||||||||||||||||||||||||||||||||||||||||||||||||||||||||||||||||||||||||||||||||||||||||||||||||||||||||||||||||||||||||||||||||||||||||||||||||||||||||||||||||||||
chr12 chr12 57898047 59196619 35M59195042F29M1537N12M                                                                                                                                                                                                                                                                                  AGTCGAGCCAGCACCTGTTTCTGGACCTGCCTAAG GGCCTGAATTTTCTGCTCCTGAATTCAAA|||||||||||||||||||||||||||||||||||||||||||||||||||||||||||||||||||||||||||||||||||||||||||||||||||||||||||||||||||||||||||||||||||||||||||||||||||||||||||||||||||||||||||||||||||||||||||||||||||||||||||||||||||||||||||||||||||||||||||||||||||||||||||||||||||||||||||||
chr12 chr12 57898047 59196619 35M59195042F29M1537N12M                                                                                                                                                                                                                                                                                  AGTCGAGCCAGCACCTGTTTCTGGACCTGCCTAAG GGCCTGAATTTTCTGCTCCTGAATTCAAA|||||||||||||||||||||||||||||||||||||||||||||||||||||||||||||||||||||||||||||||||||||||||||||||||||||||||||||||||||||||||||||||||||||||||||||||||||||||||||||||||||||||||||||||||||||||||||||||||||||||||||||||||||||||||||||||||||||||||||||||||||||||||||||||||||||||||||||
chr12 chr12 57898047 59196619 35M59195042F29M1537N12M                                                                                                                                                                                                                                                                                  AGTCGAGCCAGCACCTGTTTCTGGACCTGCCTAAG GGCCTGAATTTTCTGCTCCTGAATTCAAA|||||||||||||||||||||||||||||||||||||||||||||||||||||||||||||||||||||||||||||||||||||||||||||||||||||||||||||||||||||||||||||||||||||||||||||||||||||||||||||||||||||||||||||||||||||||||||||||||||||||||||||||||||||||||||||||||||||||||||||||||||||||||||||||||||||||||||||
chr12 chr12 57898047 59199734 35M59195042F29M4652N12M                                                                                                                                                                                                                                                                                  AGTCGAGCCAGCACCTGTTTCTGGACCTGCCTAAG GGCCTGAATTTTCTGCTCCTGAATTCAAA|||||||||||||||||||||||||||||||||||||||||||||||||||||||||||||||||||||||||||||||||||||||||||||||||||||||||||||||||||||||||||||||||||||||||||||||||||||||||||||||||||||||||||||||||||||||||||||||||||||||||||||||||||||||||||||||||||||||||||||||||||||||||||||||||||||||||||||
chr12 chr12 57898047 59206207 35M59195042F29M11125N12M                                                                                                                                                                                                                                                                                 AGTCGAGCCAGCACCTGTTTCTGGACCTGCCTAAG GGCCTGAATTTTCTGCTCCTGAATTCAAA|||||||||||||||||||||||||||||||||||||||||||||||||||||||||||||||||||||||||||||||||||||||||||||||||||||||||||||||||||||||||||||||||||||||||||||||||||||||||||||||||||||||||||||||||||||||||||||||||||||||||||||||||||||||||||||||||||||||||||||||||||||||||||||||||||||||||||||
chr12 chr12 57898048 59196620 34M59195042F29M1537N13M                                                                                                                                                                                                                                                                                   GTCGAGCCAGCACCTGTTTCTGGACCTGCCTAAG GGCCTGAATTTTCTGCTCCTGAATTCAAA|||||||||||||||||||||||||||||||||||||||||||||||||||||||||||||||||||||||||||||||||||||||||||||||||||||||||||||||||||||||||||||||||||||||||||||||||||||||||||||||||||||||||||||||||||||||||||||||||||||||||||||||||||||||||||||||||||||||||||||||||||||||||||||||||||||||||||||
chr12 chr12 57898048 59196620 34M59195042F29M1537N13M                                                                                                                                                                                                                                                                                   NTCGAGCCAGCACCTGTTTCTGGACCTGCCTAAG GGCCTGAATTTTCTGCTCCTGAATTCAAA|||||||||||||||||||||||||||||||||||||||||||||||||||||||||||||||||||||||||||||||||||||||||||||||||||||||||||||||||||||||||||||||||||||||||||||||||||||||||||||||||||||||||||||||||||||||||||||||||||||||||||||||||||||||||||||||||||||||||||||||||||||||||||||||||||||||||||||
chr12 chr12 57898048 59196620 34M59195042F29M1537N13M                                                                                                                                                                                                                                                                                   GTCGAGCCAGCACCTGTTTCTGGACCTGCCTAAG GGCCTGAATTTTCTGCTCCTGAATTCAAA|||||||||||||||||||||||||||||||||||||||||||||||||||||||||||||||||||||||||||||||||||||||||||||||||||||||||||||||||||||||||||||||||||||||||||||||||||||||||||||||||||||||||||||||||||||||||||||||||||||||||||||||||||||||||||||||||||||||||||||||||||||||||||||||||||||||||||||
chr12 chr12 57898048 59196620 34M59195042F29M1537N13M                                                                                                                                                                                                                                                                                   GTCGAGCCAGCACCTGTTTCTGGACCTGCCTAAG GGCCTGAATTTTCTGCTCCTGAATTCAAA|||||||||||||||||||||||||||||||||||||||||||||||||||||||||||||||||||||||||||||||||||||||||||||||||||||||||||||||||||||||||||||||||||||||||||||||||||||||||||||||||||||||||||||||||||||||||||||||||||||||||||||||||||||||||||||||||||||||||||||||||||||||||||||||||||||||||||||
chr12 chr12 57898048 59199735 34M59195042F29M4652N13M                                                                                                                                                                                                                                                                                   GTCGAGCCAGCACCTGTTTCTGGACCTGCCTAAG GGCCTGAATTTTCTGCTCCTGAATTCAAA|||||||||||||||||||||||||||||||||||||||||||||||||||||||||||||||||||||||||||||||||||||||||||||||||||||||||||||||||||||||||||||||||||||||||||||||||||||||||||||||||||||||||||||||||||||||||||||||||||||||||||||||||||||||||||||||||||||||||||||||||||||||||||||||||||||||||||||
chr12 chr12 57898048 59199735 34M59195042F29M4652N13M                                                                                                                                                                                                                                                                                   GTCGAGCCAGCACCTGTTTCTGGACCTGCCTAAG GGCCTGAATTTTCTGCTCCTGAATTCAAA|||||||||||||||||||||||||||||||||||||||||||||||||||||||||||||||||||||||||||||||||||||||||||||||||||||||||||||||||||||||||||||||||||||||||||||||||||||||||||||||||||||||||||||||||||||||||||||||||||||||||||||||||||||||||||||||||||||||||||||||||||||||||||||||||||||||||||||
chr12 chr12 57898048 59196620 34M59195042F29M1537N13M                                                                                                                                                                                                                                                                                   CTCGAGCCAGCACCTGTTTCTGGACCTGCCTAAG GGCCTGAATTTTCTGCTCCTGAATTCAAA|||||||||||||||||||||||||||||||||||||||||||||||||||||||||||||||||||||||||||||||||||||||||||||||||||||||||||||||||||||||||||||||||||||||||||||||||||||||||||||||||||||||||||||||||||||||||||||||||||||||||||||||||||||||||||||||||||||||||||||||||||||||||||||||||||||||||||||
chr12 chr12 57898048 59196620 34M59195042F29M1537N13M                                                                                                                                                                                                                                                                                   GTCGAGCCAGCACCTGTTTCTGGACCTGCCTAAG GGCCTGAATTTTCTGCTCCTGAATTCAAA|||||||||||||||||||||||||||||||||||||||||||||||||||||||||||||||||||||||||||||||||||||||||||||||||||||||||||||||||||||||||||||||||||||||||||||||||||||||||||||||||||||||||||||||||||||||||||||||||||||||||||||||||||||||||||||||||||||||||||||||||||||||||||||||||||||||||||||
chr12 chr12 57898048 59196620 34M59195042F29M1537N13M                                                                                                                                                                                                                                                                                   GTCGAGCCAGCACCTGTTTCTGGACCTGCCTAAG GGCCTGAATTTTCTGCTCCTGAATTCAAA|||||||||||||||||||||||||||||||||||||||||||||||||||||||||||||||||||||||||||||||||||||||||||||||||||||||||||||||||||||||||||||||||||||||||||||||||||||||||||||||||||||||||||||||||||||||||||||||||||||||||||||||||||||||||||||||||||||||||||||||||||||||||||||||||||||||||||||
chr12 chr12 57898048 59199735 34M59195042F29M4652N13M                                                                                                                                                                                                                                                                                   GTCGAGCCAGCACCTGTTTCTGGACCTGCCTAAG GGCCTGAATTTTCTGCTCCTGAATTCAAA|||||||||||||||||||||||||||||||||||||||||||||||||||||||||||||||||||||||||||||||||||||||||||||||||||||||||||||||||||||||||||||||||||||||||||||||||||||||||||||||||||||||||||||||||||||||||||||||||||||||||||||||||||||||||||||||||||||||||||||||||||||||||||||||||||||||||||||
chr12 chr12 57898048 59196620 34M59195042F29M1537N13M                                                                                                                                                                                                                                                                                   GTCGAGCCAGCACCTGTTTCTGGACCTGCCTAAG GGCCTGAATTTTCTGCTCCTGAATTCAAA|||||||||||||||||||||||||||||||||||||||||||||||||||||||||||||||||||||||||||||||||||||||||||||||||||||||||||||||||||||||||||||||||||||||||||||||||||||||||||||||||||||||||||||||||||||||||||||||||||||||||||||||||||||||||||||||||||||||||||||||||||||||||||||||||||||||||||||
chr12 chr12 57898048 59196620 34M59195042F29M1537N13M                                                                                                                                                                                                                                                                                   GTCGAGCCAGCACCTGTTTCTGGACCTGCCTAAG GGCCTGAATTTTCTGCTCCTGAATTCAAA|||||||||||||||||||||||||||||||||||||||||||||||||||||||||||||||||||||||||||||||||||||||||||||||||||||||||||||||||||||||||||||||||||||||||||||||||||||||||||||||||||||||||||||||||||||||||||||||||||||||||||||||||||||||||||||||||||||||||||||||||||||||||||||||||||||||||||||
chr12 chr12 57898048 59196620 34M59195042F29M1537N13M                                                                                                                                                                                                                                                                                   GTCGAGCCAGCACCTGTTTCTGGACCTGCCTAAG GGCCTGAATTTTCTGCTCCTGAATTCAAA|||||||||||||||||||||||||||||||||||||||||||||||||||||||||||||||||||||||||||||||||||||||||||||||||||||||||||||||||||||||||||||||||||||||||||||||||||||||||||||||||||||||||||||||||||||||||||||||||||||||||||||||||||||||||||||||||||||||||||||||||||||||||||||||||||||||||||||
chr12 chr12 57898048 59196620 34M59195042F29M1537N13M                                                                                                                                                                                                                                                                                   GTCGAGCCAGCACCTGTTTCTGGACCTGCCTAAG GGCCTGAATTTTCTGCTCCTGAATTCAAA|||||||||||||||||||||||||||||||||||||||||||||||||||||||||||||||||||||||||||||||||||||||||||||||||||||||||||||||||||||||||||||||||||||||||||||||||||||||||||||||||||||||||||||||||||||||||||||||||||||||||||||||||||||||||||||||||||||||||||||||||||||||||||||||||||||||||||||
chr12 chr12 57898048 59196620 34M59195042F29M1537N13M                                                                                                                                                                                                                                                                                   GTCGAGCCAGCACCTGTTTCTGGACCTGCCTAAG GGCCTGAATTTTCTGCTCCTGAATTCAAA|||||||||||||||||||||||||||||||||||||||||||||||||||||||||||||||||||||||||||||||||||||||||||||||||||||||||||||||||||||||||||||||||||||||||||||||||||||||||||||||||||||||||||||||||||||||||||||||||||||||||||||||||||||||||||||||||||||||||||||||||||||||||||||||||||||||||||||
chr12 chr12 57898048 59199735 34M59195042F29M4652N13M                                                                                                                                                                                                                                                                                   GTCGAGCCAGCACCTGTTTCTGGACCTGCCTAAG GGCCTGAATTTTCTGCTCCTGAATTCAAA|||||||||||||||||||||||||||||||||||||||||||||||||||||||||||||||||||||||||||||||||||||||||||||||||||||||||||||||||||||||||||||||||||||||||||||||||||||||||||||||||||||||||||||||||||||||||||||||||||||||||||||||||||||||||||||||||||||||||||||||||||||||||||||||||||||||||||||
chr12 chr12 57898048 59199735 34M59195042F29M4652N13M                                                                                                                                                                                                                                                                                   GTCGAGCCAGCACCTGTTTCTGGACCTGCCTAAG GGCCTGAATTTTCTGCTCCTGAATTCAAA|||||||||||||||||||||||||||||||||||||||||||||||||||||||||||||||||||||||||||||||||||||||||||||||||||||||||||||||||||||||||||||||||||||||||||||||||||||||||||||||||||||||||||||||||||||||||||||||||||||||||||||||||||||||||||||||||||||||||||||||||||||||||||||||||||||||||||||
chr12 chr12 57898048 59199735 34M59195042F29M4652N13M                                                                                                                                                                                                                                                                                   GTCGAGCCAGCACCTGTTTCTGGACCTGCCTAAG GGCATGAATTTTCTGCTCCTGAATTCAAA|||||||||||||||||||||||||||||||||||||||||||||||||||||||||||||||||||||||||||||||||||||||||||||||||||||||||||||||||||||||||||||||||||||||||||||||||||||||||||||||||||||||||||||||||||||||||||||||||||||||||||||||||||||||||||||||||||||||||||||||||||||||||||||||||||||||||||||
chr12 chr12 57898048 59196620 34M59195042F29M1537N13M                                                                                                                                                                                                                                                                                   GTCGAGCCAGCACCTGTTTCTGGACCTGCCTAAT GGCCTGAATTTTCTGCTCCTGAATTCAAA|||||||||||||||||||||||||||||||||||||||||||||||||||||||||||||||||||||||||||||||||||||||||||||||||||||||||||||||||||||||||||||||||||||||||||||||||||||||||||||||||||||||||||||||||||||||||||||||||||||||||||||||||||||||||||||||||||||||||||||||||||||||||||||||||||||||||||||
chr12 chr12 57898048 59196620 34M59195042F29M1537N13M                                                                                                                                                                                                                                                                                   GTCGAGCCAGCACCTGTTTCTGGACCTGCCTAAG GGCCTGAATTTTCTGCTCCTGAATTCAAA|||||||||||||||||||||||||||||||||||||||||||||||||||||||||||||||||||||||||||||||||||||||||||||||||||||||||||||||||||||||||||||||||||||||||||||||||||||||||||||||||||||||||||||||||||||||||||||||||||||||||||||||||||||||||||||||||||||||||||||||||||||||||||||||||||||||||||||
chr12 chr12 57898048 59199735 34M59195042F29M4652N13M                                                                                                                                                                                                                                                                                   GTCGAGCCAGCACCTGTTTCTGGACCTGCCTAAG GGCCTGAATTTTCTGCTCCTGAATTCAAA|||||||||||||||||||||||||||||||||||||||||||||||||||||||||||||||||||||||||||||||||||||||||||||||||||||||||||||||||||||||||||||||||||||||||||||||||||||||||||||||||||||||||||||||||||||||||||||||||||||||||||||||||||||||||||||||||||||||||||||||||||||||||||||||||||||||||||||
chr12 chr12 57898048 59196620 34M59195042F29M1537N13M                                                                                                                                                                                                                                                                                   GTCGAGCCAGCACCTGTTTCTGGACCTGCCTAAG GGCCTGAATTTTCTGCTCCTGAATTCAAA|||||||||||||||||||||||||||||||||||||||||||||||||||||||||||||||||||||||||||||||||||||||||||||||||||||||||||||||||||||||||||||||||||||||||||||||||||||||||||||||||||||||||||||||||||||||||||||||||||||||||||||||||||||||||||||||||||||||||||||||||||||||||||||||||||||||||||||
chr12 chr12 57898049 59196621 33M59195042F29M1537N14M                                                                                                                                                                                                                                                                                    TCGAGCCAGCACCTGTTTCTGGACCTGCCTAAG GGCCTGAATTTTCTGCTCCTGAATTCAAA|||||||||||||||||||||||||||||||||||||||||||||||||||||||||||||||||||||||||||||||||||||||||||||||||||||||||||||||||||||||||||||||||||||||||||||||||||||||||||||||||||||||||||||||||||||||||||||||||||||||||||||||||||||||||||||||||||||||||||||||||||||||||||||||||||||||||||||
chr12 chr12 57898049 59196621 33M59195042F29M1537N14M                                                                                                                                                                                                                                                                                    TCGAGCCAGCACCTGTTTCTGGACCTGCCTAAG GGCCTGAATTTTCTGCTCCTGAATTCAAA|||||||||||||||||||||||||||||||||||||||||||||||||||||||||||||||||||||||||||||||||||||||||||||||||||||||||||||||||||||||||||||||||||||||||||||||||||||||||||||||||||||||||||||||||||||||||||||||||||||||||||||||||||||||||||||||||||||||||||||||||||||||||||||||||||||||||||||
chr12 chr12 57898049 59196621 33M59195042F29M1537N14M                                                                                                                                                                                                                                                                                    TCGAGCCAGCACCTGTTTCTGGACCTGCCTAAG GGCCTGAATTTTCTGCTCCTGAATTCAAA|||||||||||||||||||||||||||||||||||||||||||||||||||||||||||||||||||||||||||||||||||||||||||||||||||||||||||||||||||||||||||||||||||||||||||||||||||||||||||||||||||||||||||||||||||||||||||||||||||||||||||||||||||||||||||||||||||||||||||||||||||||||||||||||||||||||||||||
chr12 chr12 57898049 59196621 33M59195042F29M1537N14M                                                                                                                                                                                                                                                                                    TCGAGCCAGCACCTGTTTCTGGACCTGCCTAAG GGCCTGAATTTTCTGCTCCTGAATTCAAA|||||||||||||||||||||||||||||||||||||||||||||||||||||||||||||||||||||||||||||||||||||||||||||||||||||||||||||||||||||||||||||||||||||||||||||||||||||||||||||||||||||||||||||||||||||||||||||||||||||||||||||||||||||||||||||||||||||||||||||||||||||||||||||||||||||||||||||
chr12 chr12 57898049 59196621 33M59195042F29M1537N14M                                                                                                                                                                                                                                                                                    TCGAGCCAGCACCTGTTTCTGGACCTGCCTAAG GGCCTGAATTTTCTGCTCCTGAATTCAAA|||||||||||||||||||||||||||||||||||||||||||||||||||||||||||||||||||||||||||||||||||||||||||||||||||||||||||||||||||||||||||||||||||||||||||||||||||||||||||||||||||||||||||||||||||||||||||||||||||||||||||||||||||||||||||||||||||||||||||||||||||||||||||||||||||||||||||||
chr12 chr12 57898050 59196622 32M59195042F29M1537N15M                                                                                                                                                                                                                                                                                     CGAGCCAGCACCTGTTTCTGGACCTGCCTAAG GGCCTGAATTTTCTGCTCCTGAATTCAAA|||||||||||||||||||||||||||||||||||||||||||||||||||||||||||||||||||||||||||||||||||||||||||||||||||||||||||||||||||||||||||||||||||||||||||||||||||||||||||||||||||||||||||||||||||||||||||||||||||||||||||||||||||||||||||||||||||||||||||||||||||||||||||||||||||||||||||||
chr12 chr12 57898050 59196727 32M59195042F29M1642N15M                                                                                                                                                                                                                                                                                     CGAGCCAGCACCTGTTTCTGGACCTGCCTAAG GGCCTGAATTTTCTGCTCCTGAATTCAAA|||||||||||||||||||||||||||||||||||||||||||||||||||||||||||||||||||||||||||||||||||||||||||||||||||||||||||||||||||||||||||||||||||||||||||||||||||||||||||||||||||||||||||||||||||||||||||||||||||||||||||||||||||||||||||||||||||||||||||||||||||||||||||||||||||||||||||||
chr12 chr12 57898050 59196622 32M59195042F29M1537N15M                                                                                                                                                                                                                                                                                     CGAGCCAGCACCTGTTTCTGGACCTGCCTAAG GGCCTGAATTTTCTGCTCCTGAATTCAAA|||||||||||||||||||||||||||||||||||||||||||||||||||||||||||||||||||||||||||||||||||||||||||||||||||||||||||||||||||||||||||||||||||||||||||||||||||||||||||||||||||||||||||||||||||||||||||||||||||||||||||||||||||||||||||||||||||||||||||||||||||||||||||||||||||||||||||||
chr12 chr12 57898050 59199737 32M59195042F29M4652N15M                                                                                                                                                                                                                                                                                     CGAGCCAGCACCTGTTTCTGGACCTGCCTAAG GGCCTGAATTTTCTGCTCCTGAATTCAAA|||||||||||||||||||||||||||||||||||||||||||||||||||||||||||||||||||||||||||||||||||||||||||||||||||||||||||||||||||||||||||||||||||||||||||||||||||||||||||||||||||||||||||||||||||||||||||||||||||||||||||||||||||||||||||||||||||||||||||||||||||||||||||||||||||||||||||||
chr12 chr12 57898050 59199737 32M59195042F29M4652N15M                                                                                                                                                                                                                                                                                     CGAGCCAGCACCTGTTTCTGGACCTGCCTAAG GGCCTGAATTTTCTGCTCCTGAATTCAAA|||||||||||||||||||||||||||||||||||||||||||||||||||||||||||||||||||||||||||||||||||||||||||||||||||||||||||||||||||||||||||||||||||||||||||||||||||||||||||||||||||||||||||||||||||||||||||||||||||||||||||||||||||||||||||||||||||||||||||||||||||||||||||||||||||||||||||||
chr12 chr12 57898050 59206210 32M59195042F29M11125N15M                                                                                                                                                                                                                                                                                    CGAGCCAGCACCTGTTTCTGGACCTGCCTAAG GGCCTGAATTTTCTGCTCCTGAATTCAAA|||||||||||||||||||||||||||||||||||||||||||||||||||||||||||||||||||||||||||||||||||||||||||||||||||||||||||||||||||||||||||||||||||||||||||||||||||||||||||||||||||||||||||||||||||||||||||||||||||||||||||||||||||||||||||||||||||||||||||||||||||||||||||||||||||||||||||||
chr12 chr12 57898050 59196622 32M59195042F29M1537N15M                                                                                                                                                                                                                                                                                     CGAGCCAGCACCTGTTTCTGGACCTGCCTAAG GGCCTGAATTTTCTGCTCCTGAATTCAAA|||||||||||||||||||||||||||||||||||||||||||||||||||||||||||||||||||||||||||||||||||||||||||||||||||||||||||||||||||||||||||||||||||||||||||||||||||||||||||||||||||||||||||||||||||||||||||||||||||||||||||||||||||||||||||||||||||||||||||||||||||||||||||||||||||||||||||||
chr12 chr12 57898050 59196622 32M59195042F29M1537N15M                                                                                                                                                                                                                                                                                     CGAGCCAGCACCTGTTTCTGGACCTGCCTAAG GGCCTGAATTTTCTGCTCCTGAATTCAAA|||||||||||||||||||||||||||||||||||||||||||||||||||||||||||||||||||||||||||||||||||||||||||||||||||||||||||||||||||||||||||||||||||||||||||||||||||||||||||||||||||||||||||||||||||||||||||||||||||||||||||||||||||||||||||||||||||||||||||||||||||||||||||||||||||||||||||||
chr12 chr12 57898050 59196622 32M59195042F29M1537N15M                                                                                                                                                                                                                                                                                     CGAGCCAGCACCTGTTTCTGGACCTGCCTAAG GGCCTGAATTTTCTGCTCCTGAATTCAAA|||||||||||||||||||||||||||||||||||||||||||||||||||||||||||||||||||||||||||||||||||||||||||||||||||||||||||||||||||||||||||||||||||||||||||||||||||||||||||||||||||||||||||||||||||||||||||||||||||||||||||||||||||||||||||||||||||||||||||||||||||||||||||||||||||||||||||||
chr12 chr12 57898050 59199737 32M59195042F29M4652N15M                                                                                                                                                                                                                                                                                     CGAGCCAGCACCTGTTTCTGGACCTGCCTAAG GGCCTGAATTTTCTGCTCCTGAATTCAAA|||||||||||||||||||||||||||||||||||||||||||||||||||||||||||||||||||||||||||||||||||||||||||||||||||||||||||||||||||||||||||||||||||||||||||||||||||||||||||||||||||||||||||||||||||||||||||||||||||||||||||||||||||||||||||||||||||||||||||||||||||||||||||||||||||||||||||||
chr12 chr12 57898050 59199737 32M59195042F29M4652N15M                                                                                                                                                                                                                                                                                     CGAGCCAGCACCTGTTTCTGGACCTGCCTAAG GGCCTGAATTTTCTGCTCCTGAATTCAAA|||||||||||||||||||||||||||||||||||||||||||||||||||||||||||||||||||||||||||||||||||||||||||||||||||||||||||||||||||||||||||||||||||||||||||||||||||||||||||||||||||||||||||||||||||||||||||||||||||||||||||||||||||||||||||||||||||||||||||||||||||||||||||||||||||||||||||||
chr12 chr12 57898050 59196622 32M59195042F29M1537N15M                                                                                                                                                                                                                                                                                     CGAGCCAGCACCTGTTTCTGGACCTGCCTAAG GGCCTGAATTTTCTGCTCCTGAATTCAAA|||||||||||||||||||||||||||||||||||||||||||||||||||||||||||||||||||||||||||||||||||||||||||||||||||||||||||||||||||||||||||||||||||||||||||||||||||||||||||||||||||||||||||||||||||||||||||||||||||||||||||||||||||||||||||||||||||||||||||||||||||||||||||||||||||||||||||||
chr12 chr12 57898050 59196622 32M59195042F29M1537N15M                                                                                                                                                                                                                                                                                     CGAGCCAGCACCTGTTTCTGGACCTGCCTAAG GGCCTGAATTTTCTGCTCCTGAATTCAAA|||||||||||||||||||||||||||||||||||||||||||||||||||||||||||||||||||||||||||||||||||||||||||||||||||||||||||||||||||||||||||||||||||||||||||||||||||||||||||||||||||||||||||||||||||||||||||||||||||||||||||||||||||||||||||||||||||||||||||||||||||||||||||||||||||||||||||||
chr12 chr12 57898050 59196622 32M59195042F29M1537N15M                                                                                                                                                                                                                                                                                     CGAGCCAGCACCTGTTTCTGGACCTGCCTAAG GGCCTGAATTTTCTGCTCCTGAATTCAAA|||||||||||||||||||||||||||||||||||||||||||||||||||||||||||||||||||||||||||||||||||||||||||||||||||||||||||||||||||||||||||||||||||||||||||||||||||||||||||||||||||||||||||||||||||||||||||||||||||||||||||||||||||||||||||||||||||||||||||||||||||||||||||||||||||||||||||||
chr12 chr12 57898050 59196622 32M59195042F29M1537N15M                                                                                                                                                                                                                                                                                     CGAGCCAGCACCTGTTTCTGGACCTGCCTAAG GGCCTGAATTTTCTGCTCCTGAATTCAAA|||||||||||||||||||||||||||||||||||||||||||||||||||||||||||||||||||||||||||||||||||||||||||||||||||||||||||||||||||||||||||||||||||||||||||||||||||||||||||||||||||||||||||||||||||||||||||||||||||||||||||||||||||||||||||||||||||||||||||||||||||||||||||||||||||||||||||||
chr12 chr12 57898050 59196622 32M59195042F29M1537N15M                                                                                                                                                                                                                                                                                     CGAGCCAGCACCTGTTTCTGGACCTGCCTAAG GGCCTGAATTTTCTGCTCCTGAATTCAAA|||||||||||||||||||||||||||||||||||||||||||||||||||||||||||||||||||||||||||||||||||||||||||||||||||||||||||||||||||||||||||||||||||||||||||||||||||||||||||||||||||||||||||||||||||||||||||||||||||||||||||||||||||||||||||||||||||||||||||||||||||||||||||||||||||||||||||||
chr12 chr12 57898050 59196622 32M59195042F29M1537N15M                                                                                                                                                                                                                                                                                     CGCGCCAGCACCTGTTTCTGGACCTGCCTAAG GGCCTGAATTTTCTGCTCCTGAATTCAAA|||||||||||||||||||||||||||||||||||||||||||||||||||||||||||||||||||||||||||||||||||||||||||||||||||||||||||||||||||||||||||||||||||||||||||||||||||||||||||||||||||||||||||||||||||||||||||||||||||||||||||||||||||||||||||||||||||||||||||||||||||||||||||||||||||||||||||||
chr12 chr12 57898050 59196622 32M59195042F29M1537N15M                                                                                                                                                                                                                                                                                     CGAGCCAGCACCTGTTTCTGGACCTGCCTAAG GGCCTGAATTTTCTGCTCCTGAATTCAAA|||||||||||||||||||||||||||||||||||||||||||||||||||||||||||||||||||||||||||||||||||||||||||||||||||||||||||||||||||||||||||||||||||||||||||||||||||||||||||||||||||||||||||||||||||||||||||||||||||||||||||||||||||||||||||||||||||||||||||||||||||||||||||||||||||||||||||||
chr12 chr12 57898050 59196622 32M59195042F29M1537N15M                                                                                                                                                                                                                                                                                     CGAGCCAGCACCTGTTTCTGGACCTGCCTAAG GGCCTGAATTTTCTGCTCCTGAATTCAAA|||||||||||||||||||||||||||||||||||||||||||||||||||||||||||||||||||||||||||||||||||||||||||||||||||||||||||||||||||||||||||||||||||||||||||||||||||||||||||||||||||||||||||||||||||||||||||||||||||||||||||||||||||||||||||||||||||||||||||||||||||||||||||||||||||||||||||||
chr12 chr12 57898050 59199737 32M59195042F29M4652N15M                                                                                                                                                                                                                                                                                     CGAGCCAGCACCTGTTTCTGGACCTGCCTAAG GGCCTGAATTTTCTGCTCCTGAATTCAAA|||||||||||||||||||||||||||||||||||||||||||||||||||||||||||||||||||||||||||||||||||||||||||||||||||||||||||||||||||||||||||||||||||||||||||||||||||||||||||||||||||||||||||||||||||||||||||||||||||||||||||||||||||||||||||||||||||||||||||||||||||||||||||||||||||||||||||||
chr12 chr12 57898050 59199737 32M59195042F29M4652N15M                                                                                                                                                                                                                                                                                     CGAGCCAGCACCTGTTTCTGGACCTGCCTAAG GGCCTGAATTTTCTGCTCCTGAATTCAAA|||||||||||||||||||||||||||||||||||||||||||||||||||||||||||||||||||||||||||||||||||||||||||||||||||||||||||||||||||||||||||||||||||||||||||||||||||||||||||||||||||||||||||||||||||||||||||||||||||||||||||||||||||||||||||||||||||||||||||||||||||||||||||||||||||||||||||||
chr12 chr12 57898050 59199737 32M59195042F29M4652N15M                                                                                                                                                                                                                                                                                     CGAGCCAGCACCTGTTTCTGGACCTGCCTAAG GGCCTGAATTTTCTGCTCCTGAATTCAAA|||||||||||||||||||||||||||||||||||||||||||||||||||||||||||||||||||||||||||||||||||||||||||||||||||||||||||||||||||||||||||||||||||||||||||||||||||||||||||||||||||||||||||||||||||||||||||||||||||||||||||||||||||||||||||||||||||||||||||||||||||||||||||||||||||||||||||||
chr12 chr12 57898050 59199737 32M59195042F29M4652N15M                                                                                                                                                                                                                                                                                     CGAGCCAGCACCTGTTTCTGGACCTGCCTAAG GGCCTGAATTTTCTGCTCCTGAATTCAAA|||||||||||||||||||||||||||||||||||||||||||||||||||||||||||||||||||||||||||||||||||||||||||||||||||||||||||||||||||||||||||||||||||||||||||||||||||||||||||||||||||||||||||||||||||||||||||||||||||||||||||||||||||||||||||||||||||||||||||||||||||||||||||||||||||||||||||||
chr12 chr12 57898050 59199737 32M59195042F29M4652N15M                                                                                                                                                                                                                                                                                     CGAGCCAGCACCTGTTTCTGGACCTGCCTAAG GGCCTGAATTTTCTGCTCCTGAATTCAAA|||||||||||||||||||||||||||||||||||||||||||||||||||||||||||||||||||||||||||||||||||||||||||||||||||||||||||||||||||||||||||||||||||||||||||||||||||||||||||||||||||||||||||||||||||||||||||||||||||||||||||||||||||||||||||||||||||||||||||||||||||||||||||||||||||||||||||||
chr12 chr12 57898050 59199737 32M59195042F29M4652N15M                                                                                                                                                                                                                                                                                     CGAGCCAGCACCTGTTTCTGGACCTGCCTAAG GGCCTGAATTTTCTGCTCCTGAATTCAAA|||||||||||||||||||||||||||||||||||||||||||||||||||||||||||||||||||||||||||||||||||||||||||||||||||||||||||||||||||||||||||||||||||||||||||||||||||||||||||||||||||||||||||||||||||||||||||||||||||||||||||||||||||||||||||||||||||||||||||||||||||||||||||||||||||||||||||||
chr12 chr12 57898050 59199737 32M59195042F29M4652N15M                                                                                                                                                                                                                                                                                     CGAGCCAGCACCTGTTTCTGGACCTGCCTAAG GGCCTGAATTTTCTGCTCCTGAATTCAAA|||||||||||||||||||||||||||||||||||||||||||||||||||||||||||||||||||||||||||||||||||||||||||||||||||||||||||||||||||||||||||||||||||||||||||||||||||||||||||||||||||||||||||||||||||||||||||||||||||||||||||||||||||||||||||||||||||||||||||||||||||||||||||||||||||||||||||||
chr12 chr12 57898050 59199737 32M59195042F29M4652N15M                                                                                                                                                                                                                                                                                     CGAGCCAGCACCTGTTTCTGGACCTGCCTAAG GGCCTGAATTTTCTGCTCCTGAATTCAAA|||||||||||||||||||||||||||||||||||||||||||||||||||||||||||||||||||||||||||||||||||||||||||||||||||||||||||||||||||||||||||||||||||||||||||||||||||||||||||||||||||||||||||||||||||||||||||||||||||||||||||||||||||||||||||||||||||||||||||||||||||||||||||||||||||||||||||||
chr12 chr12 57898050 59199737 32M59195042F29M4652N15M                                                                                                                                                                                                                                                                                     CGAGCCAGCACCTGTTTCTGGACCTGCCTAAG GGCCTGAATTTTCTGCTCCTGAATTCAAA|||||||||||||||||||||||||||||||||||||||||||||||||||||||||||||||||||||||||||||||||||||||||||||||||||||||||||||||||||||||||||||||||||||||||||||||||||||||||||||||||||||||||||||||||||||||||||||||||||||||||||||||||||||||||||||||||||||||||||||||||||||||||||||||||||||||||||||
chr12 chr12 57898050 59199737 32M59195042F29M4652N15M                                                                                                                                                                                                                                                                                     CGAGCCAGCACCTGTTTCTGGACCTGCCTAAG GGCCTGAATTTTCTGCTCCTGAATTCAAA|||||||||||||||||||||||||||||||||||||||||||||||||||||||||||||||||||||||||||||||||||||||||||||||||||||||||||||||||||||||||||||||||||||||||||||||||||||||||||||||||||||||||||||||||||||||||||||||||||||||||||||||||||||||||||||||||||||||||||||||||||||||||||||||||||||||||||||
chr12 chr12 57898050 59196622 32M59195042F29M1537N15M                                                                                                                                                                                                                                                                                     CGAGCAAGCACCTGTTTCTGGACCTGCCTAAG GGCCTGAATTTTCTGCTCCTGAATTCCAA|||||||||||||||||||||||||||||||||||||||||||||||||||||||||||||||||||||||||||||||||||||||||||||||||||||||||||||||||||||||||||||||||||||||||||||||||||||||||||||||||||||||||||||||||||||||||||||||||||||||||||||||||||||||||||||||||||||||||||||||||||||||||||||||||||||||||||||
chr12 chr12 57898050 59196622 32M59195042F29M1537N15M                                                                                                                                                                                                                                                                                     CGAGCCAGCACCTGTTTCTGGCCCTGCCTAAG GGCCTGAATTTTCTGCTCCTGAATTCAAA|||||||||||||||||||||||||||||||||||||||||||||||||||||||||||||||||||||||||||||||||||||||||||||||||||||||||||||||||||||||||||||||||||||||||||||||||||||||||||||||||||||||||||||||||||||||||||||||||||||||||||||||||||||||||||||||||||||||||||||||||||||||||||||||||||||||||||||
chr12 chr12 57898050 59206210 32M59195042F29M11125N15M                                                                                                                                                                                                                                                                                    CGAGCCAGCACCTGTTTCTGGACCTGCCTAAG GGCCTGAATTTTCTGCTCCTGAATTCAAA|||||||||||||||||||||||||||||||||||||||||||||||||||||||||||||||||||||||||||||||||||||||||||||||||||||||||||||||||||||||||||||||||||||||||||||||||||||||||||||||||||||||||||||||||||||||||||||||||||||||||||||||||||||||||||||||||||||||||||||||||||||||||||||||||||||||||||||
chr12 chr12 57898050 59196622 32M59195042F29M1537N15M                                                                                                                                                                                                                                                                                     CGAGCCAGCACCTGTTTCTGGACCTGCCTAAG GGCCTGAATTTTCTGCTCCTGAAGTCAAA|||||||||||||||||||||||||||||||||||||||||||||||||||||||||||||||||||||||||||||||||||||||||||||||||||||||||||||||||||||||||||||||||||||||||||||||||||||||||||||||||||||||||||||||||||||||||||||||||||||||||||||||||||||||||||||||||||||||||||||||||||||||||||||||||||||||||||||
chr12 chr12 57898050 59196622 32M59195042F29M1537N15M                                                                                                                                                                                                                                                                                     CGAGCCAGCACCTGTTTCTGGACCTGCCTAAG GGCCTGAATTTTCTGCTCCTGAATTCAAA|||||||||||||||||||||||||||||||||||||||||||||||||||||||||||||||||||||||||||||||||||||||||||||||||||||||||||||||||||||||||||||||||||||||||||||||||||||||||||||||||||||||||||||||||||||||||||||||||||||||||||||||||||||||||||||||||||||||||||||||||||||||||||||||||||||||||||||
chr12 chr12 57898051 59196623 31M59195042F29M1537N16M                                                                                                                                                                                                                                                                                      GAGCCAGCACCTGTTTCTGGACCTGCCTAAG GGCCTGAATTTTCTGCTCCTGAATTCAAA|||||||||||||||||||||||||||||||||||||||||||||||||||||||||||||||||||||||||||||||||||||||||||||||||||||||||||||||||||||||||||||||||||||||||||||||||||||||||||||||||||||||||||||||||||||||||||||||||||||||||||||||||||||||||||||||||||||||||||||||||||||||||||||||||||||||||||||
chr12 chr12 57898052 59196702 30M59195042F29M1615N17M                                                                                                                                                                                                                                                                                       AGCCAGCACCTGTTTCTGGACCTGCCTAAG GGCCTGAATTTTCTGCTCCTGAATTCAAA|||||||||||||||||||||||||||||||||||||||||||||||||||||||||||||||||||||||||||||||||||||||||||||||||||||||||||||||||||||||||||||||||||||||||||||||||||||||||||||||||||||||||||||||||||||||||||||||||||||||||||||||||||||||||||||||||||||||||||||||||||||||||||||||||||||||||||||
chr12 chr12 57898052 59196729 30M59195042F29M1642N17M                                                                                                                                                                                                                                                                                       AGCCAGCACCTGTTTCTGGACCTGCCTAAG GGCCTGAATTTTCTGCTCCTGAATTCAAA|||||||||||||||||||||||||||||||||||||||||||||||||||||||||||||||||||||||||||||||||||||||||||||||||||||||||||||||||||||||||||||||||||||||||||||||||||||||||||||||||||||||||||||||||||||||||||||||||||||||||||||||||||||||||||||||||||||||||||||||||||||||||||||||||||||||||||||
chr12 chr12 57898052 59199739 30M59195042F29M4652N17M                                                                                                                                                                                                                                                                                       AGCCAGCACCTGTTTCTGGACCTGCCTAAG GGCCTGAATTTTCTGCTCCTGAATTCAAA|||||||||||||||||||||||||||||||||||||||||||||||||||||||||||||||||||||||||||||||||||||||||||||||||||||||||||||||||||||||||||||||||||||||||||||||||||||||||||||||||||||||||||||||||||||||||||||||||||||||||||||||||||||||||||||||||||||||||||||||||||||||||||||||||||||||||||||
chr12 chr12 57898052 59196624 30M59195042F29M1537N17M                                                                                                                                                                                                                                                                                       AGCCAGCACCTGTTTCTGGACCTGCCTAAG GGCCTGAATTTTCTGCTCCTGAATTCAAA|||||||||||||||||||||||||||||||||||||||||||||||||||||||||||||||||||||||||||||||||||||||||||||||||||||||||||||||||||||||||||||||||||||||||||||||||||||||||||||||||||||||||||||||||||||||||||||||||||||||||||||||||||||||||||||||||||||||||||||||||||||||||||||||||||||||||||||
chr12 chr12 57898052 59196624 30M59195042F29M1537N17M                                                                                                                                                                                                                                                                                       AGCCAGCACCTGTTTCTGGACCTGCCTAAG GGCCTGAATTTTCTGCTCCTGAATTCAAA|||||||||||||||||||||||||||||||||||||||||||||||||||||||||||||||||||||||||||||||||||||||||||||||||||||||||||||||||||||||||||||||||||||||||||||||||||||||||||||||||||||||||||||||||||||||||||||||||||||||||||||||||||||||||||||||||||||||||||||||||||||||||||||||||||||||||||||
chr12 chr12 57898052 59199739 30M59195042F29M4652N17M                                                                                                                                                                                                                                                                                       AGCCAGCACCTGTTTCTGGACCTGCCTAAG GGCCTGAATTTTCTGCTCCTGAATTCAAA|||||||||||||||||||||||||||||||||||||||||||||||||||||||||||||||||||||||||||||||||||||||||||||||||||||||||||||||||||||||||||||||||||||||||||||||||||||||||||||||||||||||||||||||||||||||||||||||||||||||||||||||||||||||||||||||||||||||||||||||||||||||||||||||||||||||||||||
chr12 chr12 57898053 59196625 29M59195042F29M1537N18M                                                                                                                                                                                                                                                                                        GCCAGCACCTGTTTCTGGACCTGCCTAAG GGCCTGAATTTTCTGCTCCTGAATTCAAA|||||||||||||||||||||||||||||||||||||||||||||||||||||||||||||||||||||||||||||||||||||||||||||||||||||||||||||||||||||||||||||||||||||||||||||||||||||||||||||||||||||||||||||||||||||||||||||||||||||||||||||||||||||||||||||||||||||||||||||||||||||||||||||||||||||||||||||
chr12 chr12 57898053 59196625 29M59195042F29M1537N18M                                                                                                                                                                                                                                                                                        GCCAGCACCTGTTTCTGGACCTGCCTAAG GGCCTGAATTTTCTGCTCCTGAATTCAAA|||||||||||||||||||||||||||||||||||||||||||||||||||||||||||||||||||||||||||||||||||||||||||||||||||||||||||||||||||||||||||||||||||||||||||||||||||||||||||||||||||||||||||||||||||||||||||||||||||||||||||||||||||||||||||||||||||||||||||||||||||||||||||||||||||||||||||||
chr12 chr12 57898053 59196625 29M59195042F29M1537N18M                                                                                                                                                                                                                                                                                        GCCAGCACCTGTTTCTGGACCTGCCTAAG GGCCTGAATTTTCTGCTCCTGAATTCAAA|||||||||||||||||||||||||||||||||||||||||||||||||||||||||||||||||||||||||||||||||||||||||||||||||||||||||||||||||||||||||||||||||||||||||||||||||||||||||||||||||||||||||||||||||||||||||||||||||||||||||||||||||||||||||||||||||||||||||||||||||||||||||||||||||||||||||||||
chr12 chr12 57898053 59199740 29M59195042F29M4652N18M                                                                                                                                                                                                                                                                                        GCCAGCACCTGTTTCTGGACCTGCCTAAG GGCCTGAATTTTCTGCTCCTGAATTCAAA|||||||||||||||||||||||||||||||||||||||||||||||||||||||||||||||||||||||||||||||||||||||||||||||||||||||||||||||||||||||||||||||||||||||||||||||||||||||||||||||||||||||||||||||||||||||||||||||||||||||||||||||||||||||||||||||||||||||||||||||||||||||||||||||||||||||||||||
chr12 chr12 57898053 59196625 29M59195042F29M1537N18M                                                                                                                                                                                                                                                                                        GCCAGCACCTGTTTCTGGACCTGCCTAAG GGCCTGAATTTTCTGCTCCTGAATTCAAA|||||||||||||||||||||||||||||||||||||||||||||||||||||||||||||||||||||||||||||||||||||||||||||||||||||||||||||||||||||||||||||||||||||||||||||||||||||||||||||||||||||||||||||||||||||||||||||||||||||||||||||||||||||||||||||||||||||||||||||||||||||||||||||||||||||||||||||
chr12 chr12 57898053 59196625 29M59195042F29M1537N18M                                                                                                                                                                                                                                                                                        GCCAGCACCTGTTTCTGGACCTGCCTAAG GGCCTGAATTTTCTGCTCCTGAATTCAAA|||||||||||||||||||||||||||||||||||||||||||||||||||||||||||||||||||||||||||||||||||||||||||||||||||||||||||||||||||||||||||||||||||||||||||||||||||||||||||||||||||||||||||||||||||||||||||||||||||||||||||||||||||||||||||||||||||||||||||||||||||||||||||||||||||||||||||||
chr12 chr12 57898053 59196625 29M59195042F29M1537N18M                                                                                                                                                                                                                                                                                        GCCAGCACCTGTTTCTGGACCTGCCTAAG GGCCTGAATTTTCTGCTCCTGAATTCAAA|||||||||||||||||||||||||||||||||||||||||||||||||||||||||||||||||||||||||||||||||||||||||||||||||||||||||||||||||||||||||||||||||||||||||||||||||||||||||||||||||||||||||||||||||||||||||||||||||||||||||||||||||||||||||||||||||||||||||||||||||||||||||||||||||||||||||||||
chr12 chr12 57898053 59196625 29M59195042F29M1537N18M                                                                                                                                                                                                                                                                                        GCCAGCACCTGTTTCTGGACCTGCCTAAG GGCCTGAATTTTCTGCTCCTGAATTCAAA|||||||||||||||||||||||||||||||||||||||||||||||||||||||||||||||||||||||||||||||||||||||||||||||||||||||||||||||||||||||||||||||||||||||||||||||||||||||||||||||||||||||||||||||||||||||||||||||||||||||||||||||||||||||||||||||||||||||||||||||||||||||||||||||||||||||||||||
chr12 chr12 57898053 59196625 29M59195042F29M1537N18M                                                                                                                                                                                                                                                                                        GCCAGCACCTGTTTCTGGACCTGCCTAAG GGCCTGAATTTTCTGCTCCTGAATTCAAA|||||||||||||||||||||||||||||||||||||||||||||||||||||||||||||||||||||||||||||||||||||||||||||||||||||||||||||||||||||||||||||||||||||||||||||||||||||||||||||||||||||||||||||||||||||||||||||||||||||||||||||||||||||||||||||||||||||||||||||||||||||||||||||||||||||||||||||
chr12 chr12 57898053 59196625 29M59195042F29M1537N18M                                                                                                                                                                                                                                                                                        GCCAGCACCTGTTTCTGGACCTGCCTAAG GGCCTGAATTTTCTGCTCCTGAATTCAAA|||||||||||||||||||||||||||||||||||||||||||||||||||||||||||||||||||||||||||||||||||||||||||||||||||||||||||||||||||||||||||||||||||||||||||||||||||||||||||||||||||||||||||||||||||||||||||||||||||||||||||||||||||||||||||||||||||||||||||||||||||||||||||||||||||||||||||||
chr12 chr12 57898053 59199740 29M59195042F29M4652N18M                                                                                                                                                                                                                                                                                        GCCAGCACCTGTTTCTGGACCTGCCTAAG GGCCTGAATTTTCTGCTCCTGAATTCAAA|||||||||||||||||||||||||||||||||||||||||||||||||||||||||||||||||||||||||||||||||||||||||||||||||||||||||||||||||||||||||||||||||||||||||||||||||||||||||||||||||||||||||||||||||||||||||||||||||||||||||||||||||||||||||||||||||||||||||||||||||||||||||||||||||||||||||||||
chr12 chr12 57898053 59206213 29M59195042F29M11125N18M                                                                                                                                                                                                                                                                                       GCCAGCACCTGTTTCTGGACCTGCCTAAG GGCCTGAATTTTCTGCTCCTGAATTCAAA|||||||||||||||||||||||||||||||||||||||||||||||||||||||||||||||||||||||||||||||||||||||||||||||||||||||||||||||||||||||||||||||||||||||||||||||||||||||||||||||||||||||||||||||||||||||||||||||||||||||||||||||||||||||||||||||||||||||||||||||||||||||||||||||||||||||||||||
chr12 chr12 57898053 59199740 29M59195042F29M4652N18M                                                                                                                                                                                                                                                                                        GCCAGCACCTGTTTCTGGACCTGCCTAAG GGCCTGAATTTTCTGCTCCTGAATTCAAA|||||||||||||||||||||||||||||||||||||||||||||||||||||||||||||||||||||||||||||||||||||||||||||||||||||||||||||||||||||||||||||||||||||||||||||||||||||||||||||||||||||||||||||||||||||||||||||||||||||||||||||||||||||||||||||||||||||||||||||||||||||||||||||||||||||||||||||
chr12 chr12 57898053 59199740 29M59195042F29M4652N18M                                                                                                                                                                                                                                                                                        GCCATCACTTGTTTCTGGACCTGCCTAAG GGCCTGAATTTTCTGCTCCTGAATTCAAA|||||||||||||||||||||||||||||||||||||||||||||||||||||||||||||||||||||||||||||||||||||||||||||||||||||||||||||||||||||||||||||||||||||||||||||||||||||||||||||||||||||||||||||||||||||||||||||||||||||||||||||||||||||||||||||||||||||||||||||||||||||||||||||||||||||||||||||
chr12 chr12 57898053 59206213 29M59195042F29M11125N18M                                                                                                                                                                                                                                                                                       GCCAGCACCTGTTTCTGGACCTGCCTAAG GGCCTGAATTTTCTGCTCCTGAATTCAAA|||||||||||||||||||||||||||||||||||||||||||||||||||||||||||||||||||||||||||||||||||||||||||||||||||||||||||||||||||||||||||||||||||||||||||||||||||||||||||||||||||||||||||||||||||||||||||||||||||||||||||||||||||||||||||||||||||||||||||||||||||||||||||||||||||||||||||||
chr12 chr12 57898053 59196625 29M59195042F29M1537N18M                                                                                                                                                                                                                                                                                        GCCAGCACCTGTTTCTGGACCTCCCTAAG GGCCTGAATTTTCTGCTCCTGAATTCAAA|||||||||||||||||||||||||||||||||||||||||||||||||||||||||||||||||||||||||||||||||||||||||||||||||||||||||||||||||||||||||||||||||||||||||||||||||||||||||||||||||||||||||||||||||||||||||||||||||||||||||||||||||||||||||||||||||||||||||||||||||||||||||||||||||||||||||||||
chr12 chr12 57898053 59199740 29M59195042F29M4652N18M                                                                                                                                                                                                                                                                                        GCCAGCACCTGTTTCTGGACCTGCCTAAG GGCCTGAATTTTCTGCTCCTGAATTCAAA|||||||||||||||||||||||||||||||||||||||||||||||||||||||||||||||||||||||||||||||||||||||||||||||||||||||||||||||||||||||||||||||||||||||||||||||||||||||||||||||||||||||||||||||||||||||||||||||||||||||||||||||||||||||||||||||||||||||||||||||||||||||||||||||||||||||||||||
chr12 chr12 57898053 59199740 29M59195042F29M4652N18M                                                                                                                                                                                                                                                                                        GCCAGCACCTGTTTCTGGACCTGCCTAAG GGCCTGAATTTTCTGCTCCTGAATTCAAA|||||||||||||||||||||||||||||||||||||||||||||||||||||||||||||||||||||||||||||||||||||||||||||||||||||||||||||||||||||||||||||||||||||||||||||||||||||||||||||||||||||||||||||||||||||||||||||||||||||||||||||||||||||||||||||||||||||||||||||||||||||||||||||||||||||||||||||
chr12 chr12 57898053 59196625 29M59195042F29M1537N18M                                                                                                                                                                                                                                                                                        GCCAGCACCTGTTTCTGGACCTGCCTAAG GGCCTGAATTTTCTGCTCCTGAATTCAAA|||||||||||||||||||||||||||||||||||||||||||||||||||||||||||||||||||||||||||||||||||||||||||||||||||||||||||||||||||||||||||||||||||||||||||||||||||||||||||||||||||||||||||||||||||||||||||||||||||||||||||||||||||||||||||||||||||||||||||||||||||||||||||||||||||||||||||||
chr12 chr12 57898053 59199740 29M59195042F29M4652N18M                                                                                                                                                                                                                                                                                        GCCATCACTTGTTTCTGGACCTGCCTAAG GGCCTGAATTTTCTGCTCCTGAATTCAAA|||||||||||||||||||||||||||||||||||||||||||||||||||||||||||||||||||||||||||||||||||||||||||||||||||||||||||||||||||||||||||||||||||||||||||||||||||||||||||||||||||||||||||||||||||||||||||||||||||||||||||||||||||||||||||||||||||||||||||||||||||||||||||||||||||||||||||||
chr12 chr12 57898053 59199740 29M59195042F29M4652N18M                                                                                                                                                                                                                                                                                        GCCAGCACCTGTTTCTGGACCTGCCTAAG GGCCTGAATTTTCTGCTCCTGAATTCAAA|||||||||||||||||||||||||||||||||||||||||||||||||||||||||||||||||||||||||||||||||||||||||||||||||||||||||||||||||||||||||||||||||||||||||||||||||||||||||||||||||||||||||||||||||||||||||||||||||||||||||||||||||||||||||||||||||||||||||||||||||||||||||||||||||||||||||||||
chr12 chr12 57898053 59196625 29M59195042F29M1537N18M                                                                                                                                                                                                                                                                                        GCCAGCACTTGTTTCTGGACCTGCCTAAG GGCCTGAATTTTCTGCTCCTGAATTCAAA|||||||||||||||||||||||||||||||||||||||||||||||||||||||||||||||||||||||||||||||||||||||||||||||||||||||||||||||||||||||||||||||||||||||||||||||||||||||||||||||||||||||||||||||||||||||||||||||||||||||||||||||||||||||||||||||||||||||||||||||||||||||||||||||||||||||||||||
chr12 chr12 57898053 59199740 29M59195042F29M4652N18M                                                                                                                                                                                                                                                                                        GCCAGCACCTGTTTCTGGACCTGCCTAAG GGCCTGAATTTTCTGCTCCTGAATTCCAA|||||||||||||||||||||||||||||||||||||||||||||||||||||||||||||||||||||||||||||||||||||||||||||||||||||||||||||||||||||||||||||||||||||||||||||||||||||||||||||||||||||||||||||||||||||||||||||||||||||||||||||||||||||||||||||||||||||||||||||||||||||||||||||||||||||||||||||
chr12 chr12 57898053 59196625 29M59195042F29M1537N18M                                                                                                                                                                                                                                                                                        GCCAGCACCTGTTTCTGGACCTGCCTAAG GGCCTGAATTTTCTGCTCCTGAATTCAAA|||||||||||||||||||||||||||||||||||||||||||||||||||||||||||||||||||||||||||||||||||||||||||||||||||||||||||||||||||||||||||||||||||||||||||||||||||||||||||||||||||||||||||||||||||||||||||||||||||||||||||||||||||||||||||||||||||||||||||||||||||||||||||||||||||||||||||||
chr12 chr12 57898053 59196703 29M59195042F29M1615N18M                                                                                                                                                                                                                                                                                        GCCAGCACCTGTTTCTGGACCTGCCTAAG GGCCTGAATTTTCTGCTCCTGAATTCAAA|||||||||||||||||||||||||||||||||||||||||||||||||||||||||||||||||||||||||||||||||||||||||||||||||||||||||||||||||||||||||||||||||||||||||||||||||||||||||||||||||||||||||||||||||||||||||||||||||||||||||||||||||||||||||||||||||||||||||||||||||||||||||||||||||||||||||||||
chr12 chr12 57898053 59196625 29M59195042F29M1537N18M                                                                                                                                                                                                                                                                                        GCCAGCACCTGTTTCTGGACCTGCCTAAG GGCCTGAATTTTCTGCTCCTGAATTCAAA|||||||||||||||||||||||||||||||||||||||||||||||||||||||||||||||||||||||||||||||||||||||||||||||||||||||||||||||||||||||||||||||||||||||||||||||||||||||||||||||||||||||||||||||||||||||||||||||||||||||||||||||||||||||||||||||||||||||||||||||||||||||||||||||||||||||||||||
chr12 chr12 57898053 59199740 29M59195042F29M4652N18M                                                                                                                                                                                                                                                                                        GCCAGCACCTGTTTCTGGACCTGCCTAAG GGCCTGAATTTTCTGCTCCTGAATTCAAA|||||||||||||||||||||||||||||||||||||||||||||||||||||||||||||||||||||||||||||||||||||||||||||||||||||||||||||||||||||||||||||||||||||||||||||||||||||||||||||||||||||||||||||||||||||||||||||||||||||||||||||||||||||||||||||||||||||||||||||||||||||||||||||||||||||||||||||
chr12 chr12 57898054 59196626 28M59195042F29M1537N19M                                                                                                                                                                                                                                                                                         CCAGCACCTGTTTCTGGACCTGCCTAAG GGCCTGAATTTTCTGCTCCTGAATTCAAA|||||||||||||||||||||||||||||||||||||||||||||||||||||||||||||||||||||||||||||||||||||||||||||||||||||||||||||||||||||||||||||||||||||||||||||||||||||||||||||||||||||||||||||||||||||||||||||||||||||||||||||||||||||||||||||||||||||||||||||||||||||||||||||||||||||||||||||
chr12 chr12 57898054 59196626 28M59195042F29M1537N19M                                                                                                                                                                                                                                                                                         CCAGCACCTGTTTCTGGACCTGCCTAAG GGCCTGAATTTTCTGCTCCTGAATTCAAA|||||||||||||||||||||||||||||||||||||||||||||||||||||||||||||||||||||||||||||||||||||||||||||||||||||||||||||||||||||||||||||||||||||||||||||||||||||||||||||||||||||||||||||||||||||||||||||||||||||||||||||||||||||||||||||||||||||||||||||||||||||||||||||||||||||||||||||
chr12 chr12 57898054 59196626 28M59195042F29M1537N19M                                                                                                                                                                                                                                                                                         CCAGCACCTGTTTCTGGACCTGCCTAAG GGCCTGAATTTTCTGCTCCTGAATTCAAA|||||||||||||||||||||||||||||||||||||||||||||||||||||||||||||||||||||||||||||||||||||||||||||||||||||||||||||||||||||||||||||||||||||||||||||||||||||||||||||||||||||||||||||||||||||||||||||||||||||||||||||||||||||||||||||||||||||||||||||||||||||||||||||||||||||||||||||
chr12 chr12 57898054 59196626 28M59195042F29M1537N19M                                                                                                                                                                                                                                                                                         CCAGCACCTGTTTCTGGACCTGCCTAAG GGCCTGAATTTTCTGCTCCTGAATTCAAA|||||||||||||||||||||||||||||||||||||||||||||||||||||||||||||||||||||||||||||||||||||||||||||||||||||||||||||||||||||||||||||||||||||||||||||||||||||||||||||||||||||||||||||||||||||||||||||||||||||||||||||||||||||||||||||||||||||||||||||||||||||||||||||||||||||||||||||
chr12 chr12 57898054 59196626 28M59195042F29M1537N19M                                                                                                                                                                                                                                                                                         CCAGCACCTTTTTCTGGACCTGCCTAAG GGCCTGAATTTTCTGCTCCTGAATTCAAA|||||||||||||||||||||||||||||||||||||||||||||||||||||||||||||||||||||||||||||||||||||||||||||||||||||||||||||||||||||||||||||||||||||||||||||||||||||||||||||||||||||||||||||||||||||||||||||||||||||||||||||||||||||||||||||||||||||||||||||||||||||||||||||||||||||||||||||
chr12 chr12 57898054 59199741 28M59195042F29M4652N19M                                                                                                                                                                                                                                                                                         CCAGCACCTGTTTCTGGACCTGCCTAAG GGCCTGAATTTTCTGCTCCTGAATTCAAA|||||||||||||||||||||||||||||||||||||||||||||||||||||||||||||||||||||||||||||||||||||||||||||||||||||||||||||||||||||||||||||||||||||||||||||||||||||||||||||||||||||||||||||||||||||||||||||||||||||||||||||||||||||||||||||||||||||||||||||||||||||||||||||||||||||||||||||
chr12 chr12 57898054 59199741 28M59195042F29M4652N19M                                                                                                                                                                                                                                                                                         CCAGCACCTGTTTCTGGACCTGCCTAAG GGCCTGAATTTTCTGCTCCTGAATTCAAA|||||||||||||||||||||||||||||||||||||||||||||||||||||||||||||||||||||||||||||||||||||||||||||||||||||||||||||||||||||||||||||||||||||||||||||||||||||||||||||||||||||||||||||||||||||||||||||||||||||||||||||||||||||||||||||||||||||||||||||||||||||||||||||||||||||||||||||
chr12 chr12 57898054 59199741 28M59195042F29M4652N19M                                                                                                                                                                                                                                                                                         CCAGCACCTGTTTCTGGACCTGCCTAAG GGCCTGAATTTTCTGCTCCTGAATTCAAA|||||||||||||||||||||||||||||||||||||||||||||||||||||||||||||||||||||||||||||||||||||||||||||||||||||||||||||||||||||||||||||||||||||||||||||||||||||||||||||||||||||||||||||||||||||||||||||||||||||||||||||||||||||||||||||||||||||||||||||||||||||||||||||||||||||||||||||
chr12 chr12 57898054 59199741 28M59195042F29M4652N19M                                                                                                                                                                                                                                                                                         CCAGCACCTGTTTCTGGACCTGCCTAAG GGCCTGAATTTTCTGCTCCTGAATTCAAA|||||||||||||||||||||||||||||||||||||||||||||||||||||||||||||||||||||||||||||||||||||||||||||||||||||||||||||||||||||||||||||||||||||||||||||||||||||||||||||||||||||||||||||||||||||||||||||||||||||||||||||||||||||||||||||||||||||||||||||||||||||||||||||||||||||||||||||
chr12 chr12 57898054 59196626 28M59195042F29M1537N19M                                                                                                                                                                                                                                                                                         CCAGCACCTGTTTCTGGACCTGCCTAAG GGCCTGAATTTTCTGCTCCTGAATTCAAA|||||||||||||||||||||||||||||||||||||||||||||||||||||||||||||||||||||||||||||||||||||||||||||||||||||||||||||||||||||||||||||||||||||||||||||||||||||||||||||||||||||||||||||||||||||||||||||||||||||||||||||||||||||||||||||||||||||||||||||||||||||||||||||||||||||||||||||
chr12 chr12 57898054 59196626 28M59195042F29M1537N19M                                                                                                                                                                                                                                                                                         CCCGCACCTGTTTCTGGACCTGCCTAAG GGCCTGAATTTTCTGCTCCTGAATTCAAA|||||||||||||||||||||||||||||||||||||||||||||||||||||||||||||||||||||||||||||||||||||||||||||||||||||||||||||||||||||||||||||||||||||||||||||||||||||||||||||||||||||||||||||||||||||||||||||||||||||||||||||||||||||||||||||||||||||||||||||||||||||||||||||||||||||||||||||
chr12 chr12 57898054 59199741 28M59195042F29M4652N19M                                                                                                                                                                                                                                                                                         CCAGAACCTGTTTCTGGACCTGCCTAAG GGCCTGAATTTTCTGCTCCTGAATTCAAA|||||||||||||||||||||||||||||||||||||||||||||||||||||||||||||||||||||||||||||||||||||||||||||||||||||||||||||||||||||||||||||||||||||||||||||||||||||||||||||||||||||||||||||||||||||||||||||||||||||||||||||||||||||||||||||||||||||||||||||||||||||||||||||||||||||||||||||
chr12 chr12 57898054 59196626 28M59195042F29M1537N19M                                                                                                                                                                                                                                                                                         CCAGCACCTGTTTCTGGACCTGCCTAAG GGCCTGAATTTTCTGCTCCTGAATTCAAA|||||||||||||||||||||||||||||||||||||||||||||||||||||||||||||||||||||||||||||||||||||||||||||||||||||||||||||||||||||||||||||||||||||||||||||||||||||||||||||||||||||||||||||||||||||||||||||||||||||||||||||||||||||||||||||||||||||||||||||||||||||||||||||||||||||||||||||
chr12 chr12 57898054 59196626 28M59195042F29M1537N19M                                                                                                                                                                                                                                                                                         CCAGCACCTGTTTCTGGACCTGCCTAAG GGCCTGAATTTTCTGCTCCTGAATTCAAA|||||||||||||||||||||||||||||||||||||||||||||||||||||||||||||||||||||||||||||||||||||||||||||||||||||||||||||||||||||||||||||||||||||||||||||||||||||||||||||||||||||||||||||||||||||||||||||||||||||||||||||||||||||||||||||||||||||||||||||||||||||||||||||||||||||||||||||
chr12 chr12 57898054 59196626 28M59195042F29M1537N19M                                                                                                                                                                                                                                                                                         CCAGCACCTGTTTCTGGACCTGCCTAAG GGCCTGAATTTTCTGCTCCTGAATTCAAA|||||||||||||||||||||||||||||||||||||||||||||||||||||||||||||||||||||||||||||||||||||||||||||||||||||||||||||||||||||||||||||||||||||||||||||||||||||||||||||||||||||||||||||||||||||||||||||||||||||||||||||||||||||||||||||||||||||||||||||||||||||||||||||||||||||||||||||
chr12 chr12 57898054 59196626 28M59195042F29M1537N19M                                                                                                                                                                                                                                                                                         CCAGCACCTGTTTCTGGACCTGCCTAAG GGCCTGAATTTTCTGCTCCTGAATTCAAA|||||||||||||||||||||||||||||||||||||||||||||||||||||||||||||||||||||||||||||||||||||||||||||||||||||||||||||||||||||||||||||||||||||||||||||||||||||||||||||||||||||||||||||||||||||||||||||||||||||||||||||||||||||||||||||||||||||||||||||||||||||||||||||||||||||||||||||
chr12 chr12 57898055 59196627 27M59195042F29M1537N20M                                                                                                                                                                                                                                                                                          CAGCACCTGTTTCTGGACCTGCCTAAG GGCCTGAATTTTCTGCTCCTGAATTCAAA|||||||||||||||||||||||||||||||||||||||||||||||||||||||||||||||||||||||||||||||||||||||||||||||||||||||||||||||||||||||||||||||||||||||||||||||||||||||||||||||||||||||||||||||||||||||||||||||||||||||||||||||||||||||||||||||||||||||||||||||||||||||||||||||||||||||||||||
chr12 chr12 57898055 59196627 27M59195042F29M1537N20M                                                                                                                                                                                                                                                                                          CAGCACCTGTTTCTGGACCTGCCTAAG GGCCTGAATTTTCTGCTCCTGAATTCAAA|||||||||||||||||||||||||||||||||||||||||||||||||||||||||||||||||||||||||||||||||||||||||||||||||||||||||||||||||||||||||||||||||||||||||||||||||||||||||||||||||||||||||||||||||||||||||||||||||||||||||||||||||||||||||||||||||||||||||||||||||||||||||||||||||||||||||||||
chr12 chr12 57898055 59199742 27M59195042F29M4652N20M                                                                                                                                                                                                                                                                                          CAGCACCTGTTTCTGGACCTGCCTAAG GGCCTGAATTTTCTGCTCCTGAATTCAAA|||||||||||||||||||||||||||||||||||||||||||||||||||||||||||||||||||||||||||||||||||||||||||||||||||||||||||||||||||||||||||||||||||||||||||||||||||||||||||||||||||||||||||||||||||||||||||||||||||||||||||||||||||||||||||||||||||||||||||||||||||||||||||||||||||||||||||||
chr12 chr12 57898055 59199742 27M59195042F29M4652N20M                                                                                                                                                                                                                                                                                          CAGCACCTGTTTCTGGACCTGCCTAAG GGCCTGAATTTTCTGCTCCTGAATTCAAA|||||||||||||||||||||||||||||||||||||||||||||||||||||||||||||||||||||||||||||||||||||||||||||||||||||||||||||||||||||||||||||||||||||||||||||||||||||||||||||||||||||||||||||||||||||||||||||||||||||||||||||||||||||||||||||||||||||||||||||||||||||||||||||||||||||||||||||
chr12 chr12 57898055 59199742 27M59195042F29M4652N20M                                                                                                                                                                                                                                                                                          CAGCACCTGTTTCTGGACCTGCCTAAG GGCCTGAATTTTCTGCTCCTGAATTCAAA|||||||||||||||||||||||||||||||||||||||||||||||||||||||||||||||||||||||||||||||||||||||||||||||||||||||||||||||||||||||||||||||||||||||||||||||||||||||||||||||||||||||||||||||||||||||||||||||||||||||||||||||||||||||||||||||||||||||||||||||||||||||||||||||||||||||||||||
chr12 chr12 57898056 59196628 26M59195042F29M1537N21M                                                                                                                                                                                                                                                                                           AGCACCTGTTTCTGGACCTGCCTAAG GGCCTGAATTTTCTGCTCCTGAATTCAAA|||||||||||||||||||||||||||||||||||||||||||||||||||||||||||||||||||||||||||||||||||||||||||||||||||||||||||||||||||||||||||||||||||||||||||||||||||||||||||||||||||||||||||||||||||||||||||||||||||||||||||||||||||||||||||||||||||||||||||||||||||||||||||||||||||||||||||||
chr12 chr12 57898056 59206216 26M59195042F29M11125N21M                                                                                                                                                                                                                                                                                          AGCACCTGTTTCTGGACCTGCCTAAG GGCCTGAATTTTCTGCTCCTGAATTCAAA|||||||||||||||||||||||||||||||||||||||||||||||||||||||||||||||||||||||||||||||||||||||||||||||||||||||||||||||||||||||||||||||||||||||||||||||||||||||||||||||||||||||||||||||||||||||||||||||||||||||||||||||||||||||||||||||||||||||||||||||||||||||||||||||||||||||||||||
chr12 chr12 57898056 59196628 26M59195042F29M1537N21M                                                                                                                                                                                                                                                                                           AGCACCTGTTTCTGGACCTGCCTAAG GGCCTGAATTTTCTGCTCCTGAATTCAAA|||||||||||||||||||||||||||||||||||||||||||||||||||||||||||||||||||||||||||||||||||||||||||||||||||||||||||||||||||||||||||||||||||||||||||||||||||||||||||||||||||||||||||||||||||||||||||||||||||||||||||||||||||||||||||||||||||||||||||||||||||||||||||||||||||||||||||||
chr12 chr12 57898056 59199743 26M59195042F29M4652N21M                                                                                                                                                                                                                                                                                           AGCACCTGTTTCTGGACCTGCCTAAG GGCCTGAATTTTCTGCTCCTGAATTCAAA|||||||||||||||||||||||||||||||||||||||||||||||||||||||||||||||||||||||||||||||||||||||||||||||||||||||||||||||||||||||||||||||||||||||||||||||||||||||||||||||||||||||||||||||||||||||||||||||||||||||||||||||||||||||||||||||||||||||||||||||||||||||||||||||||||||||||||||
chr12 chr12 57898056 59196628 26M59195042F29M1537N21M                                                                                                                                                                                                                                                                                           AGCACCTGTTTCTGGACCTGCCTAAG GGCCTGAATTTTCTGCTCCTGAATTCAAA|||||||||||||||||||||||||||||||||||||||||||||||||||||||||||||||||||||||||||||||||||||||||||||||||||||||||||||||||||||||||||||||||||||||||||||||||||||||||||||||||||||||||||||||||||||||||||||||||||||||||||||||||||||||||||||||||||||||||||||||||||||||||||||||||||||||||||||
chr12 chr12 57898057 59196629 25M59195042F29M1537N22M                                                                                                                                                                                                                                                                                            GCACCTGTTTCTGGACCTGCCTAAG GGCCTGAATTTTCTGCTCCTGAATTCAAA|||||||||||||||||||||||||||||||||||||||||||||||||||||||||||||||||||||||||||||||||||||||||||||||||||||||||||||||||||||||||||||||||||||||||||||||||||||||||||||||||||||||||||||||||||||||||||||||||||||||||||||||||||||||||||||||||||||||||||||||||||||||||||||||||||||||||||||
chr12 chr12 57898057 59196629 25M59195042F29M1537N22M                                                                                                                                                                                                                                                                                            GCACCTGTTTCTGGACCTGCCTAAG GGCCTGAATTTTCTGCTCCTGAATTCAAA|||||||||||||||||||||||||||||||||||||||||||||||||||||||||||||||||||||||||||||||||||||||||||||||||||||||||||||||||||||||||||||||||||||||||||||||||||||||||||||||||||||||||||||||||||||||||||||||||||||||||||||||||||||||||||||||||||||||||||||||||||||||||||||||||||||||||||||
chr12 chr12 57898057 59199744 25M59195042F29M4652N22M                                                                                                                                                                                                                                                                                            GCACCTGTTTCTGGACCTGCCTAAG GGCCTGAATTTTCTGCTCCTGAATTCAAA|||||||||||||||||||||||||||||||||||||||||||||||||||||||||||||||||||||||||||||||||||||||||||||||||||||||||||||||||||||||||||||||||||||||||||||||||||||||||||||||||||||||||||||||||||||||||||||||||||||||||||||||||||||||||||||||||||||||||||||||||||||||||||||||||||||||||||||
chr12 chr12 57898057 59196629 25M59195042F29M1537N22M                                                                                                                                                                                                                                                                                            GCACCTGTTTCTGGACCTGCCTAAG GGCCTGAATTTTCTGCTCCTGAATTCAAA|||||||||||||||||||||||||||||||||||||||||||||||||||||||||||||||||||||||||||||||||||||||||||||||||||||||||||||||||||||||||||||||||||||||||||||||||||||||||||||||||||||||||||||||||||||||||||||||||||||||||||||||||||||||||||||||||||||||||||||||||||||||||||||||||||||||||||||
chr12 chr12 57898057 59196629 25M59195042F29M1537N22M                                                                                                                                                                                                                                                                                            GCACCTGTTTCTGGACCTGCCTAAG GGCCTGAATTTTCTGCTCCTGAATTCAAA|||||||||||||||||||||||||||||||||||||||||||||||||||||||||||||||||||||||||||||||||||||||||||||||||||||||||||||||||||||||||||||||||||||||||||||||||||||||||||||||||||||||||||||||||||||||||||||||||||||||||||||||||||||||||||||||||||||||||||||||||||||||||||||||||||||||||||||
chr12 chr12 57898057 59196629 25M59195042F29M1537N22M                                                                                                                                                                                                                                                                                            GCACCTGTTTCTGGACCTGCCTAAG GGCCTGAATTTTCTGCTCCTGAATTCAAA|||||||||||||||||||||||||||||||||||||||||||||||||||||||||||||||||||||||||||||||||||||||||||||||||||||||||||||||||||||||||||||||||||||||||||||||||||||||||||||||||||||||||||||||||||||||||||||||||||||||||||||||||||||||||||||||||||||||||||||||||||||||||||||||||||||||||||||
chr12 chr12 57898057 59199744 25M59195042F29M4652N22M                                                                                                                                                                                                                                                                                            GCACCTGTTTCTGGACCTGCCTAAG GGCCTGAATTTTCTGCTCCTGAATTCAAA|||||||||||||||||||||||||||||||||||||||||||||||||||||||||||||||||||||||||||||||||||||||||||||||||||||||||||||||||||||||||||||||||||||||||||||||||||||||||||||||||||||||||||||||||||||||||||||||||||||||||||||||||||||||||||||||||||||||||||||||||||||||||||||||||||||||||||||
chr12 chr12 57898057 59196629 25M59195042F29M1537N22M                                                                                                                                                                                                                                                                                            GCACCTGTTTCTGGACCTGCCTAAG GGCCTGAATTTTCTGCTCCTGAATTCAAA|||||||||||||||||||||||||||||||||||||||||||||||||||||||||||||||||||||||||||||||||||||||||||||||||||||||||||||||||||||||||||||||||||||||||||||||||||||||||||||||||||||||||||||||||||||||||||||||||||||||||||||||||||||||||||||||||||||||||||||||||||||||||||||||||||||||||||||
chr12 chr12 57898057 59196629 25M59195042F29M1537N22M                                                                                                                                                                                                                                                                                            GCACCTGTTTCTGGACCTGCCTAAG GGCCTGAATTTTCTGCTCCTGAATTCAAA|||||||||||||||||||||||||||||||||||||||||||||||||||||||||||||||||||||||||||||||||||||||||||||||||||||||||||||||||||||||||||||||||||||||||||||||||||||||||||||||||||||||||||||||||||||||||||||||||||||||||||||||||||||||||||||||||||||||||||||||||||||||||||||||||||||||||||||
chr12 chr12 57898057 59196629 25M59195042F29M1537N22M                                                                                                                                                                                                                                                                                            GCACCTGTTTCTGGACCTGCCTAAG GGCCTGAATTTTCTGCTCCTGAATTCAAA|||||||||||||||||||||||||||||||||||||||||||||||||||||||||||||||||||||||||||||||||||||||||||||||||||||||||||||||||||||||||||||||||||||||||||||||||||||||||||||||||||||||||||||||||||||||||||||||||||||||||||||||||||||||||||||||||||||||||||||||||||||||||||||||||||||||||||||
chr12 chr12 57898057 59199744 25M59195042F29M4652N22M                                                                                                                                                                                                                                                                                            GCACCTGTTTCTGGACCTGCCTAAG GGCCTGAATTTTCTGCTCCTGAATTCAAA|||||||||||||||||||||||||||||||||||||||||||||||||||||||||||||||||||||||||||||||||||||||||||||||||||||||||||||||||||||||||||||||||||||||||||||||||||||||||||||||||||||||||||||||||||||||||||||||||||||||||||||||||||||||||||||||||||||||||||||||||||||||||||||||||||||||||||||
chr12 chr12 57898057 59196629 25M59195042F29M1537N22M                                                                                                                                                                                                                                                                                            GCACCTGTTTCTGGACCTGCCTAAG GGCCTGAATTTTCTGCACCTGAATTCAAA|||||||||||||||||||||||||||||||||||||||||||||||||||||||||||||||||||||||||||||||||||||||||||||||||||||||||||||||||||||||||||||||||||||||||||||||||||||||||||||||||||||||||||||||||||||||||||||||||||||||||||||||||||||||||||||||||||||||||||||||||||||||||||||||||||||||||||||
chr12 chr12 57898058 59196630 24M59195042F29M1537N23M                                                                                                                                                                                                                                                                                             CACCTGTTTCTGGACCTGCCTAAG GGCCTGAATTTTCTGCTCCTGAATTCAAA|||||||||||||||||||||||||||||||||||||||||||||||||||||||||||||||||||||||||||||||||||||||||||||||||||||||||||||||||||||||||||||||||||||||||||||||||||||||||||||||||||||||||||||||||||||||||||||||||||||||||||||||||||||||||||||||||||||||||||||||||||||||||||||||||||||||||||||
chr12 chr12 57898058 59196630 24M59195042F29M1537N23M                                                                                                                                                                                                                                                                                             CACCTGTTTCTGGACCTGCCTAAG GGCCTGAATTTTCTGCTCCTGAATTCAAA|||||||||||||||||||||||||||||||||||||||||||||||||||||||||||||||||||||||||||||||||||||||||||||||||||||||||||||||||||||||||||||||||||||||||||||||||||||||||||||||||||||||||||||||||||||||||||||||||||||||||||||||||||||||||||||||||||||||||||||||||||||||||||||||||||||||||||||
chr12 chr12 57898058 59196630 24M59195042F29M1537N23M                                                                                                                                                                                                                                                                                             CACCTGTTTCTGGACCTGCCTAAG GGCCTGAATTTTCTGCTCCTGAATTCAAA|||||||||||||||||||||||||||||||||||||||||||||||||||||||||||||||||||||||||||||||||||||||||||||||||||||||||||||||||||||||||||||||||||||||||||||||||||||||||||||||||||||||||||||||||||||||||||||||||||||||||||||||||||||||||||||||||||||||||||||||||||||||||||||||||||||||||||||
chr12 chr12 57898058 59199745 24M59195042F29M4652N23M                                                                                                                                                                                                                                                                                             CACCTGTTTCTGGACCTGCCTAAG GGCCTGAATTTTCTGCTCCTGAATTCAAA|||||||||||||||||||||||||||||||||||||||||||||||||||||||||||||||||||||||||||||||||||||||||||||||||||||||||||||||||||||||||||||||||||||||||||||||||||||||||||||||||||||||||||||||||||||||||||||||||||||||||||||||||||||||||||||||||||||||||||||||||||||||||||||||||||||||||||||
chr12 chr12 57898058 59196630 24M59195042F29M1537N23M                                                                                                                                                                                                                                                                                             CACCTGTTTCTGGACCTGCCTAAG GGCCTGAATTTTCTGCTCCTGAATTCAAA|||||||||||||||||||||||||||||||||||||||||||||||||||||||||||||||||||||||||||||||||||||||||||||||||||||||||||||||||||||||||||||||||||||||||||||||||||||||||||||||||||||||||||||||||||||||||||||||||||||||||||||||||||||||||||||||||||||||||||||||||||||||||||||||||||||||||||||
chr12 chr12 57898058 59196630 24M59195042F29M1537N23M                                                                                                                                                                                                                                                                                             CACCTGTTTCTGGACCTGCCTAAG GGCCTGAATTTTCTGCTCCTGAATTCAAA|||||||||||||||||||||||||||||||||||||||||||||||||||||||||||||||||||||||||||||||||||||||||||||||||||||||||||||||||||||||||||||||||||||||||||||||||||||||||||||||||||||||||||||||||||||||||||||||||||||||||||||||||||||||||||||||||||||||||||||||||||||||||||||||||||||||||||||
chr12 chr12 57898058 59196630 24M59195042F29M1537N23M                                                                                                                                                                                                                                                                                             CACCTGTTTCTGGACCTGCCTAAG GGCCTGAATTTTCTGCTCCTGAATTCAAA|||||||||||||||||||||||||||||||||||||||||||||||||||||||||||||||||||||||||||||||||||||||||||||||||||||||||||||||||||||||||||||||||||||||||||||||||||||||||||||||||||||||||||||||||||||||||||||||||||||||||||||||||||||||||||||||||||||||||||||||||||||||||||||||||||||||||||||
chr12 chr12 57898058 59196630 24M59195042F29M1537N23M                                                                                                                                                                                                                                                                                             CACCTGTTTCTGGACCTGCCTAAG GGCCTGAATTTTCTGCTCCTGAATTCAAA|||||||||||||||||||||||||||||||||||||||||||||||||||||||||||||||||||||||||||||||||||||||||||||||||||||||||||||||||||||||||||||||||||||||||||||||||||||||||||||||||||||||||||||||||||||||||||||||||||||||||||||||||||||||||||||||||||||||||||||||||||||||||||||||||||||||||||||
chr12 chr12 57898059 59199746 23M59195042F29M4652N24M                                                                                                                                                                                                                                                                                              ACCTGTTTCTGGACCTGCCTAAG GGCCTGAATTTTCTGCTCCTGAATTCAAA|||||||||||||||||||||||||||||||||||||||||||||||||||||||||||||||||||||||||||||||||||||||||||||||||||||||||||||||||||||||||||||||||||||||||||||||||||||||||||||||||||||||||||||||||||||||||||||||||||||||||||||||||||||||||||||||||||||||||||||||||||||||||||||||||||||||||||||
chr12 chr12 57898059 59206219 23M59195042F29M11125N24M                                                                                                                                                                                                                                                                                             ACCTGTTTCTGGACCTGCCTAAG GGCCTGAATTTTCTGCTCCTGAATTCAAA|||||||||||||||||||||||||||||||||||||||||||||||||||||||||||||||||||||||||||||||||||||||||||||||||||||||||||||||||||||||||||||||||||||||||||||||||||||||||||||||||||||||||||||||||||||||||||||||||||||||||||||||||||||||||||||||||||||||||||||||||||||||||||||||||||||||||||||
chr12 chr12 57898059 59199746 23M59195042F29M4652N24M                                                                                                                                                                                                                                                                                              ACCTGTTTCTGGACCTGCCTAAG GGCCTGAATTTTCTGCTCCTGAATTCAAA|||||||||||||||||||||||||||||||||||||||||||||||||||||||||||||||||||||||||||||||||||||||||||||||||||||||||||||||||||||||||||||||||||||||||||||||||||||||||||||||||||||||||||||||||||||||||||||||||||||||||||||||||||||||||||||||||||||||||||||||||||||||||||||||||||||||||||||
chr12 chr12 57898060 59196632 22M59195042F29M1537N25M                                                                                                                                                                                                                                                                                               CCTGTTTCTGGACCTGCCTAAG GGCCTGAATTTTCTGCTCCTGAATTCAAA|||||||||||||||||||||||||||||||||||||||||||||||||||||||||||||||||||||||||||||||||||||||||||||||||||||||||||||||||||||||||||||||||||||||||||||||||||||||||||||||||||||||||||||||||||||||||||||||||||||||||||||||||||||||||||||||||||||||||||||||||||||||||||||||||||||||||||||
chr12 chr12 57898060 59196632 22M59195042F29M1537N25M                                                                                                                                                                                                                                                                                               CCTGTTTCTGGACCTGCCTAAG GGCCTGAATTTTCTGCTCCTGAATTCAAA|||||||||||||||||||||||||||||||||||||||||||||||||||||||||||||||||||||||||||||||||||||||||||||||||||||||||||||||||||||||||||||||||||||||||||||||||||||||||||||||||||||||||||||||||||||||||||||||||||||||||||||||||||||||||||||||||||||||||||||||||||||||||||||||||||||||||||||
chr12 chr12 57898060 59196632 22M59195042F29M1537N25M                                                                                                                                                                                                                                                                                               CCTGTTTCTGGACCTGCCTAAG GGCCTGAATTTTCTGCTCCTGAATTCAAA|||||||||||||||||||||||||||||||||||||||||||||||||||||||||||||||||||||||||||||||||||||||||||||||||||||||||||||||||||||||||||||||||||||||||||||||||||||||||||||||||||||||||||||||||||||||||||||||||||||||||||||||||||||||||||||||||||||||||||||||||||||||||||||||||||||||||||||
chr12 chr12 57898060 59196632 22M59195042F29M1537N25M                                                                                                                                                                                                                                                                                               CCTGTTTCTGGACCTGCCTAAG GGCCTGAATTTTCTGCTCCTGAATTCAAA|||||||||||||||||||||||||||||||||||||||||||||||||||||||||||||||||||||||||||||||||||||||||||||||||||||||||||||||||||||||||||||||||||||||||||||||||||||||||||||||||||||||||||||||||||||||||||||||||||||||||||||||||||||||||||||||||||||||||||||||||||||||||||||||||||||||||||||
chr12 chr12 57898060 59196632 22M59195042F29M1537N25M                                                                                                                                                                                                                                                                                               CCTGTTTCTGGACCTGCCTAAG GGCCTGAATTTTCTGCTCCTGAATTCAAA|||||||||||||||||||||||||||||||||||||||||||||||||||||||||||||||||||||||||||||||||||||||||||||||||||||||||||||||||||||||||||||||||||||||||||||||||||||||||||||||||||||||||||||||||||||||||||||||||||||||||||||||||||||||||||||||||||||||||||||||||||||||||||||||||||||||||||||
chr12 chr12 57898060 59196632 22M59195042F29M1537N25M                                                                                                                                                                                                                                                                                               CCTGTTTCTGGACCTGCCTAAG GGCCTGAATTTTCTGCTCCTGAATTCAAA|||||||||||||||||||||||||||||||||||||||||||||||||||||||||||||||||||||||||||||||||||||||||||||||||||||||||||||||||||||||||||||||||||||||||||||||||||||||||||||||||||||||||||||||||||||||||||||||||||||||||||||||||||||||||||||||||||||||||||||||||||||||||||||||||||||||||||||
chr12 chr12 57898060 59196632 22M59195042F29M1537N25M                                                                                                                                                                                                                                                                                               CCTGTTTCTGGACCTGCCTAAG GGCCTGAATTTTCTGCTCCTGAATTCAAA|||||||||||||||||||||||||||||||||||||||||||||||||||||||||||||||||||||||||||||||||||||||||||||||||||||||||||||||||||||||||||||||||||||||||||||||||||||||||||||||||||||||||||||||||||||||||||||||||||||||||||||||||||||||||||||||||||||||||||||||||||||||||||||||||||||||||||||
chr12 chr12 57898060 59196632 22M59195042F29M1537N25M                                                                                                                                                                                                                                                                                               CCTGTTTCTGAACCTGCCTAAG GGCCTGAATTTTCTGCTCCTGAATTCAAA|||||||||||||||||||||||||||||||||||||||||||||||||||||||||||||||||||||||||||||||||||||||||||||||||||||||||||||||||||||||||||||||||||||||||||||||||||||||||||||||||||||||||||||||||||||||||||||||||||||||||||||||||||||||||||||||||||||||||||||||||||||||||||||||||||||||||||||
chr12 chr12 57898060 59196632 22M59195042F29M1537N25M                                                                                                                                                                                                                                                                                               CCTGTTTCTGGACCTGCCTAAG GGCCTGAATTTTCTGCTCCTGAATTCAAA|||||||||||||||||||||||||||||||||||||||||||||||||||||||||||||||||||||||||||||||||||||||||||||||||||||||||||||||||||||||||||||||||||||||||||||||||||||||||||||||||||||||||||||||||||||||||||||||||||||||||||||||||||||||||||||||||||||||||||||||||||||||||||||||||||||||||||||
chr12 chr12 57898060 59196632 22M59195042F29M1537N25M                                                                                                                                                                                                                                                                                               CCTGTTTCTGGACCTGCCTAAG GGCCTGAATTTTCTGCTCCTGAATTCAAA|||||||||||||||||||||||||||||||||||||||||||||||||||||||||||||||||||||||||||||||||||||||||||||||||||||||||||||||||||||||||||||||||||||||||||||||||||||||||||||||||||||||||||||||||||||||||||||||||||||||||||||||||||||||||||||||||||||||||||||||||||||||||||||||||||||||||||||
chr12 chr12 57898060 59196632 22M59195042F29M1537N25M                                                                                                                                                                                                                                                                                               CCTGTTTCTGGACCTGCCTAAG GGCCTGAATTTTCTGCTCCTGAATTCAAA|||||||||||||||||||||||||||||||||||||||||||||||||||||||||||||||||||||||||||||||||||||||||||||||||||||||||||||||||||||||||||||||||||||||||||||||||||||||||||||||||||||||||||||||||||||||||||||||||||||||||||||||||||||||||||||||||||||||||||||||||||||||||||||||||||||||||||||
chr12 chr12 57898060 59196632 22M59195042F29M1537N25M                                                                                                                                                                                                                                                                                               CCTGTTTCTGGACCTGCCTAAG GGCCTGAATTTTCTGCTCCTGAATTCAAA|||||||||||||||||||||||||||||||||||||||||||||||||||||||||||||||||||||||||||||||||||||||||||||||||||||||||||||||||||||||||||||||||||||||||||||||||||||||||||||||||||||||||||||||||||||||||||||||||||||||||||||||||||||||||||||||||||||||||||||||||||||||||||||||||||||||||||||
chr12 chr12 57898060 59196632 22M59195042F29M1537N25M                                                                                                                                                                                                                                                                                               CCTGTTTCTGGACCTGCCTAAG GGCCTGAATTTTCTGCTCCTGAATTCAAA|||||||||||||||||||||||||||||||||||||||||||||||||||||||||||||||||||||||||||||||||||||||||||||||||||||||||||||||||||||||||||||||||||||||||||||||||||||||||||||||||||||||||||||||||||||||||||||||||||||||||||||||||||||||||||||||||||||||||||||||||||||||||||||||||||||||||||||
chr12 chr12 57898060 59196632 22M59195042F29M1537N25M                                                                                                                                                                                                                                                                                               CCTGTTTCTGGACCTGCCTAAG GGCCTGAATTTTCTGCTCCTGAATTCAAA|||||||||||||||||||||||||||||||||||||||||||||||||||||||||||||||||||||||||||||||||||||||||||||||||||||||||||||||||||||||||||||||||||||||||||||||||||||||||||||||||||||||||||||||||||||||||||||||||||||||||||||||||||||||||||||||||||||||||||||||||||||||||||||||||||||||||||||
chr12 chr12 57898060 59196632 22M59195042F29M1537N25M                                                                                                                                                                                                                                                                                               CCTGTTTCTGGACCTGCCTAAG GGCCTGAATTTTCTGCTCCTGAATTCAAA|||||||||||||||||||||||||||||||||||||||||||||||||||||||||||||||||||||||||||||||||||||||||||||||||||||||||||||||||||||||||||||||||||||||||||||||||||||||||||||||||||||||||||||||||||||||||||||||||||||||||||||||||||||||||||||||||||||||||||||||||||||||||||||||||||||||||||||
chr12 chr12 57898060 59196632 22M59195042F29M1537N25M                                                                                                                                                                                                                                                                                               CCTGTTTCTGGACCTGCCTAAG GGCCTGAATTTTCTGCTCCTGAATTCAAA|||||||||||||||||||||||||||||||||||||||||||||||||||||||||||||||||||||||||||||||||||||||||||||||||||||||||||||||||||||||||||||||||||||||||||||||||||||||||||||||||||||||||||||||||||||||||||||||||||||||||||||||||||||||||||||||||||||||||||||||||||||||||||||||||||||||||||||
chr12 chr12 57898060 59196632 22M59195042F29M1537N25M                                                                                                                                                                                                                                                                                               CCTGTTTCTGGACCTGCCTAAG GGCCTGAATTGTCTGCTCCTGAATTCAAA|||||||||||||||||||||||||||||||||||||||||||||||||||||||||||||||||||||||||||||||||||||||||||||||||||||||||||||||||||||||||||||||||||||||||||||||||||||||||||||||||||||||||||||||||||||||||||||||||||||||||||||||||||||||||||||||||||||||||||||||||||||||||||||||||||||||||||||
chr12 chr12 57898060 59196737 22M59195042F29M1642N25M                                                                                                                                                                                                                                                                                               CCTGTTTCTGGACCTGCCTAAG GGCCTGAATTTTCTGCTCCTGAATTCAAA|||||||||||||||||||||||||||||||||||||||||||||||||||||||||||||||||||||||||||||||||||||||||||||||||||||||||||||||||||||||||||||||||||||||||||||||||||||||||||||||||||||||||||||||||||||||||||||||||||||||||||||||||||||||||||||||||||||||||||||||||||||||||||||||||||||||||||||
chr12 chr12 57898060 59199747 22M59195042F29M4652N25M                                                                                                                                                                                                                                                                                               CCTGTTTCTGGACCTGCCTAAG GGCCTGAATTTTCTGCTCCTGAATTCAAA|||||||||||||||||||||||||||||||||||||||||||||||||||||||||||||||||||||||||||||||||||||||||||||||||||||||||||||||||||||||||||||||||||||||||||||||||||||||||||||||||||||||||||||||||||||||||||||||||||||||||||||||||||||||||||||||||||||||||||||||||||||||||||||||||||||||||||||
chr12 chr12 57898060 59199747 22M59195042F29M4652N25M                                                                                                                                                                                                                                                                                               CCTGTTTCTGGACCTGCCTAAG GGCCTGAATTTTCTGCTCCTGAATTCAAA|||||||||||||||||||||||||||||||||||||||||||||||||||||||||||||||||||||||||||||||||||||||||||||||||||||||||||||||||||||||||||||||||||||||||||||||||||||||||||||||||||||||||||||||||||||||||||||||||||||||||||||||||||||||||||||||||||||||||||||||||||||||||||||||||||||||||||||
chr12 chr12 57898060 59199747 22M59195042F29M4652N25M                                                                                                                                                                                                                                                                                               CCTGTTTCTGGACCTGCCTAAG GGCCTGAATTTTCTGCTCCTGAATTCAAA|||||||||||||||||||||||||||||||||||||||||||||||||||||||||||||||||||||||||||||||||||||||||||||||||||||||||||||||||||||||||||||||||||||||||||||||||||||||||||||||||||||||||||||||||||||||||||||||||||||||||||||||||||||||||||||||||||||||||||||||||||||||||||||||||||||||||||||
chr12 chr12 57898060 59199747 22M59195042F29M4652N25M                                                                                                                                                                                                                                                                                               CCTGTTTCTGGACCTGCCTAAG GGCCTGAATTTTCTGCTCCTGAATTCAAA|||||||||||||||||||||||||||||||||||||||||||||||||||||||||||||||||||||||||||||||||||||||||||||||||||||||||||||||||||||||||||||||||||||||||||||||||||||||||||||||||||||||||||||||||||||||||||||||||||||||||||||||||||||||||||||||||||||||||||||||||||||||||||||||||||||||||||||
chr12 chr12 57898060 59199747 22M59195042F29M4652N25M                                                                                                                                                                                                                                                                                               CCTGTTTCTGGACCTGCCTAAG GGCCTGAATTTTCTGCTCCTGAATTCAAA|||||||||||||||||||||||||||||||||||||||||||||||||||||||||||||||||||||||||||||||||||||||||||||||||||||||||||||||||||||||||||||||||||||||||||||||||||||||||||||||||||||||||||||||||||||||||||||||||||||||||||||||||||||||||||||||||||||||||||||||||||||||||||||||||||||||||||||
chr12 chr12 57898060 59196632 22M59195042F29M1537N25M                                                                                                                                                                                                                                                                                               CCTGTTTCTGGACCTGCCTAAG GGCCTGAATTTTCTGCTCCTGAATTCAAA|||||||||||||||||||||||||||||||||||||||||||||||||||||||||||||||||||||||||||||||||||||||||||||||||||||||||||||||||||||||||||||||||||||||||||||||||||||||||||||||||||||||||||||||||||||||||||||||||||||||||||||||||||||||||||||||||||||||||||||||||||||||||||||||||||||||||||||
chr12 chr12 57898060 59196737 22M59195042F29M1642N25M                                                                                                                                                                                                                                                                                               CCTGTTTCTGGACCTGCCTAAG GGCCTGAATTTTCTGCTCCTGAATTCAAA|||||||||||||||||||||||||||||||||||||||||||||||||||||||||||||||||||||||||||||||||||||||||||||||||||||||||||||||||||||||||||||||||||||||||||||||||||||||||||||||||||||||||||||||||||||||||||||||||||||||||||||||||||||||||||||||||||||||||||||||||||||||||||||||||||||||||||||
chr12 chr12 57898060 59196632 22M59195042F29M1537N25M                                                                                                                                                                                                                                                                                               CCTGTTTCTGGACCTGCCTAAG GGCCTGAATTTTCTGCTCCTGAATTCAAA|||||||||||||||||||||||||||||||||||||||||||||||||||||||||||||||||||||||||||||||||||||||||||||||||||||||||||||||||||||||||||||||||||||||||||||||||||||||||||||||||||||||||||||||||||||||||||||||||||||||||||||||||||||||||||||||||||||||||||||||||||||||||||||||||||||||||||||
chr12 chr12 57898060 59196632 22M59195042F29M1537N25M                                                                                                                                                                                                                                                                                               CCTGTTTCTGGACCTGCCTAAG GGCCTGAATTTTCTGCTCCTGAATTCAAA|||||||||||||||||||||||||||||||||||||||||||||||||||||||||||||||||||||||||||||||||||||||||||||||||||||||||||||||||||||||||||||||||||||||||||||||||||||||||||||||||||||||||||||||||||||||||||||||||||||||||||||||||||||||||||||||||||||||||||||||||||||||||||||||||||||||||||||
chr12 chr12 57898060 59199747 22M59195042F29M4652N25M                                                                                                                                                                                                                                                                                               CCTGTTTCTGGACCTGCCTAAG GGCCTGAATTTTCTGCTCCTGAATTCAAA|||||||||||||||||||||||||||||||||||||||||||||||||||||||||||||||||||||||||||||||||||||||||||||||||||||||||||||||||||||||||||||||||||||||||||||||||||||||||||||||||||||||||||||||||||||||||||||||||||||||||||||||||||||||||||||||||||||||||||||||||||||||||||||||||||||||||||||
chr12 chr12 57898060 59196632 22M59195042F29M1537N25M                                                                                                                                                                                                                                                                                               CCTGTTTCTGGACCTGCCTAAG GGCCTGAATTTTCTGCTCCTGAATTCAAA|||||||||||||||||||||||||||||||||||||||||||||||||||||||||||||||||||||||||||||||||||||||||||||||||||||||||||||||||||||||||||||||||||||||||||||||||||||||||||||||||||||||||||||||||||||||||||||||||||||||||||||||||||||||||||||||||||||||||||||||||||||||||||||||||||||||||||||
chr12 chr12 57898060 59199747 22M59195042F29M4652N25M                                                                                                                                                                                                                                                                                               CCTGTTTCTGGACCTGCCTAAG GGCCTGAATTTTCTGCTCCTGAATTCAAA|||||||||||||||||||||||||||||||||||||||||||||||||||||||||||||||||||||||||||||||||||||||||||||||||||||||||||||||||||||||||||||||||||||||||||||||||||||||||||||||||||||||||||||||||||||||||||||||||||||||||||||||||||||||||||||||||||||||||||||||||||||||||||||||||||||||||||||
chr12 chr12 57898060 59196632 22M59195042F29M1537N25M                                                                                                                                                                                                                                                                                               CCTGTTTCTGGACCTGCCTAAG GGCCTGAATTTTCTGCTCCTGAATTCAAA|||||||||||||||||||||||||||||||||||||||||||||||||||||||||||||||||||||||||||||||||||||||||||||||||||||||||||||||||||||||||||||||||||||||||||||||||||||||||||||||||||||||||||||||||||||||||||||||||||||||||||||||||||||||||||||||||||||||||||||||||||||||||||||||||||||||||||||
chr12 chr12 57898060 59196632 22M59195042F29M1537N25M                                                                                                                                                                                                                                                                                               CCTGTTTCTGGACCTGCCTAAG GGCCTGAATTTTCTGCTCCTGAATTCAAA|||||||||||||||||||||||||||||||||||||||||||||||||||||||||||||||||||||||||||||||||||||||||||||||||||||||||||||||||||||||||||||||||||||||||||||||||||||||||||||||||||||||||||||||||||||||||||||||||||||||||||||||||||||||||||||||||||||||||||||||||||||||||||||||||||||||||||||
chr12 chr12 57898060 59199747 22M59195042F29M4652N25M                                                                                                                                                                                                                                                                                               CCTGTTTCTGGACCTGCCTAAG GGCCTGAATTTTCTGCTCCTGAATTCAAA|||||||||||||||||||||||||||||||||||||||||||||||||||||||||||||||||||||||||||||||||||||||||||||||||||||||||||||||||||||||||||||||||||||||||||||||||||||||||||||||||||||||||||||||||||||||||||||||||||||||||||||||||||||||||||||||||||||||||||||||||||||||||||||||||||||||||||||
chr12 chr12 57898060 59199747 22M59195042F29M4652N25M                                                                                                                                                                                                                                                                                               CCTGTTTCTGGACCTGCCTAAG GGCCTGAATTTTCTGCTCCTGAATTCAAA|||||||||||||||||||||||||||||||||||||||||||||||||||||||||||||||||||||||||||||||||||||||||||||||||||||||||||||||||||||||||||||||||||||||||||||||||||||||||||||||||||||||||||||||||||||||||||||||||||||||||||||||||||||||||||||||||||||||||||||||||||||||||||||||||||||||||||||
chr12 chr12 57898061 59196633 21M59195042F29M1537N26M                                                                                                                                                                                                                                                                                                CTGTTTCTGGACCTGCCTAAG GGCCTGAATTTTCTGCTCCTGAATTCAAA|||||||||||||||||||||||||||||||||||||||||||||||||||||||||||||||||||||||||||||||||||||||||||||||||||||||||||||||||||||||||||||||||||||||||||||||||||||||||||||||||||||||||||||||||||||||||||||||||||||||||||||||||||||||||||||||||||||||||||||||||||||||||||||||||||||||||||||
chr12 chr12 57898061 59196633 21M59195042F29M1537N26M                                                                                                                                                                                                                                                                                                CTGTTTCTGGACCTGCCTAAG GGCCTGAATTTTCTGCTCCTGAATTCAAA|||||||||||||||||||||||||||||||||||||||||||||||||||||||||||||||||||||||||||||||||||||||||||||||||||||||||||||||||||||||||||||||||||||||||||||||||||||||||||||||||||||||||||||||||||||||||||||||||||||||||||||||||||||||||||||||||||||||||||||||||||||||||||||||||||||||||||||
chr12 chr12 57898061 59199748 21M59195042F29M4652N26M                                                                                                                                                                                                                                                                                                CTGTTTCTGGACCTGCCTAAG GGCCTGAATTTTCTGCTCCTGAATTCAAA|||||||||||||||||||||||||||||||||||||||||||||||||||||||||||||||||||||||||||||||||||||||||||||||||||||||||||||||||||||||||||||||||||||||||||||||||||||||||||||||||||||||||||||||||||||||||||||||||||||||||||||||||||||||||||||||||||||||||||||||||||||||||||||||||||||||||||||
chr12 chr12 57898061 59199748 21M59195042F29M4652N26M                                                                                                                                                                                                                                                                                                CTGTTTCTGGACCTGCCTAAG GGCCTGAATTTTCTGCTCCTGAATTCAAA|||||||||||||||||||||||||||||||||||||||||||||||||||||||||||||||||||||||||||||||||||||||||||||||||||||||||||||||||||||||||||||||||||||||||||||||||||||||||||||||||||||||||||||||||||||||||||||||||||||||||||||||||||||||||||||||||||||||||||||||||||||||||||||||||||||||||||||
chr12 chr12 57898061 59196633 21M59195042F29M1537N26M                                                                                                                                                                                                                                                                                                CTGTTTCTGGACCTGCCTAAG GGCCTGAATTTTCTGCTCCTGAATTCAAA|||||||||||||||||||||||||||||||||||||||||||||||||||||||||||||||||||||||||||||||||||||||||||||||||||||||||||||||||||||||||||||||||||||||||||||||||||||||||||||||||||||||||||||||||||||||||||||||||||||||||||||||||||||||||||||||||||||||||||||||||||||||||||||||||||||||||||||
chr12 chr12 57898061 59196633 21M59195042F29M1537N26M                                                                                                                                                                                                                                                                                                CTGTTTCTGGACCTGCCTAAG GGCCTGAATTTTCTGCTCCTGAATTCAAA|||||||||||||||||||||||||||||||||||||||||||||||||||||||||||||||||||||||||||||||||||||||||||||||||||||||||||||||||||||||||||||||||||||||||||||||||||||||||||||||||||||||||||||||||||||||||||||||||||||||||||||||||||||||||||||||||||||||||||||||||||||||||||||||||||||||||||||
chr12 chr12 57898061 59196633 21M59195042F29M1537N26M                                                                                                                                                                                                                                                                                                CTGTTTCTGGACCTGCCTAAG GGCCTGAATTTTCTGCTCCTGAATTCAAA|||||||||||||||||||||||||||||||||||||||||||||||||||||||||||||||||||||||||||||||||||||||||||||||||||||||||||||||||||||||||||||||||||||||||||||||||||||||||||||||||||||||||||||||||||||||||||||||||||||||||||||||||||||||||||||||||||||||||||||||||||||||||||||||||||||||||||||
chr12 chr12 57898061 59196633 21M59195042F29M1537N26M                                                                                                                                                                                                                                                                                                CTGTTTCTGGACCTGCCTAAG GGCCTGAATTTTCTGCTCCTGAATTCAAA|||||||||||||||||||||||||||||||||||||||||||||||||||||||||||||||||||||||||||||||||||||||||||||||||||||||||||||||||||||||||||||||||||||||||||||||||||||||||||||||||||||||||||||||||||||||||||||||||||||||||||||||||||||||||||||||||||||||||||||||||||||||||||||||||||||||||||||
chr12 chr12 57898061 59196633 21M59195042F29M1537N26M                                                                                                                                                                                                                                                                                                CTGTTTCTGGACCTGCCTAAG GGCCTGAATTTTCTGCTCCTGAATTCAAA|||||||||||||||||||||||||||||||||||||||||||||||||||||||||||||||||||||||||||||||||||||||||||||||||||||||||||||||||||||||||||||||||||||||||||||||||||||||||||||||||||||||||||||||||||||||||||||||||||||||||||||||||||||||||||||||||||||||||||||||||||||||||||||||||||||||||||||
chr12 chr12 57898062 59199749 20M59195042F29M4652N27M                                                                                                                                                                                                                                                                                                 TGTTTCTGGACCTGCCTAAG GGCCTGAATTTTCTGCTCCTGAATTTAAA|||||||||||||||||||||||||||||||||||||||||||||||||||||||||||||||||||||||||||||||||||||||||||||||||||||||||||||||||||||||||||||||||||||||||||||||||||||||||||||||||||||||||||||||||||||||||||||||||||||||||||||||||||||||||||||||||||||||||||||||||||||||||||||||||||||||||||||
chr12 chr12 57898062 59196634 20M59195042F29M1537N27M                                                                                                                                                                                                                                                                                                 TGTTTCTGGACCTGCCTAAG GGCCTGAATTTTCTGCTCCTGAATTCAAA|||||||||||||||||||||||||||||||||||||||||||||||||||||||||||||||||||||||||||||||||||||||||||||||||||||||||||||||||||||||||||||||||||||||||||||||||||||||||||||||||||||||||||||||||||||||||||||||||||||||||||||||||||||||||||||||||||||||||||||||||||||||||||||||||||||||||||||
chr12 chr12 57898062 59196634 20M59195042F29M1537N27M                                                                                                                                                                                                                                                                                                 TGTTTCTGGACCTGCCTAAG GGCCTGAATTTTCTGCTCCTGAATTCAAA|||||||||||||||||||||||||||||||||||||||||||||||||||||||||||||||||||||||||||||||||||||||||||||||||||||||||||||||||||||||||||||||||||||||||||||||||||||||||||||||||||||||||||||||||||||||||||||||||||||||||||||||||||||||||||||||||||||||||||||||||||||||||||||||||||||||||||||
chr12 chr12 57898062 59196634 20M59195042F29M1537N27M                                                                                                                                                                                                                                                                                                 TGTTTCTGGACCTGCCTAAG GGCCTGAATTTTCTGCTCCTGAATTCAAA|||||||||||||||||||||||||||||||||||||||||||||||||||||||||||||||||||||||||||||||||||||||||||||||||||||||||||||||||||||||||||||||||||||||||||||||||||||||||||||||||||||||||||||||||||||||||||||||||||||||||||||||||||||||||||||||||||||||||||||||||||||||||||||||||||||||||||||
chr12 chr12 57898062 59196634 20M59195042F29M1537N27M                                                                                                                                                                                                                                                                                                 TGTTTCTGGACCTGCCTAAG GGCCTGAATTTTCTGCTCCTGAATTCAAA|||||||||||||||||||||||||||||||||||||||||||||||||||||||||||||||||||||||||||||||||||||||||||||||||||||||||||||||||||||||||||||||||||||||||||||||||||||||||||||||||||||||||||||||||||||||||||||||||||||||||||||||||||||||||||||||||||||||||||||||||||||||||||||||||||||||||||||
chr12 chr12 57898062 59196634 20M59195042F29M1537N27M                                                                                                                                                                                                                                                                                                 TGTTTCTGGACCTGCCTAAG GGCCTGAATTTTCTGCTCCTGAATTCAAA|||||||||||||||||||||||||||||||||||||||||||||||||||||||||||||||||||||||||||||||||||||||||||||||||||||||||||||||||||||||||||||||||||||||||||||||||||||||||||||||||||||||||||||||||||||||||||||||||||||||||||||||||||||||||||||||||||||||||||||||||||||||||||||||||||||||||||||
chr12 chr12 57898062 59196634 20M59195042F29M1537N27M                                                                                                                                                                                                                                                                                                 TGTTTCTGGACCTGCCTAAG GGCCTGAATTTTCTGCTCCTGAATTCAAA|||||||||||||||||||||||||||||||||||||||||||||||||||||||||||||||||||||||||||||||||||||||||||||||||||||||||||||||||||||||||||||||||||||||||||||||||||||||||||||||||||||||||||||||||||||||||||||||||||||||||||||||||||||||||||||||||||||||||||||||||||||||||||||||||||||||||||||
chr12 chr12 57898062 59199749 20M59195042F29M4652N27M                                                                                                                                                                                                                                                                                                 TGTTTCTGGACCTGCCTAAG GGCCTGAATTTTCTGCTCCTGAATTCAAA|||||||||||||||||||||||||||||||||||||||||||||||||||||||||||||||||||||||||||||||||||||||||||||||||||||||||||||||||||||||||||||||||||||||||||||||||||||||||||||||||||||||||||||||||||||||||||||||||||||||||||||||||||||||||||||||||||||||||||||||||||||||||||||||||||||||||||||
chr12 chr12 57898062 59196634 20M59195042F29M1537N27M                                                                                                                                                                                                                                                                                                 TGTTTCTGGACCTGCCTAAG GGCCTGAATTTTCTGCTCCTGAATTCAAA|||||||||||||||||||||||||||||||||||||||||||||||||||||||||||||||||||||||||||||||||||||||||||||||||||||||||||||||||||||||||||||||||||||||||||||||||||||||||||||||||||||||||||||||||||||||||||||||||||||||||||||||||||||||||||||||||||||||||||||||||||||||||||||||||||||||||||||
chr12 chr12 57898062 59196739 20M59195042F29M1642N27M                                                                                                                                                                                                                                                                                                 TGTTTCTGGACCTGCCTAAG GGCCTGAATTTTCTGCTCCTGAATTCAAA|||||||||||||||||||||||||||||||||||||||||||||||||||||||||||||||||||||||||||||||||||||||||||||||||||||||||||||||||||||||||||||||||||||||||||||||||||||||||||||||||||||||||||||||||||||||||||||||||||||||||||||||||||||||||||||||||||||||||||||||||||||||||||||||||||||||||||||
chr12 chr12 57898062 59196739 20M59195042F29M1642N27M                                                                                                                                                                                                                                                                                                 TGTTTCTGGACCTGCCTAAG GGCCTGAATTTTCTGCTCCTGAATTCAAA|||||||||||||||||||||||||||||||||||||||||||||||||||||||||||||||||||||||||||||||||||||||||||||||||||||||||||||||||||||||||||||||||||||||||||||||||||||||||||||||||||||||||||||||||||||||||||||||||||||||||||||||||||||||||||||||||||||||||||||||||||||||||||||||||||||||||||||
chr12 chr12 57898062 59196634 20M59195042F29M1537N27M                                                                                                                                                                                                                                                                                                 TGTTTCTGGACCTGCCTAAG GGCCTGAATTTTCTGCTCCTGAATTCAAA|||||||||||||||||||||||||||||||||||||||||||||||||||||||||||||||||||||||||||||||||||||||||||||||||||||||||||||||||||||||||||||||||||||||||||||||||||||||||||||||||||||||||||||||||||||||||||||||||||||||||||||||||||||||||||||||||||||||||||||||||||||||||||||||||||||||||||||
chr12 chr12 57898062 59199749 20M59195042F29M4652N27M                                                                                                                                                                                                                                                                                                 TGTTTCTGGACCTGCCTAAG GGCCTGAATTTTCTGCTCCTGAATTCAAA|||||||||||||||||||||||||||||||||||||||||||||||||||||||||||||||||||||||||||||||||||||||||||||||||||||||||||||||||||||||||||||||||||||||||||||||||||||||||||||||||||||||||||||||||||||||||||||||||||||||||||||||||||||||||||||||||||||||||||||||||||||||||||||||||||||||||||||
chr12 chr12 57898062 59199749 20M59195042F29M4652N27M                                                                                                                                                                                                                                                                                                 TGTTTCTGGACCTGCCTAAG GGCCTGAATTTTCTGCTCCTGAATTCAAA|||||||||||||||||||||||||||||||||||||||||||||||||||||||||||||||||||||||||||||||||||||||||||||||||||||||||||||||||||||||||||||||||||||||||||||||||||||||||||||||||||||||||||||||||||||||||||||||||||||||||||||||||||||||||||||||||||||||||||||||||||||||||||||||||||||||||||||
chr12 chr12 57898062 59199749 20M59195042F29M4652N27M                                                                                                                                                                                                                                                                                                 TGTTTCTGGACCTGCCTAAG GGCCTGAATTTTCTGCTCCTGAATTCAAA|||||||||||||||||||||||||||||||||||||||||||||||||||||||||||||||||||||||||||||||||||||||||||||||||||||||||||||||||||||||||||||||||||||||||||||||||||||||||||||||||||||||||||||||||||||||||||||||||||||||||||||||||||||||||||||||||||||||||||||||||||||||||||||||||||||||||||||
chr12 chr12 57898062 59199749 20M59195042F29M4652N27M                                                                                                                                                                                                                                                                                                 TGTTTCTGGACCTGCCTAAG GGCCTGAATTTTCTGCTCCTGAATTCAAA|||||||||||||||||||||||||||||||||||||||||||||||||||||||||||||||||||||||||||||||||||||||||||||||||||||||||||||||||||||||||||||||||||||||||||||||||||||||||||||||||||||||||||||||||||||||||||||||||||||||||||||||||||||||||||||||||||||||||||||||||||||||||||||||||||||||||||||
chr12 chr12 57898063 59199750 19M59195042F29M4652N28M                                                                                                                                                                                                                                                                                                  GTTTCTGGACCTGCCTAAG GGCCTGAATTTTCTGCTCCTGAATTCAAA|||||||||||||||||||||||||||||||||||||||||||||||||||||||||||||||||||||||||||||||||||||||||||||||||||||||||||||||||||||||||||||||||||||||||||||||||||||||||||||||||||||||||||||||||||||||||||||||||||||||||||||||||||||||||||||||||||||||||||||||||||||||||||||||||||||||||||||
chr12 chr12 57898063 59199750 19M59195042F29M4652N28M                                                                                                                                                                                                                                                                                                  GTTTCTGGACCTGCCTAAG GGCCTGAATTTTCTGCTCCTGAATTCAAA|||||||||||||||||||||||||||||||||||||||||||||||||||||||||||||||||||||||||||||||||||||||||||||||||||||||||||||||||||||||||||||||||||||||||||||||||||||||||||||||||||||||||||||||||||||||||||||||||||||||||||||||||||||||||||||||||||||||||||||||||||||||||||||||||||||||||||||
chr12 chr12 57898063 59199750 19M59195042F29M4652N28M                                                                                                                                                                                                                                                                                                  GTTTCTGGACCTGCCTAAG GGCCTGAATTTTCTGCTCCTGAATTCAAA|||||||||||||||||||||||||||||||||||||||||||||||||||||||||||||||||||||||||||||||||||||||||||||||||||||||||||||||||||||||||||||||||||||||||||||||||||||||||||||||||||||||||||||||||||||||||||||||||||||||||||||||||||||||||||||||||||||||||||||||||||||||||||||||||||||||||||||
chr12 chr12 57898063 59196635 19M59195042F29M1537N28M                                                                                                                                                                                                                                                                                                  GTTTCTGGACCTGCCTAAG GGCCTGAATTTTCTGCTCCTGAATTCAAA|||||||||||||||||||||||||||||||||||||||||||||||||||||||||||||||||||||||||||||||||||||||||||||||||||||||||||||||||||||||||||||||||||||||||||||||||||||||||||||||||||||||||||||||||||||||||||||||||||||||||||||||||||||||||||||||||||||||||||||||||||||||||||||||||||||||||||||
chr12 chr12 57898063 59196635 19M59195042F29M1537N28M                                                                                                                                                                                                                                                                                                  GTTTCTGGACCTGCCTAAG GGCCTGAATTTTCTGCTCCTGAATTCAAA|||||||||||||||||||||||||||||||||||||||||||||||||||||||||||||||||||||||||||||||||||||||||||||||||||||||||||||||||||||||||||||||||||||||||||||||||||||||||||||||||||||||||||||||||||||||||||||||||||||||||||||||||||||||||||||||||||||||||||||||||||||||||||||||||||||||||||||
chr12 chr12 57898063 59196635 19M59195042F29M1537N28M                                                                                                                                                                                                                                                                                                  GTTTCTGGACCTGCCTAAG GGCCTGAATTTTCTGCTCCTGAATTCAAA|||||||||||||||||||||||||||||||||||||||||||||||||||||||||||||||||||||||||||||||||||||||||||||||||||||||||||||||||||||||||||||||||||||||||||||||||||||||||||||||||||||||||||||||||||||||||||||||||||||||||||||||||||||||||||||||||||||||||||||||||||||||||||||||||||||||||||||
chr12 chr12 57898063 59196635 19M59195042F29M1537N28M                                                                                                                                                                                                                                                                                                  GTTTCTGGACCTGCCTAAG GGCCTGCATTTTCTGCTCCTGAATTCAAA|||||||||||||||||||||||||||||||||||||||||||||||||||||||||||||||||||||||||||||||||||||||||||||||||||||||||||||||||||||||||||||||||||||||||||||||||||||||||||||||||||||||||||||||||||||||||||||||||||||||||||||||||||||||||||||||||||||||||||||||||||||||||||||||||||||||||||||
chr12 chr12 57898063 59199750 19M59195042F29M4652N28M                                                                                                                                                                                                                                                                                                  GTTTCTGGACCTGCCTAAG GGCCTGAATTTTCTGCTCCTGAATTCAAA|||||||||||||||||||||||||||||||||||||||||||||||||||||||||||||||||||||||||||||||||||||||||||||||||||||||||||||||||||||||||||||||||||||||||||||||||||||||||||||||||||||||||||||||||||||||||||||||||||||||||||||||||||||||||||||||||||||||||||||||||||||||||||||||||||||||||||||
chr12 chr12 57898063 59199750 19M59195042F29M4652N28M                                                                                                                                                                                                                                                                                                  GTTTCTGGACCTGCCTAAG GGCCTGAATTTTCTGCTCCTGAATTCAAA|||||||||||||||||||||||||||||||||||||||||||||||||||||||||||||||||||||||||||||||||||||||||||||||||||||||||||||||||||||||||||||||||||||||||||||||||||||||||||||||||||||||||||||||||||||||||||||||||||||||||||||||||||||||||||||||||||||||||||||||||||||||||||||||||||||||||||||
chr12 chr12 57898063 59196635 19M59195042F29M1537N28M                                                                                                                                                                                                                                                                                                  GTTTCTGGACCTGCCTAAG GGCCTGAATTTTCTGCTCCTGAATTCAAA|||||||||||||||||||||||||||||||||||||||||||||||||||||||||||||||||||||||||||||||||||||||||||||||||||||||||||||||||||||||||||||||||||||||||||||||||||||||||||||||||||||||||||||||||||||||||||||||||||||||||||||||||||||||||||||||||||||||||||||||||||||||||||||||||||||||||||||
chr12 chr12 57898063 59196635 19M59195042F29M1537N28M                                                                                                                                                                                                                                                                                                  GTTTCTGGACCTGCCTAAG GGCCTGAATTTTCTGCTCCTGAATTCAAA|||||||||||||||||||||||||||||||||||||||||||||||||||||||||||||||||||||||||||||||||||||||||||||||||||||||||||||||||||||||||||||||||||||||||||||||||||||||||||||||||||||||||||||||||||||||||||||||||||||||||||||||||||||||||||||||||||||||||||||||||||||||||||||||||||||||||||||
chr12 chr12 57898063 59196635 19M59195042F29M1537N28M                                                                                                                                                                                                                                                                                                  GTTTCTGGACCTGCCTAAG GGCCTGAATTTTCTGCTCCTGAATTCAAA|||||||||||||||||||||||||||||||||||||||||||||||||||||||||||||||||||||||||||||||||||||||||||||||||||||||||||||||||||||||||||||||||||||||||||||||||||||||||||||||||||||||||||||||||||||||||||||||||||||||||||||||||||||||||||||||||||||||||||||||||||||||||||||||||||||||||||||
chr12 chr12 57898063 59196635 19M59195042F29M1537N28M                                                                                                                                                                                                                                                                                                  GTTTCTGGACCTGCCTAAG GGCCTGAATCTTCTGCTCCTGAAGTCAAA|||||||||||||||||||||||||||||||||||||||||||||||||||||||||||||||||||||||||||||||||||||||||||||||||||||||||||||||||||||||||||||||||||||||||||||||||||||||||||||||||||||||||||||||||||||||||||||||||||||||||||||||||||||||||||||||||||||||||||||||||||||||||||||||||||||||||||||
chr12 chr12 57898063 59206223 19M59195042F29M11125N28M                                                                                                                                                                                                                                                                                                 TTTTCTGAACCTGCCTAAG GGCCTGAATTTTCTGCTCCTGAATTCAAA|||||||||||||||||||||||||||||||||||||||||||||||||||||||||||||||||||||||||||||||||||||||||||||||||||||||||||||||||||||||||||||||||||||||||||||||||||||||||||||||||||||||||||||||||||||||||||||||||||||||||||||||||||||||||||||||||||||||||||||||||||||||||||||||||||||||||||||
chr12 chr12 57898063 59199750 19M59195042F29M4652N28M                                                                                                                                                                                                                                                                                                  GTTTCTGGACCTGCCTAAG GGCCTGAATTTTCTGCTCCTGAATTCAAA|||||||||||||||||||||||||||||||||||||||||||||||||||||||||||||||||||||||||||||||||||||||||||||||||||||||||||||||||||||||||||||||||||||||||||||||||||||||||||||||||||||||||||||||||||||||||||||||||||||||||||||||||||||||||||||||||||||||||||||||||||||||||||||||||||||||||||||
chr12 chr12 57898063 59196635 19M59195042F29M1537N28M                                                                                                                                                                                                                                                                                                  GTTTCTGGACCTGCCTAAG GGCCCGAATTTTCTGCTCCTGAATTCAAA|||||||||||||||||||||||||||||||||||||||||||||||||||||||||||||||||||||||||||||||||||||||||||||||||||||||||||||||||||||||||||||||||||||||||||||||||||||||||||||||||||||||||||||||||||||||||||||||||||||||||||||||||||||||||||||||||||||||||||||||||||||||||||||||||||||||||||||
chr12 chr12 57898064 59196636 18M59195042F29M1537N29M                                                                                                                                                                                                                                                                                                   TTTCTGGACCTGCCTAAG GGCCTGAATTTTCTGCTCCTGAATTCAAA|||||||||||||||||||||||||||||||||||||||||||||||||||||||||||||||||||||||||||||||||||||||||||||||||||||||||||||||||||||||||||||||||||||||||||||||||||||||||||||||||||||||||||||||||||||||||||||||||||||||||||||||||||||||||||||||||||||||||||||||||||||||||||||||||||||||||||||
chr12 chr12 57898064 59199751 18M59195042F29M4652N29M                                                                                                                                                                                                                                                                                                   TTTCTGGACCTGCCTAAG GGCCTGAATTTTCTGCTCCTGAATTCAAA|||||||||||||||||||||||||||||||||||||||||||||||||||||||||||||||||||||||||||||||||||||||||||||||||||||||||||||||||||||||||||||||||||||||||||||||||||||||||||||||||||||||||||||||||||||||||||||||||||||||||||||||||||||||||||||||||||||||||||||||||||||||||||||||||||||||||||||
chr12 chr12 57898064 59196636 18M59195042F29M1537N29M                                                                                                                                                                                                                                                                                                   TTTCTGGACCTGCCTAAG GGCCTGAATTTTCTGCTCCTGAATTCAAA|||||||||||||||||||||||||||||||||||||||||||||||||||||||||||||||||||||||||||||||||||||||||||||||||||||||||||||||||||||||||||||||||||||||||||||||||||||||||||||||||||||||||||||||||||||||||||||||||||||||||||||||||||||||||||||||||||||||||||||||||||||||||||||||||||||||||||||
chr12 chr12 57898064 59196636 18M59195042F29M1537N29M                                                                                                                                                                                                                                                                                                   TTTCTGGACCTGCCTAAG GGCCTGAATTTTCTGCTCCTGAATTCAAA|||||||||||||||||||||||||||||||||||||||||||||||||||||||||||||||||||||||||||||||||||||||||||||||||||||||||||||||||||||||||||||||||||||||||||||||||||||||||||||||||||||||||||||||||||||||||||||||||||||||||||||||||||||||||||||||||||||||||||||||||||||||||||||||||||||||||||||
chr12 chr12 57898064 59196636 18M59195042F29M1537N29M                                                                                                                                                                                                                                                                                                   TTTCTGGACCTGCCTAAG GGCCTGAATTTTCTGCTCCTGAATTCAAA|||||||||||||||||||||||||||||||||||||||||||||||||||||||||||||||||||||||||||||||||||||||||||||||||||||||||||||||||||||||||||||||||||||||||||||||||||||||||||||||||||||||||||||||||||||||||||||||||||||||||||||||||||||||||||||||||||||||||||||||||||||||||||||||||||||||||||||
chr12 chr12 57898064 59196636 18M59195042F29M1537N29M                                                                                                                                                                                                                                                                                                   TTTCTGGACCTGCCTAAG GGCCTGAATTTTCTGCTCCTGAATTCAAA|||||||||||||||||||||||||||||||||||||||||||||||||||||||||||||||||||||||||||||||||||||||||||||||||||||||||||||||||||||||||||||||||||||||||||||||||||||||||||||||||||||||||||||||||||||||||||||||||||||||||||||||||||||||||||||||||||||||||||||||||||||||||||||||||||||||||||||
chr12 chr12 57898064 59196636 18M59195042F29M1537N29M                                                                                                                                                                                                                                                                                                   TTTCTGGACCTGCCTAAG GGCCTGAATTTTCTGCTCCTGAATTCAAA|||||||||||||||||||||||||||||||||||||||||||||||||||||||||||||||||||||||||||||||||||||||||||||||||||||||||||||||||||||||||||||||||||||||||||||||||||||||||||||||||||||||||||||||||||||||||||||||||||||||||||||||||||||||||||||||||||||||||||||||||||||||||||||||||||||||||||||
chr12 chr12 57898064 59196636 18M59195042F29M1537N29M                                                                                                                                                                                                                                                                                                   TTTCTGGACCTGCCTAAG GGCCTGAATTTTCTGCTCCTGAATTCAAA|||||||||||||||||||||||||||||||||||||||||||||||||||||||||||||||||||||||||||||||||||||||||||||||||||||||||||||||||||||||||||||||||||||||||||||||||||||||||||||||||||||||||||||||||||||||||||||||||||||||||||||||||||||||||||||||||||||||||||||||||||||||||||||||||||||||||||||
chr12 chr12 57898065 59199752 17M59195042F29M4652N30M                                                                                                                                                                                                                                                                                                    TTCTGGACCTGCCTAAG GGCCTGAATTTTCTGCTCCTGAATTCAAA|||||||||||||||||||||||||||||||||||||||||||||||||||||||||||||||||||||||||||||||||||||||||||||||||||||||||||||||||||||||||||||||||||||||||||||||||||||||||||||||||||||||||||||||||||||||||||||||||||||||||||||||||||||||||||||||||||||||||||||||||||||||||||||||||||||||||||||
chr12 chr12 57898065 59196637 17M59195042F29M1537N30M                                                                                                                                                                                                                                                                                                    TTCTGGACCTGCCTAAG GGCCTGAATTTTCTGCTCCTGAATTCAAA|||||||||||||||||||||||||||||||||||||||||||||||||||||||||||||||||||||||||||||||||||||||||||||||||||||||||||||||||||||||||||||||||||||||||||||||||||||||||||||||||||||||||||||||||||||||||||||||||||||||||||||||||||||||||||||||||||||||||||||||||||||||||||||||||||||||||||||
chr12 chr12 57898065 59196637 17M59195042F29M1537N30M                                                                                                                                                                                                                                                                                                    TTCTGGACCTGCCTAAG GGCCTGAATTTTCTGCTCCTGAATTCAAA|||||||||||||||||||||||||||||||||||||||||||||||||||||||||||||||||||||||||||||||||||||||||||||||||||||||||||||||||||||||||||||||||||||||||||||||||||||||||||||||||||||||||||||||||||||||||||||||||||||||||||||||||||||||||||||||||||||||||||||||||||||||||||||||||||||||||||||
chr12 chr12 57898065 59196637 17M59195042F29M1537N30M                                                                                                                                                                                                                                                                                                    TTCTGGACCTGCCTAAG GGCCTGAATTTTCTGCTCCTGAATTCAAA|||||||||||||||||||||||||||||||||||||||||||||||||||||||||||||||||||||||||||||||||||||||||||||||||||||||||||||||||||||||||||||||||||||||||||||||||||||||||||||||||||||||||||||||||||||||||||||||||||||||||||||||||||||||||||||||||||||||||||||||||||||||||||||||||||||||||||||
chr12 chr12 57898065 59196637 17M59195042F29M1537N30M                                                                                                                                                                                                                                                                                                    TTCTGGACCTGCCTAAG GGCCTGAATTTTCTGCTCCTGAATTCAAA|||||||||||||||||||||||||||||||||||||||||||||||||||||||||||||||||||||||||||||||||||||||||||||||||||||||||||||||||||||||||||||||||||||||||||||||||||||||||||||||||||||||||||||||||||||||||||||||||||||||||||||||||||||||||||||||||||||||||||||||||||||||||||||||||||||||||||||
chr12 chr12 57898065 59196637 17M59195042F29M1537N30M                                                                                                                                                                                                                                                                                                    TTCTGGACCTGCCTAAG GGCCTGAATTTTCTGCTCCTGAATTCAAA|||||||||||||||||||||||||||||||||||||||||||||||||||||||||||||||||||||||||||||||||||||||||||||||||||||||||||||||||||||||||||||||||||||||||||||||||||||||||||||||||||||||||||||||||||||||||||||||||||||||||||||||||||||||||||||||||||||||||||||||||||||||||||||||||||||||||||||
chr12 chr12 57898065 59196637 17M59195042F29M1537N30M                                                                                                                                                                                                                                                                                                    TTCTGGACCTGCCTAAG GGCCTGAATTTTCTGCTCCTGAATTCAAA|||||||||||||||||||||||||||||||||||||||||||||||||||||||||||||||||||||||||||||||||||||||||||||||||||||||||||||||||||||||||||||||||||||||||||||||||||||||||||||||||||||||||||||||||||||||||||||||||||||||||||||||||||||||||||||||||||||||||||||||||||||||||||||||||||||||||||||
chr12 chr12 57898065 59199752 17M59195042F29M4652N30M                                                                                                                                                                                                                                                                                                    TTCTGGACCTGCCTAAG GGCCTGAATTTTCTGCTCCTGAATTCAAA|||||||||||||||||||||||||||||||||||||||||||||||||||||||||||||||||||||||||||||||||||||||||||||||||||||||||||||||||||||||||||||||||||||||||||||||||||||||||||||||||||||||||||||||||||||||||||||||||||||||||||||||||||||||||||||||||||||||||||||||||||||||||||||||||||||||||||||
chr12 chr12 57898065 59199752 17M59195042F29M4652N30M                                                                                                                                                                                                                                                                                                    TTCTGGACCTGCCTAAG GGCCTGAATTTTCTGCTCCTGAATTCAAA|||||||||||||||||||||||||||||||||||||||||||||||||||||||||||||||||||||||||||||||||||||||||||||||||||||||||||||||||||||||||||||||||||||||||||||||||||||||||||||||||||||||||||||||||||||||||||||||||||||||||||||||||||||||||||||||||||||||||||||||||||||||||||||||||||||||||||||
chr12 chr12 57898065 59199752 17M59195042F29M4652N30M                                                                                                                                                                                                                                                                                                    TTCTGGACCTGCCTAAG GGCCTGAATTTTCTGCTCCTGAATTCAAA|||||||||||||||||||||||||||||||||||||||||||||||||||||||||||||||||||||||||||||||||||||||||||||||||||||||||||||||||||||||||||||||||||||||||||||||||||||||||||||||||||||||||||||||||||||||||||||||||||||||||||||||||||||||||||||||||||||||||||||||||||||||||||||||||||||||||||||
chr12 chr12 57898065 59199752 17M59195042F29M4652N30M                                                                                                                                                                                                                                                                                                    TTCTGGACCTGCCTAAG GGCCTGAATTTTCTGCTCCTGAATTCAAA|||||||||||||||||||||||||||||||||||||||||||||||||||||||||||||||||||||||||||||||||||||||||||||||||||||||||||||||||||||||||||||||||||||||||||||||||||||||||||||||||||||||||||||||||||||||||||||||||||||||||||||||||||||||||||||||||||||||||||||||||||||||||||||||||||||||||||||
chr12 chr12 57898065 59196637 17M59195042F29M1537N30M                                                                                                                                                                                                                                                                                                    TTCTGGACCTGCCTAAG GGCCTGAATTTTCTGCTCCTGAATTCAAA|||||||||||||||||||||||||||||||||||||||||||||||||||||||||||||||||||||||||||||||||||||||||||||||||||||||||||||||||||||||||||||||||||||||||||||||||||||||||||||||||||||||||||||||||||||||||||||||||||||||||||||||||||||||||||||||||||||||||||||||||||||||||||||||||||||||||||||
chr12 chr12 57898065 59196637 17M59195042F29M1537N30M                                                                                                                                                                                                                                                                                                    TTCTGGACCTGCCTAAG GGCCTGAATTTTCTGCTCCTGAATTCAAA|||||||||||||||||||||||||||||||||||||||||||||||||||||||||||||||||||||||||||||||||||||||||||||||||||||||||||||||||||||||||||||||||||||||||||||||||||||||||||||||||||||||||||||||||||||||||||||||||||||||||||||||||||||||||||||||||||||||||||||||||||||||||||||||||||||||||||||
chr12 chr12 57898065 59196637 17M59195042F29M1537N30M                                                                                                                                                                                                                                                                                                    TTCTGGACCTGCCTAAG GGCCTGAATTTTCTGCTCCTGAATTCAAA|||||||||||||||||||||||||||||||||||||||||||||||||||||||||||||||||||||||||||||||||||||||||||||||||||||||||||||||||||||||||||||||||||||||||||||||||||||||||||||||||||||||||||||||||||||||||||||||||||||||||||||||||||||||||||||||||||||||||||||||||||||||||||||||||||||||||||||
chr12 chr12 57898065 59196637 17M59195042F29M1537N30M                                                                                                                                                                                                                                                                                                    TTCTGGACCTGCCTAAG GGCCTGAATTTTCTGCTCCTGAATTCAAA|||||||||||||||||||||||||||||||||||||||||||||||||||||||||||||||||||||||||||||||||||||||||||||||||||||||||||||||||||||||||||||||||||||||||||||||||||||||||||||||||||||||||||||||||||||||||||||||||||||||||||||||||||||||||||||||||||||||||||||||||||||||||||||||||||||||||||||
chr12 chr12 57898065 59196637 17M59195042F29M1537N30M                                                                                                                                                                                                                                                                                                    TTCTGGACCTGCCTAAG GGCCTGAATTTTCTGCTCCTGAATTCAAA|||||||||||||||||||||||||||||||||||||||||||||||||||||||||||||||||||||||||||||||||||||||||||||||||||||||||||||||||||||||||||||||||||||||||||||||||||||||||||||||||||||||||||||||||||||||||||||||||||||||||||||||||||||||||||||||||||||||||||||||||||||||||||||||||||||||||||||
chr12 chr12 57898065 59196637 17M59195042F29M1537N30M                                                                                                                                                                                                                                                                                                    TTCTGGACCTGCCTAAG GGCCTGAATTTTCTGCTCCTGAATTCAAA|||||||||||||||||||||||||||||||||||||||||||||||||||||||||||||||||||||||||||||||||||||||||||||||||||||||||||||||||||||||||||||||||||||||||||||||||||||||||||||||||||||||||||||||||||||||||||||||||||||||||||||||||||||||||||||||||||||||||||||||||||||||||||||||||||||||||||||
chr12 chr12 57898065 59196637 17M59195042F29M1537N30M                                                                                                                                                                                                                                                                                                    TTCTGGACCTGCCTAAG GGCCTGAATTTTCTGCTCCTGAATTCAAA|||||||||||||||||||||||||||||||||||||||||||||||||||||||||||||||||||||||||||||||||||||||||||||||||||||||||||||||||||||||||||||||||||||||||||||||||||||||||||||||||||||||||||||||||||||||||||||||||||||||||||||||||||||||||||||||||||||||||||||||||||||||||||||||||||||||||||||
chr12 chr12 57898065 59199752 17M59195042F29M4652N30M                                                                                                                                                                                                                                                                                                    TTCTGGACCTGCCTAAG GGCCTGAATTTTCTGCTCCTGAATTCAAA|||||||||||||||||||||||||||||||||||||||||||||||||||||||||||||||||||||||||||||||||||||||||||||||||||||||||||||||||||||||||||||||||||||||||||||||||||||||||||||||||||||||||||||||||||||||||||||||||||||||||||||||||||||||||||||||||||||||||||||||||||||||||||||||||||||||||||||
chr12 chr12 57898065 59196637 17M59195042F29M1537N30M                                                                                                                                                                                                                                                                                                    TTCTGGACCTGCCTAAG GGCCTGAATTTTCTGCTCCTGAATTCAAA|||||||||||||||||||||||||||||||||||||||||||||||||||||||||||||||||||||||||||||||||||||||||||||||||||||||||||||||||||||||||||||||||||||||||||||||||||||||||||||||||||||||||||||||||||||||||||||||||||||||||||||||||||||||||||||||||||||||||||||||||||||||||||||||||||||||||||||
chr12 chr12 57898065 59196637 17M59195042F29M1537N30M                                                                                                                                                                                                                                                                                                    TTCTGGACCTGCCTAAG GGCCTGAATTTTCTGCTCCTGAATTCAAA|||||||||||||||||||||||||||||||||||||||||||||||||||||||||||||||||||||||||||||||||||||||||||||||||||||||||||||||||||||||||||||||||||||||||||||||||||||||||||||||||||||||||||||||||||||||||||||||||||||||||||||||||||||||||||||||||||||||||||||||||||||||||||||||||||||||||||||
chr12 chr12 57898065 59196637 17M59195042F29M1537N30M                                                                                                                                                                                                                                                                                                    TTCTGGCCCTGCCTAAG GGCCTGAATTTTCTGCTCCTGAATTCAAA|||||||||||||||||||||||||||||||||||||||||||||||||||||||||||||||||||||||||||||||||||||||||||||||||||||||||||||||||||||||||||||||||||||||||||||||||||||||||||||||||||||||||||||||||||||||||||||||||||||||||||||||||||||||||||||||||||||||||||||||||||||||||||||||||||||||||||||
chr12 chr12 57898065 59196637 17M59195042F29M1537N30M                                                                                                                                                                                                                                                                                                    TTCTTGACCTGTCTAAG GGCCTGAATTTTCTGCTCCTGAATTCAAA|||||||||||||||||||||||||||||||||||||||||||||||||||||||||||||||||||||||||||||||||||||||||||||||||||||||||||||||||||||||||||||||||||||||||||||||||||||||||||||||||||||||||||||||||||||||||||||||||||||||||||||||||||||||||||||||||||||||||||||||||||||||||||||||||||||||||||||
chr12 chr12 57898065 59196637 17M59195042F29M1537N30M                                                                                                                                                                                                                                                                                                    TTCTGGACCTGCCTAAG GGCCTGAATTTTCTGCTCCTGAATTCAAA|||||||||||||||||||||||||||||||||||||||||||||||||||||||||||||||||||||||||||||||||||||||||||||||||||||||||||||||||||||||||||||||||||||||||||||||||||||||||||||||||||||||||||||||||||||||||||||||||||||||||||||||||||||||||||||||||||||||||||||||||||||||||||||||||||||||||||||
chr12 chr12 57898065 59196637 17M59195042F29M1537N30M                                                                                                                                                                                                                                                                                                    TTCTGGACCTGCCTAAG GGCCTGAATTTTCTGCTCCTGAATTCAAA|||||||||||||||||||||||||||||||||||||||||||||||||||||||||||||||||||||||||||||||||||||||||||||||||||||||||||||||||||||||||||||||||||||||||||||||||||||||||||||||||||||||||||||||||||||||||||||||||||||||||||||||||||||||||||||||||||||||||||||||||||||||||||||||||||||||||||||
chr12 chr12 57898066 59199753 16M59195042F29M4652N31M                                                                                                                                                                                                                                                                                                     TCTGGACCTGCCTAAG GGCCTGAATTTTCTGCTCCTGAATTCAAA|||||||||||||||||||||||||||||||||||||||||||||||||||||||||||||||||||||||||||||||||||||||||||||||||||||||||||||||||||||||||||||||||||||||||||||||||||||||||||||||||||||||||||||||||||||||||||||||||||||||||||||||||||||||||||||||||||||||||||||||||||||||||||||||||||||||||||||
chr12 chr12 57898066 59199753 16M59195042F29M4652N31M                                                                                                                                                                                                                                                                                                     TCTGGACCTGCCTAAG GGCCTGAATTTTCTGCTCCTGAATTCAAA|||||||||||||||||||||||||||||||||||||||||||||||||||||||||||||||||||||||||||||||||||||||||||||||||||||||||||||||||||||||||||||||||||||||||||||||||||||||||||||||||||||||||||||||||||||||||||||||||||||||||||||||||||||||||||||||||||||||||||||||||||||||||||||||||||||||||||||
chr12 chr12 57898066 59196638 16M59195042F29M1537N31M                                                                                                                                                                                                                                                                                                     TCTGGACCTGCCTAAG GGCCTGAATTTTCTGCTCCTGAATTCAAA|||||||||||||||||||||||||||||||||||||||||||||||||||||||||||||||||||||||||||||||||||||||||||||||||||||||||||||||||||||||||||||||||||||||||||||||||||||||||||||||||||||||||||||||||||||||||||||||||||||||||||||||||||||||||||||||||||||||||||||||||||||||||||||||||||||||||||||
chr12 chr12 57898066 59196638 16M59195042F29M1537N31M                                                                                                                                                                                                                                                                                                     TCTGGACCTGCCTAAG GGCCTGAATTTTCTGCTCCTGAATTCAAA|||||||||||||||||||||||||||||||||||||||||||||||||||||||||||||||||||||||||||||||||||||||||||||||||||||||||||||||||||||||||||||||||||||||||||||||||||||||||||||||||||||||||||||||||||||||||||||||||||||||||||||||||||||||||||||||||||||||||||||||||||||||||||||||||||||||||||||
chr12 chr12 57898066 59196743 16M59195042F29M1642N31M                                                                                                                                                                                                                                                                                                     GCTGGACCTGCCTAAG GGCCTGAATTTTCTGCTCCTGAATTCAAA|||||||||||||||||||||||||||||||||||||||||||||||||||||||||||||||||||||||||||||||||||||||||||||||||||||||||||||||||||||||||||||||||||||||||||||||||||||||||||||||||||||||||||||||||||||||||||||||||||||||||||||||||||||||||||||||||||||||||||||||||||||||||||||||||||||||||||||
chr12 chr12 57898066 59196638 16M59195042F29M1537N31M                                                                                                                                                                                                                                                                                                     TCTGGACCTGCCTAAG GGCCTGAATTTTCTGCTCCTGAATTCAAA|||||||||||||||||||||||||||||||||||||||||||||||||||||||||||||||||||||||||||||||||||||||||||||||||||||||||||||||||||||||||||||||||||||||||||||||||||||||||||||||||||||||||||||||||||||||||||||||||||||||||||||||||||||||||||||||||||||||||||||||||||||||||||||||||||||||||||||
chr12 chr12 57898066 59196638 16M59195042F29M1537N31M                                                                                                                                                                                                                                                                                                     TCTGGACCTGCCTAAG GGCCTGAATTTTCTGCTCCTGAATTCAAA|||||||||||||||||||||||||||||||||||||||||||||||||||||||||||||||||||||||||||||||||||||||||||||||||||||||||||||||||||||||||||||||||||||||||||||||||||||||||||||||||||||||||||||||||||||||||||||||||||||||||||||||||||||||||||||||||||||||||||||||||||||||||||||||||||||||||||||
chr12 chr12 57898066 59196638 16M59195042F29M1537N31M                                                                                                                                                                                                                                                                                                     TCTGGACCTGCCTAAG GGCCTGAATTTTCTGCTCCTGAATTCAAA|||||||||||||||||||||||||||||||||||||||||||||||||||||||||||||||||||||||||||||||||||||||||||||||||||||||||||||||||||||||||||||||||||||||||||||||||||||||||||||||||||||||||||||||||||||||||||||||||||||||||||||||||||||||||||||||||||||||||||||||||||||||||||||||||||||||||||||
chr12 chr12 57898066 59196638 16M59195042F29M1537N31M                                                                                                                                                                                                                                                                                                     TCTGGACCTGCCTAAG GGCCTGAATTTTCTGCTCCTGAATTCAAA|||||||||||||||||||||||||||||||||||||||||||||||||||||||||||||||||||||||||||||||||||||||||||||||||||||||||||||||||||||||||||||||||||||||||||||||||||||||||||||||||||||||||||||||||||||||||||||||||||||||||||||||||||||||||||||||||||||||||||||||||||||||||||||||||||||||||||||
chr12 chr12 57898066 59196638 16M59195042F29M1537N31M                                                                                                                                                                                                                                                                                                     TCTGGACCTGCCTAAG GGCCTGAATTTTCTGCTCCTGAATTCAAA|||||||||||||||||||||||||||||||||||||||||||||||||||||||||||||||||||||||||||||||||||||||||||||||||||||||||||||||||||||||||||||||||||||||||||||||||||||||||||||||||||||||||||||||||||||||||||||||||||||||||||||||||||||||||||||||||||||||||||||||||||||||||||||||||||||||||||||
chr12 chr12 57898066 59196638 16M59195042F29M1537N31M                                                                                                                                                                                                                                                                                                     TCTGGACCTGCCTAAG GGCCTGAATTTTCTGCTCCTGAATTCAAA|||||||||||||||||||||||||||||||||||||||||||||||||||||||||||||||||||||||||||||||||||||||||||||||||||||||||||||||||||||||||||||||||||||||||||||||||||||||||||||||||||||||||||||||||||||||||||||||||||||||||||||||||||||||||||||||||||||||||||||||||||||||||||||||||||||||||||||
chr12 chr12 57898066 59196716 16M59195042F29M1615N31M                                                                                                                                                                                                                                                                                                     TCTGGACCTGCCTAAG GGCCTGAATTTTCTGCTCCTGAATTCAAA|||||||||||||||||||||||||||||||||||||||||||||||||||||||||||||||||||||||||||||||||||||||||||||||||||||||||||||||||||||||||||||||||||||||||||||||||||||||||||||||||||||||||||||||||||||||||||||||||||||||||||||||||||||||||||||||||||||||||||||||||||||||||||||||||||||||||||||
chr12 chr12 57898066 59199753 16M59195042F29M4652N31M                                                                                                                                                                                                                                                                                                     TCTGGACCTGCCTAAG GGCCTGAATTTTCTGCTCCTGAATTCAAA|||||||||||||||||||||||||||||||||||||||||||||||||||||||||||||||||||||||||||||||||||||||||||||||||||||||||||||||||||||||||||||||||||||||||||||||||||||||||||||||||||||||||||||||||||||||||||||||||||||||||||||||||||||||||||||||||||||||||||||||||||||||||||||||||||||||||||||
chr12 chr12 57898066 59199753 16M59195042F29M4652N31M                                                                                                                                                                                                                                                                                                     TCTGGACCTGCCTAAG GGCCTGAATTTTCTGCTCCTGAATTCAAA|||||||||||||||||||||||||||||||||||||||||||||||||||||||||||||||||||||||||||||||||||||||||||||||||||||||||||||||||||||||||||||||||||||||||||||||||||||||||||||||||||||||||||||||||||||||||||||||||||||||||||||||||||||||||||||||||||||||||||||||||||||||||||||||||||||||||||||
chr12 chr12 57898066 59199753 16M59195042F29M4652N31M                                                                                                                                                                                                                                                                                                     TCTGGACCTGCCTAAG GGCCTGAATTTTCTGCTCCTGAATTCAAA|||||||||||||||||||||||||||||||||||||||||||||||||||||||||||||||||||||||||||||||||||||||||||||||||||||||||||||||||||||||||||||||||||||||||||||||||||||||||||||||||||||||||||||||||||||||||||||||||||||||||||||||||||||||||||||||||||||||||||||||||||||||||||||||||||||||||||||
chr12 chr12 57898066 59199753 16M59195042F29M4652N31M                                                                                                                                                                                                                                                                                                     TCTGGACCTGCCTAAG GGCCTGAATTTTCTGCTCCTGAATTCAAA|||||||||||||||||||||||||||||||||||||||||||||||||||||||||||||||||||||||||||||||||||||||||||||||||||||||||||||||||||||||||||||||||||||||||||||||||||||||||||||||||||||||||||||||||||||||||||||||||||||||||||||||||||||||||||||||||||||||||||||||||||||||||||||||||||||||||||||
chr12 chr12 57898066 59199753 16M59195042F29M4652N31M                                                                                                                                                                                                                                                                                                     TCTGGACCTGCCTAAG GGCCTGAATTTTCTGCTCCTGAATTCAAA|||||||||||||||||||||||||||||||||||||||||||||||||||||||||||||||||||||||||||||||||||||||||||||||||||||||||||||||||||||||||||||||||||||||||||||||||||||||||||||||||||||||||||||||||||||||||||||||||||||||||||||||||||||||||||||||||||||||||||||||||||||||||||||||||||||||||||||
chr12 chr12 57898066 59199753 16M59195042F29M4652N31M                                                                                                                                                                                                                                                                                                     TCTGGACCTGCCTAAG GGCCTGAATTTTCTGCTCCTGAATTCAAA|||||||||||||||||||||||||||||||||||||||||||||||||||||||||||||||||||||||||||||||||||||||||||||||||||||||||||||||||||||||||||||||||||||||||||||||||||||||||||||||||||||||||||||||||||||||||||||||||||||||||||||||||||||||||||||||||||||||||||||||||||||||||||||||||||||||||||||
chr12 chr12 57898066 59199753 16M59195042F29M4652N31M                                                                                                                                                                                                                                                                                                     TCTGGACCTGCCTAAG GGCCTGAATTTTCTGCTCCTGAATTCAAA|||||||||||||||||||||||||||||||||||||||||||||||||||||||||||||||||||||||||||||||||||||||||||||||||||||||||||||||||||||||||||||||||||||||||||||||||||||||||||||||||||||||||||||||||||||||||||||||||||||||||||||||||||||||||||||||||||||||||||||||||||||||||||||||||||||||||||||
chr12 chr12 57898066 59199753 16M59195042F29M4652N31M                                                                                                                                                                                                                                                                                                     TCTGGACCTGCCTAAG GGCCTGAATTTTCTGCTCCTGAATTCAAA|||||||||||||||||||||||||||||||||||||||||||||||||||||||||||||||||||||||||||||||||||||||||||||||||||||||||||||||||||||||||||||||||||||||||||||||||||||||||||||||||||||||||||||||||||||||||||||||||||||||||||||||||||||||||||||||||||||||||||||||||||||||||||||||||||||||||||||
chr12 chr12 57898066 59199753 16M59195042F29M4652N31M                                                                                                                                                                                                                                                                                                     TCTGGACCTGCCTAAG GGCCTGAATTTTCTGCTCCTGAATTCAAA|||||||||||||||||||||||||||||||||||||||||||||||||||||||||||||||||||||||||||||||||||||||||||||||||||||||||||||||||||||||||||||||||||||||||||||||||||||||||||||||||||||||||||||||||||||||||||||||||||||||||||||||||||||||||||||||||||||||||||||||||||||||||||||||||||||||||||||
chr12 chr12 57898066 59196638 16M59195042F29M1537N31M                                                                                                                                                                                                                                                                                                     TCTGGACCTGCCTAAG GGCCTGAATTTTCTGCTCCTGAATTCAAA|||||||||||||||||||||||||||||||||||||||||||||||||||||||||||||||||||||||||||||||||||||||||||||||||||||||||||||||||||||||||||||||||||||||||||||||||||||||||||||||||||||||||||||||||||||||||||||||||||||||||||||||||||||||||||||||||||||||||||||||||||||||||||||||||||||||||||||
chr12 chr12 57898066 59196638 16M59195042F29M1537N31M                                                                                                                                                                                                                                                                                                     TCTGGACCTGCCTAAG GGCCTGAATTTTCTGCTCCTGAATTCAAA|||||||||||||||||||||||||||||||||||||||||||||||||||||||||||||||||||||||||||||||||||||||||||||||||||||||||||||||||||||||||||||||||||||||||||||||||||||||||||||||||||||||||||||||||||||||||||||||||||||||||||||||||||||||||||||||||||||||||||||||||||||||||||||||||||||||||||||
chr12 chr12 57898066 59196638 16M59195042F29M1537N31M                                                                                                                                                                                                                                                                                                     TCTGGACCTGCCTAAG GGCCTGAATTTTCTGCTCCTGAATTCAAA|||||||||||||||||||||||||||||||||||||||||||||||||||||||||||||||||||||||||||||||||||||||||||||||||||||||||||||||||||||||||||||||||||||||||||||||||||||||||||||||||||||||||||||||||||||||||||||||||||||||||||||||||||||||||||||||||||||||||||||||||||||||||||||||||||||||||||||
chr12 chr12 57898066 59196638 16M59195042F29M1537N31M                                                                                                                                                                                                                                                                                                     TCTGGACCTGCCTAAG GGCCTGAATTTTCTGCTCCTGAATTCAAA|||||||||||||||||||||||||||||||||||||||||||||||||||||||||||||||||||||||||||||||||||||||||||||||||||||||||||||||||||||||||||||||||||||||||||||||||||||||||||||||||||||||||||||||||||||||||||||||||||||||||||||||||||||||||||||||||||||||||||||||||||||||||||||||||||||||||||||
chr12 chr12 57898066 59196638 16M59195042F29M1537N31M                                                                                                                                                                                                                                                                                                     TCTGGACCTGCCTAAG GGCCTGAATTTTCTGCTCCTGAATTCAAA|||||||||||||||||||||||||||||||||||||||||||||||||||||||||||||||||||||||||||||||||||||||||||||||||||||||||||||||||||||||||||||||||||||||||||||||||||||||||||||||||||||||||||||||||||||||||||||||||||||||||||||||||||||||||||||||||||||||||||||||||||||||||||||||||||||||||||||
chr12 chr12 57898066 59196638 16M59195042F29M1537N31M                                                                                                                                                                                                                                                                                                     TCTGGACCTGCCTAAG GGCCTGAATTTTCTGCTCCTGAATTCAAA|||||||||||||||||||||||||||||||||||||||||||||||||||||||||||||||||||||||||||||||||||||||||||||||||||||||||||||||||||||||||||||||||||||||||||||||||||||||||||||||||||||||||||||||||||||||||||||||||||||||||||||||||||||||||||||||||||||||||||||||||||||||||||||||||||||||||||||
chr12 chr12 57898066 59196638 16M59195042F29M1537N31M                                                                                                                                                                                                                                                                                                     TCTGGACCTGCCTAAG GGCCTGAATTTTCTGCTCCTGAATTCAAA|||||||||||||||||||||||||||||||||||||||||||||||||||||||||||||||||||||||||||||||||||||||||||||||||||||||||||||||||||||||||||||||||||||||||||||||||||||||||||||||||||||||||||||||||||||||||||||||||||||||||||||||||||||||||||||||||||||||||||||||||||||||||||||||||||||||||||||
chr12 chr12 57898066 59196638 16M59195042F29M1537N31M                                                                                                                                                                                                                                                                                                     TCTGGACCTGCCTAAG GGCCTGAATTTTCTGCTCCTGAATTCAAA|||||||||||||||||||||||||||||||||||||||||||||||||||||||||||||||||||||||||||||||||||||||||||||||||||||||||||||||||||||||||||||||||||||||||||||||||||||||||||||||||||||||||||||||||||||||||||||||||||||||||||||||||||||||||||||||||||||||||||||||||||||||||||||||||||||||||||||
chr12 chr12 57898066 59196638 16M59195042F29M1537N31M                                                                                                                                                                                                                                                                                                     TCTGGACCTGCCTAAG GGCCTGAATTTTCTGCTCCTGAATTCAAA|||||||||||||||||||||||||||||||||||||||||||||||||||||||||||||||||||||||||||||||||||||||||||||||||||||||||||||||||||||||||||||||||||||||||||||||||||||||||||||||||||||||||||||||||||||||||||||||||||||||||||||||||||||||||||||||||||||||||||||||||||||||||||||||||||||||||||||
chr12 chr12 57898066 59196638 16M59195042F29M1537N31M                                                                                                                                                                                                                                                                                                     TCTGGACCTGCCTAAG GGCCTGAATTTTCTGCTCCTGAATTCAAA|||||||||||||||||||||||||||||||||||||||||||||||||||||||||||||||||||||||||||||||||||||||||||||||||||||||||||||||||||||||||||||||||||||||||||||||||||||||||||||||||||||||||||||||||||||||||||||||||||||||||||||||||||||||||||||||||||||||||||||||||||||||||||||||||||||||||||||
chr12 chr12 57898066 59196638 16M59195042F29M1537N31M                                                                                                                                                                                                                                                                                                     TCTGGACCTGCCTAAG GGCCTGAATTTTCTGCTCCTGAATTCAAA|||||||||||||||||||||||||||||||||||||||||||||||||||||||||||||||||||||||||||||||||||||||||||||||||||||||||||||||||||||||||||||||||||||||||||||||||||||||||||||||||||||||||||||||||||||||||||||||||||||||||||||||||||||||||||||||||||||||||||||||||||||||||||||||||||||||||||||
chr12 chr12 57898066 59196638 16M59195042F29M1537N31M                                                                                                                                                                                                                                                                                                     TCTGGACCTGCCTAAG GGCCTGAATTTTCTGCTCCTGAATTCAAA|||||||||||||||||||||||||||||||||||||||||||||||||||||||||||||||||||||||||||||||||||||||||||||||||||||||||||||||||||||||||||||||||||||||||||||||||||||||||||||||||||||||||||||||||||||||||||||||||||||||||||||||||||||||||||||||||||||||||||||||||||||||||||||||||||||||||||||
chr12 chr12 57898066 59196638 16M59195042F29M1537N31M                                                                                                                                                                                                                                                                                                     TCTGGACCTGCCTAAG GGCCTGAATTTTCTGCTCCTGAATTCAAA|||||||||||||||||||||||||||||||||||||||||||||||||||||||||||||||||||||||||||||||||||||||||||||||||||||||||||||||||||||||||||||||||||||||||||||||||||||||||||||||||||||||||||||||||||||||||||||||||||||||||||||||||||||||||||||||||||||||||||||||||||||||||||||||||||||||||||||
chr12 chr12 57898066 59196638 16M59195042F29M1537N31M                                                                                                                                                                                                                                                                                                     TCTGGACCTGCCTAAG GGCCTGAATTTTCTGCTCCTGAATTCAAA|||||||||||||||||||||||||||||||||||||||||||||||||||||||||||||||||||||||||||||||||||||||||||||||||||||||||||||||||||||||||||||||||||||||||||||||||||||||||||||||||||||||||||||||||||||||||||||||||||||||||||||||||||||||||||||||||||||||||||||||||||||||||||||||||||||||||||||
chr12 chr12 57898066 59196638 16M59195042F29M1537N31M                                                                                                                                                                                                                                                                                                     TCTGGACCTGCCTAAG GGCCTGAATTTTCTGCTCCTGAATTCAAA|||||||||||||||||||||||||||||||||||||||||||||||||||||||||||||||||||||||||||||||||||||||||||||||||||||||||||||||||||||||||||||||||||||||||||||||||||||||||||||||||||||||||||||||||||||||||||||||||||||||||||||||||||||||||||||||||||||||||||||||||||||||||||||||||||||||||||||
chr12 chr12 57898066 59196638 16M59195042F29M1537N31M                                                                                                                                                                                                                                                                                                     TCTGGACCTGCCTAAG GGCCTGAATTTTCTGCTCCTGAATTCAAA|||||||||||||||||||||||||||||||||||||||||||||||||||||||||||||||||||||||||||||||||||||||||||||||||||||||||||||||||||||||||||||||||||||||||||||||||||||||||||||||||||||||||||||||||||||||||||||||||||||||||||||||||||||||||||||||||||||||||||||||||||||||||||||||||||||||||||||
chr12 chr12 57898066 59196638 16M59195042F29M1537N31M                                                                                                                                                                                                                                                                                                     TCTGGACCTGCCTAAG GGCCTGAATTTTCTGCTCCTGAATTCAAA|||||||||||||||||||||||||||||||||||||||||||||||||||||||||||||||||||||||||||||||||||||||||||||||||||||||||||||||||||||||||||||||||||||||||||||||||||||||||||||||||||||||||||||||||||||||||||||||||||||||||||||||||||||||||||||||||||||||||||||||||||||||||||||||||||||||||||||
chr12 chr12 57898066 59196638 16M59195042F29M1537N31M                                                                                                                                                                                                                                                                                                     TCTGGACCTGCCTAAG GGCCTGAATTTTCTGCTCCTGAATTCAAA|||||||||||||||||||||||||||||||||||||||||||||||||||||||||||||||||||||||||||||||||||||||||||||||||||||||||||||||||||||||||||||||||||||||||||||||||||||||||||||||||||||||||||||||||||||||||||||||||||||||||||||||||||||||||||||||||||||||||||||||||||||||||||||||||||||||||||||
chr12 chr12 57898067 59196639 15M59195042F29M1537N32M                                                                                                                                                                                                                                                                                                      CTGGACCTGCCTAAG GGCCTGAATTTTCTGCTCCTGAATTCAAA|||||||||||||||||||||||||||||||||||||||||||||||||||||||||||||||||||||||||||||||||||||||||||||||||||||||||||||||||||||||||||||||||||||||||||||||||||||||||||||||||||||||||||||||||||||||||||||||||||||||||||||||||||||||||||||||||||||||||||||||||||||||||||||||||||||||||||||
chr12 chr12 57898067 59196639 15M59195042F29M1537N32M                                                                                                                                                                                                                                                                                                      CTGGACCTGCCTAAG GGCCTGAATTTTCTGCTCCTGAATTCAAA|||||||||||||||||||||||||||||||||||||||||||||||||||||||||||||||||||||||||||||||||||||||||||||||||||||||||||||||||||||||||||||||||||||||||||||||||||||||||||||||||||||||||||||||||||||||||||||||||||||||||||||||||||||||||||||||||||||||||||||||||||||||||||||||||||||||||||||
chr12 chr12 57898067 59196639 15M59195042F29M1537N32M                                                                                                                                                                                                                                                                                                      CTGGACCTGCCTAAG GGCCTGAATTTTCTGCTCCTGAATTCAAA|||||||||||||||||||||||||||||||||||||||||||||||||||||||||||||||||||||||||||||||||||||||||||||||||||||||||||||||||||||||||||||||||||||||||||||||||||||||||||||||||||||||||||||||||||||||||||||||||||||||||||||||||||||||||||||||||||||||||||||||||||||||||||||||||||||||||||||
chr12 chr12 57898067 59196639 15M59195042F29M1537N32M                                                                                                                                                                                                                                                                                                      CTGGACCTGCCTAAG GGCCTGAATTTTCTGCTCCTGAATTCAAA|||||||||||||||||||||||||||||||||||||||||||||||||||||||||||||||||||||||||||||||||||||||||||||||||||||||||||||||||||||||||||||||||||||||||||||||||||||||||||||||||||||||||||||||||||||||||||||||||||||||||||||||||||||||||||||||||||||||||||||||||||||||||||||||||||||||||||||
chr12 chr12 57898067 59196639 15M59195042F29M1537N32M                                                                                                                                                                                                                                                                                                      CTGGACCTGCCTAAG GGCCTGAATTTTCTGCTCCTGAATTCAAA|||||||||||||||||||||||||||||||||||||||||||||||||||||||||||||||||||||||||||||||||||||||||||||||||||||||||||||||||||||||||||||||||||||||||||||||||||||||||||||||||||||||||||||||||||||||||||||||||||||||||||||||||||||||||||||||||||||||||||||||||||||||||||||||||||||||||||||
chr12 chr12 57898067 59196639 15M59195042F29M1537N32M                                                                                                                                                                                                                                                                                                      CTGGACCTGCCTAAG GGCCTGAATTTTCTGCTCCTGAATTCAAA|||||||||||||||||||||||||||||||||||||||||||||||||||||||||||||||||||||||||||||||||||||||||||||||||||||||||||||||||||||||||||||||||||||||||||||||||||||||||||||||||||||||||||||||||||||||||||||||||||||||||||||||||||||||||||||||||||||||||||||||||||||||||||||||||||||||||||||
chr12 chr12 57898067 59196717 15M59195042F29M1615N32M                                                                                                                                                                                                                                                                                                      CTGGACCTGCCTAAG GGCCTGAATTTTCTGCTCCTGAATTCAAA|||||||||||||||||||||||||||||||||||||||||||||||||||||||||||||||||||||||||||||||||||||||||||||||||||||||||||||||||||||||||||||||||||||||||||||||||||||||||||||||||||||||||||||||||||||||||||||||||||||||||||||||||||||||||||||||||||||||||||||||||||||||||||||||||||||||||||||
chr12 chr12 57898067 59199754 15M59195042F29M4652N32M                                                                                                                                                                                                                                                                                                      CTGGACCTGCCTAAG GGCCTGAATTTTCTGCTCCTGAATTCAAA|||||||||||||||||||||||||||||||||||||||||||||||||||||||||||||||||||||||||||||||||||||||||||||||||||||||||||||||||||||||||||||||||||||||||||||||||||||||||||||||||||||||||||||||||||||||||||||||||||||||||||||||||||||||||||||||||||||||||||||||||||||||||||||||||||||||||||||
chr12 chr12 57898067 59206227 15M59195042F29M11125N32M                                                                                                                                                                                                                                                                                                     CTGGACCTGCCTAAG GGCCTGAATTTTCTGCTCCTGAATTCAAA|||||||||||||||||||||||||||||||||||||||||||||||||||||||||||||||||||||||||||||||||||||||||||||||||||||||||||||||||||||||||||||||||||||||||||||||||||||||||||||||||||||||||||||||||||||||||||||||||||||||||||||||||||||||||||||||||||||||||||||||||||||||||||||||||||||||||||||
chr12 chr12 57898067 59199754 15M59195042F29M4652N32M                                                                                                                                                                                                                                                                                                      CTGGACCTGCCTAAG GGCCTGAATTTTCTGCTCCTGAATTCAAA|||||||||||||||||||||||||||||||||||||||||||||||||||||||||||||||||||||||||||||||||||||||||||||||||||||||||||||||||||||||||||||||||||||||||||||||||||||||||||||||||||||||||||||||||||||||||||||||||||||||||||||||||||||||||||||||||||||||||||||||||||||||||||||||||||||||||||||
chr12 chr12 57898067 59196639 15M59195042F29M1537N32M                                                                                                                                                                                                                                                                                                      CTGGACCTGCCTAAG GGCCTGAATTTTCTGCTCCTGAATTCAAA|||||||||||||||||||||||||||||||||||||||||||||||||||||||||||||||||||||||||||||||||||||||||||||||||||||||||||||||||||||||||||||||||||||||||||||||||||||||||||||||||||||||||||||||||||||||||||||||||||||||||||||||||||||||||||||||||||||||||||||||||||||||||||||||||||||||||||||
chr12 chr12 57898067 59196639 15M59195042F29M1537N32M                                                                                                                                                                                                                                                                                                      CTGGACCTGCCTAAG GGCCTGAATTTTCTGCTCCTGAATTCAAA|||||||||||||||||||||||||||||||||||||||||||||||||||||||||||||||||||||||||||||||||||||||||||||||||||||||||||||||||||||||||||||||||||||||||||||||||||||||||||||||||||||||||||||||||||||||||||||||||||||||||||||||||||||||||||||||||||||||||||||||||||||||||||||||||||||||||||||
chr12 chr12 57898067 59199754 15M59195042F29M4652N32M                                                                                                                                                                                                                                                                                                      CTGGACCTGCCTAAG GGCCTGAATTTTCTGCTCCTGAATTCAAA|||||||||||||||||||||||||||||||||||||||||||||||||||||||||||||||||||||||||||||||||||||||||||||||||||||||||||||||||||||||||||||||||||||||||||||||||||||||||||||||||||||||||||||||||||||||||||||||||||||||||||||||||||||||||||||||||||||||||||||||||||||||||||||||||||||||||||||
chr12 chr12 57898068 59196640 14M59195042F29M1537N33M                                                                                                                                                                                                                                                                                                       TGGACCTGCCTAAG GGCCTGAATTTTCTGCTCCTGAATTCAAA|||||||||||||||||||||||||||||||||||||||||||||||||||||||||||||||||||||||||||||||||||||||||||||||||||||||||||||||||||||||||||||||||||||||||||||||||||||||||||||||||||||||||||||||||||||||||||||||||||||||||||||||||||||||||||||||||||||||||||||||||||||||||||||||||||||||||||||
chr12 chr12 57898068 59196640 14M59195042F29M1537N33M                                                                                                                                                                                                                                                                                                       TGGACCTGCCTAAG GGCCTGAATTTTCTGCTCCTGAATTCAAA|||||||||||||||||||||||||||||||||||||||||||||||||||||||||||||||||||||||||||||||||||||||||||||||||||||||||||||||||||||||||||||||||||||||||||||||||||||||||||||||||||||||||||||||||||||||||||||||||||||||||||||||||||||||||||||||||||||||||||||||||||||||||||||||||||||||||||||
chr12 chr12 57898068 59196640 14M59195042F29M1537N33M                                                                                                                                                                                                                                                                                                       TGGACCTGCCTAAG GGCCTGAATTTTCTGCTCCTGAATTCAAA|||||||||||||||||||||||||||||||||||||||||||||||||||||||||||||||||||||||||||||||||||||||||||||||||||||||||||||||||||||||||||||||||||||||||||||||||||||||||||||||||||||||||||||||||||||||||||||||||||||||||||||||||||||||||||||||||||||||||||||||||||||||||||||||||||||||||||||
chr12 chr12 57898069 59199756 13M59195042F29M4652N34M                                                                                                                                                                                                                                                                                                        GGACCTGCCTAAG GGCCTGAATTTTCTGCTCCTGAATTCAAA|||||||||||||||||||||||||||||||||||||||||||||||||||||||||||||||||||||||||||||||||||||||||||||||||||||||||||||||||||||||||||||||||||||||||||||||||||||||||||||||||||||||||||||||||||||||||||||||||||||||||||||||||||||||||||||||||||||||||||||||||||||||||||||||||||||||||||||
chr12 chr12 57898069 59196641 13M59195042F29M1537N34M                                                                                                                                                                                                                                                                                                        GGACCTGCCTAAG GGCCTGAATTTTCTGCTCCTGAATTCAAA|||||||||||||||||||||||||||||||||||||||||||||||||||||||||||||||||||||||||||||||||||||||||||||||||||||||||||||||||||||||||||||||||||||||||||||||||||||||||||||||||||||||||||||||||||||||||||||||||||||||||||||||||||||||||||||||||||||||||||||||||||||||||||||||||||||||||||||
chr12 chr12 57898069 59199756 13M59195042F29M4652N34M                                                                                                                                                                                                                                                                                                        GGACCTGCCTAAG GGCCTGAATTTTCTGCTCCTGAATTCAAA|||||||||||||||||||||||||||||||||||||||||||||||||||||||||||||||||||||||||||||||||||||||||||||||||||||||||||||||||||||||||||||||||||||||||||||||||||||||||||||||||||||||||||||||||||||||||||||||||||||||||||||||||||||||||||||||||||||||||||||||||||||||||||||||||||||||||||||
chr12 chr12 57898069 59196641 13M59195042F29M1537N34M                                                                                                                                                                                                                                                                                                        GGACCTGCCTAAT GGCCTGAATTTTCTGCTCCTGAATTCAAA|||||||||||||||||||||||||||||||||||||||||||||||||||||||||||||||||||||||||||||||||||||||||||||||||||||||||||||||||||||||||||||||||||||||||||||||||||||||||||||||||||||||||||||||||||||||||||||||||||||||||||||||||||||||||||||||||||||||||||||||||||||||||||||||||||||||||||||
chr12 chr12 57898069 59196641 13M59195042F29M1537N34M                                                                                                                                                                                                                                                                                                        GGACCTGCCTAAG GGCCTGAATTTTCTGCTCCTGAATTCAAA|||||||||||||||||||||||||||||||||||||||||||||||||||||||||||||||||||||||||||||||||||||||||||||||||||||||||||||||||||||||||||||||||||||||||||||||||||||||||||||||||||||||||||||||||||||||||||||||||||||||||||||||||||||||||||||||||||||||||||||||||||||||||||||||||||||||||||||
chr12 chr12 57898069 59196641 13M59195042F29M1537N34M                                                                                                                                                                                                                                                                                                        GGACCTGCCTAAG GGCCTGAATTTTCTGCTCCTGAATTCAAA|||||||||||||||||||||||||||||||||||||||||||||||||||||||||||||||||||||||||||||||||||||||||||||||||||||||||||||||||||||||||||||||||||||||||||||||||||||||||||||||||||||||||||||||||||||||||||||||||||||||||||||||||||||||||||||||||||||||||||||||||||||||||||||||||||||||||||||
chr12 chr12 57898069 59196641 13M59195042F29M1537N34M                                                                                                                                                                                                                                                                                                        GGACCTGCCTAAG GGCCTGAATTTTCTGCTCCTGAATTCAAA|||||||||||||||||||||||||||||||||||||||||||||||||||||||||||||||||||||||||||||||||||||||||||||||||||||||||||||||||||||||||||||||||||||||||||||||||||||||||||||||||||||||||||||||||||||||||||||||||||||||||||||||||||||||||||||||||||||||||||||||||||||||||||||||||||||||||||||
chr12 chr12 57898070 59196642 12M59195042F29M1537N35M                                                                                                                                                                                                                                                                                                         GACCTGCCTAAG GGCCTGAATTTTCTGCTCCTGAATTCAAA|||||||||||||||||||||||||||||||||||||||||||||||||||||||||||||||||||||||||||||||||||||||||||||||||||||||||||||||||||||||||||||||||||||||||||||||||||||||||||||||||||||||||||||||||||||||||||||||||||||||||||||||||||||||||||||||||||||||||||||||||||||||||||||||||||||||||||||
chr12 chr12 57898070 59196642 12M59195042F29M1537N35M                                                                                                                                                                                                                                                                                                         GACCTGCCTAAG GGCCTGAATTTTCTGCTCCTGAATTCAAA|||||||||||||||||||||||||||||||||||||||||||||||||||||||||||||||||||||||||||||||||||||||||||||||||||||||||||||||||||||||||||||||||||||||||||||||||||||||||||||||||||||||||||||||||||||||||||||||||||||||||||||||||||||||||||||||||||||||||||||||||||||||||||||||||||||||||||||
chr12 chr12 57898070 59196642 12M59195042F29M1537N35M                                                                                                                                                                                                                                                                                                         GACCTGCCTAAG GGCCTGAATTTTCTGCTCCTGAATTCAAA|||||||||||||||||||||||||||||||||||||||||||||||||||||||||||||||||||||||||||||||||||||||||||||||||||||||||||||||||||||||||||||||||||||||||||||||||||||||||||||||||||||||||||||||||||||||||||||||||||||||||||||||||||||||||||||||||||||||||||||||||||||||||||||||||||||||||||||
chr12 chr12 57898070 59199757 12M59195042F29M4652N35M                                                                                                                                                                                                                                                                                                         GACCTGCCTAAG GGCCTGAATTTTCTGCTCCTGAATTCAAA|||||||||||||||||||||||||||||||||||||||||||||||||||||||||||||||||||||||||||||||||||||||||||||||||||||||||||||||||||||||||||||||||||||||||||||||||||||||||||||||||||||||||||||||||||||||||||||||||||||||||||||||||||||||||||||||||||||||||||||||||||||||||||||||||||||||||||||
chr12 chr12 57898070 59196642 12M59195042F29M1537N35M                                                                                                                                                                                                                                                                                                         GACCTGCCTAAG GGCCTGAATTTTCTGCTCCTGAATTCAAA|||||||||||||||||||||||||||||||||||||||||||||||||||||||||||||||||||||||||||||||||||||||||||||||||||||||||||||||||||||||||||||||||||||||||||||||||||||||||||||||||||||||||||||||||||||||||||||||||||||||||||||||||||||||||||||||||||||||||||||||||||||||||||||||||||||||||||||
chr12 chr12 57898070 59196642 12M59195042F29M1537N35M                                                                                                                                                                                                                                                                                                         GACCTGCCTAAG GGCCTGAATTTTCTGCTCCTGAATTCAAA|||||||||||||||||||||||||||||||||||||||||||||||||||||||||||||||||||||||||||||||||||||||||||||||||||||||||||||||||||||||||||||||||||||||||||||||||||||||||||||||||||||||||||||||||||||||||||||||||||||||||||||||||||||||||||||||||||||||||||||||||||||||||||||||||||||||||||||
chr12 chr12 57898070 59196642 12M59195042F29M1537N35M                                                                                                                                                                                                                                                                                                         GACCTGCCTAAG GGCCTGAATTTTCTGCTCCTGAATTCAAA|||||||||||||||||||||||||||||||||||||||||||||||||||||||||||||||||||||||||||||||||||||||||||||||||||||||||||||||||||||||||||||||||||||||||||||||||||||||||||||||||||||||||||||||||||||||||||||||||||||||||||||||||||||||||||||||||||||||||||||||||||||||||||||||||||||||||||||
chr12 chr12 57898070 59199725 12M59195042F29M1642N32M2978N3M                                                                                                                                                                                                                                                                                                  GACCTGCCTAAG GGCCTGAATTTTCTGCTCCTGAATTCAAA|||||||||||||||||||||||||||||||||||||||||||||||||||||||||||||||||||||||||||||||||||||||||||||||||||||||||||||||||||||||||||||||||||||||||||||||||||||||||||||||||||||||||||||||||||||||||||||||||||||||||||||||||||||||||||||||||||||||||||||||||||||||||||||||||||||||||||||
chr12 chr12 57898071 59196643 11M59195042F29M1537N36M                                                                                                                                                                                                                                                                                                          ACCTGCCTAAG GGCCTGAATTTTCTGCTCCTGAATTCAAA|||||||||||||||||||||||||||||||||||||||||||||||||||||||||||||||||||||||||||||||||||||||||||||||||||||||||||||||||||||||||||||||||||||||||||||||||||||||||||||||||||||||||||||||||||||||||||||||||||||||||||||||||||||||||||||||||||||||||||||||||||||||||||||||||||||||||||||
chr12 chr12 57898071 59196643 11M59195042F29M1537N36M                                                                                                                                                                                                                                                                                                          ACCTGCCTAAG GGCCTGAATTTTCTGCTCCTGAATTCAAA|||||||||||||||||||||||||||||||||||||||||||||||||||||||||||||||||||||||||||||||||||||||||||||||||||||||||||||||||||||||||||||||||||||||||||||||||||||||||||||||||||||||||||||||||||||||||||||||||||||||||||||||||||||||||||||||||||||||||||||||||||||||||||||||||||||||||||||
chr12 chr12 57898071 59196643 11M59195042F29M1537N36M                                                                                                                                                                                                                                                                                                          ACCTGCCTAAG GGCCTGAATTTTCTGCTCCTGAATTCAAA|||||||||||||||||||||||||||||||||||||||||||||||||||||||||||||||||||||||||||||||||||||||||||||||||||||||||||||||||||||||||||||||||||||||||||||||||||||||||||||||||||||||||||||||||||||||||||||||||||||||||||||||||||||||||||||||||||||||||||||||||||||||||||||||||||||||||||||
chr12 chr12 57898071 59196643 11M59195042F29M1537N36M                                                                                                                                                                                                                                                                                                          ACCTGCCTAAG GGCCTGAATTTTCTGCTCCTGAATTCAAA|||||||||||||||||||||||||||||||||||||||||||||||||||||||||||||||||||||||||||||||||||||||||||||||||||||||||||||||||||||||||||||||||||||||||||||||||||||||||||||||||||||||||||||||||||||||||||||||||||||||||||||||||||||||||||||||||||||||||||||||||||||||||||||||||||||||||||||
chr12 chr12 57898071 59196643 11M59195042F29M1537N36M                                                                                                                                                                                                                                                                                                          ACCTGCCTAAG GGCCTGAATTTTCTGCTCCTGAATTCAAA|||||||||||||||||||||||||||||||||||||||||||||||||||||||||||||||||||||||||||||||||||||||||||||||||||||||||||||||||||||||||||||||||||||||||||||||||||||||||||||||||||||||||||||||||||||||||||||||||||||||||||||||||||||||||||||||||||||||||||||||||||||||||||||||||||||||||||||
chr12 chr12 57898071 59196643 11M59195042F29M1537N36M                                                                                                                                                                                                                                                                                                          ACCTGCCTAAG GGCCTGAATTTTCTGCTCCTGAATTCAAA|||||||||||||||||||||||||||||||||||||||||||||||||||||||||||||||||||||||||||||||||||||||||||||||||||||||||||||||||||||||||||||||||||||||||||||||||||||||||||||||||||||||||||||||||||||||||||||||||||||||||||||||||||||||||||||||||||||||||||||||||||||||||||||||||||||||||||||
chr12 chr12 57898071 59196643 11M59195042F29M1537N36M                                                                                                                                                                                                                                                                                                          ACCTGCCTAAG GGCCTGAATTTTCTGCTCCTGAATTCAAA|||||||||||||||||||||||||||||||||||||||||||||||||||||||||||||||||||||||||||||||||||||||||||||||||||||||||||||||||||||||||||||||||||||||||||||||||||||||||||||||||||||||||||||||||||||||||||||||||||||||||||||||||||||||||||||||||||||||||||||||||||||||||||||||||||||||||||||
chr12 chr12 57898071 59196643 11M59195042F29M1537N36M                                                                                                                                                                                                                                                                                                          ACCTGCCTAAG GGCCTGAATTTTCTGCTCCTGAATTCAAA|||||||||||||||||||||||||||||||||||||||||||||||||||||||||||||||||||||||||||||||||||||||||||||||||||||||||||||||||||||||||||||||||||||||||||||||||||||||||||||||||||||||||||||||||||||||||||||||||||||||||||||||||||||||||||||||||||||||||||||||||||||||||||||||||||||||||||||
chr12 chr12 57898071 59196643 11M59195042F29M1537N36M                                                                                                                                                                                                                                                                                                          ACCTGCCTAAG GGCCTGAATTTTCTGCTCCTGAATTCAAA|||||||||||||||||||||||||||||||||||||||||||||||||||||||||||||||||||||||||||||||||||||||||||||||||||||||||||||||||||||||||||||||||||||||||||||||||||||||||||||||||||||||||||||||||||||||||||||||||||||||||||||||||||||||||||||||||||||||||||||||||||||||||||||||||||||||||||||
chr12 chr12 57898071 59196643 11M59195042F29M1537N36M                                                                                                                                                                                                                                                                                                          ACCTGCCTAAG GGCCTGAATTTTCTGCTCCTGAATTCAAA|||||||||||||||||||||||||||||||||||||||||||||||||||||||||||||||||||||||||||||||||||||||||||||||||||||||||||||||||||||||||||||||||||||||||||||||||||||||||||||||||||||||||||||||||||||||||||||||||||||||||||||||||||||||||||||||||||||||||||||||||||||||||||||||||||||||||||||
chr12 chr12 57898071 59196643 11M59195042F29M1537N36M                                                                                                                                                                                                                                                                                                          ACCTGCCTAAG GGCCTGAATTTTCTGCTCCTGAATTCAAA|||||||||||||||||||||||||||||||||||||||||||||||||||||||||||||||||||||||||||||||||||||||||||||||||||||||||||||||||||||||||||||||||||||||||||||||||||||||||||||||||||||||||||||||||||||||||||||||||||||||||||||||||||||||||||||||||||||||||||||||||||||||||||||||||||||||||||||
chr12 chr12 57898071 59196643 11M59195042F29M1537N36M                                                                                                                                                                                                                                                                                                          ACCTGCCTAAG GGCCTGAATTTTCTGCTCCTGAATTCAAA|||||||||||||||||||||||||||||||||||||||||||||||||||||||||||||||||||||||||||||||||||||||||||||||||||||||||||||||||||||||||||||||||||||||||||||||||||||||||||||||||||||||||||||||||||||||||||||||||||||||||||||||||||||||||||||||||||||||||||||||||||||||||||||||||||||||||||||
chr12 chr12 57898071 59196643 11M59195042F29M1537N36M                                                                                                                                                                                                                                                                                                          ACCTGCCTAAG GGCCTGAATTTTCTGCTCCTGAATTCAAA|||||||||||||||||||||||||||||||||||||||||||||||||||||||||||||||||||||||||||||||||||||||||||||||||||||||||||||||||||||||||||||||||||||||||||||||||||||||||||||||||||||||||||||||||||||||||||||||||||||||||||||||||||||||||||||||||||||||||||||||||||||||||||||||||||||||||||||
chr12 chr12 57898072 59196644 10M59195042F29M1537N37M                                                                                                                                                                                                                                                                                                           CCTGCCTAAG GGCCTGAATTTTCTGCTCCTGAATTCAAA|||||||||||||||||||||||||||||||||||||||||||||||||||||||||||||||||||||||||||||||||||||||||||||||||||||||||||||||||||||||||||||||||||||||||||||||||||||||||||||||||||||||||||||||||||||||||||||||||||||||||||||||||||||||||||||||||||||||||||||||||||||||||||||||||||||||||||||
chr12 chr12 57898072 59196644 10M59195042F29M1537N37M                                                                                                                                                                                                                                                                                                           CCTGCCTAAG GGCCTGAATTTTCTGCTCCTGAATTCAAA|||||||||||||||||||||||||||||||||||||||||||||||||||||||||||||||||||||||||||||||||||||||||||||||||||||||||||||||||||||||||||||||||||||||||||||||||||||||||||||||||||||||||||||||||||||||||||||||||||||||||||||||||||||||||||||||||||||||||||||||||||||||||||||||||||||||||||||
chr12 chr12 57898072 59196644 10M59195042F29M1537N37M                                                                                                                                                                                                                                                                                                           CCTGCCTAAG GGCCTGAATTTTCTGCTCCTGAATTCAAA|||||||||||||||||||||||||||||||||||||||||||||||||||||||||||||||||||||||||||||||||||||||||||||||||||||||||||||||||||||||||||||||||||||||||||||||||||||||||||||||||||||||||||||||||||||||||||||||||||||||||||||||||||||||||||||||||||||||||||||||||||||||||||||||||||||||||||||
chr12 chr12 57898072 59196644 10M59195042F29M1537N37M                                                                                                                                                                                                                                                                                                           CCTGCCTAAG GGCCTGAATTTTCTGCTCCTGAATTCAAA|||||||||||||||||||||||||||||||||||||||||||||||||||||||||||||||||||||||||||||||||||||||||||||||||||||||||||||||||||||||||||||||||||||||||||||||||||||||||||||||||||||||||||||||||||||||||||||||||||||||||||||||||||||||||||||||||||||||||||||||||||||||||||||||||||||||||||||
chr12 chr12 57898072 59196644 10M59195042F29M1537N37M                                                                                                                                                                                                                                                                                                           CCTGCCTAAT GGCCTGAATTTTCTGCTCCTTAATTCAAA|||||||||||||||||||||||||||||||||||||||||||||||||||||||||||||||||||||||||||||||||||||||||||||||||||||||||||||||||||||||||||||||||||||||||||||||||||||||||||||||||||||||||||||||||||||||||||||||||||||||||||||||||||||||||||||||||||||||||||||||||||||||||||||||||||||||||||||
chr12 chr12 57898072 59196644 10M59195042F29M1537N37M                                                                                                                                                                                                                                                                                                           CCTGCCTAAG GGCCTGAATTTTCTGCTCCTGAATTCAAA|||||||||||||||||||||||||||||||||||||||||||||||||||||||||||||||||||||||||||||||||||||||||||||||||||||||||||||||||||||||||||||||||||||||||||||||||||||||||||||||||||||||||||||||||||||||||||||||||||||||||||||||||||||||||||||||||||||||||||||||||||||||||||||||||||||||||||||
chr12 chr12 57898072 59199759 10M59195042F29M4652N37M                                                                                                                                                                                                                                                                                                           CCTGCCTAAG GGCCTGAATTTTCTGCTCCTGAATTCAAA|||||||||||||||||||||||||||||||||||||||||||||||||||||||||||||||||||||||||||||||||||||||||||||||||||||||||||||||||||||||||||||||||||||||||||||||||||||||||||||||||||||||||||||||||||||||||||||||||||||||||||||||||||||||||||||||||||||||||||||||||||||||||||||||||||||||||||||
chr12 chr12 57898072 59199759 10M59195042F29M4652N37M                                                                                                                                                                                                                                                                                                           CCTGCCTAAG GGCCTGAATTTTCTGCTCCTGAATTCAAA|||||||||||||||||||||||||||||||||||||||||||||||||||||||||||||||||||||||||||||||||||||||||||||||||||||||||||||||||||||||||||||||||||||||||||||||||||||||||||||||||||||||||||||||||||||||||||||||||||||||||||||||||||||||||||||||||||||||||||||||||||||||||||||||||||||||||||||
chr12 chr12 57898072 59199759 10M59195042F29M4652N37M                                                                                                                                                                                                                                                                                                           CCTGCCTAAG GGCCTGAATTTTCTGCTCCTGAATTCAAA|||||||||||||||||||||||||||||||||||||||||||||||||||||||||||||||||||||||||||||||||||||||||||||||||||||||||||||||||||||||||||||||||||||||||||||||||||||||||||||||||||||||||||||||||||||||||||||||||||||||||||||||||||||||||||||||||||||||||||||||||||||||||||||||||||||||||||||
chr12 chr12 57898072 59196644 10M59195042F29M1537N37M                                                                                                                                                                                                                                                                                                           CCTGGCTAAG GGCCTGAATTTTCTGCTCCTGAATTCAAA|||||||||||||||||||||||||||||||||||||||||||||||||||||||||||||||||||||||||||||||||||||||||||||||||||||||||||||||||||||||||||||||||||||||||||||||||||||||||||||||||||||||||||||||||||||||||||||||||||||||||||||||||||||||||||||||||||||||||||||||||||||||||||||||||||||||||||||
chr12 chr12 57898072 59196644 10M59195042F29M1537N37M                                                                                                                                                                                                                                                                                                           CCTGCCTAAG GGCCTGAATTTTCTGCTCCTGAATTCAAA|||||||||||||||||||||||||||||||||||||||||||||||||||||||||||||||||||||||||||||||||||||||||||||||||||||||||||||||||||||||||||||||||||||||||||||||||||||||||||||||||||||||||||||||||||||||||||||||||||||||||||||||||||||||||||||||||||||||||||||||||||||||||||||||||||||||||||||
chr12 chr12 57898072 59196644 10M59195042F29M1537N37M                                                                                                                                                                                                                                                                                                           CCTGCCTAAG GGCCTGAATTTTCTGCTCCTGAATTCAAA|||||||||||||||||||||||||||||||||||||||||||||||||||||||||||||||||||||||||||||||||||||||||||||||||||||||||||||||||||||||||||||||||||||||||||||||||||||||||||||||||||||||||||||||||||||||||||||||||||||||||||||||||||||||||||||||||||||||||||||||||||||||||||||||||||||||||||||
chr12 chr12 57898072 59196644 10M59195042F29M1537N37M                                                                                                                                                                                                                                                                                                           CCTGCCTAAG GGCCTGAATTTTCTGCTCCTGAATTCAAA|||||||||||||||||||||||||||||||||||||||||||||||||||||||||||||||||||||||||||||||||||||||||||||||||||||||||||||||||||||||||||||||||||||||||||||||||||||||||||||||||||||||||||||||||||||||||||||||||||||||||||||||||||||||||||||||||||||||||||||||||||||||||||||||||||||||||||||
chr12 chr12 57898072 59196644 10M59195042F29M1537N37M                                                                                                                                                                                                                                                                                                           CCTGCCTAAG GGCCTGAATTTTCTGCTCCTGAATTCAAA|||||||||||||||||||||||||||||||||||||||||||||||||||||||||||||||||||||||||||||||||||||||||||||||||||||||||||||||||||||||||||||||||||||||||||||||||||||||||||||||||||||||||||||||||||||||||||||||||||||||||||||||||||||||||||||||||||||||||||||||||||||||||||||||||||||||||||||
chr12 chr12 57898072 59196644 10M59195042F29M1537N37M                                                                                                                                                                                                                                                                                                           CCTGCCTAAG GGCCTGAATTTTCTGCTCCTGAATTCAAA|||||||||||||||||||||||||||||||||||||||||||||||||||||||||||||||||||||||||||||||||||||||||||||||||||||||||||||||||||||||||||||||||||||||||||||||||||||||||||||||||||||||||||||||||||||||||||||||||||||||||||||||||||||||||||||||||||||||||||||||||||||||||||||||||||||||||||||
chr12 chr12 57898072 59199759 10M59195042F29M4652N37M                                                                                                                                                                                                                                                                                                           CCTGCCTAAG GGCCTGAATTTTCTGCTCCTGAATTCAAA|||||||||||||||||||||||||||||||||||||||||||||||||||||||||||||||||||||||||||||||||||||||||||||||||||||||||||||||||||||||||||||||||||||||||||||||||||||||||||||||||||||||||||||||||||||||||||||||||||||||||||||||||||||||||||||||||||||||||||||||||||||||||||||||||||||||||||||
chr12 chr12 57898072 59196644 10M59195042F29M1537N37M                                                                                                                                                                                                                                                                                                           CCTGCCTAAG GGCCTGAATTTTCTGCTCCTGAATTCAAA|||||||||||||||||||||||||||||||||||||||||||||||||||||||||||||||||||||||||||||||||||||||||||||||||||||||||||||||||||||||||||||||||||||||||||||||||||||||||||||||||||||||||||||||||||||||||||||||||||||||||||||||||||||||||||||||||||||||||||||||||||||||||||||||||||||||||||||
chr12 chr12 57898072 59196644 10M59195042F29M1537N37M                                                                                                                                                                                                                                                                                                           CCTGCCTAAG GGCCTGAATTTTCTGCTCCTGAATTCAAA|||||||||||||||||||||||||||||||||||||||||||||||||||||||||||||||||||||||||||||||||||||||||||||||||||||||||||||||||||||||||||||||||||||||||||||||||||||||||||||||||||||||||||||||||||||||||||||||||||||||||||||||||||||||||||||||||||||||||||||||||||||||||||||||||||||||||||||
chr12 chr12 57898072 59196644 10M59195042F29M1537N37M                                                                                                                                                                                                                                                                                                           CCTGCCTAAG GGCCTGAATTTTCTGCTCCTGAATTCAAA|||||||||||||||||||||||||||||||||||||||||||||||||||||||||||||||||||||||||||||||||||||||||||||||||||||||||||||||||||||||||||||||||||||||||||||||||||||||||||||||||||||||||||||||||||||||||||||||||||||||||||||||||||||||||||||||||||||||||||||||||||||||||||||||||||||||||||||
chr12 chr12 57898072 59196644 10M59195042F29M1537N37M                                                                                                                                                                                                                                                                                                           CCTGCCTAAG GGCCTGAATTTTCTGCTCCTGAATTCAAA|||||||||||||||||||||||||||||||||||||||||||||||||||||||||||||||||||||||||||||||||||||||||||||||||||||||||||||||||||||||||||||||||||||||||||||||||||||||||||||||||||||||||||||||||||||||||||||||||||||||||||||||||||||||||||||||||||||||||||||||||||||||||||||||||||||||||||||
chr12 chr12 57898072 59196644 10M59195042F29M1537N37M                                                                                                                                                                                                                                                                                                           CCTGCCTAAG GGCCTGAATTTTCTGCTCCTGAATTCAAA|||||||||||||||||||||||||||||||||||||||||||||||||||||||||||||||||||||||||||||||||||||||||||||||||||||||||||||||||||||||||||||||||||||||||||||||||||||||||||||||||||||||||||||||||||||||||||||||||||||||||||||||||||||||||||||||||||||||||||||||||||||||||||||||||||||||||||||
chr12 chr12 57898072 59196644 10M59195042F29M1537N37M                                                                                                                                                                                                                                                                                                           CCTGCCTAAG GGCCTGAATTTTCTGCTCCTGAATTCAAA|||||||||||||||||||||||||||||||||||||||||||||||||||||||||||||||||||||||||||||||||||||||||||||||||||||||||||||||||||||||||||||||||||||||||||||||||||||||||||||||||||||||||||||||||||||||||||||||||||||||||||||||||||||||||||||||||||||||||||||||||||||||||||||||||||||||||||||
chr12 chr12 57898072 59196644 10M59195042F29M1537N37M                                                                                                                                                                                                                                                                                                           CCTGCCTAAG GGCCTGAATTTTCTGCTCCTGAATTCAAA|||||||||||||||||||||||||||||||||||||||||||||||||||||||||||||||||||||||||||||||||||||||||||||||||||||||||||||||||||||||||||||||||||||||||||||||||||||||||||||||||||||||||||||||||||||||||||||||||||||||||||||||||||||||||||||||||||||||||||||||||||||||||||||||||||||||||||||
chr12 chr12 57898072 59196644 10M59195042F29M1537N37M                                                                                                                                                                                                                                                                                                           CCTGCCTAAG GGCCTGAATTTTCTGCTCCTGAATTCAAA|||||||||||||||||||||||||||||||||||||||||||||||||||||||||||||||||||||||||||||||||||||||||||||||||||||||||||||||||||||||||||||||||||||||||||||||||||||||||||||||||||||||||||||||||||||||||||||||||||||||||||||||||||||||||||||||||||||||||||||||||||||||||||||||||||||||||||||
chr12 chr12 57898073 59199760 9M59195042F29M4652N38M                                                                                                                                                                                                                                                                                                             CTGCCTAAG GGCCTGAATTTTCTGCTCCTGAATTCAAA|||||||||||||||||||||||||||||||||||||||||||||||||||||||||||||||||||||||||||||||||||||||||||||||||||||||||||||||||||||||||||||||||||||||||||||||||||||||||||||||||||||||||||||||||||||||||||||||||||||||||||||||||||||||||||||||||||||||||||||||||||||||||||||||||||||||||||||
chr12 chr12 57898073 59199760 9M59195042F29M4652N38M                                                                                                                                                                                                                                                                                                             CTGCCTAAG GGCCTGAATTTTCTGCTCCTGAATTCAAA|||||||||||||||||||||||||||||||||||||||||||||||||||||||||||||||||||||||||||||||||||||||||||||||||||||||||||||||||||||||||||||||||||||||||||||||||||||||||||||||||||||||||||||||||||||||||||||||||||||||||||||||||||||||||||||||||||||||||||||||||||||||||||||||||||||||||||||
chr12 chr12 57898073 59199760 9M59195042F29M4652N38M                                                                                                                                                                                                                                                                                                             CTGCCTAAG GGCCTGAATTTTCTGCTCCTGAATTCAAA|||||||||||||||||||||||||||||||||||||||||||||||||||||||||||||||||||||||||||||||||||||||||||||||||||||||||||||||||||||||||||||||||||||||||||||||||||||||||||||||||||||||||||||||||||||||||||||||||||||||||||||||||||||||||||||||||||||||||||||||||||||||||||||||||||||||||||||
chr12 chr12 57898073 59199760 9M59195042F29M4652N38M                                                                                                                                                                                                                                                                                                             CTGCCTAAG GGCCTGAATTTTCTGCTCCTGAATTCAAA|||||||||||||||||||||||||||||||||||||||||||||||||||||||||||||||||||||||||||||||||||||||||||||||||||||||||||||||||||||||||||||||||||||||||||||||||||||||||||||||||||||||||||||||||||||||||||||||||||||||||||||||||||||||||||||||||||||||||||||||||||||||||||||||||||||||||||||
chr12 chr12 57898073 59199760 9M59195042F29M4652N38M                                                                                                                                                                                                                                                                                                             CTGCCTAAG GGCCTGAATTTTCTGCTCCTGAATTCAAA|||||||||||||||||||||||||||||||||||||||||||||||||||||||||||||||||||||||||||||||||||||||||||||||||||||||||||||||||||||||||||||||||||||||||||||||||||||||||||||||||||||||||||||||||||||||||||||||||||||||||||||||||||||||||||||||||||||||||||||||||||||||||||||||||||||||||||||
chr12 chr12 57898073 59199760 9M59195042F29M4652N38M                                                                                                                                                                                                                                                                                                             CTGCCTAAG GGCCTGAATTTTCTGCTCCTGAATTCAAA|||||||||||||||||||||||||||||||||||||||||||||||||||||||||||||||||||||||||||||||||||||||||||||||||||||||||||||||||||||||||||||||||||||||||||||||||||||||||||||||||||||||||||||||||||||||||||||||||||||||||||||||||||||||||||||||||||||||||||||||||||||||||||||||||||||||||||||
chr12 chr12 57898074 59199761 8M59195042F29M4652N39M                                                                                                                                                                                                                                                                                                              TGCCTAAG GGCCTGAATTTTCTGCTCCTGAATTCAAA|||||||||||||||||||||||||||||||||||||||||||||||||||||||||||||||||||||||||||||||||||||||||||||||||||||||||||||||||||||||||||||||||||||||||||||||||||||||||||||||||||||||||||||||||||||||||||||||||||||||||||||||||||||||||||||||||||||||||||||||||||||||||||||||||||||||||||||
chr12 chr12 57898074 59196646 8M59195042F29M1537N39M                                                                                                                                                                                                                                                                                                              TGCCTAAG GGCCTGAATTTTCTGCTCCTGAATTCAAA|||||||||||||||||||||||||||||||||||||||||||||||||||||||||||||||||||||||||||||||||||||||||||||||||||||||||||||||||||||||||||||||||||||||||||||||||||||||||||||||||||||||||||||||||||||||||||||||||||||||||||||||||||||||||||||||||||||||||||||||||||||||||||||||||||||||||||||
chr12 chr12 57898074 59206234 8M59195042F29M11125N39M                                                                                                                                                                                                                                                                                                             TGCCTAAG GGCCTGAATTTTCTGCTCCTGAATTCAAA|||||||||||||||||||||||||||||||||||||||||||||||||||||||||||||||||||||||||||||||||||||||||||||||||||||||||||||||||||||||||||||||||||||||||||||||||||||||||||||||||||||||||||||||||||||||||||||||||||||||||||||||||||||||||||||||||||||||||||||||||||||||||||||||||||||||||||||
chr12 chr12 57898074 59196646 8M59195042F29M1537N39M                                                                                                                                                                                                                                                                                                              TGCCTAAG GGCCTGAATTTTCTGCTCCTGAATTCAAA|||||||||||||||||||||||||||||||||||||||||||||||||||||||||||||||||||||||||||||||||||||||||||||||||||||||||||||||||||||||||||||||||||||||||||||||||||||||||||||||||||||||||||||||||||||||||||||||||||||||||||||||||||||||||||||||||||||||||||||||||||||||||||||||||||||||||||||
chr12 chr12 57898074 59199761 8M59195042F29M4652N39M                                                                                                                                                                                                                                                                                                              TGCCTAAG GGCCTGAATTTTCTGCTCCTGAATTCAAA|||||||||||||||||||||||||||||||||||||||||||||||||||||||||||||||||||||||||||||||||||||||||||||||||||||||||||||||||||||||||||||||||||||||||||||||||||||||||||||||||||||||||||||||||||||||||||||||||||||||||||||||||||||||||||||||||||||||||||||||||||||||||||||||||||||||||||||
chr12 chr12 57898074 59199761 8M59195042F29M4652N39M                                                                                                                                                                                                                                                                                                              TGCCTAAG GGCCTGAATTTTCTGCTCCTGAATTCAAA|||||||||||||||||||||||||||||||||||||||||||||||||||||||||||||||||||||||||||||||||||||||||||||||||||||||||||||||||||||||||||||||||||||||||||||||||||||||||||||||||||||||||||||||||||||||||||||||||||||||||||||||||||||||||||||||||||||||||||||||||||||||||||||||||||||||||||||
chr12 chr12 57898074 59196646 8M59195042F29M1537N39M                                                                                                                                                                                                                                                                                                              TGCCTAAG GGCCTGAATTTTCTGCTCCTGAATTCAAA|||||||||||||||||||||||||||||||||||||||||||||||||||||||||||||||||||||||||||||||||||||||||||||||||||||||||||||||||||||||||||||||||||||||||||||||||||||||||||||||||||||||||||||||||||||||||||||||||||||||||||||||||||||||||||||||||||||||||||||||||||||||||||||||||||||||||||||
chr12 chr12 57898074 59196646 8M59195042F29M1537N39M                                                                                                                                                                                                                                                                                                              TGCCTAAG GGCCTGAATTTTCTGCTCCTGAATTCAAA|||||||||||||||||||||||||||||||||||||||||||||||||||||||||||||||||||||||||||||||||||||||||||||||||||||||||||||||||||||||||||||||||||||||||||||||||||||||||||||||||||||||||||||||||||||||||||||||||||||||||||||||||||||||||||||||||||||||||||||||||||||||||||||||||||||||||||||
chr12 chr12 57898074 59199761 8M59195042F29M4652N39M                                                                                                                                                                                                                                                                                                              TGCCTAAG GGCCTGAATTTTCTGCTCCTGAATTCAAA|||||||||||||||||||||||||||||||||||||||||||||||||||||||||||||||||||||||||||||||||||||||||||||||||||||||||||||||||||||||||||||||||||||||||||||||||||||||||||||||||||||||||||||||||||||||||||||||||||||||||||||||||||||||||||||||||||||||||||||||||||||||||||||||||||||||||||||
chr12 chr12 57898074 59199761 8M59195042F29M4652N39M                                                                                                                                                                                                                                                                                                              TGCCTAAG GGCCTGAATTTTCTGCTCCTGAATTCAAA|||||||||||||||||||||||||||||||||||||||||||||||||||||||||||||||||||||||||||||||||||||||||||||||||||||||||||||||||||||||||||||||||||||||||||||||||||||||||||||||||||||||||||||||||||||||||||||||||||||||||||||||||||||||||||||||||||||||||||||||||||||||||||||||||||||||||||||
chr12 chr12 57898074 59206234 8M59195042F29M11125N39M                                                                                                                                                                                                                                                                                                             TGCCTAAG GGCCTGAATTTTCTGCTCCTGAATTCAAA|||||||||||||||||||||||||||||||||||||||||||||||||||||||||||||||||||||||||||||||||||||||||||||||||||||||||||||||||||||||||||||||||||||||||||||||||||||||||||||||||||||||||||||||||||||||||||||||||||||||||||||||||||||||||||||||||||||||||||||||||||||||||||||||||||||||||||||
chr12 chr12 57898075 59196647 7M59195042F29M1537N40M                                                                                                                                                                                                                                                                                                               GCCTAAG GGCCTGAATTTTCTGCTCCTGAATTCAAA|||||||||||||||||||||||||||||||||||||||||||||||||||||||||||||||||||||||||||||||||||||||||||||||||||||||||||||||||||||||||||||||||||||||||||||||||||||||||||||||||||||||||||||||||||||||||||||||||||||||||||||||||||||||||||||||||||||||||||||||||||||||||||||||||||||||||||||
chr12 chr12 57898077 59199764 5M59195042F29M4652N42M                                                                                                                                                                                                                                                                                                                 CTAAG GGCCTGAATTTTCTGCTCCTGAATTCAAA|||||||||||||||||||||||||||||||||||||||||||||||||||||||||||||||||||||||||||||||||||||||||||||||||||||||||||||||||||||||||||||||||||||||||||||||||||||||||||||||||||||||||||||||||||||||||||||||||||||||||||||||||||||||||||||||||||||||||||||||||||||||||||||||||||||||||||||
chr12 chr12 57898077 59196649 5M59195042F29M1537N42M                                                                                                                                                                                                                                                                                                                 CTAAG GGCCTGAATTTTCTGCTCCTGAATTCAAA|||||||||||||||||||||||||||||||||||||||||||||||||||||||||||||||||||||||||||||||||||||||||||||||||||||||||||||||||||||||||||||||||||||||||||||||||||||||||||||||||||||||||||||||||||||||||||||||||||||||||||||||||||||||||||||||||||||||||||||||||||||||||||||||||||||||||||||
chr12 chr12 57898077 59196649 5M59195042F29M1537N42M                                                                                                                                                                                                                                                                                                                 CTAAG GGCCTGAATTTTCTGCTCCTGAATTCAAA|||||||||||||||||||||||||||||||||||||||||||||||||||||||||||||||||||||||||||||||||||||||||||||||||||||||||||||||||||||||||||||||||||||||||||||||||||||||||||||||||||||||||||||||||||||||||||||||||||||||||||||||||||||||||||||||||||||||||||||||||||||||||||||||||||||||||||||
chr12 chr12 57898078 59196650 4M59195042F29M1537N43M                                                                                                                                                                                                                                                                                                                  TAAG GGCCTGAATTTTCTGCTCCTGAATTCAAA|||||||||||||||||||||||||||||||||||||||||||||||||||||||||||||||||||||||||||||||||||||||||||||||||||||||||||||||||||||||||||||||||||||||||||||||||||||||||||||||||||||||||||||||||||||||||||||||||||||||||||||||||||||||||||||||||||||||||||||||||||||||||||||||||||||||||||||
chr12 chr12 57898078 59196650 4M59195042F29M1537N43M                                                                                                                                                                                                                                                                                                                  TAAG GGCCTGAATTTTCTGCTCCTGAATTCAAA|||||||||||||||||||||||||||||||||||||||||||||||||||||||||||||||||||||||||||||||||||||||||||||||||||||||||||||||||||||||||||||||||||||||||||||||||||||||||||||||||||||||||||||||||||||||||||||||||||||||||||||||||||||||||||||||||||||||||||||||||||||||||||||||||||||||||||||
chr12 chr12 57898079 59206197 3M59195042F29M4652N42M6431N2M                                                                                                                                                                                                                                                                                                            AAG GGCCTGAATTTTCTCCTCCTGAATTCAAA|||||||||||||||||||||||||||||||||||||||||||||||||||||||||||||||||||||||||||||||||||||||||||||||||||||||||||||||||||||||||||||||||||||||||||||||||||||||||||||||||||||||||||||||||||||||||||||||||||||||||||||||||||||||||||||||||||||||||||||||||||||||||||||||||||||||||||||
chr12 chr12 57898079 59206197 3M59195042F29M4652N42M6431N2M                                                                                                                                                                                                                                                                                                            AAG GGCCTGAATTTTCTCCTCCTGAATTCAAA|||||||||||||||||||||||||||||||||||||||||||||||||||||||||||||||||||||||||||||||||||||||||||||||||||||||||||||||||||||||||||||||||||||||||||||||||||||||||||||||||||||||||||||||||||||||||||||||||||||||||||||||||||||||||||||||||||||||||||||||||||||||||||||||||||||||||||||
chr12 chr12 57898079 59206197 3M59195042F29M4652N42M6431N2M                                                                                                                                                                                                                                                                                                            AAG GGCCTGAATTTTCTCCTCCTGAATTCAAA|||||||||||||||||||||||||||||||||||||||||||||||||||||||||||||||||||||||||||||||||||||||||||||||||||||||||||||||||||||||||||||||||||||||||||||||||||||||||||||||||||||||||||||||||||||||||||||||||||||||||||||||||||||||||||||||||||||||||||||||||||||||||||||||||||||||||||||
chr12 chr12 59195034 59196647 36M1537N40M                                                                                                                                                                                                                                                                                                                           GCCTAAGGGCCTGAATTTTCTGCTCCTGAATTCAAA|||||||||||||||||||||||||||||||||||||||||||||||||||||||||||||||||||||||||||||||||||||||||||||||||||||||||||||||||||||||||||||||||||||||||||||||||||||||||||||||||||||||||||||||||||||||||||||||||||||||||||||||||||||||||||||||||||||||||||||||||||||||||||||||||||||||||||||
chr12 chr12 59195037 59196650 33M1537N43M                                                                                                                                                                                                                                                                                                                              TAAGGGCCTGAATTTTCTGCTCCTGAATTCAAA|||||||||||||||||||||||||||||||||||||||||||||||||||||||||||||||||||||||||||||||||||||||||||||||||||||||||||||||||||||||||||||||||||||||||||||||||||||||||||||||||||||||||||||||||||||||||||||||||||||||||||||||||||||||||||||||||||||||||||||||||||||||||||||||||||||||||||||
chr12 chr12 59195040 59196653 30M1537N46M                                                                                                                                                                                                                                                                                                                                 GGGCCTGAATTTTCTGCTCCTGAATTCAAA|||||||||||||||||||||||||||||||||||||||||||||||||||||||||||||||||||||||||||||||||||||||||||||||||||||||||||||||||||||||||||||||||||||||||||||||||||||||||||||||||||||||||||||||||||||||||||||||||||||||||||||||||||||||||||||||||||||||||||||||||||||||||||||||||||||||||||||
chr12 chr12 59195043 59199739 27M1642N32M2978N17M                                                                                                                                                                                                                                                                                                                            CCTGAATTTTCTGCTCCTGAATTCAAA|||||||||||||||||||||||||||||||||||||||||||||||||||||||||||||||||||||||||||||||||||||||||||||||||||||||||||||||||||||||||||||||||||||||||||||||||||||||||||||||||||||||||||||||||||||||||||||||||||||||||||||||||||||||||||||||||||||||||||||||||||||||||||||||||||||||||||||
chr12 chr12 59195046 59196659 24M1537N52M                                                                                                                                                                                                                                                                                                                                       GAATTTTCTGCTCCTGAATTCAAA|||||||||||||||||||||||||||||||||||||||||||||||||||||||||||||||||||||||||||||||||||||||||||||||||||||||||||||||||||||||||||||||||||||||||||||||||||||||||||||||||||||||||||||||||||||||||||||||||||||||||||||||||||||||||||||||||||||||||||||||||||||||||||||||||||||||||||||
chr12 chr12 59195049 59206208 21M4652N42M6431N13M                                                                                                                                                                                                                                                                                                                                  TTTTCTGCTCCTGAATTCAAA|||||||||||||||||||||||||||||||||||||||||||||||||||||||||||||||||||||||||||||||||||||||||||||||||||||||||||||||||||||||||||||||||||||||||||||||||||||||||||||||||||||||||||||||||||||||||||||||||||||||||||||||||||||||||||||||||||||||||||||||||||||||||||||||||||||||||||||
chr12 chr12 59195052 59206211 18M4652N42M6431N16M                                                                                                                                                                                                                                                                                                                                     TCTGCTCCTGAATTCAAA|||||||||||||||||||||||||||||||||||||||||||||||||||||||||||||||||||||||||||||||||||||||||||||||||||||||||||||||||||||||||||||||||||||||||||||||||||||||||||||||||||||||||||||||||||||||||||||||||||||||||||||||||||||||||||||||||||||||||||||||||||||||||||||||||||||||||||||
chr12 chr12 59195055 59199783 15M4652N61M                                                                                                                                                                                                                                                                                                                                                GCTCCTGAATTCAAA|||||||||||||||||||||||||||||||||||||||||||||||||||||||||||||||||||||||||||||||||||||||||||||||||||||||||||||||||||||||||||||||||||||||||||||||||||||||||||||||||||||||||||||||||||||||||||||||||||||||||||||||||||||||||||||||||||||||||||||||||||||||||||||||||||||||||||||
chr12 chr12 59195058 59196671 12M1537N64M                                                                                                                                                                                                                                                                                                                                                   CCTGAATTCAAA|||||||||||||||||||||||||||||||||||||||||||||||||||||||||||||||||||||||||||||||||||||||||||||||||||||||||||||||||||||||||||||||||||||||||||||||||||||||||||||||||||||||||||||||||||||||||||||||||||||||||||||||||||||||||||||||||||||||||||||||||||||||||||||||||||||||||||||
chr12 chr12 59195061 59196674 9M1537N67M                                                                                                                                                                                                                                                                                                                                                       GAATTCAAA|||||||||||||||||||||||||||||||||||||||||||||||||||||||||||||||||||||||||||||||||||||||||||||||||||||||||||||||||||||||||||||||||||||||||||||||||||||||||||||||||||||||||||||||||||||||||||||||||||||||||||||||||||||||||||||||||||||||||||||||||||||||||||||||||||||||||||||
chr12 chr12 59195064 59196677 6M1537N70M                                                                                                                                                                                                                                                                                                                                                          TTCAAA|||||||||||||||||||||||||||||||||||||||||||||||||||||||||||||||||||||||||||||||||||||||||||||||||||||||||||||||||||||||||||||||||||||||||||||||||||||||||||||||||||||||||||||||||||||||||||||||||||||||||||||||||||||||||||||||||||||||||||||||||||||||||||||||||||||||||||||
chr12 chr12 59195067 59196680 3M1537N73M                                                                                                                                                                                                                                                                                                                                                             AAA|||||||||||||||||||||||||||||||||||||||||||||||||||||||||||||||||||||||||||||||||||||||||||||||||||||||||||||||||||||||||||||||||||||||||||||||||||||||||||||||||||||||||||||||||||||||||||||||||||||||||||||||||||||||||||||||||||||||||||||||||||||||||||||||||||||||||||||
chr12 chr12 59195074 59195150 76M                                                                                                                                                                                                                                                                                                                                                                           GTTTCCATAACCCTGATCTGCATAGTCAGGCATCTGGGACCTGAACATGGTTGGGACAATTTGCTGTCCATCATTA
chr12 chr12 59195084 59195160 76M                                                                                                                                                                                                                                                                                                                                                                                     CCCTGATCTGCATAGTCAGGCATCTGGGACCTGAACATGGTTGGGACAATTTGCTGTCCATCATTATCTTTCTCTT
chr12 chr12 59195108 59195184 76M                                                                                                                                                                                                                                                                                                                                                                                                             TGGGACCTGAACATGGTTGGGACAATTTGCTGTCAATCCTTATCTTTCTCTTTCAATTCTCCTTGAGCTGCTGTAC
chr12 chr12 59195116 59195192 76M                                                                                                                                                                                                                                                                                                                                                                                                                     GAACATGGTTGGGACAATTTGCTGTCCATCATTATCTTTCTCTTTCAATTCTCCTTGAGCTGCTGTACCATTTTCA
chr12 chr12 59195134 59195210 76M                                                                                                                                                                                                                                                                                                                                                                                                                                       TTGCTGTCCATCATTATCTTTCTCTTTCAATTCTCCTTGAGCTGCTGTACCATTTTCAGTCCTAACTCCATATTTG
chr12 chr12 59195155 59195231 76M                                                                                                                                                                                                                                                                                                                                                                                                                                                            CTCTTTCAATTCTCCTTGAGCTGCTGTACCATTTTCAGTCCTAACTCCATATTTGTGTTCTCCCCAACACCCCCCA
chr12 chr12 59195159 59195235 76M                                                                                                                                                                                                                                                                                                                                                                                                                                                                TTCAATTCTCCTTGAGCTGCTGTACCATTTTCAGTCCTAACTCCATATTTGTGTTCTCCCCAACACCCCCCACCCC
chr12 chr12 59195175 59195251 76M                                                                                                                                                                                                                                                                                                                                                                                                                                                                                CTGCTGTACCATTTTCAGTCCTAACTCCATATTTGTGTTCTCCCCAACACCCCCCACCCCCGCCACTTTGGAAAAG
chr12 chr12 59195182 59195258 76M                                                                                                                                                                                                                                                                                                                                                                                                                                                                                       ACCATTTTCAGTCCTAACTCCATATTTGTGTTCTCCCCAACACCCCCCACCCCCACCACTTTGGAAAAGCCAAATT
chr12 chr12 59195187 59195263 76M                                                                                                                                                                                                                                                                                                                                                                                                                                                                                            TTTCAGTCCTAACTCCATATTTGTGTTCTCCCCAACACCCCCCACCCCCACCACTTTGGAAAAGCCAAATTTGAAT
chr12 chr12 59195207 59195283 76M                                                                                                                                                                                                                                                                                                                                                                                                                                                                                                                TTGTGTTCTCCCCAACACCCCCCACCCCCACCACTTTGGAAAAGCCAAATTTGAATCTTTGTTCAAATTCCATACT
chr12 chr12 59195240 59195316 76M                                                                                                                                                                                                                                                                                                                                                                                                                                                                                                                                                 CTTTGGAAAAGCCAAATTTGAATCTTTGTTCAATTTCCATACTTAGCCTGCTGCCTTTTAGATATCATTCTTCTTG
chr12 chr12 59195252 59195328 76M                                                                                                                                                                                                                                                                                                                                                                                                                                                                                                                                                             CAAATTTGAATCTTTGTTCAAATTCCATACTTAGCCTGCTGCCTTTTAGATATCATTCTTCTTGGCCTTATTTCCT
chr12 chr12 59195270 59195346 76M                                                                                                                                                                                                                                                                                                                                                                                                                                                                                                                                                                               CAAATTCCATACTTAGCCTGCTGCCTTTTAGATATCATTCTTCTTGGCCTTATTTCCTACCTCAAGTTT
chr12 chr12 59195280 59195356 76M                                                                                                                                                                                                                                                                                                                                                                                                                                                                                                                                                                                         ACTTAGCCTGCTGCCTTTTAGATATCATTCTTCTTGGCCTTATTTCCTACCTCAAGTTT
chr12 chr12 59195283 59195359 76M                                                                                                                                                                                                                                                                                                                                                                                                                                                                                                                                                                                            TAGCCTGCTGCCTTTTAGATATCATTCTTCTTGGCCTTATTTCCTACCTCAAGTTT
chr12 chr12 59195295 59195371 76M                                                                                                                                                                                                                                                                                                                                                                                                                                                                                                                                                                                                        TTTTAGATATCATTCTTCTTGGCCTTATTTCCTACCTCAAGTTT
chr12 chr12 59195311 59195387 76M                                                                                                                                                                                                                                                                                                                                                                                                                                                                                                                                                                                                                        TCTTGGCCTTATTTCCTACCTCAAGTTT
chr12 chr12 59195335 59195411 76M                                                                                                                                                                                                                                                                                                                                                                                                                                                                                                                                                                                                                                                GTTT 
    28 pairs 
 2:1
2:2
1:3
-2:2
-4:2
2:12
1:17
16:3
-1:-413
-5:-626
2:-631
2:-654
-5:-656
-3:-670
-3:-670
-3:-670
-1:1566
0:1569
-4:1569
1:1628
0:1701
-1811:3
0:4685
1:4697
2:4712
2:4712
0:4717
0:4717 
     TCGA-02-2485-01A chr12-chr12 57898081 59199722 ff 
 
141 54 126 12 56 6487
CATGCCCTGTGGTGCAGTCGAGCCAGCACCTGTTTCTGGACCTGCCTAAG AATAGAATGCAACTGAAGAAATGGCTGGTGCAATTCAGCAAGGTAAAAGA
99999999999999999999999999999999999999999999999999 99999999999999999999999999999999999999999999999999
MARS exon11(57898006-57898081) ENSG00000258231 exon5(59199721-59199840)
 
 blast search - human genome  
 left 
 >ref|NT_029419.12| Homo sapiens chromosome 12 genomic contig, GRCh37.p9 Primary 
Assembly
 gb|GL000107.1| Homo sapiens chromosome 12 genomic contig, GRCh37 reference primary 
assembly
Length=71516776

 Score = 93.5 bits (50),  Expect = 5e-17
 Identities = 50/50 (100%), Gaps = 0/50 (0%)
 Strand=Plus/Plus

Query  1         CATGCCCTGTGGTGCAGTCGAGCCAGCACCTGTTTCTGGACCTGCCTAAG  50
                 ||||||||||||||||||||||||||||||||||||||||||||||||||
Sbjct  20041339  CATGCCCTGTGGTGCAGTCGAGCCAGCACCTGTTTCTGGACCTGCCTAAG  20041388


>ref|NW_001838060.2| Homo sapiens chromosome 12 genomic contig, alternate assembly 
HuRef SCAF_1103279188147, whole genome shotgun sequence
 gb|DS486061.1| Homo sapiens SCAF_1103279188147 genomic scaffold, whole genome 
shotgun sequence
Length=18942049

 Score = 93.5 bits (50),  Expect = 5e-17
 Identities = 50/50 (100%), Gaps = 0/50 (0%)
 Strand=Plus/Minus

Query  1         CATGCCCTGTGGTGCAGTCGAGCCAGCACCTGTTTCTGGACCTGCCTAAG  50
                 ||||||||||||||||||||||||||||||||||||||||||||||||||
Sbjct  18630467  CATGCCCTGTGGTGCAGTCGAGCCAGCACCTGTTTCTGGACCTGCCTAAG  18630418


>ref|NC_000012.11| Homo sapiens chromosome 12, GRCh37.p9 Primary Assembly
 gb|CM000674.1| Homo sapiens chromosome 12, GRCh37 primary reference assembly
Length=133851895

 Score = 93.5 bits (50),  Expect = 5e-17
 Identities = 50/50 (100%), Gaps = 0/50 (0%)
 Strand=Plus/Plus

Query  1         CATGCCCTGTGGTGCAGTCGAGCCAGCACCTGTTTCTGGACCTGCCTAAG  50
                 ||||||||||||||||||||||||||||||||||||||||||||||||||
Sbjct  57898033  CATGCCCTGTGGTGCAGTCGAGCCAGCACCTGTTTCTGGACCTGCCTAAG  57898082


>ref|AC_000144.1| Homo sapiens chromosome 12, alternate assembly HuRef, whole genome 
shotgun sequence
 gb|CM000473.1| Homo sapiens chromosome 12, whole genome shotgun sequence
Length=130617856

 Score = 93.5 bits (50),  Expect = 5e-17
 Identities = 50/50 (100%), Gaps = 0/50 (0%)
 Strand=Plus/Plus

Query  1         CATGCCCTGTGGTGCAGTCGAGCCAGCACCTGTTTCTGGACCTGCCTAAG  50
                 ||||||||||||||||||||||||||||||||||||||||||||||||||
Sbjct  54936110  CATGCCCTGTGGTGCAGTCGAGCCAGCACCTGTTTCTGGACCTGCCTAAG  54936159


>gb|CM000263.1| Homo sapiens chromosome 12, whole genome shotgun sequence
Length=133516800

 Score = 93.5 bits (50),  Expect = 5e-17
 Identities = 50/50 (100%), Gaps = 0/50 (0%)
 Strand=Plus/Plus

Query  1         CATGCCCTGTGGTGCAGTCGAGCCAGCACCTGTTTCTGGACCTGCCTAAG  50
                 ||||||||||||||||||||||||||||||||||||||||||||||||||
Sbjct  57553869  CATGCCCTGTGGTGCAGTCGAGCCAGCACCTGTTTCTGGACCTGCCTAAG  57553918


Lambda     K      H
    1.33    0.621     1.12 

Gapped
Lambda     K      H
    1.28    0.460    0.850 

Effective search space used: 681780354108
 
 right 
 >ref|NT_029419.12| Homo sapiens chromosome 12 genomic contig, GRCh37.p9 Primary 
Assembly
 gb|GL000107.1| Homo sapiens chromosome 12 genomic contig, GRCh37 reference primary 
assembly
Length=71516776

 Score = 93.5 bits (50),  Expect = 5e-17
 Identities = 50/50 (100%), Gaps = 0/50 (0%)
 Strand=Plus/Plus

Query  1         AATAGAATGCAACTGAAGAAATGGCTGGTGCAATTCAGCAAGGTAAAAGA  50
                 ||||||||||||||||||||||||||||||||||||||||||||||||||
Sbjct  21343029  AATAGAATGCAACTGAAGAAATGGCTGGTGCAATTCAGCAAGGTAAAAGA  21343078


>ref|NW_001838060.2| Homo sapiens chromosome 12 genomic contig, alternate assembly 
HuRef SCAF_1103279188147, whole genome shotgun sequence
 gb|DS486061.1| Homo sapiens SCAF_1103279188147 genomic scaffold, whole genome 
shotgun sequence
Length=18942049

 Score = 93.5 bits (50),  Expect = 5e-17
 Identities = 50/50 (100%), Gaps = 0/50 (0%)
 Strand=Plus/Minus

Query  1         AATAGAATGCAACTGAAGAAATGGCTGGTGCAATTCAGCAAGGTAAAAGA  50
                 ||||||||||||||||||||||||||||||||||||||||||||||||||
Sbjct  17318263  AATAGAATGCAACTGAAGAAATGGCTGGTGCAATTCAGCAAGGTAAAAGA  17318214


>ref|NC_000012.11| Homo sapiens chromosome 12, GRCh37.p9 Primary Assembly
 gb|CM000674.1| Homo sapiens chromosome 12, GRCh37 primary reference assembly
Length=133851895

 Score = 93.5 bits (50),  Expect = 5e-17
 Identities = 50/50 (100%), Gaps = 0/50 (0%)
 Strand=Plus/Plus

Query  1         AATAGAATGCAACTGAAGAAATGGCTGGTGCAATTCAGCAAGGTAAAAGA  50
                 ||||||||||||||||||||||||||||||||||||||||||||||||||
Sbjct  59199723  AATAGAATGCAACTGAAGAAATGGCTGGTGCAATTCAGCAAGGTAAAAGA  59199772


>ref|AC_000144.1| Homo sapiens chromosome 12, alternate assembly HuRef, whole genome 
shotgun sequence
 gb|CM000473.1| Homo sapiens chromosome 12, whole genome shotgun sequence
Length=130617856

 Score = 93.5 bits (50),  Expect = 5e-17
 Identities = 50/50 (100%), Gaps = 0/50 (0%)
 Strand=Plus/Plus

Query  1         AATAGAATGCAACTGAAGAAATGGCTGGTGCAATTCAGCAAGGTAAAAGA  50
                 ||||||||||||||||||||||||||||||||||||||||||||||||||
Sbjct  56248314  AATAGAATGCAACTGAAGAAATGGCTGGTGCAATTCAGCAAGGTAAAAGA  56248363


>gb|CM000263.1| Homo sapiens chromosome 12, whole genome shotgun sequence
Length=133516800

 Score = 93.5 bits (50),  Expect = 5e-17
 Identities = 50/50 (100%), Gaps = 0/50 (0%)
 Strand=Plus/Plus

Query  1         AATAGAATGCAACTGAAGAAATGGCTGGTGCAATTCAGCAAGGTAAAAGA  50
                 ||||||||||||||||||||||||||||||||||||||||||||||||||
Sbjct  58861364  AATAGAATGCAACTGAAGAAATGGCTGGTGCAATTCAGCAAGGTAAAAGA  58861413


Lambda     K      H
    1.33    0.621     1.12 

Gapped
Lambda     K      H
    1.28    0.460    0.850 

Effective search space used: 681780354108
 
 blast search - nt  
 left 
 >ref|XM_002823424.2| PREDICTED: Pongo abelii methionyl-tRNA synthetase (MARS), mRNA
Length=2917

 Score = 93.5 bits (50),  Expect = 6e-17
 Identities = 50/50 (100%), Gaps = 0/50 (0%)
 Strand=Plus/Plus

Query  1     CATGCCCTGTGGTGCAGTCGAGCCAGCACCTGTTTCTGGACCTGCCTAAG  50
             ||||||||||||||||||||||||||||||||||||||||||||||||||
Sbjct  1457  CATGCCCTGTGGTGCAGTCGAGCCAGCACCTGTTTCTGGACCTGCCTAAG  1506


>ref|NM_004990.3| Homo sapiens methionyl-tRNA synthetase (MARS), mRNA
Length=2932

 Score = 93.5 bits (50),  Expect = 6e-17
 Identities = 50/50 (100%), Gaps = 0/50 (0%)
 Strand=Plus/Plus

Query  1     CATGCCCTGTGGTGCAGTCGAGCCAGCACCTGTTTCTGGACCTGCCTAAG  50
             ||||||||||||||||||||||||||||||||||||||||||||||||||
Sbjct  1457  CATGCCCTGTGGTGCAGTCGAGCCAGCACCTGTTTCTGGACCTGCCTAAG  1506


>dbj|AB590802.1| Synthetic construct DNA, clone: pFN21AB8542, Homo sapiens MARS 
gene for methionyl-tRNA synthetase, without stop codon, in 
Flexi system
Length=2717

 Score = 93.5 bits (50),  Expect = 6e-17
 Identities = 50/50 (100%), Gaps = 0/50 (0%)
 Strand=Plus/Plus

Query  1     CATGCCCTGTGGTGCAGTCGAGCCAGCACCTGTTTCTGGACCTGCCTAAG  50
             ||||||||||||||||||||||||||||||||||||||||||||||||||
Sbjct  1328  CATGCCCTGTGGTGCAGTCGAGCCAGCACCTGTTTCTGGACCTGCCTAAG  1377


>dbj|AK303349.1| Homo sapiens cDNA FLJ55046 complete cds, highly similar to Methionyl-tRNA 
synthetase (EC 6.1.1.10)
Length=1328

 Score = 93.5 bits (50),  Expect = 6e-17
 Identities = 50/50 (100%), Gaps = 0/50 (0%)
 Strand=Plus/Plus

Query  1     CATGCCCTGTGGTGCAGTCGAGCCAGCACCTGTTTCTGGACCTGCCTAAG  50
             ||||||||||||||||||||||||||||||||||||||||||||||||||
Sbjct  961   CATGCCCTGTGGTGCAGTCGAGCCAGCACCTGTTTCTGGACCTGCCTAAG  1010


>dbj|AK293945.1| Homo sapiens cDNA FLJ53309 complete cds, highly similar to Methionyl-tRNA 
synthetase (EC 6.1.1.10)
Length=2702

 Score = 93.5 bits (50),  Expect = 6e-17
 Identities = 50/50 (100%), Gaps = 0/50 (0%)
 Strand=Plus/Plus

Query  1     CATGCCCTGTGGTGCAGTCGAGCCAGCACCTGTTTCTGGACCTGCCTAAG  50
             ||||||||||||||||||||||||||||||||||||||||||||||||||
Sbjct  1268  CATGCCCTGTGGTGCAGTCGAGCCAGCACCTGTTTCTGGACCTGCCTAAG  1317


>dbj|AK290857.1| Homo sapiens cDNA FLJ76789 complete cds, highly similar to Homo 
sapiens methionine-tRNA synthetase (MARS), mRNA
Length=2780

 Score = 93.5 bits (50),  Expect = 6e-17
 Identities = 50/50 (100%), Gaps = 0/50 (0%)
 Strand=Plus/Plus

Query  1     CATGCCCTGTGGTGCAGTCGAGCCAGCACCTGTTTCTGGACCTGCCTAAG  50
             ||||||||||||||||||||||||||||||||||||||||||||||||||
Sbjct  1328  CATGCCCTGTGGTGCAGTCGAGCCAGCACCTGTTTCTGGACCTGCCTAAG  1377


>gb|BC065187.1| Homo sapiens methionine-tRNA synthetase, mRNA (cDNA clone IMAGE:5021529), 
partial cds
Length=2830

 Score = 93.5 bits (50),  Expect = 6e-17
 Identities = 50/50 (100%), Gaps = 0/50 (0%)
 Strand=Plus/Plus

Query  1     CATGCCCTGTGGTGCAGTCGAGCCAGCACCTGTTTCTGGACCTGCCTAAG  50
             ||||||||||||||||||||||||||||||||||||||||||||||||||
Sbjct  1354  CATGCCCTGTGGTGCAGTCGAGCCAGCACCTGTTTCTGGACCTGCCTAAG  1403


>dbj|AK122956.1| Homo sapiens cDNA FLJ16674 fis, clone THYMU3008136, highly  similar 
to Methionyl-tRNA synthetase (EC 6.1.1.10)
Length=3574

 Score = 93.5 bits (50),  Expect = 6e-17
 Identities = 50/50 (100%), Gaps = 0/50 (0%)
 Strand=Plus/Plus

Query  1     CATGCCCTGTGGTGCAGTCGAGCCAGCACCTGTTTCTGGACCTGCCTAAG  50
             ||||||||||||||||||||||||||||||||||||||||||||||||||
Sbjct  2005  CATGCCCTGTGGTGCAGTCGAGCCAGCACCTGTTTCTGGACCTGCCTAAG  2054


>gb|BT007338.1| Homo sapiens methionine-tRNA synthetase mRNA, complete cds
Length=2703

 Score = 93.5 bits (50),  Expect = 6e-17
 Identities = 50/50 (100%), Gaps = 0/50 (0%)
 Strand=Plus/Plus

Query  1     CATGCCCTGTGGTGCAGTCGAGCCAGCACCTGTTTCTGGACCTGCCTAAG  50
             ||||||||||||||||||||||||||||||||||||||||||||||||||
Sbjct  1319  CATGCCCTGTGGTGCAGTCGAGCCAGCACCTGTTTCTGGACCTGCCTAAG  1368


>dbj|AK222767.1| Homo sapiens mRNA for methionine-tRNA synthetase variant, clone: 
HEP00371
Length=2738

 Score = 93.5 bits (50),  Expect = 6e-17
 Identities = 50/50 (100%), Gaps = 0/50 (0%)
 Strand=Plus/Plus

Query  1     CATGCCCTGTGGTGCAGTCGAGCCAGCACCTGTTTCTGGACCTGCCTAAG  50
             ||||||||||||||||||||||||||||||||||||||||||||||||||
Sbjct  1339  CATGCCCTGTGGTGCAGTCGAGCCAGCACCTGTTTCTGGACCTGCCTAAG  1388


>gb|BC015011.1| Homo sapiens methionyl-tRNA synthetase, mRNA (cDNA clone IMAGE:4864644), 
partial cds
Length=2790

 Score = 93.5 bits (50),  Expect = 6e-17
 Identities = 50/50 (100%), Gaps = 0/50 (0%)
 Strand=Plus/Plus

Query  1     CATGCCCTGTGGTGCAGTCGAGCCAGCACCTGTTTCTGGACCTGCCTAAG  50
             ||||||||||||||||||||||||||||||||||||||||||||||||||
Sbjct  1317  CATGCCCTGTGGTGCAGTCGAGCCAGCACCTGTTTCTGGACCTGCCTAAG  1366


>gb|AC022506.38| Homo sapiens 12 BAC RP11-181L23 (Roswell Park Cancer Institute 
Human BAC Library) complete sequence
Length=172136

 Score = 93.5 bits (50),  Expect = 6e-17
 Identities = 50/50 (100%), Gaps = 0/50 (0%)
 Strand=Plus/Minus

Query  1       CATGCCCTGTGGTGCAGTCGAGCCAGCACCTGTTTCTGGACCTGCCTAAG  50
               ||||||||||||||||||||||||||||||||||||||||||||||||||
Sbjct  103836  CATGCCCTGTGGTGCAGTCGAGCCAGCACCTGTTTCTGGACCTGCCTAAG  103787


>emb|X94754.1| H.sapiens mRNA for yeast methionyl-tRNA synthetase homologue
Length=2779

 Score = 93.5 bits (50),  Expect = 6e-17
 Identities = 50/50 (100%), Gaps = 0/50 (0%)
 Strand=Plus/Plus

Query  1     CATGCCCTGTGGTGCAGTCGAGCCAGCACCTGTTTCTGGACCTGCCTAAG  50
             ||||||||||||||||||||||||||||||||||||||||||||||||||
Sbjct  1326  CATGCCCTGTGGTGCAGTCGAGCCAGCACCTGTTTCTGGACCTGCCTAAG  1375


>emb|Z49216.1| H.sapiens mitoxantrone-resistance associated mRNA
Length=1550

 Score = 93.5 bits (50),  Expect = 6e-17
 Identities = 50/50 (100%), Gaps = 0/50 (0%)
 Strand=Plus/Plus

Query  1    CATGCCCTGTGGTGCAGTCGAGCCAGCACCTGTTTCTGGACCTGCCTAAG  50
            ||||||||||||||||||||||||||||||||||||||||||||||||||
Sbjct  53   CATGCCCTGTGGTGCAGTCGAGCCAGCACCTGTTTCTGGACCTGCCTAAG  102


>gb|AY889906.1| Synthetic construct Homo sapiens clone FLH022775.01X methionine-tRNA 
synthetase (MARS) mRNA, complete cds
Length=2703

 Score = 93.5 bits (50),  Expect = 6e-17
 Identities = 50/50 (100%), Gaps = 0/50 (0%)
 Strand=Plus/Plus

Query  1     CATGCCCTGTGGTGCAGTCGAGCCAGCACCTGTTTCTGGACCTGCCTAAG  50
             ||||||||||||||||||||||||||||||||||||||||||||||||||
Sbjct  1319  CATGCCCTGTGGTGCAGTCGAGCCAGCACCTGTTTCTGGACCTGCCTAAG  1368


>gb|BC011849.2| Homo sapiens methionyl-tRNA synthetase, mRNA (cDNA clone MGC:20313 
IMAGE:4135154), complete cds
Length=2800

 Score = 93.5 bits (50),  Expect = 6e-17
 Identities = 50/50 (100%), Gaps = 0/50 (0%)
 Strand=Plus/Plus

Query  1     CATGCCCTGTGGTGCAGTCGAGCCAGCACCTGTTTCTGGACCTGCCTAAG  50
             ||||||||||||||||||||||||||||||||||||||||||||||||||
Sbjct  1325  CATGCCCTGTGGTGCAGTCGAGCCAGCACCTGTTTCTGGACCTGCCTAAG  1374


>gb|BC011639.2| Homo sapiens methionyl-tRNA synthetase, mRNA (cDNA clone MGC:12828 
IMAGE:4120680), complete cds
Length=2799

 Score = 93.5 bits (50),  Expect = 6e-17
 Identities = 50/50 (100%), Gaps = 0/50 (0%)
 Strand=Plus/Plus

Query  1     CATGCCCTGTGGTGCAGTCGAGCCAGCACCTGTTTCTGGACCTGCCTAAG  50
             ||||||||||||||||||||||||||||||||||||||||||||||||||
Sbjct  1327  CATGCCCTGTGGTGCAGTCGAGCCAGCACCTGTTTCTGGACCTGCCTAAG  1376


>gb|BC006328.2| Homo sapiens methionyl-tRNA synthetase, mRNA (cDNA clone MGC:12889 
IMAGE:4139330), complete cds
Length=2795

 Score = 93.5 bits (50),  Expect = 6e-17
 Identities = 50/50 (100%), Gaps = 0/50 (0%)
 Strand=Plus/Plus

Query  1     CATGCCCTGTGGTGCAGTCGAGCCAGCACCTGTTTCTGGACCTGCCTAAG  50
             ||||||||||||||||||||||||||||||||||||||||||||||||||
Sbjct  1325  CATGCCCTGTGGTGCAGTCGAGCCAGCACCTGTTTCTGGACCTGCCTAAG  1374


>gb|BC002384.2| Homo sapiens methionyl-tRNA synthetase, mRNA (cDNA clone MGC:8646 
IMAGE:2961615), complete cds
Length=2799

 Score = 93.5 bits (50),  Expect = 6e-17
 Identities = 50/50 (100%), Gaps = 0/50 (0%)
 Strand=Plus/Plus

Query  1     CATGCCCTGTGGTGCAGTCGAGCCAGCACCTGTTTCTGGACCTGCCTAAG  50
             ||||||||||||||||||||||||||||||||||||||||||||||||||
Sbjct  1325  CATGCCCTGTGGTGCAGTCGAGCCAGCACCTGTTTCTGGACCTGCCTAAG  1374


>dbj|D84224.1| Homo sapiens mRNA for methionyl tRNA synthetase, complete cds
Length=2800

 Score = 93.5 bits (50),  Expect = 6e-17
 Identities = 50/50 (100%), Gaps = 0/50 (0%)
 Strand=Plus/Plus

Query  1     CATGCCCTGTGGTGCAGTCGAGCCAGCACCTGTTTCTGGACCTGCCTAAG  50
             ||||||||||||||||||||||||||||||||||||||||||||||||||
Sbjct  1342  CATGCCCTGTGGTGCAGTCGAGCCAGCACCTGTTTCTGGACCTGCCTAAG  1391


>ref|XM_003824853.1| PREDICTED: Pan paniscus methionyl-tRNA synthetase (MARS), mRNA
Length=3665

 Score = 87.9 bits (47),  Expect = 3e-15
 Identities = 49/50 (98%), Gaps = 0/50 (0%)
 Strand=Plus/Plus

Query  1     CATGCCCTGTGGTGCAGTCGAGCCAGCACCTGTTTCTGGACCTGCCTAAG  50
             ||||||||||||||||||||| ||||||||||||||||||||||||||||
Sbjct  2010  CATGCCCTGTGGTGCAGTCGACCCAGCACCTGTTTCTGGACCTGCCTAAG  2059


>ref|XM_509164.3| PREDICTED: Pan troglodytes methionyl-tRNA synthetase (MARS), 
mRNA
Length=3469

 Score = 87.9 bits (47),  Expect = 3e-15
 Identities = 49/50 (98%), Gaps = 0/50 (0%)
 Strand=Plus/Plus

Query  1     CATGCCCTGTGGTGCAGTCGAGCCAGCACCTGTTTCTGGACCTGCCTAAG  50
             ||||||||||||||||||||| ||||||||||||||||||||||||||||
Sbjct  2010  CATGCCCTGTGGTGCAGTCGACCCAGCACCTGTTTCTGGACCTGCCTAAG  2059


>gb|AC191734.3| Pan troglodytes BAC clone CH251-555H2 from chromosome 12, complete 
sequence
Length=223618

 Score = 87.9 bits (47),  Expect = 3e-15
 Identities = 49/50 (98%), Gaps = 0/50 (0%)
 Strand=Plus/Minus

Query  1      CATGCCCTGTGGTGCAGTCGAGCCAGCACCTGTTTCTGGACCTGCCTAAG  50
              ||||||||||||||||||||| ||||||||||||||||||||||||||||
Sbjct  76081  CATGCCCTGTGGTGCAGTCGACCCAGCACCTGTTTCTGGACCTGCCTAAG  76032


>ref|XM_003252767.1| PREDICTED: Nomascus leucogenys methionyl-tRNA synthetase (MARS), 
mRNA
Length=2916

 Score = 82.4 bits (44),  Expect = 1e-13
 Identities = 48/50 (96%), Gaps = 0/50 (0%)
 Strand=Plus/Plus

Query  1     CATGCCCTGTGGTGCAGTCGAGCCAGCACCTGTTTCTGGACCTGCCTAAG  50
             |||| |||||||||||||||| ||||||||||||||||||||||||||||
Sbjct  1457  CATGTCCTGTGGTGCAGTCGACCCAGCACCTGTTTCTGGACCTGCCTAAG  1506


Lambda     K      H
    1.33    0.621     1.12 

Gapped
Lambda     K      H
    1.28    0.460    0.850 

Effective search space used: 874911716973
 
 right 
 >ref|XR_110367.3| PREDICTED: Homo sapiens uncharacterized LOC100506869, transcript 
variant 1 (LOC100506869), misc_RNA
Length=766

 Score = 93.5 bits (50),  Expect = 6e-17
 Identities = 50/50 (100%), Gaps = 0/50 (0%)
 Strand=Plus/Plus

Query  1   AATAGAATGCAACTGAAGAAATGGCTGGTGCAATTCAGCAAGGTAAAAGA  50
           ||||||||||||||||||||||||||||||||||||||||||||||||||
Sbjct  3   AATAGAATGCAACTGAAGAAATGGCTGGTGCAATTCAGCAAGGTAAAAGA  52


>ref|XR_111312.3| PREDICTED: Homo sapiens uncharacterized LOC100506869, transcript 
variant 1 (LOC100506869), misc_RNA
Length=766

 Score = 93.5 bits (50),  Expect = 6e-17
 Identities = 50/50 (100%), Gaps = 0/50 (0%)
 Strand=Plus/Plus

Query  1   AATAGAATGCAACTGAAGAAATGGCTGGTGCAATTCAGCAAGGTAAAAGA  50
           ||||||||||||||||||||||||||||||||||||||||||||||||||
Sbjct  3   AATAGAATGCAACTGAAGAAATGGCTGGTGCAATTCAGCAAGGTAAAAGA  52


>gb|AC020637.9|AC020637 Homo sapiens 12 BAC RP11-530H22 (Roswell Park Cancer Institute 
Human BAC Library) complete sequence
Length=177317

 Score = 93.5 bits (50),  Expect = 6e-17
 Identities = 50/50 (100%), Gaps = 0/50 (0%)
 Strand=Plus/Plus

Query  1       AATAGAATGCAACTGAAGAAATGGCTGGTGCAATTCAGCAAGGTAAAAGA  50
               ||||||||||||||||||||||||||||||||||||||||||||||||||
Sbjct  176995  AATAGAATGCAACTGAAGAAATGGCTGGTGCAATTCAGCAAGGTAAAAGA  177044


>ref|XM_003824823.1| PREDICTED: Pan paniscus uncharacterized LOC100970154 (LOC100970154), 
mRNA
Length=760

 Score = 80.5 bits (43),  Expect = 5e-13
 Identities = 45/46 (97%), Gaps = 0/46 (0%)
 Strand=Plus/Plus

Query  5   GAATGCAACTGAAGAAATGGCTGGTGCAATTCAGCAAGGTAAAAGA  50
           |||||||||||| |||||||||||||||||||||||||||||||||
Sbjct  1   GAATGCAACTGAGGAAATGGCTGGTGCAATTCAGCAAGGTAAAAGA  46


>ref|XM_003313542.1| PREDICTED: Pan troglodytes hypothetical protein LOC100611860 
(LOC100611860), mRNA
Length=765

 Score = 80.5 bits (43),  Expect = 5e-13
 Identities = 45/46 (97%), Gaps = 0/46 (0%)
 Strand=Plus/Plus

Query  5   GAATGCAACTGAAGAAATGGCTGGTGCAATTCAGCAAGGTAAAAGA  50
           |||||||||||| |||||||||||||||||||||||||||||||||
Sbjct  1   GAATGCAACTGAGGAAATGGCTGGTGCAATTCAGCAAGGTAAAAGA  46


>ref|XR_111313.2| PREDICTED: Homo sapiens uncharacterized LOC100506869, transcript 
variant 2 (LOC100506869), misc_RNA
Length=464

 Score = 78.7 bits (42),  Expect = 2e-12
 Identities = 42/42 (100%), Gaps = 0/42 (0%)
 Strand=Plus/Plus

Query  1    AATAGAATGCAACTGAAGAAATGGCTGGTGCAATTCAGCAAG  42
            ||||||||||||||||||||||||||||||||||||||||||
Sbjct  167  AATAGAATGCAACTGAAGAAATGGCTGGTGCAATTCAGCAAG  208


>ref|XR_110368.2| PREDICTED: Homo sapiens uncharacterized LOC100506869, transcript 
variant 2 (LOC100506869), misc_RNA
Length=464

 Score = 78.7 bits (42),  Expect = 2e-12
 Identities = 42/42 (100%), Gaps = 0/42 (0%)
 Strand=Plus/Plus

Query  1    AATAGAATGCAACTGAAGAAATGGCTGGTGCAATTCAGCAAG  42
            ||||||||||||||||||||||||||||||||||||||||||
Sbjct  167  AATAGAATGCAACTGAAGAAATGGCTGGTGCAATTCAGCAAG  208


Lambda     K      H
    1.33    0.621     1.12 

Gapped
Lambda     K      H
    1.28    0.460    0.850 

Effective search space used: 874911716973
 
    reads 
 chr12 chr12 57894225 57897970 74M3669N2M                     |||||||||||||||||||||||||||||||||||||||||||||||||||||||||||||||||||||||||||||||||||||||||||||||||||||||||||||||||||||||||||||||||||||||||||||||||||||||||||||||||||||||||||||||||||||||||||CT
chr12 chr12 57894230 57898008 75M3702N1M                     ||||||||||||||||||||||||||||||||||||||||||||||||||||||||||||||||||||||||||||||||||||||||||||||||||||||||||||||||||||||||||||||||||||||||||||||||||||||||||||||||||||||||||||||||||||||||||||||||||||||||||||||||||||||||||||||||||A
chr12 chr12 57894233 57898011 72M3702N4M                     ||||||||||||||||||||||||||||||||||||||||||||||||||||||||||||||||||||||||||||||||||||||||||||||||||||||||||||||||||||||||||||||||||||||||||||||||||||||||||||||||||||||||||||||||||||||||||||||||||||||||||||||||||||||||||||||||||AAGC
chr12 chr12 57894236 57898014 69M3702N7M                     ||||||||||||||||||||||||||||||||||||||||||||||||||||||||||||||||||||||||||||||||||||||||||||||||||||||||||||||||||||||||||||||||||||||||||||||||||||||||||||||||||||||||||||||||||||||||||||||||||||||||||||||||||||||||||||||||||AAGCCTC
chr12 chr12 57894239 57898017 66M3702N10M                    ||||||||||||||||||||||||||||||||||||||||||||||||||||||||||||||||||||||||||||||||||||||||||||||||||||||||||||||||||||||||||||||||||||||||||||||||||||||||||||||||||||||||||||||||||||||||||||||||||||||||||||||||||||||||||||||||||AAGCCTCAGT
chr12 chr12 57894242 57898020 63M3702N13M                    ||||||||||||||||||||||||||||||||||||||||||||||||||||||||||||||||||||||||||||||||||||||||||||||||||||||||||||||||||||||||||||||||||||||||||||||||||||||||||||||||||||||||||||||||||||||||||||||||||||||||||||||||||||||||||||||||||AAGCCTCAGTGTA
chr12 chr12 57894245 57898023 60M3702N16M                    ||||||||||||||||||||||||||||||||||||||||||||||||||||||||||||||||||||||||||||||||||||||||||||||||||||||||||||||||||||||||||||||||||||||||||||||||||||||||||||||||||||||||||||||||||||||||||||||||||||||||||||||||||||||||||||||||||AAGCCTCAGTGTAAAG
chr12 chr12 57894248 57898026 57M3702N19M                    ||||||||||||||||||||||||||||||||||||||||||||||||||||||||||||||||||||||||||||||||||||||||||||||||||||||||||||||||||||||||||||||||||||||||||||||||||||||||||||||||||||||||||||||||||||||||||||||||||||||||||||||||||||||||||||||||||AAGCCTCAGTGTAAAGTCT
chr12 chr12 57894251 57898029 54M3702N22M                    ||||||||||||||||||||||||||||||||||||||||||||||||||||||||||||||||||||||||||||||||||||||||||||||||||||||||||||||||||||||||||||||||||||||||||||||||||||||||||||||||||||||||||||||||||||||||||||||||||||||||||||||||||||||||||||||||||AAGCCTCAGTGTAAAGTCTGCC
chr12 chr12 57894254 57898032 51M3702N25M                    ||||||||||||||||||||||||||||||||||||||||||||||||||||||||||||||||||||||||||||||||||||||||||||||||||||||||||||||||||||||||||||||||||||||||||||||||||||||||||||||||||||||||||||||||||||||||||||||||||||||||||||||||||||||||||||||||||AAGCCTCAGTGTAAAGTCTGCCGAT
chr12 chr12 57894257 57898035 48M3702N28M                    ||||||||||||||||||||||||||||||||||||||||||||||||||||||||||||||||||||||||||||||||||||||||||||||||||||||||||||||||||||||||||||||||||||||||||||||||||||||||||||||||||||||||||||||||||||||||||||||||||||||||||||||||||||||||||||||||||AAGCCTCAGTGTAAAGTCTGCCGATCAT
chr12 chr12 57894260 57898038 45M3702N31M                    ||||||||||||||||||||||||||||||||||||||||||||||||||||||||||||||||||||||||||||||||||||||||||||||||||||||||||||||||||||||||||||||||||||||||||||||||||||||||||||||||||||||||||||||||||||||||||||||||||||||||||||||||||||||||||||||||||AAGCCTCCGTGTAAAGGCTGCCGATCATGCC
chr12 chr12 57894263 57898041 42M3702N34M                    ||||||||||||||||||||||||||||||||||||||||||||||||||||||||||||||||||||||||||||||||||||||||||||||||||||||||||||||||||||||||||||||||||||||||||||||||||||||||||||||||||||||||||||||||||||||||||||||||||||||||||||||||||||||||||||||||||AAGCCTCAGTGTAAAGTCTGCCGATCATGCCCTG
chr12 chr12 57894266 57898044 39M3702N37M                    ||||||||||||||||||||||||||||||||||||||||||||||||||||||||||||||||||||||||||||||||||||||||||||||||||||||||||||||||||||||||||||||||||||||||||||||||||||||||||||||||||||||||||||||||||||||||||||||||||||||||||||||||||||||||||||||||||AAGCCTCAGTGTAAAGTCTGCCGATCATGCCCTGTGG
chr12 chr12 57894269 57898047 36M3702N40M                    ||||||||||||||||||||||||||||||||||||||||||||||||||||||||||||||||||||||||||||||||||||||||||||||||||||||||||||||||||||||||||||||||||||||||||||||||||||||||||||||||||||||||||||||||||||||||||||||||||||||||||||||||||||||||||||||||||AAGCCTCAGTGTAAAGTCTGCCGATCATGCCCTGTGGTGC
chr12 chr12 57894272 57898050 33M3702N43M                    ||||||||||||||||||||||||||||||||||||||||||||||||||||||||||||||||||||||||||||||||||||||||||||||||||||||||||||||||||||||||||||||||||||||||||||||||||||||||||||||||||||||||||||||||||||||||||||||||||||||||||||||||||||||||||||||||||AAGCCTCAGTGTAAAGTCTGCCGATCATGCCCTGTGGTGCAGA
chr12 chr12 57894275 57898053 30M3702N46M                    ||||||||||||||||||||||||||||||||||||||||||||||||||||||||||||||||||||||||||||||||||||||||||||||||||||||||||||||||||||||||||||||||||||||||||||||||||||||||||||||||||||||||||||||||||||||||||||||||||||||||||||||||||||||||||||||||||AAGCCTCAGTGTAAAGTCTGCCGATCATGCCCTGTGGTGCAGTCGA
chr12 chr12 57894278 57898056 27M3702N49M                    ||||||||||||||||||||||||||||||||||||||||||||||||||||||||||||||||||||||||||||||||||||||||||||||||||||||||||||||||||||||||||||||||||||||||||||||||||||||||||||||||||||||||||||||||||||||||||||||||||||||||||||||||||||||||||||||||||AAGCCTCAGTGTAAAGTCTGCCGATCATGCCCTGTGGTGCAGTCGAGCC
chr12 chr12 57894281 57898059 24M3702N52M                    ||||||||||||||||||||||||||||||||||||||||||||||||||||||||||||||||||||||||||||||||||||||||||||||||||||||||||||||||||||||||||||||||||||||||||||||||||||||||||||||||||||||||||||||||||||||||||||||||||||||||||||||||||||||||||||||||||AAGCCTCAGTGTAAAGTCTGCCGATCATGCCCTGTGGTGCAGTCGAGCCAGC
chr12 chr12 57894284 57898062 21M3702N55M                    ||||||||||||||||||||||||||||||||||||||||||||||||||||||||||||||||||||||||||||||||||||||||||||||||||||||||||||||||||||||||||||||||||||||||||||||||||||||||||||||||||||||||||||||||||||||||||||||||||||||||||||||||||||||||||||||||||AAGCCTCAGTGTAAAGTCTGCCGATCATGCCCTGTGGTGCAGTCGAGCCAGCACC
chr12 chr12 57894287 57898065 18M3702N58M                    ||||||||||||||||||||||||||||||||||||||||||||||||||||||||||||||||||||||||||||||||||||||||||||||||||||||||||||||||||||||||||||||||||||||||||||||||||||||||||||||||||||||||||||||||||||||||||||||||||||||||||||||||||||||||||||||||||AAGCCTCAGTGTAAAGTCTGCCGATCATGCCCTGTGGTGCAGTCGAGCCAGCACCTGT
chr12 chr12 57894290 57898068 15M3702N61M                    ||||||||||||||||||||||||||||||||||||||||||||||||||||||||||||||||||||||||||||||||||||||||||||||||||||||||||||||||||||||||||||||||||||||||||||||||||||||||||||||||||||||||||||||||||||||||||||||||||||||||||||||||||||||||||||||||||AAGCCTCAGTGTAAAGTCTGCCGATCATGCCCTGTGGTGCAGTCGAGCCAGCACCTGTTTC
chr12 chr12 57894293 57898071 12M3702N64M                    ||||||||||||||||||||||||||||||||||||||||||||||||||||||||||||||||||||||||||||||||||||||||||||||||||||||||||||||||||||||||||||||||||||||||||||||||||||||||||||||||||||||||||||||||||||||||||||||||||||||||||||||||||||||||||||||||||AAGCCTCAGTGTAAAGTCTGCCGATCATGCCCTGTGGTGCAGTCGAGCCAGCACCTGTTTCTGG
chr12 chr12 57894296 57898074 9M3702N67M                     ||||||||||||||||||||||||||||||||||||||||||||||||||||||||||||||||||||||||||||||||||||||||||||||||||||||||||||||||||||||||||||||||||||||||||||||||||||||||||||||||||||||||||||||||||||||||||||||||||||||||||||||||||||||||||||||||||AAGCCTCAGTTTAAAGTCTGCCGATCATGCCCTGTGGTGCAGTCGAGCCAGCACCTGTTTCTGGACC
chr12 chr12 57894299 57898077 6M3702N70M                     ||||||||||||||||||||||||||||||||||||||||||||||||||||||||||||||||||||||||||||||||||||||||||||||||||||||||||||||||||||||||||||||||||||||||||||||||||||||||||||||||||||||||||||||||||||||||||||||||||||||||||||||||||||||||||||||||||AAGCCTCAGTGTAAAGTCTGCCGATCATGCCCTGTGGTGCAGTCGAGCCAGCACCTGTTTCTGGACCTGC
chr12 chr12 57894302 57898080 3M3702N73M                     ||||||||||||||||||||||||||||||||||||||||||||||||||||||||||||||||||||||||||||||||||||||||||||||||||||||||||||||||||||||||||||||||||||||||||||||||||||||||||||||||||||||||||||||||||||||||||||||||||||||||||||||||||||||||||||||||||AGGCCTCAGTGTAAAGTCTGCCGATCATGCCCTGTGGTCCAGTCGAGCCAGCACCTGTTTCTGGACCTGCCTA
chr12 chr12 57897715 57897791 76M                            GGTGTGATGG
chr12 chr12 57897719 57897795 76M                            GGTGTGATGGGACA
chr12 chr12 57897742 57897818 76M                            GGTGTGATGGGACAGAGATCTGAAACTCTGAGAACAA
chr12 chr12 57897746 57897822 76M                            GGTGTGATGGGACAGAGATCTGAAACTCTGAGAACAAATGG
chr12 chr12 57897780 57897856 76M                            GGTGTGATGGGACAGAGATCTGAAACTCTGAGAACAAATGGAGTTATACTGAGAAGATTTGAGTCTCCATGTTTA
chr12 chr12 57897786 57897862 76M                                 GATGGGACAGAGATCTGAAACTCTGAGAACAAATGGAGTTATACTGAGAAGATTTGAGTCTCCATGTTTAGAGTAG
chr12 chr12 57897804 57897880 76M                                                   AACTCTGAGAACAAATGGAGTTATACTGAGAAGATTTGAGTCTCCATGTTTAGAGTAGGCCACATAGTAGAGTTAT
chr12 chr12 57897832 57897908 76M                                                                               AGAAGATTTGAGTCTCCATGTTTAGAGTAGGCCACATAGTAGAGTTATTGGAGTGTGAGCCTGAAGCTAAACTAGA
chr12 chr12 57897852 57897928 76M                                                                                                   TTTAGAGTAGGCCACATAGTAGAGTTATTGGAGTGTGAGCCTGAAGCTAAACTAGATGGCAGACTGAGGAGCTAAT
chr12 chr12 57897874 57897950 76M                                                                                                                         AGTTATTGGAGTGTGAGCCTGAAGCTAAACTAGATGGCAGACTGAGGAGCTAATCCTTCTCTGCTCCTCCCCTTGC
chr12 chr12 57897880 57897956 76M                                                                                                                               TGGAGTGTGAGCCTGAAGCTAAACTAGATGGCAGACTGAGGAGCTAATCCTTCTCTGCTCCTCCCCTTGCCTGGAC
chr12 chr12 57897893 57897969 76M                                                                                                                                            TGAAGCTAAACTAGATGGCAGACTGAGGAGCTAATCCTTCTCTGCTCCTCCCCTTGCCTGGACCCCTCCCCTGGCC
chr12 chr12 57897903 57897979 76M                                                                                                                                                      CTAGATGGCAGACTGAGGAGCTAATCCTTCTCTGCTCCTCCCCTTGCCTGGACCCCTCCCCTGGCCTGCAGGCCTG
chr12 chr12 57897909 57897985 76M                                                                                                                                                            GGCAGACTGAGGAGCTAATCCTTCTCTGCTCCTCCCCTTGCCTGGACCCCTCCCCTGGCCTGCAGGCCTGATCTGT
chr12 chr12 57897923 57897999 76M                                                                                                                                                                          CTAATCCTTCTCTGCTCCTCCCCTTGCCTGGACCCCTCCCCTGGCCTGCAGGCCTGATCTGTCCTCTGGAATTTTC
chr12 chr12 57897928 57898004 76M                                                                                                                                                                               CCTTCTCTGCTCCTCCCCTTGCCTGGACCCCTCCCCTGGCCTGCAGGCCTGATCTGTCCTCTGGAATTTTCCTTCG
chr12 chr12 57897953 57898029 76M                                                                                                                                                                                                        GACCCCTCCCCTGGCCTGCAGGCCTGATCTGTCCTCTGGAATTTTCCTTCGCAGAAGCCTCAGTGTAAAGTCTGCC
chr12 chr12 57897972 57898048 76M                                                                                                                                                                                                                           AGGCCTGATCTGTCCTCTGGAATTTTCCTTCGCAGAAGCCTCAGTGTAAAGTCTGCCGATCATGCCCTGTGGTGCA
chr12 chr12 57897976 57898052 76M                                                                                                                                                                                                                               CTGATCTGTCCTCTGGAATTTTCCTTCGCAGAAGCCTCAGTGTAAAGTCTGCCGATCATGCCCTGTGGTGCAGTCG
chr12 chr12 57897989 57898065 76M                                                                                                                                                                                                                                            TGGAATTTTCCTTCGCAGAAGCCTCAGTGTAAAGTCTGCCGATCATGCCCTGTGGTGCAGTCGAGCCAGCACCTGT
chr12 chr12 57897995 57898071 76M                                                                                                                                                                                                                                                  TTTCCTTCGCAGAAGCCTCAGTGTAAAGTCTGCCGATCATGCCCTGTGGTGCAGTCGAGCCAGCACCTGTTTCTGG
chr12 chr12 57898003 57898079 76M                                                                                                                                                                                                                                                          TAAGAAGCCTCAGTGTAAAGTCTGCCGATCATGCCCTGTGGTGCAGTCGAGCCAGCACCTGTTTCTGGACCTGCCT
chr12 chr12 57898006 57898082 76M                                                                                                                                                                                                                                                             GAAGCCTCAGTGTAAAGTCTCCCGATCATGCCCTGTGGTGCAGTCGAGCCAGCACCTGTTTCTGGACCTGCCTAAG
chr12 chr12 57898009 57898085 76M                                                                                                                                                                                                                                                                GCCTCAGTGTAAAGTCTGCCGATCATGCCCTGTGGTGCAGTCGAGCCAGCACCTGTTTCTGGACCTGCCTAAGCTG
chr12 chr12 57898010 59199726 72M59199723F4M                                                                                                                                                                                                                                                      CCTCAGTGTAAAGTCTGCCGATCATGCCCTGTGGTGCAGTCGAGCCAGCACCTGTTTCTGGACCTGCCTAAG AATA
chr12 chr12 57898010 59199726 72M59199723F4M                                                                                                                                                                                                                                                      CCTCAGTGTAAAGTCTGCCGATCATGCCCTGTGGTGCAGTCGAGCCAGCACCTGTTTCTGGACCTGCCTAAG AATA
chr12 chr12 57898010 59199726 72M59199723F4M                                                                                                                                                                                                                                                      CCTCAGTGTAAAGTCTGCCGATCATGCCCTGTGGTGCAGTCGAGCCAGCACCTGTTTCTGGACCTGCCTAAG AATA
chr12 chr12 57898010 59199726 72M59199723F4M                                                                                                                                                                                                                                                      CCTCAGTGTAAAGTCTGCCGATCATGCCCTGTGGTGCAGTCGAGCCAGCACCTGTTTCTGGACCTGCCTAAG AATA
chr12 chr12 57898010 59199726 72M59199723F4M                                                                                                                                                                                                                                                      CCTCAGTGTAAAGTCTGCCGATCATGCCCTGTGGTGCAGTCGAGCCAGCACCTGTTTCTGGACCTGCCTAAG AATA
chr12 chr12 57898010 59199726 72M59199723F4M                                                                                                                                                                                                                                                      CCTCAGTGTAAAGTCTGCCGATCATGCCCTGTGGTGCAGTCGAGCCAGCACCTGTTTCTGGACCTGCCTAAG AATA
chr12 chr12 57898010 59199726 72M59199723F4M                                                                                                                                                                                                                                                      CCTCAGTGTAAAGTCTGCCGATCATGCCCTGTGGTGCAGTCGAGCCAGCACCTGTTTCTGGACCTGCCTAAG AATA
chr12 chr12 57898010 59199726 72M59199723F4M                                                                                                                                                                                                                                                      CCTCAGTGTAAAGTCTGCCGATCATGCCCTGTGGTGCAGTCGAGCCAGCACCTGTTTCTGGACCTGCCTAAG AATA
chr12 chr12 57898010 59199726 72M59199723F4M                                                                                                                                                                                                                                                      CCTCAGTGTAAAGTCTGCCGATCATGCCCTGTGGTGCAGTCGAGCCAGCACCTGTTTCTGGACCTGCCTAAG AATA
chr12 chr12 57898010 59199726 72M59199723F4M                                                                                                                                                                                                                                                      CCTCAGTGTAAAGTCTGCCGATCATGCCCTGTGGTGCAGTCGAGCCAGCACCTGTTTCTGGACCTGCCTAAG AATA
chr12 chr12 57898011 59199727 71M59199723F5M                                                                                                                                                                                                                                                       CTCAGTGTAAAGTCTGCCGATCATGCCCTGTGGTGCAGTCGAGCCAGCACCTGTTTCTGGACCTGCCTAAG AATAG
chr12 chr12 57898011 59199727 71M59199723F5M                                                                                                                                                                                                                                                       CTCAGTGTAAAGTCTGCCGATCATGCCCTGTGGTGCAGTCGAGCCAGCACCTGTTTCTGGACCTGCCTAAG AATAG
chr12 chr12 57898011 59199727 71M59199723F5M                                                                                                                                                                                                                                                       CTCAGTGTAAAGTCTGCCGATCATGCCCTGTGGTGCAGTCGAGCCAGCACCTGTTTCTGGACCTGCCTAAG AATAG
chr12 chr12 57898013 59199729 69M59199723F7M                                                                                                                                                                                                                                                         CAGTCTAAAGTCTGCCGATCATGCCCTGTCGTGCAGTCGAGCCAGCACCTGTTTCTGGACCTGCCTAAG AATAGAA
chr12 chr12 57898014 59199730 68M59199723F8M                                                                                                                                                                                                                                                          AGTGTAAAGTCTGCCGATCATGCCCTGTGGTGCAGTCGAGCCAGCACCTGTTTCTGGACCTGCCTAAG AATAGAAT
chr12 chr12 57898015 59199731 67M59199723F9M                                                                                                                                                                                                                                                           GTGTAAAGTCTGCCGATCATGCCCTGTGGTGCAGTCGAGCCAGCACCTGTTTCTGGACCTGCCTAAG AATAGAATG
chr12 chr12 57898015 59199731 67M59199723F9M                                                                                                                                                                                                                                                           GTGTAAAGTCTGCCGATCATGCCCTGTGGTGCAGTCGAGCCAGCACCTGTTTCTGGACCTGCCTAAG AATAGAATG
chr12 chr12 57898016 59199732 66M59199723F10M                                                                                                                                                                                                                                                           TGTAAAGTCTGCCGATCATGCCCTGTGGTGCAGTCGAGCCAGCACCTGTTTCTGGACCTGCCTAAG AATAGAATGC
chr12 chr12 57898016 59199732 66M59199723F10M                                                                                                                                                                                                                                                           TGTAAAGTCTGCCGATCATGCCCTGTGGTGCAGTCGAGCCAGCACCTGTTTCTGGACCTGCCTAAG AATAGAATGC
chr12 chr12 57898017 59199733 65M59199723F11M                                                                                                                                                                                                                                                            GTAAAGTCTGCCGATCATGCCCTGTGGTGCAGTCGAGCCAGCACCTGTTTCTGGACCTGCCTAAG AATAGAATGCA
chr12 chr12 57898017 59199733 65M59199723F11M                                                                                                                                                                                                                                                            GTAAAGTCTGCCGATCATGCCCTGTGGTGCAGTCGAGCCAGCACCTGTTTCTGGACCTGCCTAAG AATAGAATGCA
chr12 chr12 57898017 59199733 65M59199723F11M                                                                                                                                                                                                                                                            GTAAAGTCTGCCGATCATGCCCTGTGGTGCAGTCGAGCCAGCACCTGTTTCTGGACCTGCCTAAG AATAGAATGCA
chr12 chr12 57898017 59199733 65M59199723F11M                                                                                                                                                                                                                                                            GTAAAGTCTGCCGATCATGCCCTGTGGTGCAGTCGAGCCAGCACCTGTTTCTGGACCTGCCTAAG AATAGAATGCA
chr12 chr12 57898017 59199733 65M59199723F11M                                                                                                                                                                                                                                                            GTAAAGTCTGCCGATCATGCCCTGTGGTGCAGTCGAGCCAGCACCTGTTTCTGGACCTGCCTAAG AATAGAATGCA
chr12 chr12 57898017 59199733 65M59199723F11M                                                                                                                                                                                                                                                            GTAAAGTCTGCCGATCATGCCCTGTGGTGCAGTCGAGCCAGCACCTGTTTCTGGACCTGCCTAAG AATAGAATGCA
chr12 chr12 57898017 59199733 65M59199723F11M                                                                                                                                                                                                                                                            GTAAAGTCTGCCGATCATGCCCTGTGGTGCAGTCGAGCCAGCACCTGTTTCTGGACCTGCCTAAG AATAGAATGCA
chr12 chr12 57898017 59199733 65M59199723F11M                                                                                                                                                                                                                                                            GTAAAGTCTGCCGATCATGCCCTGTGGTGCAGTCGAGCCAGCACCTGTTTCTGGACCTGCCTAAG AATAGAATGCA
chr12 chr12 57898017 59199733 65M59199723F11M                                                                                                                                                                                                                                                            GTAAAGTCTGCCGATCATGCCCTGTGGTGCAGTCGAGCCAGCACCTGTTTCTGGACCTGCCTAAG AATAGAATGCA
chr12 chr12 57898018 59199734 64M59199723F12M                                                                                                                                                                                                                                                             TAAAGTCTGCCGATCATGCCCTGTGGTGCAGTCGAGCCAGCACCTGTTTCTGGACCTGCCTAAG AATAGAATGCAA
chr12 chr12 57898018 59199734 64M59199723F12M                                                                                                                                                                                                                                                             TAAAGTCTGCCGATCATGCCCTGTGGTGCAGTCGAGCCAGCACCTGTTTCTGGACCTGCCTAAG AATAGAATGCAA
chr12 chr12 57898018 59199734 64M59199723F12M                                                                                                                                                                                                                                                             TAAAGTCTGCCGATCATGCCCTGTGGTGCAGTCGAGCCAGCACCTGTTTCTGGACCTGCCTAAG AATAGAATGCAA
chr12 chr12 57898019 59199735 63M59199723F13M                                                                                                                                                                                                                                                              AAAGTCTGCCGATCATGCCCTGTGGCGCAGTCGAGCCAGCACCTGTTTCTGGACCTGCCTAAG AATAGAATGCAAC
chr12 chr12 57898019 59199735 63M59199723F13M                                                                                                                                                                                                                                                              AAAGTCTGCCGATCATGCCCTGTGGTGCAGTCGAGCCAGCACCTGTTTCTGGACCTGCCTAAG AATAGAATGCAAC
chr12 chr12 57898019 59199735 63M59199723F13M                                                                                                                                                                                                                                                              AAAGTCTGCCGATCATGCCCTGTGGTGCAGTCGAGCCAGCACCTGTTTCTGGACCTGCCTAAG AATAGAATGCAAC
chr12 chr12 57898019 59199735 63M59199723F13M                                                                                                                                                                                                                                                              AAATTCTGCCGATCATGCCCTGTGGTGCATTCGAGCCAGCACCTGTTTCTGGACCTGCCTAAG AATAGAATGCAAC
chr12 chr12 57898019 59199735 63M59199723F13M                                                                                                                                                                                                                                                              AAAGTCTGCCGATCATGCCCTGTGGTGCAGTCGAGCCAGCACCTGTTTCTGGACCTGCCTAAG AATAGAATGCAAC
chr12 chr12 57898019 59199735 63M59199723F13M                                                                                                                                                                                                                                                              AAAGTCTGCCGATCATGCCCTGTGGTGCAGTCGAGCCAGCACCTGTTTCTGGACCTGCCTAAG AATAGAATGCAAC
chr12 chr12 57898021 59199737 61M59199723F15M                                                                                                                                                                                                                                                                AGTCTGCCGATCATGCCCTGTGGTGCAGTCGAGCCAGCACCTGTTTCTGGACCTGCCTAAG AATAGAATGCAACTG
chr12 chr12 57898022 59199738 60M59199723F16M                                                                                                                                                                                                                                                                 GTCTGCCGATCATGCCCTGTGGTGCAGTCGAGCCAGCACCTGTTTCTGGACCTGCCTAAG AATAGAATGCAACTGA
chr12 chr12 57898024 59199740 58M59199723F18M                                                                                                                                                                                                                                                                   CTGCCGATCATGCCCTGTGGTGCAGTCGAGCCAGCACCTGTTTCTGGACCTGCCTAAG AATAGAATGCAACTGAAG
chr12 chr12 57898024 59199740 58M59199723F18M                                                                                                                                                                                                                                                                   CTGCCGATCATGCCCTGTGGTGCAGTCGAGCCAGCACCTGTTTCTGGACCTGCCTAAG AATAGAATGCAACTGAAG
chr12 chr12 57898024 59199740 58M59199723F18M                                                                                                                                                                                                                                                                   CTGCCGATCATGCCCTGTGGTGCAGTCGAGCCAGCACCTGTTTCTGGACCTGCCTAAG AATAGAATGCAACTGAAG
chr12 chr12 57898024 59199740 58M59199723F18M                                                                                                                                                                                                                                                                   CTGCCGATCATGCCCTGTGGTGCAGTCGAGCCAGCACCTGTTTCTGGACCTGCCTAAG AATAGAATGCAACTGAAG
chr12 chr12 57898024 59199740 58M59199723F18M                                                                                                                                                                                                                                                                   CTGCCGATCATGCCCTGTGGTGCAGTCGAGCCAGCACCTGTTTCTGGACCTGCCTAAG AATAGAATGCAACTGAAG
chr12 chr12 57898025 59199741 57M59199723F19M                                                                                                                                                                                                                                                                    TGCCGATCATGCCCTGTGGTGCAGTCGAGCCAGCACCTGTTTCTGGACCTGCCTAAG AATAGAATGCAACTGAAGA
chr12 chr12 57898025 59199741 57M59199723F19M                                                                                                                                                                                                                                                                    TGCCGATCATGCCCTGTGGTGCAGTCGAGCCAGCACCTGTTTCTGGACCTGCCTAAG AATAGAATGCAACTGAAGA
chr12 chr12 57898025 59199741 57M59199723F19M                                                                                                                                                                                                                                                                    TGCCGATCATGCCCTGTGGTGCAGTCGAGCCAGCACCTGTTTCTGGACCTGCCTAAG AATAGAATGCAACTGAAGA
chr12 chr12 57898025 59199741 57M59199723F19M                                                                                                                                                                                                                                                                    TGCCGATCATGCCCTGTGGTGCAGTCGAGCCAGCACCTGTTTCTGGACCTGCCTAAG AATAGAATGCAACTGAAGA
chr12 chr12 57898026 59199742 56M59199723F20M                                                                                                                                                                                                                                                                     GCCGATCATGCCCTGTGGTGCAGTCGAGCCAGCACCTGTTTCTGGACCTGCCTAAG AATAGAATGCAACTGAAGAA
chr12 chr12 57898026 59199742 56M59199723F20M                                                                                                                                                                                                                                                                     GCCGATCATGCCCTGTGGTGCAGTCGAGCCAGCACCTGTTTCTGGACCTGCCTAAG AATAGAATGCAACTGAAGAA
chr12 chr12 57898026 59199742 56M59199723F20M                                                                                                                                                                                                                                                                     GCCGATCATGCCCTGTGGTGCAGTCGAGCCAGCACCTGTTTCTGGACCTGCCTAAG AATAGAATGCAACTGAAGAA
chr12 chr12 57898026 59199742 56M59199723F20M                                                                                                                                                                                                                                                                     GCCGATCATGCCCTGTGGTGCAGTCGAGCCAGCACCTGTTTCTGGACCTGCCTAAG AATAGAATGCAACTGAAGAA
chr12 chr12 57898026 59199742 56M59199723F20M                                                                                                                                                                                                                                                                     GCCGATCATGCCCTGTGGTGCAGTCGAGCCAGCACCTGTTTCTGGACCTGCCTAAG AATAGAATGCAACTGAAGAA
chr12 chr12 57898026 59199742 56M59199723F20M                                                                                                                                                                                                                                                                     GCCGATCATGCCCTGTGGTGCAGTCGAGCCAGCACCTGTTTCTGGACCTGCCTAAG AATAGAATGCAACTGAAGAA
chr12 chr12 57898026 59199742 56M59199723F20M                                                                                                                                                                                                                                                                     GCCGATCATGCCCTGTGGTGCAGTCGAGCCAGCACCTGTTTCTGGACCTGCCTAAG AATAGAATGCAACTGAAGAA
chr12 chr12 57898026 59199742 56M59199723F20M                                                                                                                                                                                                                                                                     GCCGATCATGCCCTGTGGTGCAGTCGAGCCAGCACCTGTTTCTGGACCTGCCTAAG AATAGAATGCAACTGAAGAA
chr12 chr12 57898026 59199742 56M59199723F20M                                                                                                                                                                                                                                                                     GCCGATCATGCCCTGTGGTGCAGTCGAGCCAGCACCTGTTTCTGGACCTGCCTAAG AATAGAATGCAACTGAAGAA
chr12 chr12 57898027 59199743 55M59199723F21M                                                                                                                                                                                                                                                                      CCGATCATGCCCTGTGGTGCAGTCGAGCCAGCACCTGTTTCTGGACCTGCCTAAG AATAGAATGCAACTGAAGAAA
chr12 chr12 57898028 59199744 54M59199723F22M                                                                                                                                                                                                                                                                       CGATCATGCCCTGTGGTGCAGTCGAGCCAGCACCTGTTTCTGGACCTGCCTAAG AATAGAATGCAACTGAAGAAAT
chr12 chr12 57898028 59199744 54M59199723F22M                                                                                                                                                                                                                                                                       CGATCATGCCCTGTGGTGCAGTCGAGCCAGCACCTGTTTCTGGACCTGCCTAAG AATAGAATGCAACTGAAGAAAT
chr12 chr12 57898028 59199744 54M59199723F22M                                                                                                                                                                                                                                                                       CGATCATGCCCTGTGGTGCAGTCGAGCCAGCACCTGTTTCTGGACCTGCCTAAG AATAGAATGCAACTGAAGAAAT
chr12 chr12 57898028 59199744 54M59199723F22M                                                                                                                                                                                                                                                                       CGATCATGCCCTGTGGTGCAGTCGAGCCAGCACCTGTTTCTGGACCTGCCTAAG AATAGAATGCAACTGAAGAAAT
chr12 chr12 57898028 59199744 54M59199723F22M                                                                                                                                                                                                                                                                       CGATCTTGCCCTGTGGTGCAGTCGAGCCAGCACCTGTTTCTGGACCTGCCTAAG AATAGAATGCAACTGAAGAAAT
chr12 chr12 57898028 59199744 54M59199723F22M                                                                                                                                                                                                                                                                       CGATCATGCCCTGTGGTGCAGTCGAGCCAGCACCTGTTTCTGGACCTGCCTAAG AATAGAATGCAACTGAAGAAAT
chr12 chr12 57898028 59199744 54M59199723F22M                                                                                                                                                                                                                                                                       CGATCATGCCCTGTGGTGCAGTCGAGCCAGCACCTGTTTCTGGACCTGCCTAAG AATAGAATGCAACTGAAGAAAT
chr12 chr12 57898028 59199744 54M59199723F22M                                                                                                                                                                                                                                                                       CGATCATGCCCTGTGGTGCAGTCGAGCCAGCACCTGTTTCTGGACCTGCCTAAG AATAGAATGCAACTGAAGAAAT
chr12 chr12 57898029 59199745 53M59199723F23M                                                                                                                                                                                                                                                                        GATCATGCCCTGTGGTGCAGTCGAGCCAGCACCTGTTTCTGGACCTGCCTAAG AATAGAATGCAACTGAAGAAATG
chr12 chr12 57898031 59199747 51M59199723F25M                                                                                                                                                                                                                                                                          TCATGCCCTGTGGTGCAGTCGAGCCAGCACCTGTTTCTGGACCTGCCCAAG AATAGAATGCAACTGAAGAAATGGC
chr12 chr12 57898031 59199747 51M59199723F25M                                                                                                                                                                                                                                                                          TCATGCCCTGTGGTGCAGTCGAGCCAGCACCTGTTTCTGGACCTGCCTAAG AATAGAATGCAACTGAAGAAATGGC
chr12 chr12 57898031 59199747 51M59199723F25M                                                                                                                                                                                                                                                                          TCATGCCCTGTGGTGCAGTCGAGCCAGCACCTGTTTCTGGACCTGCCTAAG AATAGAATGCAACTGAAGAAATGGC
chr12 chr12 57898031 59199747 51M59199723F25M                                                                                                                                                                                                                                                                          TCATGCCCTGTGGTGCAGTCGAGCCAGCACCTGTTTCTGGACCTGCCTAAG AATAGAATGCAACTGAAGAAATGGC
chr12 chr12 57898031 59199747 51M59199723F25M                                                                                                                                                                                                                                                                          TCATGCCCGGTGGTGCAGTCGAGCCAGCACCTGTTTCTGGACCTGCCTAAG AATAGAATGCAACTGAAGAAATGGC
chr12 chr12 57898031 59199747 51M59199723F25M                                                                                                                                                                                                                                                                          TCATGCCCTGTGGTGCAGTCGAGCCAGCACCTGTTTCTGGACCTGCCTAAG AATAGAATGCAACTGAAGAAATGGC
chr12 chr12 57898032 59199748 50M59199723F26M                                                                                                                                                                                                                                                                           CATGCCCTGTGGTGCAGTCGAGCCAGCACCTGTTTCTGGACCTGCCTAAG AATAGAATGCAACTGAAGAAATGGCT
chr12 chr12 57898032 59199748 50M59199723F26M                                                                                                                                                                                                                                                                           CATGCCCTGTGGTGCAGTCGAGCCAGCACCTGTTTCTGGACCTGCCTAAG AATAGAATGCAACTGAAGAAATGTCT
chr12 chr12 57898033 59199749 49M59199723F27M                                                                                                                                                                                                                                                                            ATGCCCTGTGGTGCAGTCGAGCCAGCACCTGTTTCTGGACCTGCCTAAG AATAGAATGCAACTGAAGAAATGGCTG
chr12 chr12 57898033 59199749 49M59199723F27M                                                                                                                                                                                                                                                                            ATGCCCTGTGTGGCAGTCGAGCCAGCACCTGTTTCTGGACCTGCCTAAG AATAGAATGCAACTGAAGAAATGGCTG
chr12 chr12 57898033 59199749 49M59199723F27M                                                                                                                                                                                                                                                                            ATTCCCTGTGGTGCGGTCGAGCCAGCACCTGTTTCTGGACCTGCCTAAG AATAGAATGCAACTGAAGAAATGGCTG
chr12 chr12 57898034 59199750 48M59199723F28M                                                                                                                                                                                                                                                                             TGCCCTGTGGTGCAGTCGAGCCAGCACCTGTTTCTGGACCTGCCTAAG AATAGAATGCAACTGAAGAAATGGCCGG
chr12 chr12 57898035 59199751 47M59199723F29M                                                                                                                                                                                                                                                                              GCCCTGTGGTGCAGTCGAGCCAGCACCTGTTTCTGGACCTGCCTAAG AATAGAATGCAACTGAAGAAATGGCTGGG
chr12 chr12 57898035 59199751 47M59199723F29M                                                                                                                                                                                                                                                                              GCCCTGTGGTGCAGTCGAGCCAGCACCTGTTTCTGGACCTGCCTAAG AATAGAATGCAACTGAAGAAATGGCTGGG
chr12 chr12 57898035 59199751 47M59199723F29M                                                                                                                                                                                                                                                                              GCCCTGTGGTGCAGTCGAGCCAGCACCTGTTTCTGGACCTGCCTAAG AATAGAATGCAACTGAAGAAATGGCTGGG
chr12 chr12 57898035 59199751 47M59199723F29M                                                                                                                                                                                                                                                                              GCCCTGTGGTGCAGTCGAGCCAGCACCTGTTTCTGGACCTGCCTAAG AATAGAATGCAACTGAAGAAATGGCTGGG
chr12 chr12 57898035 59199751 47M59199723F29M                                                                                                                                                                                                                                                                              GCCCTGTGGTGCAGTCGAGCCAGCACCTGTTTCTGGACCTGCCTAAG AATAGAATGCAACTGAAGAAATGGCTGGG
chr12 chr12 57898036 59199752 46M59199723F30M                                                                                                                                                                                                                                                                               CCCTGTGGTGCAGTCGAGCCAGCACCTGTTTCTGGACCTGCCTAAG AATAGAATGCAACTGAAGAAATGGCTGGGG
chr12 chr12 57898036 59199752 46M59199723F30M                                                                                                                                                                                                                                                                               CCCTGTGGTGCAGTCGAGCCAGCACCTGTTTCTGGACCTGCCTAAG AATAGAATGCAACTGAAGAAATGGCTGGGG
chr12 chr12 57898036 59199752 46M59199723F30M                                                                                                                                                                                                                                                                               CCCTGTGGTGCAGTCGAGCCAGCACCTGTTTCTGGACCTGCCTAAG AATAGAATGCAACTGAAGAAATGGCTGGGG
chr12 chr12 57898037 59199753 45M59199723F31M                                                                                                                                                                                                                                                                                CCTGTGGTGCAGTCGAGCCAGCACCTGTTTCTGGACCTGCCTAAG AATAGAATGCAACTGAAGAAATGGCTGGTGC
chr12 chr12 57898037 59199753 45M59199723F31M                                                                                                                                                                                                                                                                                CCTGTGGTGCAGTCGAGCCAGCACCTGTTTCTGGACCTGCCTAAG AATAGAATGCAACTGAAGAAATGGCTGGTGC
chr12 chr12 57898038 59199754 44M59199723F32M                                                                                                                                                                                                                                                                                 CTGTGGTGCAGTCGAGCCAGCACCTGTTTCTGGACCTGCCTAAG AATAGAATGCAACTGAAGAAATGGCTGGTGCA
chr12 chr12 57898038 59199754 44M59199723F32M                                                                                                                                                                                                                                                                                 CTGTGGTGCAGTCGAGCCAGCACCTGTTTCTGGACCTGCCTAAG AATAGAATGCAACTGAAGAAATGGCTGGGGCA
chr12 chr12 57898038 59199754 44M59199723F32M                                                                                                                                                                                                                                                                                 CTGTGGTGCAGTCGAGCCAGCACCTGTTTCTGGACCTGCCTAAG AATAGAATGCAACTGAAGAAATGGCTGGTGCA
chr12 chr12 57898040 59199756 42M59199723F34M                                                                                                                                                                                                                                                                                   GTGGTGCAGTCGAGCCAGCACCTGTTTCTGGACCTGCCTAAG AATAGAATGCAACTGAAGAAATGGCTGGGGCAAT
chr12 chr12 57898040 59199756 42M59199723F34M                                                                                                                                                                                                                                                                                   GTGGTGCAGTCGAGCCAGCACCTGTTTCTGGACCTGCCTAAG AATAGAATGCAACTGAAGAAATGGCTGGTGCAAT
chr12 chr12 57898040 59199756 42M59199723F34M                                                                                                                                                                                                                                                                                   GTGGTGCAGTCGAGCCAGCACCTGTTTCTGGACCTGCCTAAG AATAGAATGCAACTGAAGAAATGGCTGGTGCAAT
chr12 chr12 57898043 59199759 39M59199723F37M                                                                                                                                                                                                                                                                                      GTGCAGTCGAGCCAGCACCTGTTTCTGGACCTGCCTAAG AATAGAATGCAACTGAAGAAATGGCTGGGGCAATTCA
chr12 chr12 57898043 59199759 39M59199723F37M                                                                                                                                                                                                                                                                                      GTGCAGTCGAGCCAGCACCTGTTTCTGGACCTGCCTAAG AATAGAATGCAACTGAAGAAATGGCTGGTGCAATTCA
chr12 chr12 57898043 59199759 39M59199723F37M                                                                                                                                                                                                                                                                                      GTGCAGTCGAGCCAGCACCTGTTTCTGGACCTGCCTAAG AATAGAATGCAACTGAAGAAATGGCTGGTGCCATTCA
chr12 chr12 57898043 59199759 39M59199723F37M                                                                                                                                                                                                                                                                                      GTGCAGTCGAGCCAGCACCTGTTTCTGGACCTGCCTAAG AATAGAATGCAACTGAAGAAATGGCTGGTGCAATTCA
chr12 chr12 57898043 59199759 39M59199723F37M                                                                                                                                                                                                                                                                                      GTGCAGTCGAGCCAGCACCTGTTTCTGGACCTGCCTAAG AATAGAATGCAACTGAAGAAATGGCTGGTGCAATTCA
chr12 chr12 57898043 59199759 39M59199723F37M                                                                                                                                                                                                                                                                                      GTGCAGTCGAGCCAGCACCTGTTTCTGGACCTGCCTAAG AATAGAATGCAACTGAAGAAATGGCTGGTGCAATTCA
chr12 chr12 57898043 59199759 39M59199723F37M                                                                                                                                                                                                                                                                                      GTGCAGTCGAGCCAGCACCTGTTTCTGGACCTGCCTAAG AATAGAATGCAACTGAAGAAATGGCTGGGGCAATTCA
chr12 chr12 57898043 59199759 39M59199723F37M                                                                                                                                                                                                                                                                                      GTGCAGTCGAGCCAGCACCTGTTTCTGGACCTGCCTAAG AATAGAATGCAACTGAAGAAATGGCTGGGGCAATTCA
chr12 chr12 57898043 59199759 39M59199723F37M                                                                                                                                                                                                                                                                                      GTGCAGTCGAGCCAGCACCTGTTTCTGGACCTGCCTAAG AATAGAATGCAACTGAAGAAATGGCTGGGGCAATTCA
chr12 chr12 57898043 59199759 39M59199723F37M                                                                                                                                                                                                                                                                                      GTGCAGTCGAGCCAGCACCTGTTTCTGGACCTGCCTAAG AATAGAATGCAACTGAAGAAATGGCTGGTGCAAATCA
chr12 chr12 57898043 59199759 39M59199723F37M                                                                                                                                                                                                                                                                                      GTGCAGTCGAGCCAGCACCTGTTTCTGGACCTGCCTAAG AATAGAATGCAACTGAAGAAATGGCTGGGGCAATTCA
chr12 chr12 57898043 59199759 39M59199723F37M                                                                                                                                                                                                                                                                                      GTGCAGTCGAGCCAGCACCTGTTTCTGGACCTGCCTAAG AATAGAATGCAACTGAAGAAATGGCTGGTGCAATTCA
chr12 chr12 57898043 59199759 39M59199723F37M                                                                                                                                                                                                                                                                                      GTGCAGTCGAGCCAGCACCTGTTTCTGGACCTGCCTAAG AATAGAATGCAACTGAAGAAATGGCTGGGGCAATTCA
chr12 chr12 57898044 59199760 38M59199723F38M                                                                                                                                                                                                                                                                                       TGCAGTCGAGCCAGCACCTGTTTCTGGACCTGCCTAAG AATAGAATGCAACTGAAGAAATGGCTGGTGCAATTCAG
chr12 chr12 57898044 59199760 38M59199723F38M                                                                                                                                                                                                                                                                                       TGCAGTCGAGCCAGCACCTGTTTCTGGACCTGCCTAAG AATAGAATGCAACTGAAGAAATGGCTGGTGCAATTCAG
chr12 chr12 57898044 59199760 38M59199723F38M                                                                                                                                                                                                                                                                                       TGCAGTCGAGCCAGCACCTGTTTCTGGACCTGCCTAAG AATAGAATGCAACTGAAGAAATGGCTGGTGCAATTCAG
chr12 chr12 57898044 59199760 38M59199723F38M                                                                                                                                                                                                                                                                                       TCCATTCGAGCCAGCACCTGTTTCTGGACCTGCCTAAG AATAGAATGCAACTGAAGAAATGGCTGGTGCAATTCAG
chr12 chr12 57898044 59199760 38M59199723F38M                                                                                                                                                                                                                                                                                       TGCAGTCGAGCCAGCACCTGTTTCTGGACCTGCCTAAG AATAGAATGCAACTGAAGAAATGGCTGGTGCAATTCAG
chr12 chr12 57898044 59199760 38M59199723F38M                                                                                                                                                                                                                                                                                       TGCAGTCGAGCCAGCACCTGTTTCTGGACCTGCCTAAG AATAGAATGCAACTGAAGAAATGGCTGGTGCAATTCAG
chr12 chr12 57898046 59199762 36M59199723F40M                                                                                                                                                                                                                                                                                         CAGTCGAGCCAGCACCTGTTTCTGGACCTGCCTAAG AATAGAATGCAACTGAAGAAATGGCTGGTGCAATTCAGCA
chr12 chr12 57898046 59199762 36M59199723F40M                                                                                                                                                                                                                                                                                         CAGTCGAGCCAGCACCTGTTTCTGGACCTGCCTAAG AATAGAATGCAACTGAAGAAATGGCTGGTGCAATTCAGCA
chr12 chr12 57898047 59199763 35M59199723F41M                                                                                                                                                                                                                                                                                          AGTCGAGCCAGCACCTGTTTCTGGACCTGCCTAAG AATAGAATGCAACTGAAGAAATGGCTGGTGCAATTCAGCAA
chr12 chr12 57898047 59199763 35M59199723F41M                                                                                                                                                                                                                                                                                          AGTCGAGCCAGCACCTGTTTCTGGACCTGCCTAAG AATAGAATGCAACTGAAGAAATGGCTGGTGCAATTTAGCAA
chr12 chr12 57898047 59199763 35M59199723F41M                                                                                                                                                                                                                                                                                          AGTCGAGCCAGCACCTGTTTCTGGACCTGCCTAAG AATAGAATGCAACTGAAGAAATGGCTGGTGCAATTCAGCAA
chr12 chr12 57898047 59199763 35M59199723F41M                                                                                                                                                                                                                                                                                          AGTCGAGCCAGCACCTGTTTCTGGACCTGCCTAAG AATAGAATGCAACTGAAGAAATGGCTGGTGCAATTCAGCAA
chr12 chr12 57898047 59199763 35M59199723F41M                                                                                                                                                                                                                                                                                          AGTCGAGCCAGCACCTGTTTCTGGACCTGCCTAAG AATAGAATGCAACTGAAGAAATGGCTGGTGCAATTCAGCAA
chr12 chr12 57898048 59199764 34M59199723F42M                                                                                                                                                                                                                                                                                           GTCGAGCCAGCACCTGTTTCTGGACCTGCCTAAG AATAGAATGCAACTGAAGAAATGGCTGGTGCAATTCAGCAAG
chr12 chr12 57898048 59199764 34M59199723F42M                                                                                                                                                                                                                                                                                           GTCGAGCCAGCACCTGTTTCTGGACCTGCCTAAG AATAGAATGCAACTGAAGAAATGGCTGGTGCAATTCAGCCAA
chr12 chr12 57898048 59199764 34M59199723F42M                                                                                                                                                                                                                                                                                           GTCGAGCCAGCACCTGTTTCTGGACCTGCCTAAG AATAGAATGCAACTGAAGAAATGGCTGGTGCAATTCAGCAAG
chr12 chr12 57898048 59199764 34M59199723F42M                                                                                                                                                                                                                                                                                           GTCGAGCCAGCACCTGTTTCTGGACCTGCCTAAG AATAGAATGCAACTGAAGAAATGGCTGGGGCAATTCAGCAAG
chr12 chr12 57898048 59199764 34M59199723F42M                                                                                                                                                                                                                                                                                           GTCGAGCCAGCACCTGTTTCTGGACCTGCCTAAG AGTAGAATGCAACGGAAGAAATGGCTGGTGCAATTCAGCAAG
chr12 chr12 57898048 59199764 34M59199723F42M                                                                                                                                                                                                                                                                                           GTCGAGCCAGCACCTGTTTCTGGACCTGCCTAAG AATAGAATGCAACTGAAGAAATGGCTGGTGCAATTCAGCAAG
chr12 chr12 57898048 59199764 34M59199723F42M                                                                                                                                                                                                                                                                                           GTCGAGCCAGCACCTGTTTCTGGACCTGCCTAAG AATAGAATGCAACTGAAGAAATGGCTGGTGCAAATCAGCAAG
chr12 chr12 57898048 59199764 34M59199723F42M                                                                                                                                                                                                                                                                                           GTCGAGCCAGCACCTGTTTCTGGACCTGCCTAAG AATAGAATGCAACTGAAGAAATGGCTGGTGCAATTCAGCAAG
chr12 chr12 57898048 59199764 34M59199723F42M                                                                                                                                                                                                                                                                                           GTCGAGCCAGCACCTGTTTCTGGACCTGCCTAAG AATAGAATGCAACTGAAGAAATGGCTGGTGCAATTCAGCAAG
chr12 chr12 57898048 59199764 34M59199723F42M                                                                                                                                                                                                                                                                                           GTCGAGCCAGCACCTGTTTCTGGACCTGCCTAAG AATAGAATGCAACTGAAGAAATGGCTGGTGCAATTCAGCAAG
chr12 chr12 57898048 59199764 34M59199723F42M                                                                                                                                                                                                                                                                                           GTCGAGCCAGCACCTGTTTCTGGACCTGCCTAAG AATAGAATGCAACTGAAGAAATGGCTGGTGCAATTCAGCAAG
chr12 chr12 57898049 59206196 33M59199723F42M6431N1M                                                                                                                                                                                                                                                                                     TCGAGCCAGCACCTGTTTCTGGACCTGCCTAAG AATAGAATGCAACTGAAGAAATGGCTGGTGCAATTCAGCAAG||||||||||||||||||||||||||||||||||||||||||||||||||||||||||||||||||||||||||||||||||||||||||||||||||||||||||||||||||||||||||||||||||||||||||||||||||||||||||||||||||||||||||||||||||||||||||||||||||||||||||||||||||||||||||||||||||||||||||||||||||||||||||||||||
chr12 chr12 57898050 59206197 32M59199723F42M6431N2M                                                                                                                                                                                                                                                                                      CGAGCCAGCACCTGTTTCTGGACCTGCCTAAG AATAGAATGCAACTGAAGAAATGGCTGGGGCAATTCAGCAAG||||||||||||||||||||||||||||||||||||||||||||||||||||||||||||||||||||||||||||||||||||||||||||||||||||||||||||||||||||||||||||||||||||||||||||||||||||||||||||||||||||||||||||||||||||||||||||||||||||||||||||||||||||||||||||||||||||||||||||||||||||||||||||||||
chr12 chr12 57898050 59206197 32M59199723F42M6431N2M                                                                                                                                                                                                                                                                                      CGAGCCAGCACCTGTTTCTGGACCTGCCTAAG AATAGAATGCAACTGAAGAAATGGCTGGTGCAATTCAGCAAG||||||||||||||||||||||||||||||||||||||||||||||||||||||||||||||||||||||||||||||||||||||||||||||||||||||||||||||||||||||||||||||||||||||||||||||||||||||||||||||||||||||||||||||||||||||||||||||||||||||||||||||||||||||||||||||||||||||||||||||||||||||||||||||||
chr12 chr12 57898050 59205705 32M59199723F42M5939N2M                                                                                                                                                                                                                                                                                      CGAGCCAGCACCTGTTTCTGGACCTGCCTAAG AATAGAATGCAACTGAAGAAATGGCTGGTGCAATTCAGCAAG||||||||||||||||||||||||||||||||||||||||||||||||||||||||||||||||||||||||||||||||||||||||||||||||||||||||||||||||||||||||||||||||||||||||||||||||||||||||||||||||||||||||||||||||||||||||||||||||||||||||||||||||||||||||||||||||||||||||||||||||||||||||||||||||
chr12 chr12 57898050 59206197 32M59199723F42M6431N2M                                                                                                                                                                                                                                                                                      CGAGCCAGCACCTGTTTCTGGACCTGCCTAAG AATAGAATGCAACTGAAGAAATGGCTGGGGCAATTCAGCAAG||||||||||||||||||||||||||||||||||||||||||||||||||||||||||||||||||||||||||||||||||||||||||||||||||||||||||||||||||||||||||||||||||||||||||||||||||||||||||||||||||||||||||||||||||||||||||||||||||||||||||||||||||||||||||||||||||||||||||||||||||||||||||||||||
chr12 chr12 57898050 59206197 32M59199723F42M6431N2M                                                                                                                                                                                                                                                                                      TGAGCCAGCACCTGTTTCTGGACCTGCCTAAG AATAGAATGCAACTGAAGAAATGGCTGGGGCAATTCAGCAAG||||||||||||||||||||||||||||||||||||||||||||||||||||||||||||||||||||||||||||||||||||||||||||||||||||||||||||||||||||||||||||||||||||||||||||||||||||||||||||||||||||||||||||||||||||||||||||||||||||||||||||||||||||||||||||||||||||||||||||||||||||||||||||||||
chr12 chr12 57898050 59206197 32M59199723F42M6431N2M                                                                                                                                                                                                                                                                                      CGAGCCAGCACCTGTTTCTGGACCTGCCTAAG AATAGAAGGCAACTGAAGAAATGGCTGGTGCAATTCAGCAAG||||||||||||||||||||||||||||||||||||||||||||||||||||||||||||||||||||||||||||||||||||||||||||||||||||||||||||||||||||||||||||||||||||||||||||||||||||||||||||||||||||||||||||||||||||||||||||||||||||||||||||||||||||||||||||||||||||||||||||||||||||||||||||||||
chr12 chr12 57898050 59206197 32M59199723F42M6431N2M                                                                                                                                                                                                                                                                                      CGAGCCAGCACCTGTTTCTGGACCTGCCTAAG AATAGAATGCAACTGAAGAAATGGCTGGGGCAATTCAGCAAG||||||||||||||||||||||||||||||||||||||||||||||||||||||||||||||||||||||||||||||||||||||||||||||||||||||||||||||||||||||||||||||||||||||||||||||||||||||||||||||||||||||||||||||||||||||||||||||||||||||||||||||||||||||||||||||||||||||||||||||||||||||||||||||||
chr12 chr12 57898050 59206197 32M59199723F42M6431N2M                                                                                                                                                                                                                                                                                      CGAGCCAGCACCTGTTTCTGGACCTGCCTAAG AATAGAATGCAACTGAAGAAATGGCTGGGGCAATTCAGCAAG||||||||||||||||||||||||||||||||||||||||||||||||||||||||||||||||||||||||||||||||||||||||||||||||||||||||||||||||||||||||||||||||||||||||||||||||||||||||||||||||||||||||||||||||||||||||||||||||||||||||||||||||||||||||||||||||||||||||||||||||||||||||||||||||
chr12 chr12 57898050 59206197 32M59199723F42M6431N2M                                                                                                                                                                                                                                                                                      CGAGCCAGCACCTGTTTCTGGACCTGCCTAAG AATAGAATGCAACTGAAGAAATGGCTGGTGCAATTCAGCAAG||||||||||||||||||||||||||||||||||||||||||||||||||||||||||||||||||||||||||||||||||||||||||||||||||||||||||||||||||||||||||||||||||||||||||||||||||||||||||||||||||||||||||||||||||||||||||||||||||||||||||||||||||||||||||||||||||||||||||||||||||||||||||||||||
chr12 chr12 57898050 59206197 32M59199723F42M6431N2M                                                                                                                                                                                                                                                                                      CGAGCCAGCACCTGTTTCTGGACCTGCCTAAG AATAGAATGCAACTGAAGAAATGGCTGGTGCAATTCAGCAAG||||||||||||||||||||||||||||||||||||||||||||||||||||||||||||||||||||||||||||||||||||||||||||||||||||||||||||||||||||||||||||||||||||||||||||||||||||||||||||||||||||||||||||||||||||||||||||||||||||||||||||||||||||||||||||||||||||||||||||||||||||||||||||||||
chr12 chr12 57898050 59206197 32M59199723F42M6431N2M                                                                                                                                                                                                                                                                                      CGAGCCAGCACCTGTTTCTGGACCTGCCTAAG AATAGAATGCAACTGAAGAAATGGCTGGTGCAATTCAGCAAG||||||||||||||||||||||||||||||||||||||||||||||||||||||||||||||||||||||||||||||||||||||||||||||||||||||||||||||||||||||||||||||||||||||||||||||||||||||||||||||||||||||||||||||||||||||||||||||||||||||||||||||||||||||||||||||||||||||||||||||||||||||||||||||||
chr12 chr12 57898050 59206197 32M59199723F42M6431N2M                                                                                                                                                                                                                                                                                      CGAGCCAGCACCTGTTTCTGGACCTGCCTAAG AATAGAATGCAACTGAAGAAATGGCTGGTGCAATTCAGCAAG||||||||||||||||||||||||||||||||||||||||||||||||||||||||||||||||||||||||||||||||||||||||||||||||||||||||||||||||||||||||||||||||||||||||||||||||||||||||||||||||||||||||||||||||||||||||||||||||||||||||||||||||||||||||||||||||||||||||||||||||||||||||||||||||
chr12 chr12 57898051 59199767 31M59199723F45M                                                                                                                                                                                                                                                                                              GAGCCAGCACCTGTTTCTGGACCTGCCTAAG AATAGAATGCAACTGAAGAAATGGCTGGAGAAATTCAGCAAGGTA
chr12 chr12 57898051 59199767 31M59199723F45M                                                                                                                                                                                                                                                                                              GAGCCAGCACCTGTTTCTGGACCTGCCTAAG AATAGAATGCAACTGAAGAAATGGCTGGTGCAATTCAGCAAGGTA
chr12 chr12 57898051 59206198 31M59199723F42M6431N3M                                                                                                                                                                                                                                                                                       GAGCCAGCACCTGTTTCTGGACCTGCCTAAG AATAGAATGCAACTGAAGAAATGGCTGGTGCAATTCAGCAAG||||||||||||||||||||||||||||||||||||||||||||||||||||||||||||||||||||||||||||||||||||||||||||||||||||||||||||||||||||||||||||||||||||||||||||||||||||||||||||||||||||||||||||||||||||||||||||||||||||||||||||||||||||||||||||||||||||||||||||||||||||||||||||||||
chr12 chr12 57898053 59206200 29M59199723F42M6431N5M                                                                                                                                                                                                                                                                                         GCCAGCACCTGTTTCTGGACCTGCCTAAG AATAGAATGCAACTGAAGAAATGGCTGGTGCAATTCAGCAAG||||||||||||||||||||||||||||||||||||||||||||||||||||||||||||||||||||||||||||||||||||||||||||||||||||||||||||||||||||||||||||||||||||||||||||||||||||||||||||||||||||||||||||||||||||||||||||||||||||||||||||||||||||||||||||||||||||||||||||||||||||||||||||||||
chr12 chr12 57898053 59206200 29M59199723F42M6431N5M                                                                                                                                                                                                                                                                                         GCCAGCACCTGTTTCTGGACCTGCCTAAG AATAGAATGCAACTGAAGAAATGGCTGGTGCAATTCAGCAAG||||||||||||||||||||||||||||||||||||||||||||||||||||||||||||||||||||||||||||||||||||||||||||||||||||||||||||||||||||||||||||||||||||||||||||||||||||||||||||||||||||||||||||||||||||||||||||||||||||||||||||||||||||||||||||||||||||||||||||||||||||||||||||||||
chr12 chr12 57898053 59206200 29M59199723F42M6431N5M                                                                                                                                                                                                                                                                                         GCCAGCACCTGTTTCTGGACCTGCCTAAG AATAGAATGCAACTGAAGAAATGGCTGGTGCAATTCAGCAAG||||||||||||||||||||||||||||||||||||||||||||||||||||||||||||||||||||||||||||||||||||||||||||||||||||||||||||||||||||||||||||||||||||||||||||||||||||||||||||||||||||||||||||||||||||||||||||||||||||||||||||||||||||||||||||||||||||||||||||||||||||||||||||||||
chr12 chr12 57898053 59206200 29M59199723F42M6431N5M                                                                                                                                                                                                                                                                                         GCCAGCACCTGTTTCTGGACCTGCCTAAG AATAGAATGCAACTGAAGAAATGGCTGGTGCAATTCAGCAAG||||||||||||||||||||||||||||||||||||||||||||||||||||||||||||||||||||||||||||||||||||||||||||||||||||||||||||||||||||||||||||||||||||||||||||||||||||||||||||||||||||||||||||||||||||||||||||||||||||||||||||||||||||||||||||||||||||||||||||||||||||||||||||||||
chr12 chr12 57898053 59206200 29M59199723F42M6431N5M                                                                                                                                                                                                                                                                                         GCCAGCACCTGTTTCTGGACCTGCCTAAG AATAGAATGCAACTGAAGAAATGGCTGGTGCAATTCAGCAAG||||||||||||||||||||||||||||||||||||||||||||||||||||||||||||||||||||||||||||||||||||||||||||||||||||||||||||||||||||||||||||||||||||||||||||||||||||||||||||||||||||||||||||||||||||||||||||||||||||||||||||||||||||||||||||||||||||||||||||||||||||||||||||||||
chr12 chr12 57898054 59199770 28M59199723F48M                                                                                                                                                                                                                                                                                                 CCAGCACCTGTTTCTGGACCTGCCTAAG AATAGAATGCAACTGAAGAAATGGCTGGGGCAATTCAGCAAGGTAAAA
chr12 chr12 57898054 59199770 28M59199723F48M                                                                                                                                                                                                                                                                                                 CCAGCACCTGTTTCTGGACCTGCCTAAG AATAGAATGCAACTGAAGAAATGGCTGGGGCAATTCAGCAAGGTAAAA
chr12 chr12 57898054 59199770 28M59199723F48M                                                                                                                                                                                                                                                                                                 CCAGCACCTGTTTCTGGACCTGCCTAAG AATAGAATGCAACTGAAGAAATGGCTGGTGCAATTCAGCAAGGTAAAA
chr12 chr12 57898054 59206201 28M59199723F42M6431N6M                                                                                                                                                                                                                                                                                          CCAGCACCTGTTTCTGGACCTGCCTAAG AATAGAATGCAACTGAAGAAATGGCTGGTGCAATTCAGCAAG||||||||||||||||||||||||||||||||||||||||||||||||||||||||||||||||||||||||||||||||||||||||||||||||||||||||||||||||||||||||||||||||||||||||||||||||||||||||||||||||||||||||||||||||||||||||||||||||||||||||||||||||||||||||||||||||||||||||||||||||||||||||||||||||
chr12 chr12 57898054 59206201 28M59199723F42M6431N6M                                                                                                                                                                                                                                                                                          CCAGCACCTGTTTCTGGACCTGCCTAAG AATAGAATGCAACTGAAGAAATGGCTGGTGCAATTCAGCAAG||||||||||||||||||||||||||||||||||||||||||||||||||||||||||||||||||||||||||||||||||||||||||||||||||||||||||||||||||||||||||||||||||||||||||||||||||||||||||||||||||||||||||||||||||||||||||||||||||||||||||||||||||||||||||||||||||||||||||||||||||||||||||||||||
chr12 chr12 57898054 59206201 28M59199723F42M6431N6M                                                                                                                                                                                                                                                                                          CCAGCACCTGTTTCTGGACCTGCCTAAG AATAGAATGCAACTGAAGAAATGGCTGGTGCAATTCAGCAAG||||||||||||||||||||||||||||||||||||||||||||||||||||||||||||||||||||||||||||||||||||||||||||||||||||||||||||||||||||||||||||||||||||||||||||||||||||||||||||||||||||||||||||||||||||||||||||||||||||||||||||||||||||||||||||||||||||||||||||||||||||||||||||||||
chr12 chr12 57898054 59206201 28M59199723F42M6431N6M                                                                                                                                                                                                                                                                                          CCAGCACCTGTTTCTGGACCTGCCTAAG AATAGAATGCAACTGAAGAAATGGCTGGTGCAATTCAGCAAG||||||||||||||||||||||||||||||||||||||||||||||||||||||||||||||||||||||||||||||||||||||||||||||||||||||||||||||||||||||||||||||||||||||||||||||||||||||||||||||||||||||||||||||||||||||||||||||||||||||||||||||||||||||||||||||||||||||||||||||||||||||||||||||||
chr12 chr12 57898054 59206201 28M59199723F42M6431N6M                                                                                                                                                                                                                                                                                          CCAGCACCTGTTTCTGGACCTGCCTAAG AATAGAATGCAACTGAAGAAATGGCTGGTGCAATTCAGCAAG||||||||||||||||||||||||||||||||||||||||||||||||||||||||||||||||||||||||||||||||||||||||||||||||||||||||||||||||||||||||||||||||||||||||||||||||||||||||||||||||||||||||||||||||||||||||||||||||||||||||||||||||||||||||||||||||||||||||||||||||||||||||||||||||
chr12 chr12 57898054 59206201 28M59199723F42M6431N6M                                                                                                                                                                                                                                                                                          CCAGCACCTGTTTCTGGACCTGCCTAAG AATAGAATGCAACTGAAGAAATGGCTGGTGCAATTCAGCAAG||||||||||||||||||||||||||||||||||||||||||||||||||||||||||||||||||||||||||||||||||||||||||||||||||||||||||||||||||||||||||||||||||||||||||||||||||||||||||||||||||||||||||||||||||||||||||||||||||||||||||||||||||||||||||||||||||||||||||||||||||||||||||||||||
chr12 chr12 57898054 59206201 28M59199723F42M6431N6M                                                                                                                                                                                                                                                                                          CCAGCACCTGTTTCTGGACCTGCCTAAG AATAGAATGCAACTGAAGAAATGGCTGGTGCAATTCAGCAAG||||||||||||||||||||||||||||||||||||||||||||||||||||||||||||||||||||||||||||||||||||||||||||||||||||||||||||||||||||||||||||||||||||||||||||||||||||||||||||||||||||||||||||||||||||||||||||||||||||||||||||||||||||||||||||||||||||||||||||||||||||||||||||||||
chr12 chr12 57898054 59206201 28M59199723F42M6431N6M                                                                                                                                                                                                                                                                                          CCAGCACCTGTTTCTGGACCTGCCTAAG AATAGAATGCAACTGAAGAAATGGCTGGTGCAATTCAGCAAG||||||||||||||||||||||||||||||||||||||||||||||||||||||||||||||||||||||||||||||||||||||||||||||||||||||||||||||||||||||||||||||||||||||||||||||||||||||||||||||||||||||||||||||||||||||||||||||||||||||||||||||||||||||||||||||||||||||||||||||||||||||||||||||||
chr12 chr12 57898054 59206201 28M59199723F42M6431N6M                                                                                                                                                                                                                                                                                          CCAGCACCTGTTTCTGGACCTGCCTAAG AATAGAATGCAACTGAAGAAATGGCTGGTGCAATTCAGCAAG||||||||||||||||||||||||||||||||||||||||||||||||||||||||||||||||||||||||||||||||||||||||||||||||||||||||||||||||||||||||||||||||||||||||||||||||||||||||||||||||||||||||||||||||||||||||||||||||||||||||||||||||||||||||||||||||||||||||||||||||||||||||||||||||
chr12 chr12 57898054 59206201 28M59199723F42M6431N6M                                                                                                                                                                                                                                                                                          CCAGCACCTGTTTCTGGACCTGCCTAAG AATAGAATGCAACTGAAGAAATGGCTGGTGCAATTCAGCAAG||||||||||||||||||||||||||||||||||||||||||||||||||||||||||||||||||||||||||||||||||||||||||||||||||||||||||||||||||||||||||||||||||||||||||||||||||||||||||||||||||||||||||||||||||||||||||||||||||||||||||||||||||||||||||||||||||||||||||||||||||||||||||||||||
chr12 chr12 57898054 59206201 28M59199723F42M6431N6M                                                                                                                                                                                                                                                                                          CCAGCACCTGTTTCTGGACCTGCCTAAG AATAGAATGCAACTGAAGAAATGGCTGGTGCAATTCAGCAAG||||||||||||||||||||||||||||||||||||||||||||||||||||||||||||||||||||||||||||||||||||||||||||||||||||||||||||||||||||||||||||||||||||||||||||||||||||||||||||||||||||||||||||||||||||||||||||||||||||||||||||||||||||||||||||||||||||||||||||||||||||||||||||||||
chr12 chr12 57898055 59206202 27M59199723F42M6431N7M                                                                                                                                                                                                                                                                                           CAGCACCTGTTTCTGGACCTGCCTAAG AATAGAATGCAACTGAAGAAATGGCTGGTGCAATTCAGCAAG||||||||||||||||||||||||||||||||||||||||||||||||||||||||||||||||||||||||||||||||||||||||||||||||||||||||||||||||||||||||||||||||||||||||||||||||||||||||||||||||||||||||||||||||||||||||||||||||||||||||||||||||||||||||||||||||||||||||||||||||||||||||||||||||
chr12 chr12 57898055 59206202 27M59199723F42M6431N7M                                                                                                                                                                                                                                                                                           CAGCCCCTGTTTCTGGACCTGCCTAAG AATAGAATGCAACTGAAGAAATGGCTGGTGCAATTCAGCAAG||||||||||||||||||||||||||||||||||||||||||||||||||||||||||||||||||||||||||||||||||||||||||||||||||||||||||||||||||||||||||||||||||||||||||||||||||||||||||||||||||||||||||||||||||||||||||||||||||||||||||||||||||||||||||||||||||||||||||||||||||||||||||||||||
chr12 chr12 57898055 59206202 27M59199723F42M6431N7M                                                                                                                                                                                                                                                                                           CAGCACCTGTTTCTGGACCTGCCTAAG AATAGAATGCAACTGAAGAAATGGCTGGTGCAATTCAGCAAG||||||||||||||||||||||||||||||||||||||||||||||||||||||||||||||||||||||||||||||||||||||||||||||||||||||||||||||||||||||||||||||||||||||||||||||||||||||||||||||||||||||||||||||||||||||||||||||||||||||||||||||||||||||||||||||||||||||||||||||||||||||||||||||||
chr12 chr12 57898055 59206202 27M59199723F42M6431N7M                                                                                                                                                                                                                                                                                           CAGCACCTGTTTCTGGACCTGCCTAAG AATAGAATGCAACTGAAGAAATGGCTGGTGCAATTCAGCAAG||||||||||||||||||||||||||||||||||||||||||||||||||||||||||||||||||||||||||||||||||||||||||||||||||||||||||||||||||||||||||||||||||||||||||||||||||||||||||||||||||||||||||||||||||||||||||||||||||||||||||||||||||||||||||||||||||||||||||||||||||||||||||||||||
chr12 chr12 57898055 59199771 27M59199723F49M                                                                                                                                                                                                                                                                                                  CAGCACCTGTTTCTGGACCTGCCTAAG AATAGAATGCAACTGAAGAAATGGCTGGTGCAATTCAGCAAGGTAAAAG
chr12 chr12 57898057 59206204 25M59199723F42M6431N9M                                                                                                                                                                                                                                                                                             GCACCTGTTTCTGGACCTGCCTAAG AATAGAATGCAACTGAAGAAATGGCTGGGGCAATTCAGCAAG||||||||||||||||||||||||||||||||||||||||||||||||||||||||||||||||||||||||||||||||||||||||||||||||||||||||||||||||||||||||||||||||||||||||||||||||||||||||||||||||||||||||||||||||||||||||||||||||||||||||||||||||||||||||||||||||||||||||||||||||||||||||||||||||
chr12 chr12 57898057 59206204 25M59199723F42M6431N9M                                                                                                                                                                                                                                                                                             GCACCTGTTTCTGGACCTGCCTAAG AATAGAATGCAACTGAAGAAATGGCTGGTGCAATTCAGCAAG||||||||||||||||||||||||||||||||||||||||||||||||||||||||||||||||||||||||||||||||||||||||||||||||||||||||||||||||||||||||||||||||||||||||||||||||||||||||||||||||||||||||||||||||||||||||||||||||||||||||||||||||||||||||||||||||||||||||||||||||||||||||||||||||
chr12 chr12 57898057 59206204 25M59199723F42M6431N9M                                                                                                                                                                                                                                                                                             GCACCTGTTTCTGGACCTGCCTAAG AATAGAATGCAACTGAAGAAATGGCTGGTGCAATTCAGCAAG||||||||||||||||||||||||||||||||||||||||||||||||||||||||||||||||||||||||||||||||||||||||||||||||||||||||||||||||||||||||||||||||||||||||||||||||||||||||||||||||||||||||||||||||||||||||||||||||||||||||||||||||||||||||||||||||||||||||||||||||||||||||||||||||
chr12 chr12 57898058 59206205 24M59199723F42M6431N10M                                                                                                                                                                                                                                                                                             CACCTGTTTCTGGACCTGCCTAAG AATAGAATGCAACTGAAGAAATGGCTGGTGCAATTCAGCAAG||||||||||||||||||||||||||||||||||||||||||||||||||||||||||||||||||||||||||||||||||||||||||||||||||||||||||||||||||||||||||||||||||||||||||||||||||||||||||||||||||||||||||||||||||||||||||||||||||||||||||||||||||||||||||||||||||||||||||||||||||||||||||||||||
chr12 chr12 57898059 59199775 23M59199723F53M                                                                                                                                                                                                                                                                                                      ACCTGTTTCTGGACCTGCCTAAG AATAGAATGCAACTGAAGAAATGGCTGGTGCAATTCAGCAAGGTAAAAGATTA
chr12 chr12 57898060 59206207 22M59199723F42M6431N12M                                                                                                                                                                                                                                                                                               CCTGTTTCTGGACCTGCCTAAG AATAGAATGCAACTGAAGAAATGGCTGGTGCAATTCAGCAAG||||||||||||||||||||||||||||||||||||||||||||||||||||||||||||||||||||||||||||||||||||||||||||||||||||||||||||||||||||||||||||||||||||||||||||||||||||||||||||||||||||||||||||||||||||||||||||||||||||||||||||||||||||||||||||||||||||||||||||||||||||||||||||||||
chr12 chr12 57898060 59206207 22M59199723F42M6431N12M                                                                                                                                                                                                                                                                                               CCTGTTTCTGGACCTGCCTAAG AATAGAATGCAACTGAAGAAATGGCTGGTGCAATTCAGCAAG||||||||||||||||||||||||||||||||||||||||||||||||||||||||||||||||||||||||||||||||||||||||||||||||||||||||||||||||||||||||||||||||||||||||||||||||||||||||||||||||||||||||||||||||||||||||||||||||||||||||||||||||||||||||||||||||||||||||||||||||||||||||||||||||
chr12 chr12 57898060 59206207 22M59199723F42M6431N12M                                                                                                                                                                                                                                                                                               CCTGTTTCTGGACCTGCCTAAG AATAGAATGCAACTGAAGAAATGGCTGGTGCAATTCAGCAAG||||||||||||||||||||||||||||||||||||||||||||||||||||||||||||||||||||||||||||||||||||||||||||||||||||||||||||||||||||||||||||||||||||||||||||||||||||||||||||||||||||||||||||||||||||||||||||||||||||||||||||||||||||||||||||||||||||||||||||||||||||||||||||||||
chr12 chr12 57898060 59199776 22M59199723F54M                                                                                                                                                                                                                                                                                                       CCTGTTTCTGGACCTGCCTAAG AATAGAATGCAACTGAAGAAATGGCTGGTGCAATTCAGCAAGGTAAAAGATTAT
chr12 chr12 57898060 59206207 22M59199723F42M6431N12M                                                                                                                                                                                                                                                                                               CCTGTTTCTGGACCTGCCTAAG AATAGAATGCAACTGAAGAAATGGCTGGTGCAATTCAGCAAG||||||||||||||||||||||||||||||||||||||||||||||||||||||||||||||||||||||||||||||||||||||||||||||||||||||||||||||||||||||||||||||||||||||||||||||||||||||||||||||||||||||||||||||||||||||||||||||||||||||||||||||||||||||||||||||||||||||||||||||||||||||||||||||||
chr12 chr12 57898060 59206207 22M59199723F42M6431N12M                                                                                                                                                                                                                                                                                               CCTGTTTCTGGACCTGCCTAAG AATAGAATGCAACTGAAGAAATGGCTGGGGCAATTCAGCAAG||||||||||||||||||||||||||||||||||||||||||||||||||||||||||||||||||||||||||||||||||||||||||||||||||||||||||||||||||||||||||||||||||||||||||||||||||||||||||||||||||||||||||||||||||||||||||||||||||||||||||||||||||||||||||||||||||||||||||||||||||||||||||||||||
chr12 chr12 57898060 59206207 22M59199723F42M6431N12M                                                                                                                                                                                                                                                                                               CCTGTTTCTGGACCTGCCTAAG AATAGAATGCAACTGAAGAAATGGCTGGGGCAATTCAGCAAG||||||||||||||||||||||||||||||||||||||||||||||||||||||||||||||||||||||||||||||||||||||||||||||||||||||||||||||||||||||||||||||||||||||||||||||||||||||||||||||||||||||||||||||||||||||||||||||||||||||||||||||||||||||||||||||||||||||||||||||||||||||||||||||||
chr12 chr12 57898060 59199776 22M59199723F54M                                                                                                                                                                                                                                                                                                       CCTGTTTCTGGACCTGCCTAAG AATAGAATGCAACTGAAGAAATGGCTGGTGCAATTCAGCAAGGTAAAAGATTAT
chr12 chr12 57898061 59206208 21M59199723F42M6431N13M                                                                                                                                                                                                                                                                                                CTGTTTCTGGACCTGCCTAAG AATAGAATGCAACTGAAGAAATGGCTGGTGCAATTCAGCAAG||||||||||||||||||||||||||||||||||||||||||||||||||||||||||||||||||||||||||||||||||||||||||||||||||||||||||||||||||||||||||||||||||||||||||||||||||||||||||||||||||||||||||||||||||||||||||||||||||||||||||||||||||||||||||||||||||||||||||||||||||||||||||||||||
chr12 chr12 57898062 59199778 20M59199723F56M                                                                                                                                                                                                                                                                                                         TGTTTCTGGACCTGCCTAAG AATAGAATGCAACTGAAGAAATGGCTGGTGCAATTCAGCAAGGTAAAAGATTATCT
chr12 chr12 57898062 59206209 20M59199723F42M6431N14M                                                                                                                                                                                                                                                                                                 TGTTTCTGGACCTGCCTAAG AATAGAATGCAACTGAAGAAATGGCTGGTGCAATTCAGCAAG||||||||||||||||||||||||||||||||||||||||||||||||||||||||||||||||||||||||||||||||||||||||||||||||||||||||||||||||||||||||||||||||||||||||||||||||||||||||||||||||||||||||||||||||||||||||||||||||||||||||||||||||||||||||||||||||||||||||||||||||||||||||||||||||
chr12 chr12 57898062 59206209 20M59199723F42M6431N14M                                                                                                                                                                                                                                                                                                 TGTTTCTGGACCTGCCTAAG AATAGAATGCAACTGAAGAAATGGCTGGTGCAATTCAGCAAG||||||||||||||||||||||||||||||||||||||||||||||||||||||||||||||||||||||||||||||||||||||||||||||||||||||||||||||||||||||||||||||||||||||||||||||||||||||||||||||||||||||||||||||||||||||||||||||||||||||||||||||||||||||||||||||||||||||||||||||||||||||||||||||||
chr12 chr12 57898063 59206210 19M59199723F42M6431N15M                                                                                                                                                                                                                                                                                                  GTTTCTGGACCTGCCTAAG AATAGAATGCAACTGAAGAAATGGCTGGTGCAATTCAGCAAG||||||||||||||||||||||||||||||||||||||||||||||||||||||||||||||||||||||||||||||||||||||||||||||||||||||||||||||||||||||||||||||||||||||||||||||||||||||||||||||||||||||||||||||||||||||||||||||||||||||||||||||||||||||||||||||||||||||||||||||||||||||||||||||||
chr12 chr12 57898063 59206210 19M59199723F42M6431N15M                                                                                                                                                                                                                                                                                                  GTTTCTGGACCTGCCTAAG AATAGAATGCAACTGAAGAAATGGCTGGTGCAATTCAGCAAG||||||||||||||||||||||||||||||||||||||||||||||||||||||||||||||||||||||||||||||||||||||||||||||||||||||||||||||||||||||||||||||||||||||||||||||||||||||||||||||||||||||||||||||||||||||||||||||||||||||||||||||||||||||||||||||||||||||||||||||||||||||||||||||||
chr12 chr12 57898063 59206210 19M59199723F42M6431N15M                                                                                                                                                                                                                                                                                                  GTTTCTGGACCTGCCTAAG AATAGAATGCAACTGAAGAAATGGCTGGTGCAACTCAGCAAG||||||||||||||||||||||||||||||||||||||||||||||||||||||||||||||||||||||||||||||||||||||||||||||||||||||||||||||||||||||||||||||||||||||||||||||||||||||||||||||||||||||||||||||||||||||||||||||||||||||||||||||||||||||||||||||||||||||||||||||||||||||||||||||||
chr12 chr12 57898063 59206210 19M59199723F42M6431N15M                                                                                                                                                                                                                                                                                                  GTTTCTGGACCTGCCTAAG AATAGAATGCAACTGAAGAAATGGCTGGGGCAATTCAGCAAG||||||||||||||||||||||||||||||||||||||||||||||||||||||||||||||||||||||||||||||||||||||||||||||||||||||||||||||||||||||||||||||||||||||||||||||||||||||||||||||||||||||||||||||||||||||||||||||||||||||||||||||||||||||||||||||||||||||||||||||||||||||||||||||||
chr12 chr12 57898063 59206210 19M59199723F42M6431N15M                                                                                                                                                                                                                                                                                                  GTTTCTGGACCTGCCTAAG AATAGAATGCAACTGAAGAAATGGCTGGTGCAATTCAGCAAG||||||||||||||||||||||||||||||||||||||||||||||||||||||||||||||||||||||||||||||||||||||||||||||||||||||||||||||||||||||||||||||||||||||||||||||||||||||||||||||||||||||||||||||||||||||||||||||||||||||||||||||||||||||||||||||||||||||||||||||||||||||||||||||||
chr12 chr12 57898063 59206210 19M59199723F42M6431N15M                                                                                                                                                                                                                                                                                                  GTTTCTGGACCTGCCTAAG AATAGAATGCAACTGAAGAAATGGCTGGTGCAATTCAGCAAG||||||||||||||||||||||||||||||||||||||||||||||||||||||||||||||||||||||||||||||||||||||||||||||||||||||||||||||||||||||||||||||||||||||||||||||||||||||||||||||||||||||||||||||||||||||||||||||||||||||||||||||||||||||||||||||||||||||||||||||||||||||||||||||||
chr12 chr12 57898064 59206211 18M59199723F42M6431N16M                                                                                                                                                                                                                                                                                                   TTTCTGGACCTGCCTAAG AATAGAATGCAACTGAAGAAATGGCTGGTGCAATTCAGCAAG||||||||||||||||||||||||||||||||||||||||||||||||||||||||||||||||||||||||||||||||||||||||||||||||||||||||||||||||||||||||||||||||||||||||||||||||||||||||||||||||||||||||||||||||||||||||||||||||||||||||||||||||||||||||||||||||||||||||||||||||||||||||||||||||
chr12 chr12 57898065 59206212 17M59199723F42M6431N17M                                                                                                                                                                                                                                                                                                    TTCTGGACCTGCCTAAG AATAGAATGCAACTGAAGAAATGGCTGGTGCAATTCAGCAAG||||||||||||||||||||||||||||||||||||||||||||||||||||||||||||||||||||||||||||||||||||||||||||||||||||||||||||||||||||||||||||||||||||||||||||||||||||||||||||||||||||||||||||||||||||||||||||||||||||||||||||||||||||||||||||||||||||||||||||||||||||||||||||||||
chr12 chr12 57898065 59206212 17M59199723F42M6431N17M                                                                                                                                                                                                                                                                                                    TGCTGGACCTGCCTAAG AATAGAATGCAACTGAAGAAATGGCTGGTGCAATTCAGCAAG||||||||||||||||||||||||||||||||||||||||||||||||||||||||||||||||||||||||||||||||||||||||||||||||||||||||||||||||||||||||||||||||||||||||||||||||||||||||||||||||||||||||||||||||||||||||||||||||||||||||||||||||||||||||||||||||||||||||||||||||||||||||||||||||
chr12 chr12 57898066 59199782 16M59199723F60M                                                                                                                                                                                                                                                                                                             TCTGGACCTGCCTAAG AATAGAATGCAACTGAAGAAATGGCTGGTGCAATTCAGCAAGGTAAAAGATTATCTTACC
chr12 chr12 57898066 59205721 16M59199723F42M5939N18M                                                                                                                                                                                                                                                                                                     TCTGGACCTGCCTAAG AATAGAATGCAACTGAAGAAATGGCTGGTGCAATTCAGCAAG||||||||||||||||||||||||||||||||||||||||||||||||||||||||||||||||||||||||||||||||||||||||||||||||||||||||||||||||||||||||||||||||||||||||||||||||||||||||||||||||||||||||||||||||||||||||||||||||||||||||||||||||||||||||||||||||||||||||||||||||||||||||||||||||
chr12 chr12 57898066 59199782 16M59199723F60M                                                                                                                                                                                                                                                                                                             TCTGGACCTGCCTAAG AATAGAATGCAACTGAAGAAATGGCTGGTGCAATTCAGCAAGGTAAAAGATTATCTTACC
chr12 chr12 57898066 59206213 16M59199723F42M6431N18M                                                                                                                                                                                                                                                                                                     TCTGGACCTGCCTAAG AATAGAATGCAACTGAAGAAATGGCTGGGGCAATTCAGCAAG||||||||||||||||||||||||||||||||||||||||||||||||||||||||||||||||||||||||||||||||||||||||||||||||||||||||||||||||||||||||||||||||||||||||||||||||||||||||||||||||||||||||||||||||||||||||||||||||||||||||||||||||||||||||||||||||||||||||||||||||||||||||||||||||
chr12 chr12 57898066 59206213 16M59199723F42M6431N18M                                                                                                                                                                                                                                                                                                     TCTGGACCTGCCTAAG AATAGAATGCAACTGAAGAAATGGCTGGTGCAATTCAGCAAG||||||||||||||||||||||||||||||||||||||||||||||||||||||||||||||||||||||||||||||||||||||||||||||||||||||||||||||||||||||||||||||||||||||||||||||||||||||||||||||||||||||||||||||||||||||||||||||||||||||||||||||||||||||||||||||||||||||||||||||||||||||||||||||||
chr12 chr12 57898066 59206213 16M59199723F42M6431N18M                                                                                                                                                                                                                                                                                                     TCTGGACCTGCCTAAG AATAGAATGCAACTGAAGAAATGGCTGGTGCAATTCAGCAAG||||||||||||||||||||||||||||||||||||||||||||||||||||||||||||||||||||||||||||||||||||||||||||||||||||||||||||||||||||||||||||||||||||||||||||||||||||||||||||||||||||||||||||||||||||||||||||||||||||||||||||||||||||||||||||||||||||||||||||||||||||||||||||||||
chr12 chr12 57898066 59206213 16M59199723F42M6431N18M                                                                                                                                                                                                                                                                                                     TCTGGACCTGCCTAAG AATAGAATGCAACTGAAGAAATGGCTGGTGCAATTCAGCAAG||||||||||||||||||||||||||||||||||||||||||||||||||||||||||||||||||||||||||||||||||||||||||||||||||||||||||||||||||||||||||||||||||||||||||||||||||||||||||||||||||||||||||||||||||||||||||||||||||||||||||||||||||||||||||||||||||||||||||||||||||||||||||||||||
chr12 chr12 57898066 59206213 16M59199723F42M6431N18M                                                                                                                                                                                                                                                                                                     TCTGGACCTGCCTAAG AATAGAATGCAACTGAAGAAATGGCTGGTGCAATTCAGCAAG||||||||||||||||||||||||||||||||||||||||||||||||||||||||||||||||||||||||||||||||||||||||||||||||||||||||||||||||||||||||||||||||||||||||||||||||||||||||||||||||||||||||||||||||||||||||||||||||||||||||||||||||||||||||||||||||||||||||||||||||||||||||||||||||
chr12 chr12 57898066 59206213 16M59199723F42M6431N18M                                                                                                                                                                                                                                                                                                     TCTGGACCTGCCTAAG AATAGAATGCAACTGAAGAAATGGCTGGTGCAATTCAGCAAG||||||||||||||||||||||||||||||||||||||||||||||||||||||||||||||||||||||||||||||||||||||||||||||||||||||||||||||||||||||||||||||||||||||||||||||||||||||||||||||||||||||||||||||||||||||||||||||||||||||||||||||||||||||||||||||||||||||||||||||||||||||||||||||||
chr12 chr12 57898066 59206213 16M59199723F42M6431N18M                                                                                                                                                                                                                                                                                                     TCTGGACCTGCCTAAG AATAGAATGCAACTGAAGAAATGGCTGGTGCAATTCAGCAAG||||||||||||||||||||||||||||||||||||||||||||||||||||||||||||||||||||||||||||||||||||||||||||||||||||||||||||||||||||||||||||||||||||||||||||||||||||||||||||||||||||||||||||||||||||||||||||||||||||||||||||||||||||||||||||||||||||||||||||||||||||||||||||||||
chr12 chr12 57898067 59206214 15M59199723F42M6431N19M                                                                                                                                                                                                                                                                                                      CTGGACCTGCCTAAG AATAGAATGCAACTGAAGAAATGGCTGGTGCAATTCAGCAAG||||||||||||||||||||||||||||||||||||||||||||||||||||||||||||||||||||||||||||||||||||||||||||||||||||||||||||||||||||||||||||||||||||||||||||||||||||||||||||||||||||||||||||||||||||||||||||||||||||||||||||||||||||||||||||||||||||||||||||||||||||||||||||||||
chr12 chr12 57898067 59206214 15M59199723F42M6431N19M                                                                                                                                                                                                                                                                                                      CTGGACCTGCCTAAG AATAGAATGCAACTGAAGAAATGGCTGGTGCAATTCAGCAAG||||||||||||||||||||||||||||||||||||||||||||||||||||||||||||||||||||||||||||||||||||||||||||||||||||||||||||||||||||||||||||||||||||||||||||||||||||||||||||||||||||||||||||||||||||||||||||||||||||||||||||||||||||||||||||||||||||||||||||||||||||||||||||||||
chr12 chr12 57898067 59206214 15M59199723F42M6431N19M                                                                                                                                                                                                                                                                                                      CTGGACCTGCCTAAG AATAGAATGCAACTGAAGAAATGGCTGGTGCAATTCAGCAAG||||||||||||||||||||||||||||||||||||||||||||||||||||||||||||||||||||||||||||||||||||||||||||||||||||||||||||||||||||||||||||||||||||||||||||||||||||||||||||||||||||||||||||||||||||||||||||||||||||||||||||||||||||||||||||||||||||||||||||||||||||||||||||||||
chr12 chr12 57898068 59206215 14M59199723F42M6431N20M                                                                                                                                                                                                                                                                                                       TGGACCTGCCTAAG AATAGAATGCAACTGAAGAAATGGCTGGTGCAATTCAGCAAG||||||||||||||||||||||||||||||||||||||||||||||||||||||||||||||||||||||||||||||||||||||||||||||||||||||||||||||||||||||||||||||||||||||||||||||||||||||||||||||||||||||||||||||||||||||||||||||||||||||||||||||||||||||||||||||||||||||||||||||||||||||||||||||||
chr12 chr12 57898068 59206215 14M59199723F42M6431N20M                                                                                                                                                                                                                                                                                                       TGGACCTGCCTAAG AATAGAATGCAACTGAAGAAATGGCTGGTGCAATTCAGCAAG||||||||||||||||||||||||||||||||||||||||||||||||||||||||||||||||||||||||||||||||||||||||||||||||||||||||||||||||||||||||||||||||||||||||||||||||||||||||||||||||||||||||||||||||||||||||||||||||||||||||||||||||||||||||||||||||||||||||||||||||||||||||||||||||
[truncated: 114,318,355 more chars]
